# Ionic liquids of superior thermal stability. Validation of PPh_4_^+^ as an organic cation of impressive thermodynamic durability†

**DOI:** 10.1039/d0ra03220d

**Published:** 2020-05-29

**Authors:** Mohammad Soltani, Jimmie L. McGeehee, Alexandra C. Stenson, Richard A. O'Brien, Edward R. Duranty, E. Alan Salter, Andrzej Wierzbicki, T. Grant Glover, James H. Davis

**Affiliations:** a Department of Chemistry, University of South Alabama Mobile Alabama 36688 USA jdavis@southalabama.edu

## Abstract

Recent work by Wasserscheid, *et al.* suggests that PPh_4_^+^ is an organic molecular ion of truly exceptional thermal stability. Herein we provide data that cements that conclusion: specifically, we show that aliphatic moieties of modified PPh_4_^+^-based cations incorporating methyl, methylene, or methine C–H bonds burn away at high temperatures in the presence of oxygen, forming CO, CO_2_, and water, leaving behind the parent ion PPh_4_^+^. The latter then undergoes no further reaction, at least below 425 °C.

## Abstract 2

When appended to the tetraphenylphosphonium cation, organic groups containing aliphatic C–H bonds are burned away at high temperatures in the presence of O_2_. However, the parent cation remains unscathed, demonstrating its remarkable thermal stability.

## Graphical abstract

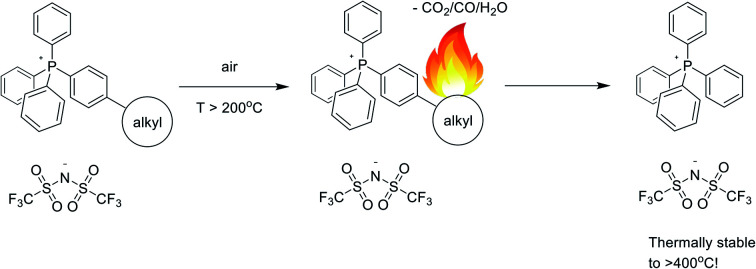

Since 2013 we have sought to design, prepare, and study ionic liquids that have degrees of thermal stability superior to those exhibited by commonplace imidazolium, quaternary ammonium, and tetraalkylphosphonium-type ILs.^1–8^ And, while high thermal stability is important to ILs slated to be used as solvents in synthetic applications,^9^ their development is especially important to their practical utility as additives to high-performance polymers, advanced lubricants, and as liquid- or ‘liquifiable’ materials for energy-related uses such as energy storage *via* phase change and heat transfer fluids in solar thermal arrays.^10^ We note that the latter applications are those in which we especially envision future practical roles for the present class of ILs.

This new family of ionic liquids of improved thermal stability is formulated around perarylated cations (cations commonly being the thermal weak link of organic salts),^11^ and the “success stories” among them (typified by compounds 1 and 2, Fig. 1) are routinely stable for months (perhaps indefinitely), in air, at temperatures of 300 °C–400 °C; as a consequence they rank among the most thermally stable organic materials heretofore described.^7^ Furthermore, they constitute a class of salts which bridge a melting point/thermal stability (“mesothermal”) gap that arguably exists between classical, all-inorganic molten salts and compounds more widely referred to as ionic liquids. The basis for the superior thermal stability of these perarylated cations is thought to be rooted in their relative invulnerability to retro-S_N_2 or Hoffman elimination reactions, these being the principal mechanistic pathways by which more widely studied IL-cations are known to thermally decompose.^7,11^

**Fig. 1:**
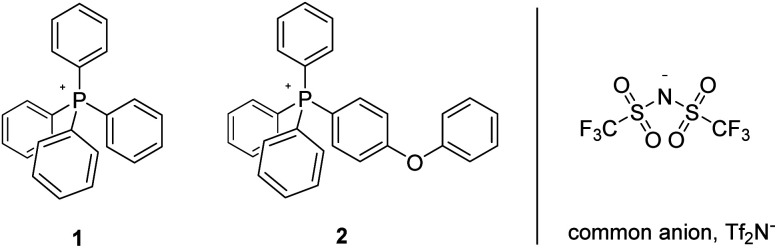
Ionic liquids previously established to be thermally stable in air for periods of 90 days and greater at *T* ≥ 350 °C.

However, even from our first study it was clear that certain modifications to these otherwise thermally stable peraryl onium cations led to changes in this attribute compared to the parent ion. Notably, those cations which are modified with aliphatic moieties are the most impacted, in-line with what is known about the relative thermal reactivity of aliphatic [C(sp^3^)–H] *versus* aromatic [C(sp^2^)–H] C–H bonds.^12–17^ Once melted, as the temperature to which the water-white liquids are subjected continues to be increased, their colour changes to brown, clear evidence of heat-induced changes to the structures and/or composition of the salts. At the same time, however, these changes are usually accompanied by only modest mass losses. In this light, it seems clear that the observed thermal ‘decomposition’ process likely produces two classes of product: volatile substances of low-molecular weight that account for the observed mass losses, and non- or less-volatile materials, likely those remaining ionic, that account for the residual mass.

Their curiosity piqued by a progress report incorporating the former observations, the reviewers of the renewal application for the grant which has provided funding to conduct work on thermally stable ILs expressed a strong interest in having us seek insights into how/why this introduction of aliphatic components into the cations diminishes their thermal stability; interest was also expressed in our gaining insight into the nature of the decomposition products. Concurring that acquiring this information would be potentially useful, we undertook the present study.

Here we have synthesized and investigated the pyrolysis of a number of tetraarylphosphonium Tf_2_N salts, the cations of 3, 6, 12–14, 16, 18, and 20 each having an arene-ring appendage containing an aliphatic C–H bond that is part of a methyl, methylene, or methine moiety. The structures of these salts, all but one of which (3) is new, are illustrated in Fig. 2. In every case, the Tf_2_N^−^ anion was employed. Our decision to employ it was driven by the established nature of its high intrinsic thermal stability, and its demonstrated lack of reactivity (*versus* several other ions) towards tetraarylphosphonium cations.^2,4^

**Fig. 2:**
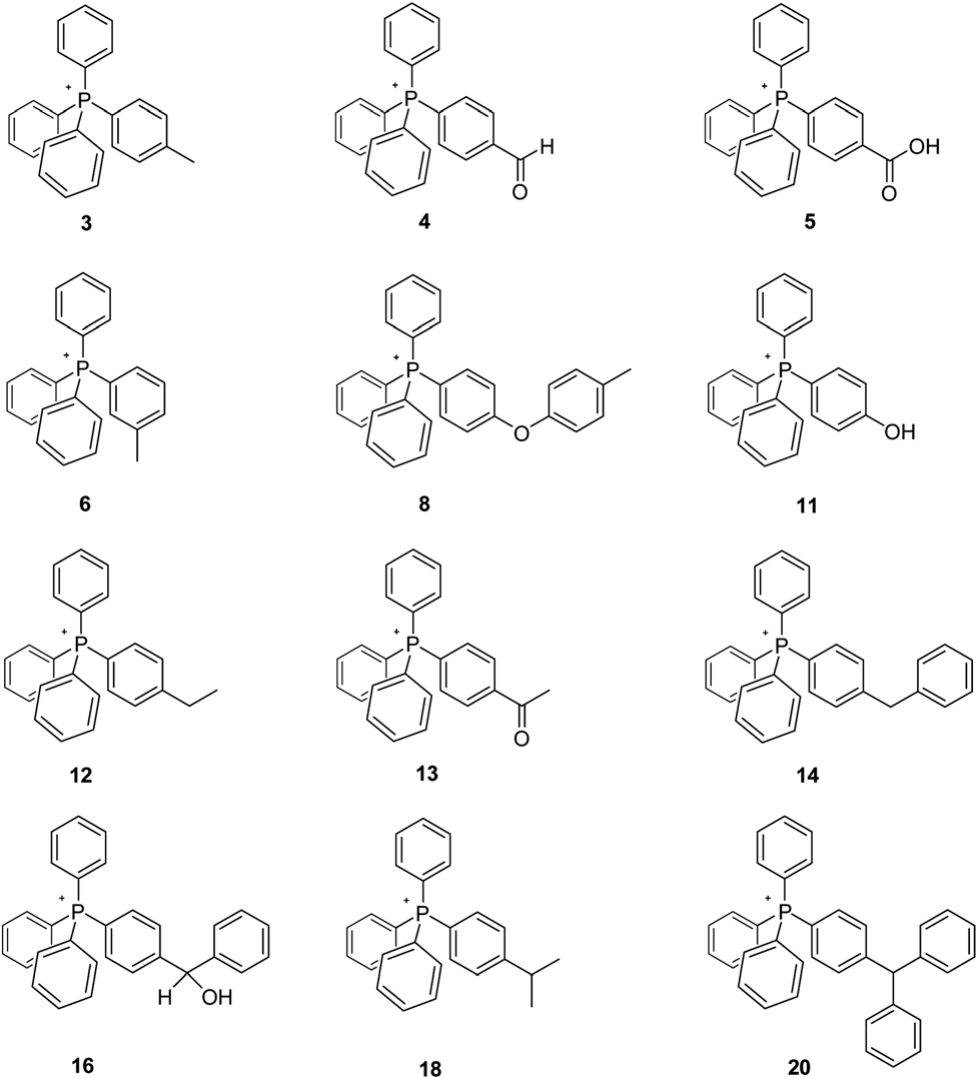
New mesothermal ionic liquids prepared for the present study. The Tf_2_N^−^ anion is common to all.

As in all our prior work, these were prepared using a modification of a synthetic methodology used by Charette and co-workers.^18^ In brief, this consists of the dissolution of equimolar amounts of triphenylphosphine and an appropriately substituted aryl bromide, chloride, or iodide in ethylene glycol, to which has been added *ca.* 5 mol% NiCl_2_·6H_2_O. The mixture is sealed in a thick-walled, screw-capped tube, in which is a magnetic stir bar. The tube is immersed in an oil bath maintained at 180 °C, then heated with stirring for 2 hours. Upon cooling, the solution is added to a two-phase system consisting of water and dichloromethane, the catalyst and ethylene glycol partitioning into the water, and the desired tetraarylphosphonium salt into the dichloromethane. The organic phase is separated, dried over anhydrous MgSO_4_, and the solvent removed to leave a white solid. Replacement of the bromide anion of this intermediate salt is accomplished by dissolving it in hot water, then adding to the stirred solution a slight (*ca.* 5%) molar excess of solid KTf_2_N. The rapid dissolution of the latter in the aqueous phase is quickly followed by the precipitation of the final phosphonium Tf_2_N salt, usually as a white solid. On occasion, the product separates as an oil which solidifies over the course of a few hours. Yields of both the intermediate halide salts and the final products are commonly quantitative.

Thermochemical evaluations were carried out in the following fashions. First, gradient and isothermal thermogravimetric analyses (TGA) were conducted in air using a TGA 550 with quartz furnace, air being used to maximize chances for compound decomposition/oxidation. During the gradient measurements, *T* was increased at a rate of 2 °C per minute from ambient to 500 °C. Isothermal experiments were carried out by ramping to the target hold temperature – 200 °C, 250 °C, or 300 °C, at a rate of 5 °C per minute. Once reached, the hold temperature was maintained for 10 hours, after which time the system *T* was ramped to 500 °C at a rate of 5 °C per minute. TGA traces are supplied in the ESI.†

In separate experiments, samples of each IL were heated in air to 200 °C, 250 °C, and 300 °C in a Thermolyne FB-1315M furnace for a 96 hour period, with each sample then being analysed post-heating by ^1^H-, ^13^C-, ^31^P-, and ^19^F-NMR (JEOL ECA 500), as well as by electrospray ionization mass spectra (ThermoFisher LTQ-Velos). This particular thermoanalytic approach – developed specifically to subject ILs to harsh, ‘real-world’ conditions – was conceived and its utility validated by Fox *et al.* at Savannah River National Laboratory, before being employed by our group.^19^ Finally, to complement both of the former experimental protocols, samples were also aerobically pyrolyzed under air in a sealed IR gas cell in order to capture and analyse the volatile components of the thermo-oxidative breakdown (ThermoFisher Nicolet is50 with MCTa detector).

The TGA data proved to be rather unrevealing in the sense that they betrayed no clear-cut relationship between mass changes at/in any given temperature or temperature range and assignable chemical changes in the salts, unlike the relationships that proved to be understandable by NMR, ESI-MS, and FTIR in conjunction with the furnace experiments (*vide infra*). In retrospect, this not particularly surprising, since concurrent volatile-fragment elimination (mass loss) and incorporation of oxygen (mass gain) into the still-ionic (hence non-volatile) perarylphosphonium cation core is likely to have occurred (*vide infra*). Such behaviour, while infrequently observed with ordinary, wholly covalent organic materials nevertheless has precedents, especially in association with intrinsically non-volatile and/or non-combustible materials.^20^ Such a course of events, depicted conjecturally for compound 3 (Scheme 1), does not appear to be commonly considered in TGA studies of ILs, probably because the experiments are routinely conducted in inert nitrogen or argon atmospheres rather than in a reactive atmosphere of air or O_2_. Although the data generated in the former instance is unarguably valuable within its context, it should be noted that those TGA outcomes may be of little utility in anticipating the thermal stability of an IL in an eventual application in which it is deliberately or incidentally exposed to air while operating at high temperatures. Making the latter such determinations is a central theme of our work.

**Scheme 1:**
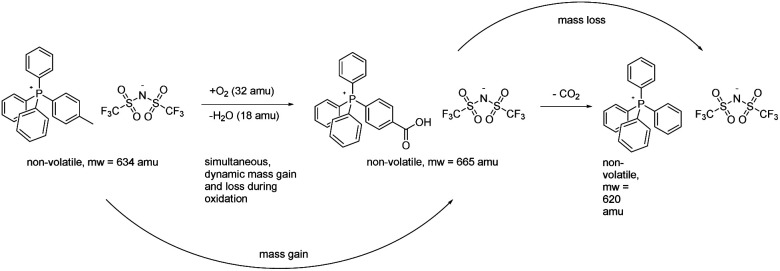
Overlapping mass gain and loss from different chemical reactions occurring during aerobic thermolysis can frustrate the use of TGA to connect specific chemical changes to particular temperatures.^18^

As described previously, each material was also heated in air (porcelain crucible) to 200 °C, 250 °C, and 300 °C in a furnace for a 96 hour period, followed by residue analysis using ^1^H-, ^13^C-, ^31^P-, and ^19^F-NMR, as well as ESI-MS. From these experiments, it was plainly apparent that the condensed-phase pyrolysis products contained no fluorine other than that contained in the Tf_2_N^−^ anion; the ^19^F spectrum of all compounds was invariant over time, consistent with there being no heat-induced changes to this anion at 200 °C, 250 °C or 300 °C. This fully comports with experiments by Wasserscheid, *et al.* establishing that the decomposition temperature of Tf_2_N^−^ is ≥420 °C.^2^ In contrast, there were routinely specific, clearly discernible changes observed in the ^1^H-, ^13^C- and ESI mass spectra of the post-heating condensed phases, and by using FTIR (*vide infra*) gaseous off-products were readily identified. By-in-large, the data emergent from these analyses was such that the identities of the major decomposition product(s) could be assigned.

The aerobic, heat-induced transformations of the *p*-tolyl salt 3 is typical of those observed to occur with most of the present alkyl-bearing tetraarylphosphonium salts (Scheme 2). Consequently, a detailed discussion of these will be a useful primer for grasping the changes observed with the other salts as well.

**Scheme 2:**
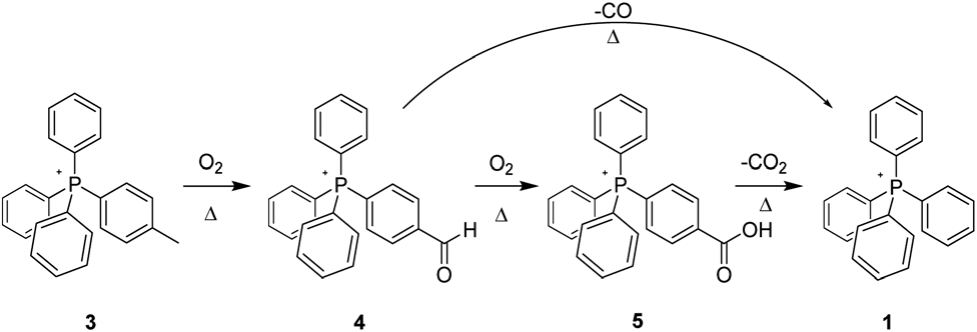
Aerobic, pyrolytic conversion of 3 into 1, and a proposed mechanism for the process.

Over the lifetime of the pyrolysis, the 3 : 4 relative intensity of the tolyl CH_3_ group in the ^1^H-NMR spectrum *versus* that of the tolyl aromatic hydrogens progressively diminished. These changes were easily identified as being associated with the substituted phenyl ring since samples of 3 were also prepared using d^15^-PPh_3_ to suppress overlapping signals from aromatic protons distal to the expected locus of change, *e.g.* the aliphatic-substituted ring. Parenthetically, the ^2^H spectra of 3 and a number of like-deuterated counterparts among the other salts were also acquired, and none showed any sign of thermolytic change on the part of the three non-alkylated phenyls.

Simultaneously, in the aromatic region, the original P-coupled AA′BB′ signals of parent 3 centred at 7.44 and 7.51 ppm grew much smaller, and were replaced with two emerging sets of AA′BB′ patterns at lower field. One pattern set, centred at 7.74 and 8.36 ppm, was considerably larger than the other, which appeared at 7.90 and 8.09 ppm. Coincident with these changes, a small new peak appeared at 10.12 ppm, while a broader, much larger resonance grew in at 9.03 ppm. The location of the former was consistent with an aldehyde –CHO; the latter, while at atypically high field for a carboxylic acid COOH, was nevertheless concluded to be such. This assignment was buttressed by (a) the intensity of the signal, which integrates as one relative to the integrated intensity of four arising specifically from the 7.74/8.36 ppm AA′BB′ signal set noted above, and (b) the complete disappearance of the signal upon addition of a trace of D_2_O. Note that D_2_O addition had no effect on the peak at 10.12 ppm, consistent with it arising from the non-exchangeable *H* of an aldehyde group.

Progressive changes in the ^13^C-NMR of 3 likewise support the emergence over time of aldehyde (4) and acid-bearing (5) derivatives originating in the reaction of the tolyl CH_3_, per Scheme 1. The clearly-identifiable resonance of the latter feature at 21.52 ppm grows steadily smaller upon heating, while a new peak emerges (168.08 ppm) that is assigned as a –COOH carbon. A second, smaller carbonyl peak (–CHO) was observed at 165.65 ppm. In addition, the very small AA′BB′ set of ^1^H peaks at 7.90 and 8.09 ppm comport well with an aldehyde-bearing arene ring, while the large AA′BB′ set of ^1^H peaks at 7.74 and 8.36 ppm are consistent with a –COOH functionalized ring. Further validating these assignments, the ESI-MS of the pyrolysis product is clearly consistent with conversion of the tolyl-bearing cation (*m*/*z* = 368) into one bearing a carboxylic acid group in its place (*m*/*z* = 398), while an additional small peak at *m*/*z* = 382 is likewise consistent with the conversion of the methyl group into an aldehyde.

The NMR and MS data mutually support the proposition that aerobic thermolysis of 3 converts the tolyl methyl group into oxygenated species, specifically an aldehyde and a carboxylic acid; further, we posit that this transformation is sequential, oxidation to the aldehyde preceding oxidation to the acid. Such a pattern of reactivity closely tracks that observed for the high-temperature oxidative transformation of structures in coal and coal model compounds.^21^ In those instances, aliphatic moieties attached to aromatic rings form benzyl radicals which then react with O_2_. Further support for the proposition that the same pattern is plausible with alkylated phosphonium salts is provided by calculations‡‡Calculations were UB3LYP/6-311+g(d,p) optimizations of the radical doublets performed using Gaussian'16; spin densities were rendered with Spartan'08. The exchange reaction energy was estimated using B3LYP/aug-cc-pvtz single-point energies and zero-point corrections from B3LYP/6-31g(d) frequency calculations. The atomic spin density assignments are based upon natural charges as determined by population analysis of the UB3LYP/aug-cc-pvtz densities. which show that the spin densities of a putative benzyl radical formed by 3 and that formed by toluene are virtually indistinguishable. Also, the idealized exchange reaction that connects these two radicals and their parents is energetically flat, with formation of toluene's benzyl radical favoured by just 0.53 kcal mol^−1^.

Even so, in order to provide direct experimental support for thermolysis involving sequential changes to 3, we independently prepared salt 4, the proposed aldehyde intermediate. This material was then subjected to pyrolysis under the conditions previously stated. Doing so, as anticipated, resulted in the conversion of the aldehyde group to the corresponding acid 5 (^1^H-, ^13^C-NMR; ESI-MS). However, a careful examination of the NMR spectra suggested an additional, unexpected change; while the peaks for the aldehyde group were now gone, and those corresponding to the acid were in evidence, the aromatic region of both the ^1^H- and ^13^C-spectra were more complex than should have been the case were only 5 now present. Proceeding on conjecture, we returned the sample to the 300 °C oven for an additional 96 hour period, at the end of which the sample was again analysed. Our suspicions were confirmed; the sample had undergone complete decarboxylation, forming 1, the parent salt PPh_4_^+^Tf_2_N^−^, a species previously having been established to be stable to *ca.* 425 °C (at which point the anion, not the PPh_4_^+^ cation, is observed to decompose).^2^ Significantly, the parallel thermal decarboxylation of benzoic acid to benzene is known.^22^

Taking a cue from the previously discussed aldehyde-to-acid experiment, we independently prepared 5, the *p*-CO_2_H-functionalized PPh_4_^+^ Tf_2_N^−^ salt, which we then thermolyzed at 300 °C in order to further validate our earlier-stated hypothesis that the acid, once formed, undergoes decarboxylation to form 1. Once again, the outcome matched our expectation. Accordingly, it is manifestly clear that if the acid-functionalized 5 underwent decarboxylation in those instances, any other alkyl-functionalized salts which produce it are destined to fall, upon extended thermolysis at high temperatures, into the rather deep thermodynamic well which PPh_4_^+^Tf_2_N^−^ (1) appears to constitute.

In addition to studying the nature of thermo-oxidative transformations of *p*-tolyl salt 3, we likewise prepared and investigated the behaviour of *m*-tolyl congener 6 (Scheme 3). As illustrated, the sequential conversion of 6 first into the corresponding aldehyde, followed by further oxidation to the derivative acid, and ending with decarboxylation of the latter to form 1, was apparent from changes over time to the ^1^H- and ^13^C-NMR of the heated material, as well as from changes in its ESI-MS. Clearly, the nature of the putative, initially-formed benzylic radical or the last-step carboxylic acid group as being in- or not-in-conjugation with the phosphonium centre had no manifest impact on the eventual outcome of the reaction.

**Scheme 3:**
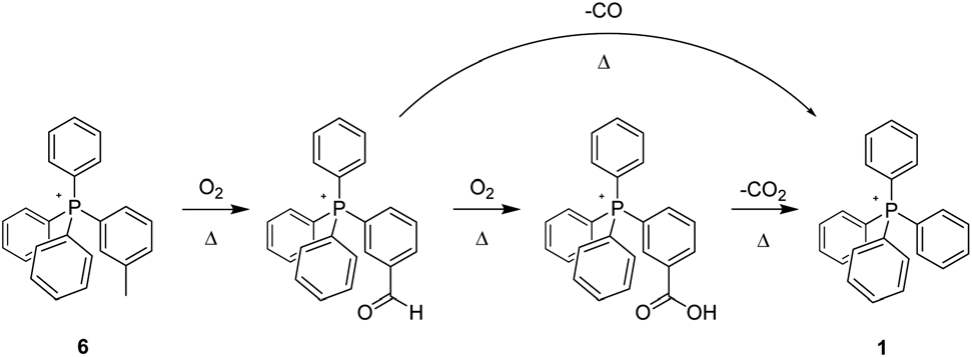
Aerobic, pyrolytic conversion of 6 into 1, and a proposed mechanism for the process.

Noting that each of the forgoing methylated tetraphenylphosphonium-cored cations bears its methyl group on a phenyl ring attached directly to the centre of cationic charge, the question arises as to whether this placement contributes to its relative thermal instability. In order to probe this possibility, we prepared a methylated analogue of 2 (compound 8, Scheme 4), that former parent cation having been previously demonstrated (like 1) to be thermally stable at 300 °C, in air, for months.

**Scheme 4:**
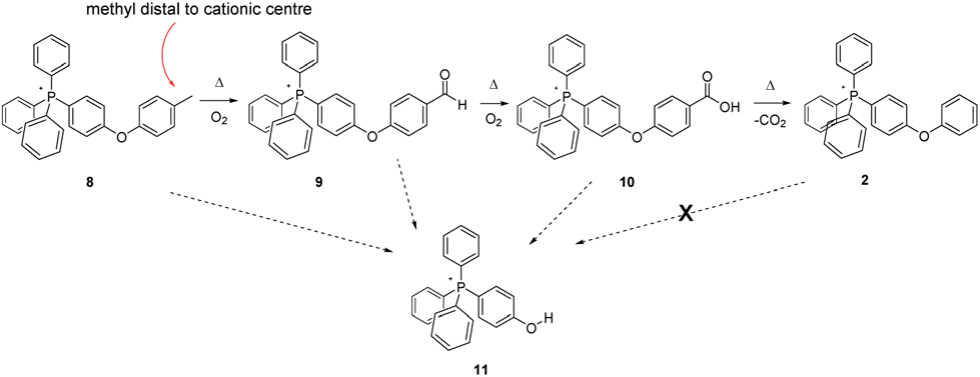
Aerobic, pyrolytic conversion of 8 into 2 and 11, and a proposed mechanism for the process.

As with 3 and 6, samples of 8 were heated to 200 °C, 250 °C, and 300 °C for 96 hours, after which time they were analysed by multi-nuclear NMR and ESI-MS. Insofar as we could determine, there were no changes to the sample subjected to heating at 200 °C. However, a by-now familiar set of changes were observed with the samples of 8 heated to 250 °C and 300 °C. In the former case, a substantial decrease in the integrated intensity (^1^H-NMR) of the tolyl methyl group peak *versus* those from the collective aromatic protons was observed – the parent compound exhibiting a ratio of ≈1 : 7, while that from the sample heated to 250 °C was ≈1 : 11, reflecting a significant diminution of the methyl group content in the sample. Equally significant, the ^1^H-NMR of the sample showed an emerging singlet at 9.94, consistent with the oxidation of the tolyl methyl group into an aldehyde, as observed when 3 and 6 were pyrolyzed. Likewise, a new resonance was observed at 169.27 ppm in the ^13^C-NMR, also consistent with the incipient transformation of the –CH_3_ group into –CHO (9, Scheme 4).

After 96 hours at 300 °C, the ^1^H- and ^13^C-spectra alike indicated the complete absence of the tolyl methyl group. Likewise, the peaks that had indicated its transformation into an aldehyde group in the 250 °C experiment were absent. Present instead was a peak (166.67 ppm) in the ^13^C-NMR consistent with the presence of a –COOH group (10), presumably formed by the oxidation of the foregoing intermediate aldehyde; its occurrence was also confirmed by ESI-MS. Perhaps most significant was a large MS peak indicating the presence of a material having a mass corresponding to that of a phenol-substituted tetraphenylphosphonium ion, *i.e.*11. So, in order to more definitively confirm the presence of 11 in the pyrolyzate, we prepared an authentic sample on which data could be acquired for purposes of comparison. The reference compound – pure 11 – proved to have a highly distinctive, P-coupled doublet in its ^13^C spectrum centred at 104.72 ppm from the P-bonded ring carbon *para* to the phenolic OH; likewise, 11 featured characteristic peaks in the ^1^H-NMR assignable to the ring protons immediately adjacent to the –OH group. The formation of 11 in the present case, when it is not produced by the pyrolysis of 2, indicates that at least under those conditions, the diphenylether linkage in 8 appears to be more labile than the comparable bond in the non-alkylated counterpart 2. Our simplest explanation is that the *para* methyl group of 8 gives rise to an easily-formed benzylic radical, which then leads to fragmentation at the ether bond. We note that radical exchange calculations‡ show that the diphenylether linkage of such a distal benzylic radical is slightly easier to break than that of parent 8, by about 2–3 kcal mol^−1^.

Once satisfied that we had clarified (to a reasonable degree) the thermal fate of simple methyl groups appended to PPh_4_^+^-type cores, we next explored the nature of transformations (if any) that would occur when a methylene group was present in the ion. In this instance, two salts, 12 and 14, were prepared and subjected to the standard thermal treatment.

Per Scheme 5, thermolysis of the ethyl-substituted salt 12 appears to proceed in a fashion closely related to that involved in the tolyl-methyl oxidation. At all temperatures, MS analysis shows the emergence of an unambiguous peak for the same carboxylic-acid functionalized salt (5) as that formed by the pyrolysis of 3, indicating that the –CH_3_ of the ethyl is altogether shed, with the –CH_2_ group being oxidized to –COOH. Even so, it is equally apparent, especially from MS and NMR of the 200 °C experiments, that the reaction proceeds by the formation of a ketone, from which the –CH_3_ group is subsequently cleaved and replaced by –OH, forming the carboxylic acid. We note that oxidation of the neutral counterpart molecule ethylbenzene has also been proposed to begin at the methylene position,^23^ a logical surmise since benzylic radicals are known to be considerably more stable than those on a primary carbon centre.^24^ In the case of the present IL, further confirmation of this pathway was obtained when we independently prepared the putative ketone-functionalized intermediate, 13, and aerobically pyrolyzed it, finding the product of this process to be carboxylic acid 5. This, in turn, upon prolonged heating underwent decarboxylation to form the PPh_4_^+^ cation (1).

**Scheme 5:**
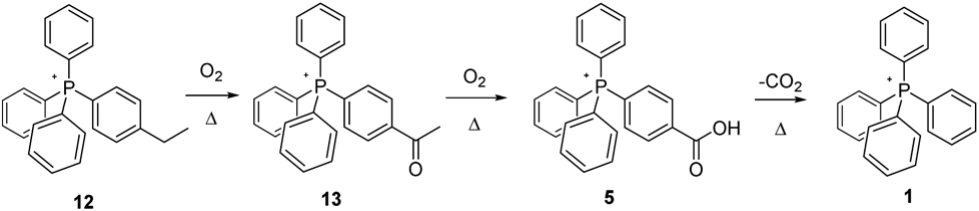
Aerobic, pyrolytic conversion of 12 into 1, and a proposed mechanism for the process.

The outcome of the thermolysis experiments focused on the second type of methylene-bearing cation, 14, were unexpected (Scheme 6). We anticipated the clean conversion of 14 to benzophenone-like 15, largely on the basis of 15 having been previously prepared and established by us to be as thermally stable as the parent cation 1.^5^ To our thinking, the degree of oxidation of 14 would be limited by the presence of two phenyl groups bracketing the aliphatic moiety; then, lacking a means to eliminate CO_2_, the system would be ‘stuck’ at that stage of transformation. That, however, did not prove to be the case. Whereas 15 as previously reported^5^ was prepared from a pre-assembled benzophenone tecton, its formation in the present case would be expected to proceed through an intermediate such as one of those shown in Scheme 6. In that event, it seems plausible to propose that the potential energy surface in the vicinity of the intermediate(s) might have multiple saddle points sufficiently low in energy to be crossed at the high temperatures of the present experiments, leading to more than one product. Significantly, the formation of more than one product is precisely what we observed.

**Scheme 6:**
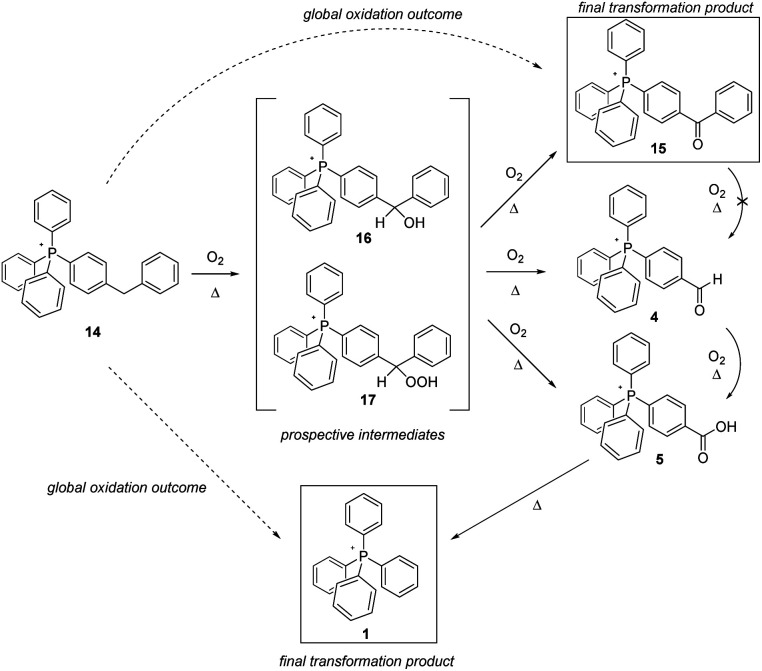
Aerobic, pyrolytic conversion of 14 into 1 and 15, and a proposed mechanism for the process.

Based upon the intensities of select signals in the pyrolysate ^1^H-NMR spectra, within 96 hours at 300 °C, the bulk of 14 was converted to the expected product 15, although acid 5 and parent cation 1 were observed in the product mixture as well. However, while the lower-temperature experiments (200 °C and 250 °C) also showed 15 being formed after 96 hours, an observable amount of 4 was also present, in keeping with it being an intermediate in the formation of 5, which in turn is eventually pyrolyzed to 1. It is notable, however, that based on prior observation, 15 is not further oxidized, nor is its side chain cleaved;^5^15 is not converted into 4. Consequently, it seems apparent that 4 must emerge from one of the proposed intermediates, or another that is not anticipated in Scheme 5.

The wholesale cleavage of an aromatic ring from 14 – made clear by the formation of 4, 5, and eventually 1 during the pyrolysis – was a surprise to us. Even so, on reflection it made clear that whatever intermediate(s) was first formed in the oxidation of 14, it had to be of a character that could lead to ring loss. In the interest of addressing this possibility, we separately prepared prospective intermediate 16, which was then pyrolyzed to ascertain whether this actually resulted in the formation of any of the arene ring-cleavage salts (note: regrettably, we were not able to prepare 17 in order to study its decomposition products).

The heating of 16 in air resulted in the formation of observable quantities of 4 in fewer than 24 hours, whether at 200 °C, 250 °C, or 300 °C. After 72 hours at 300 °C, neither 4 nor 5 were still observed in the NMR, having been fully driven to 1. Insofar as could be ascertained, the latter, along with 15, constituted the final reaction products. In all, this constellation of derivatives of 14 is very much in keeping with that established to emerge from the decomposition of the molecular counterparts (diphenylcarbinol and diphenylmethyl hydroperoxide) of the intermediates proposed in Scheme 6.^25–27^

The methyl- and methylene-appendage experiments concluded, we proceeded to explore the behaviour of the next aliphatic C–H type in the planned evaluation sequence, methine moieties as found within isopropyl- (18) and triphenylmethyl groups (19) (Schemes 7 and 8). In these instances, the study of the behaviour of 18 is of particular note since a high-volume industrial process, the Hock synthesis of phenol and acetone, proceeds through the radical-based thermal reaction of cumene (isopropyl benzene) with O_2_.^28–32^

**Scheme 7:**
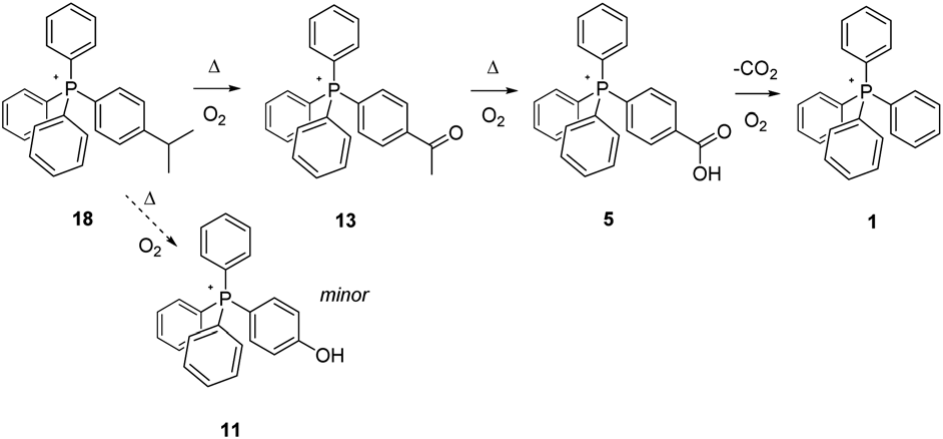
Aerobic, pyrolytic conversion of 18 into 11 and 1, and a proposed mechanism for the process.

**Scheme 8:**
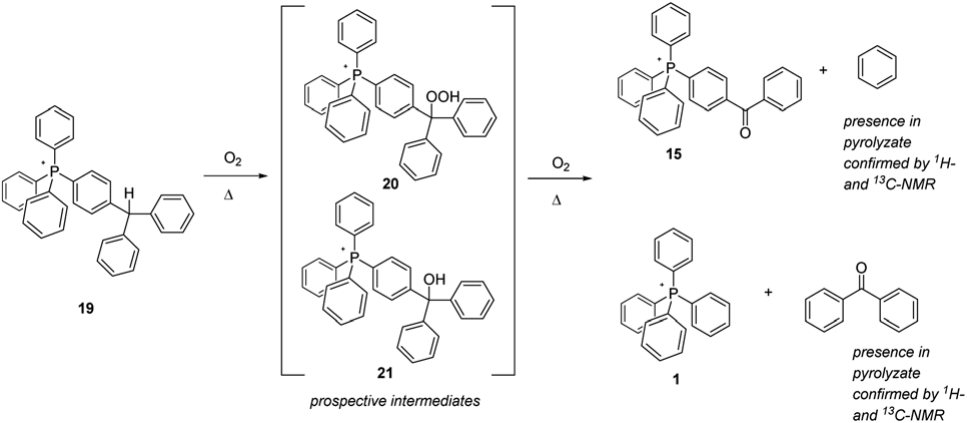
Aerobic, pyrolytic conversion of 19 into 1 and 15, and a proposed mechanism for the process.

In the Hock (cumene hydroperoxide) process, complete scission of the isopropyl group from the aromatic ring is accomplished, resulting in its replacement by a phenolic –OH, and accompanied by the liberation of acetone. However, the second step in the process requires catalysis by acid, and in the present experiments involving the pyrolysis of 18, none is added (our current interest being in outcomes driven by aerobic pyrolysis alone). That being noted, we can report that no phosphonium counterpart to phenol, *i.e.*, 11, is observed by NMR, although a small peak of the appropriate mass for this is observed by ESI-MS, a more sensitive technique.

Over the course of 96 hours, the ^1^H- and ^13^C-NMR spectra of 18 did show the steady disappearance of signals associated with the appended isopropyl group. In the ^1^H-spectrum, these signals were a doublet of intensity six at 1.31 ppm and a heptet of intensity one at 3.08 ppm; the associated signals in the ^13^C-spectrum were observed at 23.04 and 34.10 ppm. As these diminished, a large new singlet gradually grew in and then faded out over time at 2.69 ppm in the ^1^H-spectrum, and a similar signal grew in/and faded out at 26.73 ppm in the ^13^C spectrum. These signals are identical with ones from an authentic reference sample of 13, and are taken as clearly indicative of its formation in significant amounts during pyrolysis. Interestingly, the formation of acetophenone, the non-ionic counterpart of 13, is known to be a side product in the oxidation step Hock process, as is dimethylbenzyl alcohol. In the case of salt 18, only a trace of the phosphonium dimethylbenzyl alcohol counterpart was observed, and it by ESI-MS only. In the final analysis, after 96 hours at 300 °C, 18 was converted to a mixture of 5 and 1, and based upon outcomes already discussed, the 5 would have fully transformed to 1 given additional time; here again, the P-bonded phenyl constituted a firewall that sharply limited the overall degree of thermal decomposition of salt 18.

The final aliphatic-C–H-bearing salt to be studied was 19 (Scheme 8), a species in which a solitary methine-type C–H was flanked only by aromatic rings. We speculated that this compound might prove to be more robust, having its C–H moiety isolated from any other aliphatic components. However, even after only 12 hours at 250 °C, the compound showed substantial degradation; after 96 hours at 250 °C and 300 °C, it was totally decomposed (note: the sample at only 200 °C also exhibited a degree of decomposition after 96 hours). Interestingly, the pyrolytic residue consisted of only two phosphonium ions – 15 and 1 – suggesting that the postulated oxidative intermediate(s) 20 and 21 thermally rearranged by cleavage of an aromatic module from the phosphonium cation. In order to investigate this possibility, a sample of 19 was pyrolyzed at 300 °C overnight, the sample flask being provided with a cold trap to capture volatiles released during the experiment. Having successfully trapped a bolus of colourless liquid as a result, we subjected it to analysis by NMR, and were able to confirm that the liquid consisted of benzene and benzophenone, the exactly expected materials complimentary to the phosphonium ions remaining after pyrolysis.

Having sussed out the nature of the non-volatile thermolysis products, and having proposed mechanisms for their formation, we turned to an evaluation of the volatile products to see if the aforementioned mechanisms remained reasonable in light of the character of these gases. In doing so, we observed three gaseous off-products the presence of which was universal in the IR spectra of samples examined (ESI†), and which could be unambiguously assigned: H_2_O, CO_2_, and CO. Significantly, the comparative off-gas spectra in the 2000–2400 cm^−1^ range of compounds 3–5 (Fig. 3) provide especially valuable insights supportive of the decomposition mechanisms already proposed (*vide supra*). Specifically, the spectrum of 5 – bearing a COOH group – is essentially devoid of CO, and dominated by the loss of CO_2_, as previously suggested. However, the pyrolysis of 4 – incorporating a “pre-made” COH group – generates a large quantity of CO, again consistent with our proposed decomposition mechanism (see Schemes 2 and 3). Here too, however, CO_2_ is also in evidence, consistent with the proposition that the conversion of 4 to 1 can proceed by direct CO elimination or further oxidation to 5, followed by CO_2_ loss. In turn, note that the direct, start-to-finish pyrolysis of 3 shows both CO and CO_2_ generation, again as previously predicted. As a side note, the absorptions between those of CO_2_ and CO in the spectrum of the gaseous products from 3 could not be unambiguously assigned, but may be from a C/N/O unsaturated moiety. Interestingly, these peaks only appeared when pyrolysis temperatures eventually reached 400 °C, beyond which point the Tf_2_N^−^ anion is known to decompose. Given the inertness of the elemental nitrogen which would have been present in the air under which the pyrolyses were conducted, it seems reasonable to posit that these absorptions stem from a material generated by anion decomposition, since the only N-containing element of the salt was the Tf_2_N^−^ anion.

**Fig. 3:**
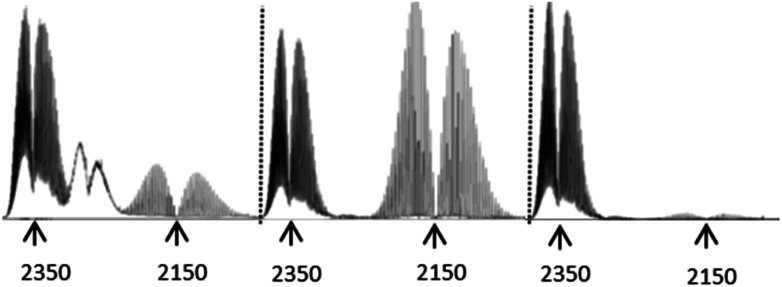
Left to right: Key region of IR spectra of off-gases from 3–5. Note that 4 gives off a considerable amount of CO, consistent with decarbonylation. In turn, 5 gives off considerable CO_2_, but virtually no CO. Pyrolysis of 3 generates observable amounts of both gases. Each of these observations is consistent with the mechanisms proposed for the respective compound's decomposition. The third substance apparent from compound 3 was not conclusively assigned. However, it appears in a range consistent with a cyanate-type moiety. Notably, it only appears after the sample is heated to *T* ≥ 400 °C, at which point the Tf_2_N^−^ anion of the salt – it's only N-containing component – is already known to decompose.

To conclude our study, we thought it worth examining what happens to select representatives of the present salts when pyrolyzed in the absence of air. Specifically, we hoped to acquire insight into whether O_2_ is strictly necessary to “decompose” an initial alkyl group (methyl, for sake of simplicity), and whether – once oxygen is incorporated into an ion structure – ongoing access to O_2_ is needed to further the progression of thermal changes. Accordingly, samples of 3–6 were sealed into glass ampoules under vacuum and heated to 300 °C for 24 hours, this shorter period being utilized in order to increase our chances for observing the (prospective) multitude of intermediate materials we thought might be generated in the reaction. Significantly, pyrolysis of 3 and 6 in the absence of oxygen resulted in no significant changes to the materials as indicated by NMR, and only a few new, very small peaks in the ESI-MS.

In contrast, samples of 4 and 5 – both of which began with oxygen as a compositional element – continued to show significant changes when heated anaerobically; recall that each of these had been prepared in a pure form to provide reference material for use in characterizing proposed products/intermediates being generated by the aerobic pyrolysis of 3. In the case of aldehyde-bearing 4, only a trace of that parent material remained after 24 hours. Most (based upon ESI-MS and NMR) had been converted into 1. We find this interesting, since (in the absence of an exogenous source of oxygen), the further oxidation of 4 into 5 that needs to occur to enable decarboxylation of the latter into 1 should not have been possible. However, we again note that another mechanism for the conversion of it into 1 is possible: decarbonylation. The latter has been reported previously as a reaction to which benzaldehyde is subject under high-temperature conditions.^33,34^

In the anaerobic thermolysis of pure 5, major product was clearly the expected decarboxylation product 1. However, an unexpected peak was also observed at *m*/*z* = 395.25, equal to the mass of 5 (*m*/*z* = 383.17) plus an additional carbon atom. We have been unable to further clarify its structure. Whatever it may be, we speculate that this material was possibly formed in the high-energy environment of the ESI-MS experiment.

Collectively, the foregoing data provide a reasonable basis to conclude that the thermally-induced cleavage of aliphatic C–H bonds in tetraarylphosphonium cations, when done in the presence of oxygen, leads to the loss or wholesale transformation of the aliphatic component, but almost exclusively without damage to the central, tetraphenylphosphonium core; the Tf_2_N^−^ salt of 1 remains, it clearly being highly rugged with respect to thermolysis. Indeed, we have been able to routinely recover pure 1 by simple recrystallization of the post-pyrolysis residues. In addition, these results may also be of utility with respect to new classes of high-performance polymers (based upon tetraarylphosphonium ions) such as those under development by Smith and co-workers, materials that are envisioned for use in the chemically and thermally demanding conditions found in fuel cells.^35–37^ We also anticipate that thermally stable, perarylphosphonium salts may prove to be interesting and thermally stable property modifiers if added to commercial high-performance polymers such as PEEK and PES.^38,39^ Not only might their addition modify properties such as plasticity, but they could prove to be effective flame retardants since many high-valent phosphorous compounds are used for that purpose.

## Conflicts of interest

There are no conflicts to declare.

## Supplementary Material

RA-010-D0RA03220D-s001
