# Correction: Ionic liquids of superior thermal stability. Validation of PPh_4_^+^ as an organic cation of impressive thermodynamic durability

**DOI:** 10.1039/d3ra90063k

**Published:** 2023-07-11

**Authors:** Mohammad Soltani, Jimmie L. McGeehee, Alexandra C. Stenson, Richard A. O'Brien, Edward R. Duranty, E. Alan Salter, Andrzej Wierzbicki, T. Grant Glover, James H. Davis

**Affiliations:** a Department of Chemistry, University of South Alabama Mobile Alabama 36688 USA jdavis@southalabama.edu

## Abstract

Correction for ‘Ionic liquids of superior thermal stability. Validation of PPh_4_^+^ as an organic cation of impressive thermodynamic durability’ by Mohammad Soltani *et al.*, *RSC Adv.*, 2020, **10**, 20521–20528. https://doi.org/10.1039/D0RA03220D

The author regrets that the funding information was incorrectly shown in the acknowledgements section of the original manuscript. The corrected funding acknowledgement is as shown below.

The Royal Society of Chemistry apologises for these errors and any consequent inconvenience to authors and readers.

## Supplementary Material

