# Supplementary material for: Ionic liquids of superior thermal stability. Validation of PPh4+ as an organic cation of impressive thermodynamic durability
Source: RSC Adv. 2020 May 29;10(35):20521–8. doi: 10.1039/d0ra03220d (PMC9054240; doi:10.1039/d0ra03220d)
Supplement: RA-010-D0RA03220D-s001 [file RA-010-D0RA03220D-s001.pdf]

Electronic Supplementary Material (ESI) for RSC Advances.
This journal is © The Royal Society of Chemistry 2020

Ionic liquids of superior thermal stability. Validation of PPh_4^+ as an organic cation of impressive thermodynamic durability

Mohammad Soltani, Jimmie L. McGeehee, Alexandra C. Stenson, Richard A. O'Brien, Edward R. Duranty, E. Alan Salter, Andrzej Wierzbicki, T. Grant Glover, and James H. Davis, Jr.*

Department of Chemistry
University of South Alabama

Supporting Information

General Information:

Commercial reagents were obtained from Aldrich Chemical and Oakwood Chemicals and used without further purification. ^{1}H, ^{19}F, and ^{13}C NMR were recorded on a 500 MHz JEOL spectrometer using $CDCl_3$ as a solvent at room temperature. All chemical shifts for ^{1}H and ^{13}C NMR were reported downfield using tetramethylsilane (TMS, at d ¼ 0.00 ppm).

General procedure for the synthesis of (4-methylphenyl)triphenylphosphonium (d_{15}) bromide

Method A:

In a 50 ml heavy wall pressure vessel with an internal thread (Teflon cap) containing a stirbar, 4-bromotoluene (1.0 equiv), triphenylphosphine (d_{15}) (1.0 equiv), $NiBr_2$ (5.0 mol%) and ethylene glycol (15 ml) were added under nitrogen atmosphere. The reaction mixture was then stirred at 180 °C for 8 hours. The mixture was then cooled to room temperature, and aqueous NaBr added to dissolve the Ni salts and ethylene glycol. Dichloromethane was added to extract the desired phosphonium salt from the milieu. After two more extractions, the combined organic extracts were dried over anhydrous Na_2SO_4' solvents were removed under reduced pressure, and pure (4-methylphenyl)triphenylphosphonium bromide was isolated as a white solid. The same procedure was utilized for the non-deuterated triphenylphosphine derivatives.

Method B:

In a 50 ml heavy wall pressure vessels with an internal thread (Teflon cap) containing a stirbar, 4-bromotoluene (1.0 equiv), triphenylphosphine (d_{15}) (1.0 equiv), $Pd(OAc)_2$ (1.5 mol%) and xylene (15 ml) were added under nitrogen atmosphere and reaction mixture stirred at 145 °C for 4 hours. The desired product precipitated as a white solid during the course of the reaction. The reaction mixture was cooled to the room temperature, filtered and washed with xylene and diethyl ether and pure product was isolated as a white solid. The same procedure is equally effective when using non-deuterated triphenylphosphine.

General procedure for the synthesis of (4-methylphenyl)triphenylphosphonium (d_{15}) bistriflimide

The bistriflimide salt was synthesized by an ion exchange of potassium bistriflimide (1.0 equiv) and phosphonium salt (1.0 equiv) in water for 15 min at room temperature. The reaction mixture was then extracted three times with dichloromethane. The combined organic extracts were dried

over anhydrous Na_2SO_4, solvents were removed under reduced pressure, and pure (4-methylphenyl)triphenylphosphonium bistriflimide was isolated as a white solid.

General procedure for the synthesis of 4-Bromophenyl diphenylmethane

In an oven-dried 100 mL flask, a solution of 4-bromobenzophenone (20 mmol, 1.0 equv) in THF (50 mL) was prepared. Phenylmagnesium bromide solution (Aldrich; 3.0 M in Et_2O, 17 ml, 2.5 equiv) was added dropwise at 0 °C under nitrogen atmosphere and the resulting mixture was then stirred at 60 °C overnight. The reaction was then quenched with a saturated aqueous solution of NH_4Cl and extracted three times with dichloromethane. The organic extracts were combined, dried over Na_2SO_4, and concentrated under reduced pressure. The residue was then purified by flash chromatography on silica gel (hexane) to give 4-Bromophenyl diphenylmethanol in 80% yield as colorless viscous liquid. The 4-bromophenyl diphenylmethanol (12 mmol, 1.0 equv) was then transferred into an oven-dried 100 mL flask equipped with a stirring bar. $Ga(OTf)_3$ (5.0 mol%), and isopropanol (60 mmol, 5.0 equv) in $PhCF_3$ (20 mL) was added. The resulting mixture was stirred at 110 °C for 1 h. The mixture was then cooled to room temperature, diluted with Et_2O, filtered and concentrated under reduced pressure. The residue was purified by flash chromatography on silica gel (hexane) to give the 4-bromophenyl diphenylmethane as a white pure product in 85% yield.

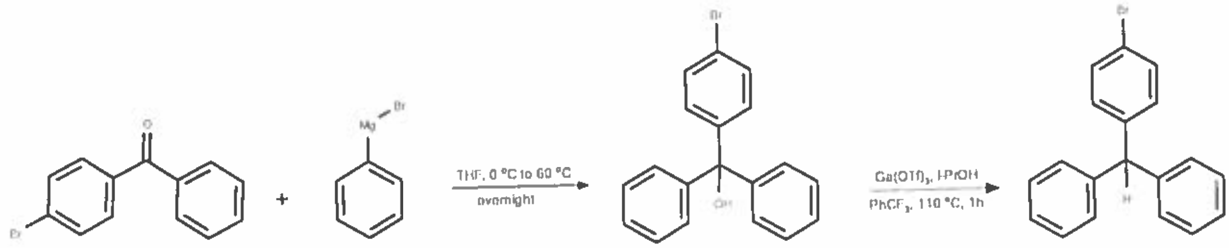

Compound 3 Pre- and Post-heating NMR Spectra

Temperature of Post-heating samples noted in upper left corner of each spectrum

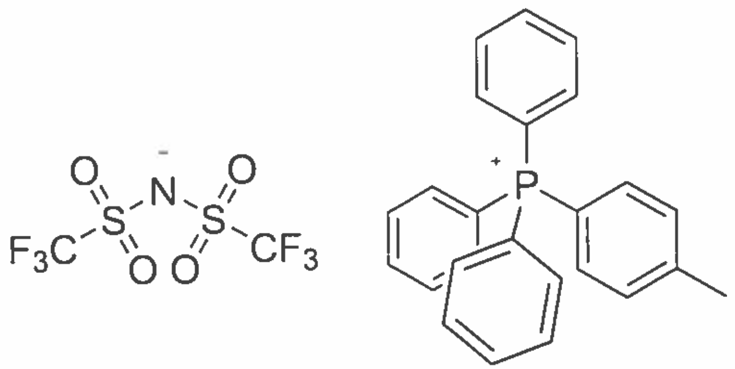

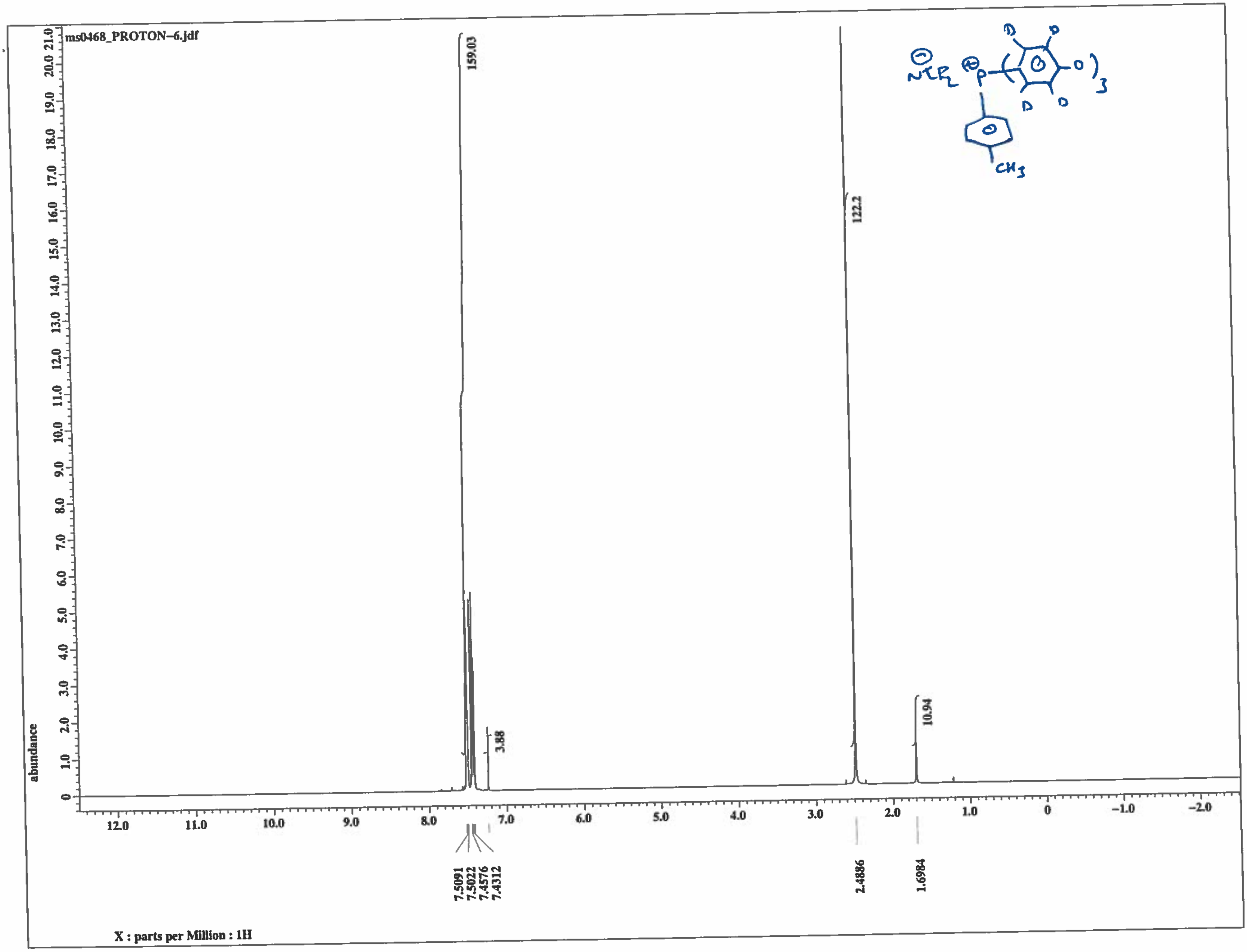
ms0468_PROTON-6.jdf
abundance
X : parts per Million : 1H
159.03
3.88
122.2
10.94
7.5091
7.5022
7.4576
7.4312
2.4886
1.6984

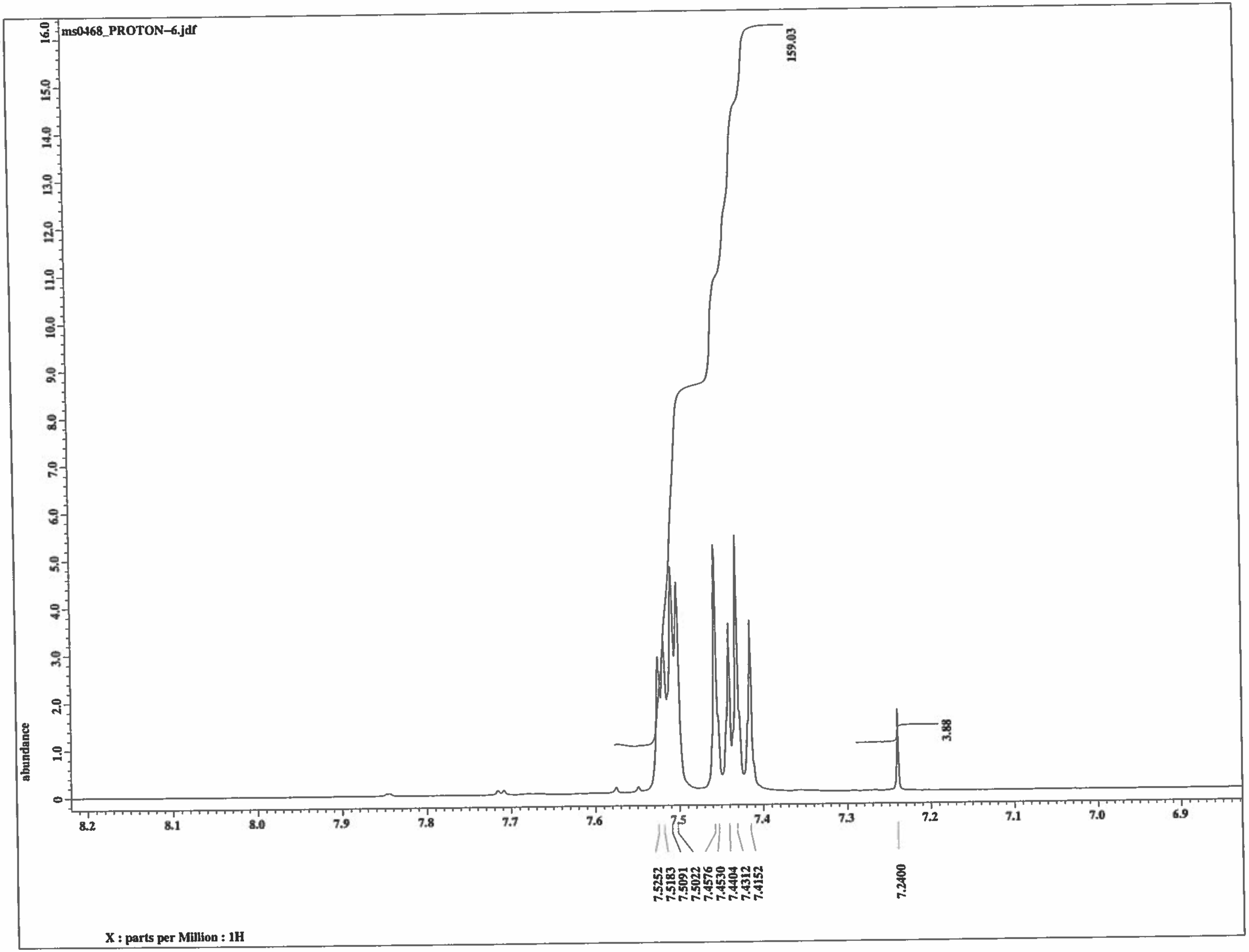
ms0468_PROTON-6.jdf
abundance
0
1.0
2.0
3.0
4.0
5.0
6.0
7.0
8.0
9.0
10.0
11.0
12.0
13.0
14.0
15.0
16.0
159.03
3.88
8.2
8.1
8.0
7.9
7.8
7.7
7.6
7.5
7.4
7.3
7.2
7.1
7.0
6.9
7.5252
7.5183
7.5091
7.5022
7.4576
7.4530
7.4404
7.4312
7.4152
7.2400
X : parts per Million : 1H

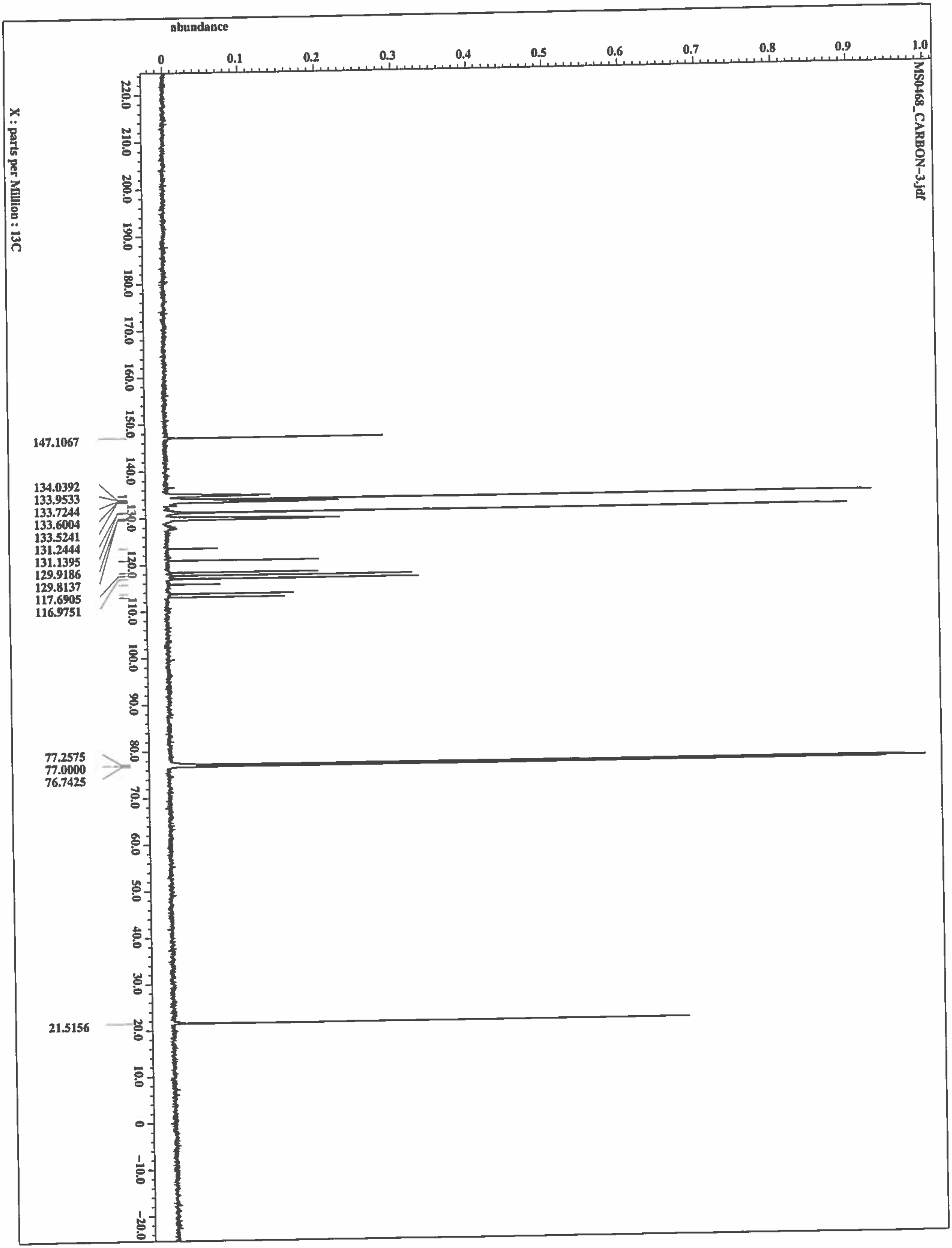

abundance
X : parts per Million : 13C
MS0468_CARBON-3.jdf
147.1067
134.0392
133.9533
133.7244
133.6004
133.5241
131.2444
131.1395
129.9186
129.8137
117.6905
116.9751
77.2575
77.0000
76.7425
21.5156

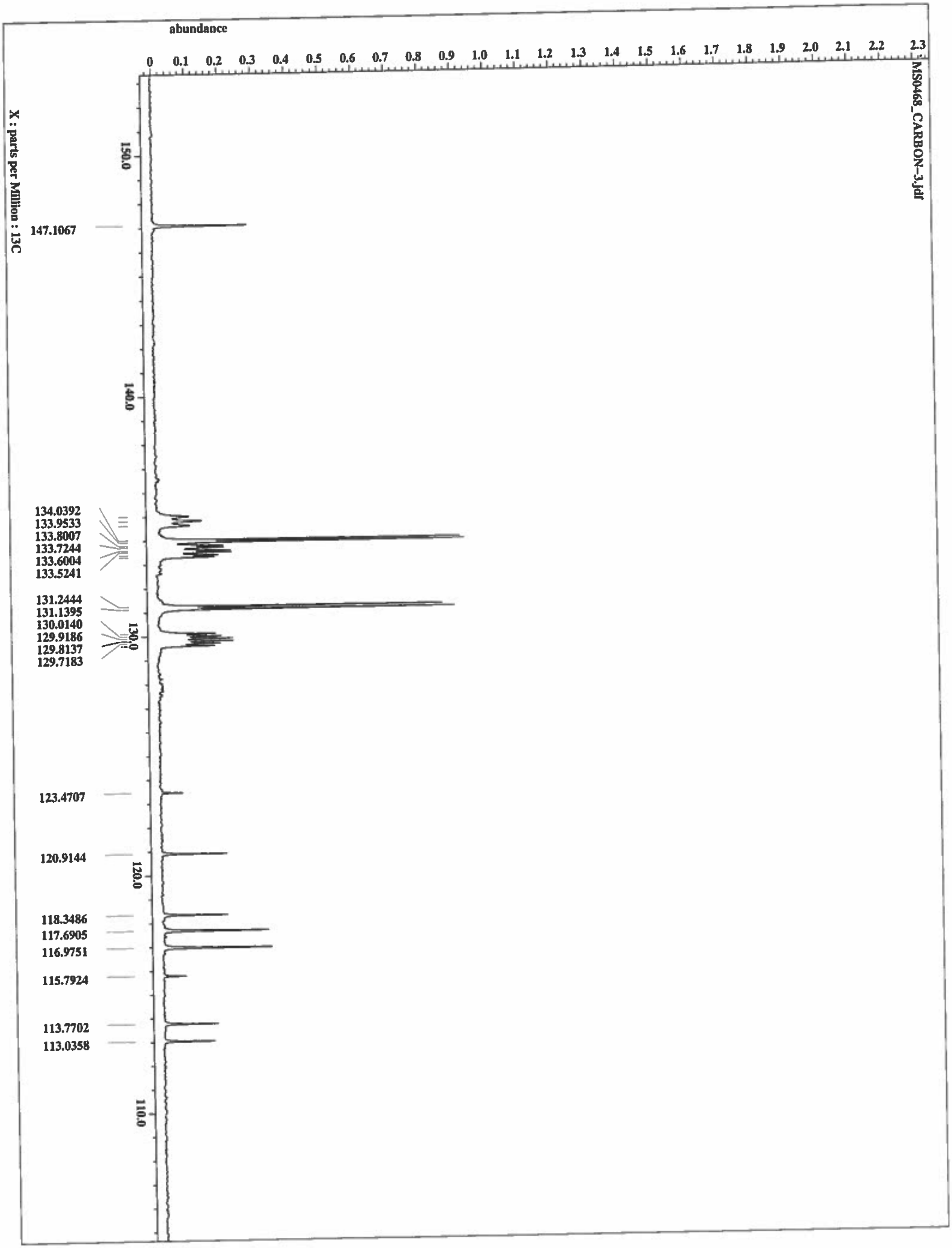

abundance
0 0.1 0.2 0.3 0.4 0.5 0.6 0.7 0.8 0.9 1.0 1.1 1.2 1.3 1.4 1.5 1.6 1.7 1.8 1.9 2.0 2.1 2.2 2.3
MS0468_CARBON-3.jdf
X : parts per Million : 13C
150.0
140.0
130.0
120.0
110.0
147.1067
134.0392
133.9533
133.8007
133.7244
133.6004
133.5241
131.2444
131.1395
130.0140
129.9186
129.8137
129.7183
123.4707
120.9144
118.3486
117.6905
116.9751
115.7924
113.7702
113.0358

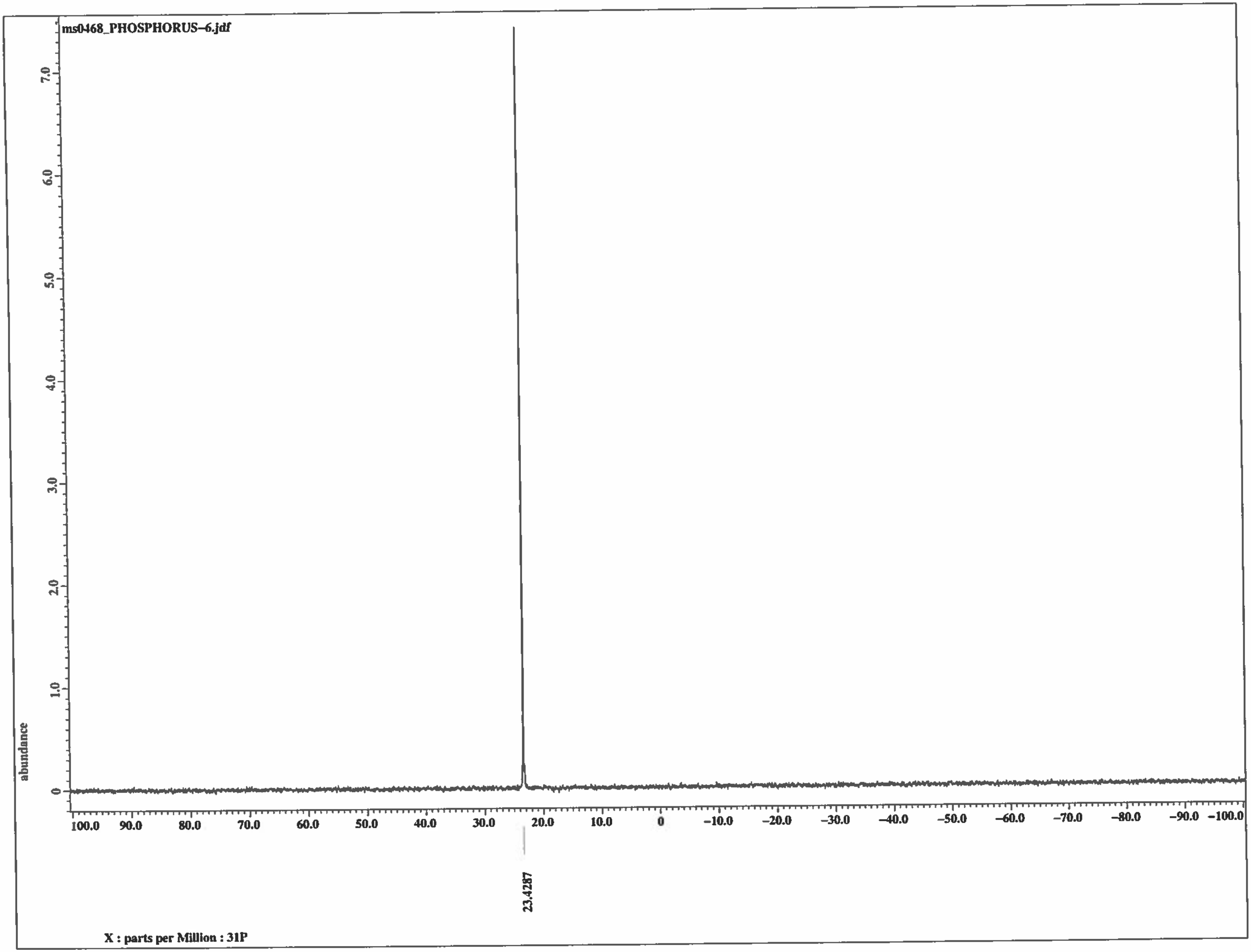

ms0468_PHOSPHORUS–6.jdf
abundance
0
1.0
2.0
3.0
4.0
5.0
6.0
7.0
100.0 90.0 80.0 70.0 60.0 50.0 40.0 30.0 20.0 10.0 0 –10.0 –20.0 –30.0 –40.0 –50.0 –60.0 –70.0 –80.0 –90.0 –100.0
23.4287
X : parts per Million : 31P

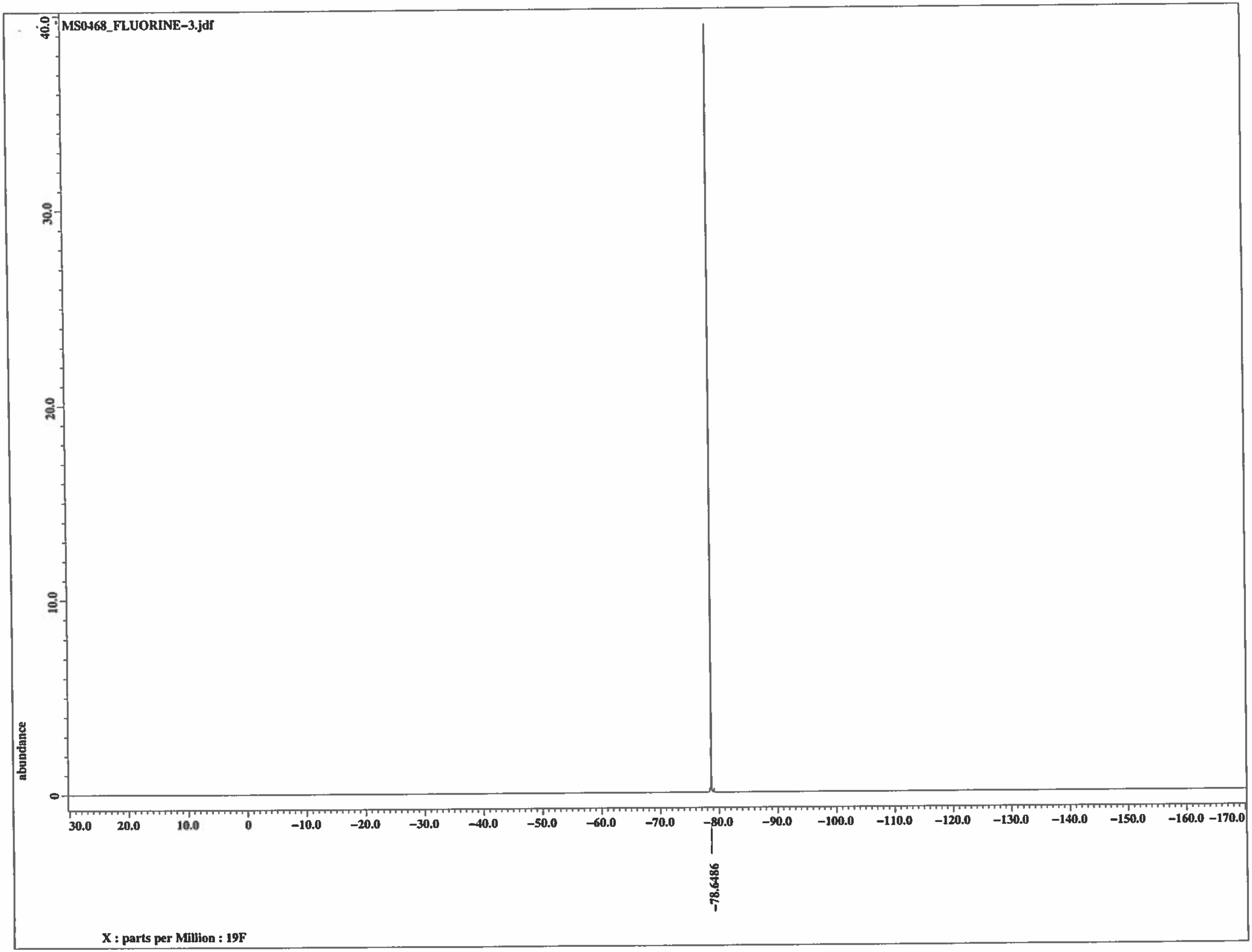
MS0468_FLUORINE-3.jdf
abundance
40.0
30.0
20.0
10.0
0
30.0
20.0
10.0
0
−10.0
−20.0
−30.0
−40.0
−50.0
−60.0
−70.0
−80.0
−90.0
−100.0
−110.0
−120.0
−130.0
−140.0
−150.0
−160.0
−170.0
−78.6486
X : parts per Million : 19F

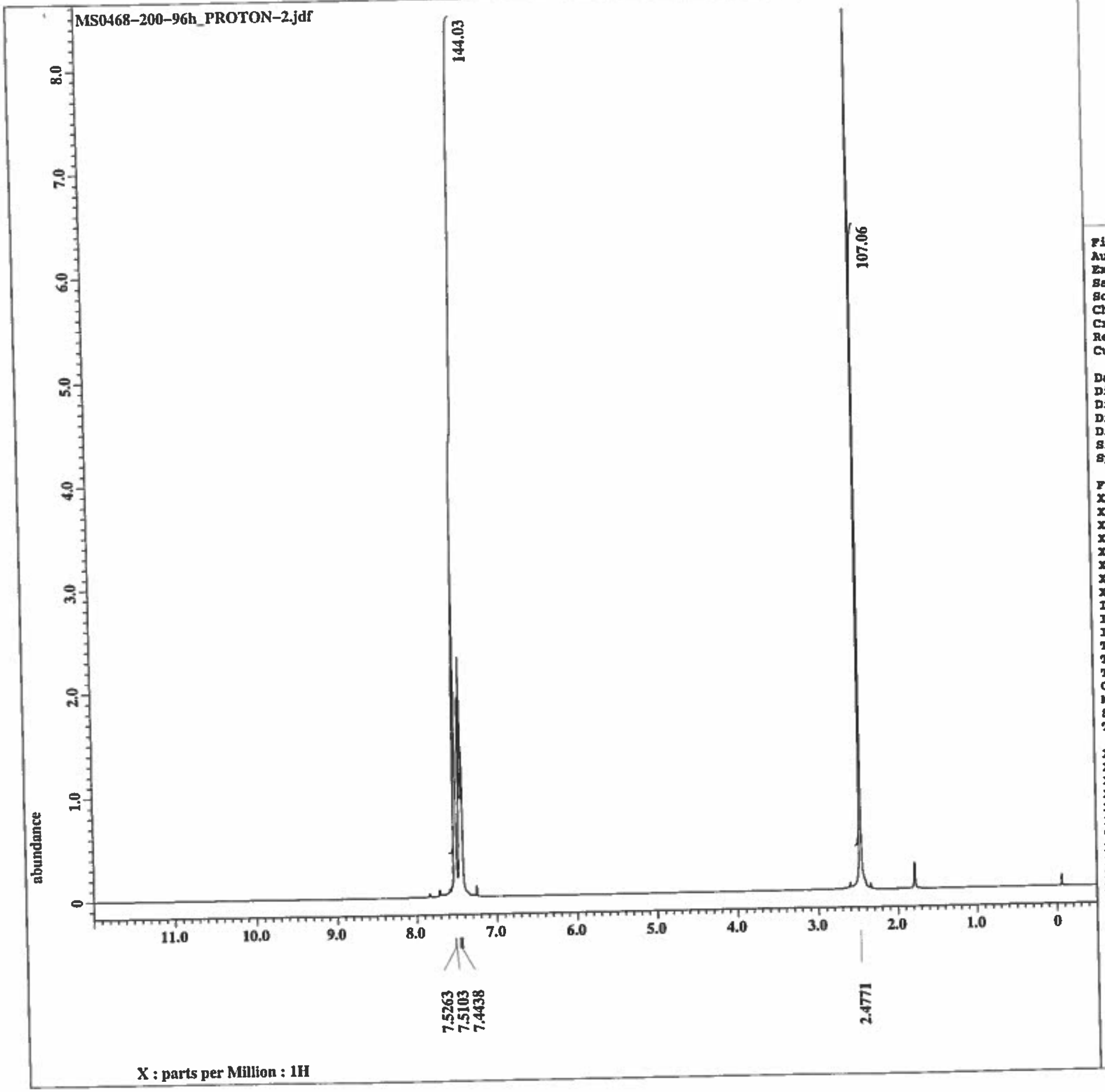

Filename	= MS0468-200-96h_PROTON
Author	= Jim Davis
Experiment	= single_pulse.ex2
Sample_id	= MS0468-200-96h
Solvent	= CHLOROFORM-D
Changer_sample	= 17
Creation_time	= 12-JUN-2018 16:07:31
Revision_time	= 12-JUN-2018 15:41:01
Current_time	= 12-JUN-2018 15:41:02
Data_format	= 1D COMPLEX
Dim_size	= 13107
Dim_title	= 1H
Dim_units	= [ppm]
Dimensions	= X
Site	= ECA 500
Spectrometer	= JNM-ECA500
Field_strength	= 11.7473579[T] (500[MH
X_acq_duration	= 1.74587904[s]
X_domain	= 1H
X_freq	= 500.15991521[MHz]
X_offset	= 5.0[ppm]
X_points	= 16384
X_prescans	= 1
X_resolution	= 0.57277737[Hz]
X_sweep	= 9.38438438[kHz]
Irr_domain	= 1H
Irr_freq	= 500.15991521[MHz]
Irr_offset	= 5.0[ppm]
Tri_domain	= 1H
Tri_freq	= 500.15991521[MHz]
Tri_offset	= 5.0[ppm]
Clipped	= FALSE
Mod_return	= 1
Scans	= 16
Total_scans	= 16
X_90_width	= 12.4[us]
X_acq_time	= 1.74587904[s]
X_angle	= 45[deg]
X_atn	= 4[dB]
X_pulse	= 6.2[us]
Irr_mode	= Off
Tri_mode	= Off
Dante_presat	= FALSE
Initial_wait	= 1[s]
Recvr_gain	= 32
Relaxation_delay	= 4[s]
Repetition_time	= 5.74587904[s]
Temp_get	= 22.1[dC]

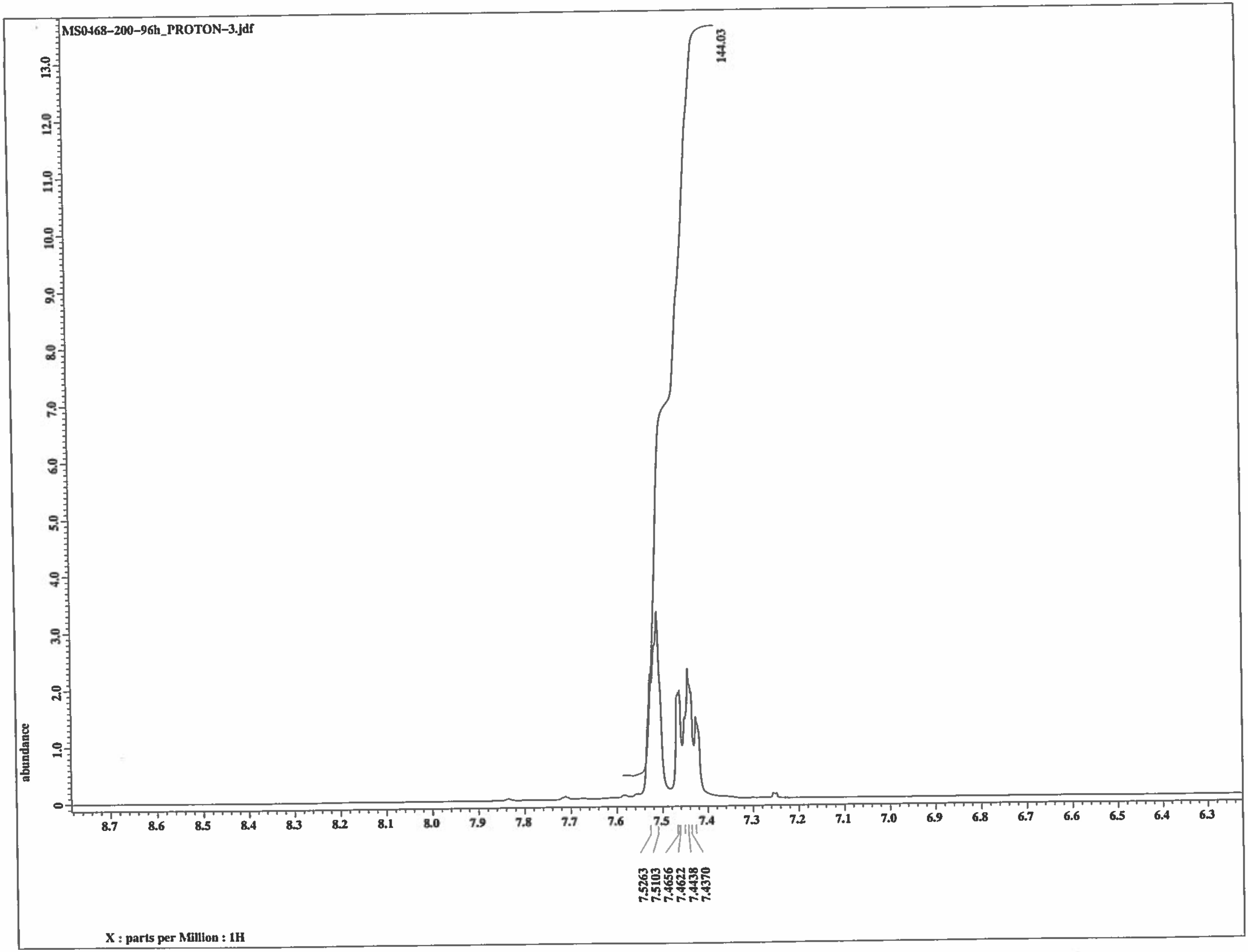

MS0468-200-96h_PROTON-3.jdf
abundance
0
1.0
2.0
3.0
4.0
5.0
6.0
7.0
8.0
9.0
10.0
11.0
12.0
13.0
144.03
8.7
8.6
8.5
8.4
8.3
8.2
8.1
8.0
7.9
7.8
7.7
7.6
7.5
7.4
7.3
7.2
7.1
7.0
6.9
6.8
6.7
6.6
6.5
6.4
6.3
7.5263
7.5103
7.4656
7.4622
7.4438
7.4370
X : parts per Million : 1H

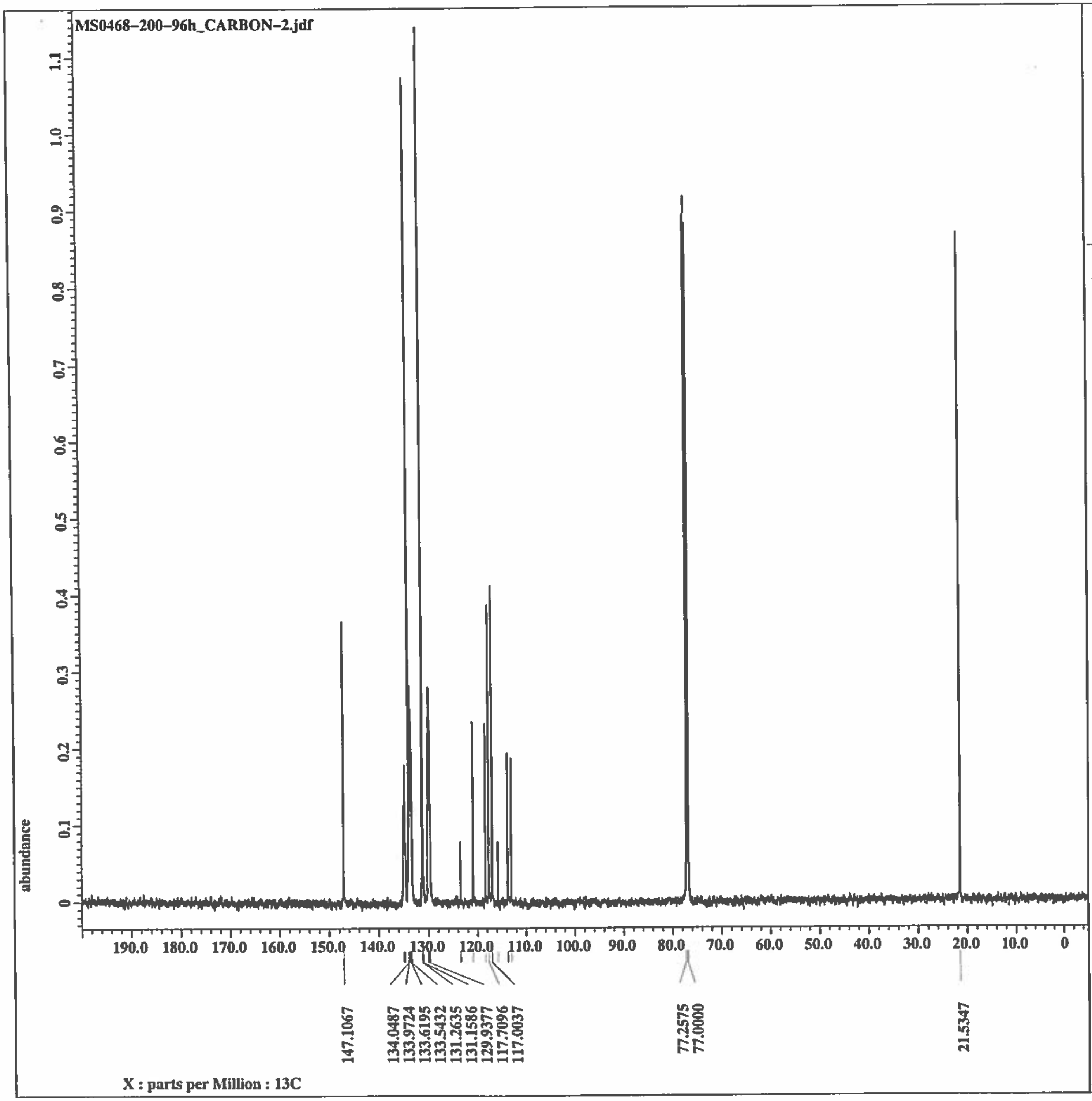


```
Filename          = MS0468-200-96h_CARBON
Author            = Jim Davis
Experiment        = single_pulse_dec
Sample_id         = MS0468-200-96h
Solvent           = CHLOROFORM-D
Changer_sample    = 17
Creation_time     = 12-JUN-2018 16:28:42
Revision_time     = 12-JUN-2018 16:02:13
Current_time      = 12-JUN-2018 16:02:14

Data_format       = 1D COMPLEX
Dim_size          = 26214
Dim_title         = 13C
Dim_units         = [ppm]
Dimensions        = X
Site              = ECA 500
Spectrometer      = JNM-ECA500

Field_strength    = 11.7473579[T] (500[MH
X_acq_duration    = 0.83361792[s]
X_domain          = 13C
X_freq            = 125.76529768[MHz]
X_offset          = 100[ppm]
X_points          = 32768
X_prescans        = 4
X_resolution      = 1.19959034[Hz]
X_sweep           = 39.3081761[kHz]
Irr_domain        = 1H
Irr_freq          = 500.15991521[MHz]
Irr_offset        = 5.0[ppm]
Clipped           = FALSE
Mod_return        = 1
Scans             = 400
Total_scans       = 400

X_90_width        = 13.2[us]
X_acq_time        = 0.83361792[s]
X_angle           = 30[deg]
X_atn             = 6[dB]
X_pulse           = 4.4[us]
Irr_atn_dec       = 20.7[dB]
Irr_atn_noe       = 20.7[dB]
Irr_noise         = WALTZ
Decoupling        = TRUE
Initial_wait      = 1[s]
Noe               = TRUE
Noe_time          = 2[s]
Recvr_gain        = 60
Relaxation_delay  = 2[s]
Repetition_time   = 2.83361792[s]
Temp_get          = 22.6[dC]
```

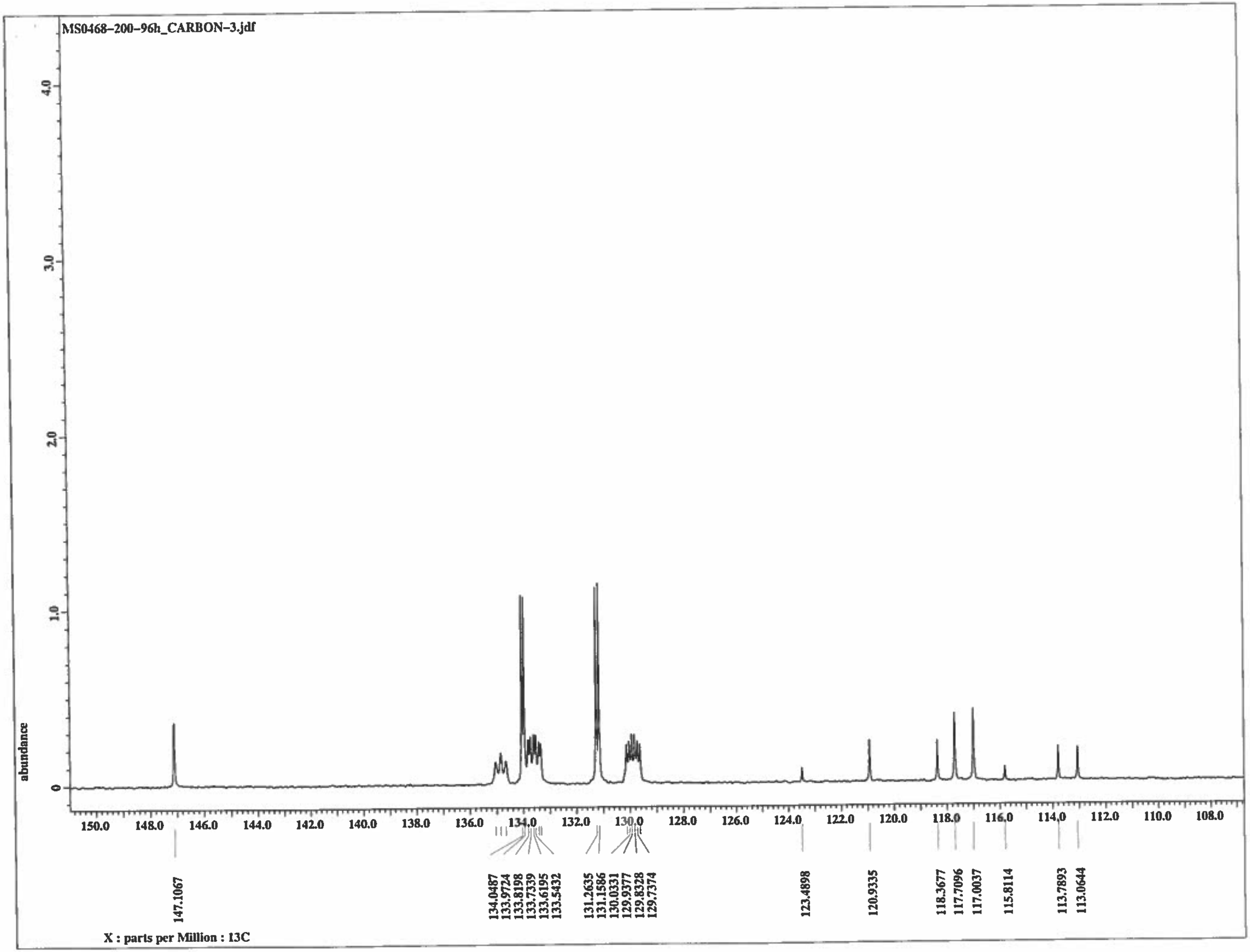
MS0468-200-96h_CARBON-3.jdf
abundance
0
1.0
2.0
3.0
4.0
150.0
148.0
146.0
144.0
142.0
140.0
138.0
136.0
134.0
132.0
130.0
128.0
126.0
124.0
122.0
120.0
118.0
116.0
114.0
112.0
110.0
108.0
147.1067
134.0487
133.9724
133.8198
133.7339
133.6195
133.5432
131.2635
131.1586
130.0331
129.9377
129.8328
129.7374
123.4898
120.9335
118.3677
117.7096
117.0037
115.8114
113.7893
113.0644
X : parts per Million : 13C

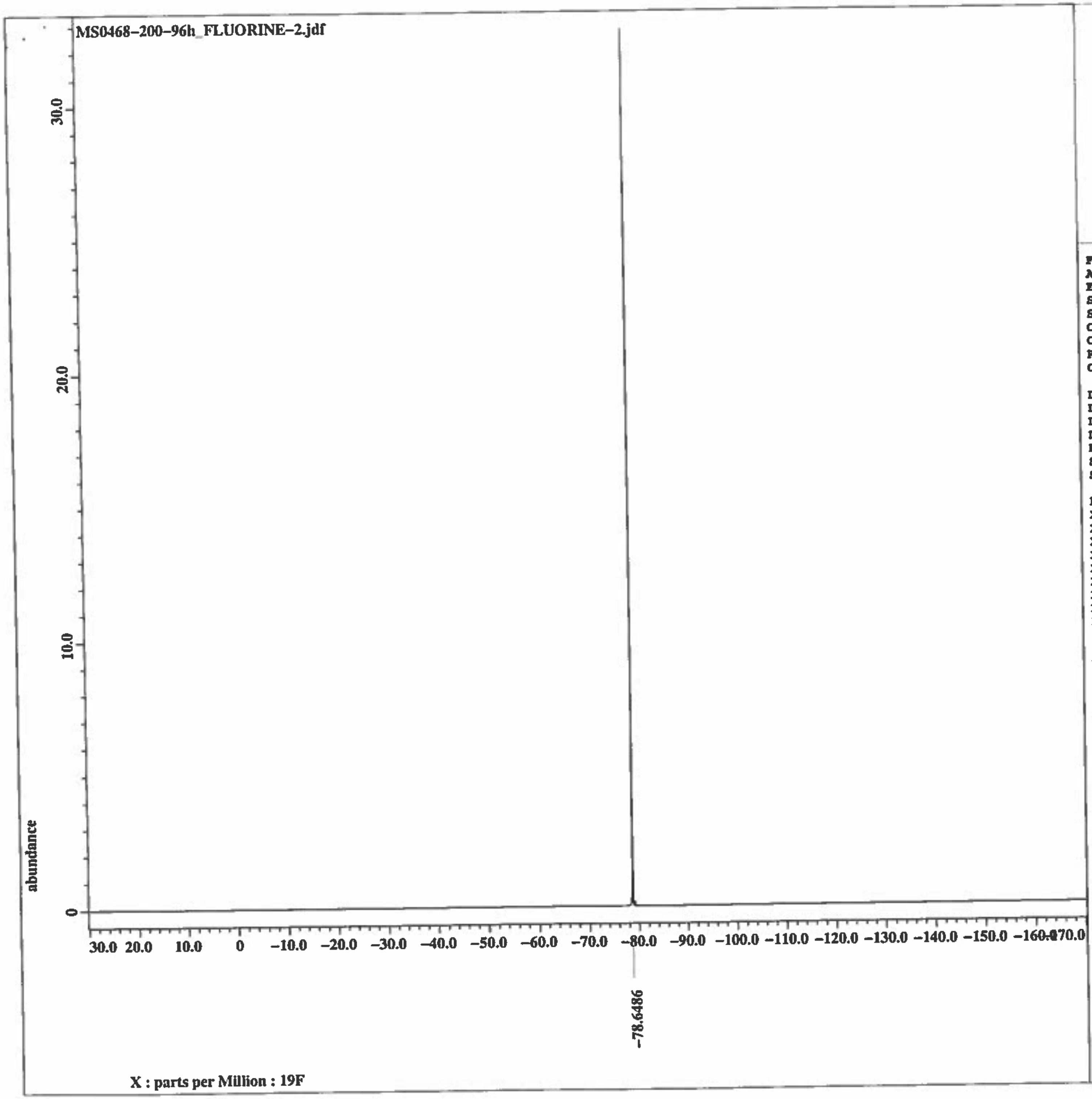

Filename	= MS0468-200-96h_FLUORI
Author	= Jim Davis
Experiment	= single_pulse.ex2
Sample_id	= MS0468-200-96h
Solvent	= CHLOROFORM-D
Changer_sample	= 17
Creation_time	= 12-JUN-2018 16:31:54
Revision_time	= 12-JUN-2018 16:05:26
Current_time	= 12-JUN-2018 16:05:26
Data_format	= 1D COMPLEX
Dim_size	= 52428
Dim_title	= 19F
Dim_units	= [ppm]
Dimensions	= X
Site	= ECA 500
Spectrometer	= JNM-ECA500
Field_strength	= 11.7473579[T] (500[MH
X_acq_duration	= 0.55574528[s]
X_domain	= 19F
X_freq	= 470.62046084[MHz]
X_offset	= -70[ppm]
X_points	= 65536
X_prescans	= 1
X_resolution	= 1.7993855[Hz]
X_sweep	= 117.9245283[kHz]
Irr_domain	= 19F
Irr_freq	= 470.62046084[MHz]
Irr_offset	= 5[ppm]
Tri_domain	= 19F
Tri_freq	= 470.62046084[MHz]
Tri_offset	= 5[ppm]
Clipped	= FALSE
Mod_return	= 1
Scans	= 16
Total_scans	= 16
X_90_width	= 13.1[us]
X_acq_time	= 0.55574528[s]
X_angle	= 45[deg]
X_atn	= 2.5[dB]
X_pulse	= 6.55[us]
Irr_mode	= Off
Tri_mode	= Off
Dante_presat	= FALSE
Initial_wait	= 1[s]
Recvr_gain	= 34
Relaxation_delay	= 4[s]
Repetition_time	= 4.55574528[s]
Temp_get	= 22.3[dC]

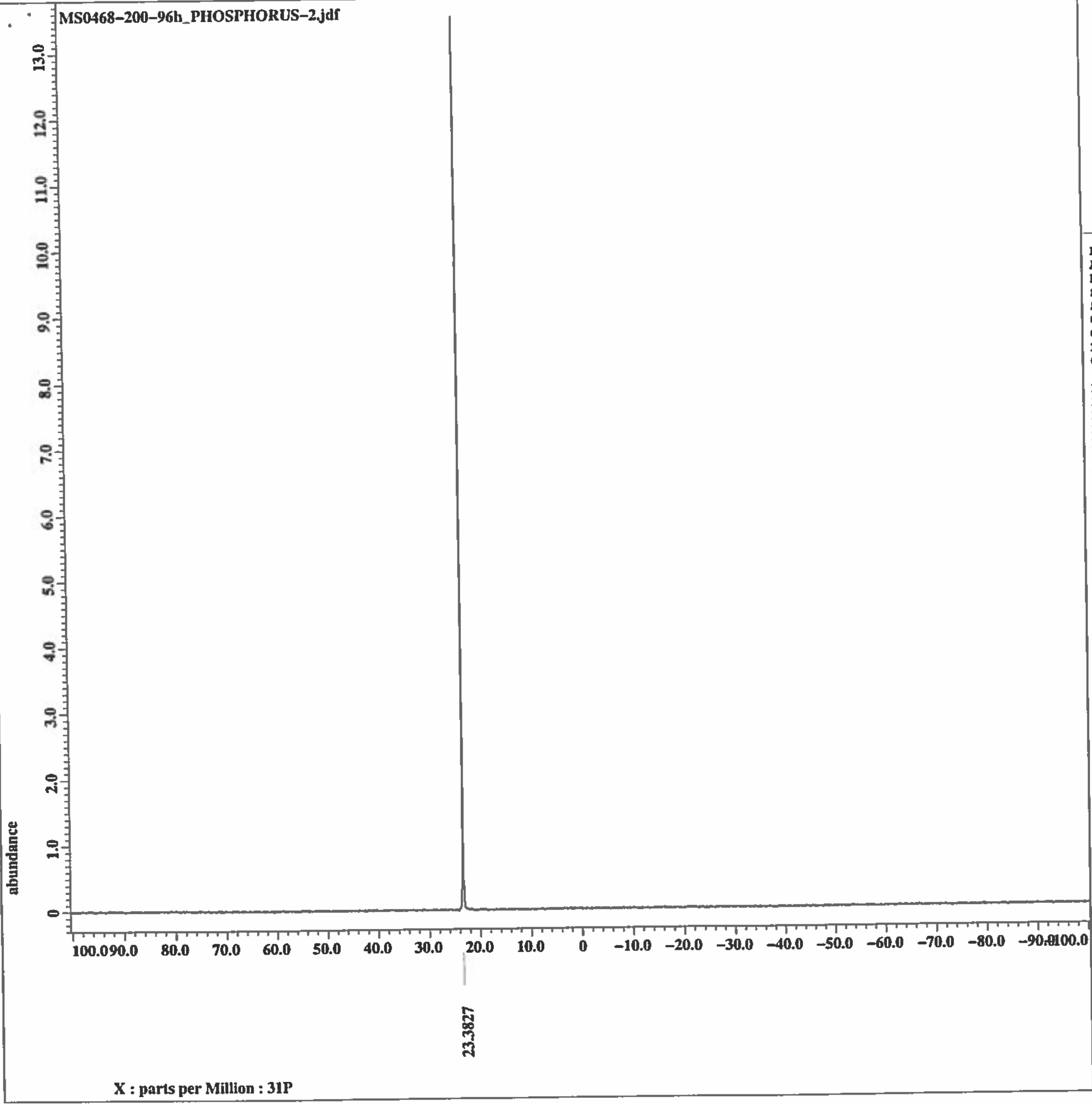

Filename	= MS0468-200-96h_PHOSPH
Author	= Jim Davis
Experiment	= single_pulse_dec
Sample_id	= MS0468-200-96h
Solvent	= CHLOROFORM-D
Changer_sample	= 17
Creation_time	= 12-JUN-2018 16:36:48
Revision_time	= 12-JUN-2018 16:10:20
Current_time	= 12-JUN-2018 16:10:20
Data_format	= 1D COMPLEX
Dim_size	= 26214
Dim_title	= 31P
Dim_units	= [ppm]
Dimensions	= X
Site	= ECA 500
Spectrometer	= JNM-ECA500
Field_strength	= 11.7473579[T] (500[MH
X_acq_duration	= 0.64487424[s]
X_domain	= 31P
X_freq	= 202.46831075[MHz]
X_offset	= 0[ppm]
X_points	= 32768
X_prescans	= 4
X_resolution	= 1.55068995[Hz]
X_sweep	= 50.81300813[kHz]
Irr_domain	= 1H
Irr_freq	= 500.15991521[MHz]
Irr_offset	= 5.0[ppm]
Clipped	= FALSE
Mod_return	= 1
Scans	= 50
Total_scans	= 50
X_90_width	= 14.687[us]
X_acq_time	= 0.64487424[s]
X_angle	= 30[deg]
X_atn	= 5[dB]
X_pulse	= 4.89566667[us]
Irr_atn_dec	= 20.7[dB]
Irr_atn_noe	= 20.7[dB]
Irr_noise	= WALTZ
Decoupling	= TRUE
Initial_wait	= 1[s]
Noe	= TRUE
Noe_time	= 2[s]
Recvr_gain	= 58
Relaxation_delay	= 2[s]
Repetition_time	= 2.64487424[s]
Temp_get	= 22.6[dC]

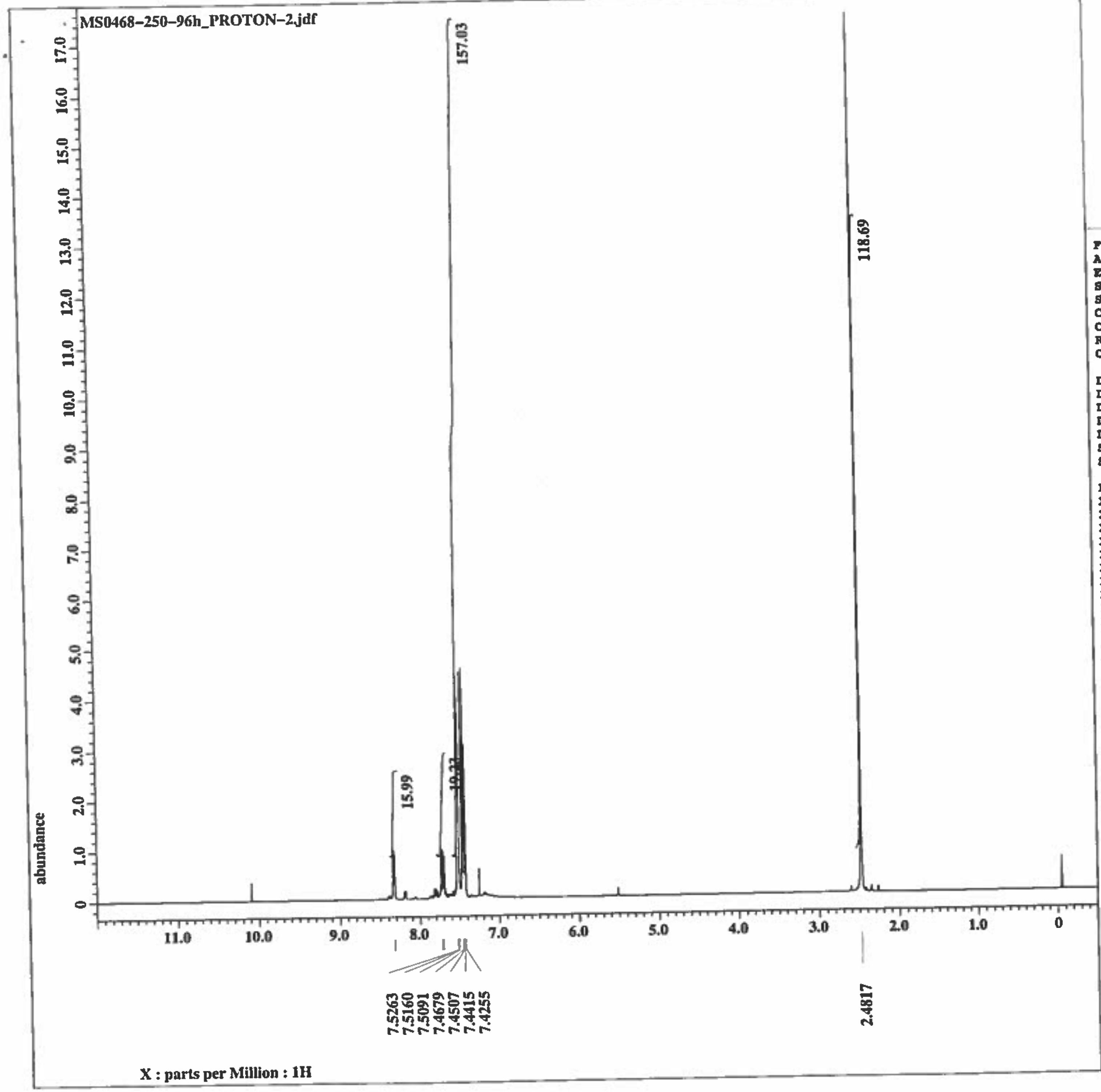

Filename	= MS0468-250-96h_PROTON
Author	= Jim Davis
Experiment	= single_pulse.ex2
Sample_id	= MS0468-250-96h
Solvent	= CHLOROFORM-D
Changer_sample	= 18
Creation_time	= 12-JUN-2018 16:44:30
Revision_time	= 12-JUN-2018 16:18:01
Current_time	= 12-JUN-2018 16:18:01
Data_format	= 1D COMPLEX
Dim_size	= 13107
Dim_title	= 1H
Dim_units	= [ppm]
Dimensions	= X
Site	= ECA 500
Spectrometer	= JNM-ECA500
Field_strength	= 11.7473579[T] (500[MH
X_acq_duration	= 1.74587904[s]
X_domain	= 1H
X_freq	= 500.15991521[MHz]
X_offset	= 5.0[ppm]
X_points	= 16384
X_prescans	= 1
X_resolution	= 0.57277737[Hz]
X_sweep	= 9.38438438[kHz]
Irr_domain	= 1H
Irr_freq	= 500.15991521[MHz]
Irr_offset	= 5.0[ppm]
Tri_domain	= 1H
Tri_freq	= 500.15991521[MHz]
Tri_offset	= 5.0[ppm]
Clipped	= FALSE
Mod_return	= 1
Scans	= 16
Total_scans	= 16
X_90_width	= 12.4[us]
X_acq_time	= 1.74587904[s]
X_angle	= 45[deg]
X_atn	= 4[dB]
X_pulse	= 6.2[us]
Irr_mode	= Off
Tri_mode	= Off
Dante_presat	= FALSE
Initial_wait	= 1[s]
Recvr_gain	= 36
Relaxation_delay	= 4[s]
Repetition_time	= 5.74587904[s]
Temp_get	= 22.2[dC]

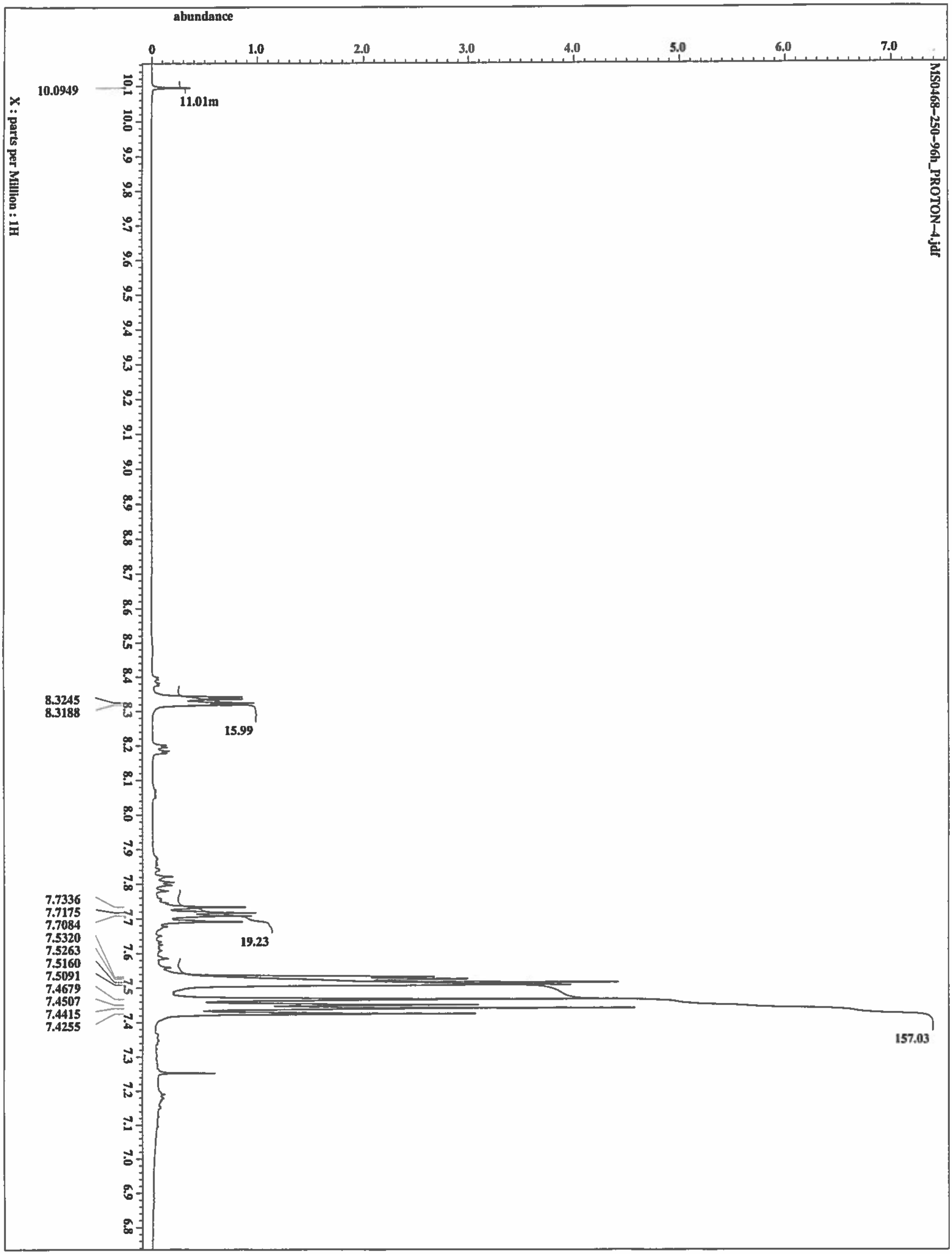

abundance
0
1.0
2.0
3.0
4.0
5.0
6.0
7.0
MS0468-250-96h_PROTON-4.jdf
X : parts per Million : 1H
10.0949
11.01m
8.3245
8.3188
15.99
7.7336
7.7175
7.7084
7.5320
7.5263
7.5160
7.5091
7.4679
7.4507
7.4415
7.4255
19.23
157.03

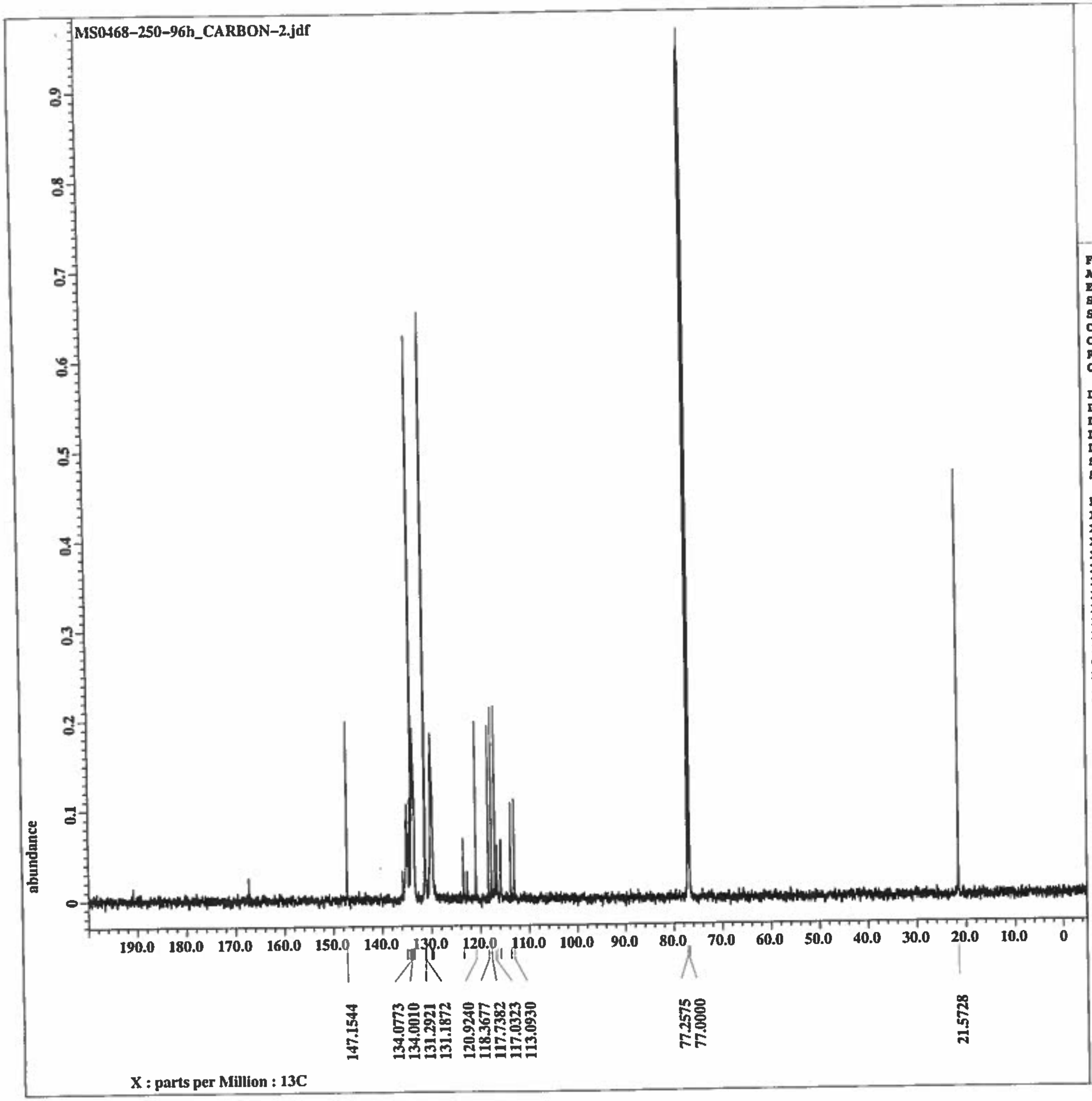

Filename	= MS0468-250-96h_CARBON
Author	= Jim Davis
Experiment	= single_pulse_dec
Sample_id	= MS0468-250-96h
Solvent	= CHLOROFORM-D
Changer_sample	= 18
Creation_time	= 12-JUN-2018 17:05:42
Revision_time	= 12-JUN-2018 16:39:13
Current_time	= 12-JUN-2018 16:39:13
Data_format	= 1D COMPLEX
Dim_size	= 26214
Dim_title	= 13C
Dim_units	= [ppm]
Dimensions	= X
Site	= ECA 500
Spectrometer	= JNM-ECA500
Field_strength	= 11.7473579[T] (500[MH
X_acq_duration	= 0.83361792[s]
X_domain	= 13C
X_freq	= 125.76529768[MHz]
X_offset	= 100[ppm]
X_points	= 32768
X_prescans	= 4
X_resolution	= 1.19959034[Hz]
X_sweep	= 39.3081761[kHz]
Irr_domain	= 1H
Irr_freq	= 500.15991521[MHz]
Irr_offset	= 5.0[ppm]
Clipped	= FALSE
Mod_return	= 1
Scans	= 400
Total_scans	= 400
X_90_width	= 13.2[us]
X_acq_time	= 0.83361792[s]
X_angle	= 30[deg]
X_atn	= 6[dB]
X_pulse	= 4.4[us]
Irr_atn_dec	= 20.7[dB]
Irr_atn_noe	= 20.7[dB]
Irr_noise	= WALTZ
Decoupling	= TRUE
Initial_wait	= 1[s]
Noe	= TRUE
Noe_time	= 2[s]
Recvr_gain	= 60
Relaxation_delay	= 2[s]
Repetition_time	= 2.83361792[s]
Temp_get	= 22.6[dC]

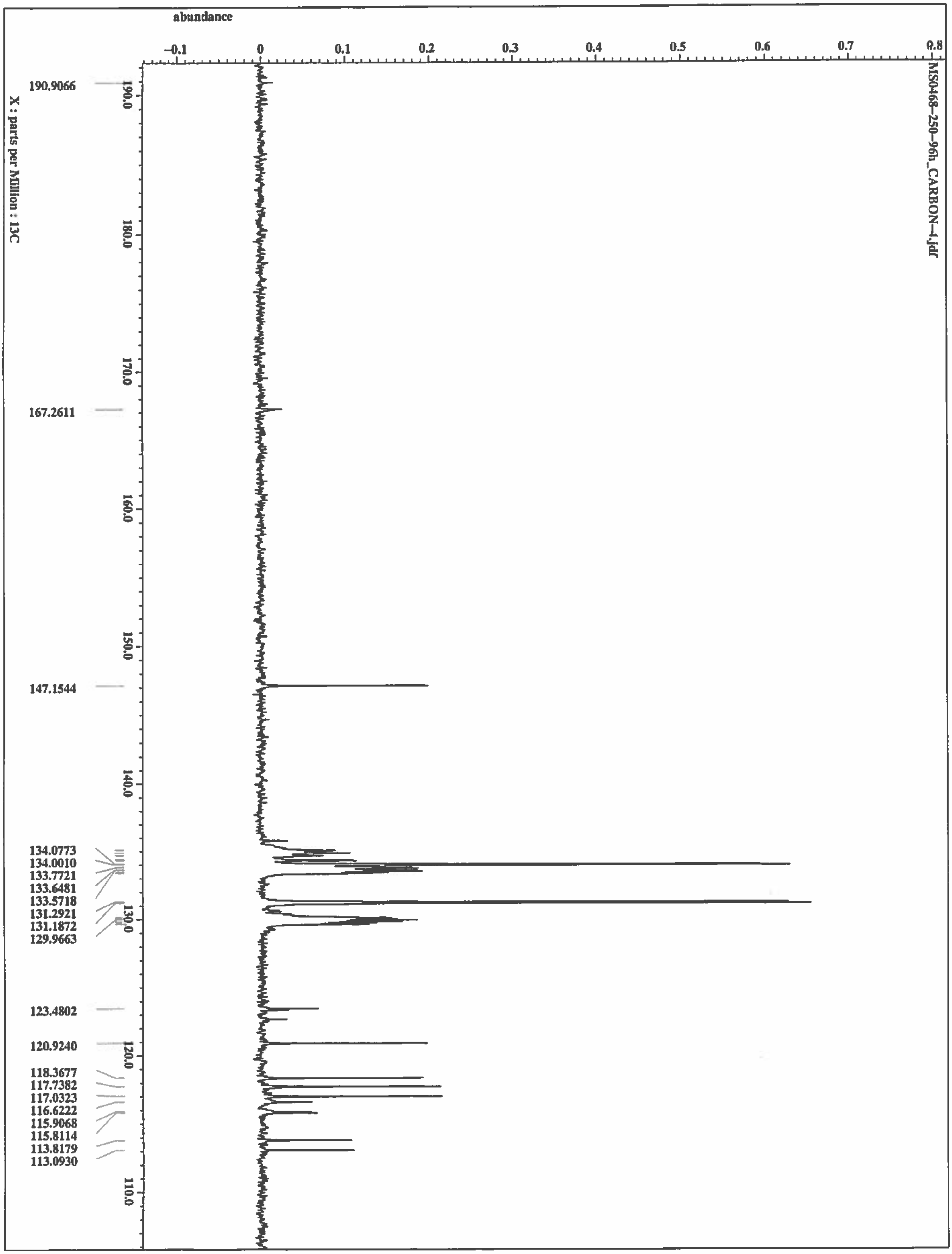

abundance
-0.1
0
0.1
0.2
0.3
0.4
0.5
0.6
0.7
0.8
MS0468-250-96h_CARBON-4.jdf
X : parts per Million : 13C
190.0
180.0
170.0
160.0
150.0
140.0
130.0
120.0
110.0
190.9066
167.2611
147.1544
134.0773
134.0010
133.7721
133.6481
133.5718
131.2921
131.1872
129.9663
123.4802
120.9240
118.3677
117.7382
117.0323
116.6222
115.9068
115.8114
113.8179
113.0930

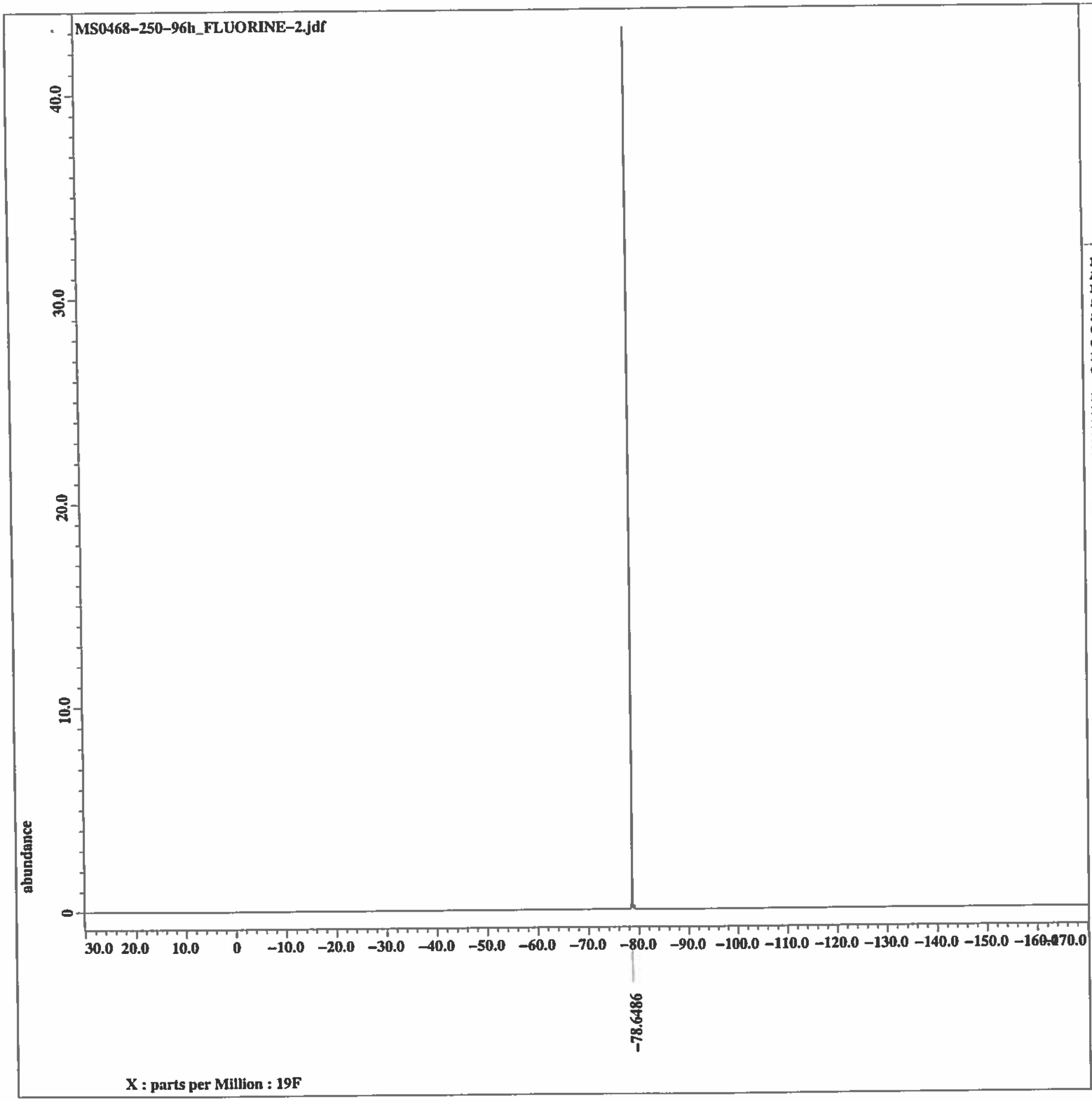

Filename	= MS0468-250-96h_FLUORI
Author	= Jim Davis
Experiment	= single_pulse.ex2
Sample_id	= MS0468-250-96h
Solvent	= CHLOROFORM-D
Changer_sample	= 18
Creation_time	= 12-JUN-2018 17:08:39
Revision_time	= 12-JUN-2018 16:42:10
Current_time	= 12-JUN-2018 16:42:11
Data_format	= 1D COMPLEX
Dim_size	= 52428
Dim_title	= 19F
Dim_units	= [ppm]
Dimensions	= X
Site	= ECA 500
Spectrometer	= JNM-ECA500
Field_strength	= 11.7473579[T] (500[MH
X_acq_duration	= 0.55574528[s]
X_domain	= 19F
X_freq	= 470.62046084[MHz]
X_offset	= -70[ppm]
X_points	= 65536
X_prescans	= 1
X_resolution	= 1.7993855[Hz]
X_sweep	= 117.9245283[kHz]
Irr_domain	= 19F
Irr_freq	= 470.62046084[MHz]
Irr_offset	= 5[ppm]
Tri_domain	= 19F
Tri_freq	= 470.62046084[MHz]
Tri_offset	= 5[ppm]
Clipped	= FALSE
Mod_return	= 1
Scans	= 16
Total_scans	= 16
X_90_width	= 13.1[us]
X_acq_time	= 0.55574528[s]
X_angle	= 45[deg]
X_atn	= 2.5[dB]
X_pulse	= 6.55[us]
Irr_mode	= Off
Tri_mode	= Off
Dante_presat	= FALSE
Initial_wait	= 1[s]
Recvr_gain	= 36
Relaxation_delay	= 4[s]
Repetition_time	= 4.55574528[s]
Temp_get	= 22.3[dC]

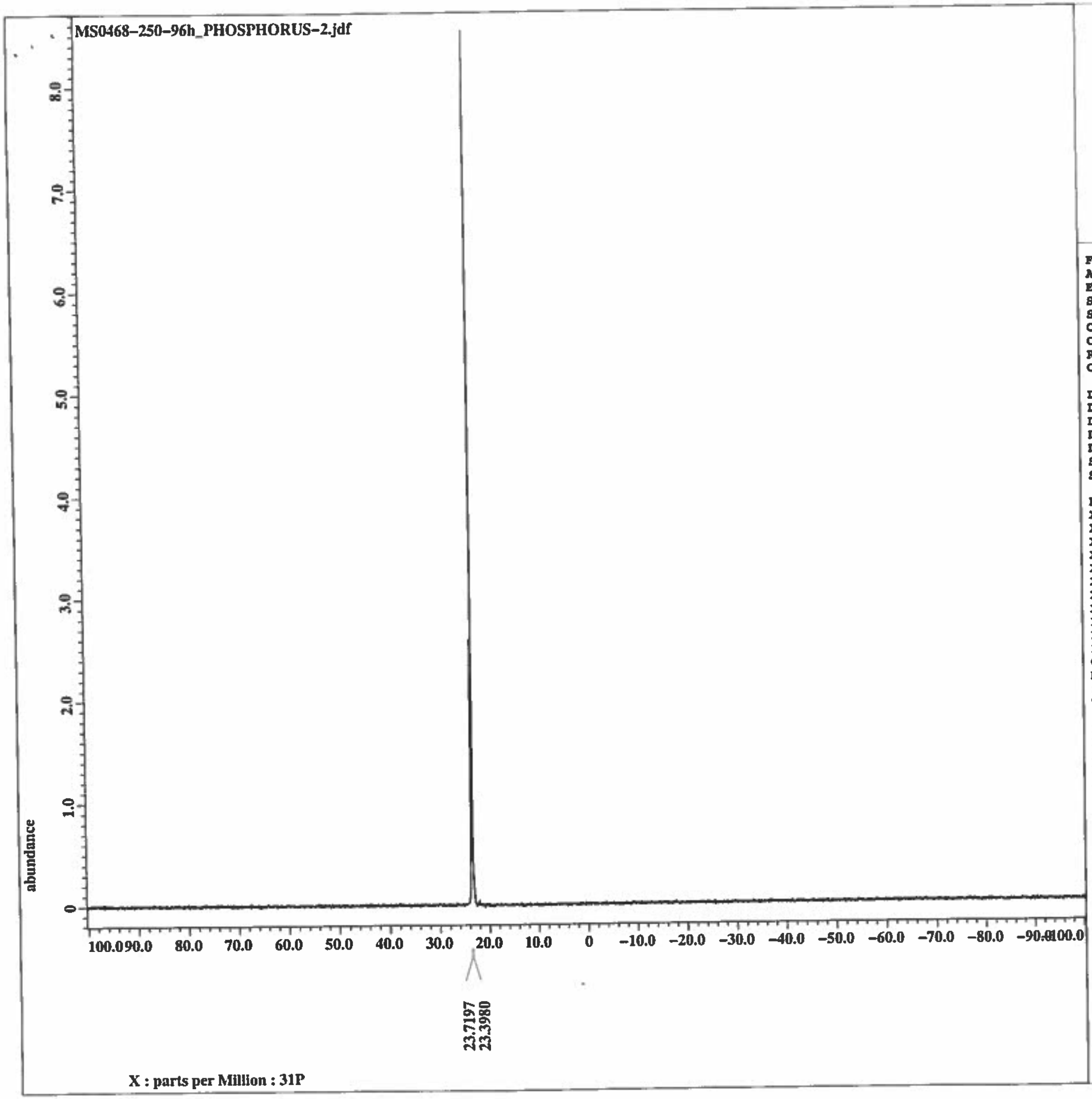

Filename	= MS0468-250-96h_PHOSPH
Author	= Jim Davis
Experiment	= single_pulse_dec
Sample_id	= MS0468-250-96h
Solvent	= CHLOROFORM-D
Changer_sample	= 18
Creation_time	= 12-JUN-2018 17:13:18
Revision_time	= 12-JUN-2018 16:46:49
Current_time	= 12-JUN-2018 16:46:49
Data_format	= 1D COMPLEX
Dim_size	= 26214
Dim_title	= 31P
Dim_units	= [ppm]
Dimensions	= X
Site	= ECA 500
Spectrometer	= JNM-ECA500
Field_strength	= 11.7473579[T] (500[MH
X_acq_duration	= 0.64487424[s]
X_domain	= 31P
X_freq	= 202.46831075[MHz]
X_offset	= 0[ppm]
X_points	= 32768
X_prescans	= 4
X_resolution	= 1.55068995[Hz]
X_sweep	= 50.81300813[kHz]
Irr_domain	= 1H
Irr_freq	= 500.15991521[MHz]
Irr_offset	= 5.0[ppm]
Clipped	= FALSE
Mod_return	= 1
Scans	= 50
Total_scans	= 50
X_90_width	= 14.687[us]
X_acq_time	= 0.64487424[s]
X_angle	= 30[deg]
X_atn	= 5[dB]
X_pulse	= 4.89566667[us]
Irr_atn_dec	= 20.7[dB]
Irr_atn_noe	= 20.7[dB]
Irr_noise	= WALTZ
Decoupling	= TRUE
Initial_wait	= 1[s]
Noe	= TRUE
Noe_time	= 2[s]
Recvr_gain	= 58
Relaxation_delay	= 2[s]
Repetition_time	= 2.64487424[s]
Temp_get	= 22.6[dC]

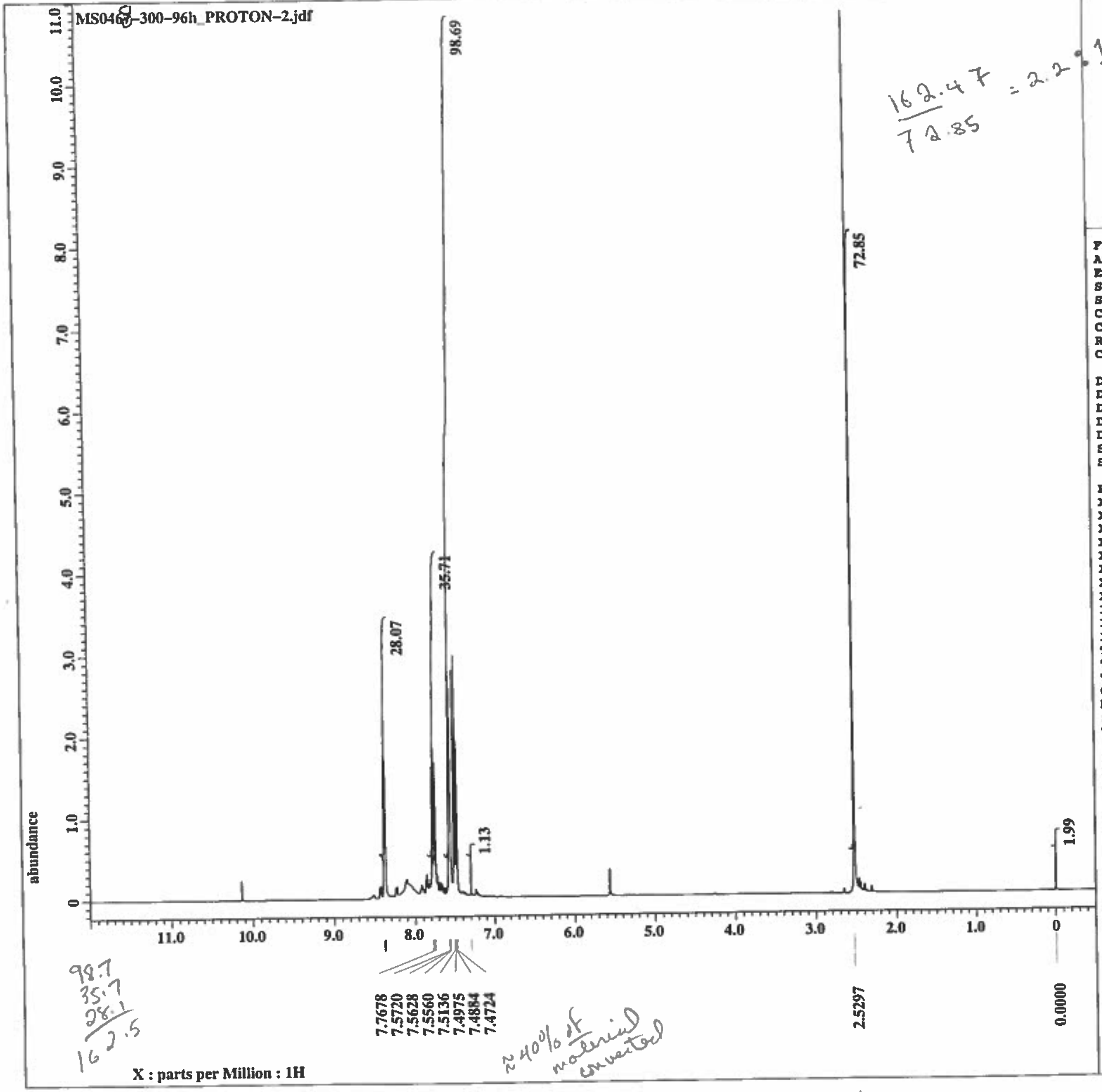

1.3

Filename	= MS0467-300-96h_PROTON
Author	= Jim Davis
Experiment	= single_pulse.ex2
Sample_id	= MS0467-300-96h
Solvent	= CHLOROFORM-D
Changer_sample	= 7
Creation_time	= 12-JUN-2018 12:28:49
Revision_time	= 12-JUN-2018 12:02:22
Current_time	= 12-JUN-2018 12:02:22
Data_format	= 1D COMPLEX
Dim_size	= 13107
Dim_title	= 1H
Dim_units	= [ppm]
Dimensions	= X
Site	= ECA 500
Spectrometer	= JNM-ECA500
Field_strength	= 11.7473579[T] (500[MH
X_acq_duration	= 1.74587904[s]
X_domain	= 1H
X_freq	= 500.15991521[MHz]
X_offset	= 5.0[ppm]
X_points	= 16384
X_prescans	= 1
X_resolution	= 0.57277737[Hz]
X_sweep	= 9.38438438[kHz]
Irr_domain	= 1H
Irr_freq	= 500.15991521[MHz]
Irr_offset	= 5.0[ppm]
Tri_domain	= 1H
Tri_freq	= 500.15991521[MHz]
Tri_offset	= 5.0[ppm]
Clipped	= FALSE
Mod_return	= 1
Scans	= 16
Total_scans	= 16
X_90_width	= 12.4[us]
X_acq_time	= 1.74587904[s]
X_angle	= 45[deg]
X_atn	= 4[dB]
X_pulse	= 6.2[us]
Irr_mode	= Off
Tri_mode	= Off
Dante_presat	= FALSE
Initial_wait	= 1[s]
Recvr_gain	= 36
Relaxation_delay	= 4[s]
Repetition_time	= 5.74587904[s]
Temp_get	= 21.7[dC]

1 ~ required 2.7 : 1 for unchanged aromatics

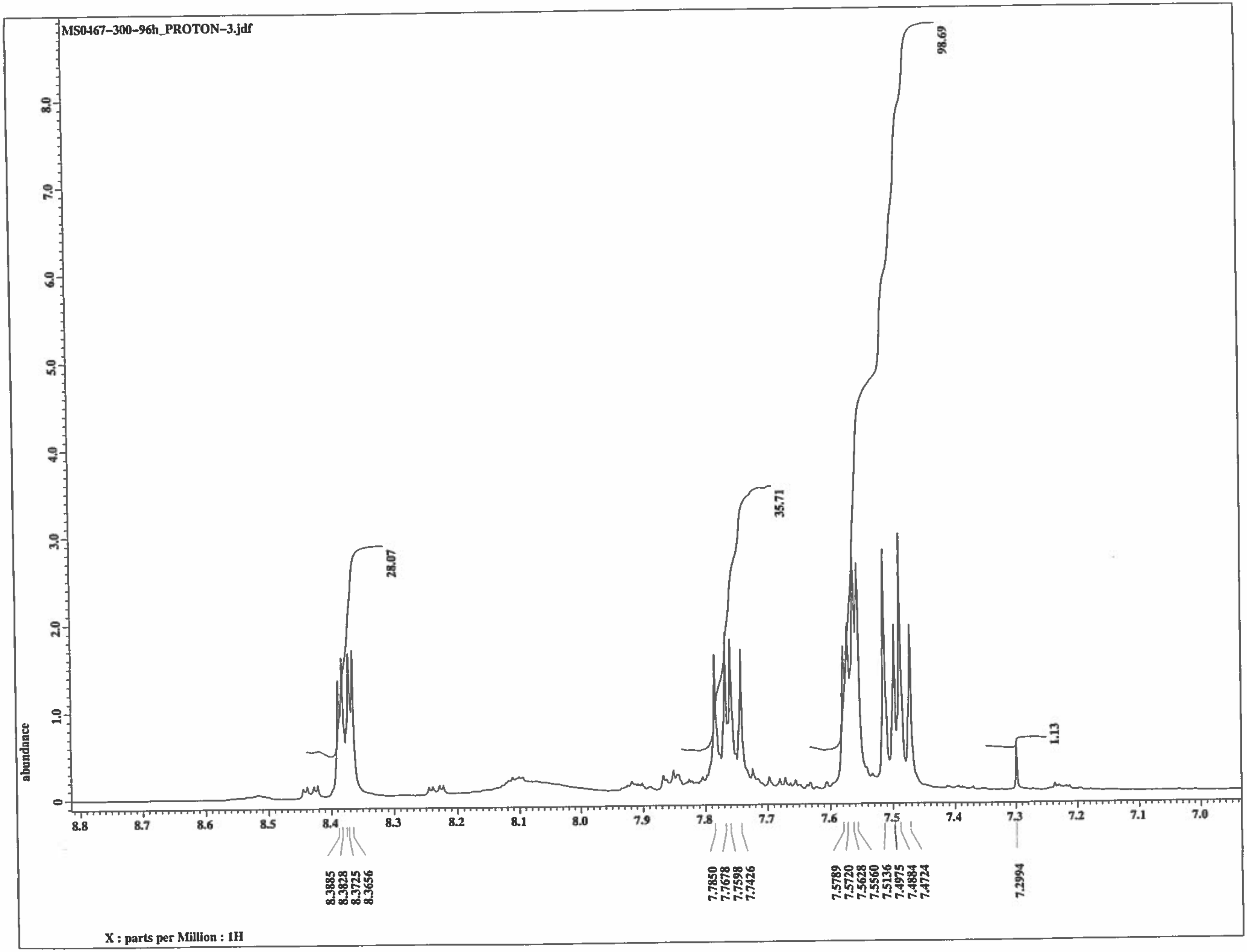
MS0467-300-96h_PROTON-3.jdf
abundance
0
1.0
2.0
3.0
4.0
5.0
6.0
7.0
8.0
28.07
35.71
98.69
1.13
8.8
8.7
8.6
8.5
8.4
8.3
8.2
8.1
8.0
7.9
7.8
7.7
7.6
7.5
7.4
7.3
7.2
7.1
7.0
8.3885
8.3828
8.3725
8.3656
7.7850
7.7678
7.7598
7.7426
7.5789
7.5720
7.5628
7.5560
7.5136
7.4975
7.4884
7.4724
7.2994
X : parts per Million : 1H

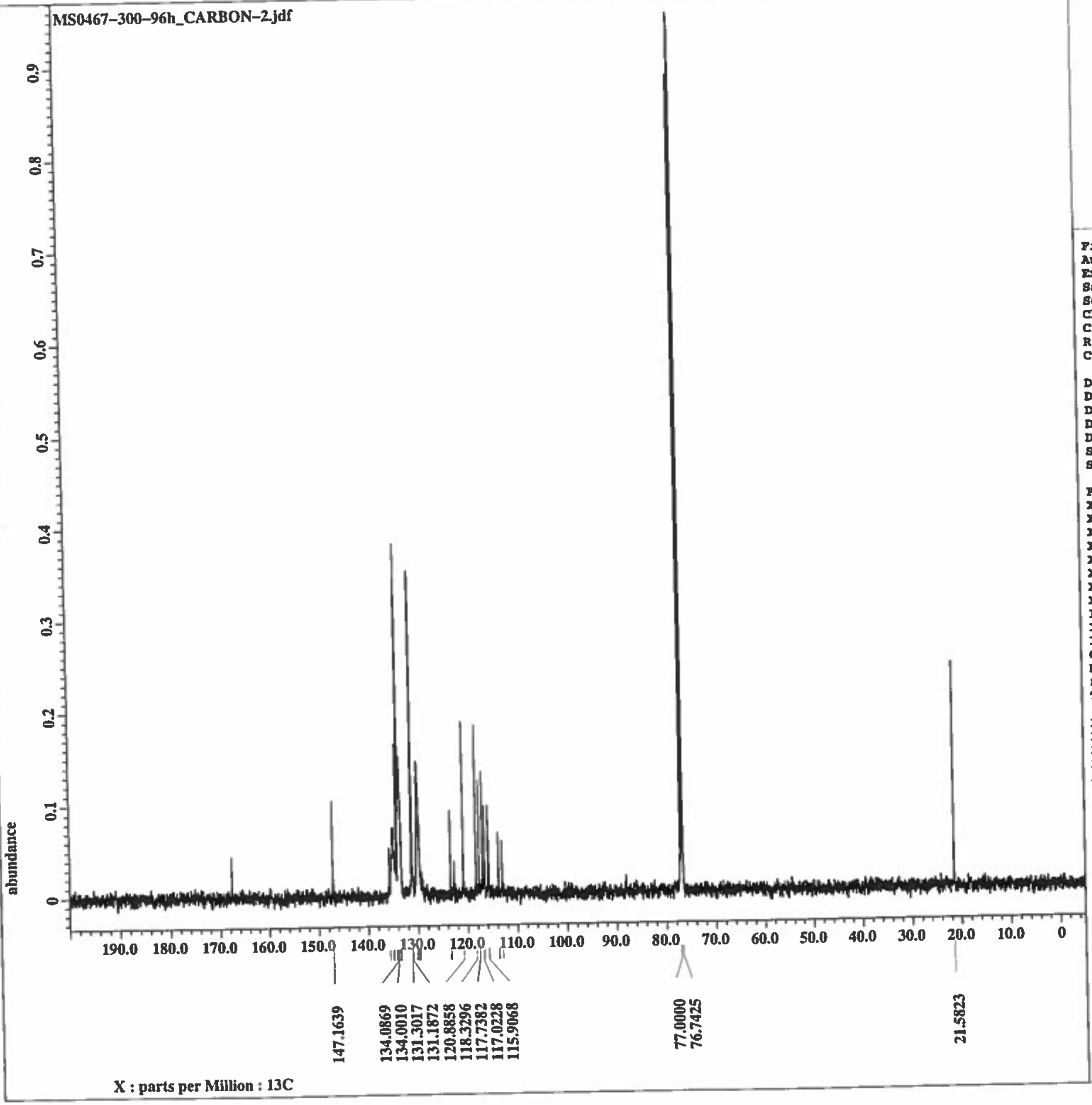

Filename	= MS0467-300-96h_CARBON
Author	= Jim Davis
Experiment	= single_pulse_dec
Sample_id	= MS0467-300-96h
Solvent	= CHLOROFORM-D
Changer_sample	= 7
Creation_time	= 12-JUN-2018 12:44:00
Revision_time	= 12-JUN-2018 12:17:33
Current_time	= 12-JUN-2018 12:17:33
Data_format	= 1D COMPLEX
Dim_size	= 26214
Dim_title	= 13C
Dim_units	= [ppm]
Dimensions	= X
Site	= ECA 500
Spectrometer	= JNM-ECA500
Field_strength	= 11.7473579[T] (500[MH
X_acq_duration	= 0.83361792[s]
X_domain	= 13C
X_freq	= 125.76529768[MHz]
X_offset	= 100[ppm]
X_points	= 32768
X_prescans	= 4
X_resolution	= 1.19959034[Hz]
X_sweep	= 39.3081761[kHz]
Irr_domain	= 1H
Irr_freq	= 500.15991521[MHz]
Irr_offset	= 5.0[ppm]
Clipped	= FALSE
Mod_return	= 1
Scans	= 256
Total_scans	= 256
X_90_width	= 13.2[us]
X_acq_time	= 0.83361792[s]
X_angle	= 30[deg]
X_atn	= 6[dB]
X_pulse	= 4.4[us]
Irr_atn_dec	= 20.7[dB]
Irr_atn_noe	= 20.7[dB]
Irr_noise	= WALTZ
Decoupling	= TRUE
Initial_wait	= 1[s]
Noe	= TRUE
Noe_time	= 2[s]
Recvr_gain	= 60
Relaxation_delay	= 2[s]
Repetition_time	= 2.83361792[s]
Temp_get	= 22.3[dC]

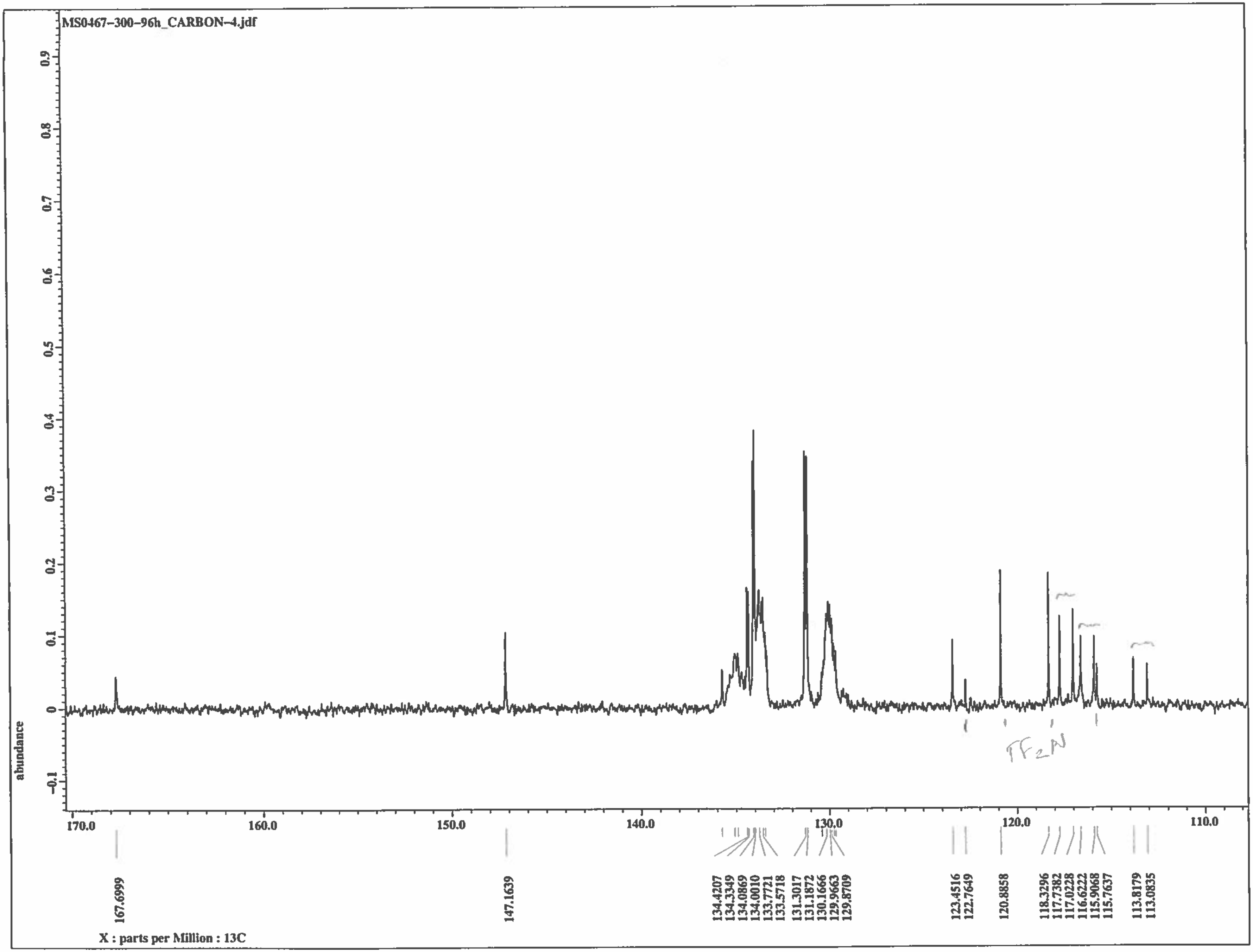
MS0467-300-96h_CARBON-4.jdf
abundance
0.9
0.8
0.7
0.6
0.5
0.4
0.3
0.2
0.1
0
-0.1
170.0
160.0
150.0
140.0
130.0
120.0
110.0
167.6999
147.1639
134.4207
134.3349
134.0869
134.0010
133.7721
133.5718
131.3017
131.1872
130.1666
129.9663
129.8709
123.4516
122.7649
120.8858
118.3296
117.7382
117.0228
116.6222
115.9068
115.7637
113.8179
113.0835
X : parts per Million : 13C

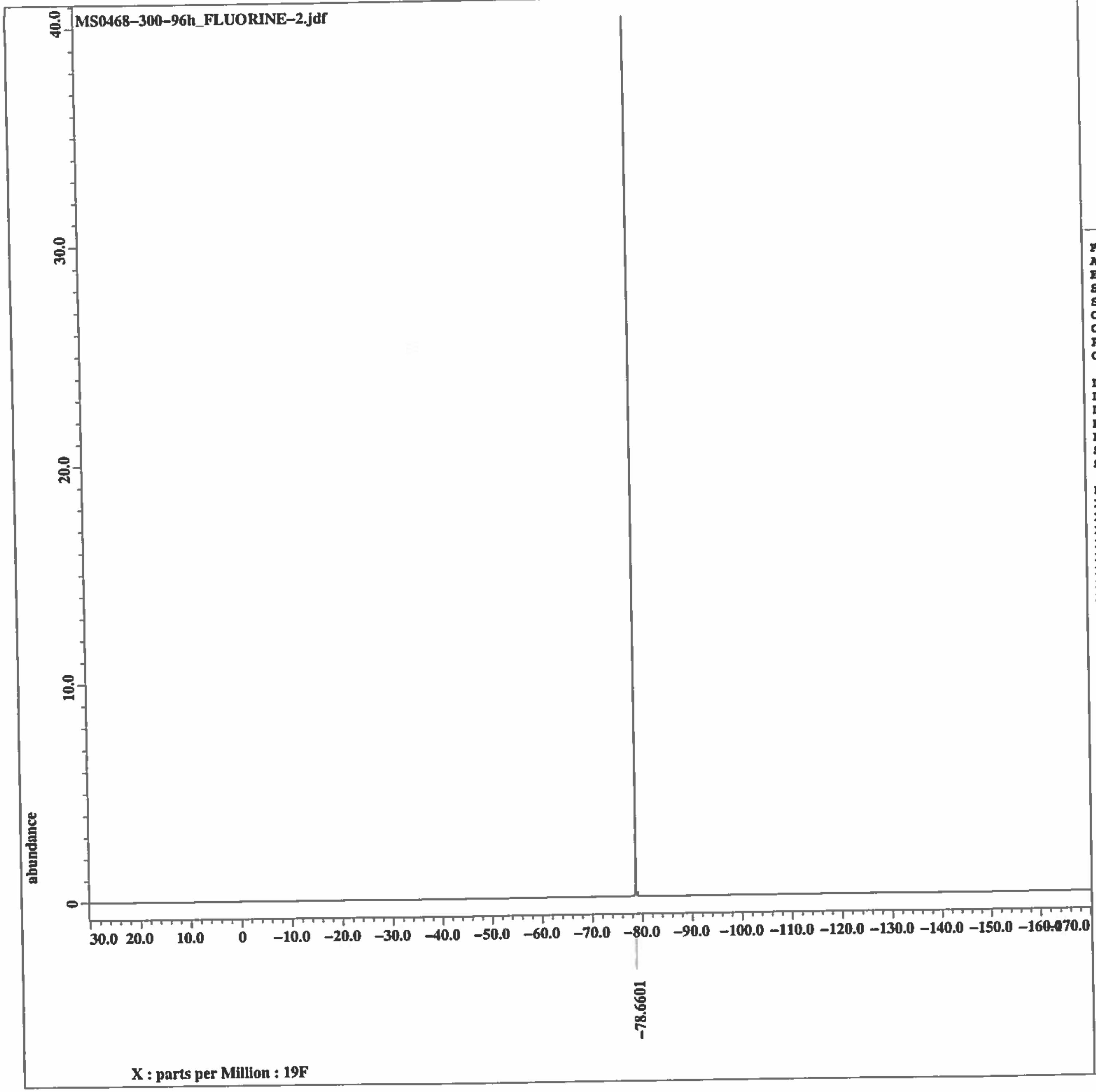

Filename	= MS0468-300-96h_FLUORI
Author	= Jim Davis
Experiment	= single_pulse.ex2
Sample_id	= MS0468-300-96h
Solvent	= CHLOROFORM-D
Changer_sample	= 7
Creation_time	= 12-JUN-2018 12:47:07
Revision_time	= 12-JUN-2018 12:20:40
Current_time	= 12-JUN-2018 12:20:41
Data_format	= 1D COMPLEX
Dim_size	= 52428
Dim_title	= 19F
Dim_units	= [ppm]
Dimensions	= X
Site	= ECA 500
Spectrometer	= JNM-ECA500
Field_strength	= 11.7473579[T] (500[MH
X_acq_duration	= 0.55574528[s]
X_domain	= 19F
X_freq	= 470.62046084[MHz]
X_offset	= -70[ppm]
X_points	= 65536
X_prescans	= 1
X_resolution	= 1.7993855[Hz]
X_sweep	= 117.9245283[kHz]
Irr_domain	= 19F
Irr_freq	= 470.62046084[MHz]
Irr_offset	= 5[ppm]
Tri_domain	= 19F
Tri_freq	= 470.62046084[MHz]
Tri_offset	= 5[ppm]
Clipped	= FALSE
Mod_return	= 1
Scans	= 16
Total_scans	= 16
X_90_width	= 13.1[us]
X_acq_time	= 0.55574528[s]
X_angle	= 45[deg]
X_atn	= 2.5[dB]
X_pulse	= 6.55[us]
Irr_mode	= Off
Tri_mode	= Off
Dante_presat	= FALSE
Initial_wait	= 1[s]
Recvr_gain	= 36
Relaxation_delay	= 4[s]
Repetition_time	= 4.55574528[s]
Temp_get	= 22[dC]

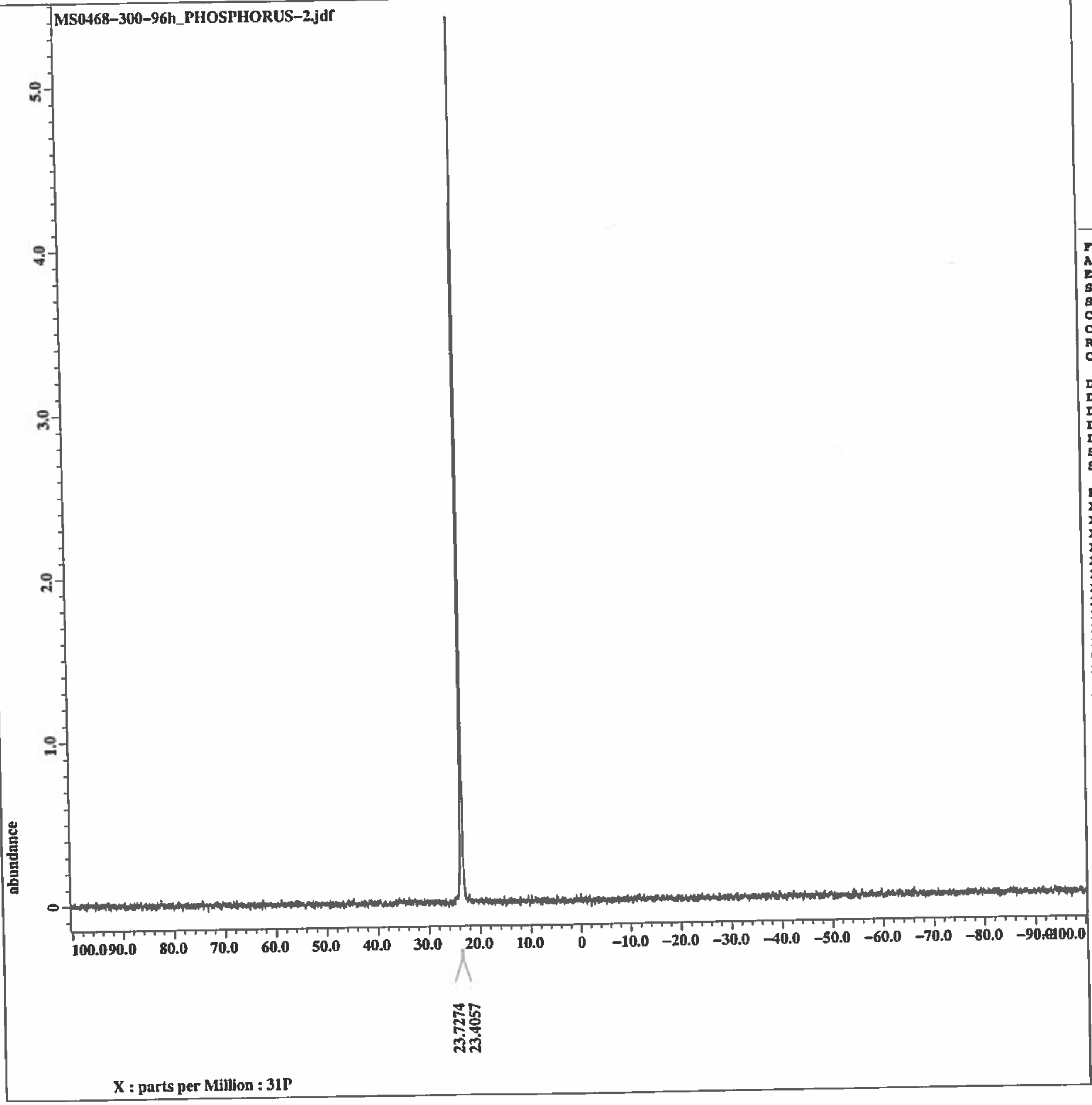

Filename	= MS0468-300-96h_PHOSPH
Author	= Jim Davis
Experiment	= single_pulse_dec
Sample_id	= MS0468-300-96h
Solvent	= CHLOROFORM-D
Changer_sample	= 7
Creation_time	= 12-JUN-2018 12:50:45
Revision_time	= 12-JUN-2018 12:24:17
Current_time	= 12-JUN-2018 12:24:17
Data_format	= 1D COMPLEX
Dim_size	= 26214
Dim_title	= 31P
Dim_units	= [ppm]
Dimensions	= X
Site	= ECA 500
Spectrometer	= JNM-ECA500
Field_strength	= 11.7473579[T] (500[MH
X_acq_duration	= 0.64487424[s]
X_domain	= 31P
X_freq	= 202.46831075[MHz]
X_offset	= 0[ppm]
X_points	= 32768
X_prescans	= 4
X_resolution	= 1.55068995[Hz]
X_sweep	= 50.81300813[kHz]
Irr_domain	= 1H
Irr_freq	= 500.15991521[MHz]
Irr_offset	= 5.0[ppm]
Clipped	= FALSE
Mod_return	= 1
Scans	= 25
Total_scans	= 25
X_90_width	= 14.687[us]
X_acq_time	= 0.64487424[s]
X_angle	= 30[deg]
X_atn	= 5[dB]
X_pulse	= 4.89566667[us]
Irr_atn_dec	= 20.7[dB]
Irr_atn_noe	= 20.7[dB]
Irr_noise	= WALTZ
Decoupling	= TRUE
Initial_wait	= 1[s]
Noe	= TRUE
Noe_time	= 2[s]
Recvr_gain	= 58
Relaxation_delay	= 2[s]
Repetition_time	= 2.64487424[s]
Temp_get	= 22.2[dC]

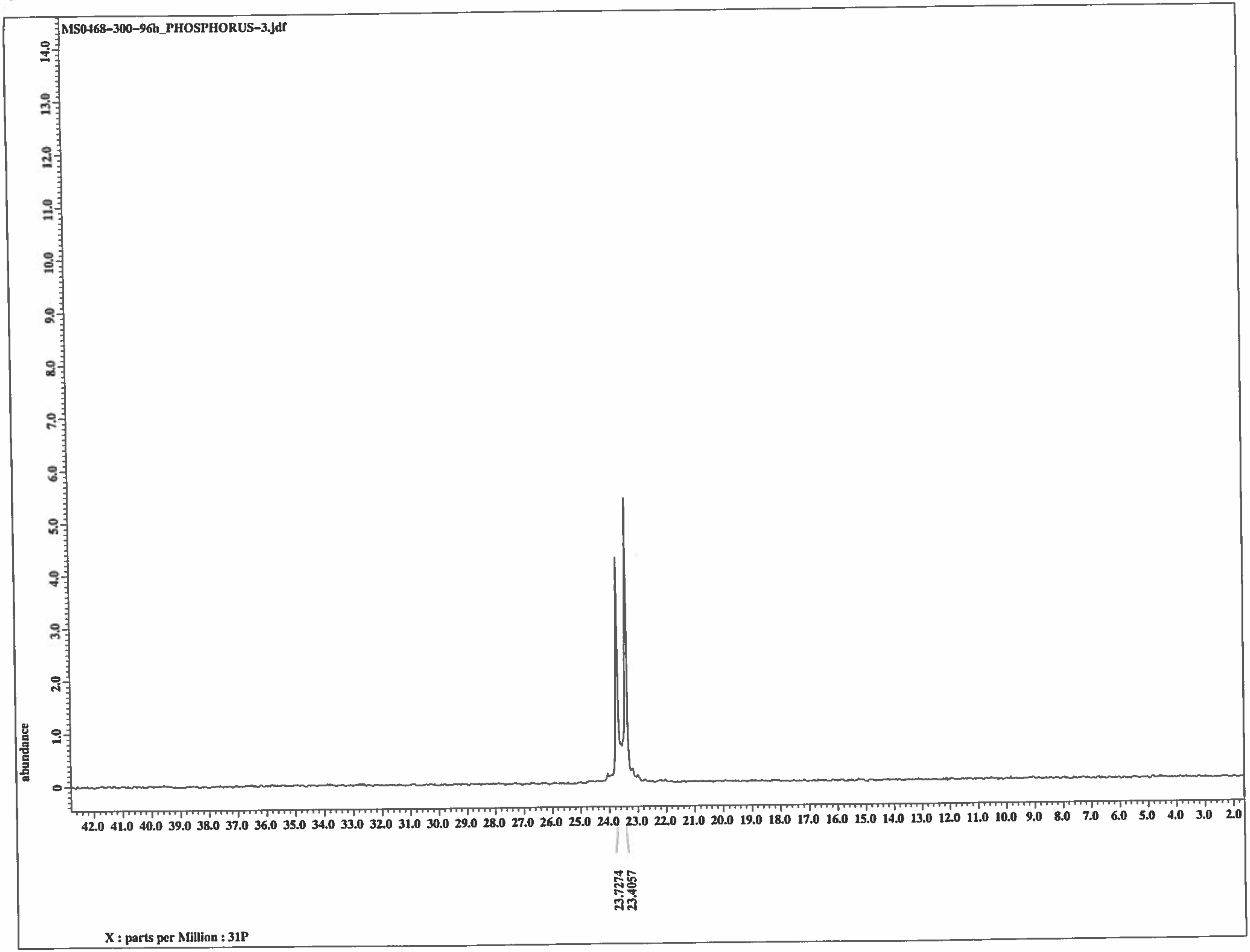
MS0468-300-96h_PHOSPHORUS-3.jdf
abundance
23.7274
23.4057
X : parts per Million : 31P

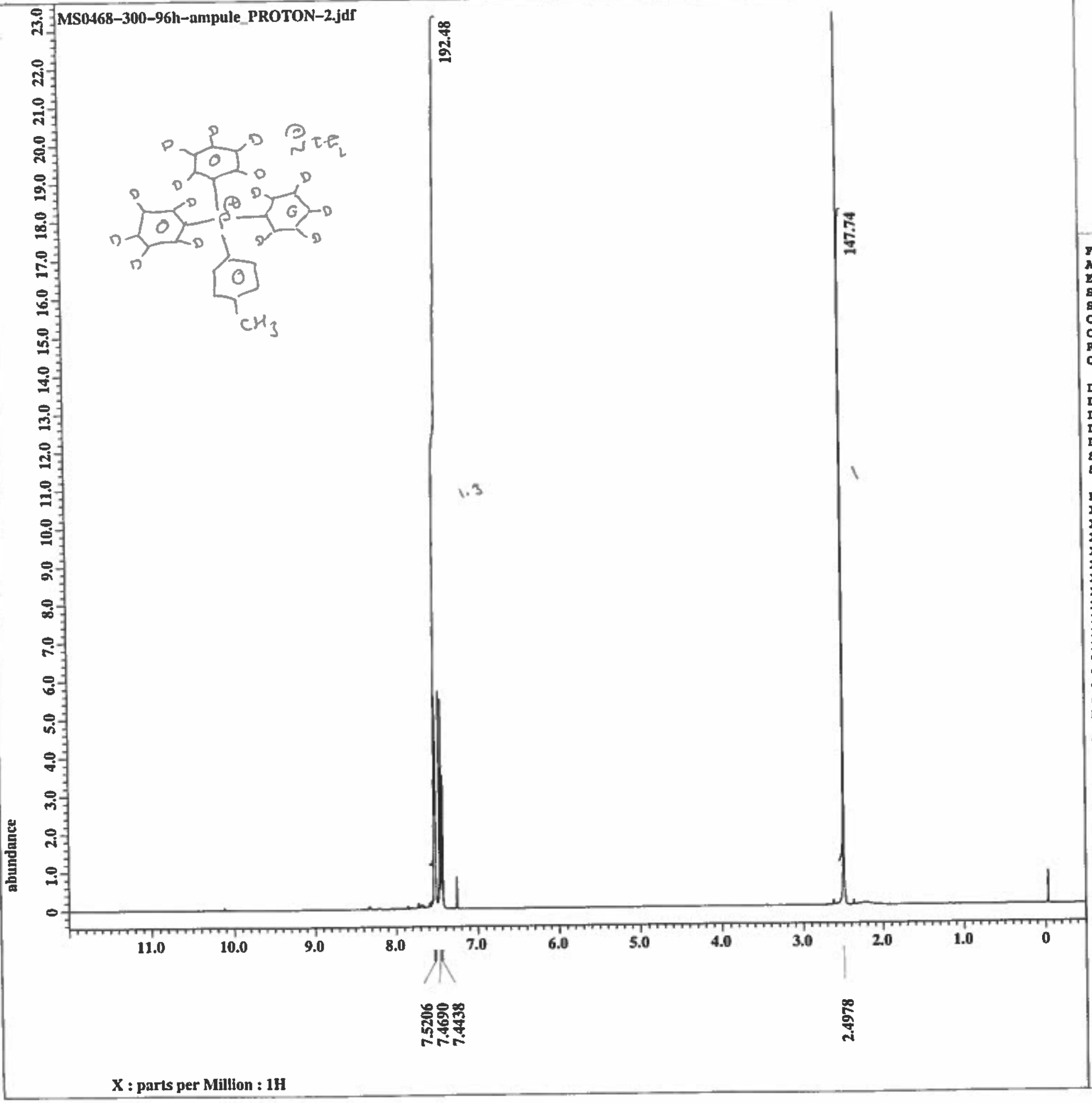

Filename	= MS0468-300-96h-ampule
Author	= Jim Davis
Experiment	= single_pulse.ex2
Sample_id	= MS0468-300-96h-ampule
Solvent	= CHLOROFORM-D
Changer_sample	= 9
Creation_time	= 25-JUN-2018 10:40:03
Revision_time	= 25-JUN-2018 10:17:21
Current_time	= 25-JUN-2018 10:17:21
Data_format	= 1D COMPLEX
Dim_size	= 13107
Dim_title	= 1H
Dim_units	= [ppm]
Dimensions	= X
Site	= ECA 500
Spectrometer	= JNM-ECA500
Field_strength	= 11.7473579[T] (500[MH
X_acq_duration	= 1.74587904[s]
X_domain	= 1H
X_freq	= 500.15991521[MHz]
X_offset	= 5.0[ppm]
X_points	= 16384
X_prescans	= 1
X_resolution	= 0.57277737[Hz]
X_sweep	= 9.38438438[kHz]
Irr_domain	= 1H
Irr_freq	= 500.15991521[MHz]
Irr_offset	= 5.0[ppm]
Tri_domain	= 1H
Tri_freq	= 500.15991521[MHz]
Tri_offset	= 5.0[ppm]
Clipped	= FALSE
Mod_return	= 1
Scans	= 16
Total_scans	= 16
X_90_width	= 12.4[us]
X_acq_time	= 1.74587904[s]
X_angle	= 45[deg]
X_atn	= 4[dB]
X_pulse	= 6.2[us]
Irr_mode	= Off
Tri_mode	= Off
Dante_presat	= FALSE
Initial_wait	= 1[s]
Recvr_gain	= 40
Relaxation_delay	= 4[s]
Repetition_time	= 5.74587904[s]
Temp_get	= 21.8[dC]

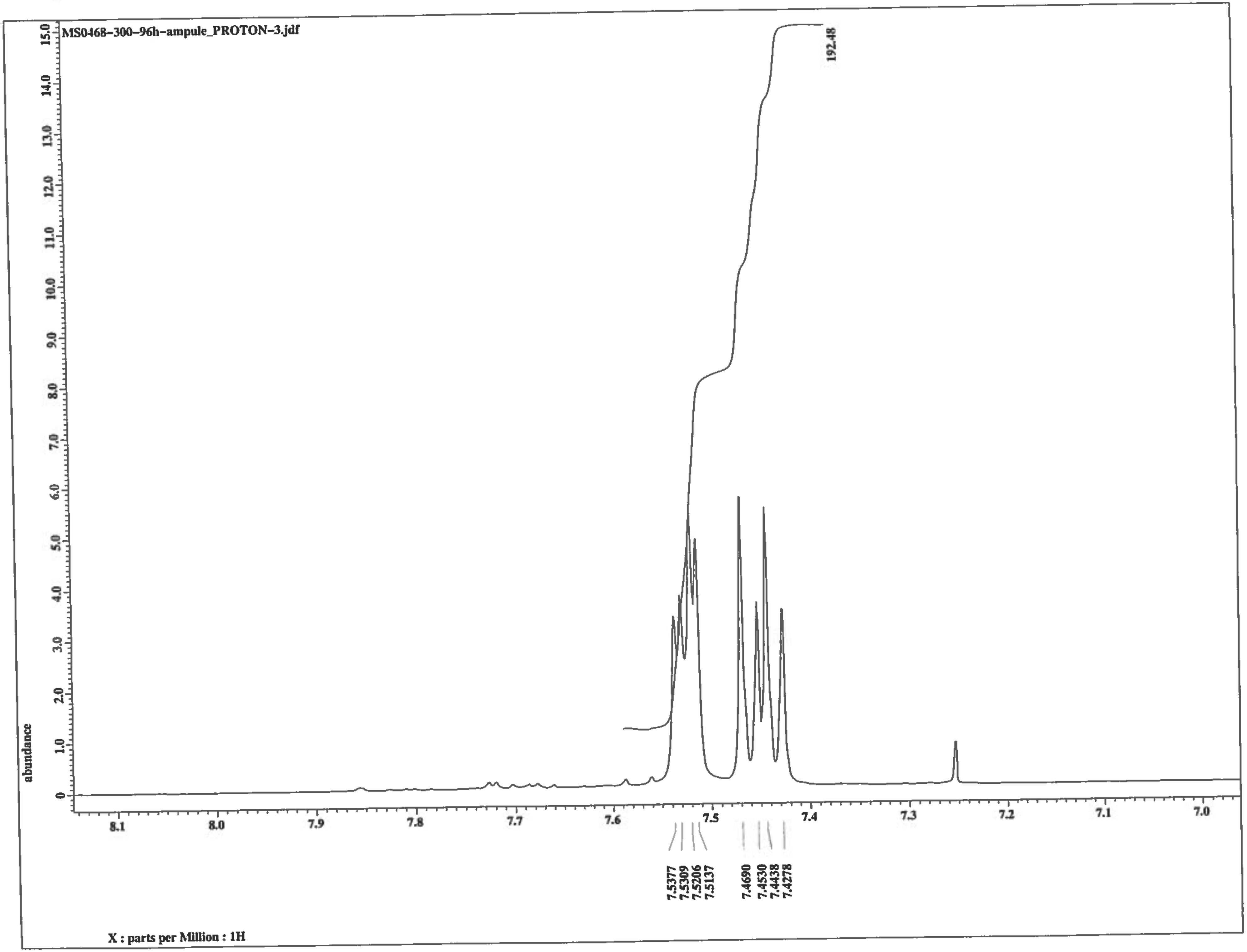

MS0468-300-96h-ampule_PROTON-3.jdf
abundance
X : parts per Million : 1H
192.48
7.5377
7.5309
7.5206
7.5137
7.4690
7.4530
7.4438
7.4278

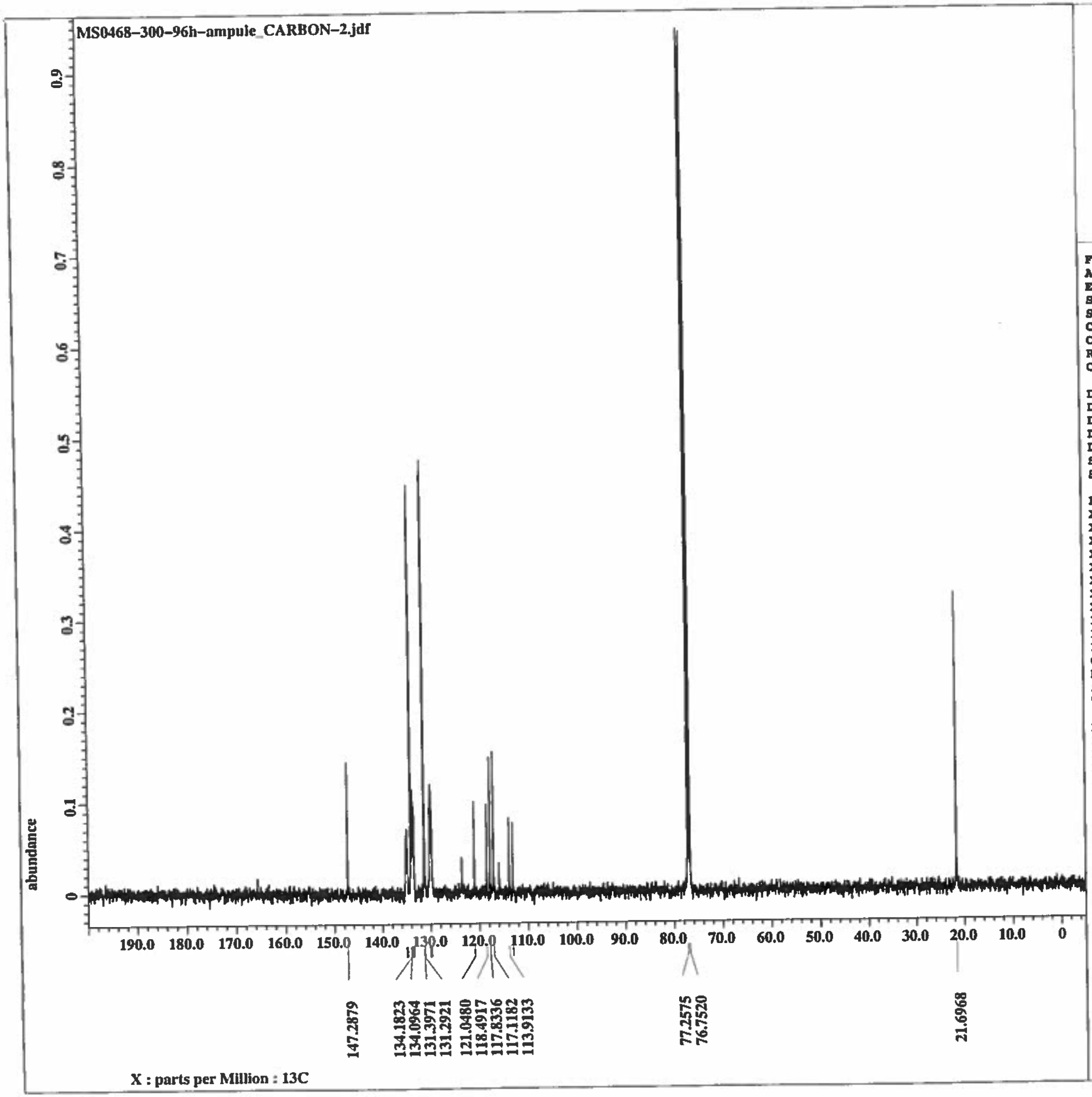

Filename	= MS0468-300-96h-ampule
Author	= Jim Davis
Experiment	= single_pulse_dec
Sample_id	= MS0468-300-96h-ampule
Solvent	= CHLOROFORM-D
Changer_sample	= 9
Creation_time	= 25-JUN-2018 10:54:25
Revision_time	= 25-JUN-2018 10:31:43
Current_time	= 25-JUN-2018 10:31:43
Data_format	= 1D COMPLEX
Dim_size	= 26214
Dim_title	= 13C
Dim_units	= [ppm]
Dimensions	= X
Site	= ECA 500
Spectrometer	= JNM-ECA500
Field_strength	= 11.7473579[T] (500[MH
X_acq_duration	= 0.83361792[s]
X_domain	= 13C
X_freq	= 125.76529768[MHz]
X_offset	= 100[ppm]
X_points	= 32768
X_prescans	= 4
X_resolution	= 1.19959034[Hz]
X_sweep	= 39.3081761[kHz]
Irr_domain	= 1H
Irr_freq	= 500.15991521[MHz]
Irr_offset	= 5.0[ppm]
Clipped	= FALSE
Mod_return	= 1
Scans	= 256
Total_scans	= 256
X_90_width	= 13.2[us]
X_acq_time	= 0.83361792[s]
X_angle	= 30[deg]
X_atn	= 6[dB]
X_pulse	= 4.4[us]
Irr_atn_dec	= 20.7[dB]
Irr_atn_noe	= 20.7[dB]
Irr_noise	= WALTZ
Decoupling	= TRUE
Initial_wait	= 1[s]
Noe	= TRUE
Noe_time	= 2[s]
Recvr_gain	= 60
Relaxation_delay	= 2[s]
Repetition_time	= 2.83361792[s]
Temp_get	= 22.5[dC]

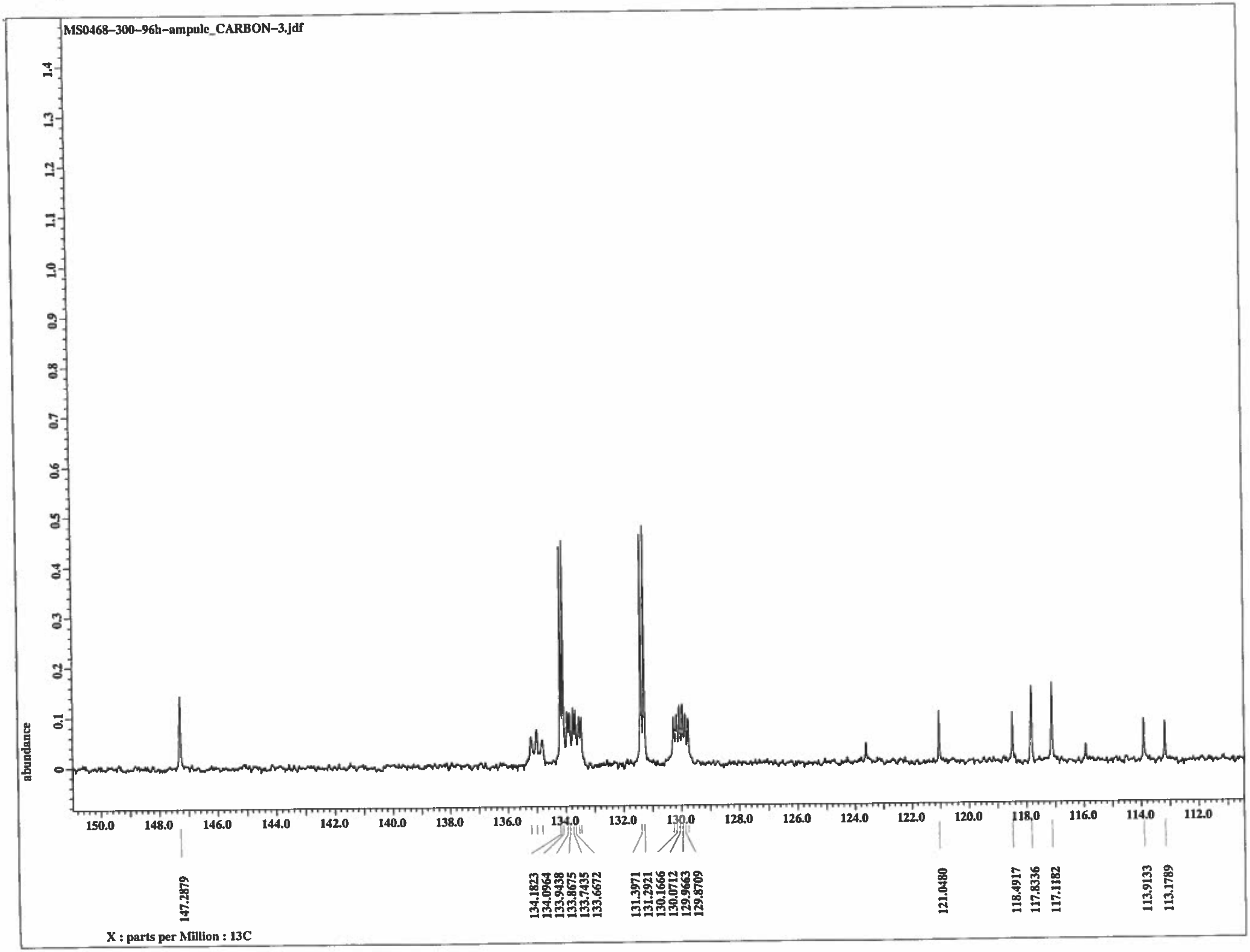
MS0468-300-96h-ampule_CARBON-3.jdf
abundance
0
0.1
0.2
0.3
0.4
0.5
0.6
0.7
0.8
0.9
1.0
1.1
1.2
1.3
1.4
150.0
148.0
146.0
144.0
142.0
140.0
138.0
136.0
134.0
132.0
130.0
128.0
126.0
124.0
122.0
120.0
118.0
116.0
114.0
112.0
147.2879
134.1823
134.0964
133.9438
133.8675
133.7435
133.6672
131.3971
131.2921
130.1666
130.0712
129.9663
129.8709
121.0480
118.4917
117.8336
117.1182
113.9133
113.1789
X : parts per Million : 13C

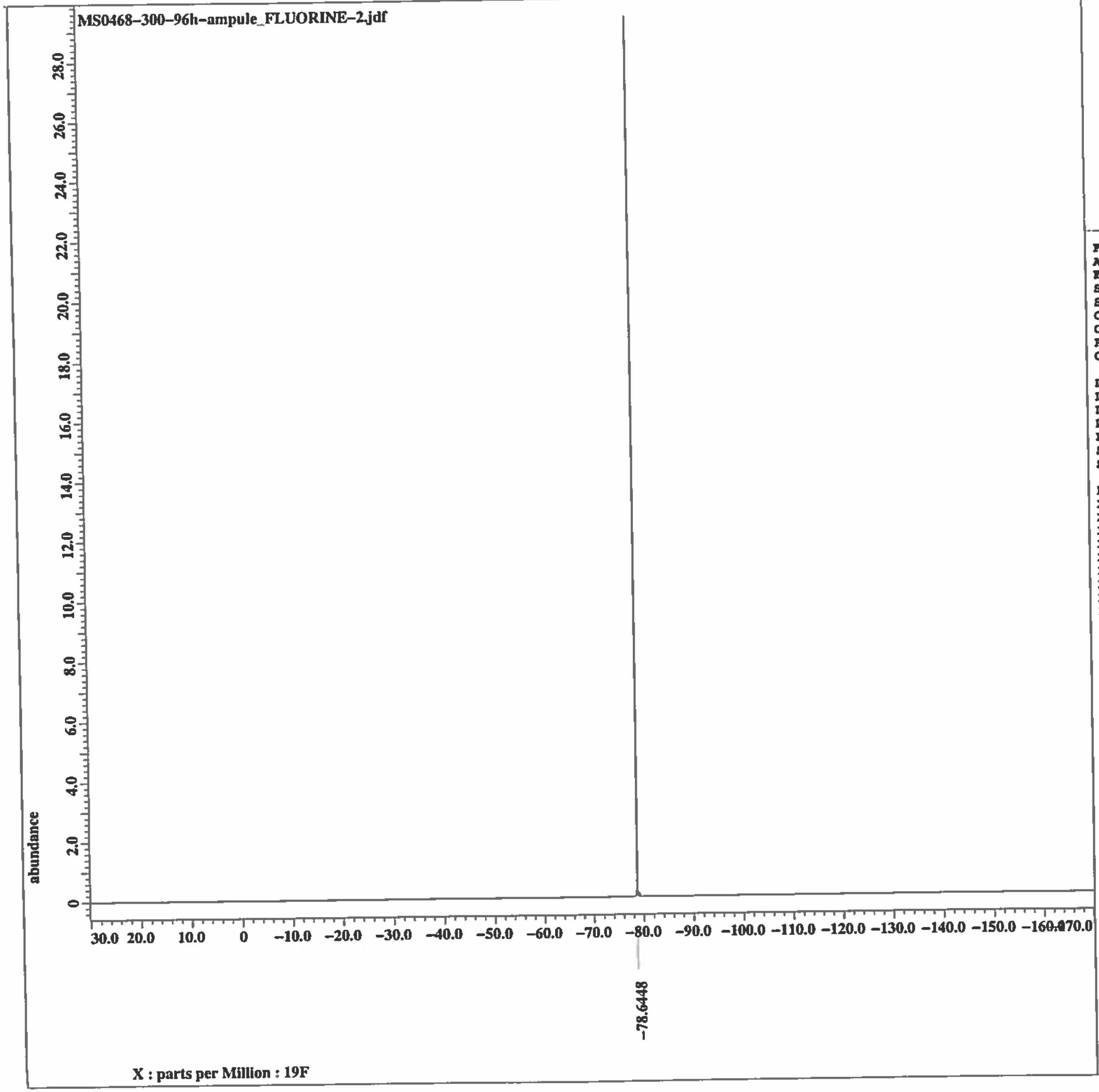

Filename	= MS0468-300-96h-ampule
Author	= Jim Davis
Experiment	= single_pulse.ex2
Sample_id	= MS0468-300-96h-ampule
Solvent	= CHLOROFORM-D
Changer_sample	= 9
Creation_time	= 25-JUN-2018 10:57:31
Revision_time	= 25-JUN-2018 10:34:51
Current_time	= 25-JUN-2018 10:34:51
Data_format	= 1D COMPLEX
Dim_size	= 52428
Dim_title	= 19F
Dim_units	= [ppm]
Dimensions	= X
Site	= ECA 500
Spectrometer	= JNM-ECA500
Field_strength	= 11.7473579[T] (500[MH
X_acq_duration	= 0.55574528[s]
X_domain	= 19F
X_freq	= 470.62046084[MHz]
X_offset	= -70[ppm]
X_points	= 65536
X_prescans	= 1
X_resolution	= 1.7993855[Hz]
X_sweep	= 117.9245283[kHz]
Irr_domain	= 19F
Irr_freq	= 470.62046084[MHz]
Irr_offset	= 5[ppm]
Tri_domain	= 19F
Tri_freq	= 470.62046084[MHz]
Tri_offset	= 5[ppm]
Clipped	= FALSE
Mod_return	= 1
Scans	= 16
Total_scans	= 16
X_90_width	= 13.1[us]
X_acq_time	= 0.55574528[s]
X_angle	= 45[deg]
X_atn	= 2.5[dB]
X_pulse	= 6.55[us]
Irr_mode	= Off
Tri_mode	= Off
Dante_presat	= FALSE
Initial_wait	= 1[s]
Recvr_gain	= 38
Relaxation_delay	= 4[s]
Repetition_time	= 4.55574528[s]
Temp_get	= 22.1[dC]

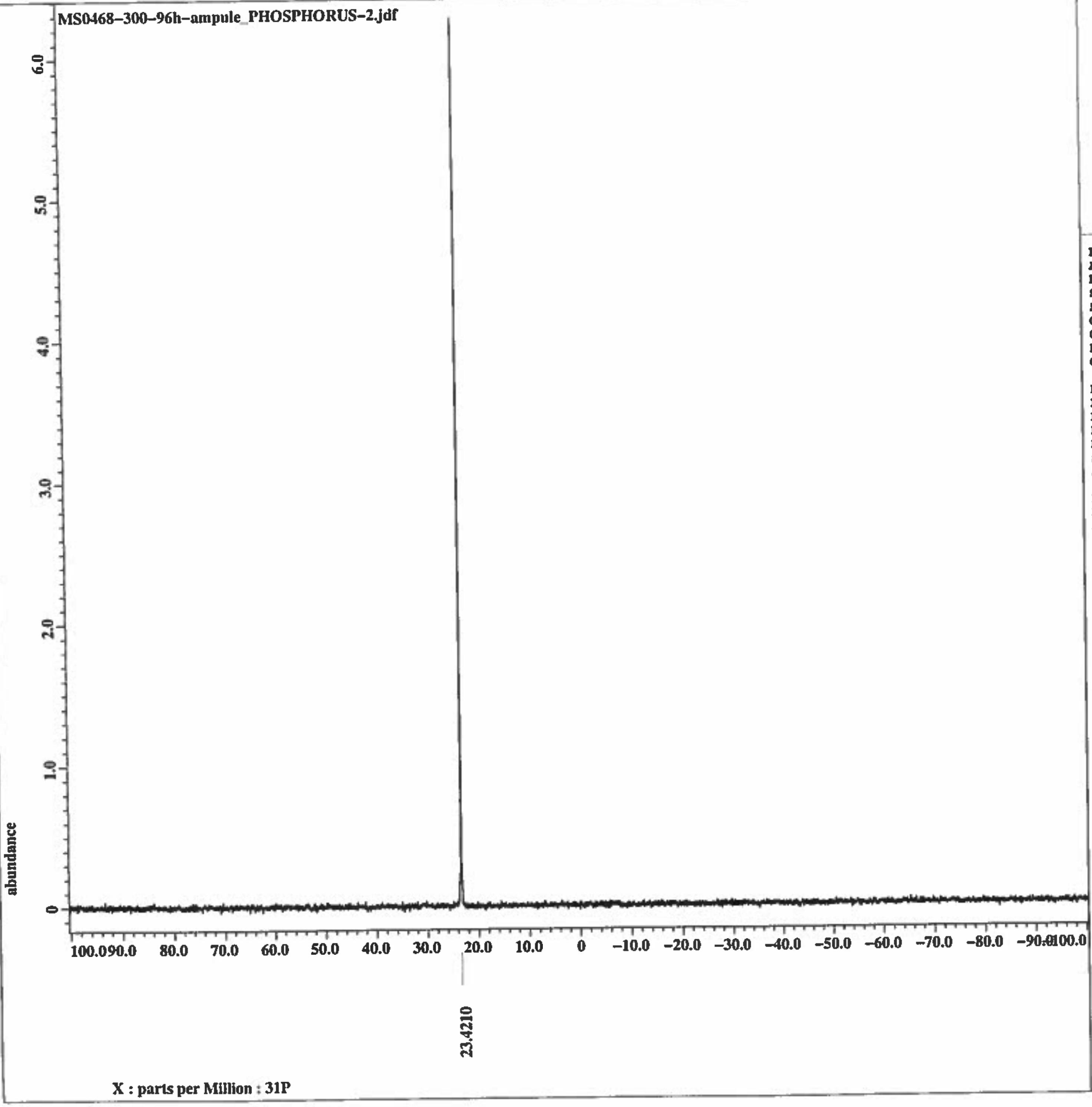

Filename	= MS0468-300-96h-ampule
Author	= Jim Davis
Experiment	= single_pulse_dec
Sample_id	= MS0468-300-96h-ampule
Solvent	= CHLOROFORM-D
Changer_sample	= 9
Creation_time	= 25-JUN-2018 11:01:05
Revision_time	= 25-JUN-2018 10:38:23
Current_time	= 25-JUN-2018 10:38:23
Data_format	= 1D COMPLEX
Dim_size	= 26214
Dim_title	= 31P
Dim_units	= [ppm]
Dimensions	= X
Site	= ECA 500
Spectrometer	= JNM-ECA500
Field_strength	= 11.7473579[T] (500[MH
X_acq_duration	= 0.64487424[s]
X_domain	= 31P
X_freq	= 202.46831075[MHz]
X_offset	= 0[ppm]
X_points	= 32768
X_prescans	= 4
X_resolution	= 1.55068995[Hz]
X_sweep	= 50.81300813[kHz]
Irr_domain	= 1H
Irr_freq	= 500.15991521[MHz]
Irr_offset	= 5.0[ppm]
Clipped	= FALSE
Mod_return	= 1
Scans	= 25
Total_scans	= 25
X_90_width	= 14.687[us]
X_acq_time	= 0.64487424[s]
X_angle	= 30[deg]
X_atn	= 5[dB]
X_pulse	= 4.89566667[us]
Irr_atn_dec	= 20.7[dB]
Irr_atn_noe	= 20.7[dB]
Irr_noise	= WALTZ
Decoupling	= TRUE
Initial_wait	= 1[s]
Noe	= TRUE
Noe_time	= 2[s]
Recvr_gain	= 58
Relaxation_delay	= 2[s]
Repetition_time	= 2.64487424[s]
Temp_get	= 22.3[dC]

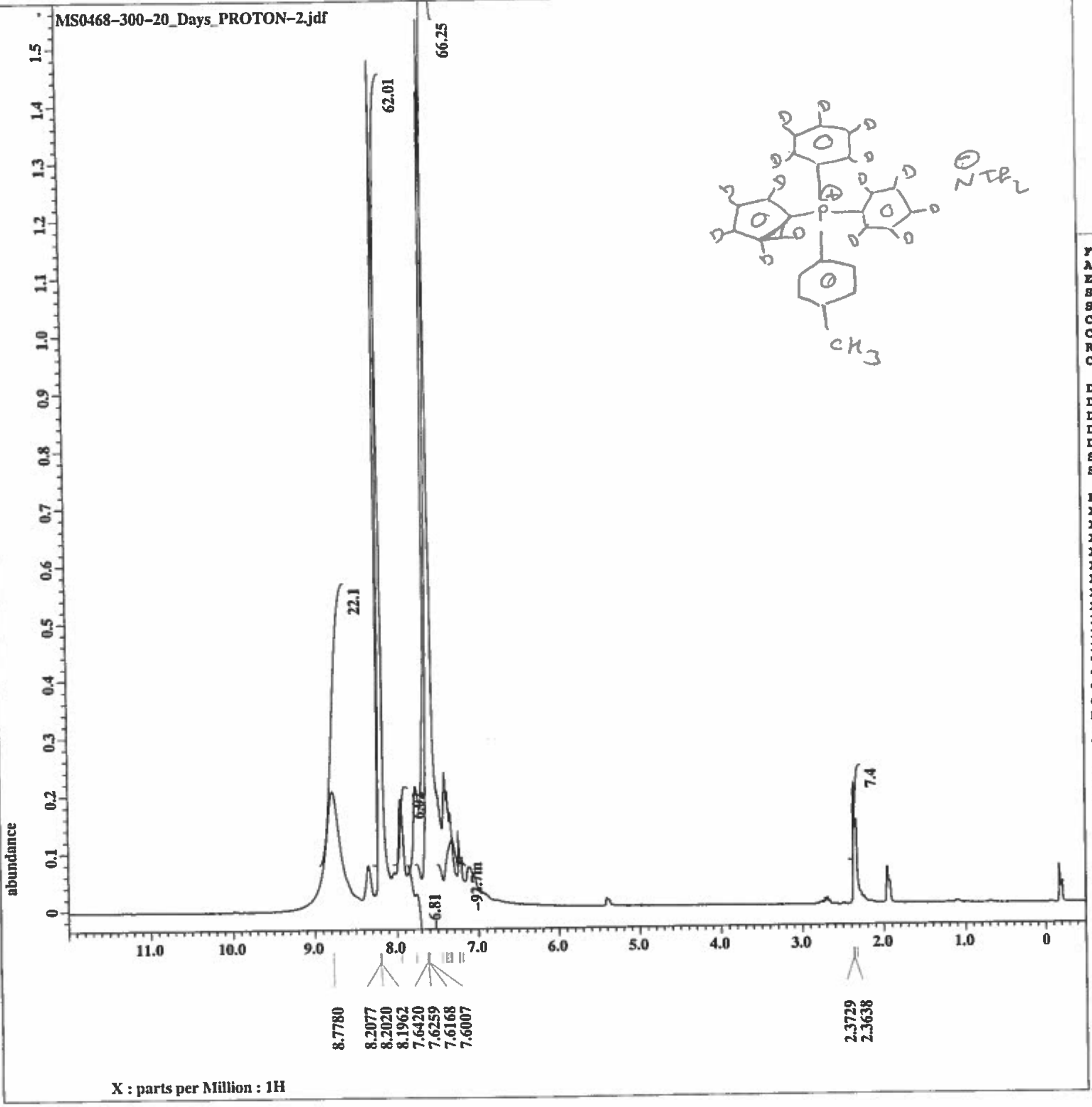

Filename	= MS0468-300-20_Days_PR
Author	= Jim Davis
Experiment	= single_pulse.ex2
Sample_id	= MS0468-300-20_Days
Solvent	= CHLOROFORM-D
Changer_sample	= 8
Creation_time	= 27-JUN-2018 17:21:47
Revision_time	= 27-JUN-2018 16:58:52
Current_time	= 27-JUN-2018 16:58:52
Data_format	= 1D COMPLEX
Dim_size	= 13107
Dim_title	= 1H
Dim_units	= [ppm]
Dimensions	= X
Site	= ECA 500
Spectrometer	= JNM-ECA500
Field_strength	= 11.7473579[T] (500[MH
X_acq_duration	= 1.74587904[s]
X_domain	= 1H
X_freq	= 500.15991521[MHz]
X_offset	= 5.0[ppm]
X_points	= 16384
X_prescans	= 1
X_resolution	= 0.57277737[Hz]
X_sweep	= 9.38438438[kHz]
Irr_domain	= 1H
Irr_freq	= 500.15991521[MHz]
Irr_offset	= 5.0[ppm]
Tri_domain	= 1H
Tri_freq	= 500.15991521[MHz]
Tri_offset	= 5.0[ppm]
Clipped	= FALSE
Mod_return	= 1
Scans	= 16
Total_scans	= 16
X_90_width	= 12.4[us]
X_acq_time	= 1.74587904[s]
X_angle	= 45[deg]
X_atn	= 4[dB]
X_pulse	= 6.2[us]
Irr_mode	= Off
Tri_mode	= Off
Dante_presat	= FALSE
Initial_wait	= 1[s]
Recvr_gain	= 38
Relaxation_delay	= 4[s]
Repetition_time	= 5.74587904[s]
Temp_get	= 22.5[dC]

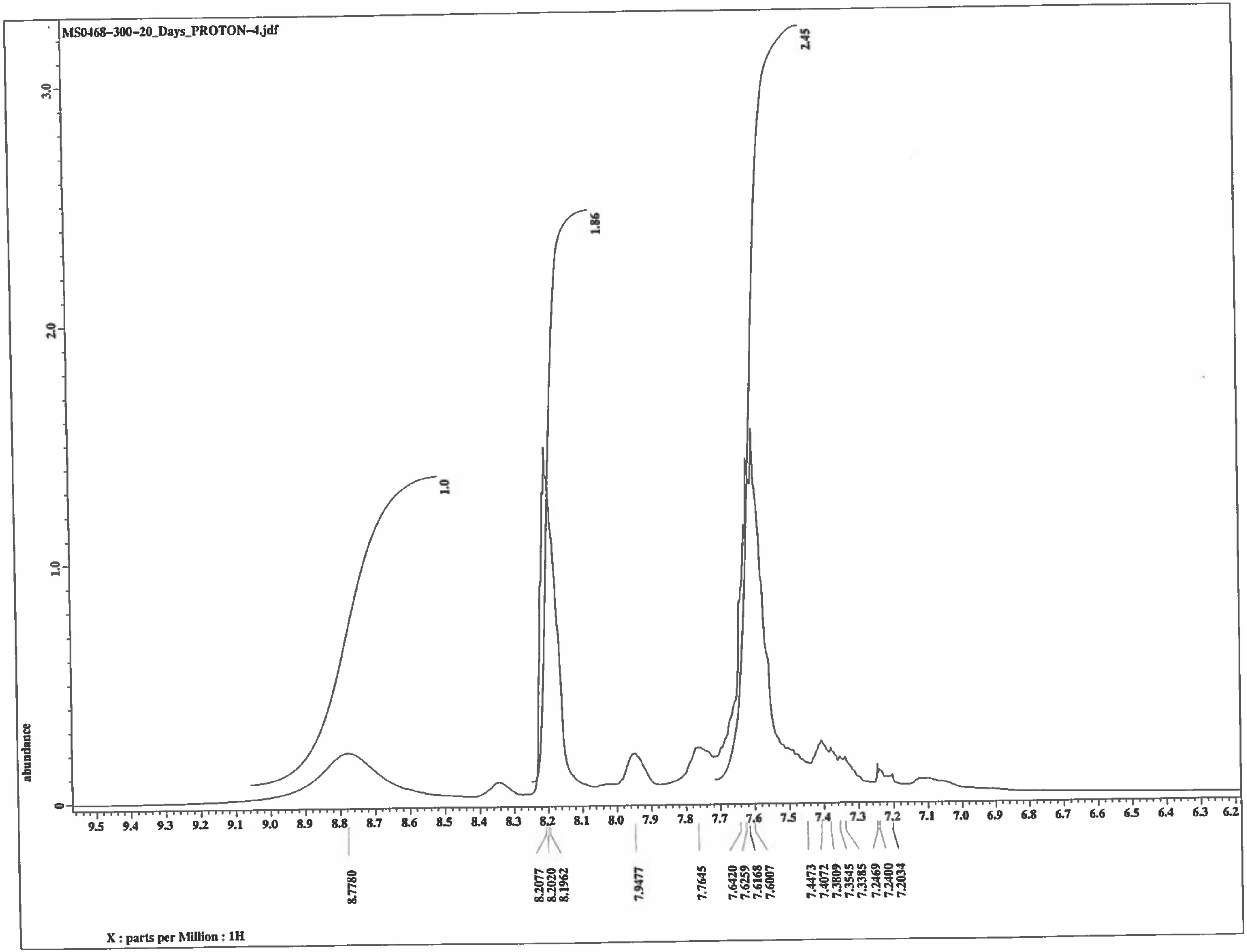
MS0468-300-20_Days_PROTON-4.jdf
abundance
0
1.0
2.0
3.0
1.0
1.86
2.45
9.5
9.4
9.3
9.2
9.1
9.0
8.9
8.8
8.7
8.6
8.5
8.4
8.3
8.2
8.1
8.0
7.9
7.8
7.7
7.6
7.5
7.4
7.3
7.2
7.1
7.0
6.9
6.8
6.7
6.6
6.5
6.4
6.3
6.2
8.7780
8.2077
8.2020
8.1962
7.9477
7.7645
7.6420
7.6259
7.6168
7.6007
7.4473
7.4072
7.3809
7.3545
7.3385
7.2469
7.2400
7.2034
X : parts per Million : 1H

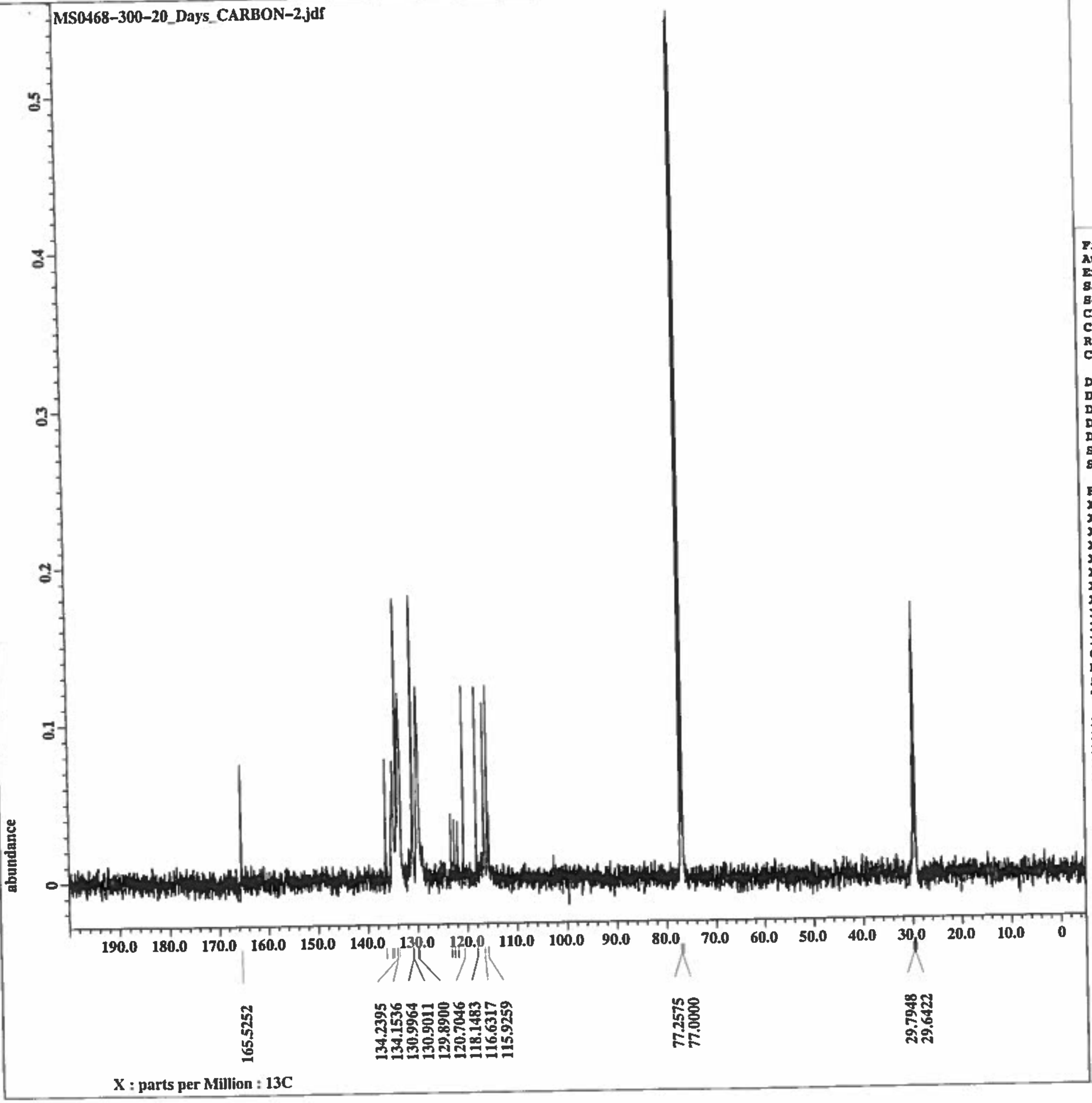

Filename	= MS0468-300-20_Days_CA
Author	= Jim Davis
Experiment	= single_pulse_dec
Sample_id	= MS0468-300-20_Days
Solvent	= CHLOROFORM-D
Changer_sample	= 8
Creation_time	= 27-JUN-2018 17:36:07
Revision_time	= 27-JUN-2018 17:13:13
Current_time	= 27-JUN-2018 17:13:13
Data_format	= 1D COMPLEX
Dim_size	= 26214
Dim_title	= 13C
Dim_units	= [ppm]
Dimensions	= X
Site	= ECA 500
Spectrometer	= JNM-ECA500
Field_strength	= 11.7473579[T] (500[MH
X_acq_duration	= 0.83361792[s]
X_domain	= 13C
X_freq	= 125.76529768[MHz]
X_offset	= 100[ppm]
X_points	= 32768
X_prescans	= 4
X_resolution	= 1.19959034[Hz]
X_sweep	= 39.3081761[kHz]
Irr_domain	= 1H
Irr_freq	= 500.15991521[MHz]
Irr_offset	= 5.0[ppm]
Clipped	= FALSE
Mod_return	= 1
Scans	= 256
Total_scans	= 256
X_90_width	= 13.2[us]
X_acq_time	= 0.83361792[s]
X_angle	= 30[deg]
X_atn	= 6[dB]
X_pulse	= 4.4[us]
Irr_atn_dec	= 20.7[dB]
Irr_atn_noe	= 20.7[dB]
Irr_noise	= WALTZ
Decoupling	= TRUE
Initial_wait	= 1[s]
Noe	= TRUE
Noe_time	= 2[s]
Recvr_gain	= 60
Relaxation_delay	= 2[s]
Repetition_time	= 2.83361792[s]
Temp_get	= 23.2[dC]

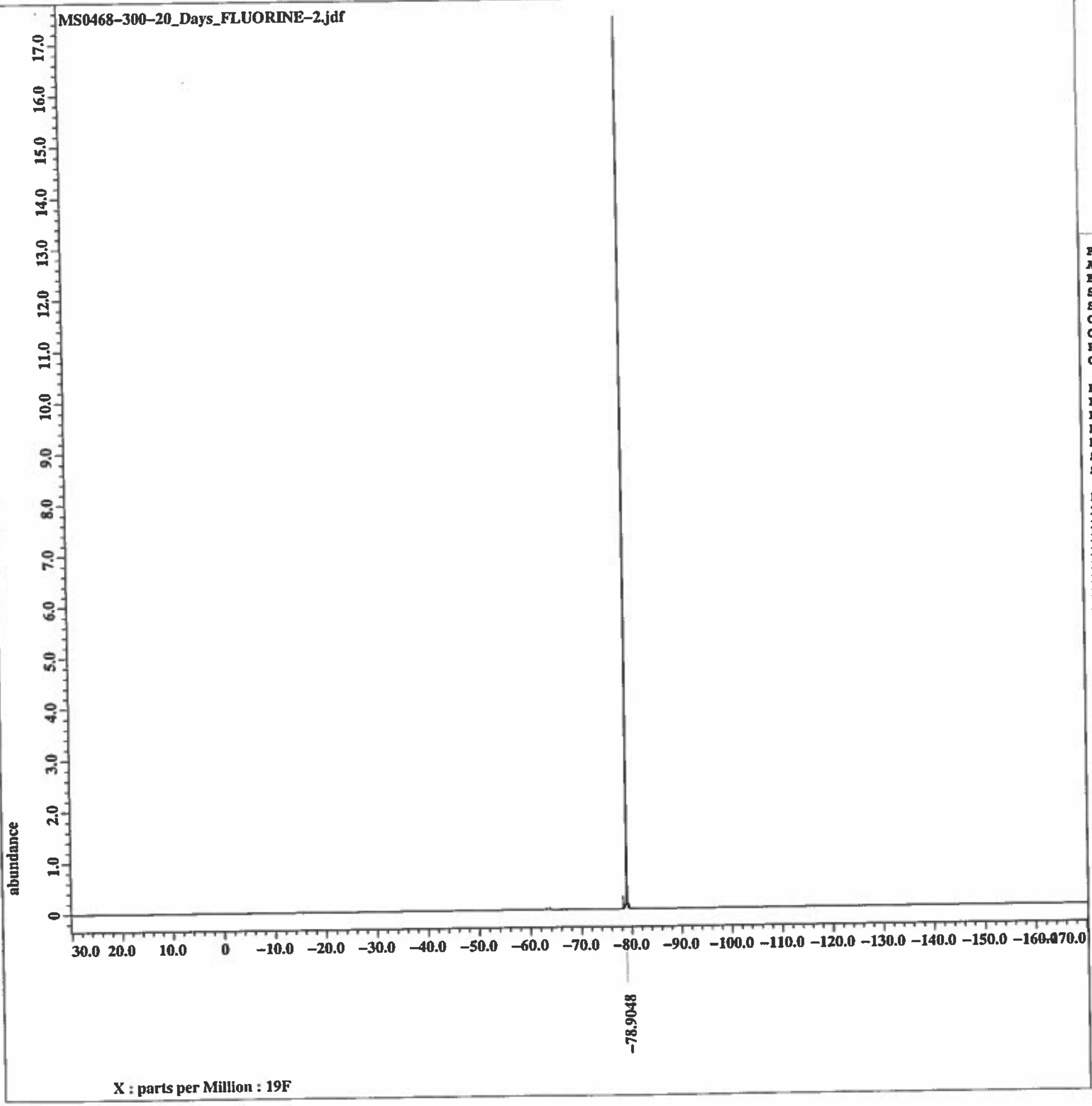

Filename	= MS0468-300-20_Days_FL
Author	= Jim Davis
Experiment	= single_pulse.ex2
Sample_id	= MS0468-300-20_Days
Solvent	= CHLOROFORM-D
Changer_sample	= 8
Creation_time	= 27-JUN-2018 17:39:03
Revision_time	= 27-JUN-2018 17:16:08
Current_time	= 27-JUN-2018 17:16:08
Data_format	= 1D COMPLEX
Dim_size	= 52428
Dim_title	= 19F
Dim_units	= [ppm]
Dimensions	= X
Site	= ECA 500
Spectrometer	= JNM-ECA500
Field_strength	= 11.7473579[T] (500[MH
X_acq_duration	= 0.55574528[s]
X_domain	= 19F
X_freq	= 470.62046084[MHz]
X_offset	= -70[ppm]
X_points	= 65536
X_prescans	= 1
X_resolution	= 1.7993855[Hz]
X_sweep	= 117.9245283[kHz]
Irr_domain	= 19F
Irr_freq	= 470.62046084[MHz]
Irr_offset	= 5[ppm]
Tri_domain	= 19F
Tri_freq	= 470.62046084[MHz]
Tri_offset	= 5[ppm]
Clipped	= FALSE
Mod_return	= 1
Scans	= 16
Total_scans	= 16
X_90_width	= 13.1[us]
X_acq_time	= 0.55574528[s]
X_angle	= 45[deg]
X_atn	= 2.5[dB]
X_pulse	= 6.55[us]
Irr_mode	= Off
Tri_mode	= Off
Dante_presat	= FALSE
Initial_wait	= 1[s]
Recvr_gain	= 36
Relaxation_delay	= 4[s]
Repetition_time	= 4.55574528[s]
Temp_get	= 22.8[dC]

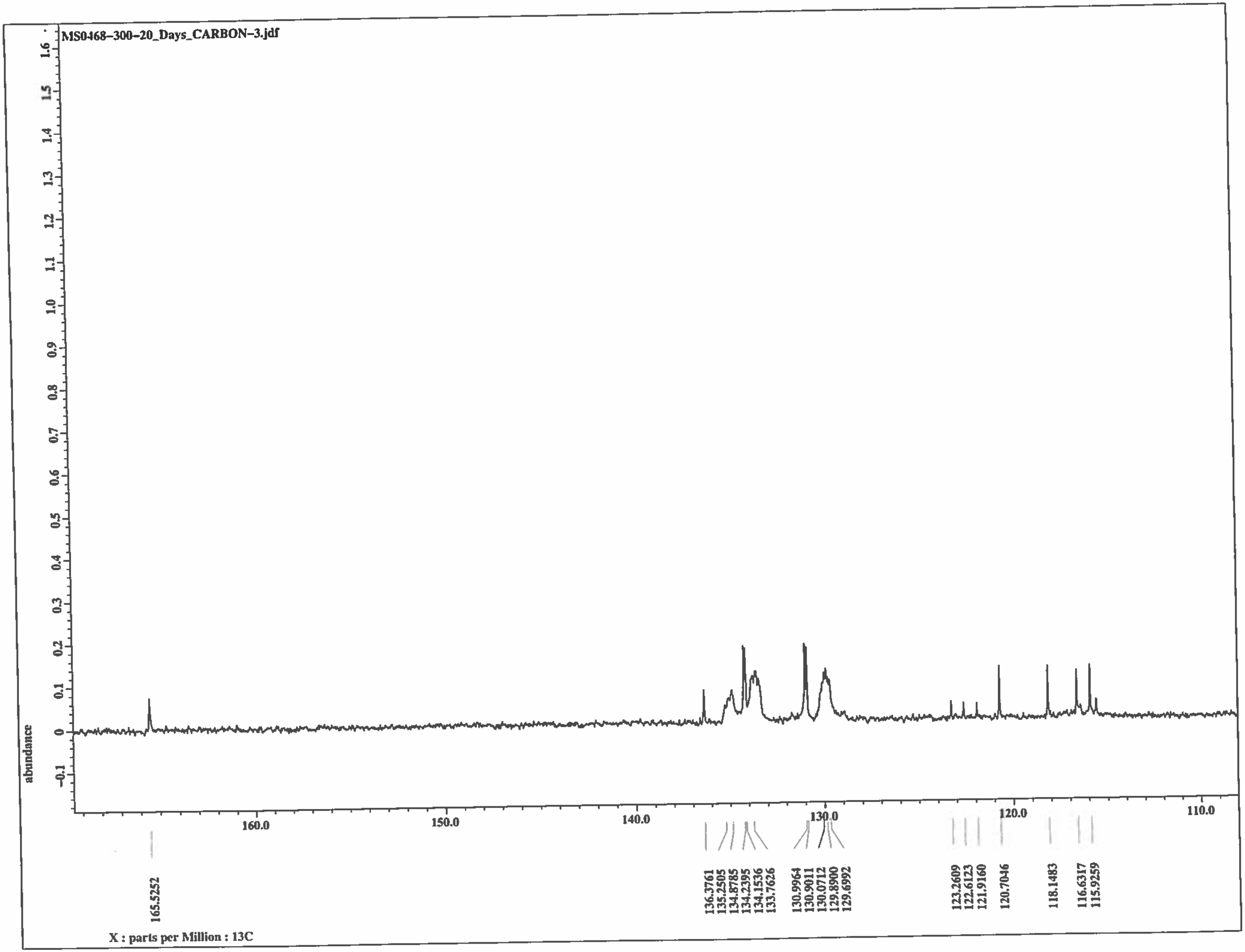
MS0468-300-20_Days_CARBON-3.jdf
abundance
-0.1
0
0.1
0.2
0.3
0.4
0.5
0.6
0.7
0.8
0.9
1.0
1.1
1.2
1.3
1.4
1.5
1.6
160.0
150.0
140.0
130.0
120.0
110.0
165.5252
136.3761
135.2505
134.8785
134.2395
134.1536
133.7626
130.9964
130.9011
130.0712
129.8900
129.6992
123.2609
122.6123
121.9160
120.7046
118.1483
116.6317
115.9259
X : parts per Million : 13C

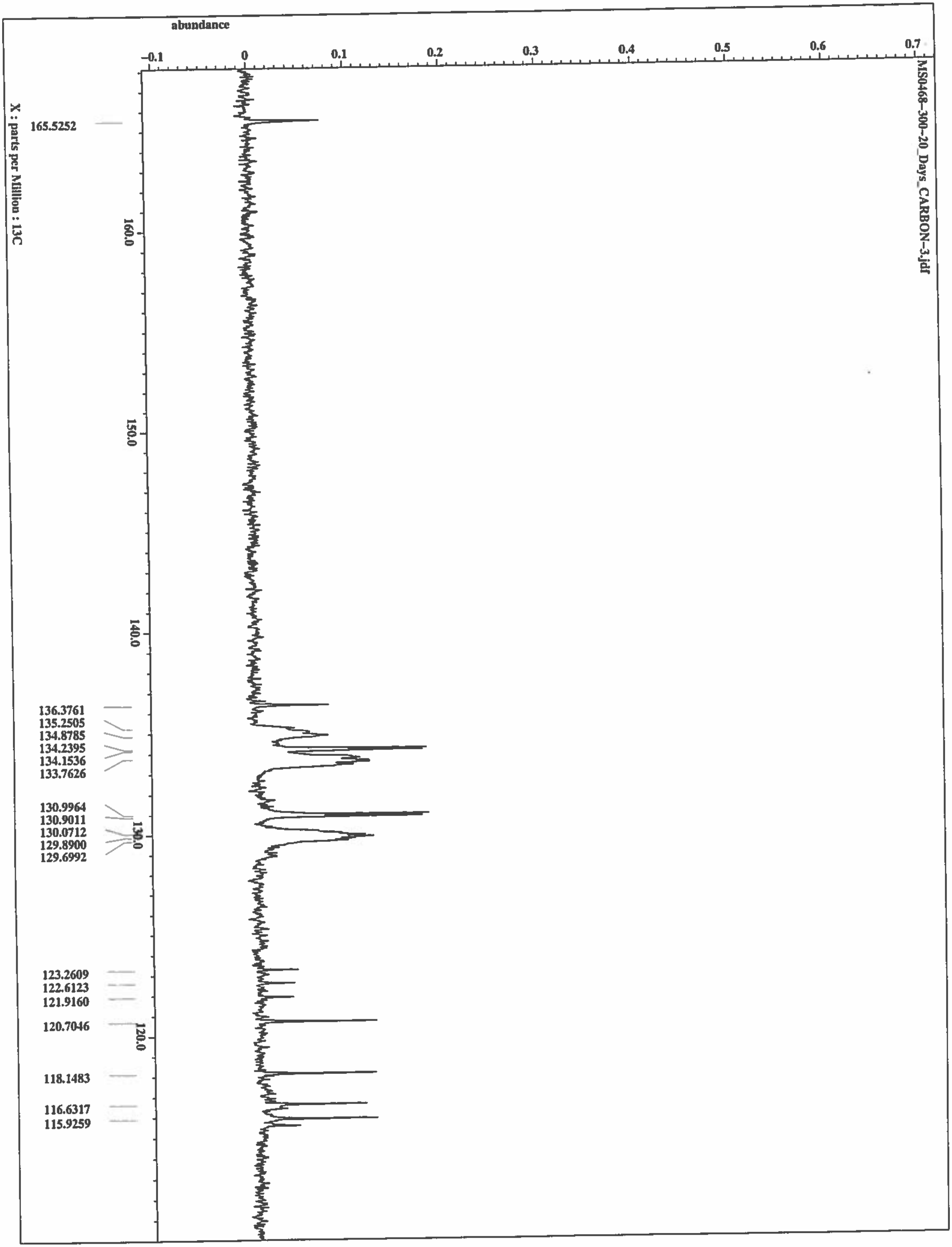
MS0468-300-20 Days_CARBON-3.jdf
abundance
-0.1
0
0.1
0.2
0.3
0.4
0.5
0.6
0.7
X : parts per Million : 13C
160.0
150.0
140.0
130.0
120.0
165.5252
136.3761
135.2505
134.8785
134.2395
134.1536
133.7626
130.9964
130.9011
130.0712
129.8900
129.6992
123.2609
122.6123
121.9160
120.7046
118.1483
116.6317
115.9259

MS0468-300-20_Days_PHOSPHORUS-2.jdf

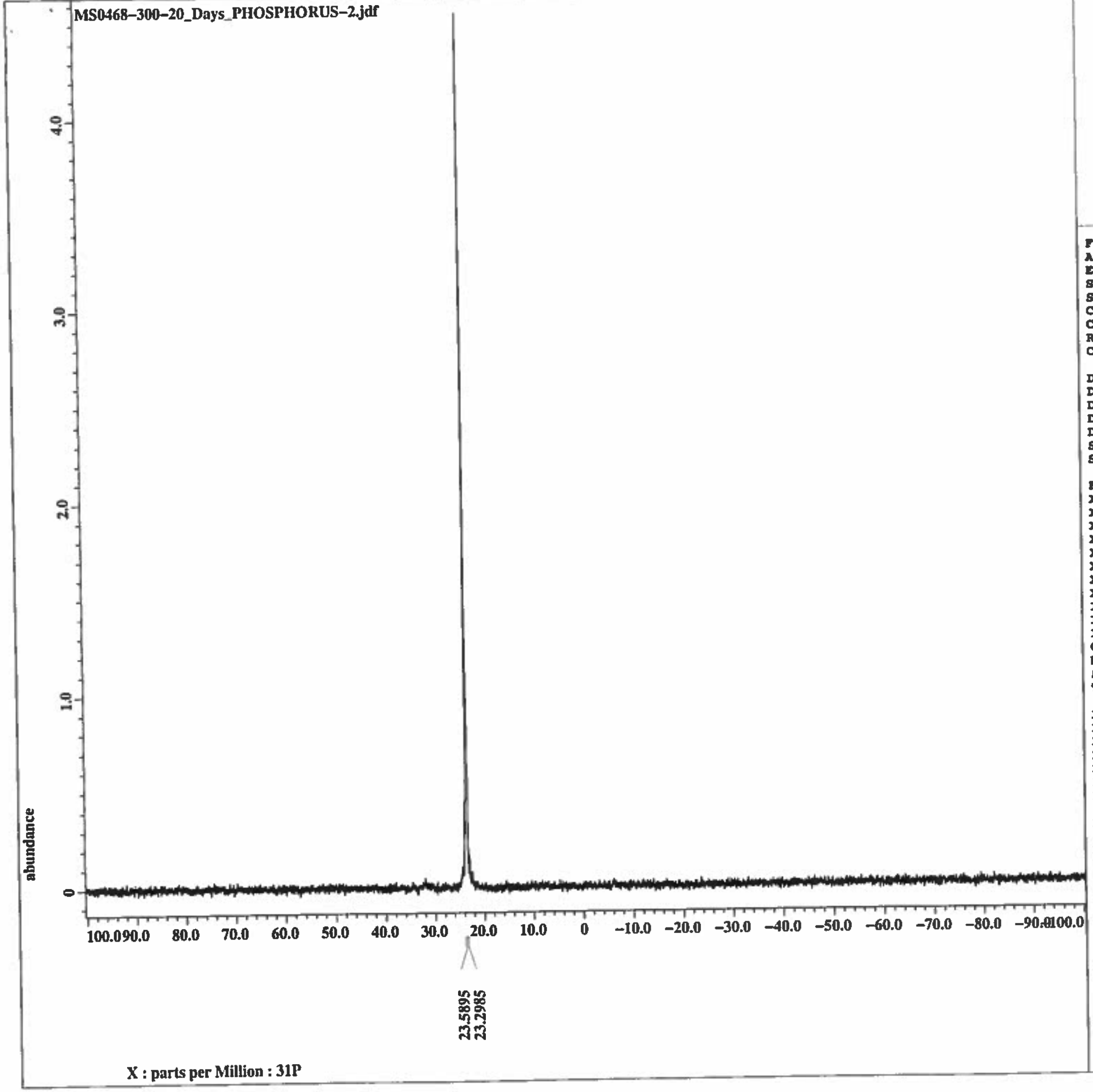

Filename	= MS0468-300-20_Days_PH
Author	= Jim Davis
Experiment	= single_pulse_dec
Sample_id	= MS0468-300-20_Days
Solvent	= CHLOROFORM-D
Changer_sample	= 8
Creation_time	= 27-JUN-2018 17:42:38
Revision_time	= 27-JUN-2018 17:19:43
Current_time	= 27-JUN-2018 17:19:44
Data_format	= 1D COMPLEX
Dim_size	= 26214
Dim_title	= 31P
Dim_units	= [ppm]
Dimensions	= X
Site	= ECA 500
Spectrometer	= JNM-ECA500
Field_strength	= 11.7473579[T] (500[MH
X_acq_duration	= 0.64487424[s]
X_domain	= 31P
X_freq	= 202.46831075[MHz]
X_offset	= 0[ppm]
X_points	= 32768
X_prescans	= 4
X_resolution	= 1.55068995[Hz]
X_sweep	= 50.81300813[kHz]
Irr_domain	= 1H
Irr_freq	= 500.15991521[MHz]
Irr_offset	= 5.0[ppm]
Clipped	= FALSE
Mod_return	= 1
Scans	= 25
Total_scans	= 25
X_90_width	= 14.687[us]
X_acq_time	= 0.64487424[s]
X_angle	= 30[deg]
X_atn	= 5[dB]
X_pulse	= 4.89566667[us]
Irr_atn_dec	= 20.7[dB]
Irr_atn_noe	= 20.7[dB]
Irr_noise	= WALTZ
Decoupling	= TRUE
Initial_wait	= 1[s]
Noe	= TRUE
Noe_time	= 2[s]
Recvr_gain	= 58
Relaxation_delay	= 2[s]
Repetition_time	= 2.64487424[s]
Temp_get	= 23[dC]

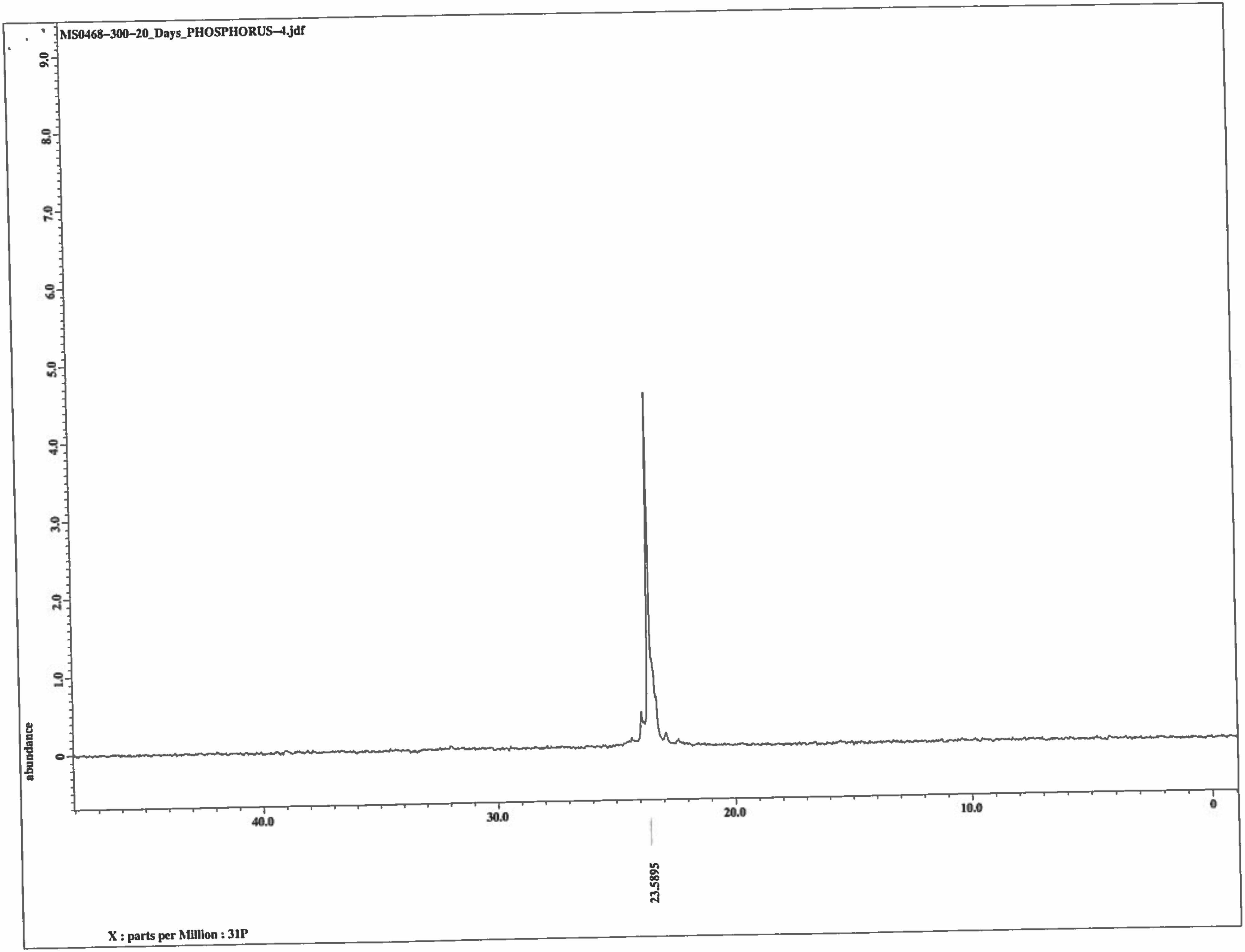
MS0468-300-20_Days_PHOSPHORUS-4.jdf
abundance
0
1.0
2.0
3.0
4.0
5.0
6.0
7.0
8.0
9.0
40.0
30.0
20.0
10.0
0
23.5895
X : parts per Million : 31P

Compound 4 Pre- and Post-heating NMR Spectra

Temperature of Post-heating samples noted in upper left corner of each spectrum

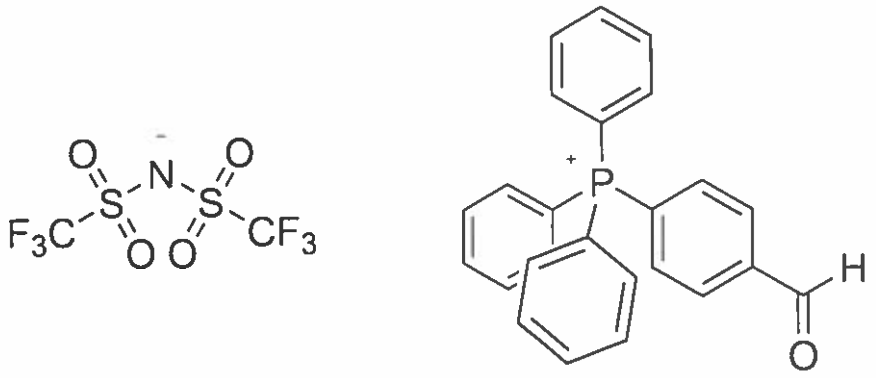

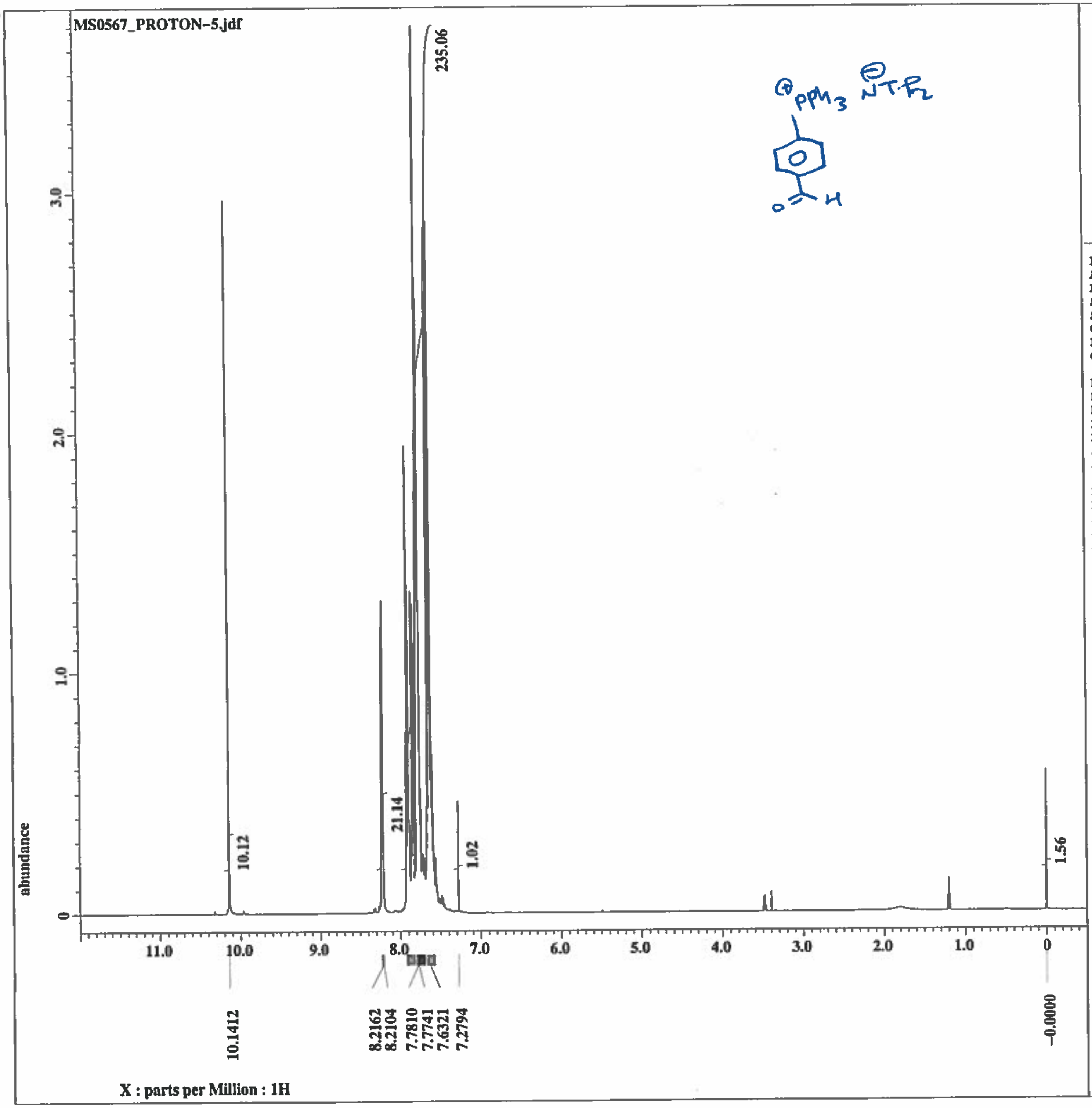

Filename	=	MS0567_PROTON-5.jdf
Author	=	Jim Davis
Experiment	=	single_pulse.ex2
Sample_id	=	MS0567
Solvent	=	CHLOROFORM-D
Creation_time	=	3-OCT-2018 19:18:04
Revision_time	=	3-OCT-2018 18:53:06
Current_time	=	3-OCT-2018 18:53:06
Data_format	=	1D COMPLEX
Dim_size	=	13107
Dim_title	=	1H
Dim_units	=	[ppm]
Dimensions	=	X
Site	=	ECA 500
Spectrometer	=	JNM-ECA500
Field_strength	=	11.7473579[T] (500[MH
X_acq_duration	=	1.74587904[s]
X_domain	=	1H
X_freq	=	500.15991521[MHz]
X_offset	=	5.0[ppm]
X_points	=	16384
X_prescans	=	1
X_resolution	=	0.57277737[Hz]
X_sweep	=	9.38438438[kHz]
Irr_domain	=	1H
Irr_freq	=	500.15991521[MHz]
Irr_offset	=	5.0[ppm]
Tri_domain	=	1H
Tri_freq	=	500.15991521[MHz]
Tri_offset	=	5.0[ppm]
Clipped	=	FALSE
Mod_return	=	1
Scans	=	16
Total_scans	=	16
X_90_width	=	12.4[us]
X_acq_time	=	1.74587904[s]
X_angle	=	45[deg]
X_atn	=	4[dB]
X_pulse	=	6.2[us]
Irr_mode	=	Off
Tri_mode	=	Off
Dante_presat	=	FALSE
Initial_wait	=	1[s]
Recvr_gain	=	30
Relaxation_delay	=	4[s]
Repetition_time	=	5.74587904[s]
Temp_get	=	22.4[dC]

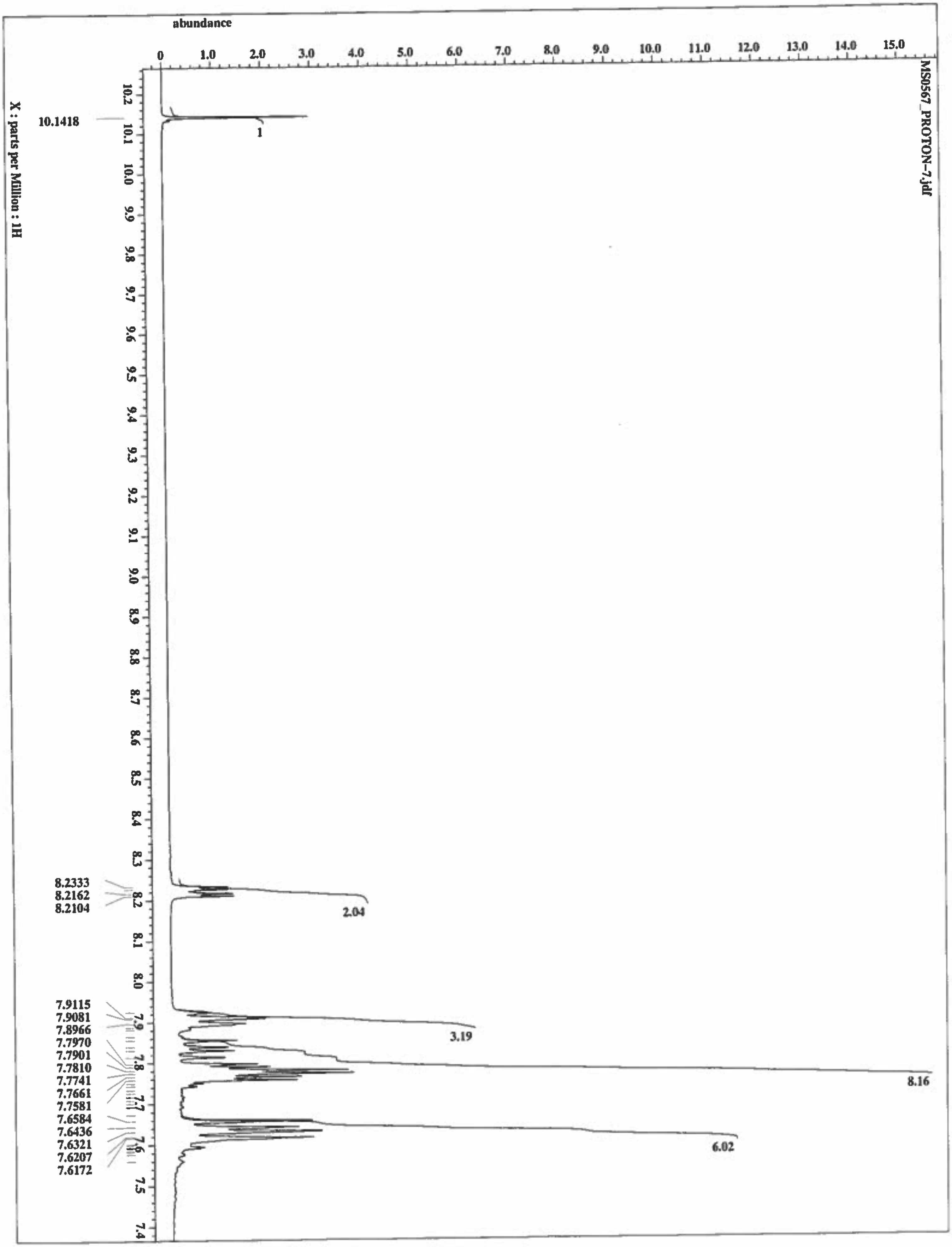

abundance
MS0567_PROTON-7.jdf
X : parts per Million : 1H
10.1418
8.2333
8.2162
8.2104
7.9115
7.9081
7.8966
7.7970
7.7901
7.7810
7.7741
7.7661
7.7581
7.6584
7.6436
7.6321
7.6207
7.6172
1
2.04
3.19
8.16
6.02

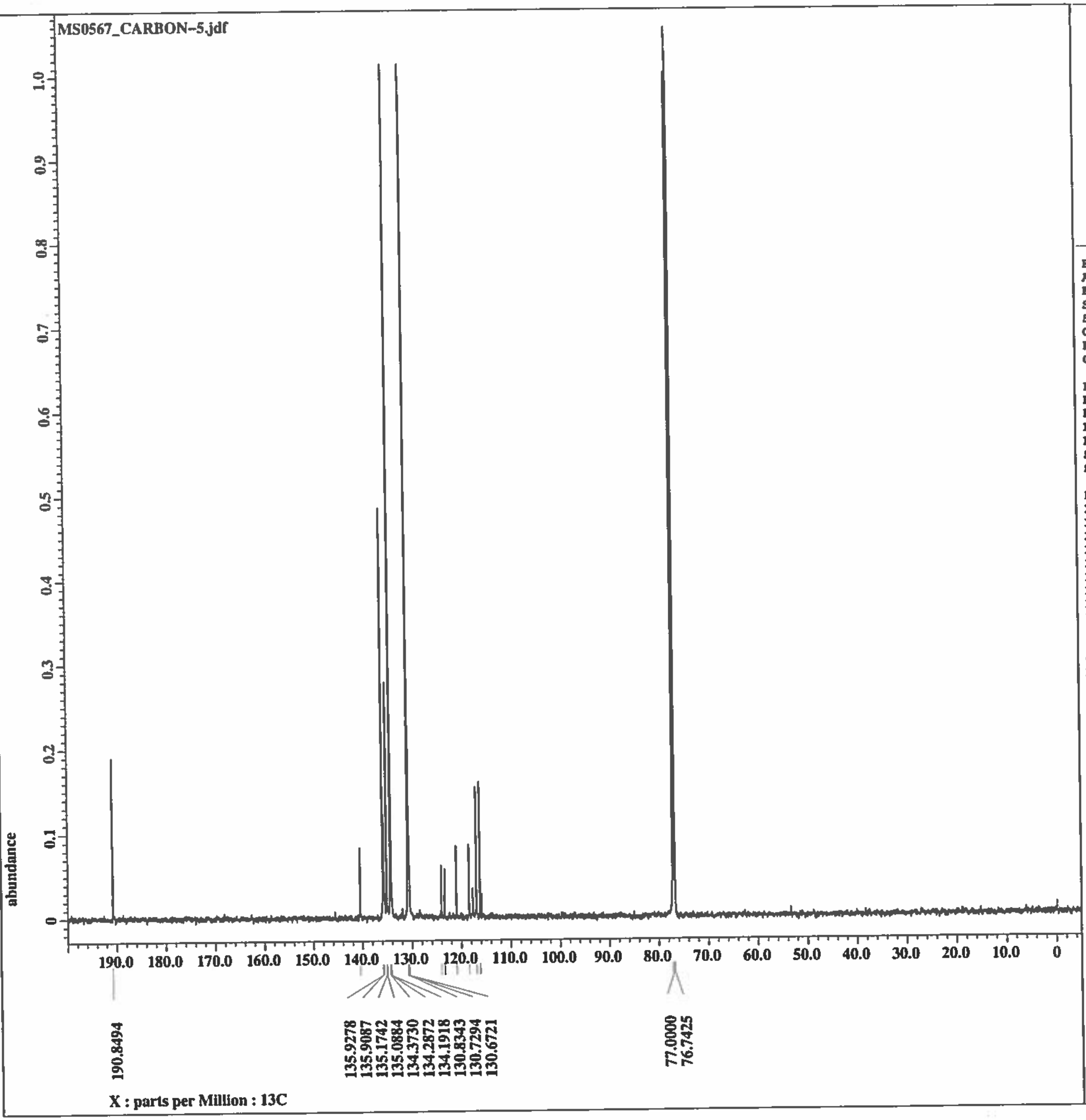

Filename	= MS0567_CARBON-5.jdf
Author	= Jim Davis
Experiment	= single_pulse_dec
Sample_id	= MS0567
Solvent	= CHLOROFORM-D
Creation_time	= 3-OCT-2018 20:08:47
Revision_time	= 3-OCT-2018 19:43:49
Current_time	= 3-OCT-2018 19:43:49
Data_format	= 1D COMPLEX
Dim_size	= 26214
Dim_title	= 13C
Dim_units	= [ppm]
Dimensions	= X
Site	= ECA 500
Spectrometer	= JNM-ECA500
Field_strength	= 11.7473579[T] (500[MH
X_acq_duration	= 0.83361792[s]
X_domain	= 13C
X_freq	= 125.76529768[MHz]
X_offset	= 100[ppm]
X_points	= 32768
X_prescans	= 4
X_resolution	= 1.19959034[Hz]
X_sweep	= 39.3081761[kHz]
Irr_domain	= 1H
Irr_freq	= 500.15991521[MHz]
Irr_offset	= 5.0[ppm]
Clipped	= FALSE
Mod_return	= 1
Scans	= 1024
Total_scans	= 1024
X_90_width	= 13.2[us]
X_acq_time	= 0.83361792[s]
X_angle	= 30[deg]
X_atn	= 6[dB]
X_pulse	= 4.4[us]
Irr_atn_dec	= 20.7[dB]
Irr_atn_noe	= 20.7[dB]
Irr_noise	= WALTZ
Decoupling	= TRUE
Initial_wait	= 1[s]
Noe	= TRUE
Noe_time	= 2[s]
Recvr_gain	= 60
Relaxation_delay	= 2[s]
Repetition_time	= 2.83361792[s]
Temp_get	= 23.1[dC]

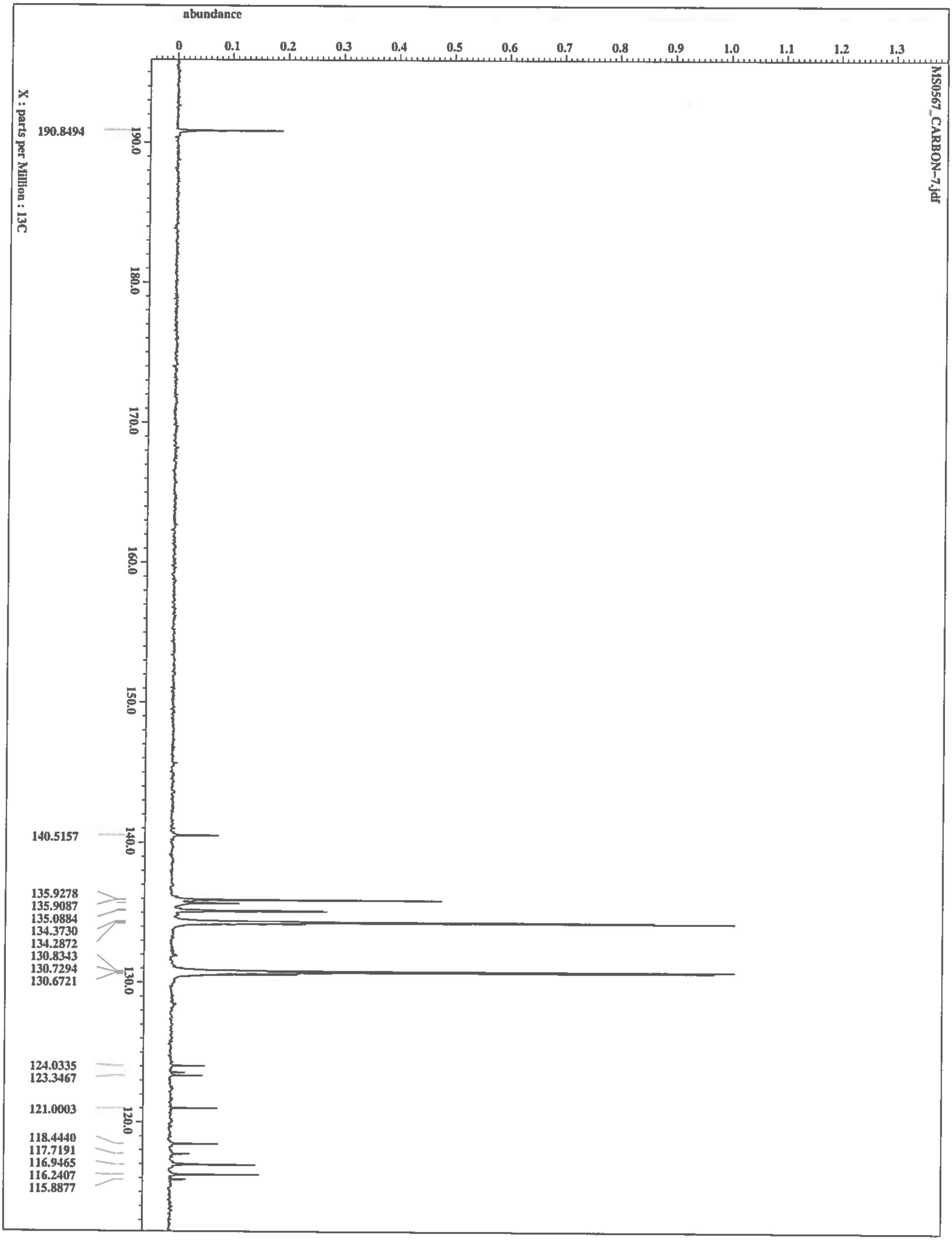

abundance
0
0.1
0.2
0.3
0.4
0.5
0.6
0.7
0.8
0.9
1.0
1.1
1.2
1.3
X : parts per Million : 13C
MS0567_CARBON-7.jdf
190.8494
140.5157
135.9278
135.9087
135.0884
134.3730
134.2872
130.8343
130.7294
130.6721
124.0335
123.3467
121.0003
118.4440
117.7191
116.9465
116.2407
115.8877
190.0
180.0
170.0
160.0
150.0
140.0
130.0
120.0

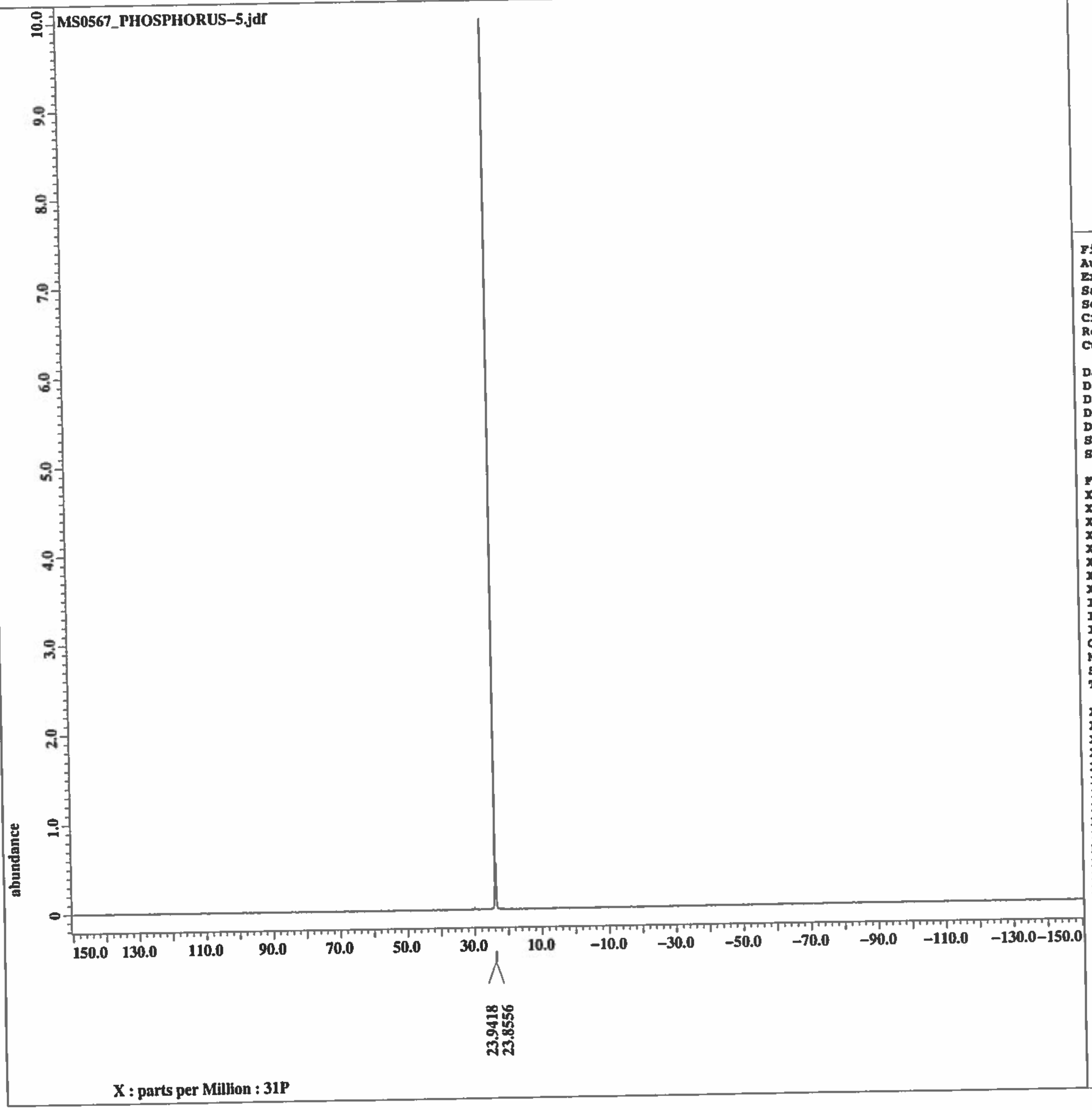

Filename	= MS0567_PHOSPHORUS-5.j
Author	= Jim Davis
Experiment	= single_pulse_dec
Sample_id	= MS0567
Solvent	= CHLOROFORM-D
Creation_time	= 4-OCT-2018 01:35:23
Revision_time	= 4-OCT-2018 01:10:23
Current_time	= 4-OCT-2018 01:10:23
Data_format	= 1D COMPLEX
Dim_size	= 52428
Dim_title	= 31P
Dim_units	= [ppm]
Dimensions	= X
Site	= ECA 500
Spectrometer	= JNM-ECA500
Field_strength	= 11.7473579[T] (500[MH
X_acq_duration	= 0.85983232[s]
X_domain	= 31P
X_freq	= 202.46831075[MHz]
X_offset	= 0[ppm]
X_points	= 65536
X_prescans	= 4
X_resolution	= 1.16301746[Hz]
X_sweep	= 76.2195122[kHz]
Irr_domain	= 1H
Irr_freq	= 500.15991521[MHz]
Irr_offset	= 5.0[ppm]
Clipped	= FALSE
Mod_return	= 1
Scans	= 128
Total_scans	= 128
X_90_width	= 14.687[us]
X_acq_time	= 0.85983232[s]
X_angle	= 30[deg]
X_atn	= 5[dB]
X_pulse	= 4.89566667[us]
Irr_atn_dec	= 20.7[dB]
Irr_atn_noe	= 20.7[dB]
Irr_noise	= WALTZ
Decoupling	= TRUE
Initial_wait	= 1[s]
Noe	= TRUE
Noe_time	= 2[s]
Recvr_gain	= 56
Relaxation_delay	= 2[s]
Repetition_time	= 2.85983232[s]
Temp_get	= 23.2[dC]

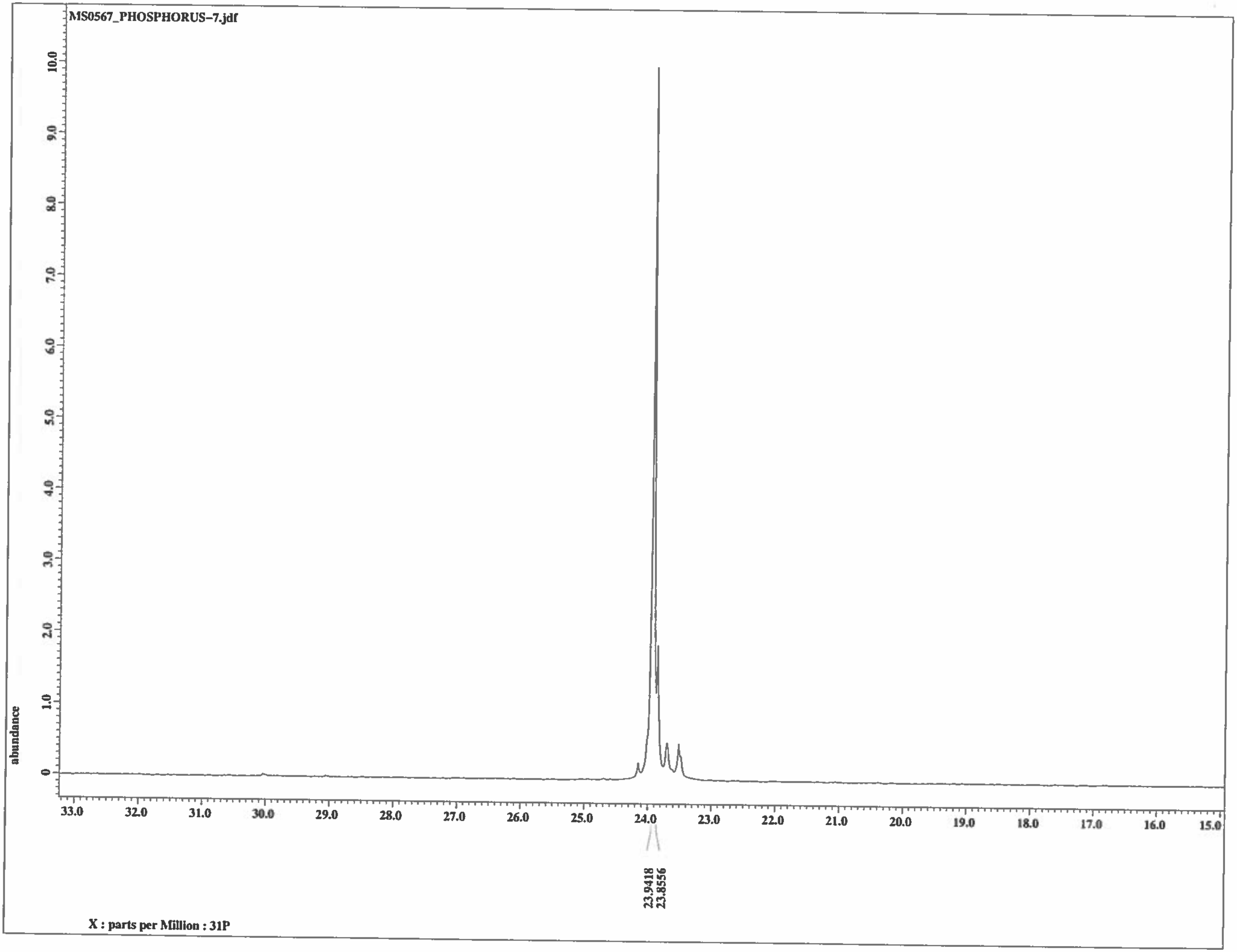
MS0567_PHOSPHORUS-7.jdf
abundance
0
1.0
2.0
3.0
4.0
5.0
6.0
7.0
8.0
9.0
10.0
33.0
32.0
31.0
30.0
29.0
28.0
27.0
26.0
25.0
24.0
23.0
22.0
21.0
20.0
19.0
18.0
17.0
16.0
15.0
23.9418
23.8556
X : parts per Million : 31P

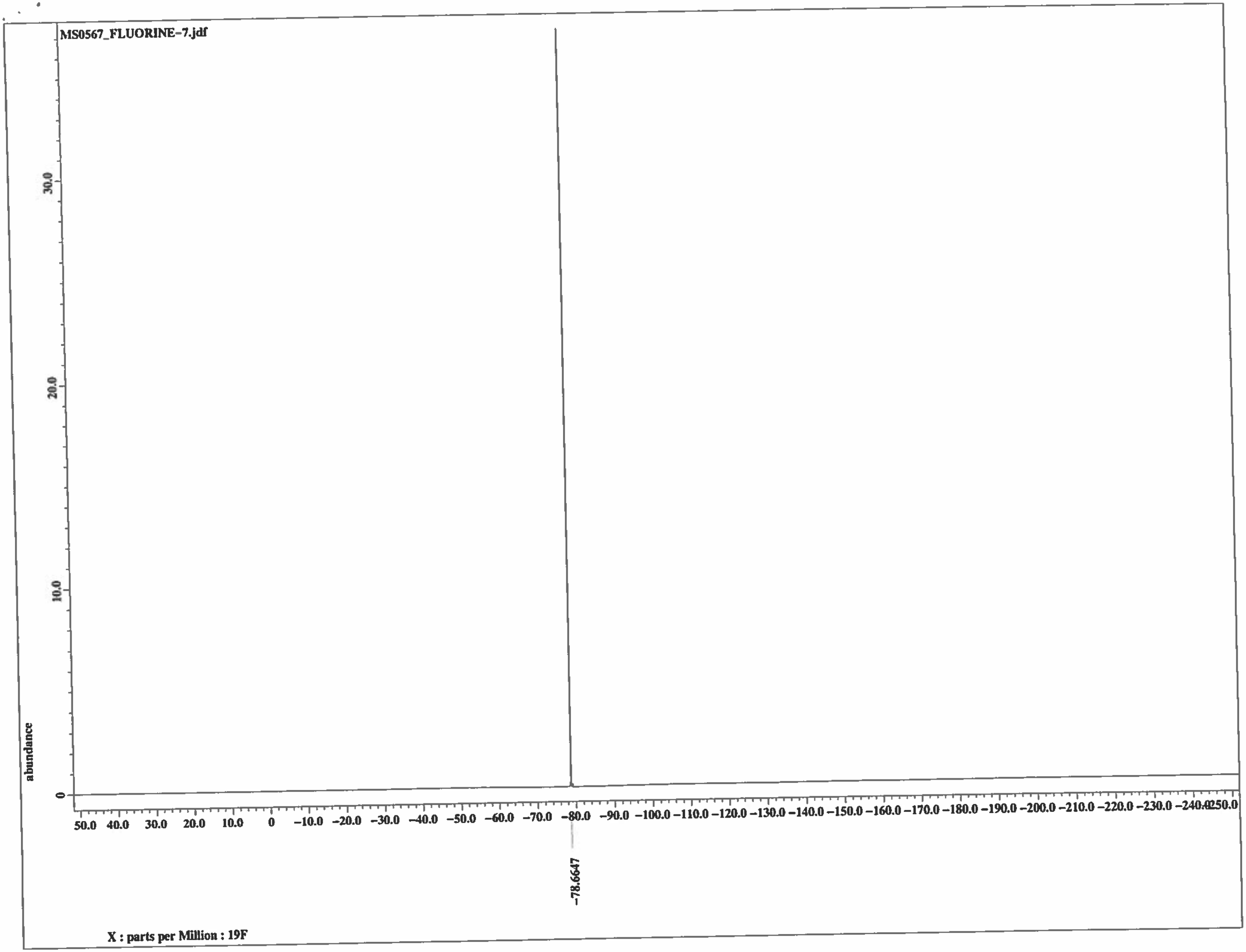
MS0567_FLUORINE-7.jdf
abundance
0
10.0
20.0
30.0
50.0 40.0 30.0 20.0 10.0 0 −10.0 −20.0 −30.0 −40.0 −50.0 −60.0 −70.0 −80.0 −90.0 −100.0 −110.0 −120.0 −130.0 −140.0 −150.0 −160.0 −170.0 −180.0 −190.0 −200.0 −210.0 −220.0 −230.0 −240.0 −250.0
−78.6647
X : parts per Million : 19F

MS0567_FLUORINE-5.jdf

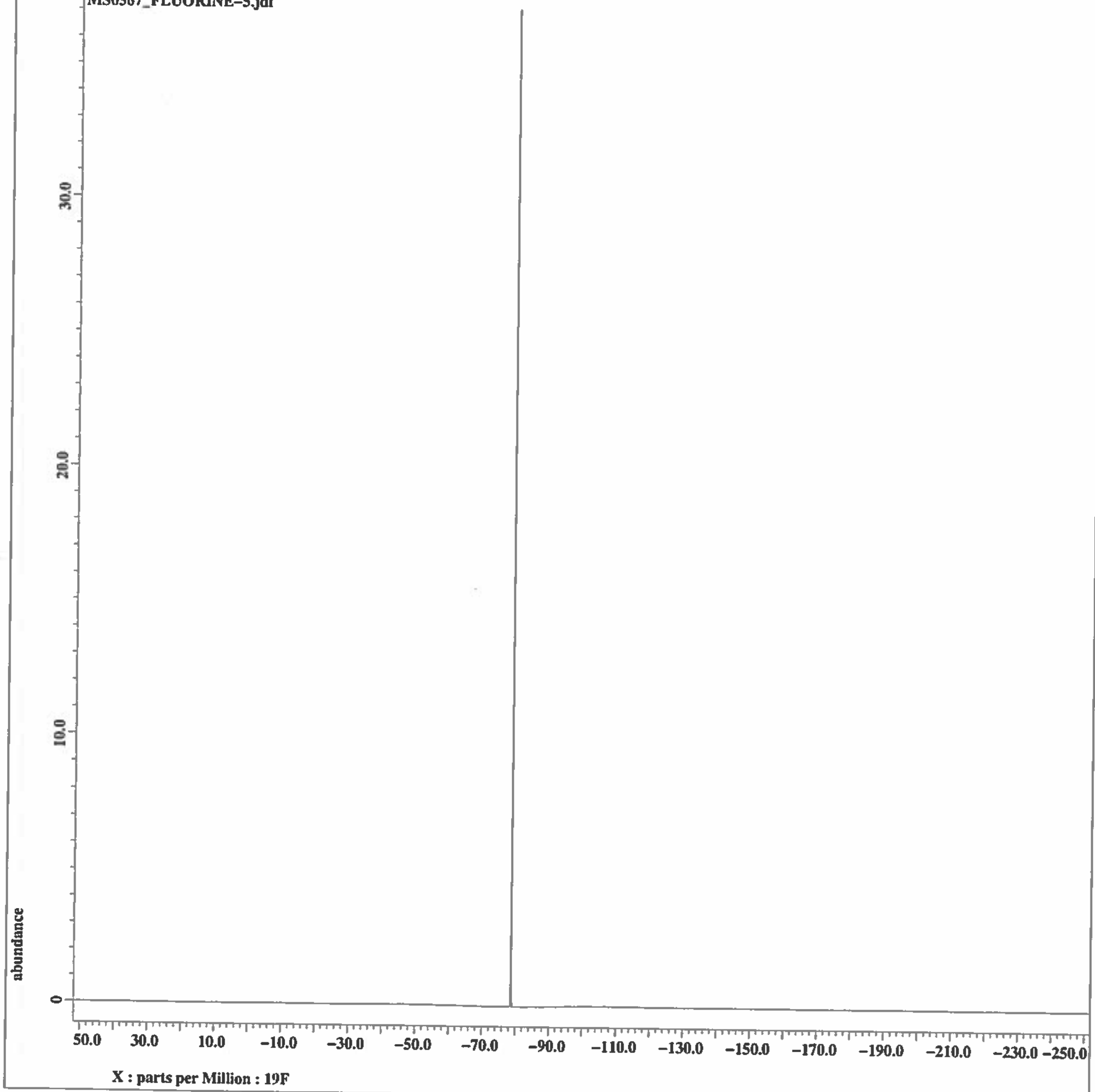


```
Filename            = MS0567_FLUORINE-5.jdf
Author              = Jim Davis
Experiment          = single_pulse.ex2
Sample_id           = MS0567
Solvent             = CHLOROFORM-D
Creation_time       =  4-OCT-2018 01:41:31
Revision_time       =  4-OCT-2018 01:16:32
Current_time        =  4-OCT-2018 01:16:33

Data_format         = 1D COMPLEX
Dim_size            = 104857
Dim_title           = 19F
Dim_units           = [ppm]
Dimensions          = X
Site                = ECA 500
Spectrometer        = JNM-ECA500

Field_strength      = 11.7473579[T] (500[MH
X_acq_duration      = 0.7340032[s]
X_domain            = 19F
X_freq              = 470.62046084[MHz]
X_offset            = -100[ppm]
X_points            = 131072
X_prescans          = 1
X_resolution        = 1.36239188[Hz]
X_sweep             = 178.57142857[kHz]
Irr_domain          = 19F
Irr_freq            = 470.62046084[MHz]
Irr_offset          = 5[ppm]
Tri_domain          = 19F
Tri_freq            = 470.62046084[MHz]
Tri_offset          = 5[ppm]
Clipped             = FALSE
Mod_return          = 1
Scans               = 50
Total_scans         = 50

X_90_width          = 13.1[us]
X_acq_time          = 0.7340032[s]
X_angle             = 45[deg]
X_atn               = 2.5[dB]
X_pulse             = 6.55[us]
Irr_mode            = Off
Tri_mode            = Off
Dante_presat        = FALSE
Initial_wait        = 1[s]
Recvr_gain          = 54
Relaxation_delay    = 4[s]
Repetition_time     = 4.7340032[s]
Temp_get            = 22.8[dC]
```

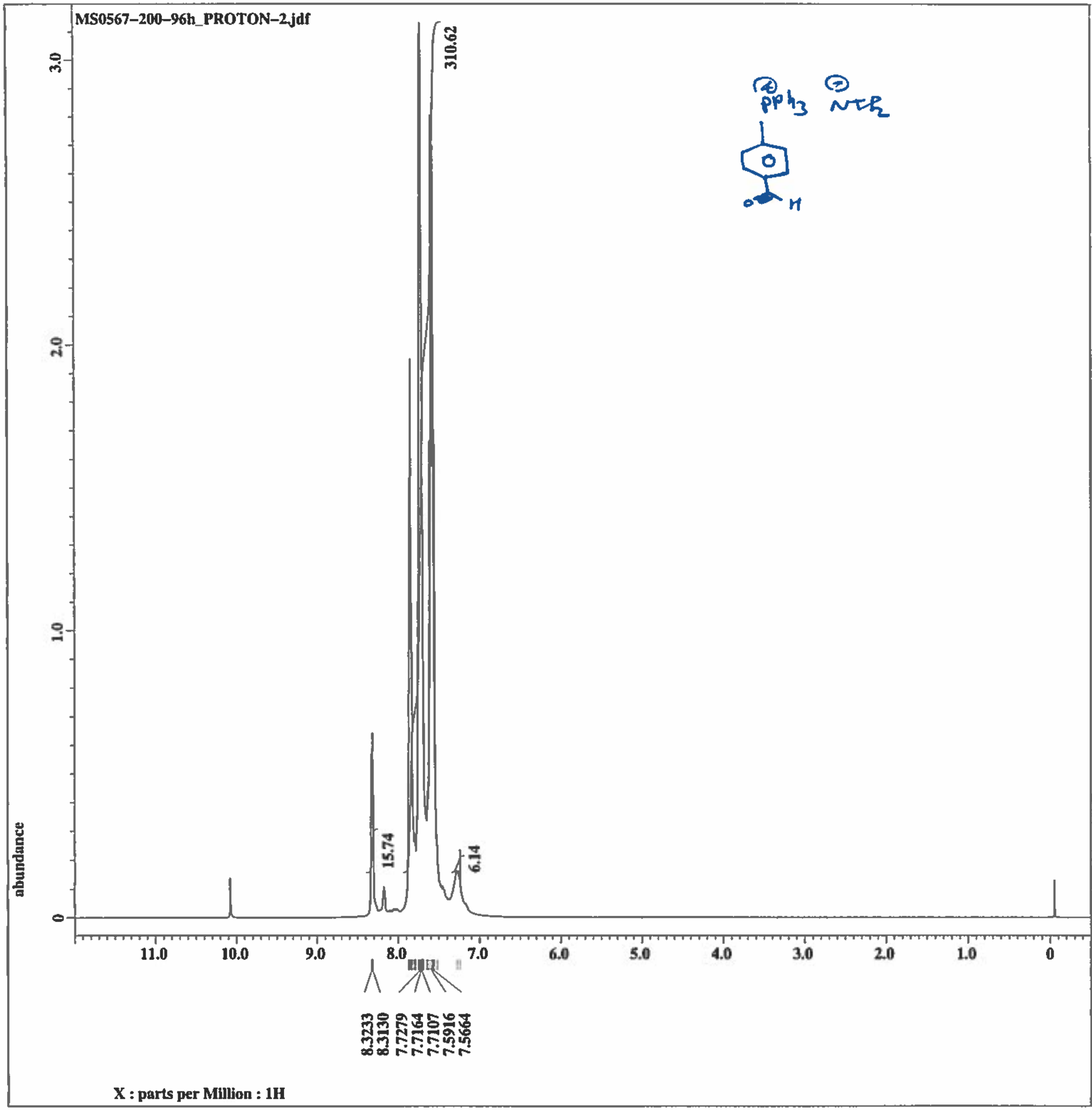

Filename	= MS0567-200-96h_PROTON
Author	= Jim Davis
Experiment	= single_pulse.ex2
Sample_id	= MS0567-200-96h
Solvent	= CHLOROFORM-D
Creation_time	= 8-OCT-2018 09:48:43
Revision_time	= 8-OCT-2018 09:23:22
Current_time	= 8-OCT-2018 09:23:22
Data_format	= 1D COMPLEX
Dim_size	= 13107
Dim_title	= 1H
Dim_units	= [ppm]
Dimensions	= X
Site	= ECA 500
Spectrometer	= JNM-ECA500
Field_strength	= 11.7473579[T] (500[MH
X_acq_duration	= 1.74587904[s]
X_domain	= 1H
X_freq	= 500.15991521[MHz]
X_offset	= 5.0[ppm]
X_points	= 16384
X_prescans	= 1
X_resolution	= 0.57277737[Hz]
X_sweep	= 9.38438438[kHz]
Irr_domain	= 1H
Irr_freq	= 500.15991521[MHz]
Irr_offset	= 5.0[ppm]
Tri_domain	= 1H
Tri_freq	= 500.15991521[MHz]
Tri_offset	= 5.0[ppm]
Clipped	= FALSE
Mod_return	= 1
Scans	= 16
Total_scans	= 16
X_90_width	= 12.4[us]
X_acq_time	= 1.74587904[s]
X_angle	= 45[deg]
X_atn	= 4[dB]
X_pulse	= 6.2[us]
Irr_mode	= Off
Tri_mode	= Off
Dante_presat	= FALSE
Initial_wait	= 1[s]
Recvr_gain	= 30
Relaxation_delay	= 4[s]
Repetition_time	= 5.74587904[s]
Temp_get	= 22.9[dC]

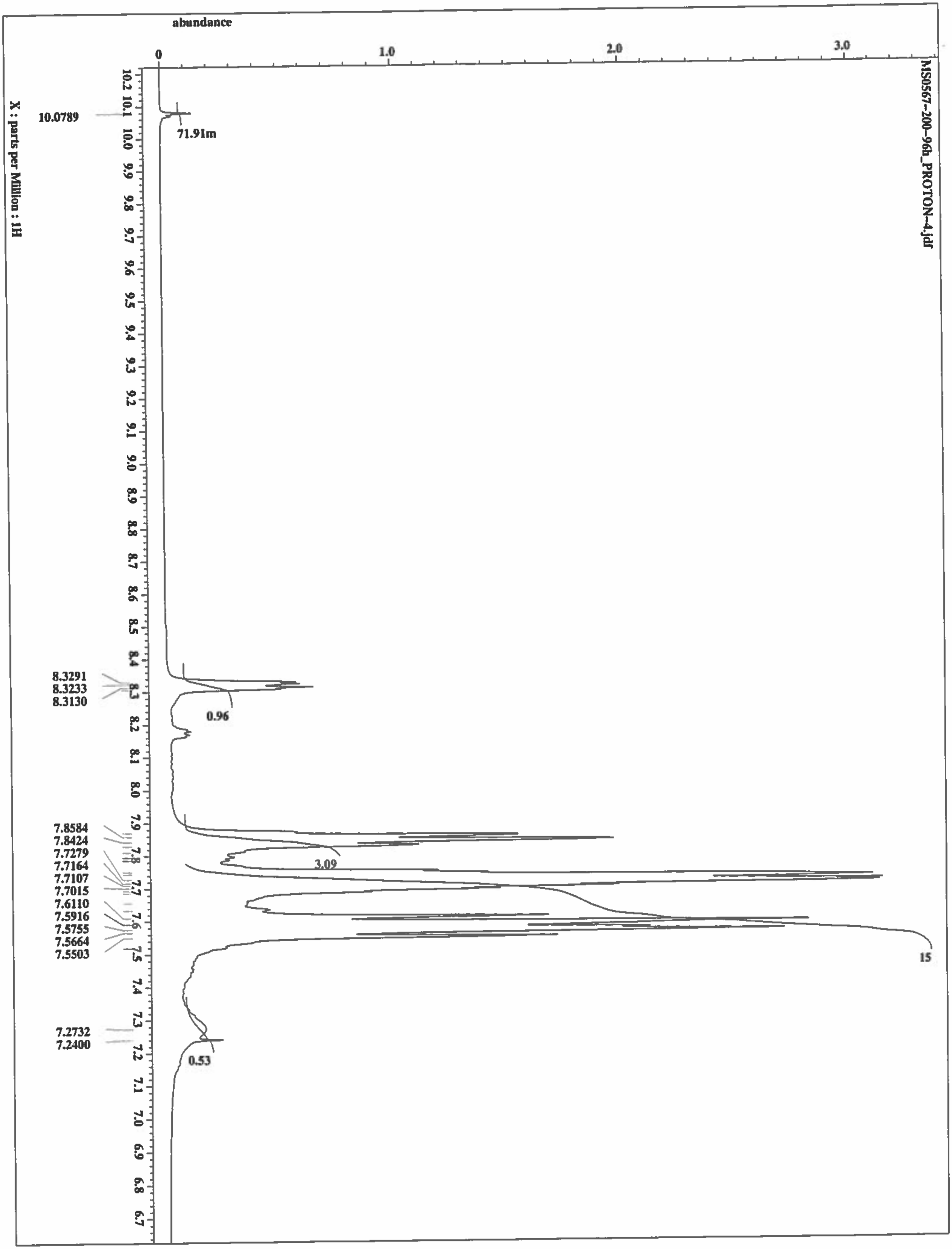

abundance
0
1.0
2.0
3.0
MS0567-200-96h_PROTON-4.jdf
X : parts per Million : 1H
10.0789
71.91m
8.3291
8.3233
8.3130
0.96
7.8584
7.8424
7.7279
7.7164
7.7107
7.7015
7.6110
7.5916
7.5755
7.5664
7.5503
3.09
15
7.2732
7.2400
0.53
10.2 10.1 10.0 9.9 9.8 9.7 9.6 9.5 9.4 9.3 9.2 9.1 9.0 8.9 8.8 8.7 8.6 8.5 8.4 8.3 8.2 8.1 8.0 7.9 7.8 7.7 7.6 7.5 7.4 7.3 7.2 7.1 7.0 6.9 6.8 6.7

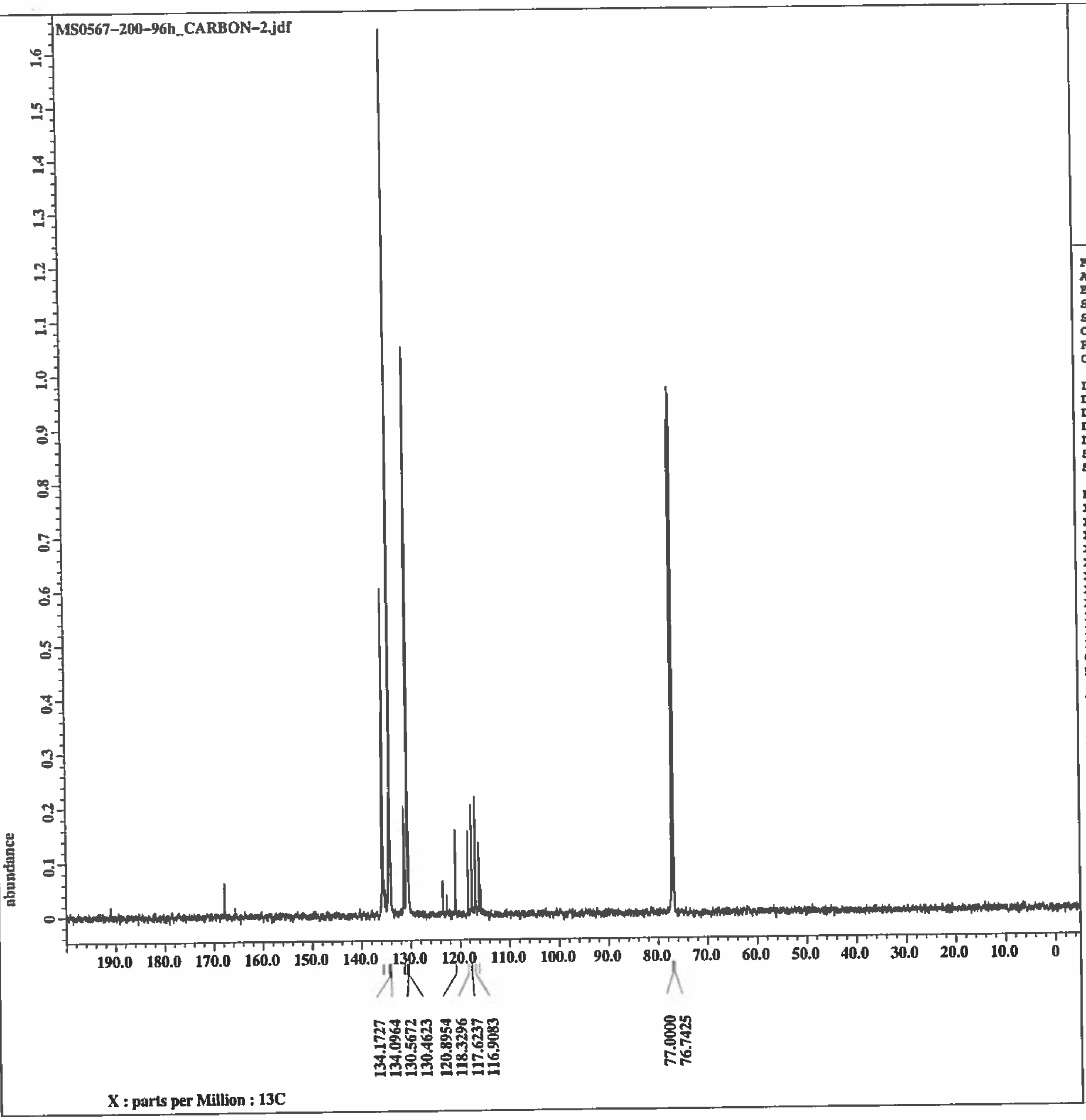

Filename	=	MS0567-200-96h_CARBON
Author	=	Jim Davis
Experiment	=	single_pulse_dec
Sample_id	=	MS0567-200-96h
Solvent	=	CHLOROFORM-D
Creation_time	=	8-OCT-2018 10:02:05
Revision_time	=	8-OCT-2018 09:36:43
Current_time	=	8-OCT-2018 09:36:43
Data_format	=	1D COMPLEX
Dim_size	=	26214
Dim_title	=	13C
Dim_units	=	[ppm]
Dimensions	=	X
Site	=	ECA 500
Spectrometer	=	JNM-ECA500
Field_strength	=	11.7473579[T] (500[MH
X_acq_duration	=	0.83361792[s]
X_domain	=	13C
X_freq	=	125.76529768[MHz]
X_offset	=	100[ppm]
X_points	=	32768
X_prescans	=	4
X_resolution	=	1.19959034[Hz]
X_sweep	=	39.3081761[kHz]
Irr_domain	=	1H
Irr_freq	=	500.15991521[MHz]
Irr_offset	=	5.0[ppm]
Clipped	=	FALSE
Mod_return	=	1
Scans	=	256
Total_scans	=	256
X_90_width	=	13.2[us]
X_acq_time	=	0.83361792[s]
X_angle	=	30[deg]
X_atn	=	6[dB]
X_pulse	=	4.4[us]
Irr_atn_dec	=	20.7[dB]
Irr_atn_noe	=	20.7[dB]
Irr_noise	=	WALTZ
Decoupling	=	TRUE
Initial_wait	=	1[s]
Noe	=	TRUE
Noe_time	=	2[s]
Recvr_gain	=	60
Relaxation_delay	=	2[s]
Repetition_time	=	2.83361792[s]
Temp_get	=	22.9[dC]

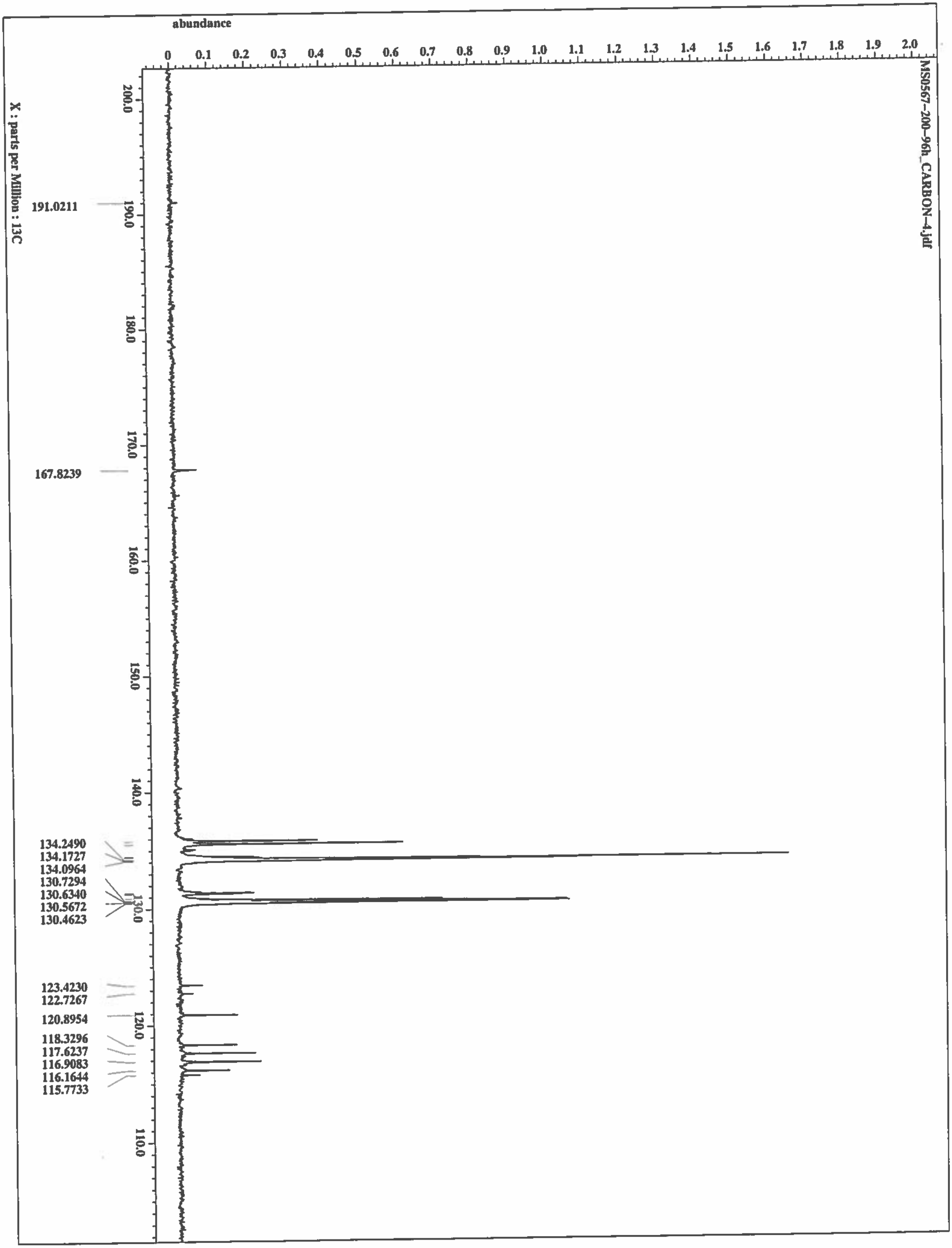

abundance
0 0.1 0.2 0.3 0.4 0.5 0.6 0.7 0.8 0.9 1.0 1.1 1.2 1.3 1.4 1.5 1.6 1.7 1.8 1.9 2.0
MS0567–200–96h_CARBON–4.jdf
X : parts per Million : 13C
200.0
190.0
180.0
170.0
160.0
150.0
140.0
130.0
120.0
110.0
191.0211
167.8239
134.2490
134.1727
134.0964
130.7294
130.6340
130.5672
130.4623
123.4230
122.7267
120.8954
118.3296
117.6237
116.9083
116.1644
115.7733

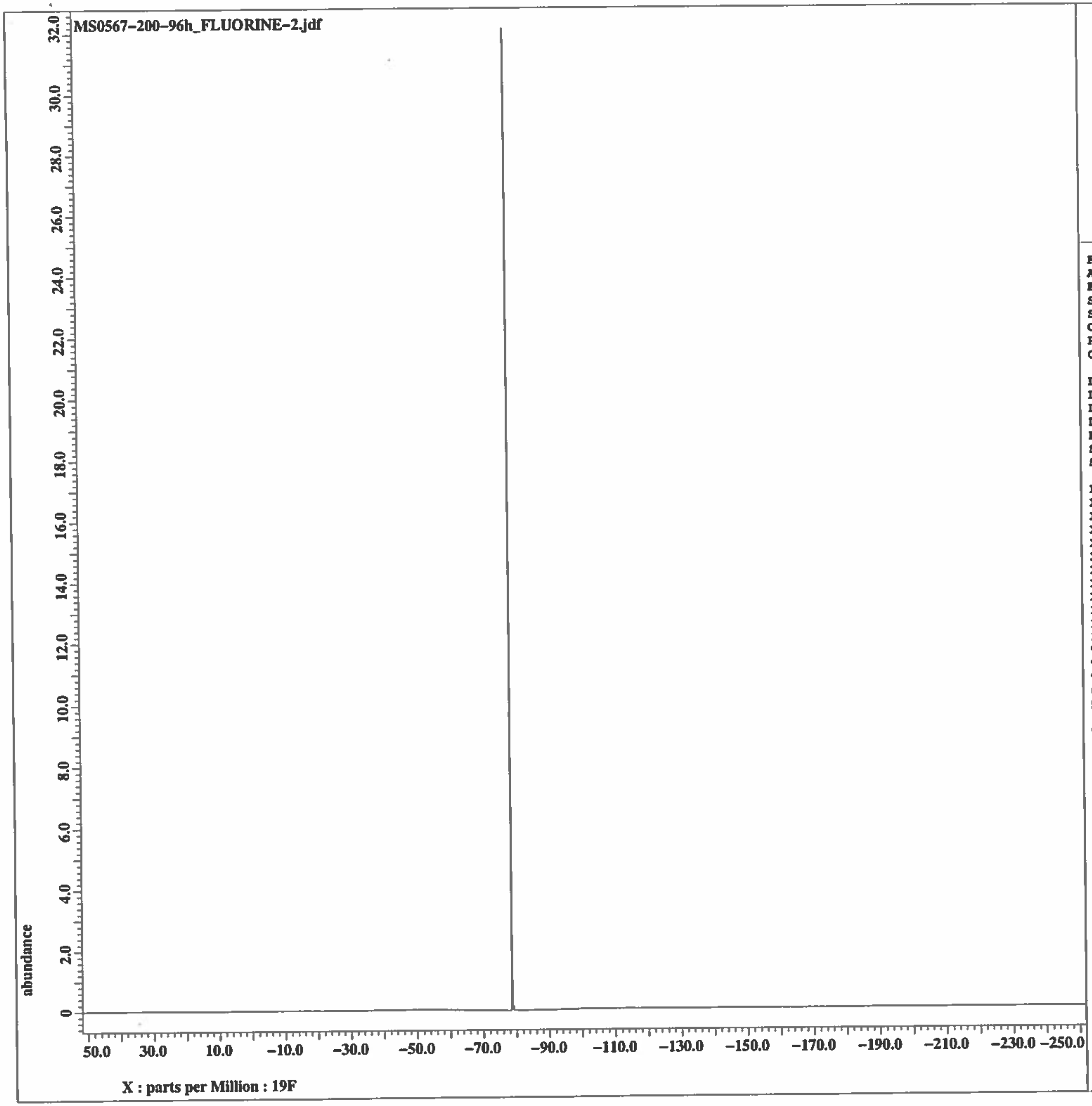

Filename	= MS0567-200-96h_FLUORI
Author	= Jim Davis
Experiment	= single_pulse.ex2
Sample_id	= MS0567-200-96h
Solvent	= CHLOROFORM-D
Creation_time	= 8-OCT-2018 10:05:45
Revision_time	= 8-OCT-2018 09:40:25
Current_time	= 8-OCT-2018 09:40:25
Data_format	= 1D COMPLEX
Dim_size	= 104857
Dim_title	= 19F
Dim_units	= [ppm]
Dimensions	= X
Site	= ECA 500
Spectrometer	= JNM-ECA500
Field_strength	= 11.7473579[T] (500[MH
X_acq_duration	= 0.7340032[s]
X_domain	= 19F
X_freq	= 470.62046084[MHz]
X_offset	= -100[ppm]
X_points	= 131072
X_prescans	= 1
X_resolution	= 1.36239188[Hz]
X_sweep	= 178.57142857[kHz]
Irr_domain	= 19F
Irr_freq	= 470.62046084[MHz]
Irr_offset	= 5[ppm]
Tri_domain	= 19F
Tri_freq	= 470.62046084[MHz]
Tri_offset	= 5[ppm]
Clipped	= FALSE
Mod_return	= 1
Scans	= 20
Total_scans	= 20
X_90_width	= 13.1[us]
X_acq_time	= 0.7340032[s]
X_angle	= 45[deg]
X_atn	= 2.5[dB]
X_pulse	= 6.55[us]
Irr_mode	= Off
Tri_mode	= Off
Dante_presat	= FALSE
Initial_wait	= 1[s]
Recvr_gain	= 54
Relaxation_delay	= 4[s]
Repetition_time	= 4.7340032[s]
Temp_get	= 22.5[dC]

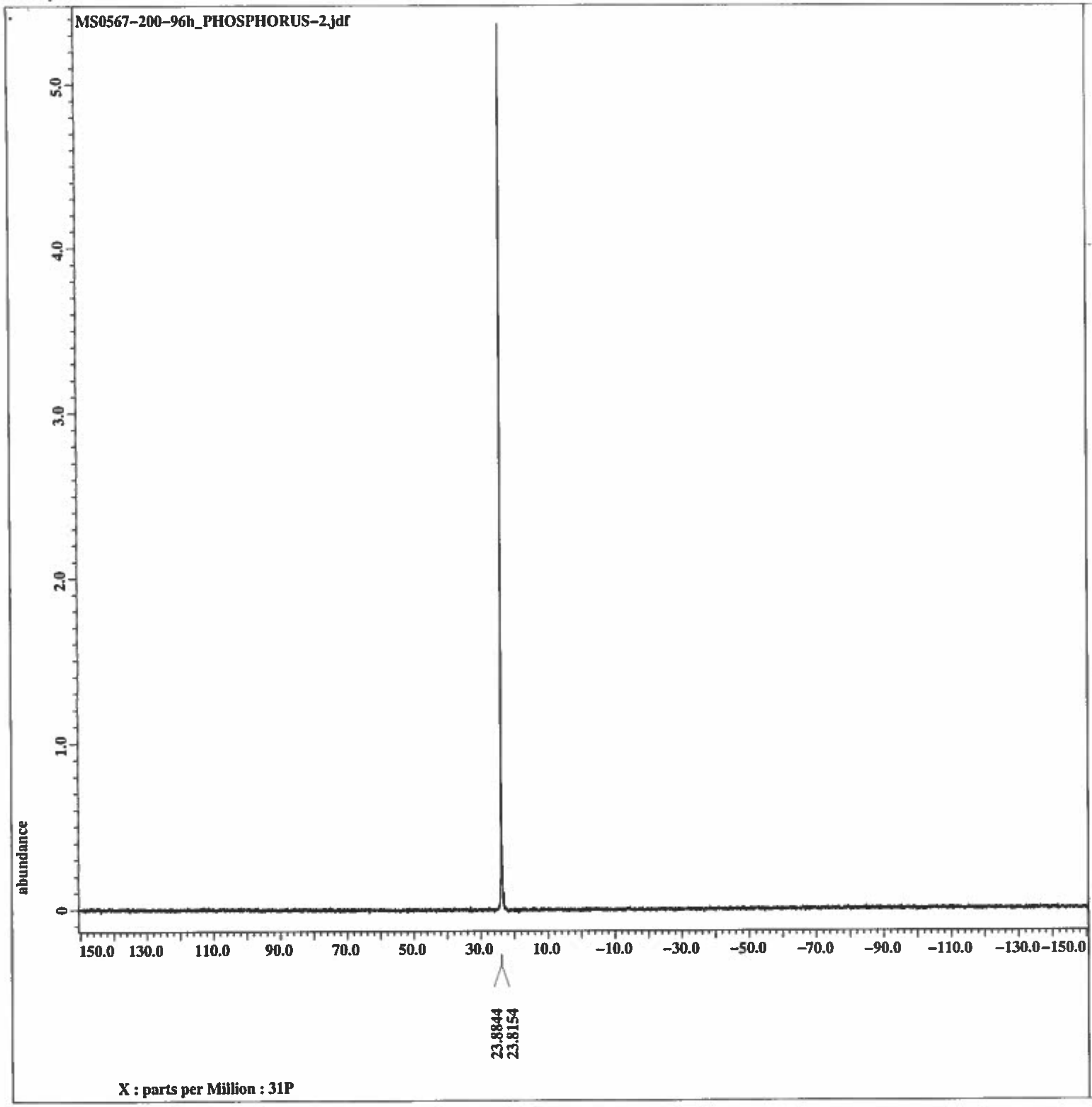


```
Filename            = MS0567-200-96h_PHOSPH
Author              = Jim Davis
Experiment          = single_pulse_dec
Sample_id           = MS0567-200-96h
Solvent             = CHLOROFORM-D
Creation_time       =  8-OCT-2018 10:09:52
Revision_time       =  8-OCT-2018 09:44:30
Current_time        =  8-OCT-2018 09:44:30

Data_format         = 1D COMPLEX
Dim_size            = 52428
Dim_title           = 31P
Dim_units           = [ppm]
Dimensions          = X
Site                = ECA 500
Spectrometer        = JNM-ECA500

Field_strength      = 11.7473579[T] (500[MH
X_acq_duration      = 0.85983232[s]
X_domain            = 31P
X_freq              = 202.46831075[MHz]
X_offset            = 0[ppm]
X_points            = 65536
X_prescans          = 4
X_resolution        = 1.16301746[Hz]
X_sweep             = 76.2195122[kHz]
Irr_domain          = 1H
Irr_freq            = 500.15991521[MHz]
Irr_offset          = 5.0[ppm]
Clipped             = FALSE
Mod_return          = 1
Scans               = 30
Total_scans         = 30

X_90_width          = 14.687[us]
X_acq_time          = 0.85983232[s]
X_angle             = 30[deg]
X_atn               = 5[dB]
X_pulse             = 4.89566667[us]
Irr_atn_dec         = 20.7[dB]
Irr_atn_noe         = 20.7[dB]
Irr_noise           = WALTZ
Decoupling          = TRUE
Initial_wait        = 1[s]
Noe                 = TRUE
Noe_time            = 2[s]
Recvr_gain          = 56
Relaxation_delay    = 2[s]
Repetition_time     = 2.85983232[s]
Temp_get            = 22.9[dC]
```

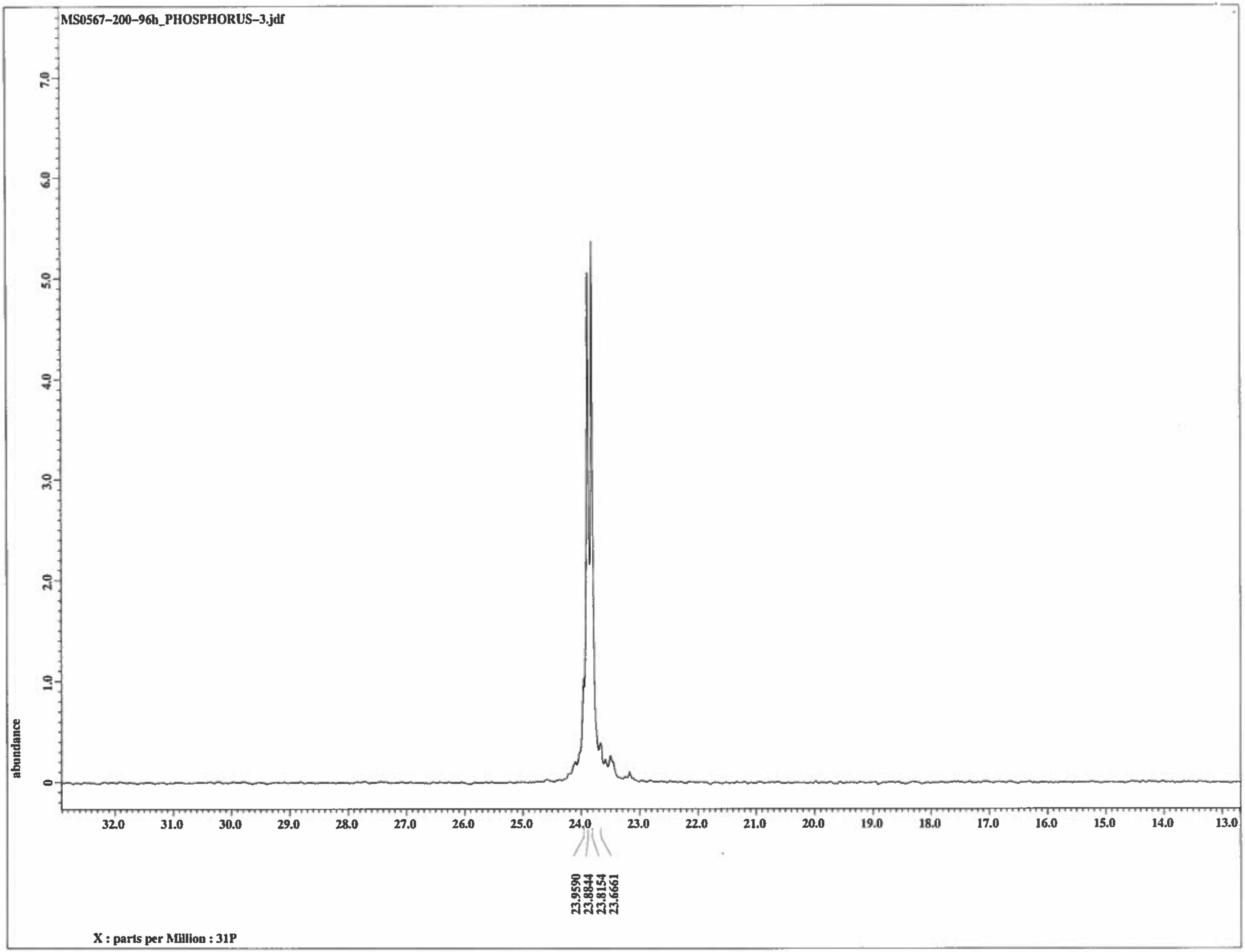
MS0567-200-96h_PHOSPHORUS-3.jdf
abundance
0
1.0
2.0
3.0
4.0
5.0
6.0
7.0
32.0
31.0
30.0
29.0
28.0
27.0
26.0
25.0
24.0
23.0
22.0
21.0
20.0
19.0
18.0
17.0
16.0
15.0
14.0
13.0
23.9590
23.8844
23.8154
23.6661
X : parts per Million : 31P

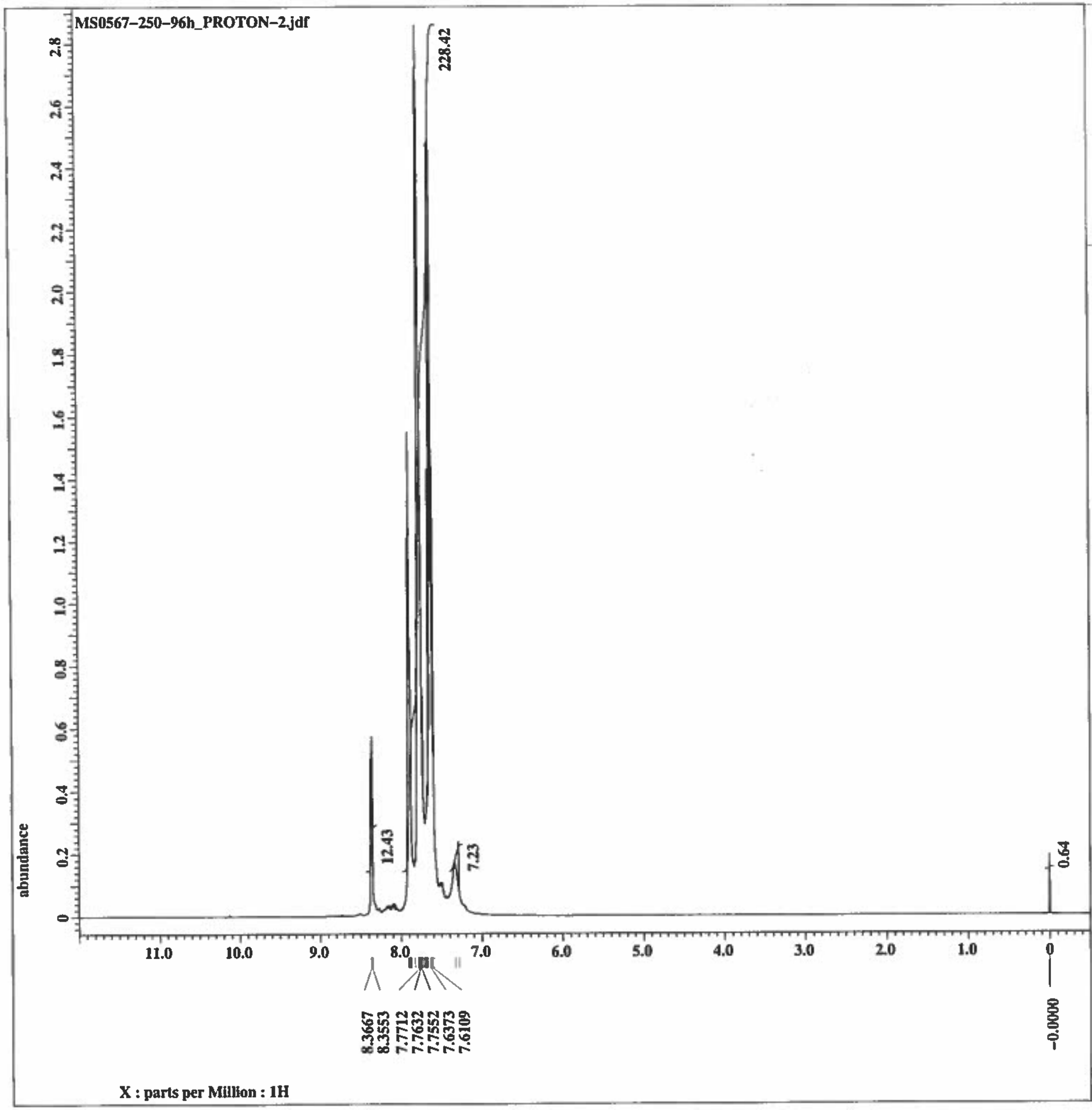

Filename = MS0567-250-96h_PROTON
Author = Jim Davis
Experiment = single_pulse.ex2
Sample_id = MS0567-250-96h
Solvent = CHLOROFORM-D
Creation_time = 8-OCT-2018 10:17:25
Revision_time = 8-OCT-2018 09:52:04
Current_time = 8-OCT-2018 09:52:04

Data_format = 1D COMPLEX
Dim_size = 13107
Dim_title = 1H
Dim_units = [ppm]
Dimensions = X
Site = ECA 500
Spectrometer = JNM-ECA500

Field_strength = 11.7473579[T] (500[MH
X_acq_duration = 1.74587904[s]
X_domain = 1H
X_freq = 500.15991521[MHz]
X_offset = 5.0[ppm]
X_points = 16384
X_prescans = 1
X_resolution = 0.57277737[Hz]
X_sweep = 9.38438438[kHz]
Irr_domain = 1H
Irr_freq = 500.15991521[MHz]
Irr_offset = 5.0[ppm]
Tri_domain = 1H
Tri_freq = 500.15991521[MHz]
Tri_offset = 5.0[ppm]
Clipped = FALSE
Mod_return = 1
Scans = 16
Total_scans = 16

X_90_width = 12.4[us]
X_acq_time = 1.74587904[s]
X_angle = 45[deg]
X_atn = 4[dB]
X_pulse = 6.2[us]
Irr_mode = Off
Tri_mode = Off
Dante_presat = FALSE
Initial_wait = 1[s]
Recvr_gain = 26
Relaxation_delay = 4[s]
Repetition_time = 5.74587904[s]
Temp_get = 22.7[dC]

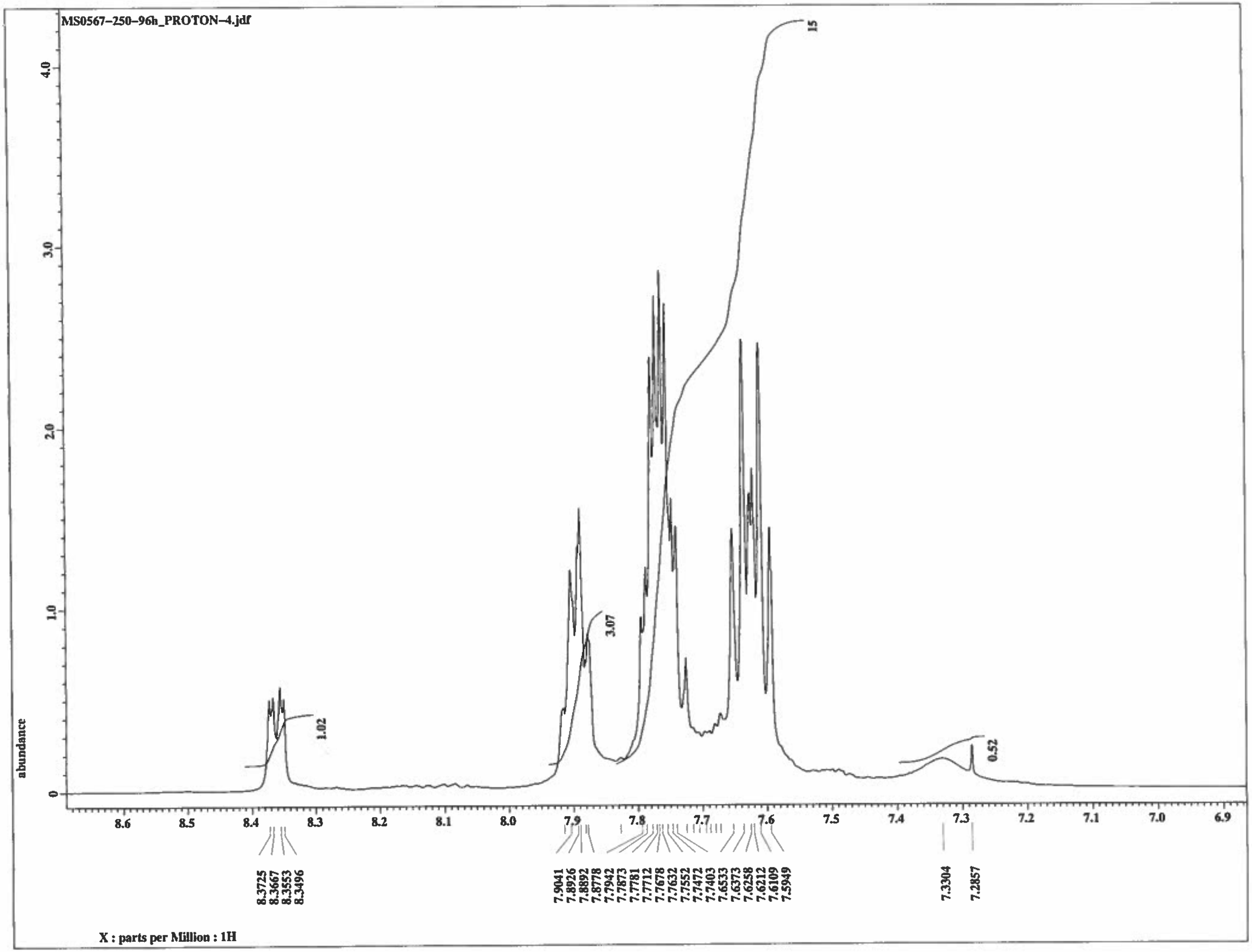
MS0567-250-96h_PROTON-4.jdf
abundance
0
1.0
2.0
3.0
4.0
1.02
3.07
15
0.52
8.6
8.5
8.4
8.3
8.2
8.1
8.0
7.9
7.8
7.7
7.6
7.5
7.4
7.3
7.2
7.1
7.0
6.9
8.3725
8.3667
8.3553
8.3496
7.9041
7.8926
7.8892
7.8778
7.7942
7.7873
7.7781
7.7712
7.7678
7.7632
7.7552
7.7472
7.7403
7.6533
7.6373
7.6258
7.6212
7.6109
7.5949
7.3304
7.2857
X : parts per Million : 1H

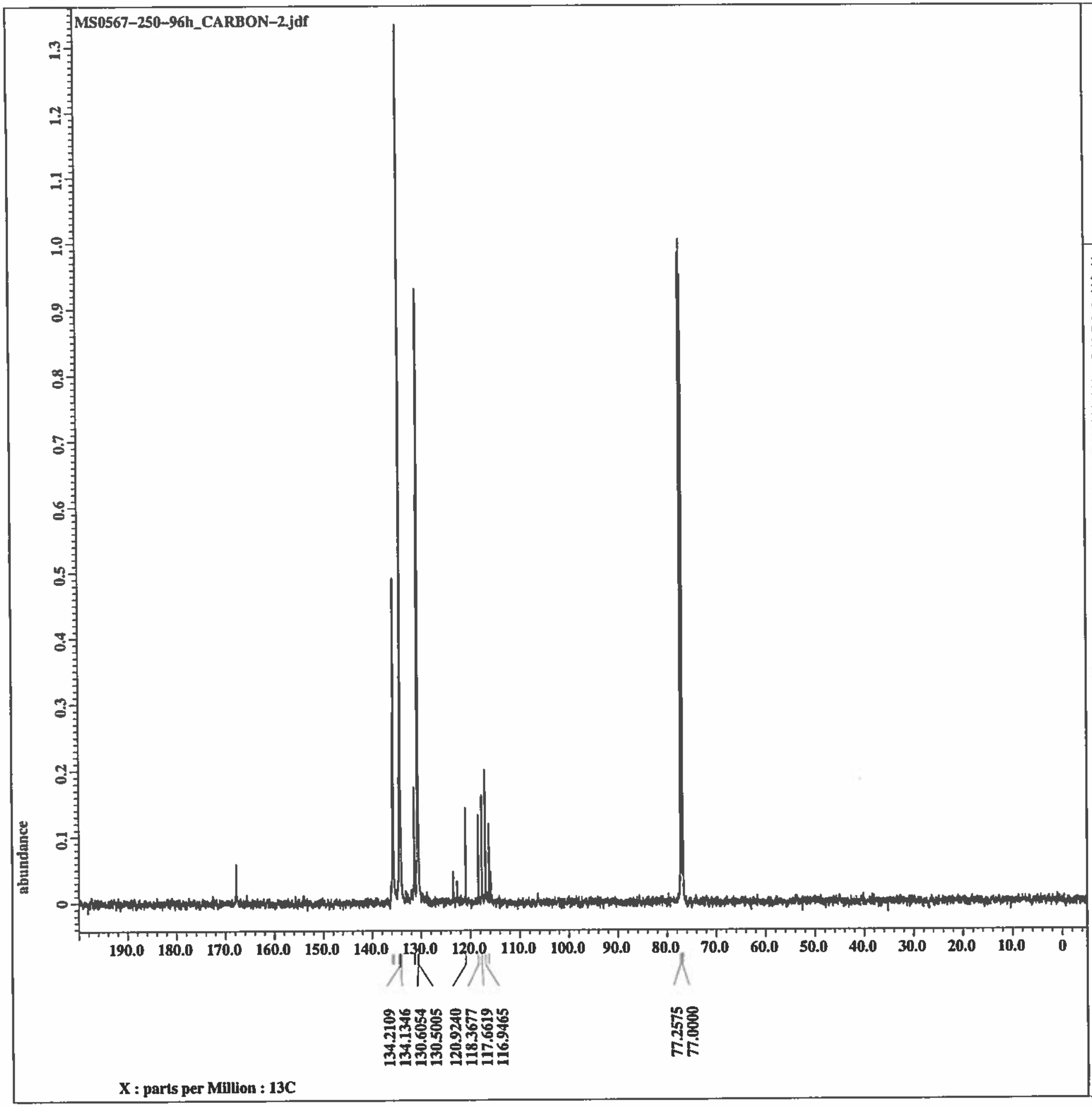

Filename	= MS0567-250-96h_CARBON
Author	= Jim Davis
Experiment	= single_pulse_dec
Sample_id	= MS0567-250-96h
Solvent	= CHLOROFORM-D
Creation_time	= 8-OCT-2018 10:30:48
Revision_time	= 8-OCT-2018 10:05:27
Current_time	= 8-OCT-2018 10:05:27
Data_format	= 1D COMPLEX
Dim_size	= 26214
Dim_title	= 13C
Dim_units	= [ppm]
Dimensions	= X
Site	= ECA 500
Spectrometer	= JNM-ECA500
Field_strength	= 11.7473579[T] (500[MH
X_acq_duration	= 0.83361792[s]
X_domain	= 13C
X_freq	= 125.76529768[MHz]
X_offset	= 100[ppm]
X_points	= 32768
X_prescans	= 4
X_resolution	= 1.19959034[Hz]
X_sweep	= 39.3081761[kHz]
Irr_domain	= 1H
Irr_freq	= 500.15991521[MHz]
Irr_offset	= 5.0[ppm]
Clipped	= FALSE
Mod_return	= 1
Scans	= 256
Total_scans	= 256
X_90_width	= 13.2[us]
X_acq_time	= 0.83361792[s]
X_angle	= 30[deg]
X_atn	= 6[dB]
X_pulse	= 4.4[us]
Irr_atn_dec	= 20.7[dB]
Irr_atn_noe	= 20.7[dB]
Irr_noise	= WALTZ
Decoupling	= TRUE
Initial_wait	= 1[s]
Noe	= TRUE
Noe_time	= 2[s]
Recvr_gain	= 60
Relaxation_delay	= 2[s]
Repetition_time	= 2.83361792[s]
Temp_get	= 23.3[dC]

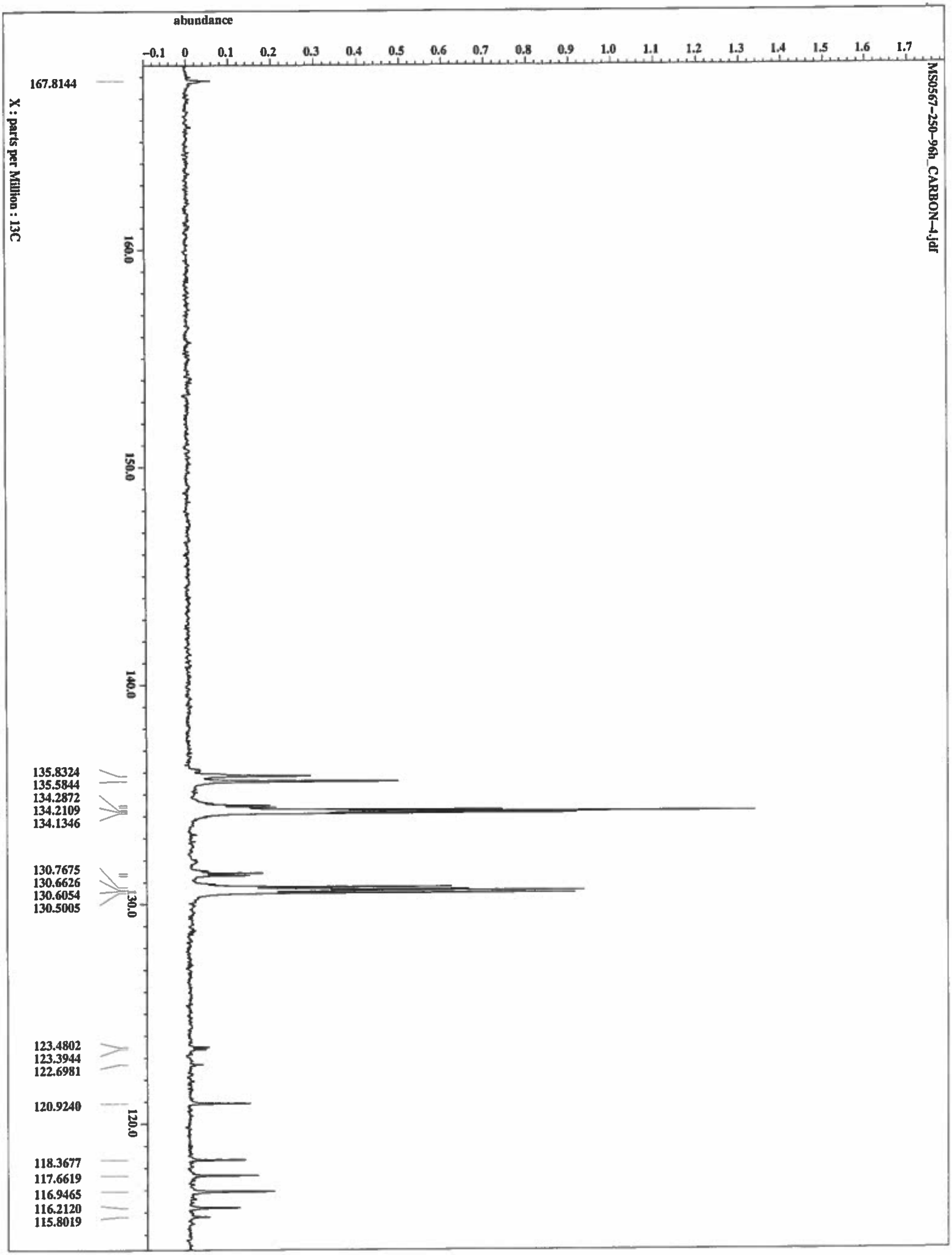

abundance
-0.1
0
0.1
0.2
0.3
0.4
0.5
0.6
0.7
0.8
0.9
1.0
1.1
1.2
1.3
1.4
1.5
1.6
1.7
MS0567-250-96h_CARBON-4.jdf
X : parts per Million : 13C
160.0
150.0
140.0
130.0
120.0
167.8144
135.8324
135.5844
134.2872
134.2109
134.1346
130.7675
130.6626
130.6054
130.5005
123.4802
123.3944
122.6981
120.9240
118.3677
117.6619
116.9465
116.2120
115.8019

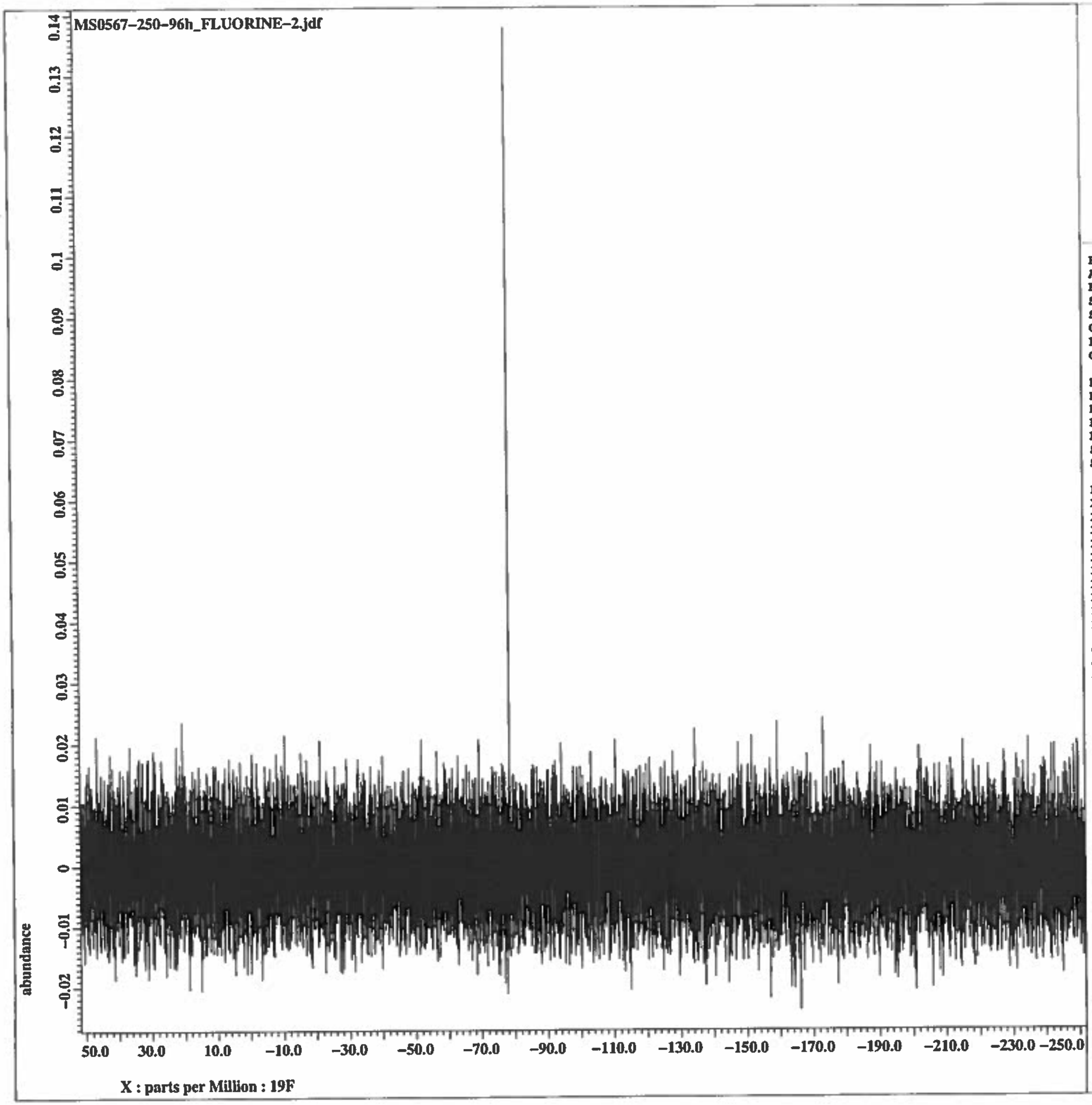

Filename	= MS0567-250-96h_FLUORI
Author	= Jim Davis
Experiment	= single_pulse.ex2
Sample_id	= MS0567-250-96h
Solvent	= CHLOROFORM-D
Creation_time	= 8-OCT-2018 10:35:00
Revision_time	= 8-OCT-2018 10:09:40
Current_time	= 8-OCT-2018 10:09:40
Data_format	= 1D COMPLEX
Dim_size	= 104857
Dim_title	= 19F
Dim_units	= [ppm]
Dimensions	= X
Site	= ECA 500
Spectrometer	= JNM-ECA500
Field_strength	= 11.7473579[T] (500[MH
X_acq_duration	= 0.7340032[s]
X_domain	= 19F
X_freq	= 470.62046084[MHz]
X_offset	= -100[ppm]
X_points	= 131072
X_prescans	= 1
X_resolution	= 1.36239188[Hz]
X_sweep	= 178.57142857[kHz]
Irr_domain	= 19F
Irr_freq	= 470.62046084[MHz]
Irr_offset	= 5[ppm]
Tri_domain	= 19F
Tri_freq	= 470.62046084[MHz]
Tri_offset	= 5[ppm]
Clipped	= FALSE
Mod_return	= 1
Scans	= 20
Total_scans	= 20
X_90_width	= 13.1[us]
X_acq_time	= 0.7340032[s]
X_angle	= 45[deg]
X_atn	= 2.5[dB]
X_pulse	= 6.55[us]
Irr_mode	= Off
Tri_mode	= Off
Dante_presat	= FALSE
Initial_wait	= 1[s]
Recvr_gain	= 70
Relaxation_delay	= 4[s]
Repetition_time	= 4.7340032[s]
Temp_get	= 22.9[dC]

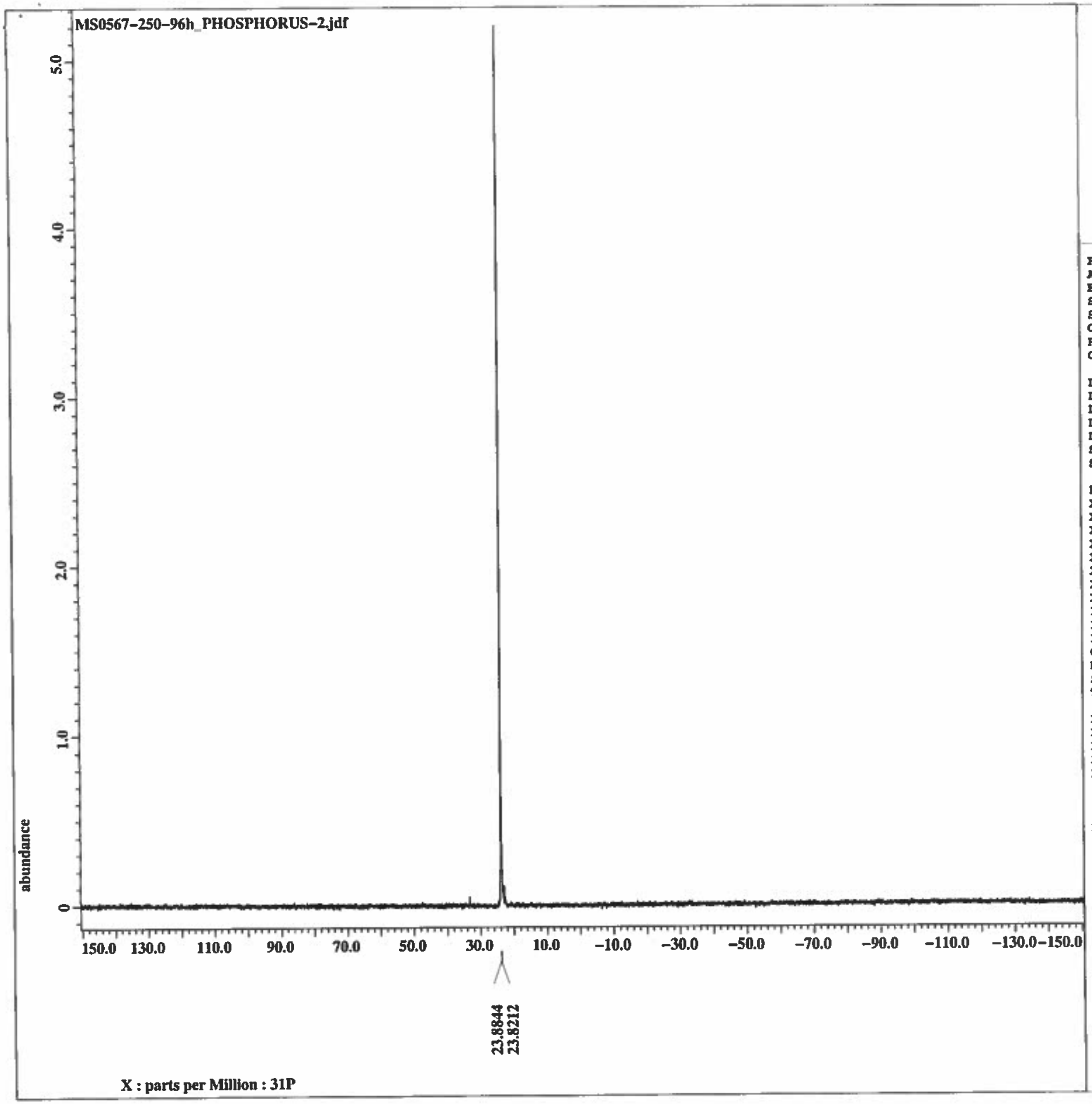

Filename	= MS0567-250-96h_PHOSPH
Author	= Jim Davis
Experiment	= single_pulse_dec
Sample_id	= MS0567-250-96h
Solvent	= CHLOROFORM-D
Creation_time	= 8-OCT-2018 10:39:10
Revision_time	= 8-OCT-2018 10:13:49
Current_time	= 8-OCT-2018 10:13:49
Data_format	= 1D COMPLEX
Dim_size	= 52428
Dim_title	= 31P
Dim_units	= [ppm]
Dimensions	= X
Site	= ECA 500
Spectrometer	= JNM-ECA500
Field_strength	= 11.7473579[T] (500[MH
X_acq_duration	= 0.85983232[s]
X_domain	= 31P
X_freq	= 202.46831075[MHz]
X_offset	= 0[ppm]
X_points	= 65536
X_prescans	= 4
X_resolution	= 1.16301746[Hz]
X_sweep	= 76.2195122[kHz]
Irr_domain	= 1H
Irr_freq	= 500.15991521[MHz]
Irr_offset	= 5.0[ppm]
Clipped	= FALSE
Mod_return	= 1
Scans	= 30
Total_scans	= 30
X_90_width	= 14.687[us]
X_acq_time	= 0.85983232[s]
X_angle	= 30[deg]
X_atn	= 5[dB]
X_pulse	= 4.89566667[us]
Irr_atn_dec	= 20.7[dB]
Irr_atn_noe	= 20.7[dB]
Irr_noise	= WALTZ
Decoupling	= TRUE
Initial_wait	= 1[s]
Noe	= TRUE
Noe_time	= 2[s]
Recvr_gain	= 58
Relaxation_delay	= 2[s]
Repetition_time	= 2.85983232[s]
Temp_get	= 23.3[dC]

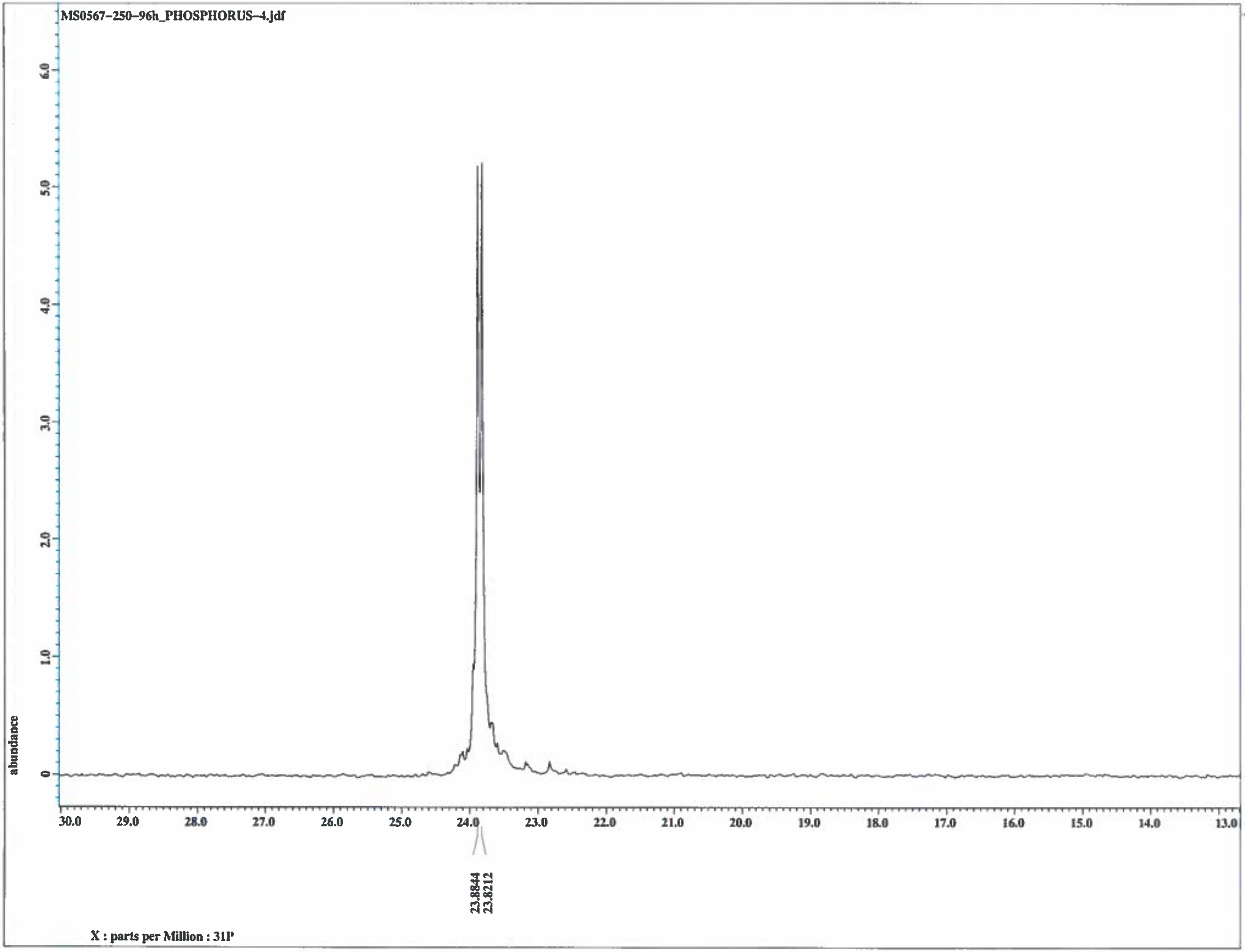
MS0567-250-96h_PHOSPHORUS-4.jdf
abundance
0
1.0
2.0
3.0
4.0
5.0
6.0
30.0
29.0
28.0
27.0
26.0
25.0
24.0
23.0
22.0
21.0
20.0
19.0
18.0
17.0
16.0
15.0
14.0
13.0
23.8844
23.8212
X : parts per Million : 31P

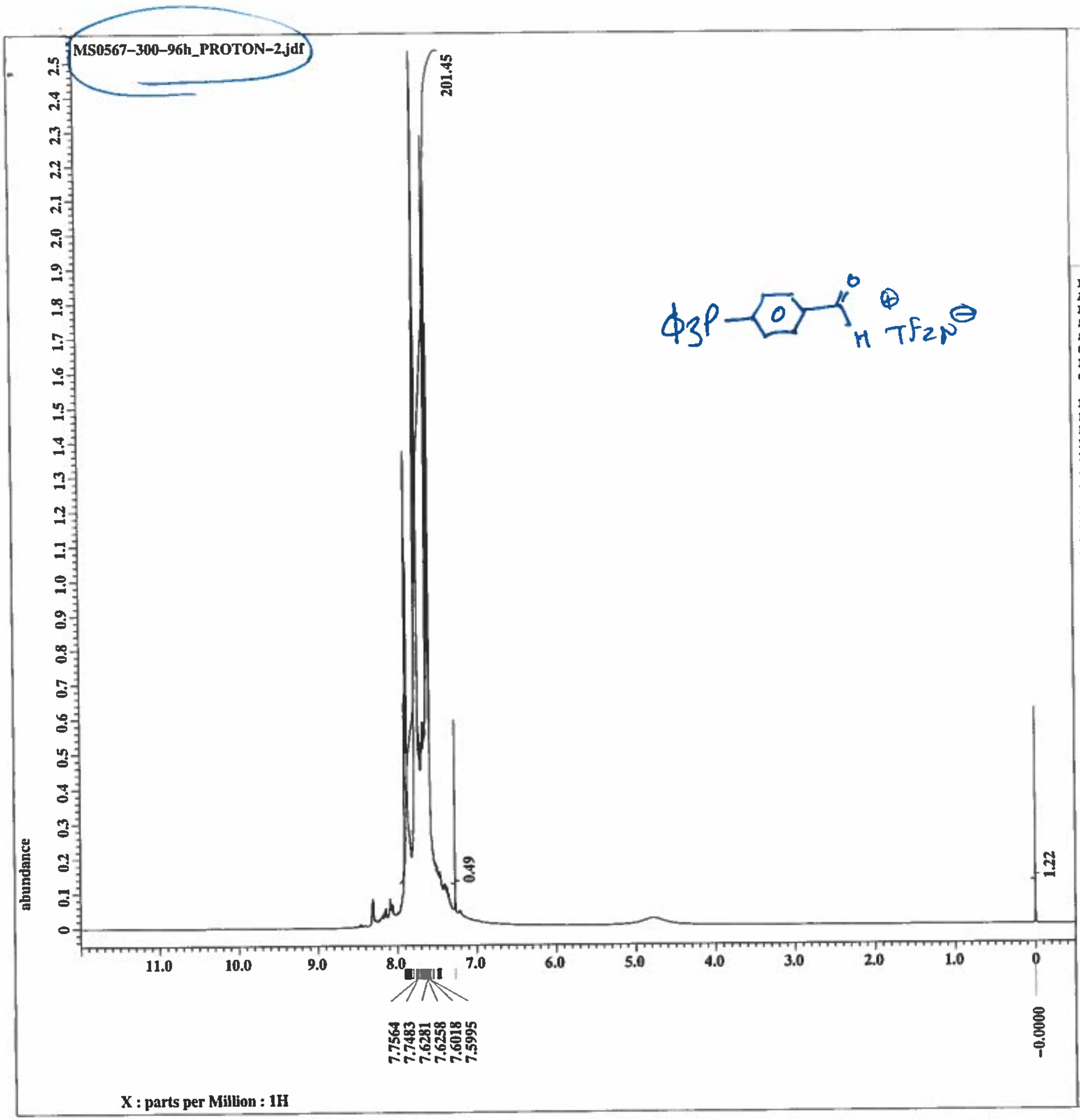

Filename	= MS0567-300-96h_PROTON
Author	= Jim Davis
Experiment	= single_pulse.ex2
Sample_id	= MS0567-300-96h
Solvent	= CHLOROFORM-D
Creation_time	= 8-OCT-2018 10:46:10
Revision_time	= 8-OCT-2018 10:20:47
Current_time	= 8-OCT-2018 10:20:47
Data_format	= 1D COMPLEX
Dim_size	= 13107
Dim_title	= 1H
Dim_units	= [ppm]
Dimensions	= X
Site	= ECA 500
Spectrometer	= JNM-ECA500
Field_strength	= 11.7473579[T] (500[MH
X_acq_duration	= 1.74587904[s]
X_domain	= 1H
X_freq	= 500.15991521[MHz]
X_offset	= 5.0[ppm]
X_points	= 16384
X_prescans	= 1
X_resolution	= 0.57277737[Hz]
X_sweep	= 9.38438438[kHz]
Irr_domain	= 1H
Irr_freq	= 500.15991521[MHz]
Irr_offset	= 5.0[ppm]
Tri_domain	= 1H
Tri_freq	= 500.15991521[MHz]
Tri_offset	= 5.0[ppm]
Clipped	= FALSE
Mod_return	= 1
Scans	= 16
Total_scans	= 16
X_90_width	= 12.4[us]
X_acq_time	= 1.74587904[s]
X_angle	= 45[deg]
X_atn	= 4[dB]
X_pulse	= 6.2[us]
Irr_mode	= Off
Tri_mode	= Off
Dante_presat	= FALSE
Initial_wait	= 1[s]
Recvr_gain	= 30
Relaxation_delay	= 4[s]
Repetition_time	= 5.74587904[s]
Temp_get	= 23[dC]

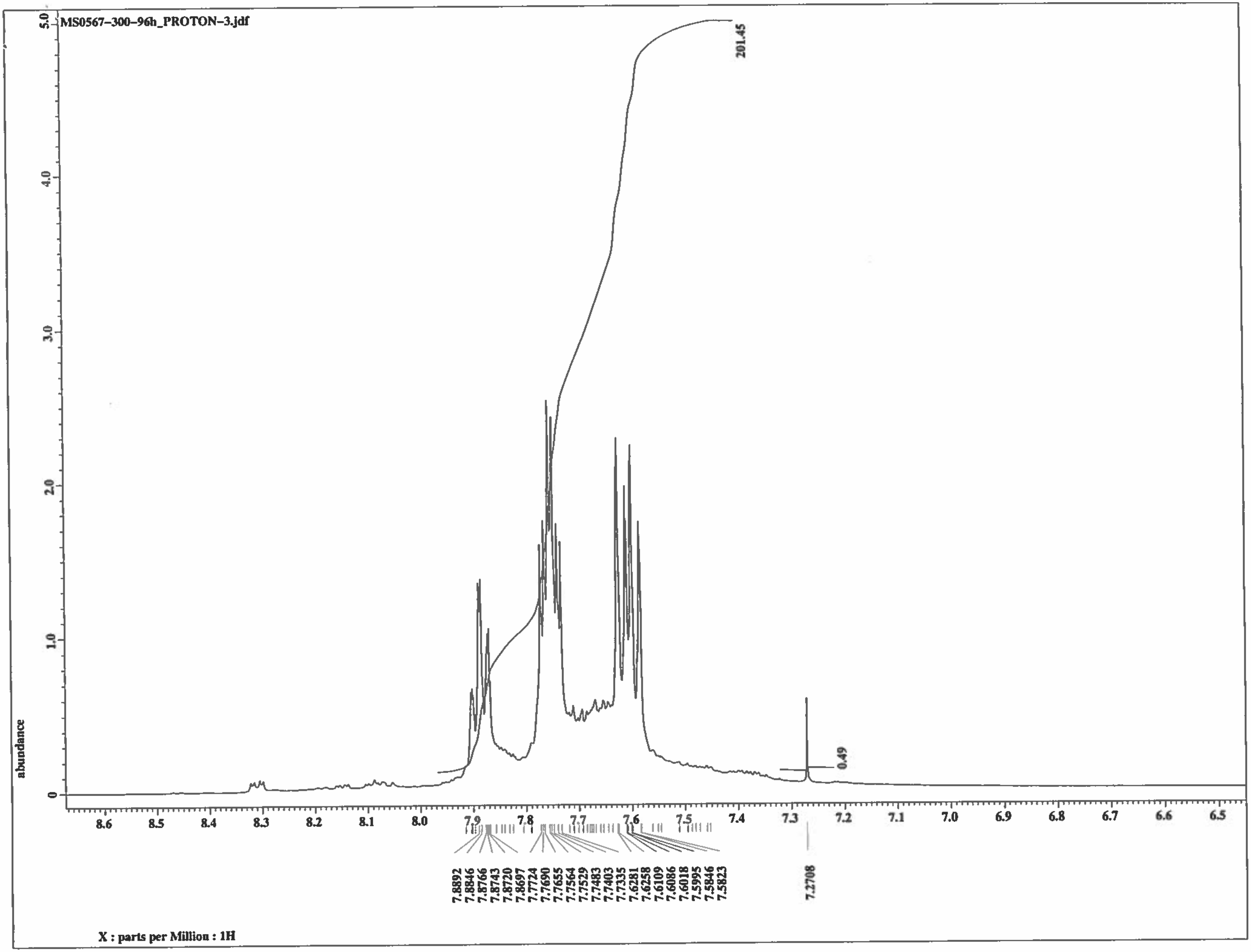
MS0567-300-96h_PROTON-3.jdf
abundance
201.45
0.49
7.8892
7.8846
7.8766
7.8743
7.8720
7.8697
7.7724
7.7690
7.7655
7.7564
7.7529
7.7483
7.7403
7.7335
7.6281
7.6258
7.6109
7.6086
7.6018
7.5995
7.5846
7.5823
7.2708
X : parts per Million : 1H

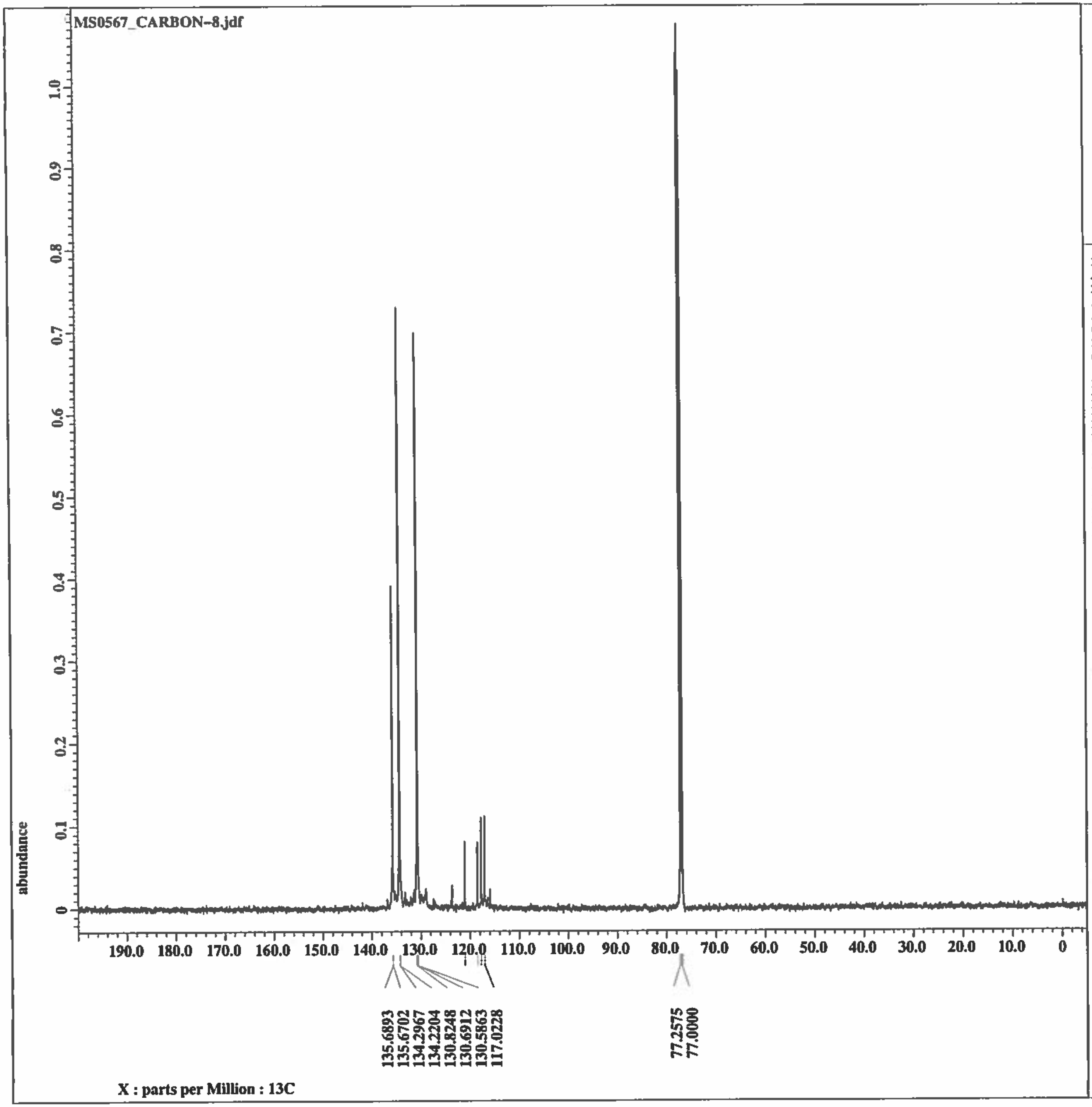

Filename	= MS0567_CARBON-8.jdf
Author	= Jim Davis
Experiment	= single_pulse_dec
Sample_id	= MS0567
Solvent	= CHLOROFORM-D
Creation_time	= 8-OCT-2018 18:35:33
Revision_time	= 8-OCT-2018 18:10:09
Current_time	= 8-OCT-2018 18:10:09
Data_format	= 1D COMPLEX
Dim_size	= 26214
Dim_title	= 13C
Dim_units	= [ppm]
Dimensions	= X
Site	= ECA 500
Spectrometer	= JNM-ECA500
Field_strength	= 11.7473579[T] (500[MH
X_acq_duration	= 0.83361792[s]
X_domain	= 13C
X_freq	= 125.76529768[MHz]
X_offset	= 100[ppm]
X_points	= 32768
X_prescans	= 4
X_resolution	= 1.19959034[Hz]
X_sweep	= 39.3081761[kHz]
Irr_domain	= 1H
Irr_freq	= 500.15991521[MHz]
Irr_offset	= 5.0[ppm]
Clipped	= FALSE
Mod_return	= 1
Scans	= 1024
Total_scans	= 1024
X_90_width	= 13.2[us]
X_acq_time	= 0.83361792[s]
X_angle	= 30[deg]
X_atn	= 6[dB]
X_pulse	= 4.4[us]
Irr_atn_dec	= 20.7[dB]
Irr_atn_noe	= 20.7[dB]
Irr_noise	= WALTZ
Decoupling	= TRUE
Initial_wait	= 1[s]
Noe	= TRUE
Noe_time	= 2[s]
Recvr_gain	= 60
Relaxation_delay	= 2[s]
Repetition_time	= 2.83361792[s]
Temp_get	= 23.5[dC]

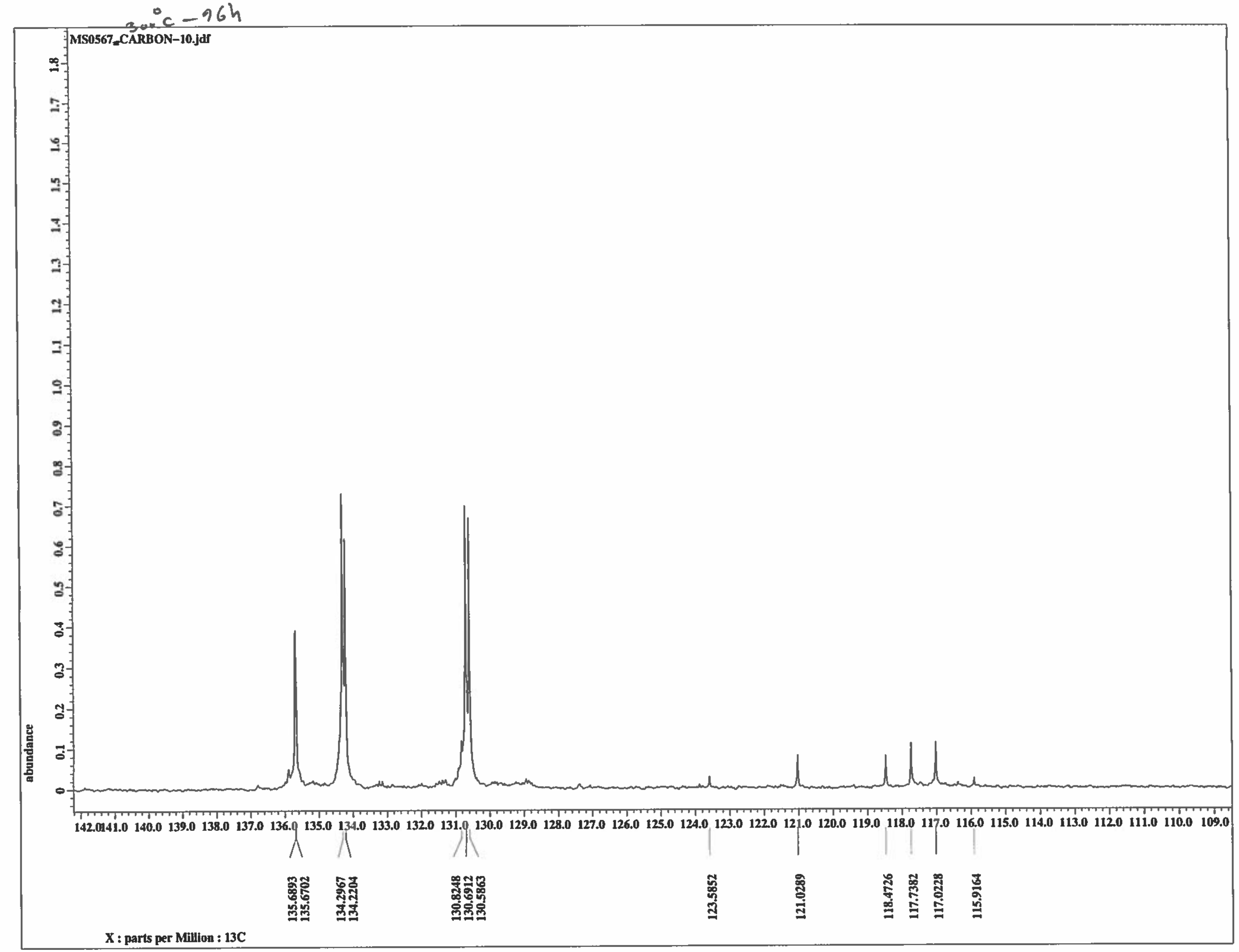
30°C – 96h
MS0567_CARBON-10.jdf
abundance
0 0.1 0.2 0.3 0.4 0.5 0.6 0.7 0.8 0.9 1.0 1.1 1.2 1.3 1.4 1.5 1.6 1.7 1.8
142.0 141.0 140.0 139.0 138.0 137.0 136.0 135.0 134.0 133.0 132.0 131.0 130.0 129.0 128.0 127.0 126.0 125.0 124.0 123.0 122.0 121.0 120.0 119.0 118.0 117.0 116.0 115.0 114.0 113.0 112.0 111.0 110.0 109.0
135.6893
135.6702
134.2967
134.2204
130.8248
130.6912
130.5863
123.5852
121.0289
118.4726
117.7382
117.0228
115.9164
X : parts per Million : 13C

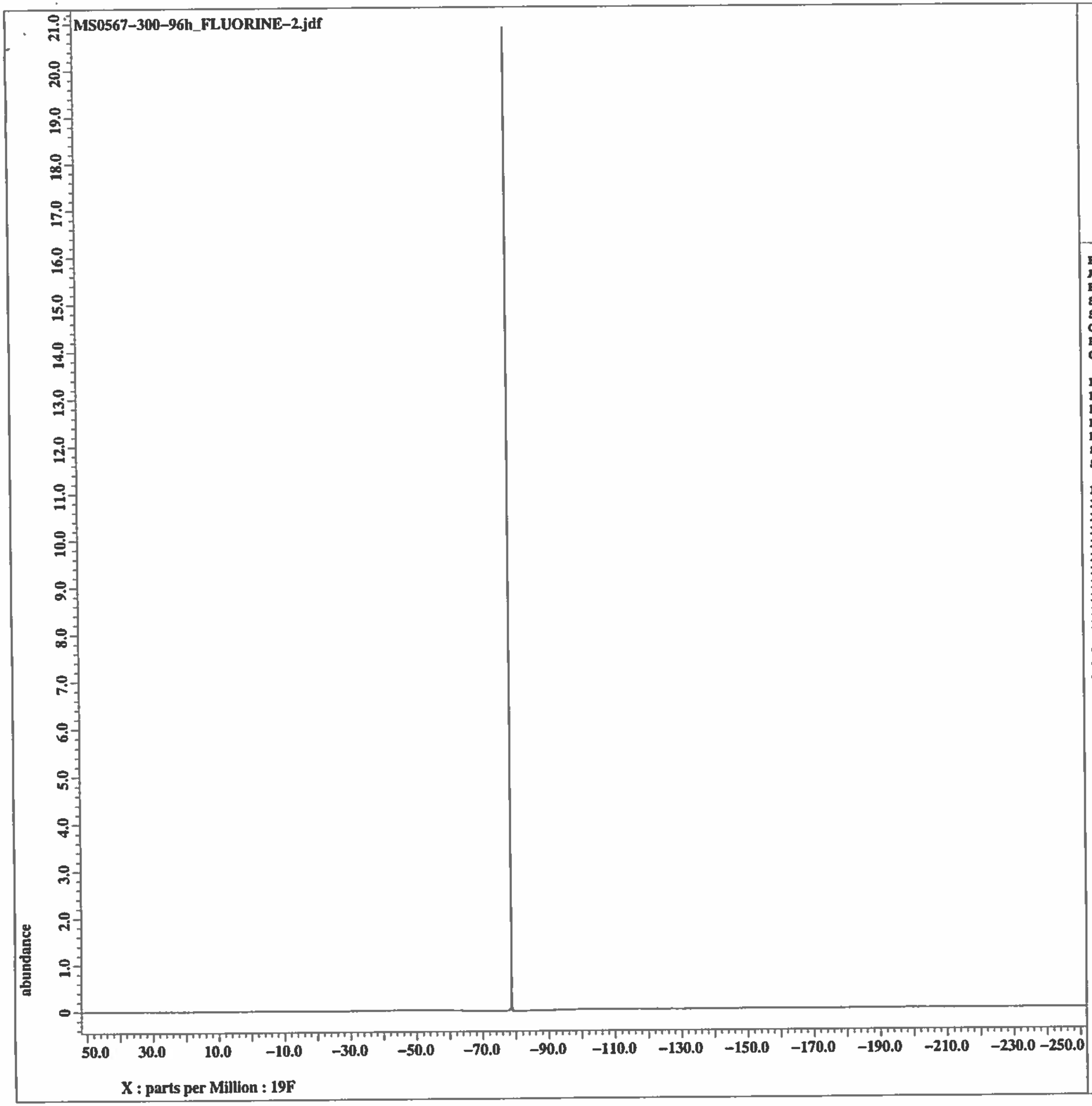

Filename	=	MS0567-300-96h_FLUORI
Author	=	Jim Davis
Experiment	=	single_pulse.ex2
Sample_id	=	MS0567-300-96h
Solvent	=	CHLOROFORM-D
Creation_time	=	8-OCT-2018 11:03:15
Revision_time	=	8-OCT-2018 10:37:54
Current_time	=	8-OCT-2018 10:37:54
Data_format	=	1D COMPLEX
Dim_size	=	104857
Dim_title	=	19F
Dim_units	=	[ppm]
Dimensions	=	X
Site	=	ECA 500
Spectrometer	=	JNM-ECA500
Field_strength	=	11.7473579[T] (500[MH
X_acq_duration	=	0.7340032[s]
X_domain	=	19F
X_freq	=	470.62046084[MHz]
X_offset	=	-100[ppm]
X_points	=	131072
X_prescans	=	1
X_resolution	=	1.36239188[Hz]
X_sweep	=	178.57142857[kHz]
Irr_domain	=	19F
Irr_freq	=	470.62046084[MHz]
Irr_offset	=	5[ppm]
Tri_domain	=	19F
Tri_freq	=	470.62046084[MHz]
Tri_offset	=	5[ppm]
Clipped	=	FALSE
Mod_return	=	1
Scans	=	20
Total_scans	=	20
X_90_width	=	13.1[us]
X_acq_time	=	0.7340032[s]
X_angle	=	45[deg]
X_atn	=	2.5[dB]
X_pulse	=	6.55[us]
Irr_mode	=	Off
Tri_mode	=	Off
Dante_presat	=	FALSE
Initial_wait	=	1[s]
Recvr_gain	=	64
Relaxation_delay	=	4[s]
Repetition_time	=	4.7340032[s]
Temp_get	=	22.8[dC]

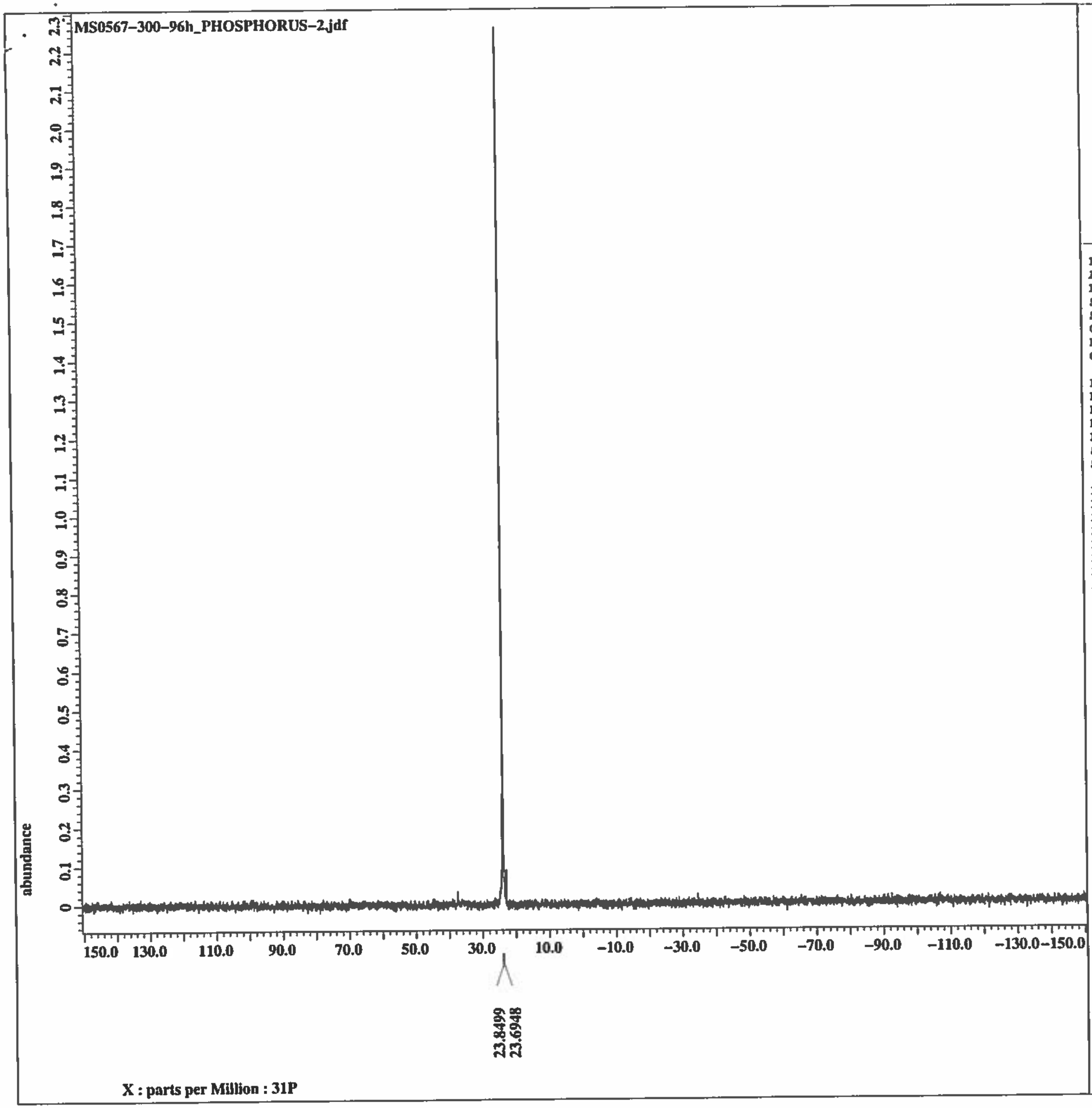

Filename	= MS0567-300-96h_PHOSPH
Author	= Jim Davis
Experiment	= single_pulse_dec
Sample_id	= MS0567-300-96h
Solvent	= CHLOROFORM-D
Creation_time	= 8-OCT-2018 11:07:22
Revision_time	= 8-OCT-2018 10:42:00
Current_time	= 8-OCT-2018 10:42:00
Data_format	= 1D COMPLEX
Dim_size	= 52428
Dim_title	= 31P
Dim_units	= [ppm]
Dimensions	= X
Site	= ECA 500
Spectrometer	= JNM-ECA500
Field_strength	= 11.7473579[T] (500[MH
X_acq_duration	= 0.85983232[s]
X_domain	= 31P
X_freq	= 202.46831075[MHz]
X_offset	= 0[ppm]
X_points	= 65536
X_prescans	= 4
X_resolution	= 1.16301746[Hz]
X_sweep	= 76.2195122[kHz]
Irr_domain	= 1H
Irr_freq	= 500.15991521[MHz]
Irr_offset	= 5.0[ppm]
Clipped	= FALSE
Mod_return	= 1
Scans	= 30
Total_scans	= 30
X_90_width	= 14.687[us]
X_acq_time	= 0.85983232[s]
X_angle	= 30[deg]
X_atn	= 5[dB]
X_pulse	= 4.89566667[us]
Irr_atn_dec	= 20.7[dB]
Irr_atn_noe	= 20.7[dB]
Irr_noise	= WALTZ
Decoupling	= TRUE
Initial_wait	= 1[s]
Noe	= TRUE
Noe_time	= 2[s]
Recvr_gain	= 56
Relaxation_delay	= 2[s]
Repetition_time	= 2.85983232[s]
Temp_get	= 23.2[dC]

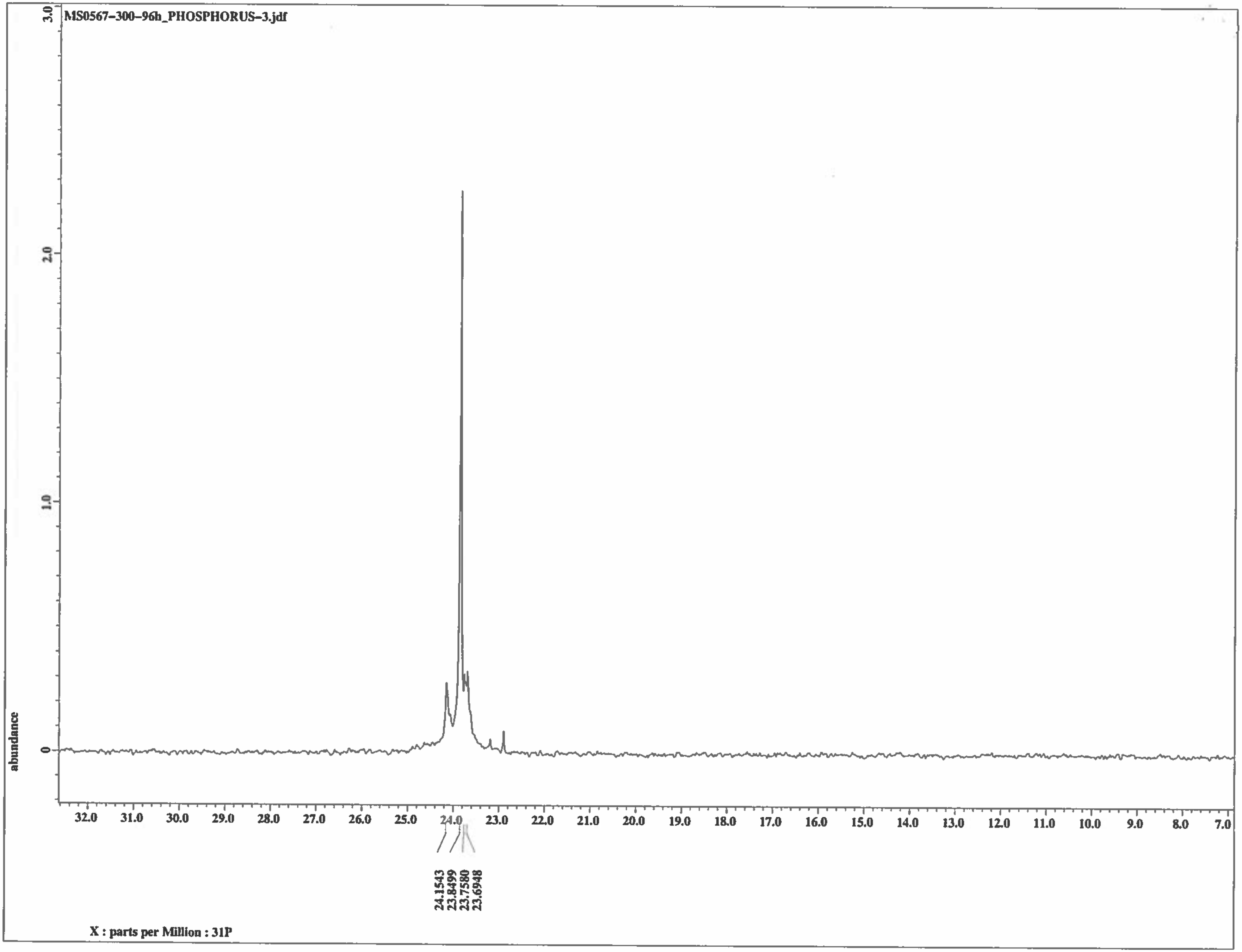
MS0567-300-96h_PHOSPHORUS-3.jdf
abundance
3.0
2.0
1.0
0
32.0
31.0
30.0
29.0
28.0
27.0
26.0
25.0
24.0
23.0
22.0
21.0
20.0
19.0
18.0
17.0
16.0
15.0
14.0
13.0
12.0
11.0
10.0
9.0
8.0
7.0
24.1543
23.8499
23.7580
23.6948
X : parts per Million : 31P

Compound 5 Pre- and Post-heating NMR Spectra

Temperature of Post-heating samples noted in upper left corner of each spectrum

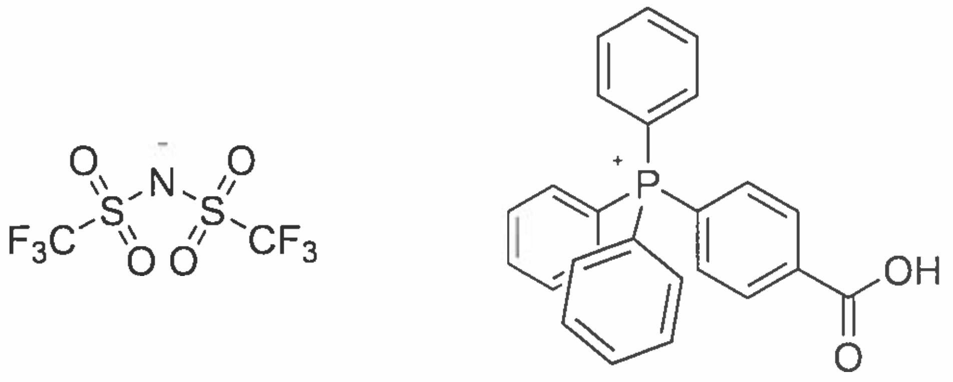

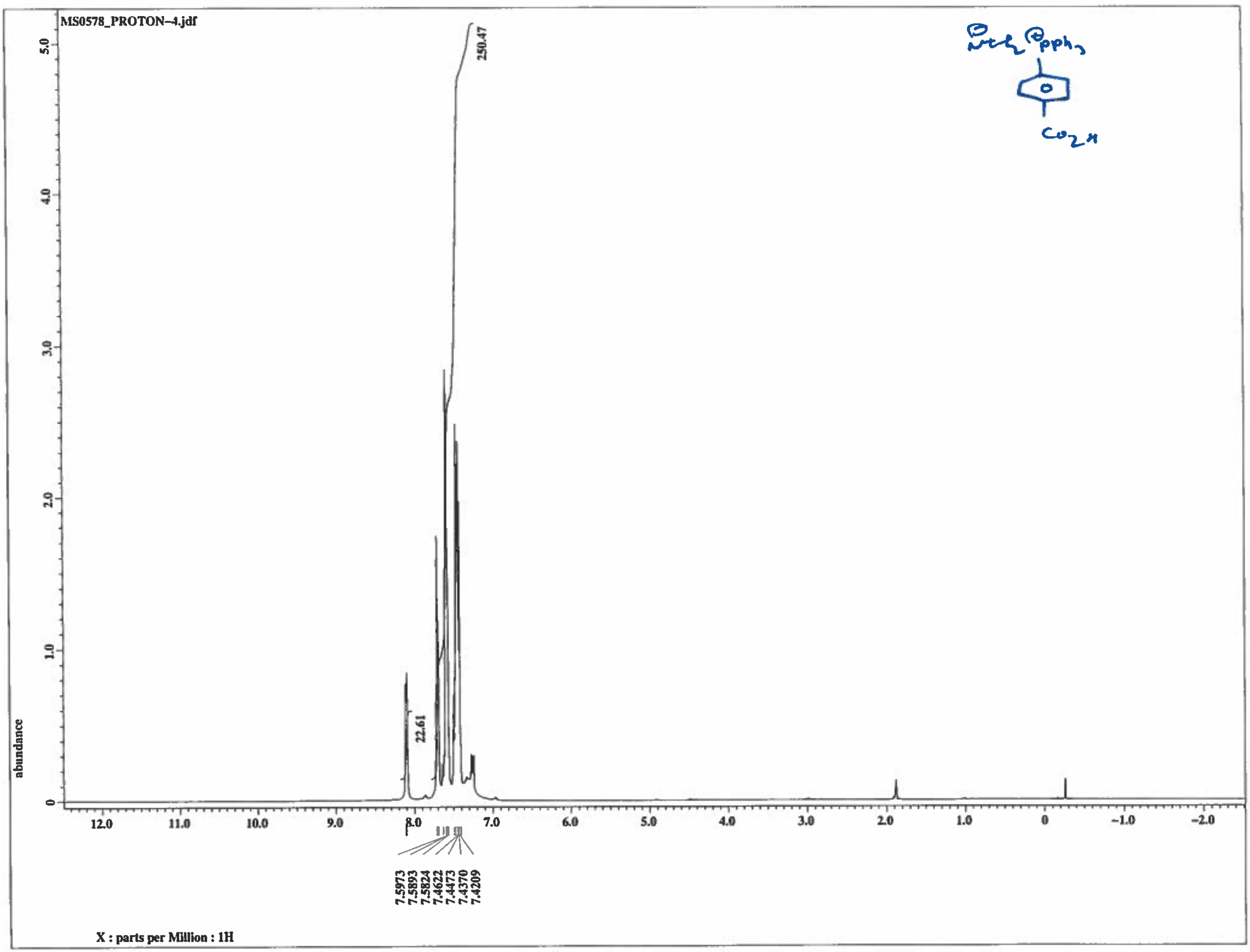
MS0578_PROTON-4.jdf
abundance
22.61
250.47
7.5973
7.5893
7.5824
7.4622
7.4473
7.4370
7.4209
X : parts per Million : 1H

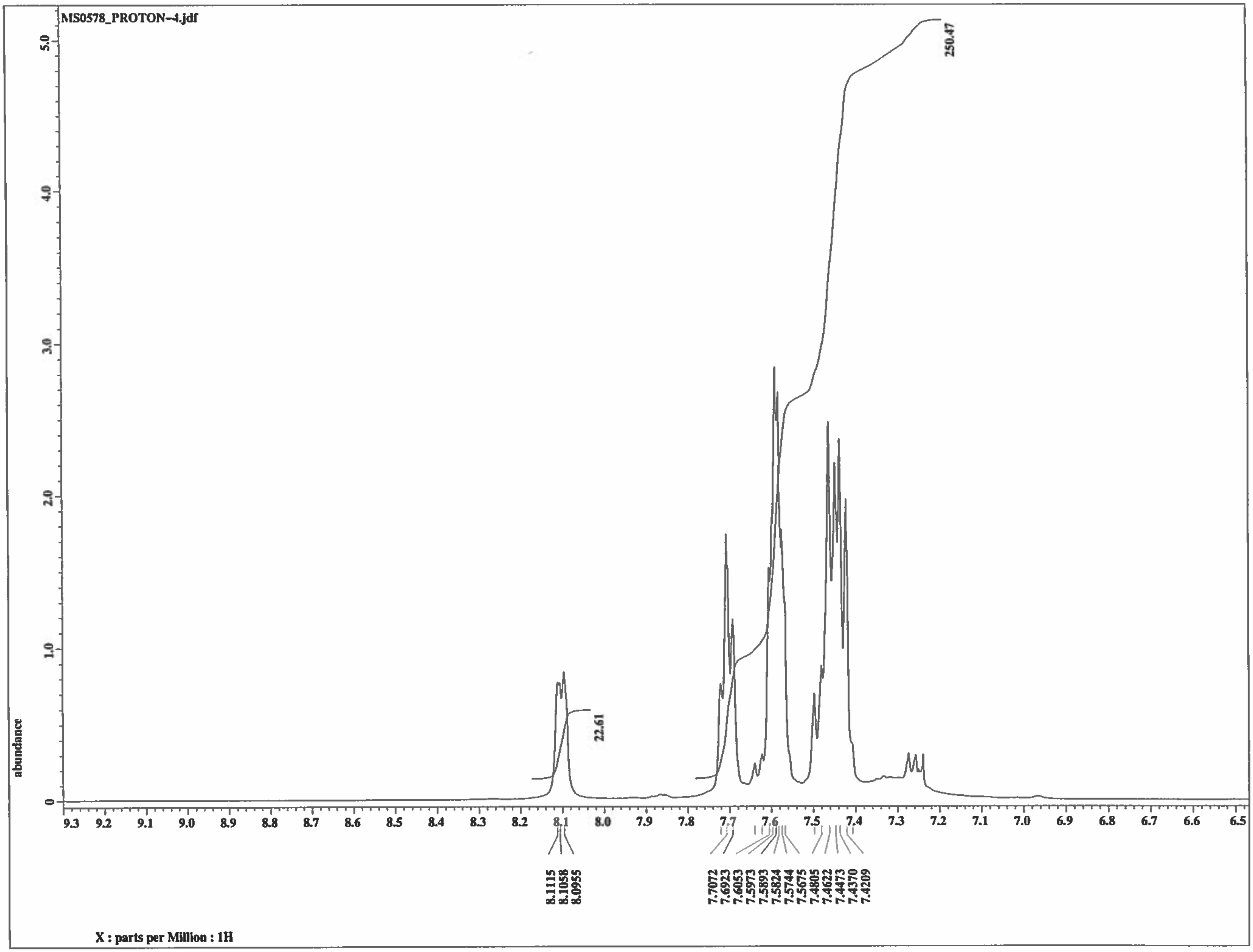

MS0578_PROTON~4.jdf
abundance
0
1.0
2.0
3.0
4.0
5.0
9.3
9.2
9.1
9.0
8.9
8.8
8.7
8.6
8.5
8.4
8.3
8.2
8.1
8.0
7.9
7.8
7.7
7.6
7.5
7.4
7.3
7.2
7.1
7.0
6.9
6.8
6.7
6.6
6.5
22.61
250.47
8.1115
8.1058
8.0955
7.7072
7.6923
7.6053
7.5973
7.5893
7.5824
7.5744
7.5675
7.4805
7.4622
7.4473
7.4370
7.4209
X : parts per Million : 1H

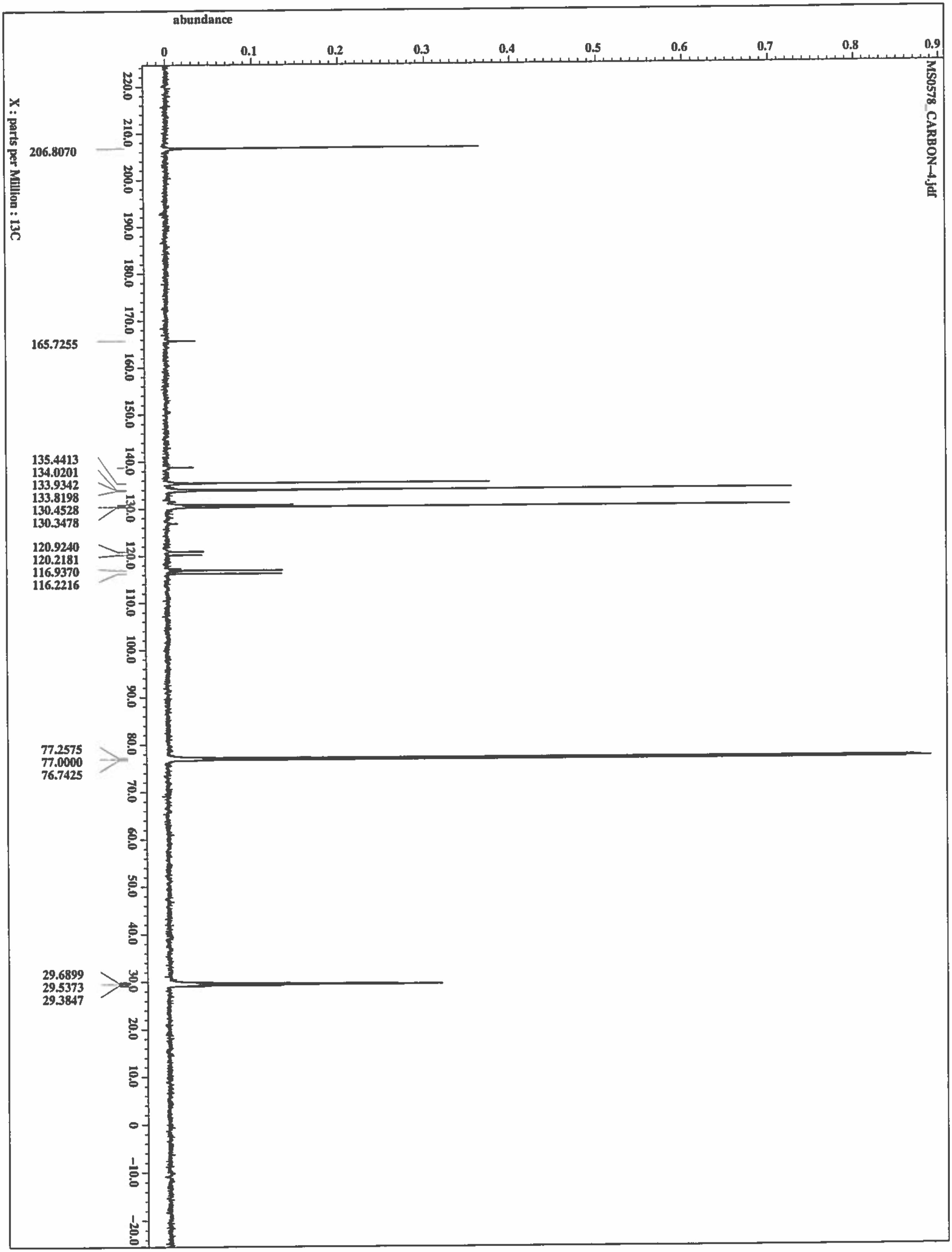

abundance
0
0.1
0.2
0.3
0.4
0.5
0.6
0.7
0.8
0.9
MS0578_CARBON-4.jdf
X : parts per Million : 13C
220.0
210.0
200.0
190.0
180.0
170.0
160.0
150.0
140.0
130.0
120.0
110.0
100.0
90.0
80.0
70.0
60.0
50.0
40.0
30.0
20.0
10.0
0
-10.0
-20.0
206.8070
165.7255
135.4413
134.0201
133.9342
133.8198
130.4528
130.3478
120.9240
120.2181
116.9370
116.2216
77.2575
77.0000
76.7425
29.6899
29.5373
29.3847

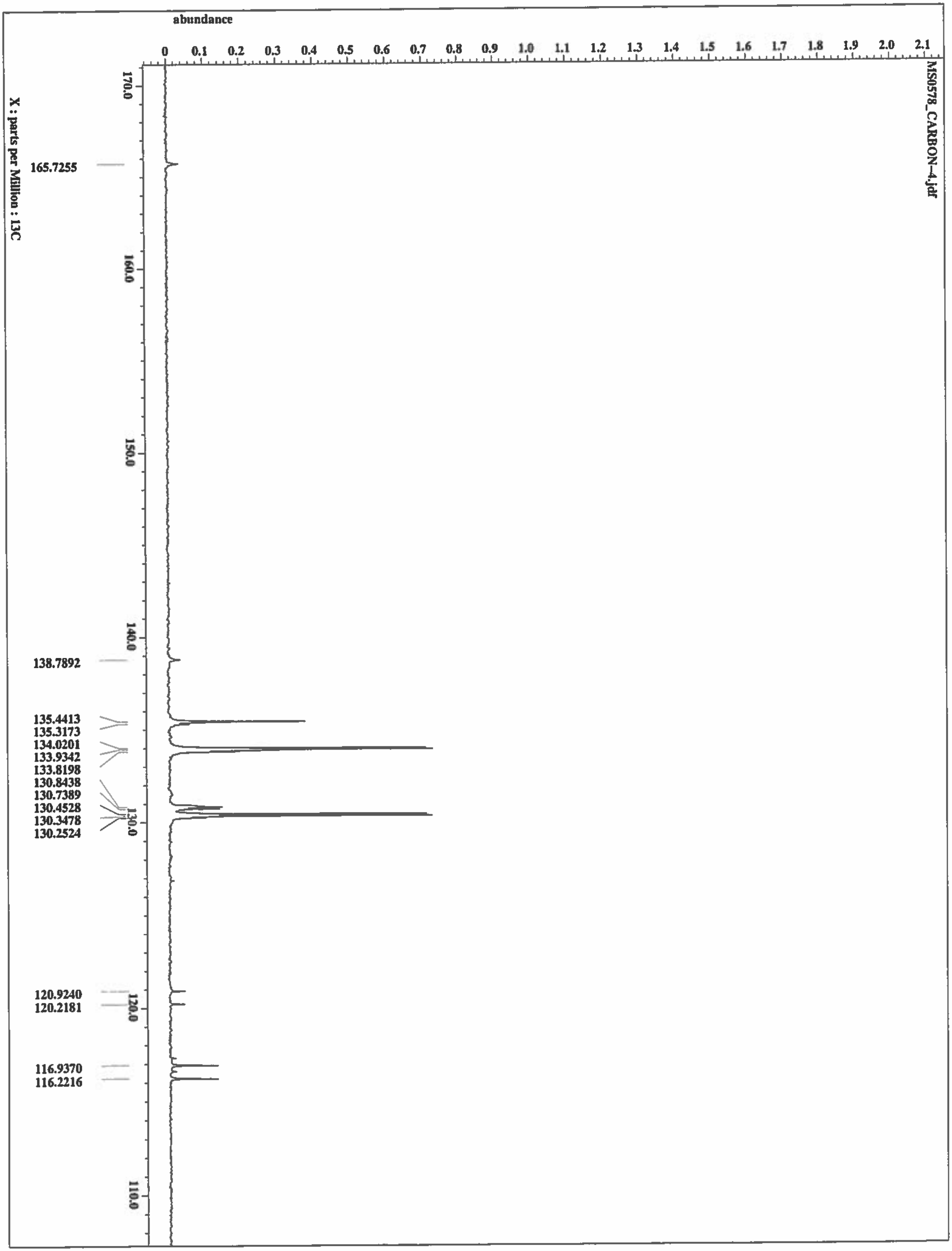
abundance
0
0.1
0.2
0.3
0.4
0.5
0.6
0.7
0.8
0.9
1.0
1.1
1.2
1.3
1.4
1.5
1.6
1.7
1.8
1.9
2.0
2.1
X : parts per Million : 13C
MS0578_CARBON-4.jdf
170.0
160.0
150.0
140.0
130.0
120.0
110.0
165.7255
138.7892
135.4413
135.3173
134.0201
133.9342
133.8198
130.8438
130.7389
130.4528
130.3478
130.2524
120.9240
120.2181
116.9370
116.2216

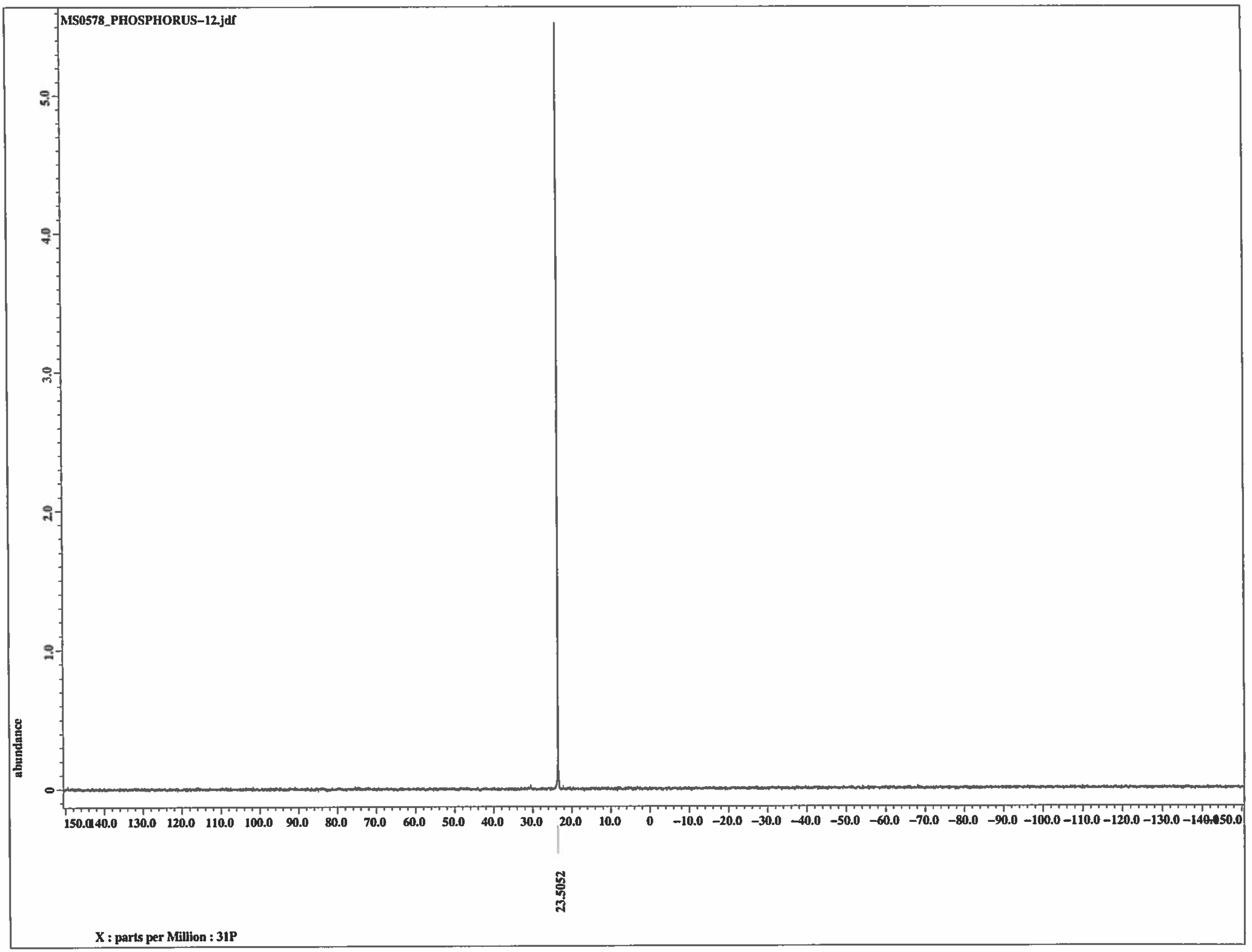
MS0578_PHOSPHORUS-12.jdf
abundance
0
1.0
2.0
3.0
4.0
5.0
150.0 140.0 130.0 120.0 110.0 100.0 90.0 80.0 70.0 60.0 50.0 40.0 30.0 20.0 10.0 0 -10.0 -20.0 -30.0 -40.0 -50.0 -60.0 -70.0 -80.0 -90.0 -100.0 -110.0 -120.0 -130.0 -140.0 -150.0
23.5052
X : parts per Million : 31P

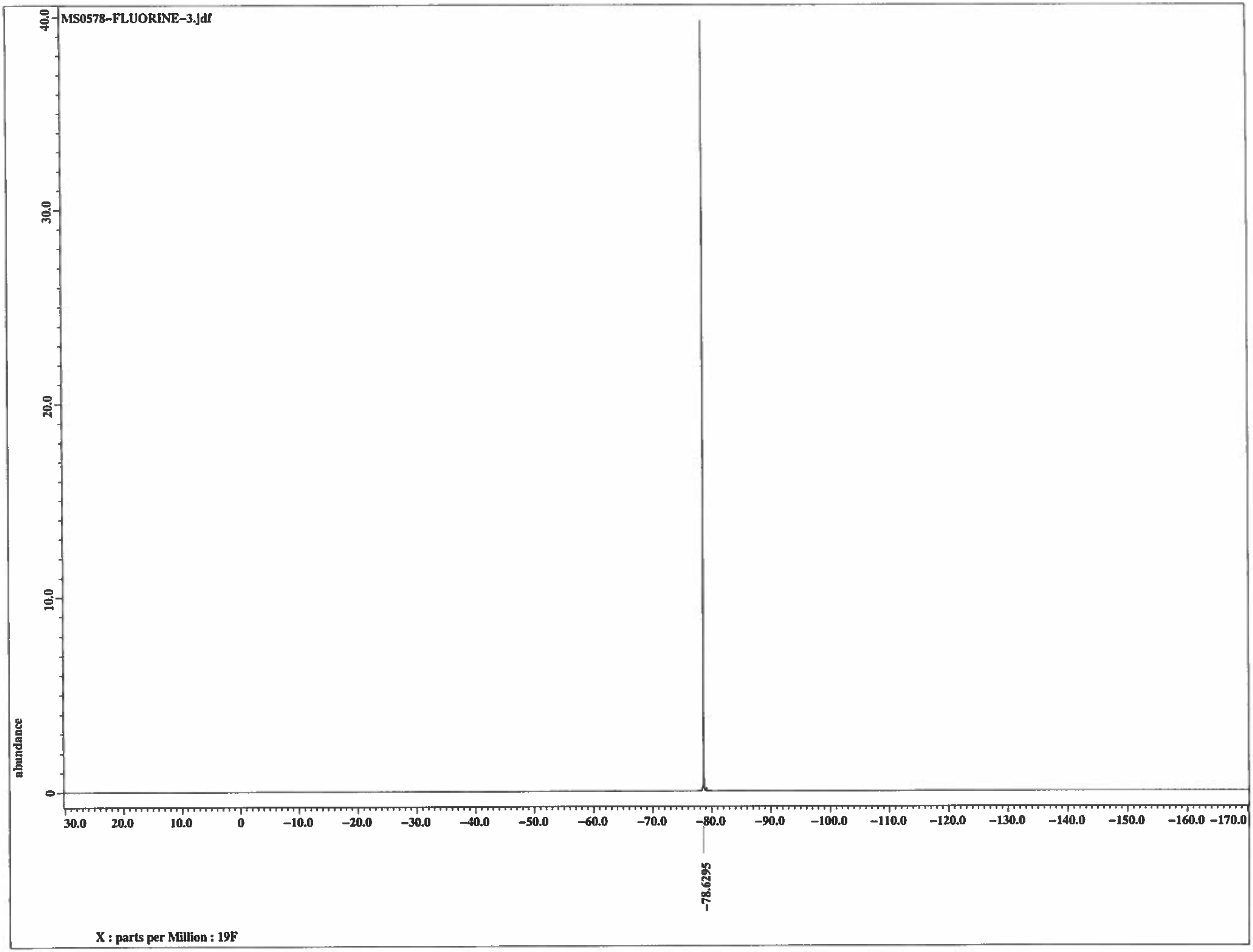
MS0578-FLUORINE-3.jdf
abundance
40.0
30.0
20.0
10.0
0
30.0 20.0 10.0 0 −10.0 −20.0 −30.0 −40.0 −50.0 −60.0 −70.0 −80.0 −90.0 −100.0 −110.0 −120.0 −130.0 −140.0 −150.0 −160.0 −170.0
−78.6295
X : parts per Million : 19F

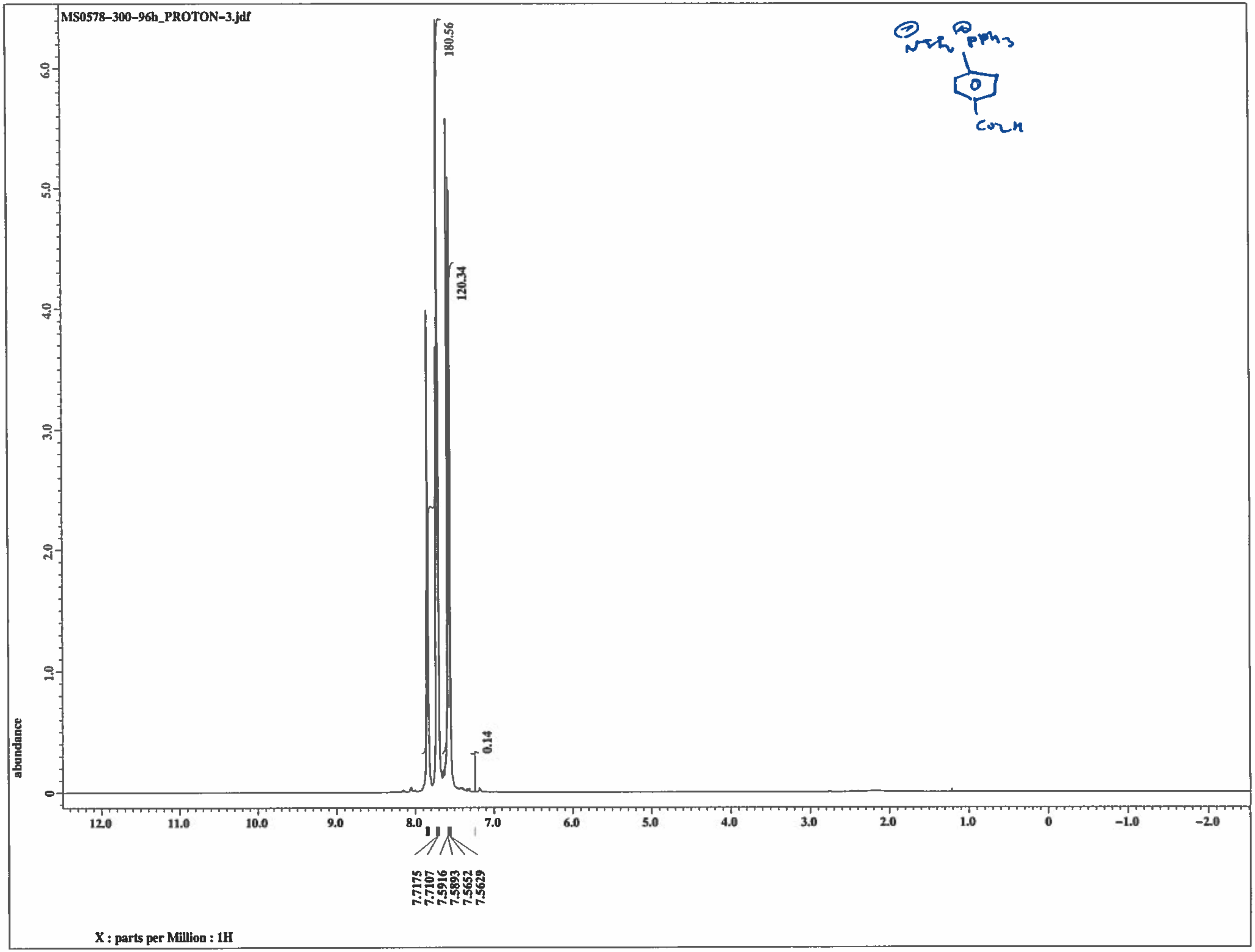
MS0578-300-96h_PROTON-3.jdf
abundance
0
1.0
2.0
3.0
4.0
5.0
6.0
180.56
120.34
0.14
12.0
11.0
10.0
9.0
8.0
7.0
6.0
5.0
4.0
3.0
2.0
1.0
0
-1.0
-2.0
7.7175
7.7107
7.5916
7.5893
7.5652
7.5629
X : parts per Million : 1H

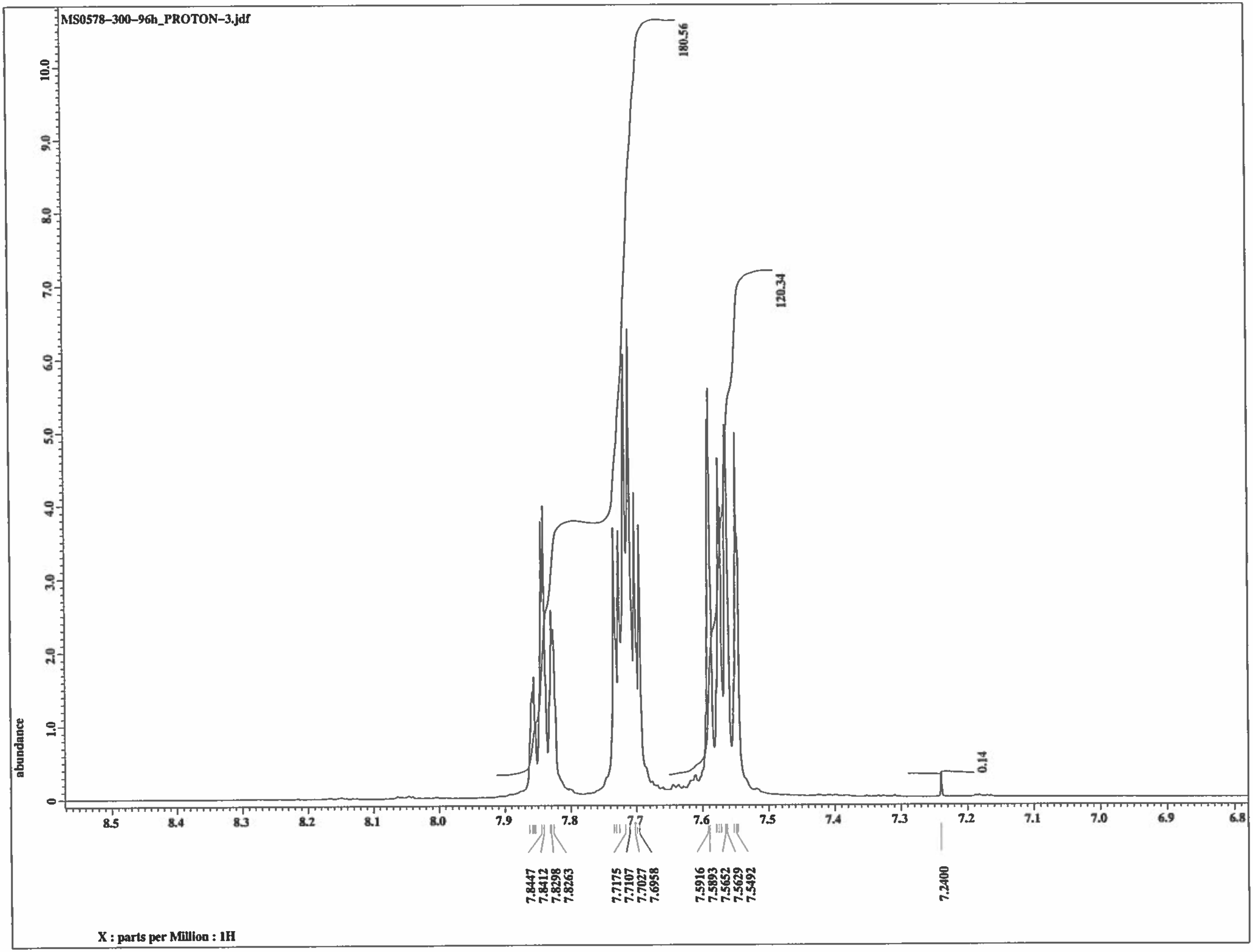
MS0578-300-96h_PROTON-3.jdf
abundance
0
1.0
2.0
3.0
4.0
5.0
6.0
7.0
8.0
9.0
10.0
180.56
120.34
0.14
8.5
8.4
8.3
8.2
8.1
8.0
7.9
7.8
7.7
7.6
7.5
7.4
7.3
7.2
7.1
7.0
6.9
6.8
7.8447
7.8412
7.8298
7.8263
7.7175
7.7107
7.7027
7.6958
7.5916
7.5893
7.5652
7.5629
7.5492
7.2400
X : parts per Million : 1H

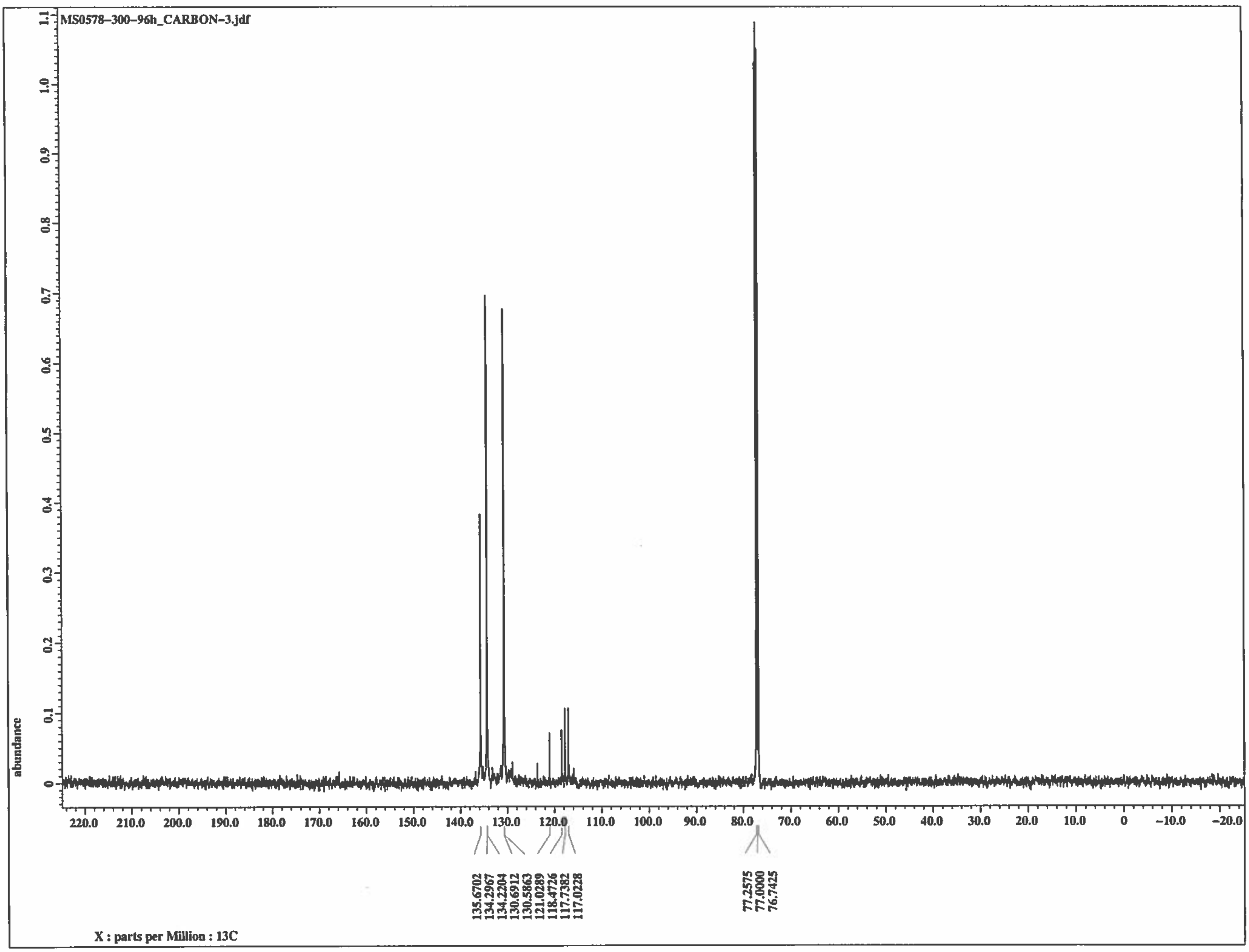
MS0578-300-96h_CARBON-3.jdf
abundance
X : parts per Million : 13C
135.6702
134.2967
134.2204
130.6912
130.5863
121.0289
118.4726
117.7382
117.0228
77.2575
77.0000
76.7425

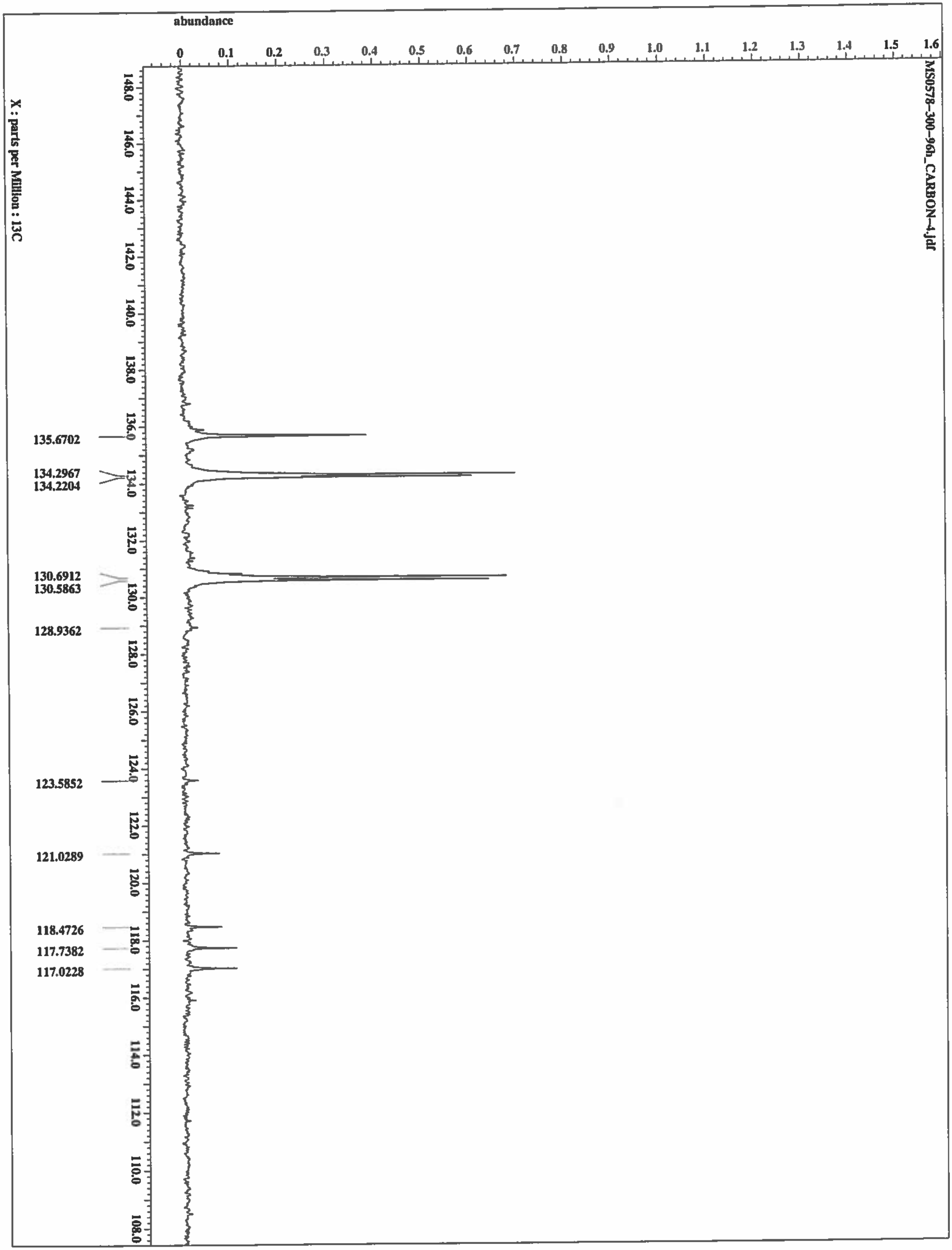
abundance
MS0578-300-96h_CARBON-4.jdf
X : parts per Million : 13C
135.6702
134.2967
134.2204
130.6912
130.5863
128.9362
123.5852
121.0289
118.4726
117.7382
117.0228

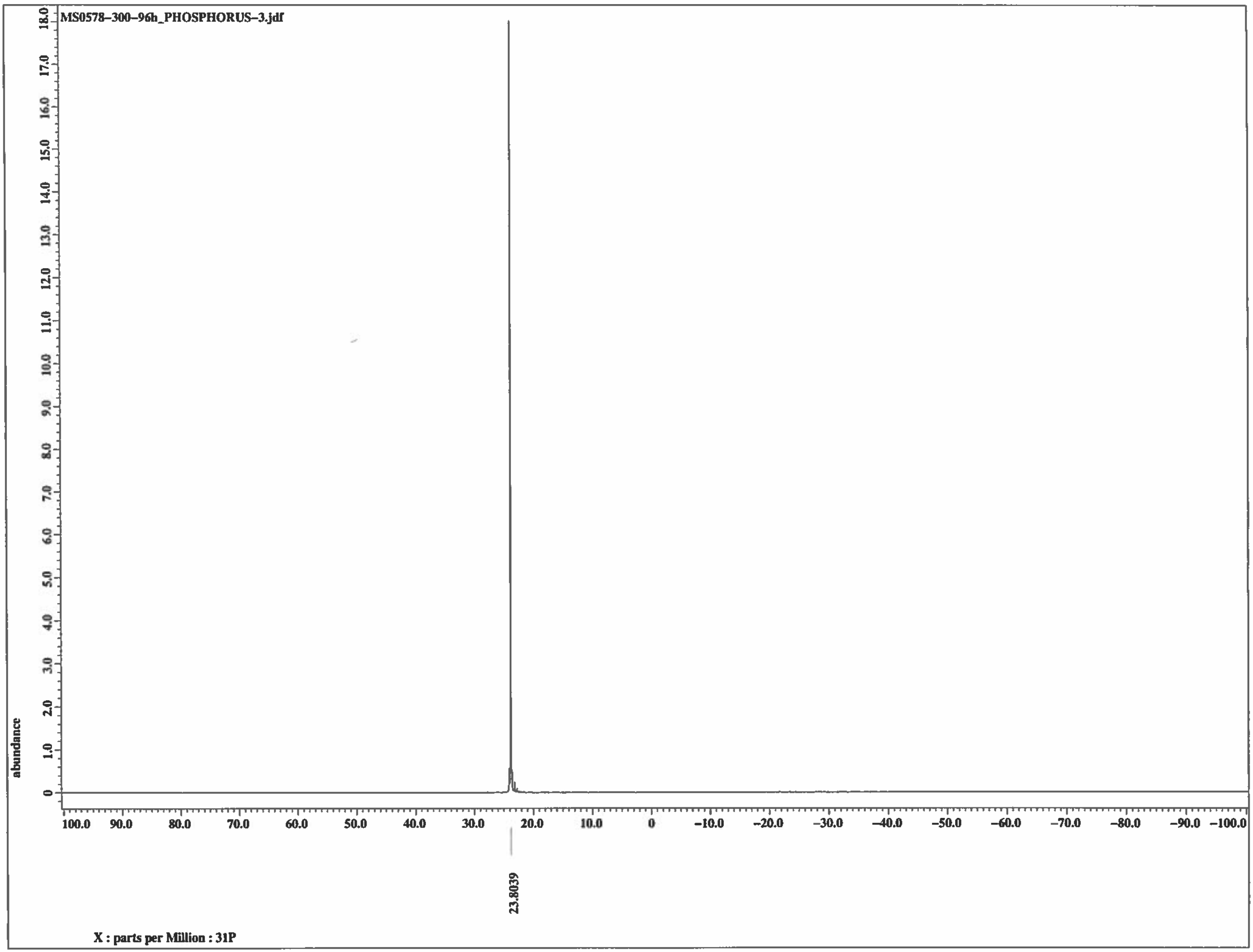

MS0578–300–96h_PHOSPHORUS–3.jdf
abundance
0
1.0
2.0
3.0
4.0
5.0
6.0
7.0
8.0
9.0
10.0
11.0
12.0
13.0
14.0
15.0
16.0
17.0
18.0
100.0
90.0
80.0
70.0
60.0
50.0
40.0
30.0
20.0
10.0
0
–10.0
–20.0
–30.0
–40.0
–50.0
–60.0
–70.0
–80.0
–90.0
–100.0
23.8039
X : parts per Million : 31P

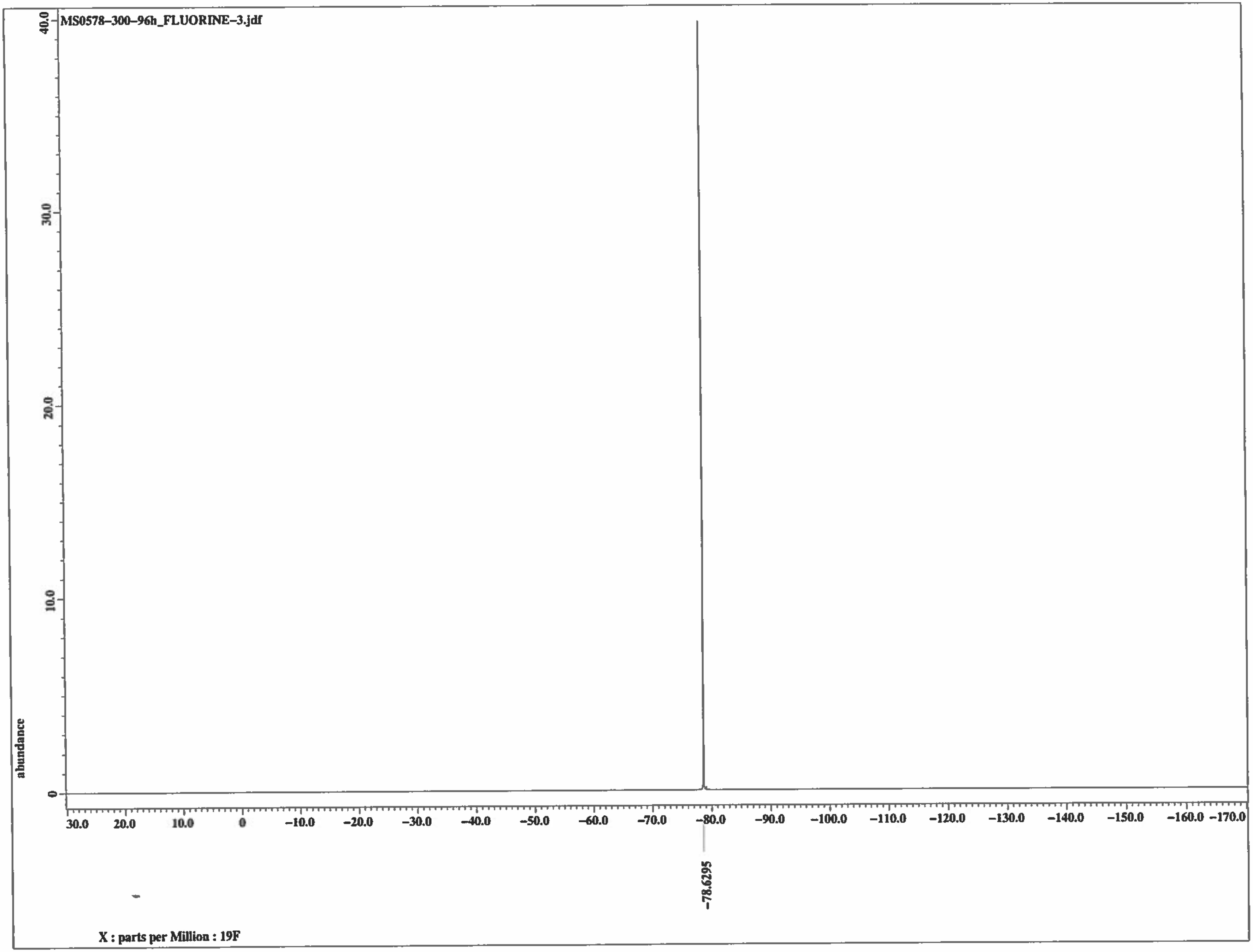
MS0578-300-96h_FLUORINE-3.jdf
abundance
40.0
30.0
20.0
10.0
0
30.0 20.0 10.0 0 -10.0 -20.0 -30.0 -40.0 -50.0 -60.0 -70.0 -80.0 -90.0 -100.0 -110.0 -120.0 -130.0 -140.0 -150.0 -160.0 -170.0
-78.6295
X : parts per Million : 19F

Compound 6 Pre- and Post-heating NMR Spectra

Temperature of Post-heating samples noted in upper left corner of each spectrum

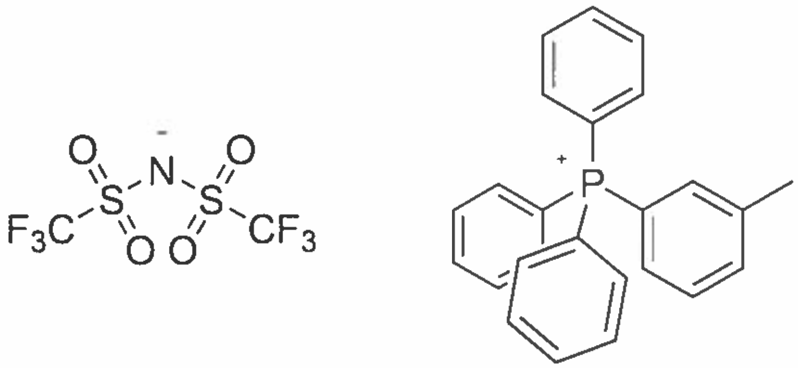

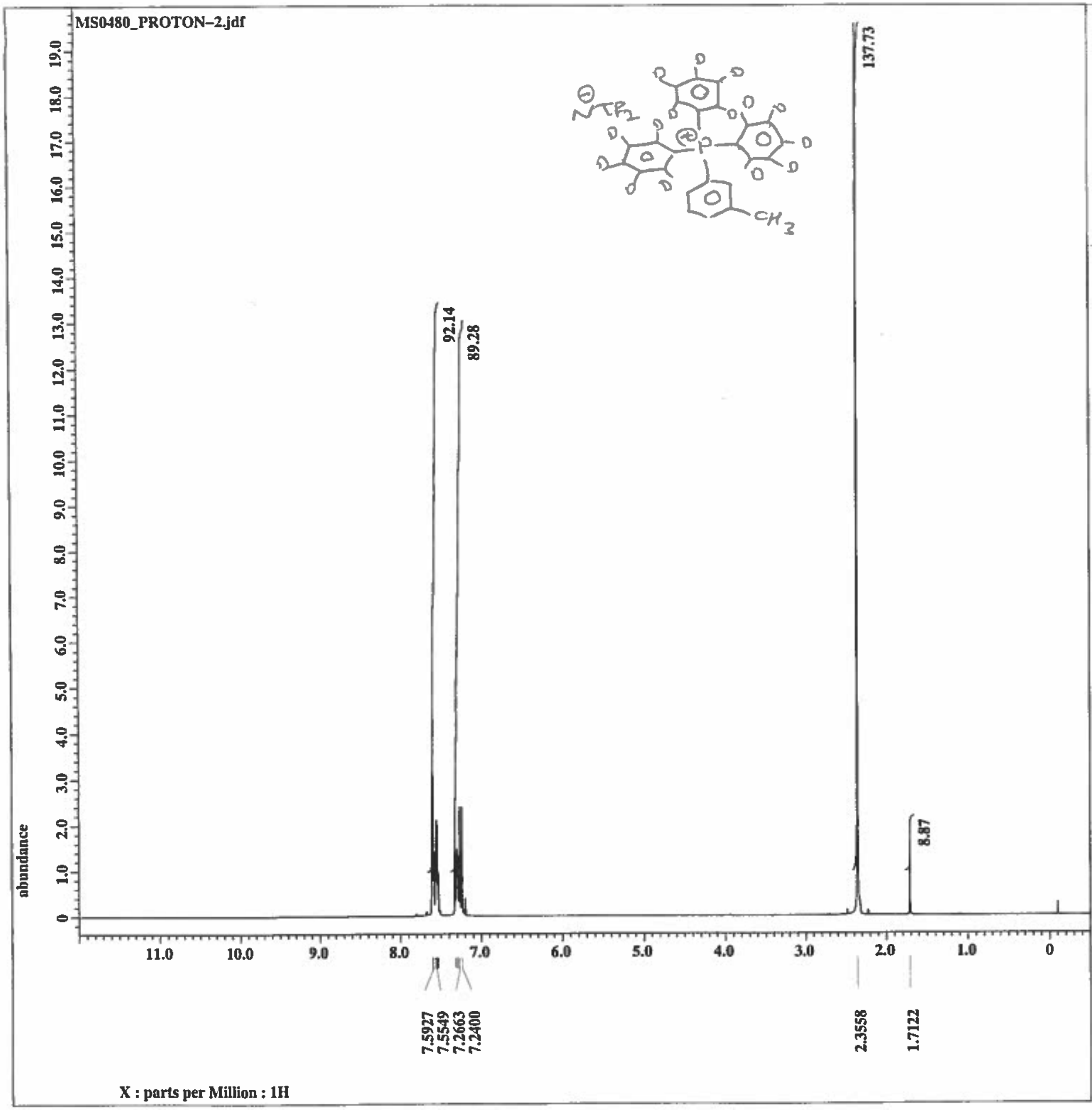

Filename	= MS0480_PROTON-2.jdf
Author	= Jim Davis
Experiment	= single_pulse.ex2
Sample_id	= MS0480
Solvent	= CHLOROFORM-D
Changer_sample	= 2
Creation_time	= 22-JUN-2018 16:29:37
Revision_time	= 22-JUN-2018 16:07:10
Current_time	= 22-JUN-2018 16:07:10
Data_format	= 1D COMPLEX
Dim_size	= 13107
Dim_title	= 1H
Dim_units	= [ppm]
Dimensions	= X
Site	= ECA 500
Spectrometer	= JNM-ECA500
Field_strength	= 11.7473579[T] (500[MH
X_acq_duration	= 1.74587904[s]
X_domain	= 1H
X_freq	= 500.15991521[MHz]
X_offset	= 5.0[ppm]
X_points	= 16384
X_prescans	= 1
X_resolution	= 0.57277737[Hz]
X_sweep	= 9.38438438[kHz]
Irr_domain	= 1H
Irr_freq	= 500.15991521[MHz]
Irr_offset	= 5.0[ppm]
Tri_domain	= 1H
Tri_freq	= 500.15991521[MHz]
Tri_offset	= 5.0[ppm]
Clipped	= FALSE
Mod_return	= 1
Scans	= 16
Total_scans	= 16
X_90_width	= 12.4[us]
X_acq_time	= 1.74587904[s]
X_angle	= 45[deg]
X_atn	= 4[dB]
X_pulse	= 6.2[us]
Irr_mode	= Off
Tri_mode	= Off
Dante_presat	= FALSE
Initial_wait	= 1[s]
Recvr_gain	= 36
Relaxation_delay	= 4[s]
Repetition_time	= 5.74587904[s]
Temp_get	= 22[dC]

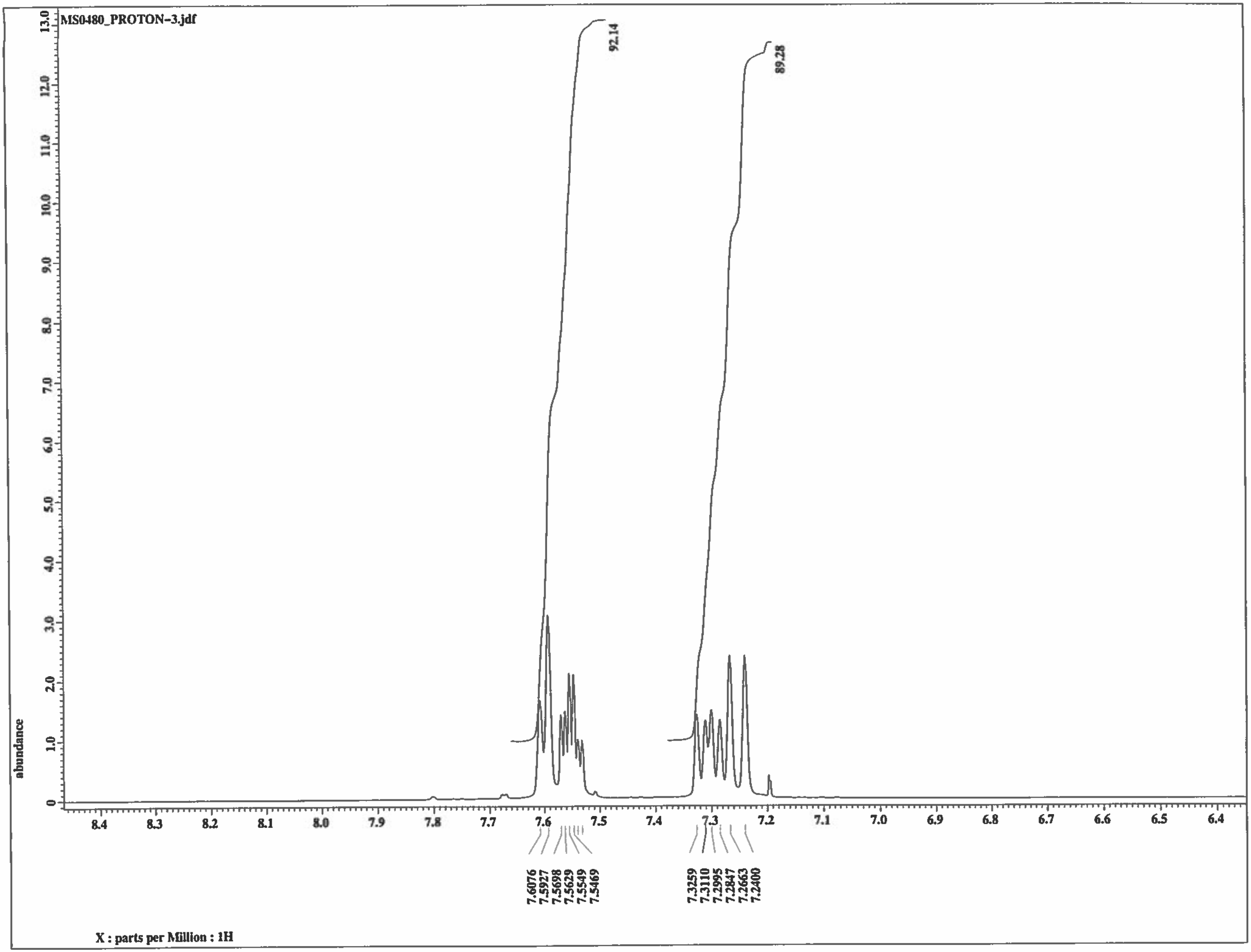
MS0480_PROTON-3.jdf
abundance
0
1.0
2.0
3.0
4.0
5.0
6.0
7.0
8.0
9.0
10.0
11.0
12.0
13.0
92.14
89.28
8.4
8.3
8.2
8.1
8.0
7.9
7.8
7.7
7.6
7.5
7.4
7.3
7.2
7.1
7.0
6.9
6.8
6.7
6.6
6.5
6.4
7.6076
7.5927
7.5698
7.5629
7.5549
7.5469
7.3259
7.3110
7.2995
7.2847
7.2663
7.2400
X : parts per Million : 1H

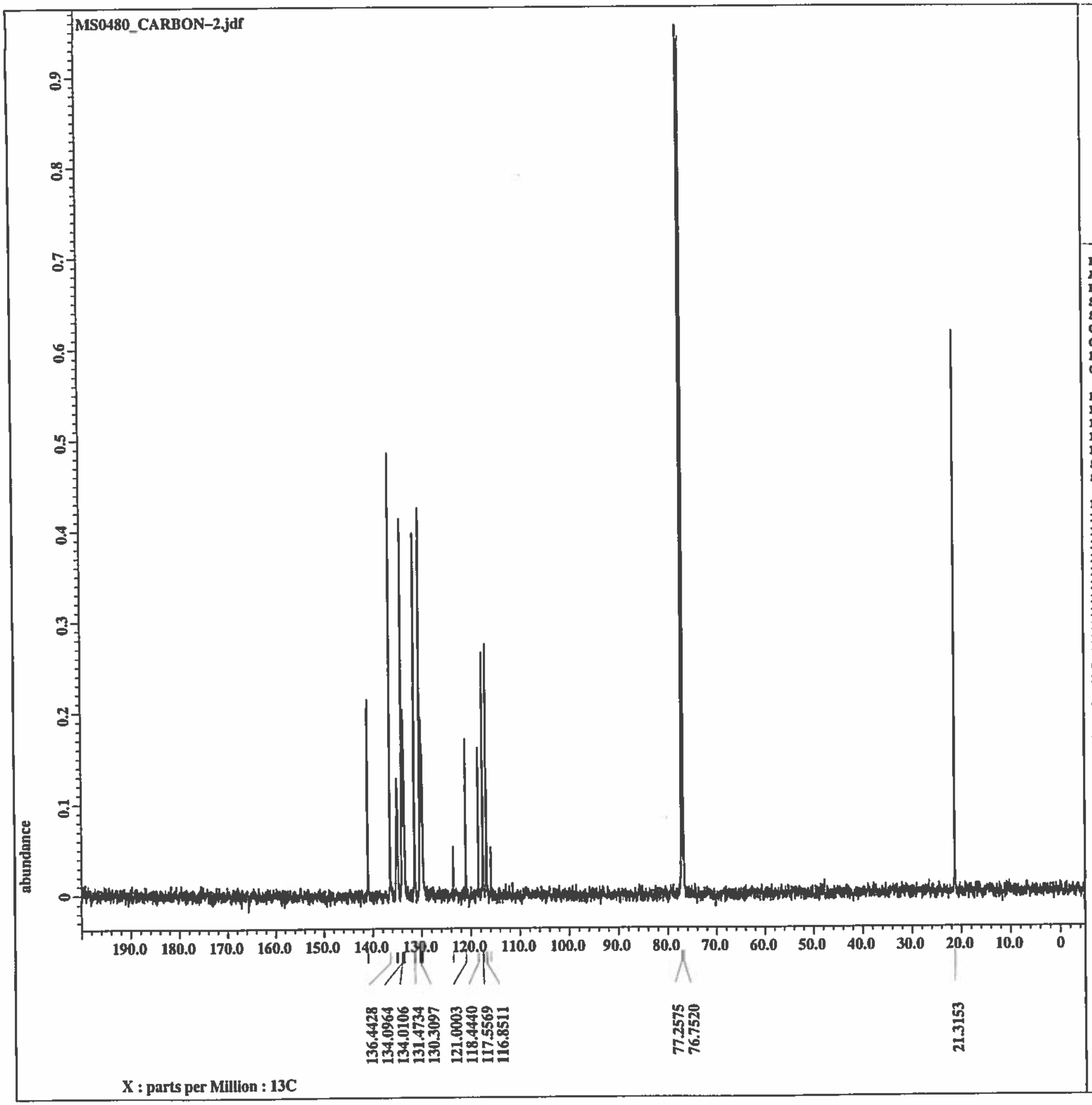

Filename = MS0480_CARBON-2.jdf
Author = Jim Davis
Experiment = single_pulse_dec
Sample_id = MS0480
Solvent = CHLOROFORM-D
Changer_sample = 2
Creation_time = 22-JUN-2018 16:44:03
Revision_time = 22-JUN-2018 16:21:36
Current_time = 22-JUN-2018 16:21:36

Data_format = 1D COMPLEX
Dim_size = 26214
Dim_title = 13C
Dim_units = [ppm]
Dimensions = X
Site = ECA 500
Spectrometer = JNM-ECA500

Field_strength = 11.7473579[T] (500[MH
X_acq_duration = 0.83361792[s]
X_domain = 13C
X_freq = 125.76529768[MHz]
X_offset = 100[ppm]
X_points = 32768
X_prescans = 4
X_resolution = 1.19959034[Hz]
X_sweep = 39.3081761[kHz]
Irr_domain = 1H
Irr_freq = 500.15991521[MHz]
Irr_offset = 5.0[ppm]
Clipped = FALSE
Mod_return = 1
Scans = 256
Total_scans = 256

X_90_width = 13.2[us]
X_acq_time = 0.83361792[s]
X_angle = 30[deg]
X_atn = 6[dB]
X_pulse = 4.4[us]
Irr_atn_dec = 20.7[dB]
Irr_atn_noe = 20.7[dB]
Irr_noise = WALTZ
Decoupling = TRUE
Initial_wait = 1[s]
Noe = TRUE
Noe_time = 2[s]
Recvr_gain = 60
Relaxation_delay = 2[s]
Repetition_time = 2.83361792[s]
Temp_get = 22.6[dC]

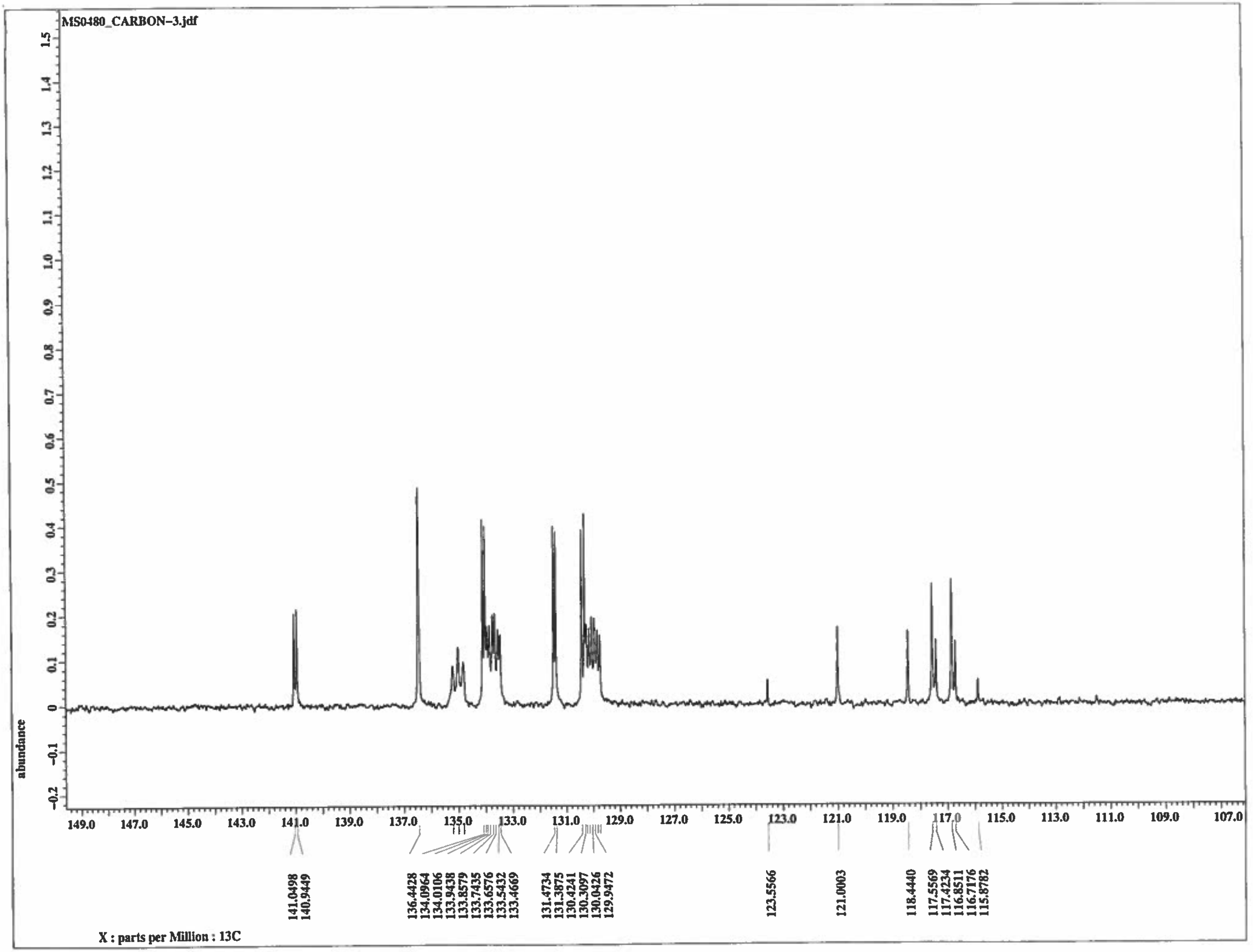

MS0480_CARBON-3.jdf
abundance
1.5 1.4 1.3 1.2 1.1 1.0 0.9 0.8 0.7 0.6 0.5 0.4 0.3 0.2 0.1 0 -0.1 -0.2
149.0 147.0 145.0 143.0 141.0 139.0 137.0 135.0 133.0 131.0 129.0 127.0 125.0 123.0 121.0 119.0 117.0 115.0 113.0 111.0 109.0 107.0
141.0498
140.9449
136.4428
134.0964
134.0106
133.9438
133.8579
133.7435
133.6576
133.5432
133.4669
131.4734
131.3875
130.4241
130.3097
130.0426
129.9472
123.5566
121.0003
118.4440
117.5569
117.4234
116.8511
116.7176
115.8782
X : parts per Million : 13C

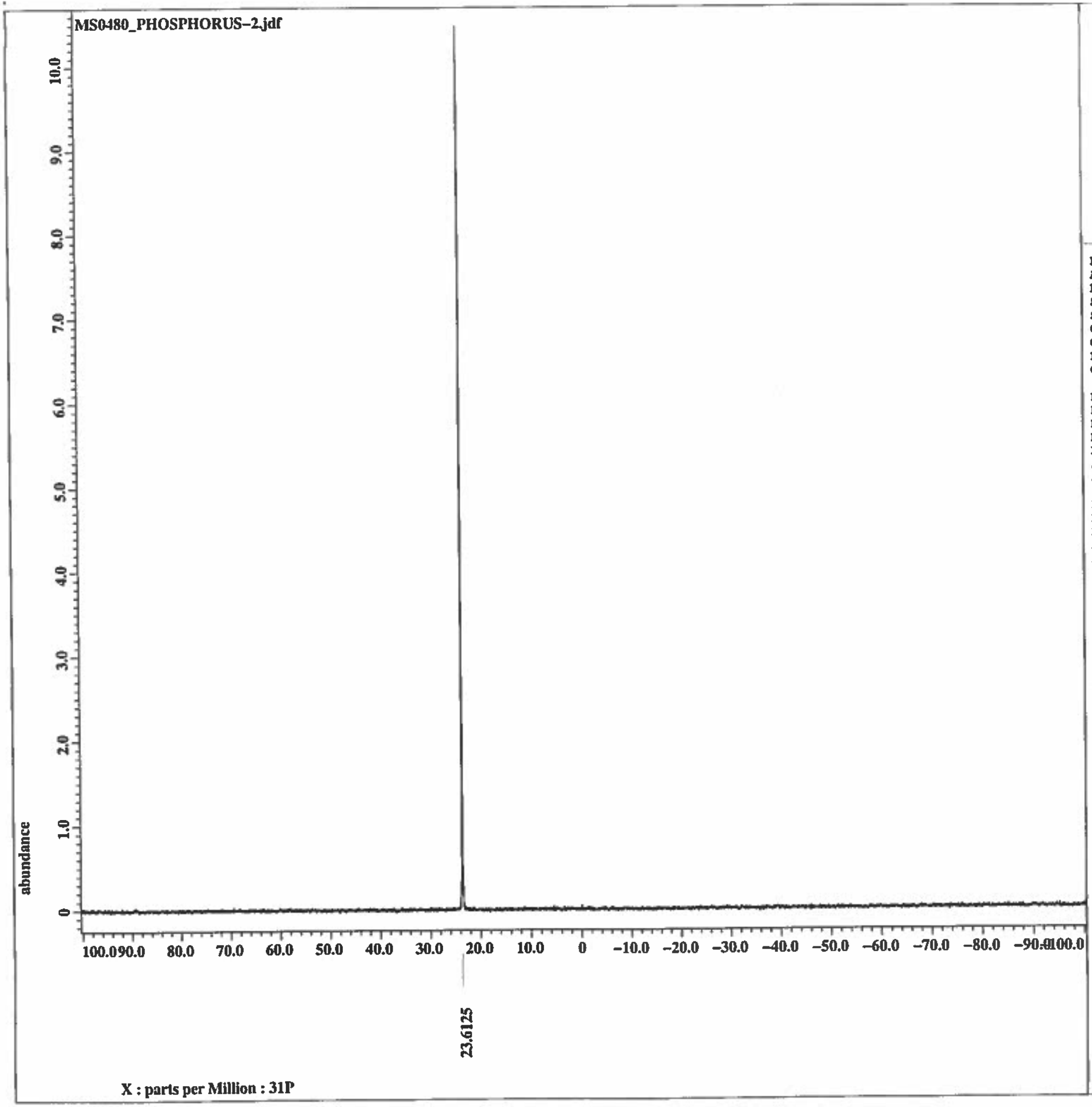

Filename	= MS0480_PHOSPHORUS-2.j
Author	= Jim Davis
Experiment	= single_pulse_dec
Sample_id	= MS0480
Solvent	= CHLOROFORM-D
Changer_sample	= 2
Creation_time	= 22-JUN-2018 16:25:52
Revision_time	= 22-JUN-2018 16:03:24
Current_time	= 22-JUN-2018 16:03:24
Data_format	= 1D COMPLEX
Dim_size	= 26214
Dim_title	= 31P
Dim_units	= [ppm]
Dimensions	= X
Site	= ECA 500
Spectrometer	= JNM-ECA500
Field_strength	= 11.7473579[T] (500[MH
X_acq_duration	= 0.64487424[s]
X_domain	= 31P
X_freq	= 202.46831075[MHz]
X_offset	= 0[ppm]
X_points	= 32768
X_prescans	= 4
X_resolution	= 1.55068995[Hz]
X_sweep	= 50.81300813[kHz]
Irr_domain	= 1H
Irr_freq	= 500.15991521[MHz]
Irr_offset	= 5.0[ppm]
Clipped	= FALSE
Mod_return	= 1
Scans	= 25
Total_scans	= 25
X_90_width	= 14.687[us]
X_acq_time	= 0.64487424[s]
X_angle	= 30[deg]
X_atn	= 5[dB]
X_pulse	= 4.89566667[us]
Irr_atn_dec	= 20.7[dB]
Irr_atn_noe	= 20.7[dB]
Irr_noise	= WALTZ
Decoupling	= TRUE
Initial_wait	= 1[s]
Noe	= TRUE
Noe_time	= 2[s]
Recvr_gain	= 58
Relaxation_delay	= 2[s]
Repetition_time	= 2.64487424[s]
Temp_get	= 22.3[dC]

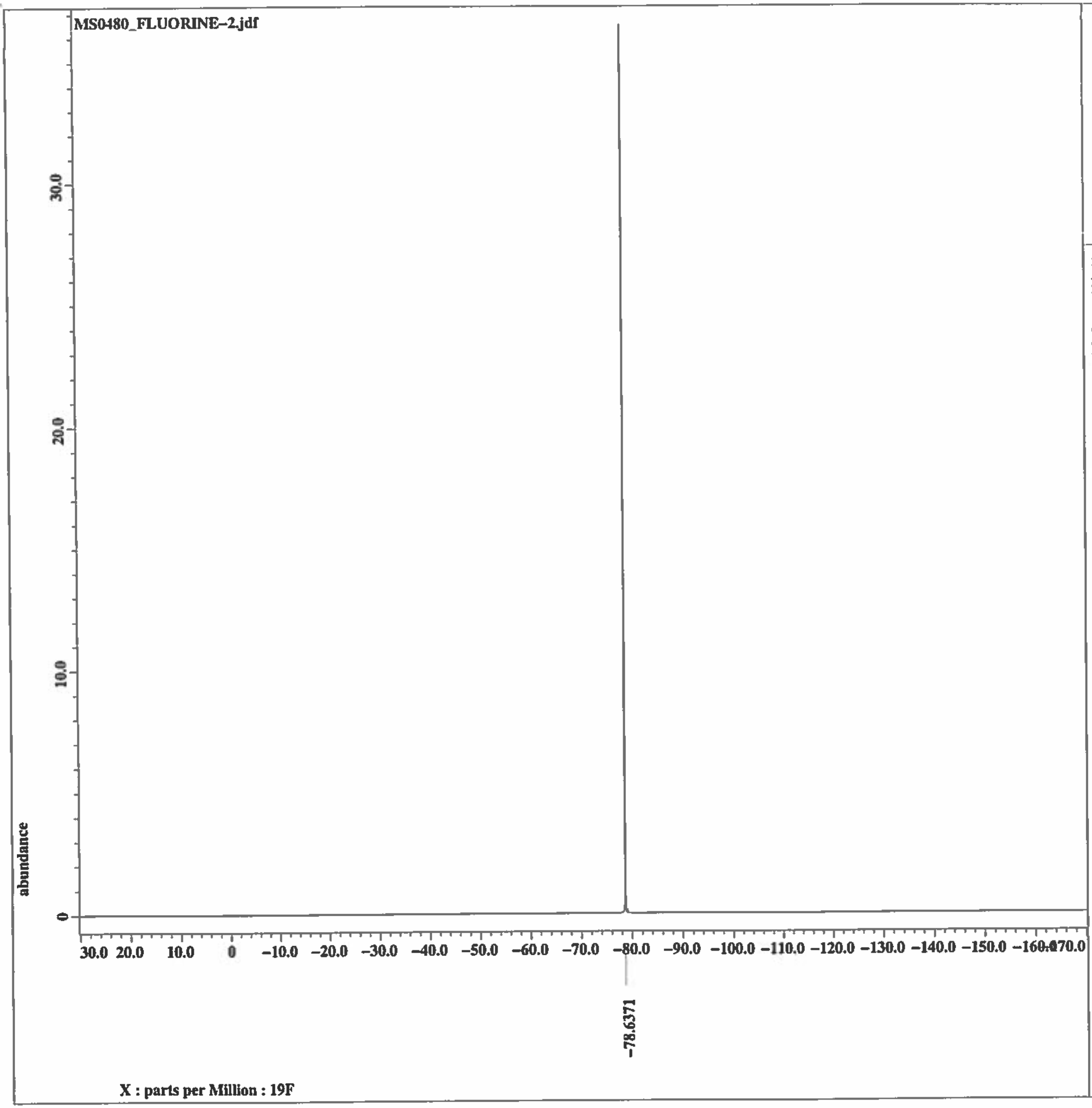

Filename	= MS0480_FLUORINE-2.jdf
Author	= Jim Davis
Experiment	= single_pulse.ex2
Sample_id	= MS0480
Solvent	= CHLOROFORM-D
Changer_sample	= 2
Creation_time	= 22-JUN-2018 16:46:59
Revision_time	= 22-JUN-2018 16:24:31
Current_time	= 22-JUN-2018 16:24:31
Data_format	= 1D COMPLEX
Dim_size	= 52428
Dim_title	= 19F
Dim_units	= [ppm]
Dimensions	= X
Site	= ECA 500
Spectrometer	= JNM-ECA500
Field_strength	= 11.7473579[T] (500[MH
X_acq_duration	= 0.55574528[s]
X_domain	= 19F
X_freq	= 470.62046084[MHz]
X_offset	= -70[ppm]
X_points	= 65536
X_prescans	= 1
X_resolution	= 1.7993855[Hz]
X_sweep	= 117.9245283[kHz]
Irr_domain	= 19F
Irr_freq	= 470.62046084[MHz]
Irr_offset	= 5[ppm]
Tri_domain	= 19F
Tri_freq	= 470.62046084[MHz]
Tri_offset	= 5[ppm]
Clipped	= FALSE
Mod_return	= 1
Scans	= 16
Total_scans	= 16
X_90_width	= 13.1[us]
X_acq_time	= 0.55574528[s]
X_angle	= 45[deg]
X_atn	= 2.5[dB]
X_pulse	= 6.55[us]
Irr_mode	= Off
Tri_mode	= Off
Dante_presat	= FALSE
Initial_wait	= 1[s]
Recvr_gain	= 38
Relaxation_delay	= 4[s]
Repetition_time	= 4.55574528[s]
Temp_get	= 22.2[dC]

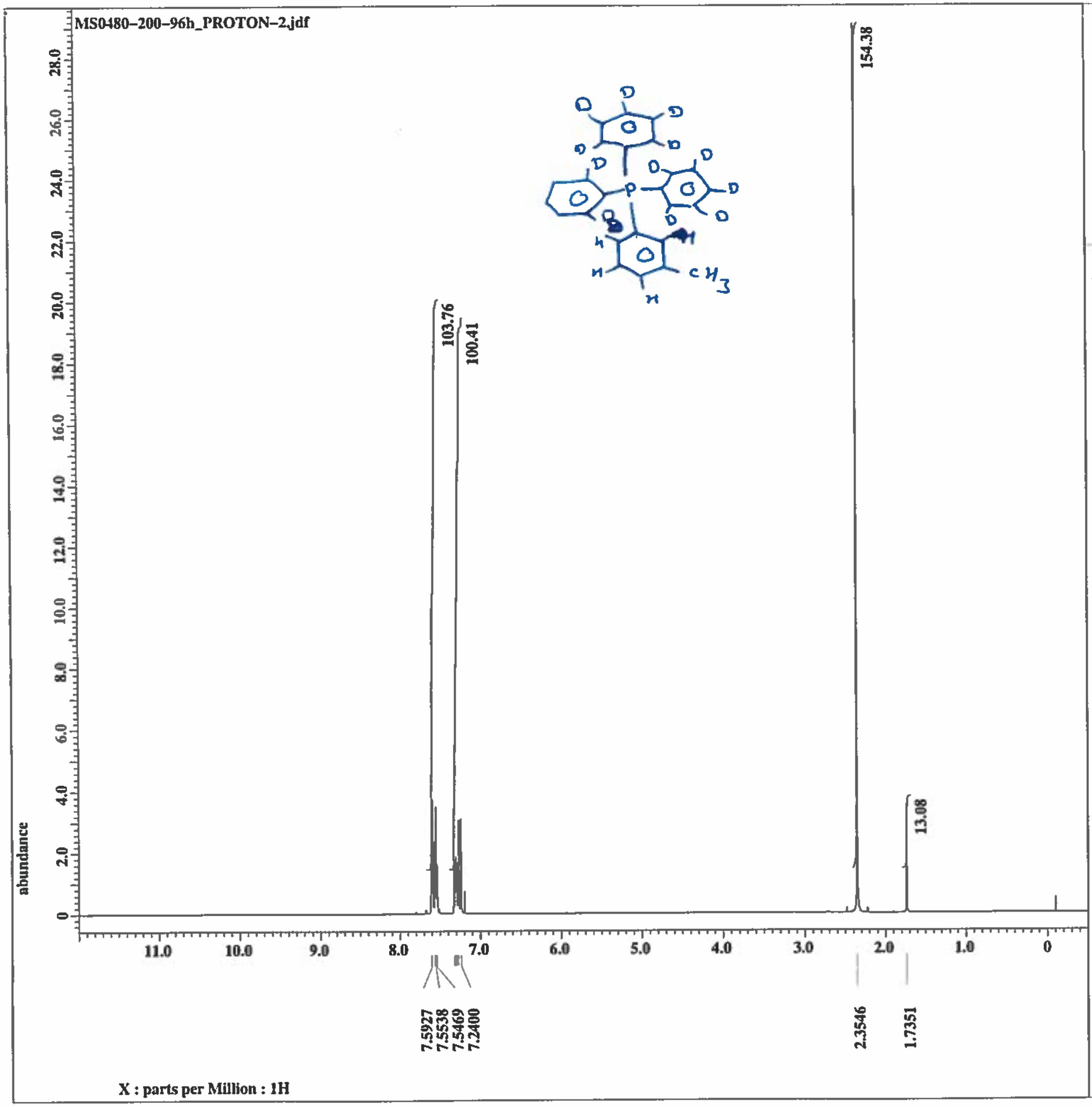

Filename	= MS0480-200-96h_PROTON
Author	= Jim Davis
Experiment	= single_pulse.ex2
Sample_id	= MS0480-200-96h
Solvent	= CHLOROFORM-D
Changer_sample	= 6
Creation_time	= 27-JUN-2018 08:42:39
Revision_time	= 27-JUN-2018 08:19:47
Current_time	= 27-JUN-2018 08:19:47
Data_format	= 1D COMPLEX
Dim_size	= 13107
Dim_title	= 1H
Dim_units	= [ppm]
Dimensions	= X
Site	= ECA 500
Spectrometer	= JNM-ECA500
Field_strength	= 11.7473579[T] (500[MH
X_acq_duration	= 1.74587904[s]
X_domain	= 1H
X_freq	= 500.15991521[MHz]
X_offset	= 5.0[ppm]
X_points	= 16384
X_prescans	= 1
X_resolution	= 0.57277737[Hz]
X_sweep	= 9.38438438[kHz]
Irr_domain	= 1H
Irr_freq	= 500.15991521[MHz]
Irr_offset	= 5.0[ppm]
Tri_domain	= 1H
Tri_freq	= 500.15991521[MHz]
Tri_offset	= 5.0[ppm]
Clipped	= FALSE
Mod_return	= 1
Scans	= 16
Total_scans	= 16
X_90_width	= 12.4[us]
X_acq_time	= 1.74587904[s]
X_angle	= 45[deg]
X_atn	= 4[dB]
X_pulse	= 6.2[us]
Irr_mode	= Off
Tri_mode	= Off
Dante_presat	= FALSE
Initial_wait	= 1[s]
Recvr_gain	= 36
Relaxation_delay	= 4[s]
Repetition_time	= 5.74587904[s]
Temp_get	= 21.6[dC]

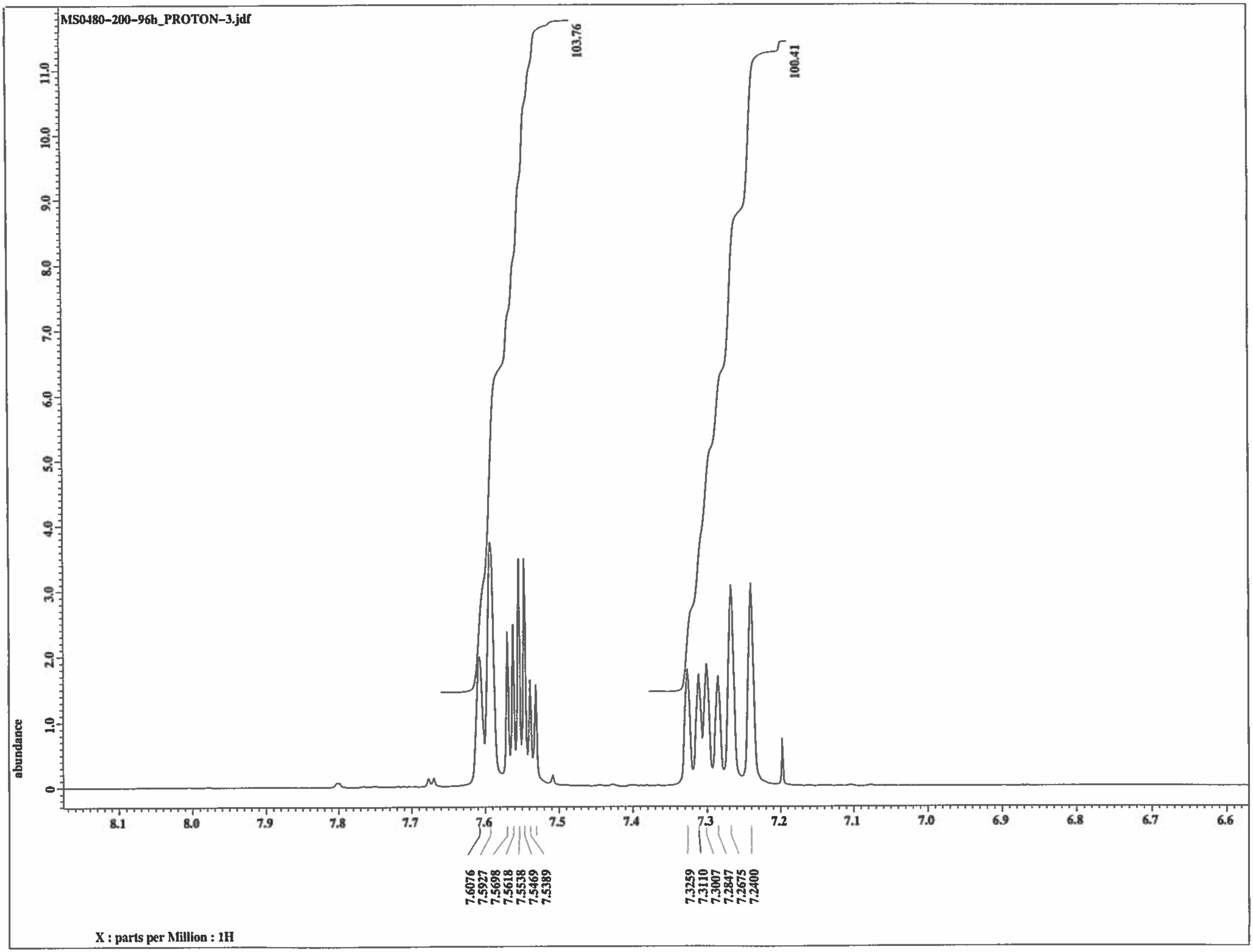

MS0480-200-96h_PROTON-3.jdf
abundance
0
1.0
2.0
3.0
4.0
5.0
6.0
7.0
8.0
9.0
10.0
11.0
103.76
100.41
8.1
8.0
7.9
7.8
7.7
7.6
7.5
7.4
7.3
7.2
7.1
7.0
6.9
6.8
6.7
6.6
7.6076
7.5927
7.5698
7.5618
7.5538
7.5469
7.5389
7.3259
7.3110
7.3007
7.2847
7.2675
7.2400
X : parts per Million : 1H

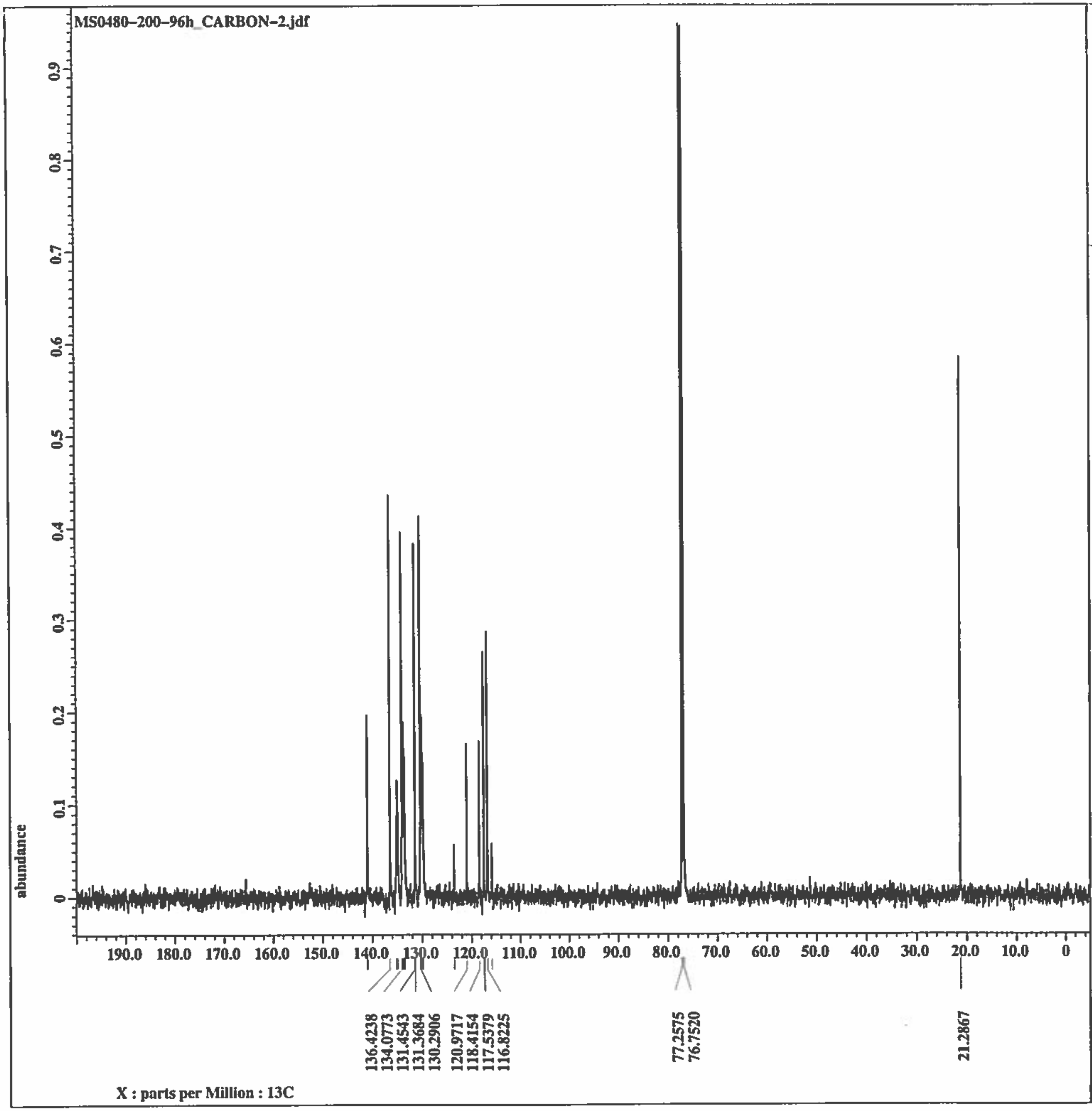

Filename	= MS0480-200-96h_CARBON
Author	= Jim Davis
Experiment	= single_pulse_dec
Sample_id	= MS0480-200-96h
Solvent	= CHLOROFORM-D
Changer_sample	= 6
Creation_time	= 27-JUN-2018 08:51:14
Revision_time	= 27-JUN-2018 08:28:22
Current_time	= 27-JUN-2018 08:28:22
Data_format	= 1D COMPLEX
Dim_size	= 26214
Dim_title	= 13C
Dim_units	= [ppm]
Dimensions	= X
Site	= ECA 500
Spectrometer	= JNM-ECA500
Field_strength	= 11.7473579[T] (500[MH
X_acq_duration	= 0.83361792[s]
X_domain	= 13C
X_freq	= 125.76529768[MHz]
X_offset	= 100[ppm]
X_points	= 32768
X_prescans	= 4
X_resolution	= 1.19959034[Hz]
X_sweep	= 39.3081761[kHz]
Irr_domain	= 1H
Irr_freq	= 500.15991521[MHz]
Irr_offset	= 5.0[ppm]
Clipped	= FALSE
Mod_return	= 1
Scans	= 134
Total_scans	= 134
X_90_width	= 13.2[us]
X_acq_time	= 0.83361792[s]
X_angle	= 30[deg]
X_atn	= 6[dB]
X_pulse	= 4.4[us]
Irr_atn_dec	= 20.7[dB]
Irr_atn_noe	= 20.7[dB]
Irr_noise	= WALTZ
Decoupling	= TRUE
Initial_wait	= 1[s]
Noe	= TRUE
Noe_time	= 2[s]
Recvr_gain	= 60
Relaxation_delay	= 2[s]
Repetition_time	= 2.83361792[s]
Temp_get	= 22.3[dC]

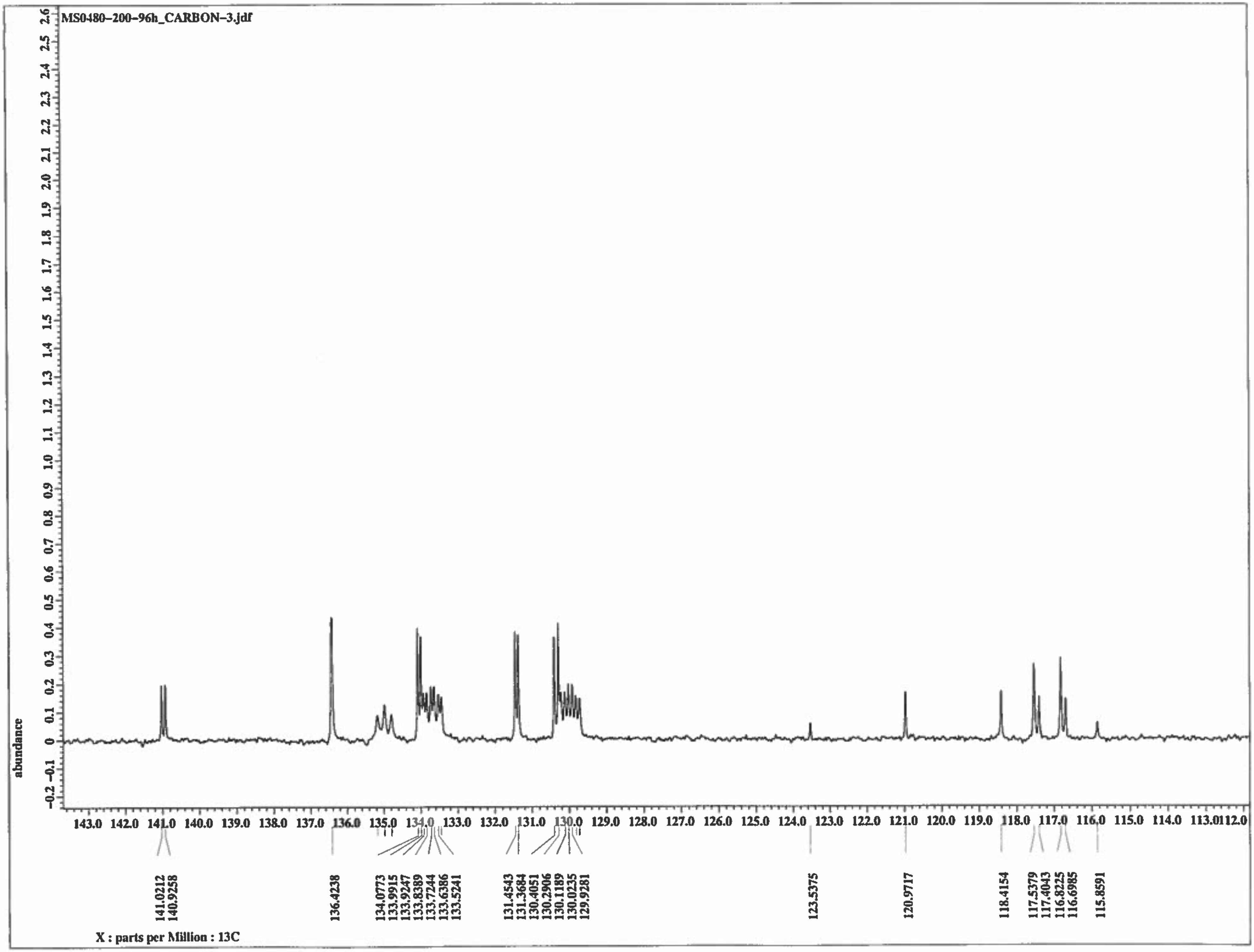
MS0480-200-96h_CARBON-3.jdf
abundance
X : parts per Million : 13C
141.0212
140.9258
136.4238
134.0773
133.9915
133.9247
133.8389
133.7244
133.6386
133.5241
131.4543
131.3684
130.4051
130.2906
130.1189
130.0235
129.9281
123.5375
120.9717
118.4154
117.5379
117.4043
116.8225
116.6985
115.8591

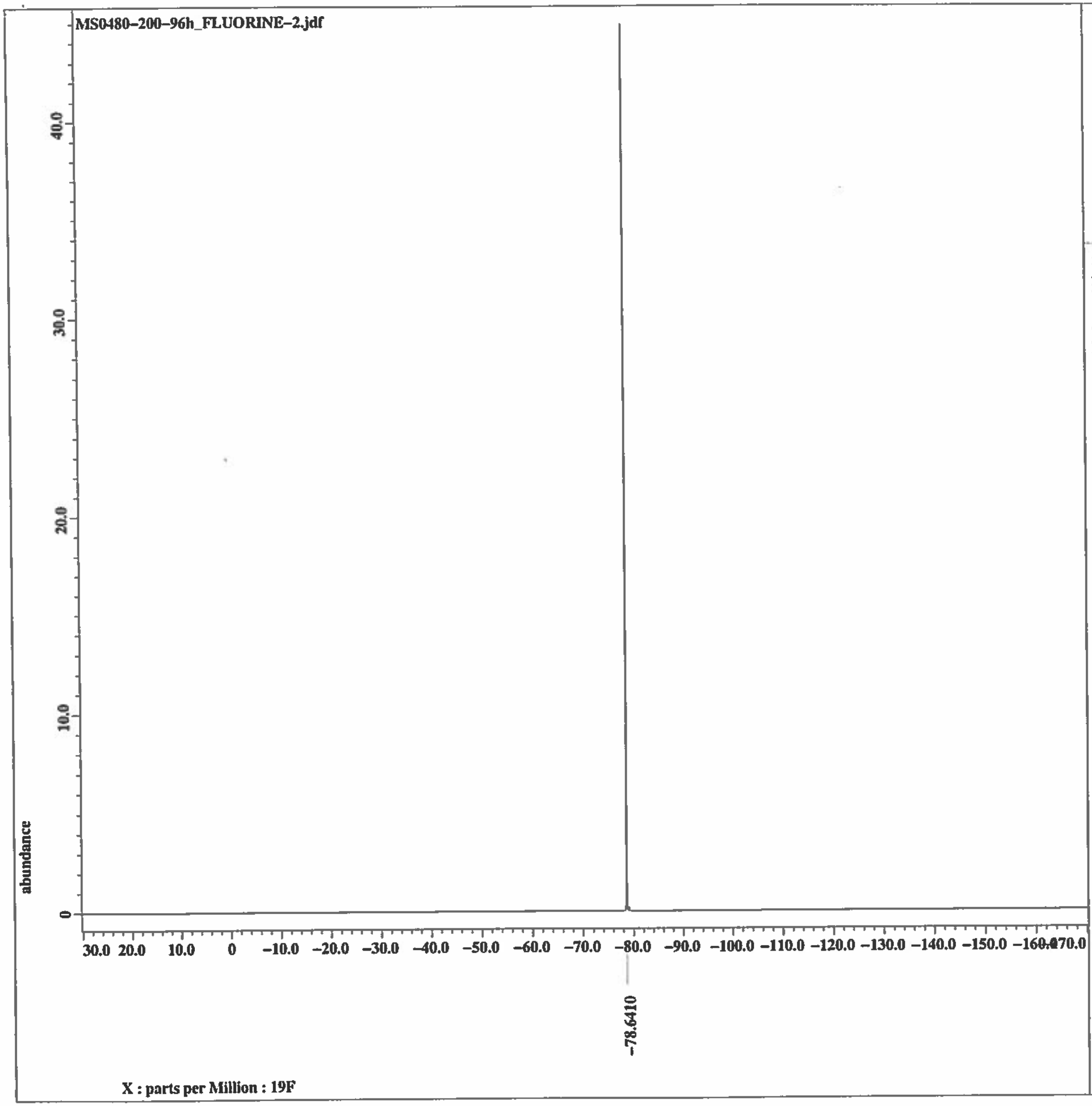

Filename	= MS0480-200-96h_FLUORI
Author	= Jim Davis
Experiment	= single_pulse.ex2
Sample_id	= MS0480-200-96h
Solvent	= CHLOROFORM-D
Changer_sample	= 6
Creation_time	= 27-JUN-2018 08:54:05
Revision_time	= 27-JUN-2018 08:31:12
Current_time	= 27-JUN-2018 08:31:12
Data_format	= 1D COMPLEX
Dim_size	= 52428
Dim_title	= 19F
Dim_units	= [ppm]
Dimensions	= X
Site	= ECA 500
Spectrometer	= JNM-ECA500
Field_strength	= 11.7473579[T] (500[MH
X_acq_duration	= 0.55574528[s]
X_domain	= 19F
X_freq	= 470.62046084[MHz]
X_offset	= -70[ppm]
X_points	= 65536
X_prescans	= 1
X_resolution	= 1.7993855[Hz]
X_sweep	= 117.9245283[kHz]
Irr_domain	= 19F
Irr_freq	= 470.62046084[MHz]
Irr_offset	= 5[ppm]
Tri_domain	= 19F
Tri_freq	= 470.62046084[MHz]
Tri_offset	= 5[ppm]
Clipped	= FALSE
Mod_return	= 1
Scans	= 16
Total_scans	= 16
X_90_width	= 13.1[us]
X_acq_time	= 0.55574528[s]
X_angle	= 45[deg]
X_atn	= 2.5[dB]
X_pulse	= 6.55[us]
Irr_mode	= Off
Tri_mode	= Off
Dante_presat	= FALSE
Initial_wait	= 1[s]
Recvr_gain	= 38
Relaxation_delay	= 4[s]
Repetition_time	= 4.55574528[s]
Temp_get	= 21.9[dC]

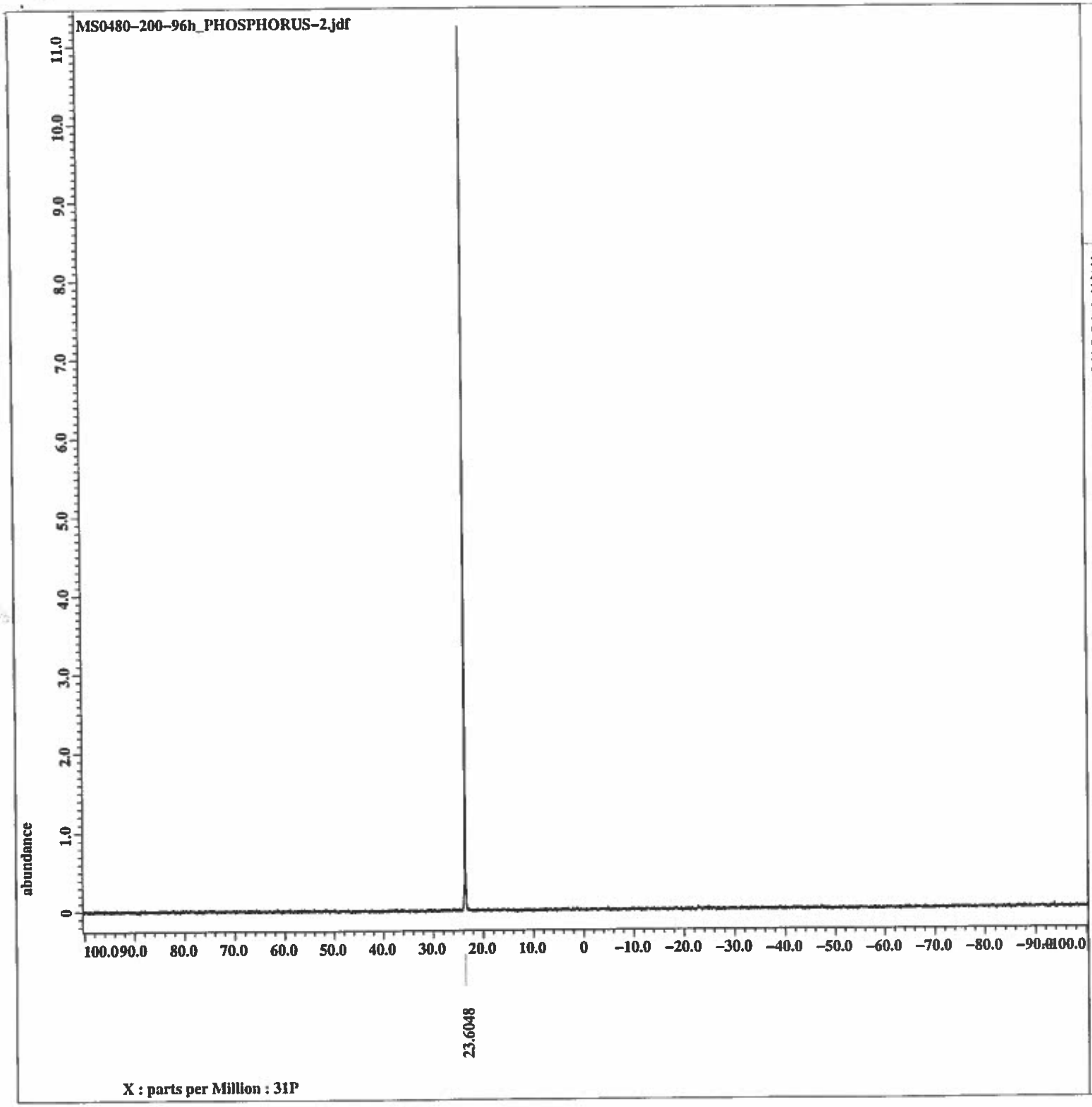

Filename	= MS0480-200-96h_PHOSPH
Author	= Jim Davis
Experiment	= single_pulse_dec
Sample_id	= MS0480-200-96h
Solvent	= CHLOROFORM-D
Changer_sample	= 6
Creation_time	= 27-JUN-2018 08:57:49
Revision_time	= 27-JUN-2018 08:34:56
Current_time	= 27-JUN-2018 08:34:58
Data_format	= 1D COMPLEX
Dim_size	= 26214
Dim_title	= 31P
Dim_units	= [ppm]
Dimensions	= X
Site	= ECA 500
Spectrometer	= JNM-ECA500
Field_strength	= 11.7473579[T] (500[MH
X_acq_duration	= 0.64487424[s]
X_domain	= 31P
X_freq	= 202.46831075[MHz]
X_offset	= 0[ppm]
X_points	= 32768
X_prescans	= 4
X_resolution	= 1.55068995[Hz]
X_sweep	= 50.81300813[kHz]
Irr_domain	= 1H
Irr_freq	= 500.15991521[MHz]
Irr_offset	= 5.0[ppm]
Clipped	= FALSE
Mod_return	= 1
Scans	= 25
Total_scans	= 25
X_90_width	= 14.687[us]
X_acq_time	= 0.64487424[s]
X_angle	= 30[deg]
X_atn	= 5[dB]
X_pulse	= 4.89566667[us]
Irr_atn_dec	= 20.7[dB]
Irr_atn_noe	= 20.7[dB]
Irr_noise	= WALTZ
Decoupling	= TRUE
Initial_wait	= 1[s]
Noe	= TRUE
Noe_time	= 2[s]
Recvr_gain	= 58
Relaxation_delay	= 2[s]
Repetition_time	= 2.64487424[s]
Temp_get	= 22.2[dC]

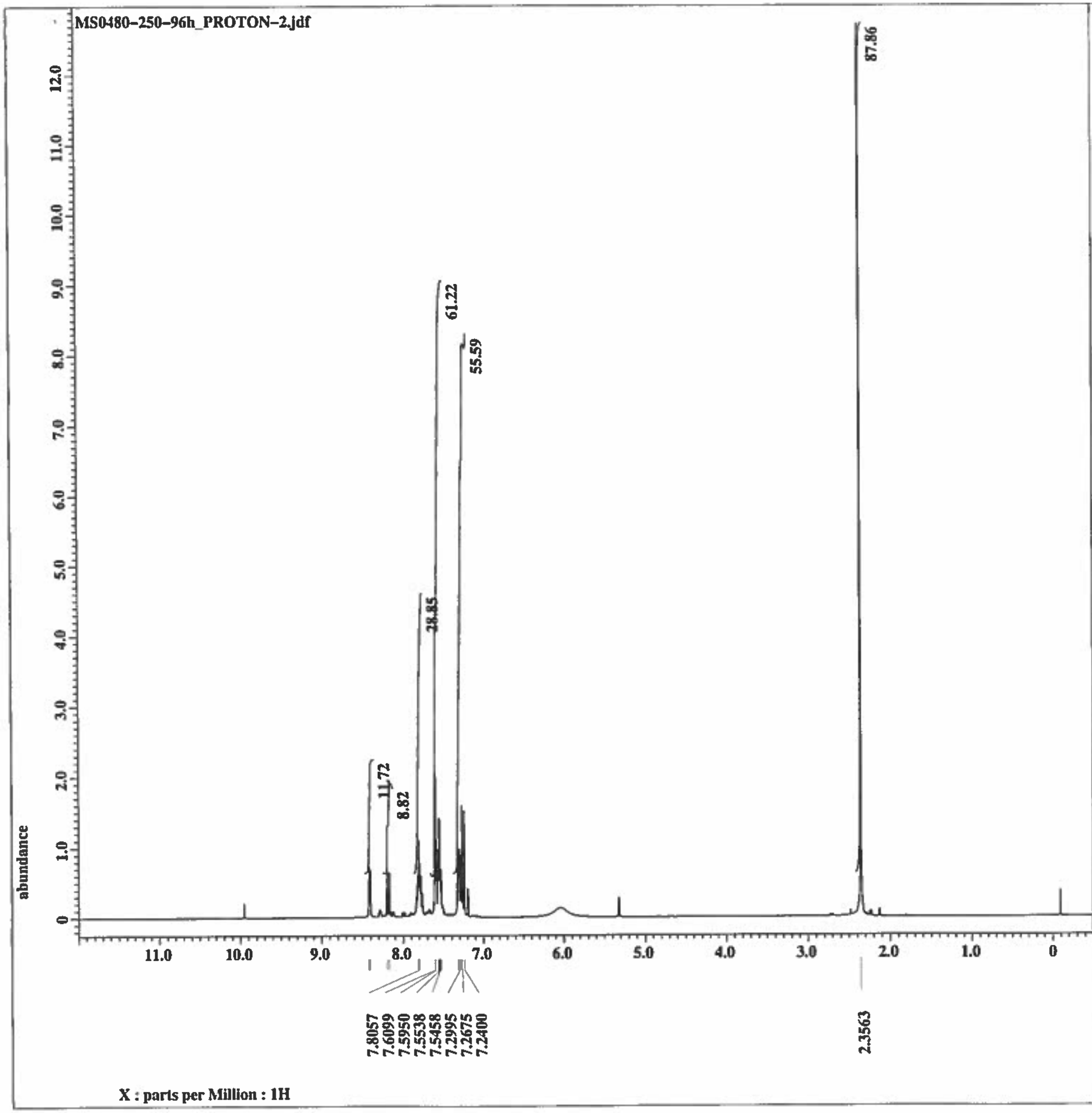

Filename	= MS0480-250-96h_PROTON
Author	= Jim Davis
Experiment	= single_pulse.ex2
Sample_id	= MS0480-250-96h
Solvent	= CHLOROFORM-D
Changer_sample	= 7
Creation_time	= 27-JUN-2018 09:05:00
Revision_time	= 27-JUN-2018 08:42:07
Current_time	= 27-JUN-2018 08:42:07
Data_format	= 1D COMPLEX
Dim_size	= 13107
Dim_title	= 1H
Dim_units	= [ppm]
Dimensions	= X
Site	= ECA 500
Spectrometer	= JNM-ECA500
Field_strength	= 11.7473579[T] (500[MH
X_acq_duration	= 1.74587904[s]
X_domain	= 1H
X_freq	= 500.15991521[MHz]
X_offset	= 5.0[ppm]
X_points	= 16384
X_prescans	= 1
X_resolution	= 0.57277737[Hz]
X_sweep	= 9.38438438[kHz]
Irr_domain	= 1H
Irr_freq	= 500.15991521[MHz]
Irr_offset	= 5.0[ppm]
Tri_domain	= 1H
Tri_freq	= 500.15991521[MHz]
Tri_offset	= 5.0[ppm]
Clipped	= FALSE
Mod_return	= 1
Scans	= 16
Total_scans	= 16
X_90_width	= 12.4[us]
X_acq_time	= 1.74587904[s]
X_angle	= 45[deg]
X_atn	= 4[dB]
X_pulse	= 6.2[us]
Irr_mode	= Off
Tri_mode	= Off
Dante_presat	= FALSE
Initial_wait	= 1[s]
Recvr_gain	= 38
Relaxation_delay	= 4[s]
Repetition_time	= 5.74587904[s]
Temp_get	= 21.9[dC]

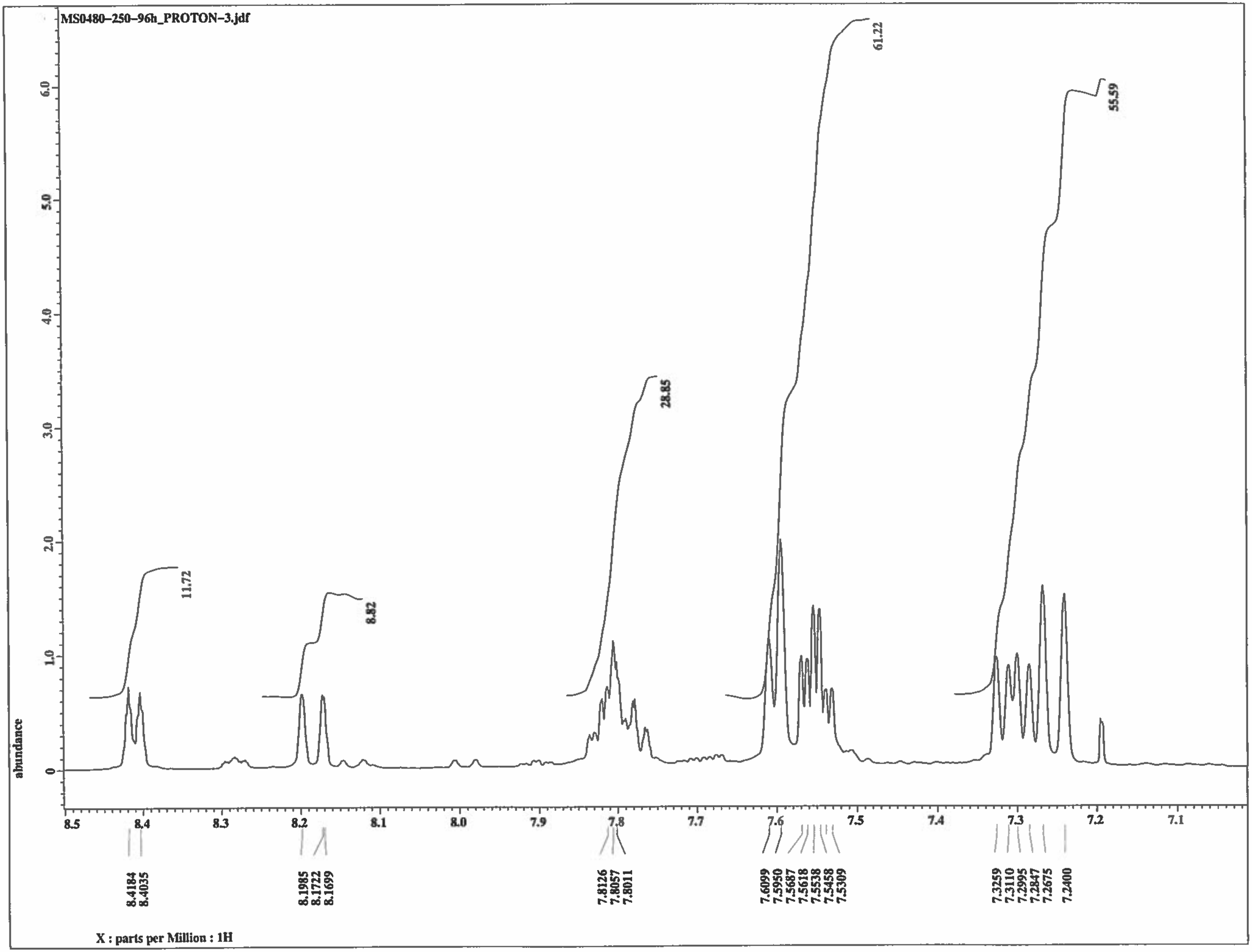

MS0480-250-96h_PROTON-3.jdf
abundance
0
1.0
2.0
3.0
4.0
5.0
6.0
11.72
8.82
28.85
61.22
55.59
8.5
8.4
8.3
8.2
8.1
8.0
7.9
7.8
7.7
7.6
7.5
7.4
7.3
7.2
7.1
8.4184
8.4035
8.1985
8.1722
8.1699
7.8126
7.8057
7.8011
7.6099
7.5950
7.5687
7.5618
7.5538
7.5458
7.5309
7.3259
7.3110
7.2995
7.2847
7.2675
7.2400
X : parts per Million : 1H

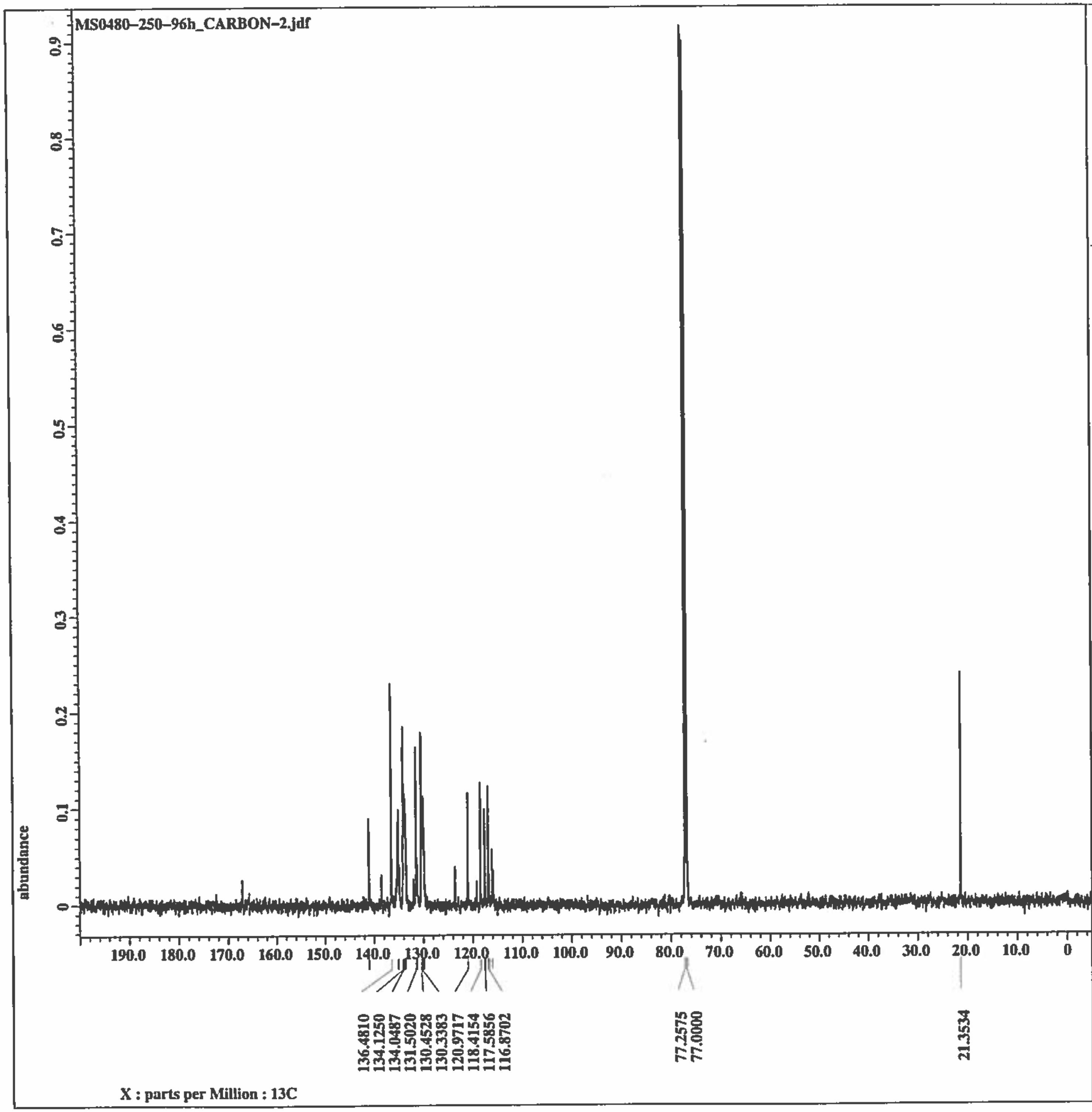

Filename	= MS0480-250-96h_CARBON
Author	= Jim Davis
Experiment	= single_pulse_dec
Sample_id	= MS0480-250-96h
Solvent	= CHLOROFORM-D
Changer_sample	= 7
Creation_time	= 27-JUN-2018 09:21:34
Revision_time	= 27-JUN-2018 08:58:41
Current_time	= 27-JUN-2018 08:58:41
Data_format	= 1D COMPLEX
Dim_size	= 26214
Dim_title	= 13C
Dim_units	= [ppm]
Dimensions	= X
Site	= ECA 500
Spectrometer	= JNM-ECA500
Field_strength	= 11.7473579[T] (500[MH
X_acq_duration	= 0.83361792[s]
X_domain	= 13C
X_freq	= 125.76529768[MHz]
X_offset	= 100[ppm]
X_points	= 32768
X_prescans	= 4
X_resolution	= 1.19959034[Hz]
X_sweep	= 39.3081761[kHz]
Irr_domain	= 1H
Irr_freq	= 500.15991521[MHz]
Irr_offset	= 5.0[ppm]
Clipped	= FALSE
Mod_return	= 1
Scans	= 300
Total_scans	= 300
X_90_width	= 13.2[us]
X_acq_time	= 0.83361792[s]
X_angle	= 30[deg]
X_atn	= 6[dB]
X_pulse	= 4.4[us]
Irr_atn_dec	= 20.7[dB]
Irr_atn_noe	= 20.7[dB]
Irr_noise	= WALTZ
Decoupling	= TRUE
Initial_wait	= 1[s]
Noe	= TRUE
Noe_time	= 2[s]
Recvr_gain	= 60
Relaxation_delay	= 2[s]
Repetition_time	= 2.83361792[s]
Temp_get	= 22.6[dC]

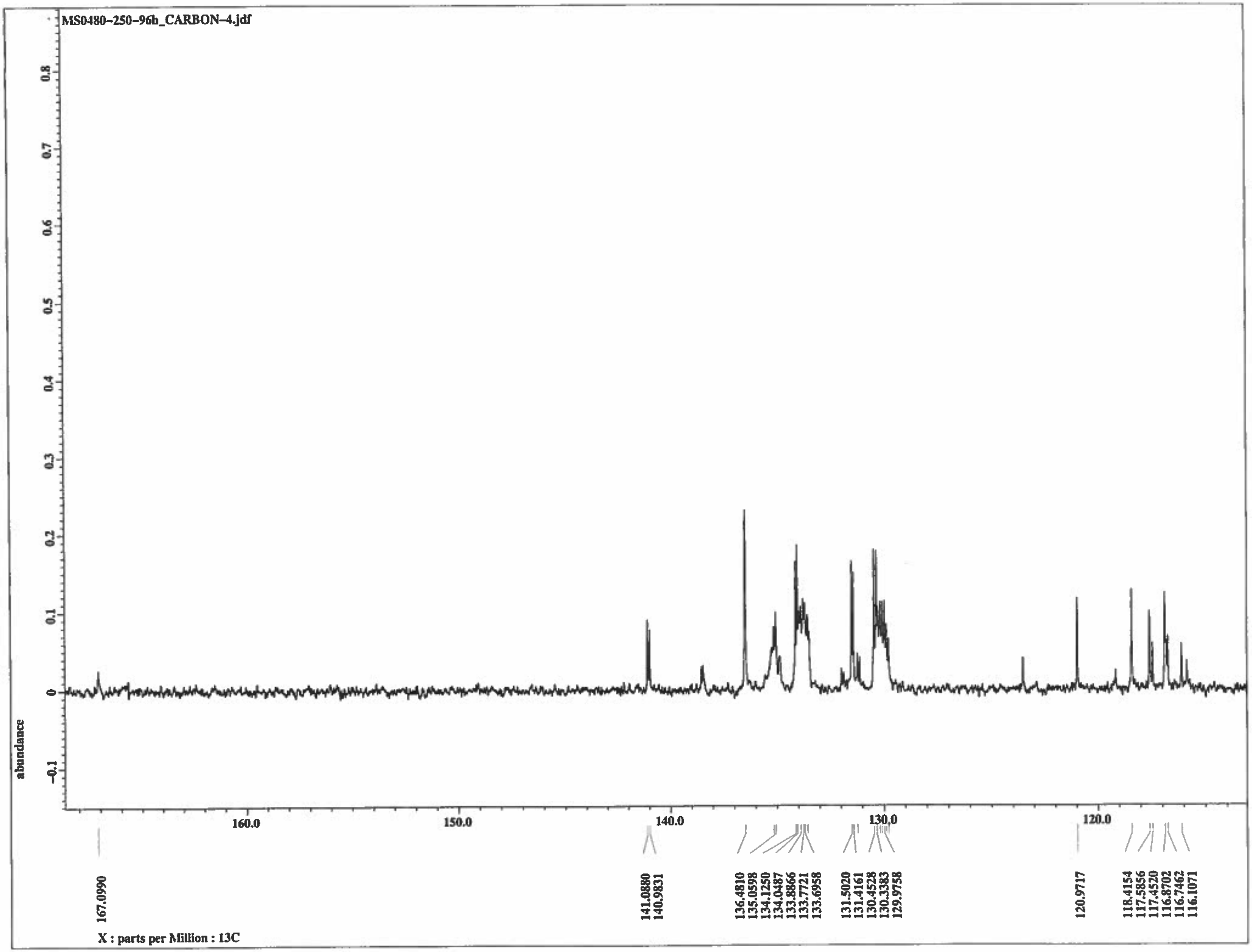
MS0480-250-96b_CARBON-4.jdf
abundance
0.8
0.7
0.6
0.5
0.4
0.3
0.2
0.1
0
-0.1
160.0
150.0
140.0
130.0
120.0
167.0990
141.0880
140.9831
136.4810
135.0598
134.1250
134.0487
133.8866
133.7721
133.6958
131.5020
131.4161
130.4528
130.3383
129.9758
120.9717
118.4154
117.5856
117.4520
116.8702
116.7462
116.1071
X : parts per Million : 13C

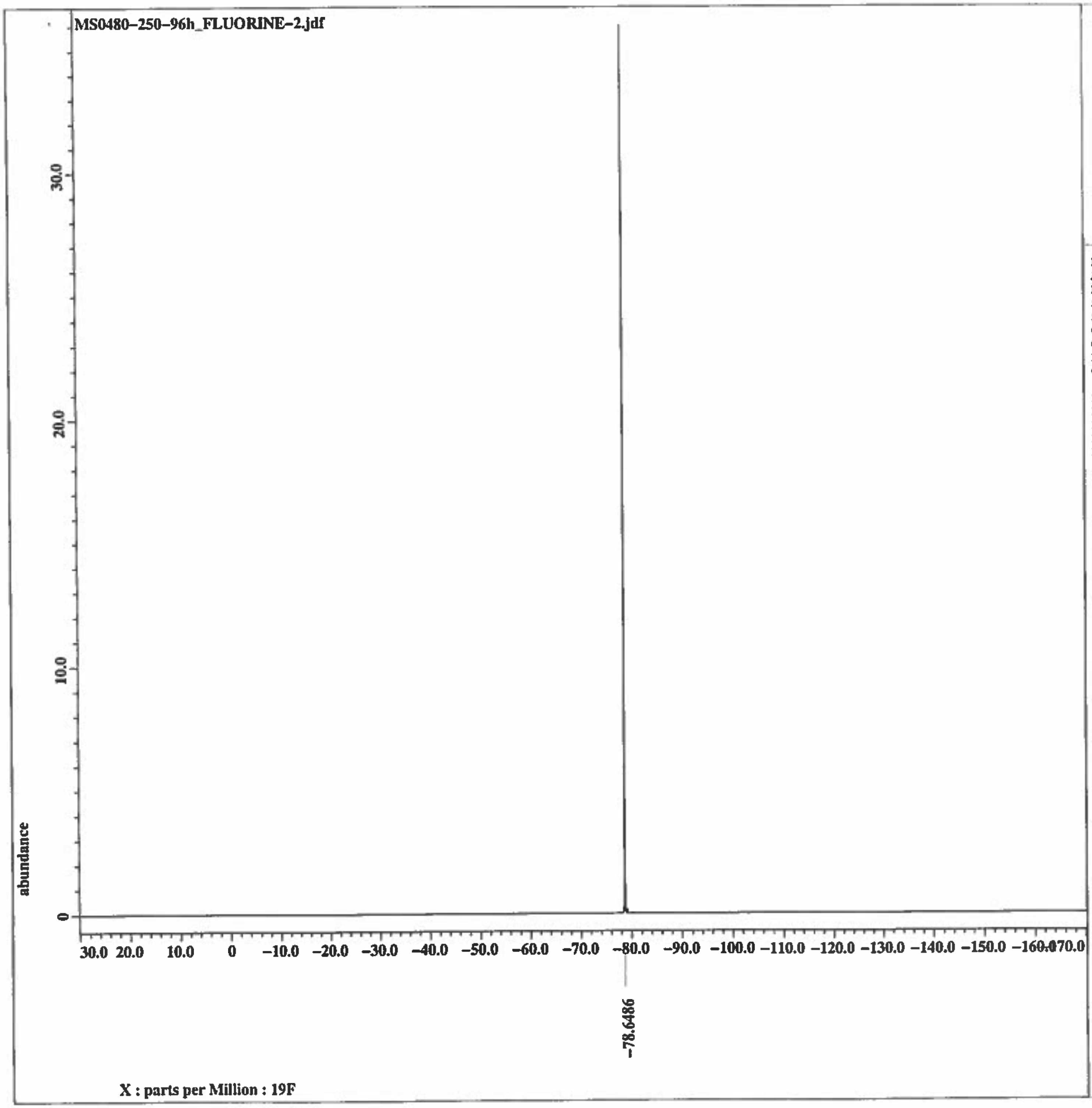

Filename	= MS0480-250-96h_FLUORI
Author	= Jim Davis
Experiment	= single_pulse.ex2
Sample_id	= MS0480-250-96h
Solvent	= CHLOROFORM-D
Changer_sample	= 7
Creation_time	= 27-JUN-2018 09:24:29
Revision_time	= 27-JUN-2018 09:01:37
Current_time	= 27-JUN-2018 09:01:38
Data_format	= 1D COMPLEX
Dim_size	= 52428
Dim_title	= 19F
Dim_units	= [ppm]
Dimensions	= X
Site	= ECA 500
Spectrometer	= JNM-ECA500
Field_strength	= 11.7473579[T] (500[MH
X_acq_duration	= 0.55574528[s]
X_domain	= 19F
X_freq	= 470.62046084[MHz]
X_offset	= -70[ppm]
X_points	= 65536
X_prescans	= 1
X_resolution	= 1.7993855[Hz]
X_sweep	= 117.9245283[kHz]
Irr_domain	= 19F
Irr_freq	= 470.62046084[MHz]
Irr_offset	= 5[ppm]
Tri_domain	= 19F
Tri_freq	= 470.62046084[MHz]
Tri_offset	= 5[ppm]
Clipped	= FALSE
Mod_return	= 1
Scans	= 16
Total_scans	= 16
X_90_width	= 13.1[us]
X_acq_time	= 0.55574528[s]
X_angle	= 45[deg]
X_atn	= 2.5[dB]
X_pulse	= 6.55[us]
Irr_mode	= Off
Tri_mode	= Off
Dante_presat	= FALSE
Initial_wait	= 1[s]
Recvr_gain	= 38
Relaxation_delay	= 4[s]
Repetition_time	= 4.55574528[s]
Temp_get	= 22.2[dC]

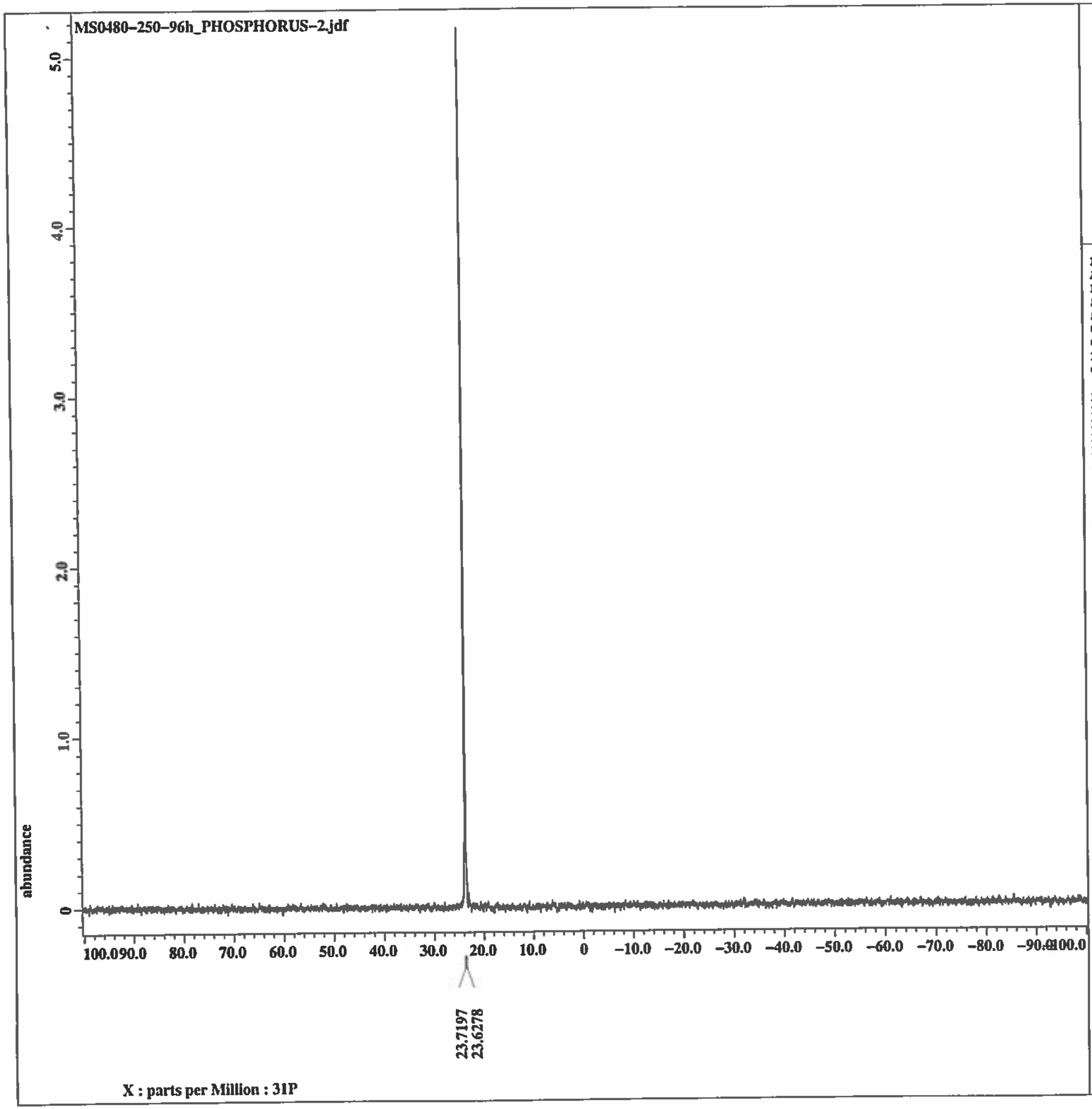

Filename	= MS0480-250-96h_PHOSPH
Author	= Jim Davis
Experiment	= single_pulse_dec
Sample_id	= MS0480-250-96h
Solvent	= CHLOROFORM-D
Changer_sample	= 7
Creation_time	= 27-JUN-2018 09:28:13
Revision_time	= 27-JUN-2018 09:05:21
Current_time	= 27-JUN-2018 09:05:21
Data_format	= 1D COMPLEX
Dim_size	= 26214
Dim_title	= 31P
Dim_units	= [ppm]
Dimensions	= X
Site	= ECA 500
Spectrometer	= JNM-ECA500
Field_strength	= 11.7473579[T] (500[MH
X_acq_duration	= 0.64487424[s]
X_domain	= 31P
X_freq	= 202.46831075[MHz]
X_offset	= 0[ppm]
X_points	= 32768
X_prescans	= 4
X_resolution	= 1.55068995[Hz]
X_sweep	= 50.81300813[kHz]
Irr_domain	= 1H
Irr_freq	= 500.15991521[MHz]
Irr_offset	= 5.0[ppm]
Clipped	= FALSE
Mod_return	= 1
Scans	= 25
Total_scans	= 25
X_90_width	= 14.687[us]
X_acq_time	= 0.64487424[s]
X_angle	= 30[deg]
X_atn	= 5[dB]
X_pulse	= 4.89566667[us]
Irr_atn_dec	= 20.7[dB]
Irr_atn_noe	= 20.7[dB]
Irr_noise	= WALTZ
Decoupling	= TRUE
Initial_wait	= 1[s]
Noe	= TRUE
Noe_time	= 2[s]
Recvr_gain	= 58
Relaxation_delay	= 2[s]
Repetition_time	= 2.64487424[s]
Temp_get	= 22.4[dC]

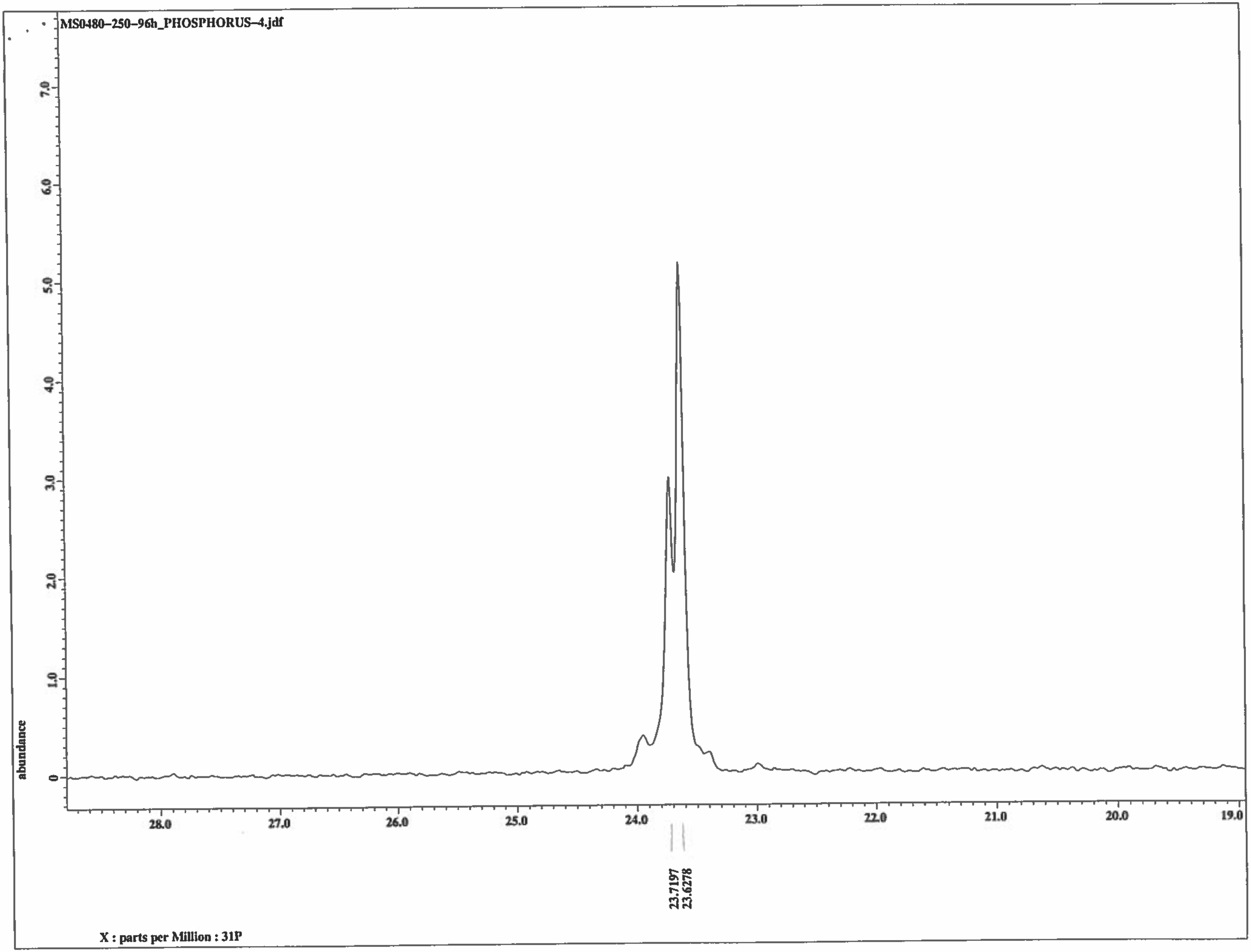
MS0480-250-96h_PHOSPHORUS-4.jdf
abundance
0
1.0
2.0
3.0
4.0
5.0
6.0
7.0
28.0
27.0
26.0
25.0
24.0
23.0
22.0
21.0
20.0
19.0
23.7197
23.6278
X : parts per Million : 31P

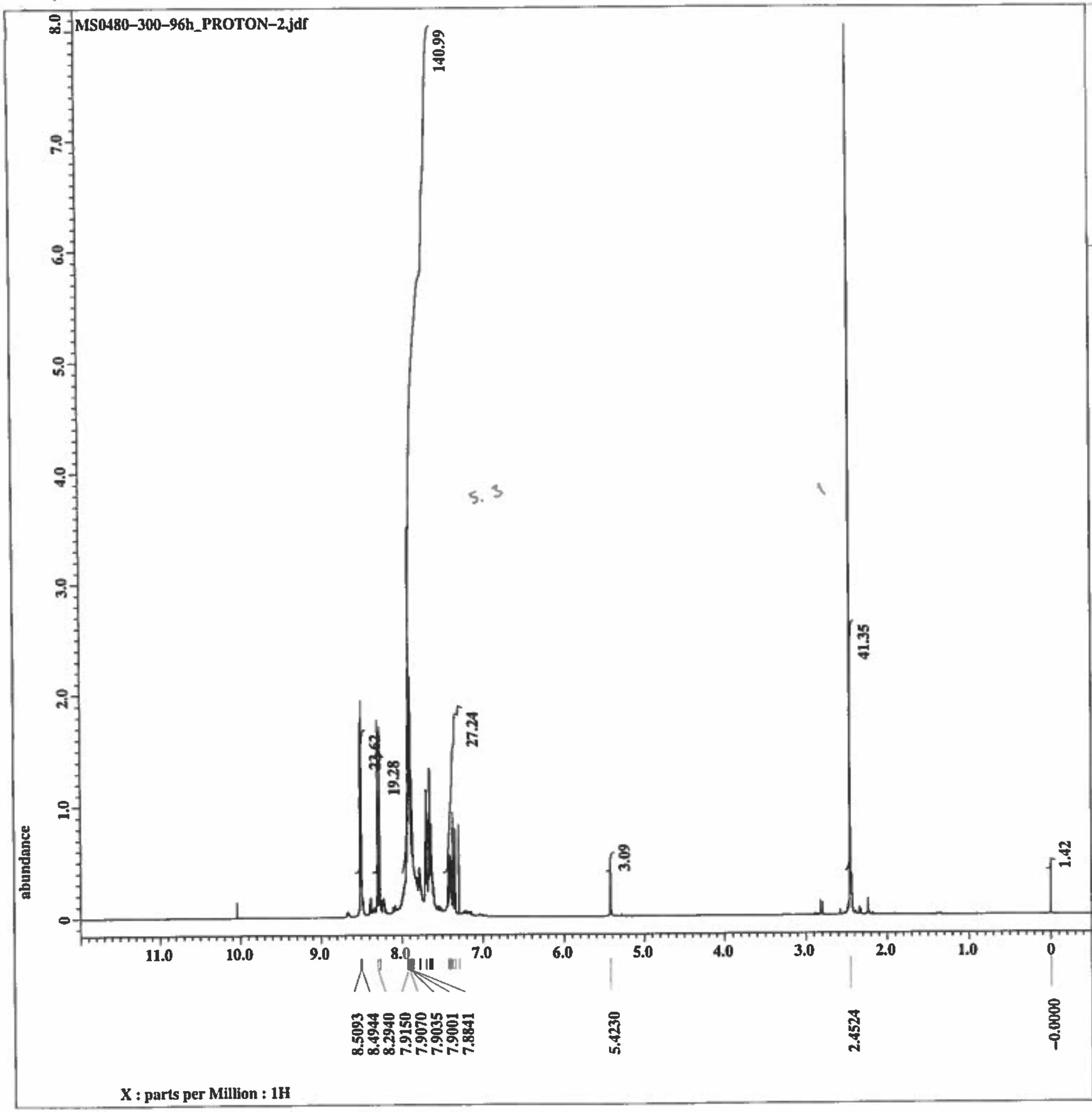

Filename	= MS0480-300-96h_PROTON
Author	= Jim Davis
Experiment	= single_pulse.ex2
Sample_id	= MS0480-300-96h
Solvent	= CHLOROFORM-D
Changer_sample	= 8
Creation_time	= 27-JUN-2018 09:35:10
Revision_time	= 27-JUN-2018 09:12:17
Current_time	= 27-JUN-2018 09:12:17
Data_format	= 1D COMPLEX
Dim_size	= 13107
Dim_title	= 1H
Dim_units	= [ppm]
Dimensions	= X
Site	= ECA 500
Spectrometer	= JNM-ECA500
Field_strength	= 11.7473579[T] (500[MH
X_acq_duration	= 1.74587904[s]
X_domain	= 1H
X_freq	= 500.15991521[MHz]
X_offset	= 5.0[ppm]
X_points	= 16384
X_prescans	= 1
X_resolution	= 0.57277737[Hz]
X_sweep	= 9.38438438[kHz]
Irr_domain	= 1H
Irr_freq	= 500.15991521[MHz]
Irr_offset	= 5.0[ppm]
Tri_domain	= 1H
Tri_freq	= 500.15991521[MHz]
Tri_offset	= 5.0[ppm]
Clipped	= FALSE
Mod_return	= 1
Scans	= 16
Total_scans	= 16
X_90_width	= 12.4[us]
X_acq_time	= 1.74587904[s]
X_angle	= 45[deg]
X_atn	= 4[dB]
X_pulse	= 6.2[us]
Irr_mode	= Off
Tri_mode	= Off
Dante_presat	= FALSE
Initial_wait	= 1[s]
Recvr_gain	= 36
Relaxation_delay	= 4[s]
Repetition_time	= 5.74587904[s]
Temp_get	= 22.1[dC]

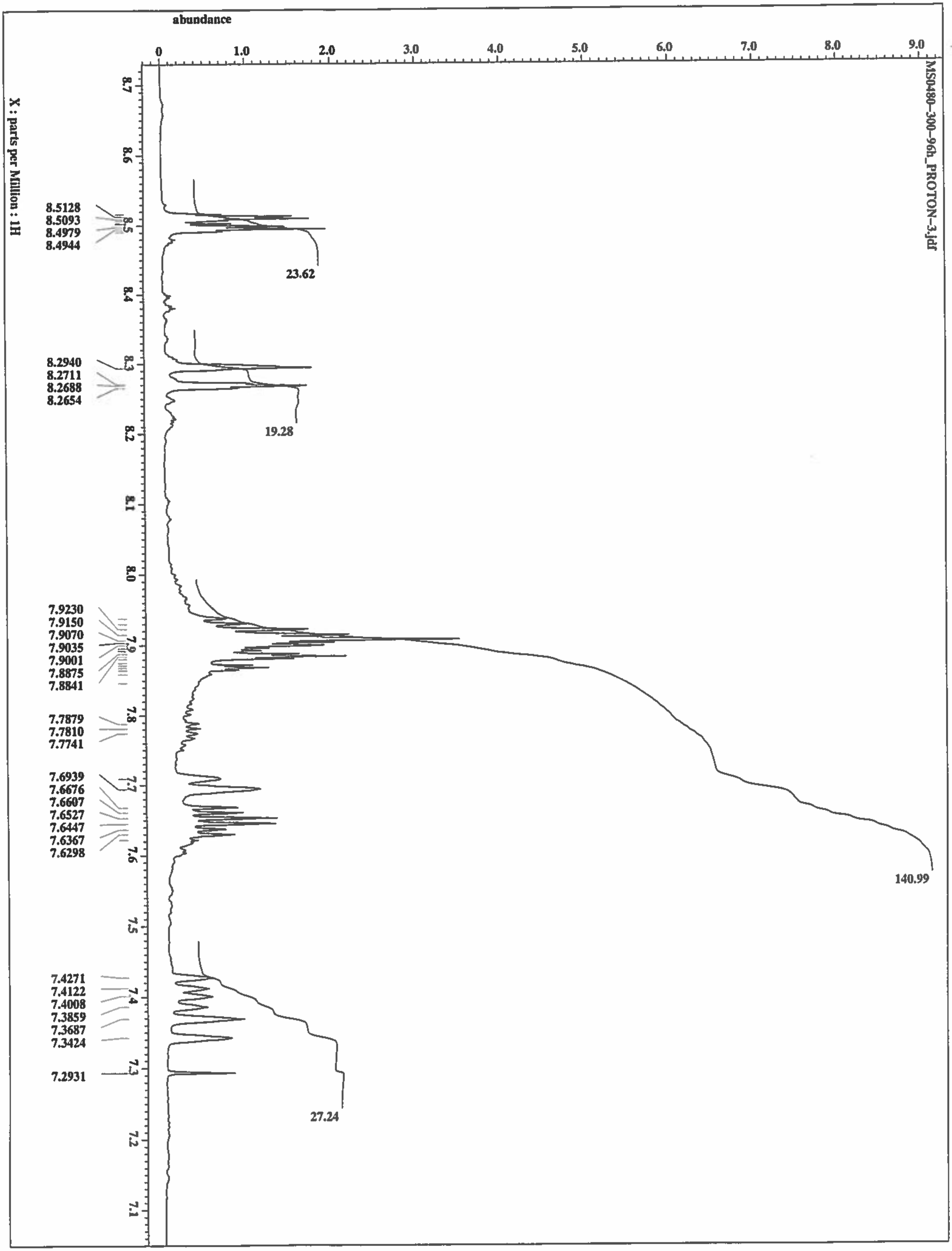
MS0480-300-96h_PROTON-3.jdf
abundance
0
1.0
2.0
3.0
4.0
5.0
6.0
7.0
8.0
9.0
X : parts per Million : 1H
8.7
8.6
8.5
8.4
8.3
8.2
8.1
8.0
7.9
7.8
7.7
7.6
7.5
7.4
7.3
7.2
7.1
8.5128
8.5093
8.4979
8.4944
23.62
8.2940
8.2711
8.2688
8.2654
19.28
7.9230
7.9150
7.9070
7.9035
7.9001
7.8875
7.8841
7.7879
7.7810
7.7741
7.6939
7.6676
7.6607
7.6527
7.6447
7.6367
7.6298
140.99
7.4271
7.4122
7.4008
7.3859
7.3687
7.3424
7.2931
27.24

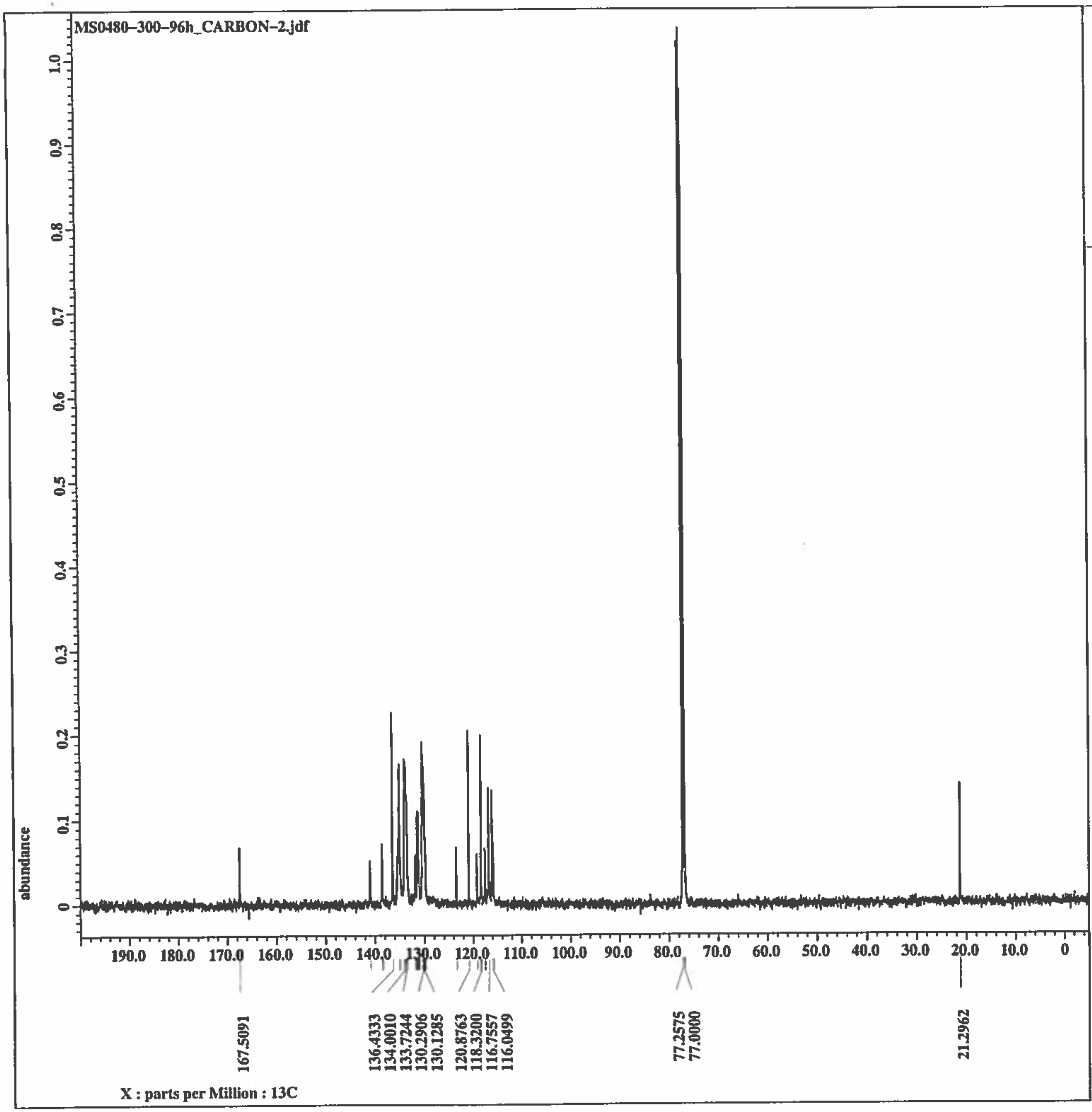

Filename	= MS0480-300-96h_CARBON
Author	= Jim Davis
Experiment	= single_pulse_dec
Sample_id	= MS0480-300-96h
Solvent	= CHLOROFORM-D
Changer_sample	= 8
Creation_time	= 27-JUN-2018 09:56:43
Revision_time	= 27-JUN-2018 09:33:51
Current_time	= 27-JUN-2018 09:33:51
Data_format	= 1D COMPLEX
Dim_size	= 26214
Dim_title	= 13C
Dim_units	= [ppm]
Dimensions	= X
Site	= ECA 500
Spectrometer	= JNM-ECA500
Field_strength	= 11.7473579[T] (500[MH
X_acq_duration	= 0.83361792[s]
X_domain	= 13C
X_freq	= 125.76529768[MHz]
X_offset	= 100[ppm]
X_points	= 32768
X_prescans	= 4
X_resolution	= 1.19959034[Hz]
X_sweep	= 39.3081761[kHz]
Irr_domain	= 1H
Irr_freq	= 500.15991521[MHz]
Irr_offset	= 5.0[ppm]
Clipped	= FALSE
Mod_return	= 1
Scans	= 400
Total_scans	= 400
X_90_width	= 13.2[us]
X_acq_time	= 0.83361792[s]
X_angle	= 30[deg]
X_atn	= 6[dB]
X_pulse	= 4.4[us]
Irr_atn_dec	= 20.7[dB]
Irr_atn_noe	= 20.7[dB]
Irr_noise	= WALTZ
Decoupling	= TRUE
Initial_wait	= 1[s]
Noe	= TRUE
Noe_time	= 2[s]
Recvr_gain	= 60
Relaxation_delay	= 2[s]
Repetition_time	= 2.83361792[s]
Temp_get	= 22.8[dC]

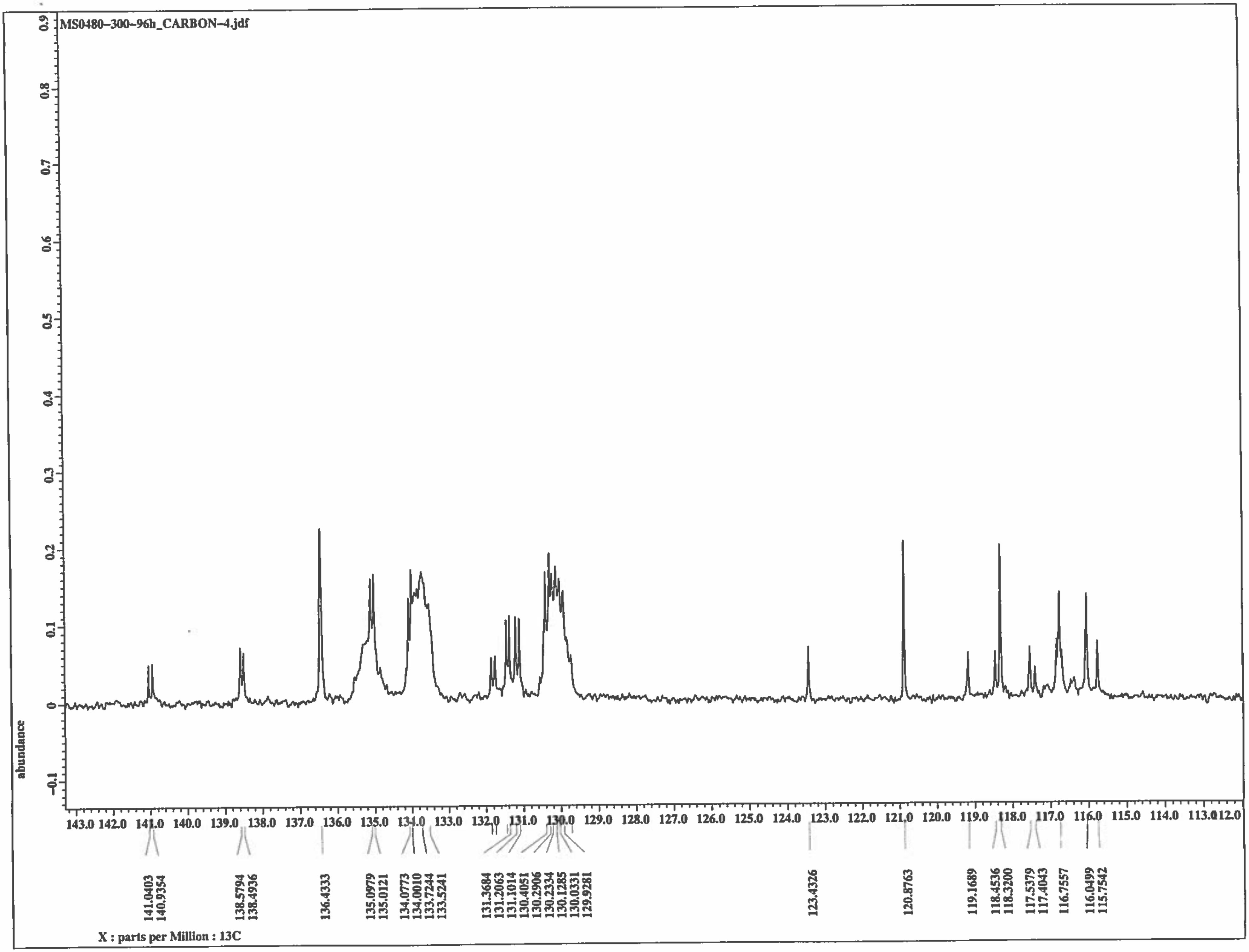
MS0480-300-96h_CARBON-4.jdf
abundance
0.9
0.8
0.7
0.6
0.5
0.4
0.3
0.2
0.1
0
-0.1
143.0 142.0 141.0 140.0 139.0 138.0 137.0 136.0 135.0 134.0 133.0 132.0 131.0 130.0 129.0 128.0 127.0 126.0 125.0 124.0 123.0 122.0 121.0 120.0 119.0 118.0 117.0 116.0 115.0 114.0 113.0 112.0
141.0403
140.9354
138.5794
138.4936
136.4333
135.0979
135.0121
134.0773
134.0010
133.7244
133.5241
131.3684
131.2063
131.1014
130.4051
130.2906
130.2334
130.1285
130.0331
129.9281
123.4326
120.8763
119.1689
118.4536
118.3200
117.5379
117.4043
116.7557
116.0499
115.7542
X : parts per Million : 13C

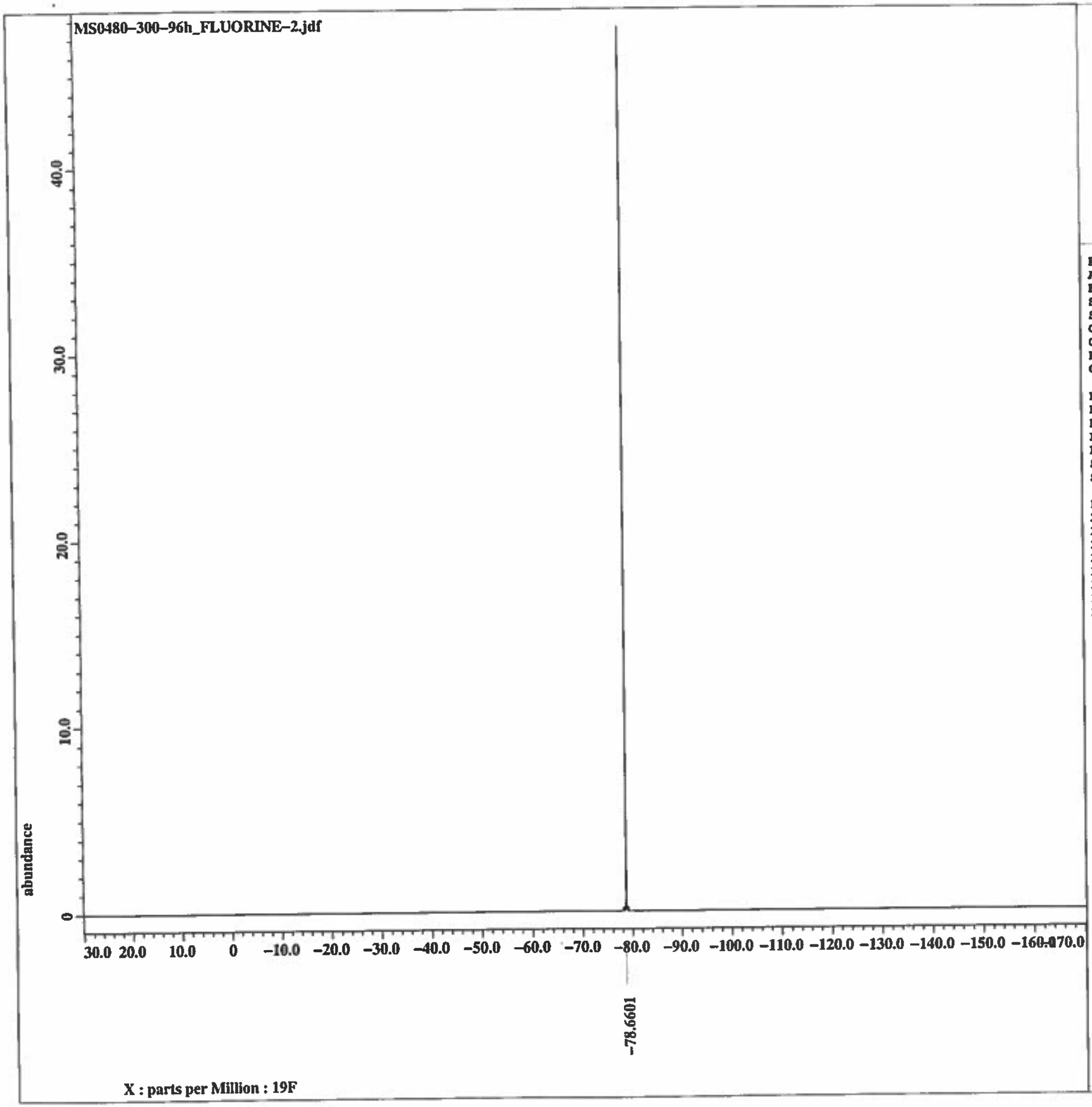

Filename	= MS0480-300-96h_FLUORI
Author	= Jim Davis
Experiment	= single_pulse.ex2
Sample_id	= MS0480-300-96h
Solvent	= CHLOROFORM-D
Changer_sample	= 8
Creation_time	= 27-JUN-2018 09:59:49
Revision_time	= 27-JUN-2018 09:36:57
Current_time	= 27-JUN-2018 09:36:57
Data_format	= 1D COMPLEX
Dim_size	= 52428
Dim_title	= 19F
Dim_units	= [ppm]
Dimensions	= X
Site	= ECA 500
Spectrometer	= JNM-ECA500
Field_strength	= 11.7473579[T] (500[MH
X_acq_duration	= 0.55574528[s]
X_domain	= 19F
X_freq	= 470.62046084[MHz]
X_offset	= -70[ppm]
X_points	= 65536
X_prescans	= 1
X_resolution	= 1.7993855[Hz]
X_sweep	= 117.9245283[kHz]
Irr_domain	= 19F
Irr_freq	= 470.62046084[MHz]
Irr_offset	= 5[ppm]
Tri_domain	= 19F
Tri_freq	= 470.62046084[MHz]
Tri_offset	= 5[ppm]
Clipped	= FALSE
Mod_return	= 1
Scans	= 16
Total_scans	= 16
X_90_width	= 13.1[us]
X_acq_time	= 0.55574528[s]
X_angle	= 45[deg]
X_atn	= 2.5[dB]
X_pulse	= 6.55[us]
Irr_mode	= Off
Tri_mode	= Off
Dante_presat	= FALSE
Initial_wait	= 1[s]
Recvr_gain	= 36
Relaxation_delay	= 4[s]
Repetition_time	= 4.55574528[s]
Temp_get	= 22.4[dC]

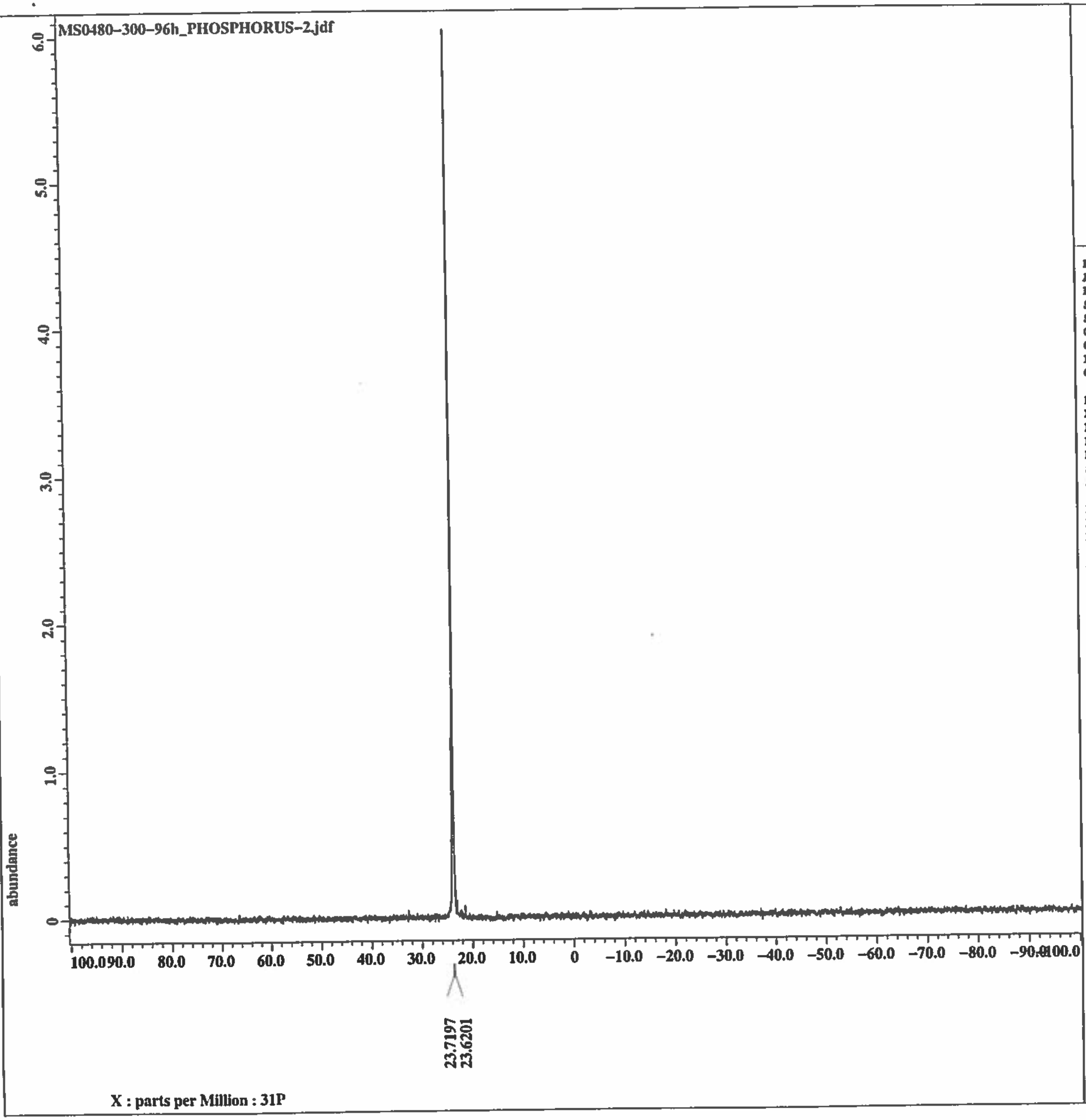

Filename	= MS0480-300-96h_PHOSPH
Author	= Jim Davis
Experiment	= single_pulse_dec
Sample_id	= MS0480-300-96h
Solvent	= CHLOROFORM-D
Changer_sample	= 8
Creation_time	= 27-JUN-2018 10:03:29
Revision_time	= 27-JUN-2018 09:40:36
Current_time	= 27-JUN-2018 09:40:36
Data_format	= 1D COMPLEX
Dim_size	= 26214
Dim_title	= 31P
Dim_units	= [ppm]
Dimensions	= X
Site	= ECA 500
Spectrometer	= JNM-ECA500
Field_strength	= 11.7473579[T] (500[MH
X_acq_duration	= 0.64487424[s]
X_domain	= 31P
X_freq	= 202.46831075[MHz]
X_offset	= 0[ppm]
X_points	= 32768
X_prescans	= 4
X_resolution	= 1.55068995[Hz]
X_sweep	= 50.81300813[kHz]
Irr_domain	= 1H
Irr_freq	= 500.15991521[MHz]
Irr_offset	= 5.0[ppm]
Clipped	= FALSE
Mod_return	= 1
Scans	= 25
Total_scans	= 25
X_90_width	= 14.687[us]
X_acq_time	= 0.64487424[s]
X_angle	= 30[deg]
X_atn	= 5[dB]
X_pulse	= 4.89566667[us]
Irr_atn_dec	= 20.7[dB]
Irr_atn_noe	= 20.7[dB]
Irr_noise	= WALTZ
Decoupling	= TRUE
Initial_wait	= 1[s]
Noe	= TRUE
Noe_time	= 2[s]
Recvr_gain	= 58
Relaxation_delay	= 2[s]
Repetition_time	= 2.64487424[s]
Temp_get	= 22.6[dC]

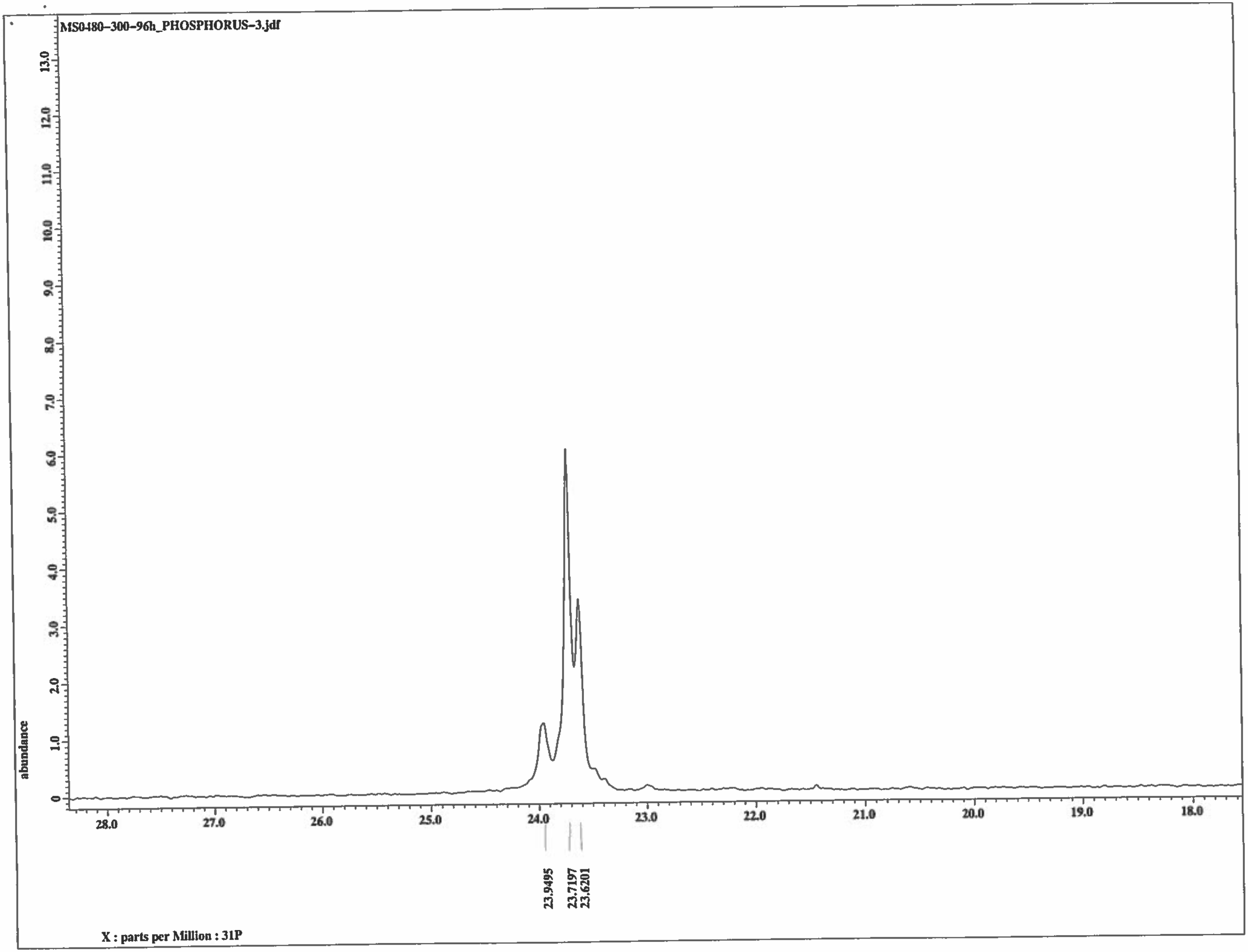

MS0480-300-96h_PHOSPHORUS-3.jdf
abundance
0
1.0
2.0
3.0
4.0
5.0
6.0
7.0
8.0
9.0
10.0
11.0
12.0
13.0
28.0
27.0
26.0
25.0
24.0
23.0
22.0
21.0
20.0
19.0
18.0
23.9495
23.7197
23.6201
X : parts per Million : 31P

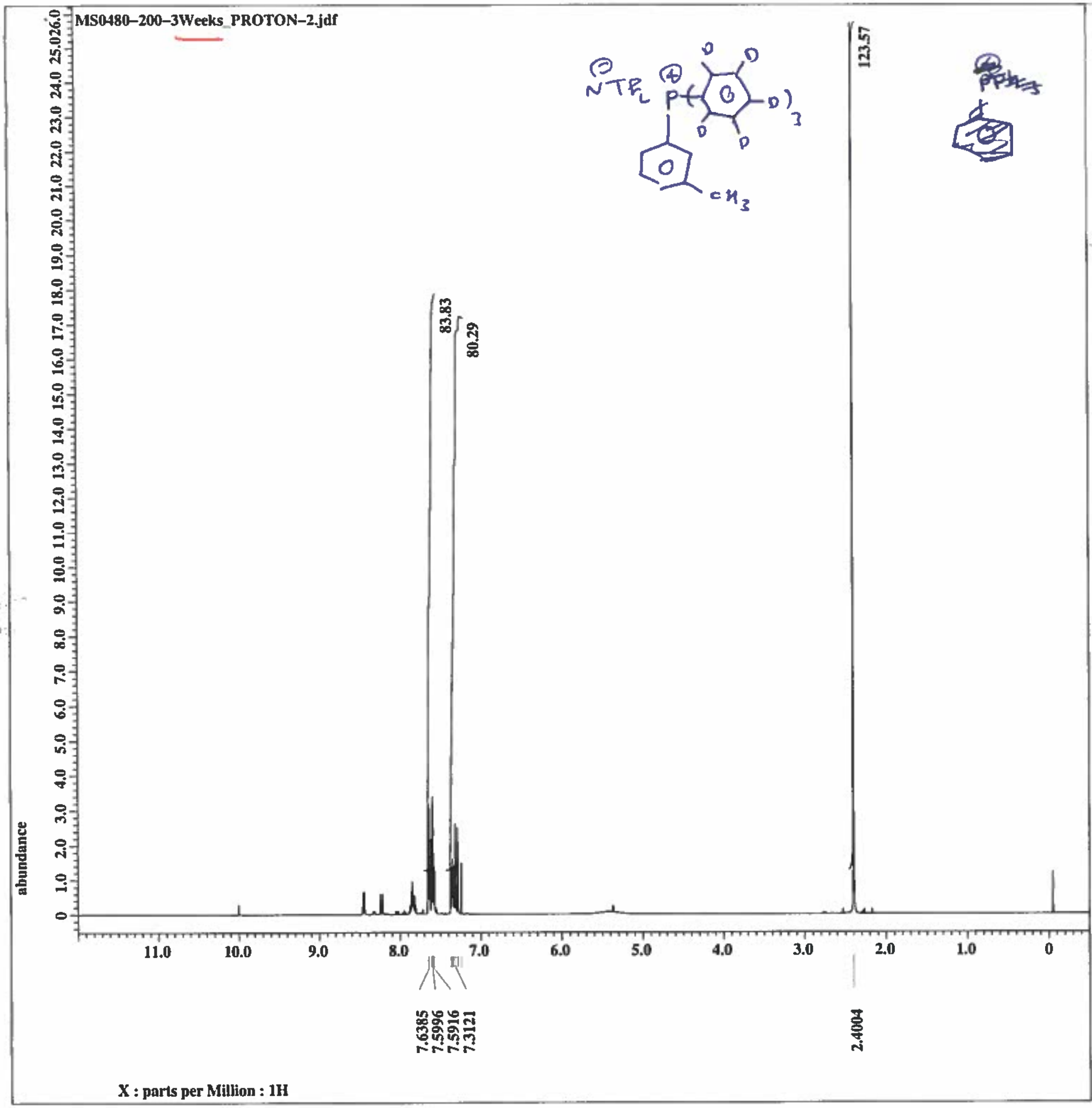

Filename	= MS0480-200-3Weeks_PRO
Author	= Jim Davis
Experiment	= single_pulse.ex2
Sample_id	= MS0480-200-3Weeks
Solvent	= CHLOROFORM-D
Changer_sample	= 21
Creation_time	= 13-JUL-2018 10:20:02
Revision_time	= 13-JUL-2018 09:57:35
Current_time	= 13-JUL-2018 09:57:35
Data_format	= 1D COMPLEX
Dim_size	= 13107
Dim_title	= 1H
Dim_units	= [ppm]
Dimensions	= X
Site	= ECA 500
Spectrometer	= JNM-ECA500
Field_strength	= 11.7473579[T] (500[MH
X_acq_duration	= 1.74587904[s]
X_domain	= 1H
X_freq	= 500.15991521[MHz]
X_offset	= 5.0[ppm]
X_points	= 16384
X_prescans	= 1
X_resolution	= 0.57277737[Hz]
X_sweep	= 9.38438438[kHz]
Irr_domain	= 1H
Irr_freq	= 500.15991521[MHz]
Irr_offset	= 5.0[ppm]
Tri_domain	= 1H
Tri_freq	= 500.15991521[MHz]
Tri_offset	= 5.0[ppm]
Clipped	= FALSE
Mod_return	= 1
Scans	= 16
Total_scans	= 16
X_90_width	= 12.4[us]
X_acq_time	= 1.74587904[s]
X_angle	= 45[deg]
X_atn	= 4[dB]
X_pulse	= 6.2[us]
Irr_mode	= Off
Tri_mode	= Off
Dante_presat	= FALSE
Initial_wait	= 1[s]
Recvr_gain	= 38
Relaxation_delay	= 4[s]
Repetition_time	= 5.74587904[s]
Temp_get	= 22.7[dC]

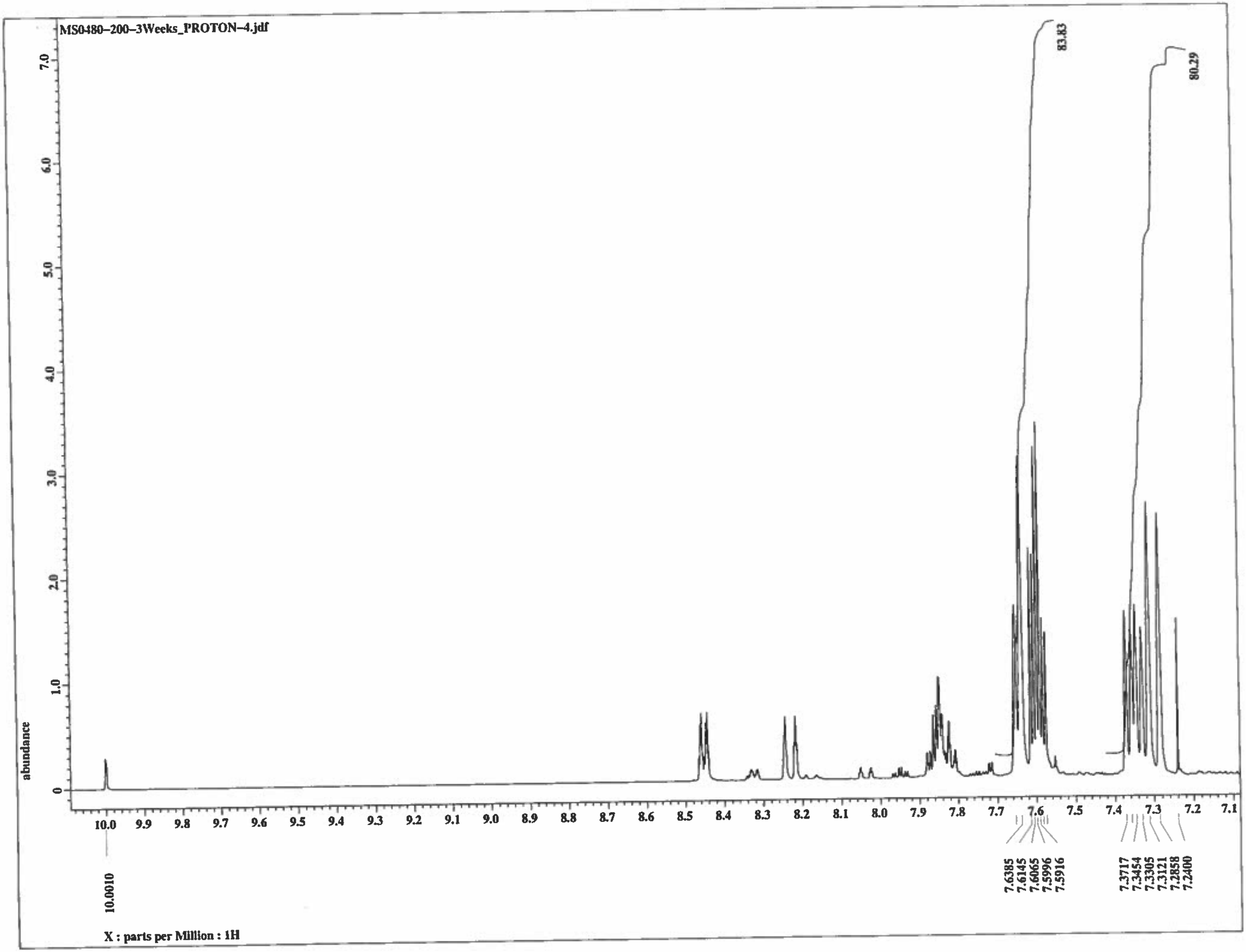

MS0480-200-3Weeks_PROTON-4.jdf
abundance
0
1.0
2.0
3.0
4.0
5.0
6.0
7.0
83.83
80.29
10.0
9.9
9.8
9.7
9.6
9.5
9.4
9.3
9.2
9.1
9.0
8.9
8.8
8.7
8.6
8.5
8.4
8.3
8.2
8.1
8.0
7.9
7.8
7.7
7.6
7.5
7.4
7.3
7.2
7.1
10.0010
7.6385
7.6145
7.6065
7.5996
7.5916
7.3717
7.3454
7.3305
7.3121
7.2858
7.2400
X : parts per Million : 1H

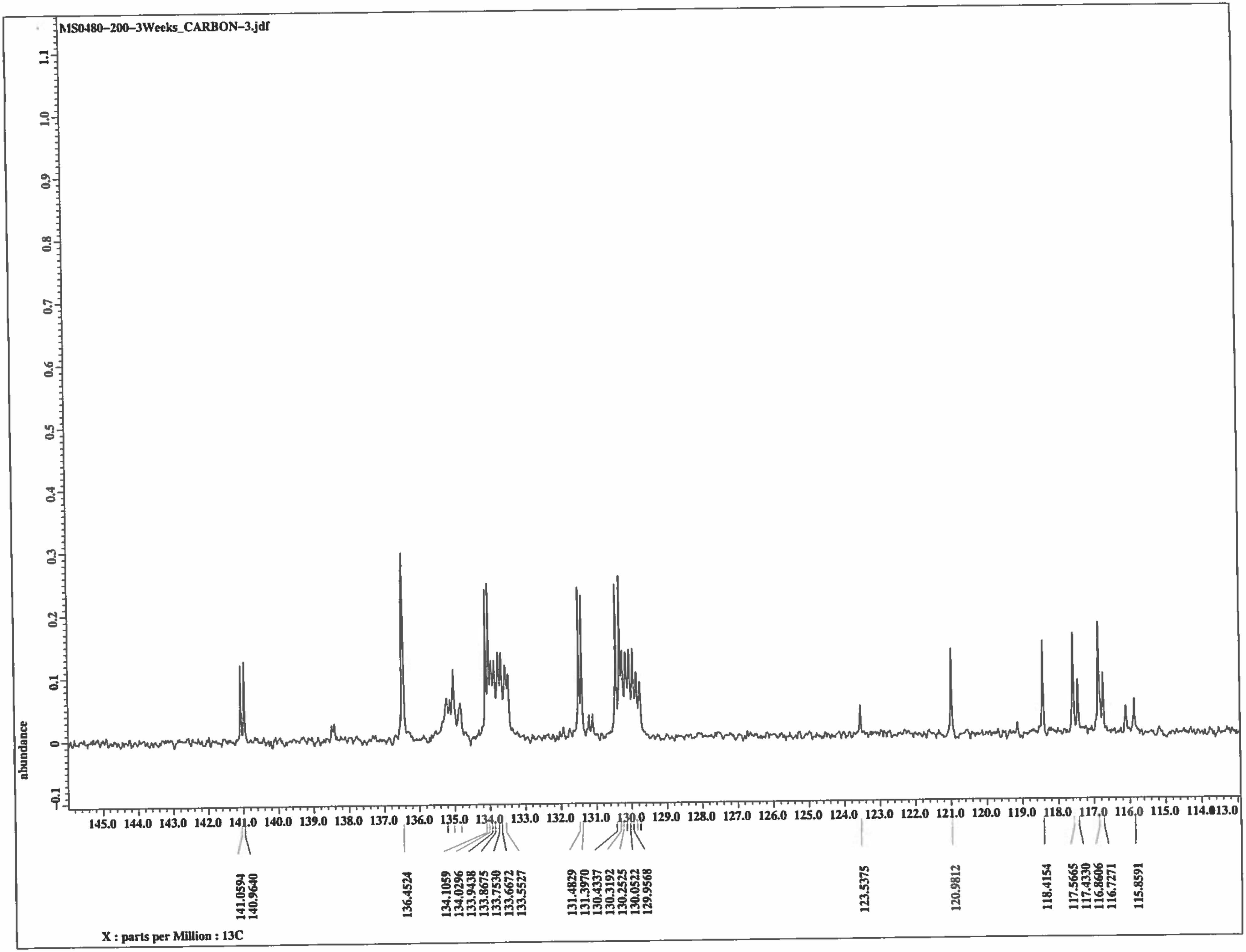

MS0480-200-3Weeks_CARBON-3.jdf
abundance
X : parts per Million : 13C
141.0594
140.9640
136.4524
134.1059
134.0296
133.9438
133.8675
133.7530
133.6672
133.5527
131.4829
131.3970
130.4337
130.3192
130.2525
130.0522
129.9568
123.5375
120.9812
118.4154
117.5665
117.4330
116.8606
116.7271
115.8591

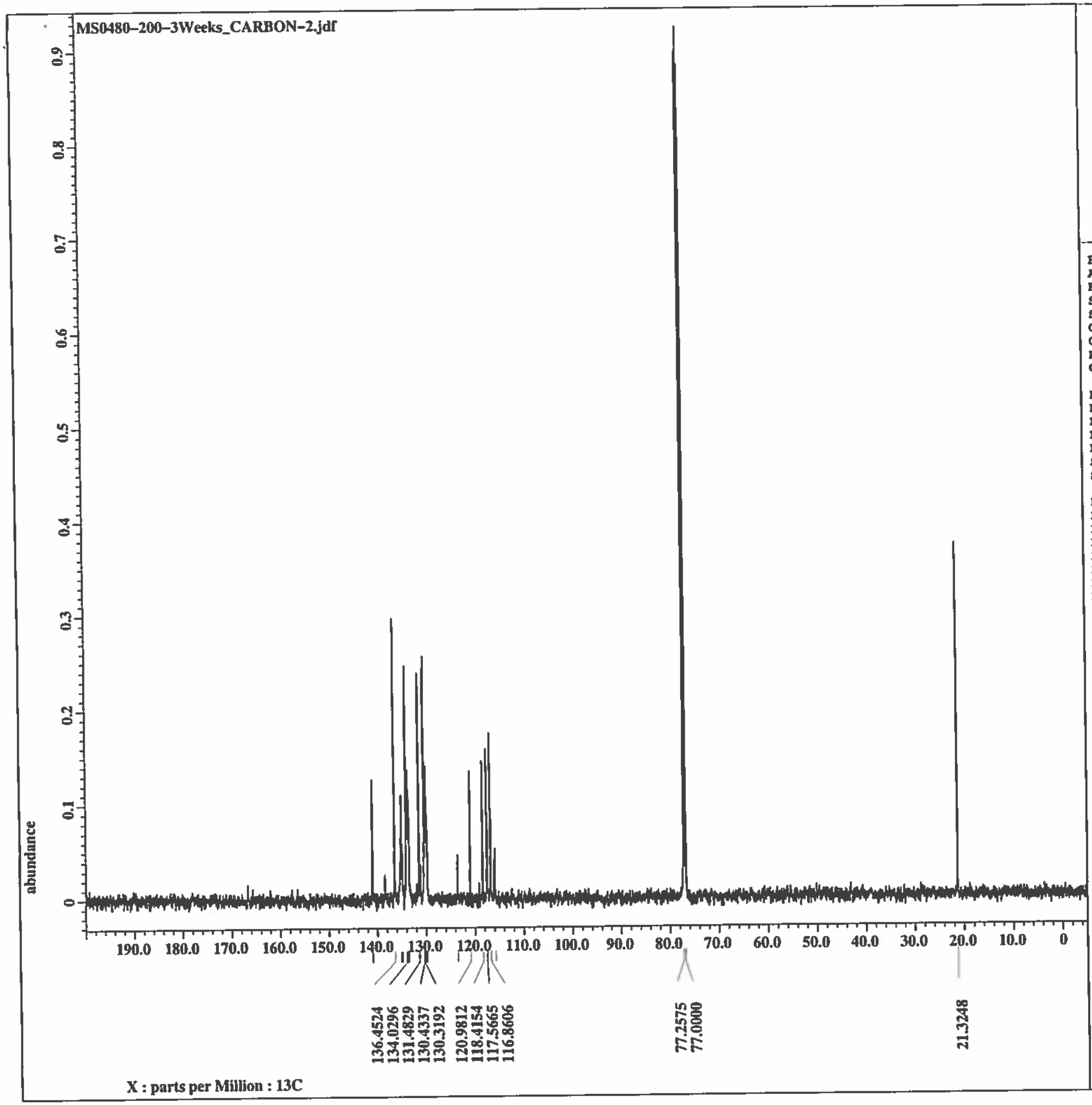

Filename	= MS0480-200-3Weeks_CAR
Author	= Jim Davis
Experiment	= single_pulse_dec
Sample_id	= MS0480-200-3Weeks
Solvent	= CHLOROFORM-D
Changer_sample	= 21
Creation_time	= 13-JUL-2018 10:34:27
Revision_time	= 13-JUL-2018 10:11:58
Current_time	= 13-JUL-2018 10:11:58
Data_format	= 1D COMPLEX
Dim_size	= 26214
Dim_title	= 13C
Dim_units	= [ppm]
Dimensions	= X
Site	= ECA 500
Spectrometer	= JNM-ECA500
Field_strength	= 11.7473579[T] (500[MH
X_acq_duration	= 0.83361792[s]
X_domain	= 13C
X_freq	= 125.76529768[MHz]
X_offset	= 100[ppm]
X_points	= 32768
X_prescans	= 4
X_resolution	= 1.19959034[Hz]
X_sweep	= 39.3081761[kHz]
Irr_domain	= 1H
Irr_freq	= 500.15991521[MHz]
Irr_offset	= 5.0[ppm]
Clipped	= FALSE
Mod_return	= 1
Scans	= 256
Total_scans	= 256
X_90_width	= 13.2[us]
X_acq_time	= 0.83361792[s]
X_angle	= 30[deg]
X_atn	= 6[dB]
X_pulse	= 4.4[us]
Irr_atn_dec	= 20.7[dB]
Irr_atn_noe	= 20.7[dB]
Irr_noise	= WALTZ
Decoupling	= TRUE
Initial_wait	= 1[s]
Noe	= TRUE
Noe_time	= 2[s]
Recvr_gain	= 60
Relaxation_delay	= 2[s]
Repetition_time	= 2.83361792[s]
Temp_get	= 23.2[dC]

MS0480-200-3Weeks_FLUORINE-2.jdf

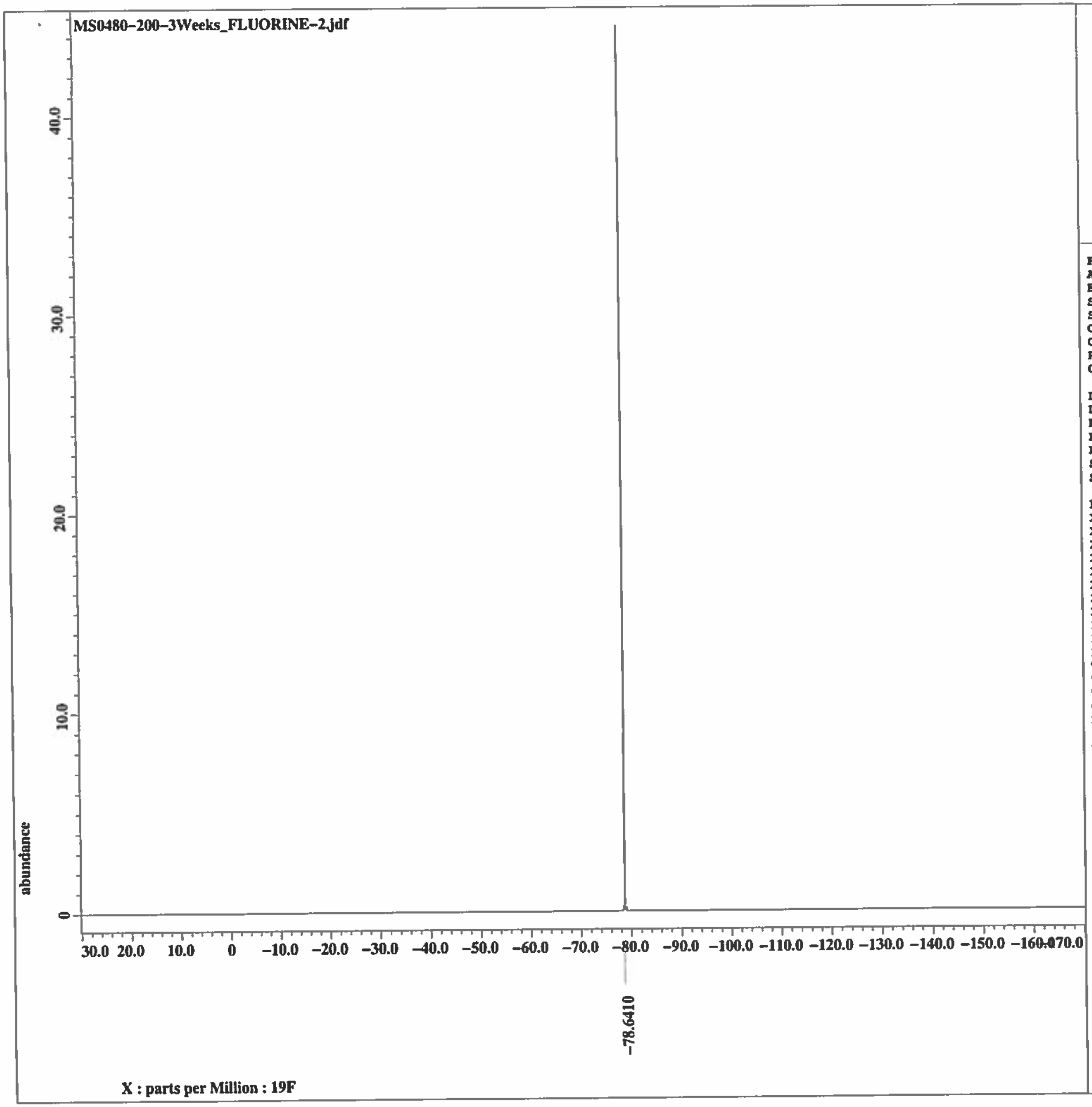

Filename	= MS0480-200-3Weeks_FLU
Author	= Jim Davis
Experiment	= single_pulse.ex2
Sample_id	= MS0480-200-3Weeks
Solvent	= CHLOROFORM-D
Changer_sample	= 21
Creation_time	= 13-JUL-2018 10:37:37
Revision_time	= 13-JUL-2018 10:15:09
Current_time	= 13-JUL-2018 10:15:09
Data_format	= 1D COMPLEX
Dim_size	= 52428
Dim_title	= 19F
Dim_units	= [ppm]
Dimensions	= X
Site	= ECA 500
Spectrometer	= JNM-ECA500
Field_strength	= 11.7473579[T] (500[MH
X_acq_duration	= 0.55574528[s]
X_domain	= 19F
X_freq	= 470.62046084[MHz]
X_offset	= -70[ppm]
X_points	= 65536
X_prescans	= 1
X_resolution	= 1.7993855[Hz]
X_sweep	= 117.9245283[kHz]
Irr_domain	= 19F
Irr_freq	= 470.62046084[MHz]
Irr_offset	= 5[ppm]
Tri_domain	= 19F
Tri_freq	= 470.62046084[MHz]
Tri_offset	= 5[ppm]
Clipped	= FALSE
Mod_return	= 1
Scans	= 16
Total_scans	= 16
X_90_width	= 13.1[us]
X_acq_time	= 0.55574528[s]
X_angle	= 45[deg]
X_atn	= 2.5[dB]
X_pulse	= 6.55[us]
Irr_mode	= Off
Tri_mode	= Off
Dante_presat	= FALSE
Initial_wait	= 1[s]
Recvr_gain	= 38
Relaxation_delay	= 4[s]
Repetition_time	= 4.55574528[s]
Temp_get	= 22.8[dC]

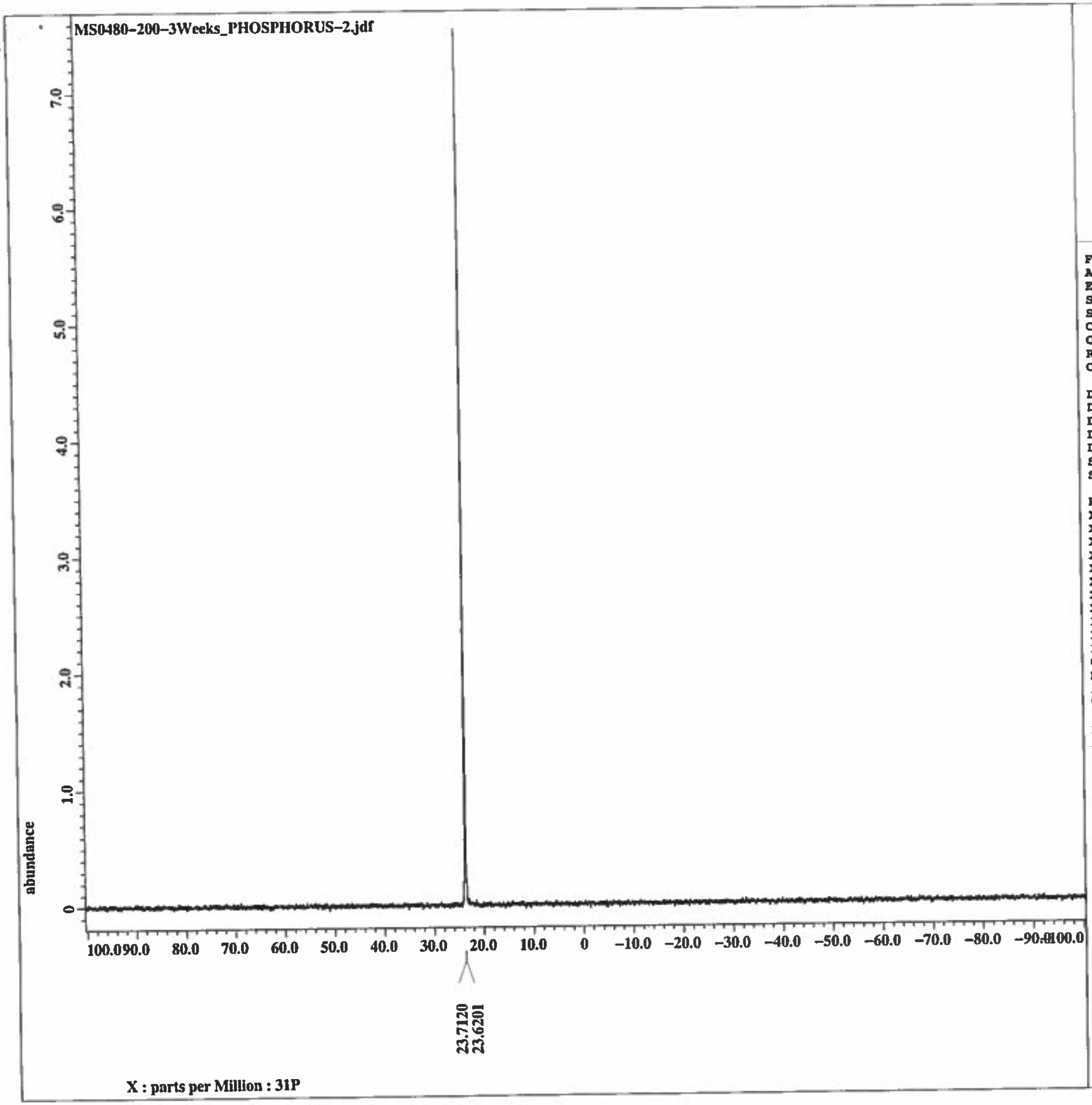

Filename	= MS0480-200-3Weeks_PHO
Author	= Jim Davis
Experiment	= single_pulse_dec
Sample_id	= MS0480-200-3Weeks
Solvent	= CHLOROFORM-D
Changer_sample	= 21
Creation_time	= 13-JUL-2018 10:41:16
Revision_time	= 13-JUL-2018 10:18:48
Current_time	= 13-JUL-2018 10:18:48
Data_format	= 1D COMPLEX
Dim_size	= 26214
Dim_title	= 31P
Dim_units	= [ppm]
Dimensions	= X
Site	= ECA 500
Spectrometer	= JNM-ECA500
Field_strength	= 11.7473579[T] (500[MH
X_acq_duration	= 0.64487424[s]
X_domain	= 31P
X_freq	= 202.46831075[MHz]
X_offset	= 0[ppm]
X_points	= 32768
X_prescans	= 4
X_resolution	= 1.55068995[Hz]
X_sweep	= 50.81300813[kHz]
Irr_domain	= 1H
Irr_freq	= 500.15991521[MHz]
Irr_offset	= 5.0[ppm]
Clipped	= FALSE
Mod_return	= 1
Scans	= 25
Total_scans	= 25
X_90_width	= 14.687[us]
X_acq_time	= 0.64487424[s]
X_angle	= 30[deg]
X_atn	= 5[dB]
X_pulse	= 4.89566667[us]
Irr_atn_dec	= 20.7[dB]
Irr_atn_noe	= 20.7[dB]
Irr_noise	= WALTZ
Decoupling	= TRUE
Initial_wait	= 1[s]
Noe	= TRUE
Noe_time	= 2[s]
Recvr_gain	= 58
Relaxation_delay	= 2[s]
Repetition_time	= 2.64487424[s]
Temp_get	= 23[dC]

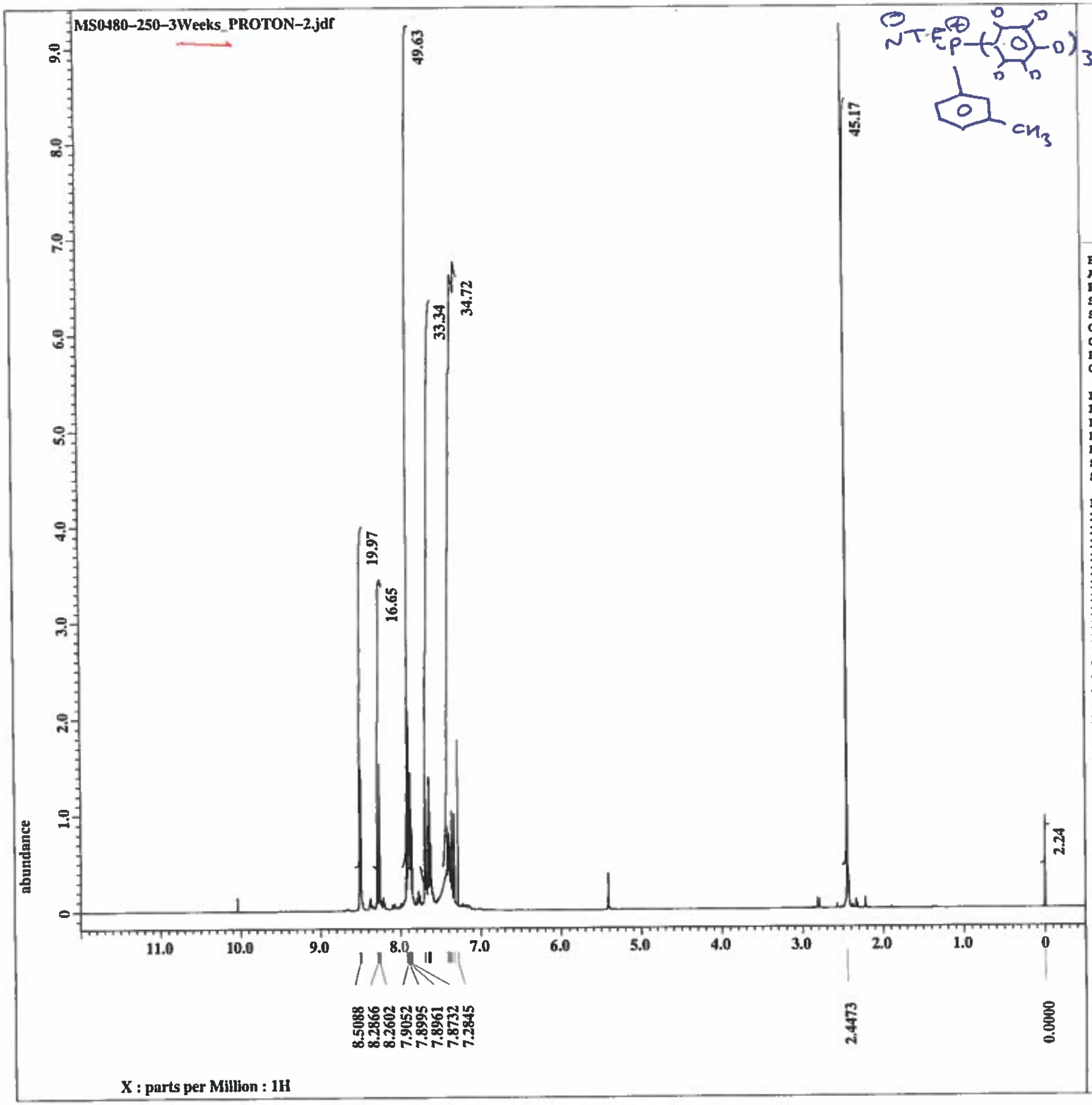

Filename	= MS0480-250-3Weeks_PRO
Author	= Jim Davis
Experiment	= single_pulse.ex2
Sample_id	= MS0480-250-3Weeks
Solvent	= CHLOROFORM-D
Changer_sample	= 22
Creation_time	= 13-JUL-2018 10:48:37
Revision_time	= 13-JUL-2018 10:26:10
Current_time	= 13-JUL-2018 10:26:10
Data_format	= 1D COMPLEX
Dim_size	= 13107
Dim_title	= 1H
Dim_units	= [ppm]
Dimensions	= X
Site	= ECA 500
Spectrometer	= JNM-ECA500
Field_strength	= 11.7473579[T] (500[MH
X_acq_duration	= 1.74587904[s]
X_domain	= 1H
X_freq	= 500.15991521[MHz]
X_offset	= 5.0[ppm]
X_points	= 16384
X_prescans	= 1
X_resolution	= 0.57277737[Hz]
X_sweep	= 9.38438438[kHz]
Irr_domain	= 1H
Irr_freq	= 500.15991521[MHz]
Irr_offset	= 5.0[ppm]
Tri_domain	= 1H
Tri_freq	= 500.15991521[MHz]
Tri_offset	= 5.0[ppm]
Clipped	= FALSE
Mod_return	= 1
Scans	= 16
Total_scans	= 16
X_90_width	= 12.4[us]
X_acq_time	= 1.74587904[s]
X_angle	= 45[deg]
X_atn	= 4[dB]
X_pulse	= 6.2[us]
Irr_mode	= Off
Tri_mode	= Off
Dante_presat	= FALSE
Initial_wait	= 1[s]
Recvr_gain	= 38
Relaxation_delay	= 4[s]
Repetition_time	= 5.74587904[s]
Temp_get	= 22.7[dC]

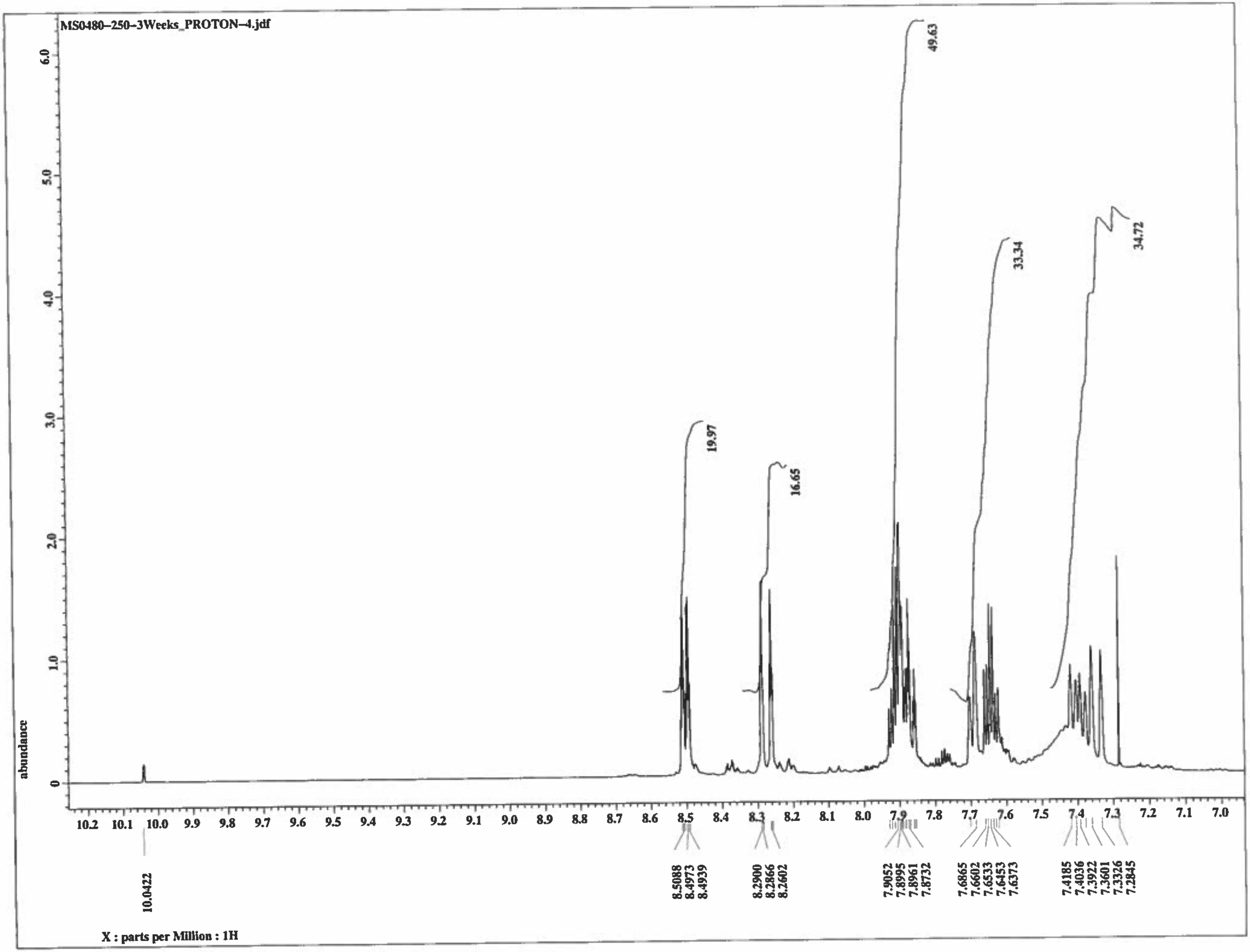
MS0480-250-3Weeks_PROTON-4.jdf
abundance
0
1.0
2.0
3.0
4.0
5.0
6.0
19.97
16.65
49.63
33.34
34.72
10.2
10.1
10.0
9.9
9.8
9.7
9.6
9.5
9.4
9.3
9.2
9.1
9.0
8.9
8.8
8.7
8.6
8.5
8.4
8.3
8.2
8.1
8.0
7.9
7.8
7.7
7.6
7.5
7.4
7.3
7.2
7.1
7.0
10.0422
8.5088
8.4973
8.4939
8.2900
8.2866
8.2602
7.9052
7.8995
7.8961
7.8732
7.6865
7.6602
7.6533
7.6453
7.6373
7.4185
7.4036
7.3922
7.3601
7.3326
7.2845
X : parts per Million : 1H

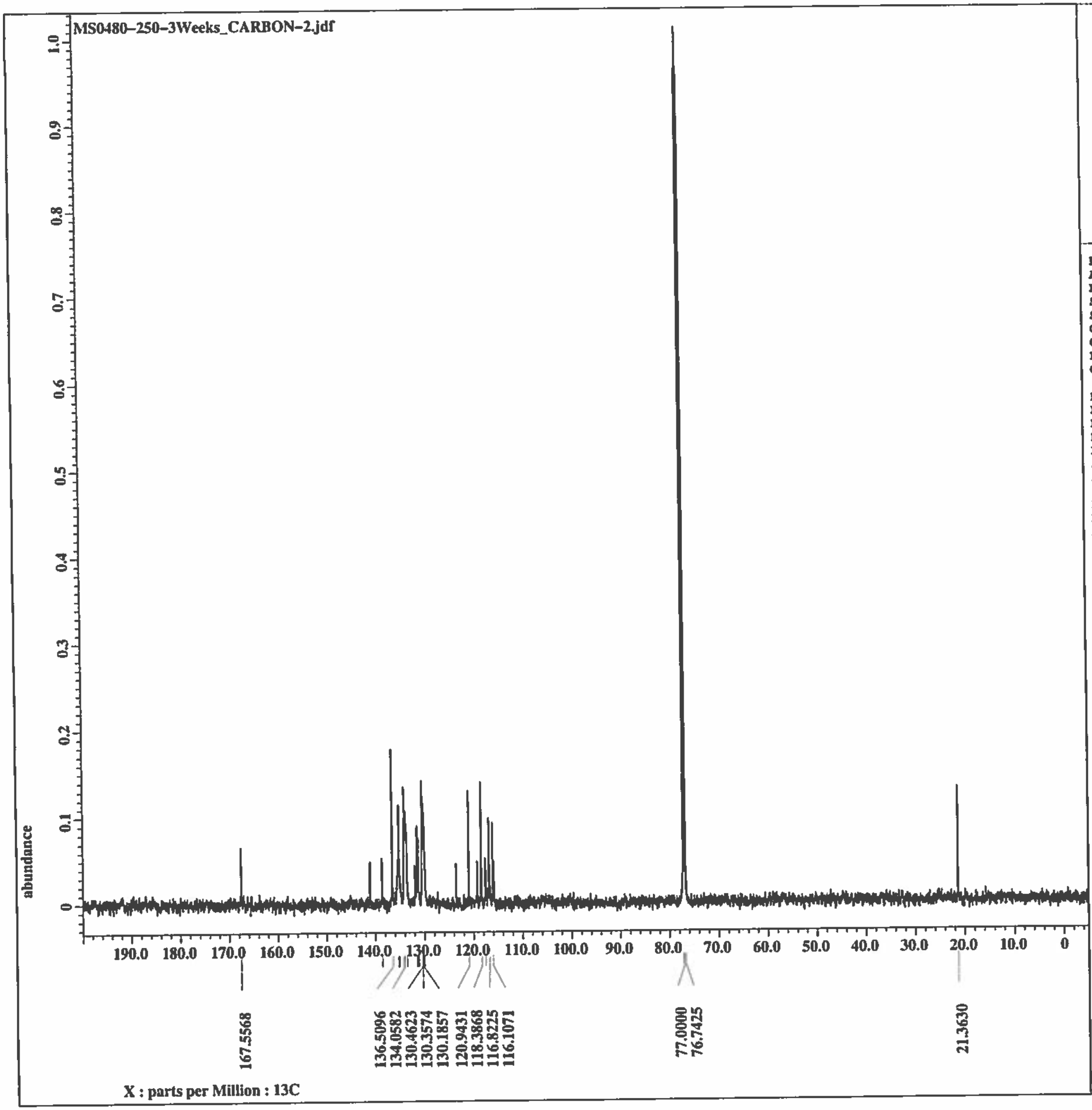

Filename	= MS0480-250-3Weeks_CAR
Author	= Jim Davis
Experiment	= single_pulse_dec
Sample_id	= MS0480-250-3Weeks
Solvent	= CHLOROFORM-D
Changer_sample	= 22
Creation_time	= 13-JUL-2018 11:03:06
Revision_time	= 13-JUL-2018 10:40:37
Current_time	= 13-JUL-2018 10:40:37
Data_format	= 1D COMPLEX
Dim_size	= 26214
Dim_title	= 13C
Dim_units	= [ppm]
Dimensions	= X
Site	= ECA 500
Spectrometer	= JNM-ECA500
Field_strength	= 11.7473579[T] (500[MH
X_acq_duration	= 0.83361792[s]
X_domain	= 13C
X_freq	= 125.76529768[MHz]
X_offset	= 100[ppm]
X_points	= 32768
X_prescans	= 4
X_resolution	= 1.19959034[Hz]
X_sweep	= 39.3081761[kHz]
Irr_domain	= 1H
Irr_freq	= 500.15991521[MHz]
Irr_offset	= 5.0[ppm]
Clipped	= FALSE
Mod_return	= 1
Scans	= 256
Total_scans	= 256
X_90_width	= 13.2[us]
X_acq_time	= 0.83361792[s]
X_angle	= 30[deg]
X_atn	= 6[dB]
X_pulse	= 4.4[us]
Irr_atn_dec	= 20.7[dB]
Irr_atn_noe	= 20.7[dB]
Irr_noise	= WALTZ
Decoupling	= TRUE
Initial_wait	= 1[s]
Noe	= TRUE
Noe_time	= 2[s]
Recvr_gain	= 60
Relaxation_delay	= 2[s]
Repetition_time	= 2.83361792[s]
Temp_get	= 23.2[dC]

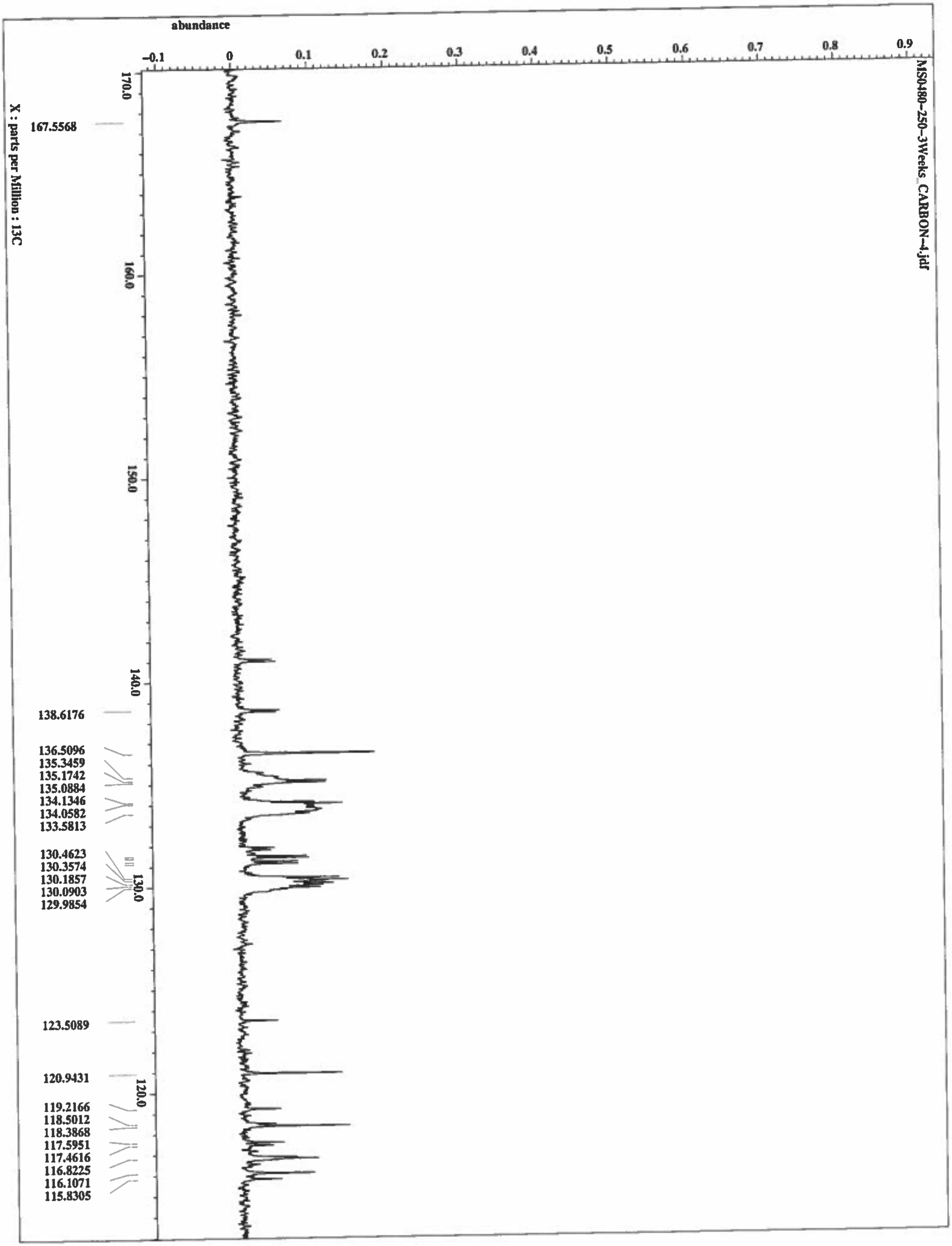
abundance
-0.1
0
0.1
0.2
0.3
0.4
0.5
0.6
0.7
0.8
0.9
MS0480-250-3Weeks_CARBON-4.jdf
X : parts per Million : 13C
170.0
160.0
150.0
140.0
130.0
120.0
167.5568
138.6176
136.5096
135.3459
135.1742
135.0884
134.1346
134.0582
133.5813
130.4623
130.3574
130.1857
130.0903
129.9854
123.5089
120.9431
119.2166
118.5012
118.3868
117.5951
117.4616
116.8225
116.1071
115.8305

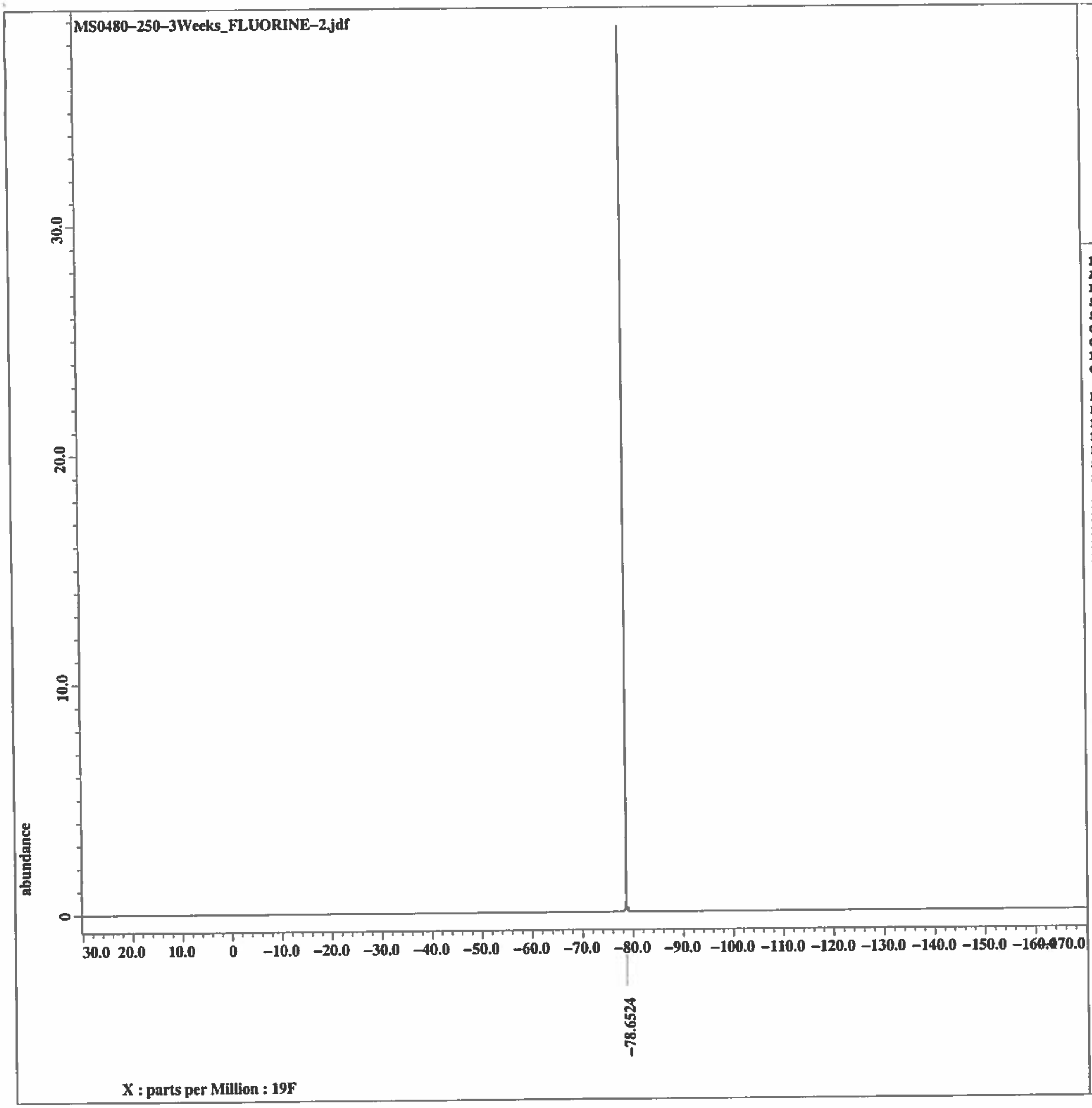


```
Filename           = MS0480-250-3Weeks_FLU
Author             = Jim Davis
Experiment         = single_pulse.ex2
Sample_id          = MS0480-250-3Weeks
Solvent            = CHLOROFORM-D
Changer_sample     = 22
Creation_time      = 13-JUL-2018 11:06:09
Revision_time      = 13-JUL-2018 10:43:42
Current_time       = 13-JUL-2018 10:43:43

Data_format        = 1D COMPLEX
Dim_size           = 52428
Dim_title          = 19F
Dim_units          = [ppm]
Dimensions         = X
Site               = ECA 500
Spectrometer       = JNM-ECA500

Field_strength     = 11.7473579[T] (500[MH
X_acq_duration     = 0.55574528[s]
X_domain           = 19F
X_freq             = 470.62046084[MHz]
X_offset           = -70[ppm]
X_points           = 65536
X_prescans         = 1
X_resolution       = 1.7993855[Hz]
X_sweep            = 117.9245283[kHz]
Irr_domain         = 19F
Irr_freq           = 470.62046084[MHz]
Irr_offset         = 5[ppm]
Tri_domain         = 19F
Tri_freq           = 470.62046084[MHz]
Tri_offset         = 5[ppm]
Clipped            = FALSE
Mod_return         = 1
Scans              = 16
Total_scans        = 16

X_90_width         = 13.1[us]
X_acq_time         = 0.55574528[s]
X_angle            = 45[deg]
X_atn              = 2.5[dB]
X_pulse            = 6.55[us]
Irr_mode           = Off
Tri_mode           = Off
Dante_presat       = FALSE
Initial_wait       = 1[s]
Recvr_gain         = 38
Relaxation_delay   = 4[s]
Repetition_time    = 4.55574528[s]
Temp_get           = 22.8[dC]
```

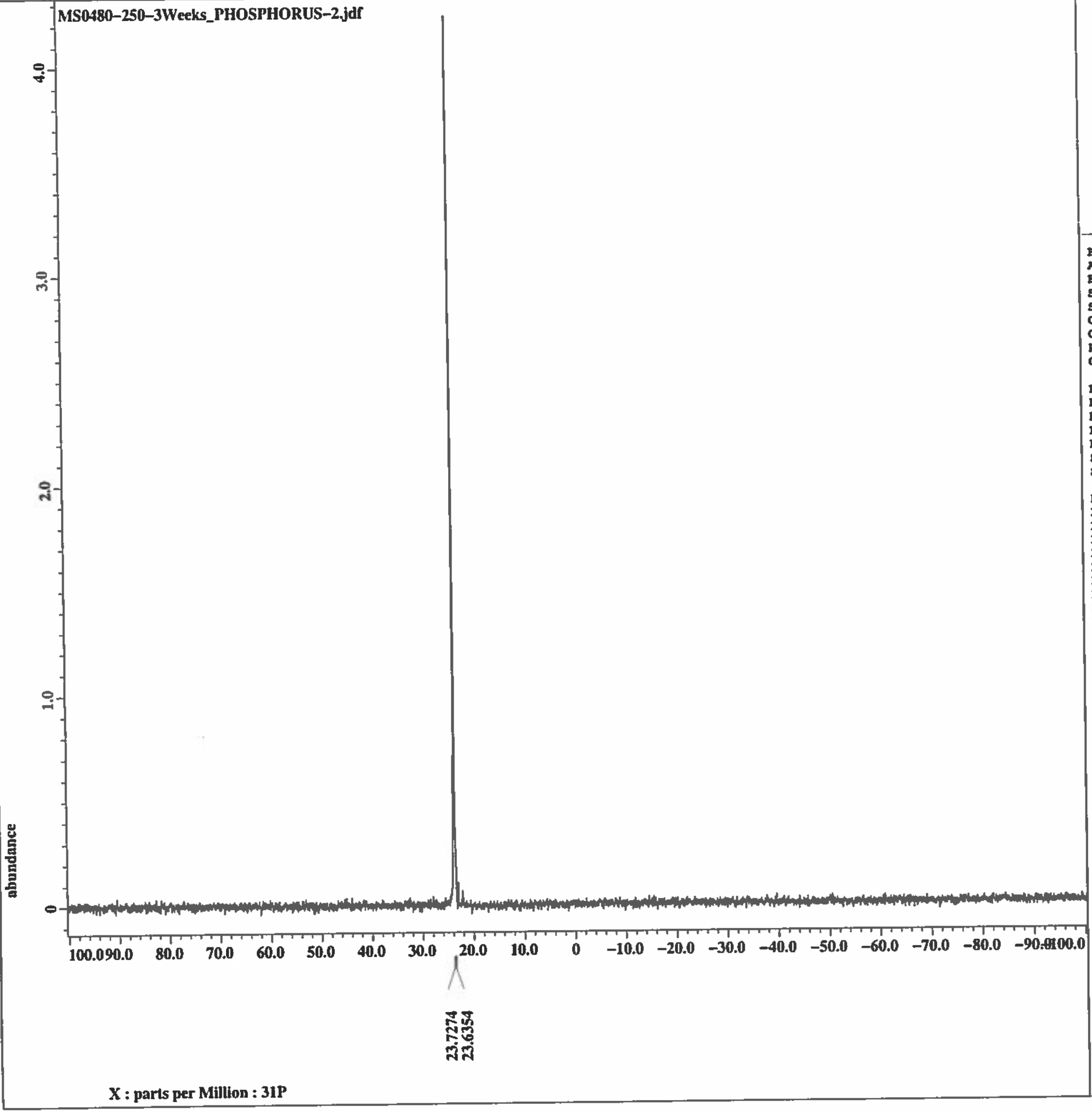

Filename	= MS0480-250-3Weeks_PHO
Author	= Jim Davis
Experiment	= single_pulse_dec
Sample_id	= MS0480-250-3Weeks
Solvent	= CHLOROFORM-D
Changer_sample	= 22
Creation_time	= 13-JUL-2018 11:10:13
Revision_time	= 13-JUL-2018 10:47:44
Current_time	= 13-JUL-2018 10:47:44
Data_format	= 1D COMPLEX
Dim_size	= 26214
Dim_title	= 31P
Dim_units	= [ppm]
Dimensions	= X
Site	= ECA 500
Spectrometer	= JNM-ECA500
Field_strength	= 11.7473579[T] (500[MH
X_acq_duration	= 0.64487424[s]
X_domain	= 31P
X_freq	= 202.46831075[MHz]
X_offset	= 0[ppm]
X_points	= 32768
X_prescans	= 4
X_resolution	= 1.55068995[Hz]
X_sweep	= 50.81300813[kHz]
Irr_domain	= 1H
Irr_freq	= 500.15991521[MHz]
Irr_offset	= 5.0[ppm]
Clipped	= FALSE
Mod_return	= 1
Scans	= 25
Total_scans	= 25
X_90_width	= 14.687[us]
X_acq_time	= 0.64487424[s]
X_angle	= 30[deg]
X_atn	= 5[dB]
X_pulse	= 4.89566667[us]
Irr_atn_dec	= 20.7[dB]
Irr_atn_noe	= 20.7[dB]
Irr_noise	= WALTZ
Decoupling	= TRUE
Initial_wait	= 1[s]
Noe	= TRUE
Noe_time	= 2[s]
Recvr_gain	= 58
Relaxation_delay	= 2[s]
Repetition_time	= 2.64487424[s]
Temp_get	= 23[dC]

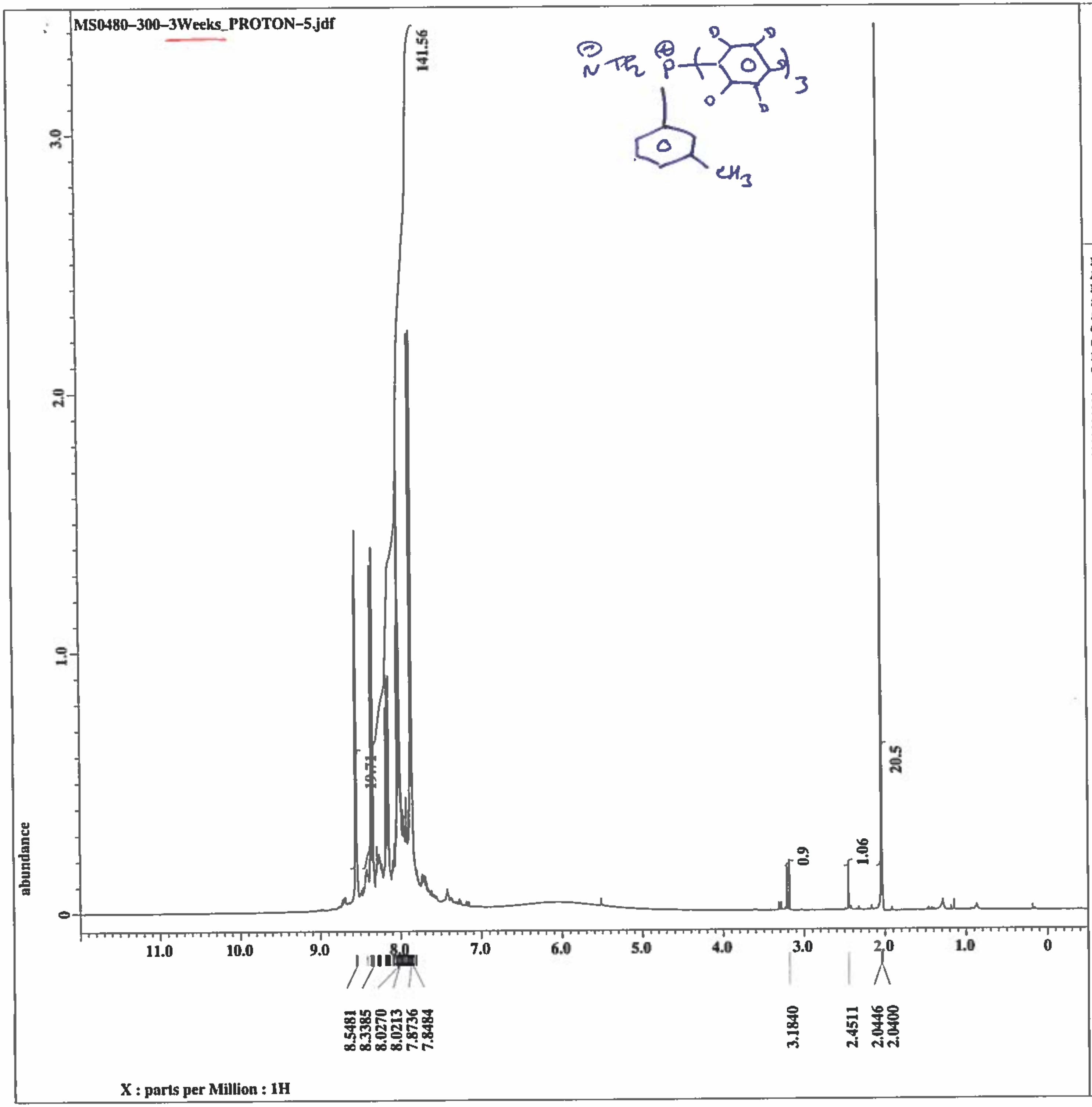

Filename	= MS0480-300-3Weeks_PRO
Author	= Jim Davis
Experiment	= single_pulse.ex2
Sample_id	= MS0480-300-3Weeks
Solvent	= ACETONE-D6
Changer_sample	= 6
Creation_time	= 14-JUL-2018 11:14:36
Revision_time	= 14-JUL-2018 10:52:04
Current_time	= 14-JUL-2018 10:52:04
Data_format	= 1D COMPLEX
Dim_size	= 13107
Dim_title	= 1H
Dim_units	= [ppm]
Dimensions	= X
Site	= ECA 500
Spectrometer	= JNM-ECA500
Field_strength	= 11.7473579[T] (500[MH
X_acq_duration	= 1.74587904[s]
X_domain	= 1H
X_freq	= 500.15991521[MHz]
X_offset	= 5.0[ppm]
X_points	= 16384
X_prescans	= 1
X_resolution	= 0.57277737[Hz]
X_sweep	= 9.38438438[kHz]
Irr_domain	= 1H
Irr_freq	= 500.15991521[MHz]
Irr_offset	= 5.0[ppm]
Tri_domain	= 1H
Tri_freq	= 500.15991521[MHz]
Tri_offset	= 5.0[ppm]
Clipped	= FALSE
Mod_return	= 1
Scans	= 16
Total_scans	= 16
X_90_width	= 12.4[us]
X_acq_time	= 1.74587904[s]
X_angle	= 45[deg]
X_atn	= 4[dB]
X_pulse	= 6.2[us]
Irr_mode	= Off
Tri_mode	= Off
Dante_presat	= FALSE
Initial_wait	= 1[s]
Recvr_gain	= 40
Relaxation_delay	= 4[s]
Repetition_time	= 5.74587904[s]
Temp_get	= 22.5[dC]

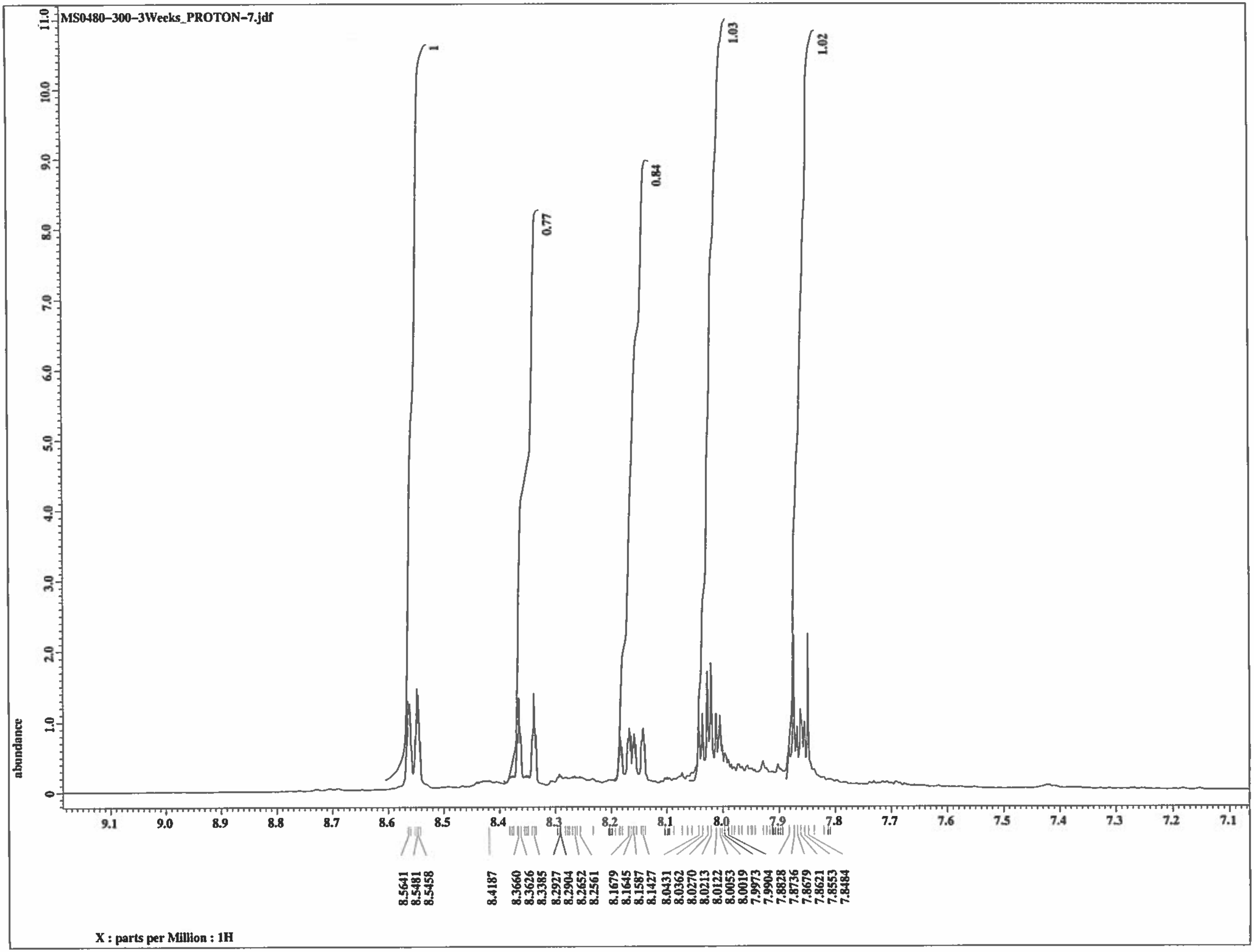
MS0480-300-3Weeks_PROTON-7.jdf
abundance
0
1.0
2.0
3.0
4.0
5.0
6.0
7.0
8.0
9.0
10.0
11.0
1
0.77
0.84
1.03
1.02
9.1
9.0
8.9
8.8
8.7
8.6
8.5
8.4
8.3
8.2
8.1
8.0
7.9
7.8
7.7
7.6
7.5
7.4
7.3
7.2
7.1
8.5641
8.5481
8.5458
8.4187
8.3660
8.3626
8.3385
8.2927
8.2904
8.2652
8.2561
8.1679
8.1645
8.1587
8.1427
8.0431
8.0362
8.0270
8.0213
8.0122
8.0053
8.0019
7.9973
7.9904
7.8828
7.8736
7.8679
7.8621
7.8553
7.8484
X : parts per Million : 1H

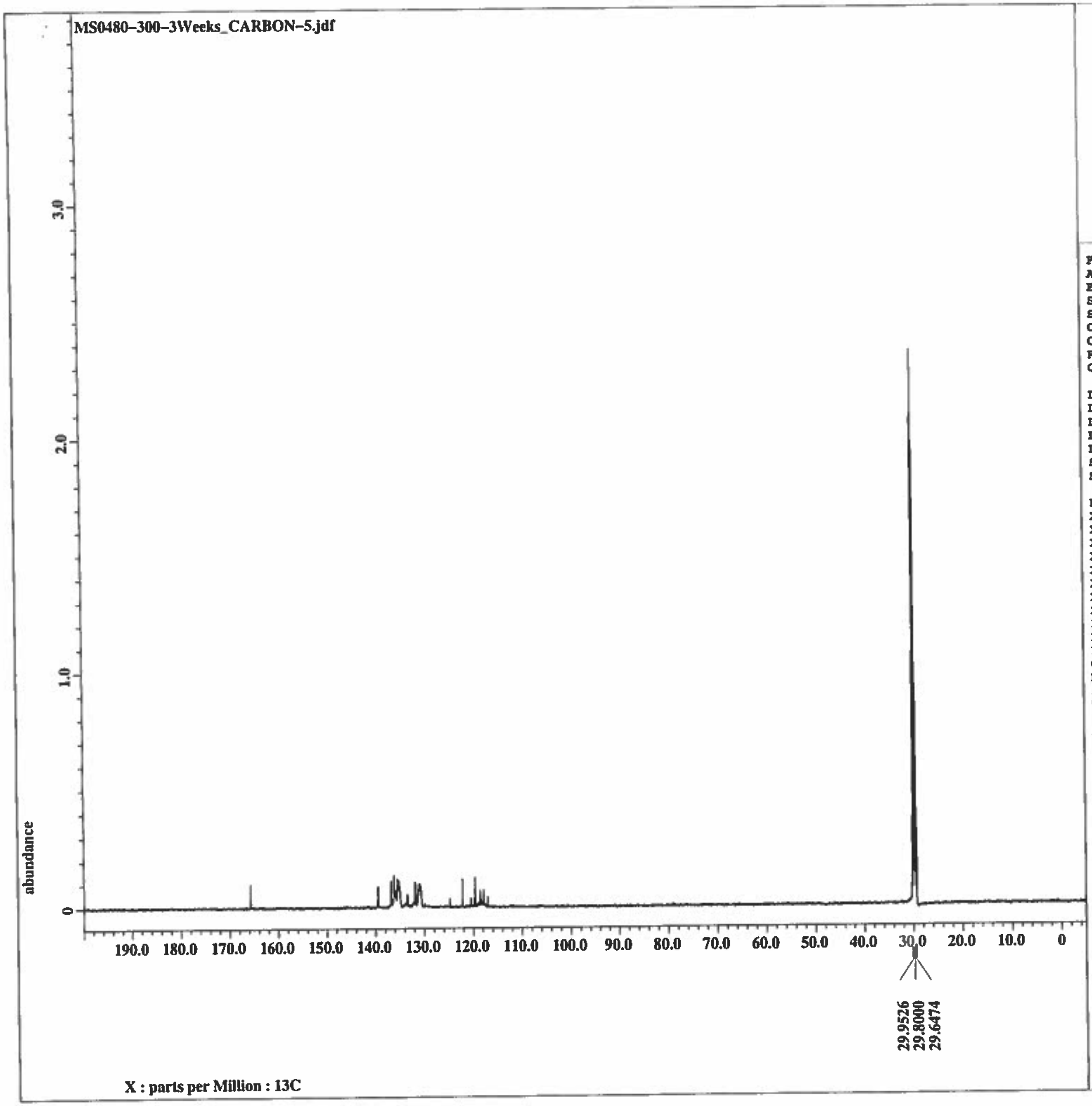

Filename	= MS0480-300-3Weeks_CAR
Author	= Jim Davis
Experiment	= single_pulse_dec
Sample_id	= MS0480-300-3Weeks
Solvent	= ACETONE-D6
Changer_sample	= 6
Creation_time	= 14-JUL-2018 11:36:02
Revision_time	= 14-JUL-2018 11:13:31
Current_time	= 14-JUL-2018 11:13:31
Data_format	= 1D COMPLEX
Dim_size	= 26214
Dim_title	= 13C
Dim_units	= [ppm]
Dimensions	= X
Site	= ECA 500
Spectrometer	= JNM-ECA500
Field_strength	= 11.7473579[T] (500[MH
X_acq_duration	= 0.83361792[s]
X_domain	= 13C
X_freq	= 125.76529768[MHz]
X_offset	= 100[ppm]
X_points	= 32768
X_prescans	= 4
X_resolution	= 1.19959034[Hz]
X_sweep	= 39.3081761[kHz]
Irr_domain	= 1H
Irr_freq	= 500.15991521[MHz]
Irr_offset	= 5.0[ppm]
Clipped	= FALSE
Mod_return	= 1
Scans	= 400
Total_scans	= 400
X_90_width	= 13.2[us]
X_acq_time	= 0.83361792[s]
X_angle	= 30[deg]
X_atn	= 6[dB]
X_pulse	= 4.4[us]
Irr_atn_dec	= 20.7[dB]
Irr_atn_noe	= 20.7[dB]
Irr_noise	= WALTZ
Decoupling	= TRUE
Initial_wait	= 1[s]
Noe	= TRUE
Noe_time	= 2[s]
Recvr_gain	= 60
Relaxation_delay	= 2[s]
Repetition_time	= 2.83361792[s]
Temp_get	= 23.1[dC]

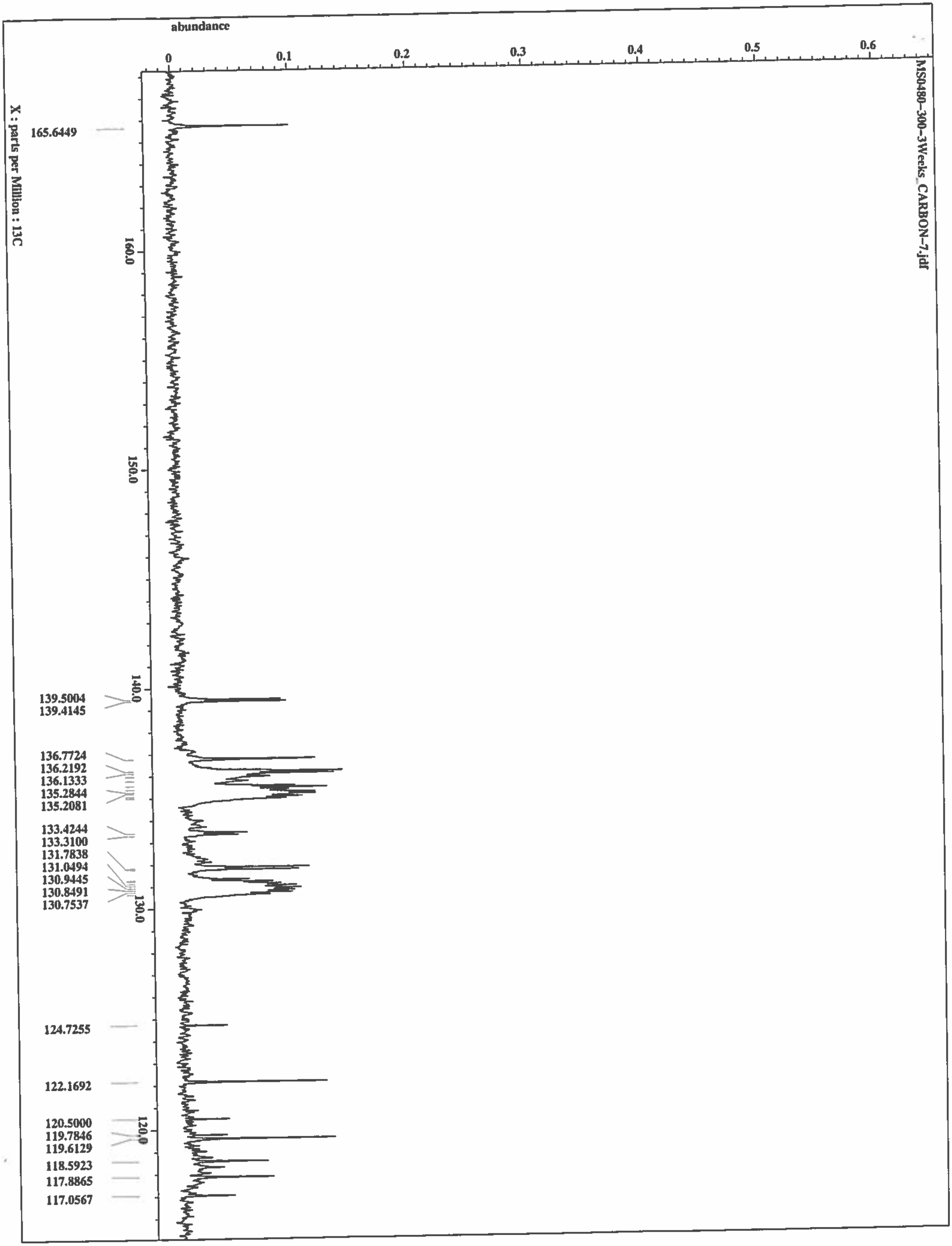
abundance
0
0.1
0.2
0.3
0.4
0.5
0.6
MS0480-300-3Weeks_CARBON-7.jdf
X : parts per Million : 13C
165.6449
160.0
150.0
140.0
139.5004
139.4145
136.7724
136.2192
136.1333
135.2844
135.2081
133.4244
133.3100
131.7838
131.0494
130.9445
130.8491
130.7537
130.0
124.7255
122.1692
120.5000
119.7846
119.6129
118.5923
117.8865
117.0567
120.0

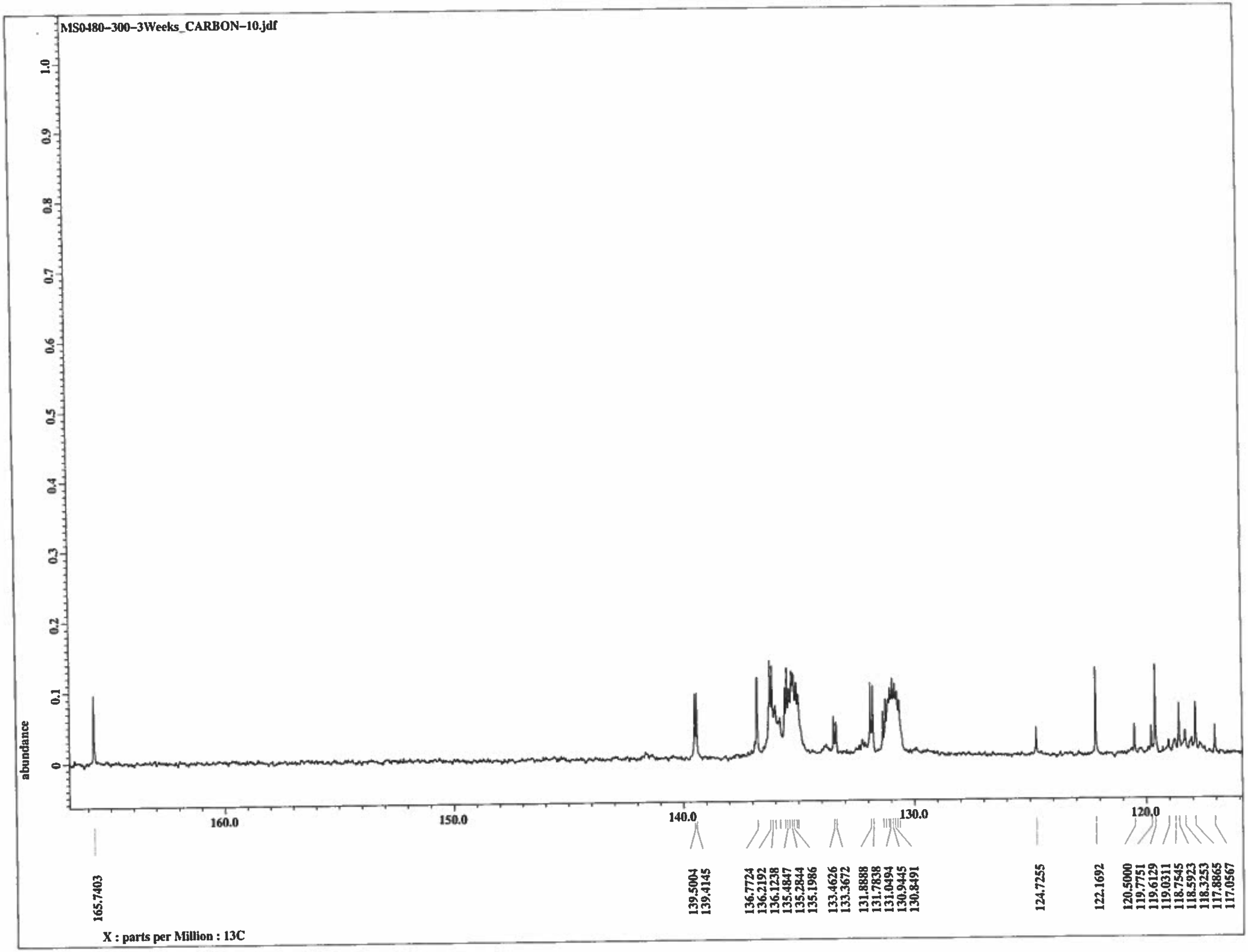
MS0480-300-3Weeks_CARBON-10.jdf
abundance
0
0.1
0.2
0.3
0.4
0.5
0.6
0.7
0.8
0.9
1.0
160.0
150.0
140.0
130.0
120.0
165.7403
139.5004
139.4145
136.7724
136.2192
136.1238
135.4847
135.2844
135.1986
133.4626
133.3672
131.8888
131.7838
131.0494
130.9445
130.8491
124.7255
122.1692
120.5000
119.7751
119.6129
119.0311
118.7545
118.5923
118.3253
117.8865
117.0567
X : parts per Million : 13C

MS0480-300-3Weeks_FLUORINE-5.jdf

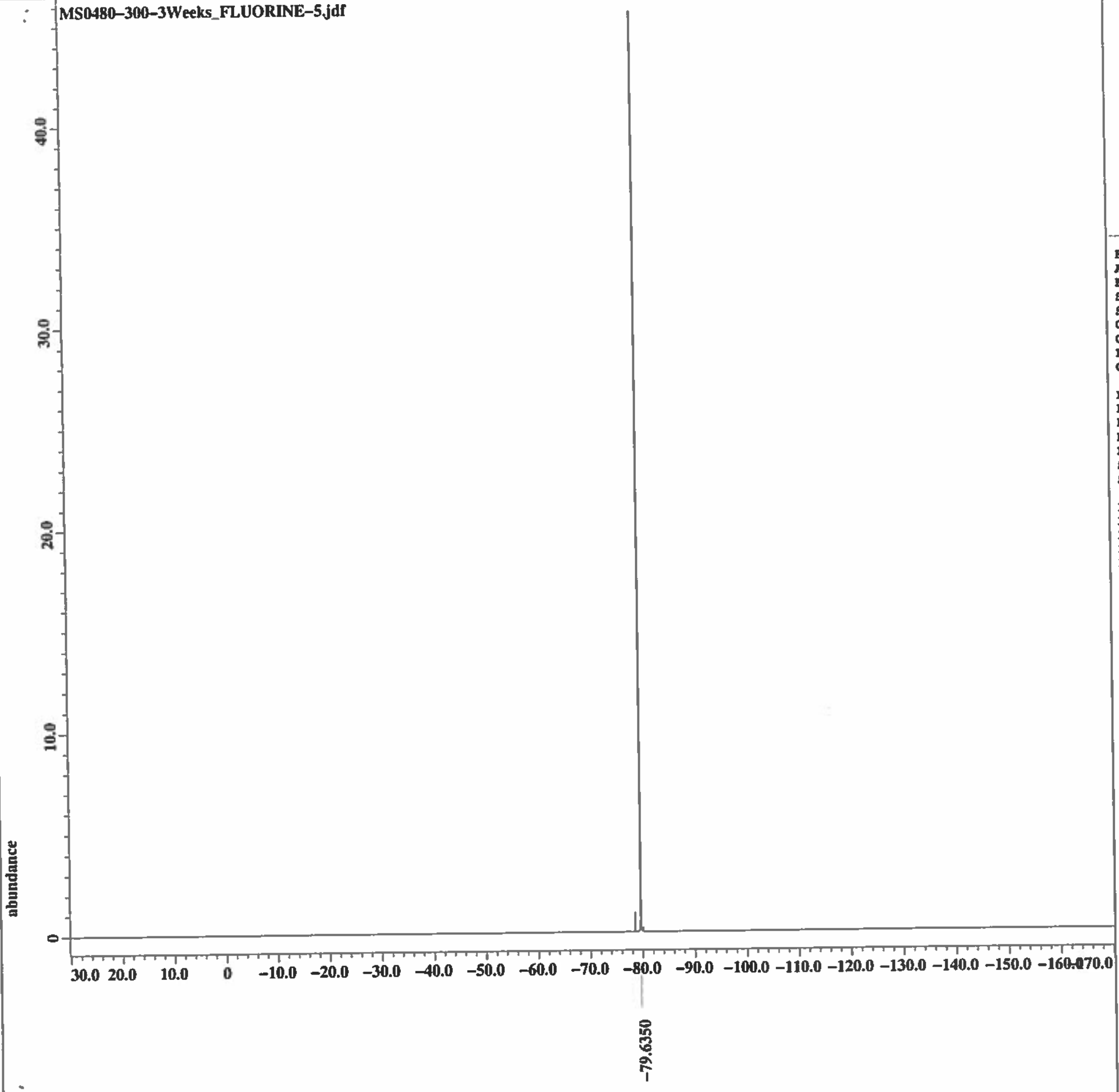

X : parts per Million : 19F

Filename	= MS0480-300-3Weeks_FLU
Author	= Jim Davis
Experiment	= single_pulse.ex2
Sample_id	= MS0480-300-3Weeks
Solvent	= ACETONE-D6
Changer_sample	= 6
Creation_time	= 14-JUL-2018 11:39:17
Revision_time	= 14-JUL-2018 11:16:46
Current_time	= 14-JUL-2018 11:16:47
Data_format	= 1D COMPLEX
Dim_size	= 52428
Dim_title	= 19F
Dim_units	= [ppm]
Dimensions	= X
Site	= ECA 500
Spectrometer	= JNM-ECA500
Field_strength	= 11.7473579[T] (500[MH
X_acq_duration	= 0.55574528[s]
X_domain	= 19F
X_freq	= 470.62046084[MHz]
X_offset	= -70[ppm]
X_points	= 65536
X_prescans	= 1
X_resolution	= 1.7993855[Hz]
X_sweep	= 117.9245283[kHz]
Irr_domain	= 19F
Irr_freq	= 470.62046084[MHz]
Irr_offset	= 5[ppm]
Tri_domain	= 19F
Tri_freq	= 470.62046084[MHz]
Tri_offset	= 5[ppm]
Clipped	= FALSE
Mod_return	= 1
Scans	= 16
Total_scans	= 16
X_90_width	= 13.1[us]
X_acq_time	= 0.55574528[s]
X_angle	= 45[deg]
X_atn	= 2.5[dB]
X_pulse	= 6.55[us]
Irr_mode	= Off
Tri_mode	= Off
Dante_presat	= FALSE
Initial_wait	= 1[s]
Recvr_gain	= 42
Relaxation_delay	= 4[s]
Repetition_time	= 4.55574528[s]
Temp_get	= 22.7[dC]

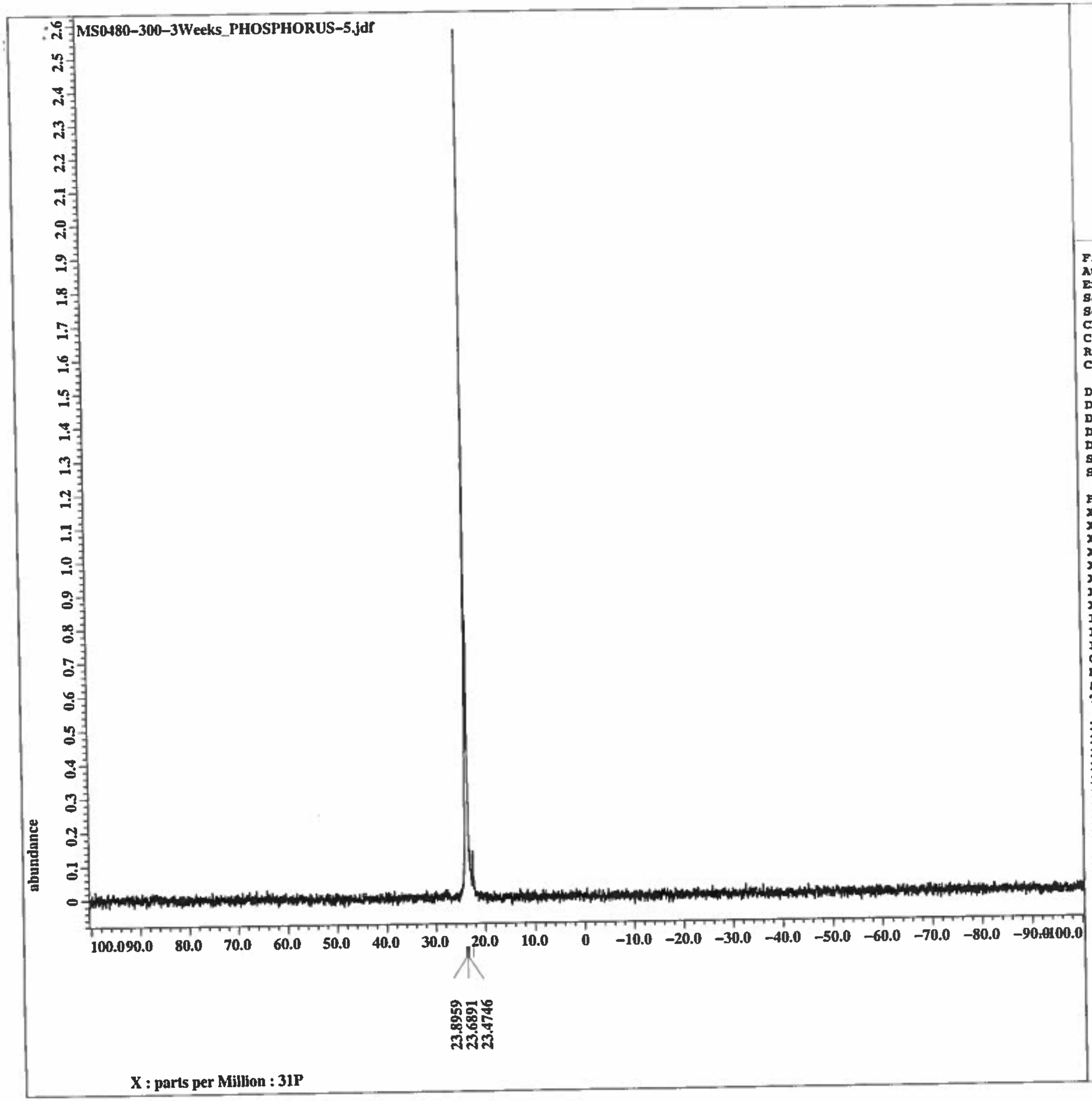

Filename	= MS0480-300-3Weeks_PHO
Author	= Jim Davis
Experiment	= single_pulse_dec
Sample_id	= MS0480-300-3Weeks
Solvent	= ACETONE-D6
Changer_sample	= 6
Creation_time	= 14-JUL-2018 11:44:00
Revision_time	= 14-JUL-2018 11:21:28
Current_time	= 14-JUL-2018 11:21:28
Data_format	= 1D COMPLEX
Dim_size	= 26214
Dim_title	= 31P
Dim_units	= [ppm]
Dimensions	= X
Site	= ECA 500
Spectrometer	= JNM-ECA500
Field_strength	= 11.7473579[T] (500[MH
X_acq_duration	= 0.64487424[s]
X_domain	= 31P
X_freq	= 202.46831075[MHz]
X_offset	= 0[ppm]
X_points	= 32768
X_prescans	= 4
X_resolution	= 1.55068995[Hz]
X_sweep	= 50.81300813[kHz]
Irr_domain	= 1H
Irr_freq	= 500.15991521[MHz]
Irr_offset	= 5.0[ppm]
Clipped	= FALSE
Mod_return	= 1
Scans	= 50
Total_scans	= 50
X_90_width	= 14.687[us]
X_acq_time	= 0.64487424[s]
X_angle	= 30[deg]
X_atn	= 5[dB]
X_pulse	= 4.89566667[us]
Irr_atn_dec	= 20.7[dB]
Irr_atn_noe	= 20.7[dB]
Irr_noise	= WALTZ
Decoupling	= TRUE
Initial_wait	= 1[s]
Noe	= TRUE
Noe_time	= 2[s]
Recvr_gain	= 58
Relaxation_delay	= 2[s]
Repetition_time	= 2.64487424[s]
Temp_get	= 23[dC]

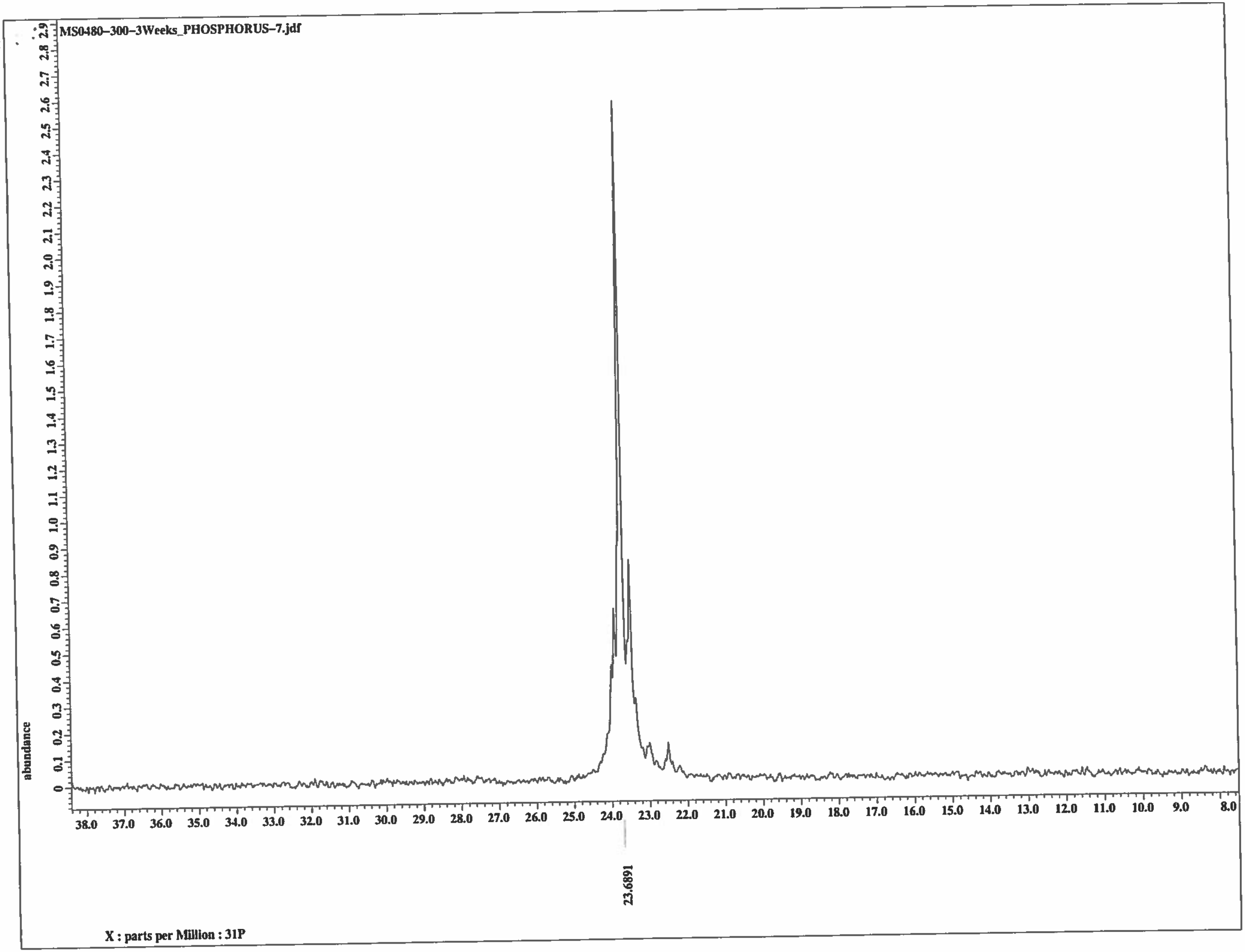

MS0480-300-3Weeks_PHOSPHORUS-7.jdf
abundance
0 0.1 0.2 0.3 0.4 0.5 0.6 0.7 0.8 0.9 1.0 1.1 1.2 1.3 1.4 1.5 1.6 1.7 1.8 1.9 2.0 2.1 2.2 2.3 2.4 2.5 2.6 2.7 2.8 2.9
38.0 37.0 36.0 35.0 34.0 33.0 32.0 31.0 30.0 29.0 28.0 27.0 26.0 25.0 24.0 23.0 22.0 21.0 20.0 19.0 18.0 17.0 16.0 15.0 14.0 13.0 12.0 11.0 10.0 9.0 8.0
23.6891
X : parts per Million : 31P

Compound 7 Pre- and Post-heating NMR Spectra

Temperature of Post-heating samples noted in upper left corner of each spectrum

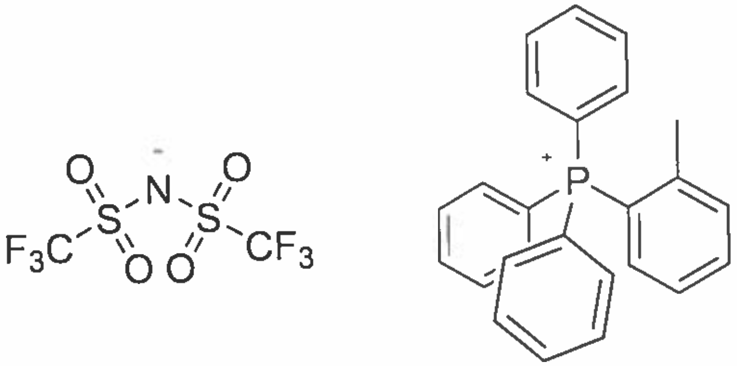

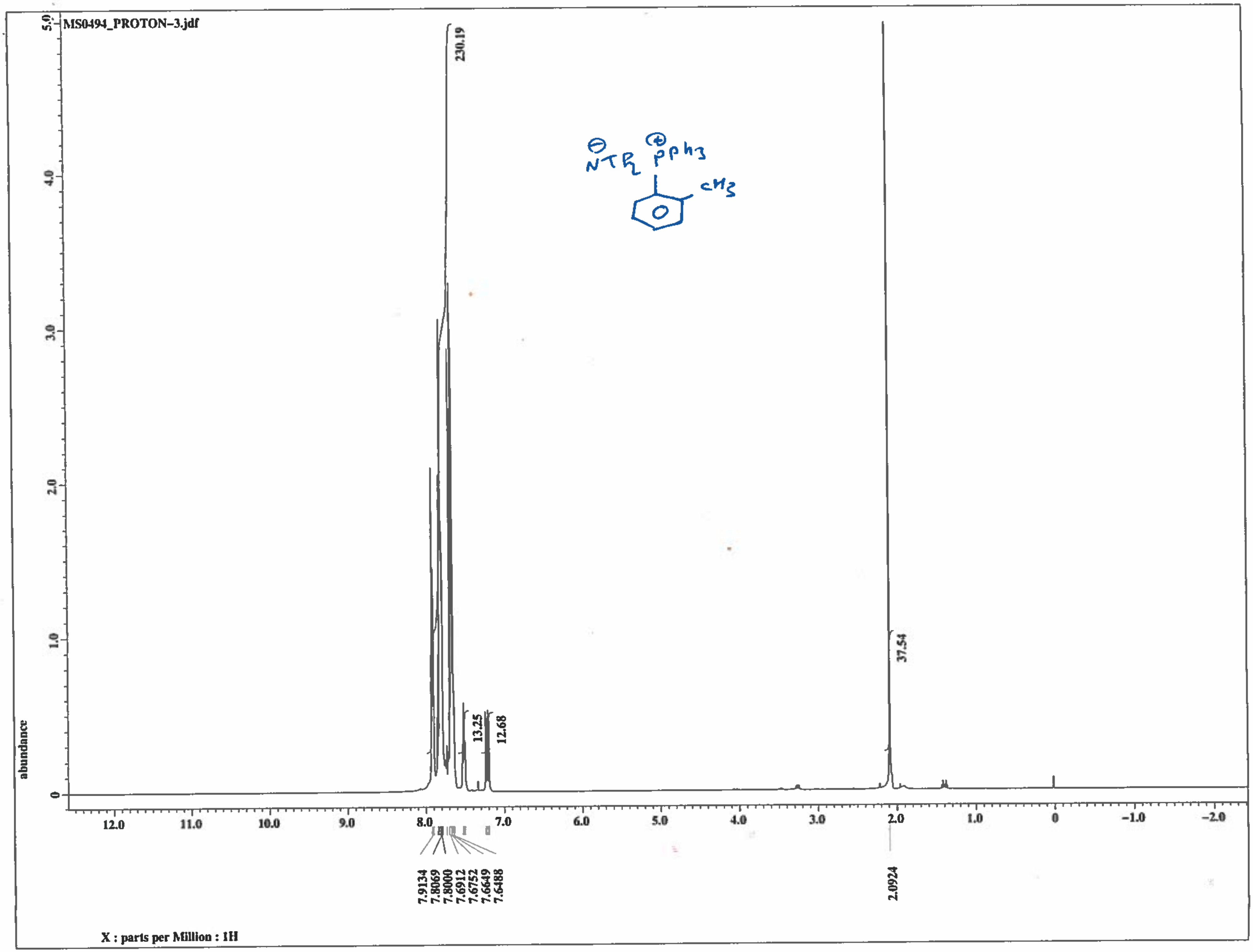
MS0494_PROTON-3.jdf
abundance
230.19
13.25
12.68
37.54
7.9134
7.8069
7.8000
7.6912
7.6752
7.6649
7.6488
2.0924
X : parts per Million : 1H

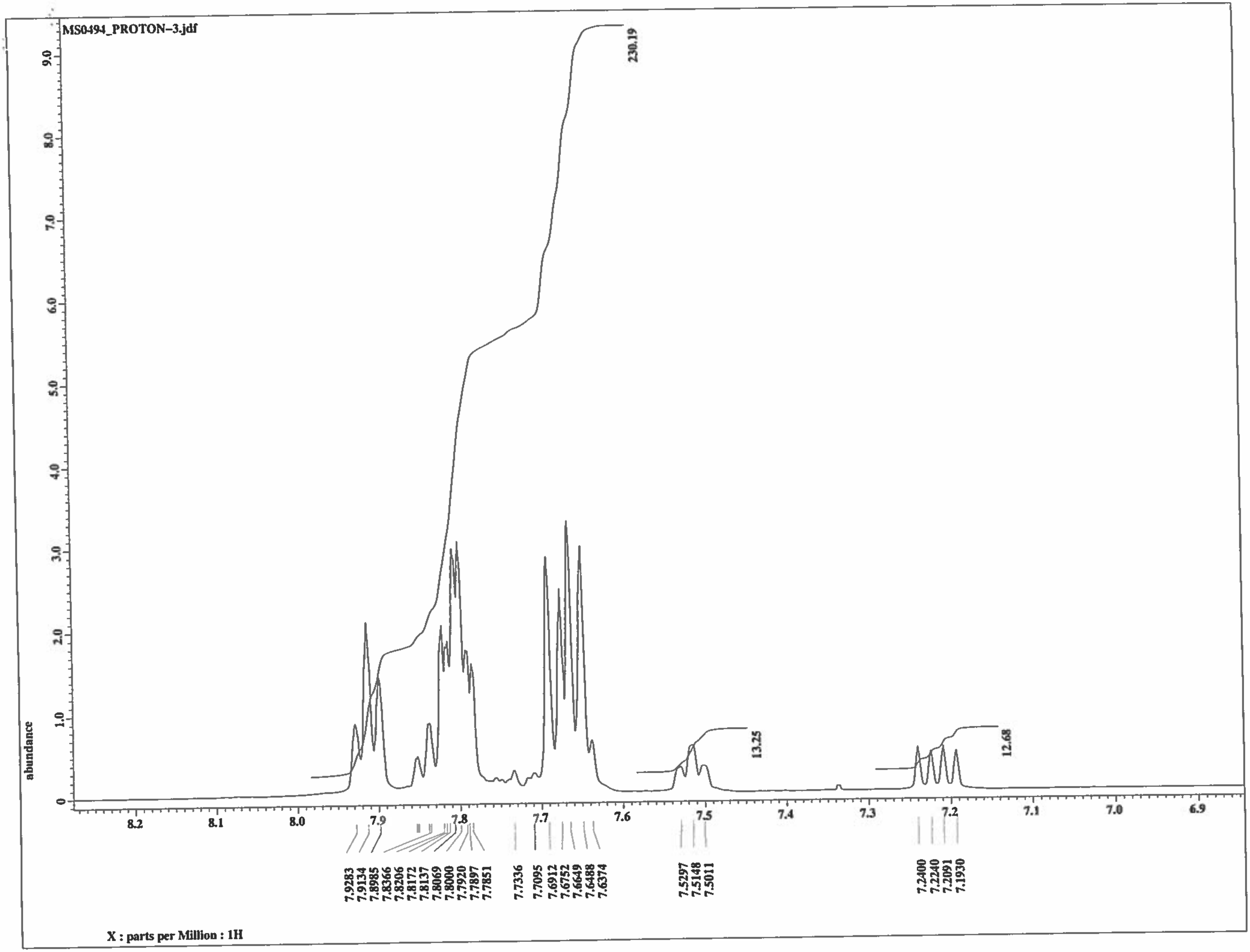
MS0494_PROTON-3.jdf
abundance
0
1.0
2.0
3.0
4.0
5.0
6.0
7.0
8.0
9.0
230.19
13.25
12.68
8.2
8.1
8.0
7.9
7.8
7.7
7.6
7.5
7.4
7.3
7.2
7.1
7.0
6.9
7.9283
7.9134
7.8985
7.8366
7.8206
7.8172
7.8137
7.8069
7.8000
7.7920
7.7897
7.7851
7.7336
7.7095
7.6912
7.6752
7.6649
7.6488
7.6374
7.5297
7.5148
7.5011
7.2400
7.2240
7.2091
7.1930
X : parts per Million : 1H

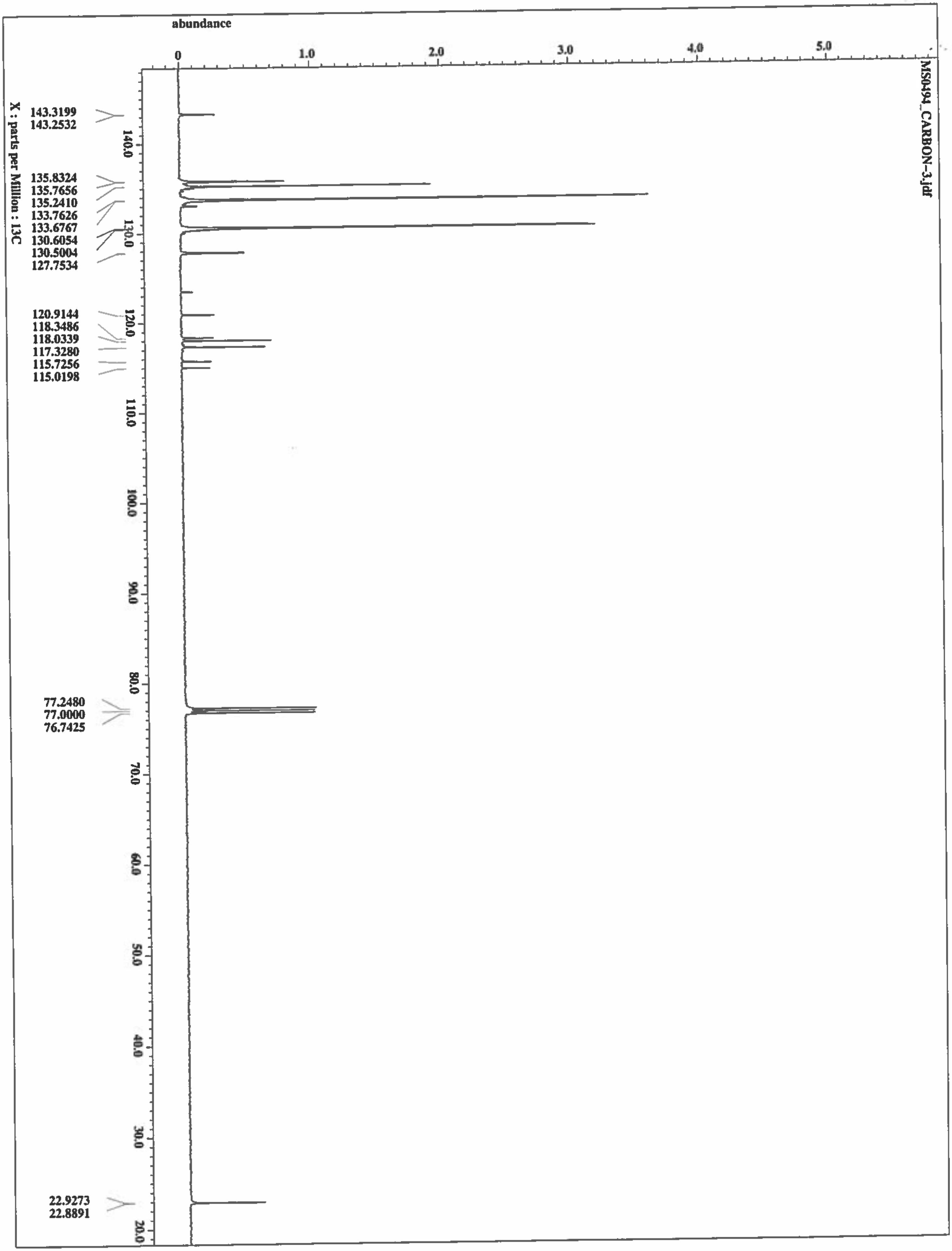

abundance
0
1.0
2.0
3.0
4.0
5.0
MS0494_CARBON-3.jdf
X : parts per Million : 13C
143.3199
143.2532
135.8324
135.7656
135.2410
133.7626
133.6767
130.6054
130.5004
127.7534
120.9144
118.3486
118.0339
117.3280
115.7256
115.0198
77.2480
77.0000
76.7425
22.9273
22.8891
140.0
130.0
120.0
110.0
100.0
90.0
80.0
70.0
60.0
50.0
40.0
30.0
20.0

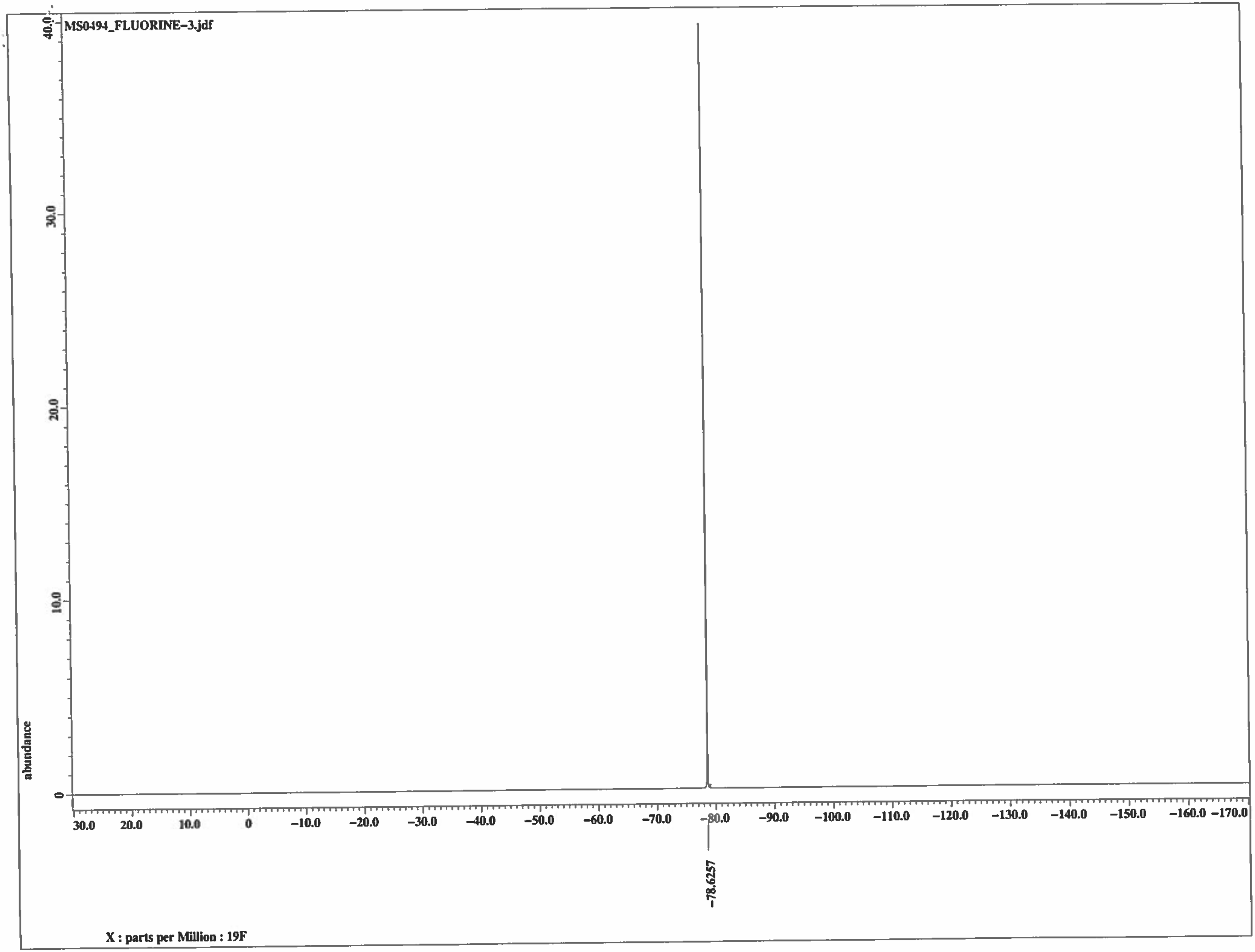

MS0494_FLUORINE-3.jdf
abundance
0
10.0
20.0
30.0
40.0
30.0
20.0
10.0
0
−10.0
−20.0
−30.0
−40.0
−50.0
−60.0
−70.0
−80.0
−90.0
−100.0
−110.0
−120.0
−130.0
−140.0
−150.0
−160.0
−170.0
−78.6257
X : parts per Million : 19F

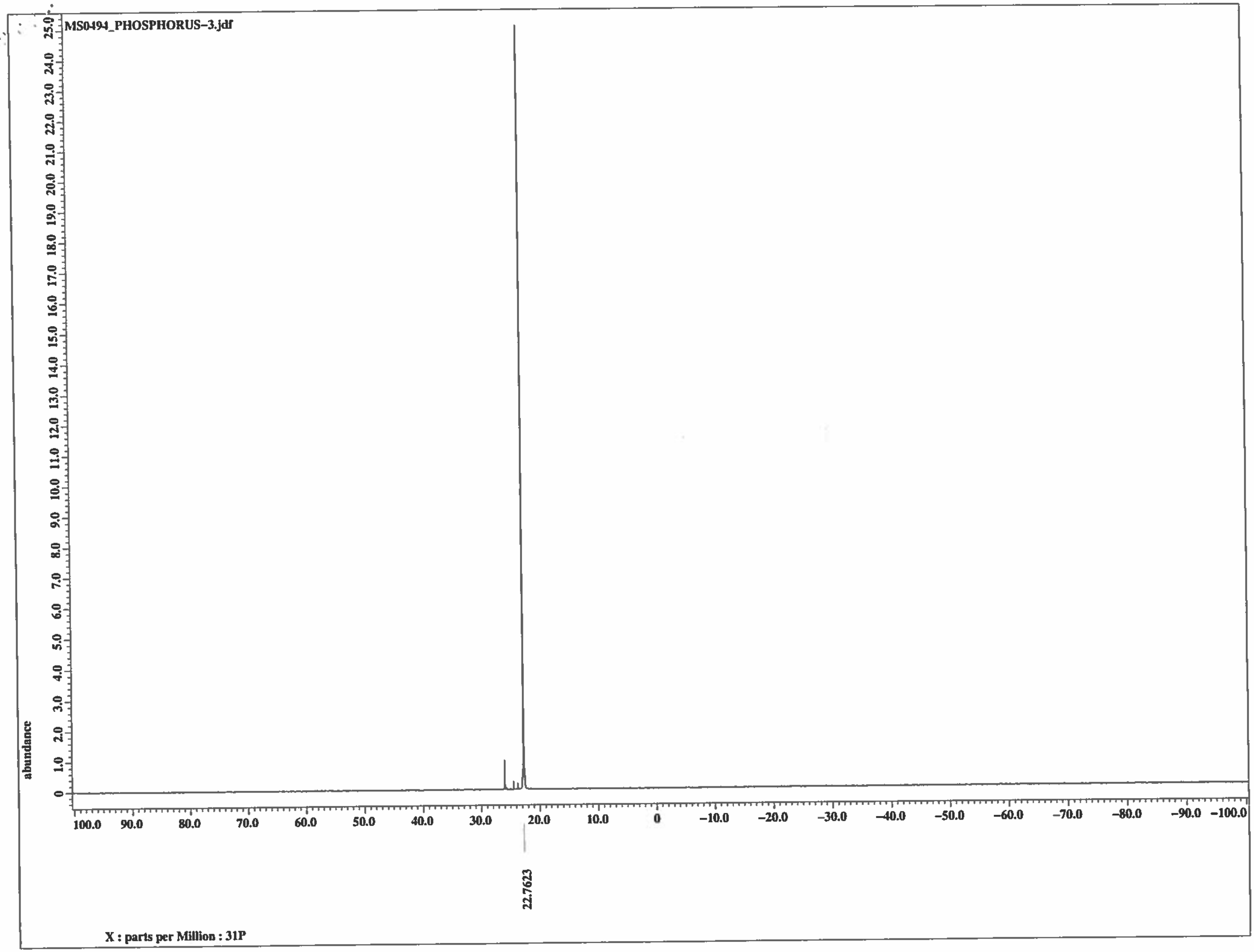

MS0494_PHOSPHORUS-3.jdf
abundance
0 1.0 2.0 3.0 4.0 5.0 6.0 7.0 8.0 9.0 10.0 11.0 12.0 13.0 14.0 15.0 16.0 17.0 18.0 19.0 20.0 21.0 22.0 23.0 24.0 25.0
100.0 90.0 80.0 70.0 60.0 50.0 40.0 30.0 20.0 10.0 0 −10.0 −20.0 −30.0 −40.0 −50.0 −60.0 −70.0 −80.0 −90.0 −100.0
22.7623
X : parts per Million : 31P

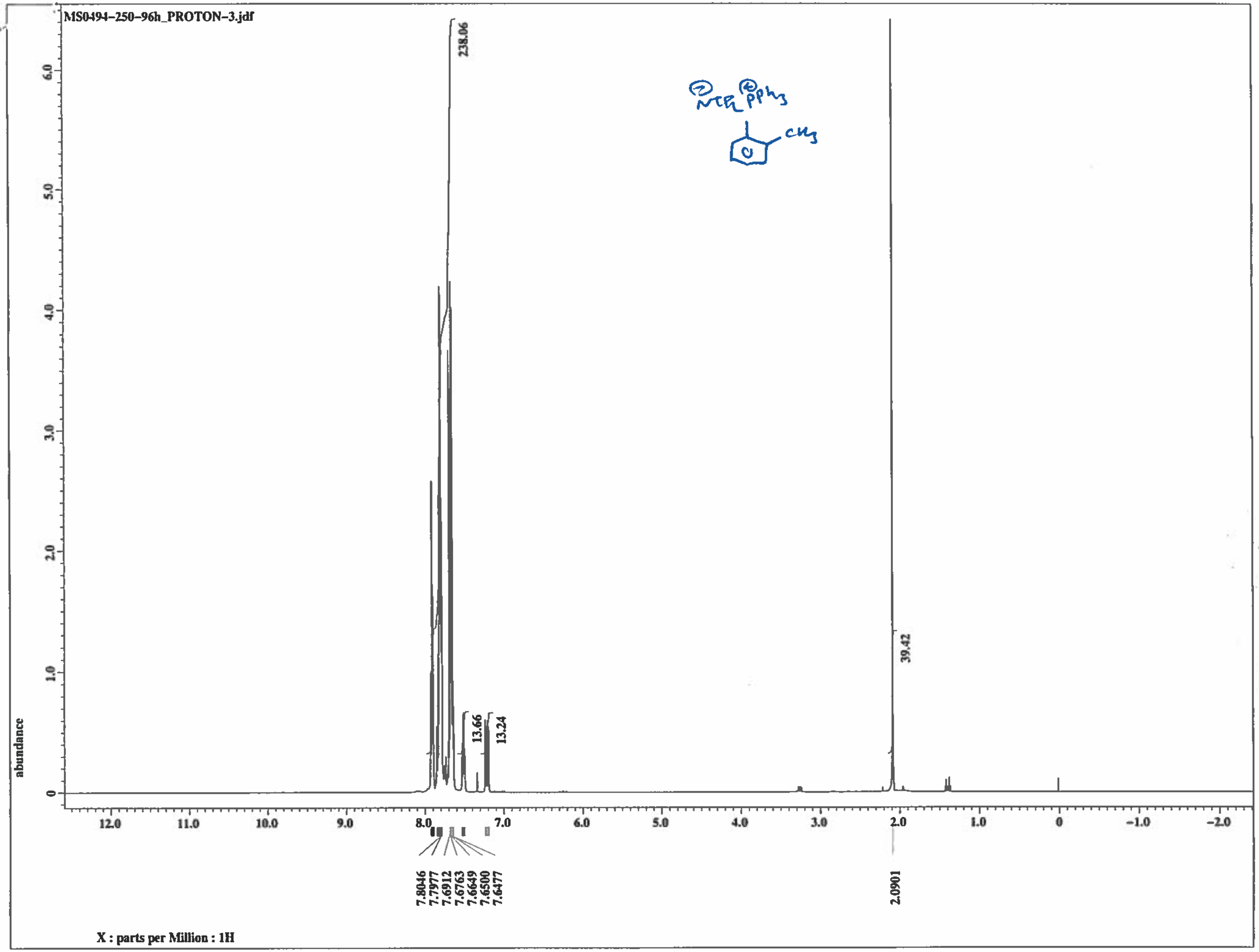

MS0494-250-96h_PROTON-3.jdf
abundance
238.06
13.66
13.24
39.42
7.8046
7.7977
7.6912
7.6763
7.6649
7.6500
7.6477
2.0901
X : parts per Million : 1H

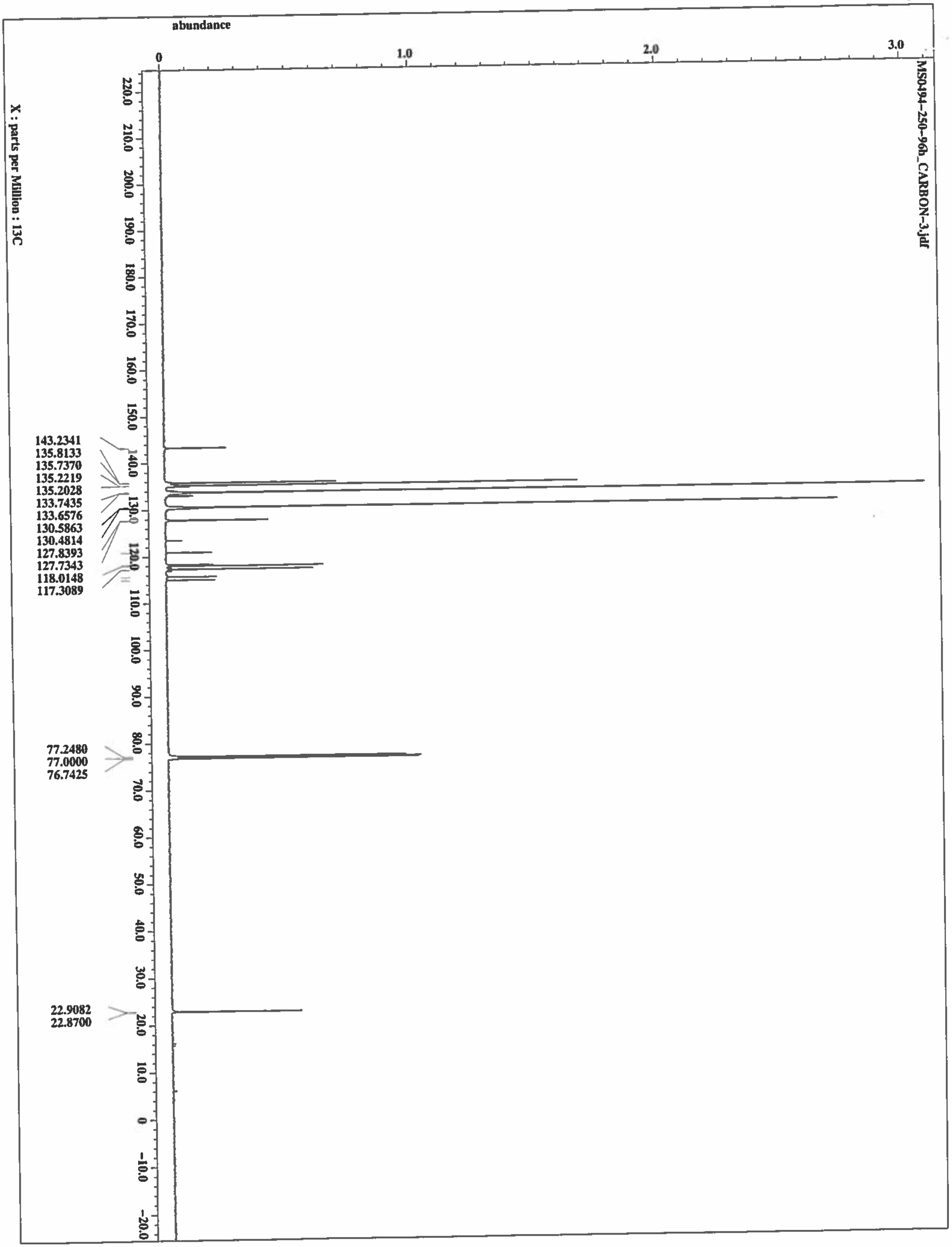

abundance
0
1.0
2.0
3.0
MS0494-250-96h_CARBON-3.jdf
X : parts per Million : 13C
220.0
210.0
200.0
190.0
180.0
170.0
160.0
150.0
140.0
130.0
120.0
110.0
100.0
90.0
80.0
70.0
60.0
50.0
40.0
30.0
20.0
10.0
0
-10.0
-20.0
143.2341
135.8133
135.7370
135.2219
135.2028
133.7435
133.6576
130.5863
130.4814
127.8393
127.7343
118.0148
117.3089
77.2480
77.0000
76.7425
22.9082
22.8700

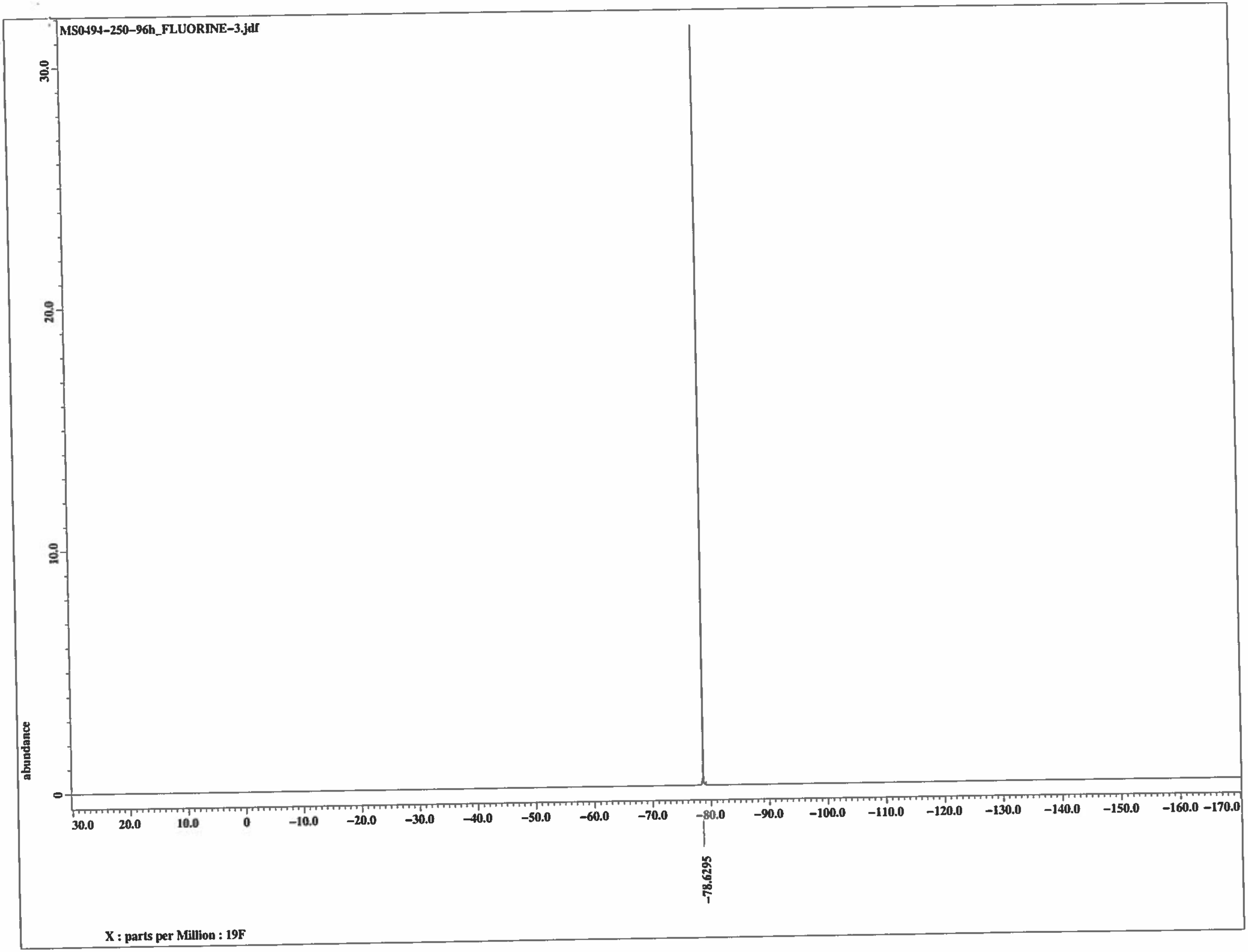
MS0494-250-96h_FLUORINE-3.jdf
abundance
30.0
20.0
10.0
0
30.0 20.0 10.0 0 -10.0 -20.0 -30.0 -40.0 -50.0 -60.0 -70.0 -80.0 -90.0 -100.0 -110.0 -120.0 -130.0 -140.0 -150.0 -160.0 -170.0
-78.6295
X : parts per Million : 19F

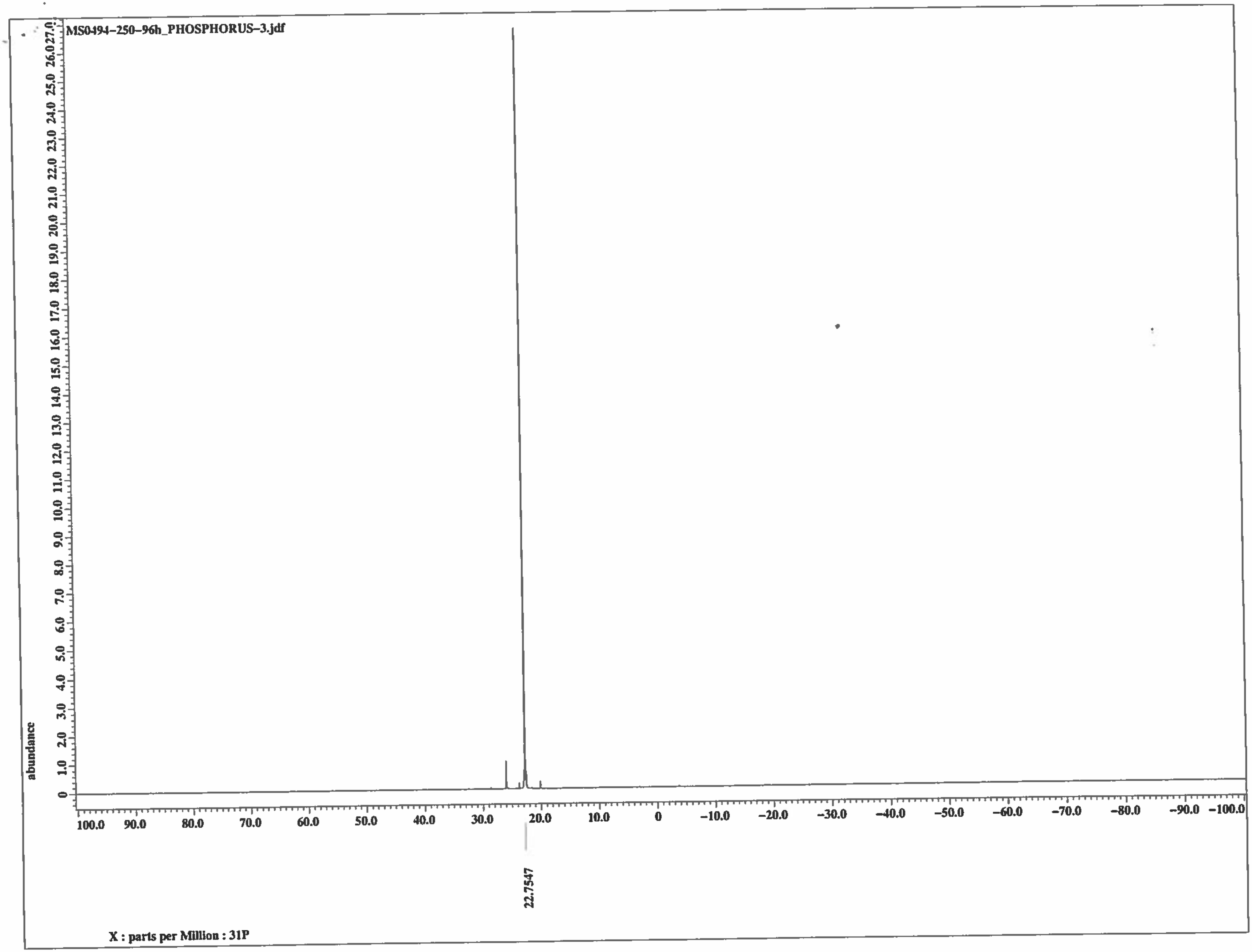
MS0494-250-96h_PHOSPHORUS-3.jdf
abundance
0
1.0
2.0
3.0
4.0
5.0
6.0
7.0
8.0
9.0
10.0
11.0
12.0
13.0
14.0
15.0
16.0
17.0
18.0
19.0
20.0
21.0
22.0
23.0
24.0
25.0
26.0
27.0
100.0
90.0
80.0
70.0
60.0
50.0
40.0
30.0
20.0
10.0
0
-10.0
-20.0
-30.0
-40.0
-50.0
-60.0
-70.0
-80.0
-90.0
-100.0
22.7547
X : parts per Million : 31P

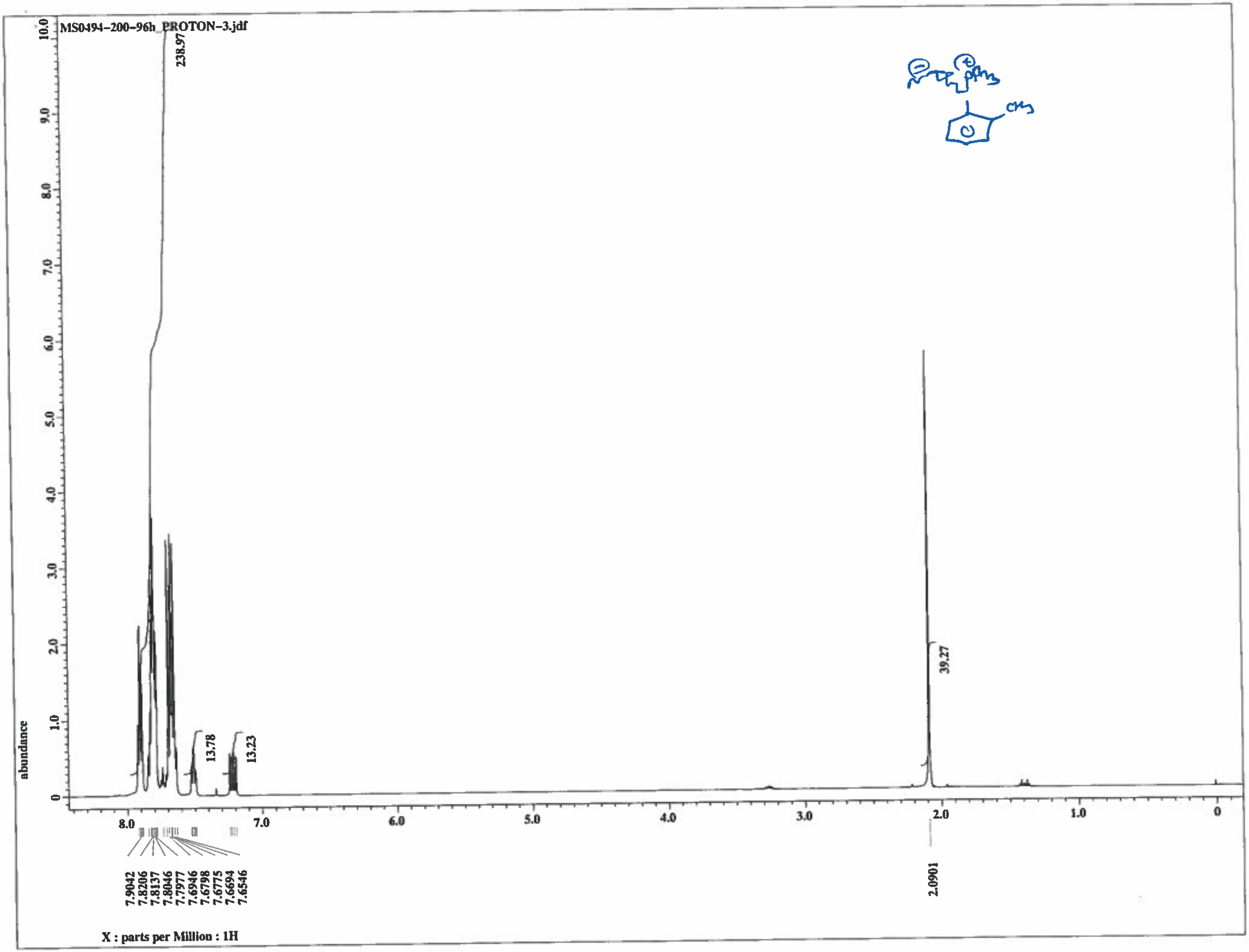
MS0494-200-96h_PROTON-3.jdf
abundance
238.97
13.78
13.23
39.27
7.9042
7.8206
7.8137
7.8046
7.7977
7.6946
7.6798
7.6775
7.6694
7.6546
2.0901
X : parts per Million : 1H

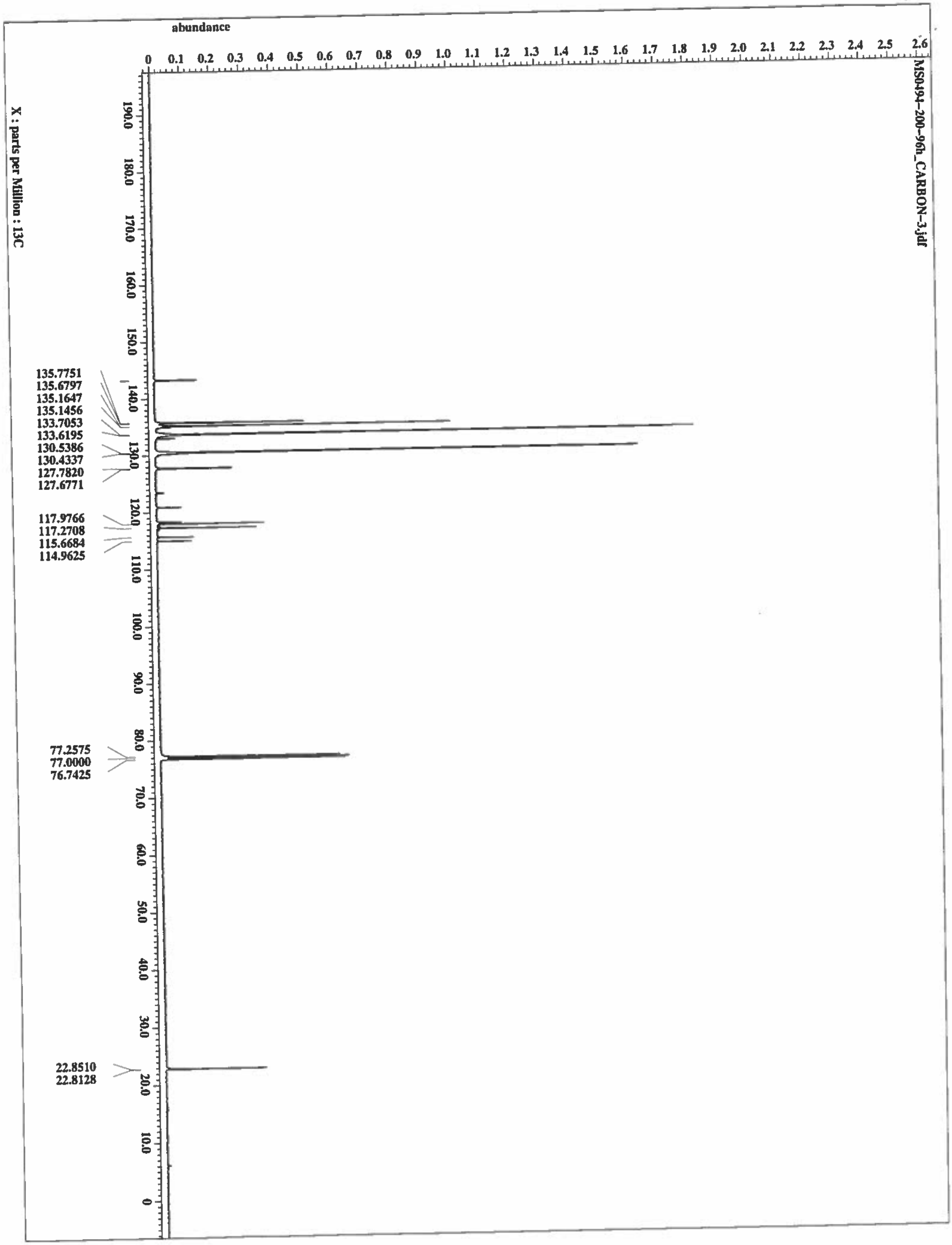

abundance
0 0.1 0.2 0.3 0.4 0.5 0.6 0.7 0.8 0.9 1.0 1.1 1.2 1.3 1.4 1.5 1.6 1.7 1.8 1.9 2.0 2.1 2.2 2.3 2.4 2.5 2.6
MS0494-200-96h_CARBON-3.jdf
X : parts per Million : 13C
190.0 180.0 170.0 160.0 150.0 140.0 130.0 120.0 110.0 100.0 90.0 80.0 70.0 60.0 50.0 40.0 30.0 20.0 10.0 0
135.7751
135.6797
135.1647
135.1456
133.7053
133.6195
130.5386
130.4337
127.7820
127.6771
117.9766
117.2708
115.6684
114.9625
77.2575
77.0000
76.7425
22.8510
22.8128

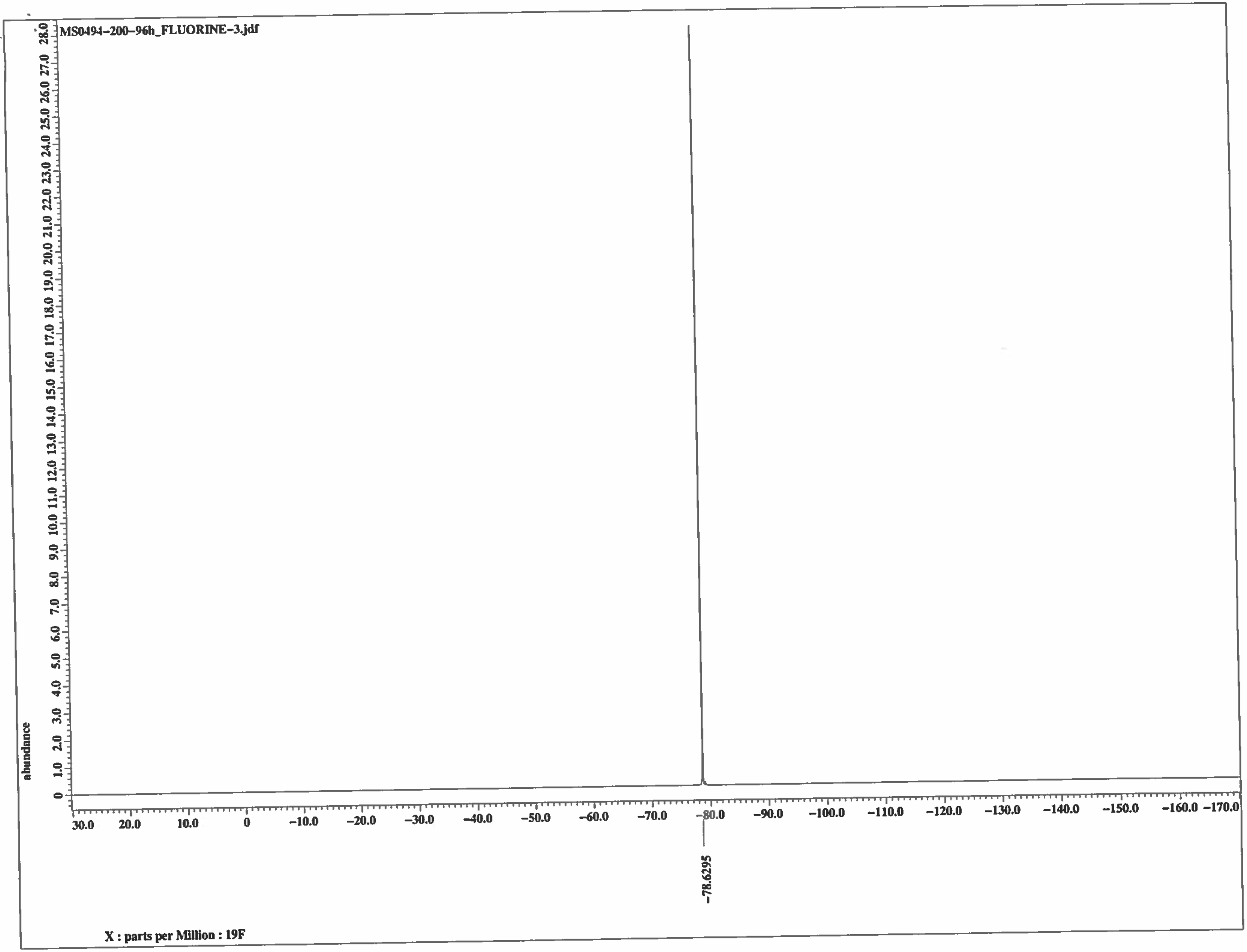
MS0494-200-96h_FLUORINE-3.jdf
abundance
0
1.0
2.0
3.0
4.0
5.0
6.0
7.0
8.0
9.0
10.0
11.0
12.0
13.0
14.0
15.0
16.0
17.0
18.0
19.0
20.0
21.0
22.0
23.0
24.0
25.0
26.0
27.0
28.0
30.0
20.0
10.0
0
−10.0
−20.0
−30.0
−40.0
−50.0
−60.0
−70.0
−80.0
−90.0
−100.0
−110.0
−120.0
−130.0
−140.0
−150.0
−160.0
−170.0
−78.6295
X : parts per Million : 19F

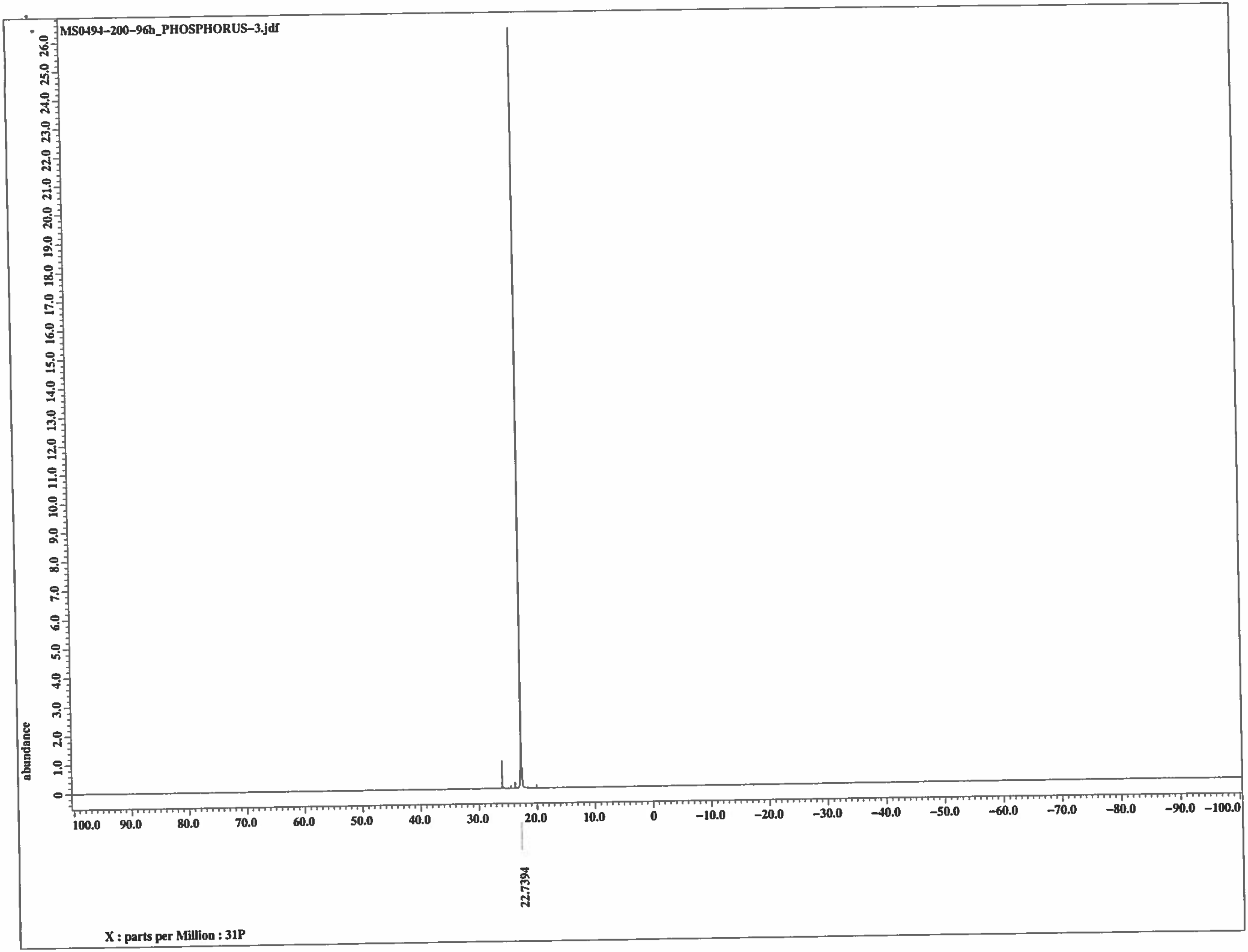
MS0494-200-96h_PHOSPHORUS-3.jdf
abundance
0
1.0
2.0
3.0
4.0
5.0
6.0
7.0
8.0
9.0
10.0
11.0
12.0
13.0
14.0
15.0
16.0
17.0
18.0
19.0
20.0
21.0
22.0
23.0
24.0
25.0
26.0
100.0
90.0
80.0
70.0
60.0
50.0
40.0
30.0
20.0
10.0
0
−10.0
−20.0
−30.0
−40.0
−50.0
−60.0
−70.0
−80.0
−90.0
−100.0
22.7394
X : parts per Million : 31P

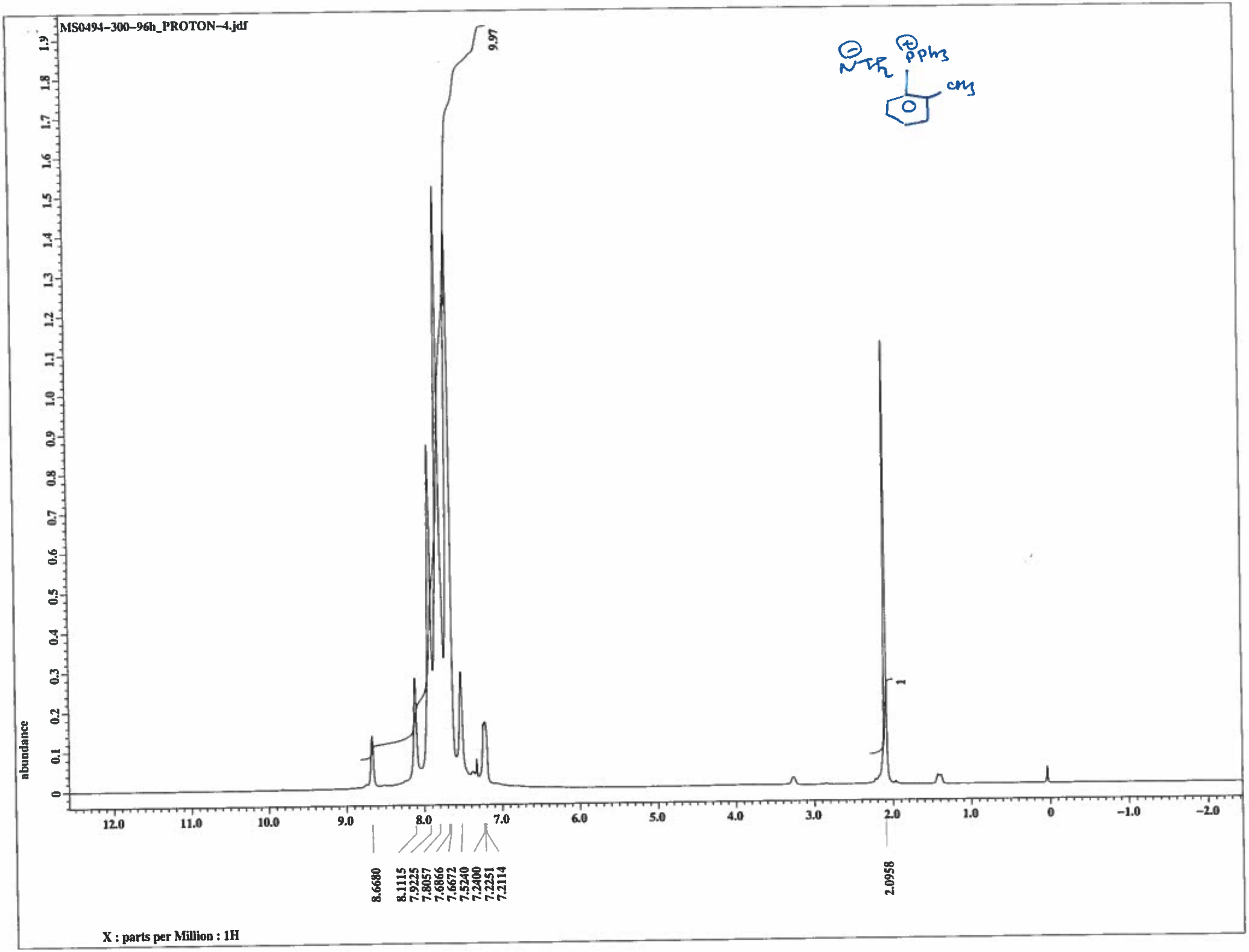

MS0494-300-96h_PROTON-4.jdf
abundance
0
0.1
0.2
0.3
0.4
0.5
0.6
0.7
0.8
0.9
1.0
1.1
1.2
1.3
1.4
1.5
1.6
1.7
1.8
1.9
9.97
1
12.0
11.0
10.0
9.0
8.0
7.0
6.0
5.0
4.0
3.0
2.0
1.0
0
-1.0
-2.0
8.6680
8.1115
7.9225
7.8057
7.6866
7.6672
7.5240
7.2400
7.2251
7.2114
2.0958
X : parts per Million : 1H

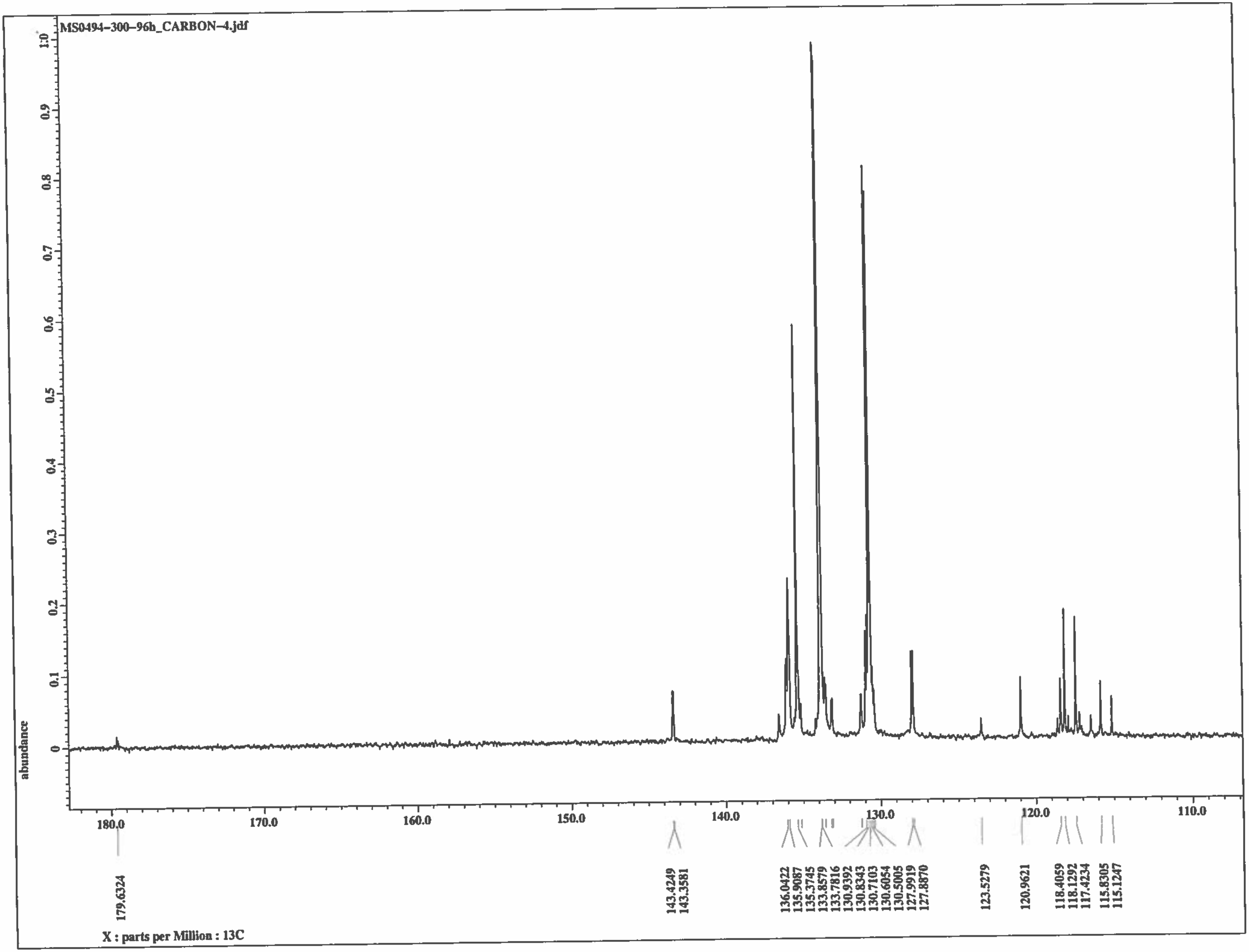

MS0494-300-96b_CARBON-4.jdf
abundance
0
0.1
0.2
0.3
0.4
0.5
0.6
0.7
0.8
0.9
1.0
180.0
170.0
160.0
150.0
140.0
130.0
120.0
110.0
179.6324
143.4249
143.3581
136.0422
135.9087
135.3745
133.8579
133.7816
130.9392
130.8343
130.7103
130.6054
130.5005
127.9919
127.8870
123.5279
120.9621
118.4059
118.1292
117.4234
115.8305
115.1247
X : parts per Million : 13C

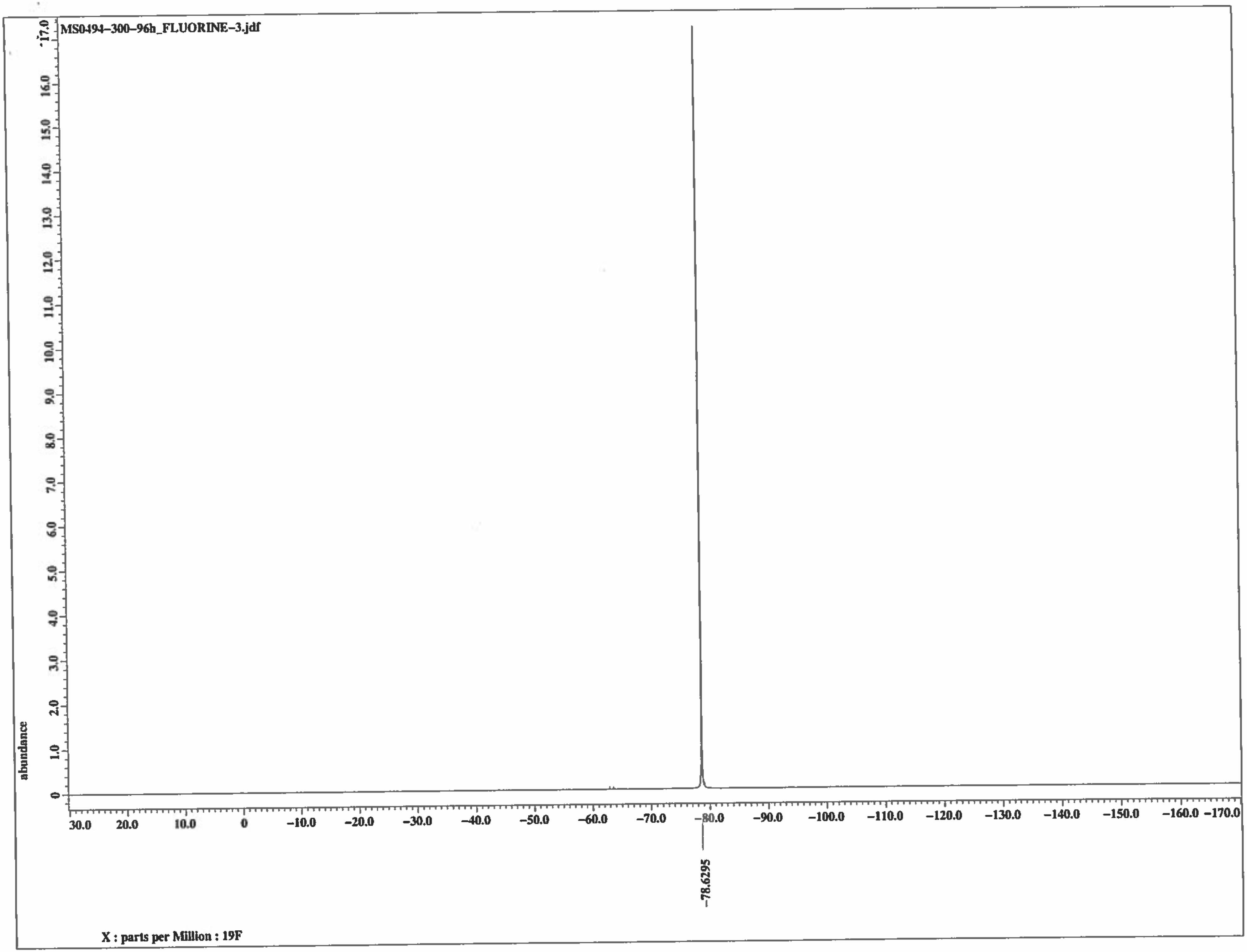
MS0494-300-96h_FLUORINE-3.jdf
abundance
0
1.0
2.0
3.0
4.0
5.0
6.0
7.0
8.0
9.0
10.0
11.0
12.0
13.0
14.0
15.0
16.0
17.0
30.0
20.0
10.0
0
-10.0
-20.0
-30.0
-40.0
-50.0
-60.0
-70.0
-80.0
-90.0
-100.0
-110.0
-120.0
-130.0
-140.0
-150.0
-160.0
-170.0
-78.6295
X : parts per Million : 19F

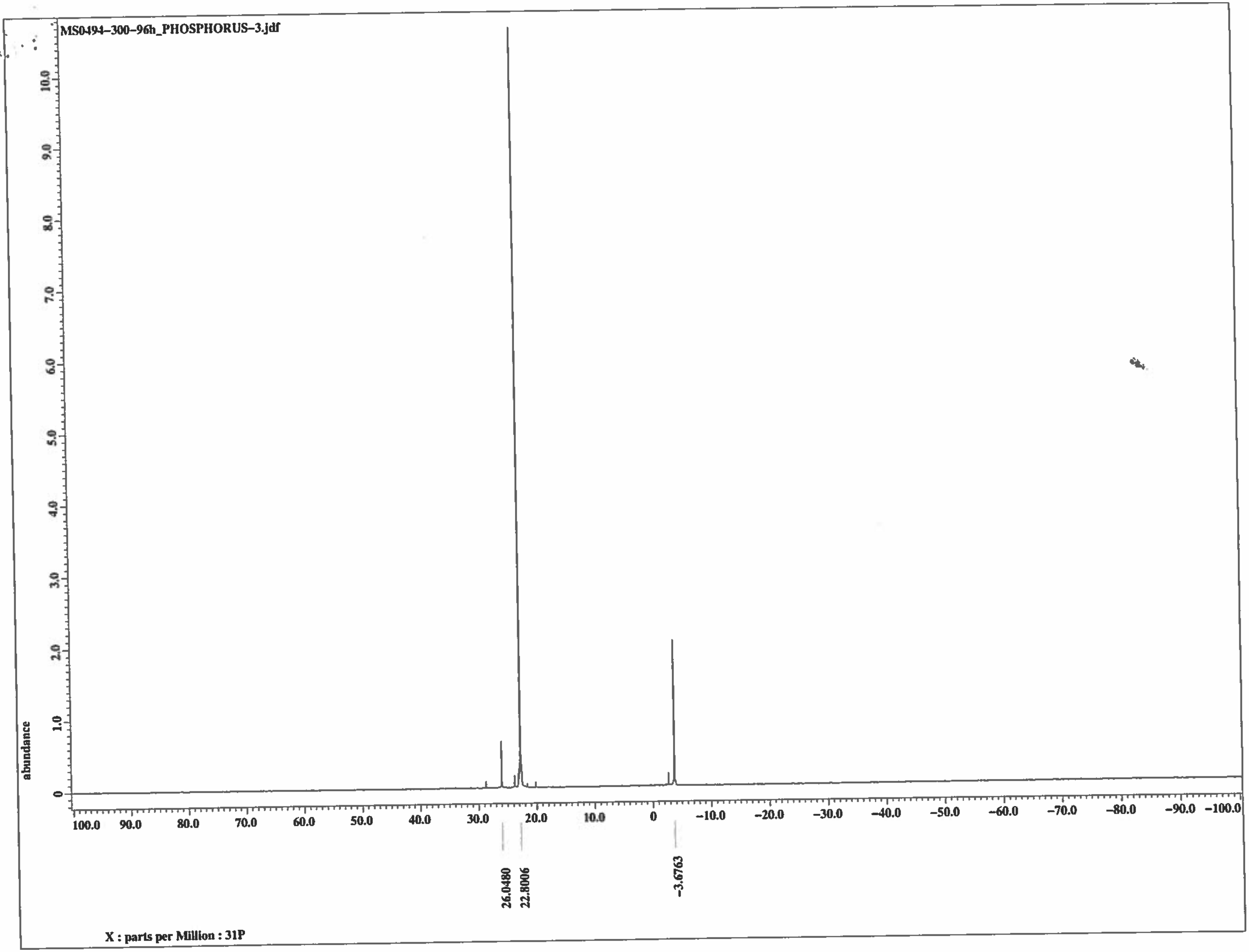

MS0494-300-96h_PHOSPHORUS-3.jdf
abundance
0
1.0
2.0
3.0
4.0
5.0
6.0
7.0
8.0
9.0
10.0
100.0
90.0
80.0
70.0
60.0
50.0
40.0
30.0
20.0
10.0
0
-10.0
-20.0
-30.0
-40.0
-50.0
-60.0
-70.0
-80.0
-90.0
-100.0
26.0480
22.8006
-3.6763
X : parts per Million : 31P

Compound 11 Pre- and Post-heating NMR Spectra

Temperature of Post-heating samples noted in upper left corner of each spectrum

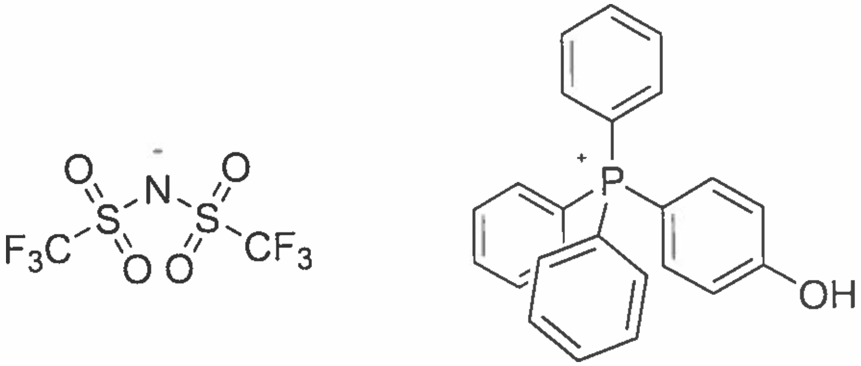

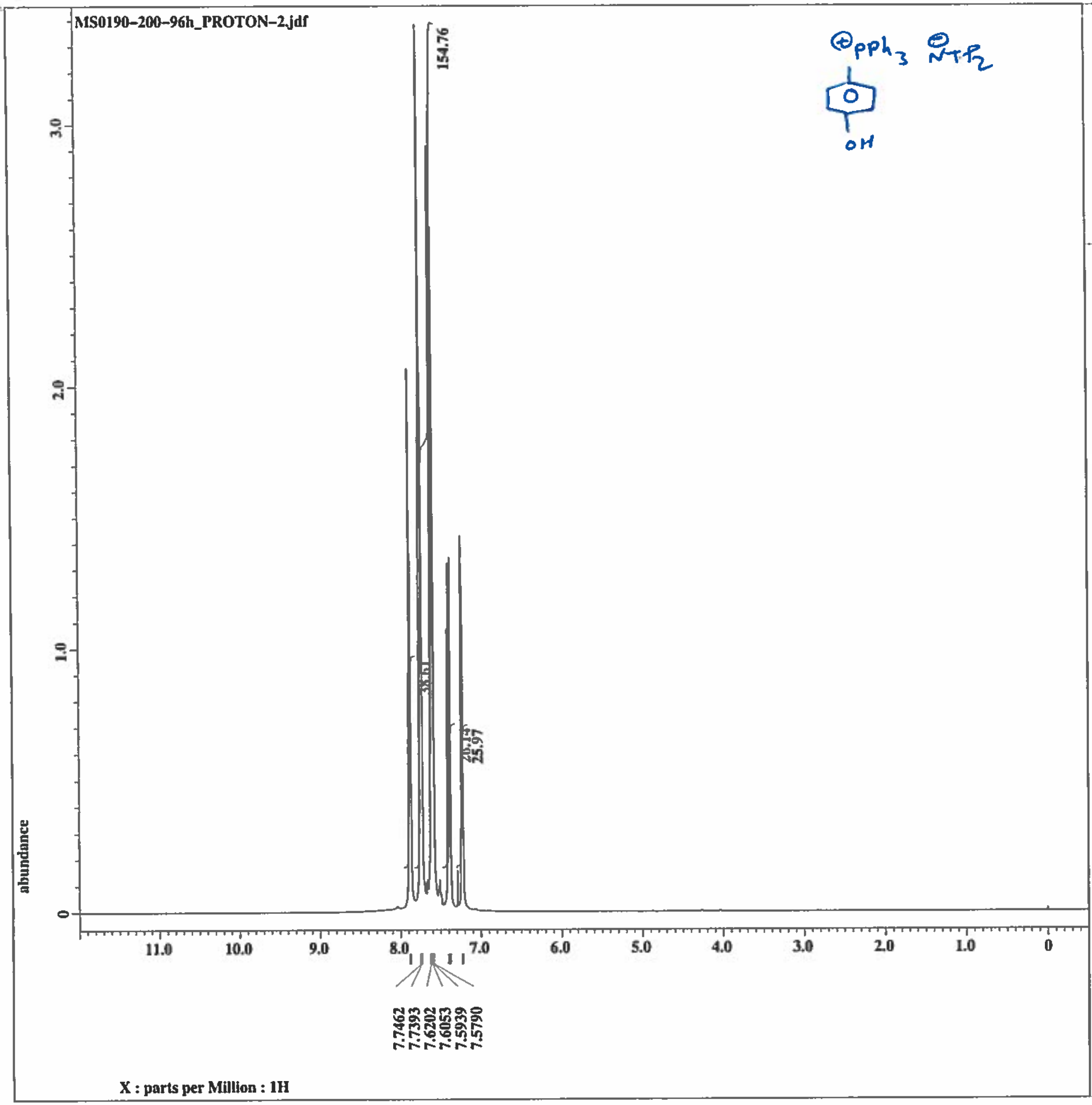

Filename	= MS0190-200-96h_PROTON
Author	= Jim Davis
Experiment	= single_pulse.ex2
Sample_id	= MS0190-200-96h
Solvent	= CHLOROFORM-D
Creation_time	= 6-JAN-2019 14:41:07
Revision_time	= 6-JAN-2019 14:15:56
Current_time	= 6-JAN-2019 14:15:56
Data_format	= 1D COMPLEX
Dim_size	= 13107
Dim_title	= 1H
Dim_units	= [ppm]
Dimensions	= X
Site	= ECA 500
Spectrometer	= JNM-ECA500
Field_strength	= 11.7473579[T] (500[MH
X_acq_duration	= 1.74587904[s]
X_domain	= 1H
X_freq	= 500.15991521[MHz]
X_offset	= 5.0[ppm]
X_points	= 16384
X_prescans	= 1
X_resolution	= 0.57277737[Hz]
X_sweep	= 9.38438438[kHz]
Irr_domain	= 1H
Irr_freq	= 500.15991521[MHz]
Irr_offset	= 5.0[ppm]
Tri_domain	= 1H
Tri_freq	= 500.15991521[MHz]
Tri_offset	= 5.0[ppm]
Clipped	= FALSE
Mod_return	= 1
Scans	= 16
Total_scans	= 16
X_90_width	= 12.4[us]
X_acq_time	= 1.74587904[s]
X_angle	= 45[deg]
X_atn	= 4[dB]
X_pulse	= 6.2[us]
Irr_mode	= Off
Tri_mode	= Off
Dante_presat	= FALSE
Initial_wait	= 1[s]
Recvr_gain	= 24
Relaxation_delay	= 4[s]
Repetition_time	= 5.74587904[s]
Temp_get	= 19.9[dC]

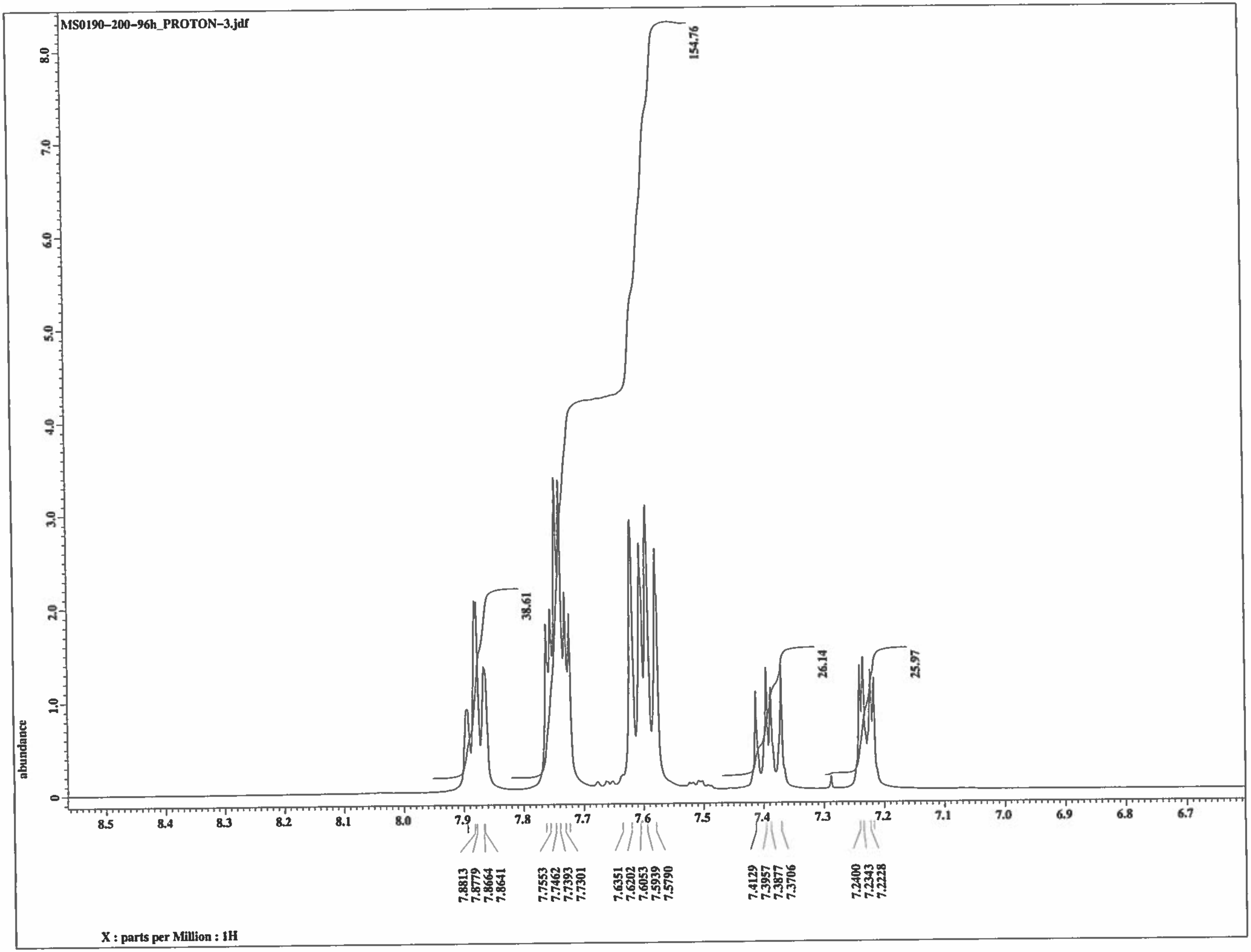
MS0190-200-96h_PROTON-3.jdf
abundance
0
1.0
2.0
3.0
4.0
5.0
6.0
7.0
8.0
154.76
38.61
26.14
25.97
8.5
8.4
8.3
8.2
8.1
8.0
7.9
7.8
7.7
7.6
7.5
7.4
7.3
7.2
7.1
7.0
6.9
6.8
6.7
7.8813
7.8779
7.8664
7.8641
7.7553
7.7462
7.7393
7.7301
7.6351
7.6202
7.6053
7.5939
7.5790
7.4129
7.3957
7.3877
7.3706
7.2400
7.2343
7.2228
X : parts per Million : 1H

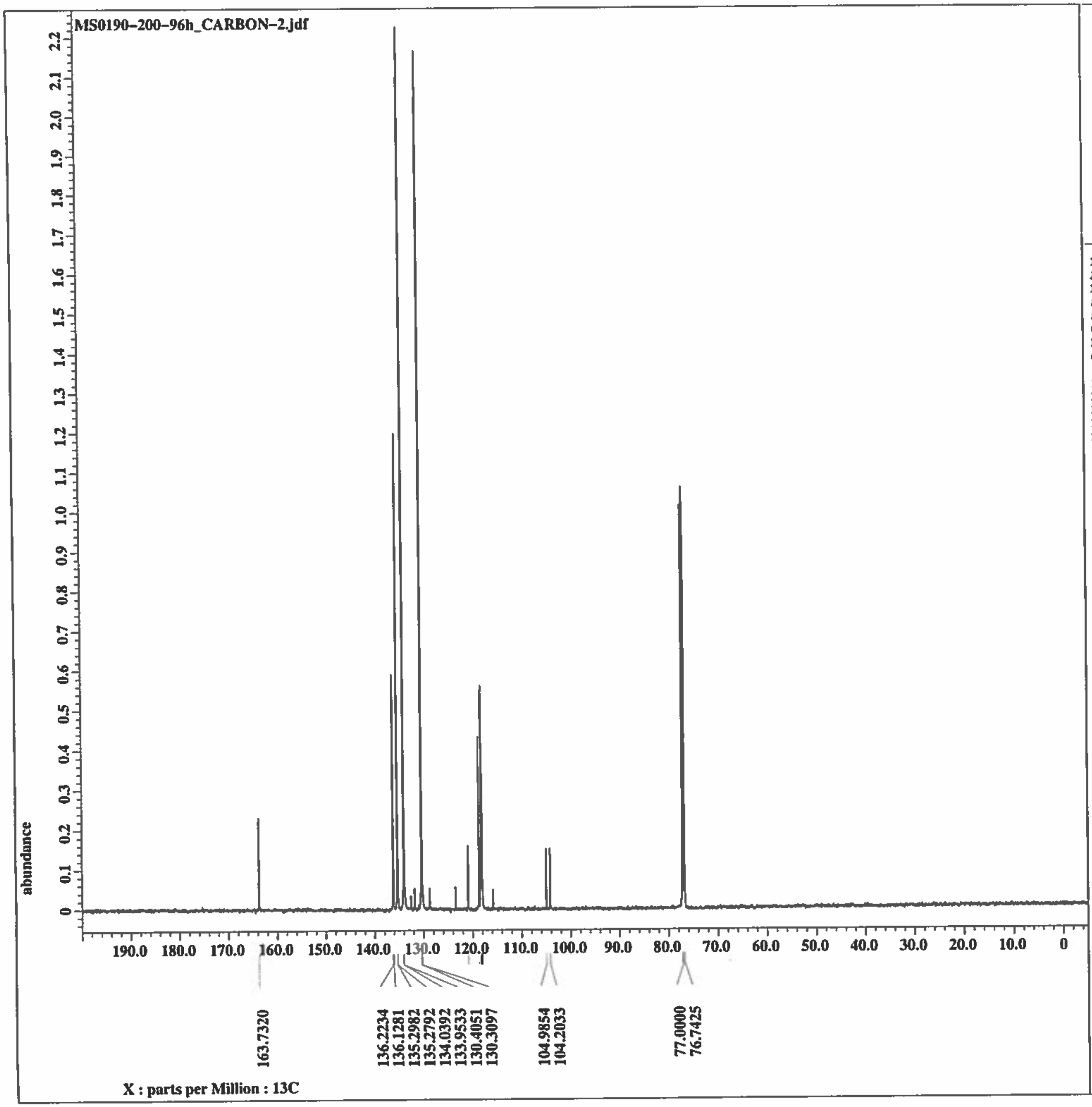

Filename	= MS0190-200-96h_CARBON
Author	= Jim Davis
Experiment	= single_pulse_dec
Sample_id	= MS0190-200-96h
Solvent	= CHLOROFORM-D
Creation_time	= 6-JAN-2019 15:11:58
Revision_time	= 6-JAN-2019 14:46:47
Current_time	= 6-JAN-2019 14:46:47
Data_format	= 1D COMPLEX
Dim_size	= 26214
Dim_title	= 13C
Dim_units	= [ppm]
Dimensions	= X
Site	= ECA 500
Spectrometer	= JNM-ECA500
Field_strength	= 11.7473579[T] (500[MH
X_acq_duration	= 0.83361792[s]
X_domain	= 13C
X_freq	= 125.76529768[MHz]
X_offset	= 100[ppm]
X_points	= 32768
X_prescans	= 4
X_resolution	= 1.19959034[Hz]
X_sweep	= 39.3081761[kHz]
Irr_domain	= 1H
Irr_freq	= 500.15991521[MHz]
Irr_offset	= 5.0[ppm]
Clipped	= FALSE
Mod_return	= 1
Scans	= 600
Total_scans	= 600
X_90_width	= 13.2[us]
X_acq_time	= 0.83361792[s]
X_angle	= 30[deg]
X_atn	= 6[dB]
X_pulse	= 4.4[us]
Irr_atn_dec	= 20.7[dB]
Irr_atn_noe	= 20.7[dB]
Irr_noise	= WALTZ
Decoupling	= TRUE
Initial_wait	= 1[s]
Noe	= TRUE
Noe_time	= 2[s]
Recvr_gain	= 60
Relaxation_delay	= 2[s]
Repetition_time	= 2.83361792[s]
Temp_get	= 20.7[dC]

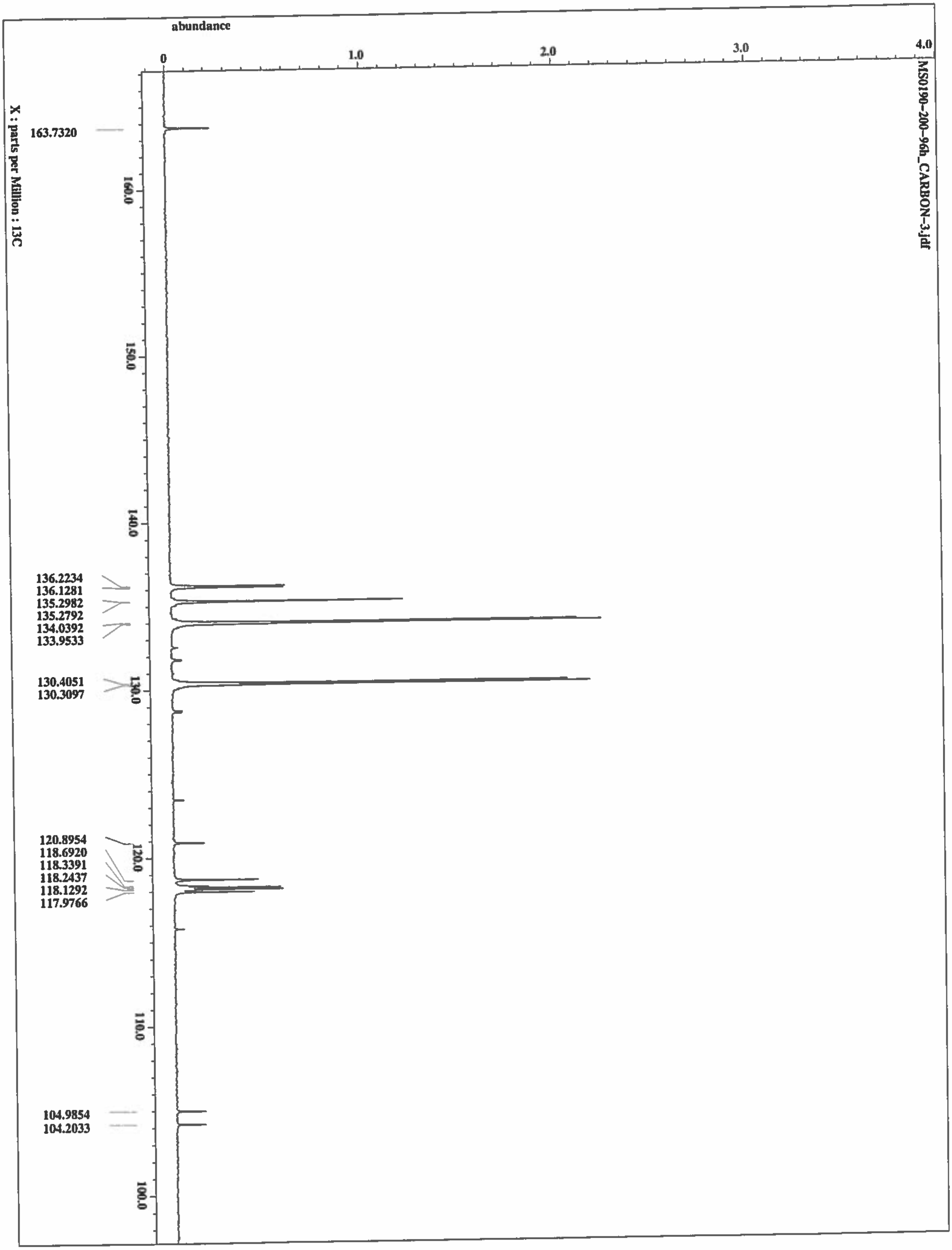
abundance
0
1.0
2.0
3.0
4.0
MS0190-200-96h_CARBON-3.jdf
X : parts per Million : 13C
163.7320
160.0
150.0
140.0
136.2234
136.1281
135.2982
135.2792
134.0392
133.9533
130.4051
130.3097
130.0
120.8954
118.6920
118.3391
118.2437
118.1292
117.9766
120.0
110.0
104.9854
104.2033
100.0

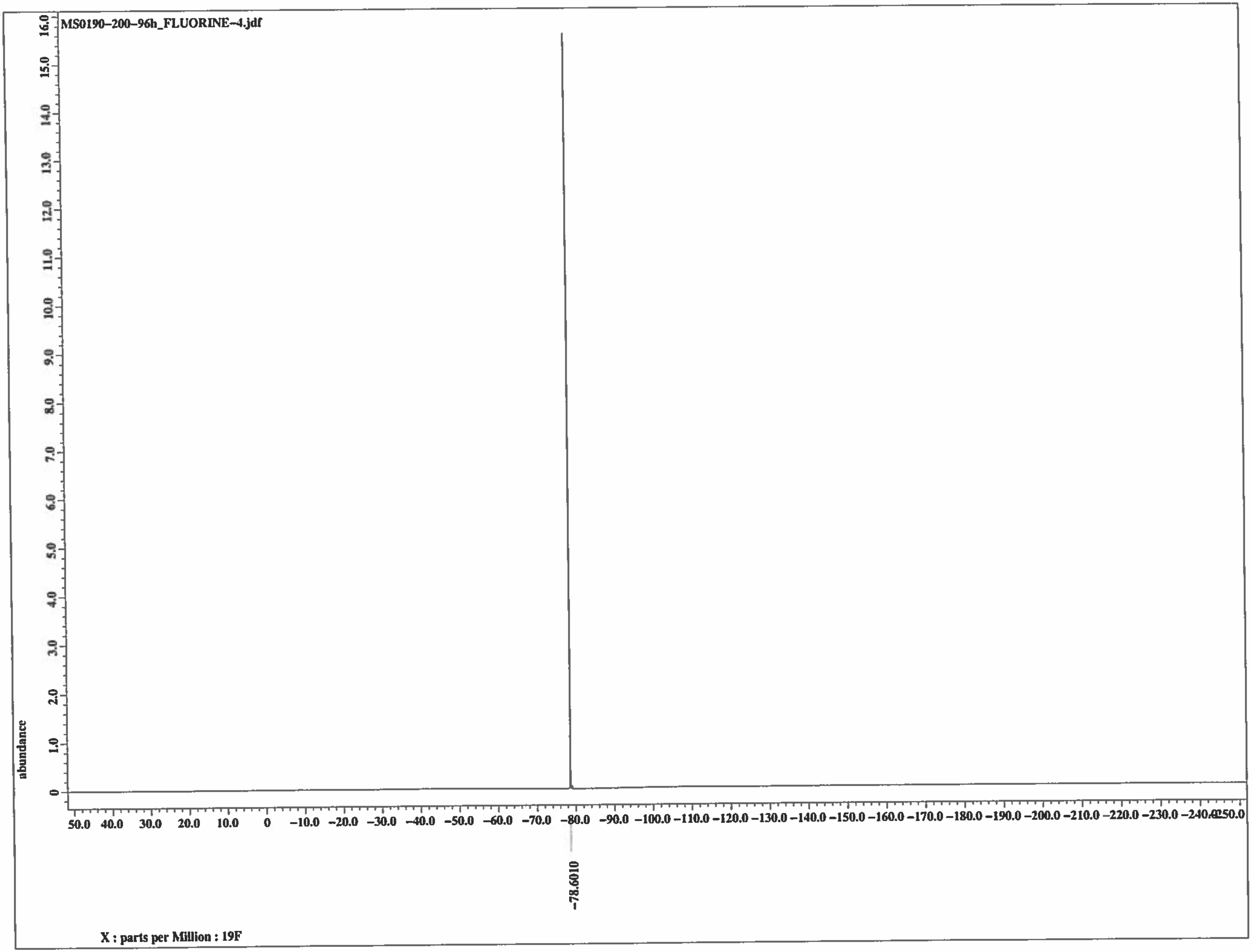

MS0190-200-96h_FLUORINE-4.jdf
abundance
-78.6010
X : parts per Million : 19F

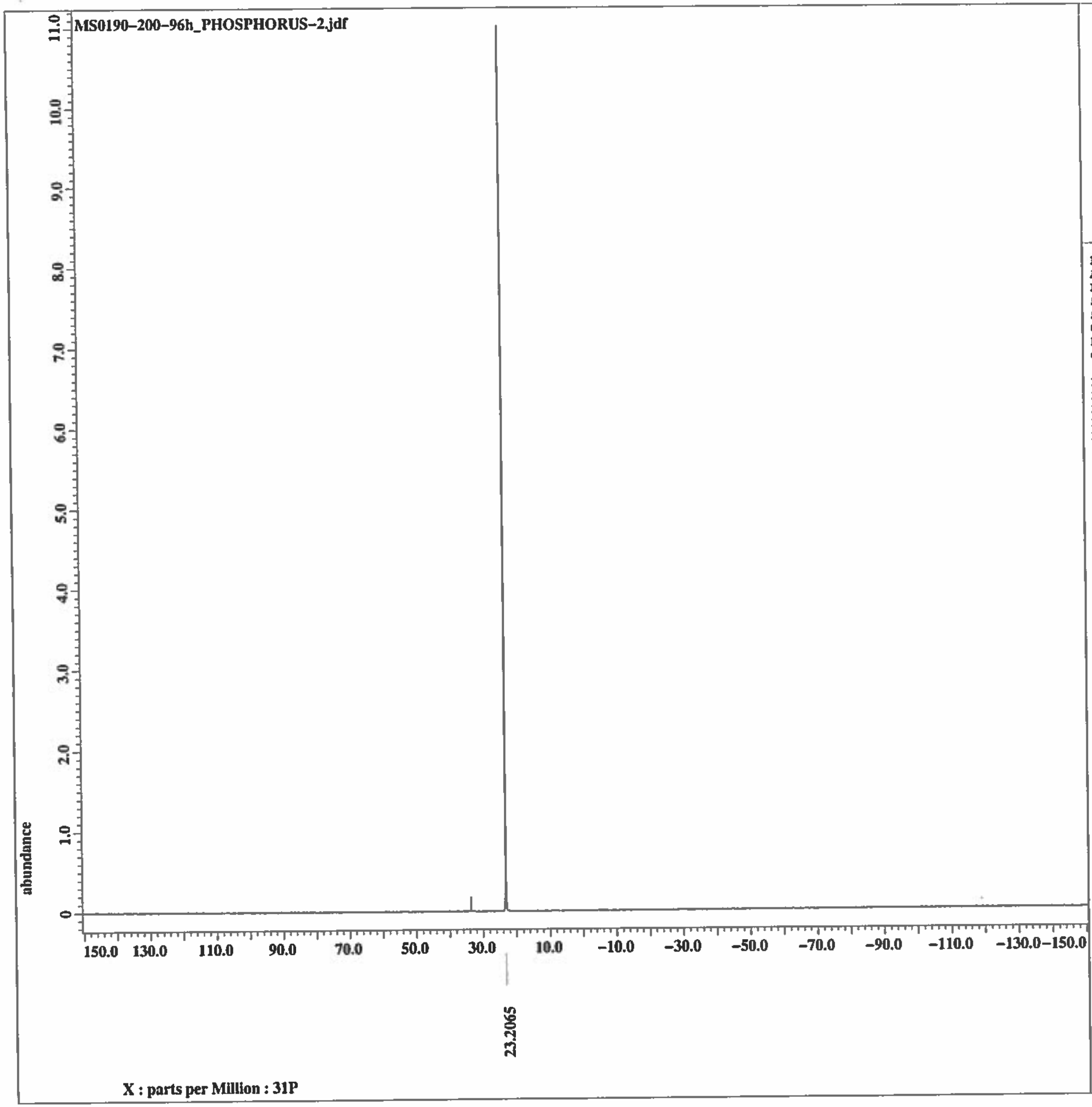

Filename	= MS0190-200-96h_PHOSPH
Author	= Jim Davis
Experiment	= single_pulse_dec
Sample_id	= MS0190-200-96h
Solvent	= CHLOROFORM-D
Creation_time	= 6-JAN-2019 15:33:59
Revision_time	= 6-JAN-2019 15:08:48
Current_time	= 6-JAN-2019 15:08:48
Data_format	= 1D COMPLEX
Dim_size	= 52428
Dim_title	= 31P
Dim_units	= [ppm]
Dimensions	= X
Site	= ECA 500
Spectrometer	= JNM-ECA500
Field_strength	= 11.7473579[T] (500[MH
X_acq_duration	= 0.85983232[s]
X_domain	= 31P
X_freq	= 202.46831075[MHz]
X_offset	= 0[ppm]
X_points	= 65536
X_prescans	= 4
X_resolution	= 1.16301746[Hz]
X_sweep	= 76.2195122[kHz]
Irr_domain	= 1H
Irr_freq	= 500.15991521[MHz]
Irr_offset	= 5.0[ppm]
Clipped	= FALSE
Mod_return	= 1
Scans	= 128
Total_scans	= 128
X_90_width	= 14.687[us]
X_acq_time	= 0.85983232[s]
X_angle	= 30[deg]
X_atn	= 5[dB]
X_pulse	= 4.89566667[us]
Irr_atn_dec	= 20.7[dB]
Irr_atn_noe	= 20.7[dB]
Irr_noise	= WALTZ
Decoupling	= TRUE
Initial_wait	= 1[s]
Noe	= TRUE
Noe_time	= 2[s]
Recvr_gain	= 58
Relaxation_delay	= 2[s]
Repetition_time	= 2.85983232[s]
Temp_get	= 20.4[dC]

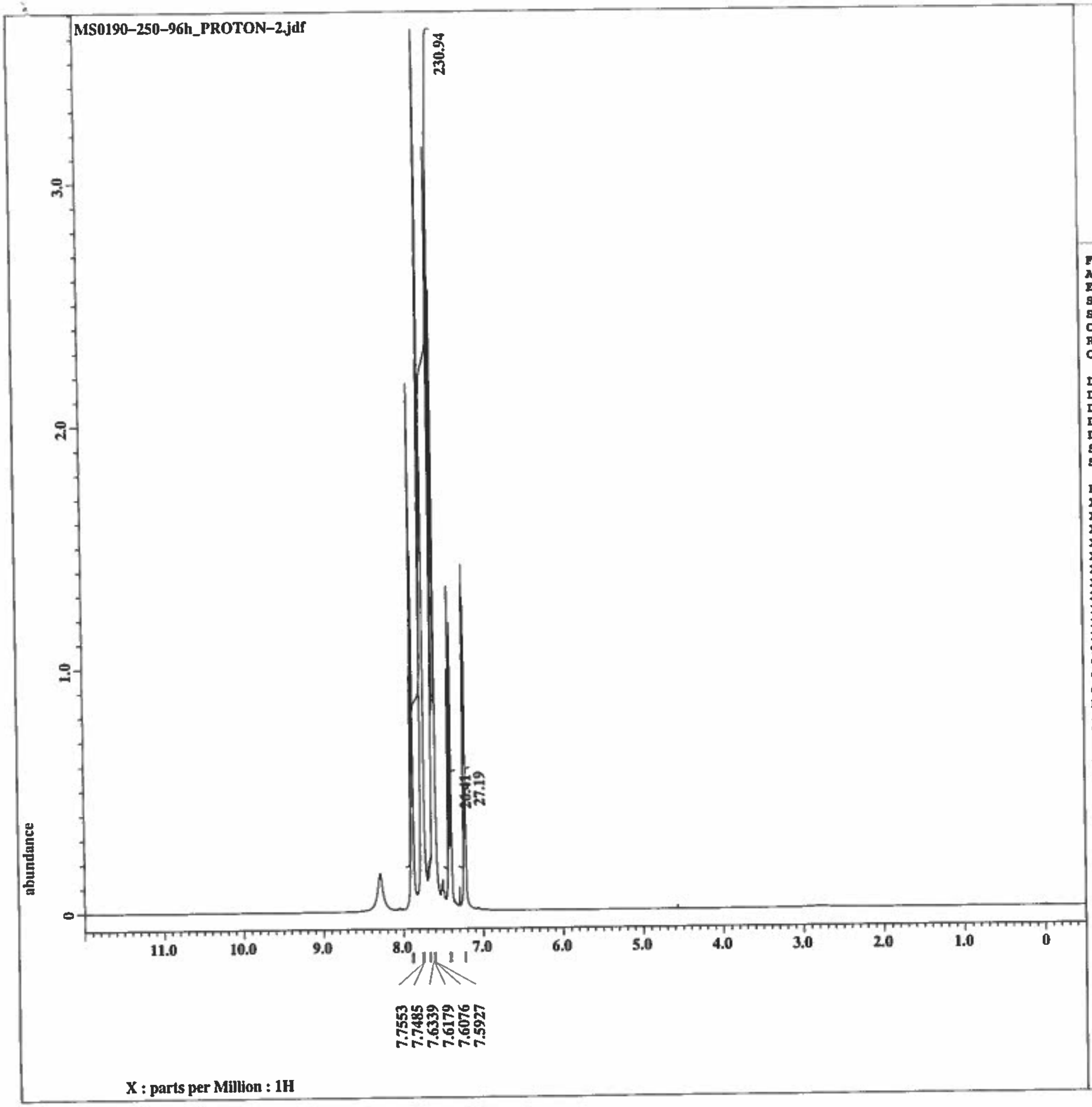

Filename	= MS0190-250-96h_PROTON
Author	= Jim Davis
Experiment	= single_pulse.ex2
Sample_id	= MS0190-250-96h
Solvent	= CHLOROFORM-D
Creation_time	= 6-JAN-2019 15:41:34
Revision_time	= 6-JAN-2019 15:16:23
Current_time	= 6-JAN-2019 15:16:23
Data_format	= 1D COMPLEX
Dim_size	= 13107
Dim_title	= 1H
Dim_units	= [ppm]
Dimensions	= X
Site	= ECA 500
Spectrometer	= JNM-ECA500
Field_strength	= 11.7473579[T] (500[MH
X_acq_duration	= 1.74587904[s]
X_domain	= 1H
X_freq	= 500.15991521[MHz]
X_offset	= 5.0[ppm]
X_points	= 16384
X_prescans	= 1
X_resolution	= 0.57277737[Hz]
X_sweep	= 9.38438438[kHz]
Irr_domain	= 1H
Irr_freq	= 500.15991521[MHz]
Irr_offset	= 5.0[ppm]
Tri_domain	= 1H
Tri_freq	= 500.15991521[MHz]
Tri_offset	= 5.0[ppm]
Clipped	= FALSE
Mod_return	= 1
Scans	= 16
Total_scans	= 16
X_90_width	= 12.4[us]
X_acq_time	= 1.74587904[s]
X_angle	= 45[deg]
X_atn	= 4[dB]
X_pulse	= 6.2[us]
Irr_mode	= Off
Tri_mode	= Off
Dante_presat	= FALSE
Initial_wait	= 1[s]
Recvr_gain	= 28
Relaxation_delay	= 4[s]
Repetition_time	= 5.74587904[s]
Temp_get	= 20.3[dC]

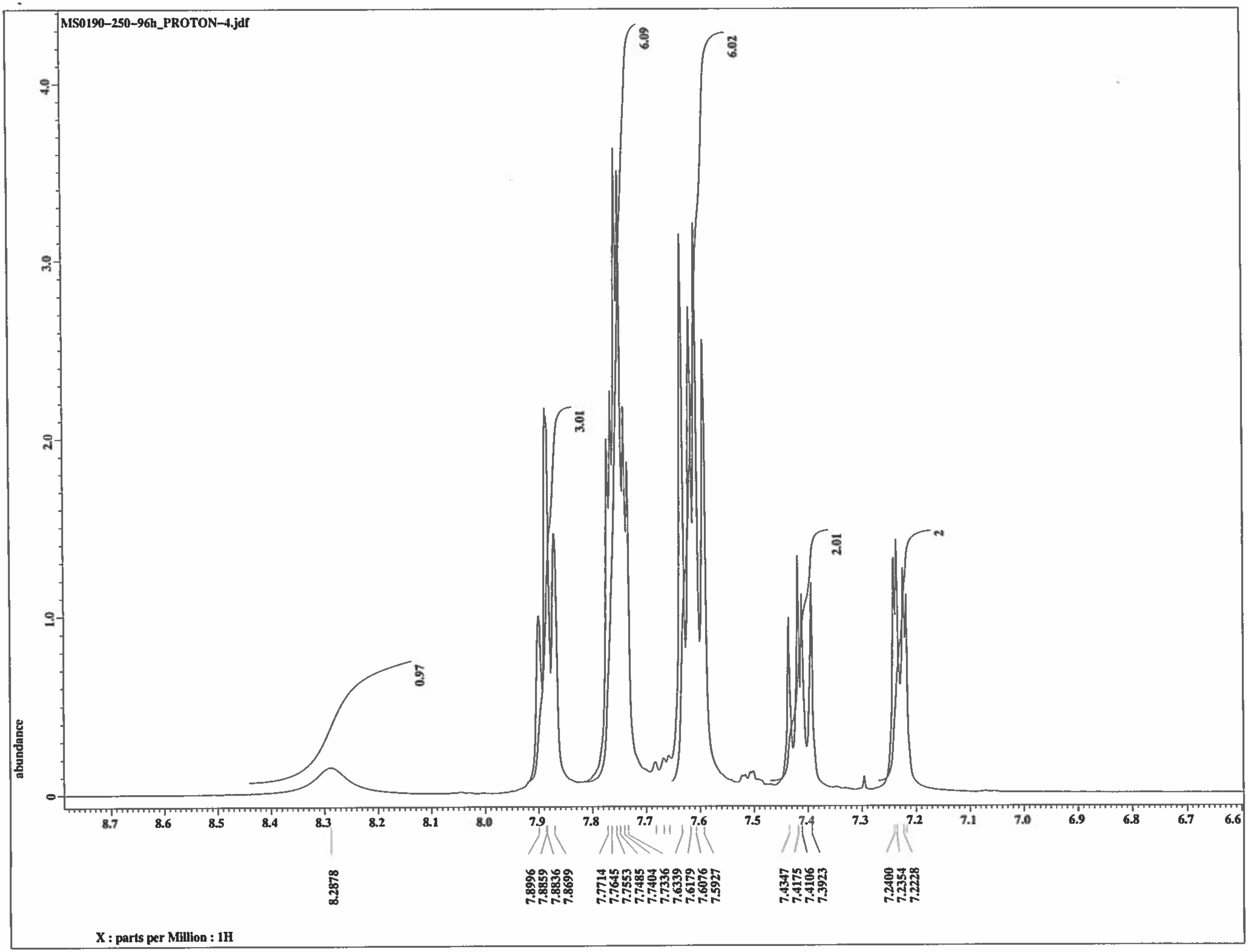
MS0190-250-96h_PROTON-4.jdf
abundance
0
1.0
2.0
3.0
4.0
0.97
3.01
6.09
6.02
2.01
2
8.7
8.6
8.5
8.4
8.3
8.2
8.1
8.0
7.9
7.8
7.7
7.6
7.5
7.4
7.3
7.2
7.1
7.0
6.9
6.8
6.7
6.6
8.2878
7.8996
7.8859
7.8836
7.8699
7.7714
7.7645
7.7553
7.7485
7.7404
7.7336
7.6339
7.6179
7.6076
7.5927
7.4347
7.4175
7.4106
7.3923
7.2400
7.2354
7.2228
X : parts per Million : 1H

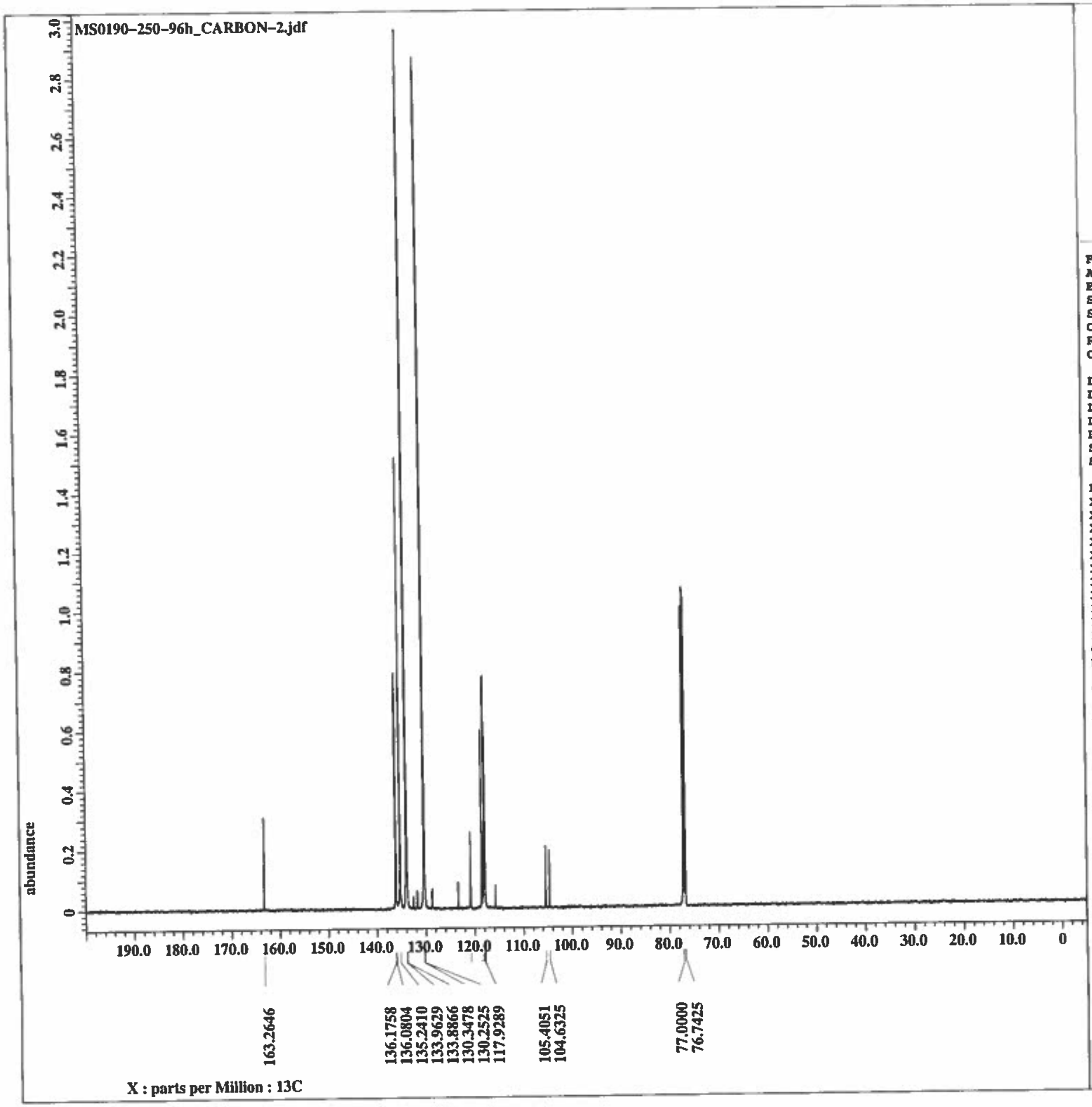

Filename	= MS0190-250-96h_CARBON
Author	= Jim Davis
Experiment	= single_pulse_dec
Sample_id	= MS0190-250-96h
Solvent	= CHLOROFORM-D
Creation_time	= 6-JAN-2019 16:12:33
Revision_time	= 6-JAN-2019 15:47:22
Current_time	= 6-JAN-2019 15:47:22
Data_format	= 1D COMPLEX
Dim_size	= 26214
Dim_title	= 13C
Dim_units	= [ppm]
Dimensions	= X
Site	= ECA 500
Spectrometer	= JNM-ECA500
Field_strength	= 11.7473579[T] (500[MH
X_acq_duration	= 0.83361792[s]
X_domain	= 13C
X_freq	= 125.76529768[MHz]
X_offset	= 100[ppm]
X_points	= 32768
X_prescans	= 4
X_resolution	= 1.19959034[Hz]
X_sweep	= 39.3081761[kHz]
Irr_domain	= 1H
Irr_freq	= 500.15991521[MHz]
Irr_offset	= 5.0[ppm]
Clipped	= FALSE
Mod_return	= 1
Scans	= 600
Total_scans	= 600
X_90_width	= 13.2[us]
X_acq_time	= 0.83361792[s]
X_angle	= 30[deg]
X_atn	= 6[dB]
X_pulse	= 4.4[us]
Irr_atn_dec	= 20.7[dB]
Irr_atn_noe	= 20.7[dB]
Irr_noise	= WALTZ
Decoupling	= TRUE
Initial_wait	= 1[s]
Noe	= TRUE
Noe_time	= 2[s]
Recvr_gain	= 60
Relaxation_delay	= 2[s]
Repetition_time	= 2.83361792[s]
Temp_get	= 20.9[dC]

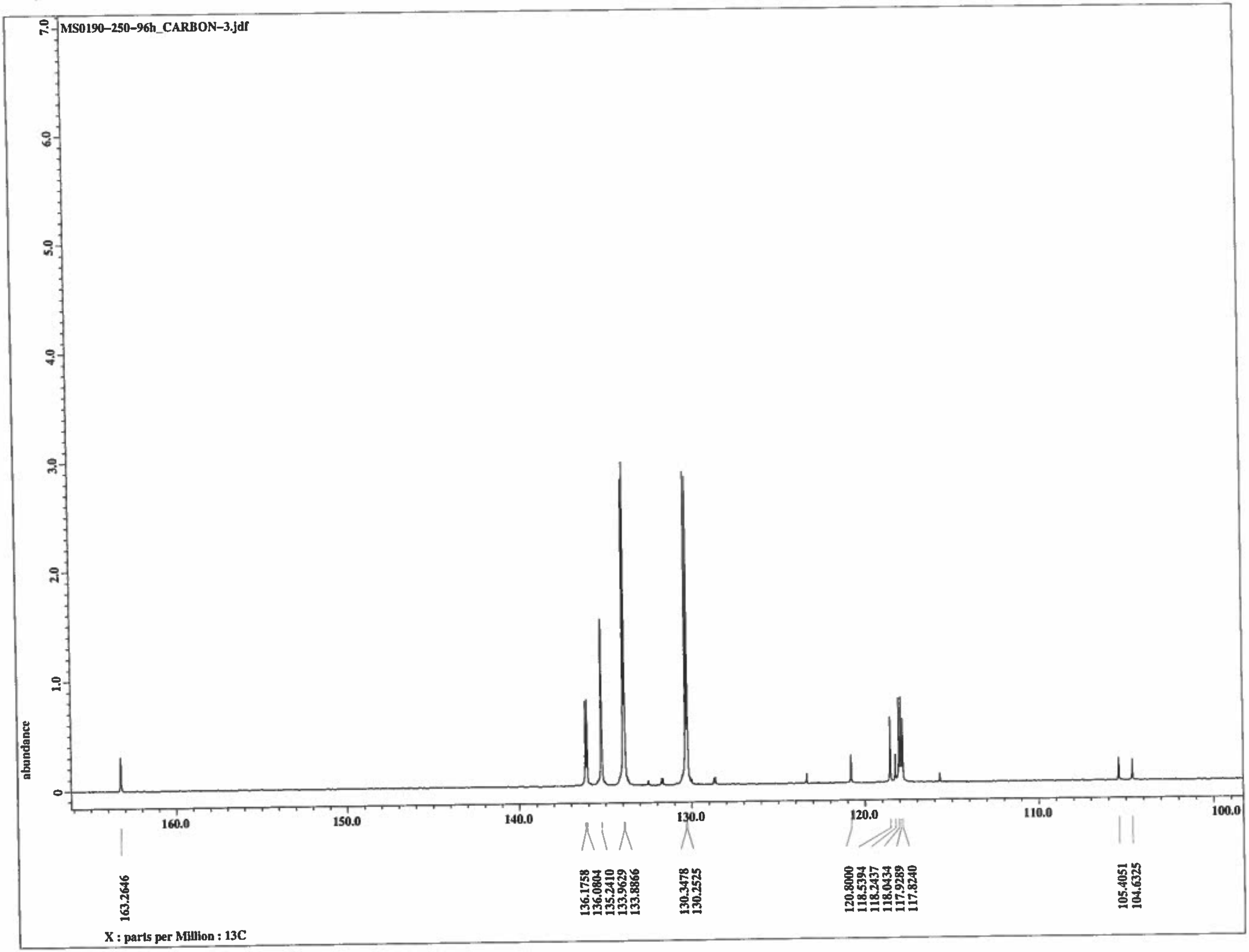
MS0190-250-96h_CARBON-3.jdf
abundance
0
1.0
2.0
3.0
4.0
5.0
6.0
7.0
160.0
150.0
140.0
130.0
120.0
110.0
100.0
163.2646
136.1758
136.0804
135.2410
133.9629
133.8866
130.3478
130.2525
120.8000
118.5394
118.2437
118.0434
117.9289
117.8240
105.4051
104.6325
X : parts per Million : 13C

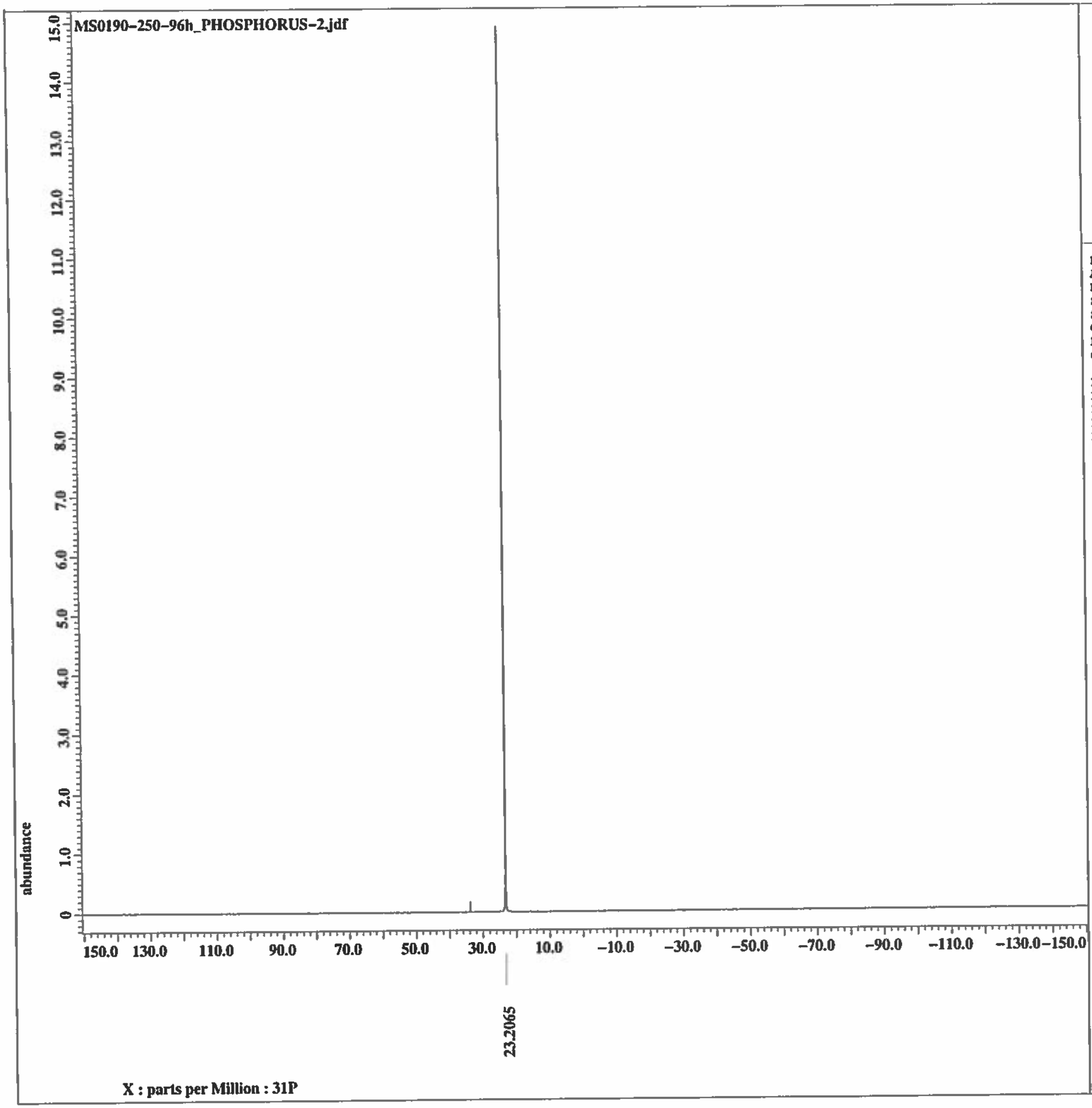

Filename	= MS0190-250-96h_PHOSPH
Author	= Jim Davis
Experiment	= single_pulse_dec
Sample_id	= MS0190-250-96h
Solvent	= CHLOROFORM-D
Creation_time	= 6-JAN-2019 16:33:32
Revision_time	= 6-JAN-2019 16:08:21
Current_time	= 6-JAN-2019 16:08:21
Data_format	= 1D COMPLEX
Dim_size	= 52428
Dim_title	= 31P
Dim_units	= [ppm]
Dimensions	= X
Site	= ECA 500
Spectrometer	= JNM-ECA500
Field_strength	= 11.7473579[T] (500[MH
X_acq_duration	= 0.85983232[s]
X_domain	= 31P
X_freq	= 202.46831075[MHz]
X_offset	= 0[ppm]
X_points	= 65536
X_prescans	= 4
X_resolution	= 1.16301746[Hz]
X_sweep	= 76.2195122[kHz]
Irr_domain	= 1H
Irr_freq	= 500.15991521[MHz]
Irr_offset	= 5.0[ppm]
Clipped	= TRUE
Mod_return	= 1
Scans	= 128
Total_scans	= 128
X_90_width	= 14.687[us]
X_acq_time	= 0.85983232[s]
X_angle	= 30[deg]
X_atn	= 5[dB]
X_pulse	= 4.89566667[us]
Irr_atn_dec	= 20.7[dB]
Irr_atn_noe	= 20.7[dB]
Irr_noise	= WALTZ
Decoupling	= TRUE
Initial_wait	= 1[s]
Noe	= TRUE
Noe_time	= 2[s]
Recvr_gain	= 54
Relaxation_delay	= 2[s]
Repetition_time	= 2.85983232[s]
Temp_get	= 20.7[dC]

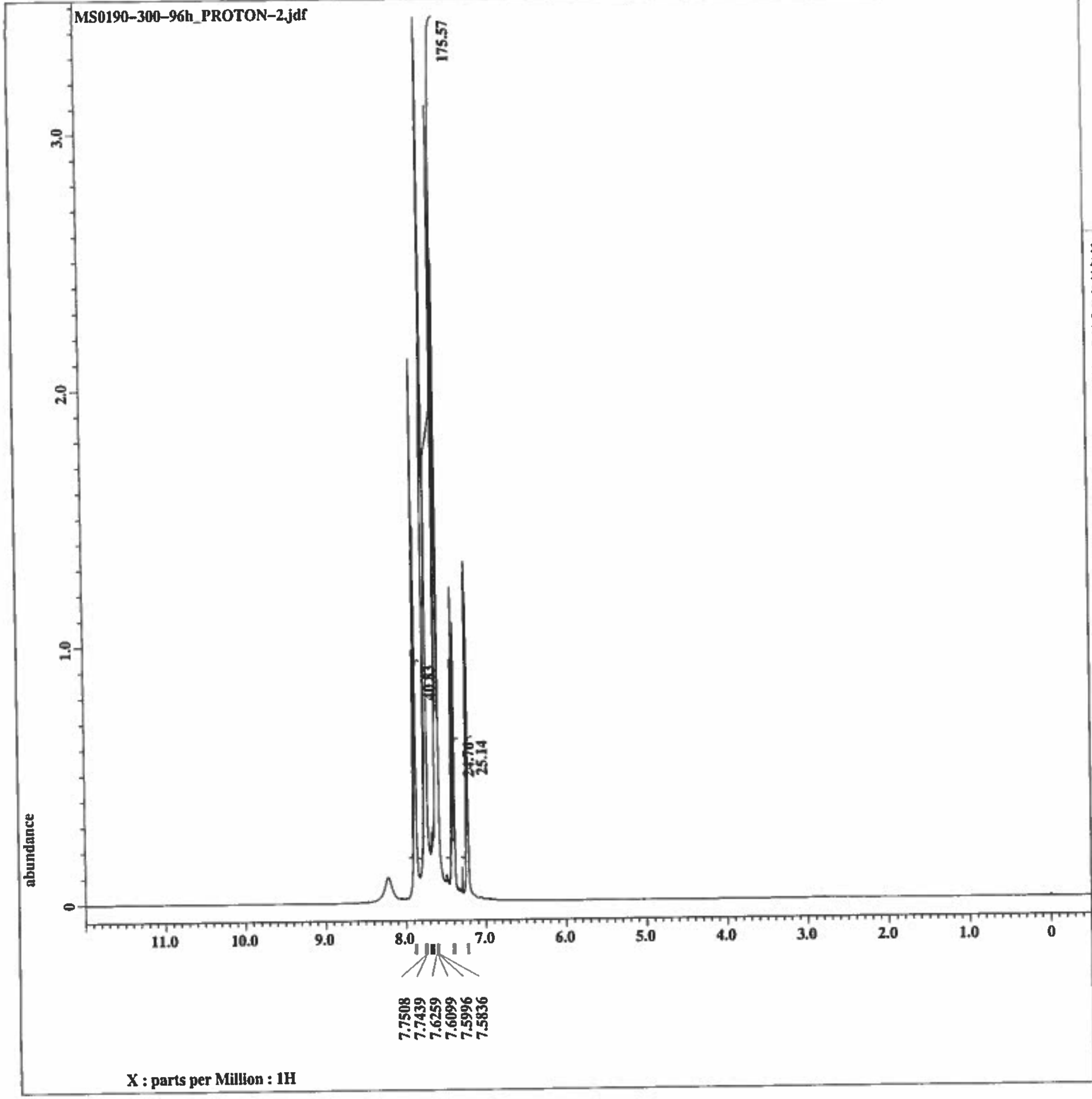

Filename	= MS0190-300-96h_PROTON
Author	= Jim Davis
Experiment	= single_pulse.ex2
Sample_id	= MS0190-300-96h
Solvent	= CHLOROFORM-D
Creation_time	= 6-JAN-2019 16:40:50
Revision_time	= 6-JAN-2019 16:15:39
Current_time	= 6-JAN-2019 16:15:39
Data_format	= 1D COMPLEX
Dim_size	= 13107
Dim_title	= 1H
Dim_units	= [ppm]
Dimensions	= X
Site	= ECA 500
Spectrometer	= JNM-ECA500
Field_strength	= 11.7473579[T] (500[MH
X_acq_duration	= 1.74587904[s]
X_domain	= 1H
X_freq	= 500.15991521[MHz]
X_offset	= 5.0[ppm]
X_points	= 16384
X_prescans	= 1
X_resolution	= 0.57277737[Hz]
X_sweep	= 9.38438438[kHz]
Irr_domain	= 1H
Irr_freq	= 500.15991521[MHz]
Irr_offset	= 5.0[ppm]
Tri_domain	= 1H
Tri_freq	= 500.15991521[MHz]
Tri_offset	= 5.0[ppm]
Clipped	= FALSE
Mod_return	= 1
Scans	= 16
Total_scans	= 16
X_90_width	= 12.4[us]
X_acq_time	= 1.74587904[s]
X_angle	= 45[deg]
X_atn	= 4[dB]
X_pulse	= 6.2[us]
Irr_mode	= Off
Tri_mode	= Off
Dante_presat	= FALSE
Initial_wait	= 1[s]
Recvr_gain	= 28
Relaxation_delay	= 4[s]
Repetition_time	= 5.74587904[s]
Temp_get	= 20.5[dC]

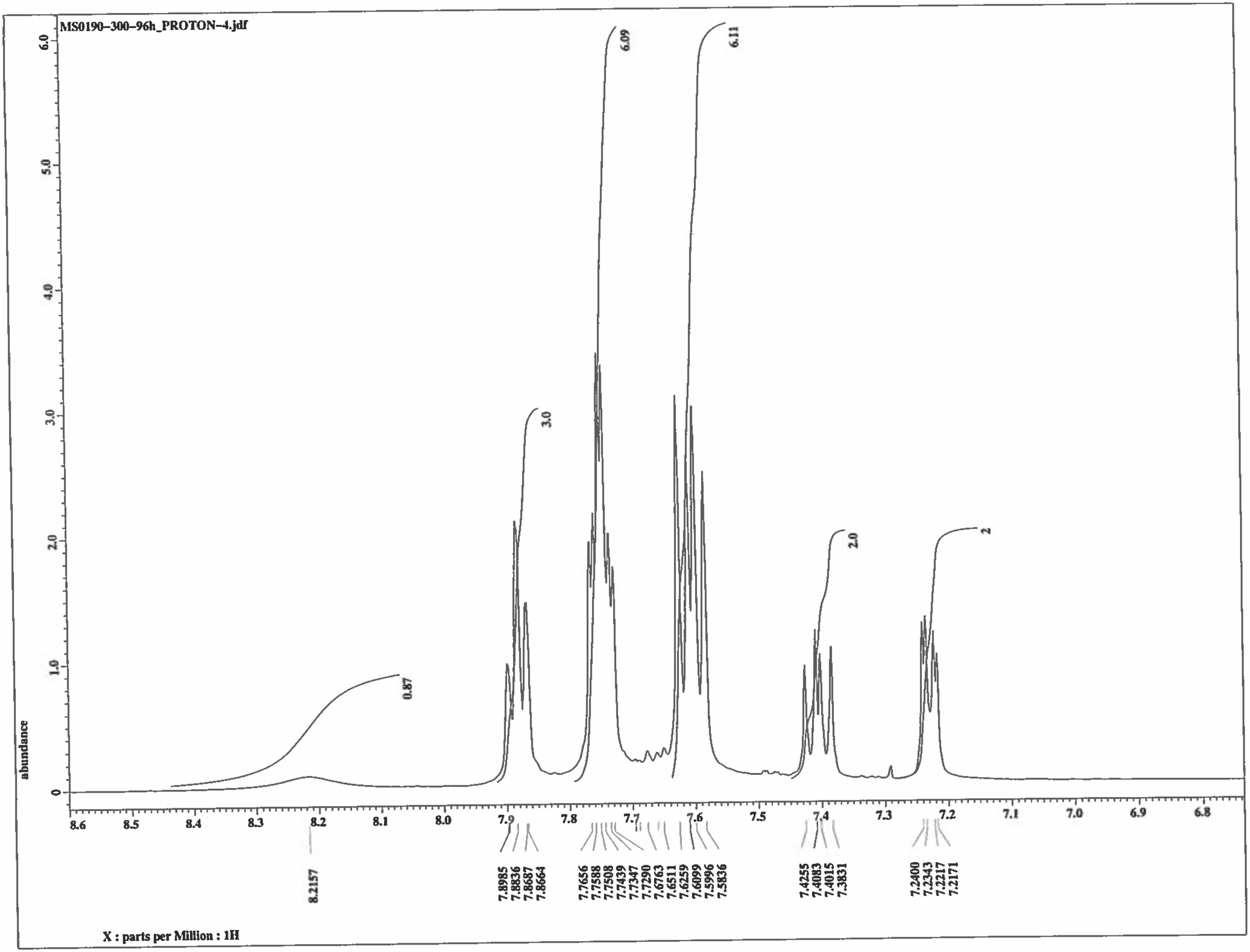
MS0190-300-96h_PROTON-4.jdf
abundance
X : parts per Million : 1H
0.87
3.0
6.09
6.11
2.0
2
8.2157
7.8985
7.8836
7.8687
7.8664
7.7656
7.7588
7.7508
7.7439
7.7347
7.7290
7.6763
7.6511
7.6259
7.6099
7.5996
7.5836
7.4255
7.4083
7.4015
7.3831
7.2400
7.2343
7.2217
7.2171

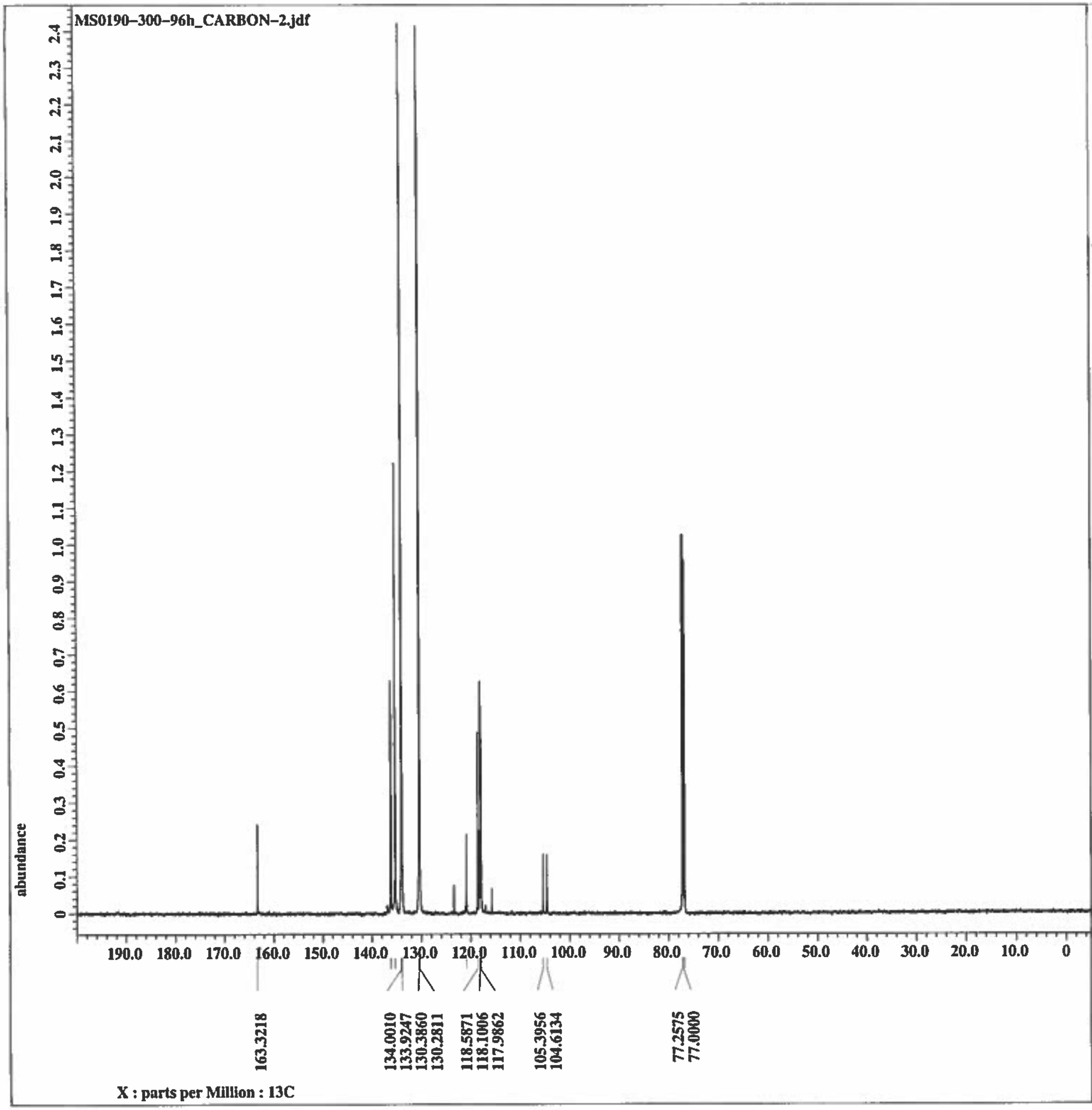

Filename	= MS0190-300-96h_CARBON
Author	= Jim Davis
Experiment	= single_pulse_dec
Sample_id	= MS0190-300-96h
Solvent	= CHLOROFORM-D
Creation_time	= 6-JAN-2019 17:11:33
Revision_time	= 6-JAN-2019 16:46:20
Current_time	= 6-JAN-2019 16:46:22
Data_format	= 1D COMPLEX
Dim_size	= 26214
Dim_title	= 13C
Dim_units	= [ppm]
Dimensions	= X
Site	= ECA 500
Spectrometer	= JNM-ECA500
Field_strength	= 11.7473579[T] (500[MH
X_acq_duration	= 0.83361792[s]
X_domain	= 13C
X_freq	= 125.76529768[MHz]
X_offset	= 100[ppm]
X_points	= 32768
X_prescans	= 4
X_resolution	= 1.19959034[Hz]
X_sweep	= 39.3081761[kHz]
Irr_domain	= 1H
Irr_freq	= 500.15991521[MHz]
Irr_offset	= 5.0[ppm]
Clipped	= FALSE
Mod_return	= 1
Scans	= 600
Total_scans	= 600
X_90_width	= 13.2[us]
X_acq_time	= 0.83361792[s]
X_angle	= 30[deg]
X_atn	= 6[dB]
X_pulse	= 4.4[us]
Irr_atn_dec	= 20.7[dB]
Irr_atn_noe	= 20.7[dB]
Irr_noise	= WALTZ
Decoupling	= TRUE
Initial_wait	= 1[s]
Noe	= TRUE
Noe_time	= 2[s]
Recvr_gain	= 60
Relaxation_delay	= 2[s]
Repetition_time	= 2.83361792[s]
Temp_get	= 21[dC]

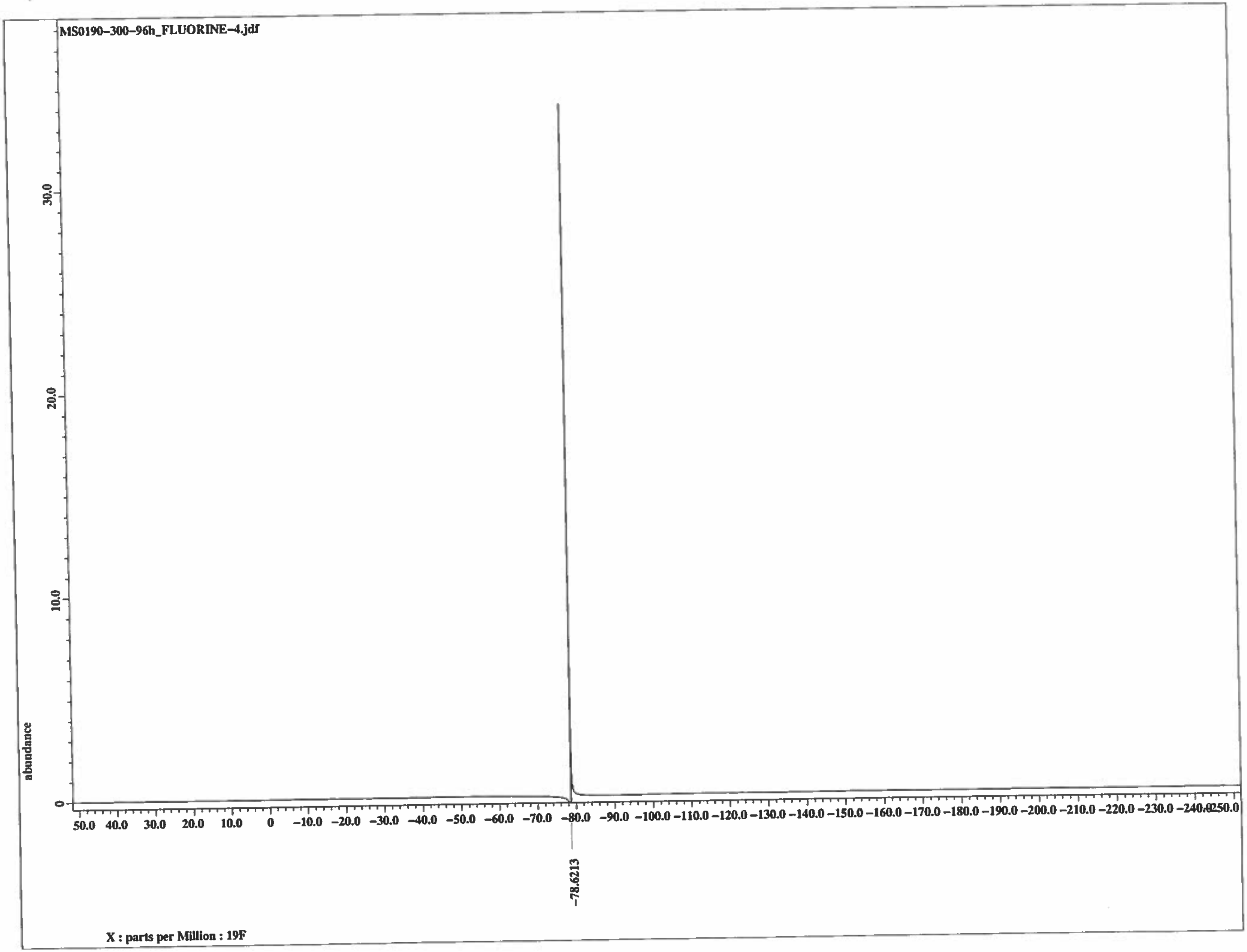

MS0190-300-96h_FLUORINE-4.jdf
abundance
30.0
20.0
10.0
0
50.0 40.0 30.0 20.0 10.0 0 -10.0 -20.0 -30.0 -40.0 -50.0 -60.0 -70.0 -80.0 -90.0 -100.0 -110.0 -120.0 -130.0 -140.0 -150.0 -160.0 -170.0 -180.0 -190.0 -200.0 -210.0 -220.0 -230.0 -240.0 250.0
-78.6213
X : parts per Million : 19F

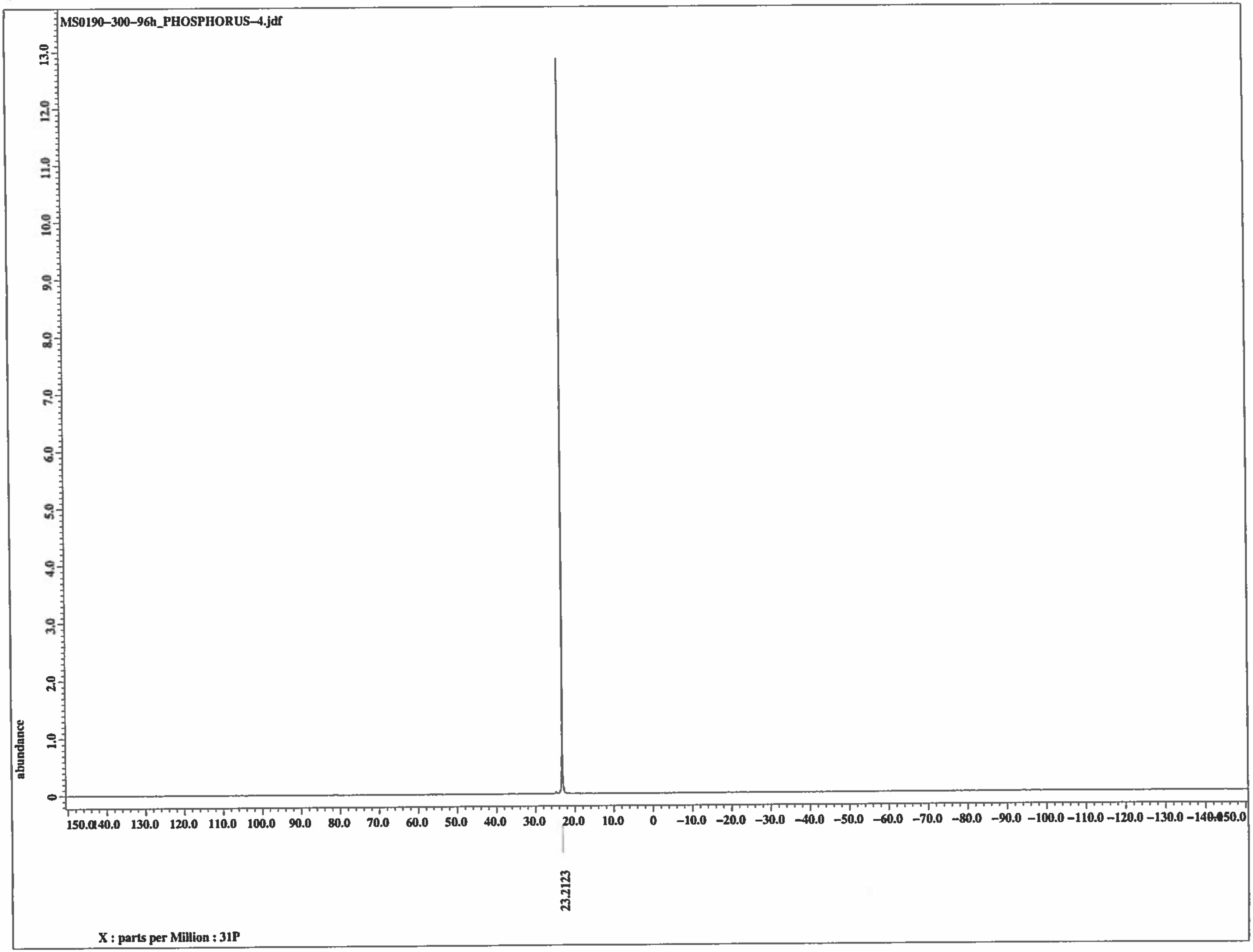
MS0190–300–96h_PHOSPHORUS–4.jdf
abundance
0
1.0
2.0
3.0
4.0
5.0
6.0
7.0
8.0
9.0
10.0
11.0
12.0
13.0
150.0
140.0
130.0
120.0
110.0
100.0
90.0
80.0
70.0
60.0
50.0
40.0
30.0
20.0
10.0
0
−10.0
−20.0
−30.0
−40.0
−50.0
−60.0
−70.0
−80.0
−90.0
−100.0
−110.0
−120.0
−130.0
−140.0
−150.0
23.2123
X : parts per Million : 31P

Compound 18 Pre- and Post-heating NMR Spectra

Temperature of Post-heating samples noted in upper left corner of each spectrum

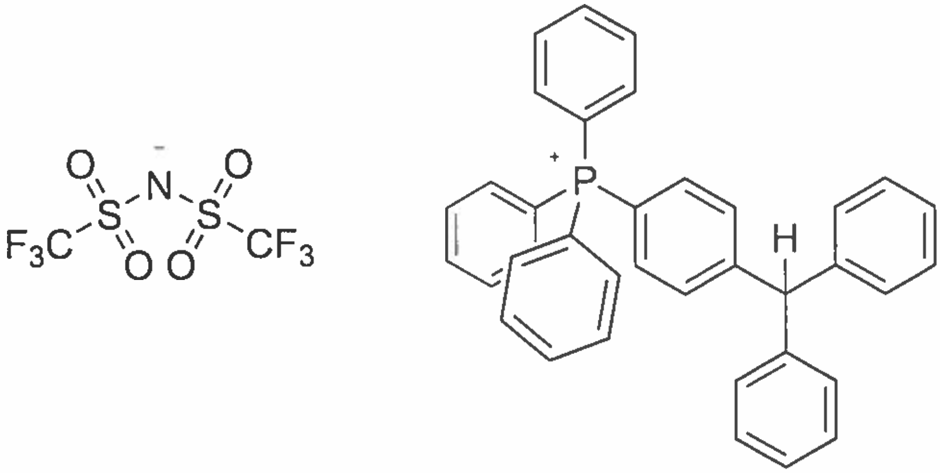

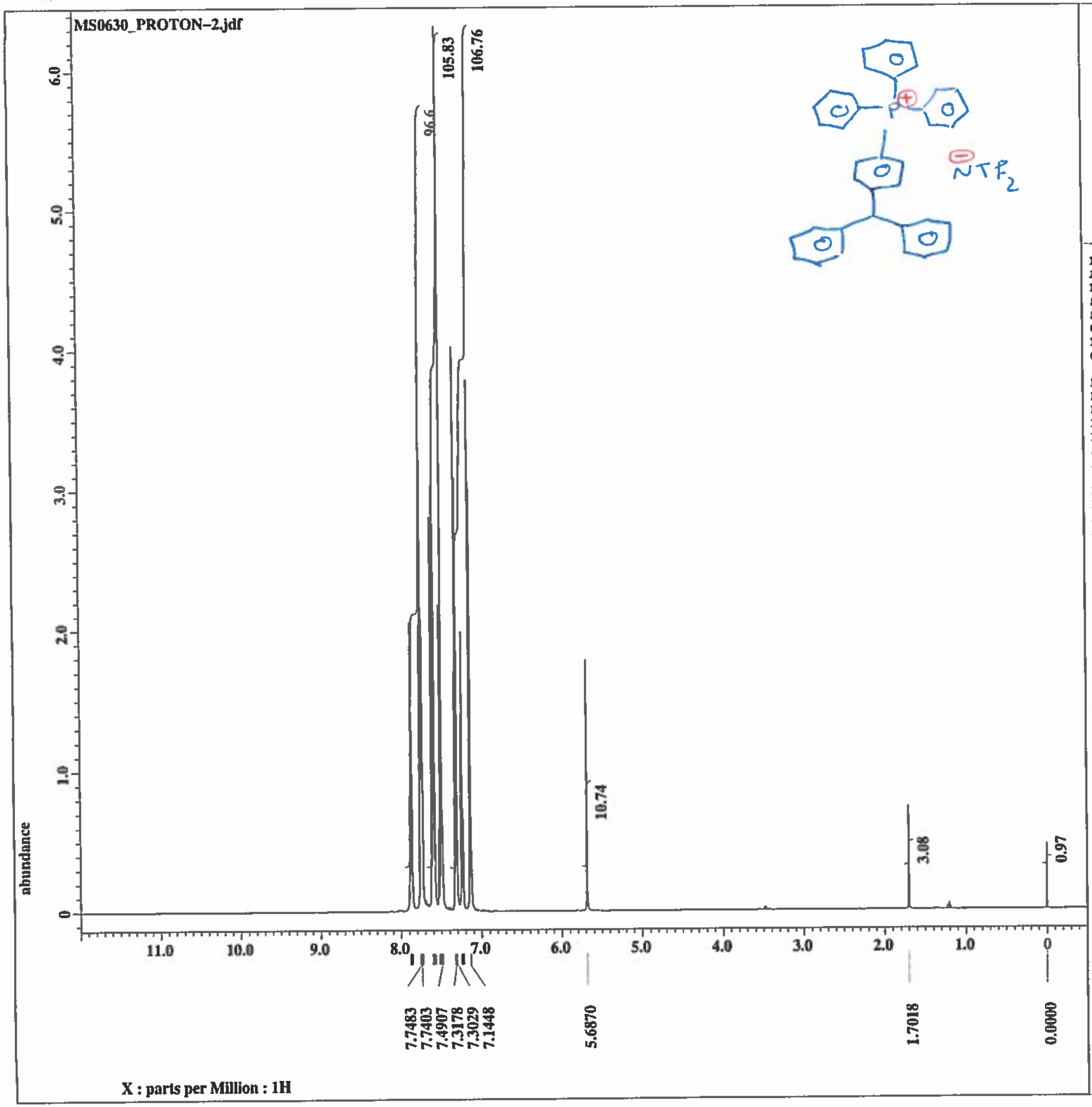

Filename	= MS0630_PROTON-2.jdf
Author	= Jim Davis
Experiment	= single_pulse.ex2
Sample_id	= MS0630
Solvent	= CHLOROFORM-D
Creation_time	= 13-DEC-2018 14:04:58
Revision_time	= 13-DEC-2018 13:39:03
Current_time	= 13-DEC-2018 13:39:03
Data_format	= 1D COMPLEX
Dim_size	= 13107
Dim_title	= 1H
Dim_units	= [ppm]
Dimensions	= X
Site	= ECA 500
Spectrometer	= JNM-ECA500
Field_strength	= 11.7473579[T] (500[MH
X_acq_duration	= 1.74587904[s]
X_domain	= 1H
X_freq	= 500.15991521[MHz]
X_offset	= 5.0[ppm]
X_points	= 16384
X_prescans	= 1
X_resolution	= 0.57277737[Hz]
X_sweep	= 9.38438438[kHz]
Irr_domain	= 1H
Irr_freq	= 500.15991521[MHz]
Irr_offset	= 5.0[ppm]
Tri_domain	= 1H
Tri_freq	= 500.15991521[MHz]
Tri_offset	= 5.0[ppm]
Clipped	= FALSE
Mod_return	= 1
Scans	= 16
Total_scans	= 16
X_90_width	= 12.4[us]
X_acq_time	= 1.74587904[s]
X_angle	= 45[deg]
X_atn	= 4[dB]
X_pulse	= 6.2[us]
Irr_mode	= Off
Tri_mode	= Off
Dante_presat	= FALSE
Initial_wait	= 1[s]
Recvr_gain	= 30
Relaxation_delay	= 4[s]
Repetition_time	= 5.74587904[s]
Temp_get	= 20.1[dC]

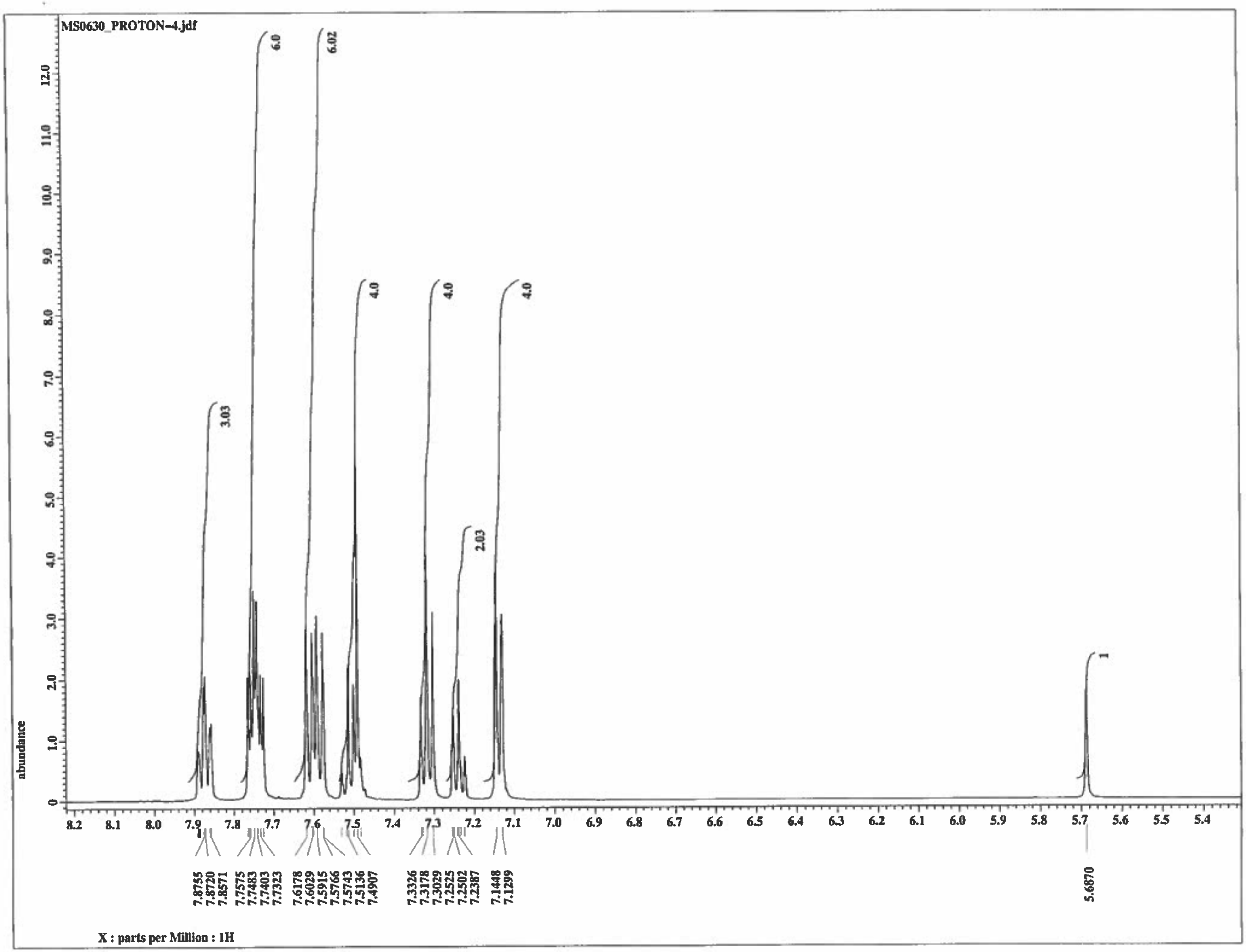
MS0630_PROTON-4.jdf
abundance
X : parts per Million : 1H
3.03
6.0
6.02
4.0
4.0
2.03
4.0
1
7.8755
7.8720
7.8571
7.7575
7.7483
7.7403
7.7323
7.6178
7.6029
7.5915
7.5766
7.5743
7.5136
7.4907
7.3326
7.3178
7.3029
7.2525
7.2502
7.2387
7.1448
7.1299
5.6870

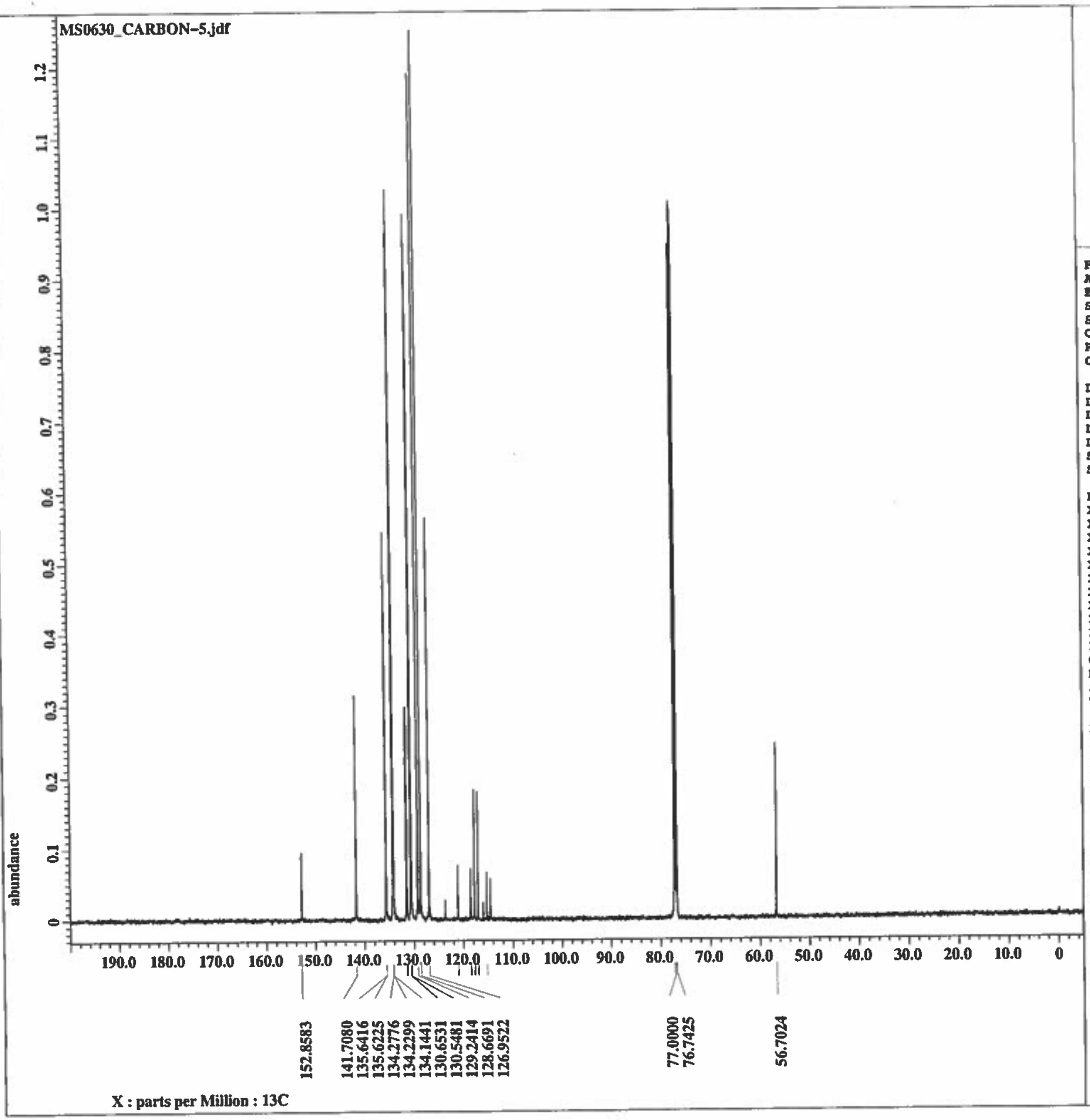

Filename	= MS0630_CARBON-5.jdf
Author	= Jim Davis
Experiment	= single_pulse_dec
Sample_id	= MS0630
Solvent	= CHLOROFORM-D
Creation_time	= 13-DEC-2018 16:30:15
Revision_time	= 13-DEC-2018 16:04:18
Current_time	= 13-DEC-2018 16:04:18
Data_format	= 1D COMPLEX
Dim_size	= 26214
Dim_title	= 13C
Dim_units	= [ppm]
Dimensions	= X
Site	= ECA 500
Spectrometer	= JNM-ECA500
Field_strength	= 11.7473579[T] (500[MH
X_acq_duration	= 0.83361792[s]
X_domain	= 13C
X_freq	= 125.76529768[MHz]
X_offset	= 100[ppm]
X_points	= 32768
X_prescans	= 4
X_resolution	= 1.19959034[Hz]
X_sweep	= 39.3081761[kHz]
Irr_domain	= 1H
Irr_freq	= 500.15991521[MHz]
Irr_offset	= 5.0[ppm]
Clipped	= FALSE
Mod_return	= 1
Scans	= 1500
Total_scans	= 1500
X_90_width	= 13.2[us]
X_acq_time	= 0.83361792[s]
X_angle	= 30[deg]
X_atn	= 6[dB]
X_pulse	= 4.4[us]
Irr_atn_dec	= 20.7[dB]
Irr_atn_noe	= 20.7[dB]
Irr_noise	= WALTZ
Decoupling	= TRUE
Initial_wait	= 1[s]
Noe	= TRUE
Noe_time	= 2[s]
Recvr_gain	= 60
Relaxation_delay	= 2[s]
Repetition_time	= 2.83361792[s]
Temp_get	= 20.6[dC]

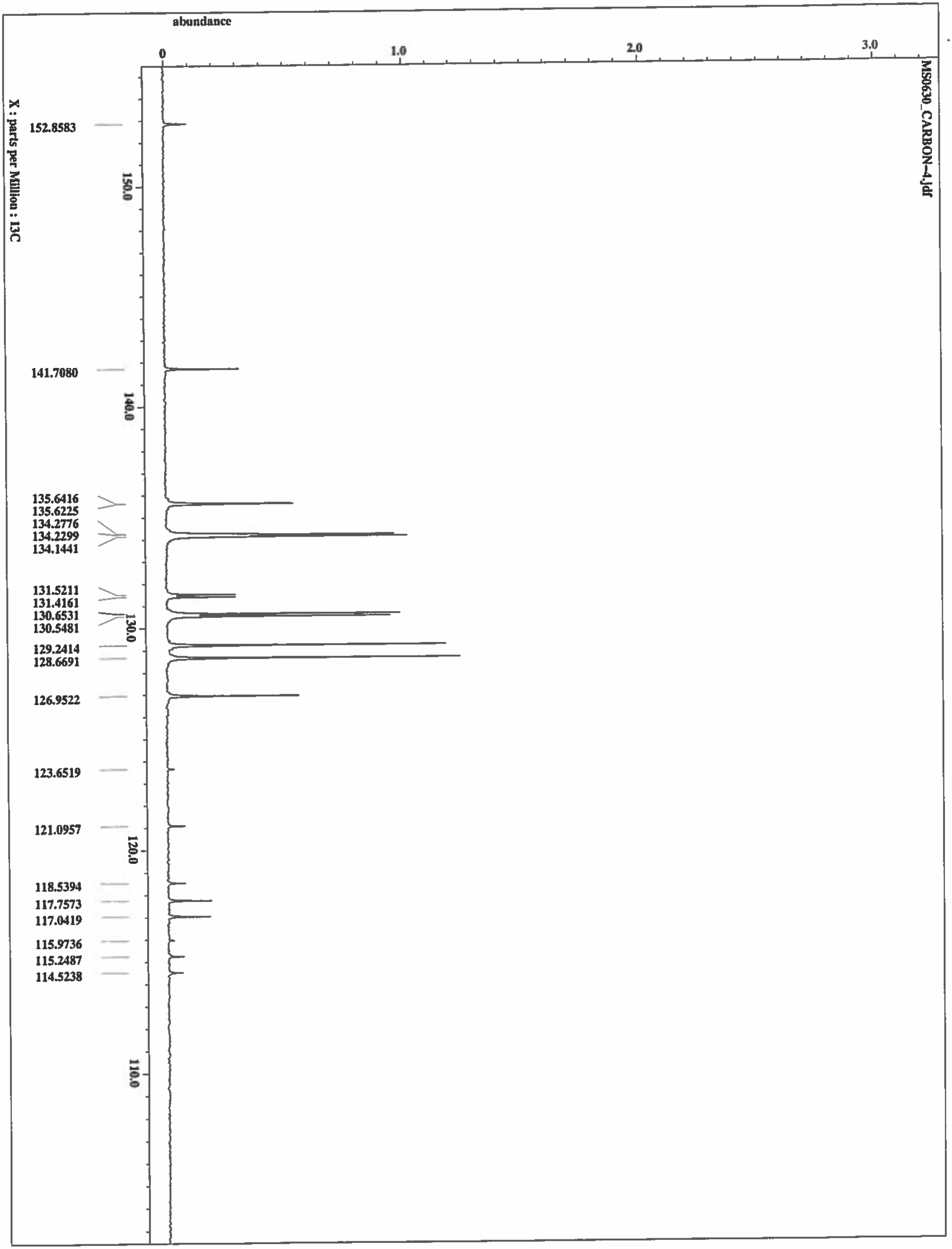

abundance
0
1.0
2.0
3.0
MS0630_CARBON-4.jdf
X : parts per Million : 13C
152.8583
150.0
141.7080
140.0
135.6416
135.6225
134.2776
134.2299
134.1441
131.5211
131.4161
130.6531
130.5481
130.0
129.2414
128.6691
126.9522
123.6519
121.0957
120.0
118.5394
117.7573
117.0419
115.9736
115.2487
114.5238
110.0

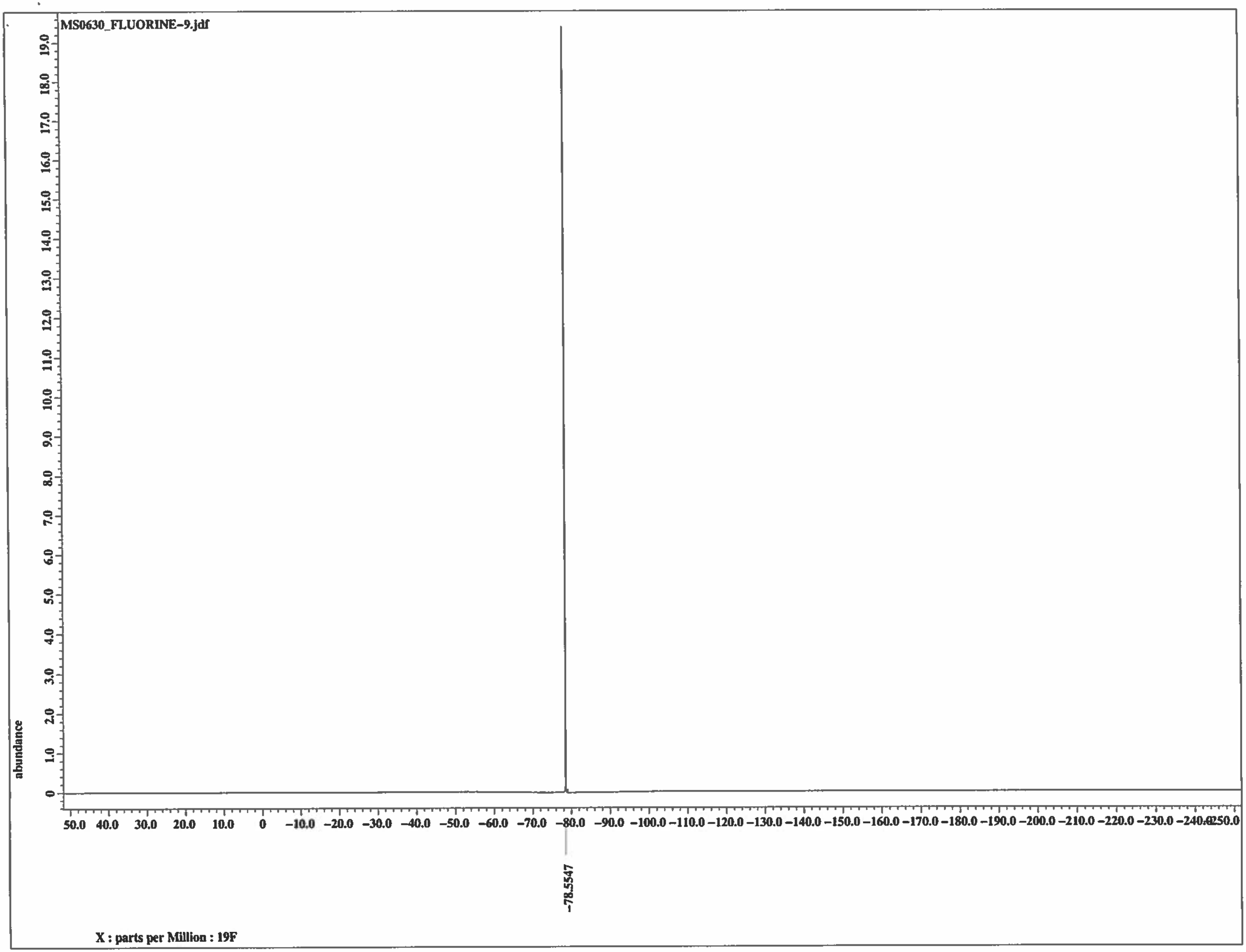

MS0630_FLUORINE-9.jdf
abundance
-78.5547
X : parts per Million : 19F

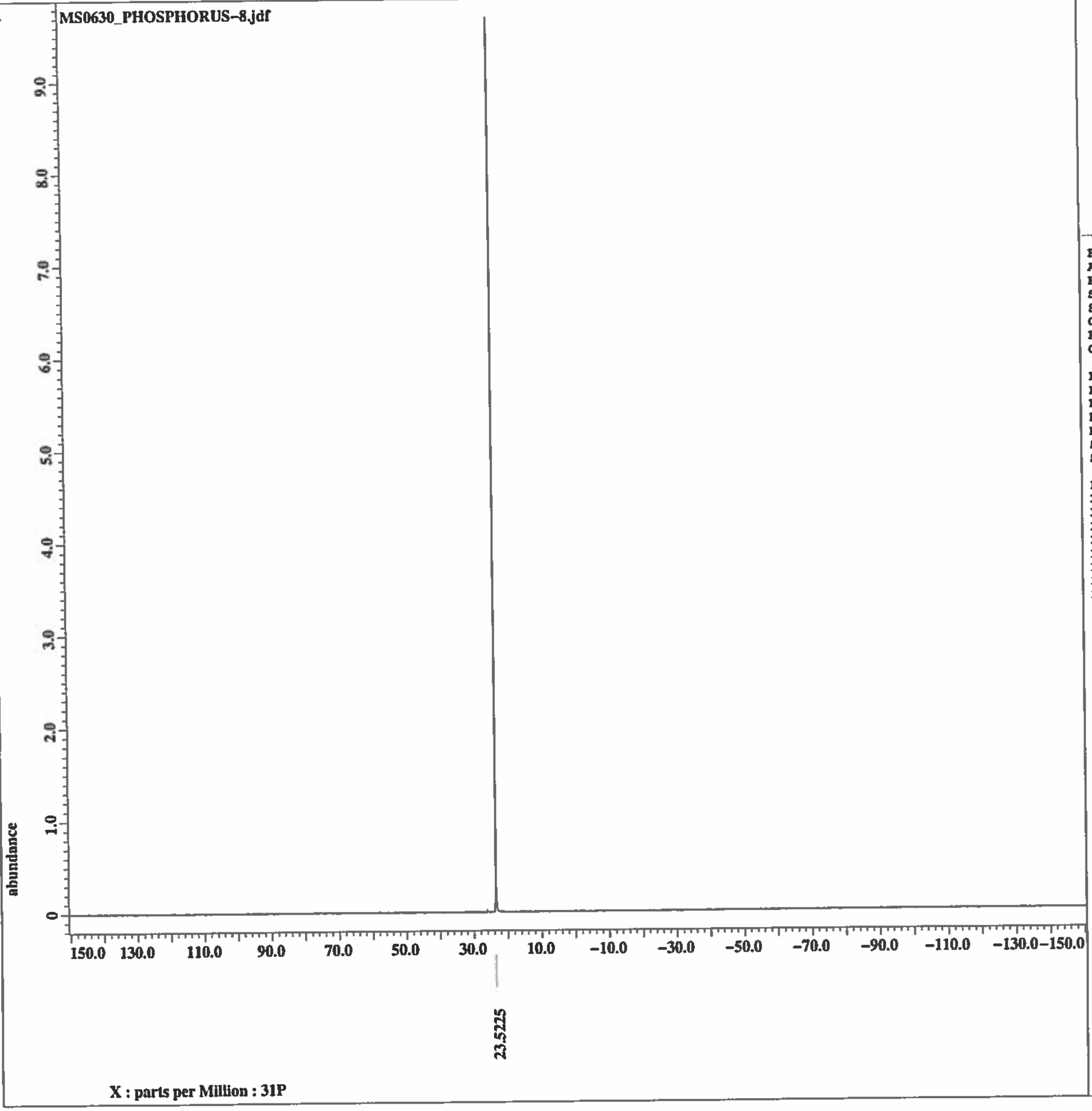

SOUTH ALABAMA JAGUARS

Filename	= MS0630_PHOSPHORUS-8.j
Author	= Jim Davis
Experiment	= single_pulse_dec
Sample_id	= MS0630
Solvent	= CHLOROFORM-D
Creation_time	= 13-DEC-2018 16:38:55
Revision_time	= 13-DEC-2018 16:12:58
Current_time	= 13-DEC-2018 16:12:58
Data_format	= 1D COMPLEX
Dim_size	= 52428
Dim_title	= 31P
Dim_units	= [ppm]
Dimensions	= X
Site	= ECA 500
Spectrometer	= JNM-ECA500
Field_strength	= 11.7473579[T] (500[MH
X_acq_duration	= 0.85983232[s]
X_domain	= 31P
X_freq	= 202.46831075[MHz]
X_offset	= 0[ppm]
X_points	= 65536
X_prescans	= 4
X_resolution	= 1.16301746[Hz]
X_sweep	= 76.2195122[kHz]
Irr_domain	= 1H
Irr_freq	= 500.15991521[MHz]
Irr_offset	= 5.0[ppm]
Clipped	= FALSE
Mod_return	= 1
Scans	= 128
Total_scans	= 128
X_90_width	= 14.687[us]
X_acq_time	= 0.85983232[s]
X_angle	= 30[deg]
X_atn	= 5[dB]
X_pulse	= 4.89566667[us]
Irr_atn_dec	= 20.7[dB]
Irr_atn_noe	= 20.7[dB]
Irr_noise	= WALTZ
Decoupling	= TRUE
Initial_wait	= 1[s]
Noe	= TRUE
Noe_time	= 2[s]
Recvr_gain	= 54
Relaxation_delay	= 2[s]
Repetition_time	= 2.85983232[s]
Temp_get	= 20.6[dC]

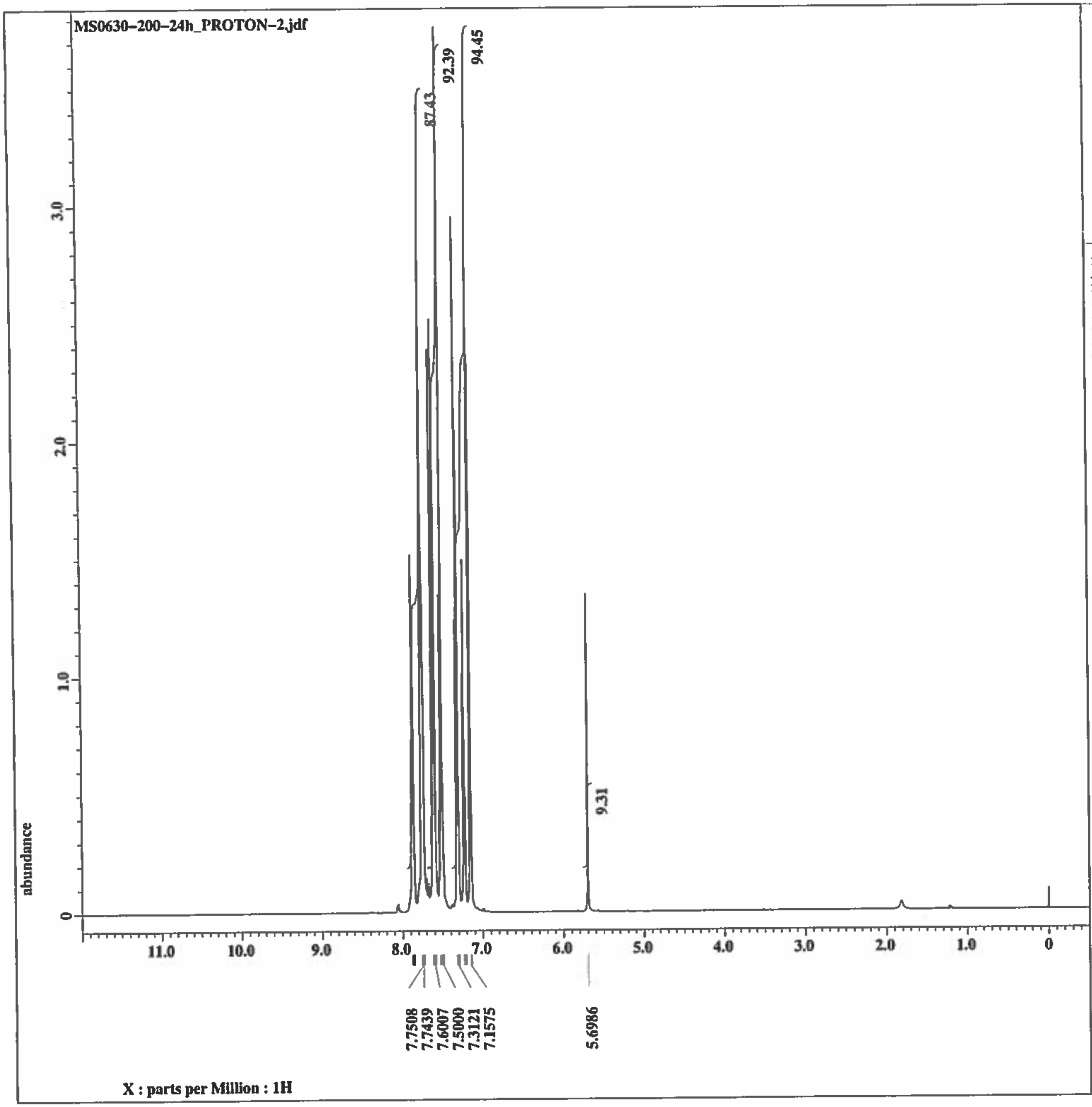

Filename	= MS0630-200-24h_PROTON
Author	= Jim Davis
Experiment	= single_pulse.ex2
Sample_id	= MS0630-200-24h
Solvent	= CHLOROFORM-D
Creation_time	= 14-DEC-2018 14:09:36
Revision_time	= 14-DEC-2018 13:43:35
Current_time	= 14-DEC-2018 13:43:35
Data_format	= 1D COMPLEX
Dim_size	= 13107
Dim_title	= 1H
Dim_units	= [ppm]
Dimensions	= X
Site	= ECA 500
Spectrometer	= JNM-ECA500
Field_strength	= 11.7473579[T] (500[MH
X_acq_duration	= 1.74587904[s]
X_domain	= 1H
X_freq	= 500.15991521[MHz]
X_offset	= 5.0[ppm]
X_points	= 16384
X_prescans	= 1
X_resolution	= 0.57277737[Hz]
X_sweep	= 9.38438438[kHz]
Irr_domain	= 1H
Irr_freq	= 500.15991521[MHz]
Irr_offset	= 5.0[ppm]
Tri_domain	= 1H
Tri_freq	= 500.15991521[MHz]
Tri_offset	= 5.0[ppm]
Clipped	= FALSE
Mod_return	= 1
Scans	= 16
Total_scans	= 16
X_90_width	= 12.4[us]
X_acq_time	= 1.74587904[s]
X_angle	= 45[deg]
X_atn	= 4[dB]
X_pulse	= 6.2[us]
Irr_mode	= Off
Tri_mode	= Off
Dante_presat	= FALSE
Initial_wait	= 1[s]
Recvr_gain	= 34
Relaxation_delay	= 4[s]
Repetition_time	= 5.74587904[s]
Temp_get	= 19.9[dC]

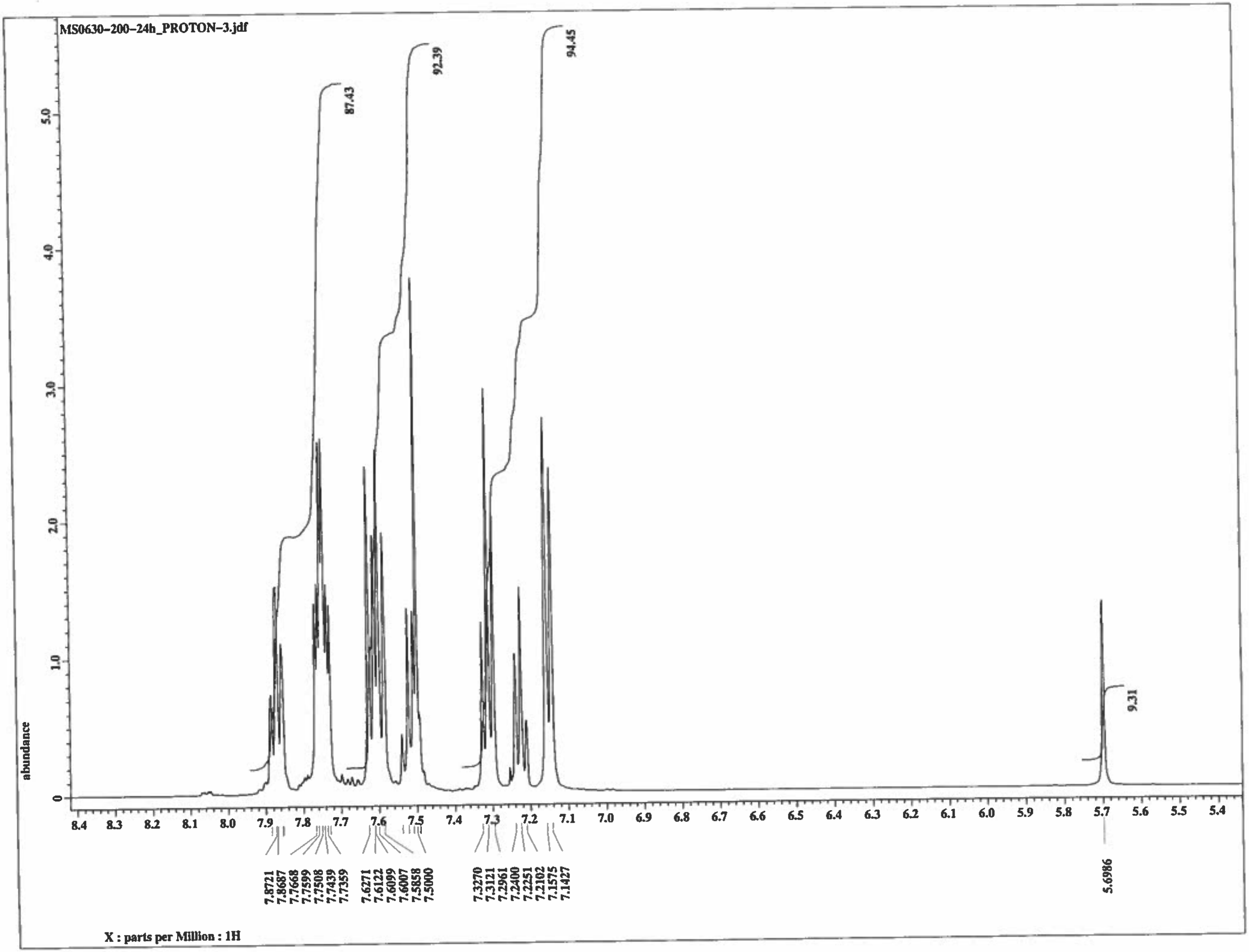

MS0630-200-24h_PROTON-3.jdf
abundance
0
1.0
2.0
3.0
4.0
5.0
87.43
92.39
94.45
9.31
8.4
8.3
8.2
8.1
8.0
7.9
7.8
7.7
7.6
7.5
7.4
7.3
7.2
7.1
7.0
6.9
6.8
6.7
6.6
6.5
6.4
6.3
6.2
6.1
6.0
5.9
5.8
5.7
5.6
5.5
5.4
7.8721
7.8687
7.7668
7.7599
7.7508
7.7439
7.7359
7.6271
7.6122
7.6099
7.6007
7.5858
7.5000
7.3270
7.3121
7.2961
7.2400
7.2251
7.2102
7.1575
7.1427
5.6986
X : parts per Million : 1H

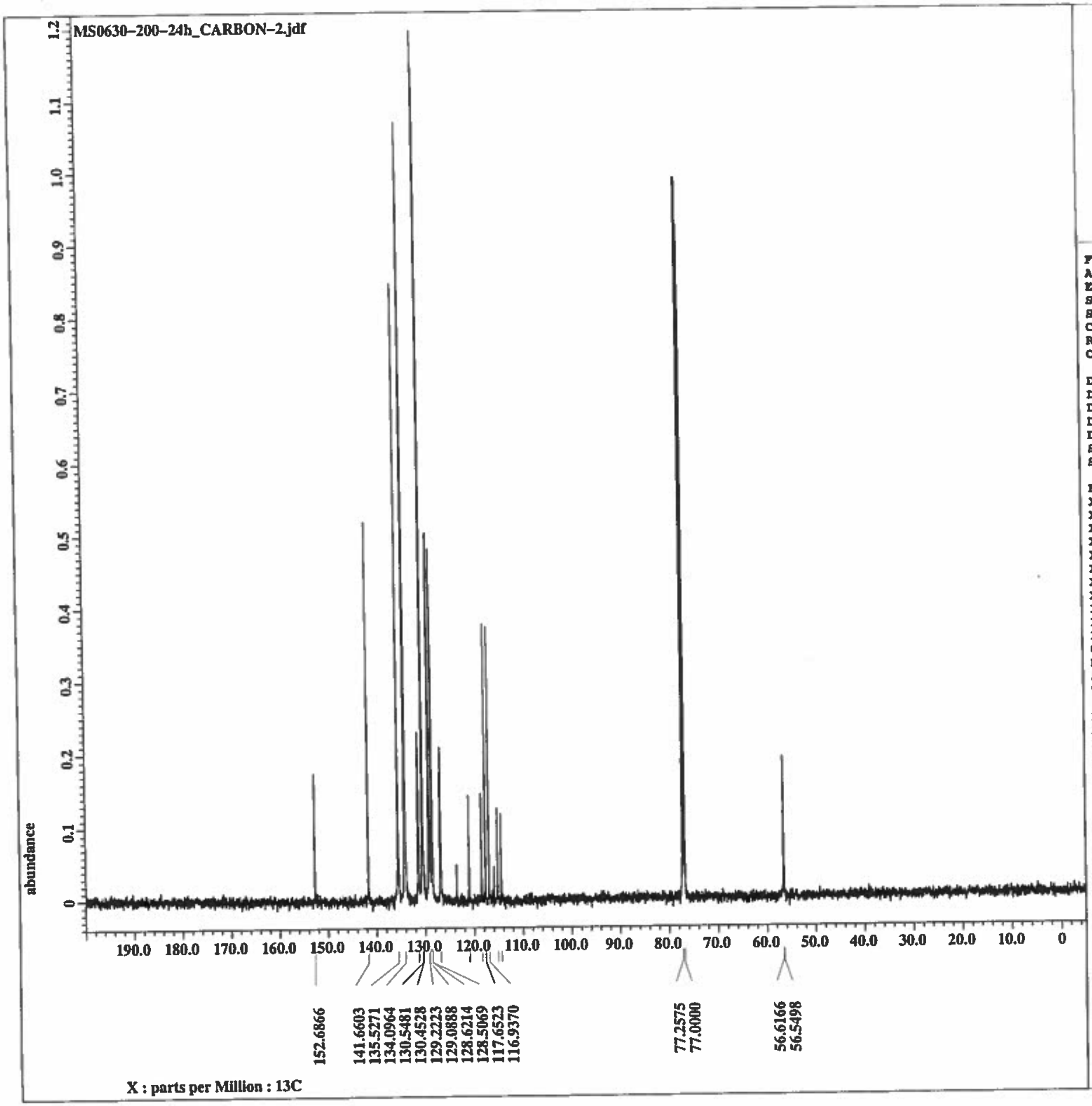

Filename	= MS0630-200-24h_CARBON
Author	= Jim Davis
Experiment	= single_pulse_dec
Sample_id	= MS0630-200-24h
Solvent	= CHLOROFORM-D
Creation_time	= 14-DEC-2018 14:23:49
Revision_time	= 14-DEC-2018 13:57:48
Current_time	= 14-DEC-2018 13:57:48
Data_format	= 1D COMPLEX
Dim_size	= 26214
Dim_title	= 13C
Dim_units	= [ppm]
Dimensions	= X
Site	= ECA 500
Spectrometer	= JNM-ECA500
Field_strength	= 11.7473579[T] (500[MH
X_acq_duration	= 0.83361792[s]
X_domain	= 13C
X_freq	= 125.76529768[MHz]
X_offset	= 100[ppm]
X_points	= 32768
X_prescans	= 4
X_resolution	= 1.19959034[Hz]
X_sweep	= 39.3081761[kHz]
Irr_domain	= 1H
Irr_freq	= 500.15991521[MHz]
Irr_offset	= 5.0[ppm]
Clipped	= FALSE
Mod_return	= 1
Scans	= 256
Total_scans	= 256
X_90_width	= 13.2[us]
X_acq_time	= 0.83361792[s]
X_angle	= 30[deg]
X_atn	= 6[dB]
X_pulse	= 4.4[us]
Irr_atn_dec	= 20.7[dB]
Irr_atn_noe	= 20.7[dB]
Irr_noise	= WALTZ
Decoupling	= TRUE
Initial_wait	= 1[s]
Noe	= TRUE
Noe_time	= 2[s]
Recvr_gain	= 60
Relaxation_delay	= 2[s]
Repetition_time	= 2.83361792[s]
Temp_get	= 20.2[dC]

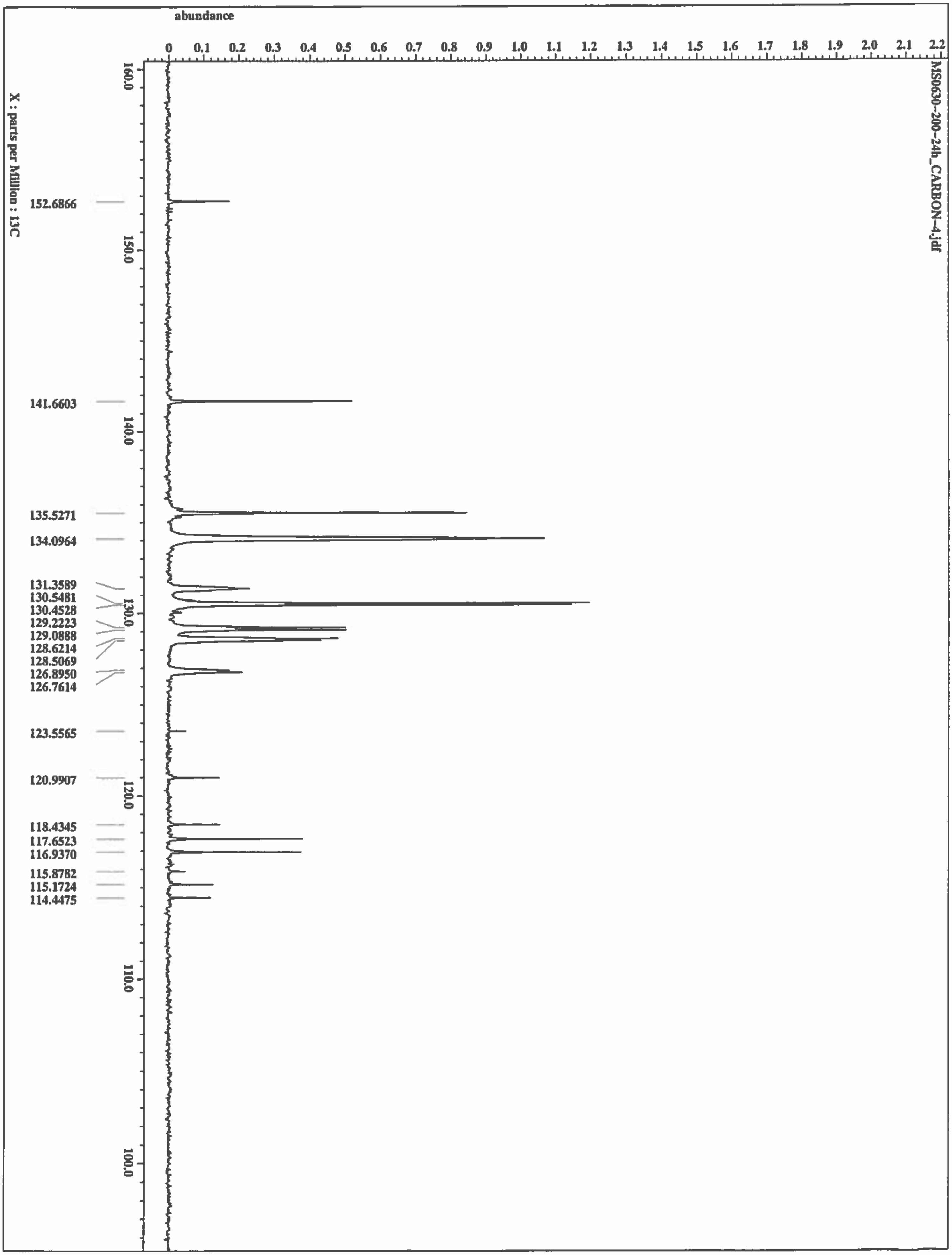

abundance
0
0.1
0.2
0.3
0.4
0.5
0.6
0.7
0.8
0.9
1.0
1.1
1.2
1.3
1.4
1.5
1.6
1.7
1.8
1.9
2.0
2.1
2.2
MS0630-200-24h_CARBON-4.jdf
X : parts per Million : 13C
160.0
150.0
140.0
130.0
120.0
110.0
100.0
152.6866
141.6603
135.5271
134.0964
131.3589
130.5481
130.4528
129.2223
129.0888
128.6214
128.5069
126.8950
126.7614
123.5565
120.9907
118.4345
117.6523
116.9370
115.8782
115.1724
114.4475

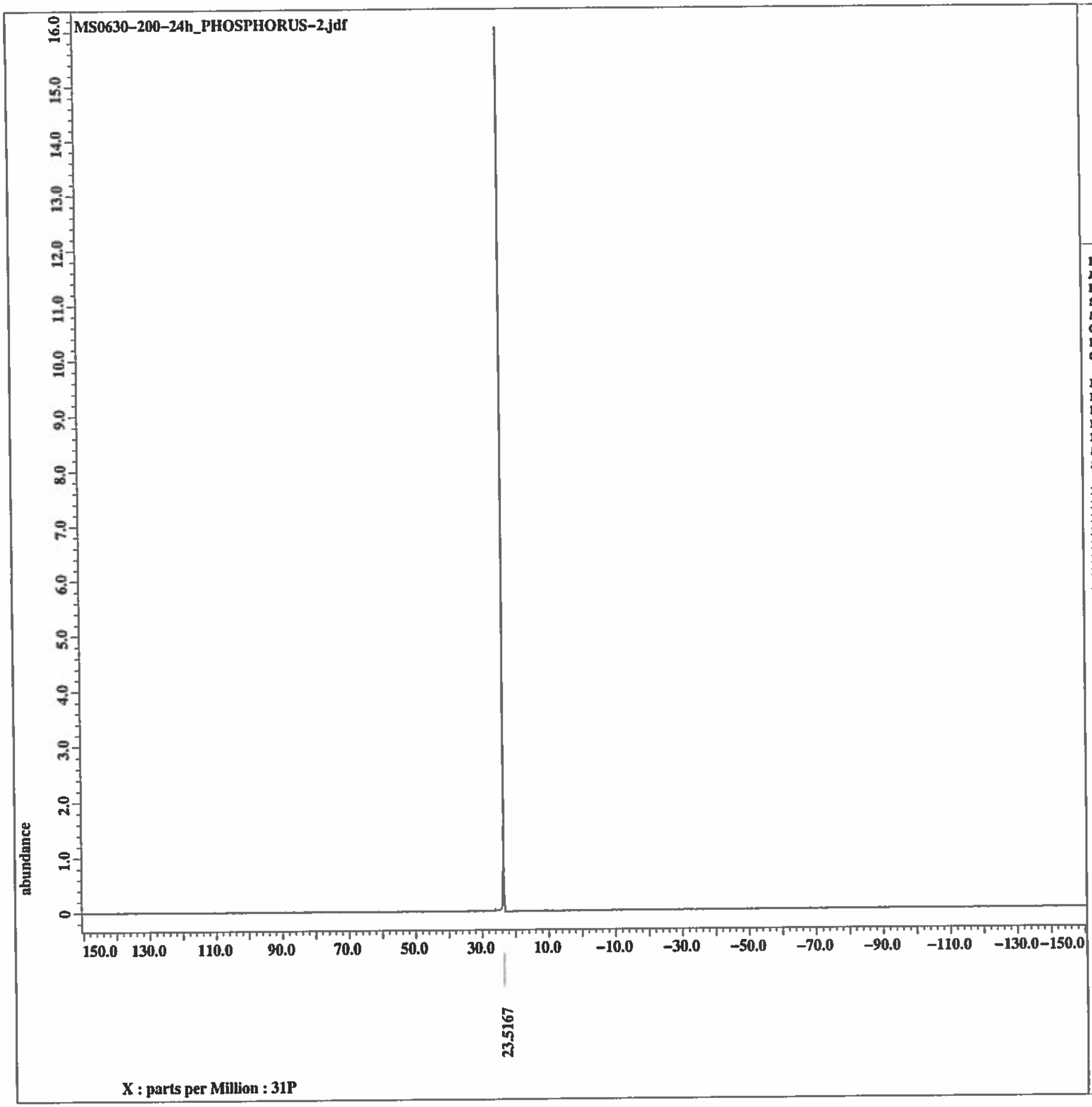


```
Filename         = MS0630-200-24h_PHOSPH
Author           = Jim Davis
Experiment       = single_pulse_dec
Sample_id        = MS0630-200-24h
Solvent          = CHLOROFORM-D
Creation_time    = 14-DEC-2018 14:29:22
Revision_time    = 14-DEC-2018 14:03:21
Current_time     = 14-DEC-2018 14:03:21

Data_format      = 1D COMPLEX
Dim_size         = 52428
Dim_title        = 31P
Dim_units        = [ppm]
Dimensions       = X
Site             = ECA 500
Spectrometer     = JNM-ECA500

Field_strength   = 11.7473579[T] (500[MH
X_acq_duration   = 0.85983232[s]
X_domain         = 31P
X_freq           = 202.46831075[MHz]
X_offset         = 0[ppm]
X_points         = 65536
X_prescans       = 4
X_resolution     = 1.16301746[Hz]
X_sweep          = 76.2195122[kHz]
Irr_domain       = 1H
Irr_freq         = 500.15991521[MHz]
Irr_offset       = 5.0[ppm]
Clipped          = FALSE
Mod_return       = 1
Scans            = 60
Total_scans      = 60

X_90_width       = 14.687[us]
X_acq_time       = 0.85983232[s]
X_angle          = 30[deg]
X_atn            = 5[dB]
X_pulse          = 4.89566667[us]
Irr_atn_dec      = 20.7[dB]
Irr_atn_noe      = 20.7[dB]
Irr_noise        = WALTZ
Decoupling       = TRUE
Initial_wait     = 1[s]
Noe              = TRUE
Noe_time         = 2[s]
Recvr_gain       = 54
Relaxation_delay = 2[s]
Repetition_time  = 2.85983232[s]
Temp_get         = 20.2[dC]
```

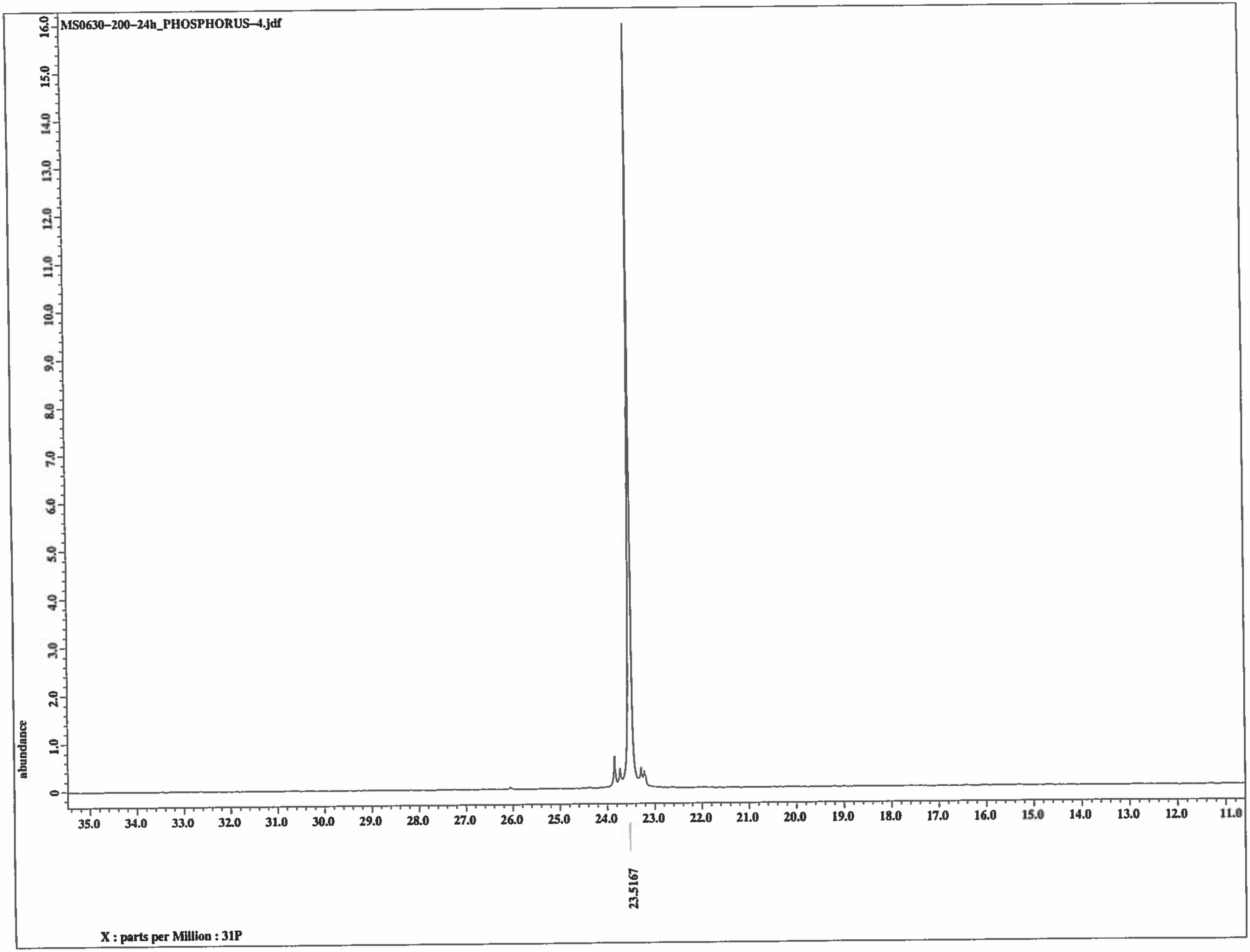
MS0630-200-24h_PHOSPHORUS-4.jdf
abundance
0
1.0
2.0
3.0
4.0
5.0
6.0
7.0
8.0
9.0
10.0
11.0
12.0
13.0
14.0
15.0
16.0
35.0
34.0
33.0
32.0
31.0
30.0
29.0
28.0
27.0
26.0
25.0
24.0
23.0
22.0
21.0
20.0
19.0
18.0
17.0
16.0
15.0
14.0
13.0
12.0
11.0
23.5167
X : parts per Million : 31P

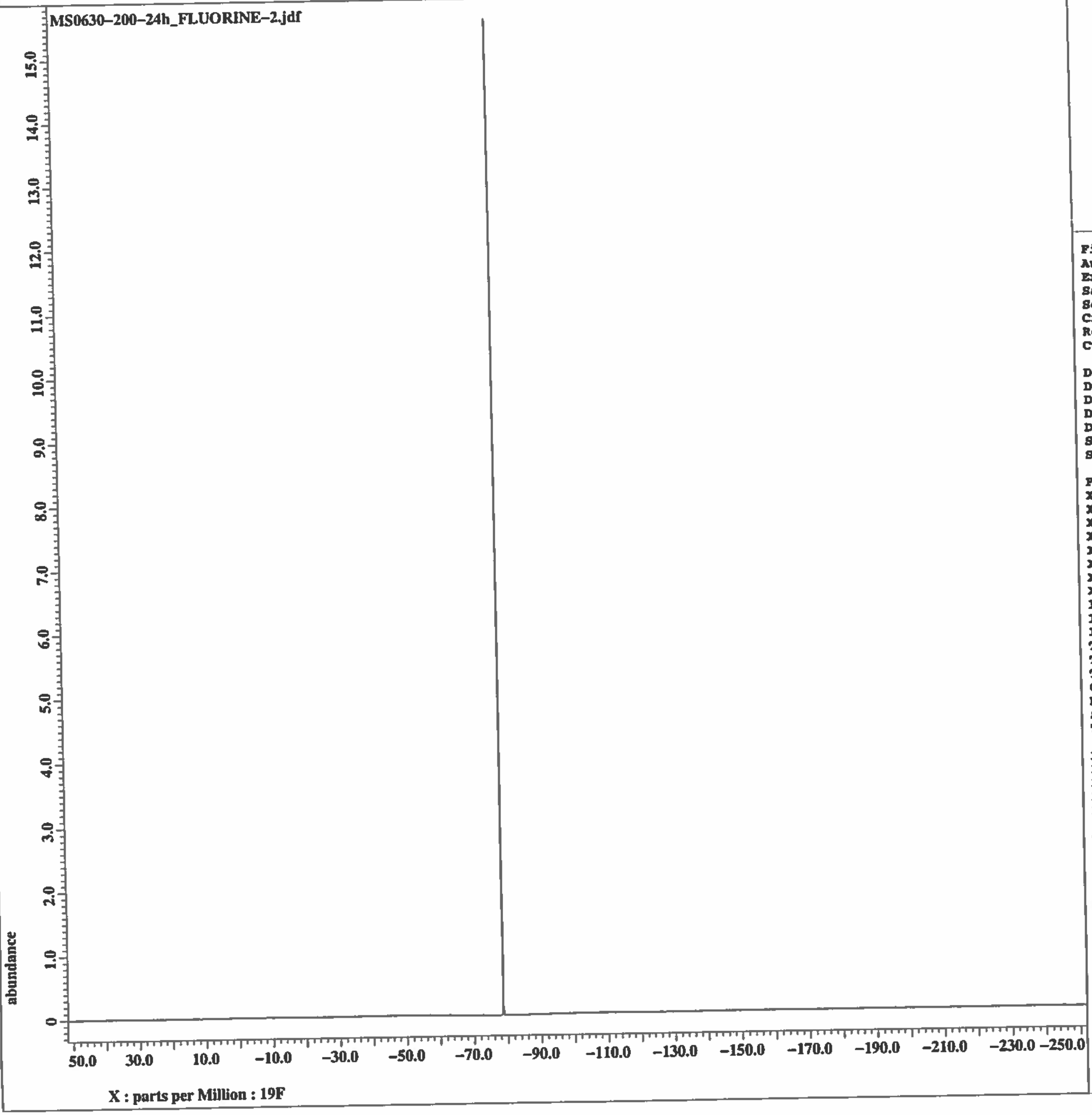

Filename	= MS0630-200-24h_FLUORI
Author	= Jim Davis
Experiment	= single_pulse.ex2
Sample_id	= MS0630-200-24h
Solvent	= CHLOROFORM-D
Creation_time	= 14-DEC-2018 14:36:28
Revision_time	= 14-DEC-2018 14:10:27
Current_time	= 14-DEC-2018 14:10:27
Data_format	= 1D COMPLEX
Dim_size	= 104857
Dim_title	= 19F
Dim_units	= [ppm]
Dimensions	= X
Site	= ECA 500
Spectrometer	= JNM-ECA500
Field_strength	= 11.7473579[T] (500[MH
X_acq_duration	= 0.7340032[s]
X_domain	= 19F
X_freq	= 470.62046084[MHz]
X_offset	= -100[ppm]
X_points	= 131072
X_prescans	= 1
X_resolution	= 1.36239188[Hz]
X_sweep	= 178.57142857[kHz]
Irr_domain	= 19F
Irr_freq	= 470.62046084[MHz]
Irr_offset	= 5[ppm]
Tri_domain	= 19F
Tri_freq	= 470.62046084[MHz]
Tri_offset	= 5[ppm]
Clipped	= FALSE
Mod_return	= 1
Scans	= 60
Total_scans	= 60
X_90_width	= 13.1[us]
X_acq_time	= 0.7340032[s]
X_angle	= 45[deg]
X_atn	= 2.5[dB]
X_pulse	= 6.55[us]
Irr_mode	= Off
Tri_mode	= Off
Dante_presat	= FALSE
Initial_wait	= 1[s]
Recvr_gain	= 70
Relaxation_delay	= 4[s]
Repetition_time	= 4.7340032[s]
Temp_get	= 20.1[dC]

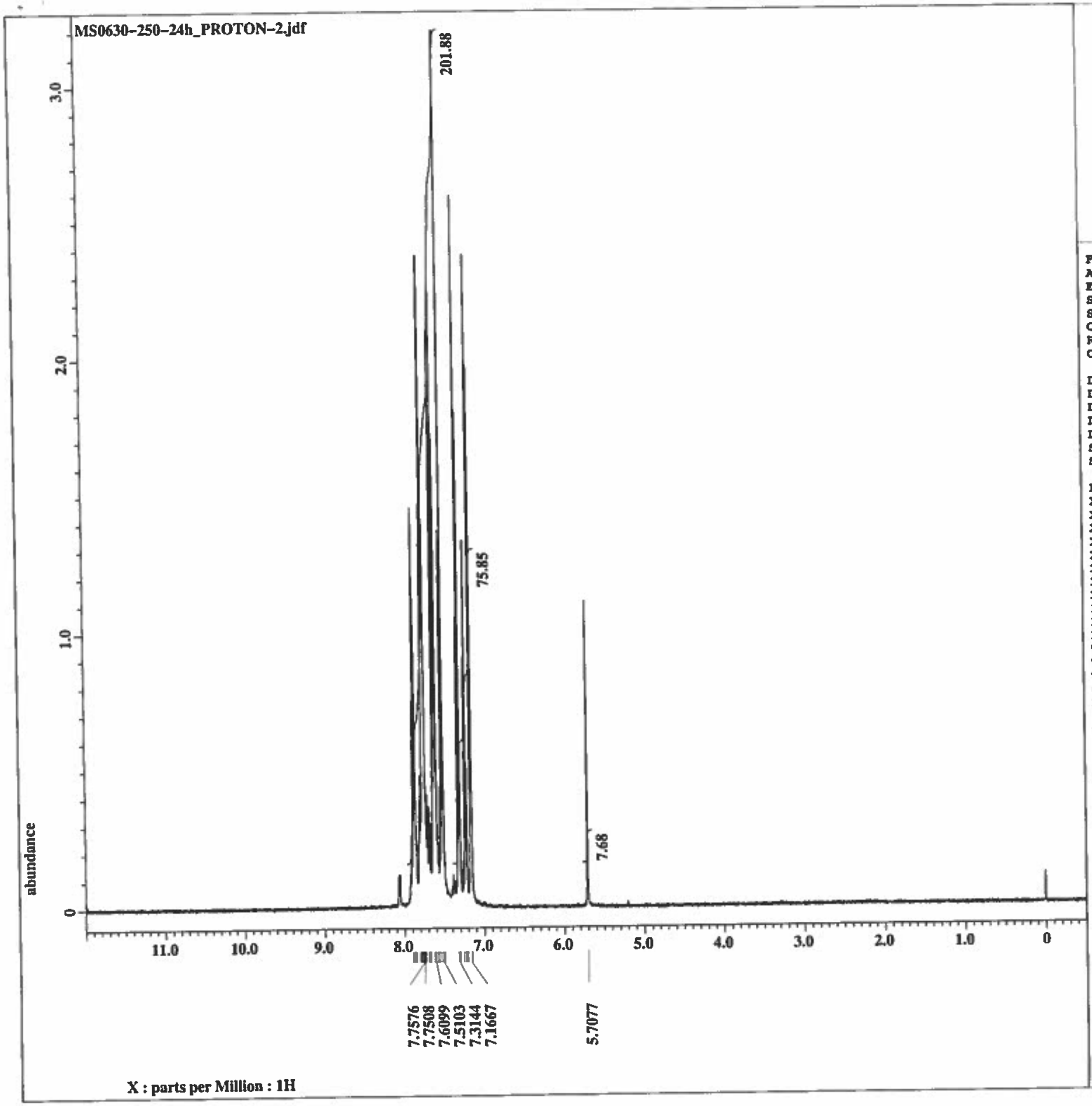

Filename	= MS0630-250-24h_PROTON
Author	= Jim Davis
Experiment	= single_pulse.ex2
Sample_id	= MS0630-250-24h
Solvent	= CHLOROFORM-D
Creation_time	= 14-DEC-2018 14:51:58
Revision_time	= 14-DEC-2018 14:25:57
Current_time	= 14-DEC-2018 14:25:57
Data_format	= 1D COMPLEX
Dim_size	= 13107
Dim_title	= 1H
Dim_units	= [ppm]
Dimensions	= X
Site	= ECA 500
Spectrometer	= JNM-ECA500
Field_strength	= 11.7473579[T] (500[MH
X_acq_duration	= 1.74587904[s]
X_domain	= 1H
X_freq	= 500.15991521[MHz]
X_offset	= 5.0[ppm]
X_points	= 16384
X_prescans	= 1
X_resolution	= 0.57277737[Hz]
X_sweep	= 9.38438438[kHz]
Irr_domain	= 1H
Irr_freq	= 500.15991521[MHz]
Irr_offset	= 5.0[ppm]
Tri_domain	= 1H
Tri_freq	= 500.15991521[MHz]
Tri_offset	= 5.0[ppm]
Clipped	= FALSE
Mod_return	= 1
Scans	= 16
Total_scans	= 16
X_90_width	= 12.4[us]
X_acq_time	= 1.74587904[s]
X_angle	= 45[deg]
X_atn	= 4[dB]
X_pulse	= 6.2[us]
Irr_mode	= Off
Tri_mode	= Off
Dante_presat	= FALSE
Initial_wait	= 1[s]
Recvr_gain	= 62
Relaxation_delay	= 4[s]
Repetition_time	= 5.74587904[s]
Temp_get	= 20.1[dC]

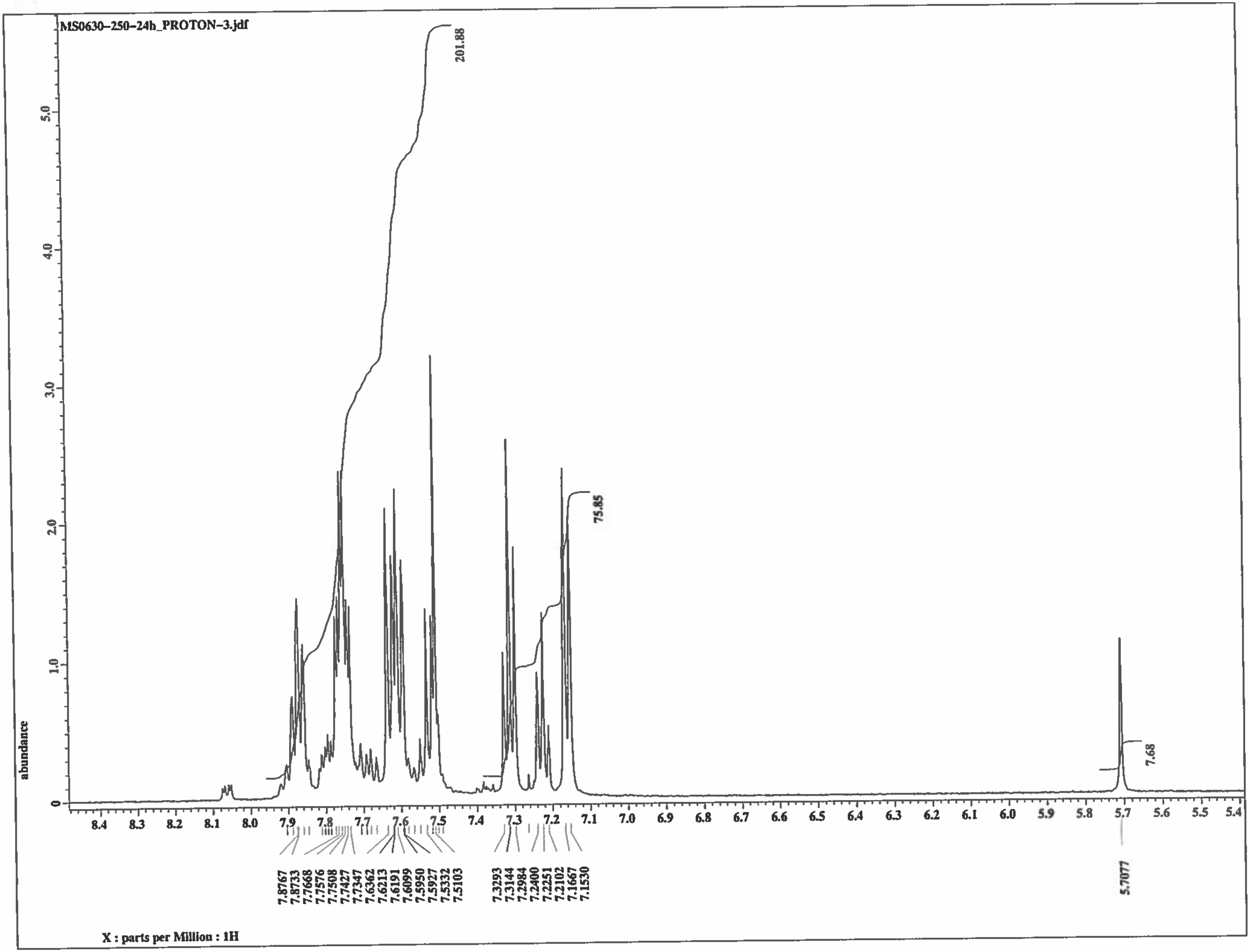

MS0630-250-24h_PROTON-3.jdf
abundance
X : parts per Million : 1H
201.88
75.85
7.68
7.8767
7.8733
7.7668
7.7576
7.7508
7.7427
7.7347
7.6362
7.6213
7.6191
7.6099
7.5950
7.5927
7.5332
7.5103
7.3293
7.3144
7.2984
7.2400
7.2251
7.2102
7.1667
7.1530
5.7077

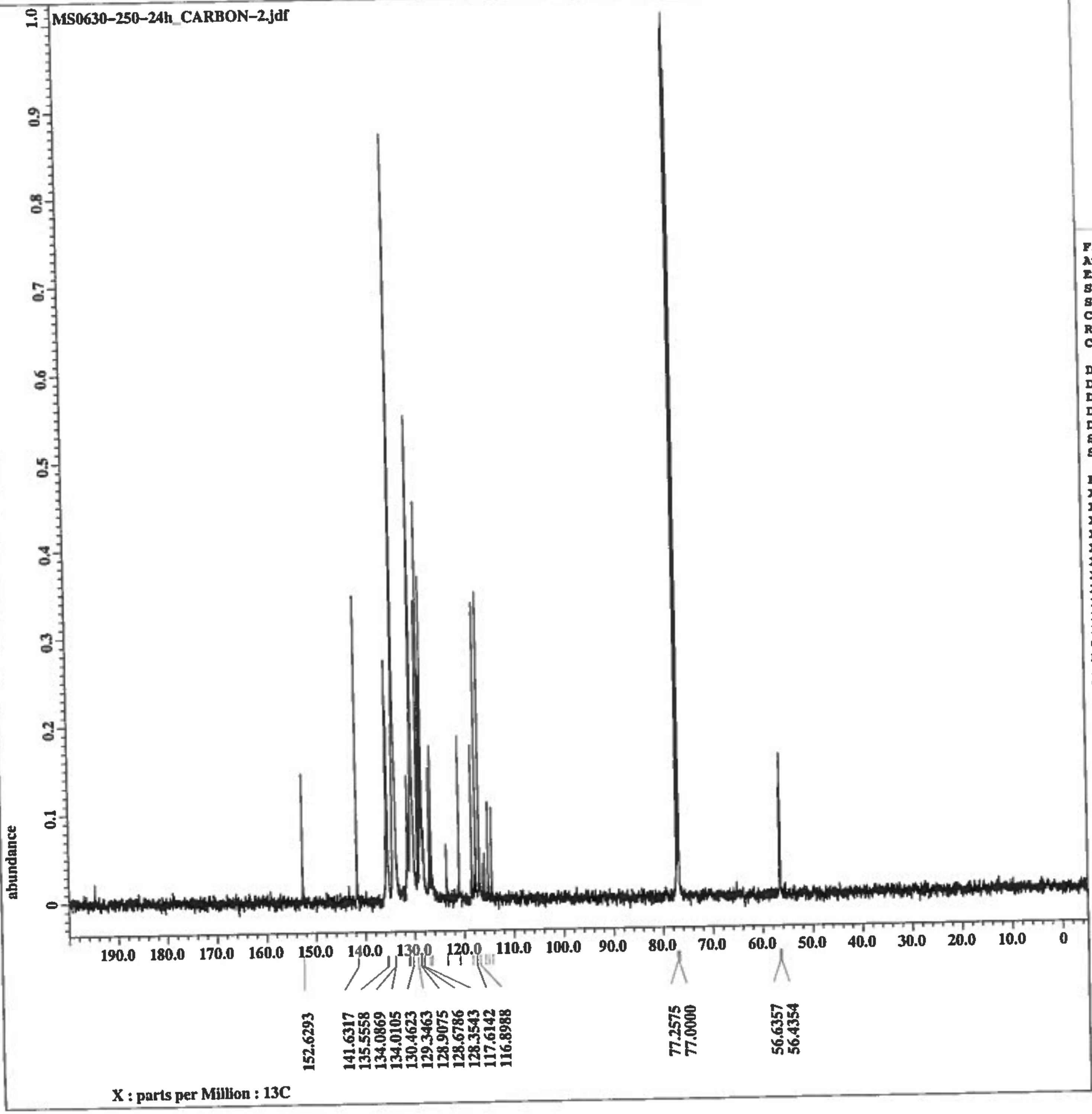

Filename	= MS0630-250-24h_CARBON
Author	= Jim Davis
Experiment	= single_pulse_dec
Sample_id	= MS0630-250-24h
Solvent	= CHLOROFORM-D
Creation_time	= 14-DEC-2018 15:07:36
Revision_time	= 14-DEC-2018 14:41:35
Current_time	= 14-DEC-2018 14:41:35
Data_format	= 1D COMPLEX
Dim_size	= 26214
Dim_title	= 13C
Dim_units	= [ppm]
Dimensions	= X
Site	= ECA 500
Spectrometer	= JNM-ECA500
Field_strength	= 11.7473579[T] (500[MH
X_acq_duration	= 0.83361792[s]
X_domain	= 13C
X_freq	= 125.76529768[MHz]
X_offset	= 100[ppm]
X_points	= 32768
X_prescans	= 4
X_resolution	= 1.19959034[Hz]
X_sweep	= 39.3081761[kHz]
Irr_domain	= 1H
Irr_freq	= 500.15991521[MHz]
Irr_offset	= 5.0[ppm]
Clipped	= FALSE
Mod_return	= 1
Scans	= 256
Total_scans	= 256
X_90_width	= 13.2[us]
X_acq_time	= 0.83361792[s]
X_angle	= 30[deg]
X_atn	= 6[dB]
X_pulse	= 4.4[us]
Irr_atn_dec	= 20.7[dB]
Irr_atn_noe	= 20.7[dB]
Irr_noise	= WALTZ
Decoupling	= TRUE
Initial_wait	= 1[s]
Noe	= TRUE
Noe_time	= 2[s]
Recvr_gain	= 60
Relaxation_delay	= 2[s]
Repetition_time	= 2.83361792[s]
Temp_get	= 20.2[dC]

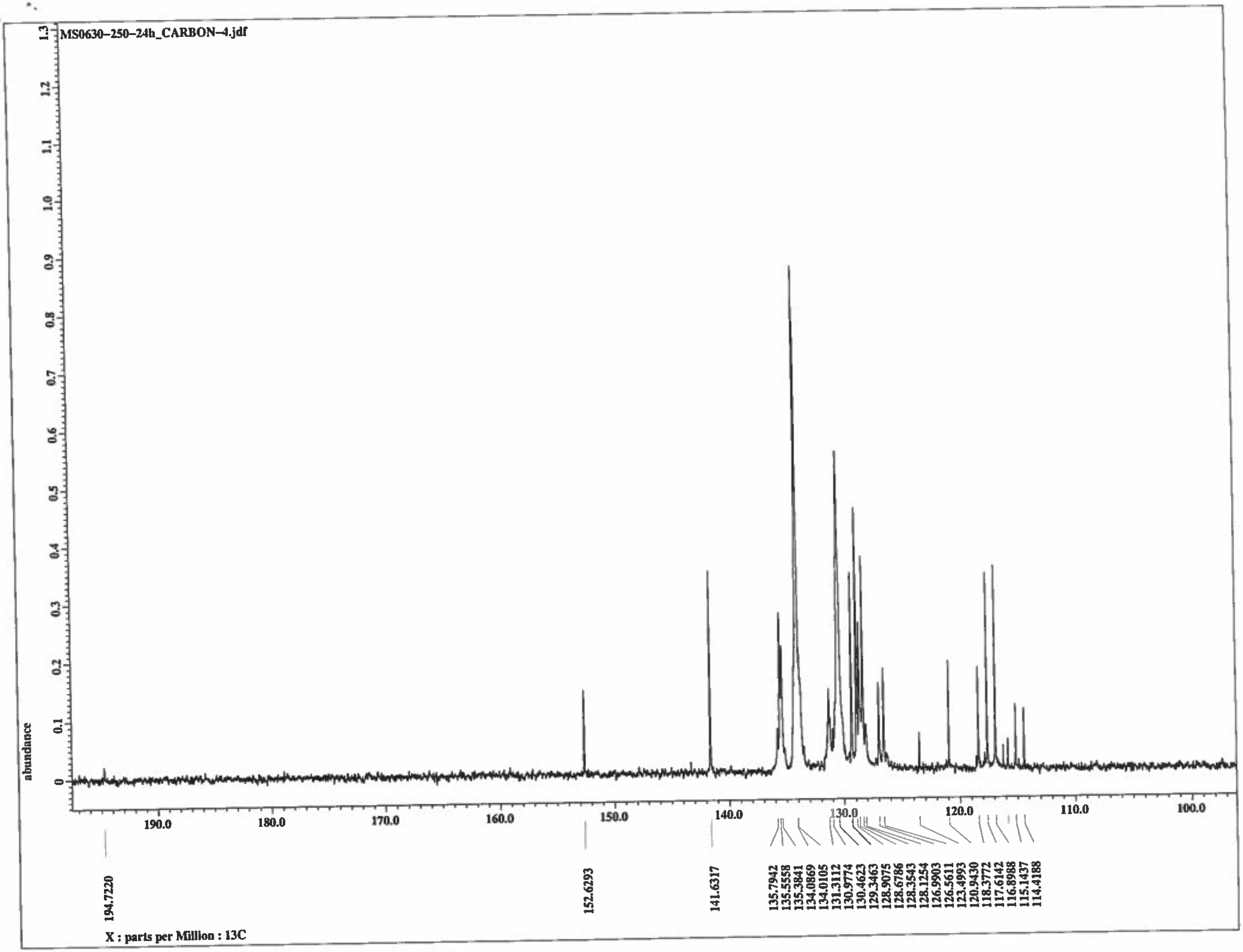

MS0630-250-24h_CARBON-4.jdf
abundance
0
0.1
0.2
0.3
0.4
0.5
0.6
0.7
0.8
0.9
1.0
1.1
1.2
1.3
190.0
180.0
170.0
160.0
150.0
140.0
130.0
120.0
110.0
100.0
194.7220
152.6293
141.6317
135.7942
135.5558
135.3841
134.0869
134.0105
131.3112
130.9774
130.4623
129.3463
128.9075
128.6786
128.3543
128.1254
126.9903
126.5611
123.4993
120.9430
118.3772
117.6142
116.8988
115.1437
114.4188
X : parts per Million : 13C

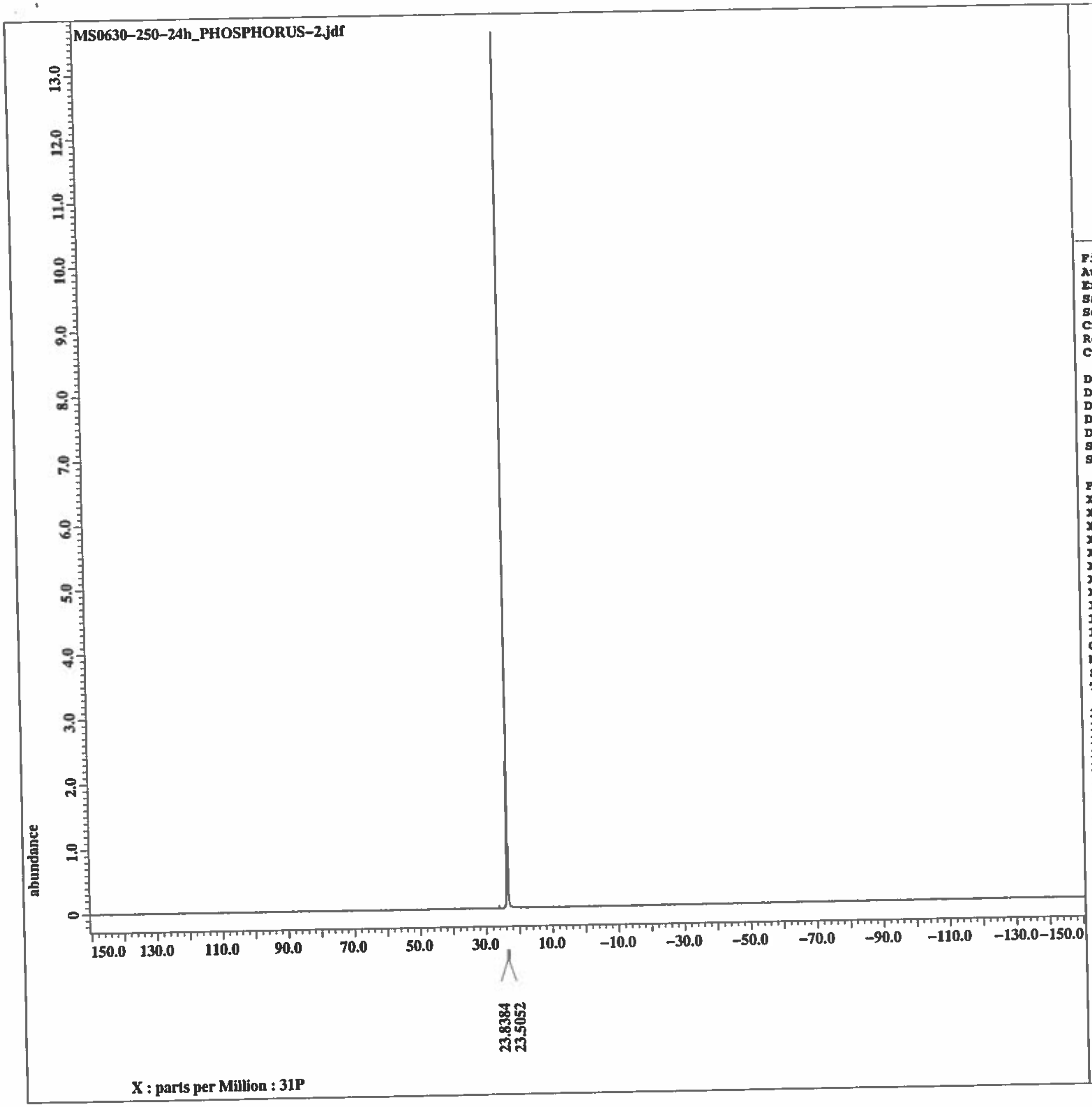

Filename	= MS0630-250-24h_PHOSPH
Author	= Jim Davis
Experiment	= single_pulse_dec
Sample_id	= MS0630-250-24h
Solvent	= CHLOROFORM-D
Creation_time	= 14-DEC-2018 15:12:45
Revision_time	= 14-DEC-2018 14:46:45
Current_time	= 14-DEC-2018 14:46:45
Data_format	= 1D COMPLEX
Dim_size	= 52428
Dim_title	= 31P
Dim_units	= [ppm]
Dimensions	= X
Site	= ECA 500
Spectrometer	= JNM-ECA500
Field_strength	= 11.7473579[T] (500[MH
X_acq_duration	= 0.85983232[s]
X_domain	= 31P
X_freq	= 202.46831075[MHz]
X_offset	= 0[ppm]
X_points	= 65536
X_prescans	= 4
X_resolution	= 1.16301746[Hz]
X_sweep	= 76.2195122[kHz]
Irr_domain	= 1H
Irr_freq	= 500.15991521[MHz]
Irr_offset	= 5.0[ppm]
Clipped	= FALSE
Mod_return	= 1
Scans	= 60
Total_scans	= 60
X_90_width	= 14.687[us]
X_acq_time	= 0.85983232[s]
X_angle	= 30[deg]
X_atn	= 5[dB]
X_pulse	= 4.89566667[us]
Irr_atn_dec	= 20.7[dB]
Irr_atn_noe	= 20.7[dB]
Irr_noise	= WALTZ
Decoupling	= TRUE
Initial_wait	= 1[s]
Noe	= TRUE
Noe_time	= 2[s]
Recvr_gain	= 54
Relaxation_delay	= 2[s]
Repetition_time	= 2.85983232[s]
Temp_get	= 20.3[dC]

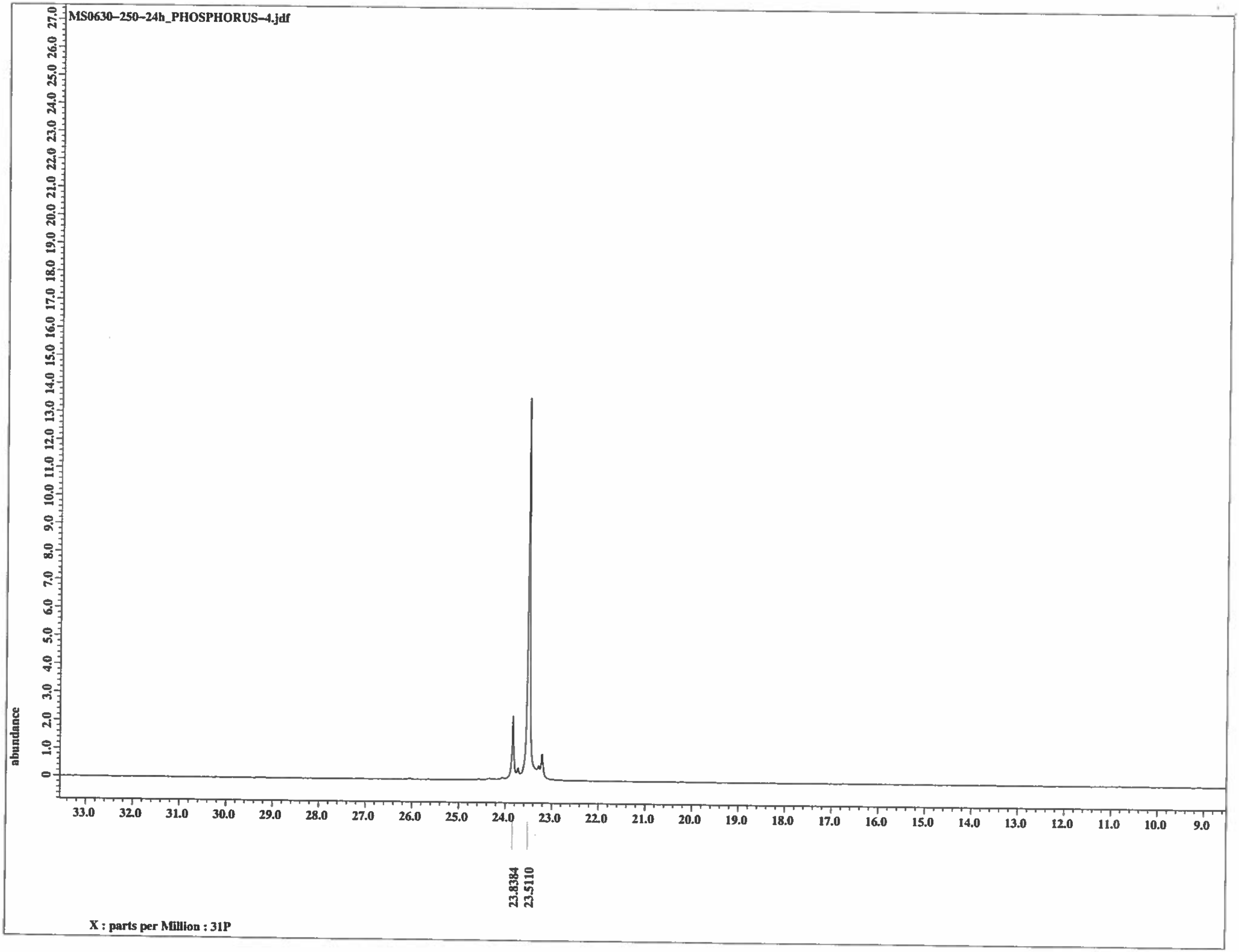
MS0630-250-24h_PHOSPHORUS-4.jdf
abundance
0
1.0
2.0
3.0
4.0
5.0
6.0
7.0
8.0
9.0
10.0
11.0
12.0
13.0
14.0
15.0
16.0
17.0
18.0
19.0
20.0
21.0
22.0
23.0
24.0
25.0
26.0
27.0
33.0
32.0
31.0
30.0
29.0
28.0
27.0
26.0
25.0
24.0
23.0
22.0
21.0
20.0
19.0
18.0
17.0
16.0
15.0
14.0
13.0
12.0
11.0
10.0
9.0
23.8384
23.5110
X : parts per Million : 31P

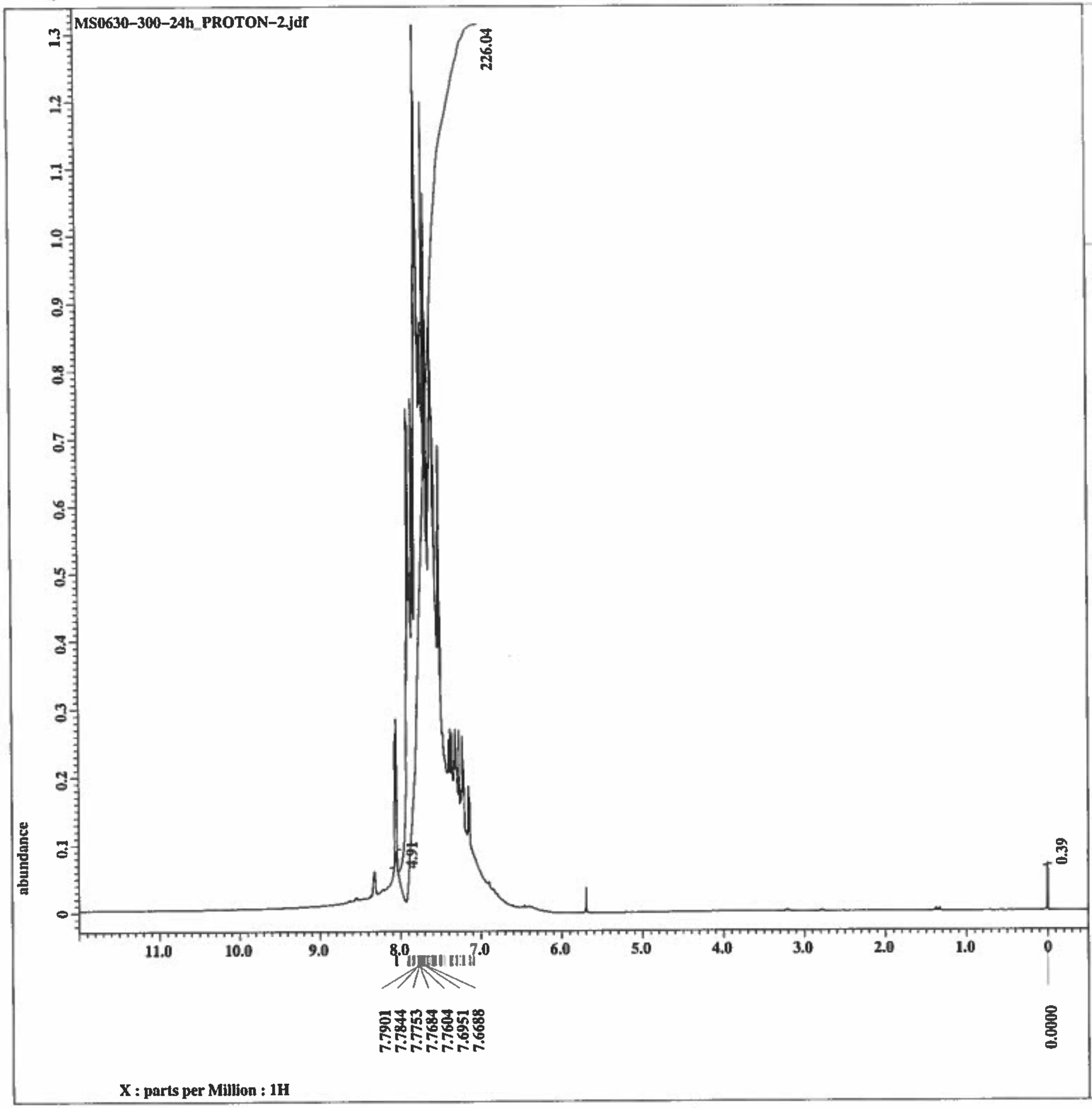

Filename	= MS0630-300-24h_PROTON
Author	= Jim Davis
Experiment	= single_pulse.ex2
Sample_id	= MS0630-300-24h
Solvent	= CHLOROFORM-D
Creation_time	= 14-DEC-2018 15:25:13
Revision_time	= 14-DEC-2018 14:59:11
Current_time	= 14-DEC-2018 14:59:11
Data_format	= 1D COMPLEX
Dim_size	= 13107
Dim_title	= 1H
Dim_units	= [ppm]
Dimensions	= X
Site	= ECA 500
Spectrometer	= JNM-ECA500
Field_strength	= 11.7473579[T] (500[MH
X_acq_duration	= 1.74587904[s]
X_domain	= 1H
X_freq	= 500.15991521[MHz]
X_offset	= 5.0[ppm]
X_points	= 16384
X_prescans	= 1
X_resolution	= 0.57277737[Hz]
X_sweep	= 9.38438438[kHz]
Irr_domain	= 1H
Irr_freq	= 500.15991521[MHz]
Irr_offset	= 5.0[ppm]
Tri_domain	= 1H
Tri_freq	= 500.15991521[MHz]
Tri_offset	= 5.0[ppm]
Clipped	= FALSE
Mod_return	= 1
Scans	= 16
Total_scans	= 16
X_90_width	= 12.4[us]
X_acq_time	= 1.74587904[s]
X_angle	= 45[deg]
X_atn	= 4[dB]
X_pulse	= 6.2[us]
Irr_mode	= Off
Tri_mode	= Off
Dante_presat	= FALSE
Initial_wait	= 1[s]
Recvr_gain	= 30
Relaxation_delay	= 4[s]
Repetition_time	= 5.74587904[s]
Temp_get	= 20.2[dC]

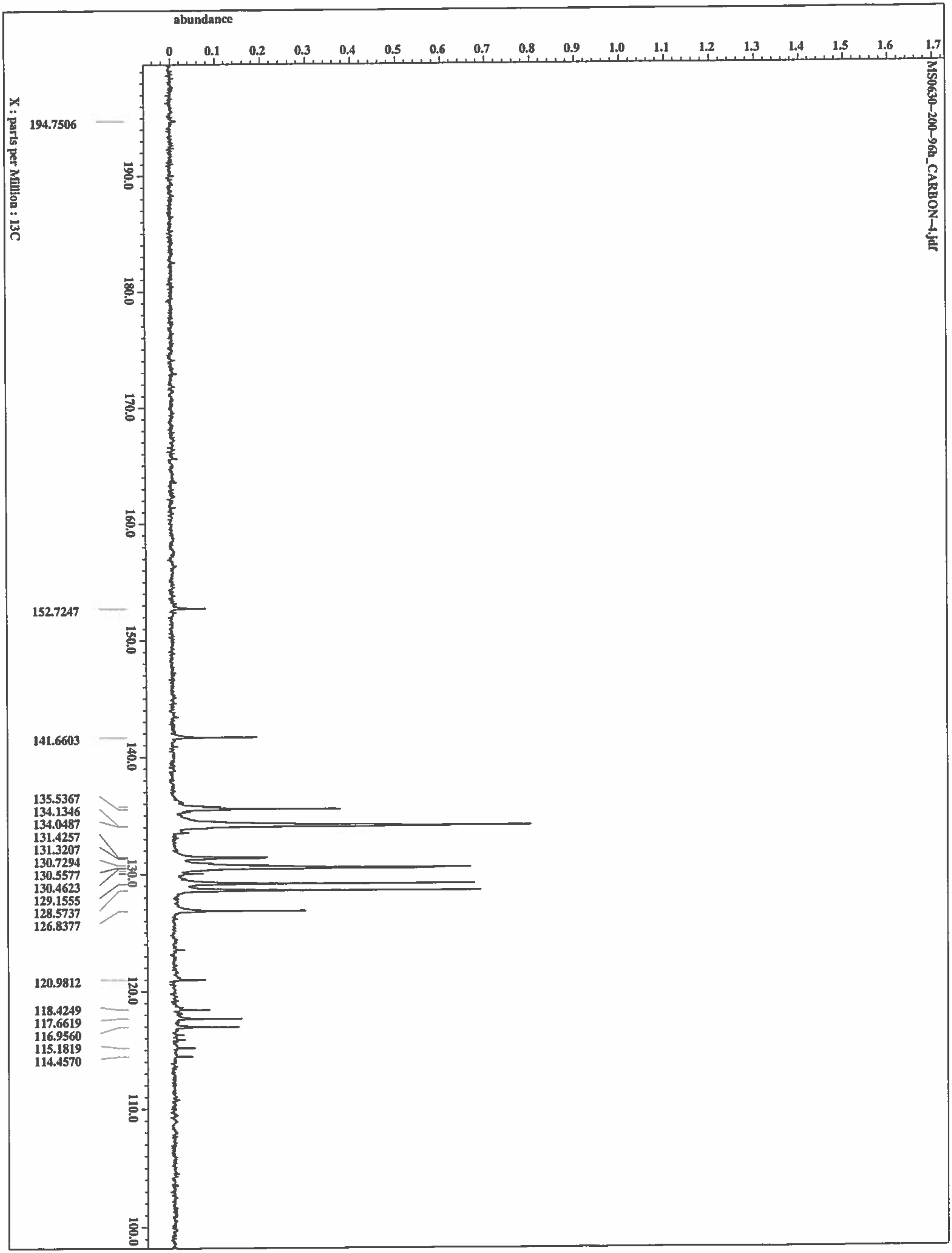

abundance
0
0.1
0.2
0.3
0.4
0.5
0.6
0.7
0.8
0.9
1.0
1.1
1.2
1.3
1.4
1.5
1.6
1.7
MS0630-200-96h_CARBON-4.jdf
X : parts per Million : 13C
194.7506
152.7247
141.6603
135.5367
134.1346
134.0487
131.4257
131.3207
130.7294
130.5577
130.4623
129.1555
128.5737
126.8377
120.9812
118.4249
117.6619
116.9560
115.1819
114.4570
190.0
180.0
170.0
160.0
150.0
140.0
130.0
120.0
110.0
100.0

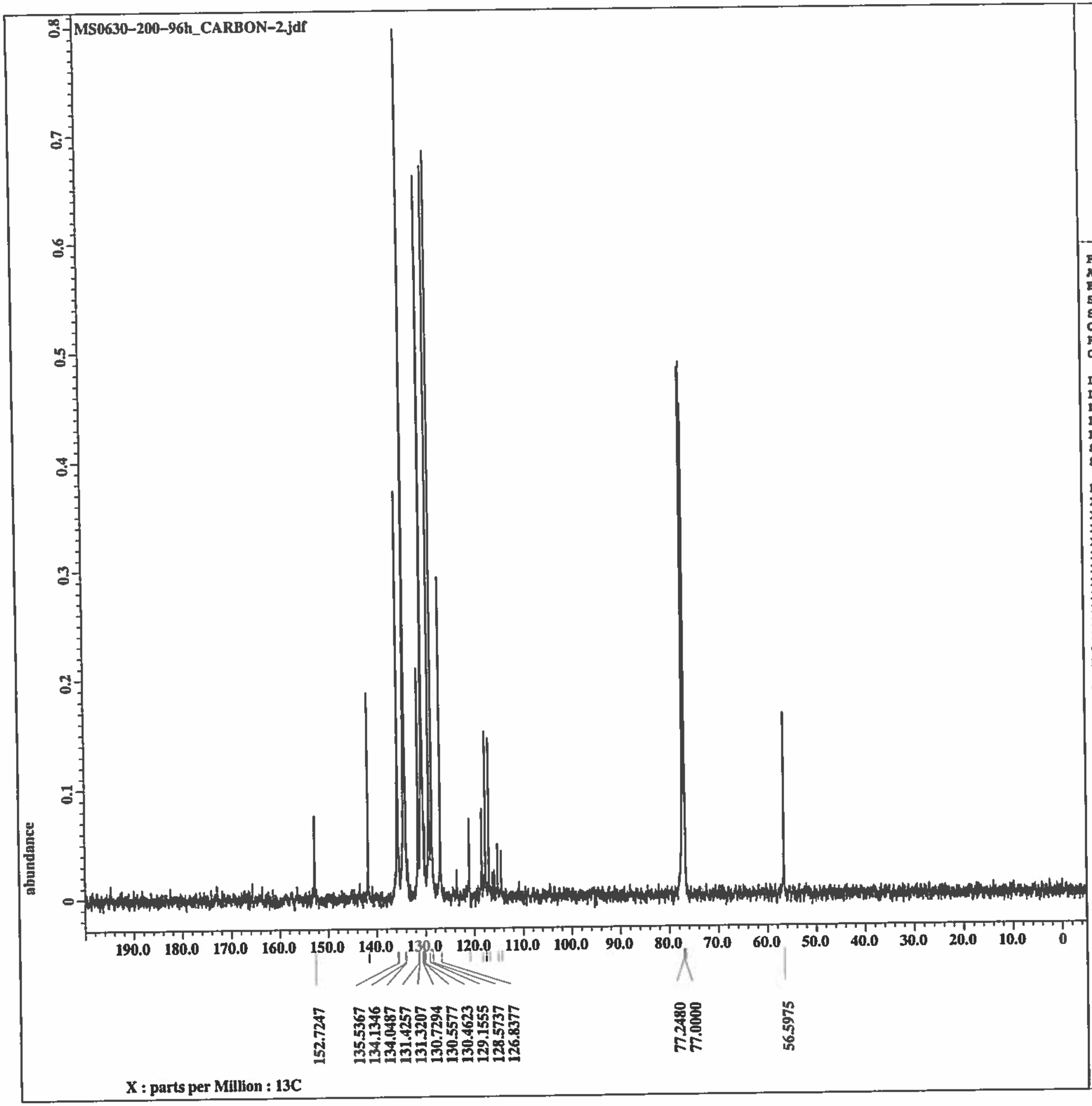

Filename	= MS0630-200-96h_CARBON
Author	= Jim Davis
Experiment	= single_pulse_dec
Sample_id	= MS0630-200-96h
Solvent	= CHLOROFORM-D
Creation_time	= 18-DEC-2018 09:34:34
Revision_time	= 18-DEC-2018 09:08:15
Current_time	= 18-DEC-2018 09:08:15
Data_format	= 1D COMPLEX
Dim_size	= 26214
Dim_title	= 13C
Dim_units	= [ppm]
Dimensions	= X
Site	= ECA 500
Spectrometer	= JNM-ECA500
Field_strength	= 11.7473579[T] (500[MH
X_acq_duration	= 0.83361792[s]
X_domain	= 13C
X_freq	= 125.76529768[MHz]
X_offset	= 100[ppm]
X_points	= 32768
X_prescans	= 4
X_resolution	= 1.19959034[Hz]
X_sweep	= 39.3081761[kHz]
Irr_domain	= 1H
Irr_freq	= 500.15991521[MHz]
Irr_offset	= 5.0[ppm]
Clipped	= FALSE
Mod_return	= 1
Scans	= 300
Total_scans	= 300
X_90_width	= 13.2[us]
X_acq_time	= 0.83361792[s]
X_angle	= 30[deg]
X_atn	= 6[dB]
X_pulse	= 4.4[us]
Irr_atn_dec	= 20.7[dB]
Irr_atn_noe	= 20.7[dB]
Irr_noise	= WALTZ
Decoupling	= TRUE
Initial_wait	= 1[s]
Noe	= TRUE
Noe_time	= 2[s]
Recvr_gain	= 60
Relaxation_delay	= 2[s]
Repetition_time	= 2.83361792[s]
Temp_get	= 20[dC]

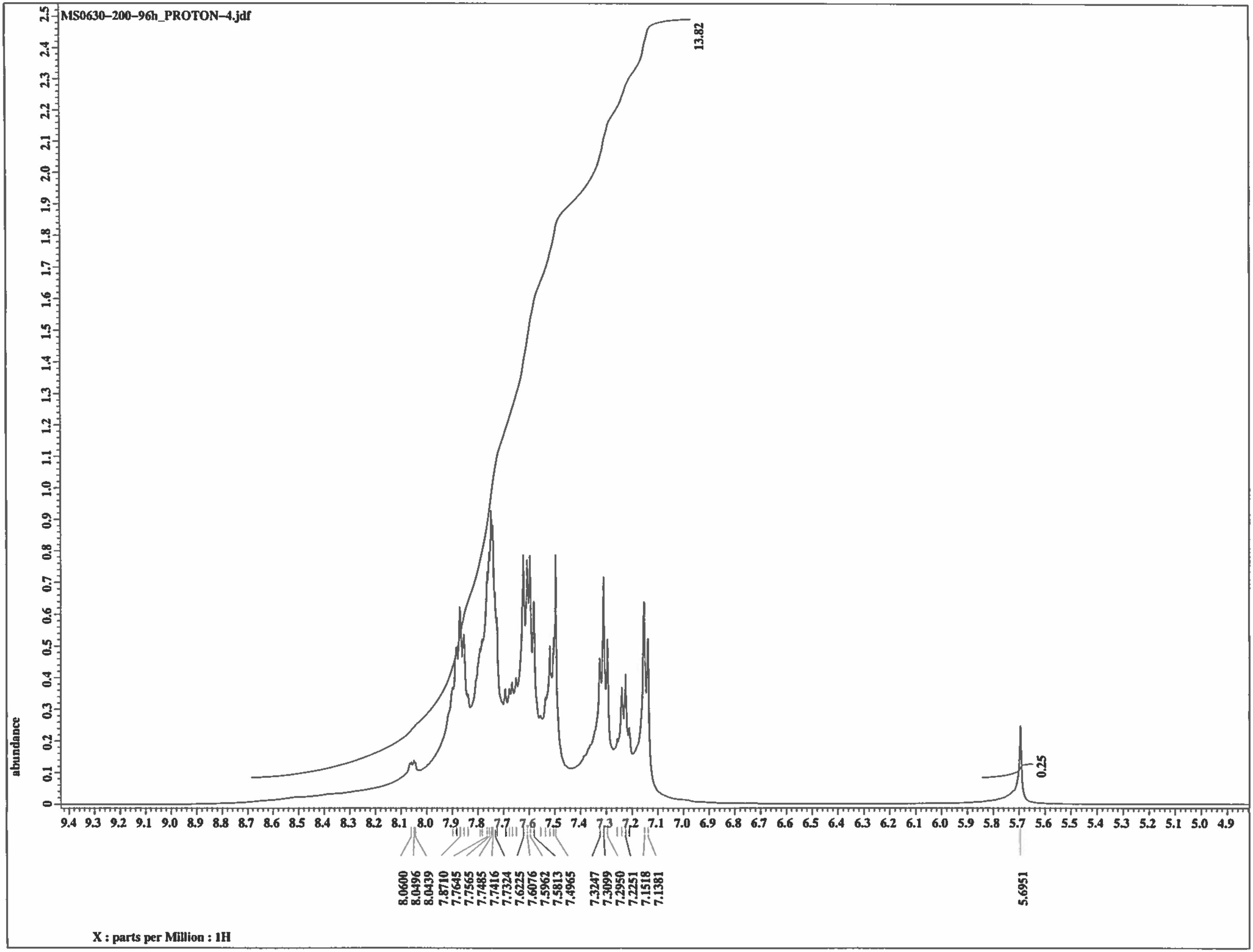
MS0630-200-96h_PROTON-4.jdf
abundance
13.82
0.25
8.0600
8.0496
8.0439
7.8710
7.7645
7.7565
7.7485
7.7416
7.7324
7.6225
7.6076
7.5962
7.5813
7.4965
7.3247
7.3099
7.2950
7.2251
7.1518
7.1381
5.6951
X : parts per Million : 1H

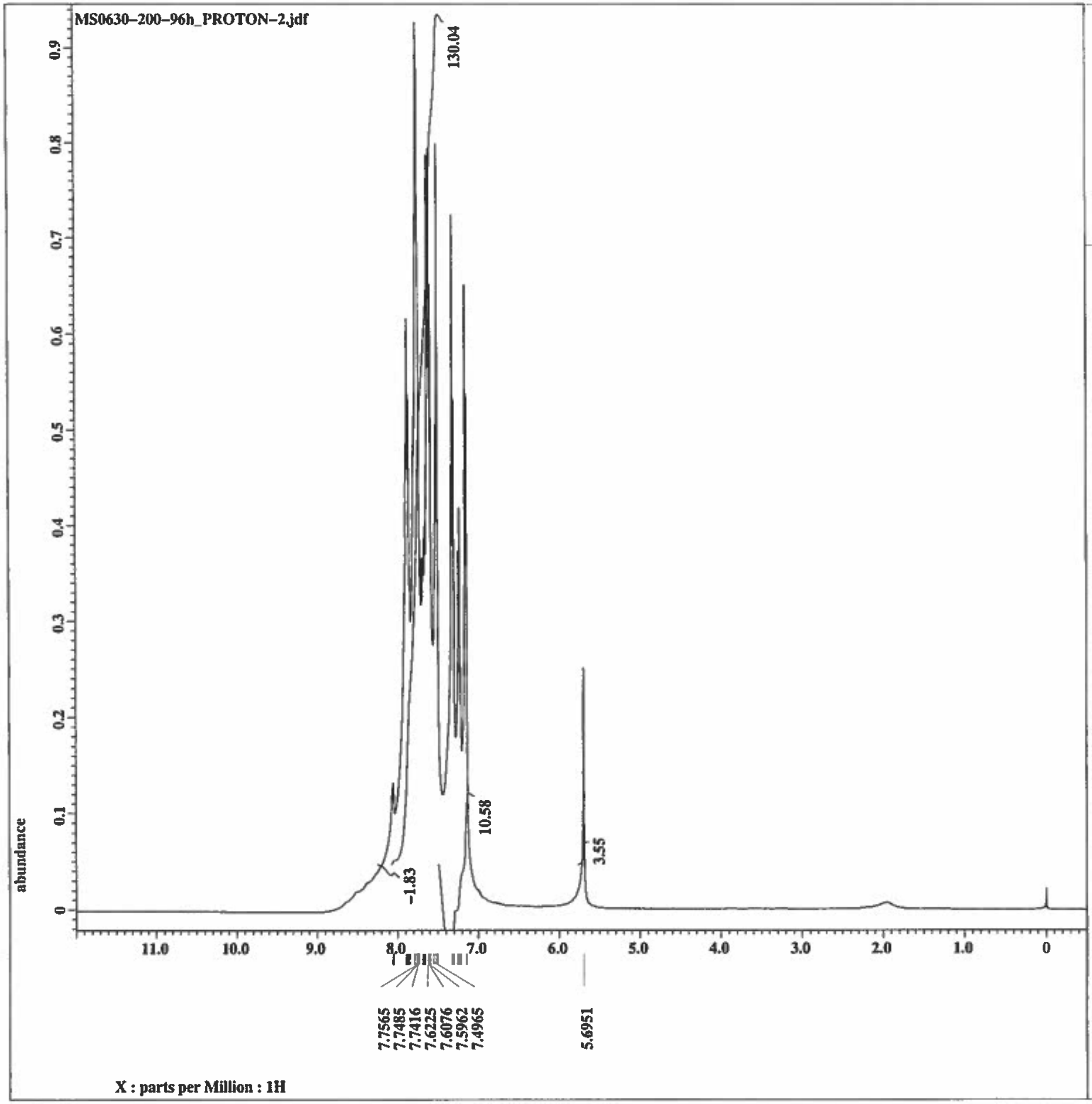

Filename	=	MS0630-200-96h_PROTON
Author	=	Jim Davis
Experiment	=	single_pulse.ex2
Sample_id	=	MS0630-200-96h
Solvent	=	CHLOROFORM-D
Creation_time	=	18-DEC-2018 09:17:38
Revision_time	=	18-DEC-2018 08:51:20
Current_time	=	18-DEC-2018 08:51:20
Data_format	=	1D COMPLEX
Dim_size	=	13107
Dim_title	=	1H
Dim_units	=	[ppm]
Dimensions	=	X
Site	=	ECA 500
Spectrometer	=	JNM-ECA500
Field_strength	=	11.7473579[T] (500[MH
X_acq_duration	=	1.74587904[s]
X_domain	=	1H
X_freq	=	500.15991521[MHz]
X_offset	=	5.0[ppm]
X_points	=	16384
X_prescans	=	1
X_resolution	=	0.57277737[Hz]
X_sweep	=	9.38438438[kHz]
Irr_domain	=	1H
Irr_freq	=	500.15991521[MHz]
Irr_offset	=	5.0[ppm]
Tri_domain	=	1H
Tri_freq	=	500.15991521[MHz]
Tri_offset	=	5.0[ppm]
Clipped	=	FALSE
Mod_return	=	1
Scans	=	16
Total_scans	=	16
X_90_width	=	12.4[us]
X_acq_time	=	1.74587904[s]
X_angle	=	45[deg]
X_atn	=	4[dB]
X_pulse	=	6.2[us]
Irr_mode	=	Off
Tri_mode	=	Off
Dante_presat	=	FALSE
Initial_wait	=	1[s]
Recvr_gain	=	32
Relaxation_delay	=	4[s]
Repetition_time	=	5.74587904[s]
Temp_get	=	19.3[dC]

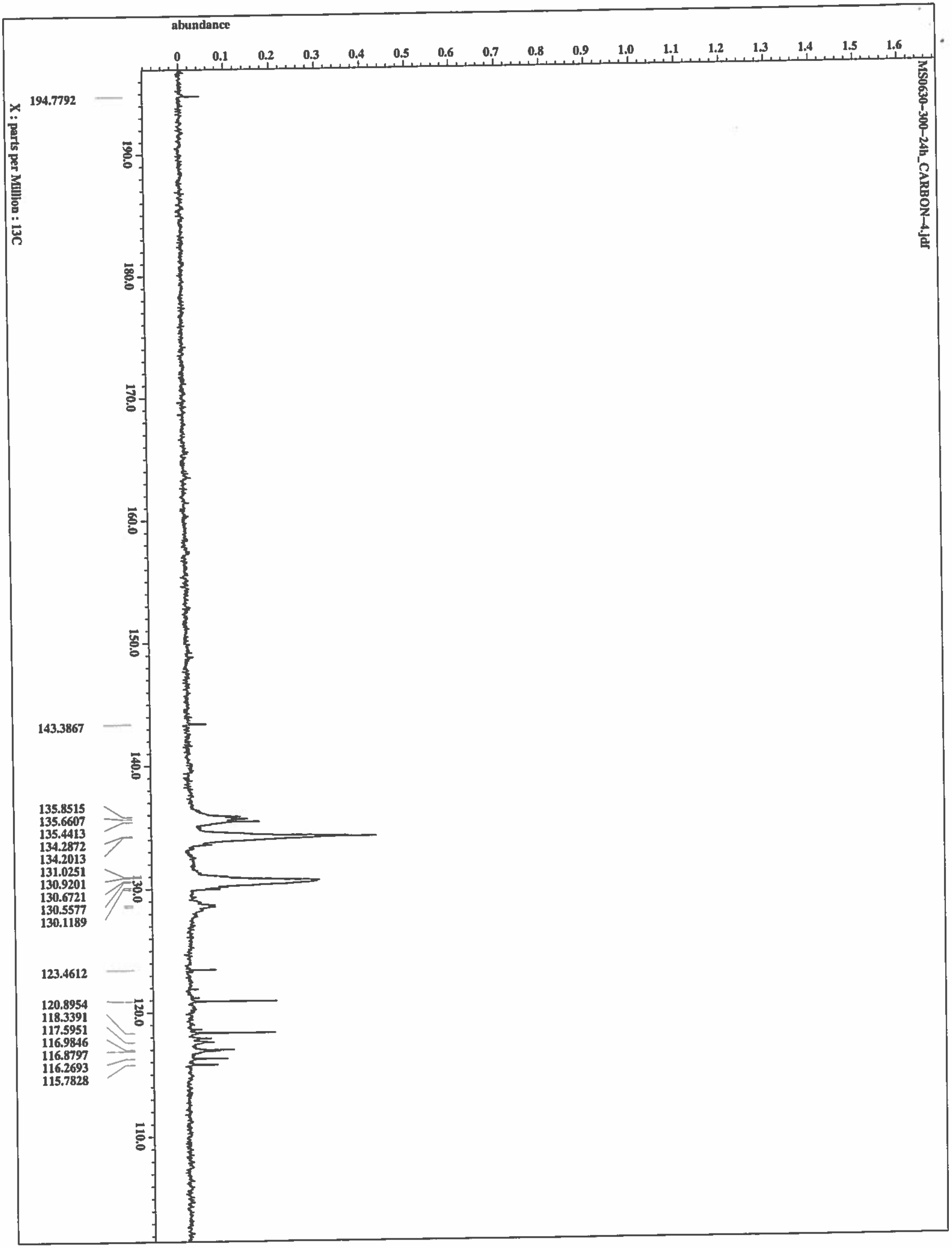
abundance
0
0.1
0.2
0.3
0.4
0.5
0.6
0.7
0.8
0.9
1.0
1.1
1.2
1.3
1.4
1.5
1.6
MS0630-300-24h_CARBON-4.jdf
X : parts per Million : 13C
190.0
180.0
170.0
160.0
150.0
140.0
130.0
120.0
110.0
194.7792
143.3867
135.8515
135.6607
135.4413
134.2872
134.2013
131.0251
130.9201
130.6721
130.5577
130.1189
123.4612
120.8954
118.3391
117.5951
116.9846
116.8797
116.2693
115.7828

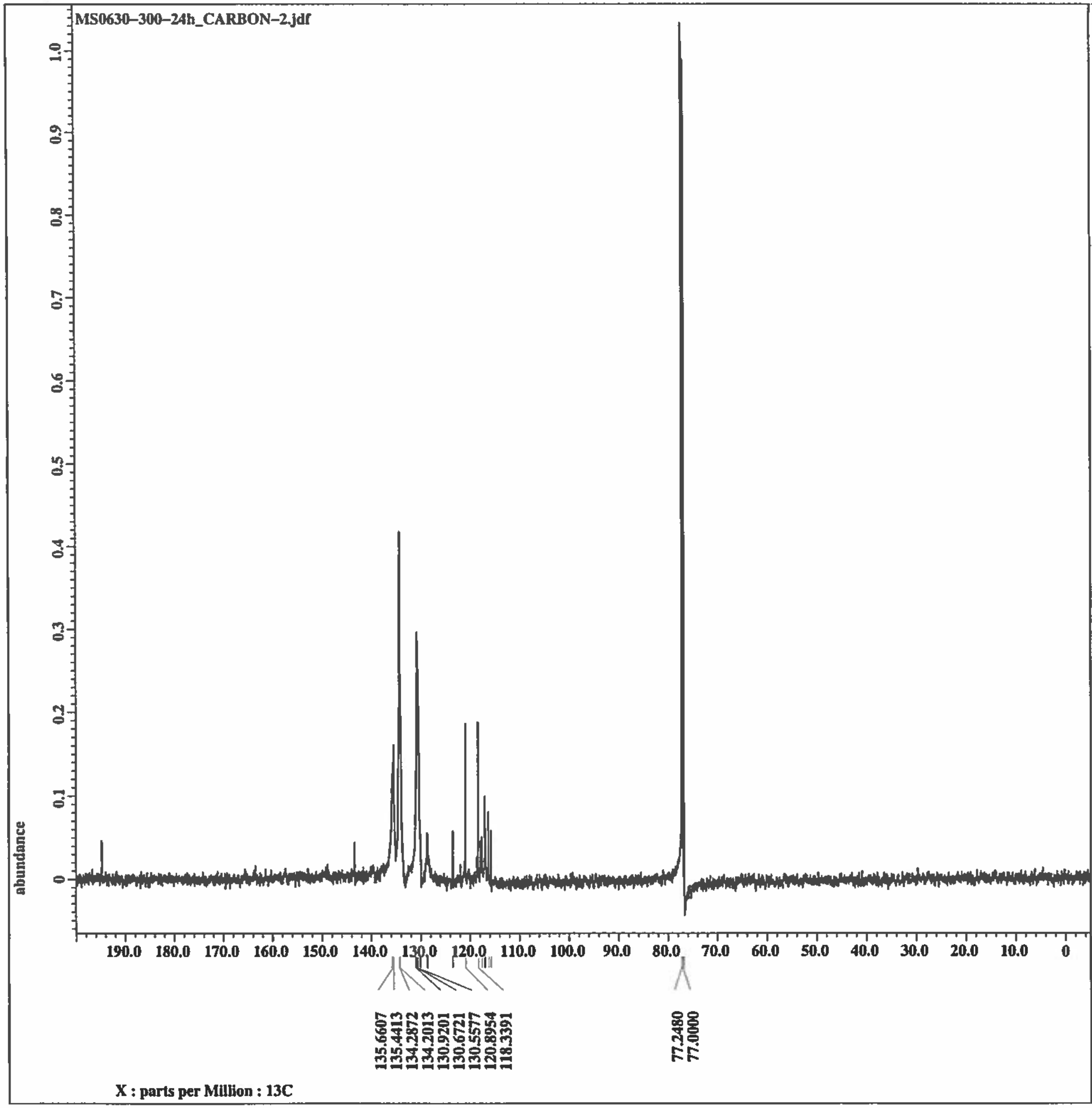

Filename	= MS0630-300-24h_CARBON
Author	= Jim Davis
Experiment	= single_pulse_dec
Sample_id	= MS0630-300-24h
Solvent	= CHLOROFORM-D
Creation_time	= 14-DEC-2018 15:39:46
Revision_time	= 14-DEC-2018 15:13:45
Current_time	= 14-DEC-2018 15:13:45
Data_format	= 1D COMPLEX
Dim_size	= 26214
Dim_title	= 13C
Dim_units	= [ppm]
Dimensions	= X
Site	= ECA 500
Spectrometer	= JNM-ECA500
Field_strength	= 11.7473579[T] (500[MH
X_acq_duration	= 0.83361792[s]
X_domain	= 13C
X_freq	= 125.76529768[MHz]
X_offset	= 100[ppm]
X_points	= 32768
X_prescans	= 4
X_resolution	= 1.19959034[Hz]
X_sweep	= 39.3081761[kHz]
Irr_domain	= 1H
Irr_freq	= 500.15991521[MHz]
Irr_offset	= 5.0[ppm]
Clipped	= FALSE
Mod_return	= 1
Scans	= 256
Total_scans	= 256
X_90_width	= 13.2[us]
X_acq_time	= 0.83361792[s]
X_angle	= 30[deg]
X_atn	= 6[dB]
X_pulse	= 4.4[us]
Irr_atn_dec	= 20.7[dB]
Irr_atn_noe	= 20.7[dB]
Irr_noise	= WALTZ
Decoupling	= TRUE
Initial_wait	= 1[s]
Noe	= TRUE
Noe_time	= 2[s]
Recvr_gain	= 60
Relaxation_delay	= 2[s]
Repetition_time	= 2.83361792[s]
Temp_get	= 20.3[dC]

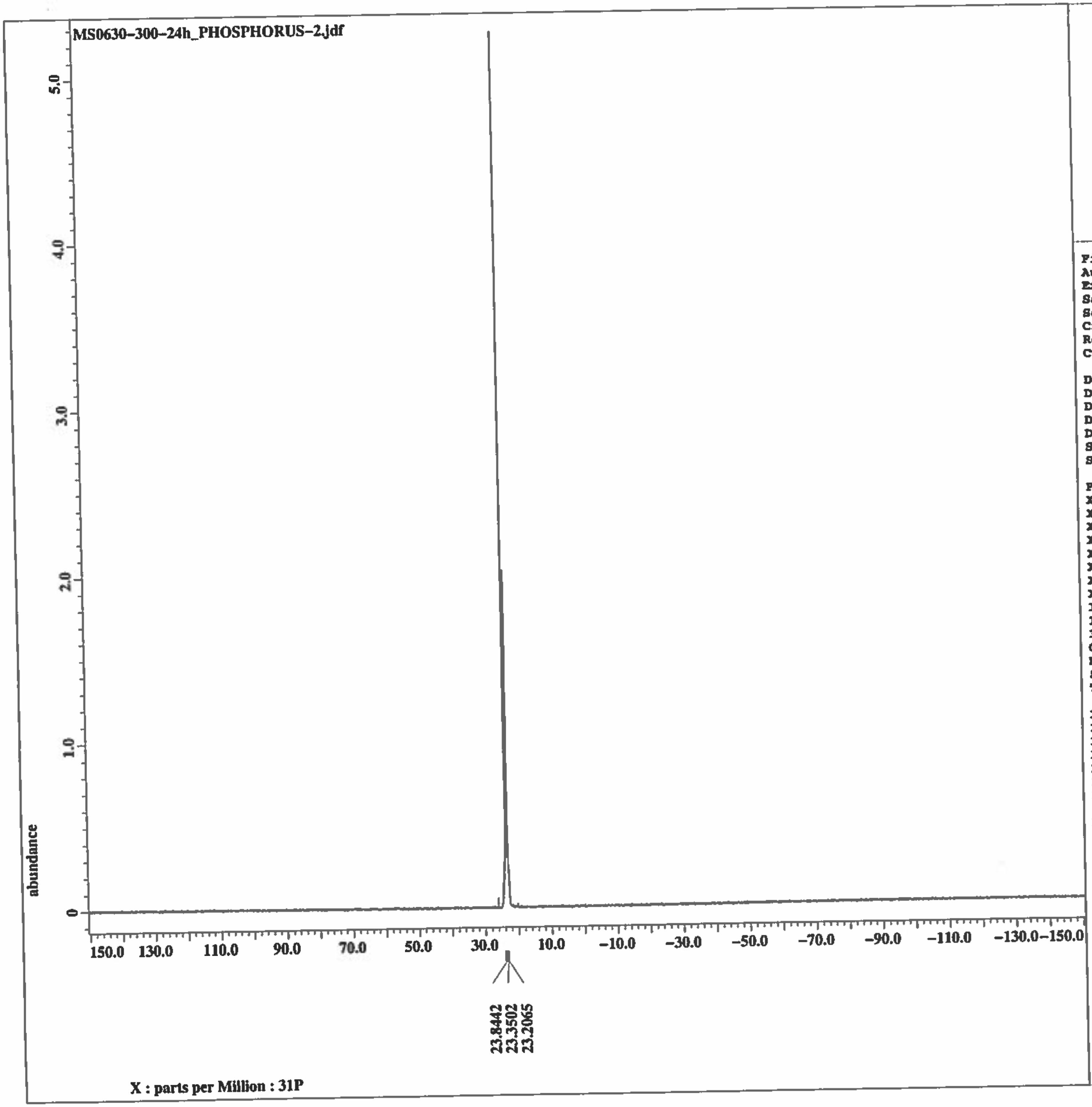

Filename	= MS0630-300-24h_PHOSPH
Author	= Jim Davis
Experiment	= single_pulse_dec
Sample_id	= MS0630-300-24h
Solvent	= CHLOROFORM-D
Creation_time	= 14-DEC-2018 15:22:02
Revision_time	= 14-DEC-2018 14:56:01
Current_time	= 14-DEC-2018 14:56:01
Data_format	= 1D COMPLEX
Dim_size	= 52428
Dim_title	= 31P
Dim_units	= [ppm]
Dimensions	= X
Site	= ECA 500
Spectrometer	= JNM-ECA500
Field_strength	= 11.7473579[T] (500[MH
X_acq_duration	= 0.85983232[s]
X_domain	= 31P
X_freq	= 202.46831075[MHz]
X_offset	= 0[ppm]
X_points	= 65536
X_prescans	= 4
X_resolution	= 1.16301746[Hz]
X_sweep	= 76.2195122[kHz]
Irr_domain	= 1H
Irr_freq	= 500.15991521[MHz]
Irr_offset	= 5.0[ppm]
Clipped	= FALSE
Mod_return	= 1
Scans	= 60
Total_scans	= 60
X_90_width	= 14.687[us]
X_acq_time	= 0.85983232[s]
X_angle	= 30[deg]
X_atn	= 5[dB]
X_pulse	= 4.89566667[us]
Irr_atn_dec	= 20.7[dB]
Irr_atn_noe	= 20.7[dB]
Irr_noise	= WALTZ
Decoupling	= TRUE
Initial_wait	= 1[s]
Noe	= TRUE
Noe_time	= 2[s]
Recvr_gain	= 54
Relaxation_delay	= 2[s]
Repetition_time	= 2.85983232[s]
Temp_get	= 20.4[dC]

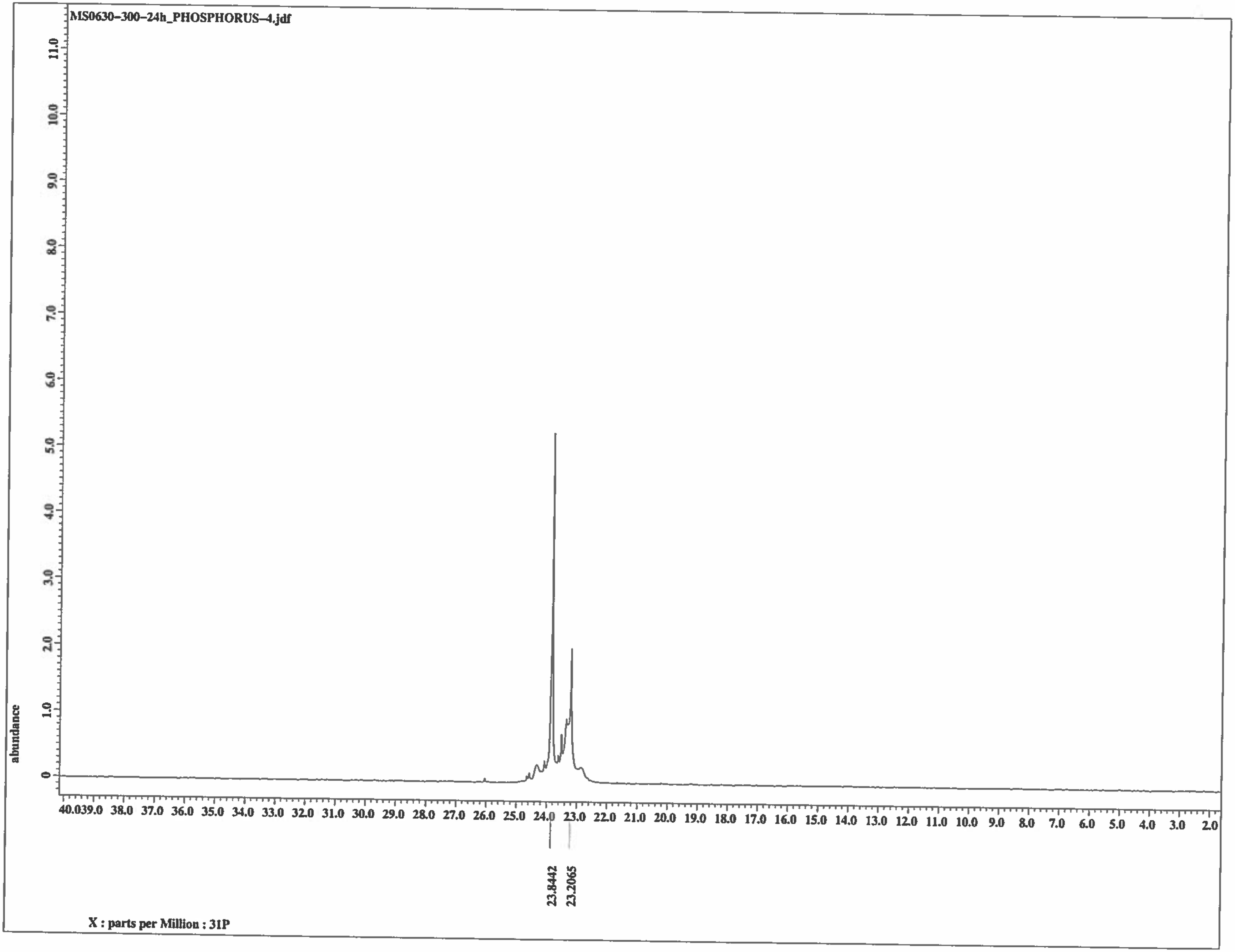

MS0630-300-24h_PHOSPHORUS-4.jdf
abundance
23.8442
23.2065
X : parts per Million : 31P

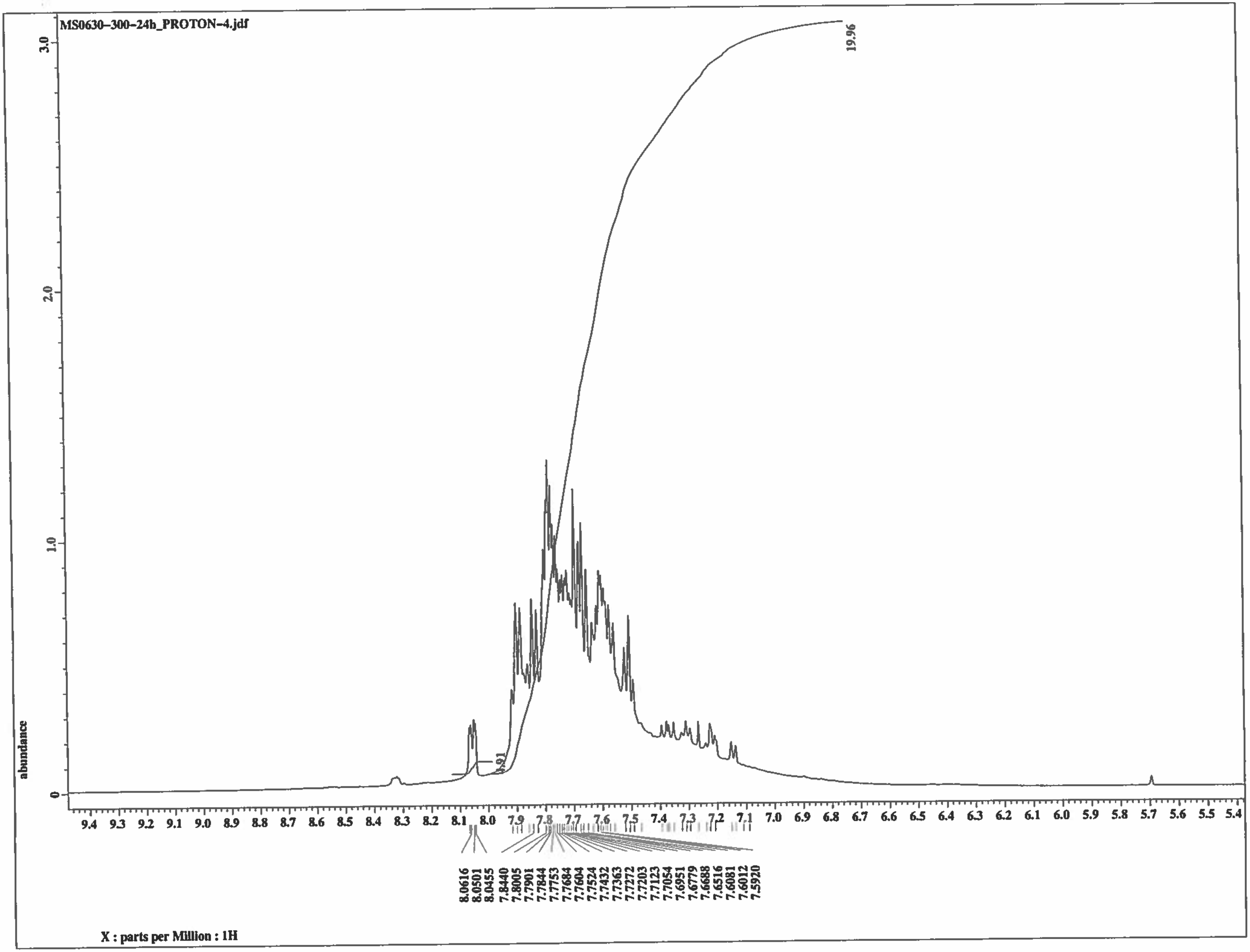

MS0630-300-24h_PROTON-4.jdf
abundance
0
1.0
2.0
3.0
19.96
1.91
9.4 9.3 9.2 9.1 9.0 8.9 8.8 8.7 8.6 8.5 8.4 8.3 8.2 8.1 8.0 7.9 7.8 7.7 7.6 7.5 7.4 7.3 7.2 7.1 7.0 6.9 6.8 6.7 6.6 6.5 6.4 6.3 6.2 6.1 6.0 5.9 5.8 5.7 5.6 5.5 5.4
8.0616
8.0501
8.0455
7.8440
7.8005
7.7901
7.7844
7.7753
7.7684
7.7604
7.7524
7.7432
7.7363
7.7272
7.7203
7.7123
7.7054
7.6951
7.6779
7.6688
7.6516
7.6081
7.6012
7.5920
X : parts per Million : 1H

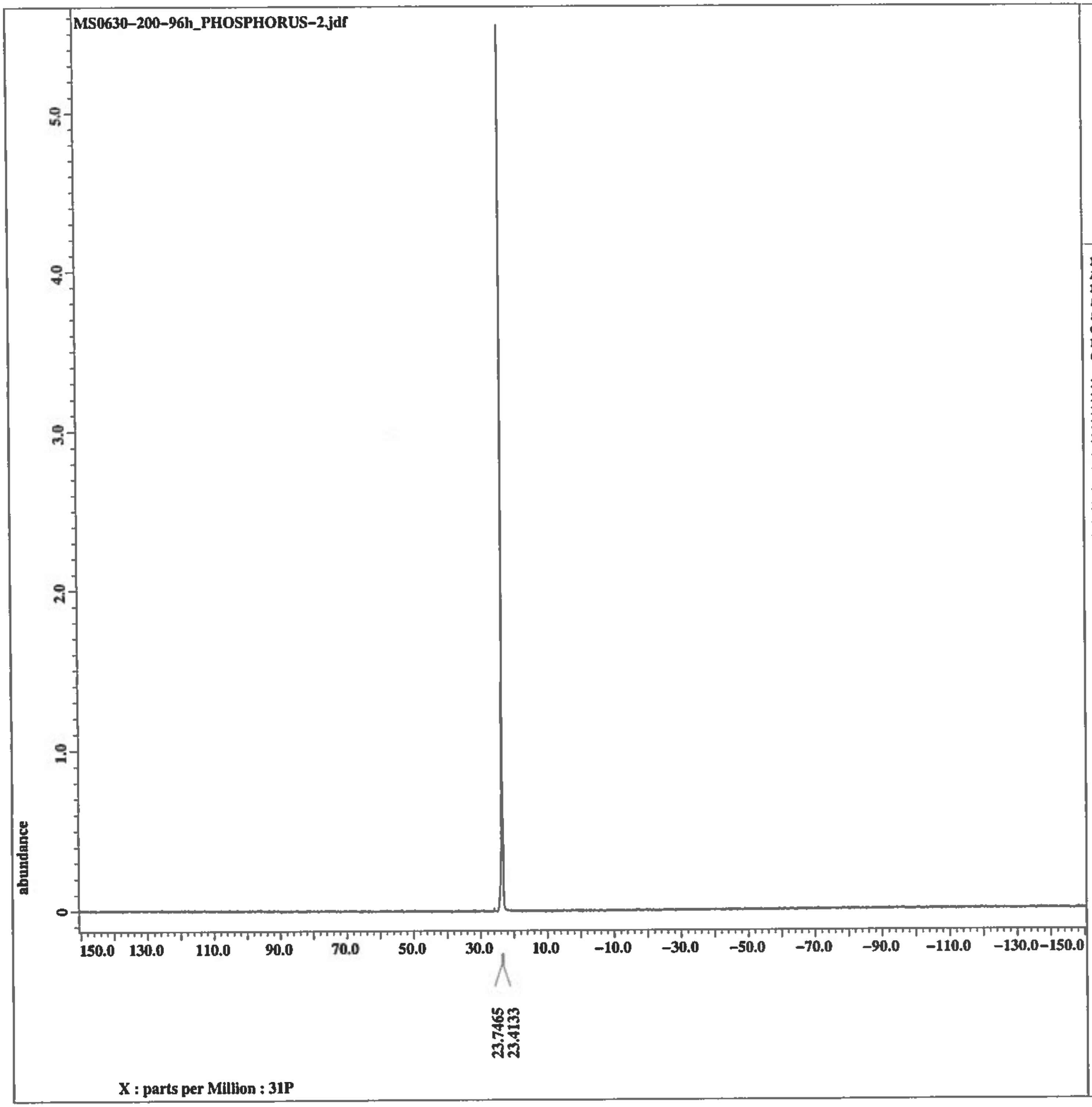


```
Filename           = MS0630-200-96h_PHOSPH
Author             = Jim Davis
Experiment         = single_pulse_dec
Sample_id          = MS0630-200-96h
Solvent            = CHLOROFORM-D
Creation_time      = 18-DEC-2018 09:40:42
Revision_time      = 18-DEC-2018 09:14:24
Current_time       = 18-DEC-2018 09:14:24

Data_format        = 1D COMPLEX
Dim_size           = 52428
Dim_title          = 31P
Dim_units          = [ppm]
Dimensions         = X
Site               = ECA 500
Spectrometer       = JNM-ECA500

Field_strength     = 11.7473579[T] (500[MH
X_acq_duration     = 0.85983232[s]
X_domain           = 31P
X_freq             = 202.46831075[MHz]
X_offset           = 0[ppm]
X_points           = 65536
X_prescans         = 4
X_resolution       = 1.16301746[Hz]
X_sweep            = 76.2195122[kHz]
Irr_domain         = 1H
Irr_freq           = 500.15991521[MHz]
Irr_offset         = 5.0[ppm]
Clipped            = FALSE
Mod_return         = 1
Scans              = 70
Total_scans        = 70

X_90_width         = 14.687[us]
X_acq_time         = 0.85983232[s]
X_angle            = 30[deg]
X_atn              = 5[dB]
X_pulse            = 4.89566667[us]
Irr_atn_dec        = 20.7[dB]
Irr_atn_noe        = 20.7[dB]
Irr_noise          = WALTZ
Decoupling         = TRUE
Initial_wait       = 1[s]
Noe                = TRUE
Noe_time           = 2[s]
Recvr_gain         = 54
Relaxation_delay   = 2[s]
Repetition_time    = 2.85983232[s]
Temp_get           = 19.8[dC]
```

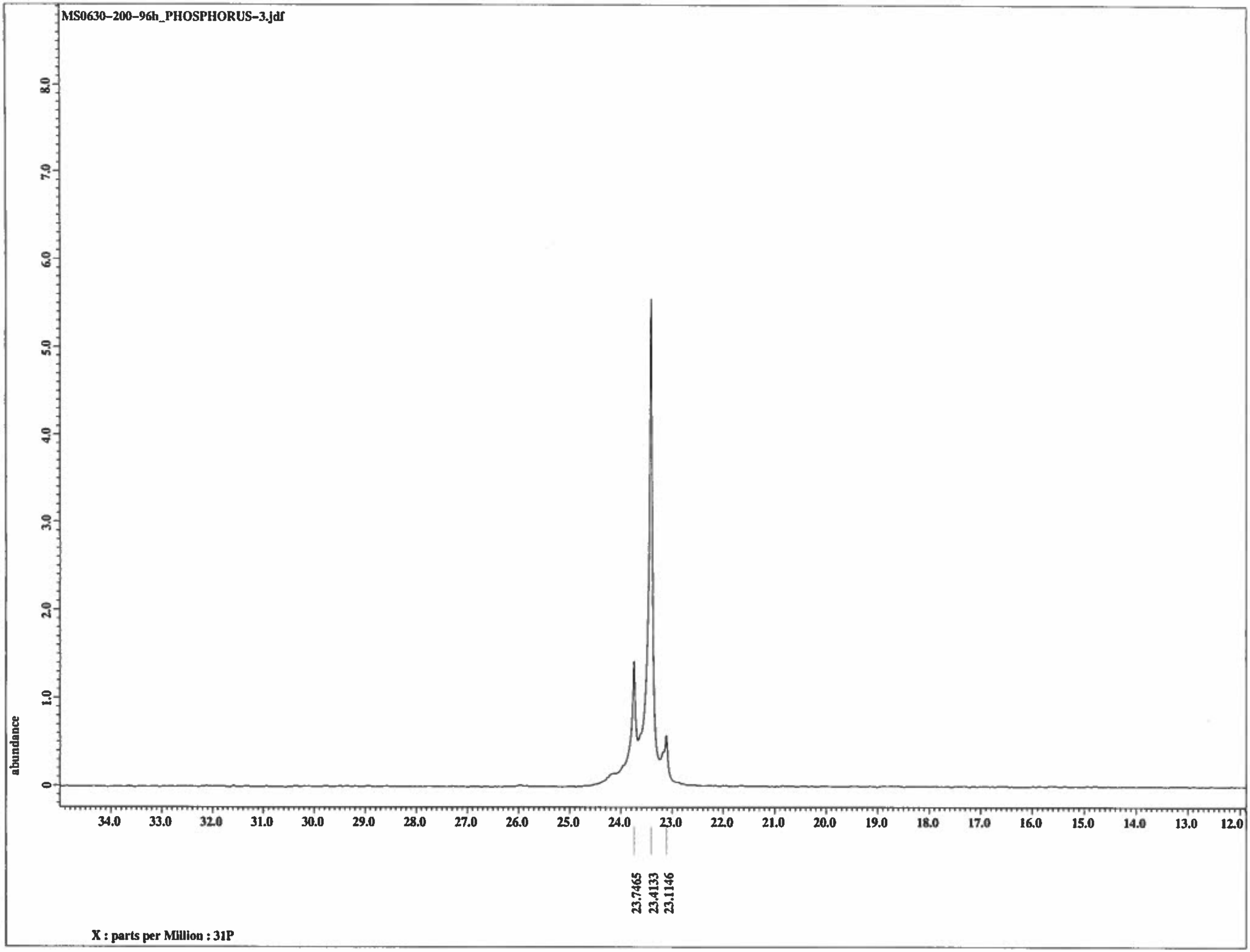
MS0630-200-96h_PHOSPHORUS-3.jdf
abundance
0
1.0
2.0
3.0
4.0
5.0
6.0
7.0
8.0
34.0
33.0
32.0
31.0
30.0
29.0
28.0
27.0
26.0
25.0
24.0
23.0
22.0
21.0
20.0
19.0
18.0
17.0
16.0
15.0
14.0
13.0
12.0
23.7465
23.4133
23.1146
X : parts per Million : 31P

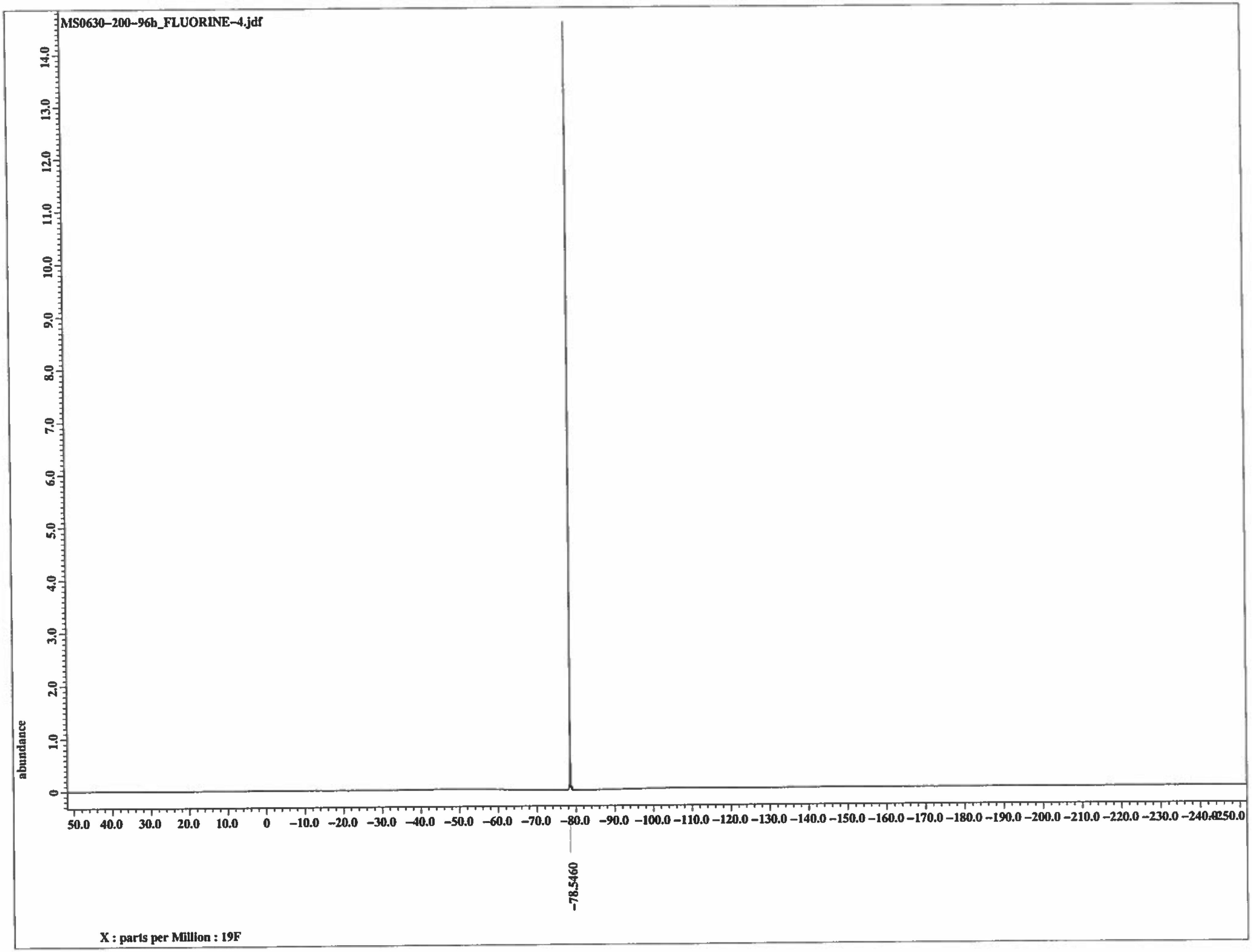

MS0630-200-96b_FLUORINE-4.jdf
abundance
0
1.0
2.0
3.0
4.0
5.0
6.0
7.0
8.0
9.0
10.0
11.0
12.0
13.0
14.0
50.0 40.0 30.0 20.0 10.0 0 -10.0 -20.0 -30.0 -40.0 -50.0 -60.0 -70.0 -80.0 -90.0 -100.0 -110.0 -120.0 -130.0 -140.0 -150.0 -160.0 -170.0 -180.0 -190.0 -200.0 -210.0 -220.0 -230.0 -240.0 -250.0
-78.5460
X : parts per Million : 19F

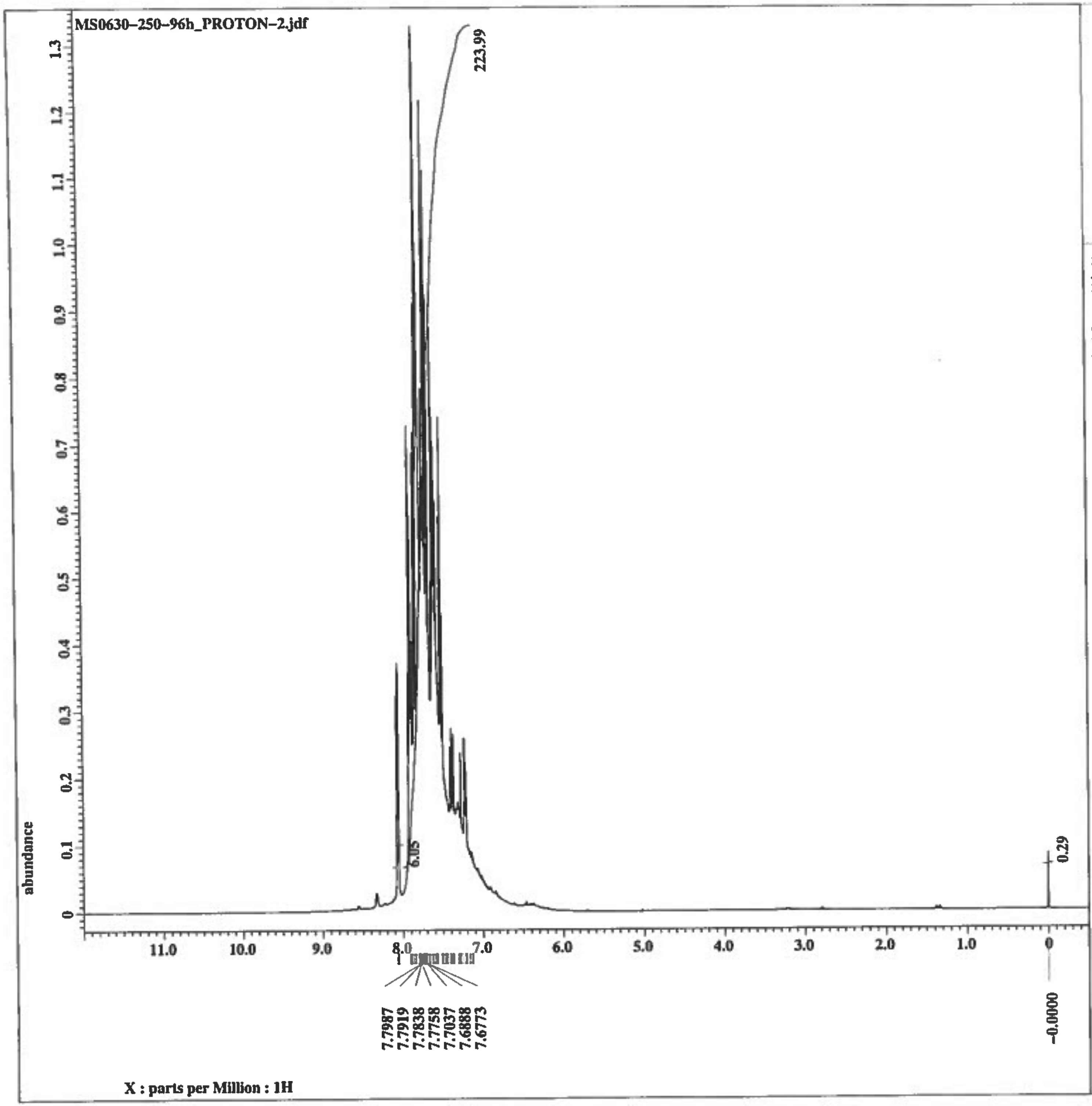

Filename	= MS0630-250-96h_PROTON
Author	= Jim Davis
Experiment	= single_pulse.ex2
Sample_id	= MS0630-250-96h
Solvent	= CHLOROFORM-D
Creation_time	= 18-DEC-2018 11:36:10
Revision_time	= 18-DEC-2018 11:09:52
Current_time	= 18-DEC-2018 11:09:52
Data_format	= 1D COMPLEX
Dim_size	= 13107
Dim_title	= 1H
Dim_units	= [ppm]
Dimensions	= X
Site	= ECA 500
Spectrometer	= JNM-ECA500
Field_strength	= 11.7473579[T] (500[MH
X_acq_duration	= 1.74587904[s]
X_domain	= 1H
X_freq	= 500.15991521[MHz]
X_offset	= 5.0[ppm]
X_points	= 16384
X_prescans	= 1
X_resolution	= 0.57277737[Hz]
X_sweep	= 9.38438438[kHz]
Irr_domain	= 1H
Irr_freq	= 500.15991521[MHz]
Irr_offset	= 5.0[ppm]
Tri_domain	= 1H
Tri_freq	= 500.15991521[MHz]
Tri_offset	= 5.0[ppm]
Clipped	= FALSE
Mod_return	= 1
Scans	= 16
Total_scans	= 16
X_90_width	= 12.4[us]
X_acq_time	= 1.74587904[s]
X_angle	= 45[deg]
X_atn	= 4[dB]
X_pulse	= 6.2[us]
Irr_mode	= Off
Tri_mode	= Off
Dante_presat	= FALSE
Initial_wait	= 1[s]
Recvr_gain	= 22
Relaxation_delay	= 4[s]
Repetition_time	= 5.74587904[s]
Temp_get	= 19.9[dC]

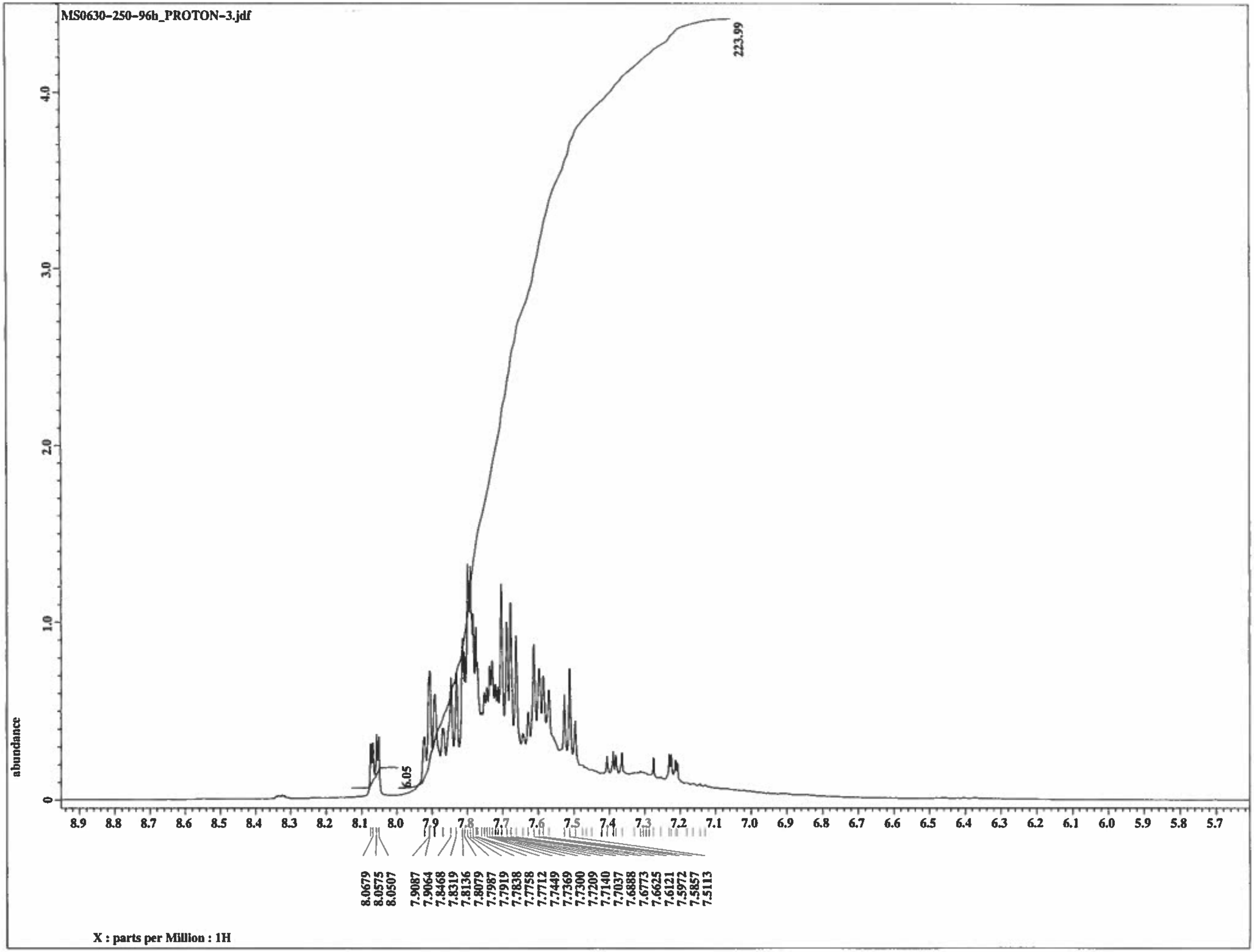
MS0630-250-96b_PROTON-3.jdf
abundance
0
1.0
2.0
3.0
4.0
223.99
6.05
8.9 8.8 8.7 8.6 8.5 8.4 8.3 8.2 8.1 8.0 7.9 7.8 7.7 7.6 7.5 7.4 7.3 7.2 7.1 7.0 6.9 6.8 6.7 6.6 6.5 6.4 6.3 6.2 6.1 6.0 5.9 5.8 5.7
8.0679
8.0575
8.0507
7.9087
7.9064
7.8468
7.8319
7.8136
7.8079
7.7987
7.7919
7.7838
7.7758
7.7712
7.7449
7.7369
7.7300
7.7209
7.7140
7.7037
7.6888
7.6773
7.6625
7.6121
7.5972
7.5857
7.5113
X : parts per Million : 1H

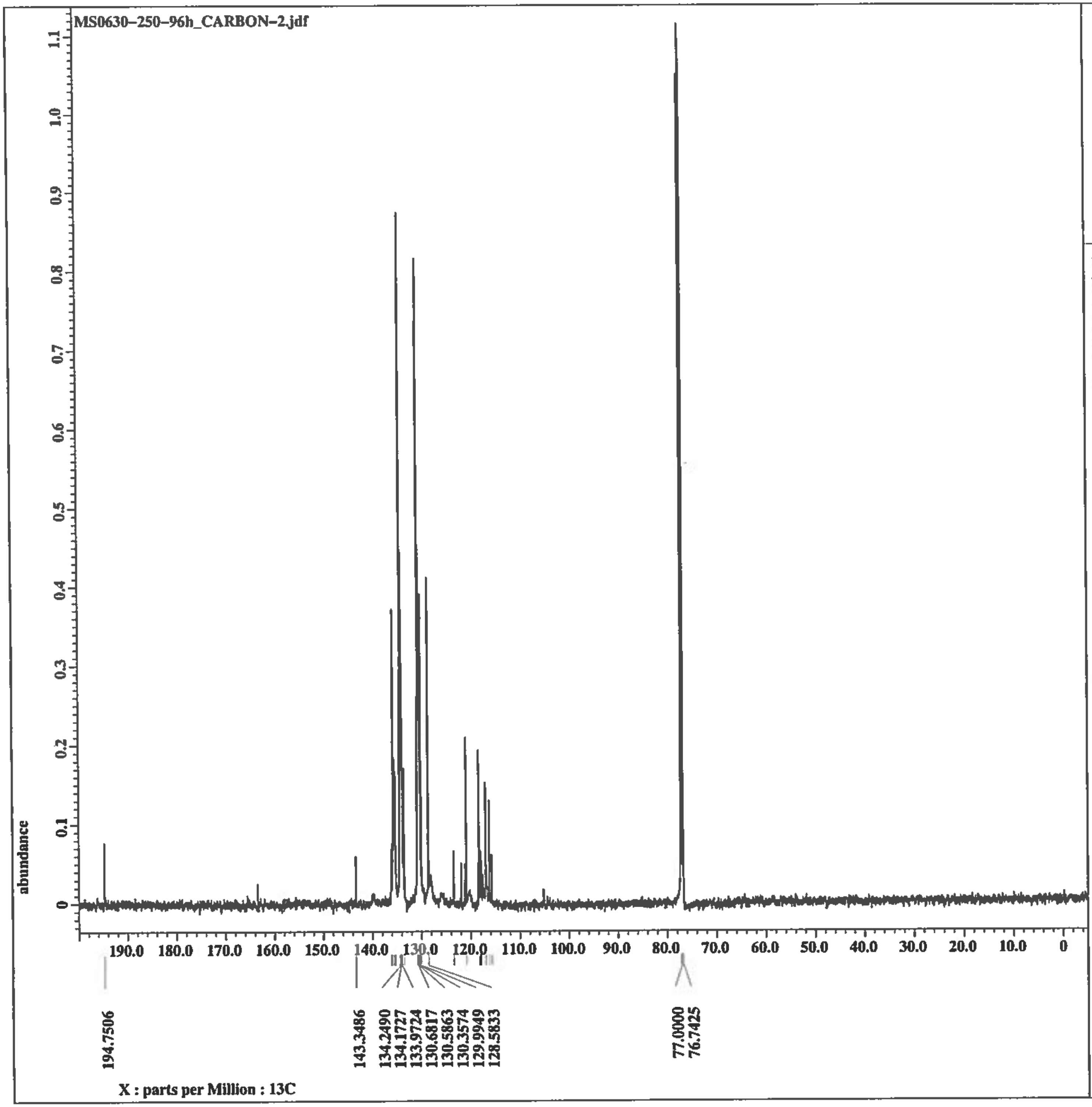

Filename	= MS0630-250-96h_CARBON
Author	= Jim Davis
Experiment	= single_pulse_dec
Sample_id	= MS0630-250-96h
Solvent	= CHLOROFORM-D
Creation_time	= 18-DEC-2018 11:57:53
Revision_time	= 18-DEC-2018 11:31:35
Current_time	= 18-DEC-2018 11:31:35
Data_format	= 1D COMPLEX
Dim_size	= 26214
Dim_title	= 13C
Dim_units	= [ppm]
Dimensions	= X
Site	= ECA 500
Spectrometer	= JNM-ECA500
Field_strength	= 11.7473579[T] (500[MH
X_acq_duration	= 0.83361792[s]
X_domain	= 13C
X_freq	= 125.76529768[MHz]
X_offset	= 100[ppm]
X_points	= 32768
X_prescans	= 4
X_resolution	= 1.19959034[Hz]
X_sweep	= 39.3081761[kHz]
Irr_domain	= 1H
Irr_freq	= 500.15991521[MHz]
Irr_offset	= 5.0[ppm]
Clipped	= FALSE
Mod_return	= 1
Scans	= 400
Total_scans	= 400
X_90_width	= 13.2[us]
X_acq_time	= 0.83361792[s]
X_angle	= 30[deg]
X_atn	= 6[dB]
X_pulse	= 4.4[us]
Irr_atn_dec	= 20.7[dB]
Irr_atn_noe	= 20.7[dB]
Irr_noise	= WALTZ
Decoupling	= TRUE
Initial_wait	= 1[s]
Noe	= TRUE
Noe_time	= 2[s]
Recvr_gain	= 60
Relaxation_delay	= 2[s]
Repetition_time	= 2.83361792[s]
Temp_get	= 20.7[dC]

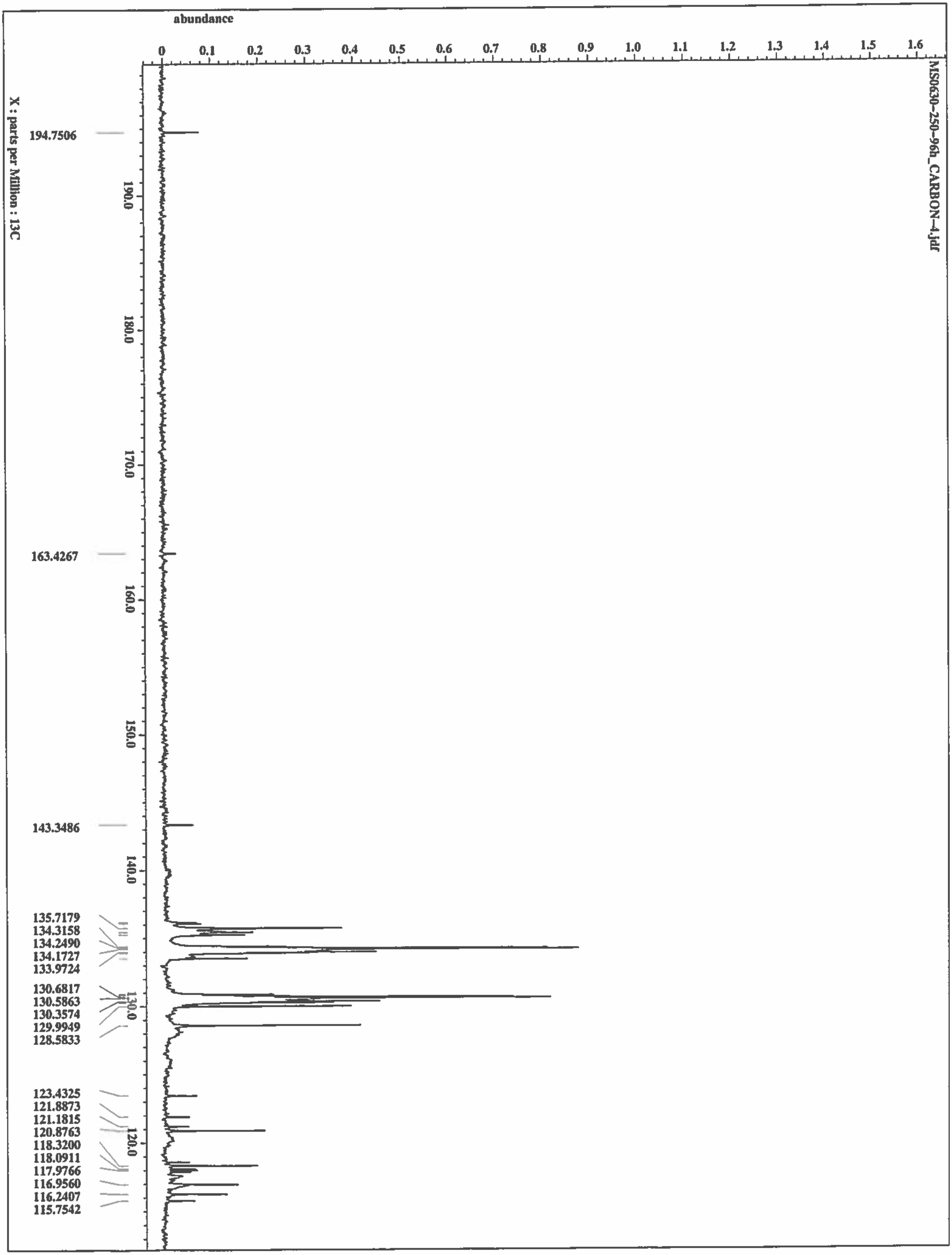

abundance
0 0.1 0.2 0.3 0.4 0.5 0.6 0.7 0.8 0.9 1.0 1.1 1.2 1.3 1.4 1.5 1.6
MS0630-250-96h_CARBON-4.jdf
X : parts per Million : 13C
190.0
180.0
170.0
160.0
150.0
140.0
130.0
120.0
194.7506
163.4267
143.3486
135.7179
134.3158
134.2490
134.1727
133.9724
130.6817
130.5863
130.3574
129.9949
128.5833
123.4325
121.8873
121.1815
120.8763
118.3200
118.0911
117.9766
116.9560
116.2407
115.7542

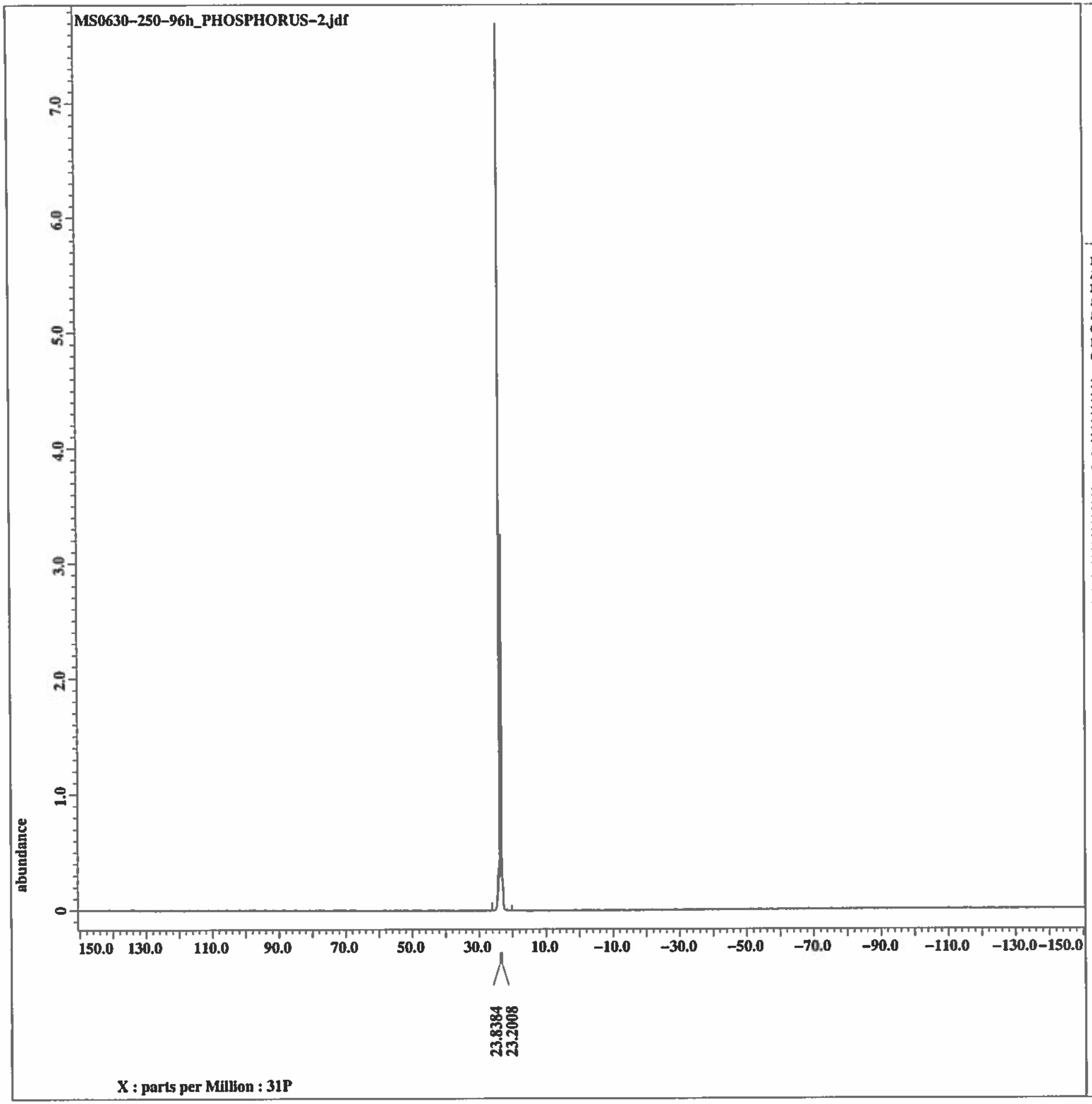

Filename	=	MS0630-250-96h_PHOSPH
Author	=	Jim Davis
Experiment	=	single_pulse_dec
Sample_id	=	MS0630-250-96h
Solvent	=	CHLOROFORM-D
Creation_time	=	18-DEC-2018 12:06:35
Revision_time	=	18-DEC-2018 11:40:17
Current_time	=	18-DEC-2018 11:40:17
Data_format	=	1D COMPLEX
Dim_size	=	52428
Dim_title	=	31P
Dim_units	=	[ppm]
Dimensions	=	X
Site	=	ECA 500
Spectrometer	=	JNM-ECA500
Field_strength	=	11.7473579[T] (500[MH
X_acq_duration	=	0.85983232[s]
X_domain	=	31P
X_freq	=	202.46831075[MHz]
X_offset	=	0[ppm]
X_points	=	65536
X_prescans	=	4
X_resolution	=	1.16301746[Hz]
X_sweep	=	76.2195122[kHz]
Irr_domain	=	1H
Irr_freq	=	500.15991521[MHz]
Irr_offset	=	5.0[ppm]
Clipped	=	TRUE
Mod_return	=	1
Scans	=	128
Total_scans	=	128
X_90_width	=	14.687[us]
X_acq_time	=	0.85983232[s]
X_angle	=	30[deg]
X_atn	=	5[dB]
X_pulse	=	4.89566667[us]
Irr_atn_dec	=	20.7[dB]
Irr_atn_noe	=	20.7[dB]
Irr_noise	=	WALTZ
Decoupling	=	TRUE
Initial_wait	=	1[s]
Noe	=	TRUE
Noe_time	=	2[s]
Recvr_gain	=	54
Relaxation_delay	=	2[s]
Repetition_time	=	2.85983232[s]
Temp_get	=	20.6[dC]

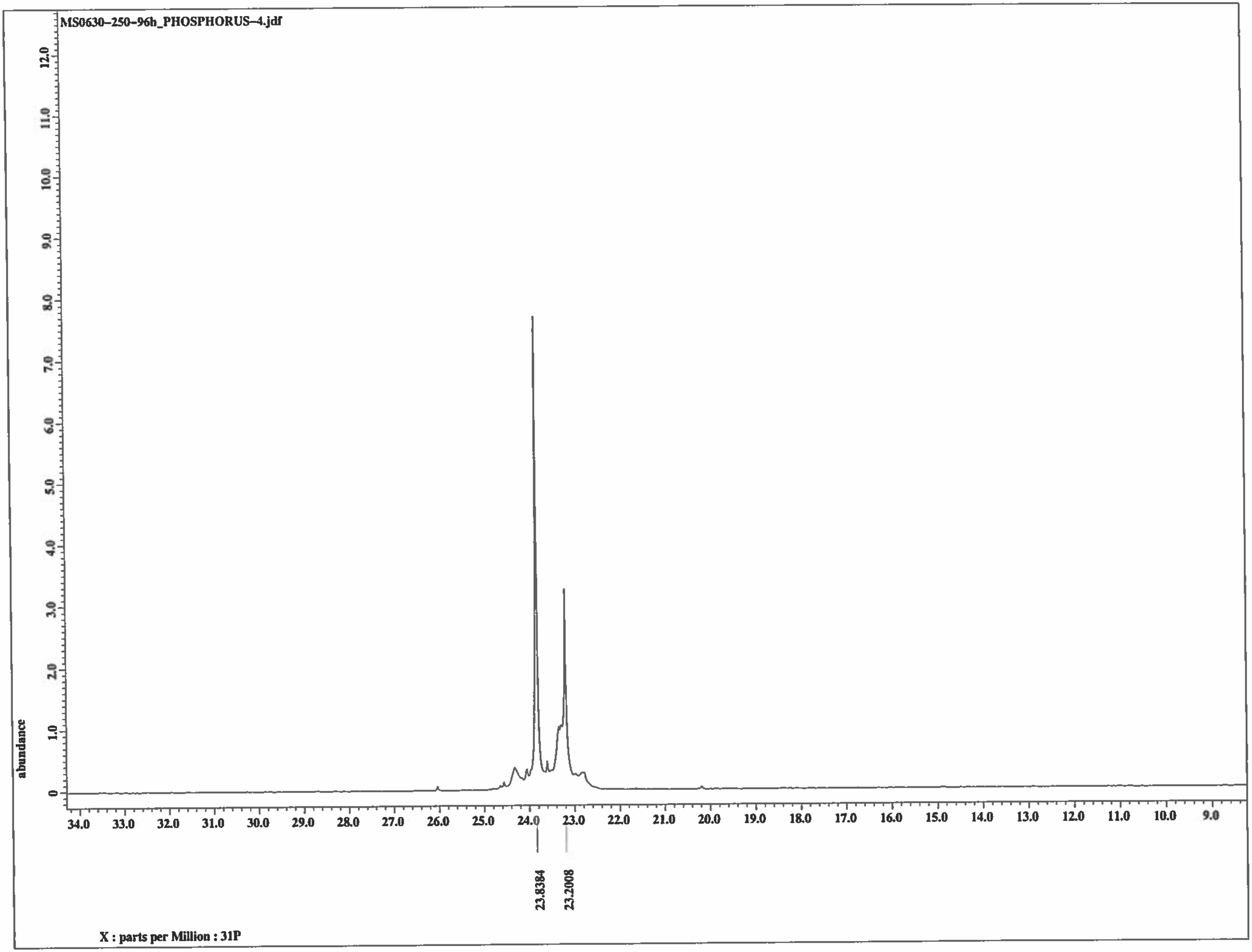
MS0630–250–96h_PHOSPHORUS–4.jdf
abundance
23.8384
23.2008
X : parts per Million : 31P

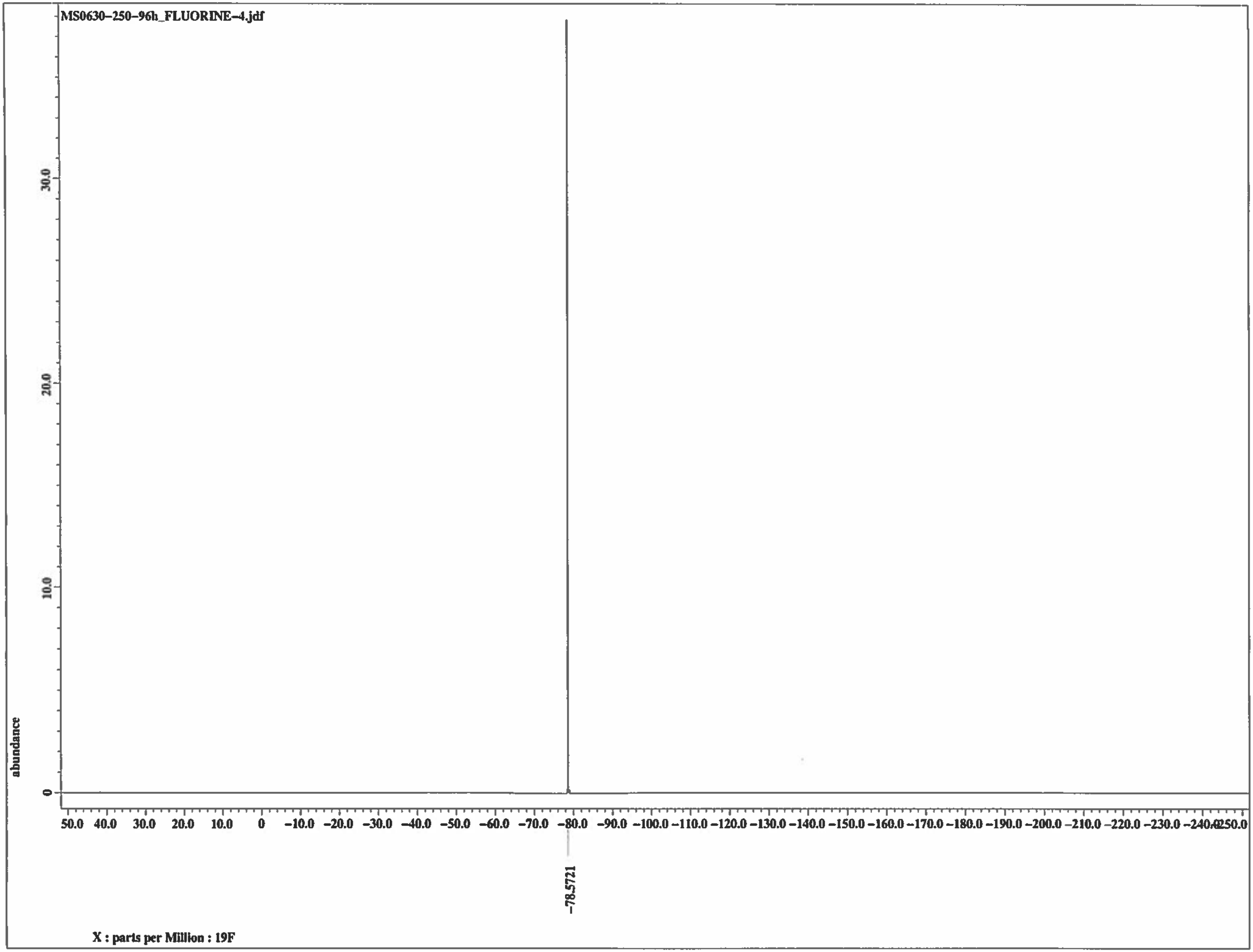
MS0630-250-96h_FLUORINE-4.jdf
abundance
0
10.0
20.0
30.0
50.0 40.0 30.0 20.0 10.0 0 -10.0 -20.0 -30.0 -40.0 -50.0 -60.0 -70.0 -80.0 -90.0 -100.0 -110.0 -120.0 -130.0 -140.0 -150.0 -160.0 -170.0 -180.0 -190.0 -200.0 -210.0 -220.0 -230.0 -240.0 -250.0
-78.5721
X : parts per Million : 19F

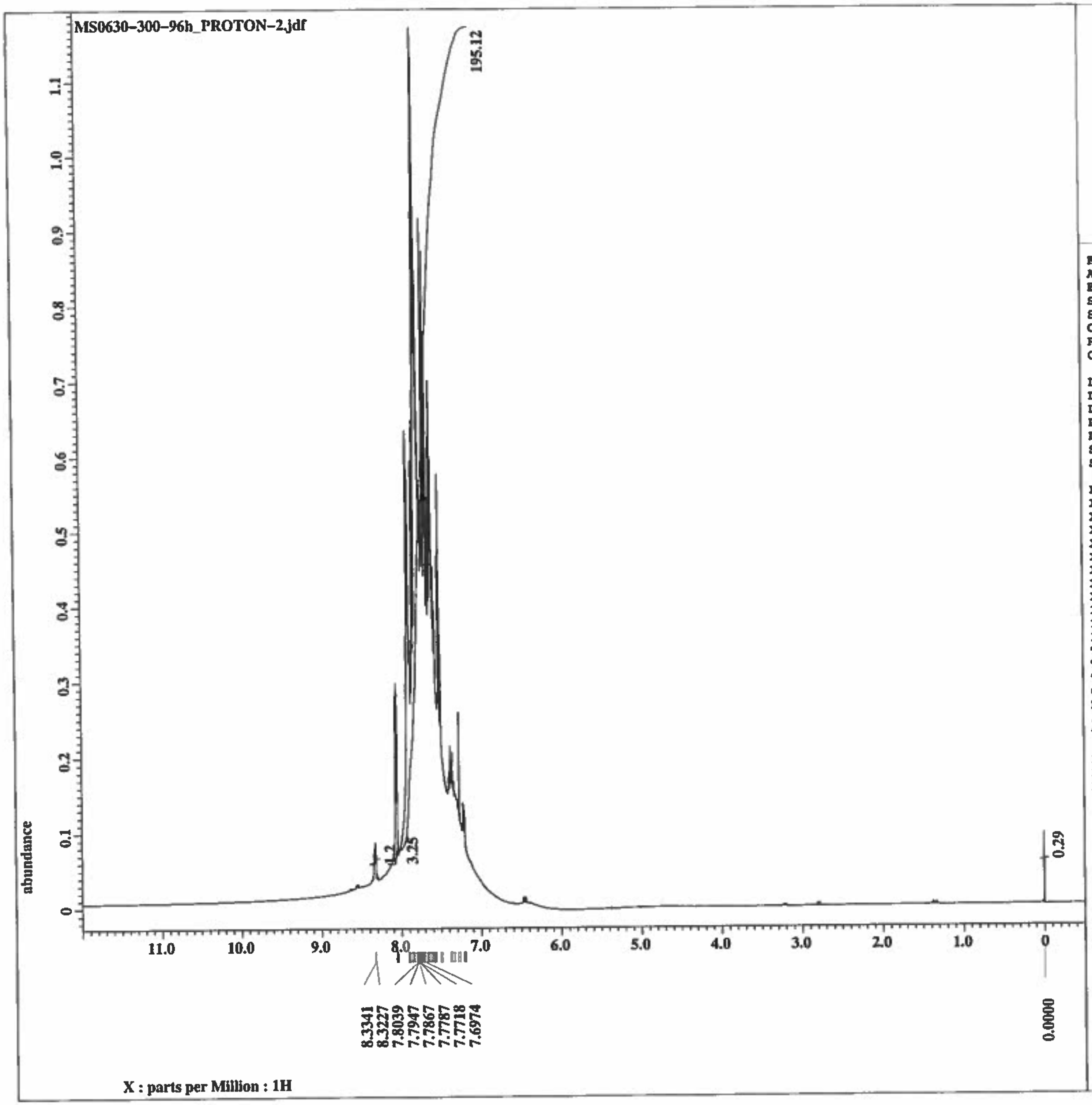

Filename	= MS0630-300-96h_PROTON
Author	= Jim Davis
Experiment	= single_pulse.ex2
Sample_id	= MS0630-300-96h
Solvent	= CHLOROFORM-D
Creation_time	= 18-DEC-2018 12:26:17
Revision_time	= 18-DEC-2018 11:59:58
Current_time	= 18-DEC-2018 11:59:58
Data_format	= 1D COMPLEX
Dim_size	= 13107
Dim_title	= 1H
Dim_units	= [ppm]
Dimensions	= X
Site	= ECA 500
Spectrometer	= JNM-ECA500
Field_strength	= 11.7473579[T] (500[MH
X_acq_duration	= 1.74587904[s]
X_domain	= 1H
X_freq	= 500.15991521[MHz]
X_offset	= 5.0[ppm]
X_points	= 16384
X_prescans	= 1
X_resolution	= 0.57277737[Hz]
X_sweep	= 9.38438438[kHz]
Irr_domain	= 1H
Irr_freq	= 500.15991521[MHz]
Irr_offset	= 5.0[ppm]
Tri_domain	= 1H
Tri_freq	= 500.15991521[MHz]
Tri_offset	= 5.0[ppm]
Clipped	= FALSE
Mod_return	= 1
Scans	= 16
Total_scans	= 16
X_90_width	= 12.4[us]
X_acq_time	= 1.74587904[s]
X_angle	= 45[deg]
X_atn	= 4[dB]
X_pulse	= 6.2[us]
Irr_mode	= Off
Tri_mode	= Off
Dante_presat	= FALSE
Initial_wait	= 1[s]
Recvr_gain	= 24
Relaxation_delay	= 4[s]
Repetition_time	= 5.74587904[s]
Temp_get	= 20.3[dC]

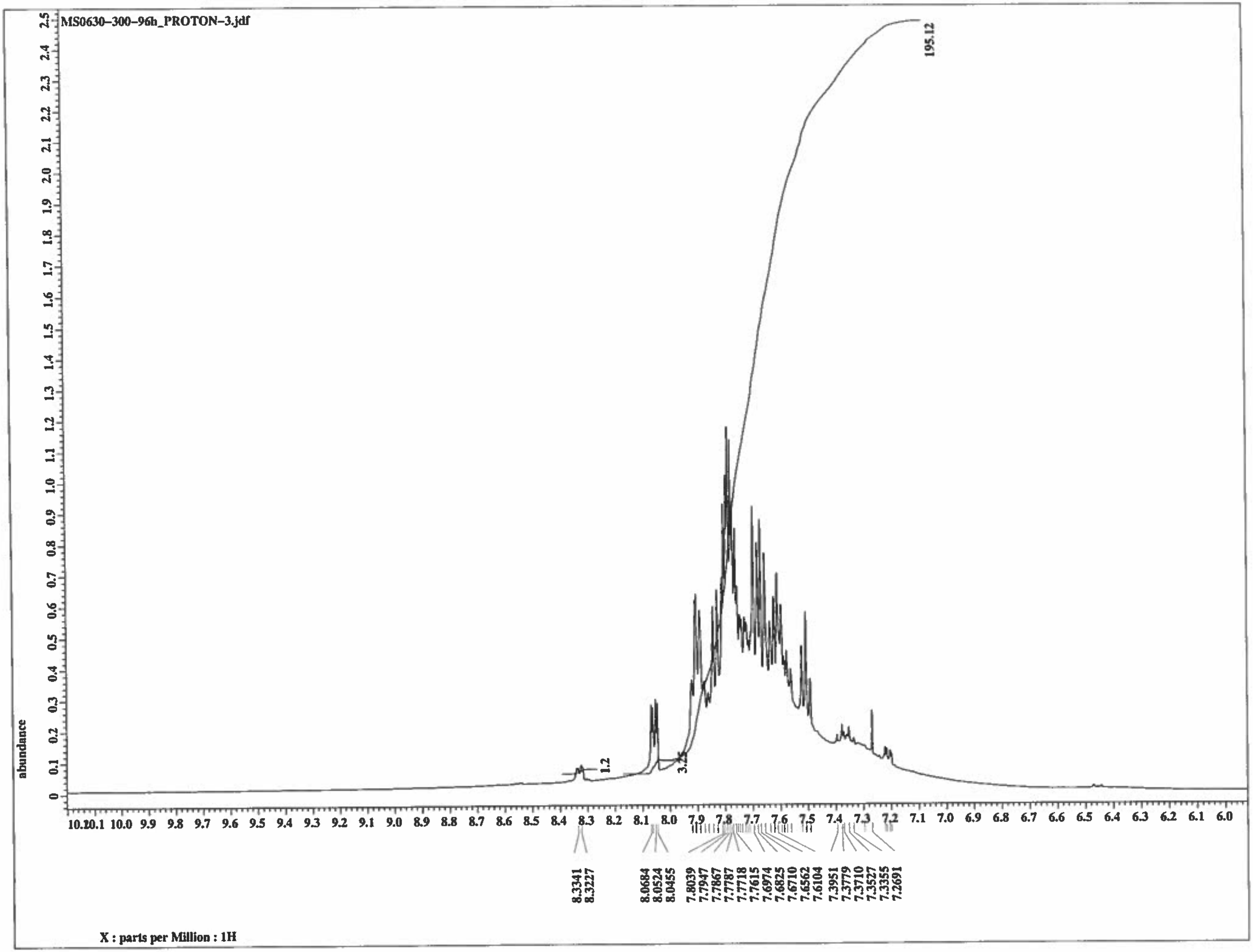
MS0630-300-96h_PROTON-3.jdf
abundance
X : parts per Million : 1H
195.12
1.2
3.25
8.3341
8.3227
8.0684
8.0524
8.0455
7.8039
7.7947
7.7867
7.7787
7.7718
7.7615
7.6974
7.6825
7.6710
7.6562
7.6104
7.3951
7.3779
7.3710
7.3527
7.3355
7.2691

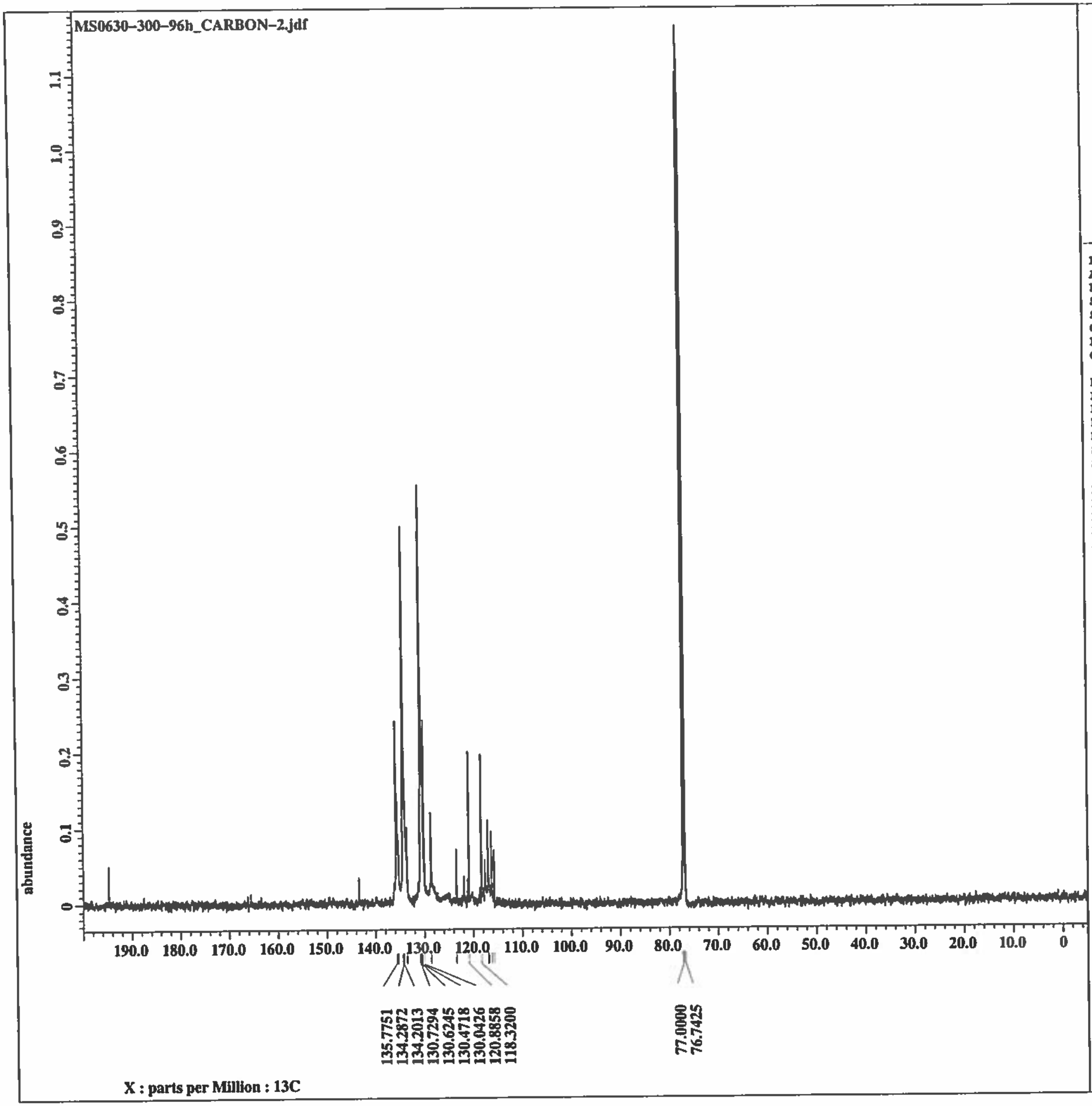

Filename	= MS0630-300-96h_CARBON
Author	= Jim Davis
Experiment	= single_pulse_dec
Sample_id	= MS0630-300-96h
Solvent	= CHLOROFORM-D
Creation_time	= 18-DEC-2018 12:47:22
Revision_time	= 18-DEC-2018 12:21:02
Current_time	= 18-DEC-2018 12:21:02
Data_format	= 1D COMPLEX
Dim_size	= 26214
Dim_title	= 13C
Dim_units	= [ppm]
Dimensions	= X
Site	= ECA 500
Spectrometer	= JNM-ECA500
Field_strength	= 11.7473579[T] (500[MH
X_acq_duration	= 0.83361792[s]
X_domain	= 13C
X_freq	= 125.76529768[MHz]
X_offset	= 100[ppm]
X_points	= 32768
X_prescans	= 4
X_resolution	= 1.19959034[Hz]
X_sweep	= 39.3081761[kHz]
Irr_domain	= 1H
Irr_freq	= 500.15991521[MHz]
Irr_offset	= 5.0[ppm]
Clipped	= FALSE
Mod_return	= 1
Scans	= 400
Total_scans	= 400
X_90_width	= 13.2[us]
X_acq_time	= 0.83361792[s]
X_angle	= 30[deg]
X_atn	= 6[dB]
X_pulse	= 4.4[us]
Irr_atn_dec	= 20.7[dB]
Irr_atn_noe	= 20.7[dB]
Irr_noise	= WALTZ
Decoupling	= TRUE
Initial_wait	= 1[s]
Noe	= TRUE
Noe_time	= 2[s]
Recvr_gain	= 60
Relaxation_delay	= 2[s]
Repetition_time	= 2.83361792[s]
Temp_get	= 20.6[dC]

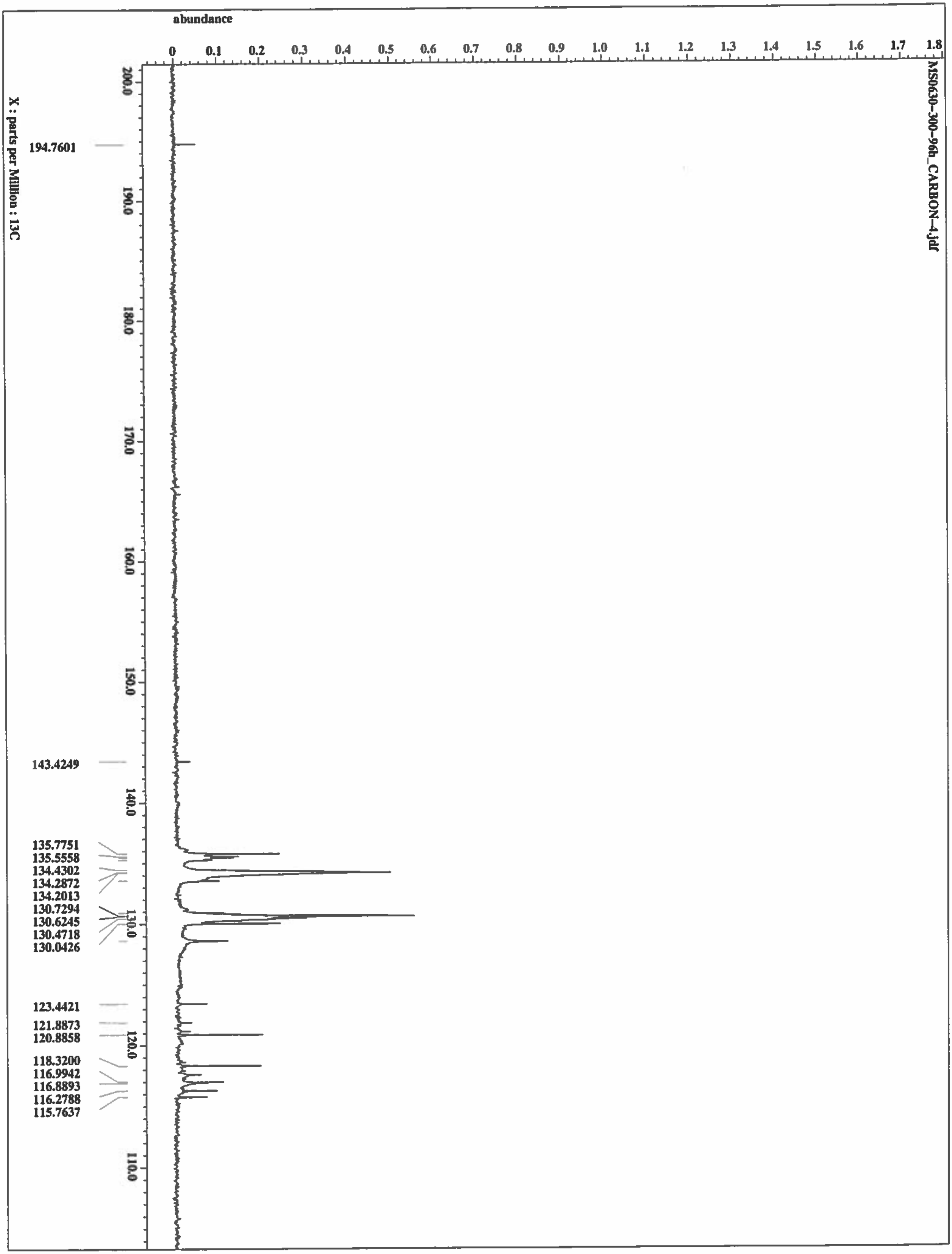
abundance
0
0.1
0.2
0.3
0.4
0.5
0.6
0.7
0.8
0.9
1.0
1.1
1.2
1.3
1.4
1.5
1.6
1.7
1.8
NS0630-300-96h_CARBON-4.jdf
X : parts per Million : 13C
200.0
190.0
180.0
170.0
160.0
150.0
140.0
130.0
120.0
110.0
194.7601
143.4249
135.7751
135.5558
134.4302
134.2872
134.2013
130.7294
130.6245
130.4718
130.0426
123.4421
121.8873
120.8858
118.3200
116.9942
116.8893
116.2788
115.7637

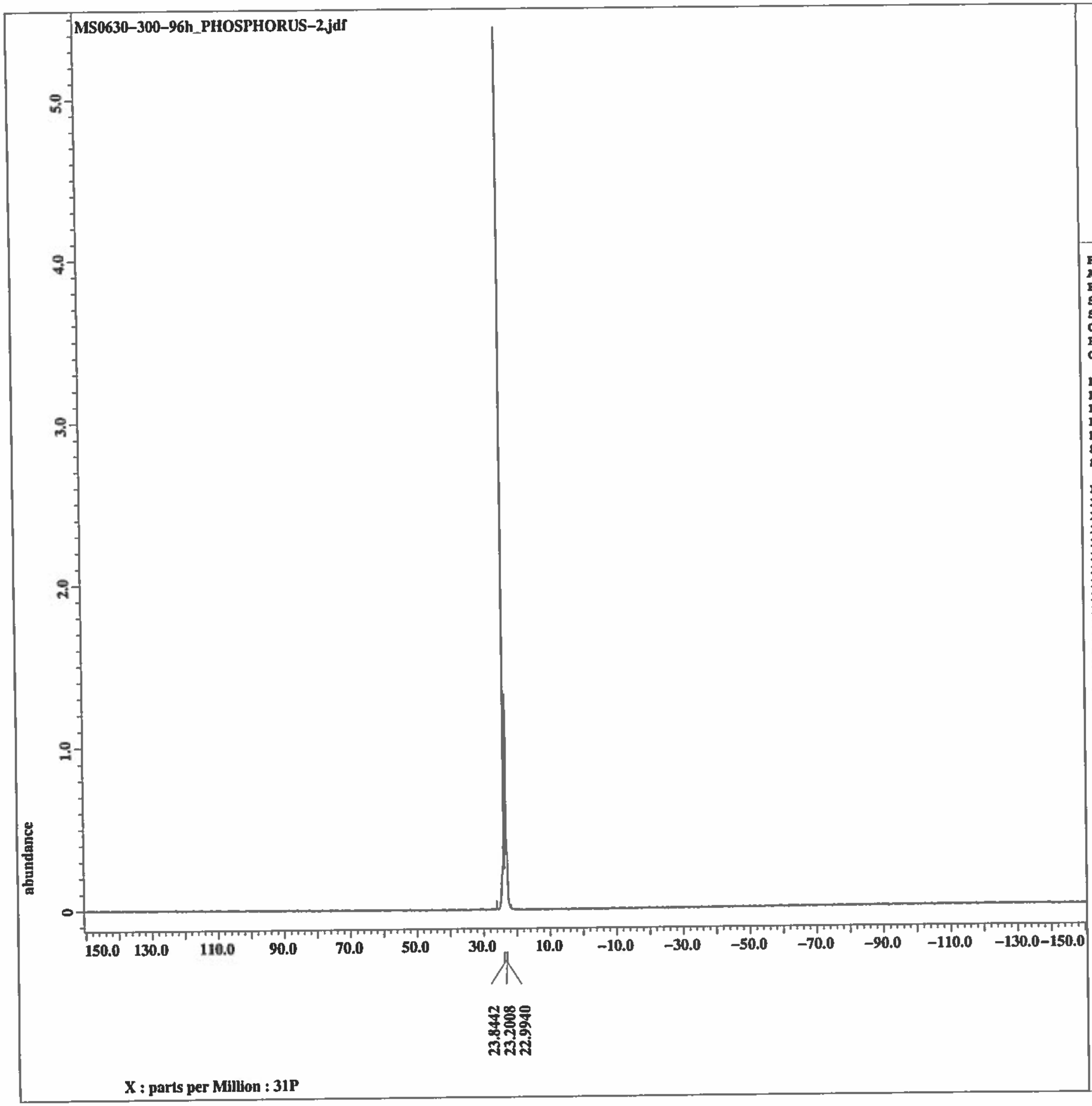

Filename	= MS0630-300-96h_PHOSPH
Author	= Jim Davis
Experiment	= single_pulse_dec
Sample_id	= MS0630-300-96h
Solvent	= CHLOROFORM-D
Creation_time	= 18-DEC-2018 12:55:48
Revision_time	= 18-DEC-2018 12:29:29
Current_time	= 18-DEC-2018 12:29:29
Data_format	= 1D COMPLEX
Dim_size	= 52428
Dim_title	= 31P
Dim_units	= [ppm]
Dimensions	= X
Site	= ECA 500
Spectrometer	= JNM-ECA500
Field_strength	= 11.7473579[T] (500[MH
X_acq_duration	= 0.85983232[s]
X_domain	= 31P
X_freq	= 202.46831075[MHz]
X_offset	= 0[ppm]
X_points	= 65536
X_prescans	= 4
X_resolution	= 1.16301746[Hz]
X_sweep	= 76.2195122[kHz]
Irr_domain	= 1H
Irr_freq	= 500.15991521[MHz]
Irr_offset	= 5.0[ppm]
Clipped	= TRUE
Mod_return	= 1
Scans	= 128
Total_scans	= 128
X_90_width	= 14.687[us]
X_acq_time	= 0.85983232[s]
X_angle	= 30[deg]
X_atn	= 5[dB]
X_pulse	= 4.89566667[us]
Irr_atn_dec	= 20.7[dB]
Irr_atn_noe	= 20.7[dB]
Irr_noise	= WALTZ
Decoupling	= TRUE
Initial_wait	= 1[s]
Noe	= TRUE
Noe_time	= 2[s]
Recvr_gain	= 54
Relaxation_delay	= 2[s]
Repetition_time	= 2.85983232[s]
Temp_get	= 20.9[dC]

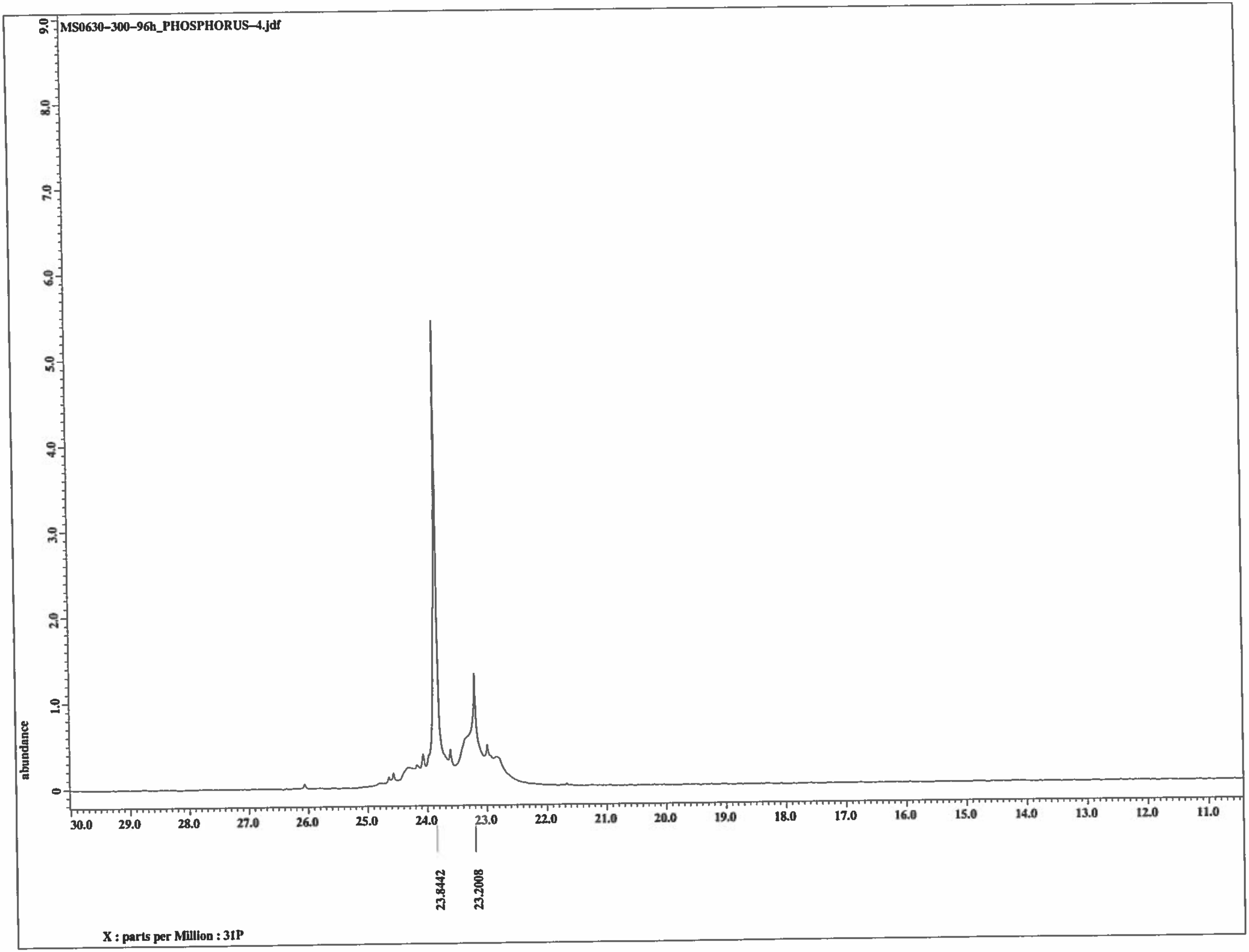
MS0630-300-96h_PHOSPHORUS-4.jdf
abundance
0
1.0
2.0
3.0
4.0
5.0
6.0
7.0
8.0
9.0
30.0
29.0
28.0
27.0
26.0
25.0
24.0
23.0
22.0
21.0
20.0
19.0
18.0
17.0
16.0
15.0
14.0
13.0
12.0
11.0
23.8442
23.2008
X : parts per Million : 31P

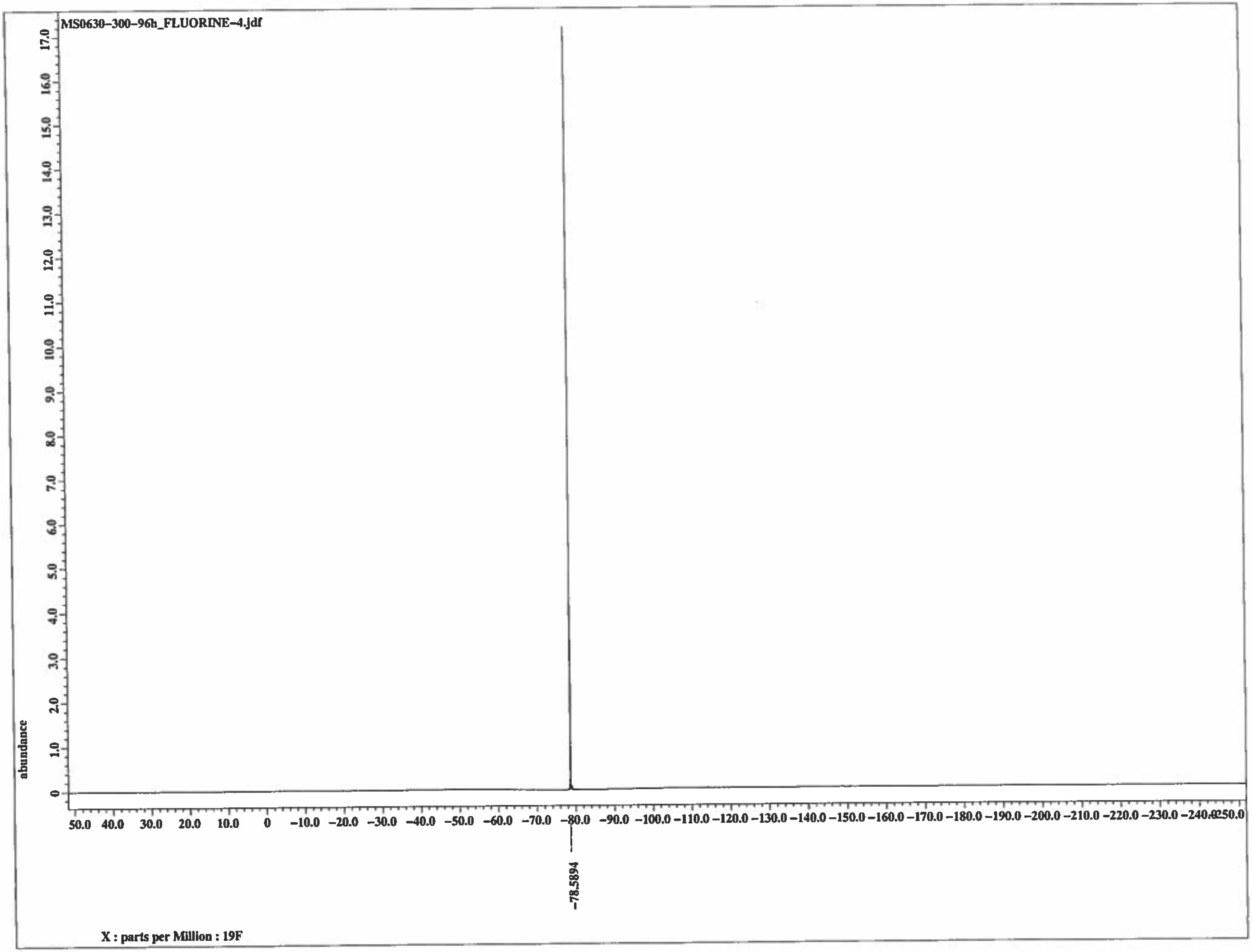

MS0630-300-96h_FLUORINE-4.jdf
abundance
-78.5894
X : parts per Million : 19F

Compound 14 Pre- and Post-heating NMR Spectra

Temperature of Post-heating samples noted in upper left corner of each spectrum

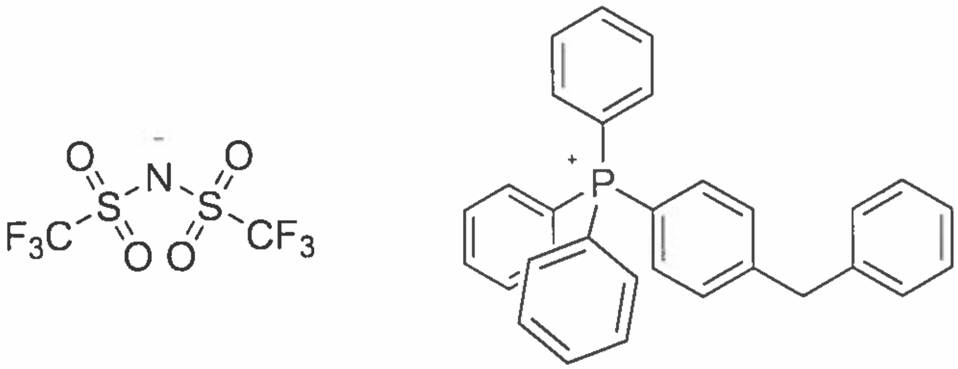

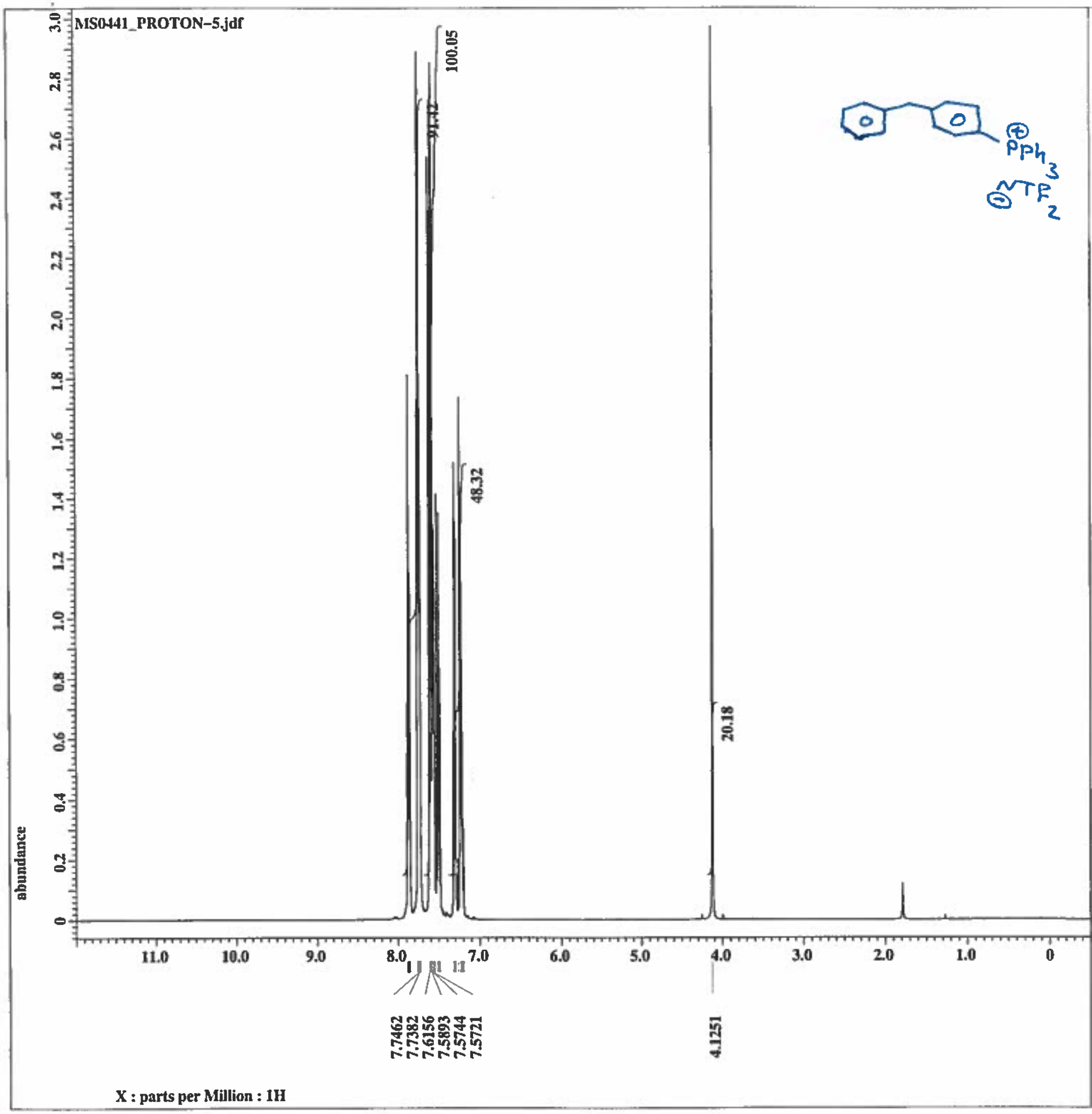

SOUTH ALABAMA JAGUARS

Filename	= MS0441_PROTON-5.jdf
Author	= Jim Davis
Experiment	= single_pulse.ex2
Sample_id	= MS0441
Solvent	= CHLOROFORM-D
Changer_sample	= 15
Creation_time	= 9-MAY-2018 19:31:18
Revision_time	= 9-MAY-2018 19:07:27
Current_time	= 9-MAY-2018 19:07:27
Data_format	= 1D COMPLEX
Dim_size	= 13107
Dim_title	= 1H
Dim_units	= [ppm]
Dimensions	= X
Site	= ECA 500
Spectrometer	= JNM-ECA500
Field_strength	= 11.7473579[T] (500[MH
X_acq_duration	= 1.74587904[s]
X_domain	= 1H
X_freq	= 500.15991521[MHz]
X_offset	= 5.0[ppm]
X_points	= 16384
X_prescans	= 1
X_resolution	= 0.57277737[Hz]
X_sweep	= 9.38438438[kHz]
Irr_domain	= 1H
Irr_freq	= 500.15991521[MHz]
Irr_offset	= 5.0[ppm]
Tri_domain	= 1H
Tri_freq	= 500.15991521[MHz]
Tri_offset	= 5.0[ppm]
Clipped	= FALSE
Mod_return	= 1
Scans	= 16
Total_scans	= 16
X_90_width	= 12.4[us]
X_acq_time	= 1.74587904[s]
X_angle	= 45[deg]
X_atn	= 4[dB]
X_pulse	= 6.2[us]
Irr_mode	= Off
Tri_mode	= Off
Dante_presat	= FALSE
Initial_wait	= 1[s]
Recvr_gain	= 22
Relaxation_delay	= 4[s]
Repetition_time	= 5.74587904[s]
Temp_get	= 22.9[dC]

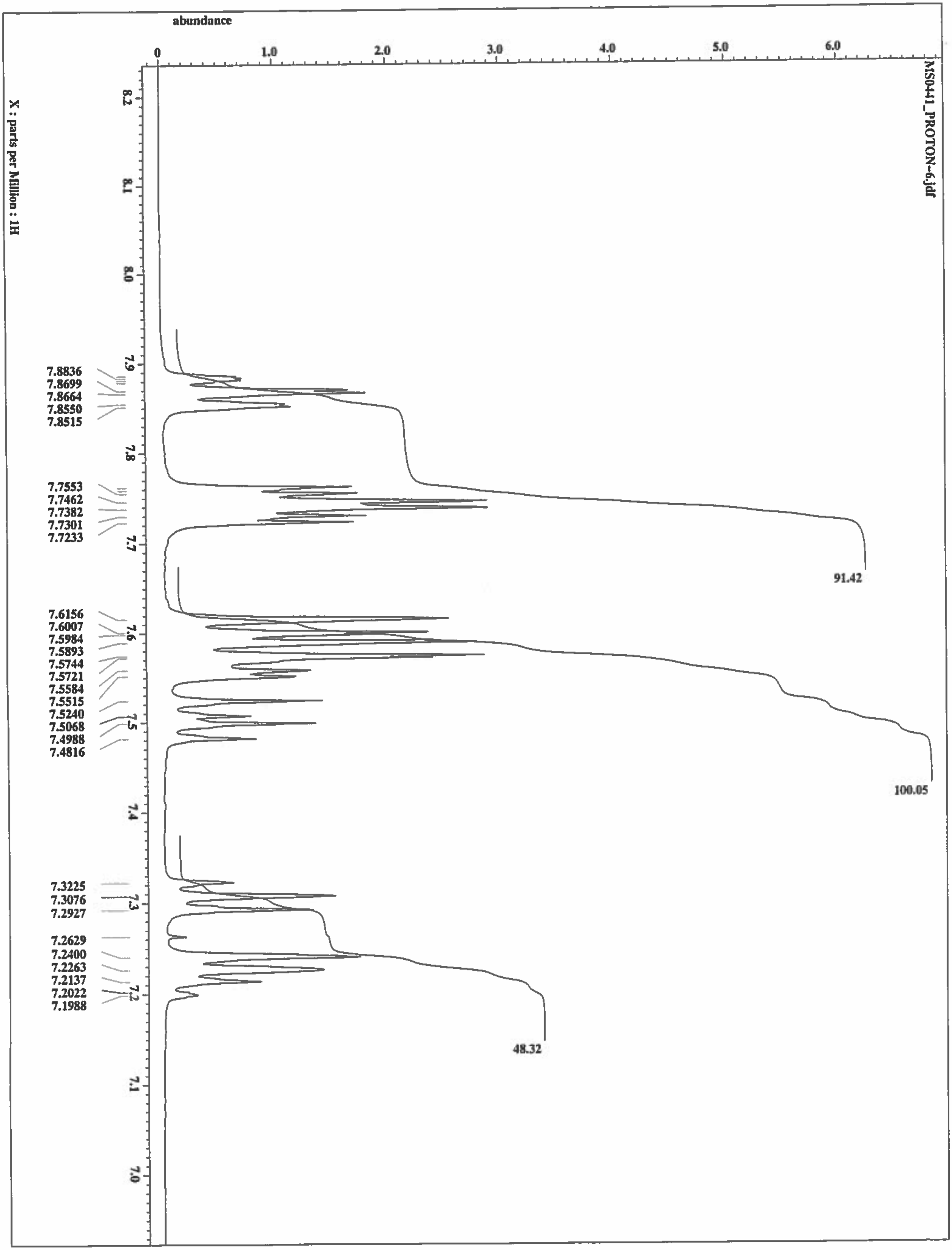
abundance
0
1.0
2.0
3.0
4.0
5.0
6.0
MS0441_PROTON-6.jdf
X : parts per Million : 1H
8.2
8.1
8.0
7.9
7.8
7.7
7.6
7.5
7.4
7.3
7.2
7.1
7.0
7.8836
7.8699
7.8664
7.8550
7.8515
7.7553
7.7462
7.7382
7.7301
7.7233
91.42
7.6156
7.6007
7.5984
7.5893
7.5744
7.5721
7.5584
7.5515
7.5240
7.5068
7.4988
7.4816
100.05
7.3225
7.3076
7.2927
7.2629
7.2400
7.2263
7.2137
7.2022
7.1988
48.32

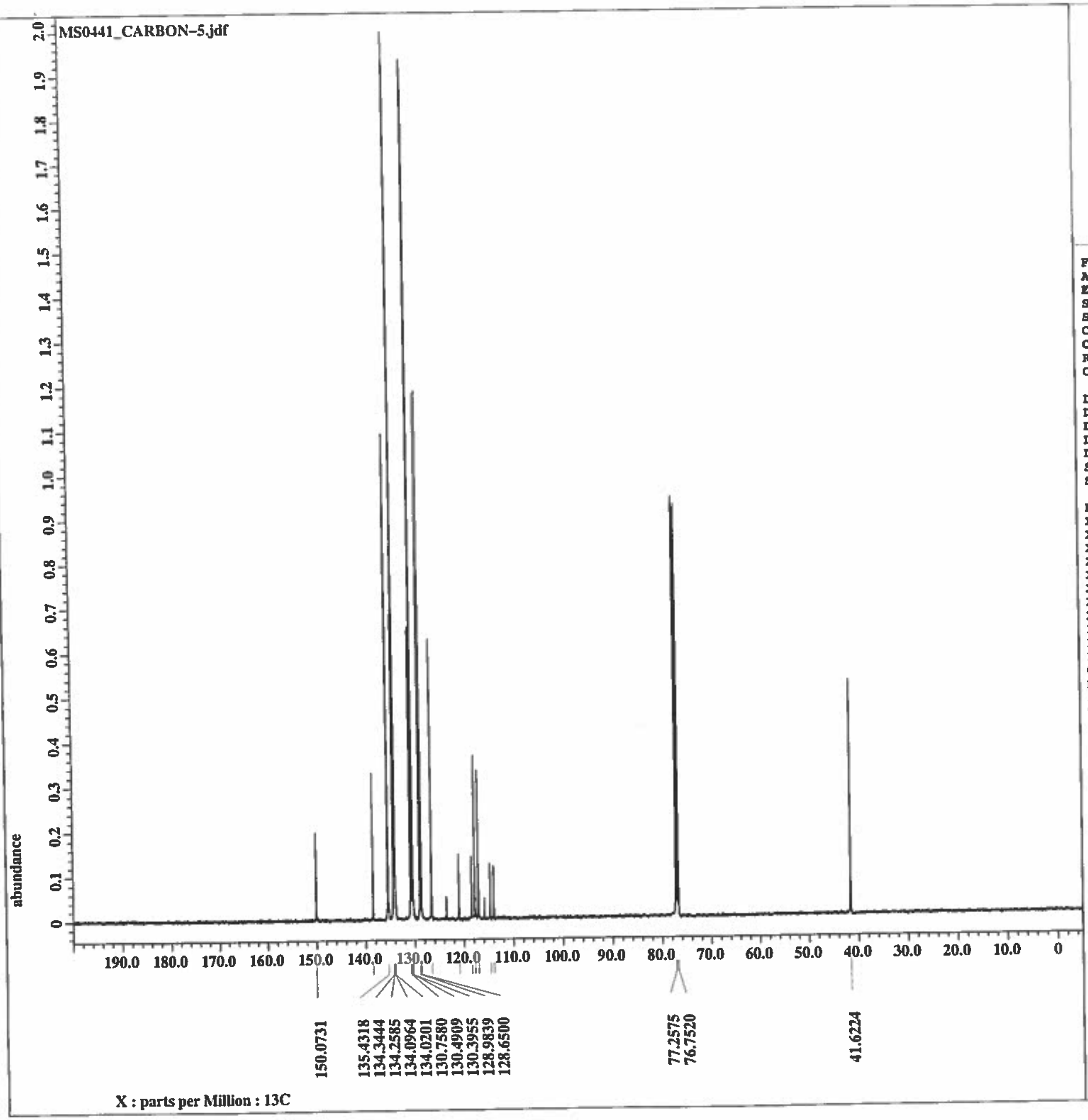

Filename	= MS0441_CARBON-5.jdf
Author	= Jim Davis
Experiment	= single_pulse_dec
Sample_id	= MS0441
Solvent	= CHLOROFORM-D
Changer_sample	= 15
Creation_time	= 9-MAY-2018 20:21:48
Revision_time	= 9-MAY-2018 19:57:58
Current_time	= 9-MAY-2018 19:57:58
Data_format	= 1D COMPLEX
Dim_size	= 26214
Dim_title	= 13C
Dim_units	= [ppm]
Dimensions	= X
Site	= ECA 500
Spectrometer	= JNM-ECA500
Field_strength	= 11.7473579[T] (500[MH
X_acq_duration	= 0.83361792[s]
X_domain	= 13C
X_freq	= 125.76529768[MHz]
X_offset	= 100[ppm]
X_points	= 32768
X_prescans	= 4
X_resolution	= 1.19959034[Hz]
X_sweep	= 39.3081761[kHz]
Irr_domain	= 1H
Irr_freq	= 500.15991521[MHz]
Irr_offset	= 5.0[ppm]
Clipped	= FALSE
Mod_return	= 1
Scans	= 1024
Total_scans	= 1024
X_90_width	= 13.2[us]
X_acq_time	= 0.83361792[s]
X_angle	= 30[deg]
X_atn	= 6[dB]
X_pulse	= 4.4[us]
Irr_atn_dec	= 20.7[dB]
Irr_atn_noe	= 20.7[dB]
Irr_noise	= WALTZ
Decoupling	= TRUE
Initial_wait	= 1[s]
Noe	= TRUE
Noe_time	= 2[s]
Recvr_gain	= 60
Relaxation_delay	= 2[s]
Repetition_time	= 2.83361792[s]
Temp_get	= 23.4[dC]

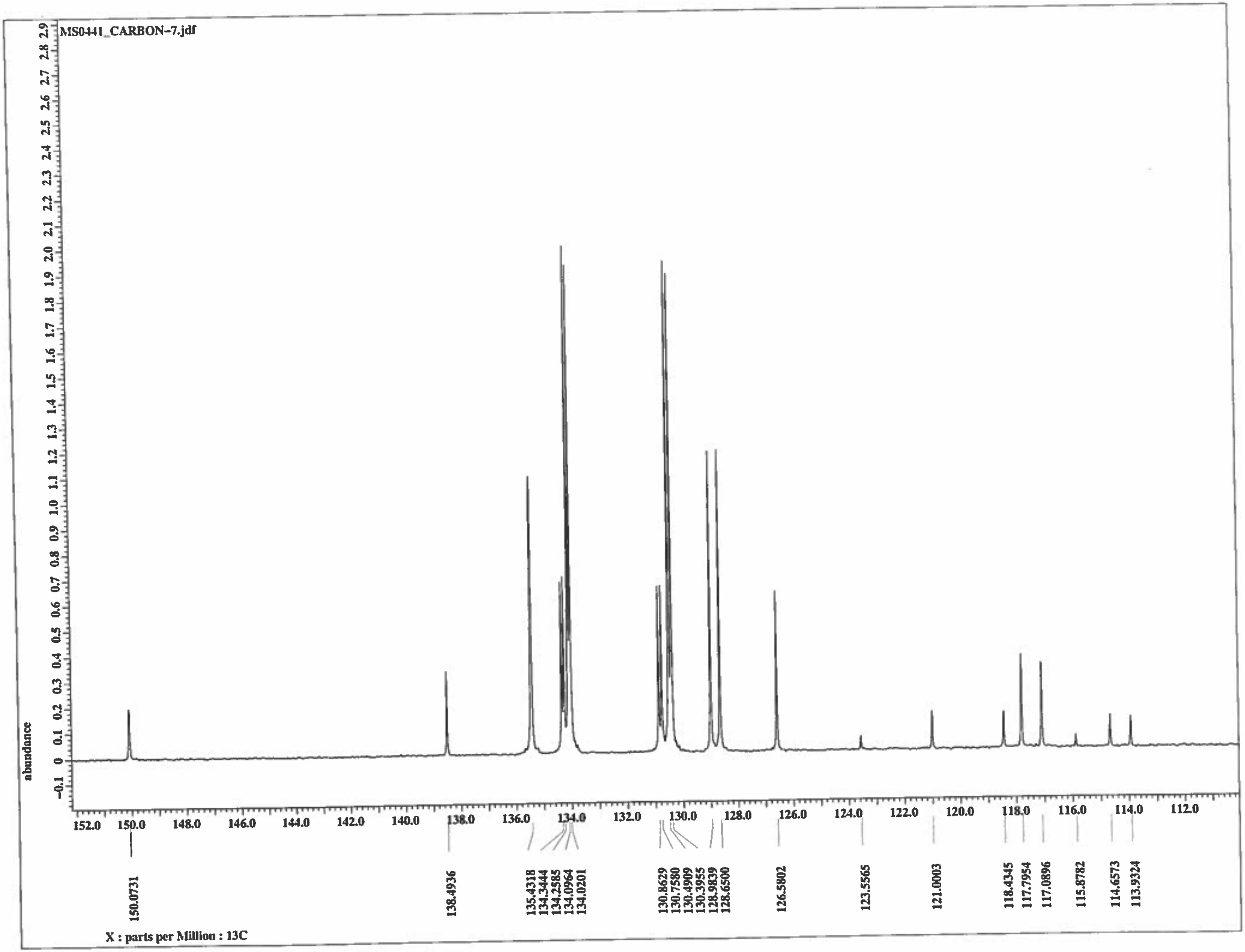

MS0441_CARBON-7.jdf
abundance
-0.1 0 0.1 0.2 0.3 0.4 0.5 0.6 0.7 0.8 0.9 1.0 1.1 1.2 1.3 1.4 1.5 1.6 1.7 1.8 1.9 2.0 2.1 2.2 2.3 2.4 2.5 2.6 2.7 2.8 2.9
152.0 150.0 148.0 146.0 144.0 142.0 140.0 138.0 136.0 134.0 132.0 130.0 128.0 126.0 124.0 122.0 120.0 118.0 116.0 114.0 112.0
150.0731
138.4936
135.4318
134.3444
134.2585
134.0964
134.0201
130.8629
130.7580
130.4909
130.3955
128.9839
128.6500
126.5802
123.5565
121.0003
118.4345
117.7954
117.0896
115.8782
114.6573
113.9324
X : parts per Million : 13C

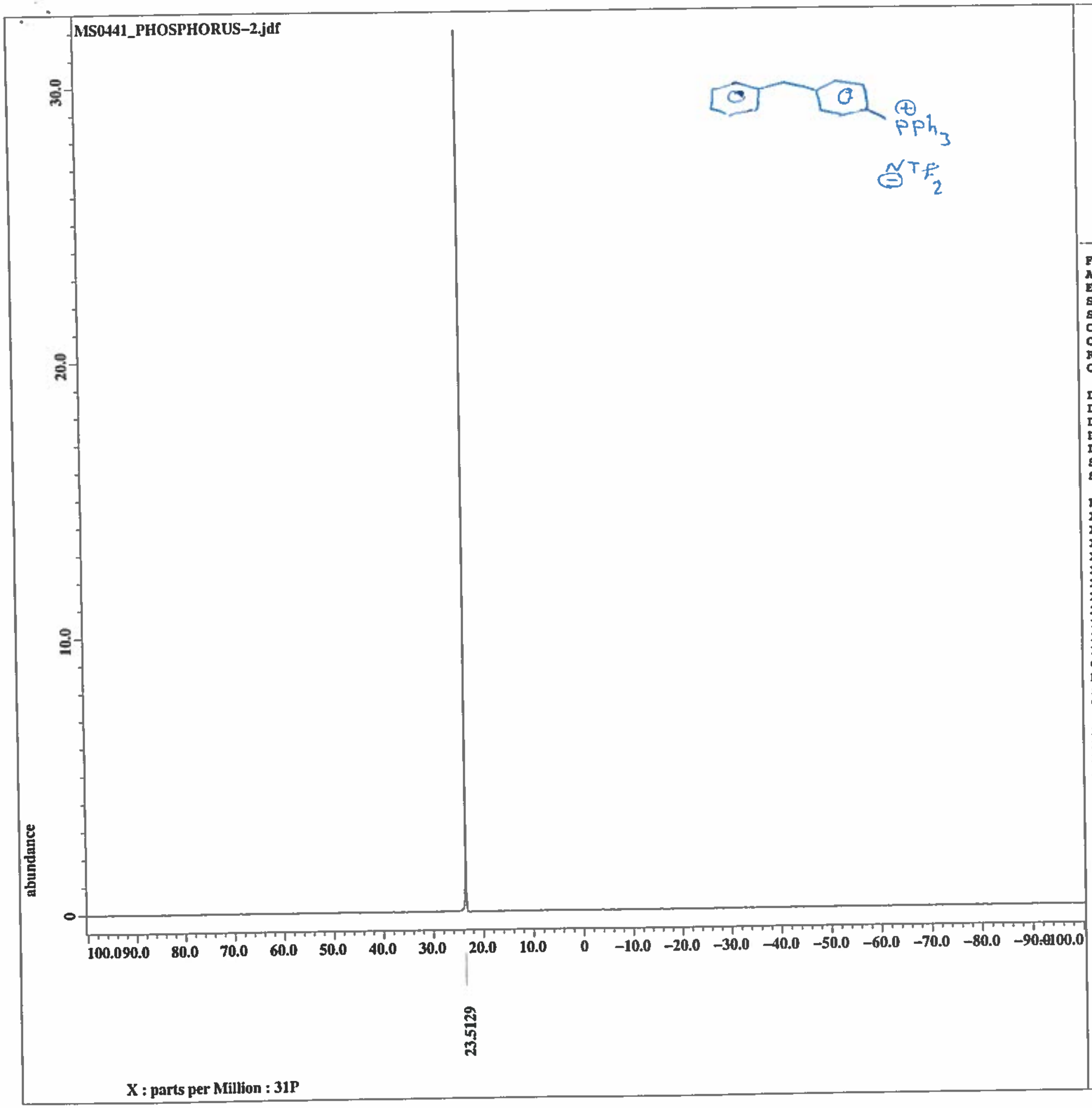

Filename	= MS0441_PHOSPHORUS-2.j
Author	= Jim Davis
Experiment	= single_pulse_dec
Sample_id	= MS0441
Solvent	= CHLOROFORM-D
Changer_sample	= 15
Creation_time	= 9-MAY-2018 17:17:05
Revision_time	= 9-MAY-2018 16:53:16
Current_time	= 9-MAY-2018 16:53:16
Data_format	= 1D COMPLEX
Dim_size	= 26214
Dim_title	= 31P
Dim_units	= [ppm]
Dimensions	= X
Site	= ECA 500
Spectrometer	= JNM-ECA500
Field_strength	= 11.7473579[T] (500[MH
X_acq_duration	= 0.64487424[s]
X_domain	= 31P
X_freq	= 202.46831075[MHz]
X_offset	= 0[ppm]
X_points	= 32768
X_prescans	= 4
X_resolution	= 1.55068995[Hz]
X_sweep	= 50.81300813[kHz]
Irr_domain	= 1H
Irr_freq	= 500.15991521[MHz]
Irr_offset	= 5.0[ppm]
Clipped	= FALSE
Mod_return	= 1
Scans	= 25
Total_scans	= 25
X_90_width	= 14.687[us]
X_acq_time	= 0.64487424[s]
X_angle	= 30[deg]
X_atn	= 5[dB]
X_pulse	= 4.89566667[us]
Irr_atn_dec	= 20.7[dB]
Irr_atn_noe	= 20.7[dB]
Irr_noise	= WALTZ
Decoupling	= TRUE
Initial_wait	= 1[s]
Noe	= TRUE
Noe_time	= 2[s]
Recvr_gain	= 54
Relaxation_delay	= 2[s]
Repetition_time	= 2.64487424[s]
Temp_get	= 22.6[dC]

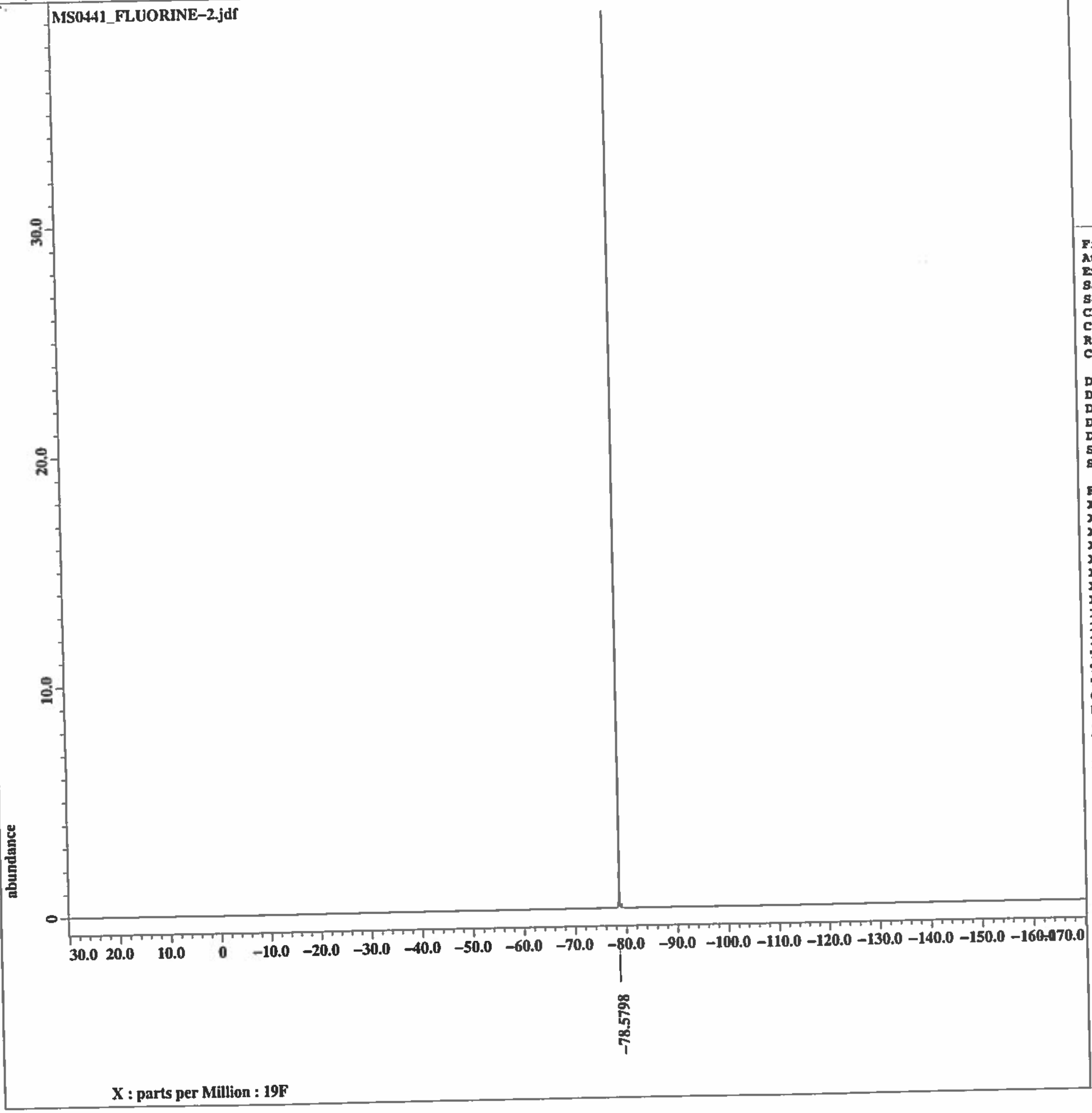

Filename	= MS0441_FLUORINE-2.jdf
Author	= Jim Davis
Experiment	= single_pulse.ex2
Sample_id	= MS0441
Solvent	= CHLOROFORM-D
Changer_sample	= 15
Creation_time	= 9-MAY-2018 17:19:52
Revision_time	= 9-MAY-2018 16:56:03
Current_time	= 9-MAY-2018 16:56:03
Data_format	= 1D COMPLEX
Dim_size	= 52428
Dim_title	= 19F
Dim_units	= [ppm]
Dimensions	= X
Site	= ECA 500
Spectrometer	= JNM-ECA500
Field_strength	= 11.7473579[T] (500[MH
X_acq_duration	= 0.55574528[s]
X_domain	= 19F
X_freq	= 470.62046084[MHz]
X_offset	= -70[ppm]
X_points	= 65536
X_prescans	= 1
X_resolution	= 1.7993855[Hz]
X_sweep	= 117.9245283[kHz]
Irr_domain	= 19F
Irr_freq	= 470.62046084[MHz]
Irr_offset	= 5[ppm]
Tri_domain	= 19F
Tri_freq	= 470.62046084[MHz]
Tri_offset	= 5[ppm]
Clipped	= FALSE
Mod_return	= 1
Scans	= 16
Total_scans	= 16
X_90_width	= 13.1[us]
X_acq_time	= 0.55574528[s]
X_angle	= 45[deg]
X_atn	= 2.5[dB]
X_pulse	= 6.55[us]
Irr_mode	= Off
Tri_mode	= Off
Dante_presat	= FALSE
Initial_wait	= 1[s]
Recvr_gain	= 36
Relaxation_delay	= 4[s]
Repetition_time	= 4.55574528[s]
Temp_get	= 22.4[dC]

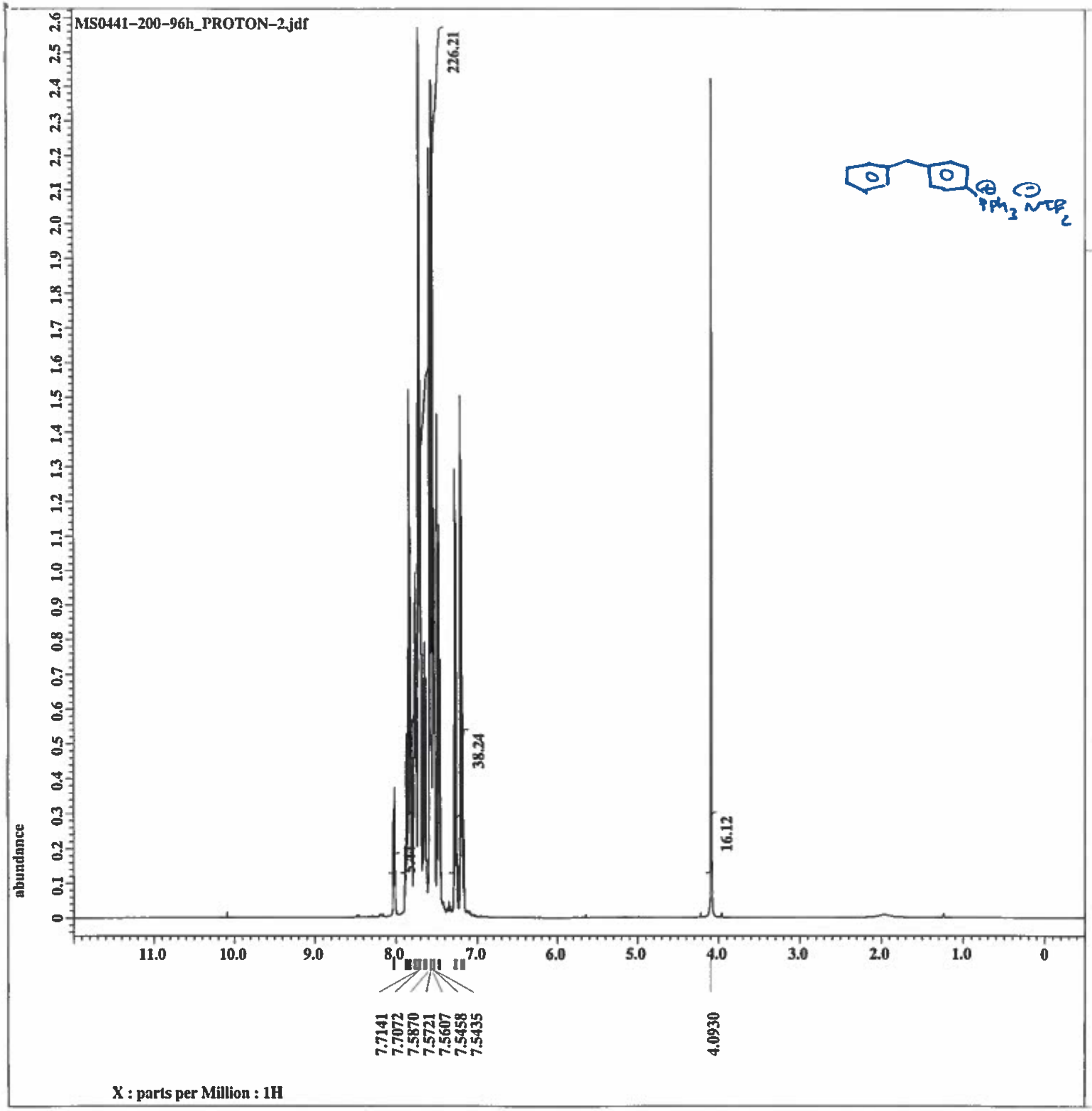

Filename	=	MS0441-200-96h_PROTON
Author	=	Jim Davis
Experiment	=	single_pulse.ex2
Sample_id	=	MS0441-200-96h
Solvent	=	CHLOROFORM-D
Changer_sample	=	2
Creation_time	=	14-MAY-2018 22:01:47
Revision_time	=	14-MAY-2018 21:37:32
Current_time	=	14-MAY-2018 21:37:32
Data_format	=	1D COMPLEX
Dim_size	=	13107
Dim_title	=	1H
Dim_units	=	[ppm]
Dimensions	=	X
Site	=	ECA 500
Spectrometer	=	JNM-ECA500
Field_strength	=	11.7473579[T] (500[MH
X_acq_duration	=	1.74587904[s]
X_domain	=	1H
X_freq	=	500.15991521[MHz]
X_offset	=	5.0[ppm]
X_points	=	16384
X_prescans	=	1
X_resolution	=	0.57277737[Hz]
X_sweep	=	9.38438438[kHz]
Irr_domain	=	1H
Irr_freq	=	500.15991521[MHz]
Irr_offset	=	5.0[ppm]
Tri_domain	=	1H
Tri_freq	=	500.15991521[MHz]
Tri_offset	=	5.0[ppm]
Clipped	=	FALSE
Mod_return	=	1
Scans	=	16
Total_scans	=	16
X_90_width	=	12.4[us]
X_acq_time	=	1.74587904[s]
X_angle	=	45[deg]
X_atn	=	4[dB]
X_pulse	=	6.2[us]
Irr_mode	=	Off
Tri_mode	=	Off
Dante_presat	=	FALSE
Initial_wait	=	1[s]
Recvr_gain	=	22
Relaxation_delay	=	4[s]
Repetition_time	=	5.74587904[s]
Temp_get	=	22.2[dC]

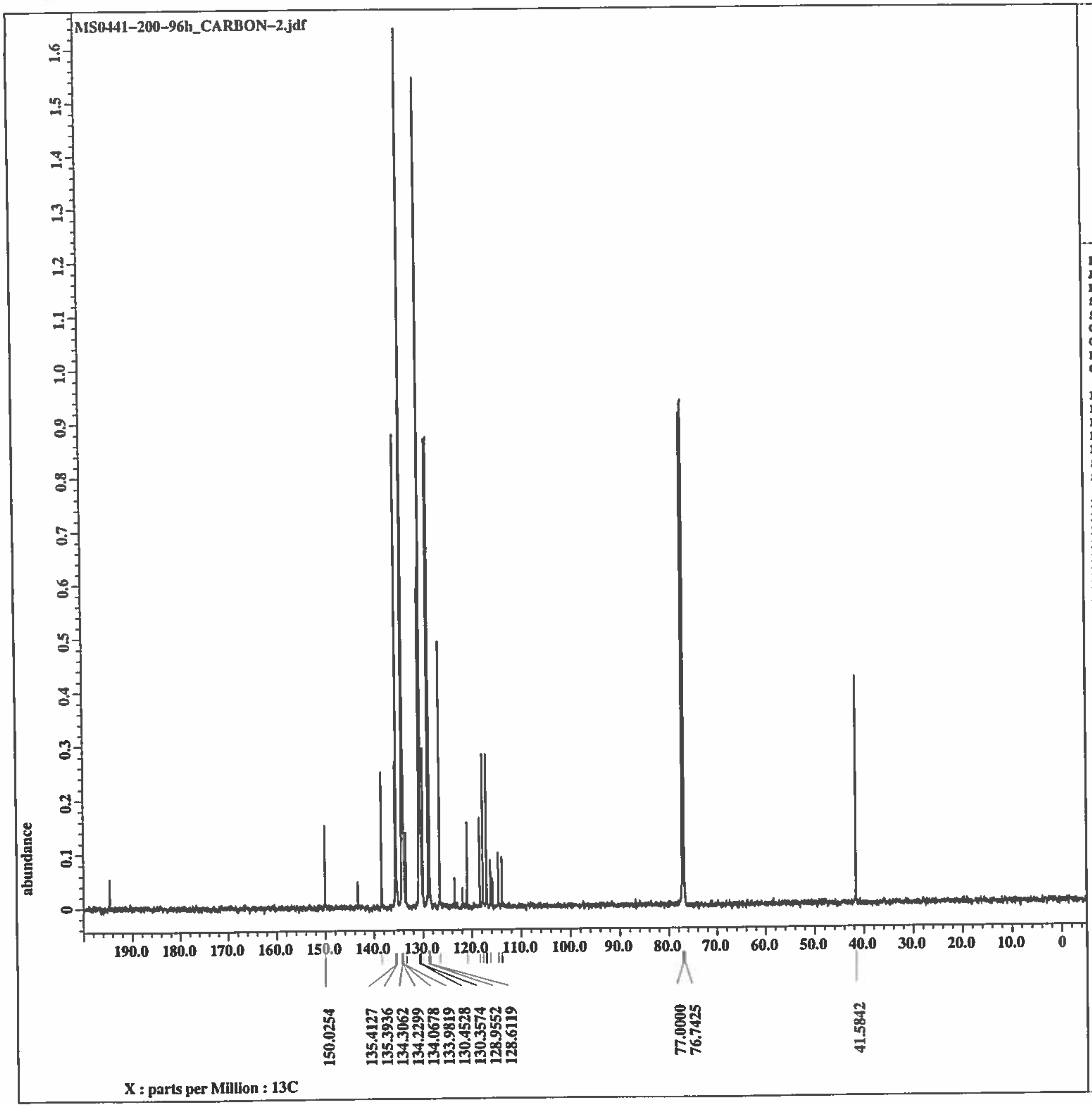

Filename	= MS0441-200-96h_CARBON
Author	= Jim Davis
Experiment	= single_pulse_dec
Sample_id	= MS0441-200-96h
Solvent	= CHLOROFORM-D
Changer_sample	= 2
Creation_time	= 14-MAY-2018 22:22:55
Revision_time	= 14-MAY-2018 21:58:40
Current_time	= 14-MAY-2018 21:58:40
Data_format	= 1D COMPLEX
Dim_size	= 26214
Dim_title	= 13C
Dim_units	= [ppm]
Dimensions	= X
Site	= ECA 500
Spectrometer	= JNM-ECA500
Field_strength	= 11.7473579[T] (500[MH
X_acq_duration	= 0.83361792[s]
X_domain	= 13C
X_freq	= 125.76529768[MHz]
X_offset	= 100[ppm]
X_points	= 32768
X_prescans	= 4
X_resolution	= 1.19959034[Hz]
X_sweep	= 39.3081761[kHz]
Irr_domain	= 1H
Irr_freq	= 500.15991521[MHz]
Irr_offset	= 5.0[ppm]
Clipped	= FALSE
Mod_return	= 1
Scans	= 400
Total_scans	= 400
X_90_width	= 13.2[us]
X_acq_time	= 0.83361792[s]
X_angle	= 30[deg]
X_atn	= 6[dB]
X_pulse	= 4.4[us]
Irr_atn_dec	= 20.7[dB]
Irr_atn_noe	= 20.7[dB]
Irr_noise	= WALTZ
Decoupling	= TRUE
Initial_wait	= 1[s]
Noe	= TRUE
Noe_time	= 2[s]
Recvr_gain	= 60
Relaxation_delay	= 2[s]
Repetition_time	= 2.83361792[s]
Temp_get	= 23.3[dC]

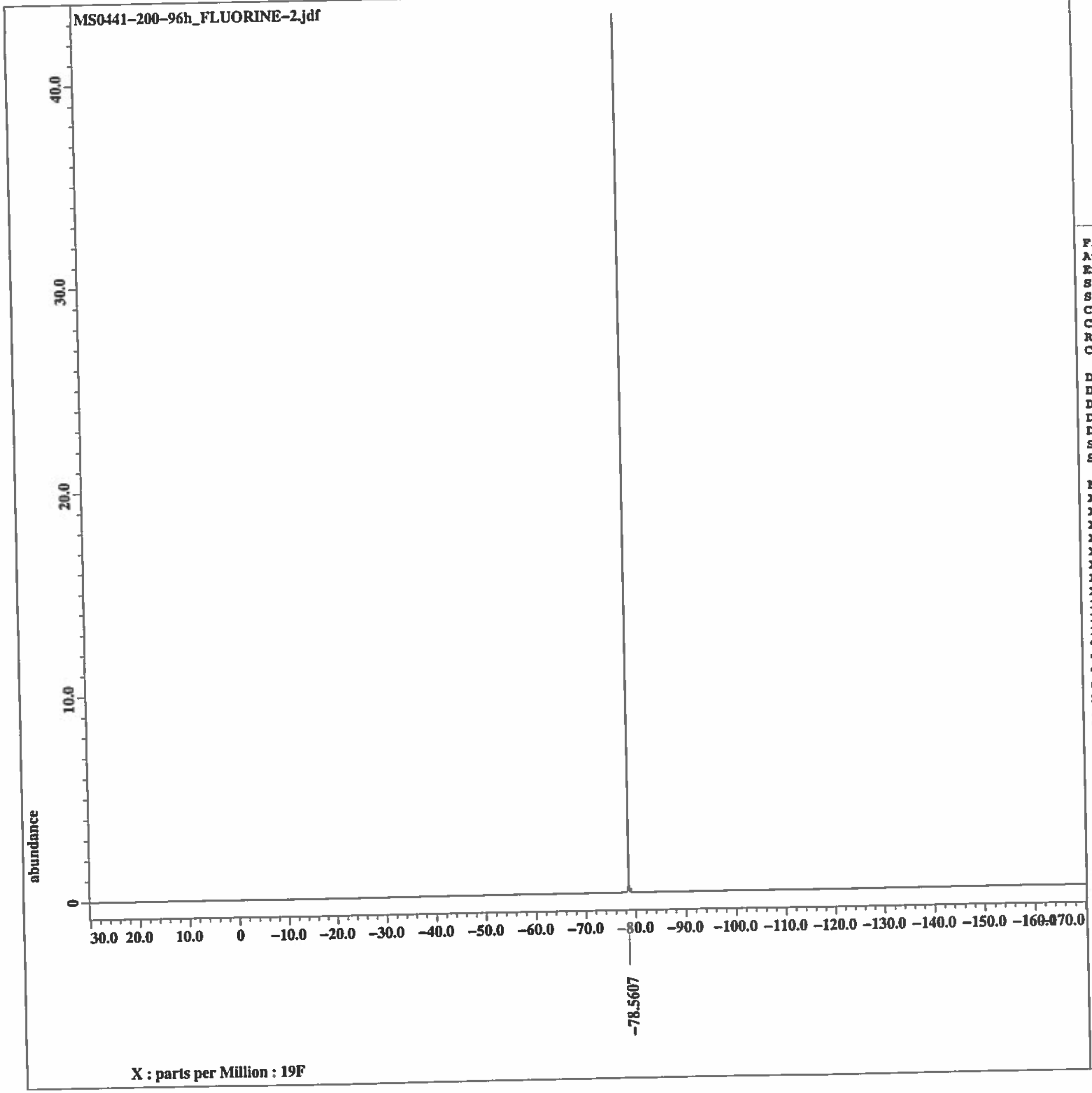

SOUTH ALABAMA JAGUARS

Filename	= MS0441-200-96h_FLUORI
Author	= Jim Davis
Experiment	= single_pulse.ex2
Sample_id	= MS0441-200-96h
Solvent	= CHLOROFORM-D
Changer_sample	= 2
Creation_time	= 14-MAY-2018 22:25:53
Revision_time	= 14-MAY-2018 22:01:37
Current_time	= 14-MAY-2018 22:01:37
Data_format	= 1D COMPLEX
Dim_size	= 52428
Dim_title	= 19F
Dim_units	= [ppm]
Dimensions	= X
Site	= ECA 500
Spectrometer	= JNM-ECA500
Field_strength	= 11.7473579[T] (500[MH
X_acq_duration	= 0.55574528[s]
X_domain	= 19F
X_freq	= 470.62046084[MHz]
X_offset	= -70[ppm]
X_points	= 65536
X_prescans	= 1
X_resolution	= 1.7993855[Hz]
X_sweep	= 117.9245283[kHz]
Irr_domain	= 19F
Irr_freq	= 470.62046084[MHz]
Irr_offset	= 5[ppm]
Tri_domain	= 19F
Tri_freq	= 470.62046084[MHz]
Tri_offset	= 5[ppm]
Clipped	= FALSE
Mod_return	= 1
Scans	= 16
Total_scans	= 16
X_90_width	= 13.1[us]
X_acq_time	= 0.55574528[s]
X_angle	= 45[deg]
X_atn	= 2.5[dB]
X_pulse	= 6.55[us]
Irr_mode	= Off
Tri_mode	= Off
Dante_presat	= FALSE
Initial_wait	= 1[s]
Recvr_gain	= 38
Relaxation_delay	= 4[s]
Repetition_time	= 4.55574528[s]
Temp_get	= 22.9[dC]

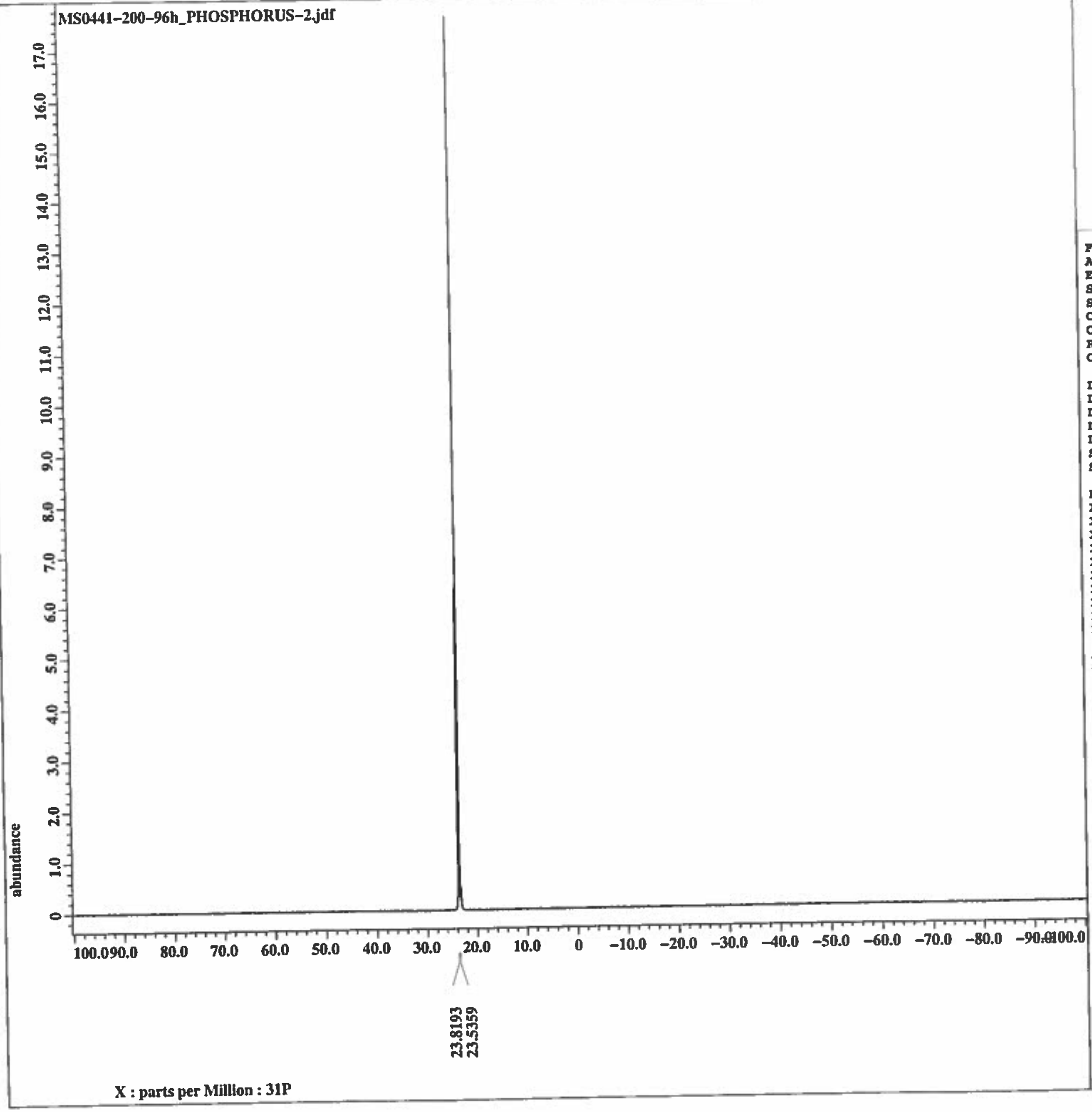

Filename	= MS0441-200-96h_PHOSPH
Author	= Jim Davis
Experiment	= single_pulse_dec
Sample_id	= MS0441-200-96h
Solvent	= CHLOROFORM-D
Changer_sample	= 2
Creation_time	= 14-MAY-2018 22:30:32
Revision_time	= 14-MAY-2018 22:06:15
Current_time	= 14-MAY-2018 22:06:15
Data_format	= 1D COMPLEX
Dim_size	= 26214
Dim_title	= 31P
Dim_units	= [ppm]
Dimensions	= X
Site	= ECA 500
Spectrometer	= JNM-ECA500
Field_strength	= 11.7473579[T] (500[MH
X_acq_duration	= 0.64487424[s]
X_domain	= 31P
X_freq	= 202.46831075[MHz]
X_offset	= 0[ppm]
X_points	= 32768
X_prescans	= 4
X_resolution	= 1.55068995[Hz]
X_sweep	= 50.81300813[kHz]
Irr_domain	= 1H
Irr_freq	= 500.15991521[MHz]
Irr_offset	= 5.0[ppm]
Clipped	= FALSE
Mod_return	= 1
Scans	= 50
Total_scans	= 50
X_90_width	= 14.687[us]
X_acq_time	= 0.64487424[s]
X_angle	= 30[deg]
X_atn	= 5[dB]
X_pulse	= 4.89566667[us]
Irr_atn_dec	= 20.7[dB]
Irr_atn_noe	= 20.7[dB]
Irr_noise	= WALTZ
Decoupling	= TRUE
Initial_wait	= 1[s]
Noe	= TRUE
Noe_time	= 2[s]
Recvr_gain	= 56
Relaxation_delay	= 2[s]
Repetition_time	= 2.64487424[s]
Temp_get	= 23.1[dC]

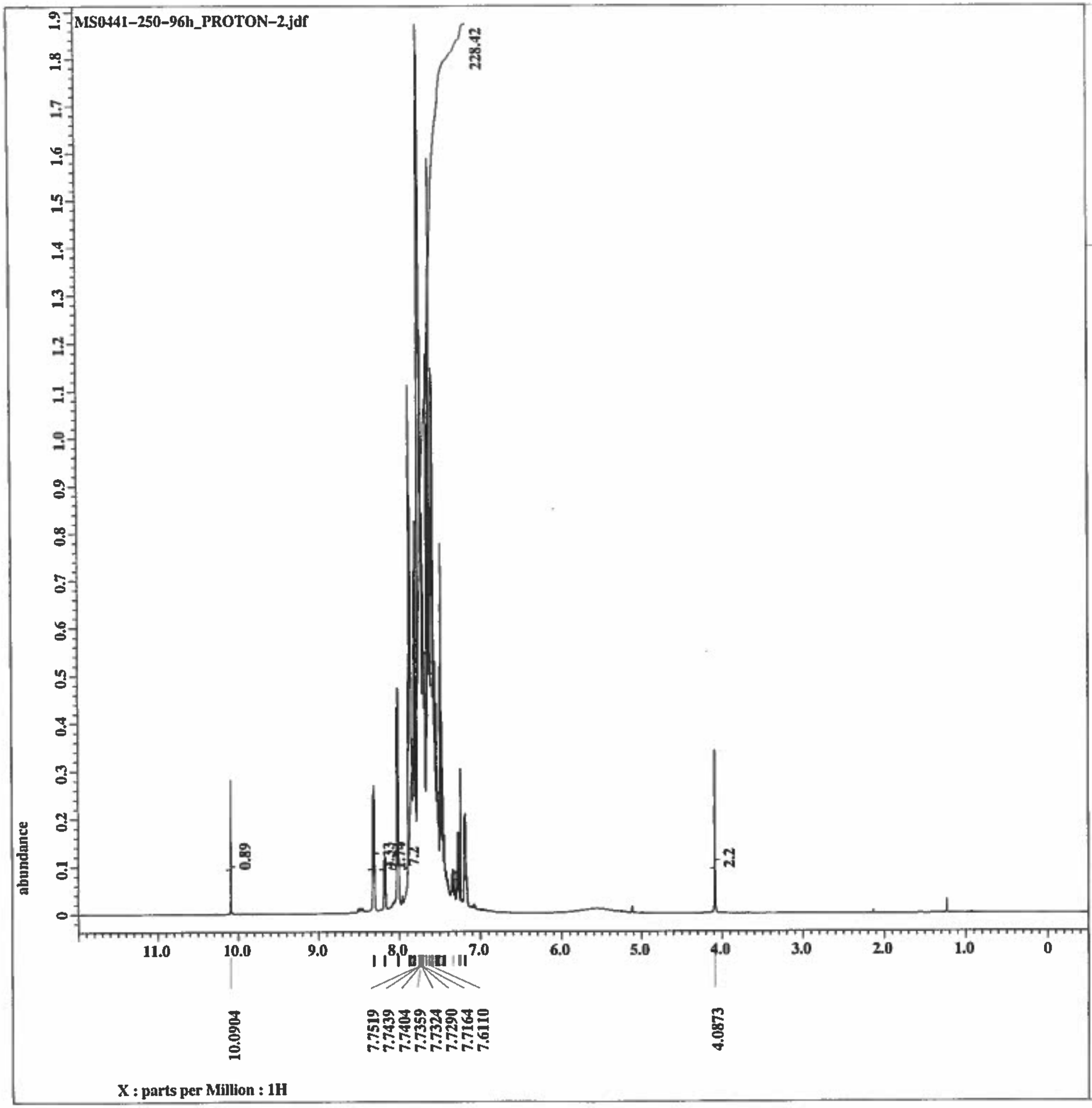

Filename	= MS0441-250-96h_PROTON
Author	= Jim Davis
Experiment	= single_pulse.ex2
Sample_id	= MS0441-250-96h
Solvent	= CHLOROFORM-D
Changer_sample	= 3
Creation_time	= 14-MAY-2018 22:37:44
Revision_time	= 14-MAY-2018 22:13:27
Current_time	= 14-MAY-2018 22:13:27
Data_format	= 1D COMPLEX
Dim_size	= 13107
Dim_title	= 1H
Dim_units	= [ppm]
Dimensions	= X
Site	= ECA 500
Spectrometer	= JNM-ECA500
Field_strength	= 11.7473579[T] (500[MH
X_acq_duration	= 1.74587904[s]
X_domain	= 1H
X_freq	= 500.15991521[MHz]
X_offset	= 5.0[ppm]
X_points	= 16384
X_prescans	= 1
X_resolution	= 0.57277737[Hz]
X_sweep	= 9.38438438[kHz]
Irr_domain	= 1H
Irr_freq	= 500.15991521[MHz]
Irr_offset	= 5.0[ppm]
Tri_domain	= 1H
Tri_freq	= 500.15991521[MHz]
Tri_offset	= 5.0[ppm]
Clipped	= FALSE
Mod_return	= 1
Scans	= 16
Total_scans	= 16
X_90_width	= 12.4[us]
X_acq_time	= 1.74587904[s]
X_angle	= 45[deg]
X_atn	= 4[dB]
X_pulse	= 6.2[us]
Irr_mode	= Off
Tri_mode	= Off
Dante_presat	= FALSE
Initial_wait	= 1[s]
Recvr_gain	= 26
Relaxation_delay	= 4[s]
Repetition_time	= 5.74587904[s]
Temp_get	= 22.2[dC]

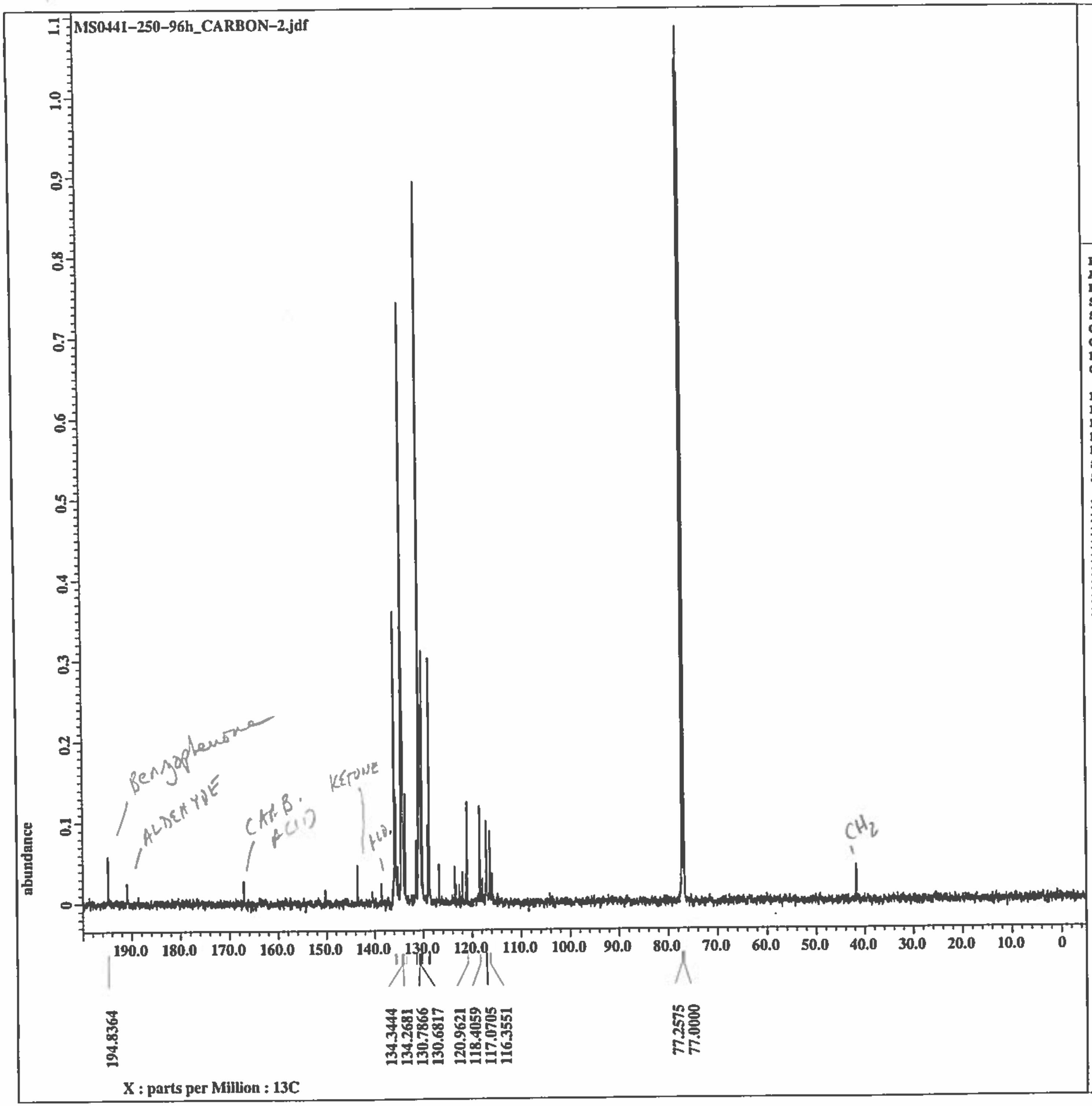

Filename	= MS0441-250-96h_CARBON
Author	= Jim Davis
Experiment	= single_pulse_dec
Sample_id	= MS0441-250-96h
Solvent	= CHLOROFORM-D
Changer_sample	= 3
Creation_time	= 14-MAY-2018 22:58:55
Revision_time	= 14-MAY-2018 22:34:38
Current_time	= 14-MAY-2018 22:34:38
Data_format	= 1D COMPLEX
Dim_size	= 26214
Dim_title	= 13C
Dim_units	= [ppm]
Dimensions	= X
Site	= ECA 500
Spectrometer	= JNM-ECA500
Field_strength	= 11.7473579[T] (500[MH
X_acq_duration	= 0.83361792[s]
X_domain	= 13C
X_freq	= 125.76529768[MHz]
X_offset	= 100[ppm]
X_points	= 32768
X_prescans	= 4
X_resolution	= 1.19959034[Hz]
X_sweep	= 39.3081761[kHz]
Irr_domain	= 1H
Irr_freq	= 500.15991521[MHz]
Irr_offset	= 5.0[ppm]
Clipped	= FALSE
Mod_return	= 1
Scans	= 400
Total_scans	= 400
X_90_width	= 13.2[us]
X_acq_time	= 0.83361792[s]
X_angle	= 30[deg]
X_atn	= 6[dB]
X_pulse	= 4.4[us]
Irr_atn_dec	= 20.7[dB]
Irr_atn_noe	= 20.7[dB]
Irr_noise	= WALTZ
Decoupling	= TRUE
Initial_wait	= 1[s]
Noe	= TRUE
Noe_time	= 2[s]
Recvr_gain	= 60
Relaxation_delay	= 2[s]
Repetition_time	= 2.83361792[s]
Temp_get	= 23.3[dC]

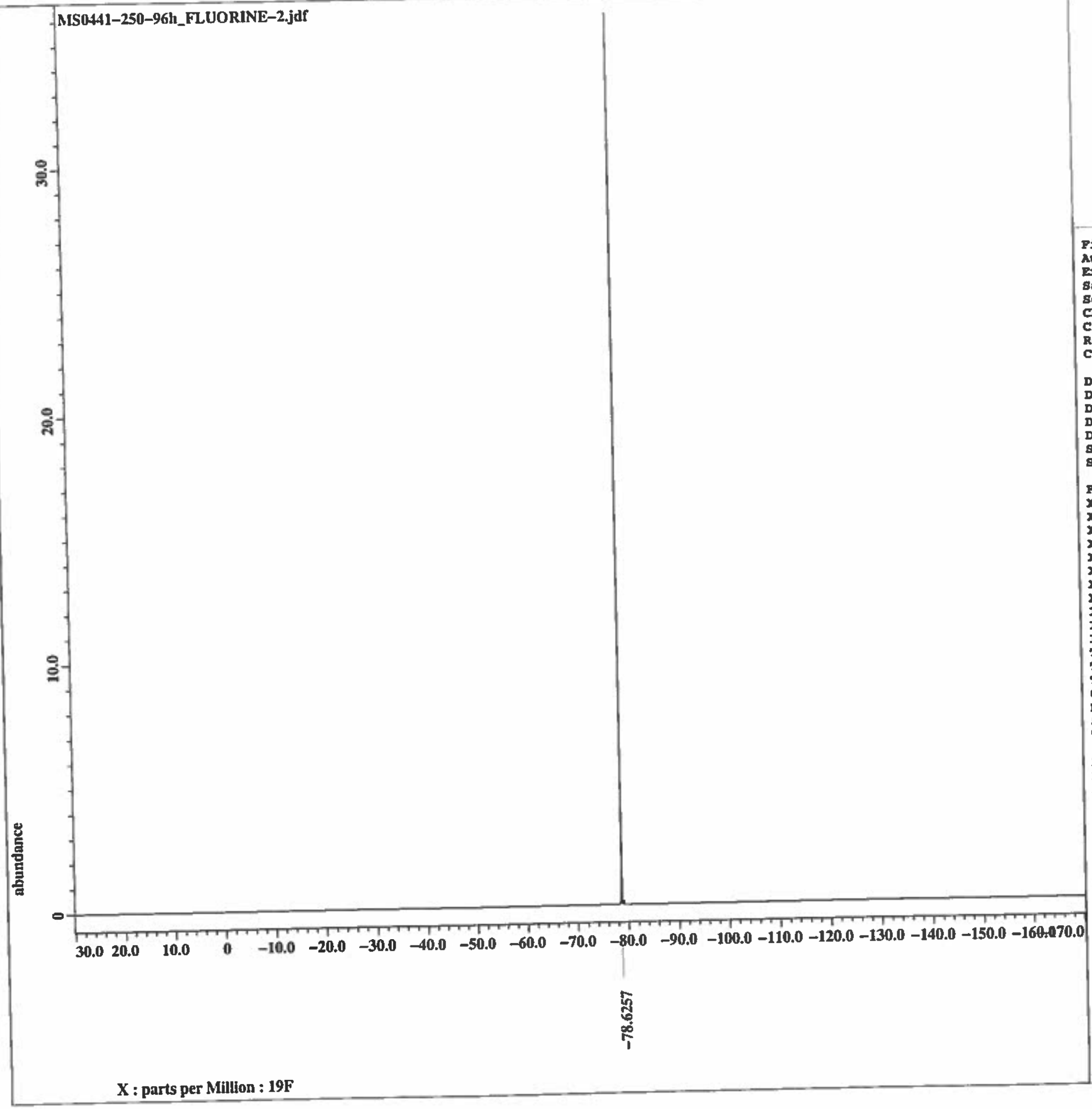

Filename	= MS0441-250-96h_FLUORI
Author	= Jim Davis
Experiment	= single_pulse.ex2
Sample_id	= MS0441-250-96h
Solvent	= CHLOROFORM-D
Changer_sample	= 3
Creation_time	= 14-MAY-2018 23:01:53
Revision_time	= 14-MAY-2018 22:37:37
Current_time	= 14-MAY-2018 22:37:37
Data_format	= 1D COMPLEX
Dim_size	= 52428
Dim_title	= 19F
Dim_units	= [ppm]
Dimensions	= X
Site	= ECA 500
Spectrometer	= JNM-ECA500
Field_strength	= 11.7473579[T] (500[MH
X_acq_duration	= 0.55574528[s]
X_domain	= 19F
X_freq	= 470.62046084[MHz]
X_offset	= -70[ppm]
X_points	= 65536
X_prescans	= 1
X_resolution	= 1.7993855[Hz]
X_sweep	= 117.9245283[kHz]
Irr_domain	= 19F
Irr_freq	= 470.62046084[MHz]
Irr_offset	= 5[ppm]
Tri_domain	= 19F
Tri_freq	= 470.62046084[MHz]
Tri_offset	= 5[ppm]
Clipped	= FALSE
Mod_return	= 1
Scans	= 16
Total_scans	= 16
X_90_width	= 13.1[us]
X_acq_time	= 0.55574528[s]
X_angle	= 45[deg]
X_atn	= 2.5[dB]
X_pulse	= 6.55[us]
Irr_mode	= Off
Tri_mode	= Off
Dante_presat	= FALSE
Initial_wait	= 1[s]
Recvr_gain	= 38
Relaxation_delay	= 4[s]
Repetition_time	= 4.55574528[s]
Temp_get	= 22.9[dC]

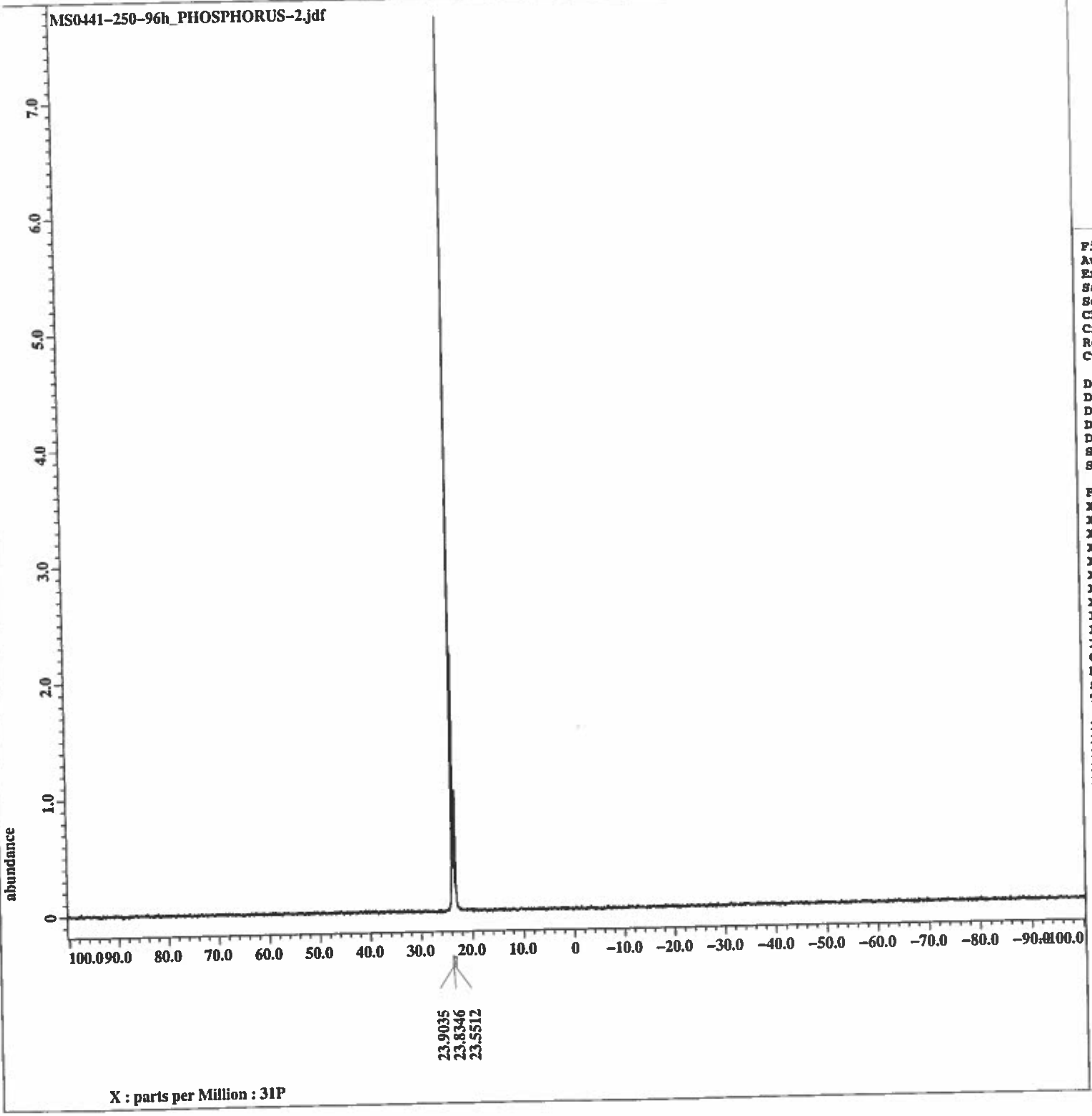

Filename	= MS0441-250-96h_PHOSPH
Author	= Jim Davis
Experiment	= single_pulse_dec
Sample_id	= MS0441-250-96h
Solvent	= CHLOROFORM-D
Changer_sample	= 3
Creation_time	= 14-MAY-2018 23:06:31
Revision_time	= 14-MAY-2018 22:42:14
Current_time	= 14-MAY-2018 22:42:14
Data_format	= 1D COMPLEX
Dim_size	= 26214
Dim_title	= 31P
Dim_units	= [ppm]
Dimensions	= X
Site	= ECA 500
Spectrometer	= JNM-ECA500
Field_strength	= 11.7473579[T] (500[MH
X_acq_duration	= 0.64487424[s]
X_domain	= 31P
X_freq	= 202.46831075[MHz]
X_offset	= 0[ppm]
X_points	= 32768
X_prescans	= 4
X_resolution	= 1.55068995[Hz]
X_sweep	= 50.81300813[kHz]
Irr_domain	= 1H
Irr_freq	= 500.15991521[MHz]
Irr_offset	= 5.0[ppm]
Clipped	= FALSE
Mod_return	= 1
Scans	= 50
Total_scans	= 50
X_90_width	= 14.687[us]
X_acq_time	= 0.64487424[s]
X_angle	= 30[deg]
X_atn	= 5[dB]
X_pulse	= 4.89566667[us]
Irr_atn_dec	= 20.7[dB]
Irr_atn_noe	= 20.7[dB]
Irr_noise	= WALTZ
Decoupling	= TRUE
Initial_wait	= 1[s]
Noe	= TRUE
Noe_time	= 2[s]
Recvr_gain	= 58
Relaxation_delay	= 2[s]
Repetition_time	= 2.64487424[s]
Temp_get	= 23[dC]

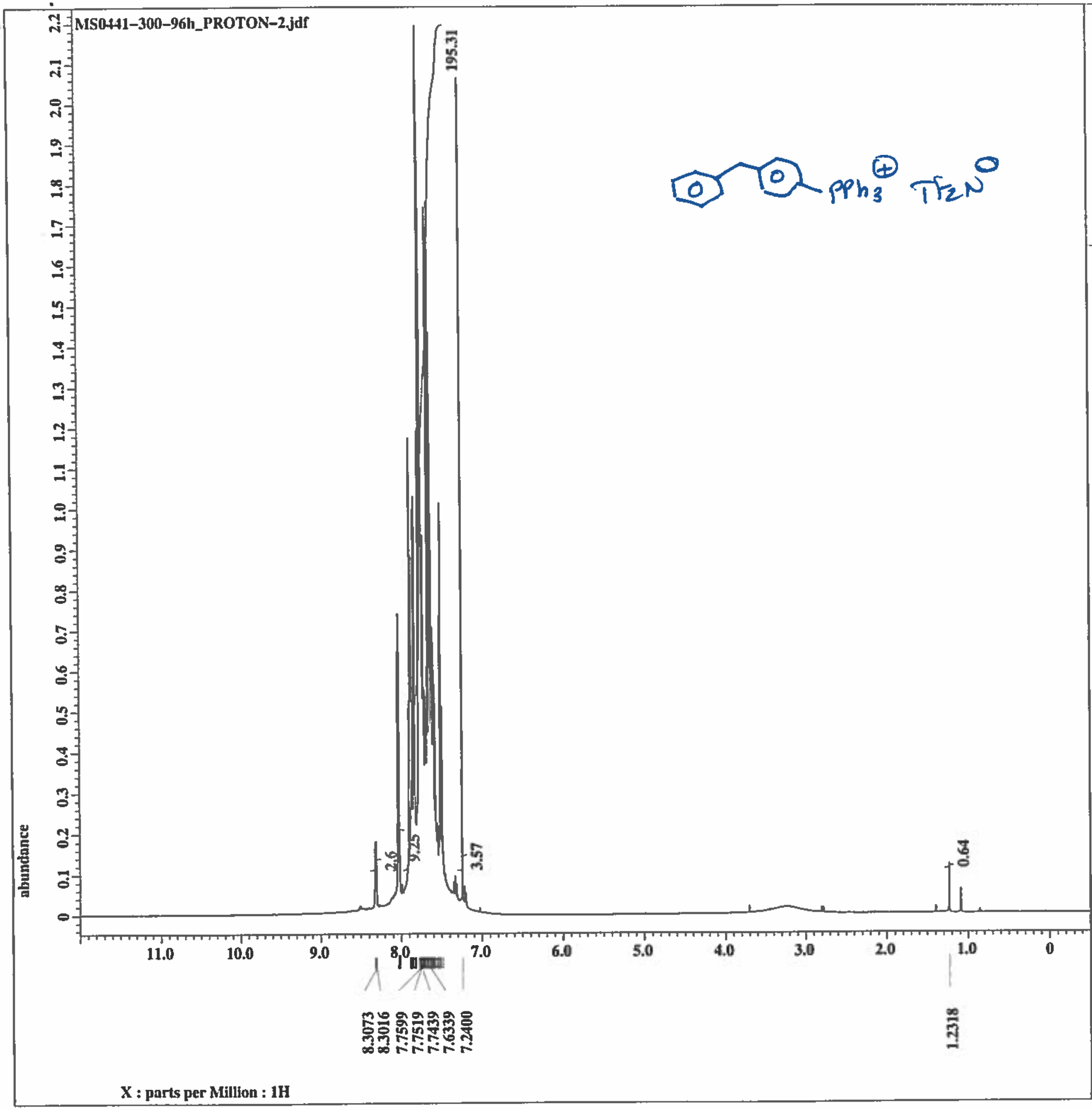

Filename	= MS0441-300-96h_PROTON
Author	= Jim Davis
Experiment	= single_pulse.ex2
Sample_id	= MS0441-300-96h
Solvent	= CHLOROFORM-D
Changer_sample	= 4
Creation_time	= 14-MAY-2018 23:13:50
Revision_time	= 14-MAY-2018 22:49:33
Current_time	= 14-MAY-2018 22:49:33
Data_format	= 1D COMPLEX
Dim_size	= 13107
Dim_title	= 1H
Dim_units	= [ppm]
Dimensions	= X
Site	= ECA 500
Spectrometer	= JNM-ECA500
Field_strength	= 11.7473579[T] (500[MH
X_acq_duration	= 1.74587904[s]
X_domain	= 1H
X_freq	= 500.15991521[MHz]
X_offset	= 5.0[ppm]
X_points	= 16384
X_prescans	= 1
X_resolution	= 0.57277737[Hz]
X_sweep	= 9.38438438[kHz]
Irr_domain	= 1H
Irr_freq	= 500.15991521[MHz]
Irr_offset	= 5.0[ppm]
Tri_domain	= 1H
Tri_freq	= 500.15991521[MHz]
Tri_offset	= 5.0[ppm]
Clipped	= FALSE
Mod_return	= 1
Scans	= 16
Total_scans	= 16
X_90_width	= 12.4[us]
X_acq_time	= 1.74587904[s]
X_angle	= 45[deg]
X_atn	= 4[dB]
X_pulse	= 6.2[us]
Irr_mode	= Off
Tri_mode	= Off
Dante_presat	= FALSE
Initial_wait	= 1[s]
Recvr_gain	= 38
Relaxation_delay	= 4[s]
Repetition_time	= 5.74587904[s]
Temp_get	= 22.2[dC]

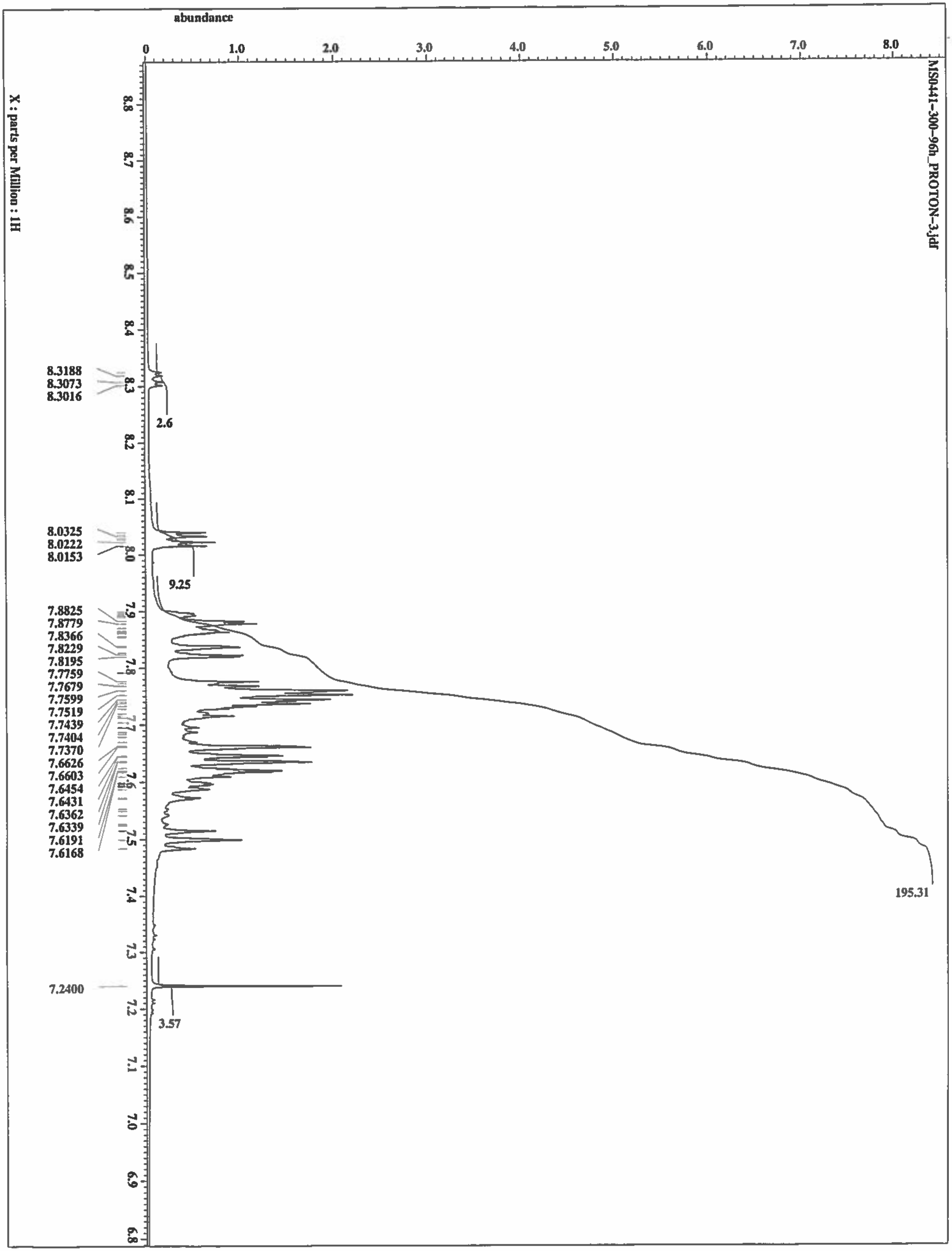

abundance
0
1.0
2.0
3.0
4.0
5.0
6.0
7.0
8.0
MS0441-300-96h_PROTON-3.jdf
X : parts per Million : 1H
8.8
8.7
8.6
8.5
8.4
8.3
8.2
8.1
8.0
7.9
7.8
7.7
7.6
7.5
7.4
7.3
7.2
7.1
7.0
6.9
6.8
8.3188
8.3073
8.3016
2.6
8.0325
8.0222
8.0153
9.25
7.8825
7.8779
7.8366
7.8229
7.8195
7.7759
7.7679
7.7599
7.7519
7.7439
7.7404
7.7370
7.6626
7.6603
7.6454
7.6431
7.6362
7.6339
7.6191
7.6168
195.31
7.2400
3.57

MS0441-300-96h_CARBON-2.jdf

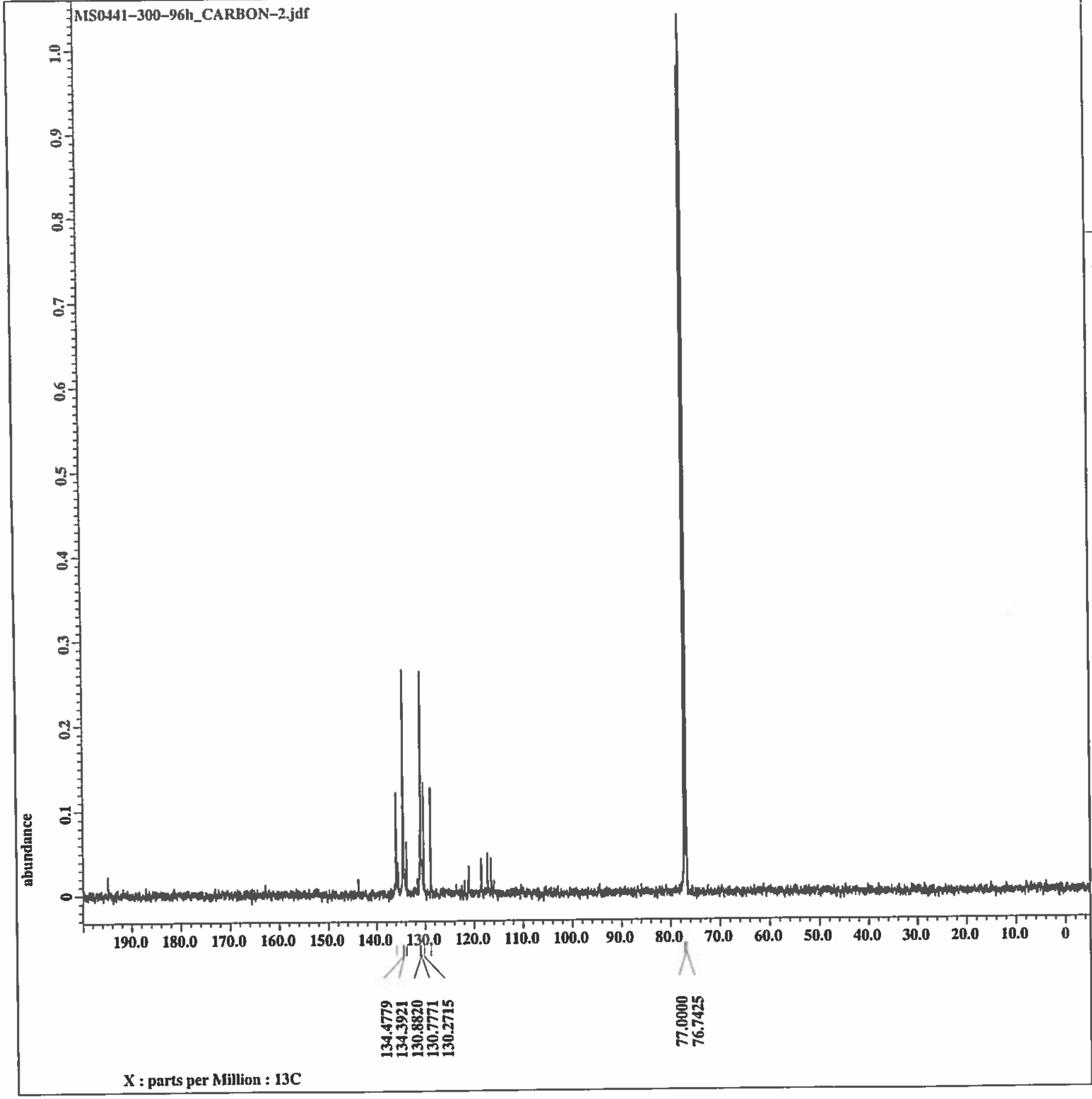

Filename	= MS0441-300-96h_CARBON
Author	= Jim Davis
Experiment	= single_pulse_dec
Sample_id	= MS0441-300-96h
Solvent	= CHLOROFORM-D
Changer_sample	= 4
Creation_time	= 14-MAY-2018 23:35:02
Revision_time	= 14-MAY-2018 23:10:45
Current_time	= 14-MAY-2018 23:10:45
Data_format	= 1D COMPLEX
Dim_size	= 26214
Dim_title	= 13C
Dim_units	= [ppm]
Dimensions	= X
Site	= ECA 500
Spectrometer	= JNM-ECA500
Field_strength	= 11.7473579[T] (500[MH
X_acq_duration	= 0.83361792[s]
X_domain	= 13C
X_freq	= 125.76529768[MHz]
X_offset	= 100[ppm]
X_points	= 32768
X_prescans	= 4
X_resolution	= 1.19959034[Hz]
X_sweep	= 39.3081761[kHz]
Irr_domain	= 1H
Irr_freq	= 500.15991521[MHz]
Irr_offset	= 5.0[ppm]
Clipped	= FALSE
Mod_return	= 1
Scans	= 400
Total_scans	= 400
X_90_width	= 13.2[us]
X_acq_time	= 0.83361792[s]
X_angle	= 30[deg]
X_atn	= 6[dB]
X_pulse	= 4.4[us]
Irr_atn_dec	= 20.7[dB]
Irr_atn_noe	= 20.7[dB]
Irr_noise	= WALTZ
Decoupling	= TRUE
Initial_wait	= 1[s]
Noe	= TRUE
Noe_time	= 2[s]
Recvr_gain	= 60
Relaxation_delay	= 2[s]
Repetition_time	= 2.83361792[s]
Temp_get	= 23.3[dC]

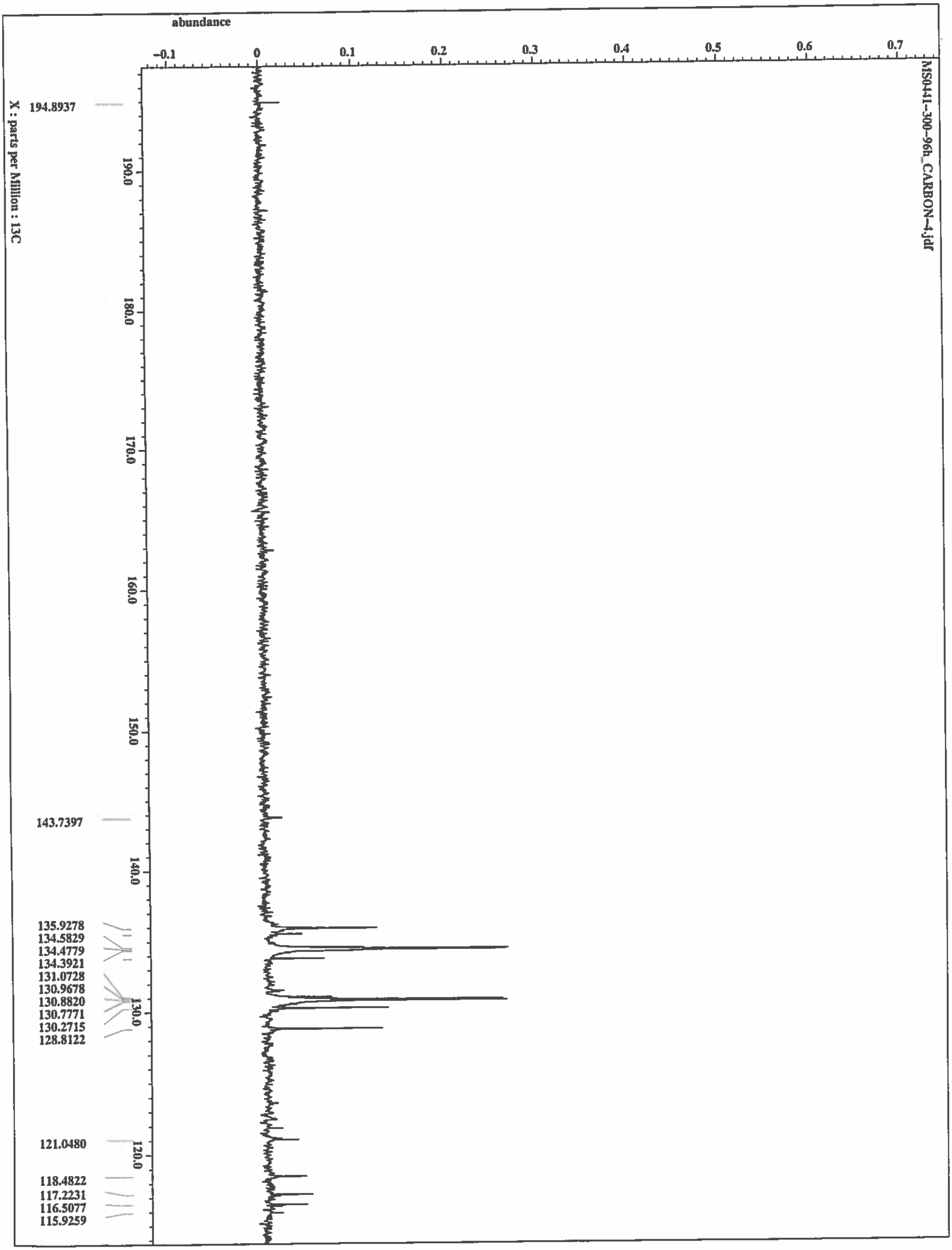

abundance
-0.1
0
0.1
0.2
0.3
0.4
0.5
0.6
0.7
MS0441-300-96h_CARBON-4.jdf
X : parts per Million : 13C
190.0
180.0
170.0
160.0
150.0
140.0
130.0
120.0
194.8937
143.7397
135.9278
134.5829
134.4779
134.3921
131.0728
130.9678
130.8820
130.7771
130.2715
128.8122
121.0480
118.4822
117.2231
116.5077
115.9259

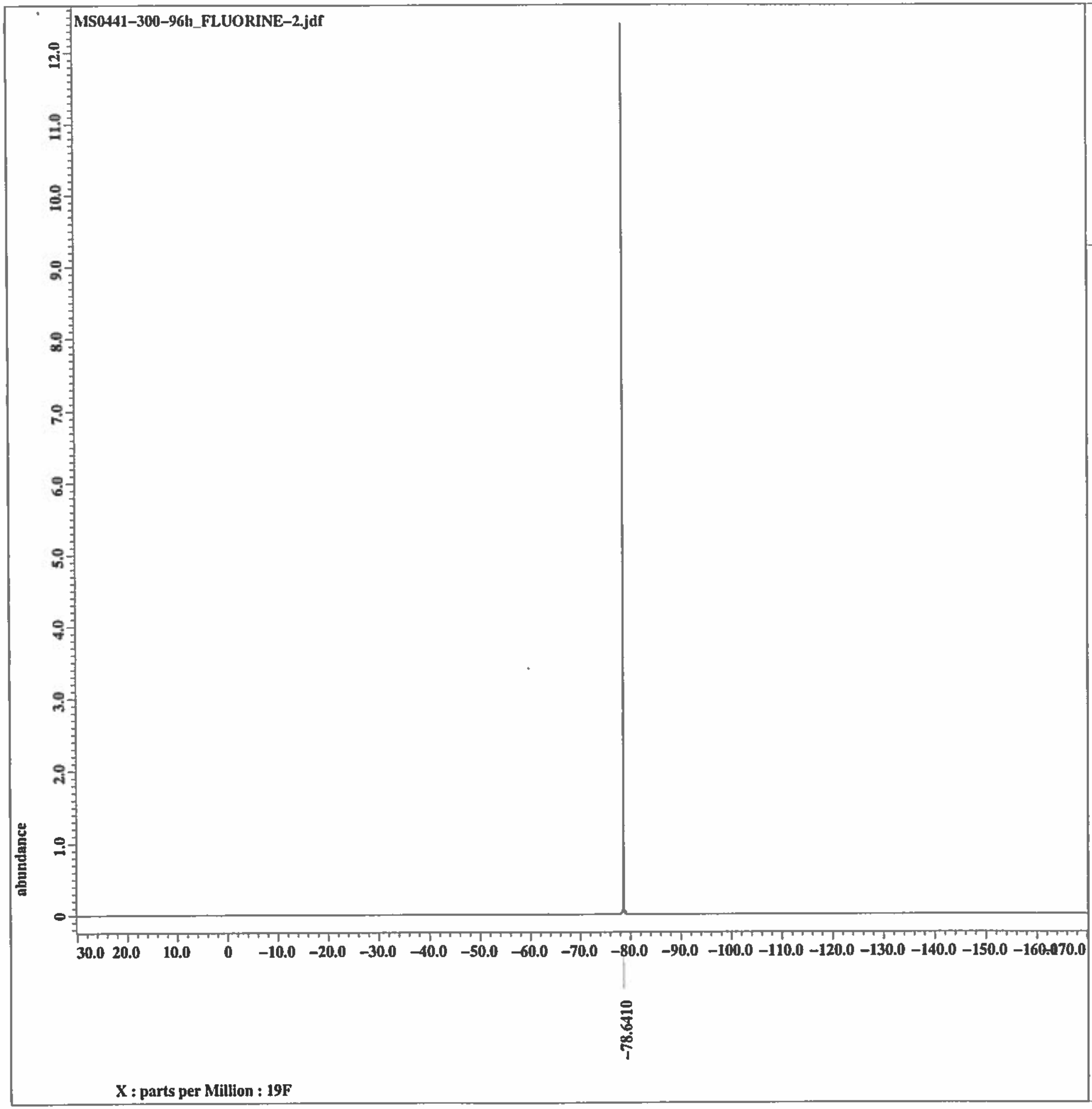

Filename	= MS0441-300-96h_FLUORI
Author	= Jim Davis
Experiment	= single_pulse.ex2
Sample_id	= MS0441-300-96h
Solvent	= CHLOROFORM-D
Changer_sample	= 4
Creation_time	= 14-MAY-2018 23:38:13
Revision_time	= 14-MAY-2018 23:13:57
Current_time	= 14-MAY-2018 23:13:57
Data_format	= 1D COMPLEX
Dim_size	= 52428
Dim_title	= 19F
Dim_units	= [ppm]
Dimensions	= X
Site	= ECA 500
Spectrometer	= JNM-ECA500
Field_strength	= 11.7473579[T] (500[MH
X_acq_duration	= 0.55574528[s]
X_domain	= 19F
X_freq	= 470.62046084[MHz]
X_offset	= -70[ppm]
X_points	= 65536
X_prescans	= 1
X_resolution	= 1.7993855[Hz]
X_sweep	= 117.9245283[kHz]
Irr_domain	= 19F
Irr_freq	= 470.62046084[MHz]
Irr_offset	= 5[ppm]
Tri_domain	= 19F
Tri_freq	= 470.62046084[MHz]
Tri_offset	= 5[ppm]
Clipped	= FALSE
Mod_return	= 1
Scans	= 16
Total_scans	= 16
X_90_width	= 13.1[us]
X_acq_time	= 0.55574528[s]
X_angle	= 45[deg]
X_atn	= 2.5[dB]
X_pulse	= 6.55[us]
Irr_mode	= Off
Tri_mode	= Off
Dante_presat	= FALSE
Initial_wait	= 1[s]
Recvr_gain	= 42
Relaxation_delay	= 4[s]
Repetition_time	= 4.55574528[s]
Temp_get	= 22.9[dC]

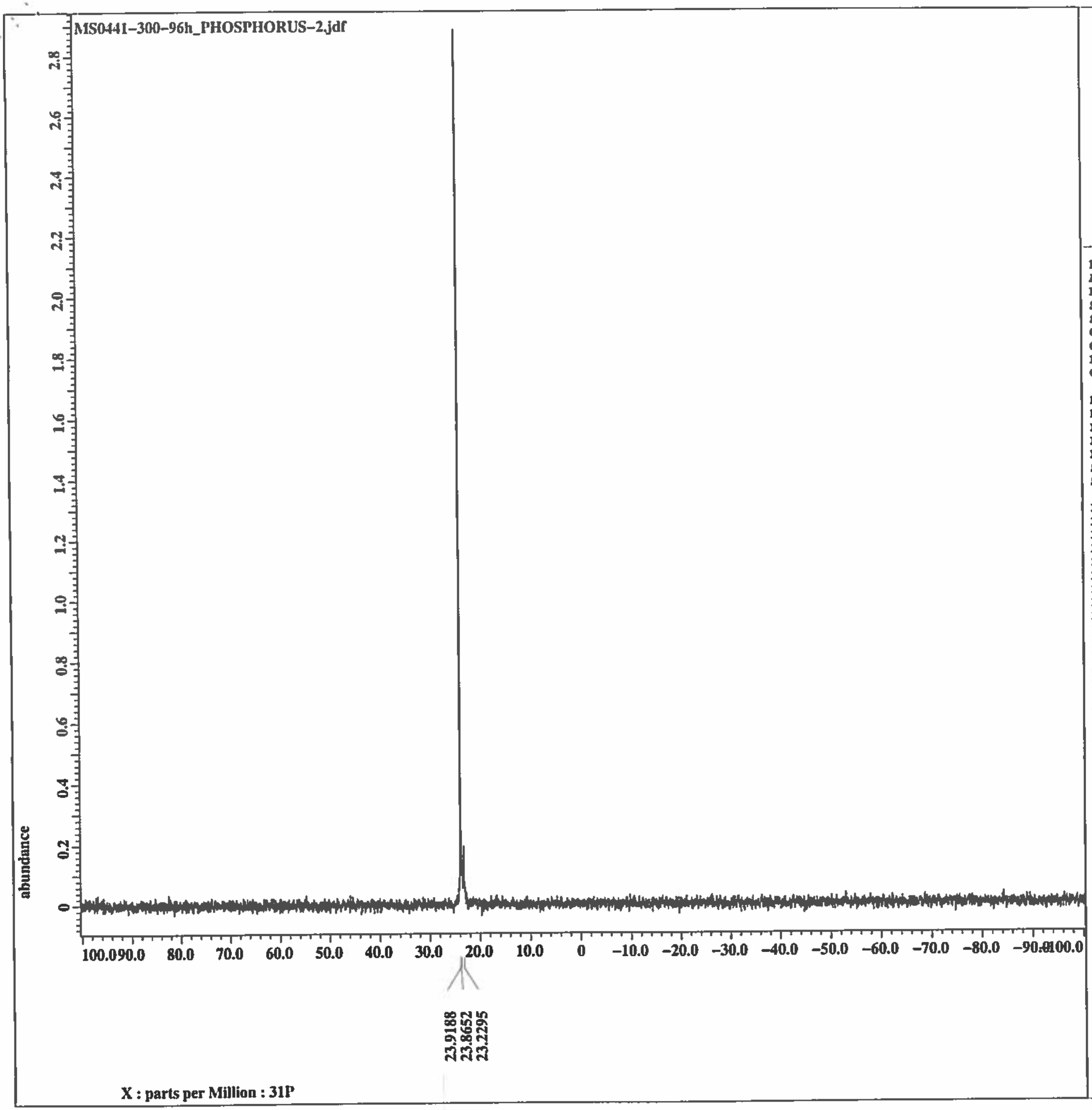

Filename	= MS0441-300-96h_PHOSPH
Author	= Jim Davis
Experiment	= single_pulse_dec
Sample_id	= MS0441-300-96h
Solvent	= CHLOROFORM-D
Changer_sample	= 4
Creation_time	= 14-MAY-2018 23:42:58
Revision_time	= 14-MAY-2018 23:18:43
Current_time	= 14-MAY-2018 23:18:43
Data_format	= 1D COMPLEX
Dim_size	= 26214
Dim_title	= 31P
Dim_units	= [ppm]
Dimensions	= X
Site	= ECA 500
Spectrometer	= JNM-ECA500
Field_strength	= 11.7473579[T] (500[MH
X_acq_duration	= 0.64487424[s]
X_domain	= 31P
X_freq	= 202.46831075[MHz]
X_offset	= 0[ppm]
X_points	= 32768
X_prescans	= 4
X_resolution	= 1.55068995[Hz]
X_sweep	= 50.81300813[kHz]
Irr_domain	= 1H
Irr_freq	= 500.15991521[MHz]
Irr_offset	= 5.0[ppm]
Clipped	= FALSE
Mod_return	= 1
Scans	= 50
Total_scans	= 50
X_90_width	= 14.687[us]
X_acq_time	= 0.64487424[s]
X_angle	= 30[deg]
X_atn	= 5[dB]
X_pulse	= 4.89566667[us]
Irr_atn_dec	= 20.7[dB]
Irr_atn_noe	= 20.7[dB]
Irr_noise	= WALTZ
Decoupling	= TRUE
Initial_wait	= 1[s]
Noe	= TRUE
Noe_time	= 2[s]
Recvr_gain	= 60
Relaxation_delay	= 2[s]
Repetition_time	= 2.64487424[s]
Temp_get	= 22.9[dC]

Compound 12 Pre- and Post-heating NMR Spectra

Temperature of Post-heating samples noted in upper left corner of each spectrum

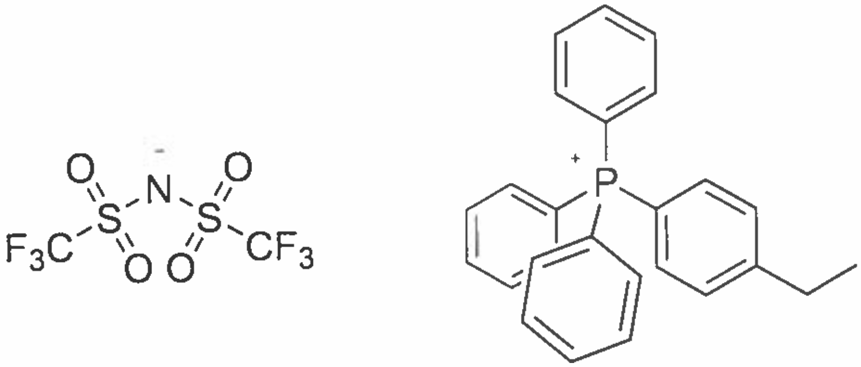

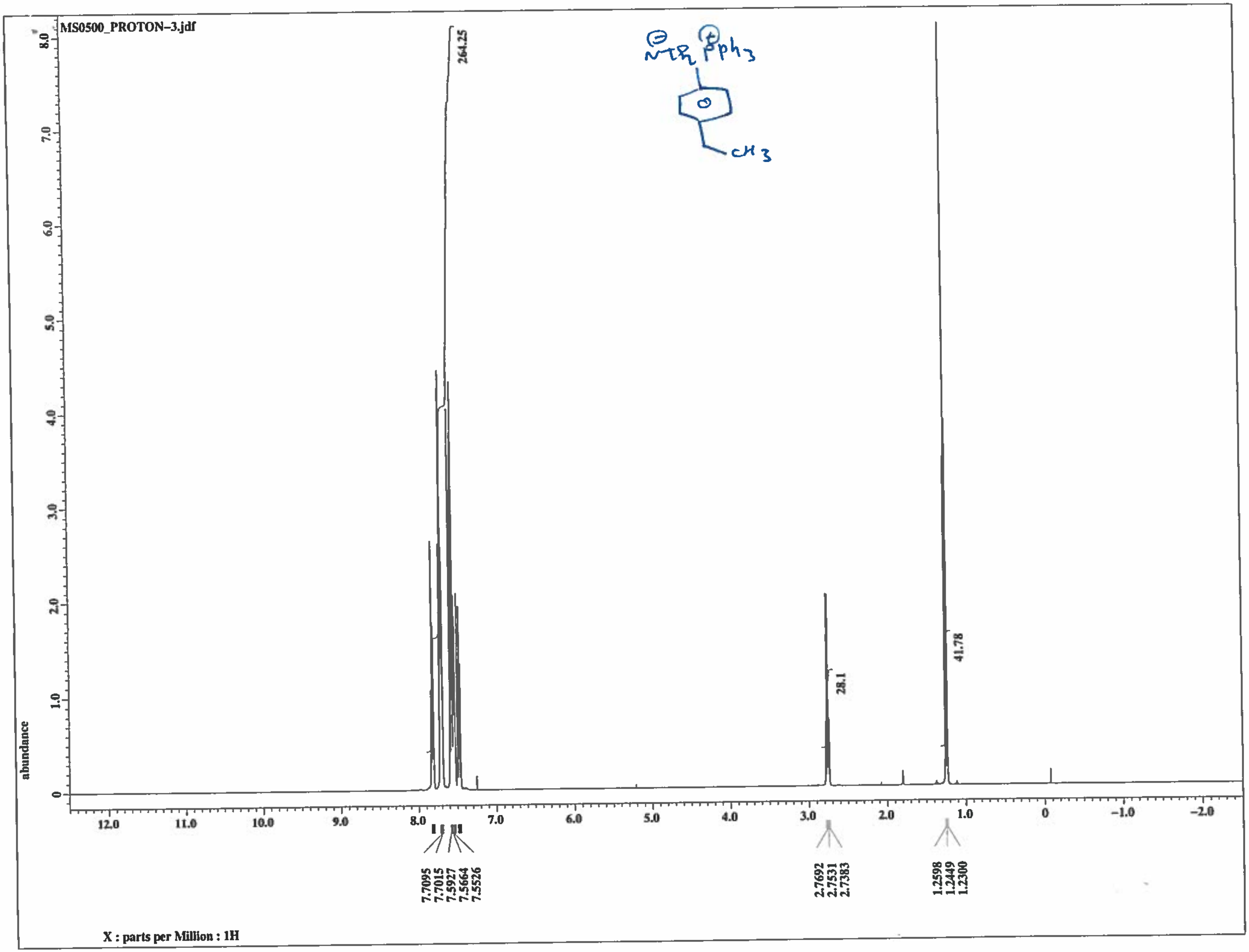
MS0500_PROTON-3.jdf
abundance
264.25
28.1
41.78
7.7095
7.7015
7.5927
7.5664
7.5526
2.7692
2.7531
2.7383
1.2598
1.2449
1.2300
X : parts per Million : 1H

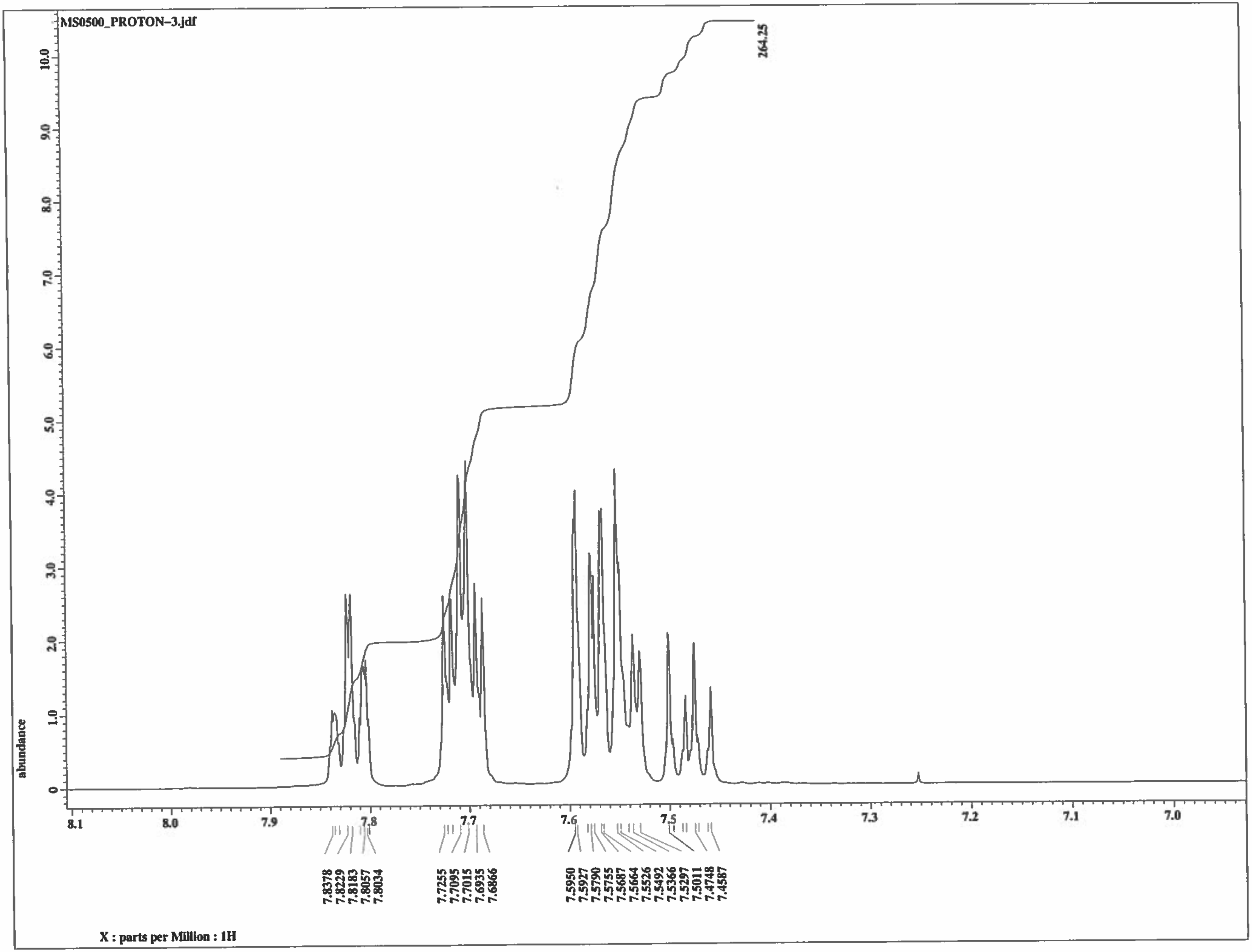
MS0500_PROTON-3.jdf
abundance
0
1.0
2.0
3.0
4.0
5.0
6.0
7.0
8.0
9.0
10.0
264.25
8.1
8.0
7.9
7.8
7.7
7.6
7.5
7.4
7.3
7.2
7.1
7.0
7.8378
7.8229
7.8183
7.8057
7.8034
7.7255
7.7095
7.7015
7.6935
7.6866
7.5950
7.5927
7.5790
7.5755
7.5687
7.5664
7.5526
7.5492
7.5366
7.5297
7.5011
7.4748
7.4587
X : parts per Million : 1H

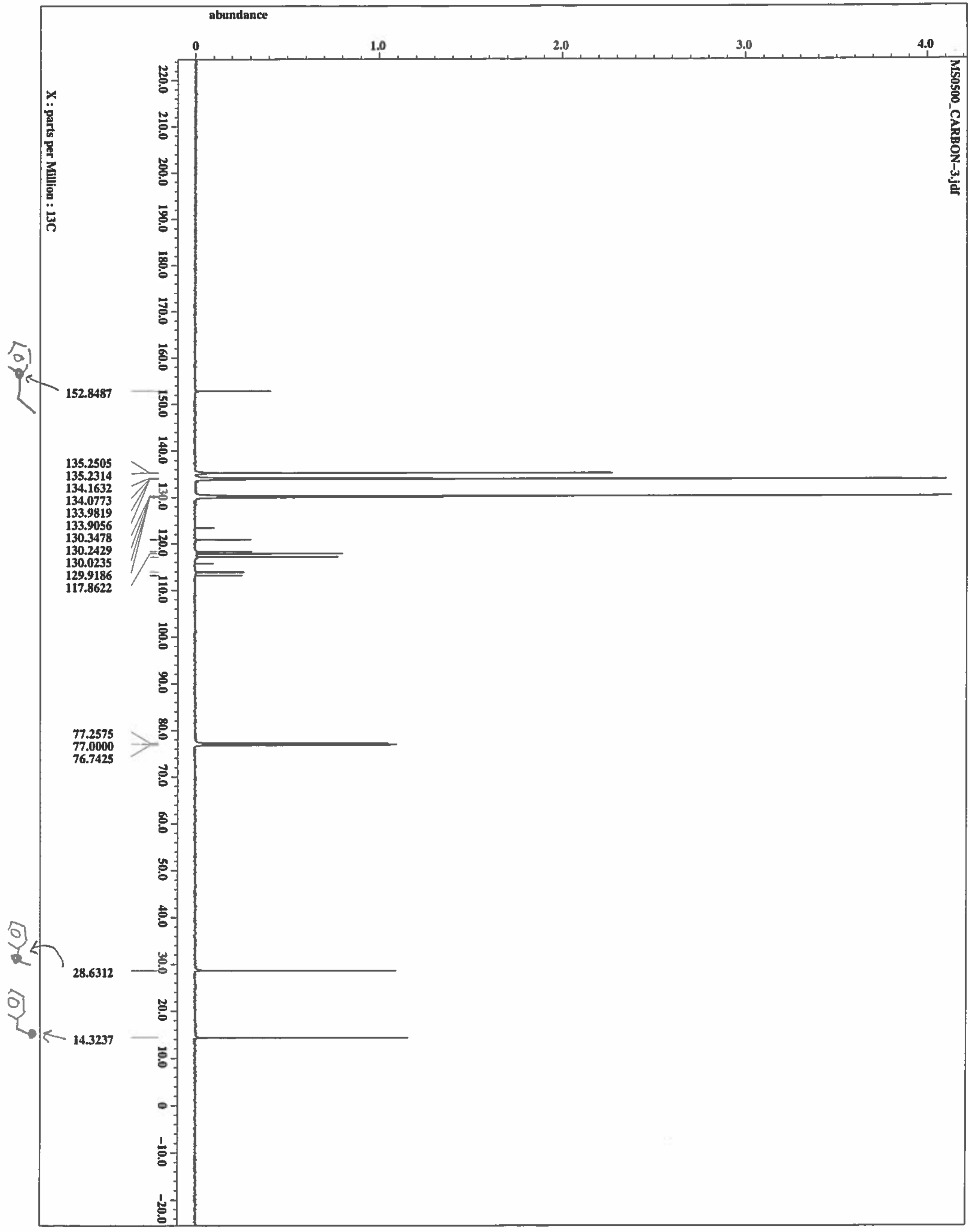
abundance
0
1.0
2.0
3.0
4.0
MS0500_CARBON-3.jdf
X : parts per Million : 13C
220.0
210.0
200.0
190.0
180.0
170.0
160.0
150.0
140.0
130.0
120.0
110.0
100.0
90.0
80.0
70.0
60.0
50.0
40.0
30.0
20.0
10.0
0
-10.0
-20.0
152.8487
135.2505
135.2314
134.1632
134.0773
133.9819
133.9056
130.3478
130.2429
130.0235
129.9186
117.8622
77.2575
77.0000
76.7425
28.6312
14.3237

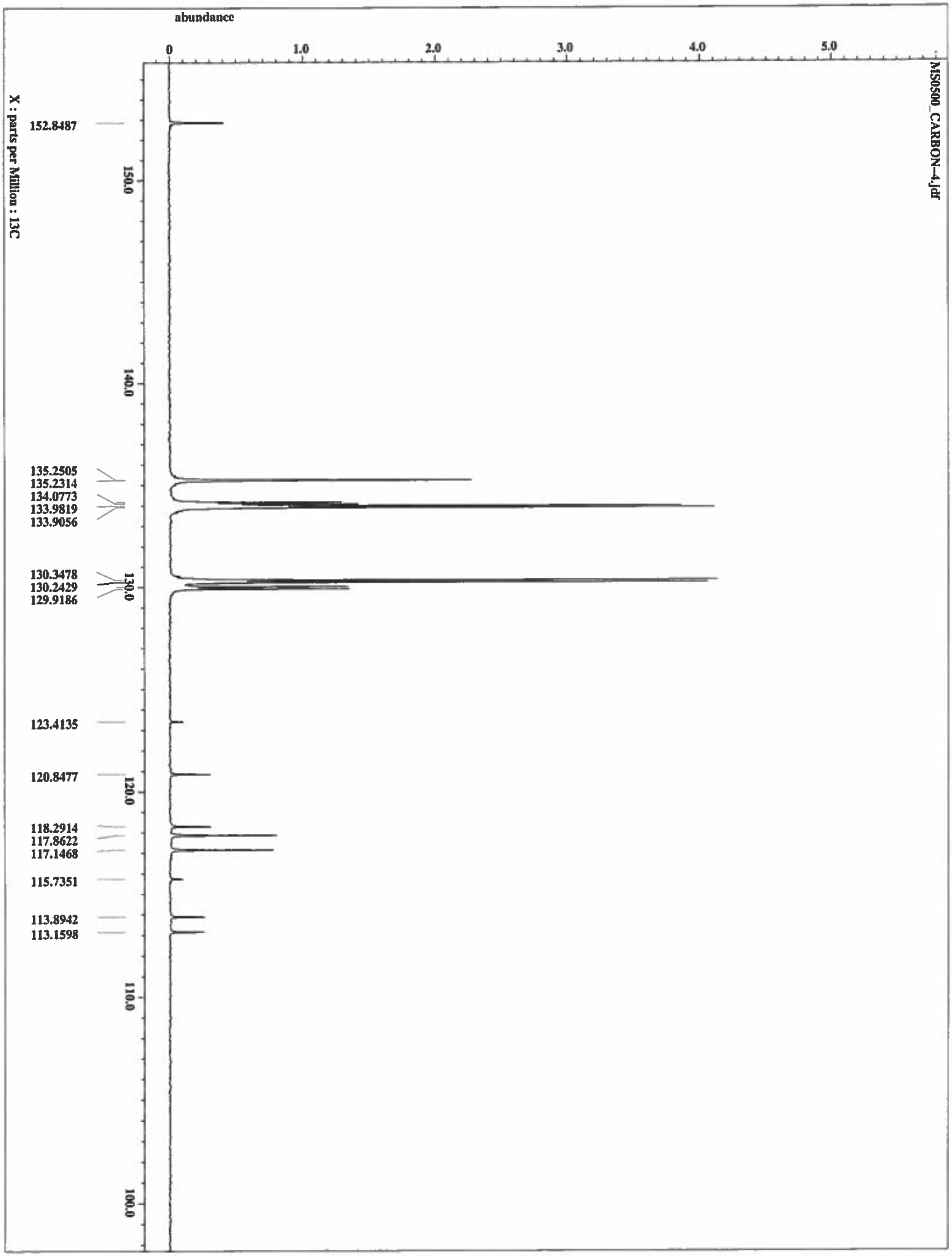
abundance
0
1.0
2.0
3.0
4.0
5.0
MS0500_CARBON-4.jdf
X : parts per Million : 13C
152.8487
150.0
140.0
135.2505
135.2314
134.0773
133.9819
133.9056
130.3478
130.2429
129.9186
130.0
123.4135
120.8477
120.0
118.2914
117.8622
117.1468
115.7351
113.8942
113.1598
110.0
100.0

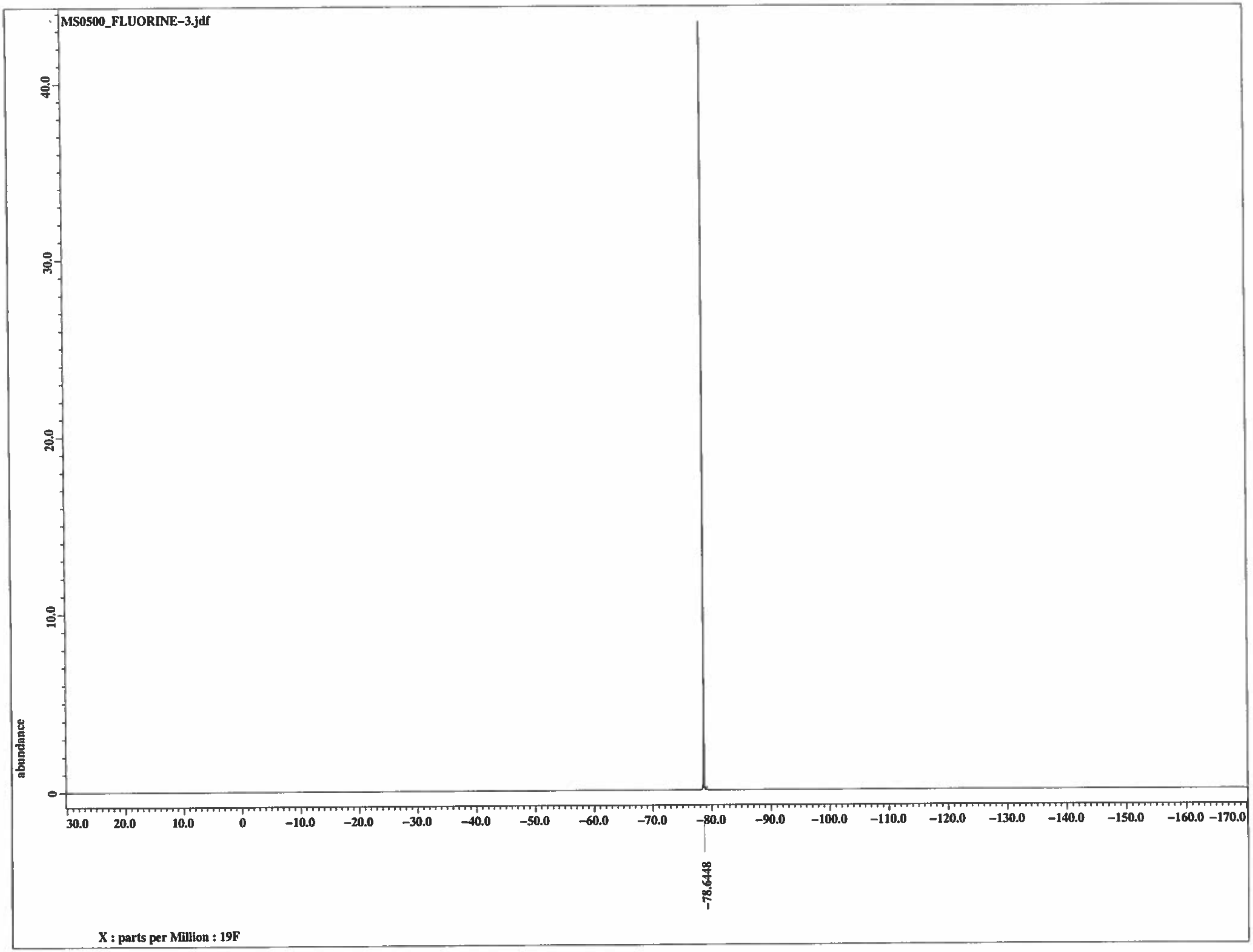
MS0500_FLUORINE-3.jdf
abundance
40.0
30.0
20.0
10.0
0
30.0 20.0 10.0 0 −10.0 −20.0 −30.0 −40.0 −50.0 −60.0 −70.0 −80.0 −90.0 −100.0 −110.0 −120.0 −130.0 −140.0 −150.0 −160.0 −170.0
−78.6448
X : parts per Million : 19F

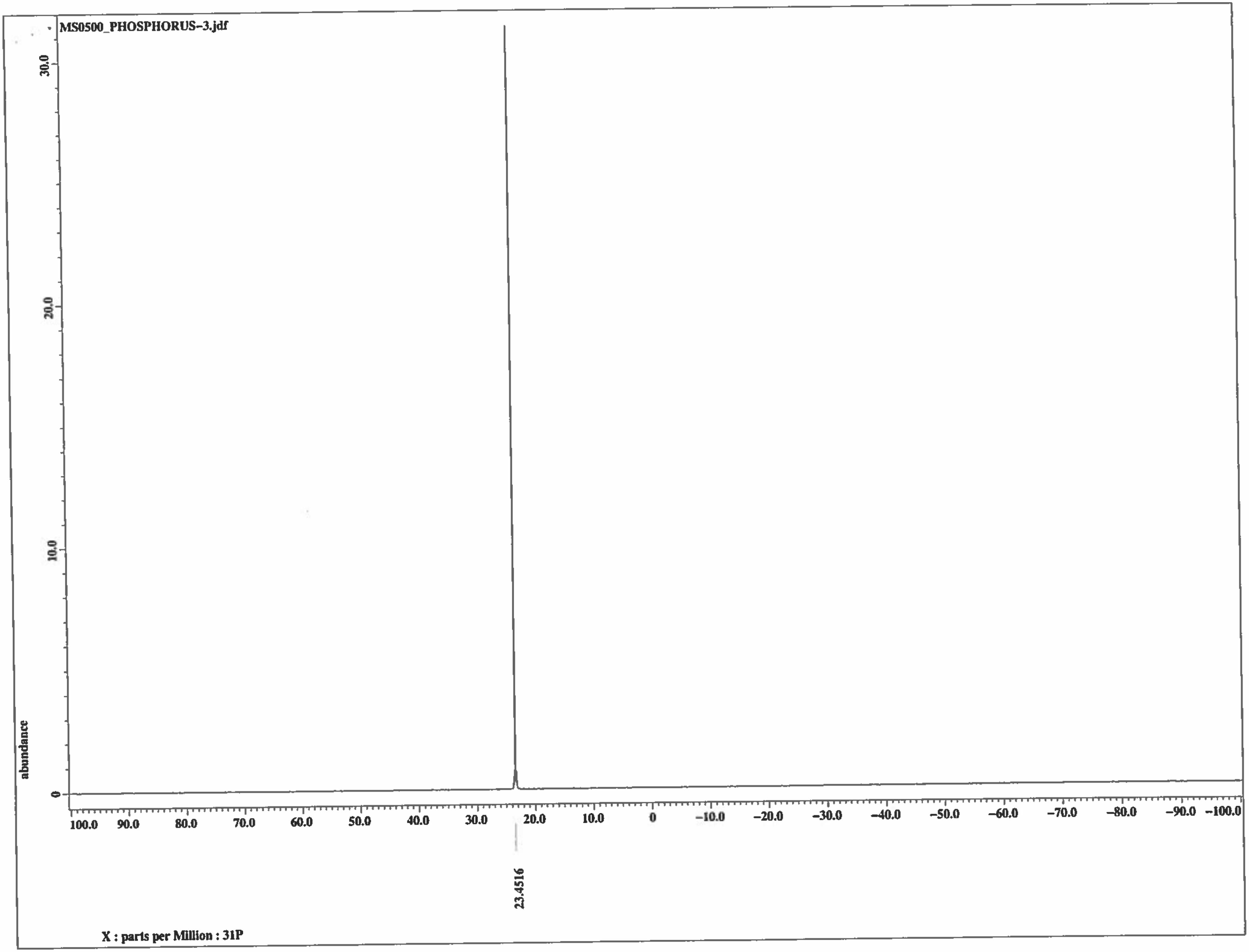
MS0500_PHOSPHORUS-3.jdf
abundance
30.0
20.0
10.0
0
100.0 90.0 80.0 70.0 60.0 50.0 40.0 30.0 20.0 10.0 0 −10.0 −20.0 −30.0 −40.0 −50.0 −60.0 −70.0 −80.0 −90.0 −100.0
23.4516
X : parts per Million : 31P

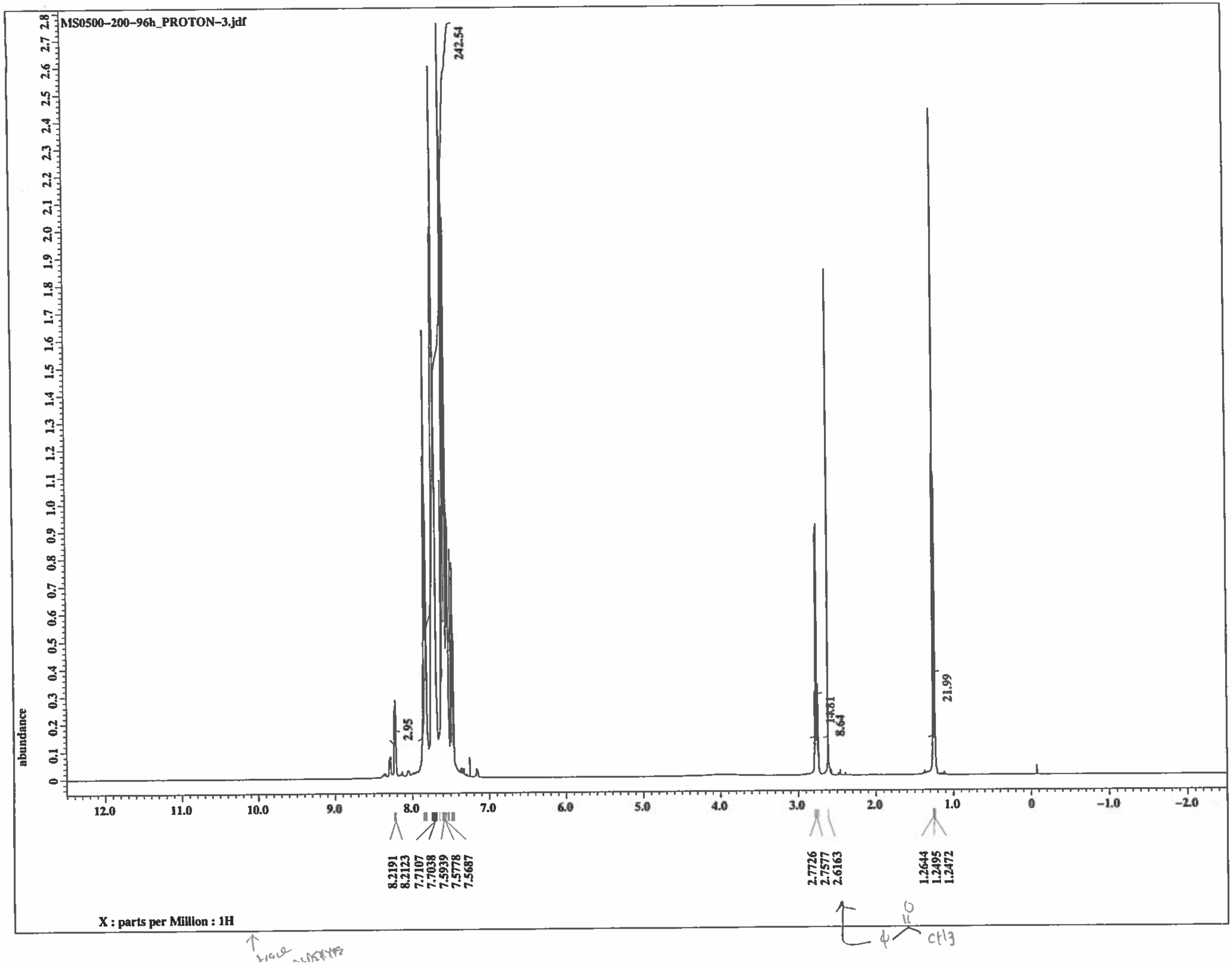

MS0500-200-96h_PROTON-3.jdf
abundance
0 0.1 0.2 0.3 0.4 0.5 0.6 0.7 0.8 0.9 1.0 1.1 1.2 1.3 1.4 1.5 1.6 1.7 1.8 1.9 2.0 2.1 2.2 2.3 2.4 2.5 2.6 2.7 2.8
242.54
2.95
14.81
8.64
21.99
12.0 11.0 10.0 9.0 8.0 7.0 6.0 5.0 4.0 3.0 2.0 1.0 0 -1.0 -2.0
8.2191
8.2123
7.7107
7.7038
7.5939
7.5778
7.5687
2.7726
2.7577
2.6163
1.2644
1.2495
1.2472
X : parts per Million : 1H

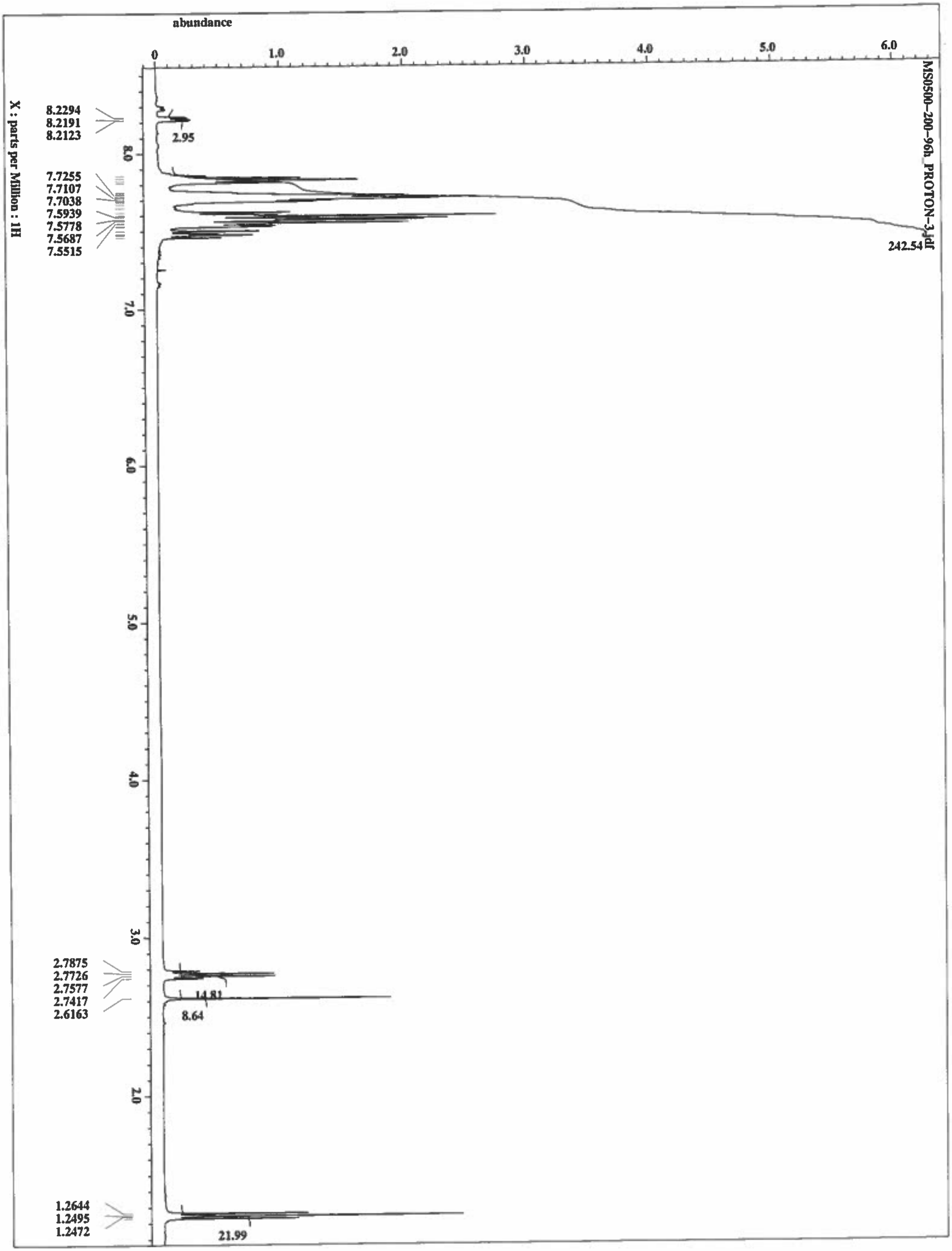

abundance
0
1.0
2.0
3.0
4.0
5.0
6.0
MS0500-200-96h_PROTON-3.jdf
X : parts per Million : 1H
8.2294
8.2191
8.2123
2.95
8.0
7.7255
7.7107
7.7038
7.5939
7.5778
7.5687
7.5515
242.54
7.0
6.0
5.0
4.0
3.0
2.7875
2.7726
2.7577
2.7417
2.6163
14.81
8.64
2.0
1.2644
1.2495
1.2472
21.99

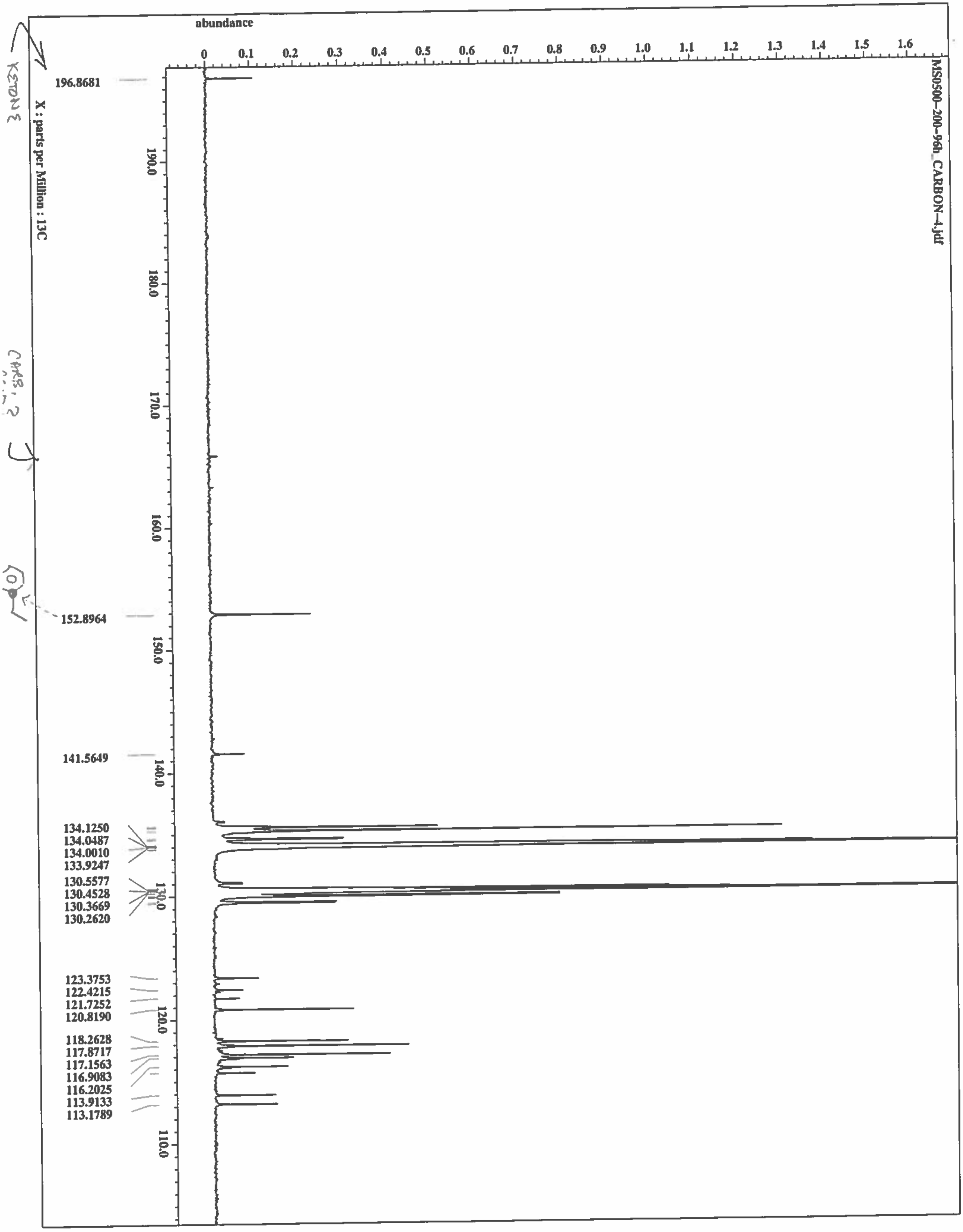

abundance
0 0.1 0.2 0.3 0.4 0.5 0.6 0.7 0.8 0.9 1.0 1.1 1.2 1.3 1.4 1.5 1.6
MS0500-200-96h_CARBON-4.jdf
X : parts per Million : 13C
190.0
180.0
170.0
160.0
150.0
140.0
130.0
120.0
110.0
196.8681
152.8964
141.5649
134.1250
134.0487
134.0010
133.9247
130.5577
130.4528
130.3669
130.2620
123.3753
122.4215
121.7252
120.8190
118.2628
117.8717
117.1563
116.9083
116.2025
113.9133
113.1789

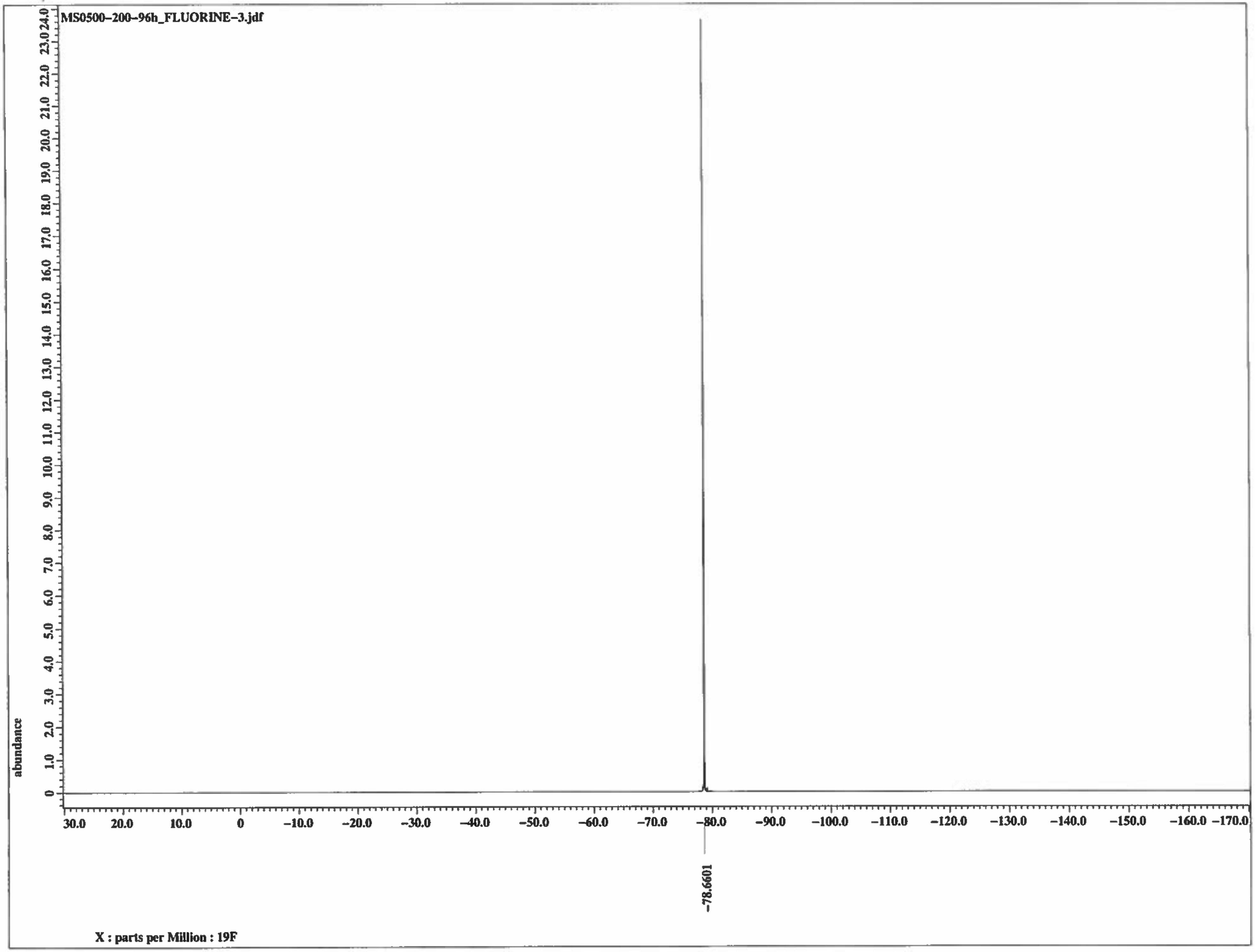
MS0500-200-96h_FLUORINE-3.jdf
abundance
-78.6601
X : parts per Million : 19F

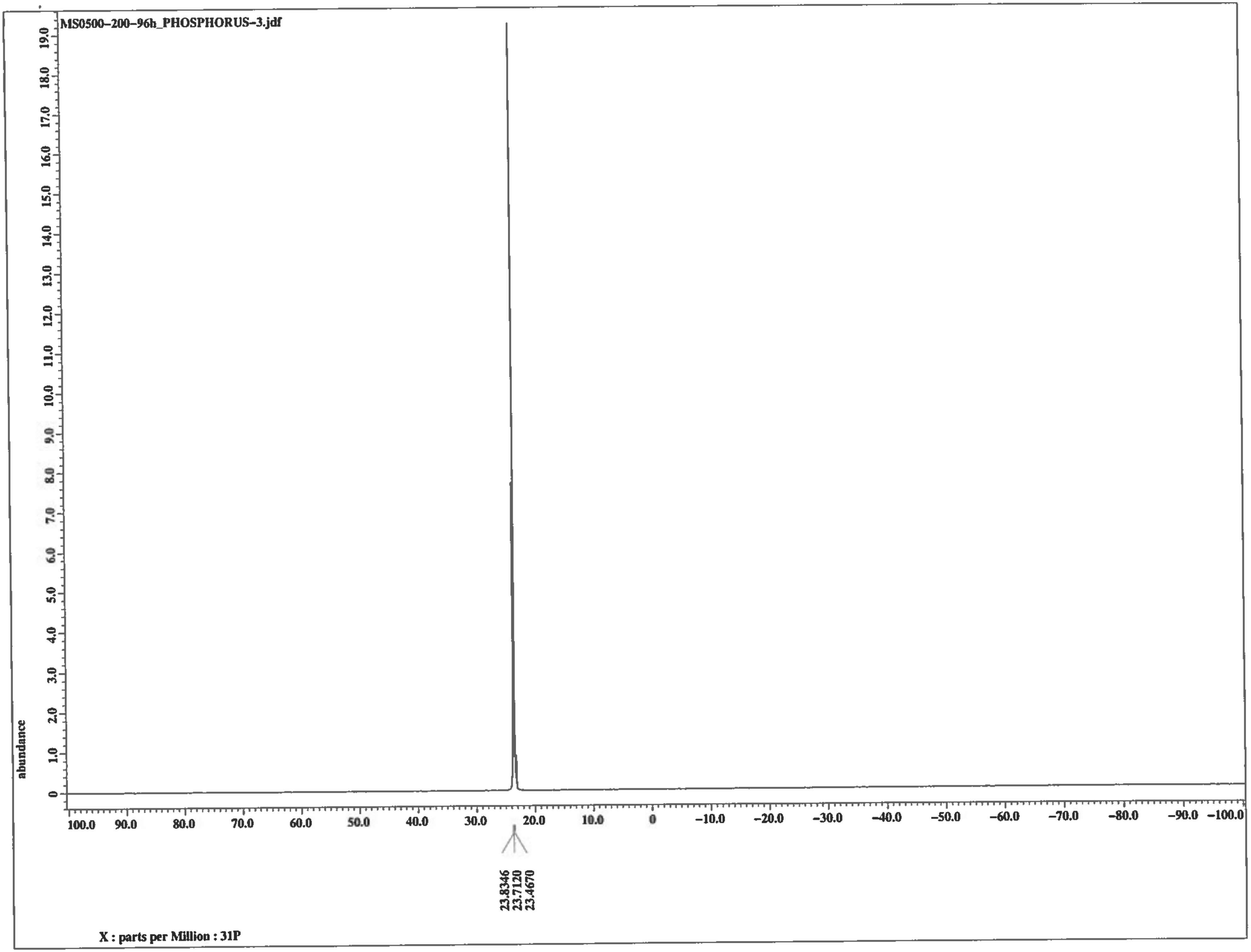
MS0500-200-96h_PHOSPHORUS-3.jdf
abundance
19.0
18.0
17.0
16.0
15.0
14.0
13.0
12.0
11.0
10.0
9.0
8.0
7.0
6.0
5.0
4.0
3.0
2.0
1.0
0
100.0
90.0
80.0
70.0
60.0
50.0
40.0
30.0
20.0
10.0
0
−10.0
−20.0
−30.0
−40.0
−50.0
−60.0
−70.0
−80.0
−90.0
−100.0
23.8346
23.7120
23.4670
X : parts per Million : 31P

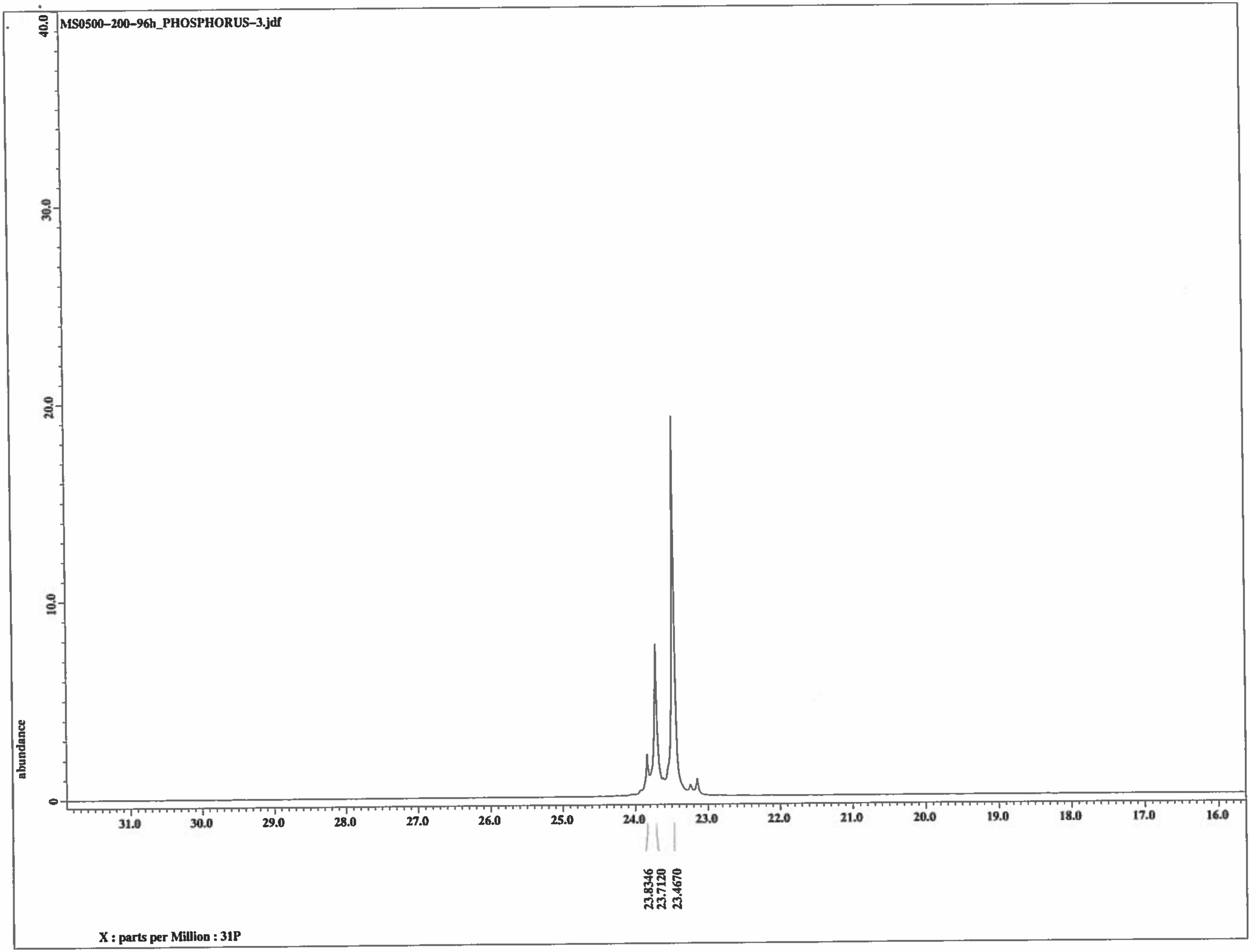
MS0500-200-96h_PHOSPHORUS-3.jdf
abundance
0
10.0
20.0
30.0
40.0
31.0
30.0
29.0
28.0
27.0
26.0
25.0
24.0
23.0
22.0
21.0
20.0
19.0
18.0
17.0
16.0
23.8346
23.7120
23.4670
X : parts per Million : 31P

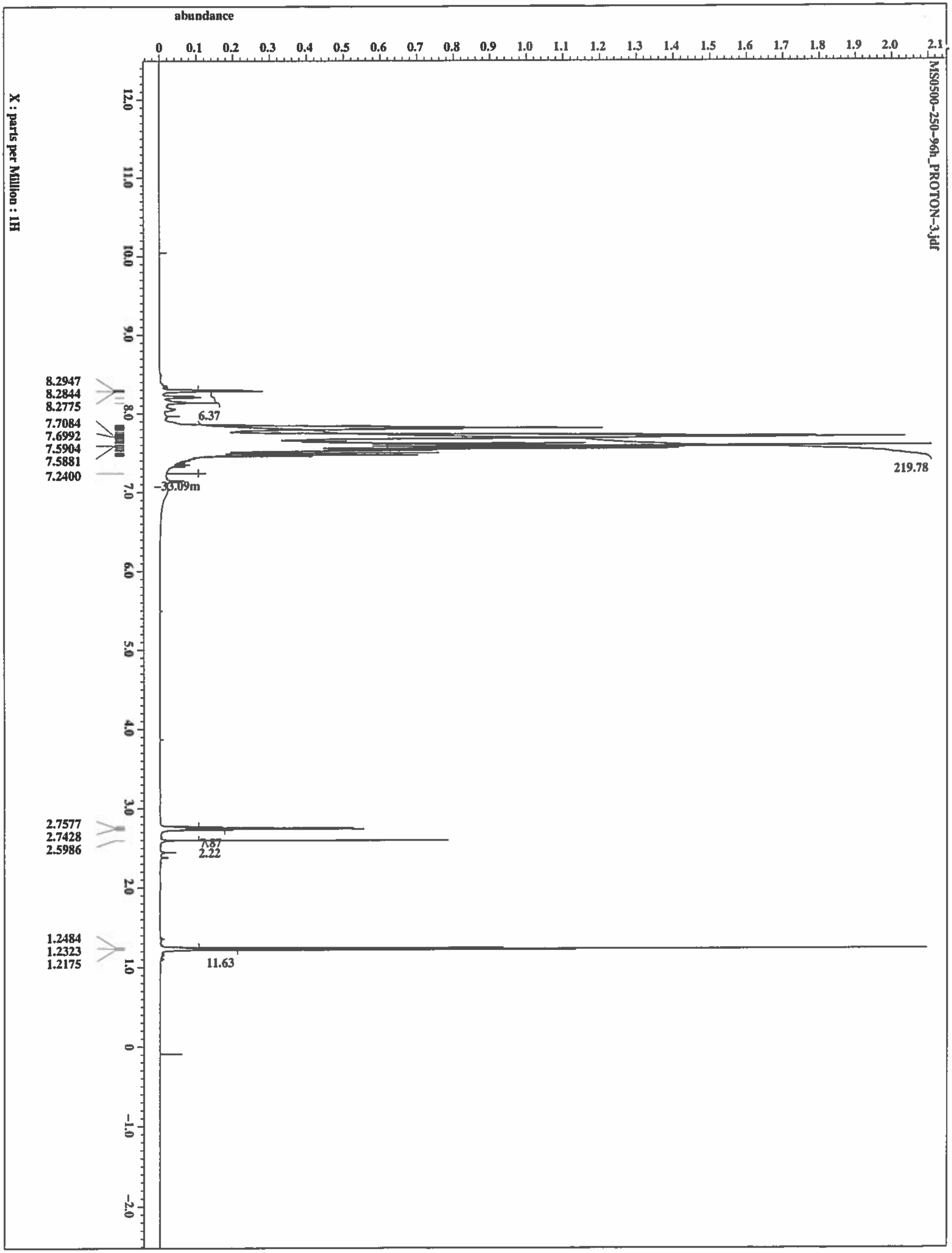
abundance
X : parts per Million : 1H
MS0500-250-96h_PROTON-3.jdf
8.2947
8.2844
8.2775
7.7084
7.6992
7.5904
7.5881
7.2400
2.7577
2.7428
2.5986
1.2484
1.2323
1.2175
6.37
219.78
-33.09m
7.87
2.22
11.63

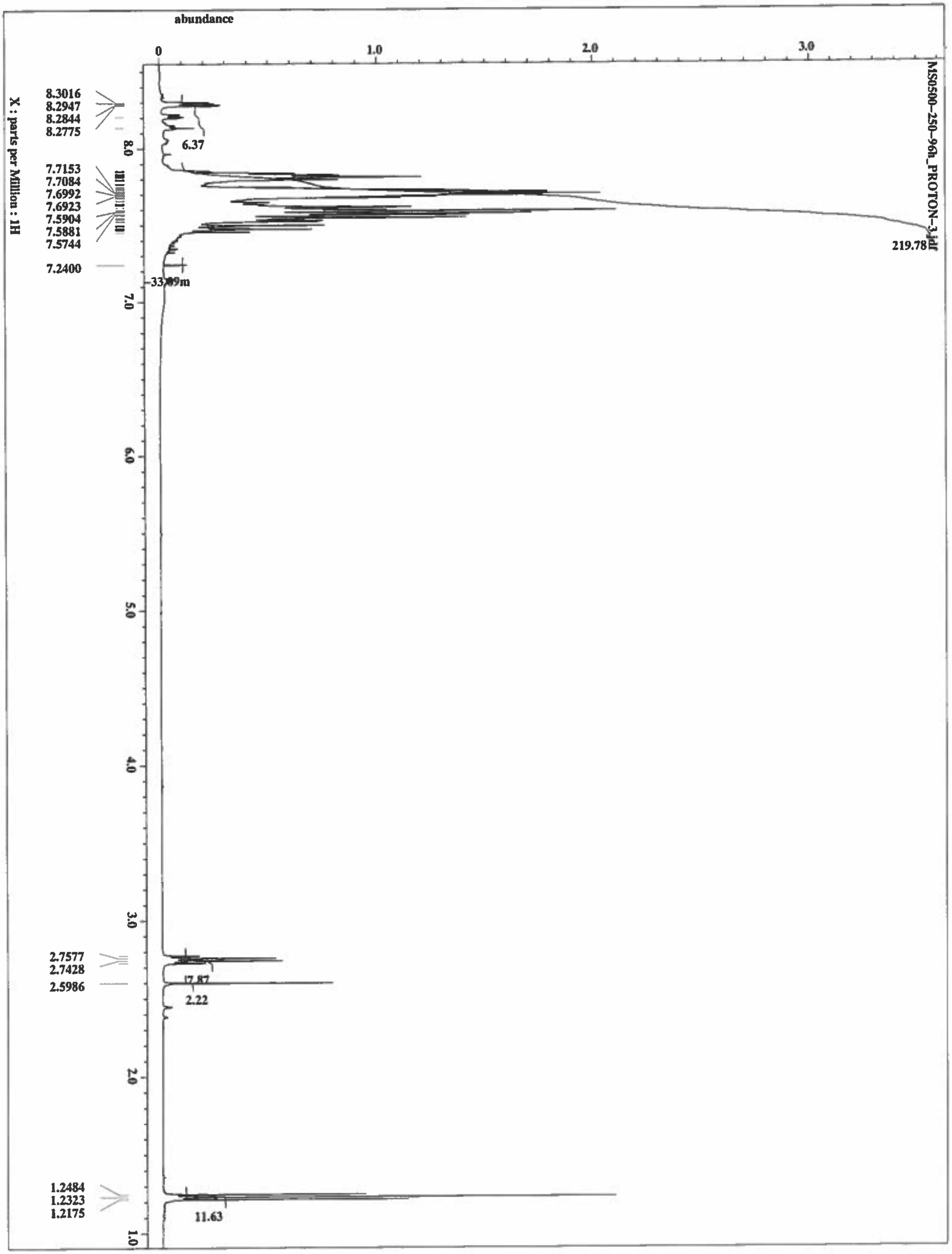

abundance
0
1.0
2.0
3.0
MS0500-250-96h_PROTON-3.jdf
X : parts per Million : 1H
8.3016
8.2947
8.2844
8.2775
8.0
6.37
7.7153
7.7084
7.6992
7.6923
7.5904
7.5881
7.5744
219.78
7.2400
-33.09m
7.0
6.0
5.0
4.0
3.0
2.7577
2.7428
17.87
2.5986
2.22
2.0
1.2484
1.2323
1.2175
11.63
1.0

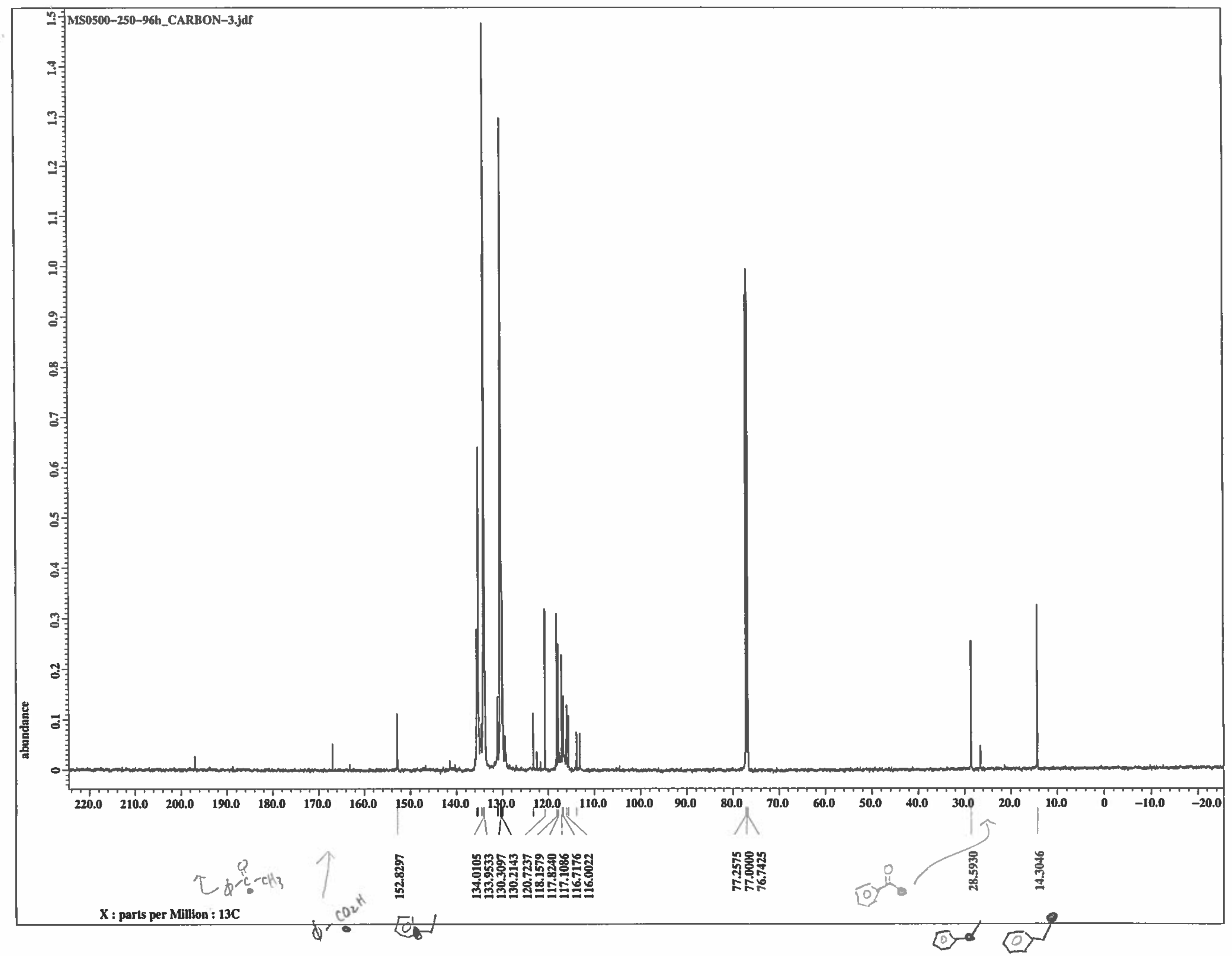

MS0500-250-96h_CARBON-3.jdf
abundance
X : parts per Million : 13C
152.8297
134.0105
133.9533
130.3097
130.2143
120.7237
118.1579
117.8240
117.1086
116.7176
116.0022
77.2575
77.0000
76.7425
28.5930
14.3046

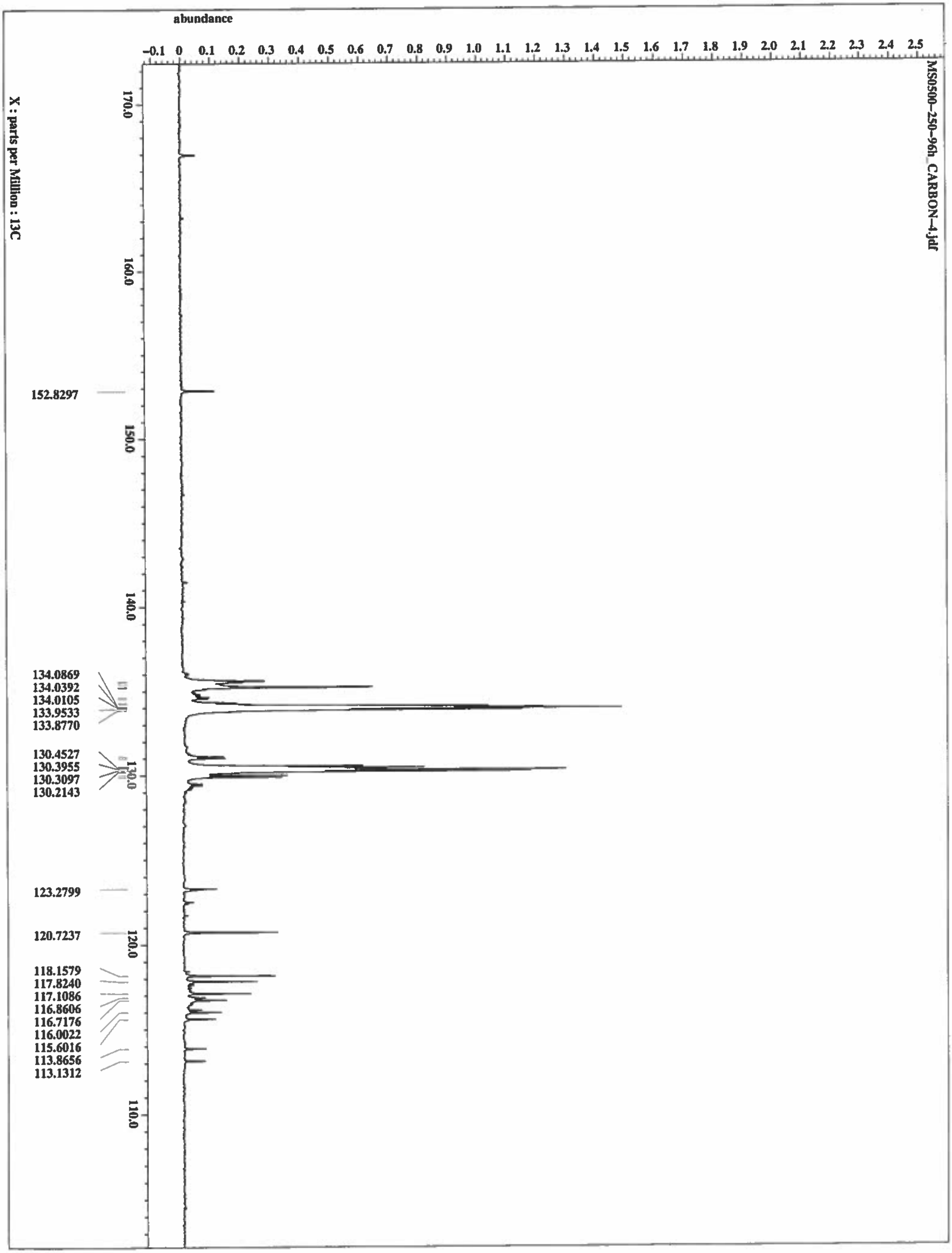

abundance
-0.1 0 0.1 0.2 0.3 0.4 0.5 0.6 0.7 0.8 0.9 1.0 1.1 1.2 1.3 1.4 1.5 1.6 1.7 1.8 1.9 2.0 2.1 2.2 2.3 2.4 2.5
MS0500-250-96h_CARBON-4.jdf
X : parts per Million : 13C
170.0
160.0
150.0
140.0
130.0
120.0
110.0
152.8297
134.0869
134.0392
134.0105
133.9533
133.8770
130.4527
130.3955
130.3097
130.2143
123.2799
120.7237
118.1579
117.8240
117.1086
116.8606
116.7176
116.0022
115.6016
113.8656
113.1312

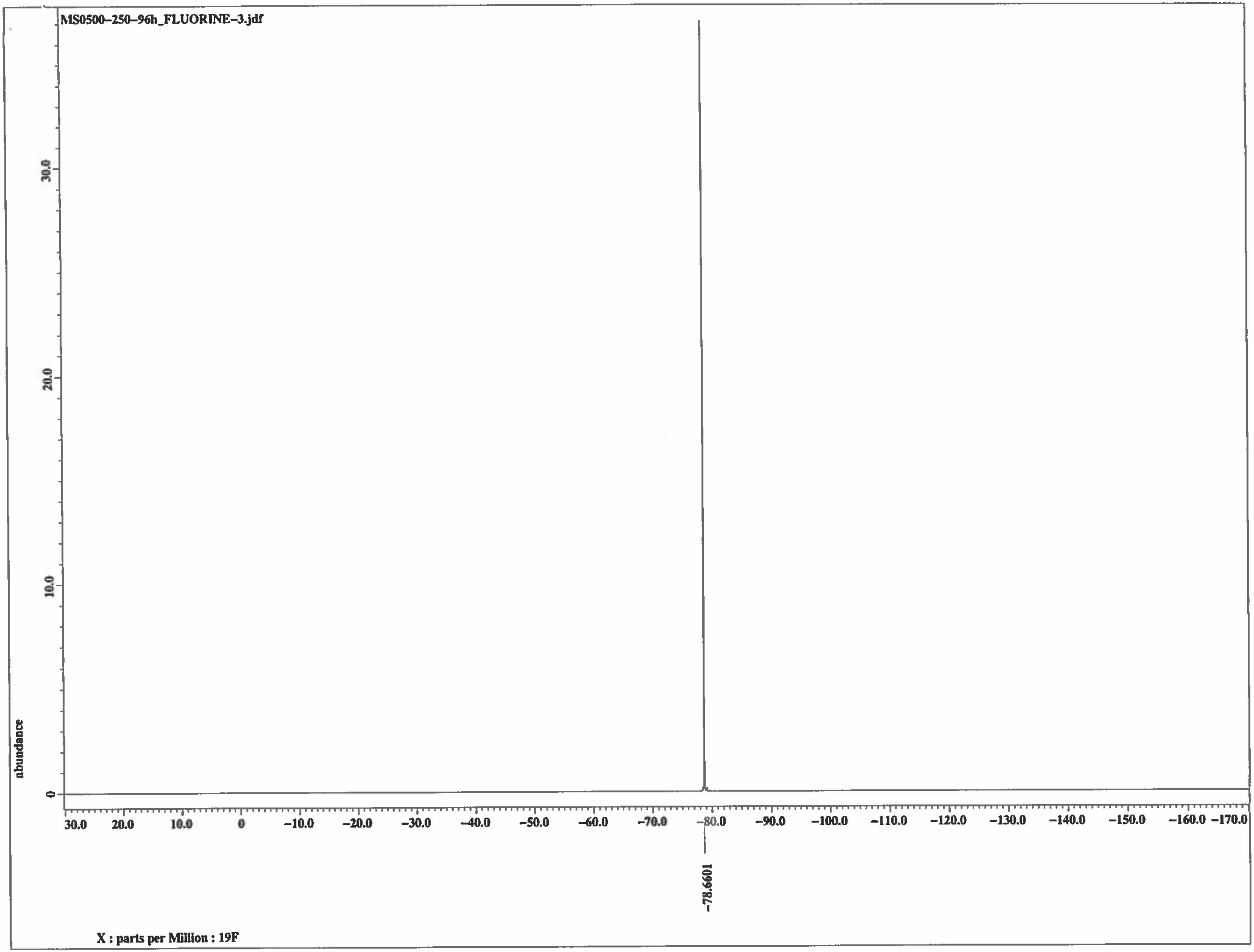
MS0500-250-96h_FLUORINE-3.jdf
abundance
30.0
20.0
10.0
0
30.0 20.0 10.0 0 −10.0 −20.0 −30.0 −40.0 −50.0 −60.0 −70.0 −80.0 −90.0 −100.0 −110.0 −120.0 −130.0 −140.0 −150.0 −160.0 −170.0
−78.6601
X : parts per Million : 19F

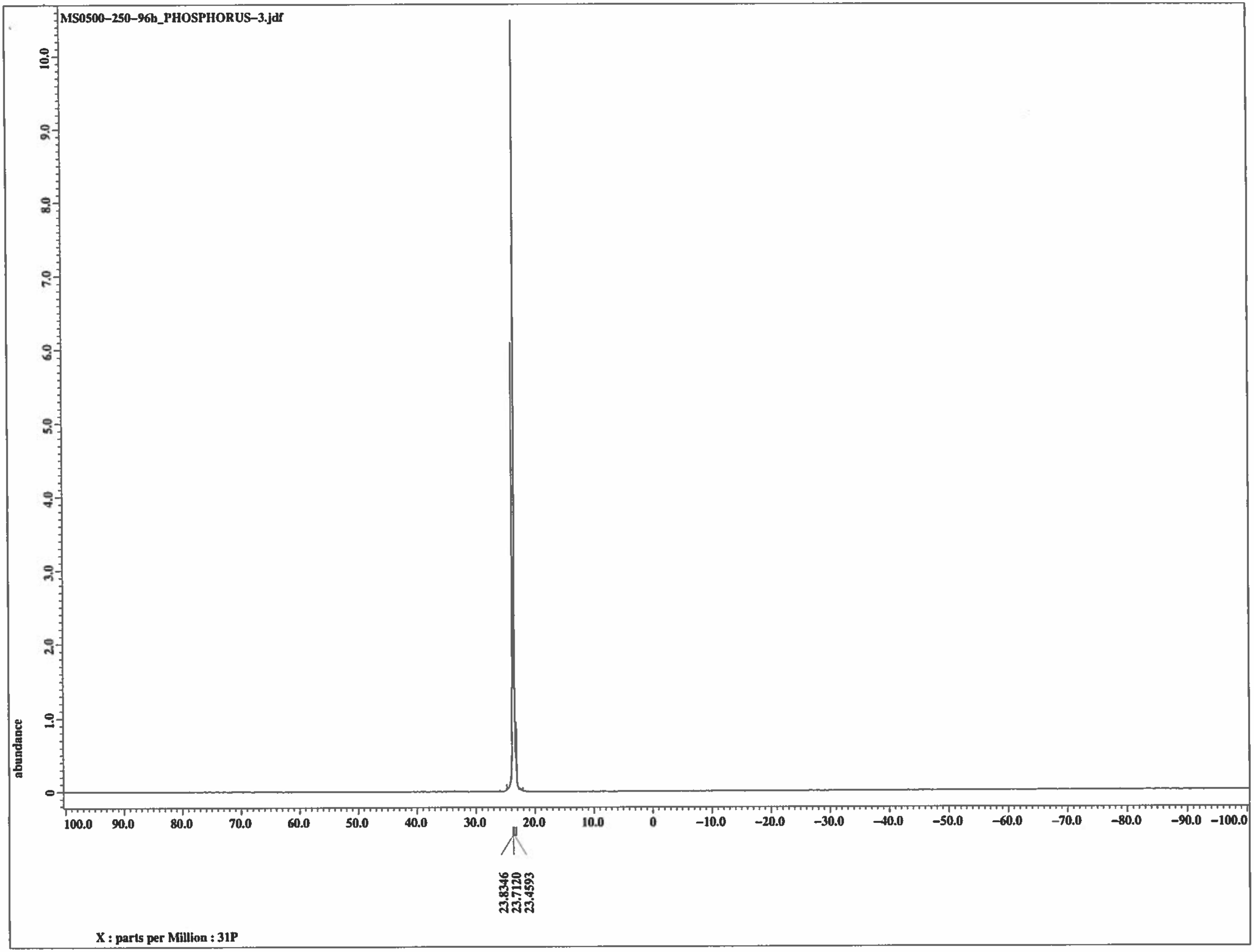
MS0500-250-96b_PHOSPHORUS-3.jdf
abundance
23.8346
23.7120
23.4593
X : parts per Million : 31P

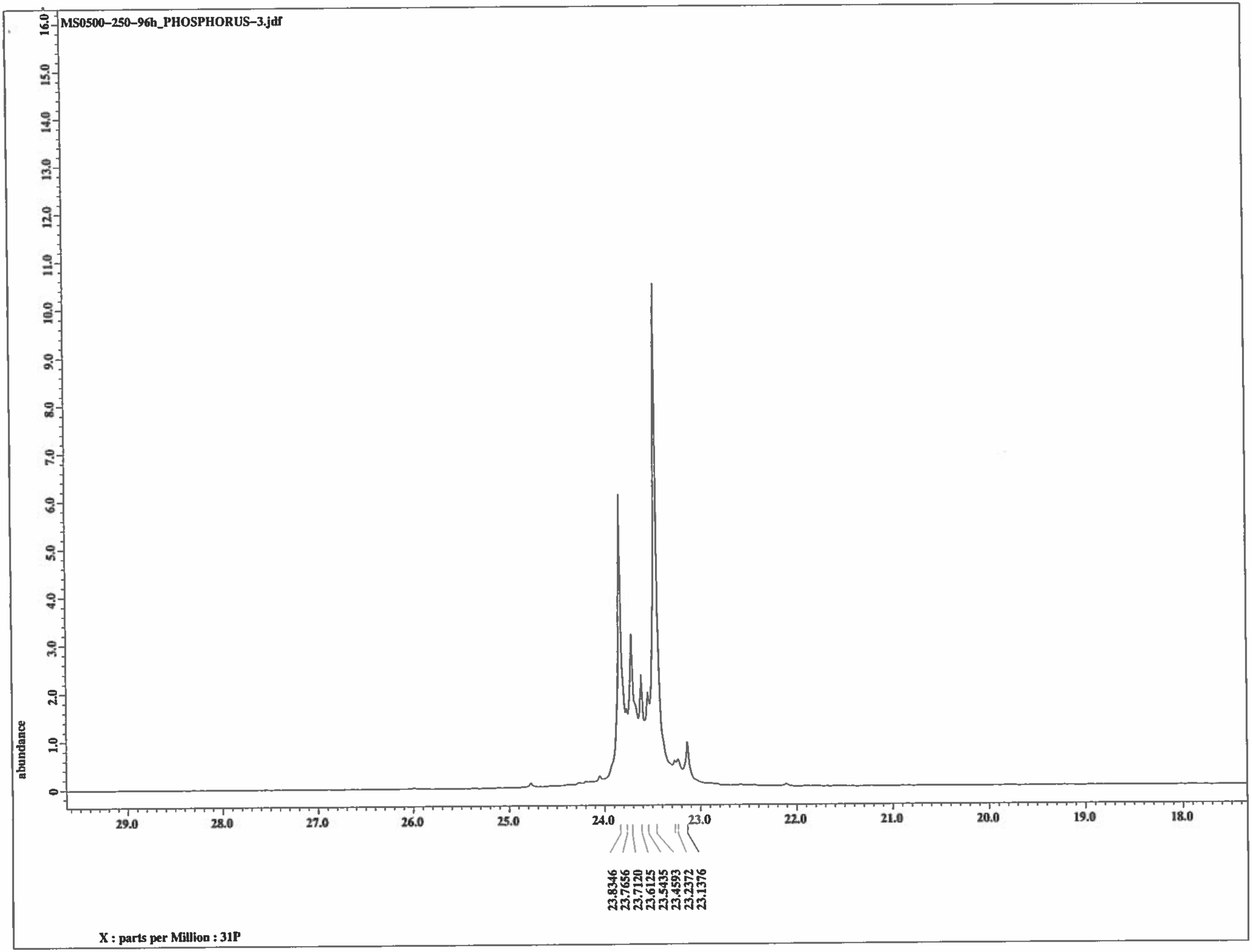
MS0500-250-96h_PHOSPHORUS-3.jdf
abundance
0
1.0
2.0
3.0
4.0
5.0
6.0
7.0
8.0
9.0
10.0
11.0
12.0
13.0
14.0
15.0
16.0
29.0
28.0
27.0
26.0
25.0
24.0
23.0
22.0
21.0
20.0
19.0
18.0
23.8346
23.7656
23.7120
23.6125
23.5435
23.4593
23.2372
23.1376
X : parts per Million : 31P

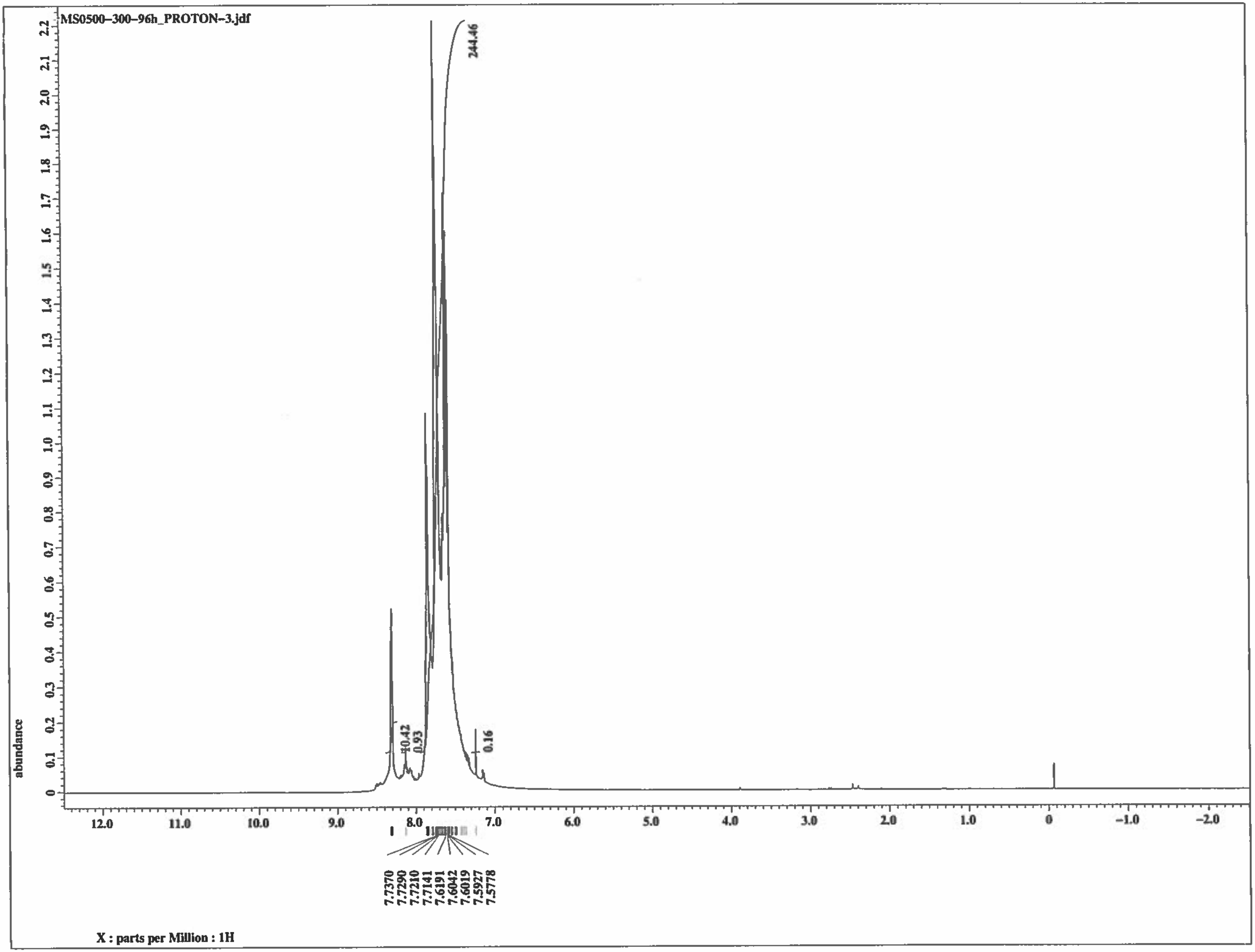
MS0500-300-96h_PROTON-3.jdf
abundance
0
0.1
0.2
0.3
0.4
0.5
0.6
0.7
0.8
0.9
1.0
1.1
1.2
1.3
1.4
1.5
1.6
1.7
1.8
1.9
2.0
2.1
2.2
244.46
10.42
0.93
0.16
12.0
11.0
10.0
9.0
8.0
7.0
6.0
5.0
4.0
3.0
2.0
1.0
0
-1.0
-2.0
7.7370
7.7290
7.7210
7.7141
7.6191
7.6042
7.6019
7.5927
7.5778
X : parts per Million : 1H

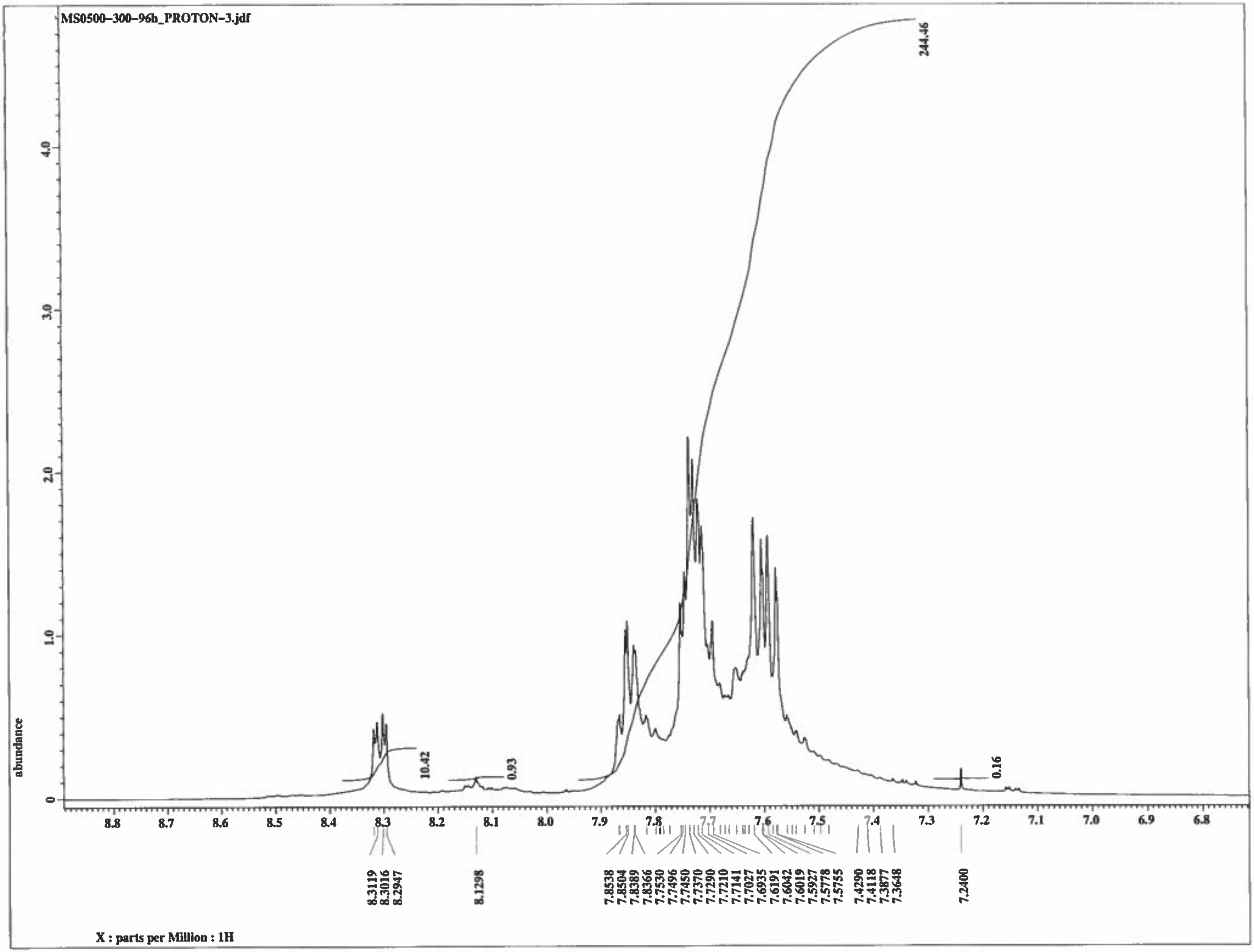

MS0500-300-96b_PROTON-3.jdf
abundance
0
1.0
2.0
3.0
4.0
10.42
0.93
244.46
0.16
8.8
8.7
8.6
8.5
8.4
8.3
8.2
8.1
8.0
7.9
7.8
7.7
7.6
7.5
7.4
7.3
7.2
7.1
7.0
6.9
6.8
8.3119
8.3016
8.2947
8.1298
7.8538
7.8504
7.8389
7.8366
7.7530
7.7496
7.7450
7.7370
7.7290
7.7210
7.7141
7.7027
7.6935
7.6191
7.6042
7.6019
7.5927
7.5778
7.5755
7.4290
7.4118
7.3877
7.3648
7.2400
X : parts per Million : 1H

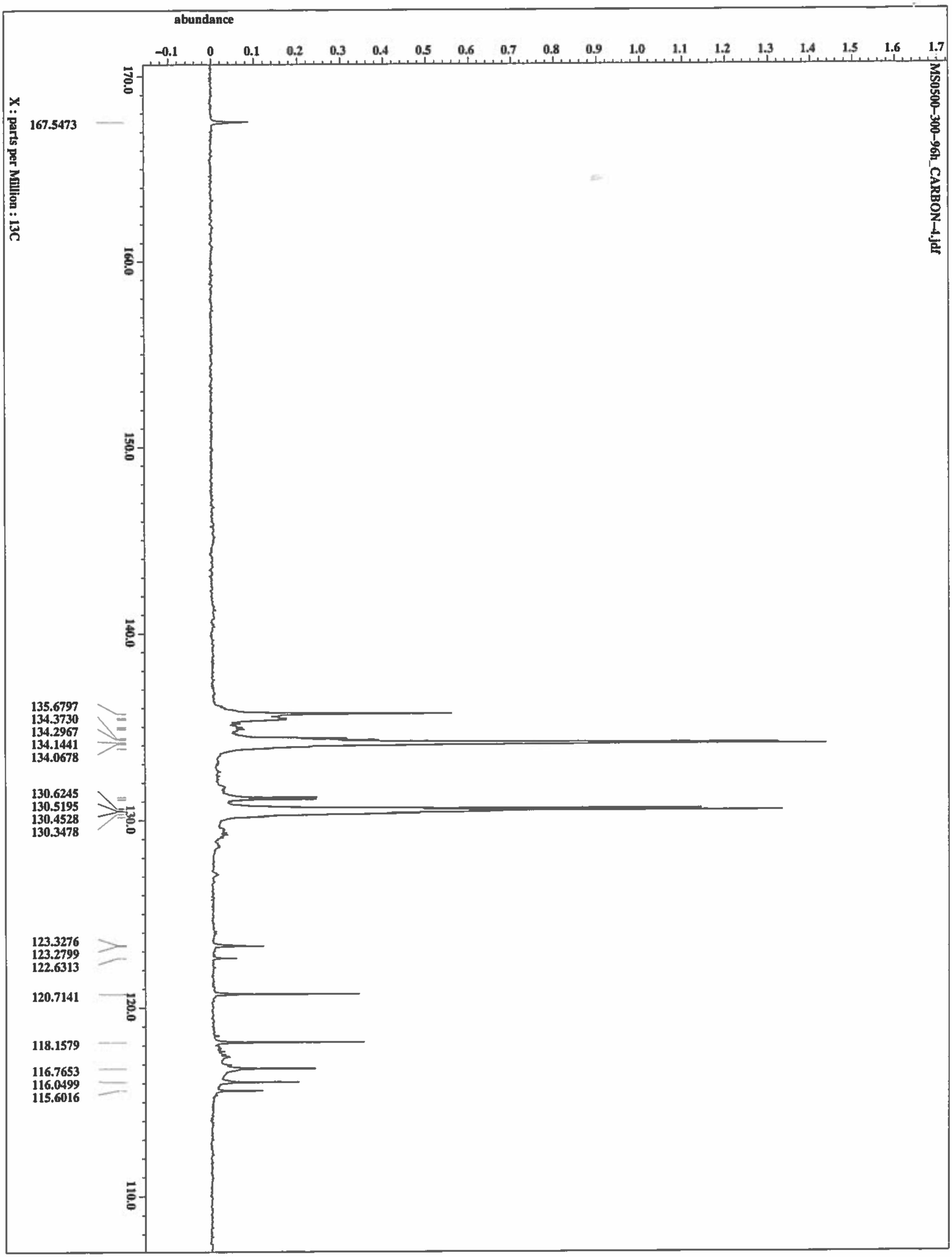

X : parts per Million : 13C
abundance
MS0500-300-96h_CARBON-4.jdf
167.5473
135.6797
134.3730
134.2967
134.1441
134.0678
130.6245
130.5195
130.4528
130.3478
123.3276
123.2799
122.6313
120.7141
118.1579
116.7653
116.0499
115.6016

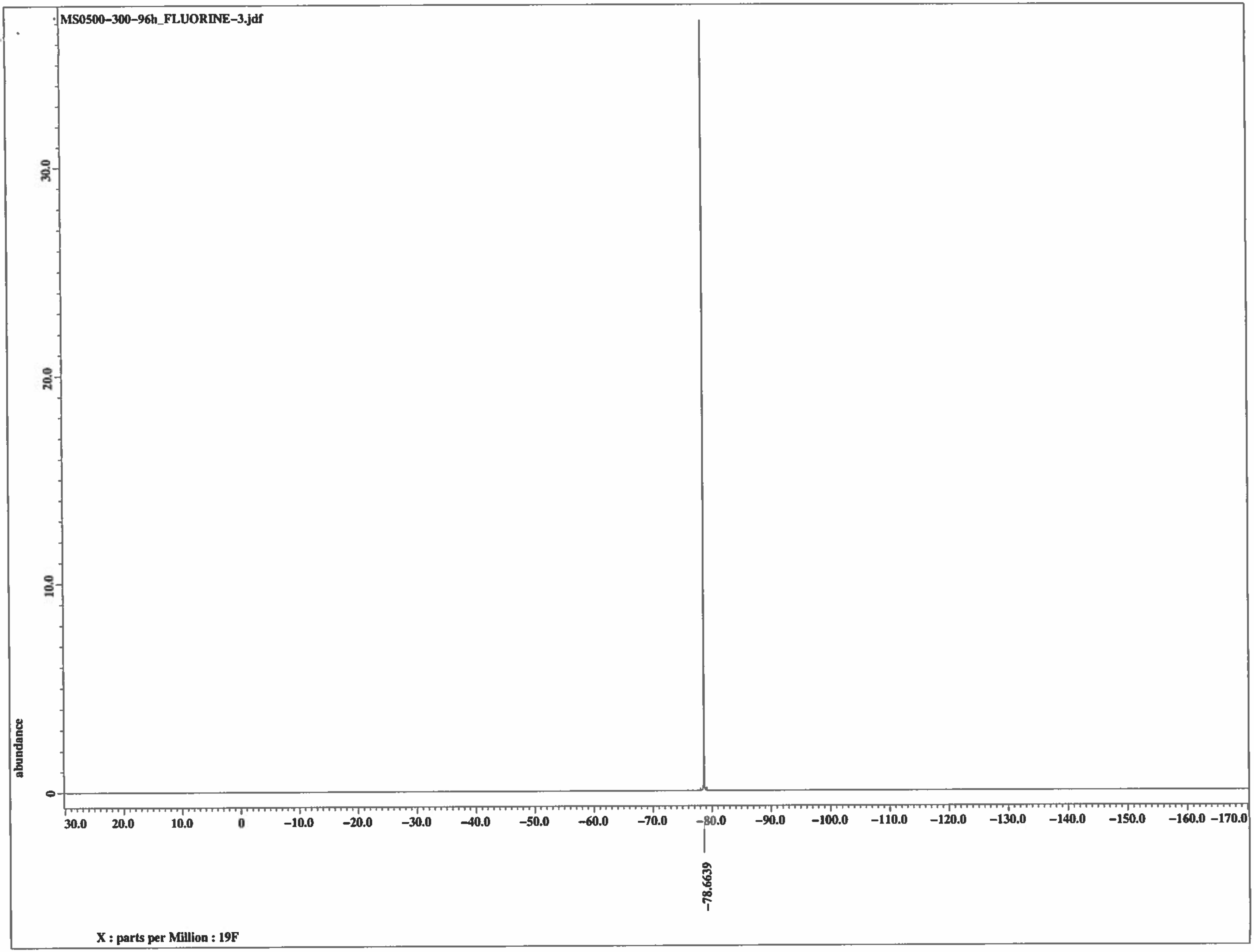
MS0500-300-96h_FLUORINE-3.jdf
abundance
0
10.0
20.0
30.0
30.0 20.0 10.0 0 −10.0 −20.0 −30.0 −40.0 −50.0 −60.0 −70.0 −80.0 −90.0 −100.0 −110.0 −120.0 −130.0 −140.0 −150.0 −160.0 −170.0
−78.6639
X : parts per Million : 19F

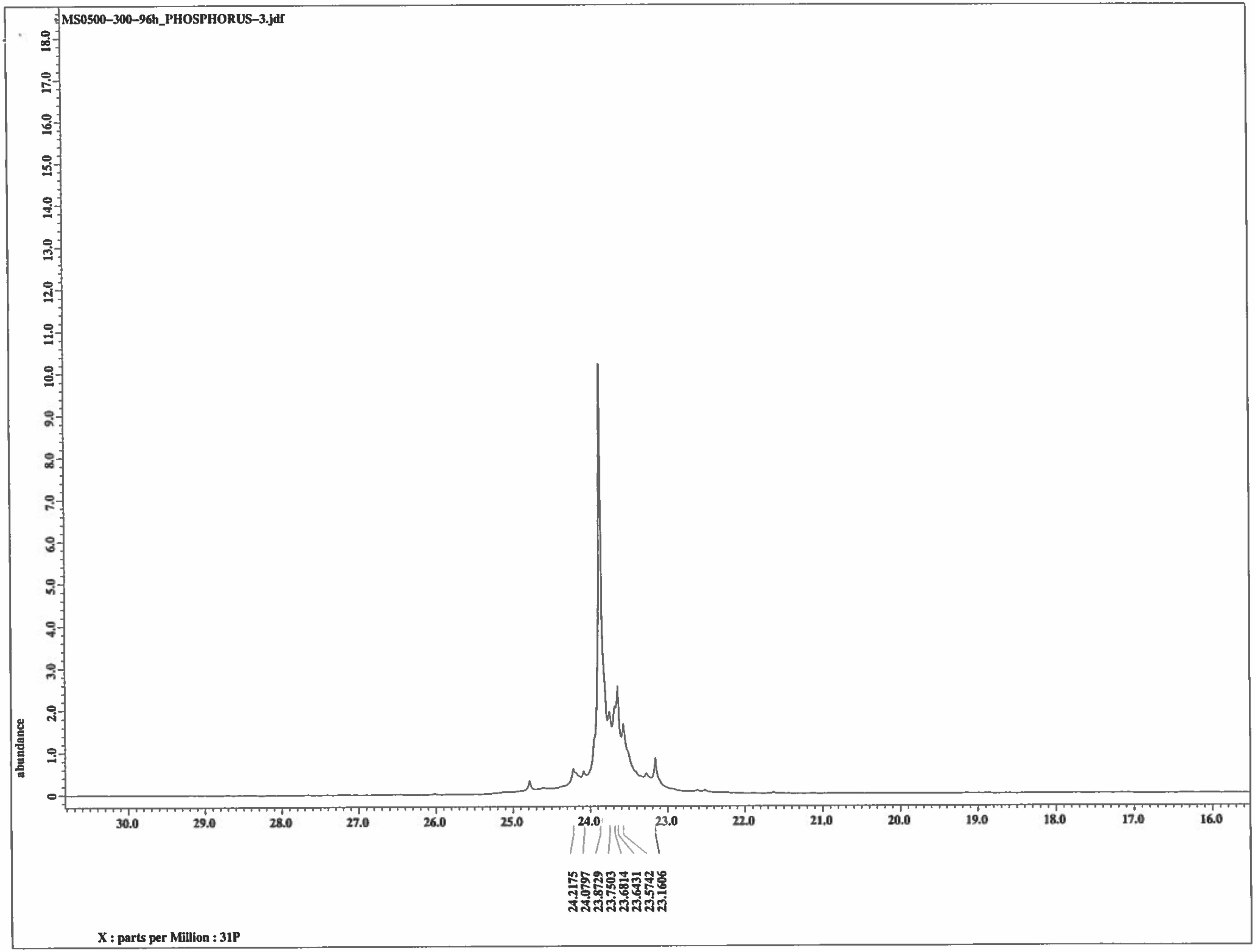

MS0500-300-96h_PHOSPHORUS-3.jdf
abundance
0
1.0
2.0
3.0
4.0
5.0
6.0
7.0
8.0
9.0
10.0
11.0
12.0
13.0
14.0
15.0
16.0
17.0
18.0
30.0
29.0
28.0
27.0
26.0
25.0
24.0
23.0
22.0
21.0
20.0
19.0
18.0
17.0
16.0
24.2175
24.0797
23.8729
23.7503
23.6814
23.6431
23.5742
23.1606
X : parts per Million : 31P

Compound 13 Pre- and Post-heating NMR Spectra

Temperature of Post-heating samples noted in upper left corner of each spectrum

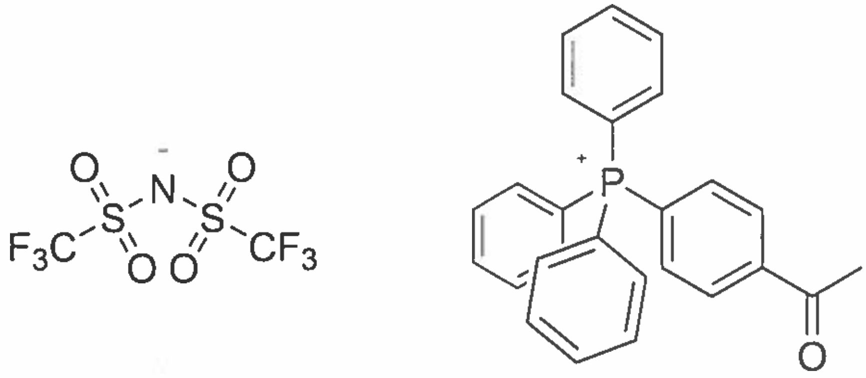

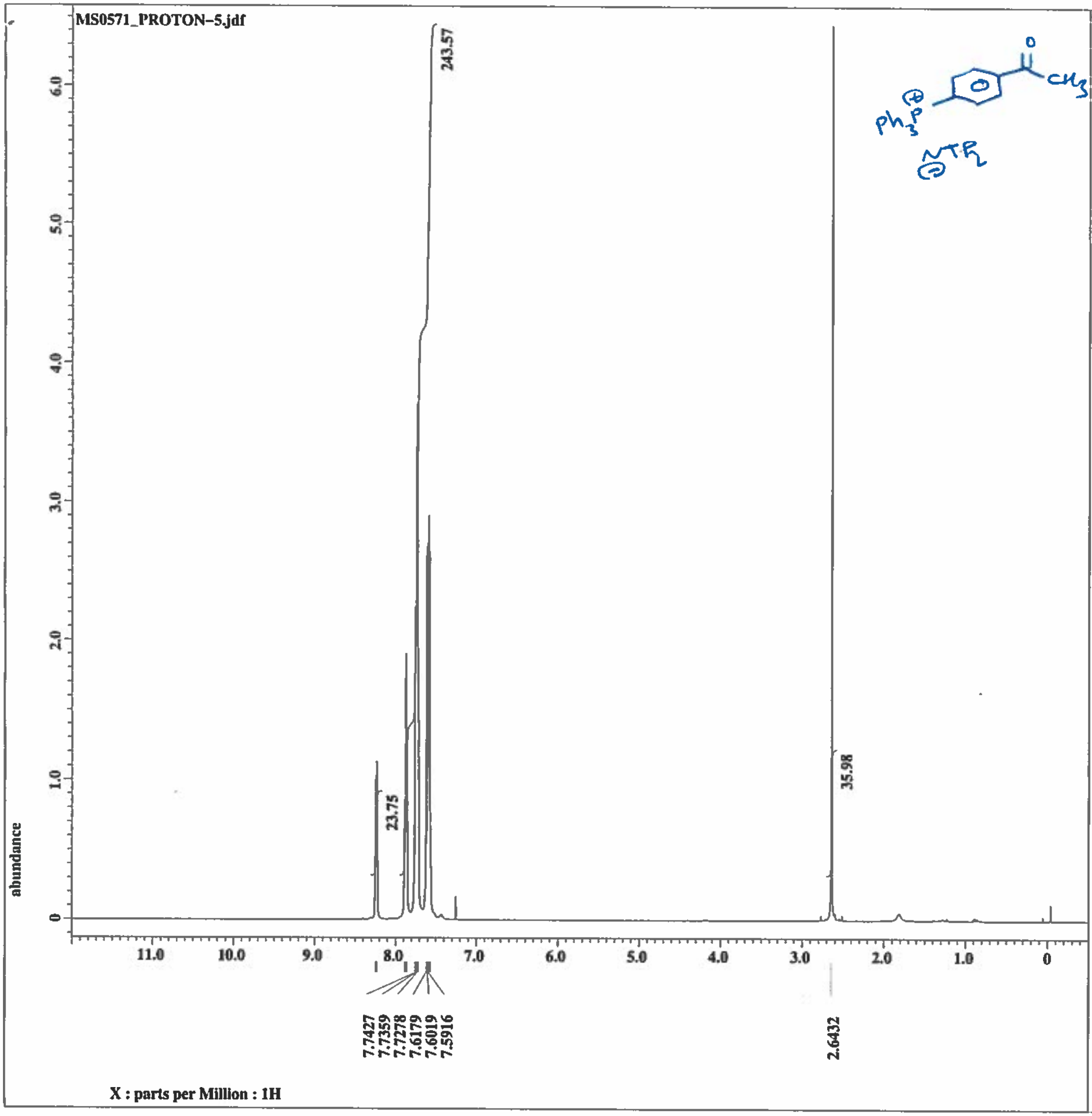

Filename	= MS0571_PROTON-5.jdf
Author	= Jim Davis
Experiment	= single_pulse.ex2
Sample_id	= MS0571
Solvent	= CHLOROFORM-D
Creation_time	= 24-OCT-2018 11:17:36
Revision_time	= 24-OCT-2018 10:54:07
Current_time	= 24-OCT-2018 10:54:07
Data_format	= 1D COMPLEX
Dim_size	= 13107
Dim_title	= 1H
Dim_units	= [ppm]
Dimensions	= X
Site	= ECA 500
Spectrometer	= JNM-ECA500
Field_strength	= 11.7473579[T] (500[MH
X_acq_duration	= 1.74587904[s]
X_domain	= 1H
X_freq	= 500.15991521[MHz]
X_offset	= 5.0[ppm]
X_points	= 16384
X_prescans	= 1
X_resolution	= 0.57277737[Hz]
X_sweep	= 9.38438438[kHz]
Irr_domain	= 1H
Irr_freq	= 500.15991521[MHz]
Irr_offset	= 5.0[ppm]
Tri_domain	= 1H
Tri_freq	= 500.15991521[MHz]
Tri_offset	= 5.0[ppm]
Clipped	= FALSE
Mod_return	= 1
Scans	= 16
Total_scans	= 16
X_90_width	= 12.4[us]
X_acq_time	= 1.74587904[s]
X_angle	= 45[deg]
X_atn	= 4[dB]
X_pulse	= 6.2[us]
Irr_mode	= Off
Tri_mode	= Off
Dante_presat	= FALSE
Initial_wait	= 1[s]
Recvr_gain	= 28
Relaxation_delay	= 4[s]
Repetition_time	= 5.74587904[s]
Temp_get	= 21.5[dC]

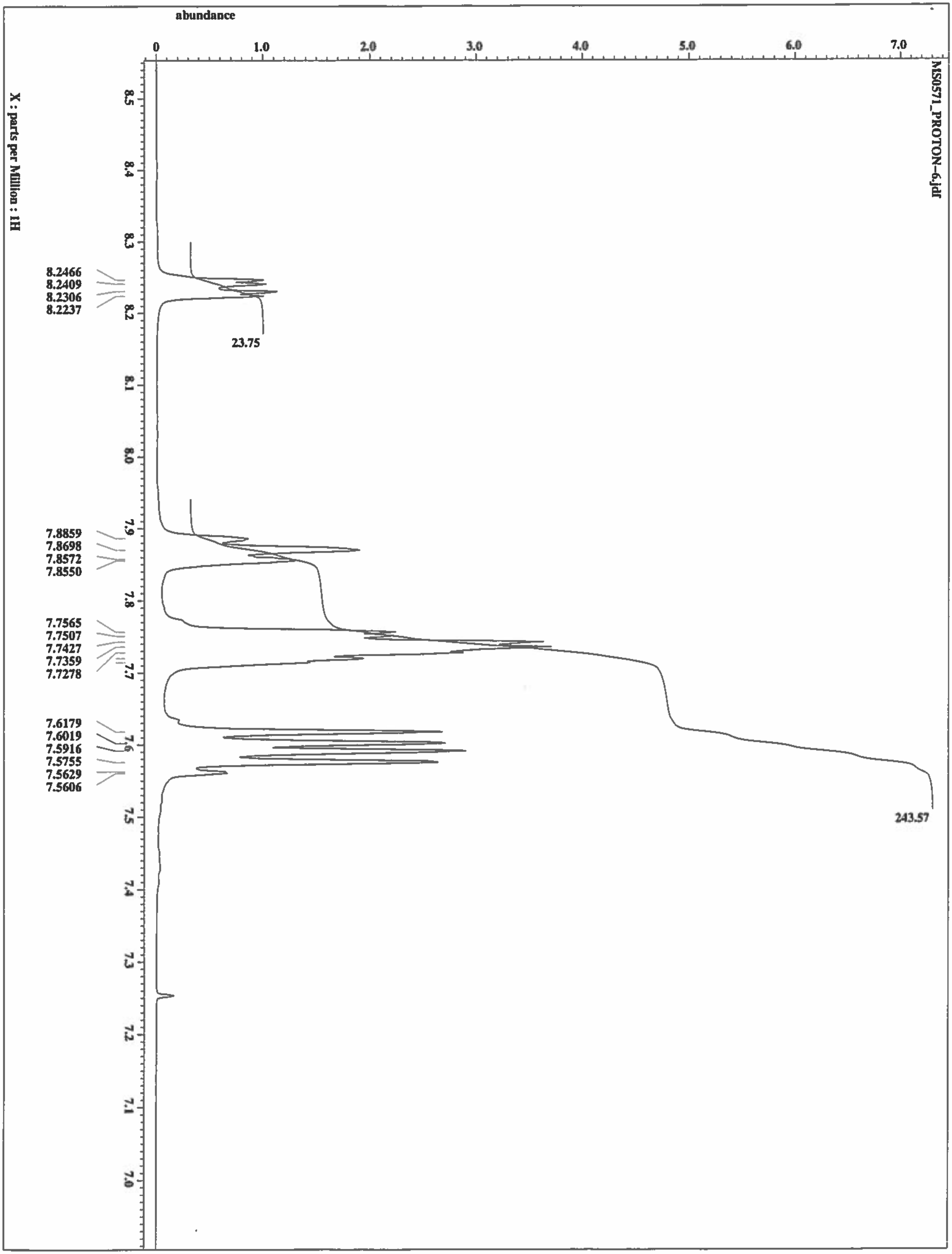
abundance
0
1.0
2.0
3.0
4.0
5.0
6.0
7.0
MS0571_PROTON-6.jdf
X : parts per Million : 1H
8.5
8.4
8.3
8.2
8.1
8.0
7.9
7.8
7.7
7.6
7.5
7.4
7.3
7.2
7.1
7.0
8.2466
8.2409
8.2306
8.2237
23.75
7.8859
7.8698
7.8572
7.8550
7.7565
7.7507
7.7427
7.7359
7.7278
7.6179
7.6019
7.5916
7.5755
7.5629
7.5606
243.57

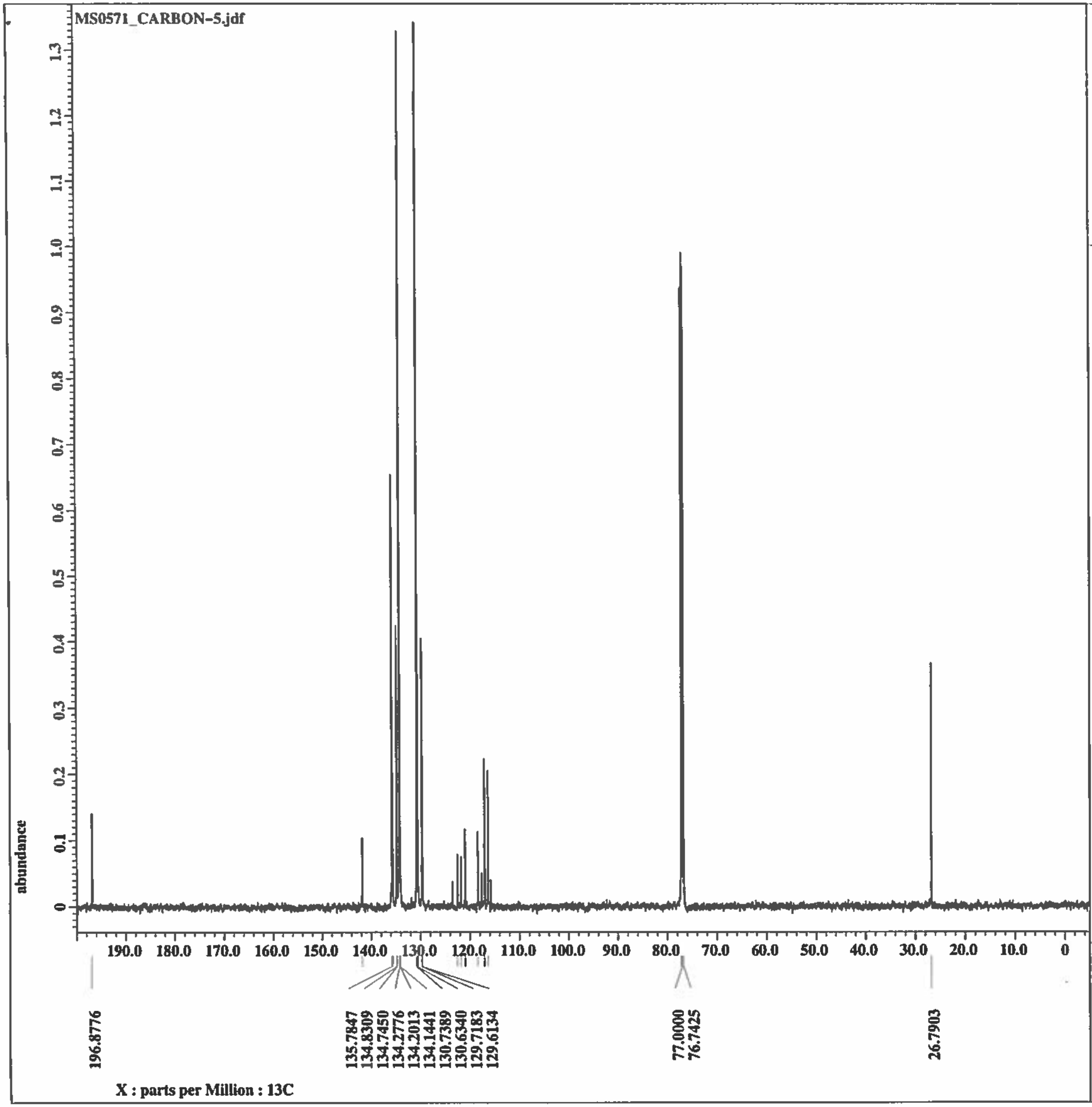

Filename	= MS0571_CARBON-5.jdf
Author	= Jim Davis
Experiment	= single_pulse_dec
Sample_id	= MS0571
Solvent	= CHLOROFORM-D
Creation_time	= 24-OCT-2018 11:39:16
Revision_time	= 24-OCT-2018 11:15:47
Current_time	= 24-OCT-2018 11:15:47
Data_format	= 1D COMPLEX
Dim_size	= 26214
Dim_title	= 13C
Dim_units	= [ppm]
Dimensions	= X
Site	= ECA 500
Spectrometer	= JNM-ECA500
Field_strength	= 11.7473579[T] (500[MH
X_acq_duration	= 0.83361792[s]
X_domain	= 13C
X_freq	= 125.76529768[MHz]
X_offset	= 100[ppm]
X_points	= 32768
X_prescans	= 4
X_resolution	= 1.19959034[Hz]
X_sweep	= 39.3081761[kHz]
Irr_domain	= 1H
Irr_freq	= 500.15991521[MHz]
Irr_offset	= 5.0[ppm]
Clipped	= FALSE
Mod_return	= 1
Scans	= 400
Total_scans	= 400
X_90_width	= 13.2[us]
X_acq_time	= 0.83361792[s]
X_angle	= 30[deg]
X_atn	= 6[dB]
X_pulse	= 4.4[us]
Irr_atn_dec	= 20.7[dB]
Irr_atn_noe	= 20.7[dB]
Irr_noise	= WALTZ
Decoupling	= TRUE
Initial_wait	= 1[s]
Noe	= TRUE
Noe_time	= 2[s]
Recvr_gain	= 60
Relaxation_delay	= 2[s]
Repetition_time	= 2.83361792[s]
Temp_get	= 22.1[dC]

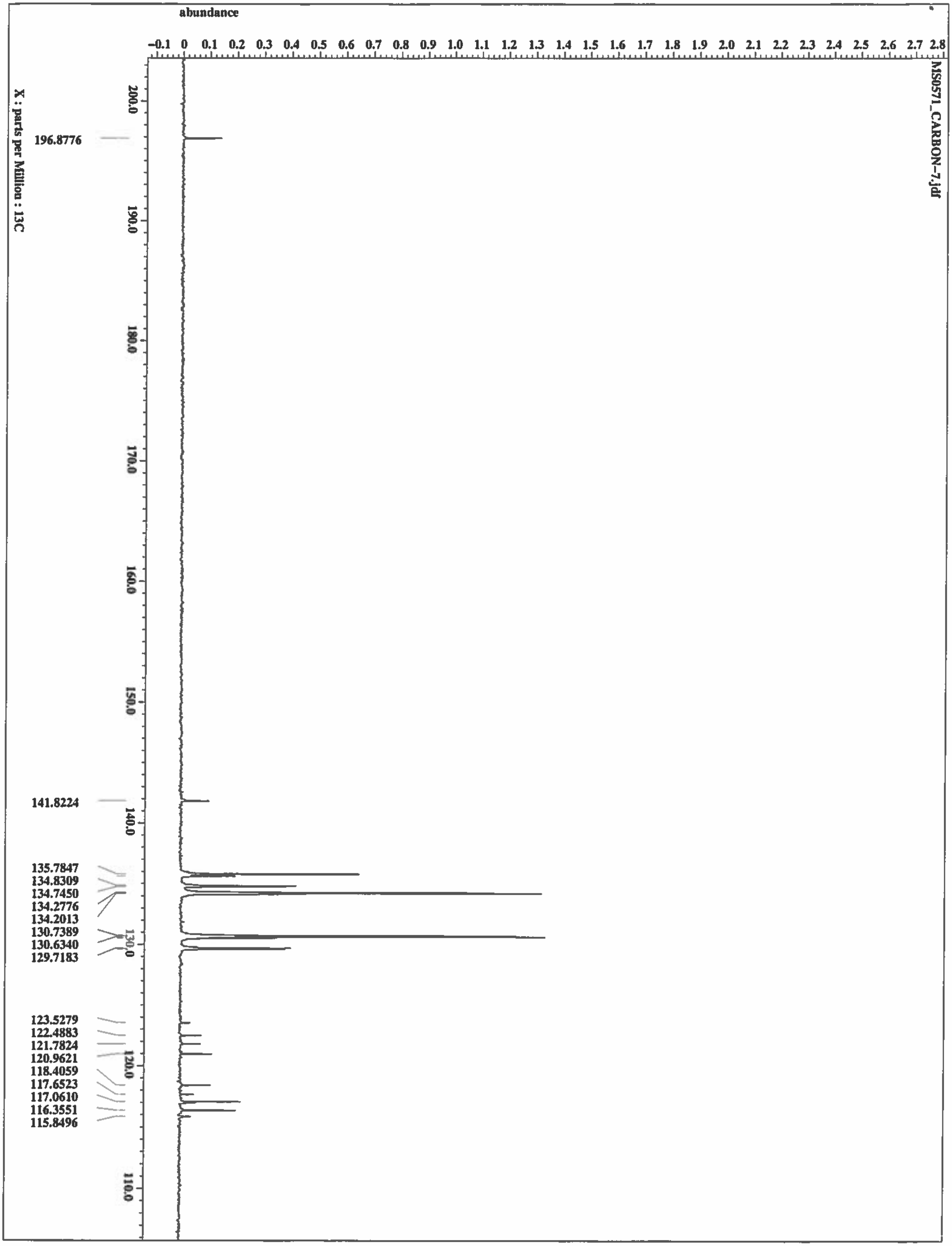

abundance
-0.1 0 0.1 0.2 0.3 0.4 0.5 0.6 0.7 0.8 0.9 1.0 1.1 1.2 1.3 1.4 1.5 1.6 1.7 1.8 1.9 2.0 2.1 2.2 2.3 2.4 2.5 2.6 2.7 2.8
MS0571_CARBON-7.jdf
X : parts per Million : 13C
200.0
190.0
180.0
170.0
160.0
150.0
140.0
130.0
120.0
110.0
196.8776
141.8224
135.7847
134.8309
134.7450
134.2776
134.2013
130.7389
130.6340
129.7183
123.5279
122.4883
121.7824
120.9621
118.4059
117.6523
117.0610
116.3551
115.8496

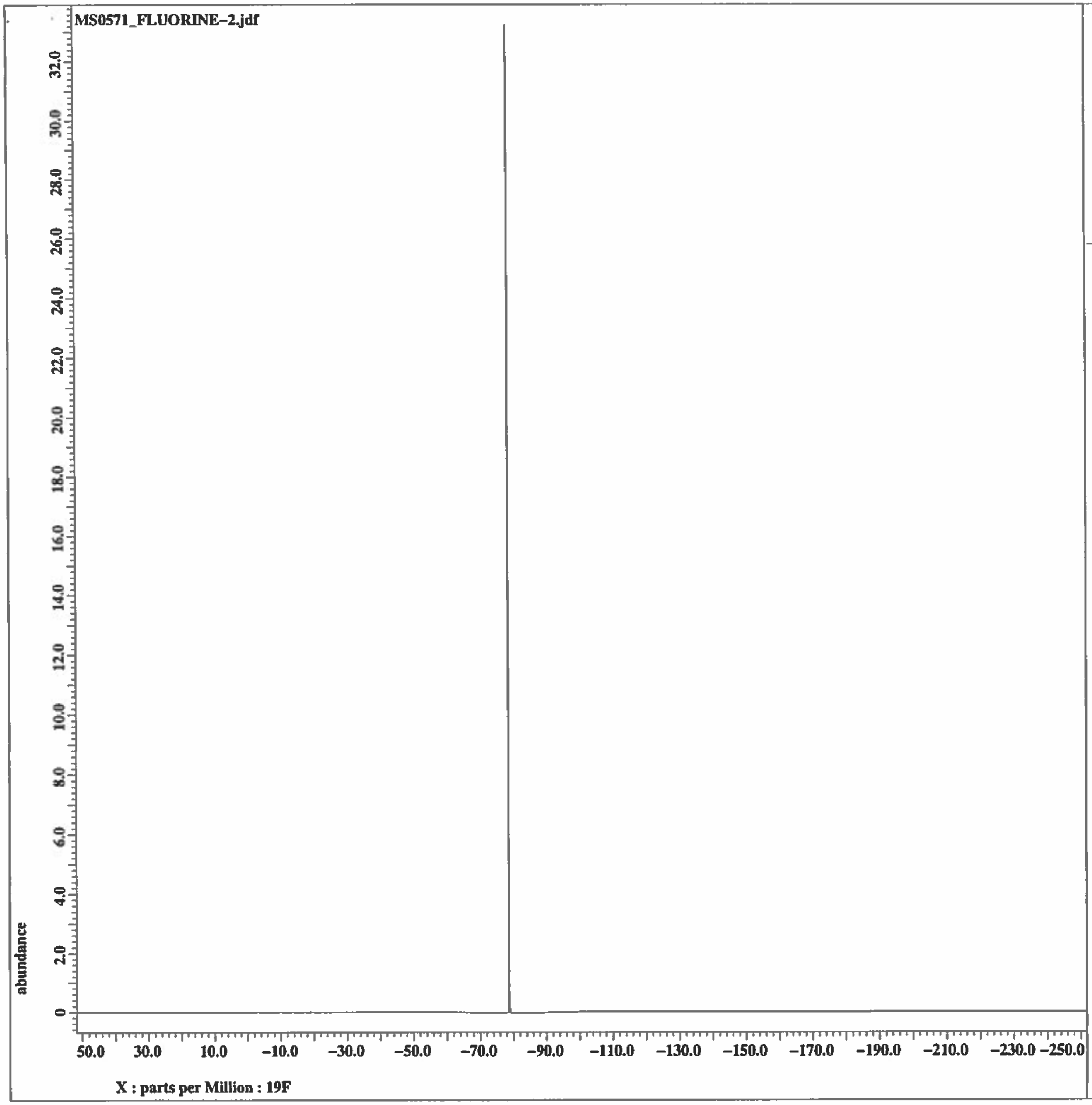

Filename	= MS0571_FLUORINE-2.jdf
Author	= Jim Davis
Experiment	= single_pulse.ex2
Sample_id	= MS0571
Solvent	= CHLOROFORM-D
Creation_time	= 5-OCT-2018 12:15:39
Revision_time	= 5-OCT-2018 11:50:32
Current_time	= 5-OCT-2018 11:50:34
Data_format	= 1D COMPLEX
Dim_size	= 104857
Dim_title	= 19F
Dim_units	= [ppm]
Dimensions	= X
Site	= ECA 500
Spectrometer	= JNM-ECA500
Field_strength	= 11.7473579[T] (500[MH
X_acq_duration	= 0.7340032[s]
X_domain	= 19F
X_freq	= 470.62046084[MHz]
X_offset	= -100[ppm]
X_points	= 131072
X_prescans	= 1
X_resolution	= 1.36239188[Hz]
X_sweep	= 178.57142857[kHz]
Irr_domain	= 19F
Irr_freq	= 470.62046084[MHz]
Irr_offset	= 5[ppm]
Tri_domain	= 19F
Tri_freq	= 470.62046084[MHz]
Tri_offset	= 5[ppm]
Clipped	= FALSE
Mod_return	= 1
Scans	= 32
Total_scans	= 32
X_90_width	= 13.1[us]
X_acq_time	= 0.7340032[s]
X_angle	= 45[deg]
X_atn	= 2.5[dB]
X_pulse	= 6.55[us]
Irr_mode	= Off
Tri_mode	= Off
Dante_presat	= FALSE
Initial_wait	= 1[s]
Recvr_gain	= 62
Relaxation_delay	= 4[s]
Repetition_time	= 4.7340032[s]
Temp_get	= 22.9[dC]

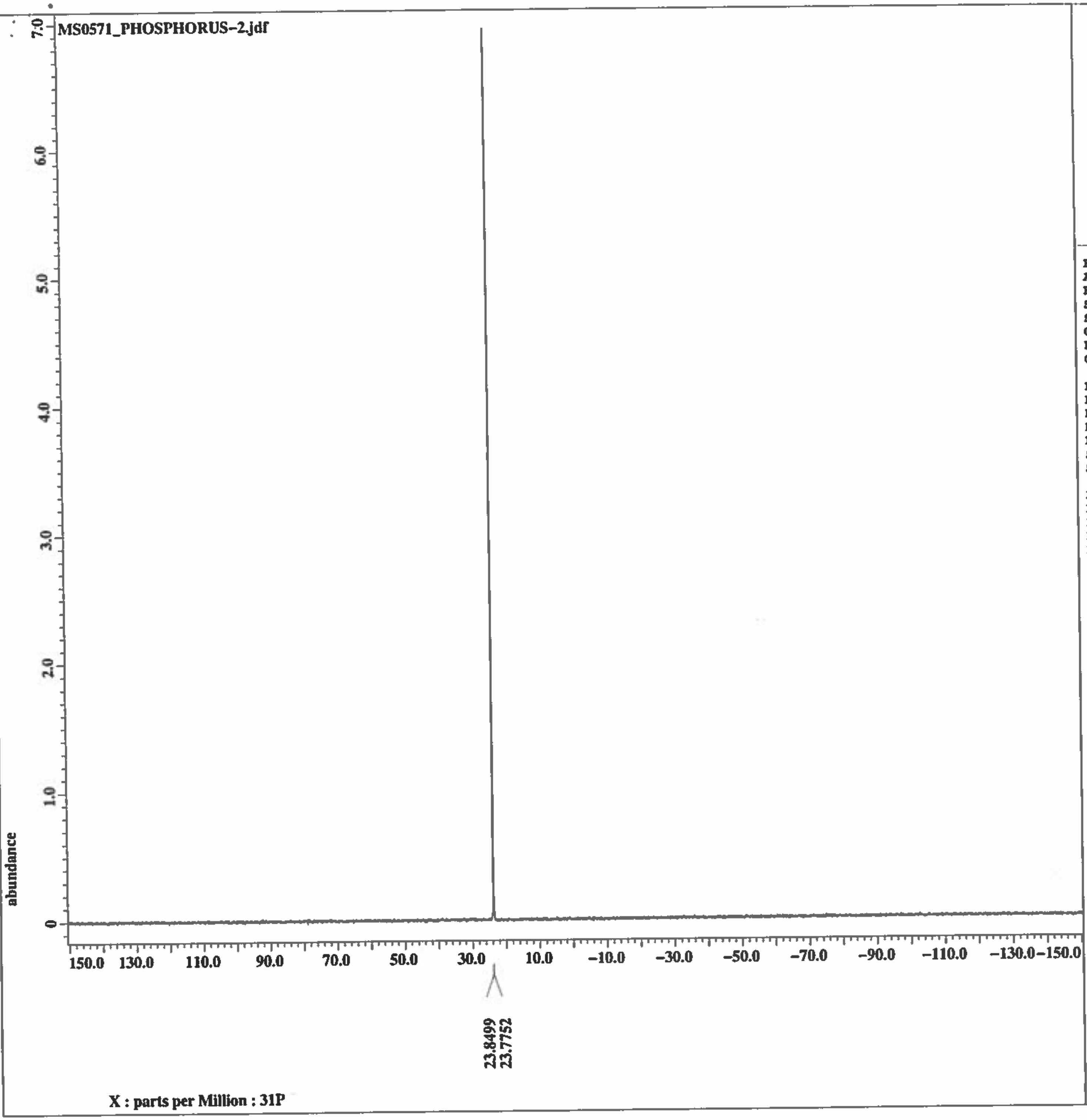

Filename	= MS0571_PHOSPHORUS-2.j
Author	= Jim Davis
Experiment	= single_pulse_dec
Sample_id	= MS0571
Solvent	= CHLOROFORM-D
Creation_time	= 5-OCT-2018 12:19:45
Revision_time	= 5-OCT-2018 11:54:39
Current_time	= 5-OCT-2018 11:54:39
Data_format	= 1D COMPLEX
Dim_size	= 52428
Dim_title	= 31P
Dim_units	= [ppm]
Dimensions	= X
Site	= ECA 500
Spectrometer	= JNM-ECA500
Field_strength	= 11.7473579[T] (500[MH
X_acq_duration	= 0.85983232[s]
X_domain	= 31P
X_freq	= 202.46831075[MHz]
X_offset	= 0[ppm]
X_points	= 65536
X_prescans	= 4
X_resolution	= 1.16301746[Hz]
X_sweep	= 76.2195122[kHz]
Irr_domain	= 1H
Irr_freq	= 500.15991521[MHz]
Irr_offset	= 5.0[ppm]
Clipped	= FALSE
Mod_return	= 1
Scans	= 30
Total_scans	= 30
X_90_width	= 14.687[us]
X_acq_time	= 0.85983232[s]
X_angle	= 30[deg]
X_atn	= 5[dB]
X_pulse	= 4.89566667[us]
Irr_atn_dec	= 20.7[dB]
Irr_atn_noe	= 20.7[dB]
Irr_noise	= WALTZ
Decoupling	= TRUE
Initial_wait	= 1[s]
Noe	= TRUE
Noe_time	= 2[s]
Recvr_gain	= 56
Relaxation_delay	= 2[s]
Repetition_time	= 2.85983232[s]
Temp_get	= 23.2[dC]

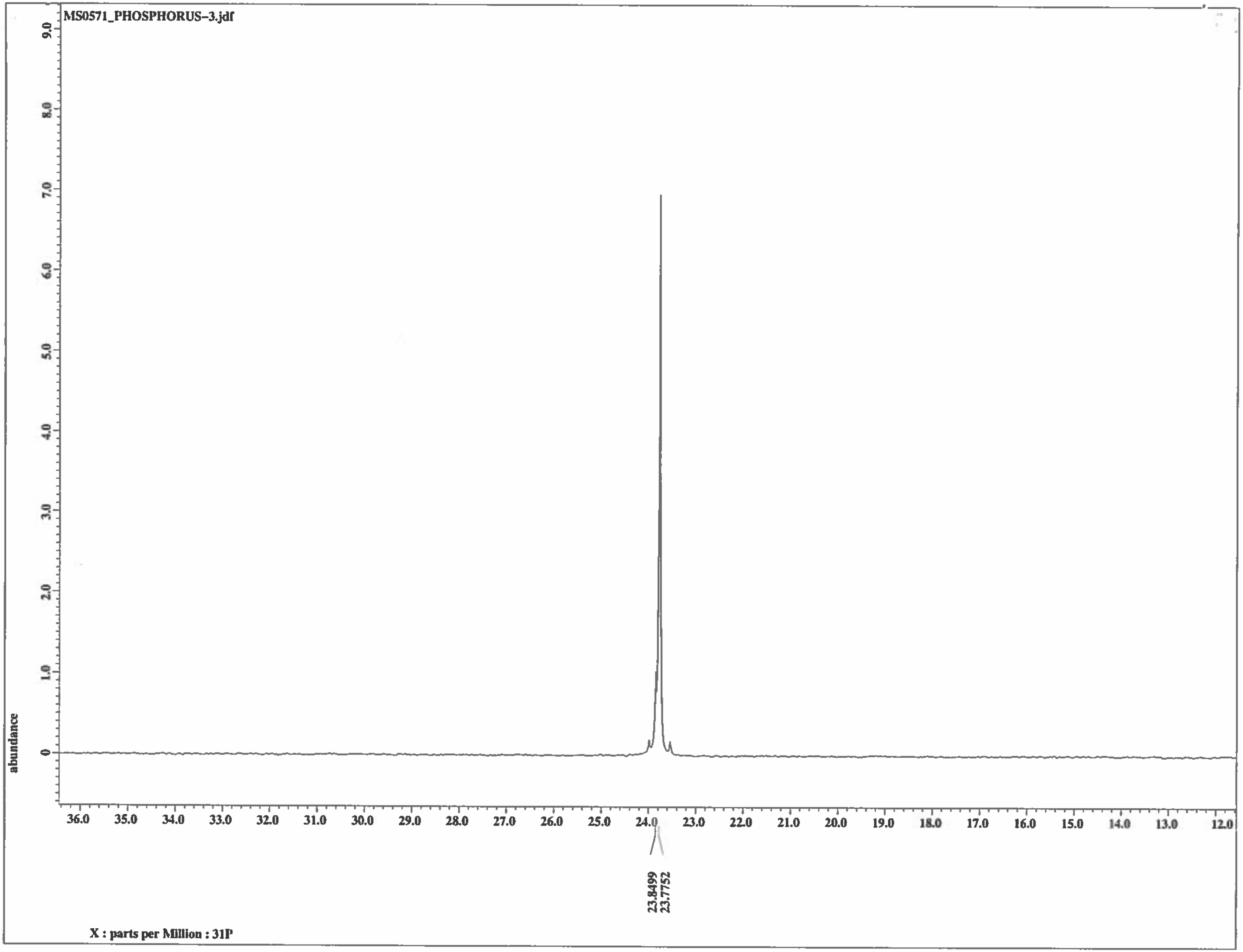
MS0571_PHOSPHORUS-3.jdf
abundance
0
1.0
2.0
3.0
4.0
5.0
6.0
7.0
8.0
9.0
36.0
35.0
34.0
33.0
32.0
31.0
30.0
29.0
28.0
27.0
26.0
25.0
24.0
23.0
22.0
21.0
20.0
19.0
18.0
17.0
16.0
15.0
14.0
13.0
12.0
23.8499
23.7752
X : parts per Million : 31P

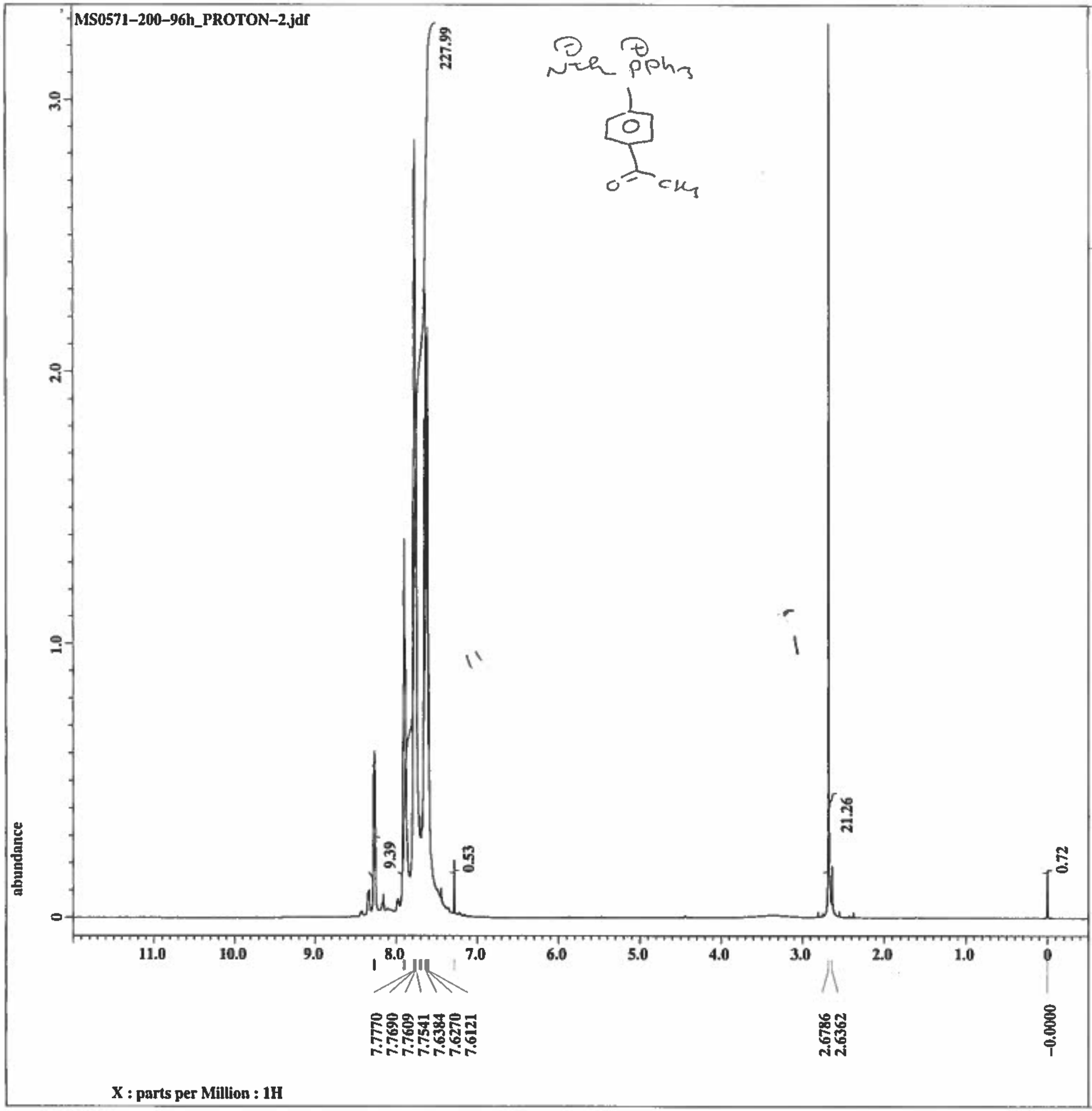

Filename	= MS0571-200-96h_PROTON
Author	= Jim Davis
Experiment	= single_pulse.ex2
Sample_id	= MS0571-200-96h
Solvent	= CHLOROFORM-D
Creation_time	= 9-OCT-2018 17:03:20
Revision_time	= 9-OCT-2018 16:37:49
Current_time	= 9-OCT-2018 16:37:49
Data_format	= 1D COMPLEX
Dim_size	= 13107
Dim_title	= 1H
Dim_units	= [ppm]
Dimensions	= X
Site	= ECA 500
Spectrometer	= JNM-ECA500
Field_strength	= 11.7473579[T] (500[MH
X_acq_duration	= 1.74587904[s]
X_domain	= 1H
X_freq	= 500.15991521[MHz]
X_offset	= 5.0[ppm]
X_points	= 16384
X_prescans	= 1
X_resolution	= 0.57277737[Hz]
X_sweep	= 9.38438438[kHz]
Irr_domain	= 1H
Irr_freq	= 500.15991521[MHz]
Irr_offset	= 5.0[ppm]
Tri_domain	= 1H
Tri_freq	= 500.15991521[MHz]
Tri_offset	= 5.0[ppm]
Clipped	= FALSE
Mod_return	= 1
Scans	= 16
Total_scans	= 16
X_90_width	= 12.4[us]
X_acq_time	= 1.74587904[s]
X_angle	= 45[deg]
X_atn	= 4[dB]
X_pulse	= 6.2[us]
Irr_mode	= Off
Tri_mode	= Off
Dante_presat	= FALSE
Initial_wait	= 1[s]
Recvr_gain	= 28
Relaxation_delay	= 4[s]
Repetition_time	= 5.74587904[s]
Temp_get	= 22.7[dC]

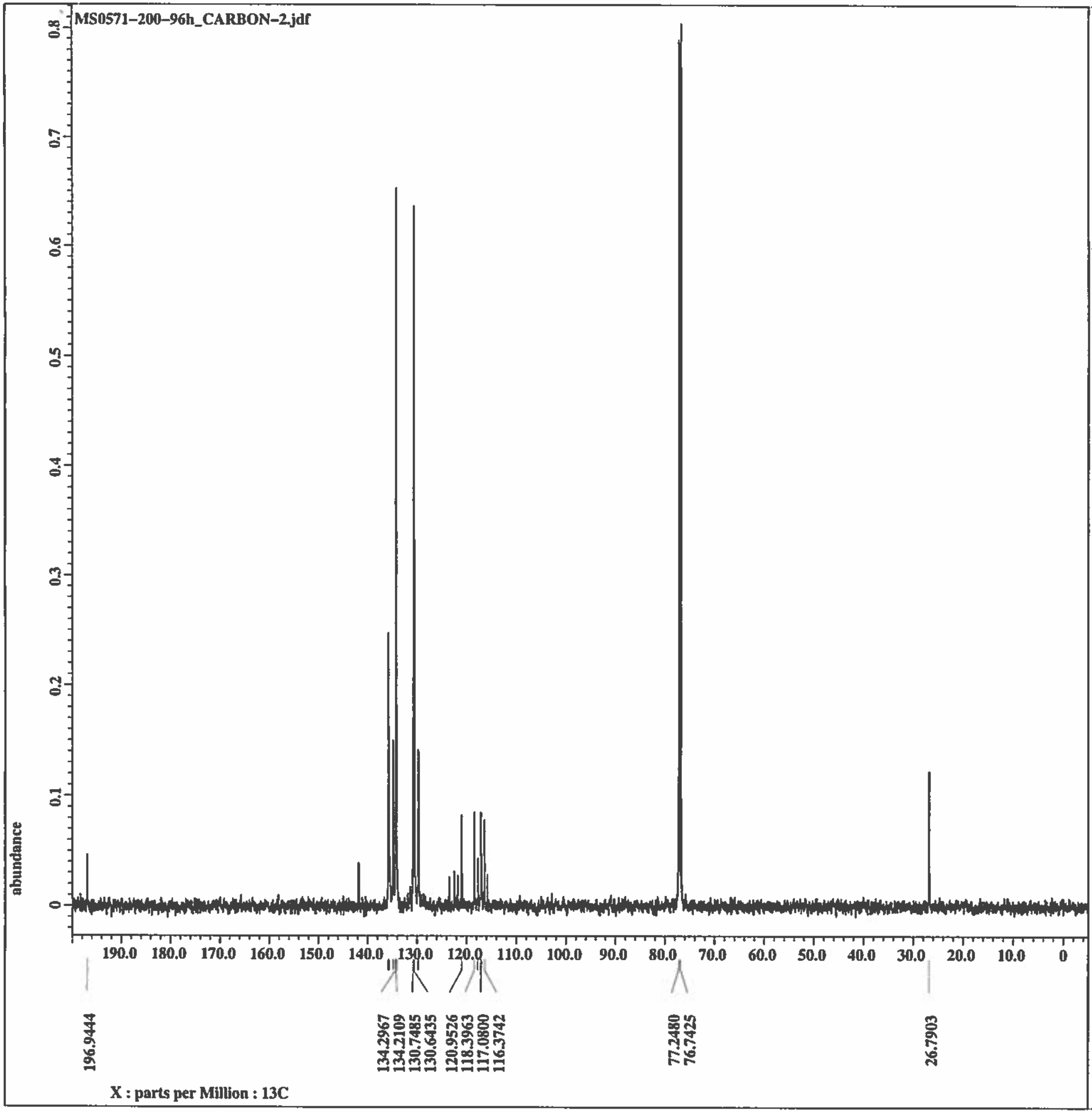

Filename	= MS0571-200-96h_CARBON
Author	= Jim Davis
Experiment	= single_pulse_dec
Sample_id	= MS0571-200-96h
Solvent	= CHLOROFORM-D
Creation_time	= 9-OCT-2018 17:15:55
Revision_time	= 9-OCT-2018 16:50:24
Current_time	= 9-OCT-2018 16:50:24
Data_format	= 1D COMPLEX
Dim_size	= 26214
Dim_title	= 13C
Dim_units	= [ppm]
Dimensions	= X
Site	= ECA 500
Spectrometer	= JNM-ECA500
Field_strength	= 11.7473579[T] (500[MH
X_acq_duration	= 0.83361792[s]
X_domain	= 13C
X_freq	= 125.76529768[MHz]
X_offset	= 100[ppm]
X_points	= 32768
X_prescans	= 4
X_resolution	= 1.19959034[Hz]
X_sweep	= 39.3081761[kHz]
Irr_domain	= 1H
Irr_freq	= 500.15991521[MHz]
Irr_offset	= 5.0[ppm]
Clipped	= FALSE
Mod_return	= 1
Scans	= 256
Total_scans	= 256
X_90_width	= 13.2[us]
X_acq_time	= 0.83361792[s]
X_angle	= 30[deg]
X_atn	= 6[dB]
X_pulse	= 4.4[us]
Irr_atn_dec	= 20.7[dB]
Irr_atn_noe	= 20.7[dB]
Irr_noise	= WALTZ
Decoupling	= TRUE
Initial_wait	= 1[s]
Noe	= TRUE
Noe_time	= 2[s]
Recvr_gain	= 60
Relaxation_delay	= 2[s]
Repetition_time	= 2.83361792[s]
Temp_get	= 23[dC]

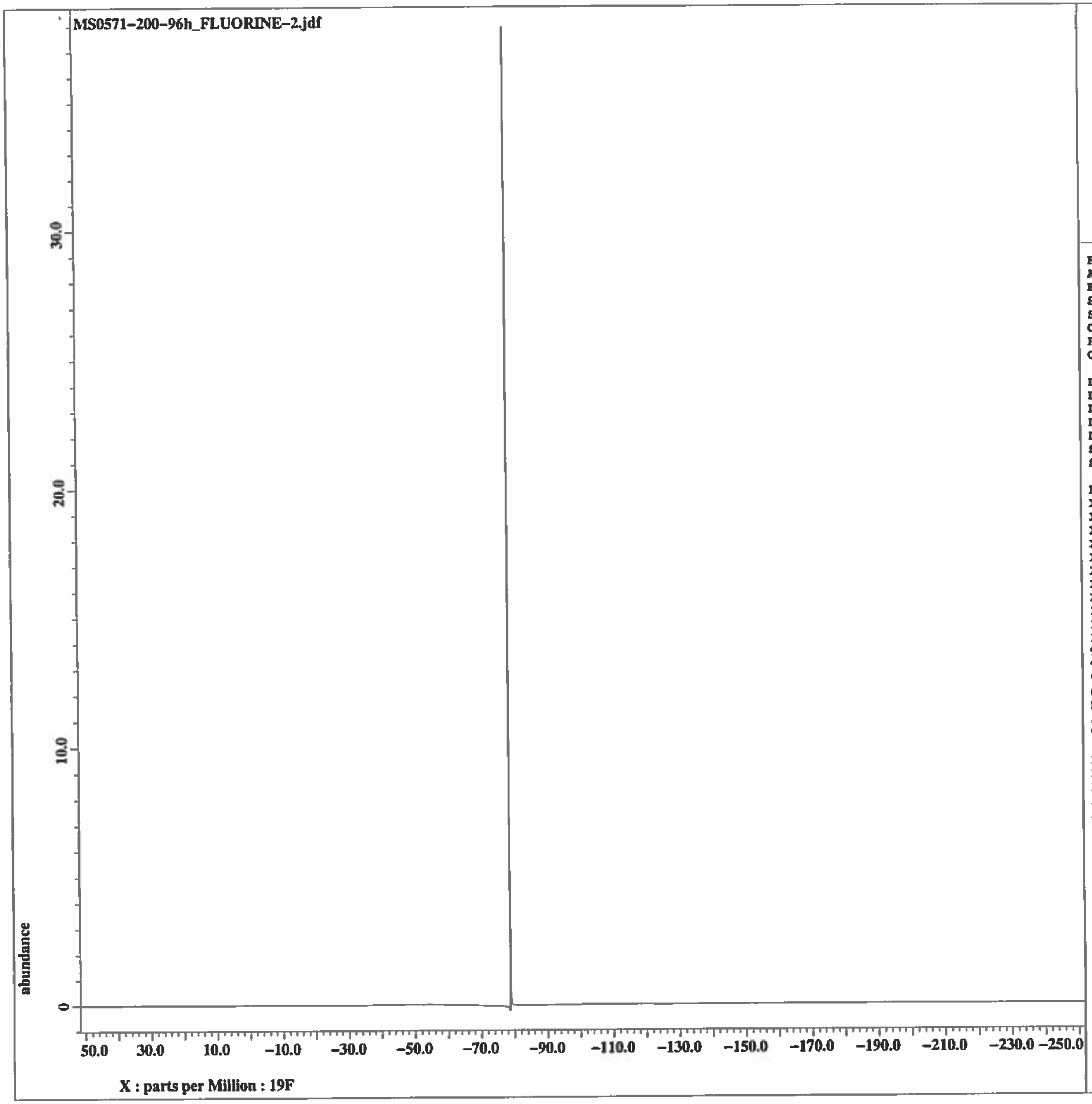


```
Filename           = MS0571-200-96h_FLUORI
Author             = Jim Davis
Experiment         = single_pulse.ex2
Sample_id          = MS0571-200-96h
Solvent            = CHLOROFORM-D
Creation_time      =  9-OCT-2018 17:20:21
Revision_time      =  9-OCT-2018 16:54:52
Current_time       =  9-OCT-2018 16:54:52

Data_format        = 1D COMPLEX
Dim_size           = 104857
Dim_title          = 19F
Dim_units          = [ppm]
Dimensions         = X
Site               = ECA 500
Spectrometer       = JNM-ECA500

Field_strength     = 11.7473579[T] (500[MH
X_acq_duration     = 0.7340032[s]
X_domain           = 19F
X_freq             = 470.62046084[MHz]
X_offset           = -100[ppm]
X_points           = 131072
X_prescans         = 1
X_resolution       = 1.36239188[Hz]
X_sweep            = 178.57142857[kHz]
Irr_domain         = 19F
Irr_freq           = 470.62046084[MHz]
Irr_offset         = 5[ppm]
Tri_domain         = 19F
Tri_freq           = 470.62046084[MHz]
Tri_offset         = 5[ppm]
Clipped            = FALSE
Mod_return         = 1
Scans              = 32
Total_scans        = 32

X_90_width         = 13.1[us]
X_acq_time         = 0.7340032[s]
X_angle            = 45[deg]
X_atn              = 2.5[dB]
X_pulse            = 6.55[us]
Irr_mode           = Off
Tri_mode           = Off
Dante_presat       = FALSE
Initial_wait       = 1[s]
Recvr_gain         = 40
Relaxation_delay   = 4[s]
Repetition_time    = 4.7340032[s]
Temp_get           = 22.7[dC]
```

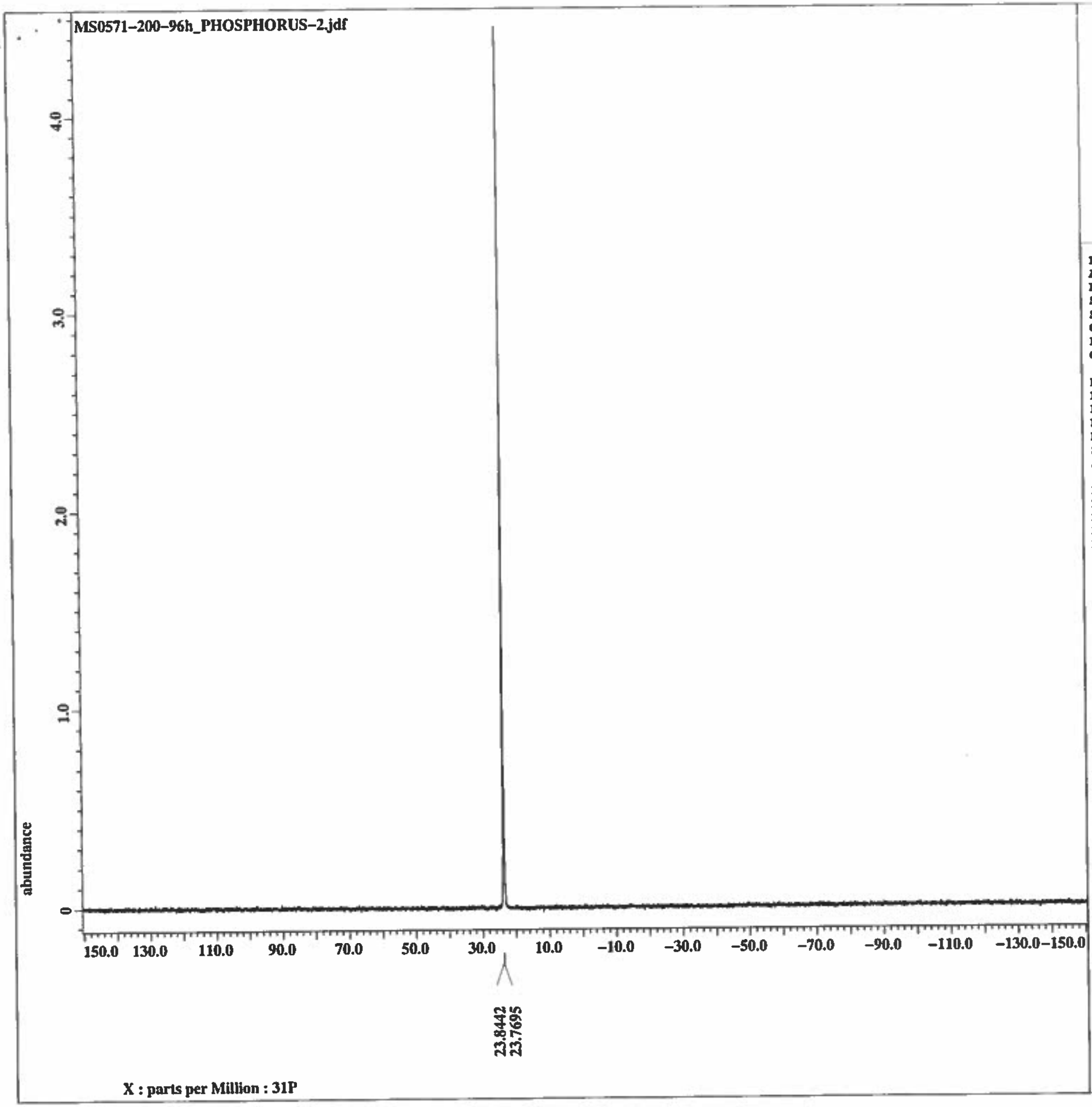

Filename	= MS0571-200-96h_PHOSPH
Author	= Jim Davis
Experiment	= single_pulse_dec
Sample_id	= MS0571-200-96h
Solvent	= CHLOROFORM-D
Creation_time	= 9-OCT-2018 17:24:42
Revision_time	= 9-OCT-2018 16:59:12
Current_time	= 9-OCT-2018 16:59:12
Data_format	= 1D COMPLEX
Dim_size	= 52428
Dim_title	= 31P
Dim_units	= [ppm]
Dimensions	= X
Site	= ECA 500
Spectrometer	= JNM-ECA500
Field_strength	= 11.7473579[T] (500[MH
X_acq_duration	= 0.85983232[s]
X_domain	= 31P
X_freq	= 202.46831075[MHz]
X_offset	= 0[ppm]
X_points	= 65536
X_prescans	= 4
X_resolution	= 1.16301746[Hz]
X_sweep	= 76.2195122[kHz]
Irr_domain	= 1H
Irr_freq	= 500.15991521[MHz]
Irr_offset	= 5.0[ppm]
Clipped	= FALSE
Mod_return	= 1
Scans	= 35
Total_scans	= 35
X_90_width	= 14.687[us]
X_acq_time	= 0.85983232[s]
X_angle	= 30[deg]
X_atn	= 5[dB]
X_pulse	= 4.89566667[us]
Irr_atn_dec	= 20.7[dB]
Irr_atn_noe	= 20.7[dB]
Irr_noise	= WALTZ
Decoupling	= TRUE
Initial_wait	= 1[s]
Noe	= TRUE
Noe_time	= 2[s]
Recvr_gain	= 56
Relaxation_delay	= 2[s]
Repetition_time	= 2.85983232[s]
Temp_get	= 23.1[dC]

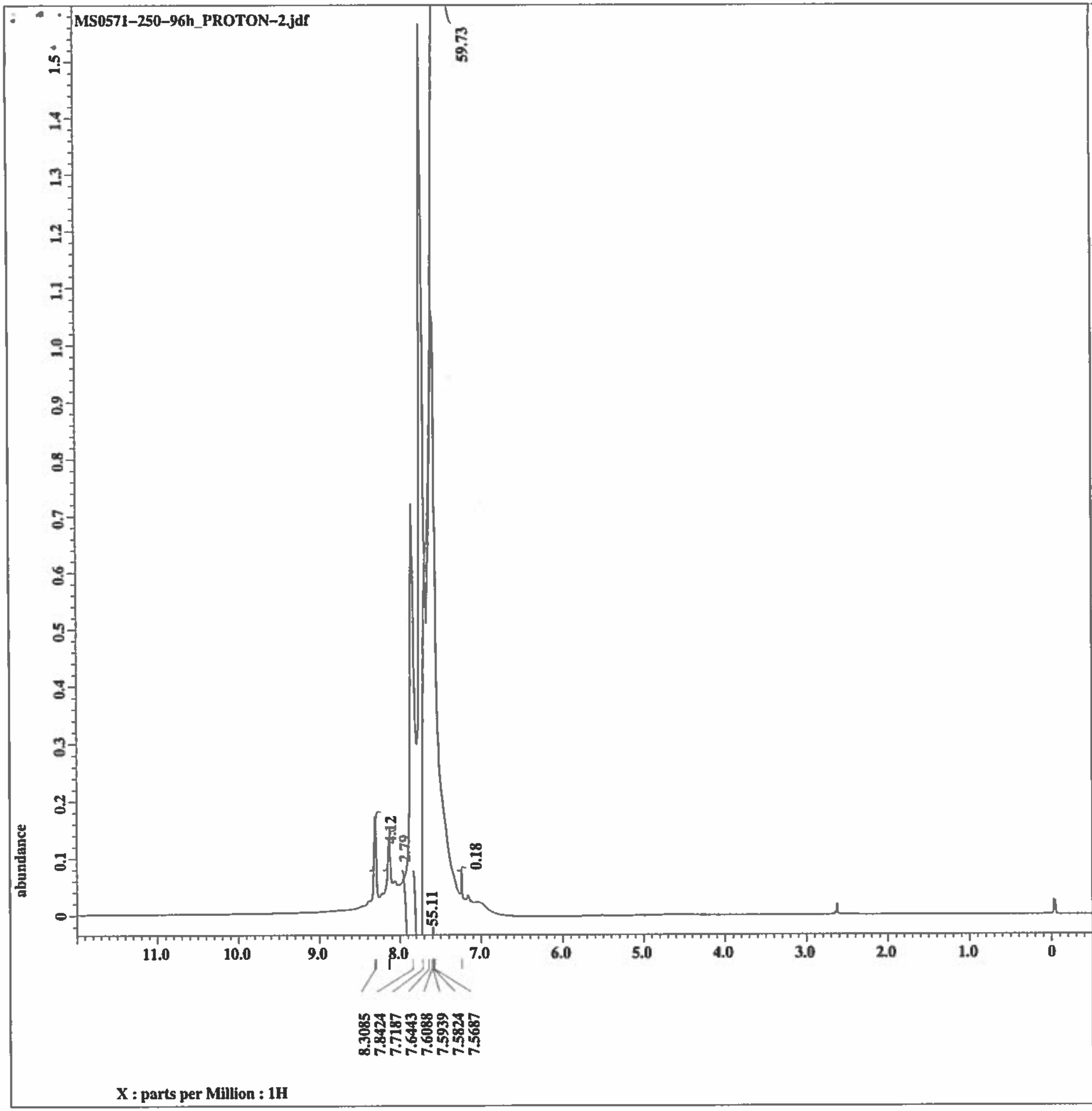

SOUTHALABAMA JAGUARS

```
Filename           = MS0571-250-96h_PROTON
Author             = Jim Davis
Experiment         = single_pulse.ex2
Sample_id          = MS0571-250-96h
Solvent            = CHLOROFORM-D
Creation_time      =  9-OCT-2018 17:31:57
Revision_time      =  9-OCT-2018 17:06:28
Current_time       =  9-OCT-2018 17:06:28

Data_format        = 1D COMPLEX
Dim_size           = 13107
Dim_title          = 1H
Dim_units          = [ppm]
Dimensions         = X
Site               = ECA 500
Spectrometer       = JNM-ECA500

Field_strength     = 11.7473579[T] (500[MH
X_acq_duration     = 1.74587904[s]
X_domain           = 1H
X_freq             = 500.15991521[MHz]
X_offset           = 5.0[ppm]
X_points           = 16384
X_prescans         = 1
X_resolution       = 0.57277737[Hz]
X_sweep            = 9.38438438[kHz]
Irr_domain         = 1H
Irr_freq           = 500.15991521[MHz]
Irr_offset         = 5.0[ppm]
Tri_domain         = 1H
Tri_freq           = 500.15991521[MHz]
Tri_offset         = 5.0[ppm]
Clipped            = FALSE
Mod_return         = 1
Scans              = 16
Total_scans        = 16

X_90_width         = 12.4[us]
X_acq_time         = 1.74587904[s]
X_angle            = 45[deg]
X_atn              = 4[dB]
X_pulse            = 6.2[us]
Irr_mode           = Off
Tri_mode           = Off
Dante_presat       = FALSE
Initial_wait       = 1[s]
Recvr_gain         = 24
Relaxation_delay   = 4[s]
Repetition_time    = 5.74587904[s]
Temp_get           = 22.8[dC]
```

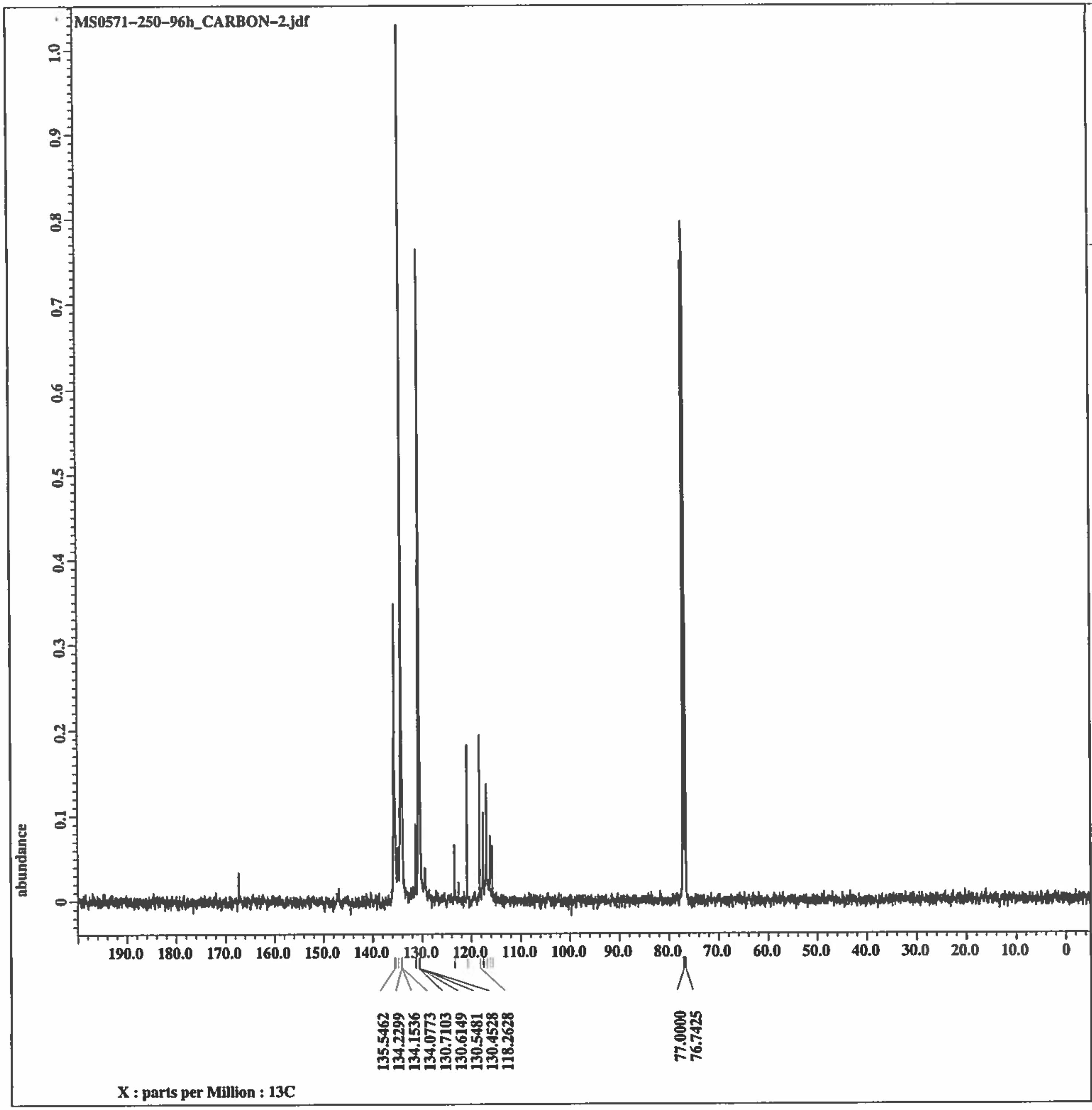

Filename	= MS0571-250-96h_CARBON
Author	= Jim Davis
Experiment	= single_pulse_dec
Sample_id	= MS0571-250-96h
Solvent	= CHLOROFORM-D
Creation_time	= 9-OCT-2018 17:45:20
Revision_time	= 9-OCT-2018 17:19:50
Current_time	= 9-OCT-2018 17:19:51
Data_format	= 1D COMPLEX
Dim_size	= 26214
Dim_title	= 13C
Dim_units	= [ppm]
Dimensions	= X
Site	= ECA 500
Spectrometer	= JNM-ECA500
Field_strength	= 11.7473579[T] (500[MH
X_acq_duration	= 0.83361792[s]
X_domain	= 13C
X_freq	= 125.76529768[MHz]
X_offset	= 100[ppm]
X_points	= 32768
X_prescans	= 4
X_resolution	= 1.19959034[Hz]
X_sweep	= 39.3081761[kHz]
Irr_domain	= 1H
Irr_freq	= 500.15991521[MHz]
Irr_offset	= 5.0[ppm]
Clipped	= FALSE
Mod_return	= 1
Scans	= 256
Total_scans	= 256
X_90_width	= 13.2[us]
X_acq_time	= 0.83361792[s]
X_angle	= 30[deg]
X_atn	= 6[dB]
X_pulse	= 4.4[us]
Irr_atn_dec	= 20.7[dB]
Irr_atn_noe	= 20.7[dB]
Irr_noise	= WALTZ
Decoupling	= TRUE
Initial_wait	= 1[s]
Noe	= TRUE
Noe_time	= 2[s]
Recvr_gain	= 60
Relaxation_delay	= 2[s]
Repetition_time	= 2.83361792[s]
Temp_get	= 23[dC]

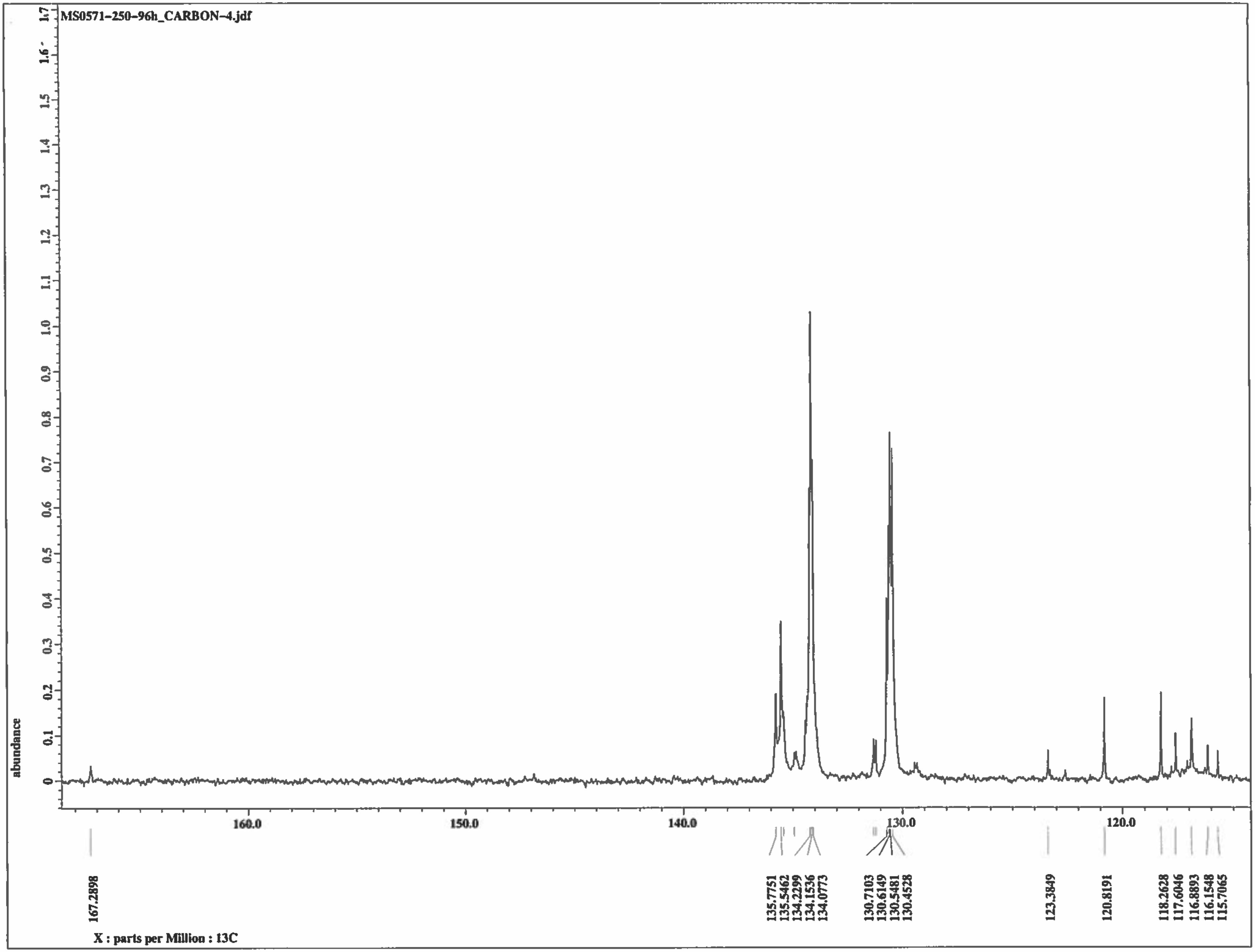
MS0571-250-96h_CARBON-4.jdf
abundance
0
0.1
0.2
0.3
0.4
0.5
0.6
0.7
0.8
0.9
1.0
1.1
1.2
1.3
1.4
1.5
1.6
1.7
160.0
150.0
140.0
130.0
120.0
167.2898
135.7751
135.5462
134.2299
134.1536
134.0773
130.7103
130.6149
130.5481
130.4528
123.3849
120.8191
118.2628
117.6046
116.8893
116.1548
115.7065
X : parts per Million : 13C

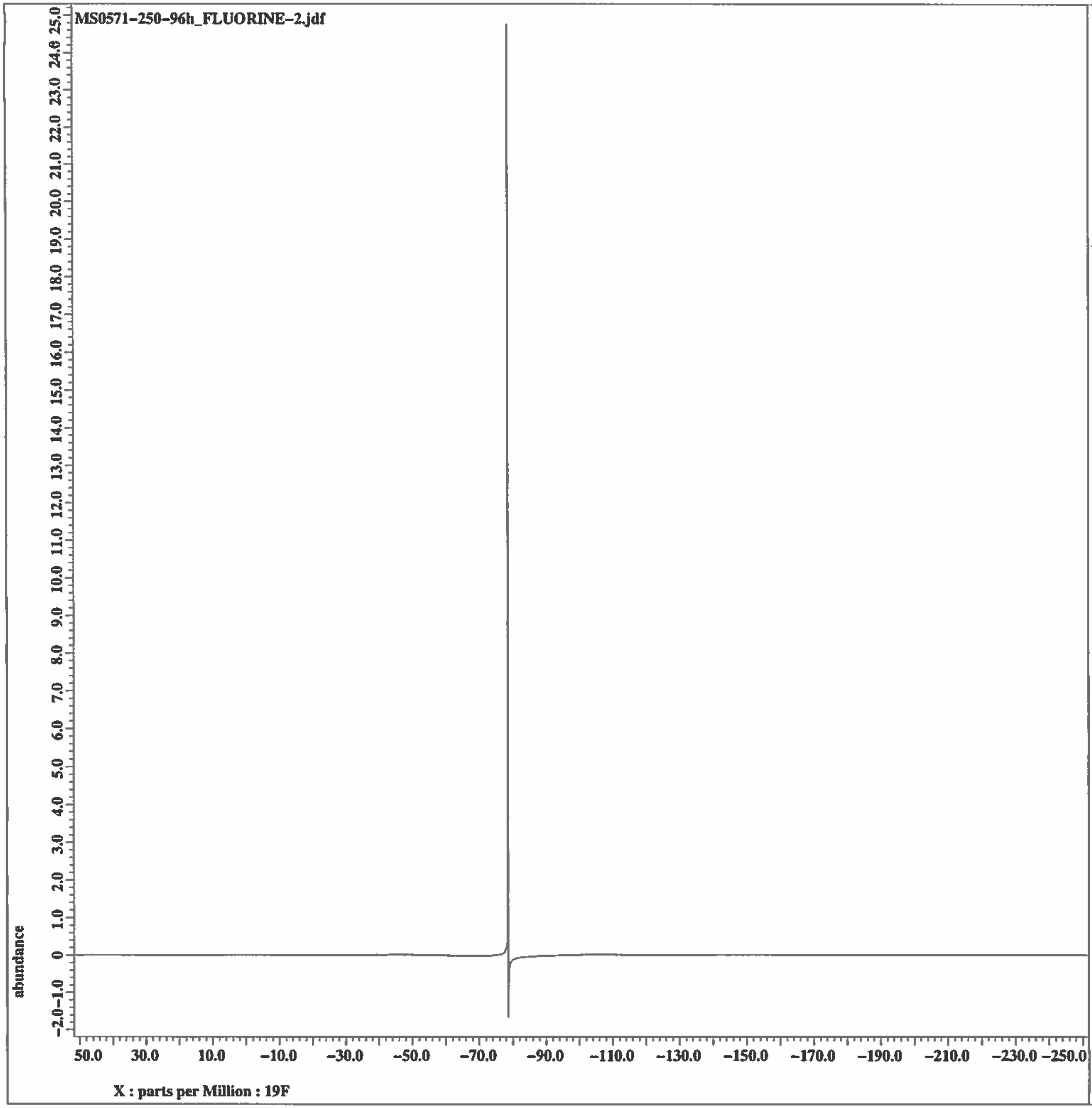

SOUTHALABAMA JAGUARS

Filename	=	MS0571-250-96h_FLUORI
Author	=	Jim Davis
Experiment	=	single_pulse.ex2
Sample_id	=	MS0571-250-96h
Solvent	=	CHLOROFORM-D
Creation_time	=	9-OCT-2018 17:49:41
Revision_time	=	9-OCT-2018 17:24:12
Current_time	=	9-OCT-2018 17:24:12
Data_format	=	1D COMPLEX
Dim_size	=	104857
Dim_title	=	19F
Dim_units	=	[ppm]
Dimensions	=	X
Site	=	ECA 500
Spectrometer	=	JNM-ECA500
Field_strength	=	11.7473579[T] (500[MH
X_acq_duration	=	0.7340032[s]
X_domain	=	19F
X_freq	=	470.62046084[MHz]
X_offset	=	-100[ppm]
X_points	=	131072
X_prescans	=	1
X_resolution	=	1.36239188[Hz]
X_sweep	=	178.57142857[kHz]
Irr_domain	=	19F
Irr_freq	=	470.62046084[MHz]
Irr_offset	=	5[ppm]
Tri_domain	=	19F
Tri_freq	=	470.62046084[MHz]
Tri_offset	=	5[ppm]
Clipped	=	FALSE
Mod_return	=	1
Scans	=	32
Total_scans	=	32
X_90_width	=	13.1[us]
X_acq_time	=	0.7340032[s]
X_angle	=	45[deg]
X_atn	=	2.5[dB]
X_pulse	=	6.55[us]
Irr_mode	=	Off
Tri_mode	=	Off
Dante_presat	=	FALSE
Initial_wait	=	1[s]
Recvr_gain	=	36
Relaxation_delay	=	4[s]
Repetition_time	=	4.7340032[s]
Temp_get	=	22.7[dC]

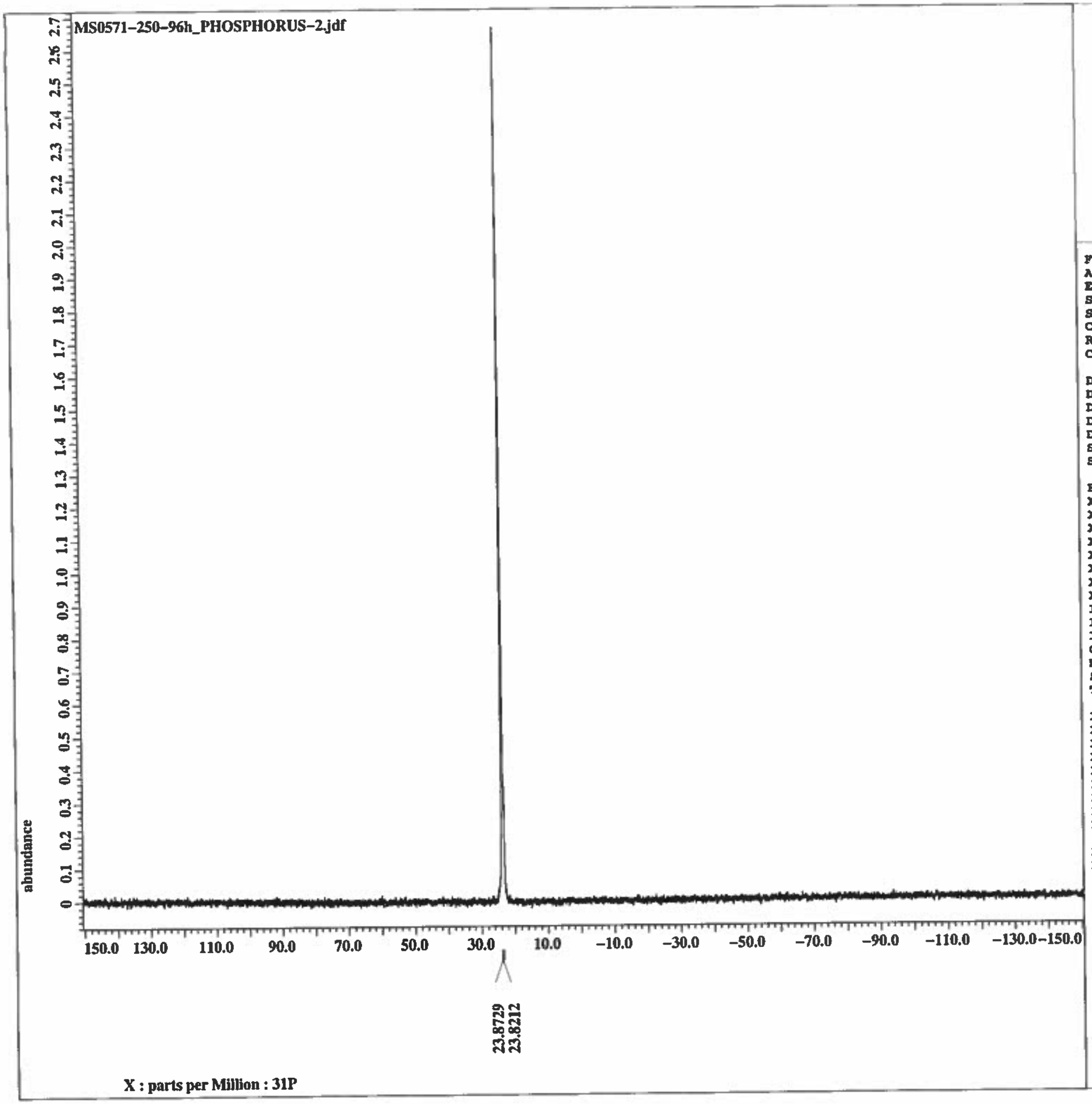


```
Filename            = MS0571-250-96h_PHOSPH
Author              = Jim Davis
Experiment          = single_pulse_dec
Sample_id           = MS0571-250-96h
Solvent             = CHLOROFORM-D
Creation_time       =  9-OCT-2018 17:53:48
Revision_time       =  9-OCT-2018 17:28:19
Current_time        =  9-OCT-2018 17:28:19

Data_format         = 1D COMPLEX
Dim_size            = 52428
Dim_title           = 31P
Dim_units           = [ppm]
Dimensions          = X
Site                = ECA 500
Spectrometer        = JNM-ECA500

Field_strength      = 11.7473579[T] (500[MH
X_acq_duration      = 0.85983232[s]
X_domain            = 31P
X_freq              = 202.46831075[MHz]
X_offset            = 0[ppm]
X_points            = 65536
X_prescans          = 4
X_resolution        = 1.16301746[Hz]
X_sweep             = 76.2195122[kHz]
Irr_domain          = 1H
Irr_freq            = 500.15991521[MHz]
Irr_offset          = 5.0[ppm]
Clipped             = FALSE
Mod_return          = 1
Scans               = 35
Total_scans         = 35

X_90_width          = 14.687[us]
X_acq_time          = 0.85983232[s]
X_angle             = 30[deg]
X_atn               = 5[dB]
X_pulse             = 4.89566667[us]
Irr_atn_dec         = 20.7[dB]
Irr_atn_noe         = 20.7[dB]
Irr_noise           = WALTZ
Decoupling          = TRUE
Initial_wait        = 1[s]
Noe                 = TRUE
Noe_time            = 2[s]
Recvr_gain          = 56
Relaxation_delay    = 2[s]
Repetition_time     = 2.85983232[s]
Temp_get            = 23[dC]
```

MS0571-300-96h_PROTON-2.jdf

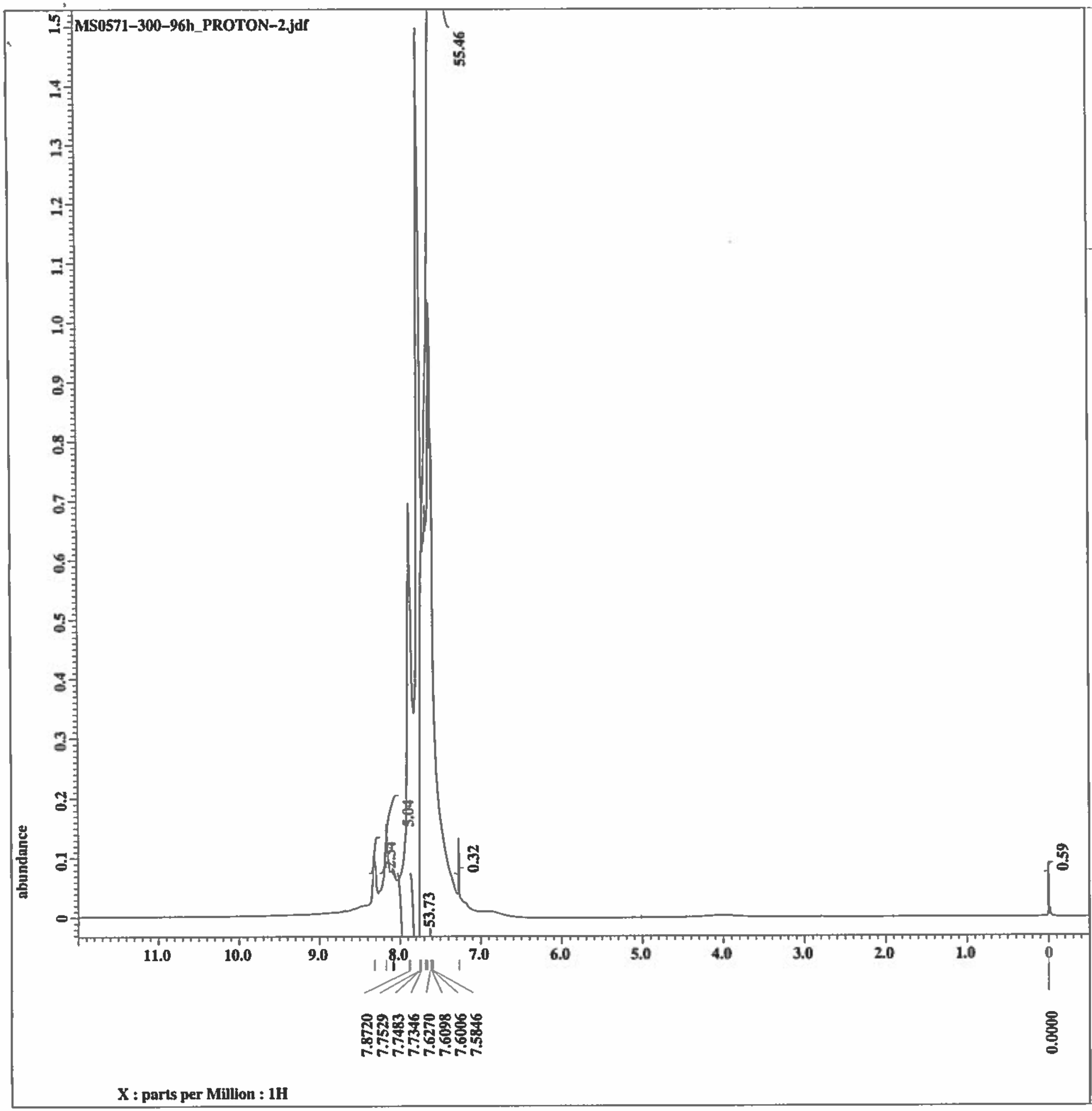

Filename	= MS0571-300-96h_PROTON
Author	= Jim Davis
Experiment	= single_pulse.ex2
Sample_id	= MS0571-300-96h
Solvent	= CHLOROFORM-D
Creation_time	= 9-OCT-2018 18:01:12
Revision_time	= 9-OCT-2018 17:35:42
Current_time	= 9-OCT-2018 17:35:43
Data_format	= 1D COMPLEX
Dim_size	= 13107
Dim_title	= 1H
Dim_units	= [ppm]
Dimensions	= X
Site	= ECA 500
Spectrometer	= JNM-ECA500
Field_strength	= 11.7473579[T] (500[MH
X_acq_duration	= 1.74587904[s]
X_domain	= 1H
X_freq	= 500.15991521[MHz]
X_offset	= 5.0[ppm]
X_points	= 16384
X_prescans	= 1
X_resolution	= 0.57277737[Hz]
X_sweep	= 9.38438438[kHz]
Irr_domain	= 1H
Irr_freq	= 500.15991521[MHz]
Irr_offset	= 5.0[ppm]
Tri_domain	= 1H
Tri_freq	= 500.15991521[MHz]
Tri_offset	= 5.0[ppm]
Clipped	= FALSE
Mod_return	= 1
Scans	= 16
Total_scans	= 16
X_90_width	= 12.4[us]
X_acq_time	= 1.74587904[s]
X_angle	= 45[deg]
X_atn	= 4[dB]
X_pulse	= 6.2[us]
Irr_mode	= Off
Tri_mode	= Off
Dante_presat	= FALSE
Initial_wait	= 1[s]
Recvr_gain	= 26
Relaxation_delay	= 4[s]
Repetition_time	= 5.74587904[s]
Temp_get	= 22.7[dC]

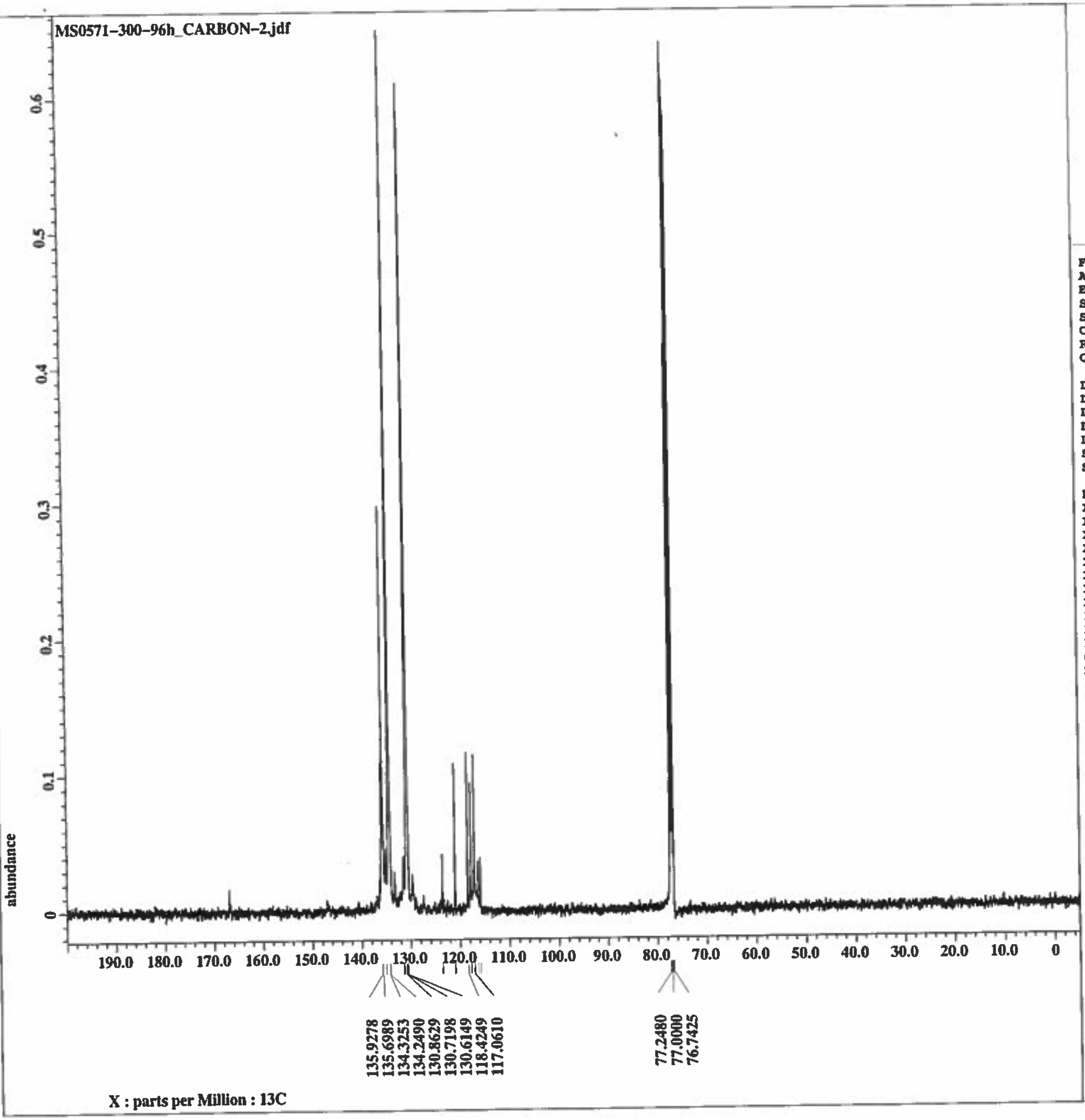

Filename	= MS0571-300-96h_CARBON
Author	= Jim Davis
Experiment	= single_pulse_dec
Sample_id	= MS0571-300-96h
Solvent	= CHLOROFORM-D
Creation_time	= 9-OCT-2018 18:50:52
Revision_time	= 9-OCT-2018 18:25:22
Current_time	= 9-OCT-2018 18:25:22
Data_format	= 1D COMPLEX
Dim_size	= 26214
Dim_title	= 13C
Dim_units	= [ppm]
Dimensions	= X
Site	= ECA 500
Spectrometer	= JNM-ECA500
Field_strength	= 11.7473579[T] (500[MH
X_acq_duration	= 0.83361792[s]
X_domain	= 13C
X_freq	= 125.76529768[MHz]
X_offset	= 100[ppm]
X_points	= 32768
X_prescans	= 4
X_resolution	= 1.19959034[Hz]
X_sweep	= 39.3081761[kHz]
Irr_domain	= 1H
Irr_freq	= 500.15991521[MHz]
Irr_offset	= 5.0[ppm]
Clipped	= FALSE
Mod_return	= 1
Scans	= 1024
Total_scans	= 1024
X_90_width	= 13.2[us]
X_acq_time	= 0.83361792[s]
X_angle	= 30[deg]
X_atn	= 6[dB]
X_pulse	= 4.4[us]
Irr_atn_dec	= 20.7[dB]
Irr_atn_noe	= 20.7[dB]
Irr_noise	= WALTZ
Decoupling	= TRUE
Initial_wait	= 1[s]
Noe	= TRUE
Noe_time	= 2[s]
Recvr_gain	= 60
Relaxation_delay	= 2[s]
Repetition_time	= 2.83361792[s]
Temp_get	= 23.4[dC]

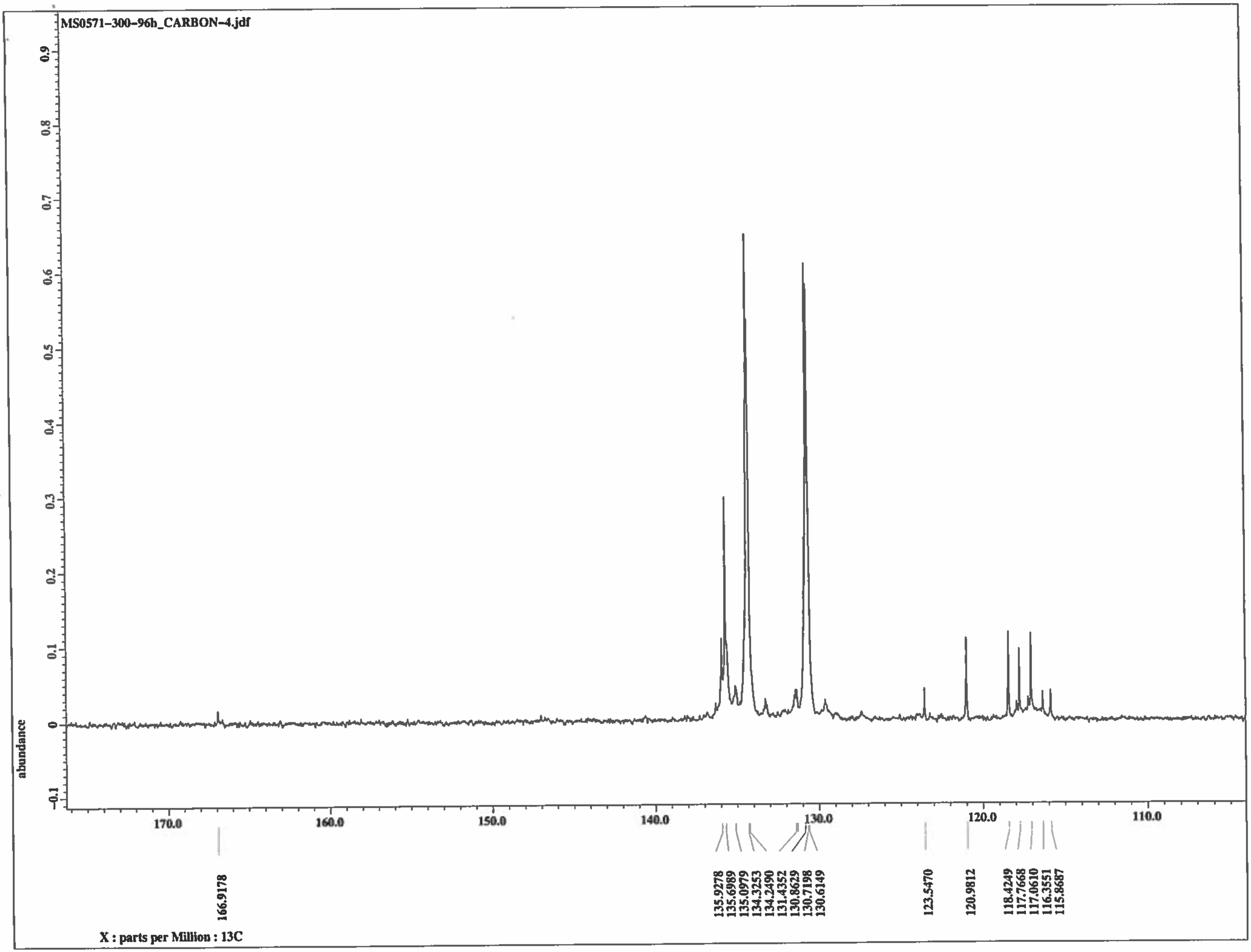
MS0571-300-96h_CARBON-4.jdf
abundance
0.9
0.8
0.7
0.6
0.5
0.4
0.3
0.2
0.1
0
-0.1
170.0
160.0
150.0
140.0
130.0
120.0
110.0
166.9178
135.9278
135.6989
135.0979
134.3253
134.2490
131.4352
130.8629
130.7198
130.6149
123.5470
120.9812
118.4249
117.7668
117.0610
116.3551
115.8687
X : parts per Million : 13C

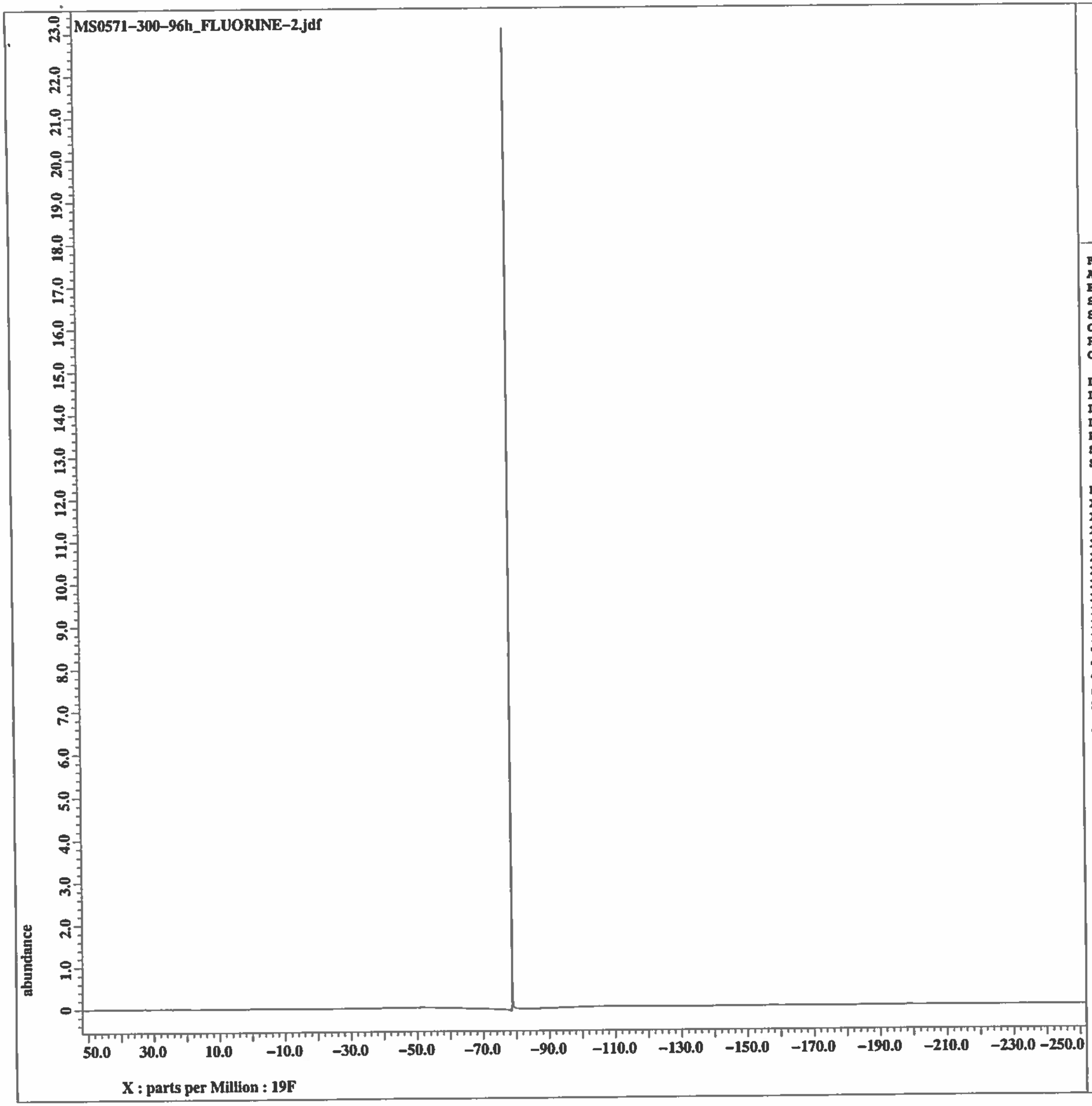

Filename	= MS0571-300-96h_FLUORI
Author	= Jim Davis
Experiment	= single_pulse.ex2
Sample_id	= MS0571-300-96h
Solvent	= CHLOROFORM-D
Creation_time	= 9-OCT-2018 18:55:50
Revision_time	= 9-OCT-2018 18:30:20
Current_time	= 9-OCT-2018 18:30:20
Data_format	= 1D COMPLEX
Dim_size	= 104857
Dim_title	= 19F
Dim_units	= [ppm]
Dimensions	= X
Site	= ECA 500
Spectrometer	= JNM-ECA500
Field_strength	= 11.7473579[T] (500[MH
X_acq_duration	= 0.7340032[s]
X_domain	= 19F
X_freq	= 470.62046084[MHz]
X_offset	= -100[ppm]
X_points	= 131072
X_prescans	= 1
X_resolution	= 1.36239188[Hz]
X_sweep	= 178.57142857[kHz]
Irr_domain	= 19F
Irr_freq	= 470.62046084[MHz]
Irr_offset	= 5[ppm]
Tri_domain	= 19F
Tri_freq	= 470.62046084[MHz]
Tri_offset	= 5[ppm]
Clipped	= FALSE
Mod_return	= 1
Scans	= 40
Total_scans	= 40
X_90_width	= 13.1[us]
X_acq_time	= 0.7340032[s]
X_angle	= 45[deg]
X_atn	= 2.5[dB]
X_pulse	= 6.55[us]
Irr_mode	= Off
Tri_mode	= Off
Dante_presat	= FALSE
Initial_wait	= 1[s]
Recvr_gain	= 40
Relaxation_delay	= 4[s]
Repetition_time	= 4.7340032[s]
Temp_get	= 23[dC]

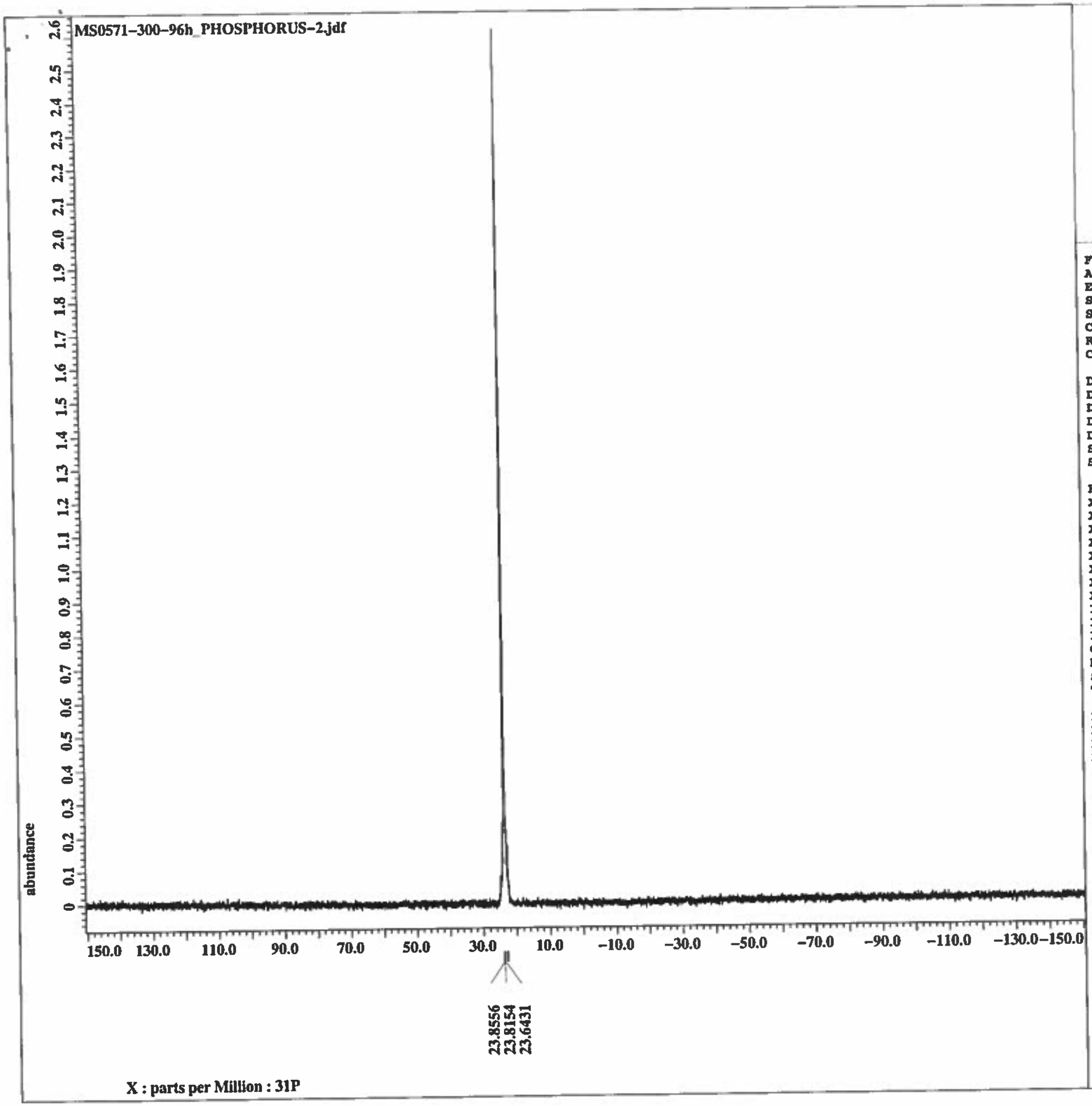

SOUTHALABAMA JAGUARS

Filename	= MS0571-300-96h_PHOSPH
Author	= Jim Davis
Experiment	= single_pulse_dec
Sample_id	= MS0571-300-96h
Solvent	= CHLOROFORM-D
Creation_time	= 9-OCT-2018 19:00:54
Revision_time	= 9-OCT-2018 18:35:25
Current_time	= 9-OCT-2018 18:35:25
Data_format	= 1D COMPLEX
Dim_size	= 52428
Dim_title	= 31P
Dim_units	= [ppm]
Dimensions	= X
Site	= ECA 500
Spectrometer	= JNM-ECA500
Field_strength	= 11.7473579[T] (500[MH
X_acq_duration	= 0.85983232[s]
X_domain	= 31P
X_freq	= 202.46831075[MHz]
X_offset	= 0[ppm]
X_points	= 65536
X_prescans	= 4
X_resolution	= 1.16301746[Hz]
X_sweep	= 76.2195122[kHz]
Irr_domain	= 1H
Irr_freq	= 500.15991521[MHz]
Irr_offset	= 5.0[ppm]
Clipped	= FALSE
Mod_return	= 1
Scans	= 50
Total_scans	= 50
X_90_width	= 14.687[us]
X_acq_time	= 0.85983232[s]
X_angle	= 30[deg]
X_atn	= 5[dB]
X_pulse	= 4.89566667[us]
Irr_atn_dec	= 20.7[dB]
Irr_atn_noe	= 20.7[dB]
Irr_noise	= WALTZ
Decoupling	= TRUE
Initial_wait	= 1[s]
Noe	= TRUE
Noe_time	= 2[s]
Recvr_gain	= 58
Relaxation_delay	= 2[s]
Repetition_time	= 2.85983232[s]
Temp_get	= 23.3[dC]

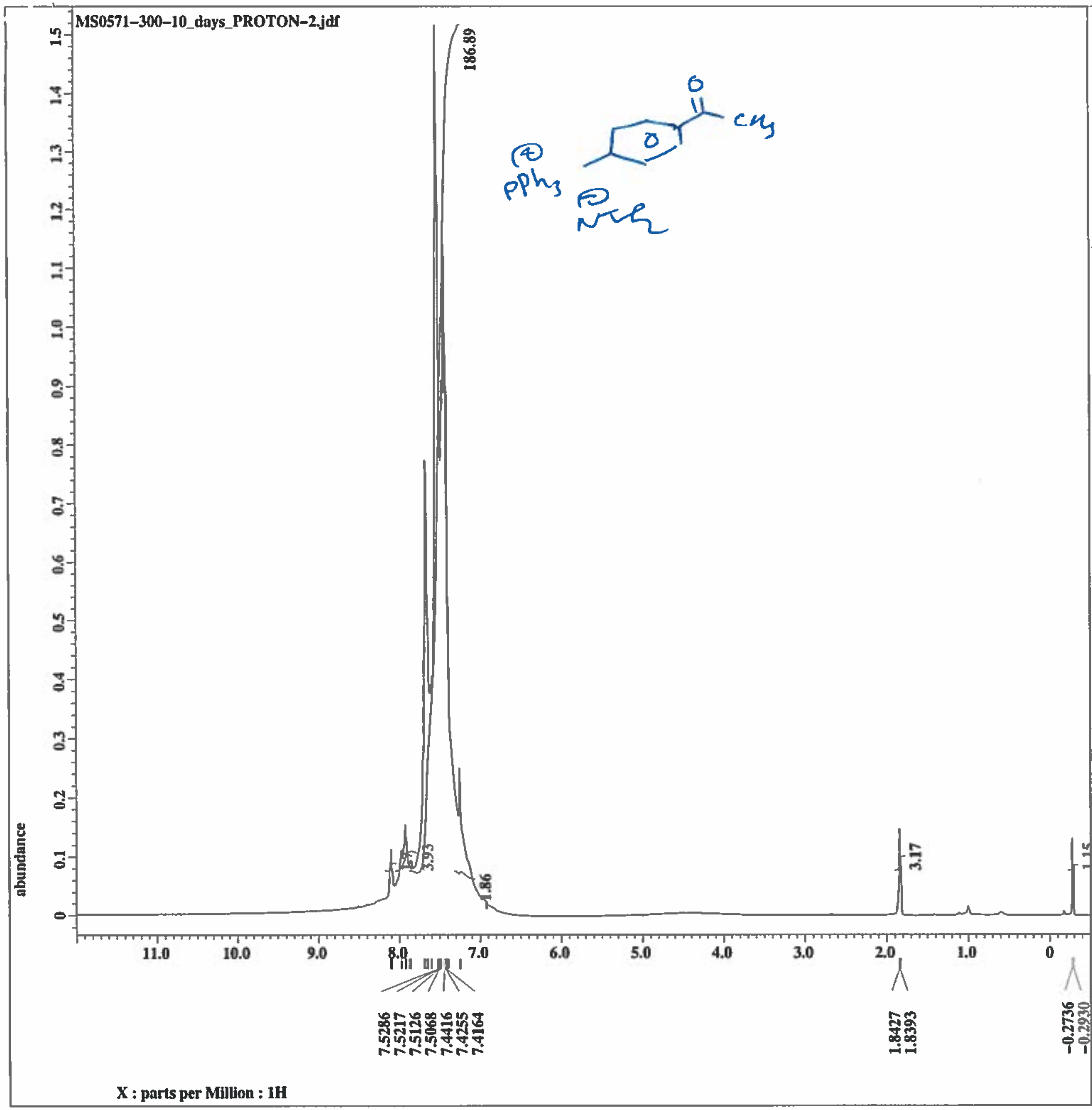

Filename	= MS0571-300-10_days_PR
Author	= Jim Davis
Experiment	= single_pulse.ex2
Sample_id	= MS0571-300-10_days
Solvent	= CHLOROFORM-D
Creation_time	= 19-OCT-2018 10:46:06
Revision_time	= 19-OCT-2018 10:19:50
Current_time	= 19-OCT-2018 10:19:50
Data_format	= 1D COMPLEX
Dim_size	= 13107
Dim_title	= 1H
Dim_units	= [ppm]
Dimensions	= X
Site	= ECA 500
Spectrometer	= JNM-ECA500
Field_strength	= 11.7473579[T] (500[MH
X_acq_duration	= 1.74587904[s]
X_domain	= 1H
X_freq	= 500.15991521[MHz]
X_offset	= 5.0[ppm]
X_points	= 16384
X_prescans	= 1
X_resolution	= 0.57277737[Hz]
X_sweep	= 9.38438438[kHz]
Irr_domain	= 1H
Irr_freq	= 500.15991521[MHz]
Irr_offset	= 5.0[ppm]
Tri_domain	= 1H
Tri_freq	= 500.15991521[MHz]
Tri_offset	= 5.0[ppm]
Clipped	= FALSE
Mod_return	= 1
Scans	= 16
Total_scans	= 16
X_90_width	= 12.4[us]
X_acq_time	= 1.74587904[s]
X_angle	= 45[deg]
X_atn	= 4[dB]
X_pulse	= 6.2[us]
Irr_mode	= Off
Tri_mode	= Off
Dante_presat	= FALSE
Initial_wait	= 1[s]
Recvr_gain	= 32
Relaxation_delay	= 4[s]
Repetition_time	= 5.74587904[s]
Temp_get	= 22.3[dC]

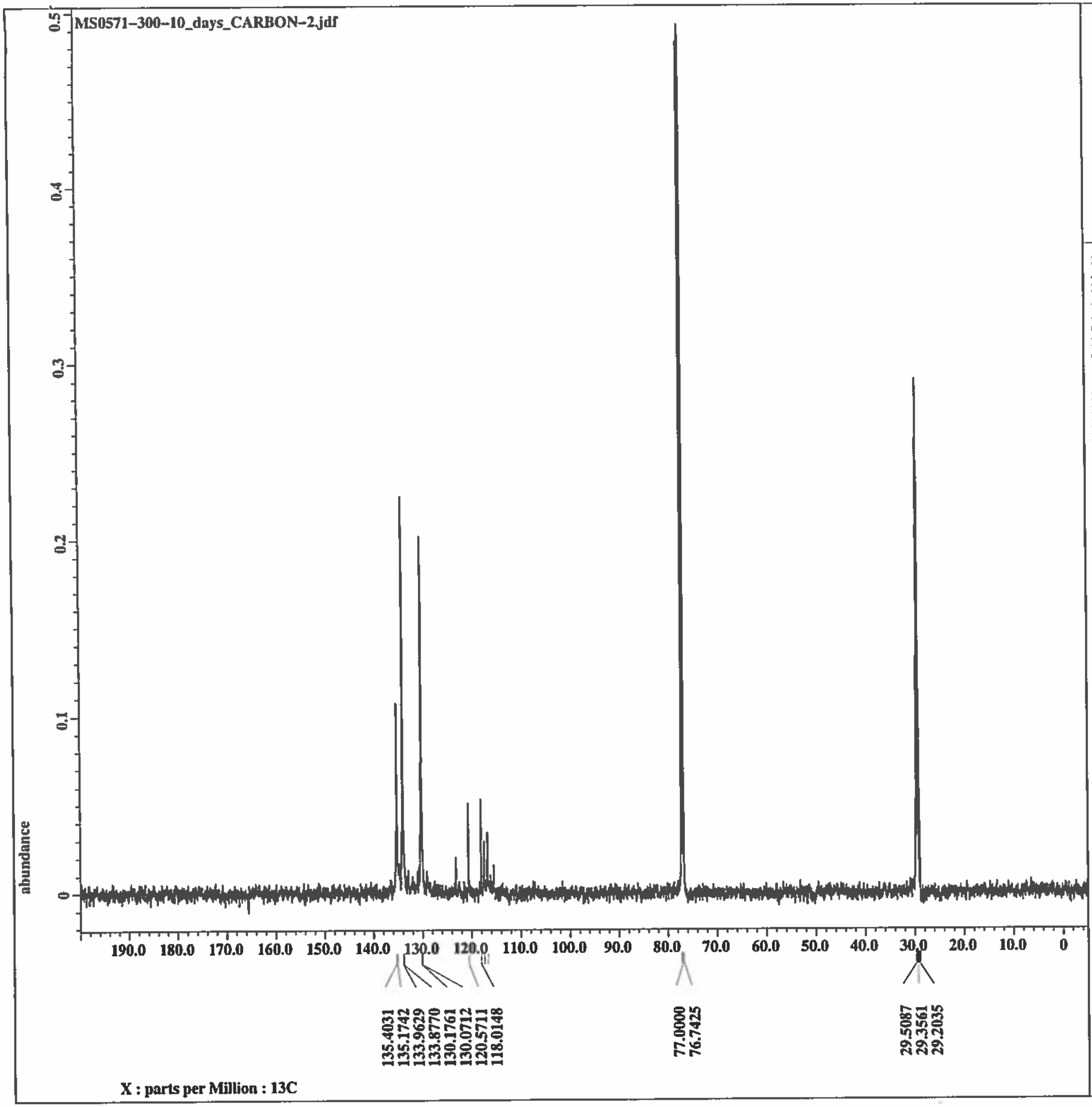

SOUTH ALABAMA JAGUARS

Filename	= MS0571-300-10_days_CA
Author	= Jim Davis
Experiment	= single_pulse_dec
Sample_id	= MS0571-300-10_days
Solvent	= CHLOROFORM-D
Creation_time	= 19-OCT-2018 11:05:28
Revision_time	= 19-OCT-2018 10:39:14
Current_time	= 19-OCT-2018 10:39:14
Data_format	= 1D COMPLEX
Dim_size	= 26214
Dim_title	= 13C
Dim_units	= [ppm]
Dimensions	= X
Site	= ECA 500
Spectrometer	= JNM-ECA500
Field_strength	= 11.7473579[T] (500[MH
X_acq_duration	= 0.83361792[s]
X_domain	= 13C
X_freq	= 125.76529768[MHz]
X_offset	= 100[ppm]
X_points	= 32768
X_prescans	= 4
X_resolution	= 1.19959034[Hz]
X_sweep	= 39.3081761[kHz]
Irr_domain	= 1H
Irr_freq	= 500.15991521[MHz]
Irr_offset	= 5.0[ppm]
Clipped	= FALSE
Mod_return	= 1
Scans	= 400
Total_scans	= 400
X_90_width	= 13.2[us]
X_acq_time	= 0.83361792[s]
X_angle	= 30[deg]
X_atn	= 6[dB]
X_pulse	= 4.4[us]
Irr_atn_dec	= 20.7[dB]
Irr_atn_noe	= 20.7[dB]
Irr_noise	= WALTZ
Decoupling	= TRUE
Initial_wait	= 1[s]
Noe	= TRUE
Noe_time	= 2[s]
Recvr_gain	= 60
Relaxation_delay	= 2[s]
Repetition_time	= 2.83361792[s]
Temp_get	= 22.9[dC]

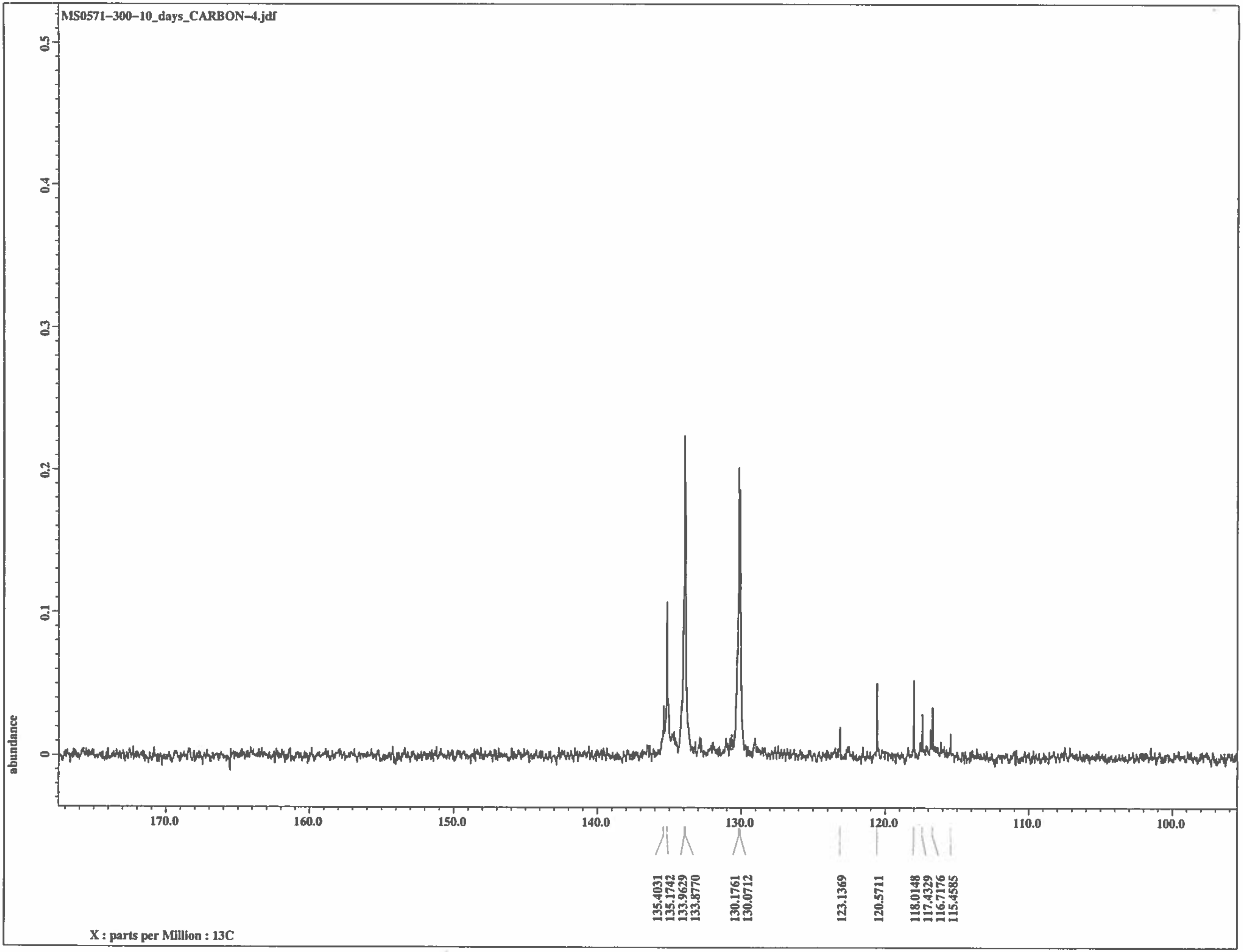
MS0571-300-10_days_CARBON-4.jdf
abundance
0
0.1
0.2
0.3
0.4
0.5
170.0
160.0
150.0
140.0
130.0
120.0
110.0
100.0
135.4031
135.1742
133.9629
133.8770
130.1761
130.0712
123.1369
120.5711
118.0148
117.4329
116.7176
115.4585
X : parts per Million : 13C

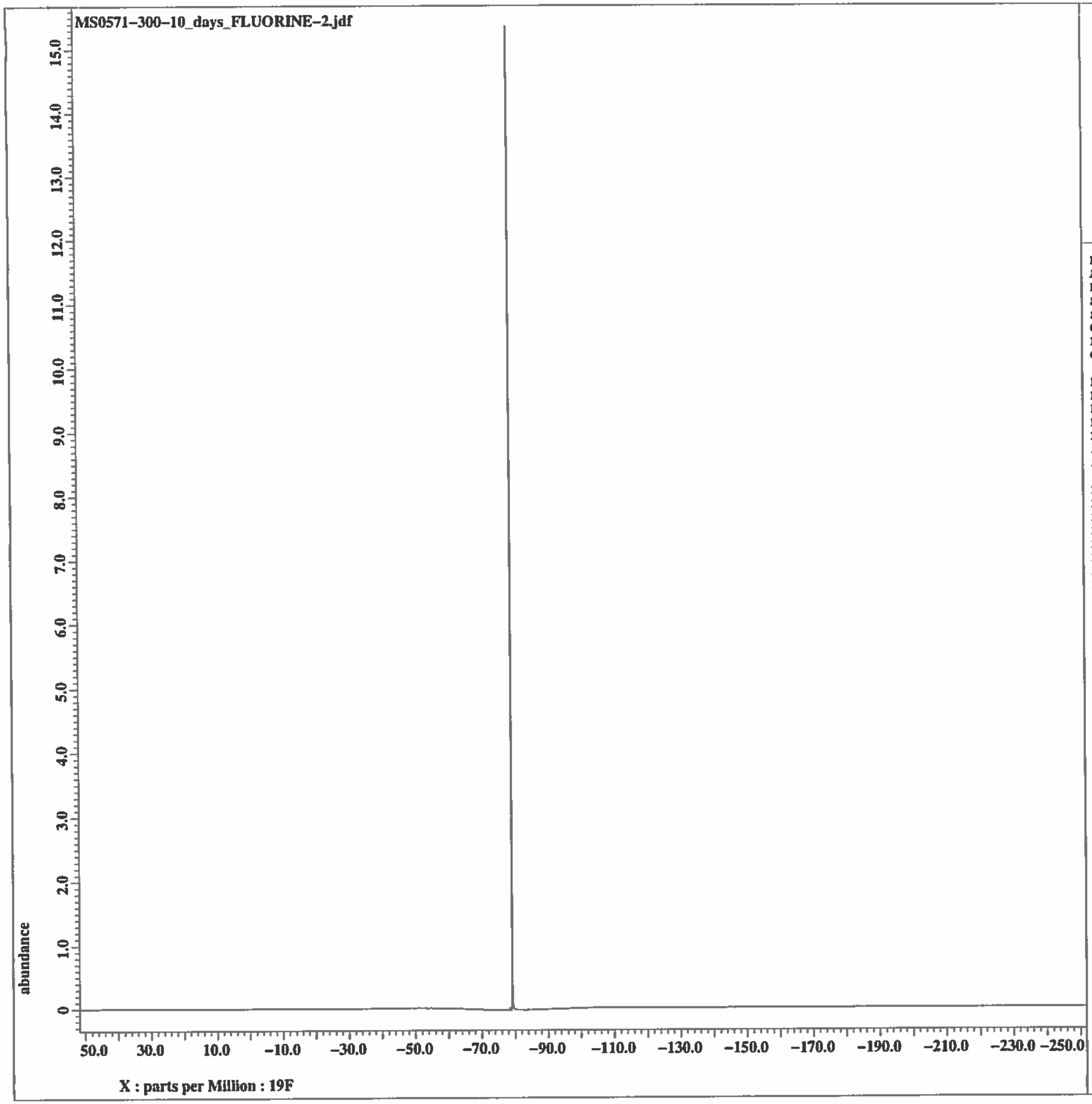

Filename	= MS0571-300-10_days_FL
Author	= Jim Davis
Experiment	= single_pulse.ex2
Sample_id	= MS0571-300-10_days
Solvent	= CHLOROFORM-D
Creation_time	= 19-OCT-2018 11:10:46
Revision_time	= 19-OCT-2018 10:44:32
Current_time	= 19-OCT-2018 10:44:32
Data_format	= 1D COMPLEX
Dim_size	= 104857
Dim_title	= 19F
Dim_units	= [ppm]
Dimensions	= X
Site	= ECA 500
Spectrometer	= JNM-ECA500
Field_strength	= 11.7473579[T] (500[MH
X_acq_duration	= 0.7340032[s]
X_domain	= 19F
X_freq	= 470.62046084[MHz]
X_offset	= -100[ppm]
X_points	= 131072
X_prescans	= 1
X_resolution	= 1.36239188[Hz]
X_sweep	= 178.57142857[kHz]
Irr_domain	= 19F
Irr_freq	= 470.62046084[MHz]
Irr_offset	= 5[ppm]
Tri_domain	= 19F
Tri_freq	= 470.62046084[MHz]
Tri_offset	= 5[ppm]
Clipped	= FALSE
Mod_return	= 1
Scans	= 40
Total_scans	= 40
X_90_width	= 13.1[us]
X_acq_time	= 0.7340032[s]
X_angle	= 45[deg]
X_atn	= 2.5[dB]
X_pulse	= 6.55[us]
Irr_mode	= Off
Tri_mode	= Off
Dante_presat	= FALSE
Initial_wait	= 1[s]
Recvr_gain	= 62
Relaxation_delay	= 4[s]
Repetition_time	= 4.7340032[s]
Temp_get	= 22.5[dC]

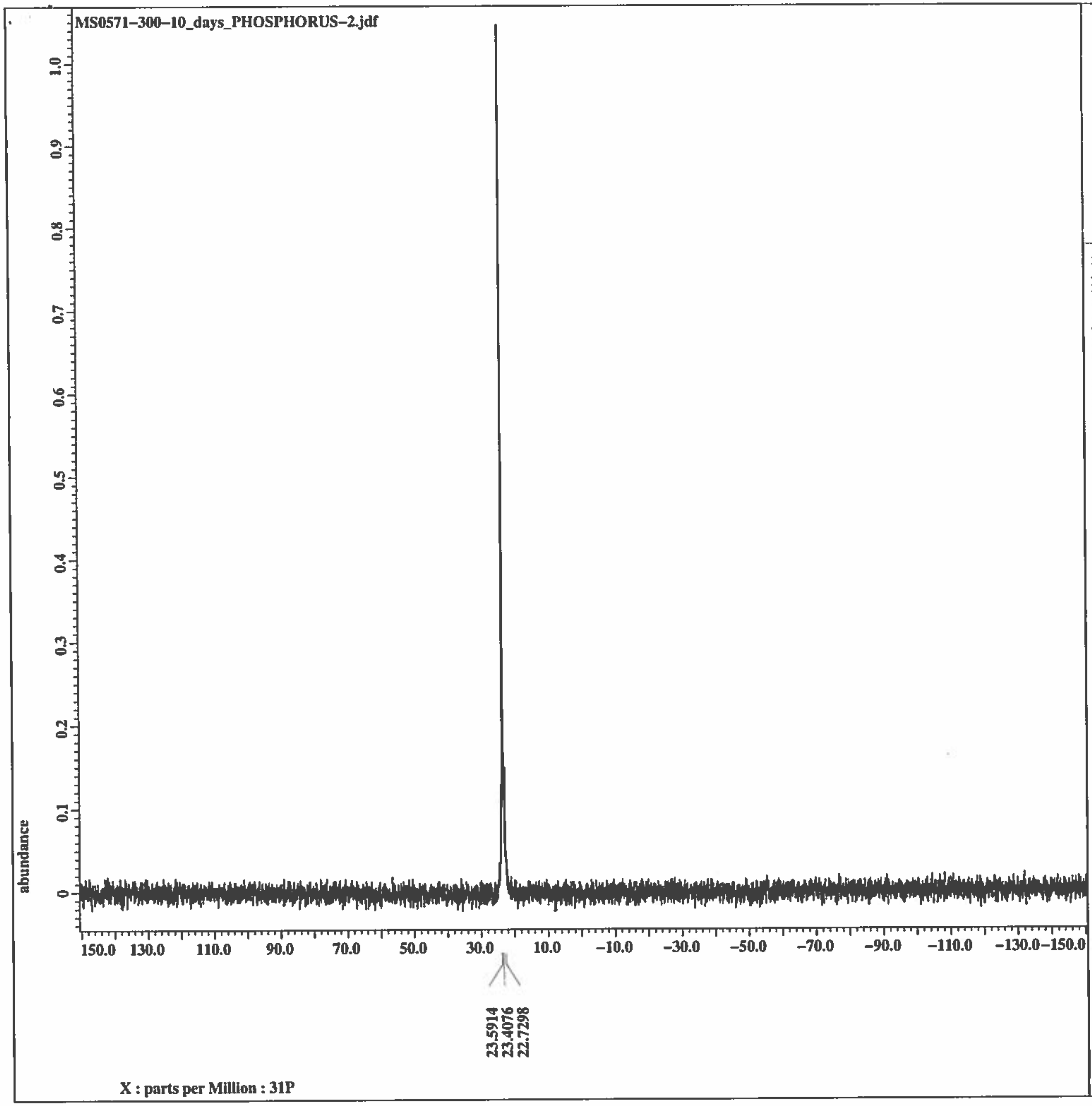

Filename	= MS0571-300-10_days_PH
Author	= Jim Davis
Experiment	= single_pulse_dec
Sample_id	= MS0571-300-10_days
Solvent	= CHLOROFORM-D
Creation_time	= 19-OCT-2018 11:15:16
Revision_time	= 19-OCT-2018 10:49:02
Current_time	= 19-OCT-2018 10:49:02
Data_format	= 1D COMPLEX
Dim_size	= 52428
Dim_title	= 31P
Dim_units	= [ppm]
Dimensions	= X
Site	= ECA 500
Spectrometer	= JNM-ECA500
Field_strength	= 11.7473579[T] (500[MH
X_acq_duration	= 0.85983232[s]
X_domain	= 31P
X_freq	= 202.46831075[MHz]
X_offset	= 0[ppm]
X_points	= 65536
X_prescans	= 4
X_resolution	= 1.16301746[Hz]
X_sweep	= 76.2195122[kHz]
Irr_domain	= 1H
Irr_freq	= 500.15991521[MHz]
Irr_offset	= 5.0[ppm]
Clipped	= FALSE
Mod_return	= 1
Scans	= 40
Total_scans	= 40
X_90_width	= 14.687[us]
X_acq_time	= 0.85983232[s]
X_angle	= 30[deg]
X_atn	= 5[dB]
X_pulse	= 4.89566667[us]
Irr_atn_dec	= 20.7[dB]
Irr_atn_noe	= 20.7[dB]
Irr_noise	= WALTZ
Decoupling	= TRUE
Initial_wait	= 1[s]
Noe	= TRUE
Noe_time	= 2[s]
Recvr_gain	= 58
Relaxation_delay	= 2[s]
Repetition_time	= 2.85983232[s]
Temp_get	= 22.8[dC]

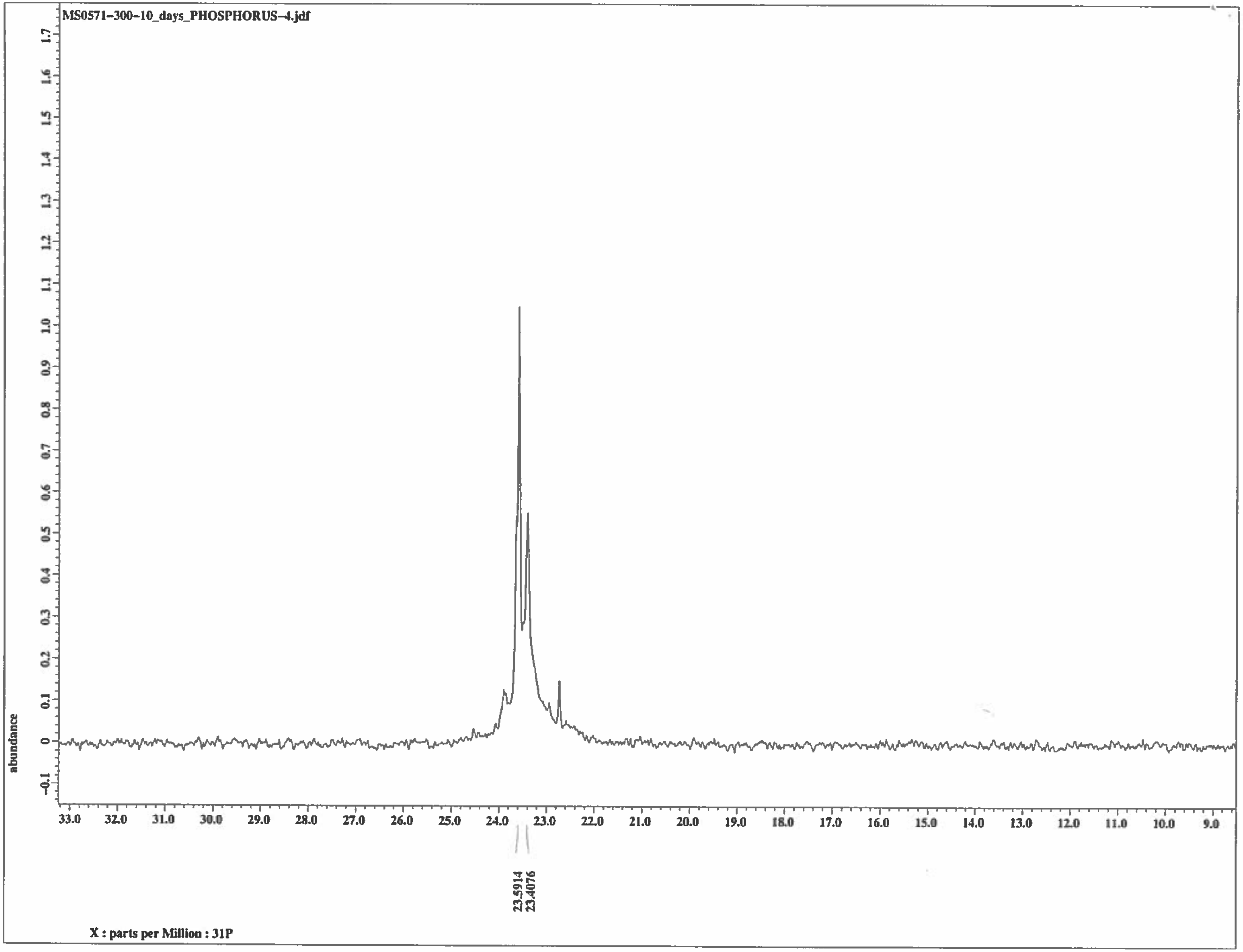
MS0571-300-10_days_PHOSPHORUS-4.jdf
abundance
23.5914
23.4076
X : parts per Million : 31P

Compound 17 Pre- and Post-heating NMR Spectra

Temperature of Post-heating samples noted in upper left corner of each spectrum

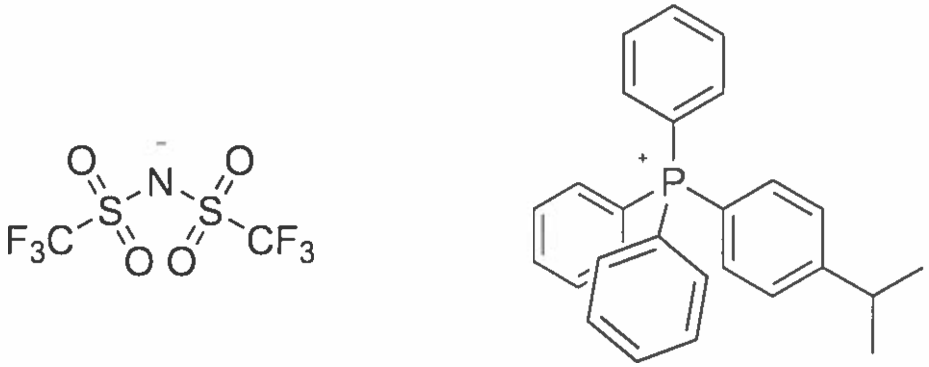

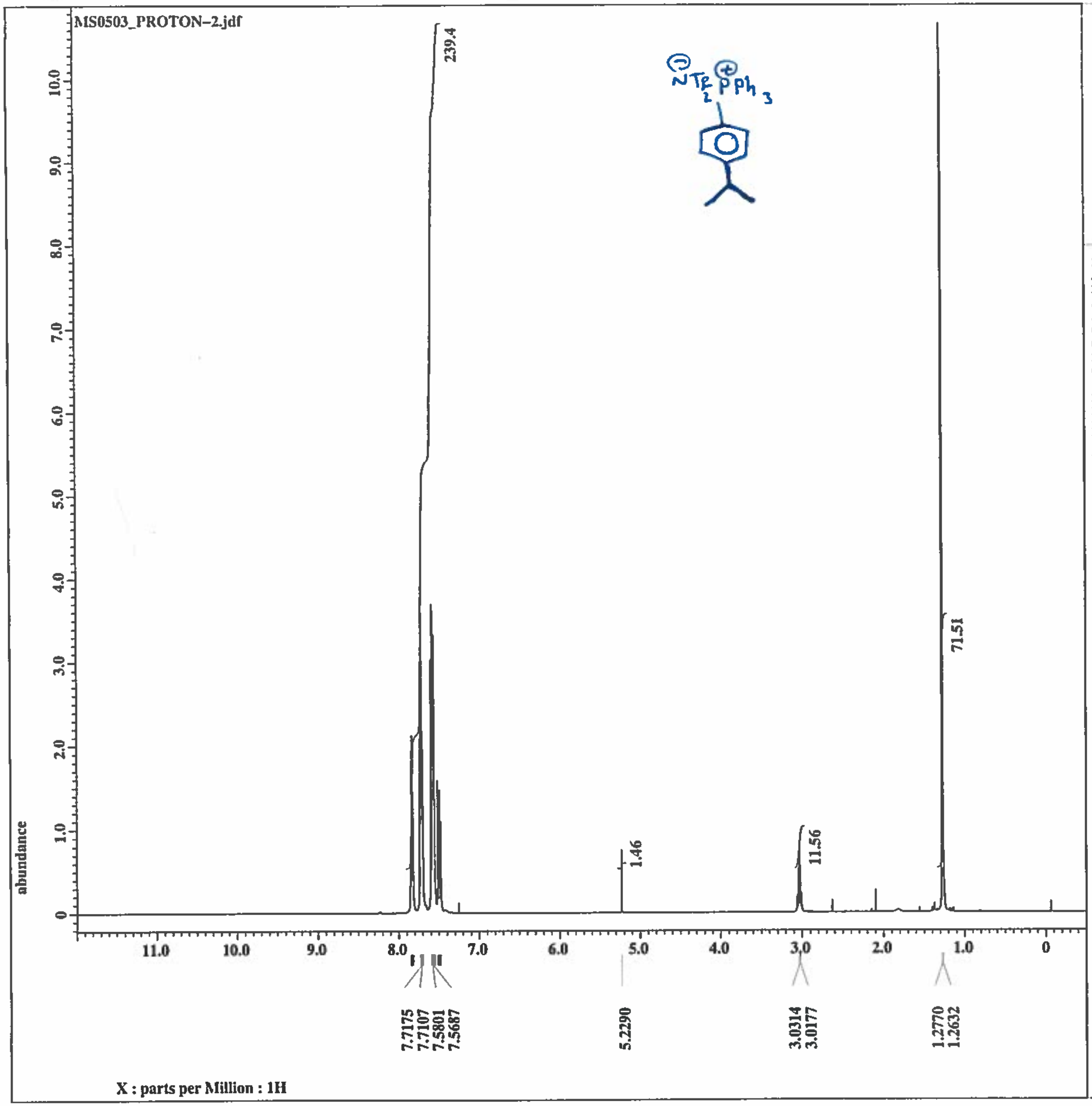

Filename	= MS0503_PROTON-2.jdf
Author	= Jim Davis
Experiment	= single_pulse.ex2
Sample_id	= MS0503
Solvent	= CHLOROFORM-D
Changer_sample	= 1
Creation_time	= 6-JUL-2018 16:26:04
Revision_time	= 6-JUL-2018 16:02:29
Current_time	= 6-JUL-2018 16:02:29
Data_format	= 1D COMPLEX
Dim_size	= 13107
Dim_title	= 1H
Dim_units	= [ppm]
Dimensions	= X
Site	= ECA 500
Spectrometer	= JNM-ECA500
Field_strength	= 11.7473579[T] (500[MH
X_acq_duration	= 1.74587904[s]
X_domain	= 1H
X_freq	= 500.15991521[MHz]
X_offset	= 5.0[ppm]
X_points	= 16384
X_prescans	= 1
X_resolution	= 0.57277737[Hz]
X_sweep	= 9.38438438[kHz]
Irr_domain	= 1H
Irr_freq	= 500.15991521[MHz]
Irr_offset	= 5.0[ppm]
Tri_domain	= 1H
Tri_freq	= 500.15991521[MHz]
Tri_offset	= 5.0[ppm]
Clipped	= FALSE
Mod_return	= 1
Scans	= 16
Total_scans	= 16
X_90_width	= 12.4[us]
X_acq_time	= 1.74587904[s]
X_angle	= 45[deg]
X_atn	= 4[dB]
X_pulse	= 6.2[us]
Irr_mode	= Off
Tri_mode	= Off
Dante_presat	= FALSE
Initial_wait	= 1[s]
Recvr_gain	= 22
Relaxation_delay	= 4[s]
Repetition_time	= 5.74587904[s]
Temp_get	= 22.6[dC]

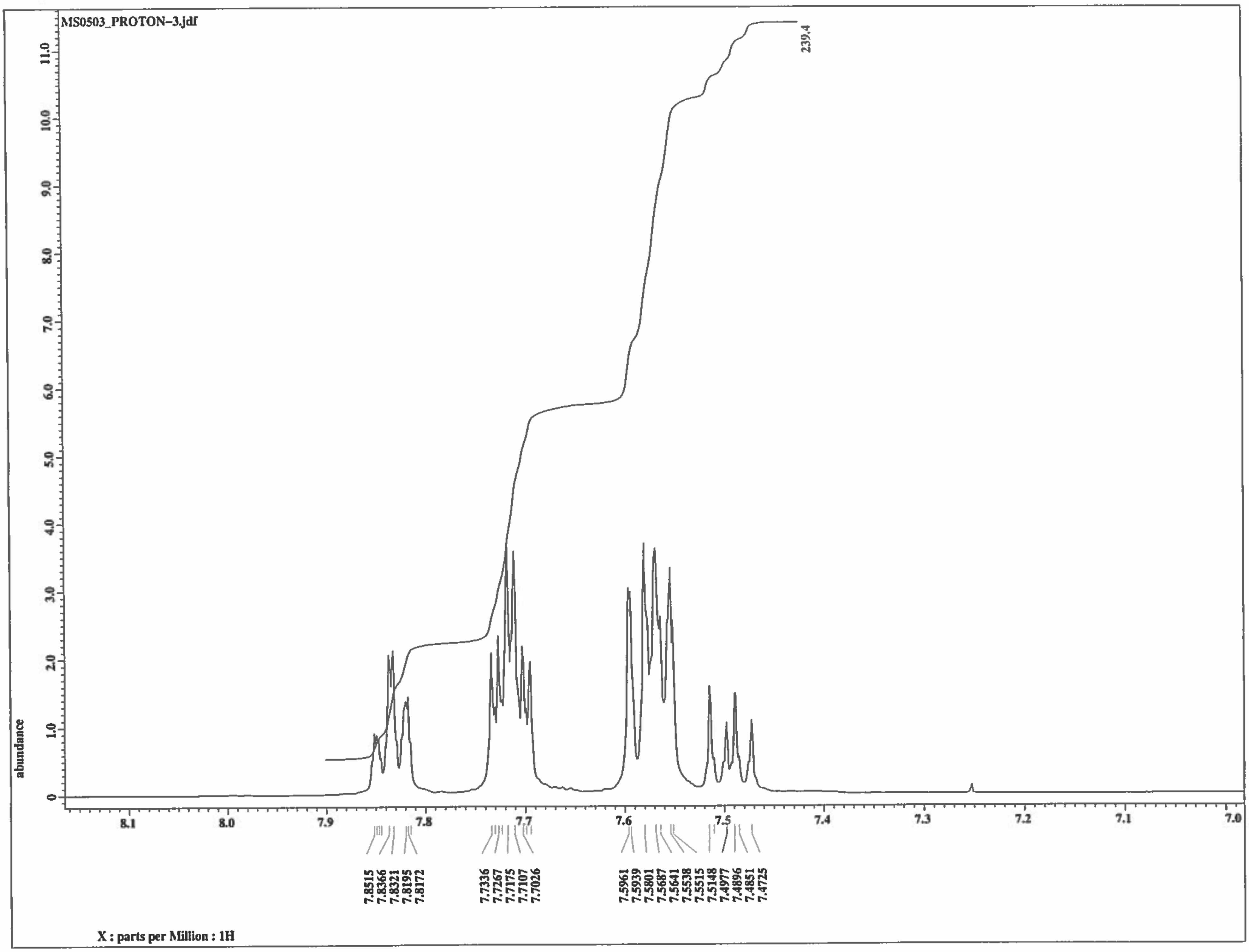
MS0503_PROTON-3.jdf
abundance
0
1.0
2.0
3.0
4.0
5.0
6.0
7.0
8.0
9.0
10.0
11.0
239.4
8.1
8.0
7.9
7.8
7.7
7.6
7.5
7.4
7.3
7.2
7.1
7.0
7.8515
7.8366
7.8321
7.8195
7.8172
7.7336
7.7267
7.7175
7.7107
7.7026
7.5961
7.5939
7.5801
7.5687
7.5641
7.5538
7.5515
7.5148
7.4977
7.4896
7.4851
7.4725
X : parts per Million : 1H

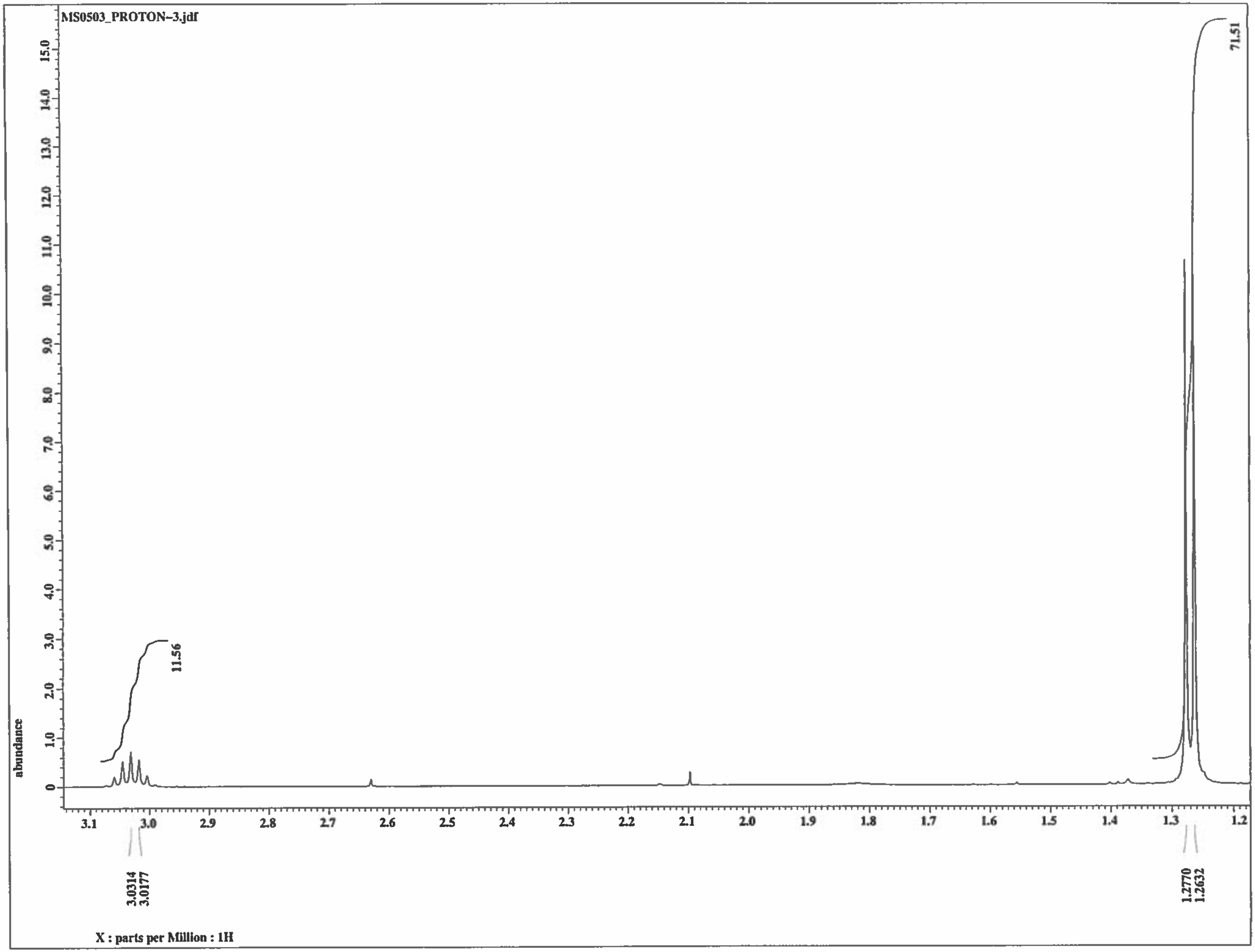
MS0503_PROTON-3.jdf
abundance
0
1.0
2.0
3.0
4.0
5.0
6.0
7.0
8.0
9.0
10.0
11.0
12.0
13.0
14.0
15.0
11.56
71.51
3.1
3.0
2.9
2.8
2.7
2.6
2.5
2.4
2.3
2.2
2.1
2.0
1.9
1.8
1.7
1.6
1.5
1.4
1.3
1.2
3.0314
3.0177
1.2770
1.2632
X : parts per Million : 1H

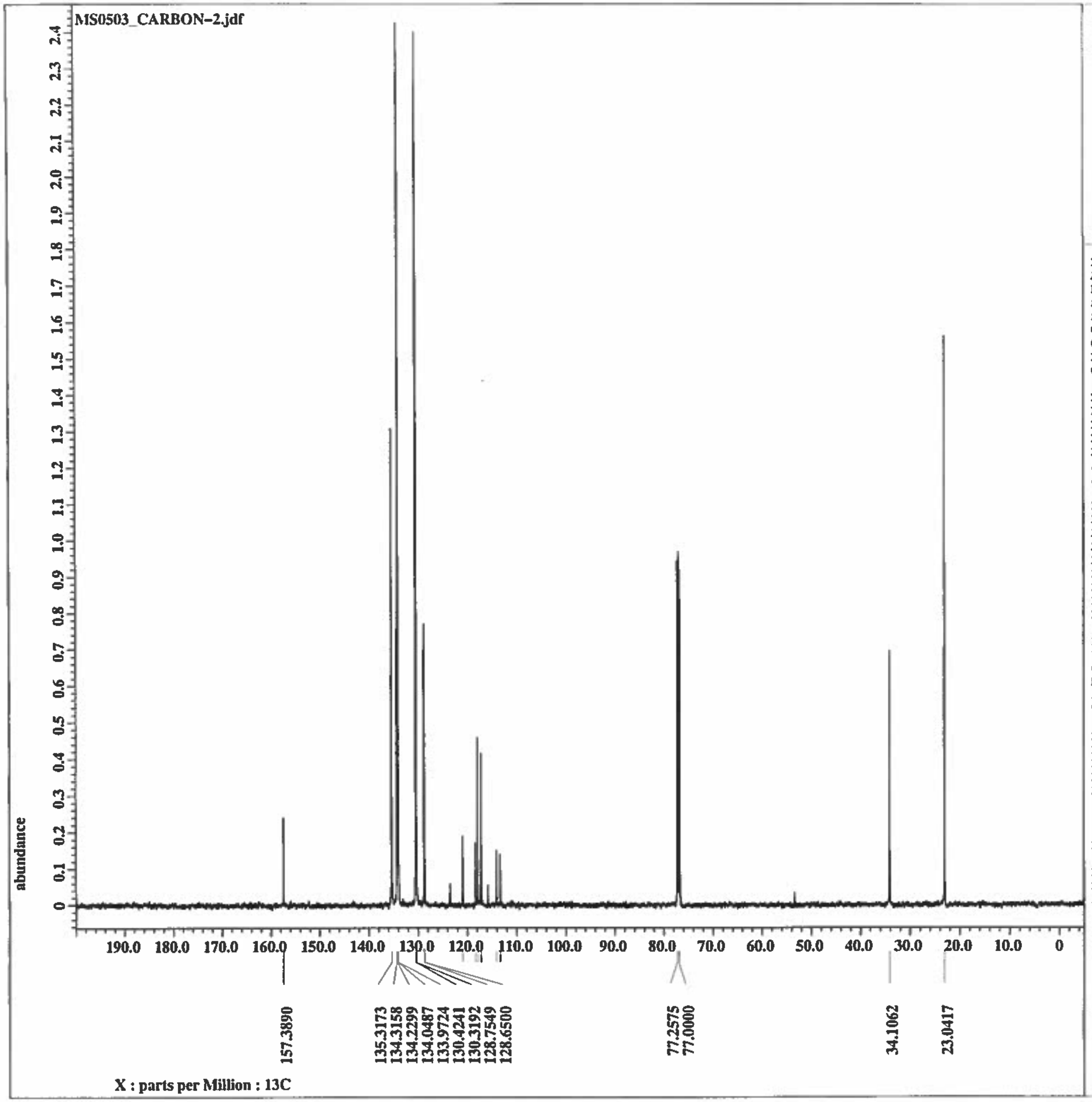

Filename	= MS0503_CARBON-2.jdf
Author	= Jim Davis
Experiment	= single_pulse_dec
Sample_id	= MS0503
Solvent	= CHLOROFORM-D
Changer_sample	= 1
Creation_time	= 6-JUL-2018 16:39:26
Revision_time	= 6-JUL-2018 16:15:53
Current_time	= 6-JUL-2018 16:15:53
Data_format	= 1D COMPLEX
Dim_size	= 26214
Dim_title	= 13C
Dim_units	= [ppm]
Dimensions	= X
Site	= ECA 500
Spectrometer	= JNM-ECA500
Field_strength	= 11.7473579[T] (500[MH
X_acq_duration	= 0.83361792[s]
X_domain	= 13C
X_freq	= 125.76529768[MHz]
X_offset	= 100[ppm]
X_points	= 32768
X_prescans	= 4
X_resolution	= 1.19959034[Hz]
X_sweep	= 39.3081761[kHz]
Irr_domain	= 1H
Irr_freq	= 500.15991521[MHz]
Irr_offset	= 5.0[ppm]
Clipped	= FALSE
Mod_return	= 1
Scans	= 256
Total_scans	= 256
X_90_width	= 13.2[us]
X_acq_time	= 0.83361792[s]
X_angle	= 30[deg]
X_atn	= 6[dB]
X_pulse	= 4.4[us]
Irr_atn_dec	= 20.7[dB]
Irr_atn_noe	= 20.7[dB]
Irr_noise	= WALTZ
Decoupling	= TRUE
Initial_wait	= 1[s]
Noe	= TRUE
Noe_time	= 2[s]
Recvr_gain	= 60
Relaxation_delay	= 2[s]
Repetition_time	= 2.83361792[s]
Temp_get	= 23[dC]

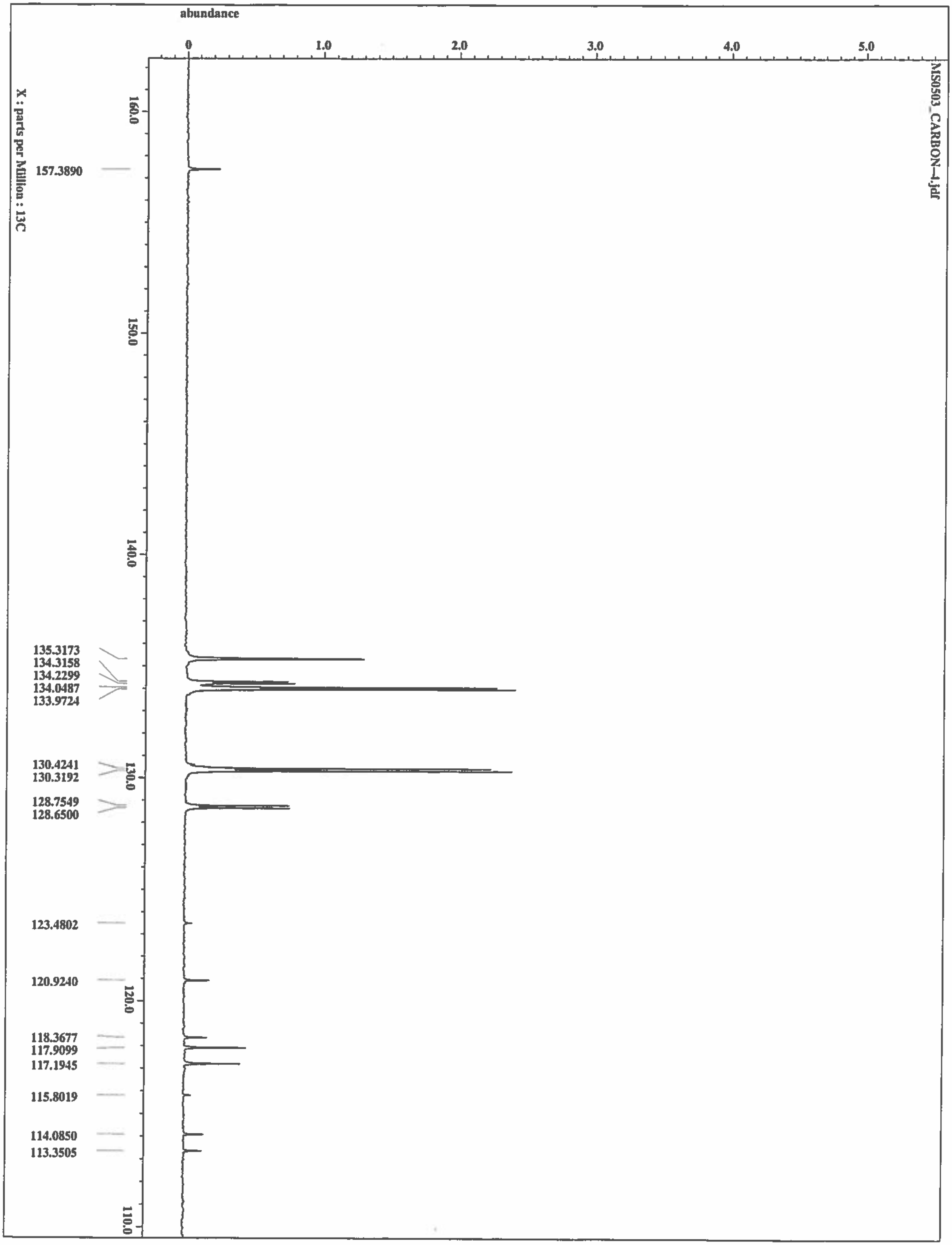

abundance
0
1.0
2.0
3.0
4.0
5.0
NS0503_CARBON-4.jdf
X : parts per Million : 13C
160.0
150.0
140.0
130.0
120.0
110.0
157.3890
135.3173
134.3158
134.2299
134.0487
133.9724
130.4241
130.3192
128.7549
128.6500
123.4802
120.9240
118.3677
117.9099
117.1945
115.8019
114.0850
113.3505

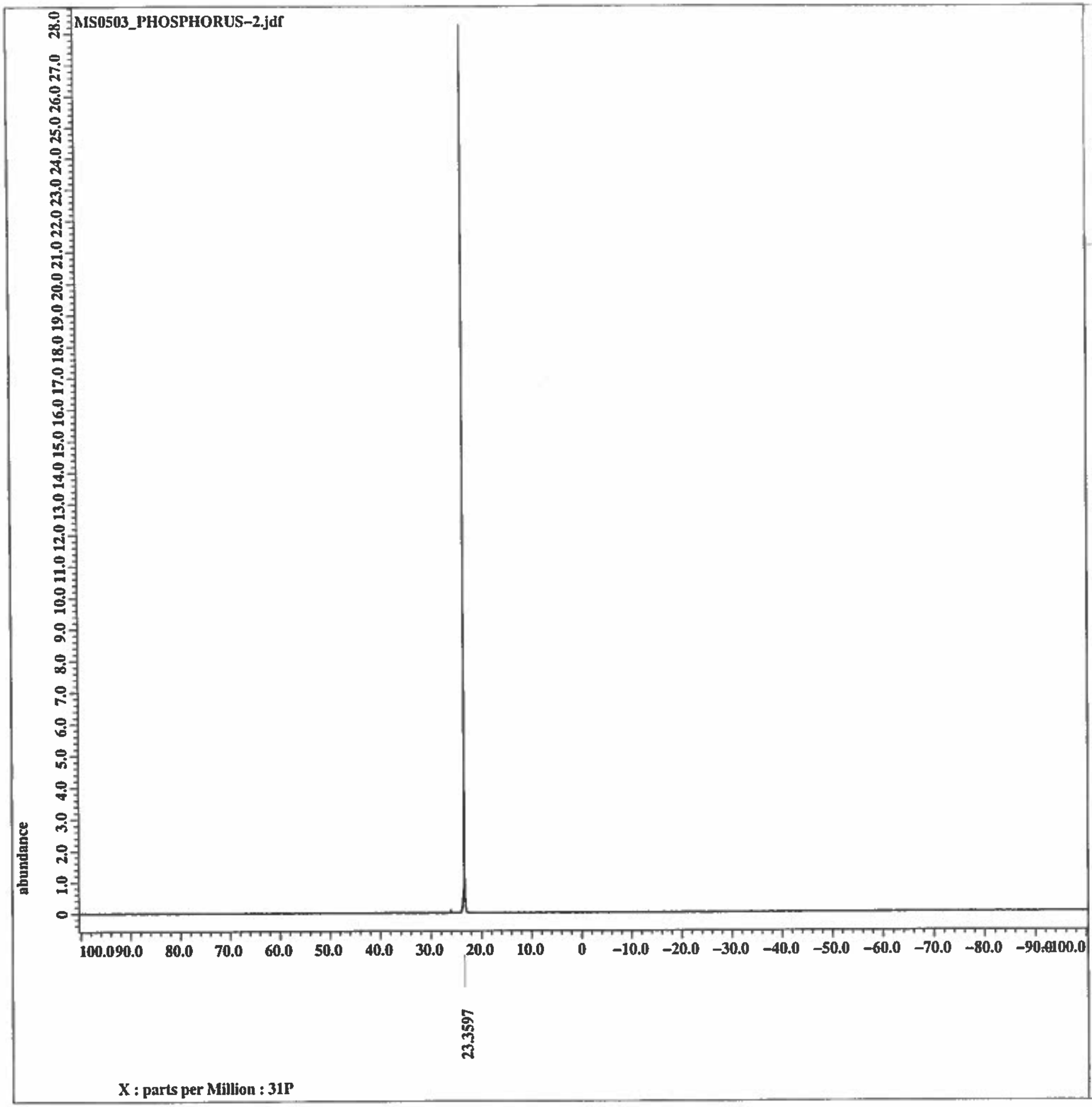

Filename	= MS0503_PHOSPHORUS-2.j
Author	= Jim Davis
Experiment	= single_pulse_dec
Sample_id	= MS0503
Solvent	= CHLOROFORM-D
Changer_sample	= 1
Creation_time	= 6-JUL-2018 16:19:19
Revision_time	= 6-JUL-2018 15:55:44
Current_time	= 6-JUL-2018 15:55:44
Data_format	= 1D COMPLEX
Dim_size	= 26214
Dim_title	= 31P
Dim_units	= [ppm]
Dimensions	= X
Site	= ECA 500
Spectrometer	= JNM-ECA500
Field_strength	= 11.7473579[T] (500[MH
X_acq_duration	= 0.64487424[s]
X_domain	= 31P
X_freq	= 202.46831075[MHz]
X_offset	= 0[ppm]
X_points	= 32768
X_prescans	= 4
X_resolution	= 1.55068995[Hz]
X_sweep	= 50.81300813[kHz]
Irr_domain	= 1H
Irr_freq	= 500.15991521[MHz]
Irr_offset	= 5.0[ppm]
Clipped	= FALSE
Mod_return	= 1
Scans	= 25
Total_scans	= 25
X_90_width	= 14.687[us]
X_acq_time	= 0.64487424[s]
X_angle	= 30[deg]
X_atn	= 5[dB]
X_pulse	= 4.89566667[us]
Irr_atn_dec	= 20.7[dB]
Irr_atn_noe	= 20.7[dB]
Irr_noise	= WALTZ
Decoupling	= TRUE
Initial_wait	= 1[s]
Noe	= TRUE
Noe_time	= 2[s]
Recvr_gain	= 54
Relaxation_delay	= 2[s]
Repetition_time	= 2.64487424[s]
Temp_get	= 22.9[dC]

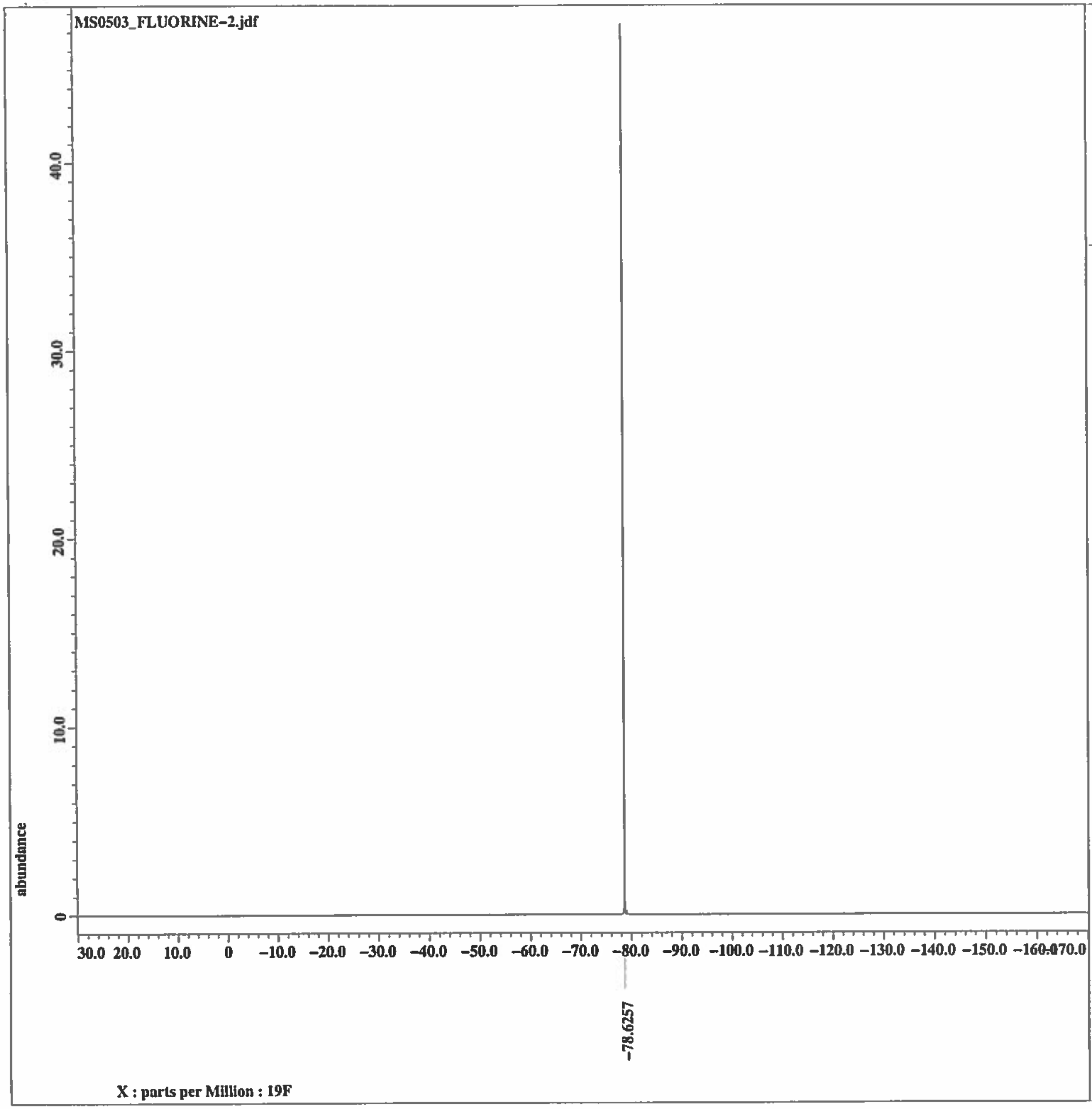

Filename	= MS0503_FLUORINE-2.jdf
Author	= Jim Davis
Experiment	= single_pulse.ex2
Sample_id	= MS0503
Solvent	= CHLOROFORM-D
Changer_sample	= 1
Creation_time	= 6-JUL-2018 16:22:31
Revision_time	= 6-JUL-2018 15:58:58
Current_time	= 6-JUL-2018 15:58:58
Data_format	= 1D COMPLEX
Dim_size	= 52428
Dim_title	= 19F
Dim_units	= [ppm]
Dimensions	= X
Site	= ECA 500
Spectrometer	= JNM-ECA500
Field_strength	= 11.7473579[T] (500[MH
X_acq_duration	= 0.55574528[s]
X_domain	= 19F
X_freq	= 470.62046084[MHz]
X_offset	= -70[ppm]
X_points	= 65536
X_prescans	= 1
X_resolution	= 1.7993855[Hz]
X_sweep	= 117.9245283[kHz]
Irr_domain	= 19F
Irr_freq	= 470.62046084[MHz]
Irr_offset	= 5[ppm]
Tri_domain	= 19F
Tri_freq	= 470.62046084[MHz]
Tri_offset	= 5[ppm]
Clipped	= FALSE
Mod_return	= 1
Scans	= 16
Total_scans	= 16
X_90_width	= 13.1[us]
X_acq_time	= 0.55574528[s]
X_angle	= 45[deg]
X_atn	= 2.5[dB]
X_pulse	= 6.55[us]
Irr_mode	= Off
Tri_mode	= Off
Dante_presat	= FALSE
Initial_wait	= 1[s]
Recvr_gain	= 36
Relaxation_delay	= 4[s]
Repetition_time	= 4.55574528[s]
Temp_get	= 22.7[dC]

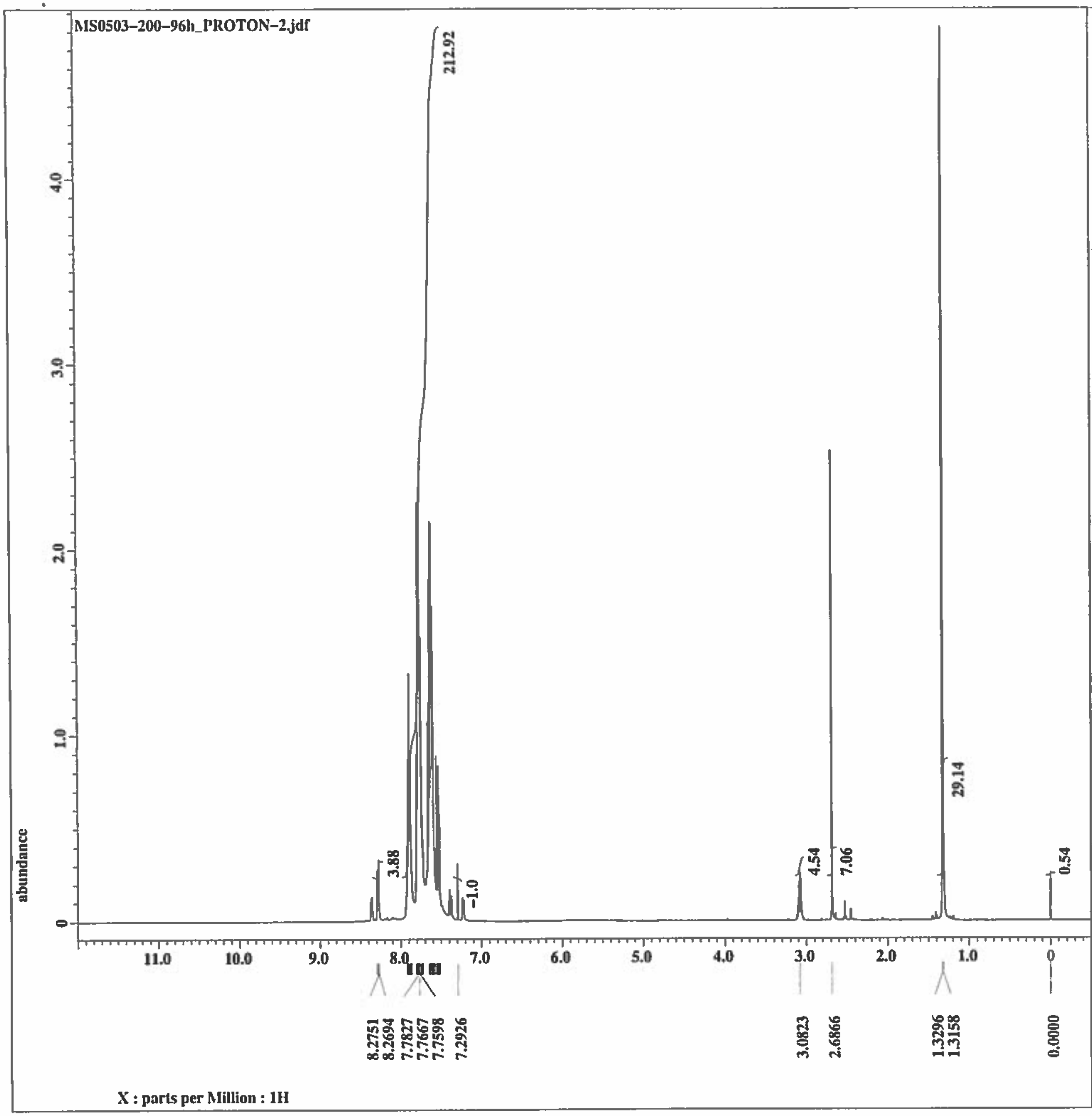

Filename	= MS0503-200-96h_PROTON
Author	= Jim Davis
Experiment	= single_pulse.ex2
Sample_id	= MS0503-200-96h
Solvent	= CHLOROFORM-D
Changer_sample	= 4
Creation_time	= 11-JUL-2018 01:51:20
Revision_time	= 11-JUL-2018 01:29:03
Current_time	= 11-JUL-2018 01:29:05
Data_format	= 1D COMPLEX
Dim_size	= 13107
Dim_title	= 1H
Dim_units	= [ppm]
Dimensions	= X
Site	= ECA 500
Spectrometer	= JNM-ECA500
Field_strength	= 11.7473579[T] (500[MH
X_acq_duration	= 1.74587904[s]
X_domain	= 1H
X_freq	= 500.15991521[MHz]
X_offset	= 5.0[ppm]
X_points	= 16384
X_prescans	= 1
X_resolution	= 0.57277737[Hz]
X_sweep	= 9.38438438[kHz]
Irr_domain	= 1H
Irr_freq	= 500.15991521[MHz]
Irr_offset	= 5.0[ppm]
Tri_domain	= 1H
Tri_freq	= 500.15991521[MHz]
Tri_offset	= 5.0[ppm]
Clipped	= FALSE
Mod_return	= 1
Scans	= 16
Total_scans	= 16
X_90_width	= 12.4[us]
X_acq_time	= 1.74587904[s]
X_angle	= 45[deg]
X_atn	= 4[dB]
X_pulse	= 6.2[us]
Irr_mode	= Off
Tri_mode	= Off
Dante_presat	= FALSE
Initial_wait	= 1[s]
Recvr_gain	= 24
Relaxation_delay	= 4[s]
Repetition_time	= 5.74587904[s]
Temp_get	= 22.2[dC]

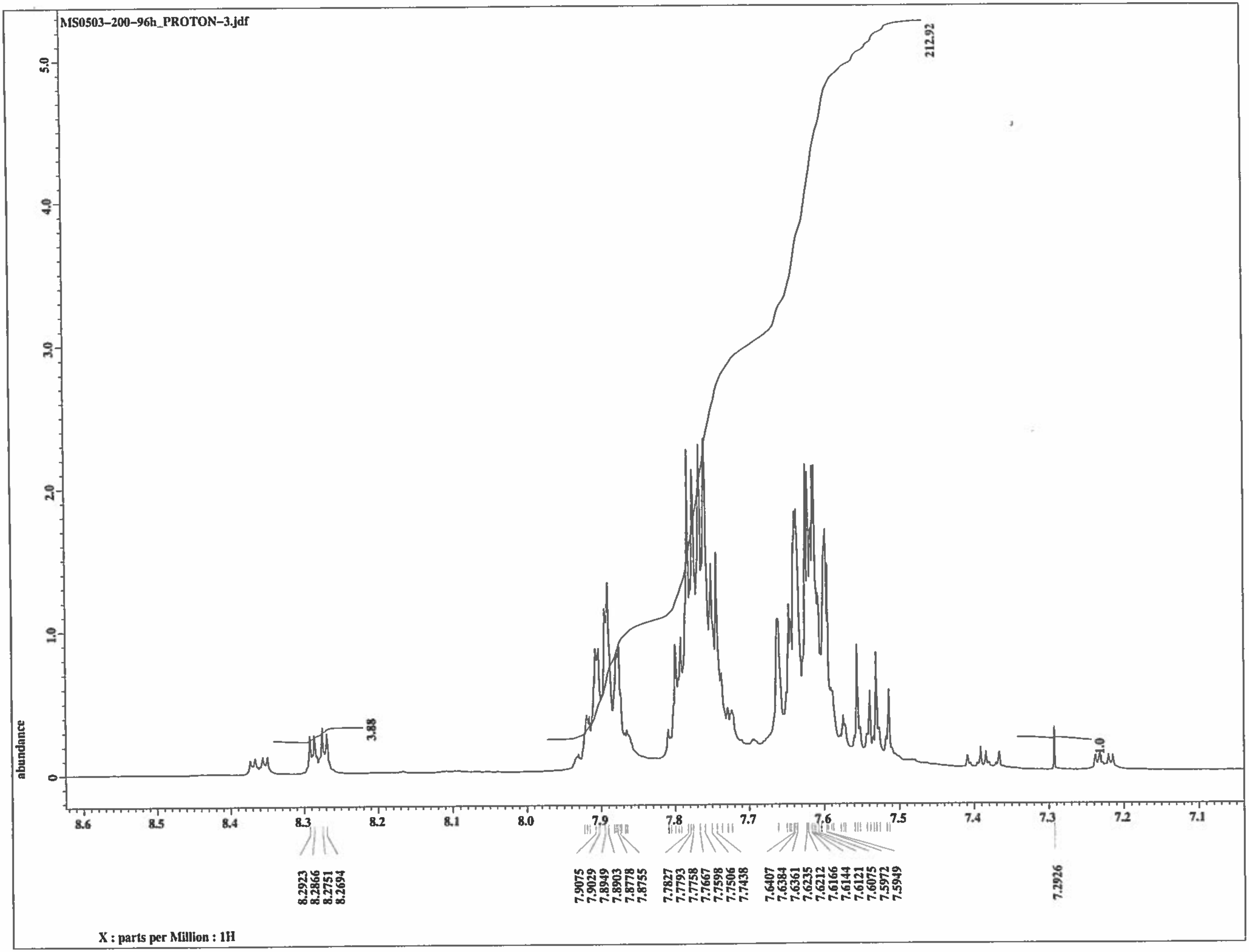
MS0503-200-96h_PROTON-3.jdf
abundance
0
1.0
2.0
3.0
4.0
5.0
8.6
8.5
8.4
8.3
8.2
8.1
8.0
7.9
7.8
7.7
7.6
7.5
7.4
7.3
7.2
7.1
3.88
212.92
1.0
8.2923
8.2866
8.2751
8.2694
7.9075
7.9029
7.8949
7.8903
7.8778
7.8755
7.7827
7.7793
7.7758
7.7667
7.7598
7.7506
7.7438
7.6407
7.6384
7.6361
7.6235
7.6212
7.6166
7.6144
7.6121
7.6075
7.5972
7.5949
7.2926
X : parts per Million : 1H

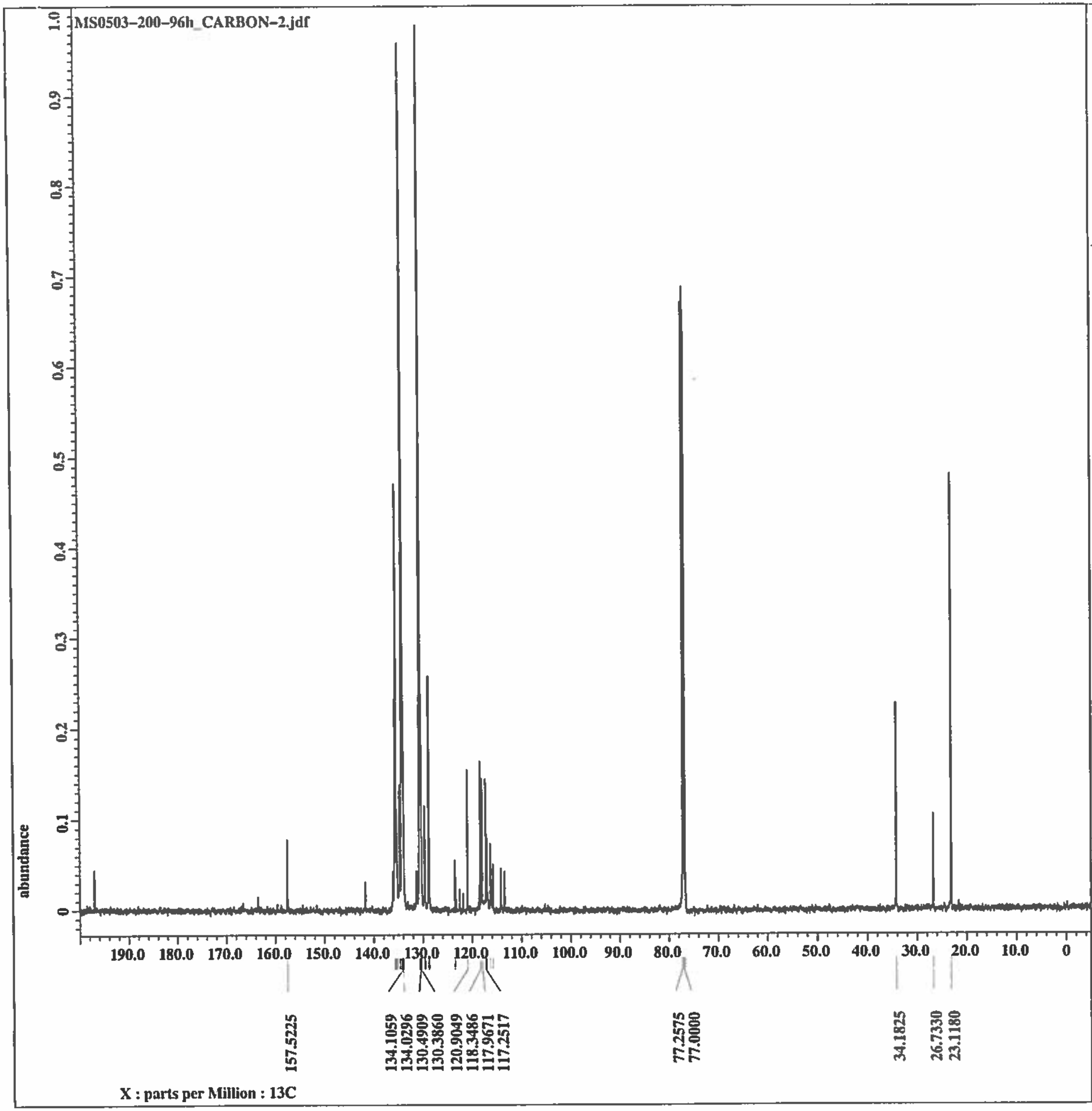

Filename	= MS0503-200-96h_CARBON
Author	= Jim Davis
Experiment	= single_pulse_dec
Sample_id	= MS0503-200-96h
Solvent	= CHLOROFORM-D
Changer_sample	= 4
Creation_time	= 11-JUL-2018 02:42:11
Revision_time	= 11-JUL-2018 02:19:54
Current_time	= 11-JUL-2018 02:19:54
Data_format	= 1D COMPLEX
Dim_size	= 26214
Dim_title	= 13C
Dim_units	= [ppm]
Dimensions	= X
Site	= ECA 500
Spectrometer	= JNM-ECA500
Field_strength	= 11.7473579[T] (500[MH
X_acq_duration	= 0.83361792[s]
X_domain	= 13C
X_freq	= 125.76529768[MHz]
X_offset	= 100[ppm]
X_points	= 32768
X_prescans	= 4
X_resolution	= 1.19959034[Hz]
X_sweep	= 39.3081761[kHz]
Irr_domain	= 1H
Irr_freq	= 500.15991521[MHz]
Irr_offset	= 5.0[ppm]
Clipped	= FALSE
Mod_return	= 1
Scans	= 1024
Total_scans	= 1024
X_90_width	= 13.2[us]
X_acq_time	= 0.83361792[s]
X_angle	= 30[deg]
X_atn	= 6[dB]
X_pulse	= 4.4[us]
Irr_atn_dec	= 20.7[dB]
Irr_atn_noe	= 20.7[dB]
Irr_noise	= WALTZ
Decoupling	= TRUE
Initial_wait	= 1[s]
Noe	= TRUE
Noe_time	= 2[s]
Recvr_gain	= 60
Relaxation_delay	= 2[s]
Repetition_time	= 2.83361792[s]
Temp_get	= 22.8[dC]

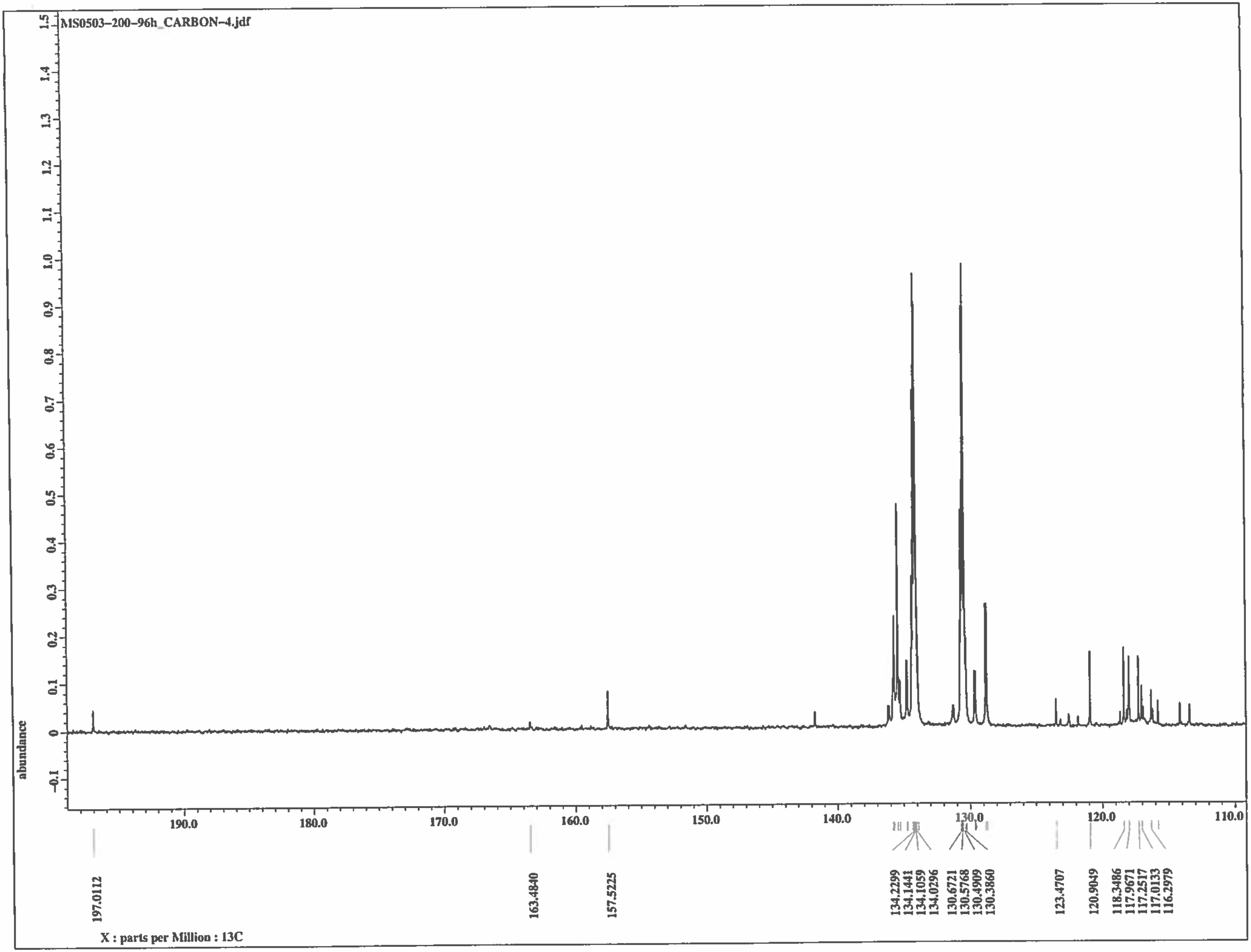
MS0503-200-96h_CARBON-4.jdf
abundance
X : parts per Million : 13C
197.0112
163.4840
157.5225
134.2299
134.1441
134.1059
134.0296
130.6721
130.5768
130.4909
130.3860
123.4707
120.9049
118.3486
117.9671
117.2517
117.0133
116.2979

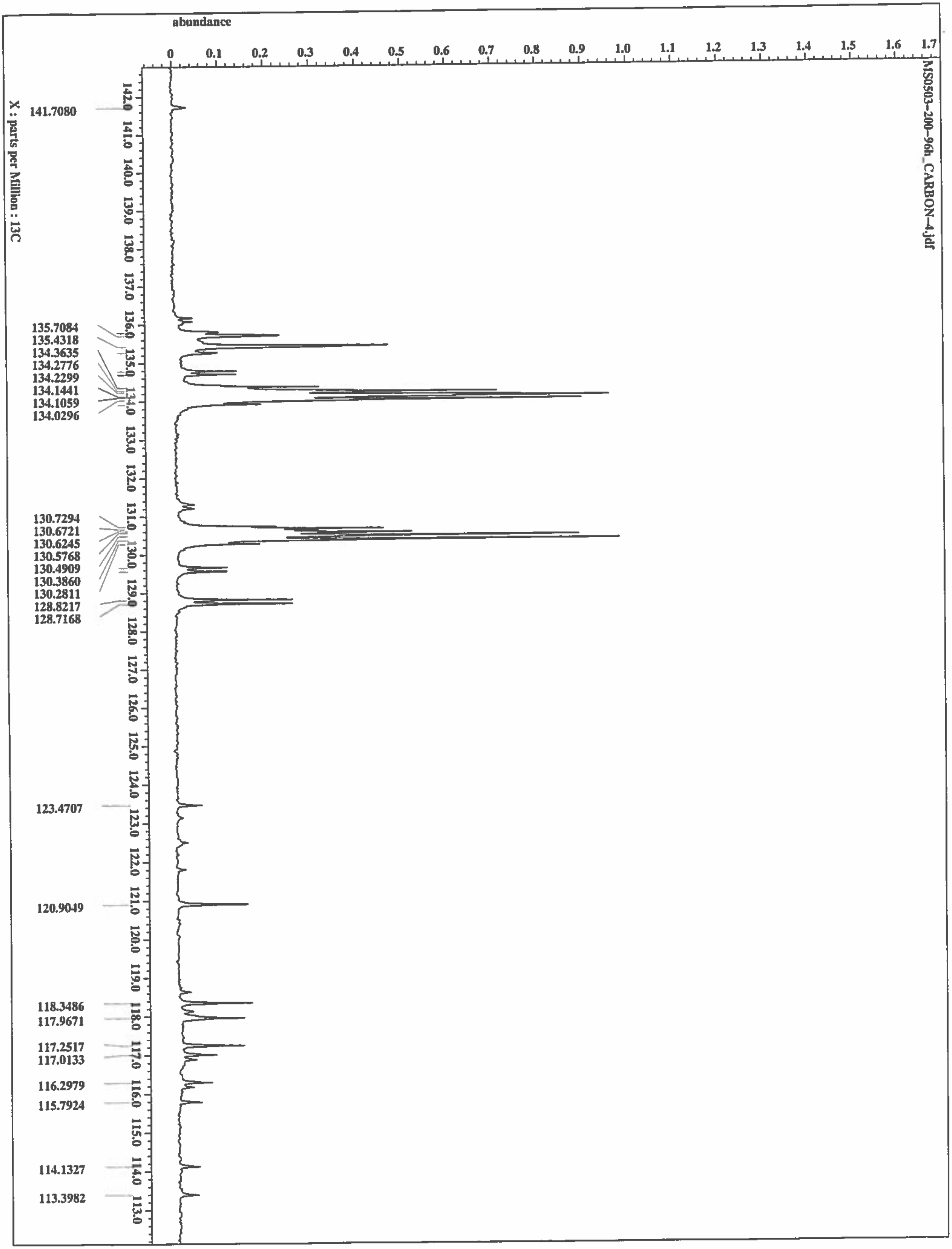

abundance
X : parts per Million : 13C
MS0503-200-96h_CARBON-4.jdf
141.7080
135.7084
135.4318
134.3635
134.2776
134.2299
134.1441
134.1059
134.0296
130.7294
130.6721
130.6245
130.5768
130.4909
130.3860
130.2811
128.8217
128.7168
123.4707
120.9049
118.3486
117.9671
117.2517
117.0133
116.2979
115.7924
114.1327
113.3982

MS0503-200-96h_FLUORINE-2.jdf

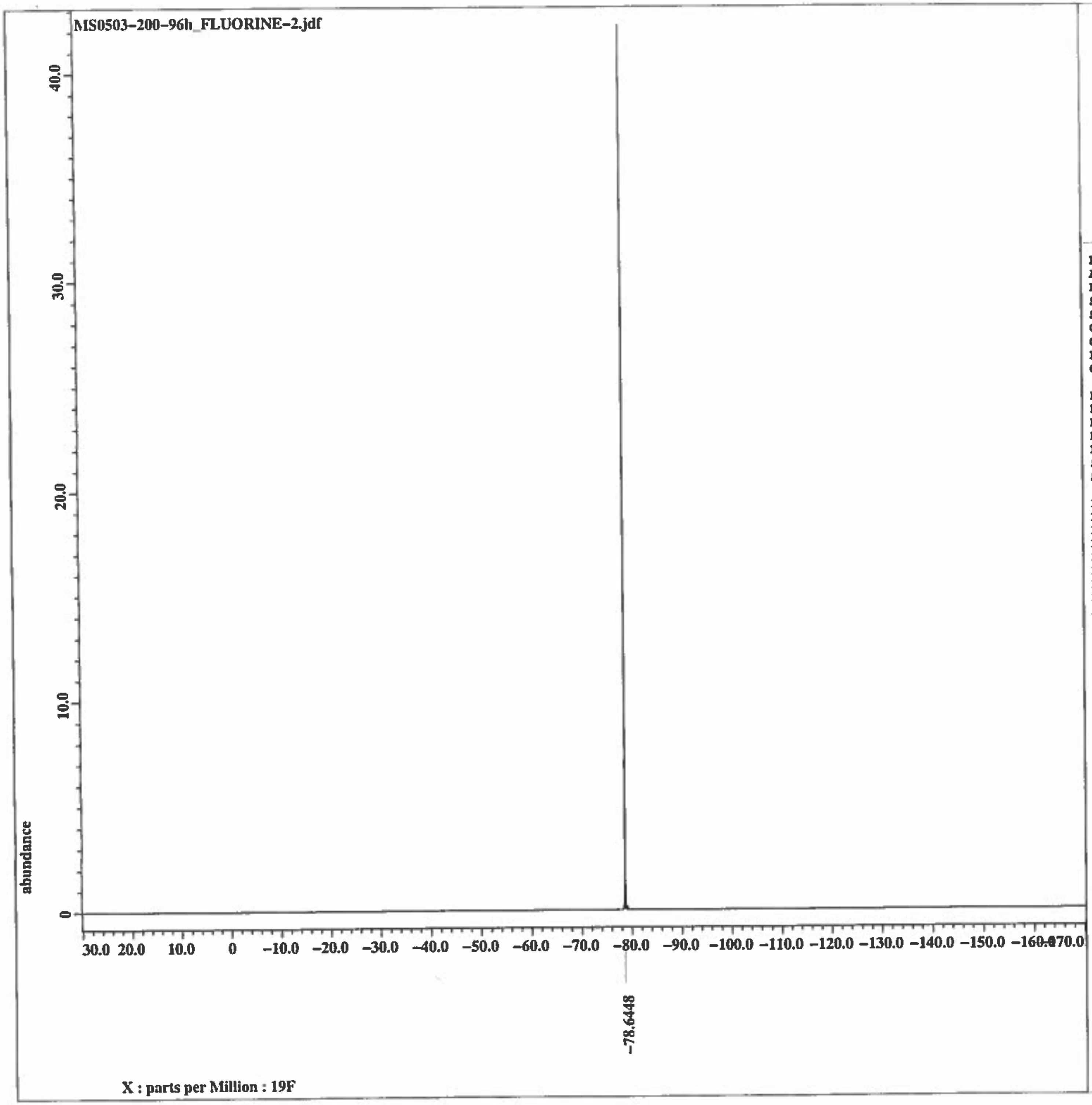

Filename	= MS0503-200-96h_FLUORI
Author	= Jim Davis
Experiment	= single_pulse.ex2
Sample_id	= MS0503-200-96h
Solvent	= CHLOROFORM-D
Changer_sample	= 4
Creation_time	= 11-JUL-2018 02:45:18
Revision_time	= 11-JUL-2018 02:23:01
Current_time	= 11-JUL-2018 02:23:01
Data_format	= 1D COMPLEX
Dim_size	= 52428
Dim_title	= 19F
Dim_units	= [ppm]
Dimensions	= X
Site	= ECA 500
Spectrometer	= JNM-ECA500
Field_strength	= 11.7473579[T] (500[MH
X_acq_duration	= 0.55574528[s]
X_domain	= 19F
X_freq	= 470.62046084[MHz]
X_offset	= -70[ppm]
X_points	= 65536
X_prescans	= 1
X_resolution	= 1.7993855[Hz]
X_sweep	= 117.9245283[kHz]
Irr_domain	= 19F
Irr_freq	= 470.62046084[MHz]
Irr_offset	= 5[ppm]
Tri_domain	= 19F
Tri_freq	= 470.62046084[MHz]
Tri_offset	= 5[ppm]
Clipped	= FALSE
Mod_return	= 1
Scans	= 16
Total_scans	= 16
X_90_width	= 13.1[us]
X_acq_time	= 0.55574528[s]
X_angle	= 45[deg]
X_atn	= 2.5[dB]
X_pulse	= 6.55[us]
Irr_mode	= Off
Tri_mode	= Off
Dante_presat	= FALSE
Initial_wait	= 1[s]
Recvr_gain	= 36
Relaxation_delay	= 4[s]
Repetition_time	= 4.55574528[s]
Temp_get	= 22.4[dC]

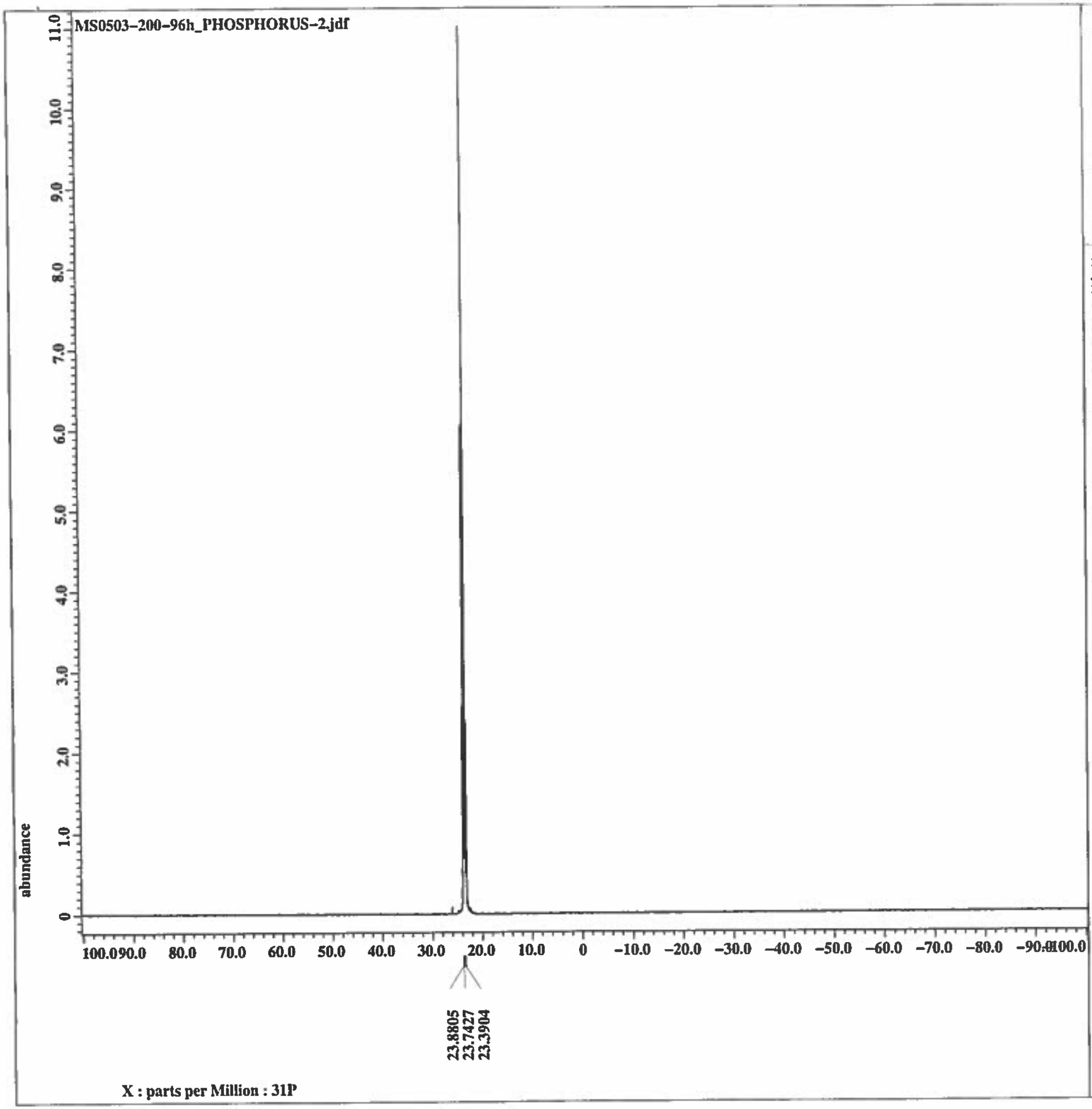

Filename	= MS0503-200-96h_PHOSPH
Author	= Jim Davis
Experiment	= single_pulse_dec
Sample_id	= MS0503-200-96h
Solvent	= CHLOROFORM-D
Changer_sample	= 4
Creation_time	= 11-JUL-2018 02:59:05
Revision_time	= 11-JUL-2018 02:36:48
Current_time	= 11-JUL-2018 02:36:48
Data_format	= 1D COMPLEX
Dim_size	= 26214
Dim_title	= 31P
Dim_units	= [ppm]
Dimensions	= X
Site	= ECA 500
Spectrometer	= JNM-ECA500
Field_strength	= 11.7473579[T] (500[MH
X_acq_duration	= 0.64487424[s]
X_domain	= 31P
X_freq	= 202.46831075[MHz]
X_offset	= 0[ppm]
X_points	= 32768
X_prescans	= 4
X_resolution	= 1.55068995[Hz]
X_sweep	= 50.81300813[kHz]
Irr_domain	= 1H
Irr_freq	= 500.15991521[MHz]
Irr_offset	= 5.0[ppm]
Clipped	= FALSE
Mod_return	= 1
Scans	= 256
Total_scans	= 256
X_90_width	= 14.687[us]
X_acq_time	= 0.64487424[s]
X_angle	= 30[deg]
X_atn	= 5[dB]
X_pulse	= 4.89566667[us]
Irr_atn_dec	= 20.7[dB]
Irr_atn_noe	= 20.7[dB]
Irr_noise	= WALTZ
Decoupling	= TRUE
Initial_wait	= 1[s]
Noe	= TRUE
Noe_time	= 2[s]
Recvr_gain	= 58
Relaxation_delay	= 2[s]
Repetition_time	= 2.64487424[s]
Temp_get	= 22.8[dC]

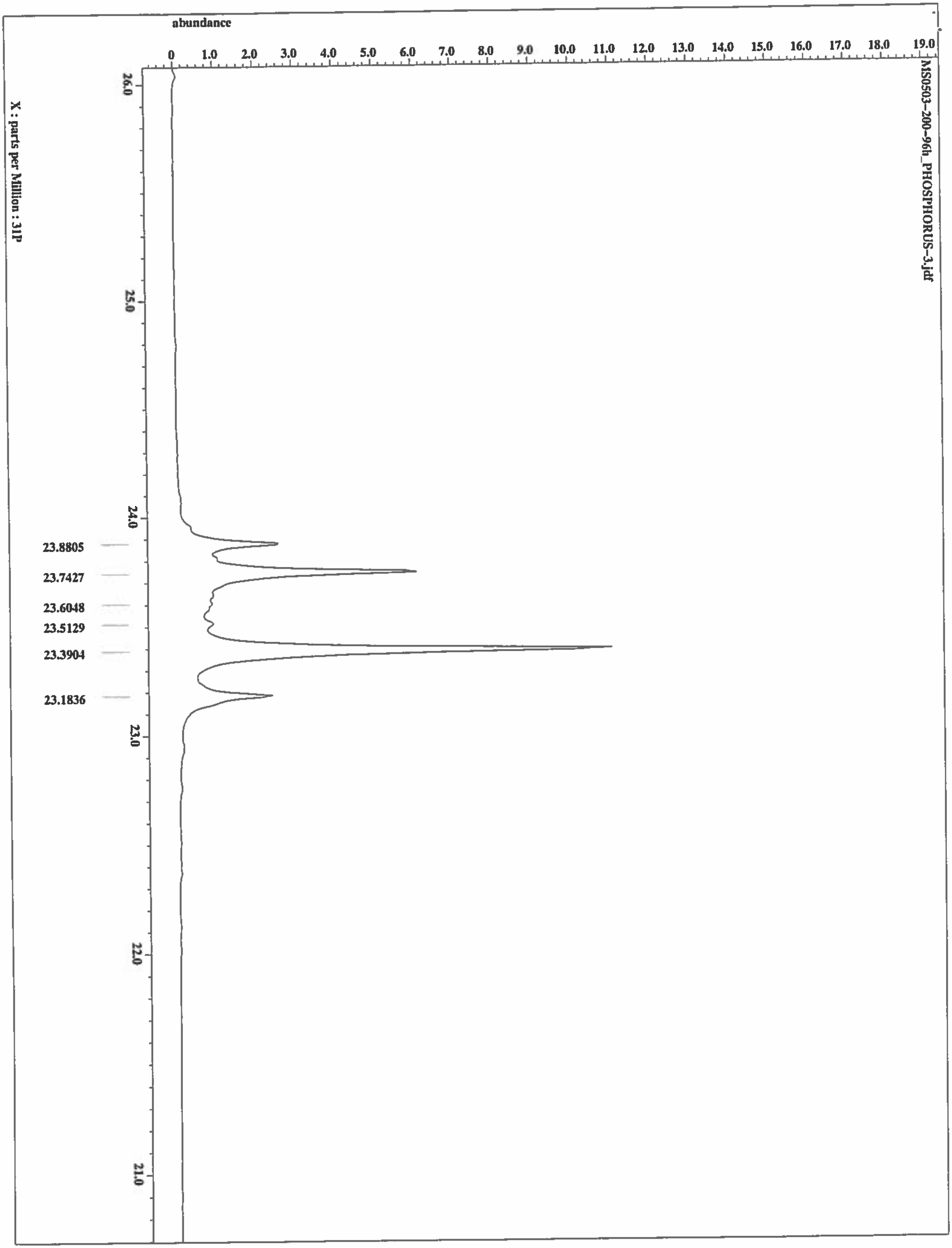

abundance
0
1.0
2.0
3.0
4.0
5.0
6.0
7.0
8.0
9.0
10.0
11.0
12.0
13.0
14.0
15.0
16.0
17.0
18.0
19.0
MS0503-200-96h_PHOSPHORUS-3.jdf
X : parts per Million : 31P
26.0
25.0
24.0
23.0
22.0
21.0
23.8805
23.7427
23.6048
23.5129
23.3904
23.1836

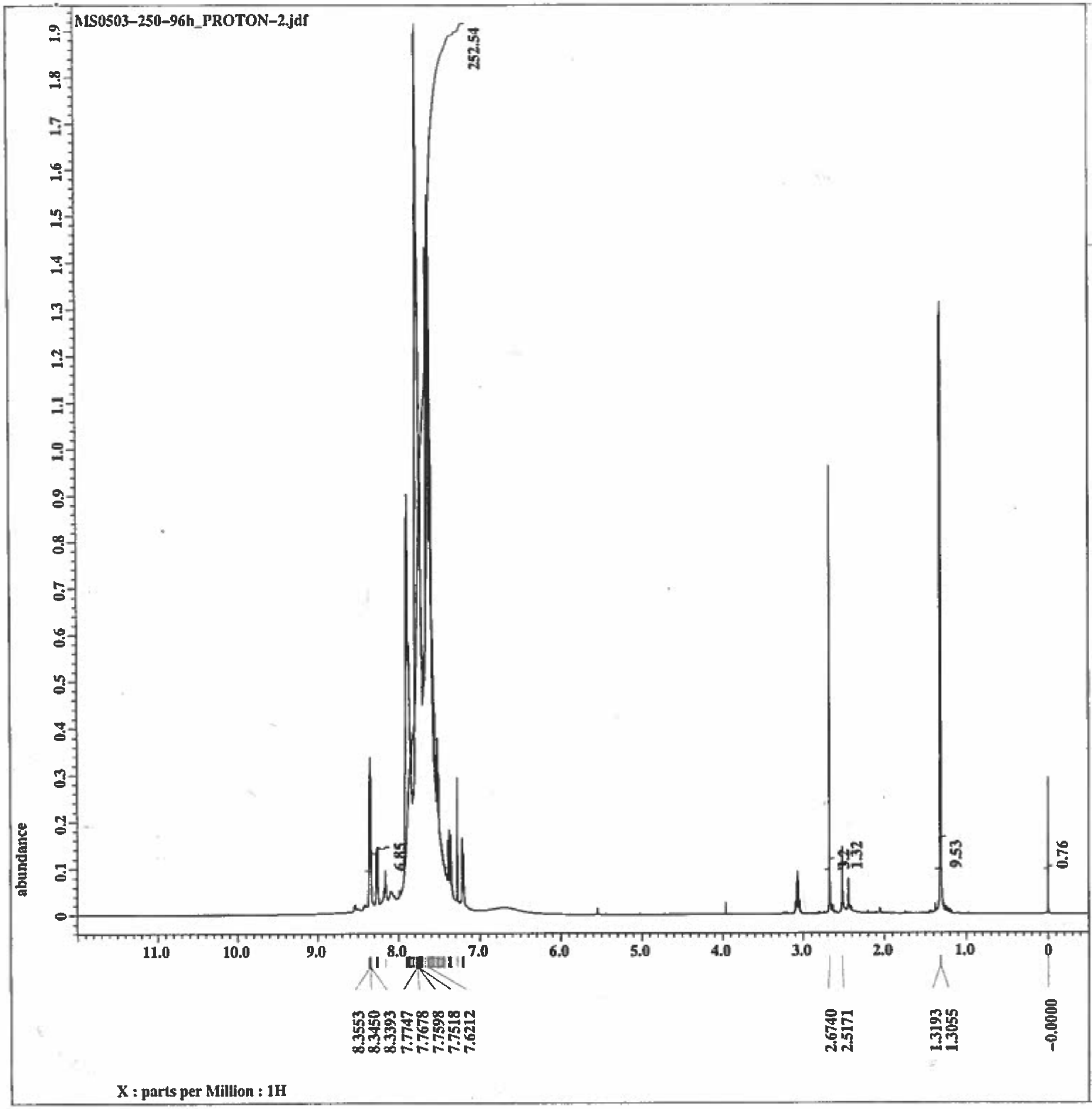

Filename	= MS0503-250-96h_PROTON
Author	= Jim Davis
Experiment	= single_pulse.ex2
Sample_id	= MS0503-250-96h
Solvent	= CHLOROFORM-D
Changer_sample	= 5
Creation_time	= 10-JUL-2018 18:34:37
Revision_time	= 10-JUL-2018 18:12:23
Current_time	= 10-JUL-2018 18:12:23
Data_format	= 1D COMPLEX
Dim_size	= 13107
Dim_title	= 1H
Dim_units	= [ppm]
Dimensions	= X
Site	= ECA 500
Spectrometer	= JNM-ECA500
Field_strength	= 11.7473579[T] (500[MH
X_acq_duration	= 1.74587904[s]
X_domain	= 1H
X_freq	= 500.15991521[MHz]
X_offset	= 5.0[ppm]
X_points	= 16384
X_prescans	= 1
X_resolution	= 0.57277737[Hz]
X_sweep	= 9.38438438[kHz]
Irr_domain	= 1H
Irr_freq	= 500.15991521[MHz]
Irr_offset	= 5.0[ppm]
Tri_domain	= 1H
Tri_freq	= 500.15991521[MHz]
Tri_offset	= 5.0[ppm]
Clipped	= FALSE
Mod_return	= 1
Scans	= 16
Total_scans	= 16
X_90_width	= 12.4[us]
X_acq_time	= 1.74587904[s]
X_angle	= 45[deg]
X_atn	= 4[dB]
X_pulse	= 6.2[us]
Irr_mode	= Off
Tri_mode	= Off
Dante_presat	= FALSE
Initial_wait	= 1[s]
Recvr_gain	= 28
Relaxation_delay	= 4[s]
Repetition_time	= 5.74587904[s]
Temp_get	= 22.8[dC]

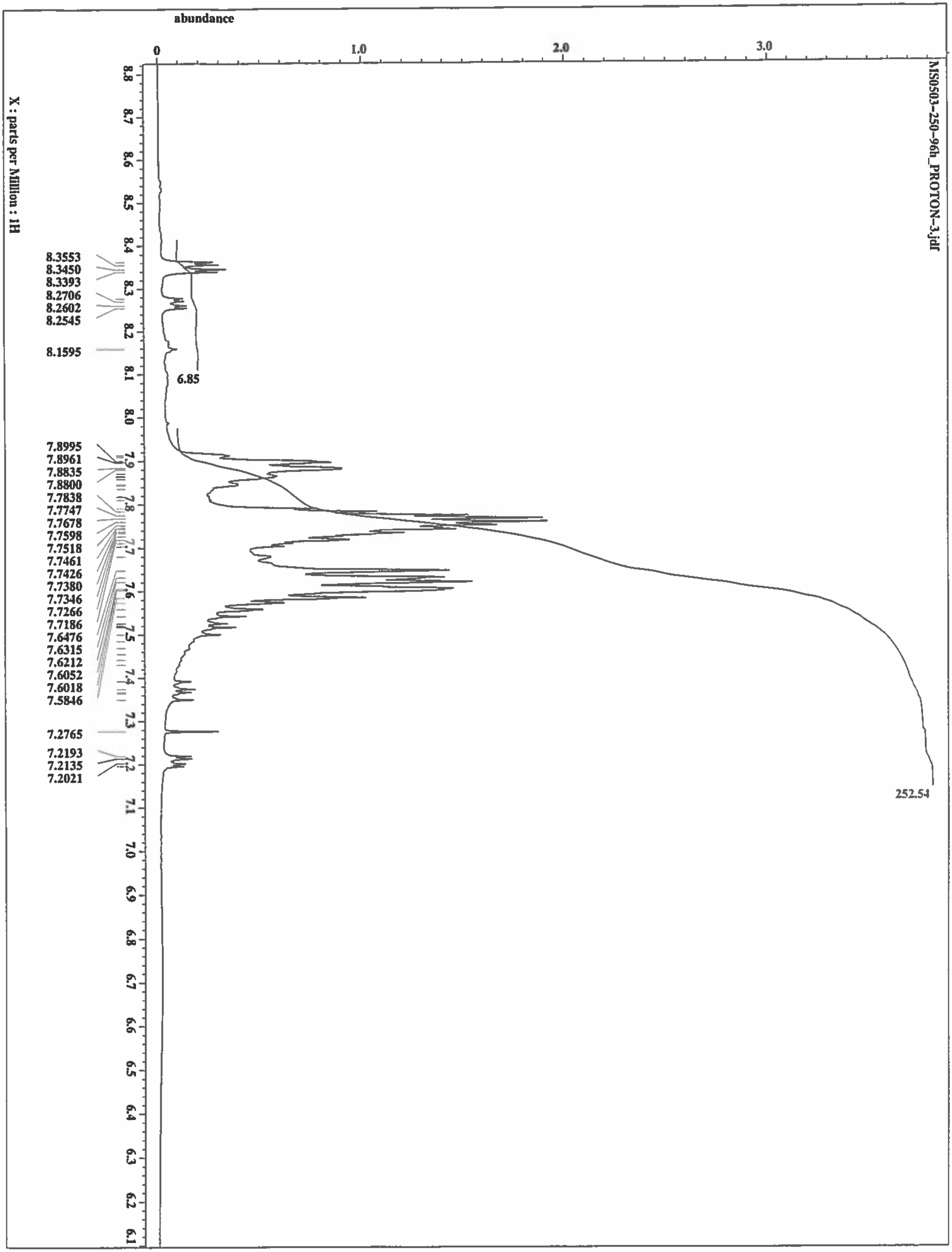

abundance
0
1.0
2.0
3.0
MS0503-250-96h_PROTON-3.jdf
X : parts per Million : 1H
8.8
8.7
8.6
8.5
8.4
8.3
8.2
8.1
8.0
7.9
7.8
7.7
7.6
7.5
7.4
7.3
7.2
7.1
7.0
6.9
6.8
6.7
6.6
6.5
6.4
6.3
6.2
6.1
8.3553
8.3450
8.3393
8.2706
8.2602
8.2545
8.1595
6.85
7.8995
7.8961
7.8835
7.8800
7.7838
7.7747
7.7678
7.7598
7.7518
7.7461
7.7426
7.7380
7.7346
7.7266
7.7186
7.6476
7.6315
7.6212
7.6052
7.6018
7.5846
7.2765
7.2193
7.2135
7.2021
252.54

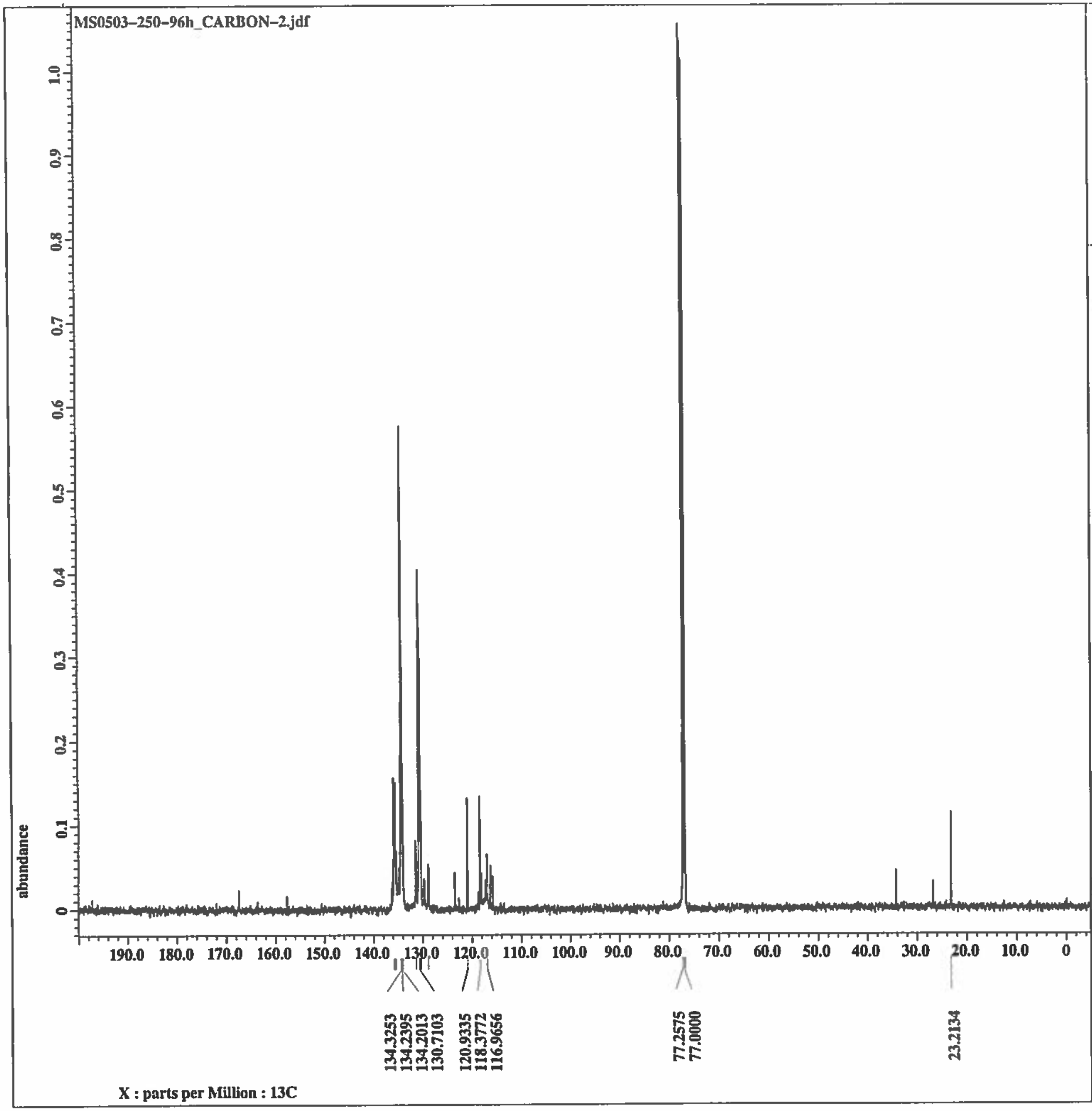

Filename	= MS0503-250-96h_CARBON
Author	= Jim Davis
Experiment	= single_pulse_dec
Sample_id	= MS0503-250-96h
Solvent	= CHLOROFORM-D
Changer_sample	= 5
Creation_time	= 10-JUL-2018 19:05:21
Revision_time	= 10-JUL-2018 18:43:05
Current_time	= 10-JUL-2018 18:43:05
Data_format	= 1D COMPLEX
Dim_size	= 26214
Dim_title	= 13C
Dim_units	= [ppm]
Dimensions	= X
Site	= ECA 500
Spectrometer	= JNM-ECA500
Field_strength	= 11.7473579[T] (500[MH
X_acq_duration	= 0.83361792[s]
X_domain	= 13C
X_freq	= 125.76529768[MHz]
X_offset	= 100[ppm]
X_points	= 32768
X_prescans	= 4
X_resolution	= 1.19959034[Hz]
X_sweep	= 39.3081761[kHz]
Irr_domain	= 1H
Irr_freq	= 500.15991521[MHz]
Irr_offset	= 5.0[ppm]
Clipped	= FALSE
Mod_return	= 1
Scans	= 600
Total_scans	= 600
X_90_width	= 13.2[us]
X_acq_time	= 0.83361792[s]
X_angle	= 30[deg]
X_atn	= 6[dB]
X_pulse	= 4.4[us]
Irr_atn_dec	= 20.7[dB]
Irr_atn_noe	= 20.7[dB]
Irr_noise	= WALTZ
Decoupling	= TRUE
Initial_wait	= 1[s]
Noe	= TRUE
Noe_time	= 2[s]
Recvr_gain	= 60
Relaxation_delay	= 2[s]
Repetition_time	= 2.83361792[s]
Temp_get	= 23.4[dC]

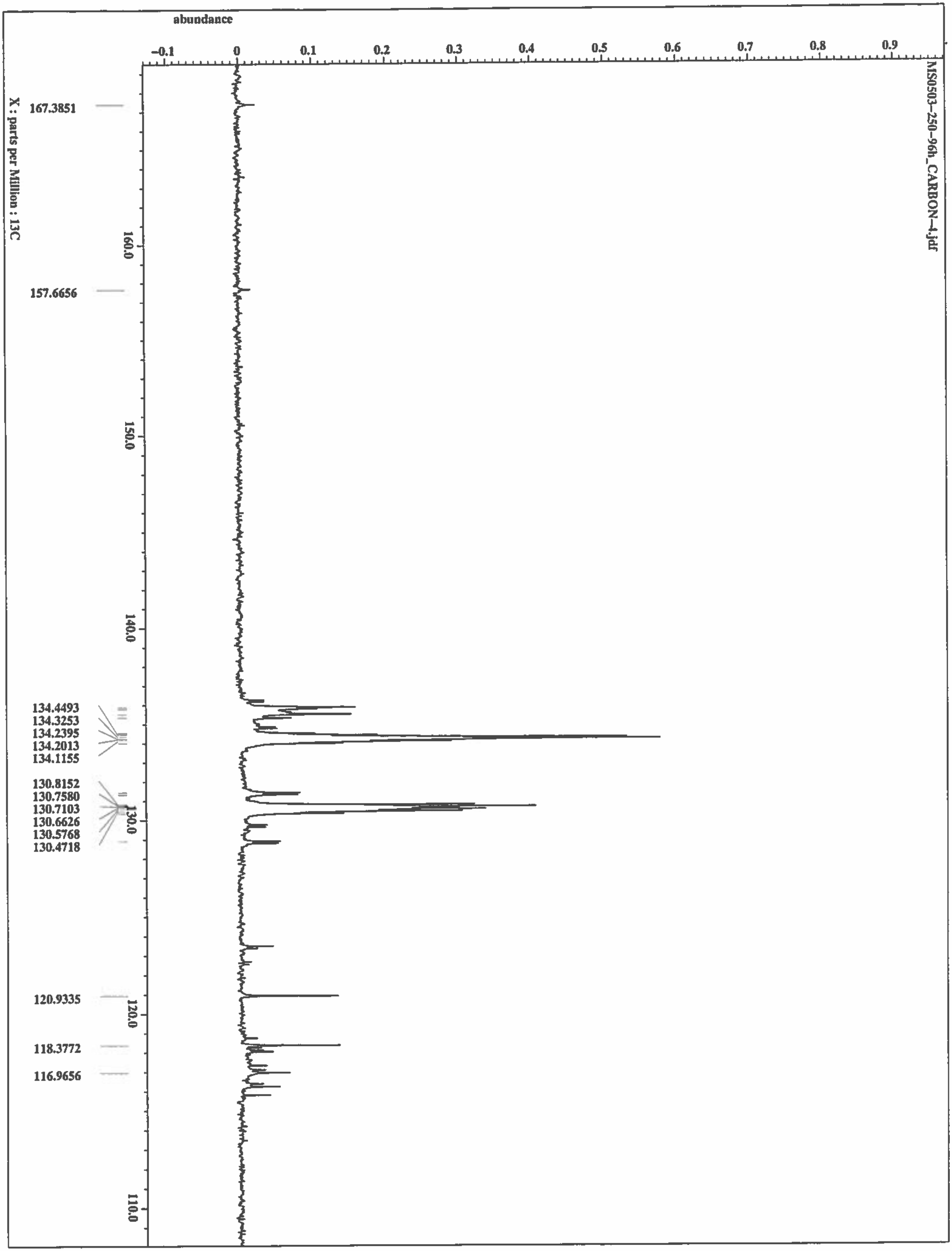

abundance
-0.1
0
0.1
0.2
0.3
0.4
0.5
0.6
0.7
0.8
0.9
X : parts per Million : 13C
MS0503-250-96h_CARBON-4.jdf
167.3851
157.6656
134.4493
134.3253
134.2395
134.2013
134.1155
130.8152
130.7580
130.7103
130.6626
130.5768
130.4718
120.9335
118.3772
116.9656
160.0
150.0
140.0
130.0
120.0
110.0

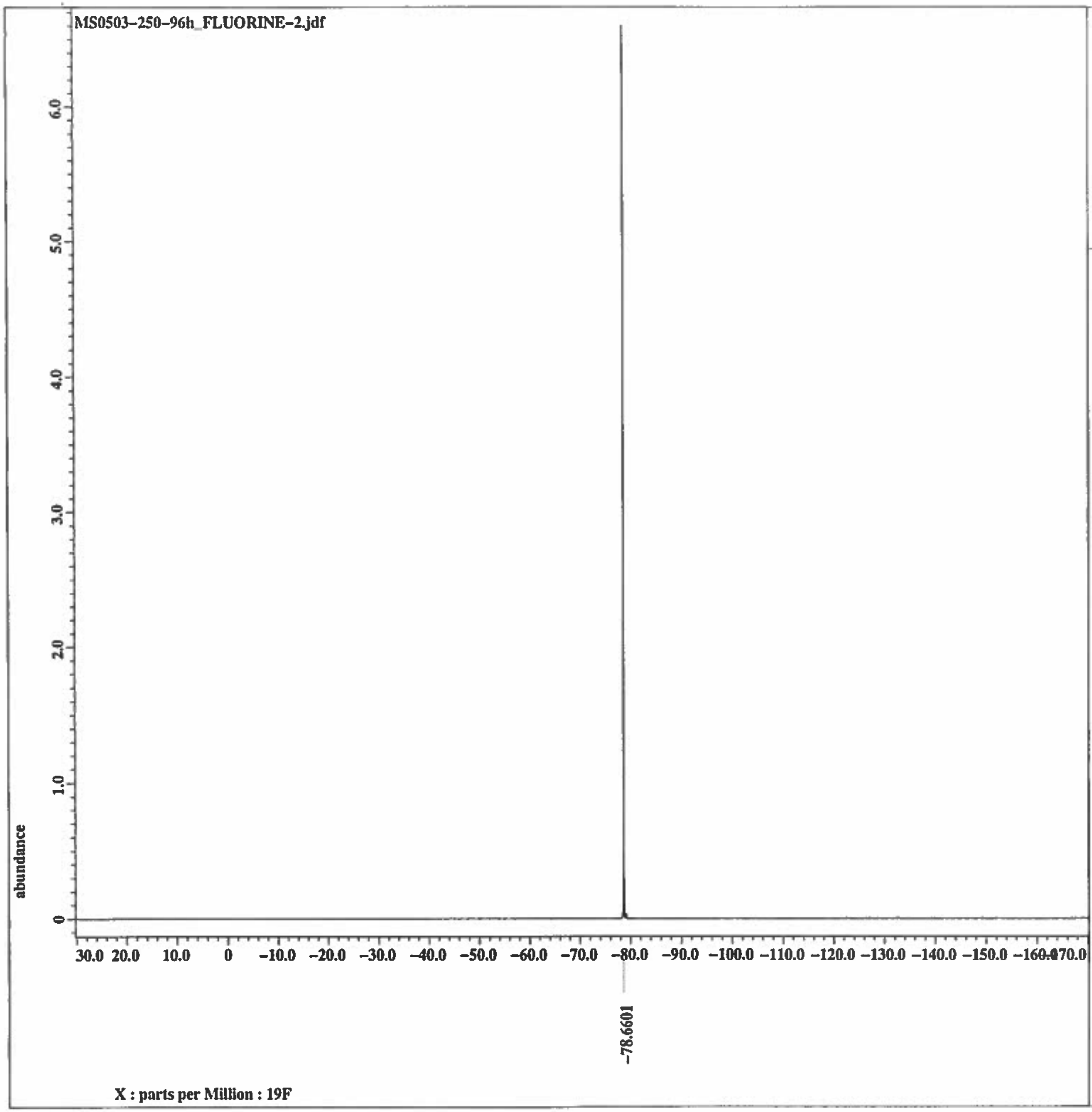

Filename	= MS0503-250-96h_FLUORI
Author	= Jim Davis
Experiment	= single_pulse.ex2
Sample_id	= MS0503-250-96h
Solvent	= CHLOROFORM-D
Changer_sample	= 5
Creation_time	= 10-JUL-2018 19:08:18
Revision_time	= 10-JUL-2018 18:46:04
Current_time	= 10-JUL-2018 18:46:04
Data_format	= 1D COMPLEX
Dim_size	= 52428
Dim_title	= 19F
Dim_units	= [ppm]
Dimensions	= X
Site	= ECA 500
Spectrometer	= JNM-ECA500
Field_strength	= 11.7473579[T] (500[MH
X_acq_duration	= 0.55574528[s]
X_domain	= 19F
X_freq	= 470.62046084[MHz]
X_offset	= -70[ppm]
X_points	= 65536
X_prescans	= 1
X_resolution	= 1.7993855[Hz]
X_sweep	= 117.9245283[kHz]
Irr_domain	= 19F
Irr_freq	= 470.62046084[MHz]
Irr_offset	= 5[ppm]
Tri_domain	= 19F
Tri_freq	= 470.62046084[MHz]
Tri_offset	= 5[ppm]
Clipped	= FALSE
Mod_return	= 1
Scans	= 16
Total_scans	= 16
X_90_width	= 13.1[us]
X_acq_time	= 0.55574528[s]
X_angle	= 45[deg]
X_atn	= 2.5[dB]
X_pulse	= 6.55[us]
Irr_mode	= Off
Tri_mode	= Off
Dante_presat	= FALSE
Initial_wait	= 1[s]
Recvr_gain	= 38
Relaxation_delay	= 4[s]
Repetition_time	= 4.55574528[s]
Temp_get	= 23[dC]

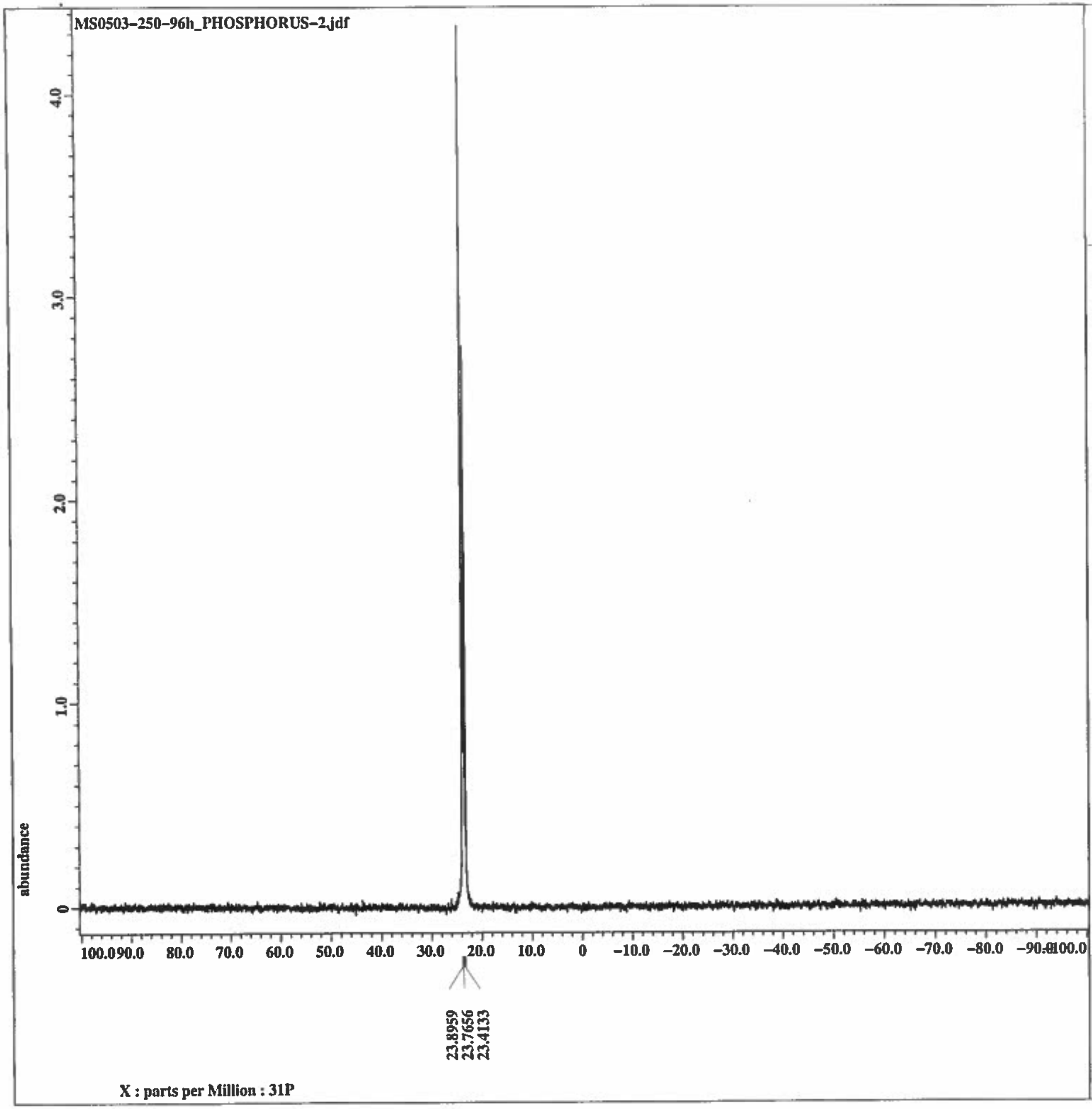

Filename	= MS0503-250-96h_PHOSPH
Author	= Jim Davis
Experiment	= single_pulse_dec
Sample_id	= MS0503-250-96h
Solvent	= CHLOROFORM-D
Changer_sample	= 5
Creation_time	= 10-JUL-2018 19:13:18
Revision_time	= 10-JUL-2018 18:51:02
Current_time	= 10-JUL-2018 18:51:02
Data_format	= 1D COMPLEX
Dim_size	= 26214
Dim_title	= 31P
Dim_units	= [ppm]
Dimensions	= X
Site	= ECA 500
Spectrometer	= JNM-ECA500
Field_strength	= 11.7473579[T] (500[MH
X_acq_duration	= 0.64487424[s]
X_domain	= 31P
X_freq	= 202.46831075[MHz]
X_offset	= 0[ppm]
X_points	= 32768
X_prescans	= 4
X_resolution	= 1.55068995[Hz]
X_sweep	= 50.81300813[kHz]
Irr_domain	= 1H
Irr_freq	= 500.15991521[MHz]
Irr_offset	= 5.0[ppm]
Clipped	= FALSE
Mod_return	= 1
Scans	= 50
Total_scans	= 50
X_90_width	= 14.687[us]
X_acq_time	= 0.64487424[s]
X_angle	= 30[deg]
X_atn	= 5[dB]
X_pulse	= 4.89566667[us]
Irr_atn_dec	= 20.7[dB]
Irr_atn_noe	= 20.7[dB]
Irr_noise	= WALTZ
Decoupling	= TRUE
Initial_wait	= 1[s]
Noe	= TRUE
Noe_time	= 2[s]
Recvr_gain	= 60
Relaxation_delay	= 2[s]
Repetition_time	= 2.64487424[s]
Temp_get	= 23.2[dC]

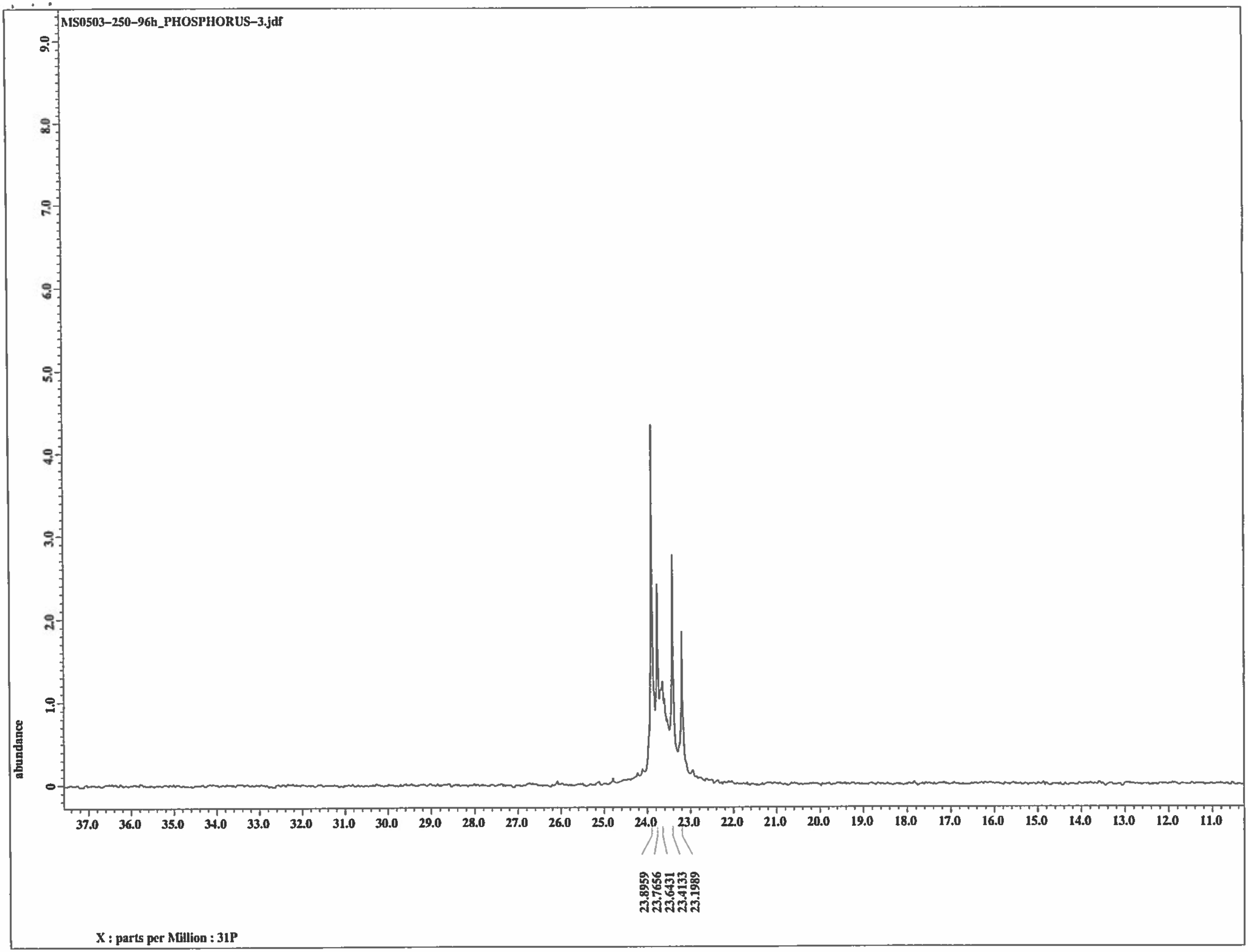

MS0503-250-96h_PHOSPHORUS-3.jdf
abundance
0
1.0
2.0
3.0
4.0
5.0
6.0
7.0
8.0
9.0
37.0
36.0
35.0
34.0
33.0
32.0
31.0
30.0
29.0
28.0
27.0
26.0
25.0
24.0
23.0
22.0
21.0
20.0
19.0
18.0
17.0
16.0
15.0
14.0
13.0
12.0
11.0
23.8959
23.7656
23.6431
23.4133
23.1989
X : parts per Million : 31P

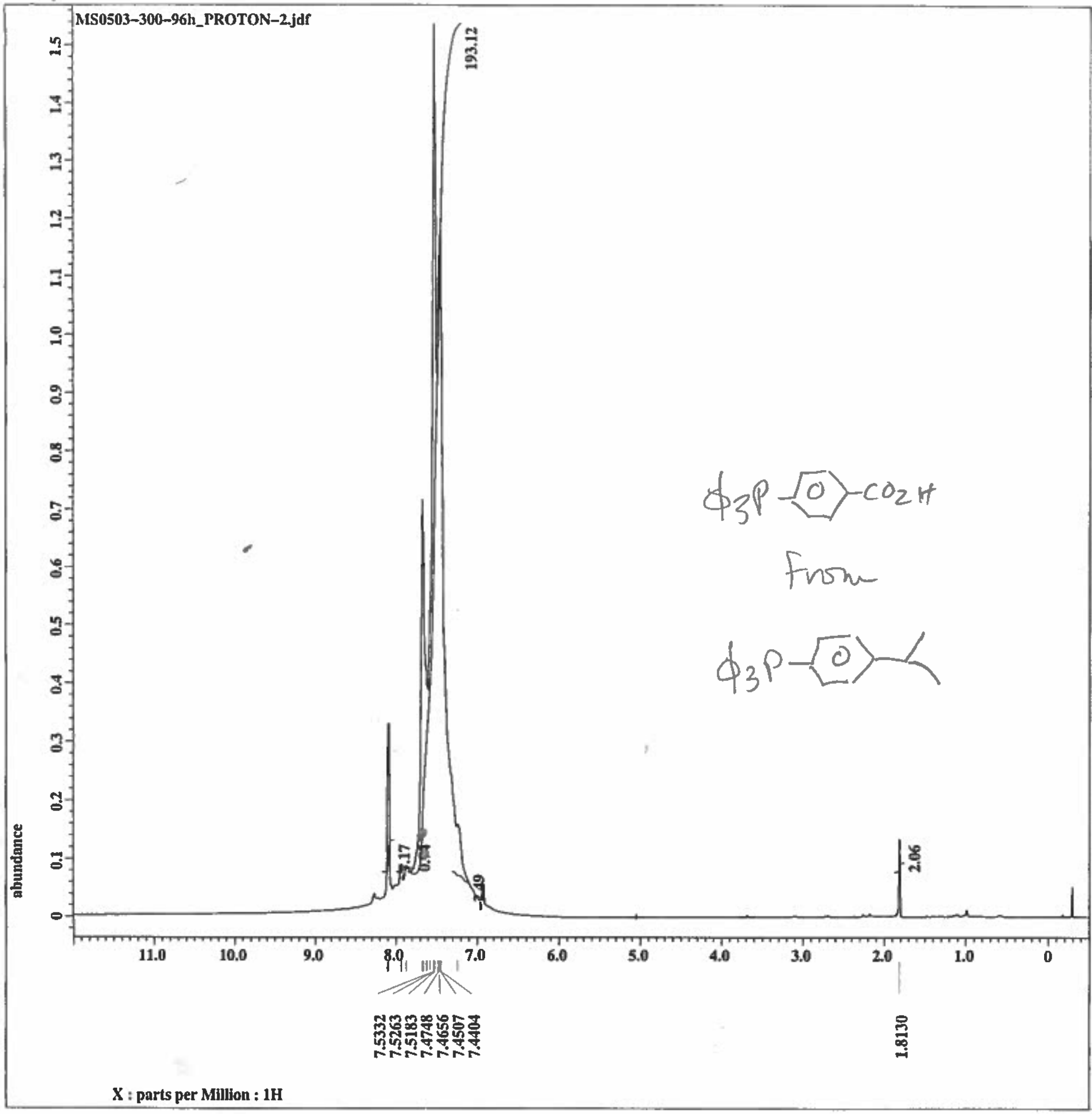

Filename	= MS0503-300-96h_PROTON
Author	= Jim Davis
Experiment	= single_pulse.ex2
Sample_id	= MS0503-300-96h
Solvent	= CHLOROFORM-D
Changer_sample	= 6
Creation_time	= 10-JUL-2018 19:20:04
Revision_time	= 10-JUL-2018 18:57:48
Current_time	= 10-JUL-2018 18:57:48
Data_format	= 1D COMPLEX
Dim_size	= 13107
Dim_title	= 1H
Dim_units	= [ppm]
Dimensions	= X
Site	= ECA 500
Spectrometer	= JNM-ECA500
Field_strength	= 11.7473579[T] (500[MH
X_acq_duration	= 1.74587904[s]
X_domain	= 1H
X_freq	= 500.15991521[MHz]
X_offset	= 5.0[ppm]
X_points	= 16384
X_prescans	= 1
X_resolution	= 0.57277737[Hz]
X_sweep	= 9.38438438[kHz]
Irr_domain	= 1H
Irr_freq	= 500.15991521[MHz]
Irr_offset	= 5.0[ppm]
Tri_domain	= 1H
Tri_freq	= 500.15991521[MHz]
Tri_offset	= 5.0[ppm]
Clipped	= FALSE
Mod_return	= 1
Scans	= 16
Total_scans	= 16
X_90_width	= 12.4[us]
X_acq_time	= 1.74587904[s]
X_angle	= 45[deg]
X_atn	= 4[dB]
X_pulse	= 6.2[us]
Irr_mode	= Off
Tri_mode	= Off
Dante_presat	= FALSE
Initial_wait	= 1[s]
Recvr_gain	= 28
Relaxation_delay	= 4[s]
Repetition_time	= 5.74587904[s]
Temp_get	= 22.8[dC]

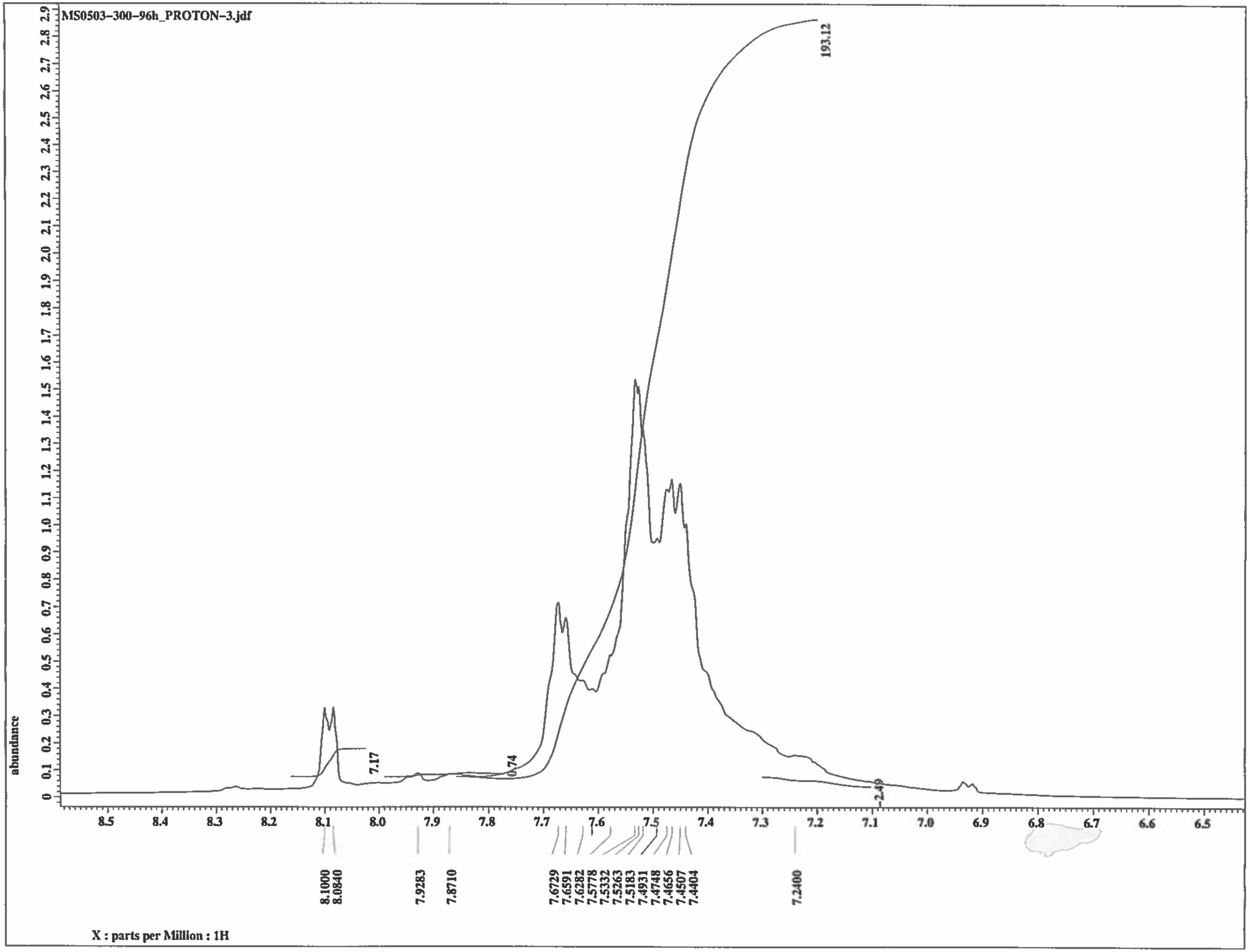
MS0503-300-96h_PROTON-3.jdf
abundance
X : parts per Million : 1H
7.17
0.74
193.12
-2.49
8.1000
8.0840
7.9283
7.8710
7.6729
7.6591
7.6282
7.5778
7.5332
7.5263
7.5183
7.4931
7.4748
7.4656
7.4507
7.4404
7.2400

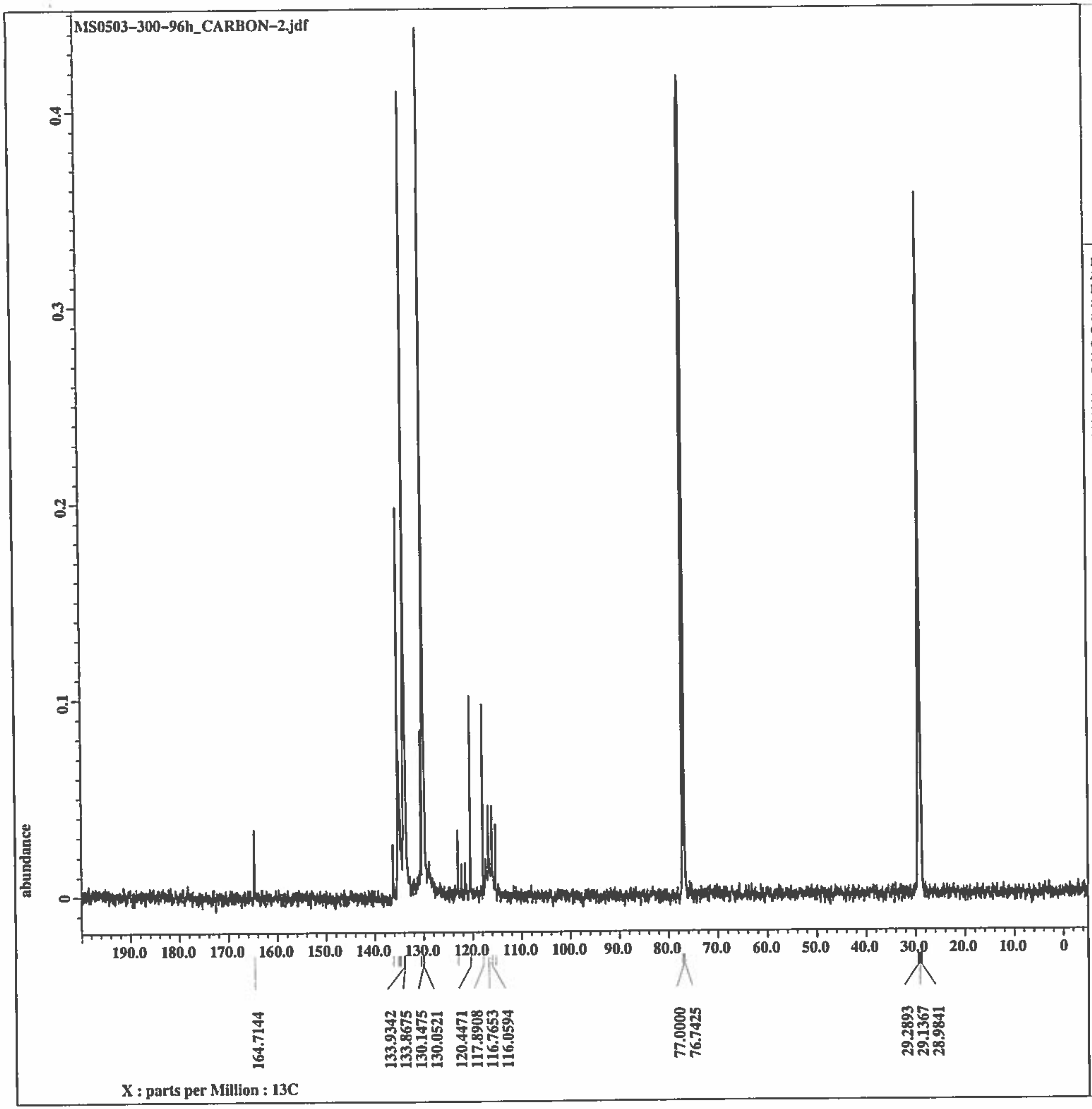

Filename	= MS0503-300-96h_CARBON
Author	= Jim Davis
Experiment	= single_pulse_dec
Sample_id	= MS0503-300-96h
Solvent	= CHLOROFORM-D
Changer_sample	= 6
Creation_time	= 10-JUL-2018 20:10:45
Revision_time	= 10-JUL-2018 19:48:30
Current_time	= 10-JUL-2018 19:48:30
Data_format	= 1D COMPLEX
Dim_size	= 26214
Dim_title	= 13C
Dim_units	= [ppm]
Dimensions	= X
Site	= ECA 500
Spectrometer	= JNM-ECA500
Field_strength	= 11.7473579[T] (500[MH
X_acq_duration	= 0.83361792[s]
X_domain	= 13C
X_freq	= 125.76529768[MHz]
X_offset	= 100[ppm]
X_points	= 32768
X_prescans	= 4
X_resolution	= 1.19959034[Hz]
X_sweep	= 39.3081761[kHz]
Irr_domain	= 1H
Irr_freq	= 500.15991521[MHz]
Irr_offset	= 5.0[ppm]
Clipped	= FALSE
Mod_return	= 1
Scans	= 1024
Total_scans	= 1024
X_90_width	= 13.2[us]
X_acq_time	= 0.83361792[s]
X_angle	= 30[deg]
X_atn	= 6[dB]
X_pulse	= 4.4[us]
Irr_atn_dec	= 20.7[dB]
Irr_atn_noe	= 20.7[dB]
Irr_noise	= WALTZ
Decoupling	= TRUE
Initial_wait	= 1[s]
Noe	= TRUE
Noe_time	= 2[s]
Recvr_gain	= 60
Relaxation_delay	= 2[s]
Repetition_time	= 2.83361792[s]
Temp_get	= 23.2[dC]

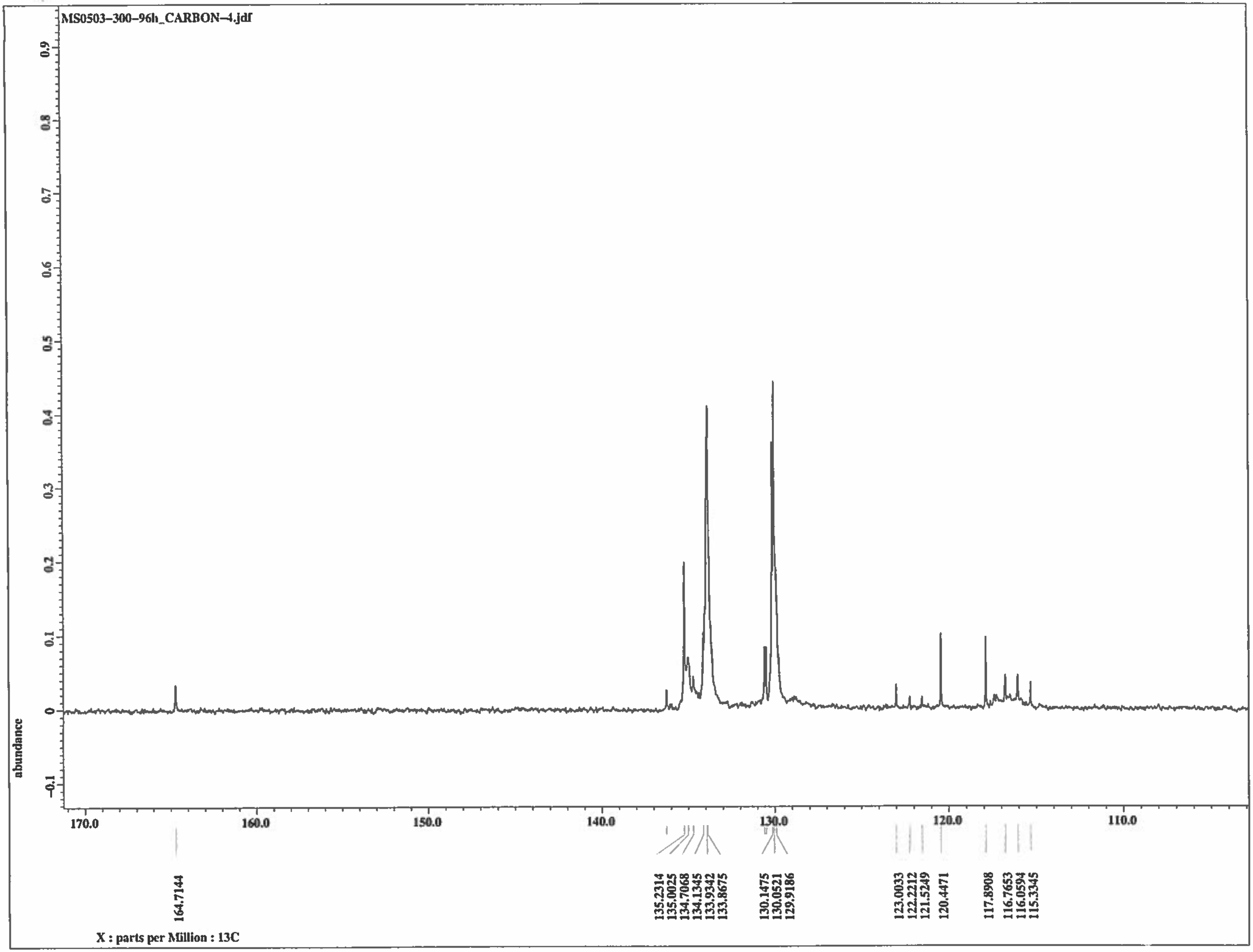

MS0503-300-96h_CARBON-4.jdf
abundance
0.9
0.8
0.7
0.6
0.5
0.4
0.3
0.2
0.1
0
−0.1
170.0
160.0
150.0
140.0
130.0
120.0
110.0
164.7144
135.2314
135.0025
134.7068
134.1345
133.9342
133.8675
130.1475
130.0521
129.9186
123.0033
122.2212
121.5249
120.4471
117.8908
116.7653
116.0594
115.3345
X : parts per Million : 13C

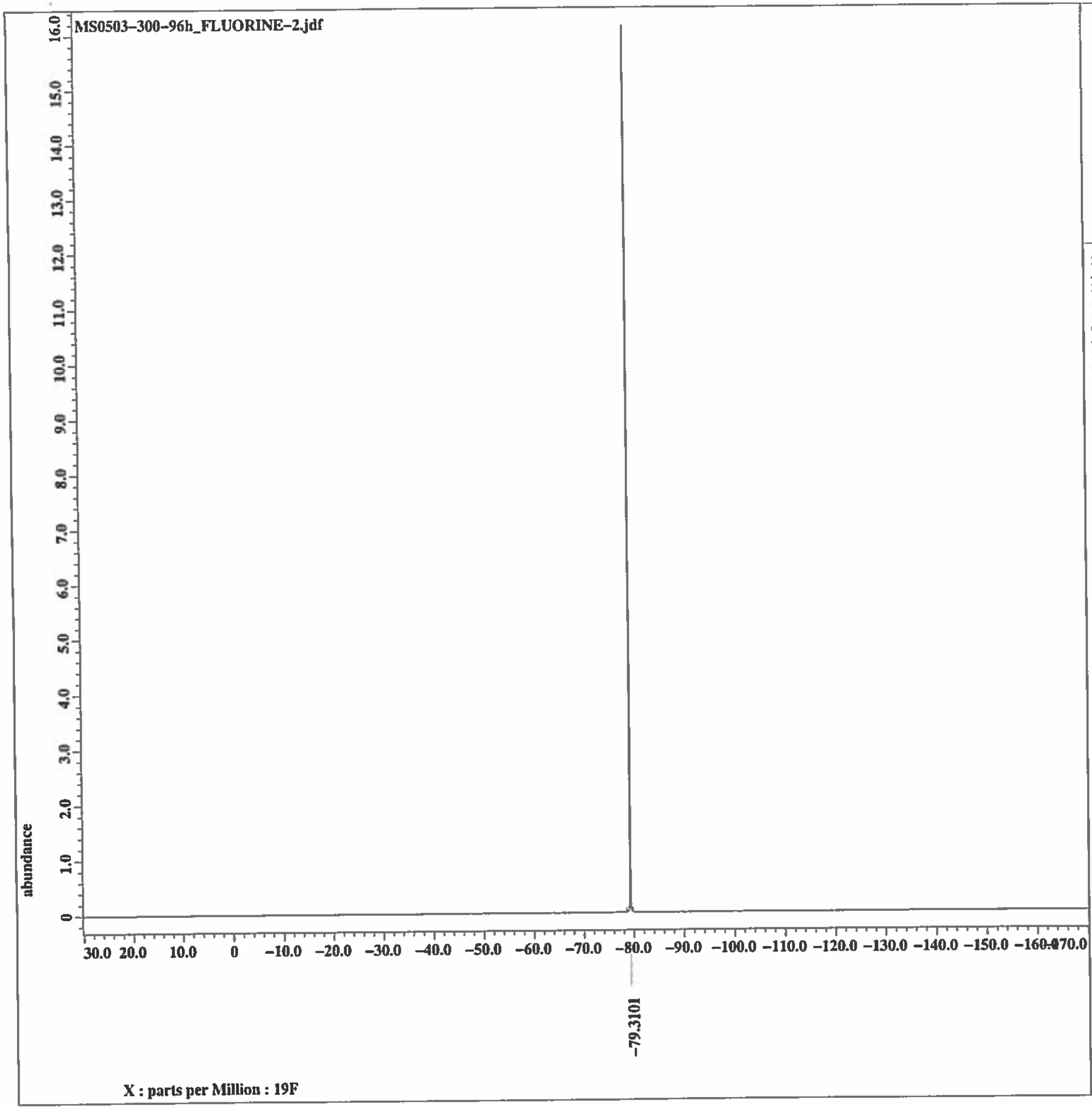

Filename	= MS0503-300-96h_FLUORI
Author	= Jim Davis
Experiment	= single_pulse.ex2
Sample_id	= MS0503-300-96h
Solvent	= CHLOROFORM-D
Changer_sample	= 6
Creation_time	= 11-JUL-2018 00:23:39
Revision_time	= 11-JUL-2018 00:01:23
Current_time	= 11-JUL-2018 00:01:23
Data_format	= 1D COMPLEX
Dim_size	= 52428
Dim_title	= 19F
Dim_units	= [ppm]
Dimensions	= X
Site	= ECA 500
Spectrometer	= JNM-ECA500
Field_strength	= 11.7473579[T] (500[MH
X_acq_duration	= 0.55574528[s]
X_domain	= 19F
X_freq	= 470.62046084[MHz]
X_offset	= -70[ppm]
X_points	= 65536
X_prescans	= 1
X_resolution	= 1.7993855[Hz]
X_sweep	= 117.9245283[kHz]
Irr_domain	= 19F
Irr_freq	= 470.62046084[MHz]
Irr_offset	= 5[ppm]
Tri_domain	= 19F
Tri_freq	= 470.62046084[MHz]
Tri_offset	= 5[ppm]
Clipped	= FALSE
Mod_return	= 1
Scans	= 16
Total_scans	= 16
X_90_width	= 13.1[us]
X_acq_time	= 0.55574528[s]
X_angle	= 45[deg]
X_atn	= 2.5[dB]
X_pulse	= 6.55[us]
Irr_mode	= Off
Tri_mode	= Off
Dante_presat	= FALSE
Initial_wait	= 1[s]
Recvr_gain	= 38
Relaxation_delay	= 4[s]
Repetition_time	= 4.55574528[s]
Temp_get	= 22.2[dC]

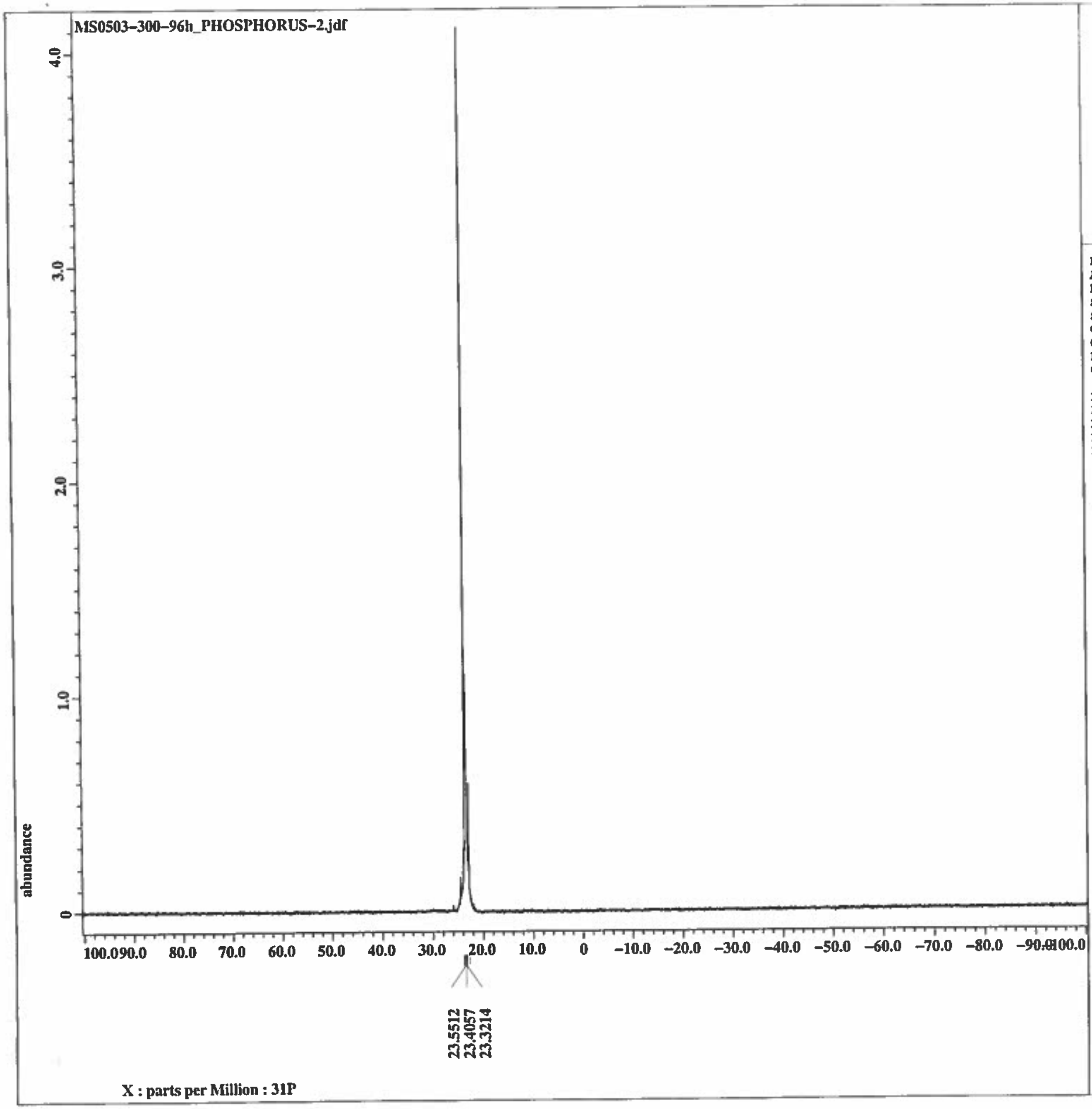

Filename	= MS0503-300-96h_PHOSPH
Author	= Jim Davis
Experiment	= single_pulse_dec
Sample_id	= MS0503-300-96h
Solvent	= CHLOROFORM-D
Changer_sample	= 6
Creation_time	= 11-JUL-2018 00:37:42
Revision_time	= 11-JUL-2018 00:15:27
Current_time	= 11-JUL-2018 00:15:27
Data_format	= 1D COMPLEX
Dim_size	= 26214
Dim_title	= 31P
Dim_units	= [ppm]
Dimensions	= X
Site	= ECA 500
Spectrometer	= JNM-ECA500
Field_strength	= 11.7473579[T] (500[MH
X_acq_duration	= 0.64487424[s]
X_domain	= 31P
X_freq	= 202.46831075[MHz]
X_offset	= 0[ppm]
X_points	= 32768
X_prescans	= 4
X_resolution	= 1.55068995[Hz]
X_sweep	= 50.81300813[kHz]
Irr_domain	= 1H
Irr_freq	= 500.15991521[MHz]
Irr_offset	= 5.0[ppm]
Clipped	= FALSE
Mod_return	= 1
Scans	= 256
Total_scans	= 256
X_90_width	= 14.687[us]
X_acq_time	= 0.64487424[s]
X_angle	= 30[deg]
X_atn	= 5[dB]
X_pulse	= 4.89566667[us]
Irr_atn_dec	= 20.7[dB]
Irr_atn_noe	= 20.7[dB]
Irr_noise	= WALTZ
Decoupling	= TRUE
Initial_wait	= 1[s]
Noe	= TRUE
Noe_time	= 2[s]
Recvr_gain	= 58
Relaxation_delay	= 2[s]
Repetition_time	= 2.64487424[s]
Temp_get	= 22.7[dC]

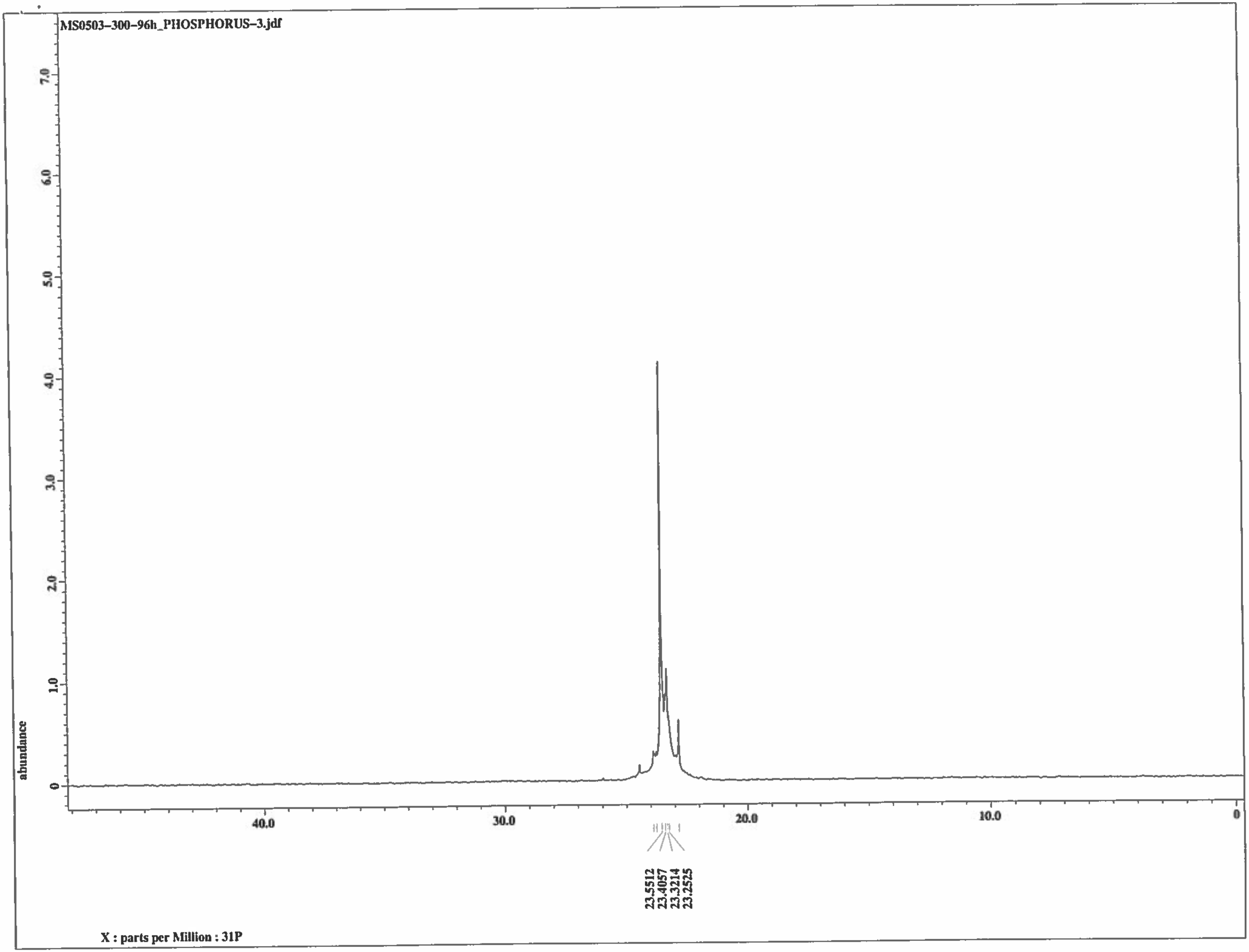

MS0503-300-96h_PHOSPHORUS-3.jdf
abundance
0
1.0
2.0
3.0
4.0
5.0
6.0
7.0
40.0
30.0
20.0
10.0
0
23.5512
23.4057
23.3214
23.2525
X : parts per Million : 31P

Compound 8 Pre- and Post-heating NMR Spectra

Temperature of Post-heating samples noted in upper left corner of each spectrum

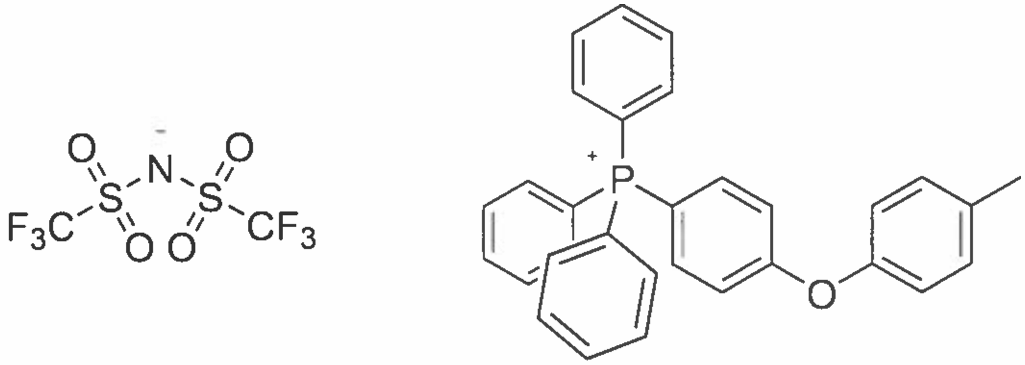

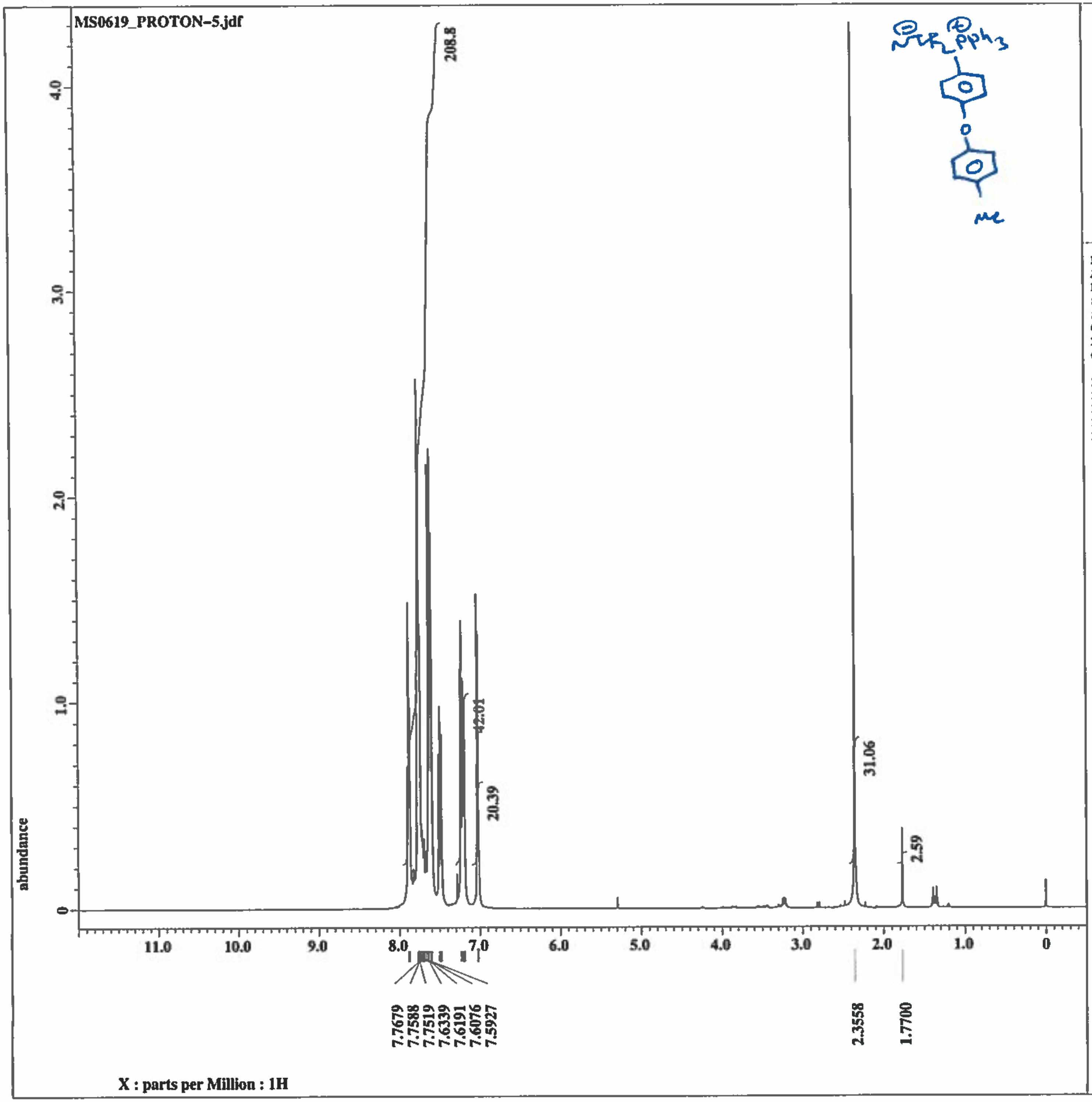

Filename	= MS0619_PROTON-5.jdf
Author	= Jim Davis
Experiment	= single_pulse.ex2
Sample_id	= MS0619
Solvent	= CHLOROFORM-D
Creation_time	= 30-NOV-2018 15:05:13
Revision_time	= 30-NOV-2018 14:40:17
Current_time	= 30-NOV-2018 14:40:17
Data_format	= 1D COMPLEX
Dim_size	= 13107
Dim_title	= 1H
Dim_units	= [ppm]
Dimensions	= X
Site	= ECA 500
Spectrometer	= JNM-ECA500
Field_strength	= 11.7473579[T] (500[MH
X_acq_duration	= 1.74587904[s]
X_domain	= 1H
X_freq	= 500.15991521[MHz]
X_offset	= 5.0[ppm]
X_points	= 16384
X_prescans	= 1
X_resolution	= 0.57277737[Hz]
X_sweep	= 9.38438438[kHz]
Irr_domain	= 1H
Irr_freq	= 500.15991521[MHz]
Irr_offset	= 5.0[ppm]
Tri_domain	= 1H
Tri_freq	= 500.15991521[MHz]
Tri_offset	= 5.0[ppm]
Clipped	= FALSE
Mod_return	= 1
Scans	= 16
Total_scans	= 16
X_90_width	= 12.4[us]
X_acq_time	= 1.74587904[s]
X_angle	= 45[deg]
X_atn	= 4[dB]
X_pulse	= 6.2[us]
Irr_mode	= Off
Tri_mode	= Off
Dante_presat	= FALSE
Initial_wait	= 1[s]
Recvr_gain	= 28
Relaxation_delay	= 4[s]
Repetition_time	= 5.74587904[s]
Temp_get	= 22.1[dC]

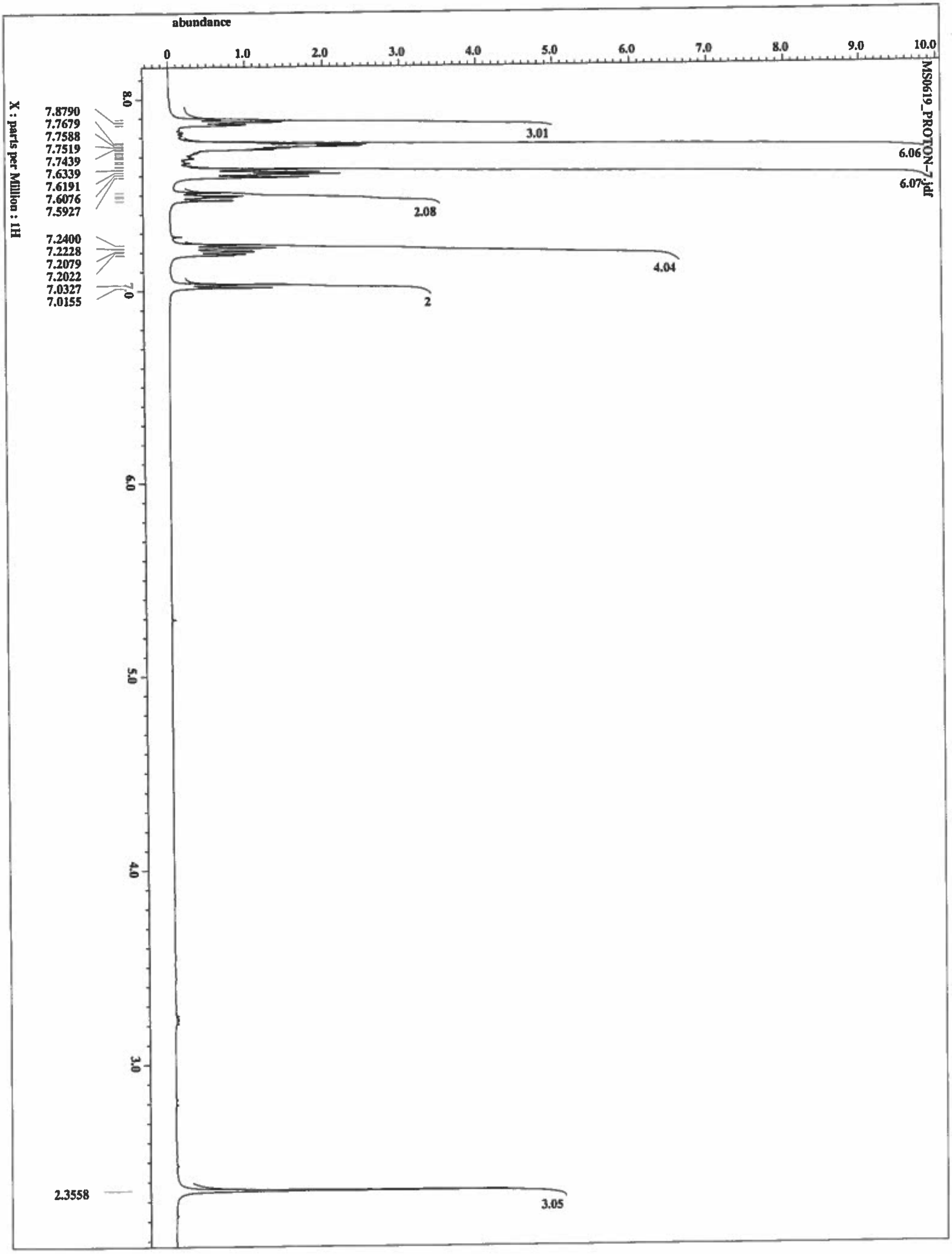

abundance
0
1.0
2.0
3.0
4.0
5.0
6.0
7.0
8.0
9.0
10.0
MS0619_PROTON-7.jdf
X : parts per Million : 1H
7.8790
7.7679
7.7588
7.7519
7.7439
7.6339
7.6191
7.6076
7.5927
7.2400
7.2228
7.2079
7.2022
7.0327
7.0155
2.3558
8.0
7.0
6.0
5.0
4.0
3.0
3.01
6.06
6.07
2.08
4.04
2
3.05

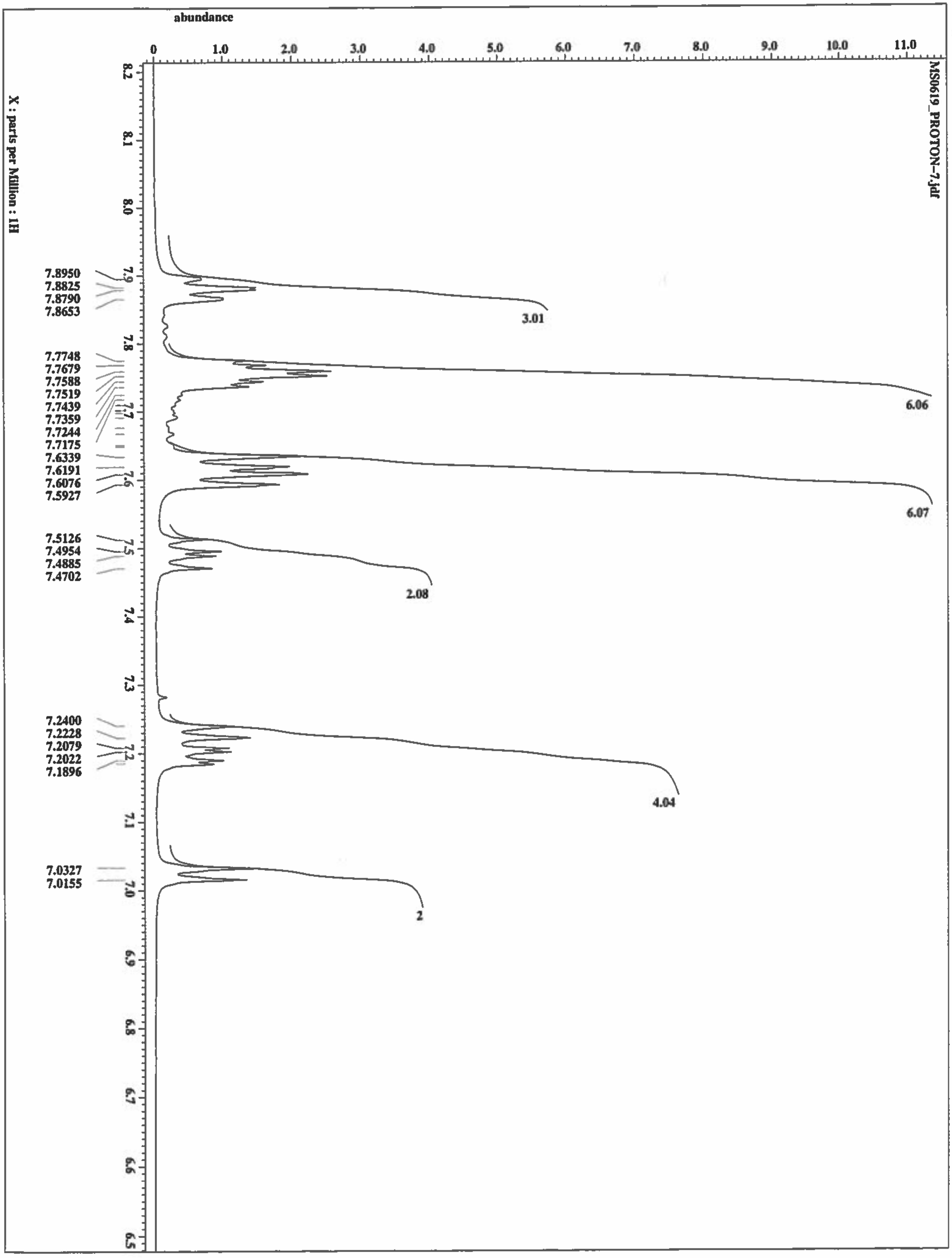

abundance
0
1.0
2.0
3.0
4.0
5.0
6.0
7.0
8.0
9.0
10.0
11.0
MS0619_PROTON-7.jdf
X : parts per Million : 1H
8.2
8.1
8.0
7.9
7.8
7.7
7.6
7.5
7.4
7.3
7.2
7.1
7.0
6.9
6.8
6.7
6.6
6.5
7.8950
7.8825
7.8790
7.8653
7.7748
7.7679
7.7588
7.7519
7.7439
7.7359
7.7244
7.7175
7.6339
7.6191
7.6076
7.5927
7.5126
7.4954
7.4885
7.4702
7.2400
7.2228
7.2079
7.2022
7.1896
7.0327
7.0155
3.01
6.06
6.07
2.08
4.04
2

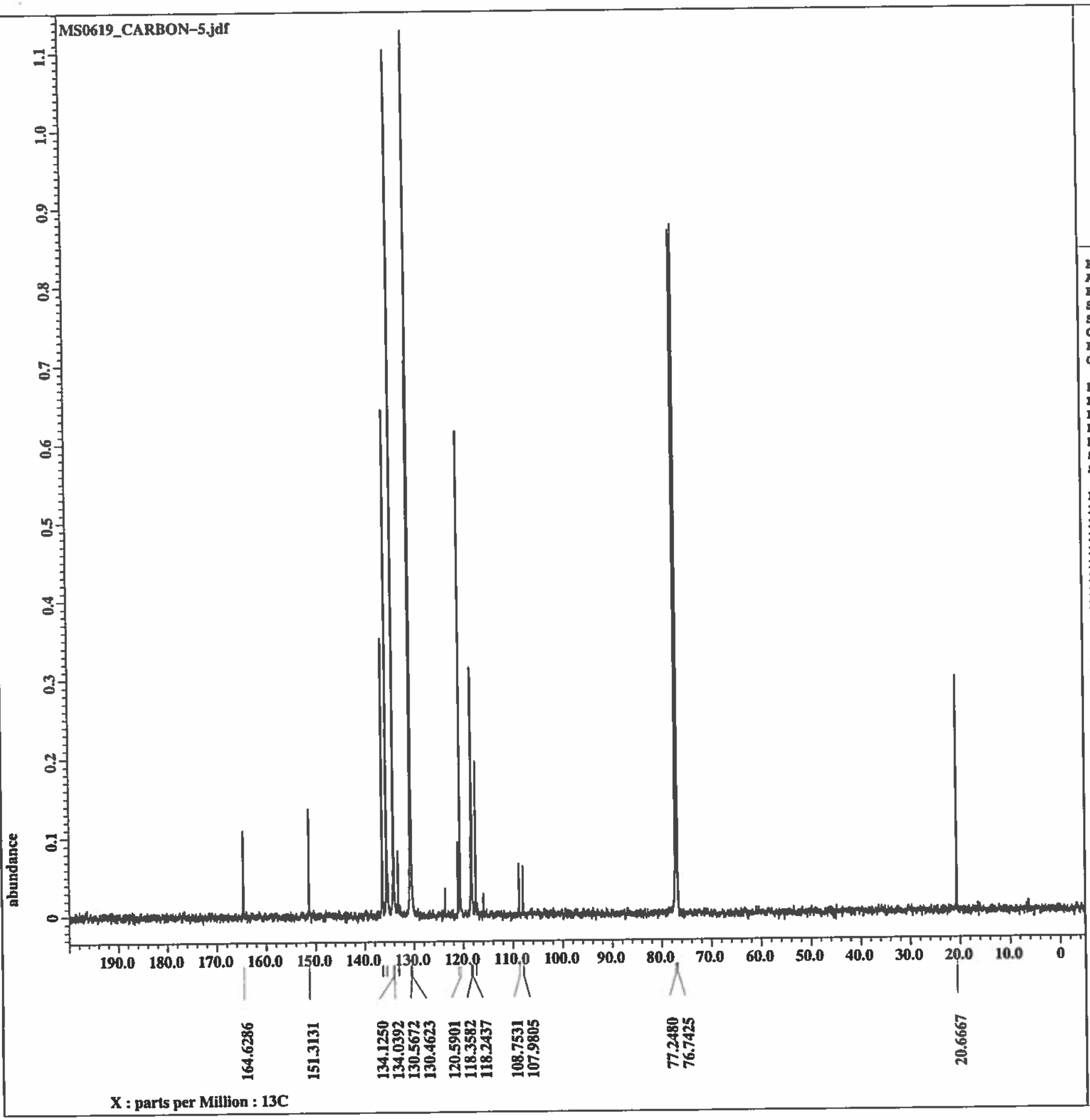

Filename	= MS0619_CARBON-5.jdf
Author	= Jim Davis
Experiment	= single_pulse_dec
Sample_id	= MS0619
Solvent	= CHLOROFORM-D
Creation_time	= 30-NOV-2018 15:26:35
Revision_time	= 30-NOV-2018 15:01:38
Current_time	= 30-NOV-2018 15:01:38
Data_format	= 1D COMPLEX
Dim_size	= 26214
Dim_title	= 13C
Dim_units	= [ppm]
Dimensions	= X
Site	= ECA 500
Spectrometer	= JNM-ECA500
Field_strength	= 11.7473579[T] (500[MH
X_acq_duration	= 0.83361792[s]
X_domain	= 13C
X_freq	= 125.76529768[MHz]
X_offset	= 100[ppm]
X_points	= 32768
X_prescans	= 4
X_resolution	= 1.19959034[Hz]
X_sweep	= 39.3081761[kHz]
Irr_domain	= 1H
Irr_freq	= 500.15991521[MHz]
Irr_offset	= 5.0[ppm]
Clipped	= FALSE
Mod_return	= 1
Scans	= 400
Total_scans	= 400
X_90_width	= 13.2[us]
X_acq_time	= 0.83361792[s]
X_angle	= 30[deg]
X_atn	= 6[dB]
X_pulse	= 4.4[us]
Irr_atn_dec	= 20.7[dB]
Irr_atn_noe	= 20.7[dB]
Irr_noise	= WALTZ
Decoupling	= TRUE
Initial_wait	= 1[s]
Noe	= TRUE
Noe_time	= 2[s]
Recvr_gain	= 60
Relaxation_delay	= 2[s]
Repetition_time	= 2.83361792[s]
Temp_get	= 23.1[dC]

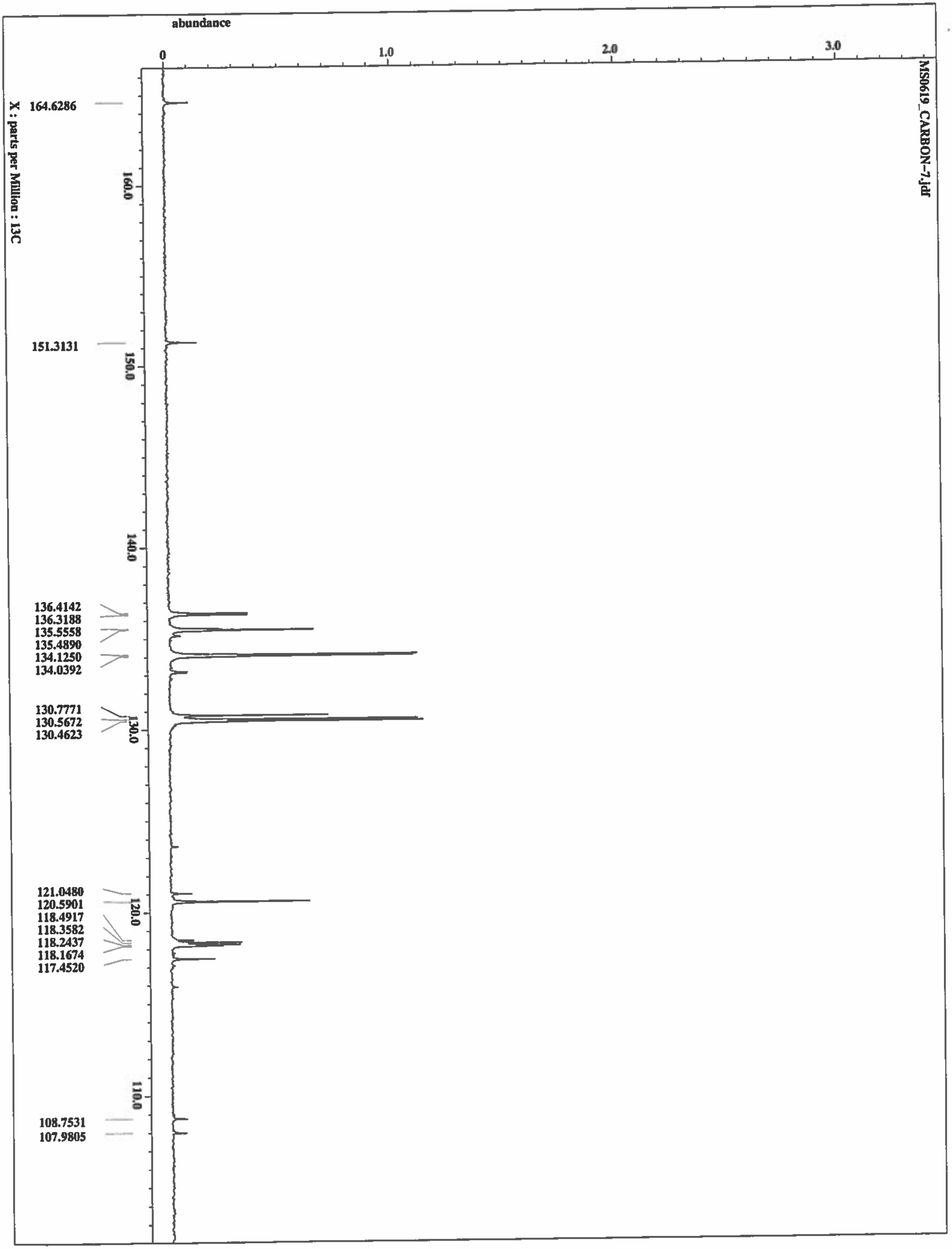

abundance
0
1.0
2.0
3.0
MS0619_CARBON-7.jdf
X : parts per Million : 13C
164.6286
160.0
151.3131
150.0
140.0
136.4142
136.3188
135.5558
135.4890
134.1250
134.0392
130.7771
130.5672
130.4623
130.0
121.0480
120.5901
118.4917
118.3582
118.2437
118.1674
117.4520
120.0
110.0
108.7531
107.9805

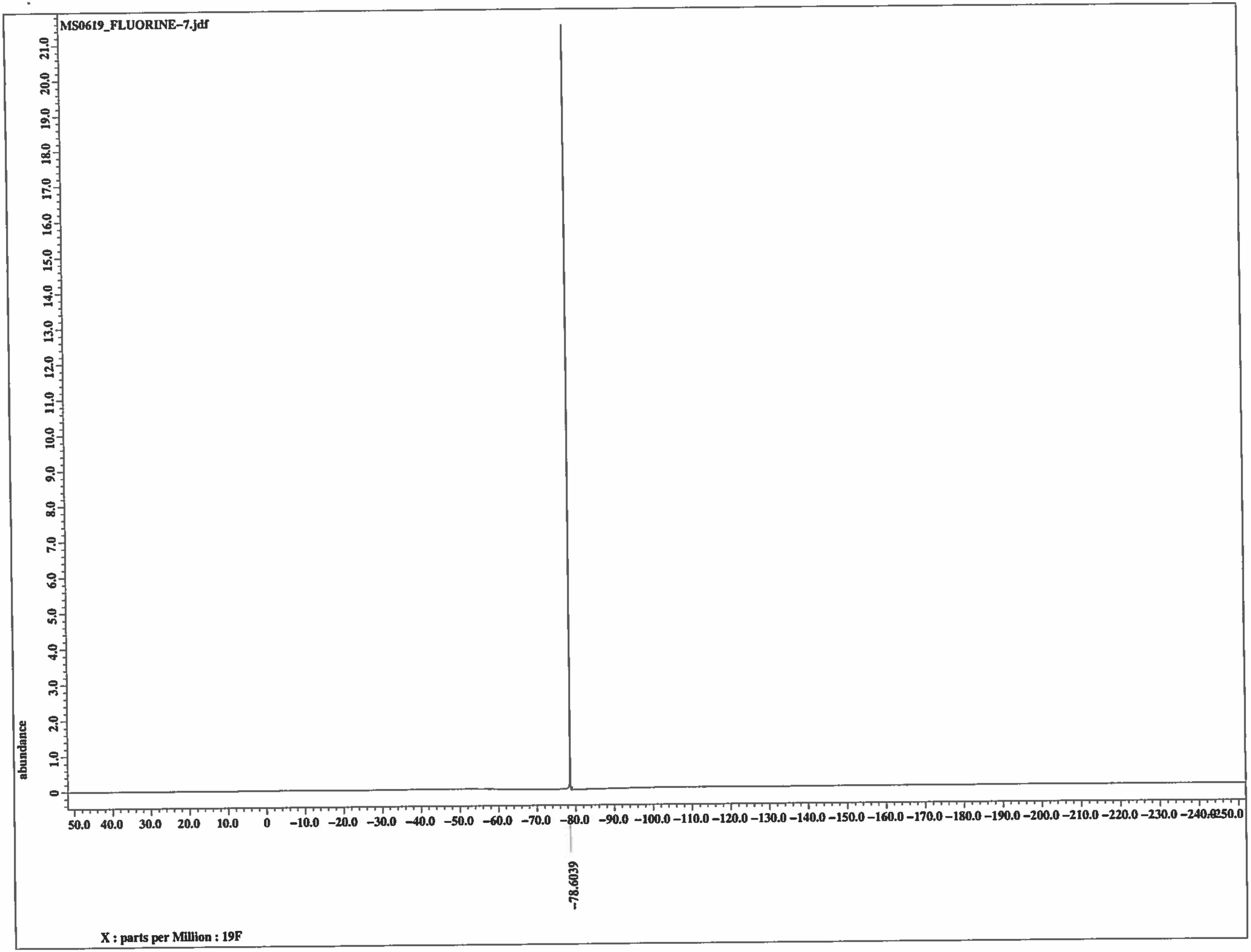
MS0619_FLUORINE-7.jdf
abundance
21.0
20.0
19.0
18.0
17.0
16.0
15.0
14.0
13.0
12.0
11.0
10.0
9.0
8.0
7.0
6.0
5.0
4.0
3.0
2.0
1.0
0
50.0 40.0 30.0 20.0 10.0 0 −10.0 −20.0 −30.0 −40.0 −50.0 −60.0 −70.0 −80.0 −90.0 −100.0 −110.0 −120.0 −130.0 −140.0 −150.0 −160.0 −170.0 −180.0 −190.0 −200.0 −210.0 −220.0 −230.0 −240.0 250.0
−78.6039
X : parts per Million : 19F

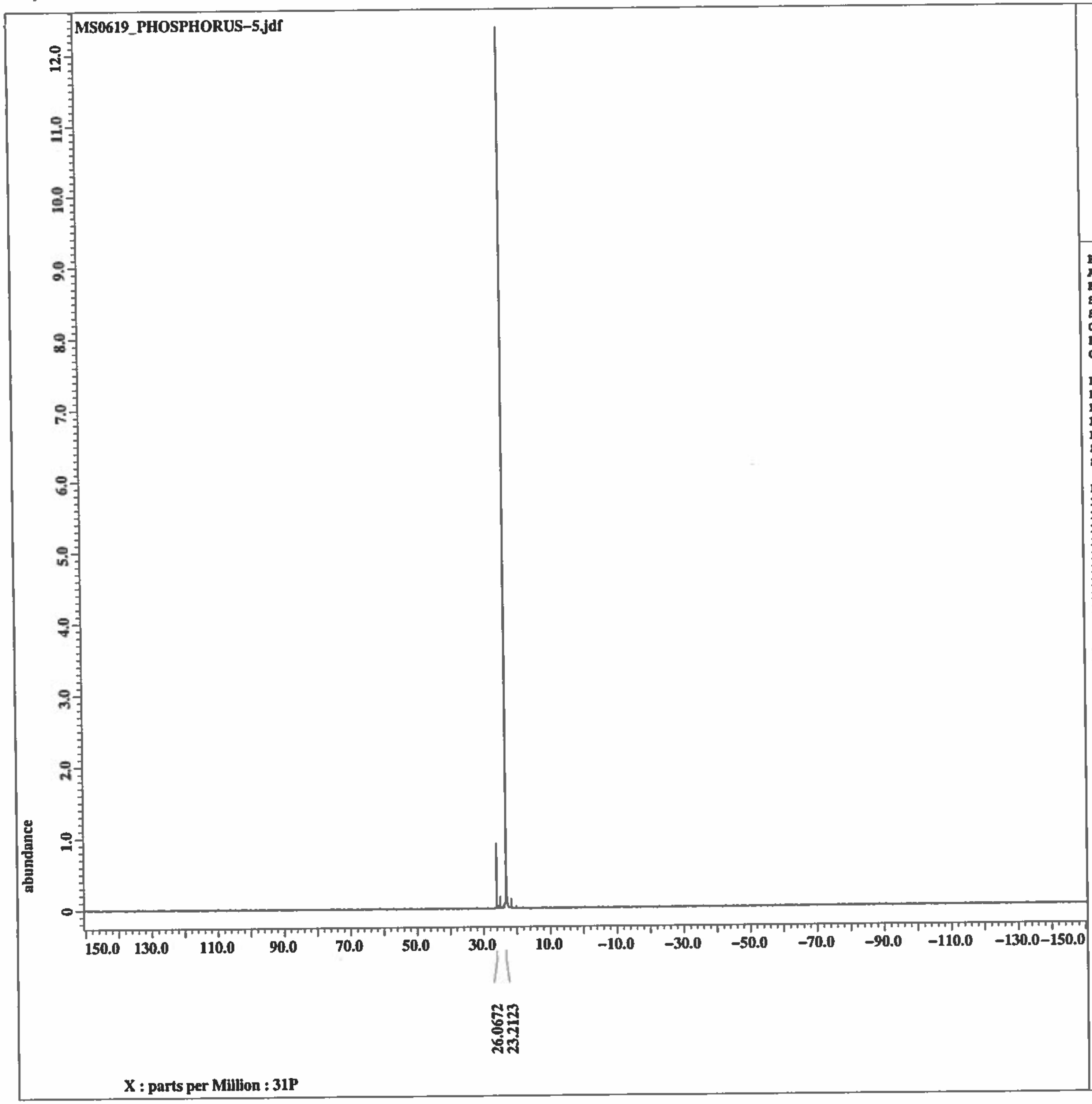

Filename	= MS0619_PHOSPHORUS-5.j
Author	= Jim Davis
Experiment	= single_pulse_dec
Sample_id	= MS0619
Solvent	= CHLOROFORM-D
Creation_time	= 30-NOV-2018 14:42:20
Revision_time	= 30-NOV-2018 14:17:23
Current_time	= 30-NOV-2018 14:17:24
Data_format	= 1D COMPLEX
Dim_size	= 52428
Dim_title	= 31P
Dim_units	= [ppm]
Dimensions	= X
Site	= ECA 500
Spectrometer	= JNM-ECA500
Field_strength	= 11.7473579[T] (500[MH
X_acq_duration	= 0.85983232[s]
X_domain	= 31P
X_freq	= 202.46831075[MHz]
X_offset	= 0[ppm]
X_points	= 65536
X_prescans	= 4
X_resolution	= 1.16301746[Hz]
X_sweep	= 76.2195122[kHz]
Irr_domain	= 1H
Irr_freq	= 500.15991521[MHz]
Irr_offset	= 5.0[ppm]
Clipped	= FALSE
Mod_return	= 1
Scans	= 50
Total_scans	= 50
X_90_width	= 14.687[us]
X_acq_time	= 0.85983232[s]
X_angle	= 30[deg]
X_atn	= 5[dB]
X_pulse	= 4.89566667[us]
Irr_atn_dec	= 20.7[dB]
Irr_atn_noe	= 20.7[dB]
Irr_noise	= WALTZ
Decoupling	= TRUE
Initial_wait	= 1[s]
Noe	= TRUE
Noe_time	= 2[s]
Recvr_gain	= 56
Relaxation_delay	= 2[s]
Repetition_time	= 2.85983232[s]
Temp_get	= 22.7[dC]

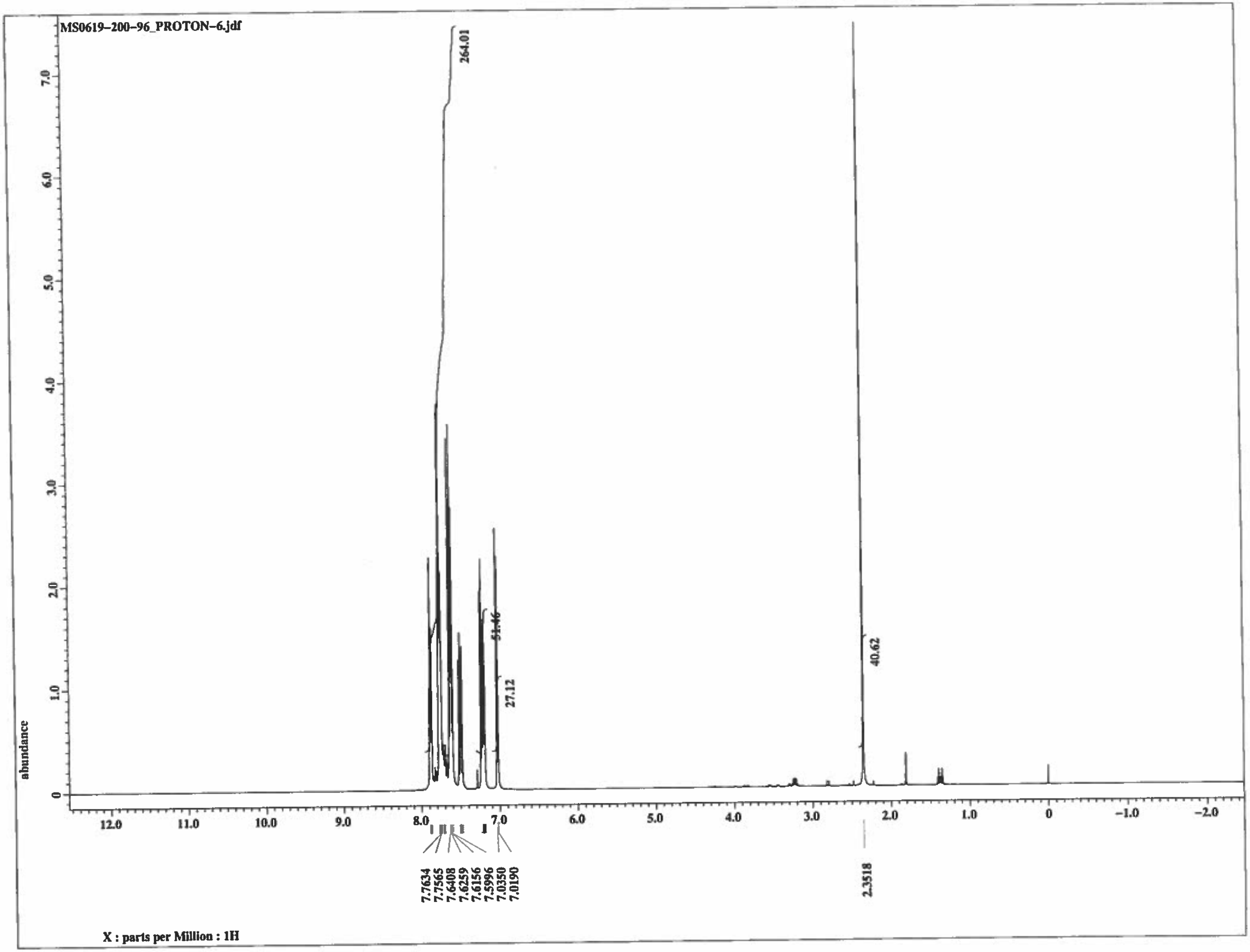

MS0619-200-96_PROTON-6.jdf
abundance
264.01
51.46
27.12
40.62
7.7634
7.7565
7.6408
7.6259
7.6156
7.5996
7.0350
7.0190
2.3518
X : parts per Million : 1H

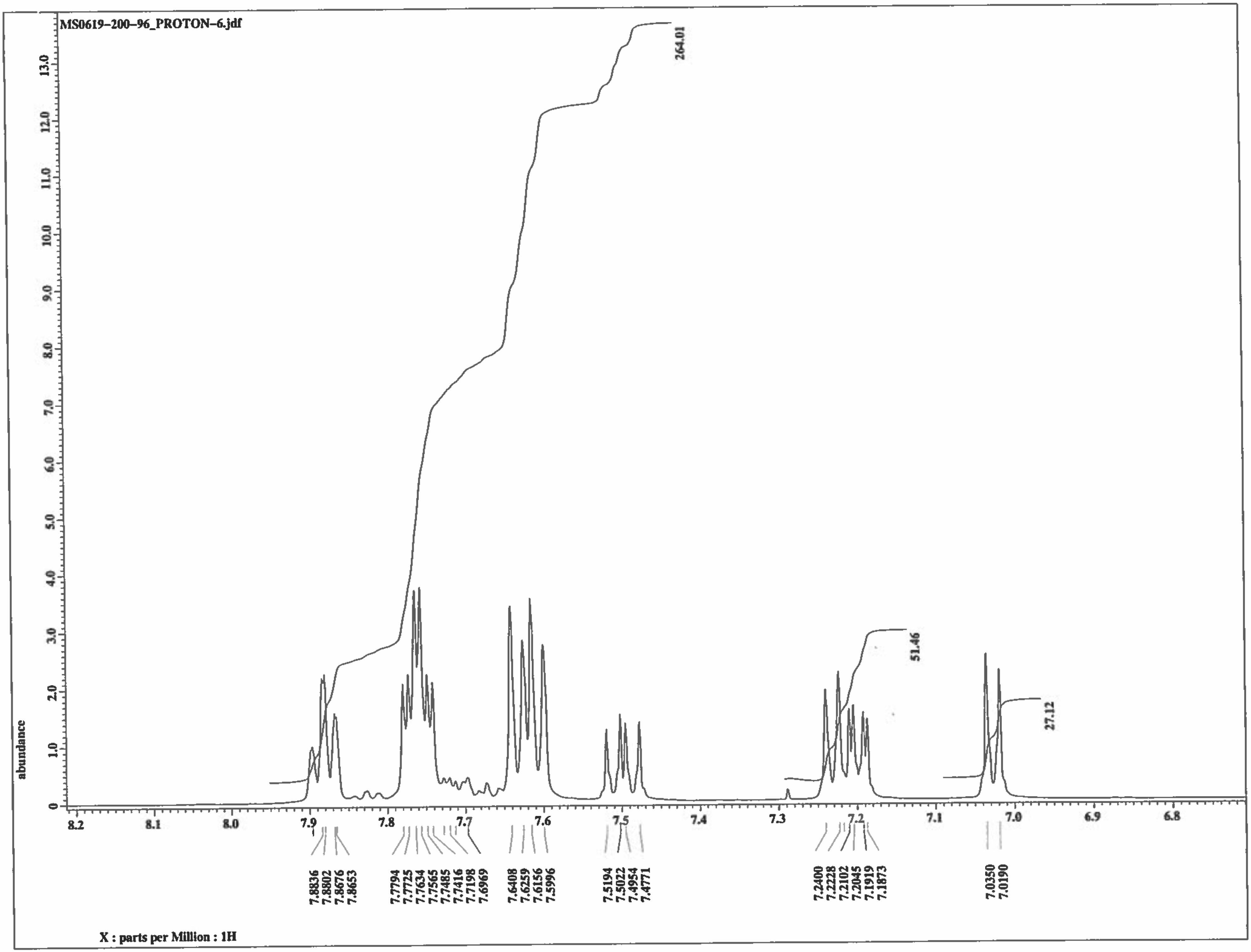

MS0619-200-96_PROTON-6.jdf
abundance
0
1.0
2.0
3.0
4.0
5.0
6.0
7.0
8.0
9.0
10.0
11.0
12.0
13.0
264.01
51.46
27.12
8.2
8.1
8.0
7.9
7.8
7.7
7.6
7.5
7.4
7.3
7.2
7.1
7.0
6.9
6.8
7.8836
7.8802
7.8676
7.8653
7.7794
7.7725
7.7634
7.7565
7.7485
7.7416
7.7198
7.6969
7.6408
7.6259
7.6156
7.5996
7.5194
7.5022
7.4954
7.4771
7.2400
7.2228
7.2102
7.2045
7.1919
7.1873
7.0350
7.0190
X : parts per Million : 1H

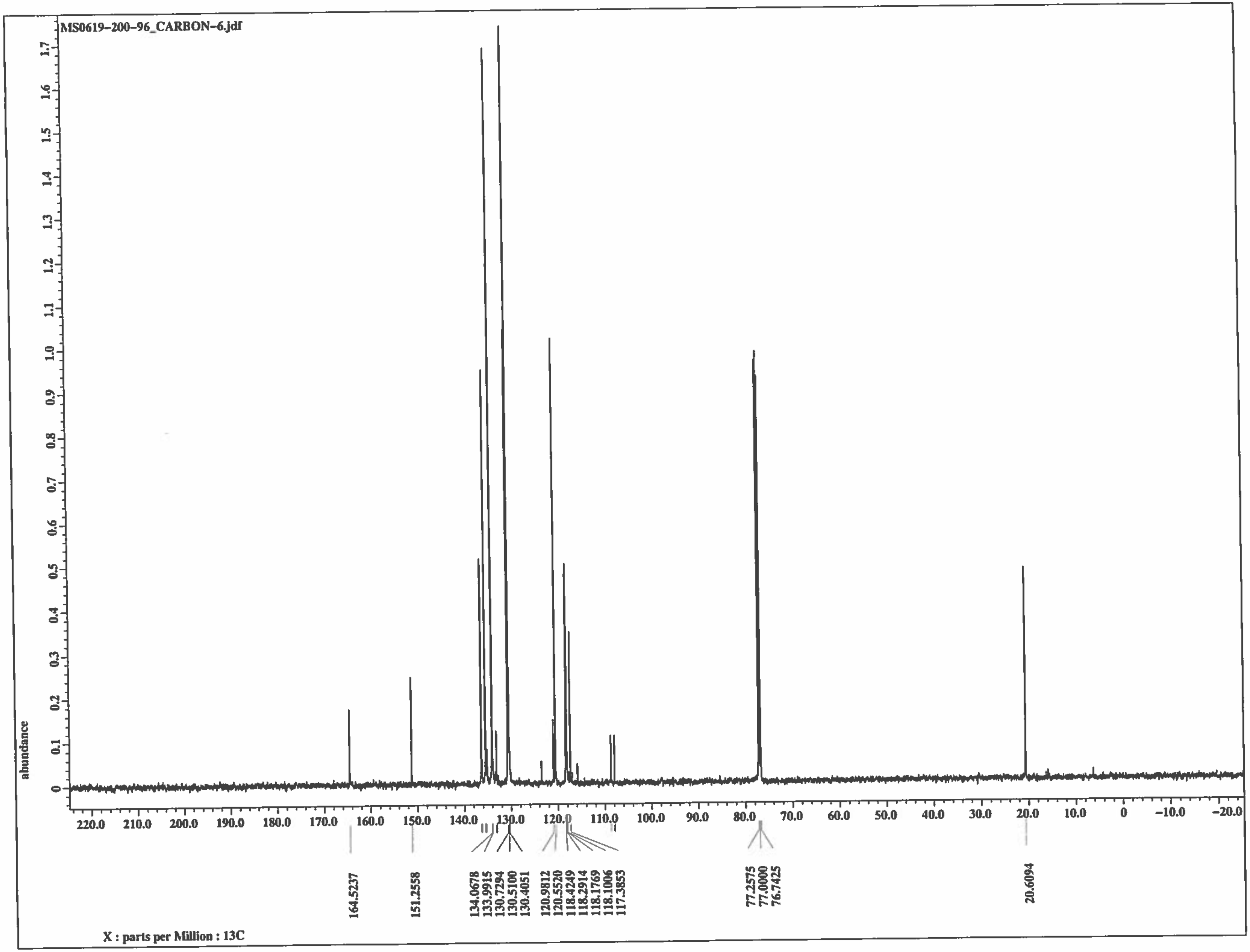

MS0619-200-96_CARBON-6.jdf
abundance
X : parts per Million : 13C
164.5237
151.2558
134.0678
133.9915
130.7294
130.5100
130.4051
120.9812
120.5520
118.4249
118.2914
118.1769
118.1006
117.3853
77.2575
77.0000
76.7425
20.6094

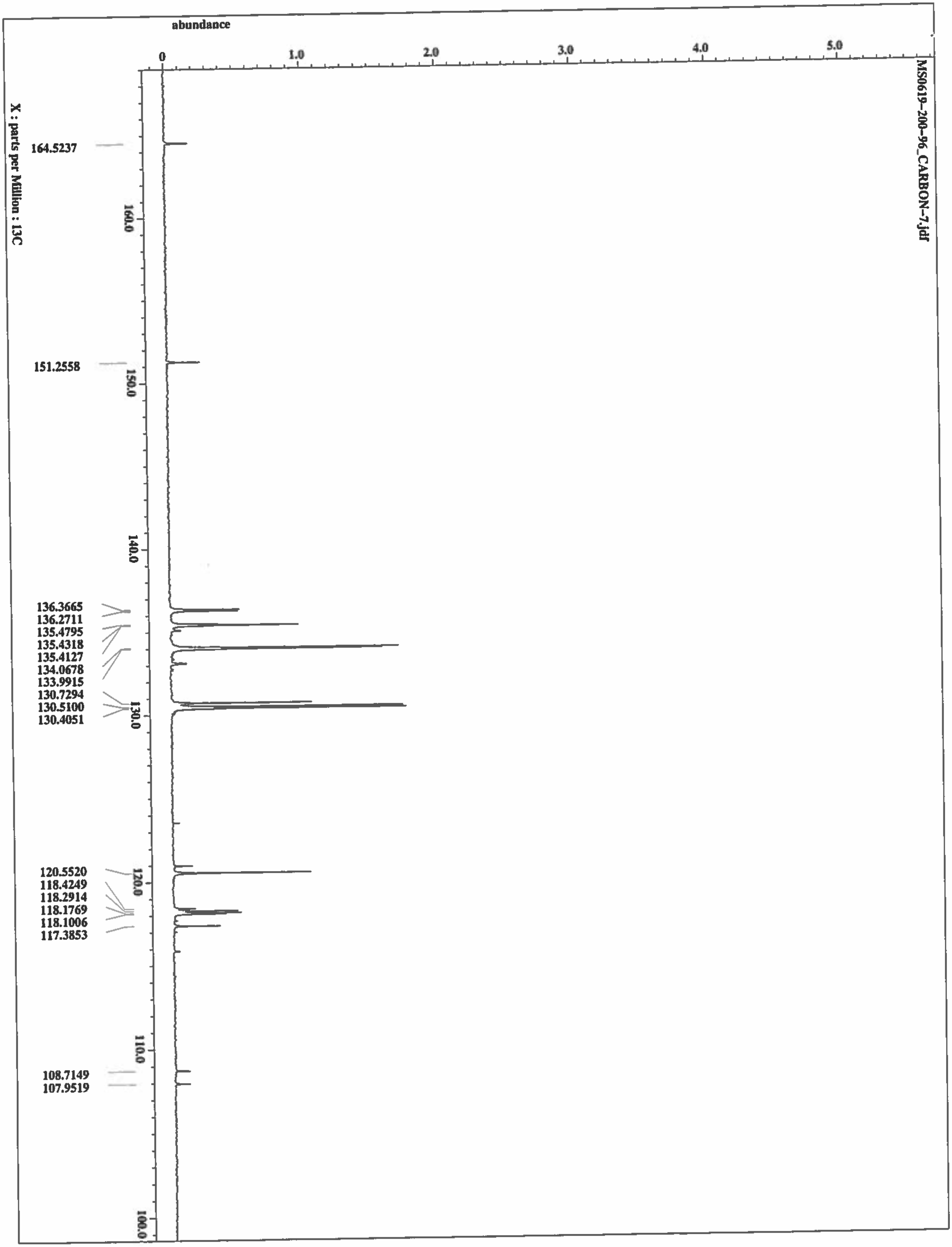

MS0619-200-96_CARBON-7.jdf
abundance
0
1.0
2.0
3.0
4.0
5.0
X : parts per Million : 13C
160.0
150.0
140.0
130.0
120.0
110.0
100.0
164.5237
151.2558
136.3665
136.2711
135.4795
135.4318
135.4127
134.0678
133.9915
130.7294
130.5100
130.4051
120.5520
118.4249
118.2914
118.1769
118.1006
117.3853
108.7149
107.9519

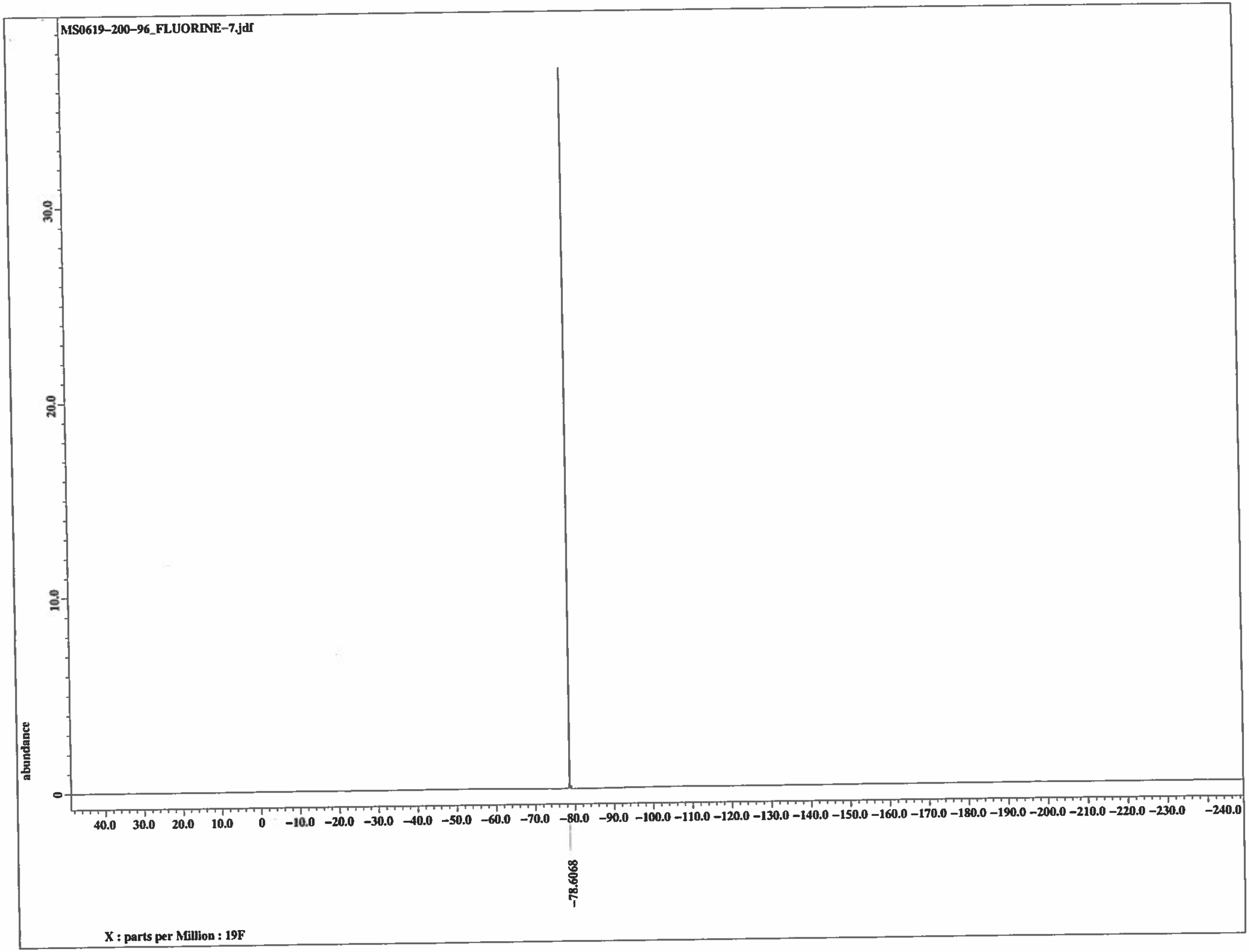
MS0619-200-96_FLUORINE-7.jdf
abundance
0
10.0
20.0
30.0
40.0 30.0 20.0 10.0 0 -10.0 -20.0 -30.0 -40.0 -50.0 -60.0 -70.0 -80.0 -90.0 -100.0 -110.0 -120.0 -130.0 -140.0 -150.0 -160.0 -170.0 -180.0 -190.0 -200.0 -210.0 -220.0 -230.0 -240.0
-78.6068
X : parts per Million : 19F

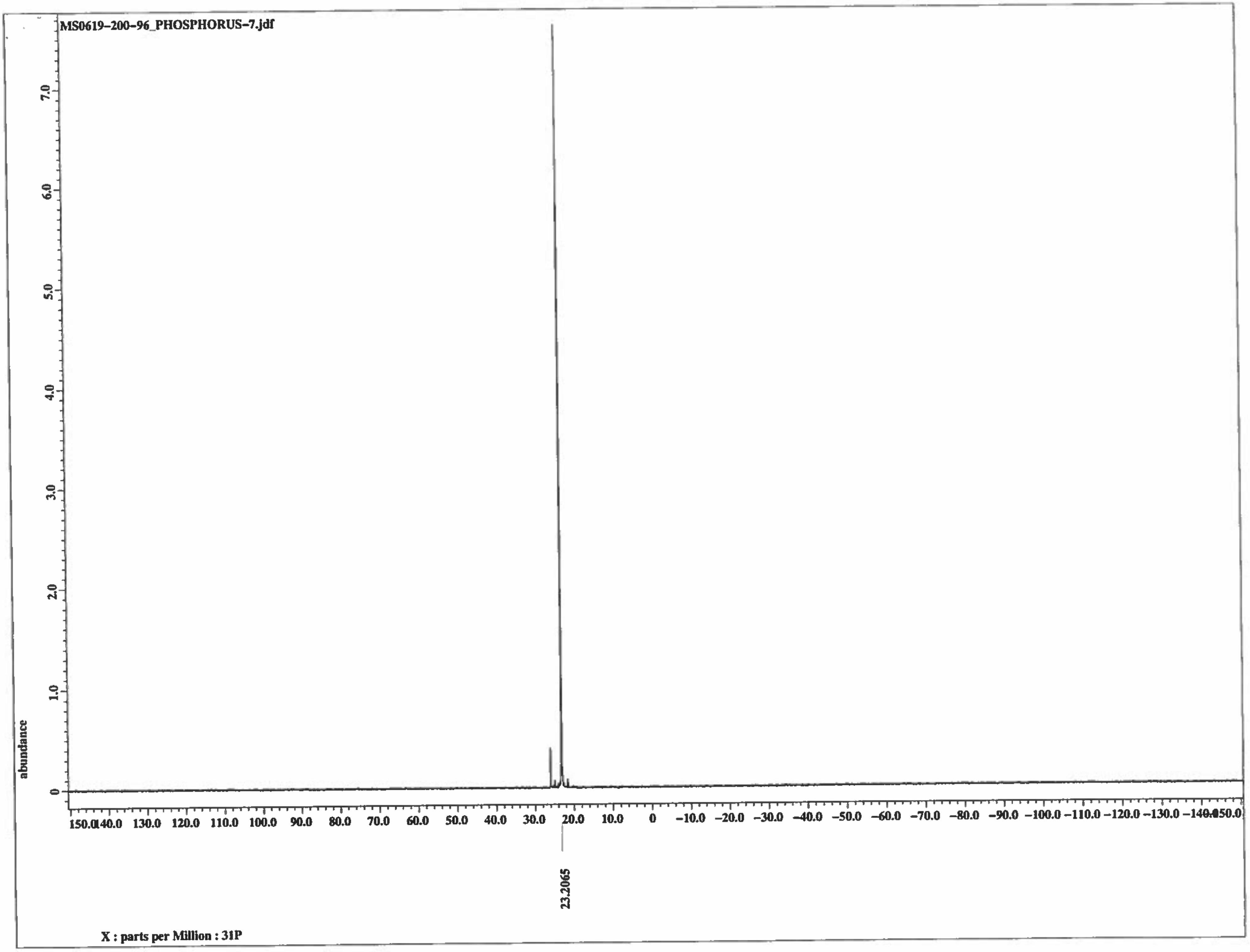

MS0619-200-96_PHOSPHORUS-7.jdf
abundance
0
1.0
2.0
3.0
4.0
5.0
6.0
7.0
150.0 140.0 130.0 120.0 110.0 100.0 90.0 80.0 70.0 60.0 50.0 40.0 30.0 20.0 10.0 0 −10.0 −20.0 −30.0 −40.0 −50.0 −60.0 −70.0 −80.0 −90.0 −100.0 −110.0 −120.0 −130.0 −140.0 −150.0
23.2065
X : parts per Million : 31P

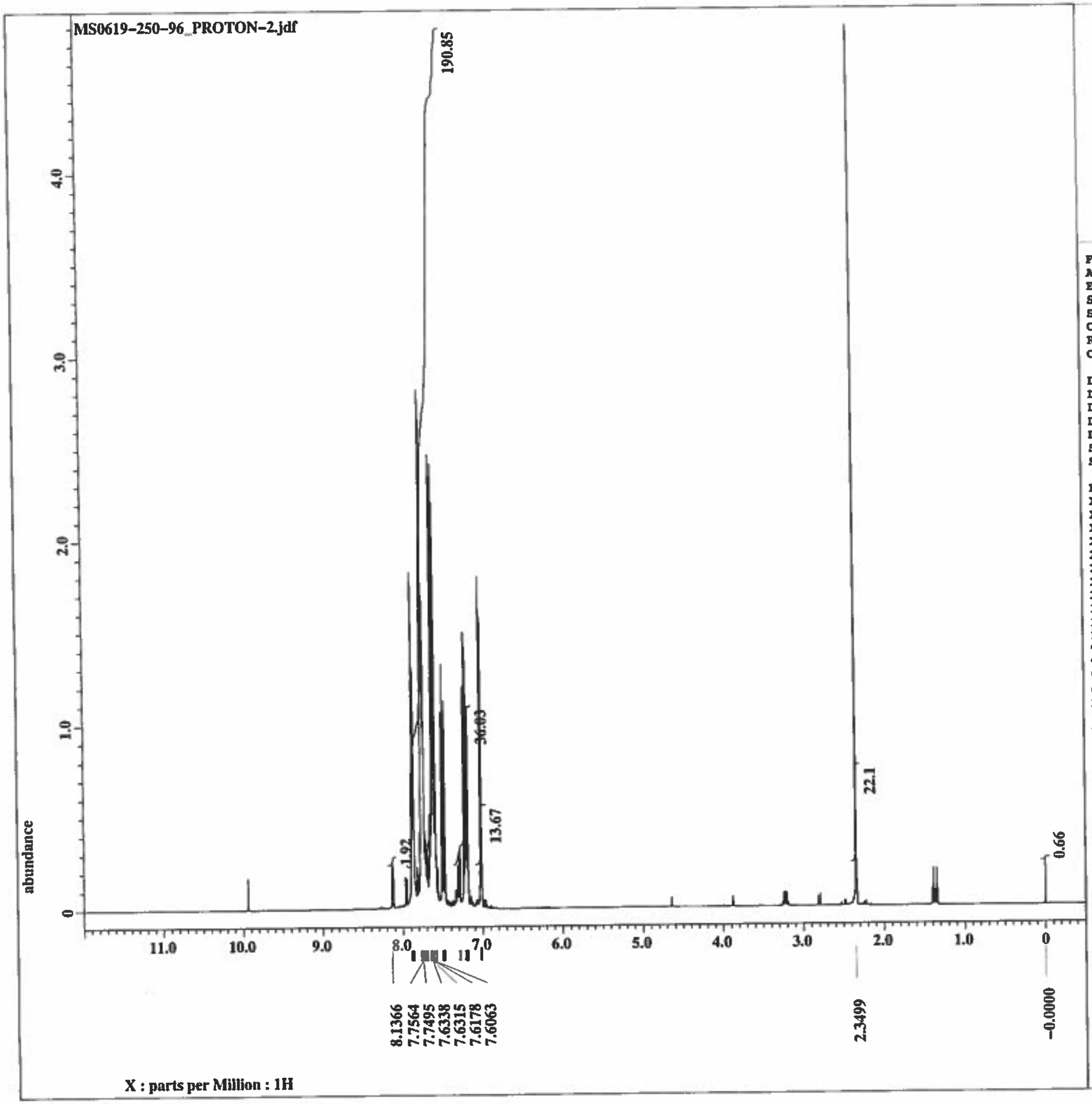

Filename	= MS0619-250-96_PROTON-
Author	= Jim Davis
Experiment	= single_pulse.ex2
Sample_id	= MS0619-250-96
Solvent	= CHLOROFORM-D
Creation_time	= 4-DEC-2018 12:07:09
Revision_time	= 4-DEC-2018 11:41:52
Current_time	= 4-DEC-2018 11:41:52
Data_format	= 1D COMPLEX
Dim_size	= 13107
Dim_title	= 1H
Dim_units	= [ppm]
Dimensions	= X
Site	= ECA 500
Spectrometer	= JNM-ECA500
Field_strength	= 11.7473579[T] (500[MH
X_acq_duration	= 1.74587904[s]
X_domain	= 1H
X_freq	= 500.15991521[MHz]
X_offset	= 5.0[ppm]
X_points	= 16384
X_prescans	= 1
X_resolution	= 0.57277737[Hz]
X_sweep	= 9.38438438[kHz]
Irr_domain	= 1H
Irr_freq	= 500.15991521[MHz]
Irr_offset	= 5.0[ppm]
Tri_domain	= 1H
Tri_freq	= 500.15991521[MHz]
Tri_offset	= 5.0[ppm]
Clipped	= FALSE
Mod_return	= 1
Scans	= 16
Total_scans	= 16
X_90_width	= 12.4[us]
X_acq_time	= 1.74587904[s]
X_angle	= 45[deg]
X_atn	= 4[dB]
X_pulse	= 6.2[us]
Irr_mode	= Off
Tri_mode	= Off
Dante_presat	= FALSE
Initial_wait	= 1[s]
Recvr_gain	= 30
Relaxation_delay	= 4[s]
Repetition_time	= 5.74587904[s]
Temp_get	= 20.6[dC]

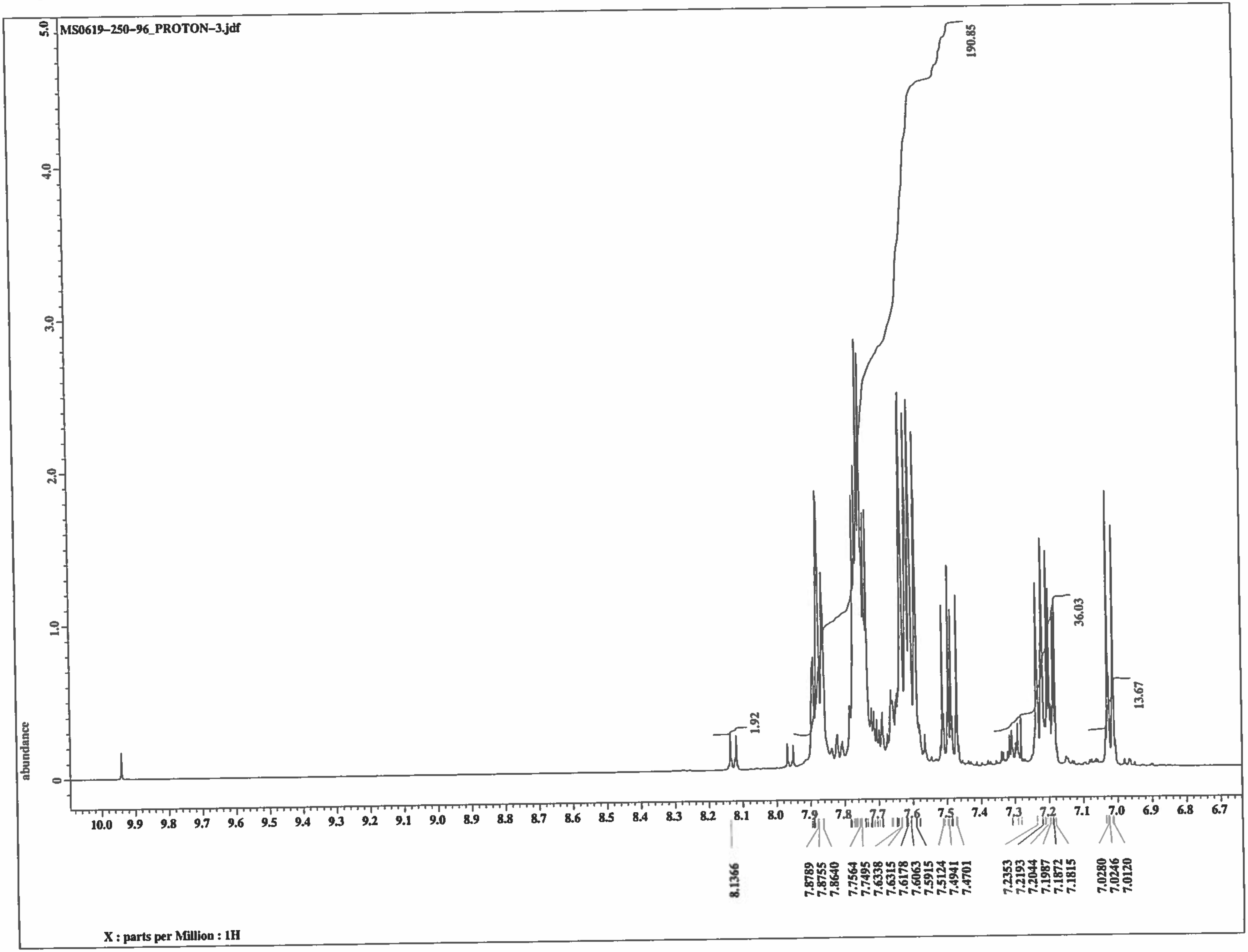
MS0619-250-96_PROTON-3.jdf
abundance
X : parts per Million : 1H
190.85
36.03
13.67
1.92
8.1366
7.8789
7.8755
7.8640
7.7564
7.7495
7.6338
7.6315
7.6178
7.6063
7.5915
7.5124
7.4941
7.4701
7.2353
7.2193
7.2044
7.1987
7.1872
7.1815
7.0280
7.0246
7.0120

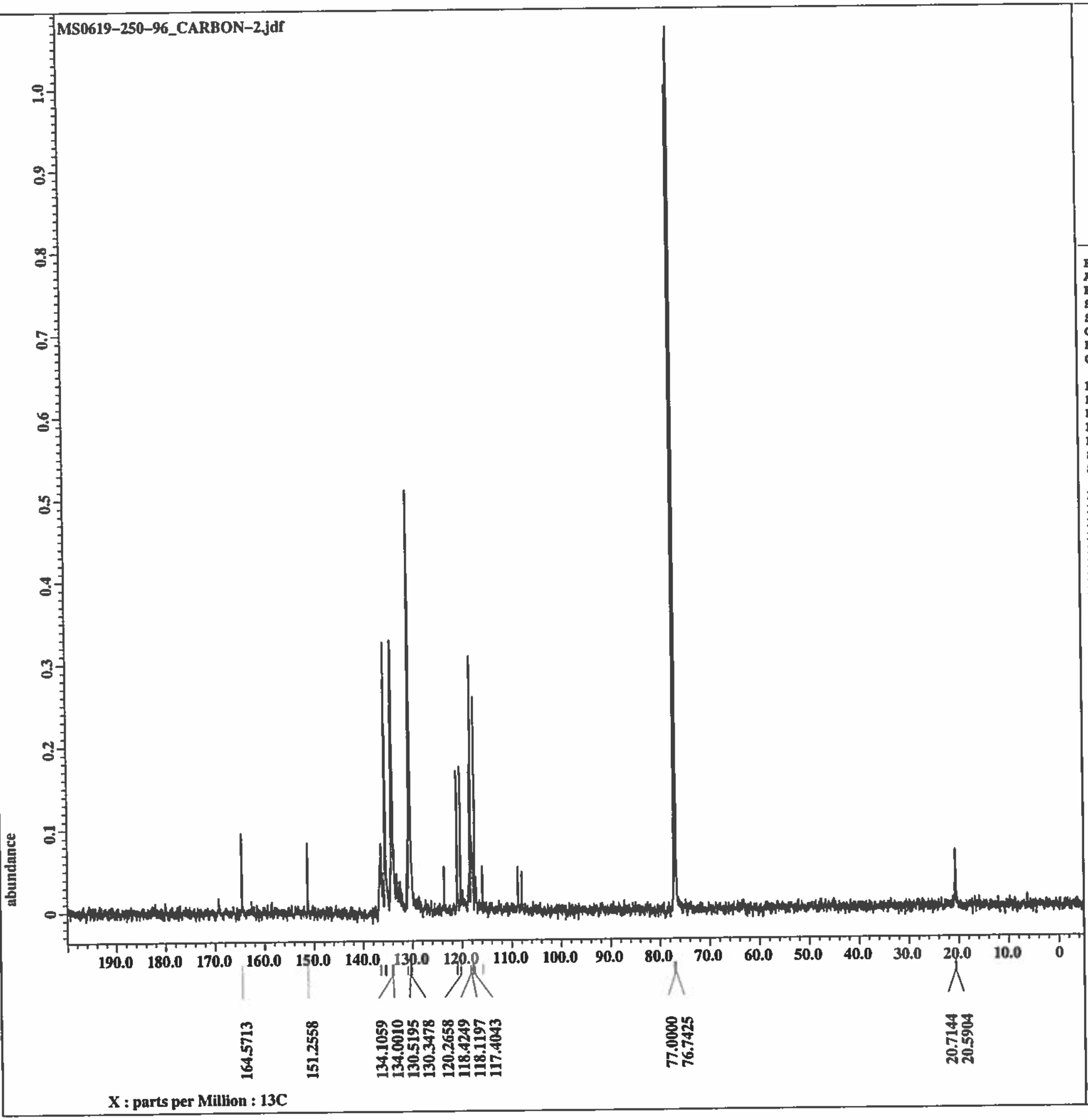

Filename	= MS0619-250-96_CARBON-
Author	= Jim Davis
Experiment	= single_pulse_dec
Sample_id	= MS0619-250-96
Solvent	= CHLOROFORM-D
Creation_time	= 4-DEC-2018 12:21:52
Revision_time	= 4-DEC-2018 11:56:35
Current_time	= 4-DEC-2018 11:56:35
Data_format	= 1D COMPLEX
Dim_size	= 26214
Dim_title	= 13C
Dim_units	= [ppm]
Dimensions	= X
Site	= ECA 500
Spectrometer	= JNM-ECA500
Field_strength	= 11.7473579[T] (500[MH
X_acq_duration	= 0.83361792[s]
X_domain	= 13C
X_freq	= 125.76529768[MHz]
X_offset	= 100[ppm]
X_points	= 32768
X_prescans	= 4
X_resolution	= 1.19959034[Hz]
X_sweep	= 39.3081761[kHz]
Irr_domain	= 1H
Irr_freq	= 500.15991521[MHz]
Irr_offset	= 5.0[ppm]
Clipped	= FALSE
Mod_return	= 1
Scans	= 256
Total_scans	= 256
X_90_width	= 13.2[us]
X_acq_time	= 0.83361792[s]
X_angle	= 30[deg]
X_atn	= 6[dB]
X_pulse	= 4.4[us]
Irr_atn_dec	= 20.7[dB]
Irr_atn_noe	= 20.7[dB]
Irr_noise	= WALTZ
Decoupling	= TRUE
Initial_wait	= 1[s]
Noe	= TRUE
Noe_time	= 2[s]
Recvr_gain	= 60
Relaxation_delay	= 2[s]
Repetition_time	= 2.83361792[s]
Temp_get	= 20.7[dC]

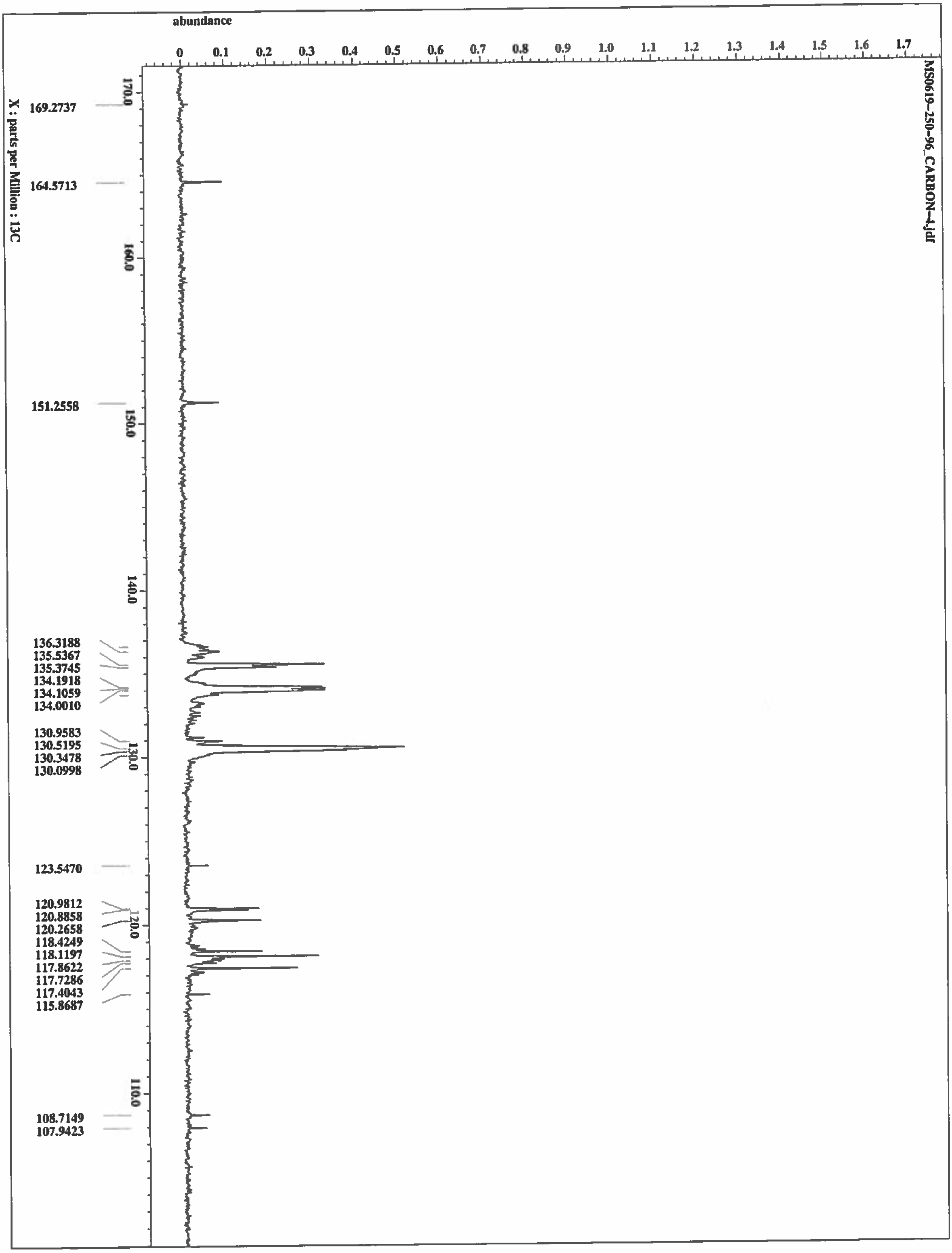
abundance
0
0.1
0.2
0.3
0.4
0.5
0.6
0.7
0.8
0.9
1.0
1.1
1.2
1.3
1.4
1.5
1.6
1.7
MS0619-250-96_CARBON-4.jdf
X : parts per Million : 13C
170.0
160.0
150.0
140.0
130.0
120.0
110.0
169.2737
164.5713
151.2558
136.3188
135.5367
135.3745
134.1918
134.1059
134.0010
130.9583
130.5195
130.3478
130.0998
123.5470
120.9812
120.8858
120.2658
118.4249
118.1197
117.8622
117.7286
117.4043
115.8687
108.7149
107.9423

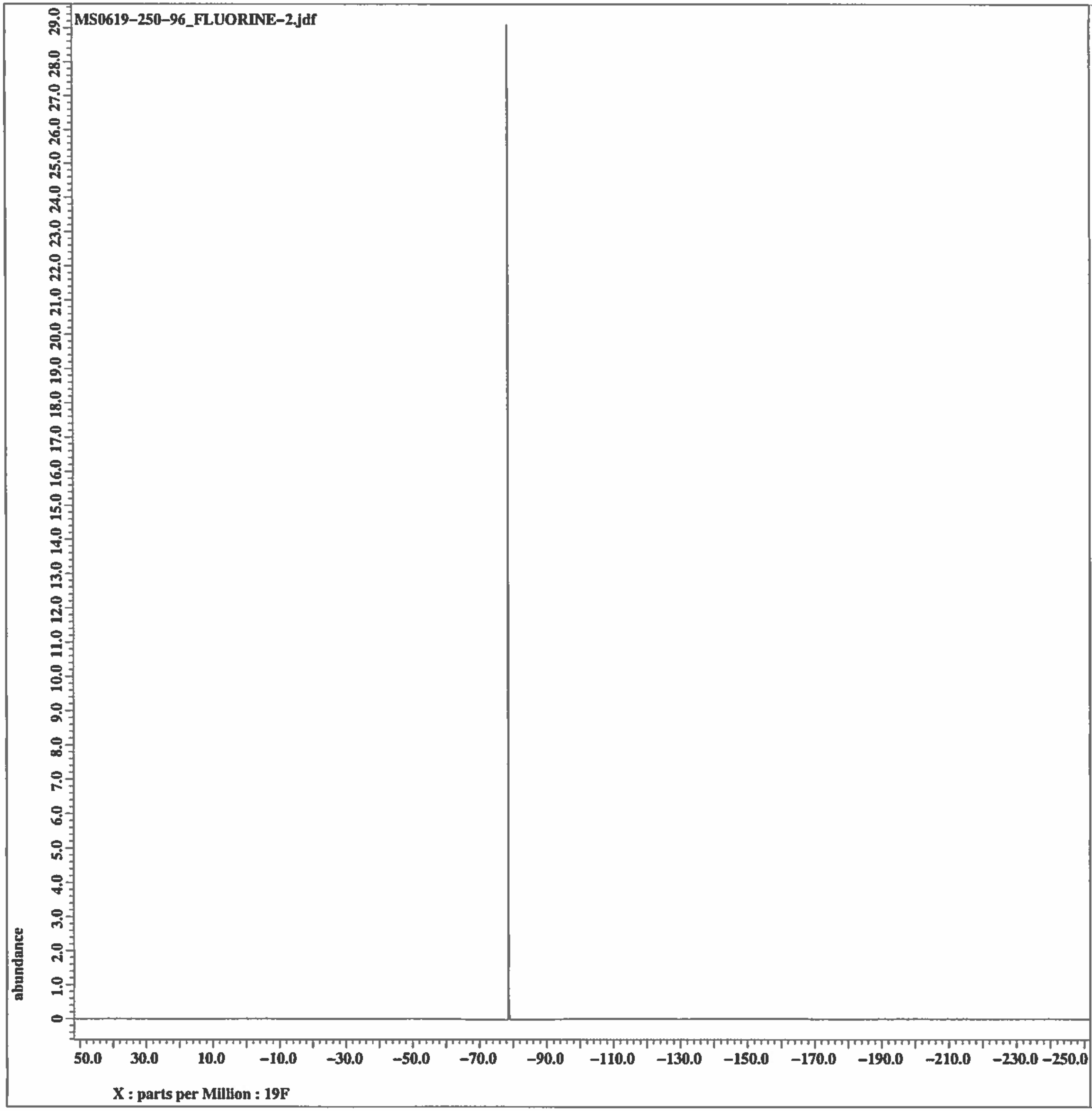

SOUTH ALABAMA JAGUARS

```
Filename            = MS0619-250-96_FLUORIN
Author              = Jim Davis
Experiment          = single_pulse.ex2
Sample_id           = MS0619-250-96
Solvent             = CHLOROFORM-D
Creation_time       =  4-DEC-2018 12:30:09
Revision_time       =  4-DEC-2018 12:04:53
Current_time        =  4-DEC-2018 12:04:53

Data_format         = 1D COMPLEX
Dim_size            = 104857
Dim_title           = 19F
Dim_units           = [ppm]
Dimensions          = X
Site                = ECA 500
Spectrometer        = JNM-ECA500

Field_strength      = 11.7473579[T] (500[MH
X_acq_duration      = 0.7340032[s]
X_domain            = 19F
X_freq              = 470.62046084[MHz]
X_offset            = -100[ppm]
X_points            = 131072
X_prescans          = 1
X_resolution        = 1.36239188[Hz]
X_sweep             = 178.57142857[kHz]
Irr_domain          = 19F
Irr_freq            = 470.62046084[MHz]
Irr_offset          = 5[ppm]
Tri_domain          = 19F
Tri_freq            = 470.62046084[MHz]
Tri_offset          = 5[ppm]
Clipped             = FALSE
Mod_return          = 1
Scans               = 70
Total_scans         = 70

X_90_width          = 13.1[us]
X_acq_time          = 0.7340032[s]
X_angle             = 45[deg]
X_atn               = 2.5[dB]
X_pulse             = 6.55[us]
Irr_mode            = Off
Tri_mode            = Off
Dante_presat        = FALSE
Initial_wait        = 1[s]
Recvr_gain          = 66
Relaxation_delay    = 4[s]
Repetition_time     = 4.7340032[s]
Temp_get            = 20.6[dC]
```

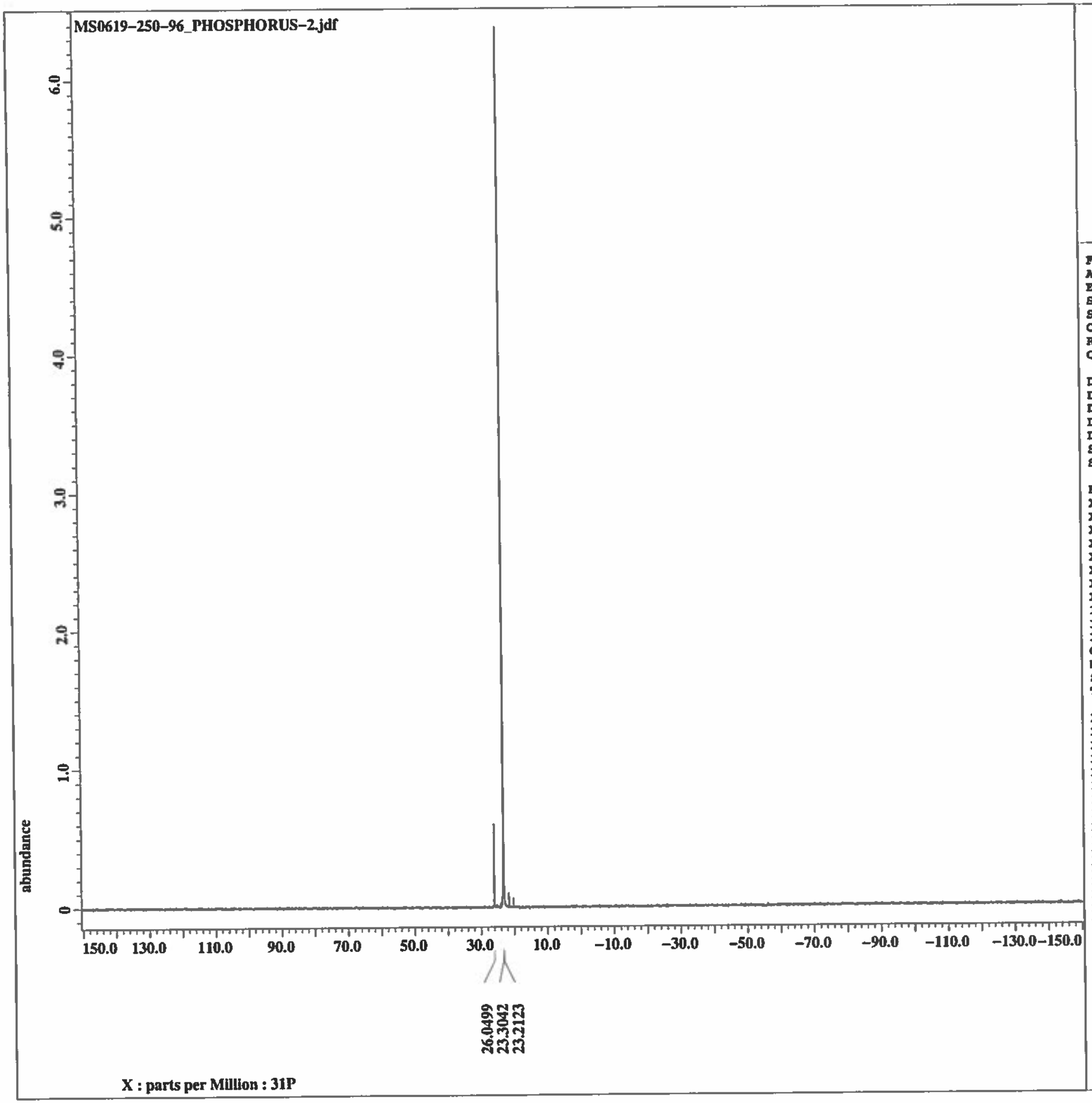

Filename	= MS0619-250-96_PHOSPHO
Author	= Jim Davis
Experiment	= single_pulse_dec
Sample_id	= MS0619-250-96
Solvent	= CHLOROFORM-D
Creation_time	= 4-DEC-2018 12:35:25
Revision_time	= 4-DEC-2018 12:10:07
Current_time	= 4-DEC-2018 12:10:07
Data_format	= 1D COMPLEX
Dim_size	= 52428
Dim_title	= 31P
Dim_units	= [ppm]
Dimensions	= X
Site	= ECA 500
Spectrometer	= JNM-ECA500
Field_strength	= 11.7473579[T] (500[MH
X_acq_duration	= 0.85983232[s]
X_domain	= 31P
X_freq	= 202.46831075[MHz]
X_offset	= 0[ppm]
X_points	= 65536
X_prescans	= 4
X_resolution	= 1.16301746[Hz]
X_sweep	= 76.2195122[kHz]
Irr_domain	= 1H
Irr_freq	= 500.15991521[MHz]
Irr_offset	= 5.0[ppm]
Clipped	= FALSE
Mod_return	= 1
Scans	= 50
Total_scans	= 50
X_90_width	= 14.687[us]
X_acq_time	= 0.85983232[s]
X_angle	= 30[deg]
X_atn	= 5[dB]
X_pulse	= 4.89566667[us]
Irr_atn_dec	= 20.7[dB]
Irr_atn_noe	= 20.7[dB]
Irr_noise	= WALTZ
Decoupling	= TRUE
Initial_wait	= 1[s]
Noe	= TRUE
Noe_time	= 2[s]
Recvr_gain	= 56
Relaxation_delay	= 2[s]
Repetition_time	= 2.85983232[s]
Temp_get	= 20.9[dC]

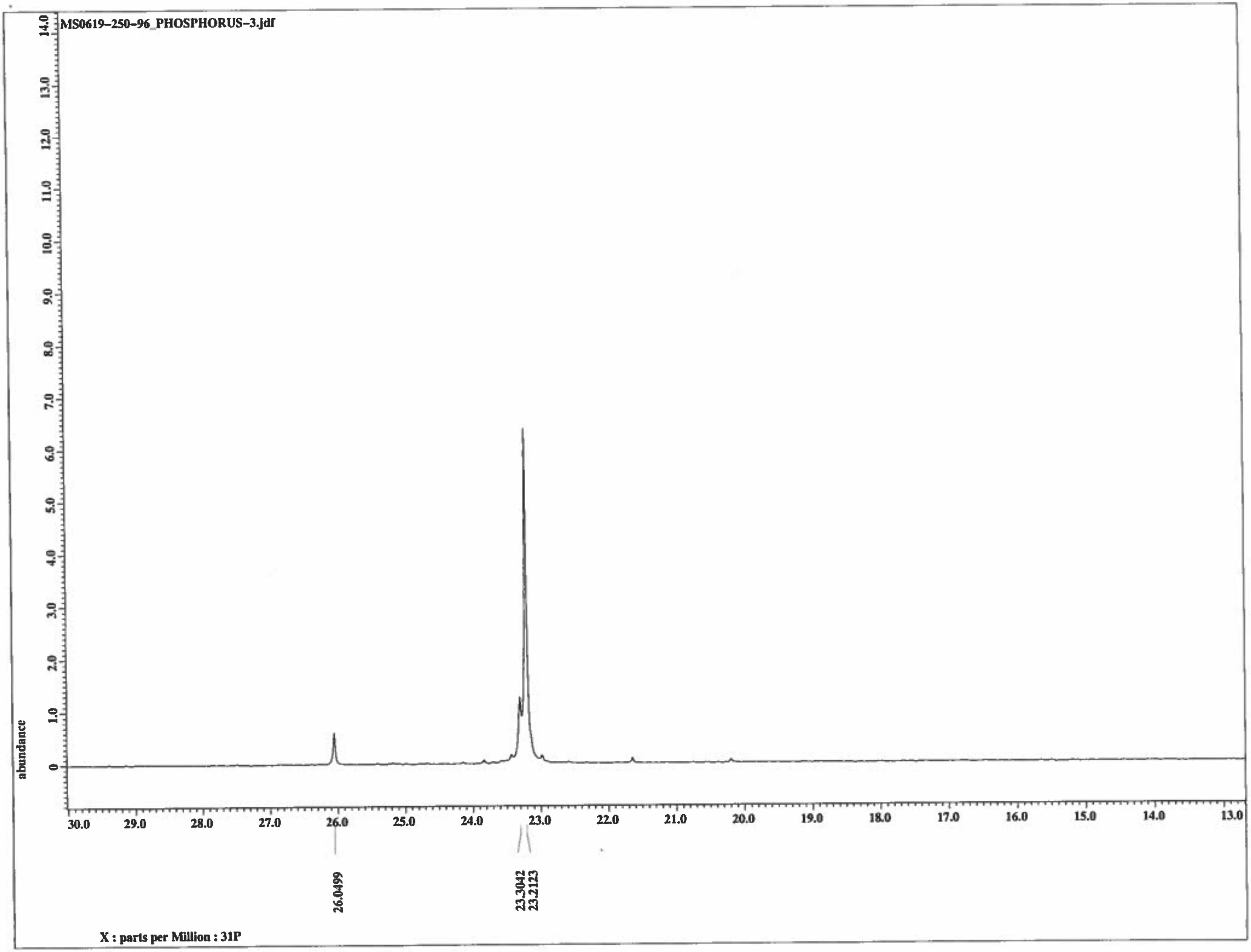

MS0619-250-96_PHOSPHORUS-3.jdf
abundance
0
1.0
2.0
3.0
4.0
5.0
6.0
7.0
8.0
9.0
10.0
11.0
12.0
13.0
14.0
30.0
29.0
28.0
27.0
26.0
25.0
24.0
23.0
22.0
21.0
20.0
19.0
18.0
17.0
16.0
15.0
14.0
13.0
26.0499
23.3042
23.2123
X : parts per Million : 31P

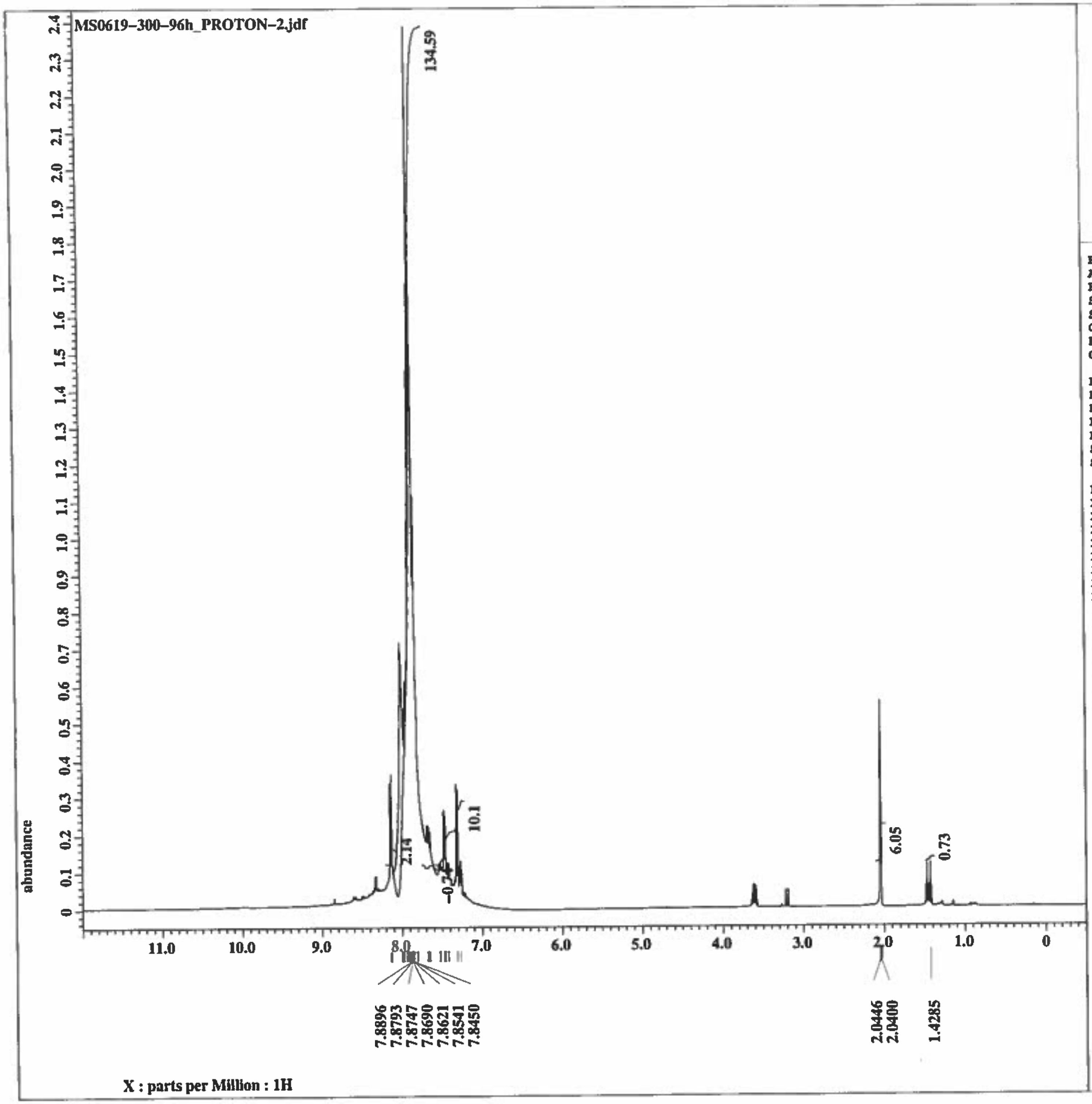


```
Filename            = MS0619-300-96h_PROTON
Author              = Jim Davis
Experiment          = single_pulse.ex2
Sample_id           = MS0619-300-96h
Solvent             = ACETONE-D6
Creation_time       =  4-DEC-2018 15:36:44
Revision_time       =  4-DEC-2018 15:11:24
Current_time        =  4-DEC-2018 15:11:25

Data_format         = 1D COMPLEX
Dim_size            = 13107
Dim_title           = 1H
Dim_units           = [ppm]
Dimensions          = X
Site                = ECA 500
Spectrometer        = JNM-ECA500

Field_strength      = 11.7473579[T] (500[MH
X_acq_duration      = 1.74587904[s]
X_domain            = 1H
X_freq              = 500.15991521[MHz]
X_offset            = 5.0[ppm]
X_points            = 16384
X_prescans          = 1
X_resolution        = 0.57277737[Hz]
X_sweep             = 9.38438438[kHz]
Irr_domain          = 1H
Irr_freq            = 500.15991521[MHz]
Irr_offset          = 5.0[ppm]
Tri_domain          = 1H
Tri_freq            = 500.15991521[MHz]
Tri_offset          = 5.0[ppm]
Clipped             = FALSE
Mod_return          = 1
Scans               = 16
Total_scans         = 16

X_90_width          = 12.4[us]
X_acq_time          = 1.74587904[s]
X_angle             = 45[deg]
X_atn               = 4[dB]
X_pulse             = 6.2[us]
Irr_mode            = Off
Tri_mode            = Off
Dante_presat        = FALSE
Initial_wait        = 1[s]
Recvr_gain          = 30
Relaxation_delay    = 4[s]
Repetition_time     = 5.74587904[s]
Temp_get            = 20.7[dC]
```

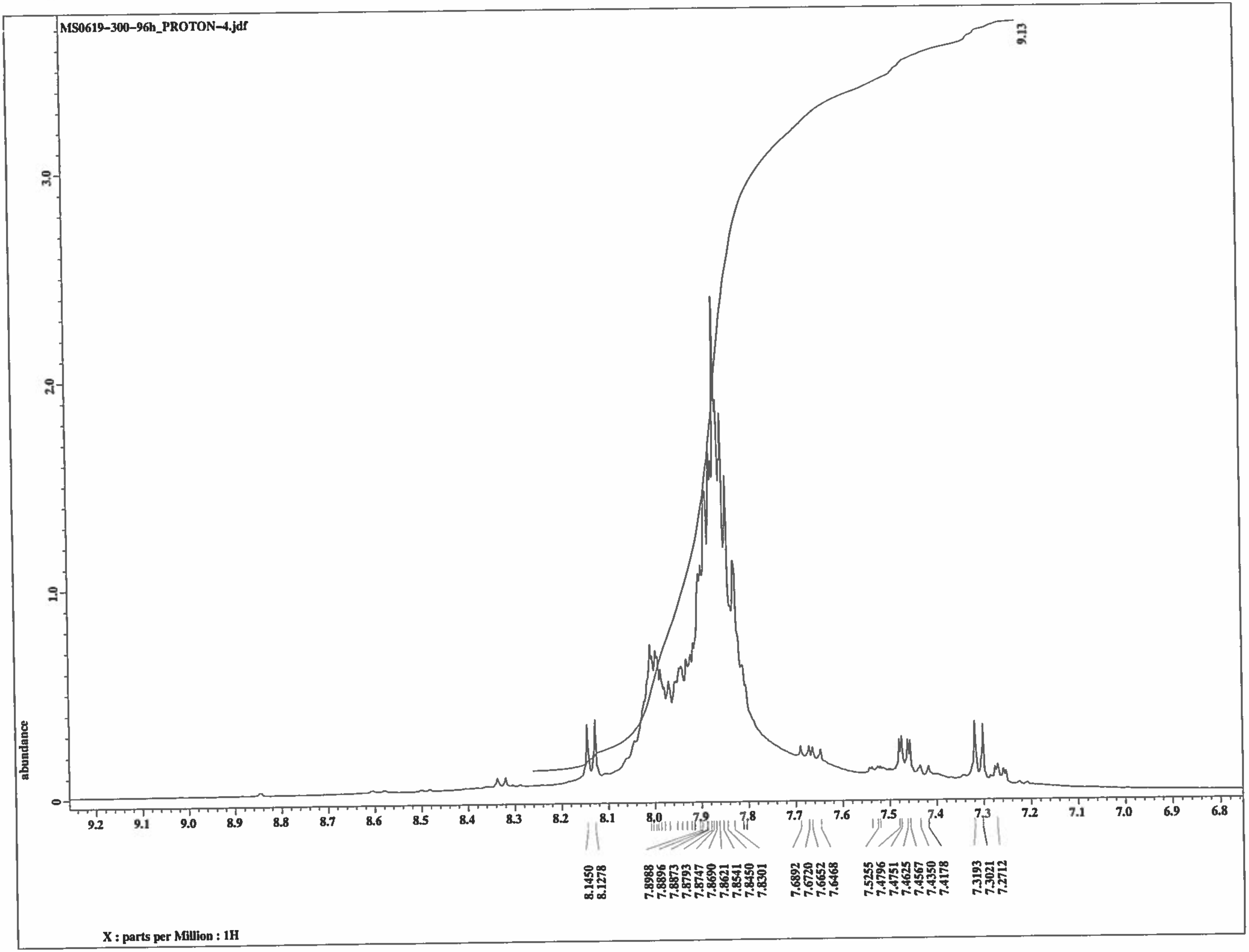

MS0619-300-96h_PROTON-4.jdf
abundance
0
1.0
2.0
3.0
9.13
9.2
9.1
9.0
8.9
8.8
8.7
8.6
8.5
8.4
8.3
8.2
8.1
8.0
7.9
7.8
7.7
7.6
7.5
7.4
7.3
7.2
7.1
7.0
6.9
6.8
8.1450
8.1278
7.8988
7.8896
7.8873
7.8793
7.8747
7.8690
7.8621
7.8541
7.8450
7.8301
7.6892
7.6720
7.6652
7.6468
7.5255
7.4796
7.4751
7.4625
7.4567
7.4350
7.4178
7.3193
7.3021
7.2712
X : parts per Million : 1H

MS0619-300-96h_CARBON-2.jdf

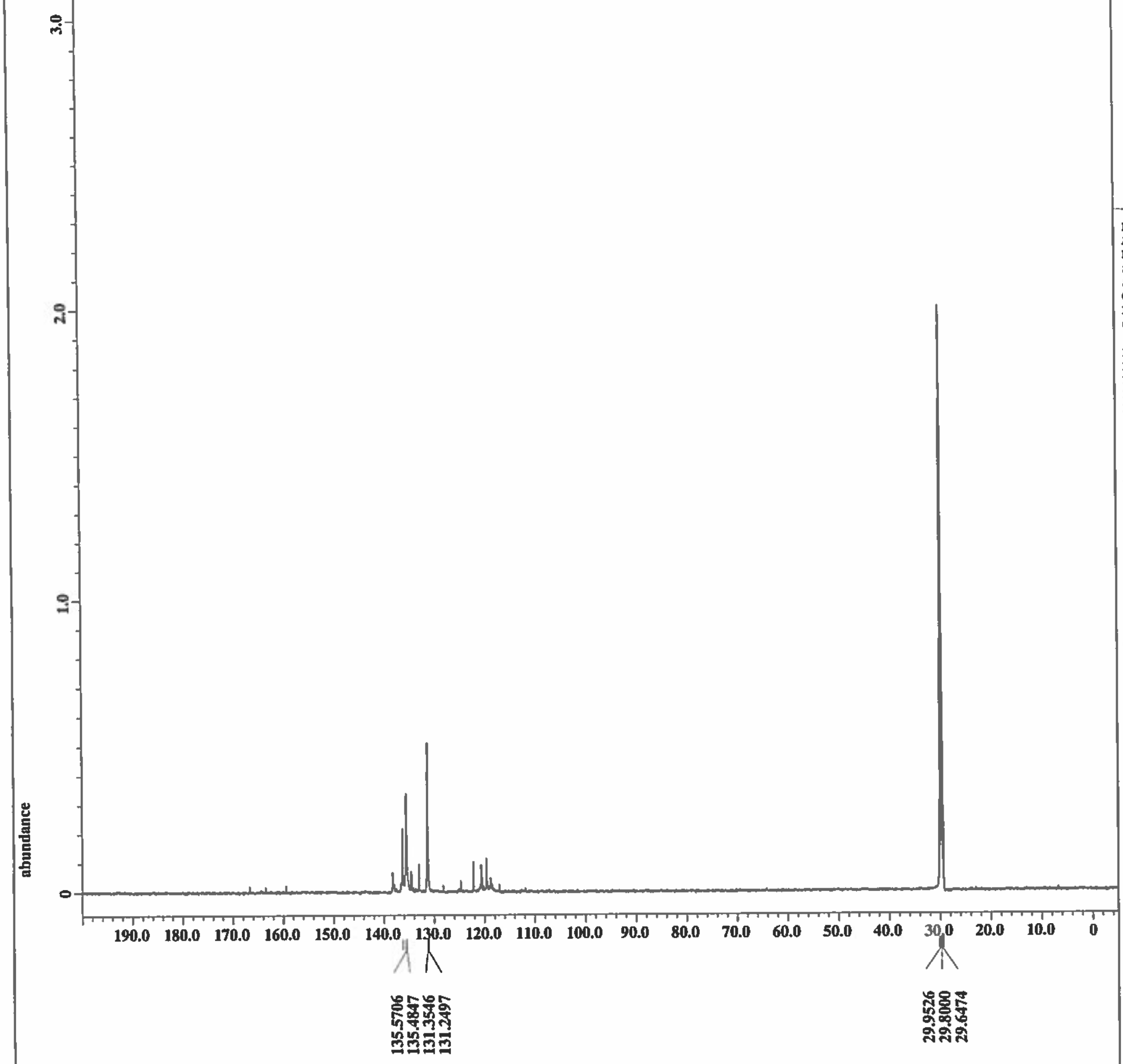

Filename	= MS0619-300-96h_CARBON
Author	= Jim Davis
Experiment	= single_pulse_dec
Sample_id	= MS0619-300-96h
Solvent	= ACETONE-D6
Creation_time	= 4-DEC-2018 16:08:01
Revision_time	= 4-DEC-2018 15:42:44
Current_time	= 4-DEC-2018 15:42:44
Data_format	= 1D COMPLEX
Dim_size	= 26214
Dim_title	= 13C
Dim_units	= [ppm]
Dimensions	= X
Site	= ECA 500
Spectrometer	= JNM-ECA500
Field_strength	= 11.7473579[T] (500[MH
X_acq_duration	= 0.83361792[s]
X_domain	= 13C
X_freq	= 125.76529768[MHz]
X_offset	= 100[ppm]
X_points	= 32768
X_prescans	= 4
X_resolution	= 1.19959034[Hz]
X_sweep	= 39.3081761[kHz]
Irr_domain	= 1H
Irr_freq	= 500.15991521[MHz]
Irr_offset	= 5.0[ppm]
Clipped	= FALSE
Mod_return	= 1
Scans	= 600
Total_scans	= 600
X_90_width	= 13.2[us]
X_acq_time	= 0.83361792[s]
X_angle	= 30[deg]
X_atn	= 6[dB]
X_pulse	= 4.4[us]
Irr_atn_dec	= 20.7[dB]
Irr_atn_noe	= 20.7[dB]
Irr_noise	= WALTZ
Decoupling	= TRUE
Initial_wait	= 1[s]
Noe	= TRUE
Noe_time	= 2[s]
Recvr_gain	= 60
Relaxation_delay	= 2[s]
Repetition_time	= 2.83361792[s]
Temp_get	= 21.1[dC]

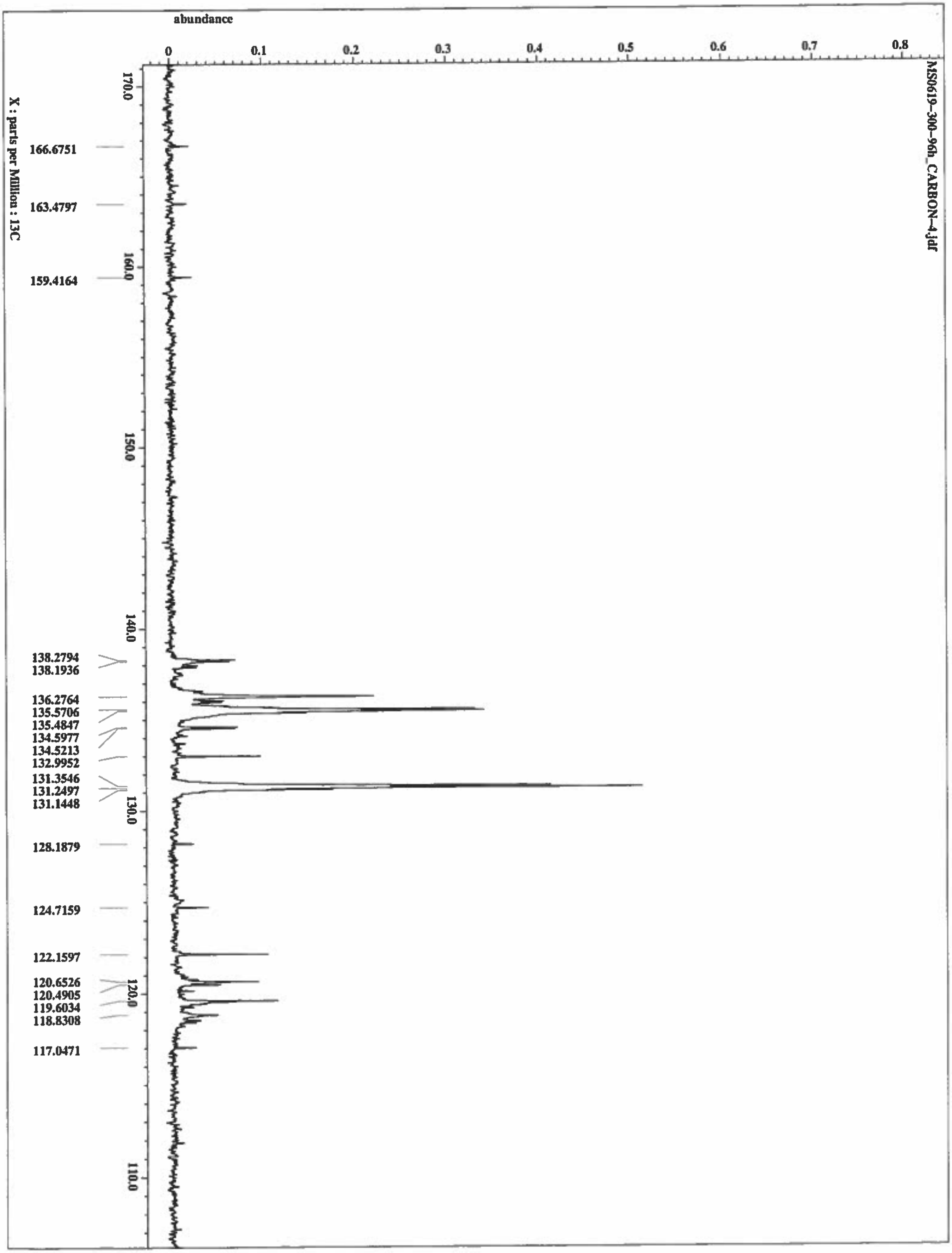
MS0619-300-96h_CARBON-4.jdf
abundance
0
0.1
0.2
0.3
0.4
0.5
0.6
0.7
0.8
X : parts per Million : 13C
170.0
160.0
150.0
140.0
130.0
120.0
110.0
166.6751
163.4797
159.4164
138.2794
138.1936
136.2764
135.5706
135.4847
134.5977
134.5213
132.9952
131.3546
131.2497
131.1448
128.1879
124.7159
122.1597
120.6526
120.4905
119.6034
118.8308
117.0471

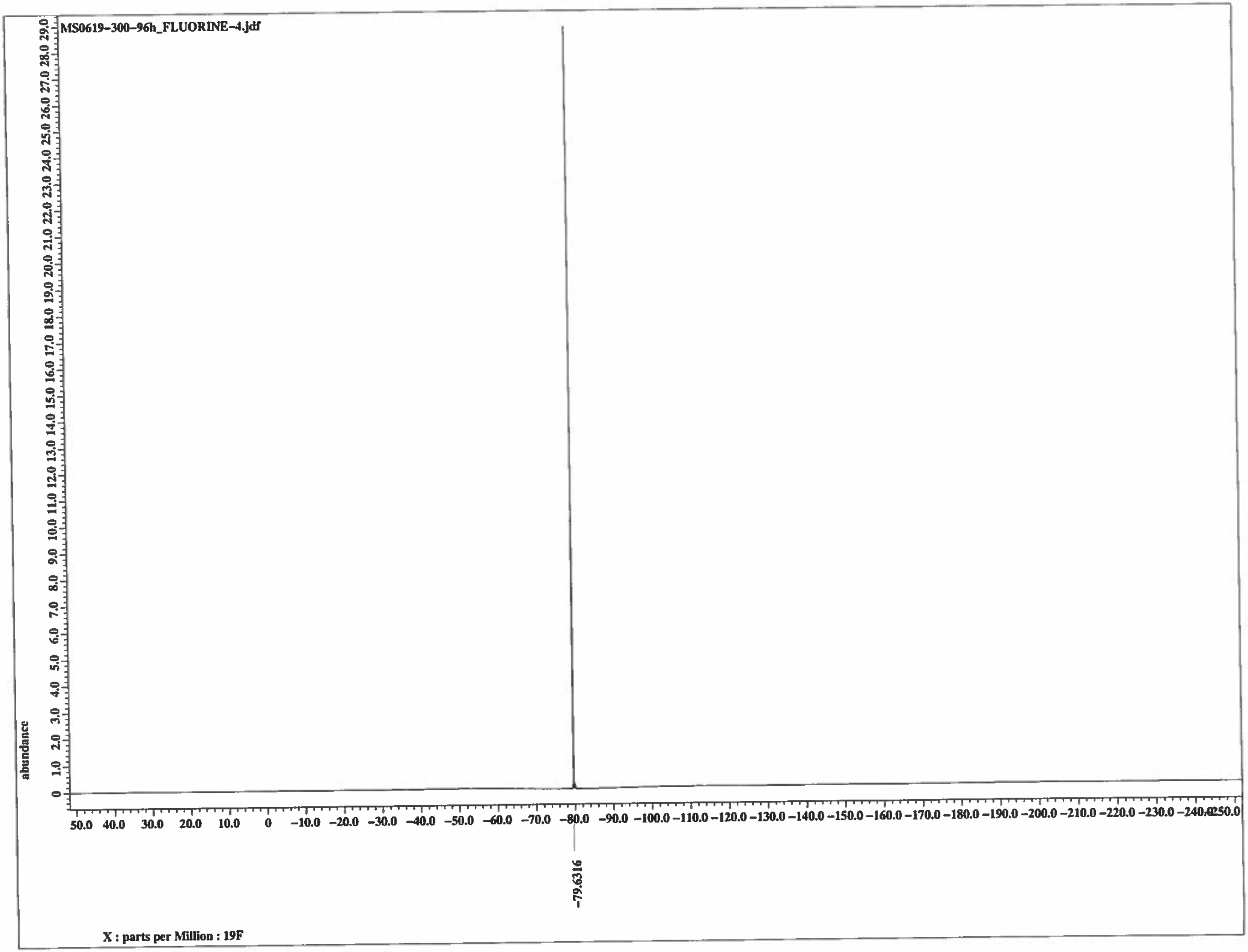

MS0619-300-96h_FLUORINE-4.jdf
abundance
0 1.0 2.0 3.0 4.0 5.0 6.0 7.0 8.0 9.0 10.0 11.0 12.0 13.0 14.0 15.0 16.0 17.0 18.0 19.0 20.0 21.0 22.0 23.0 24.0 25.0 26.0 27.0 28.0 29.0
50.0 40.0 30.0 20.0 10.0 0 -10.0 -20.0 -30.0 -40.0 -50.0 -60.0 -70.0 -80.0 -90.0 -100.0 -110.0 -120.0 -130.0 -140.0 -150.0 -160.0 -170.0 -180.0 -190.0 -200.0 -210.0 -220.0 -230.0 -240.0 -250.0
-79.6316
X : parts per Million : 19F

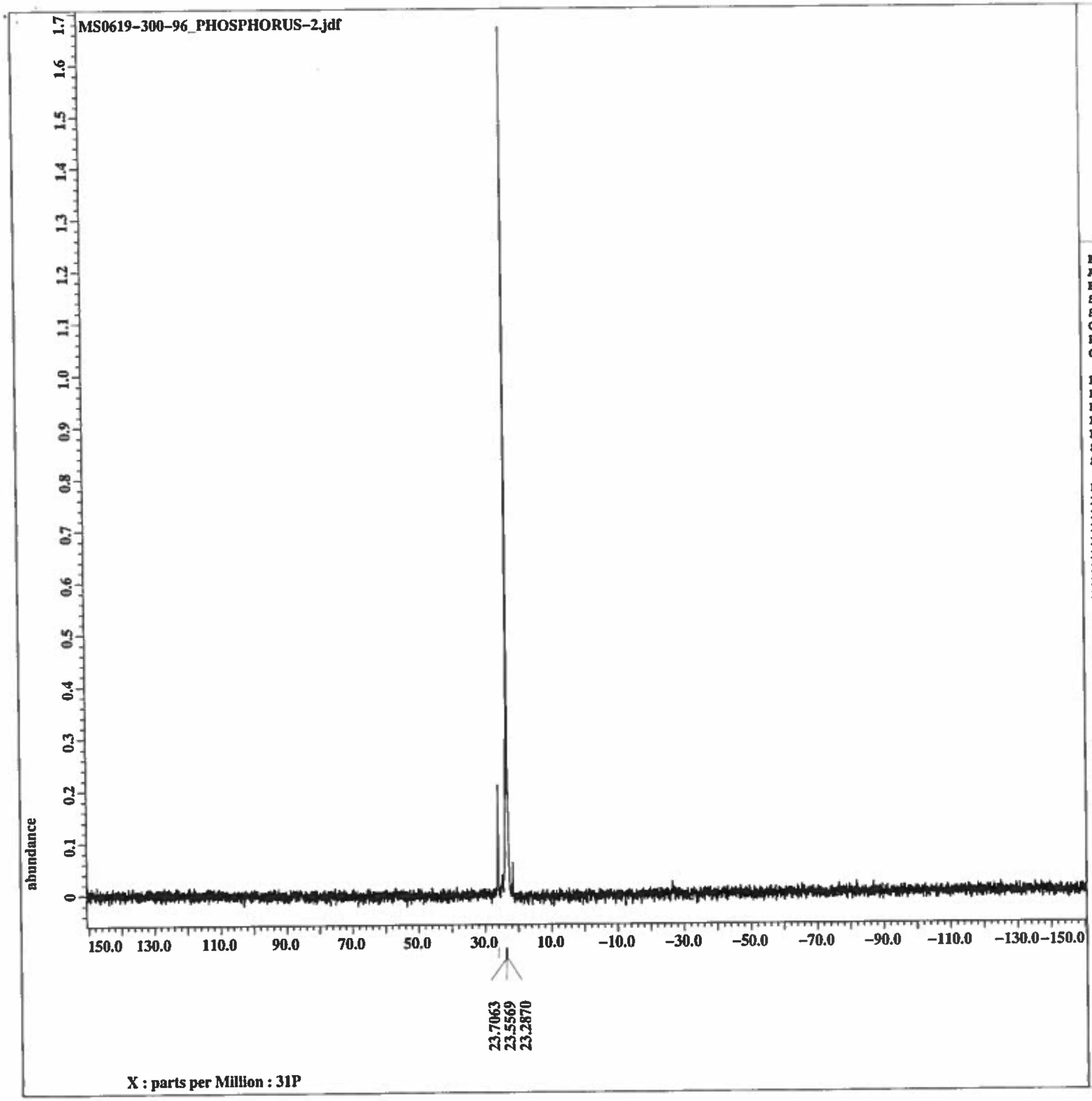

SOUTH ALABAMA JAGUARS

```
Filename            = MS0619-300-96_PHOSPHO
Author              = Jim Davis
Experiment          = single_pulse_dec
Sample_id           = MS0619-300-96
Solvent             = CHLOROFORM-D
Creation_time       =  4-DEC-2018 12:00:07
Revision_time       =  4-DEC-2018 11:34:50
Current_time        =  4-DEC-2018 11:34:50

Data_format         = 1D COMPLEX
Dim_size            = 52428
Dim_title           = 31P
Dim_units           = [ppm]
Dimensions          = X
Site                = ECA 500
Spectrometer        = JNM-ECA500

Field_strength      = 11.7473579[T] (500[MH
X_acq_duration      = 0.85983232[s]
X_domain            = 31P
X_freq              = 202.46831075[MHz]
X_offset            = 0[ppm]
X_points            = 65536
X_prescans          = 4
X_resolution        = 1.16301746[Hz]
X_sweep             = 76.2195122[kHz]
Irr_domain          = 1H
Irr_freq            = 500.15991521[MHz]
Irr_offset          = 5.0[ppm]
Clipped             = TRUE
Mod_return          = 1
Scans               = 50
Total_scans         = 50

X_90_width          = 14.687[us]
X_acq_time          = 0.85983232[s]
X_angle             = 30[deg]
X_atn               = 5[dB]
X_pulse             = 4.89566667[us]
Irr_atn_dec         = 20.7[dB]
Irr_atn_noe         = 20.7[dB]
Irr_noise           = WALTZ
Decoupling          = TRUE
Initial_wait        = 1[s]
Noe                 = TRUE
Noe_time            = 2[s]
Recvr_gain          = 58
Relaxation_delay    = 2[s]
Repetition_time     = 2.85983232[s]
Temp_get            = 20.7[dC]
```

Compound 16 Pre- and Post-heating NMR Spectra

Temperature of Post-heating samples noted in upper left corner of each spectrum

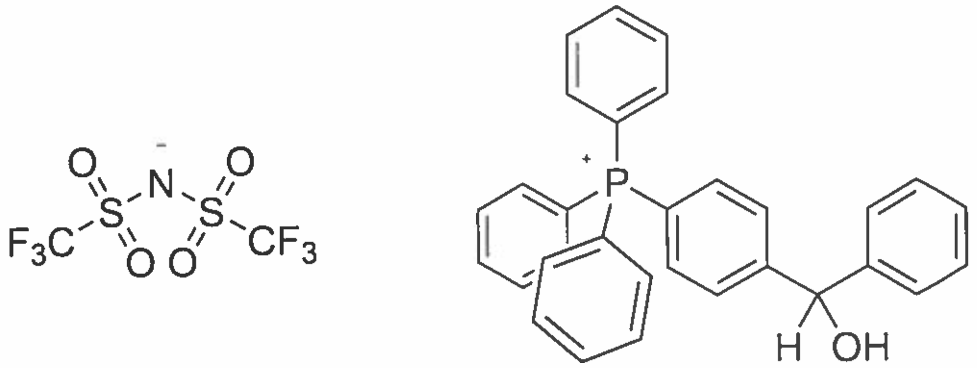

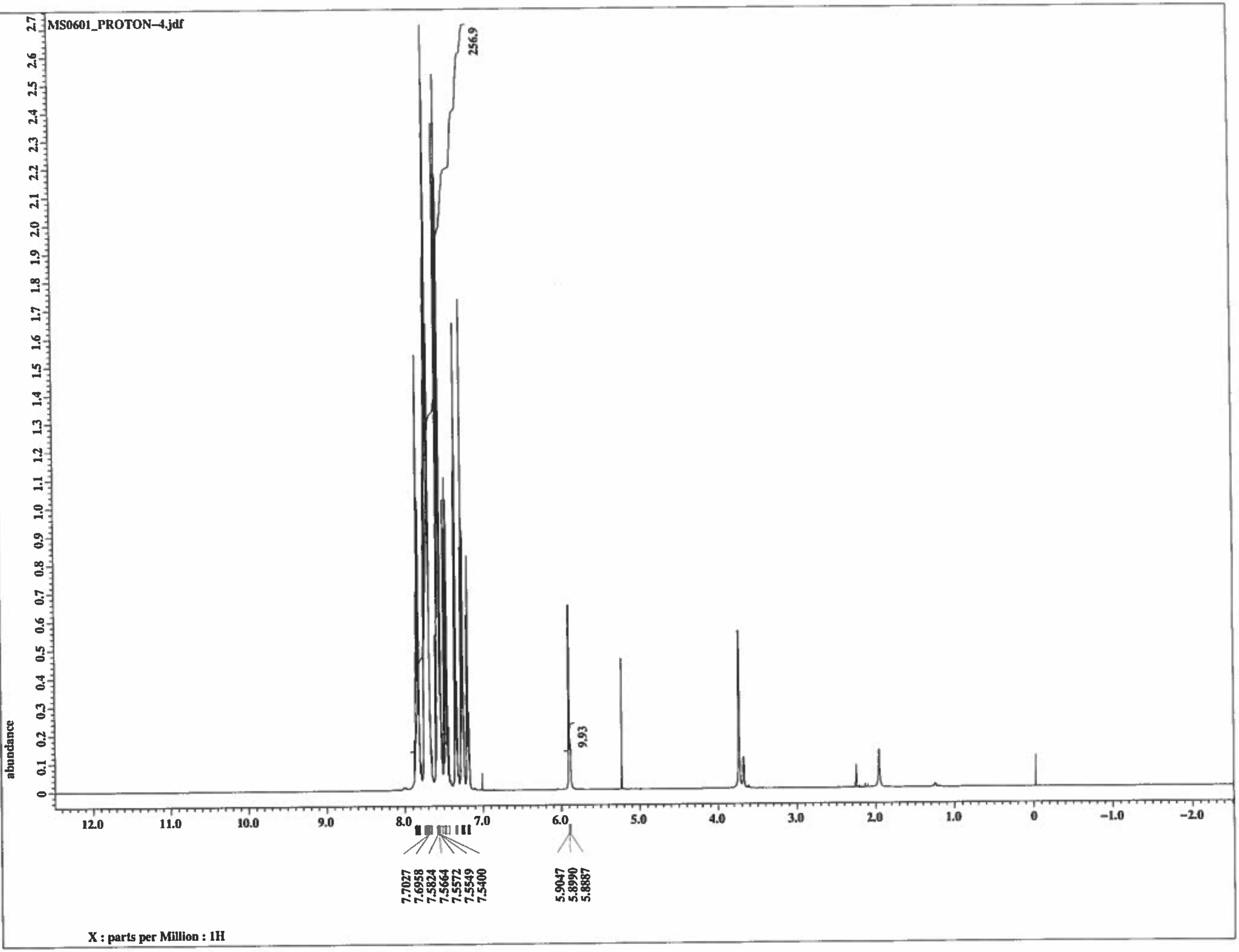
MS0601_PROTON-4.jdf
abundance
256.9
9.93
7.7027
7.6958
7.5824
7.5664
7.5572
7.5549
7.5400
5.9047
5.8990
5.8887
X : parts per Million : 1H

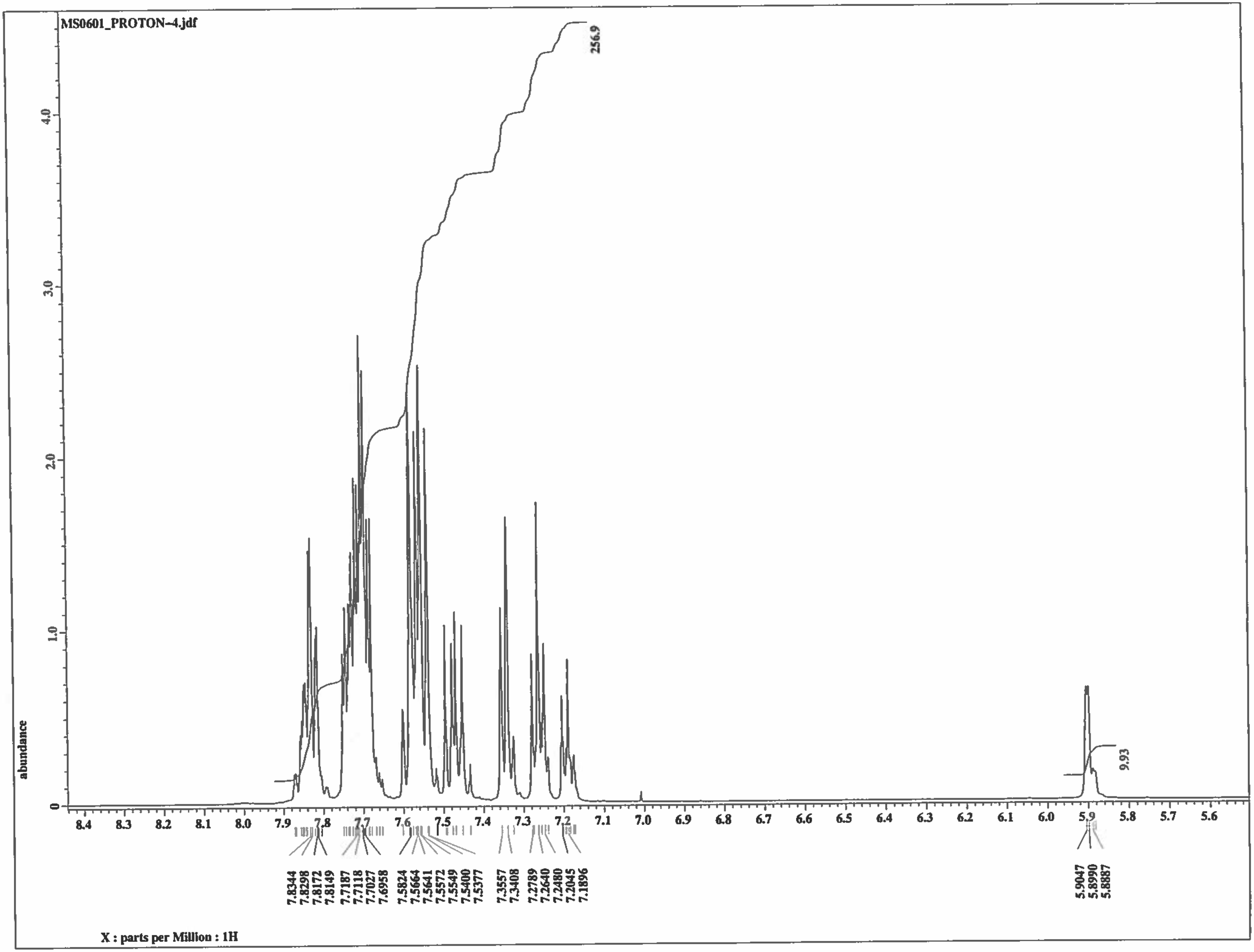
MS0601_PROTON-4.jdf
256.9
abundance
0
1.0
2.0
3.0
4.0
8.4
8.3
8.2
8.1
8.0
7.9
7.8
7.7
7.6
7.5
7.4
7.3
7.2
7.1
7.0
6.9
6.8
6.7
6.6
6.5
6.4
6.3
6.2
6.1
6.0
5.9
5.8
5.7
5.6
7.8344
7.8298
7.8172
7.8149
7.7187
7.7118
7.7027
7.6958
7.5824
7.5664
7.5641
7.5572
7.5549
7.5400
7.5377
7.3557
7.3408
7.2789
7.2640
7.2480
7.2045
7.1896
9.93
5.9047
5.8990
5.8887
X : parts per Million : 1H

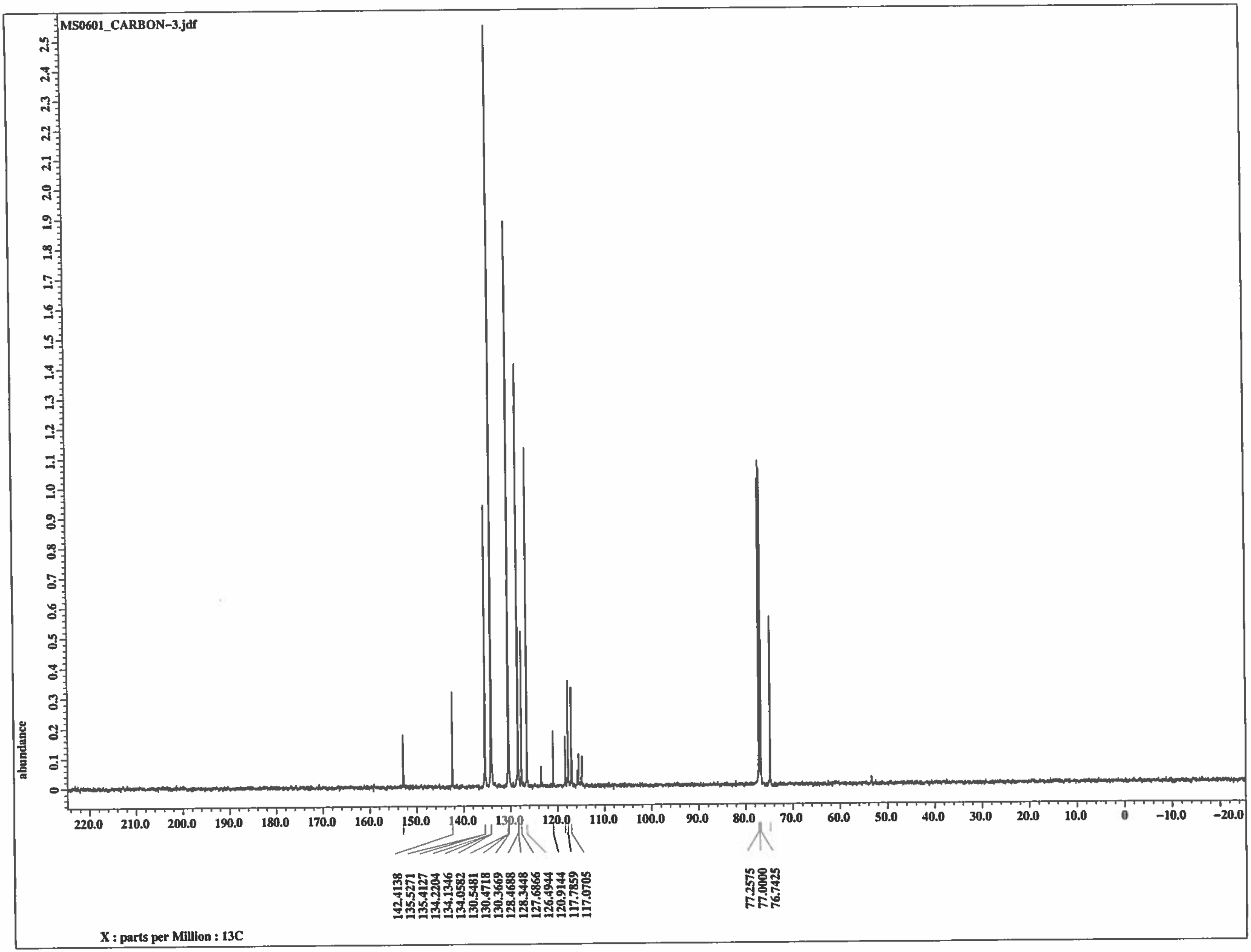
MS0601_CARBON-3.jdf
abundance
0
0.1
0.2
0.3
0.4
0.5
0.6
0.7
0.8
0.9
1.0
1.1
1.2
1.3
1.4
1.5
1.6
1.7
1.8
1.9
2.0
2.1
2.2
2.3
2.4
2.5
220.0
210.0
200.0
190.0
180.0
170.0
160.0
150.0
140.0
130.0
120.0
110.0
100.0
90.0
80.0
70.0
60.0
50.0
40.0
30.0
20.0
10.0
0
-10.0
-20.0
142.4138
135.5271
135.4127
134.2204
134.1346
134.0582
130.5481
130.4718
130.3669
128.4688
128.3448
127.6866
126.4944
120.9144
117.7859
117.0705
77.2575
77.0000
76.7425
X : parts per Million : 13C

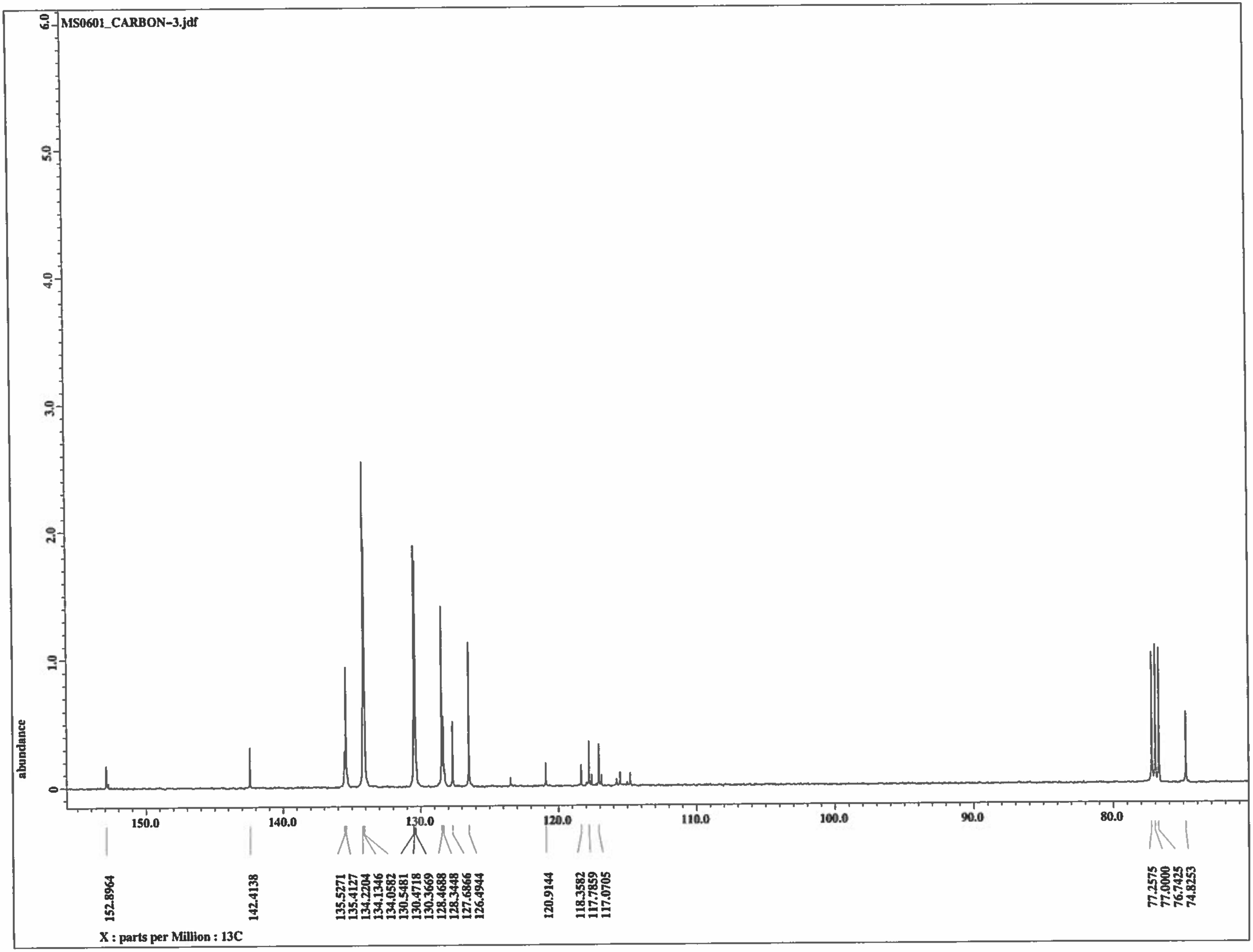

MS0601_CARBON-3.jdf
abundance
0
1.0
2.0
3.0
4.0
5.0
6.0
150.0
140.0
130.0
120.0
110.0
100.0
90.0
80.0
152.8964
142.4138
135.5271
135.4127
134.2204
134.1346
134.0582
130.5481
130.4718
130.3669
128.4688
128.3448
127.6866
126.4944
120.9144
118.3582
117.7859
117.0705
77.2575
77.0000
76.7425
74.8253
X : parts per Million : 13C

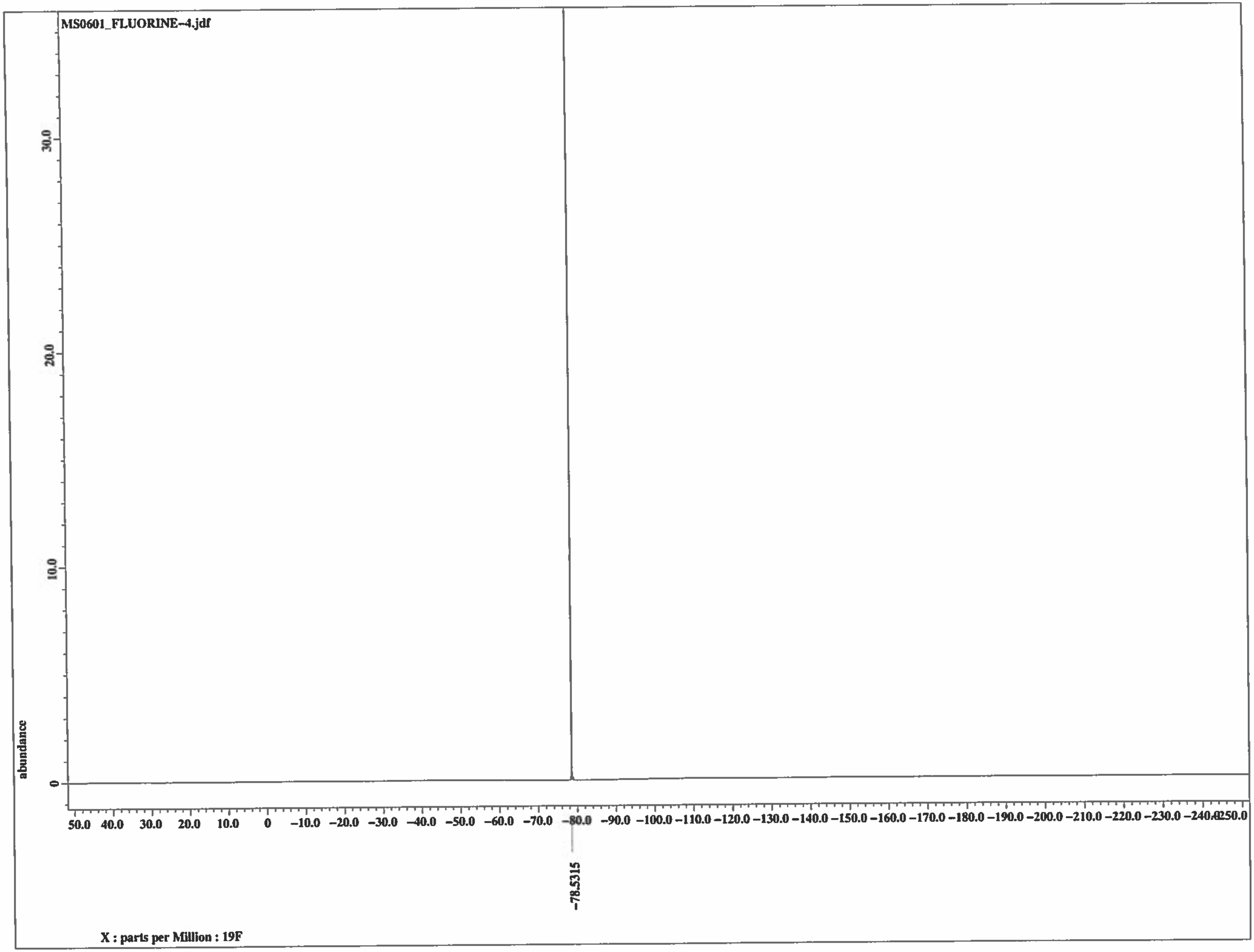
MS0601_FLUORINE-4.jdf
abundance
0
10.0
20.0
30.0
50.0 40.0 30.0 20.0 10.0 0 −10.0 −20.0 −30.0 −40.0 −50.0 −60.0 −70.0 −80.0 −90.0 −100.0 −110.0 −120.0 −130.0 −140.0 −150.0 −160.0 −170.0 −180.0 −190.0 −200.0 −210.0 −220.0 −230.0 −240.0 −250.0
−78.5315
X : parts per Million : 19F

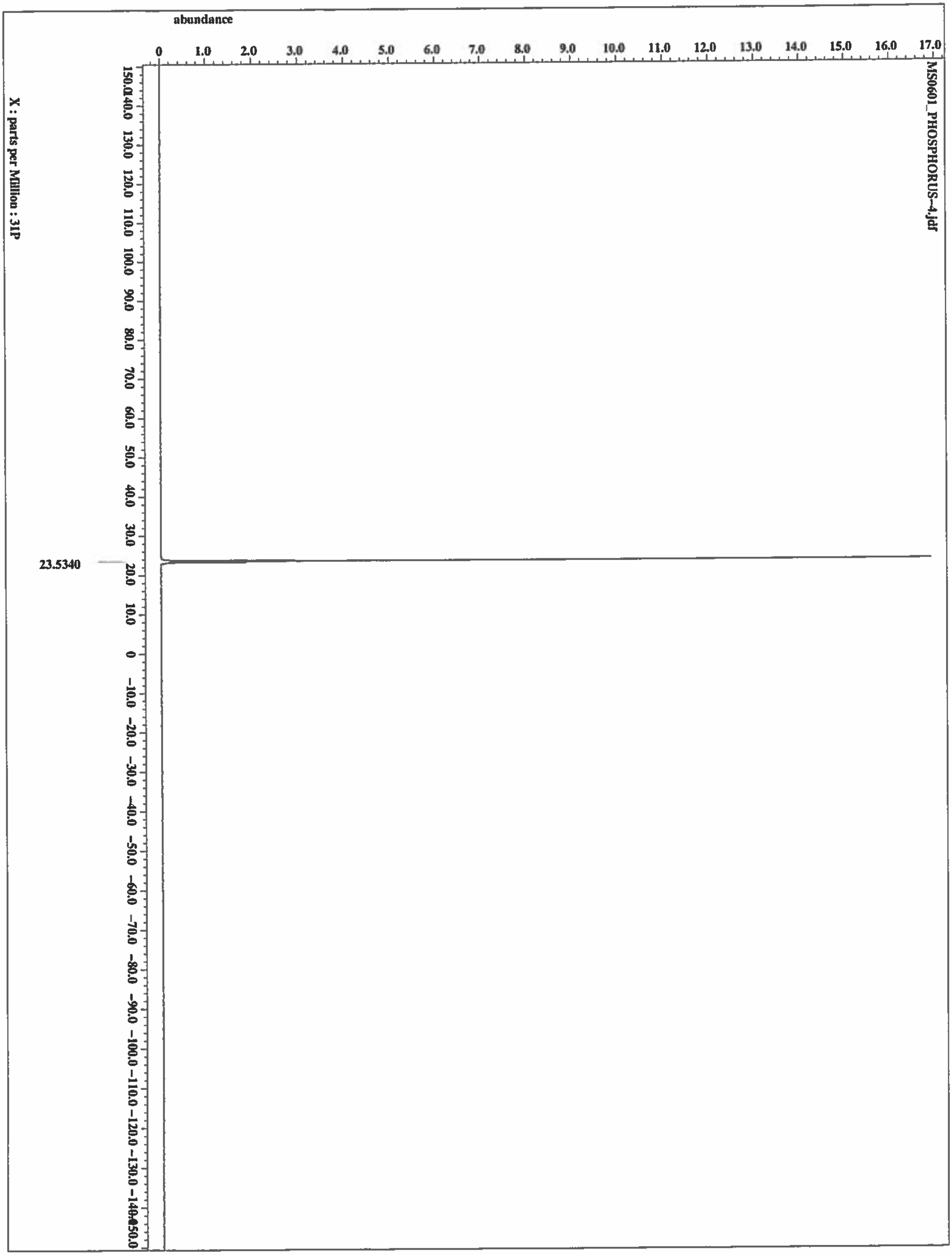
abundance
0 1.0 2.0 3.0 4.0 5.0 6.0 7.0 8.0 9.0 10.0 11.0 12.0 13.0 14.0 15.0 16.0 17.0
MS0601_PHOSPHORUS-4.jdf
X : parts per Million : 31P
23.5340
150.0 140.0 130.0 120.0 110.0 100.0 90.0 80.0 70.0 60.0 50.0 40.0 30.0 20.0 10.0 0 -10.0 -20.0 -30.0 -40.0 -50.0 -60.0 -70.0 -80.0 -90.0 -100.0 -110.0 -120.0 -130.0 -140.0 -150.0

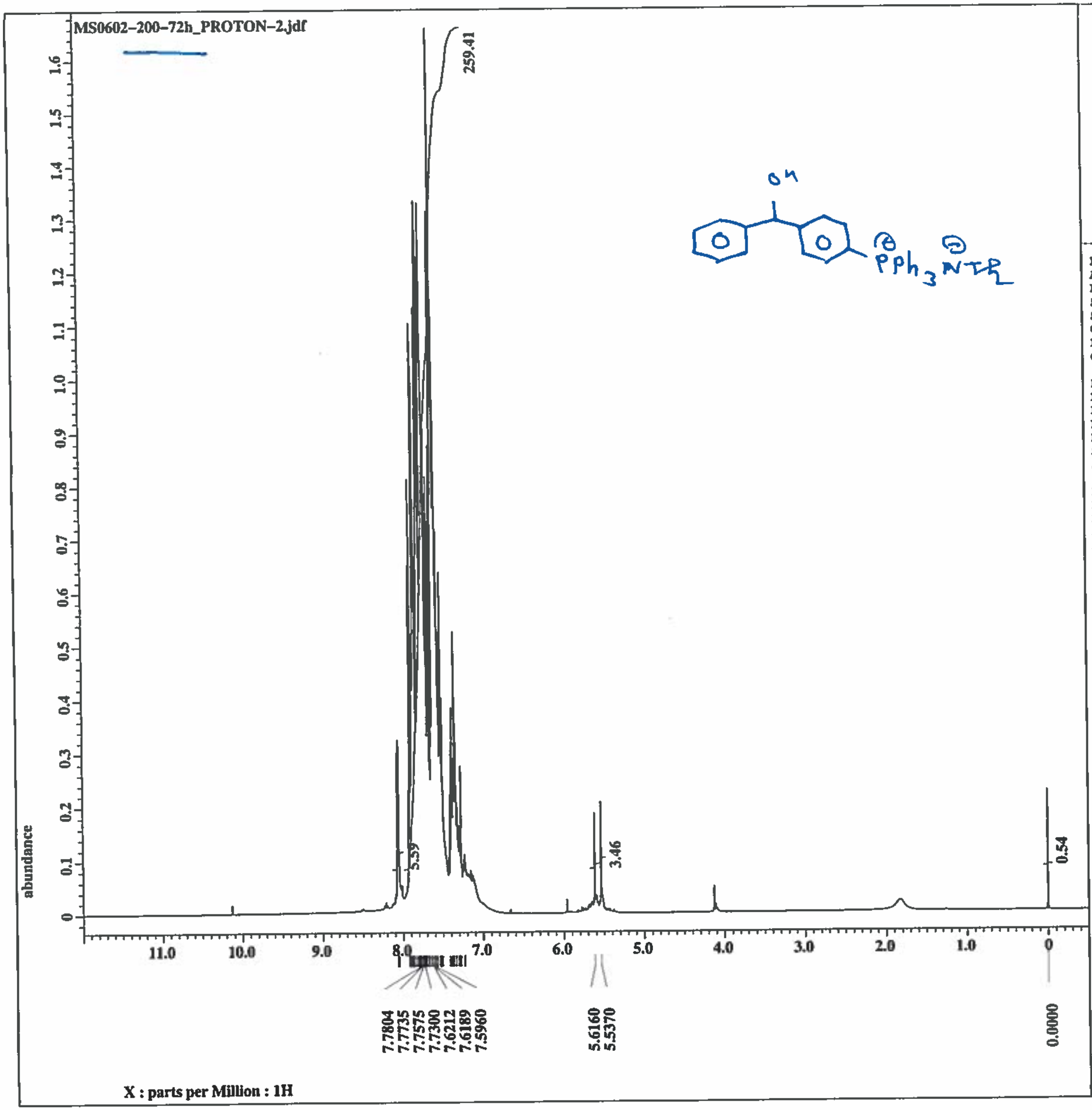

Filename	= MS0602-200-72h_PROTON
Author	= Jim Davis
Experiment	= single_pulse.ex2
Sample_id	= MS0602-200-72h
Solvent	= CHLOROFORM-D
Creation_time	= 8-NOV-2018 18:35:06
Revision_time	= 8-NOV-2018 18:10:23
Current_time	= 8-NOV-2018 18:10:23
Data_format	= 1D COMPLEX
Dim_size	= 13107
Dim_title	= 1H
Dim_units	= [ppm]
Dimensions	= X
Site	= ECA 500
Spectrometer	= JNM-ECA500
Field_strength	= 11.7473579[T] (500[MH
X_acq_duration	= 1.74587904[s]
X_domain	= 1H
X_freq	= 500.15991521[MHz]
X_offset	= 5.0[ppm]
X_points	= 16384
X_prescans	= 1
X_resolution	= 0.57277737[Hz]
X_sweep	= 9.38438438[kHz]
Irr_domain	= 1H
Irr_freq	= 500.15991521[MHz]
Irr_offset	= 5.0[ppm]
Tri_domain	= 1H
Tri_freq	= 500.15991521[MHz]
Tri_offset	= 5.0[ppm]
Clipped	= FALSE
Mod_return	= 1
Scans	= 16
Total_scans	= 16
X_90_width	= 12.4[us]
X_acq_time	= 1.74587904[s]
X_angle	= 45[deg]
X_atn	= 4[dB]
X_pulse	= 6.2[us]
Irr_mode	= Off
Tri_mode	= Off
Dante_presat	= FALSE
Initial_wait	= 1[s]
Recvr_gain	= 26
Relaxation_delay	= 4[s]
Repetition_time	= 5.74587904[s]
Temp_get	= 22.8[dC]

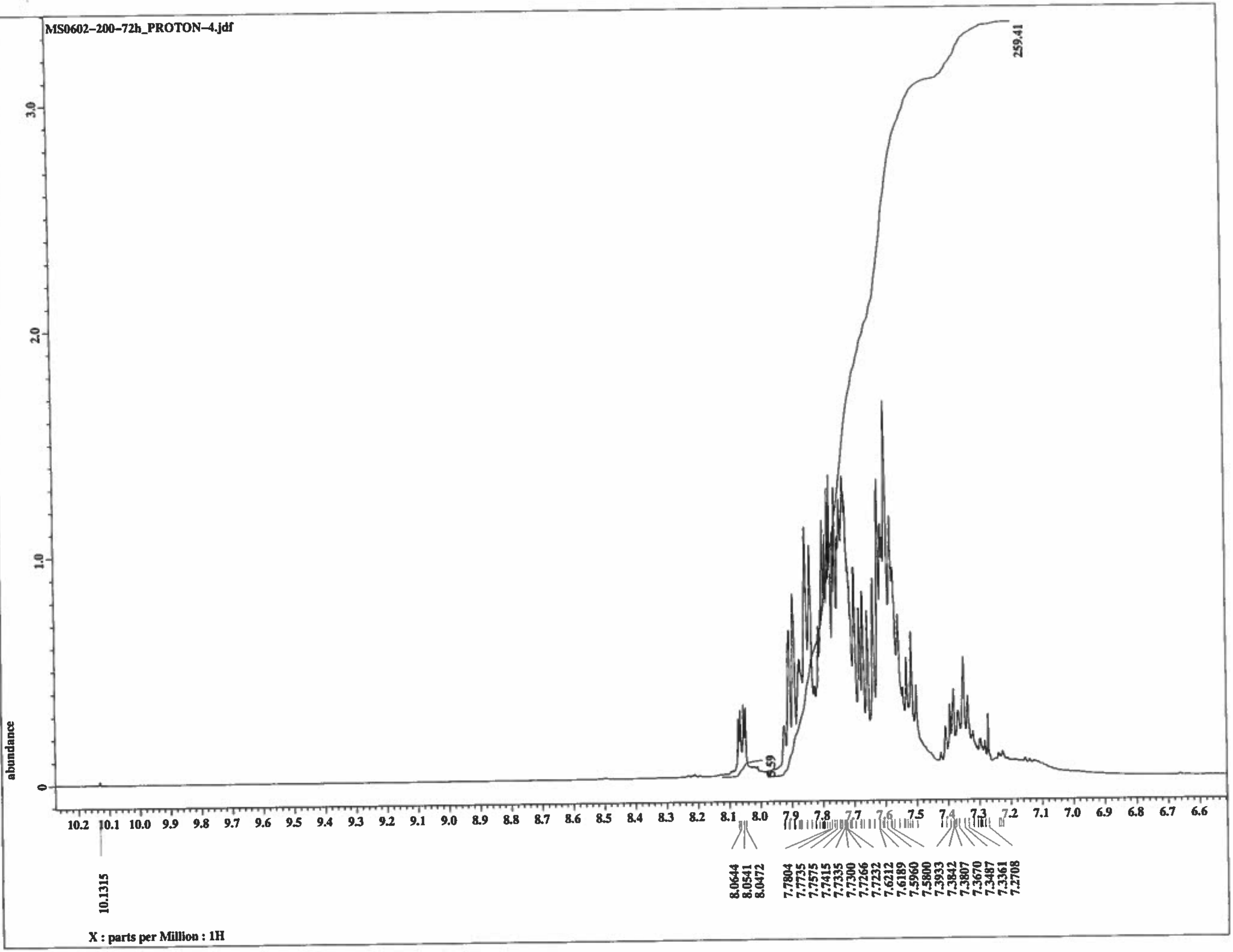
MS0602-200-72h_PROTON-4.jdf
abundance
0
1.0
2.0
3.0
259.41
5.59
10.2 10.1 10.0 9.9 9.8 9.7 9.6 9.5 9.4 9.3 9.2 9.1 9.0 8.9 8.8 8.7 8.6 8.5 8.4 8.3 8.2 8.1 8.0 7.9 7.8 7.7 7.6 7.5 7.4 7.3 7.2 7.1 7.0 6.9 6.8 6.7 6.6
10.1315
8.0644
8.0541
8.0472
7.7804
7.7735
7.7575
7.7415
7.7335
7.7300
7.7266
7.7232
7.6212
7.6189
7.5960
7.5800
7.3933
7.3842
7.3807
7.3670
7.3487
7.3361
7.2708
X : parts per Million : 1H

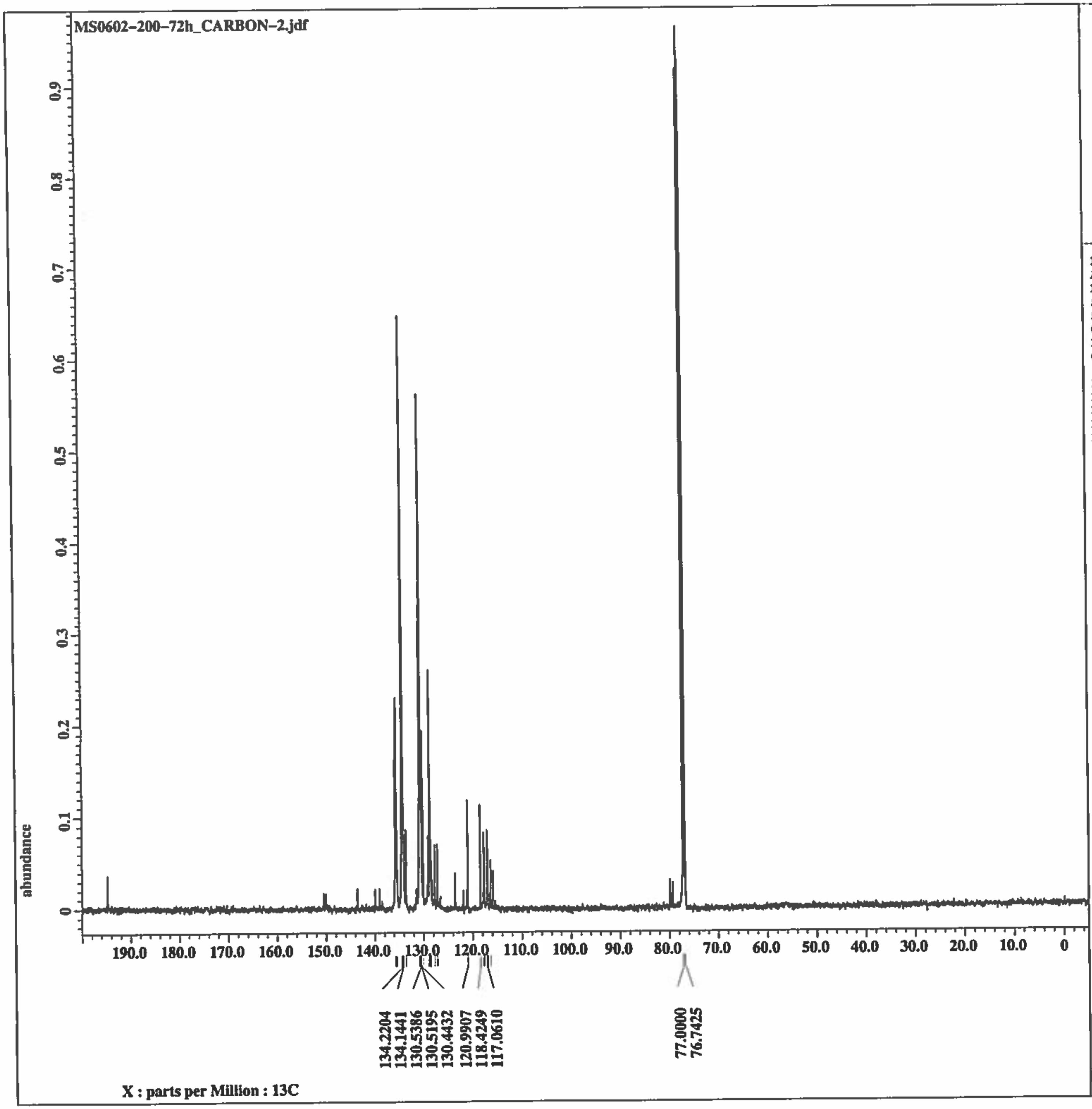


```
Filename            = MS0602-200-72h_CARBON
Author              = Jim Davis
Experiment          = single_pulse_dec
Sample_id           = MS0602-200-72h
Solvent             = CHLOROFORM-D
Creation_time       =  8-NOV-2018 19:23:57
Revision_time       =  8-NOV-2018 18:59:15
Current_time        =  8-NOV-2018 18:59:15

Data_format         = 1D COMPLEX
Dim_size            = 26214
Dim_title           = 13C
Dim_units           = [ppm]
Dimensions          = X
Site                = ECA 500
Spectrometer        = JNM-ECA500

Field_strength      = 11.7473579[T] (500[MH
X_acq_duration      = 0.83361792[s]
X_domain            = 13C
X_freq              = 125.76529768[MHz]
X_offset            = 100[ppm]
X_points            = 32768
X_prescans          = 4
X_resolution        = 1.19959034[Hz]
X_sweep             = 39.3081761[kHz]
Irr_domain          = 1H
Irr_freq            = 500.15991521[MHz]
Irr_offset          = 5.0[ppm]
Clipped             = FALSE
Mod_return          = 1
Scans               = 1024
Total_scans         = 1024

X_90_width          = 13.2[us]
X_acq_time          = 0.83361792[s]
X_angle             = 30[deg]
X_atn               = 6[dB]
X_pulse             = 4.4[us]
Irr_atn_dec         = 20.7[dB]
Irr_atn_noe         = 20.7[dB]
Irr_noise           = WALTZ
Decoupling          = TRUE
Initial_wait        = 1[s]
Noe                 = TRUE
Noe_time            = 2[s]
Recvr_gain          = 60
Relaxation_delay    = 2[s]
Repetition_time     = 2.83361792[s]
Temp_get            = 23.3[dC]
```

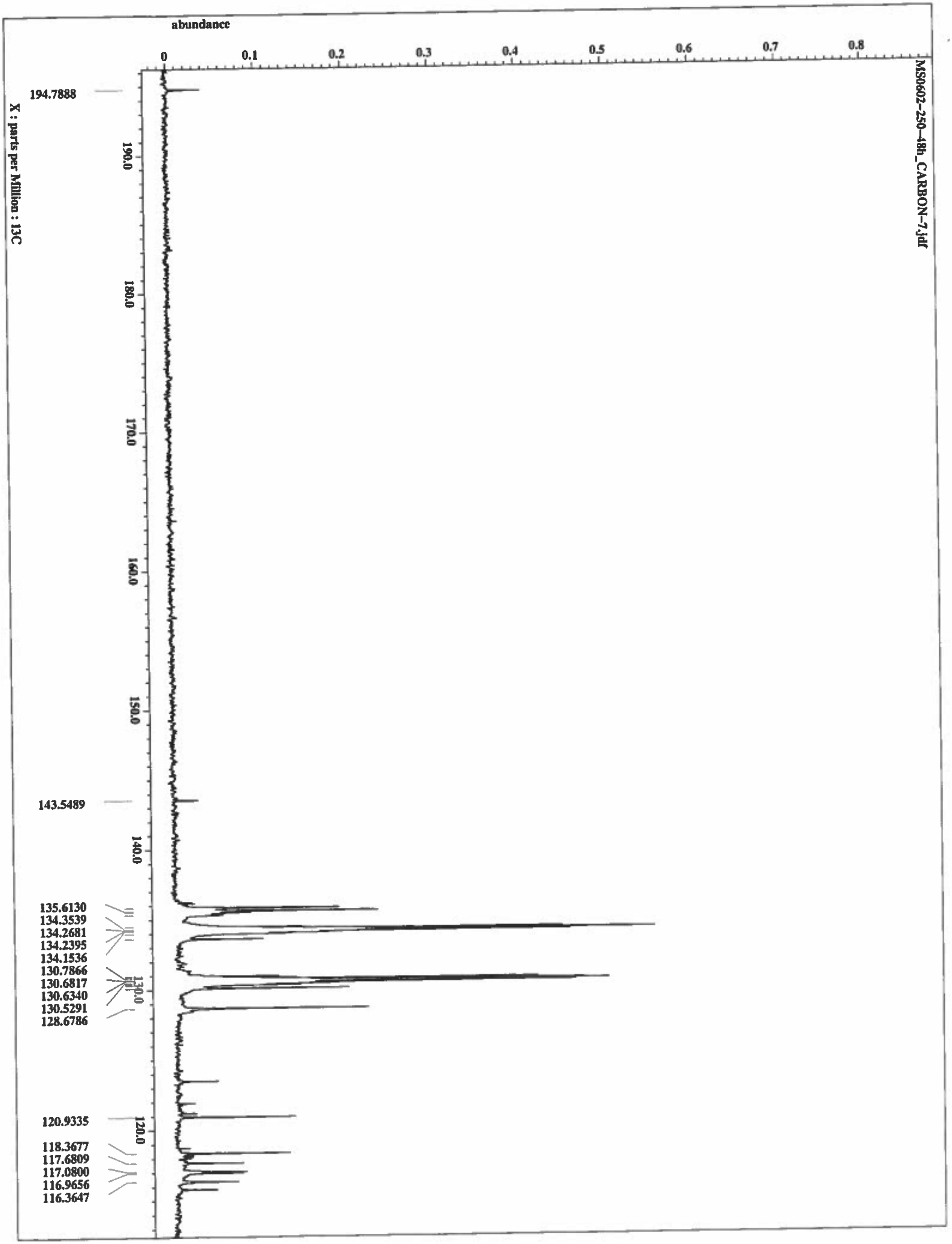
abundance
0
0.1
0.2
0.3
0.4
0.5
0.6
0.7
0.8
MS0602-250-48h_CARBON-7.jdf
X : parts per Million : 13C
194.7888
190.0
180.0
170.0
160.0
150.0
143.5489
140.0
135.6130
134.3539
134.2681
134.2395
134.1536
130.7866
130.6817
130.6340
130.5291
128.6786
130.0
120.9335
120.0
118.3677
117.6809
117.0800
116.9656
116.3647

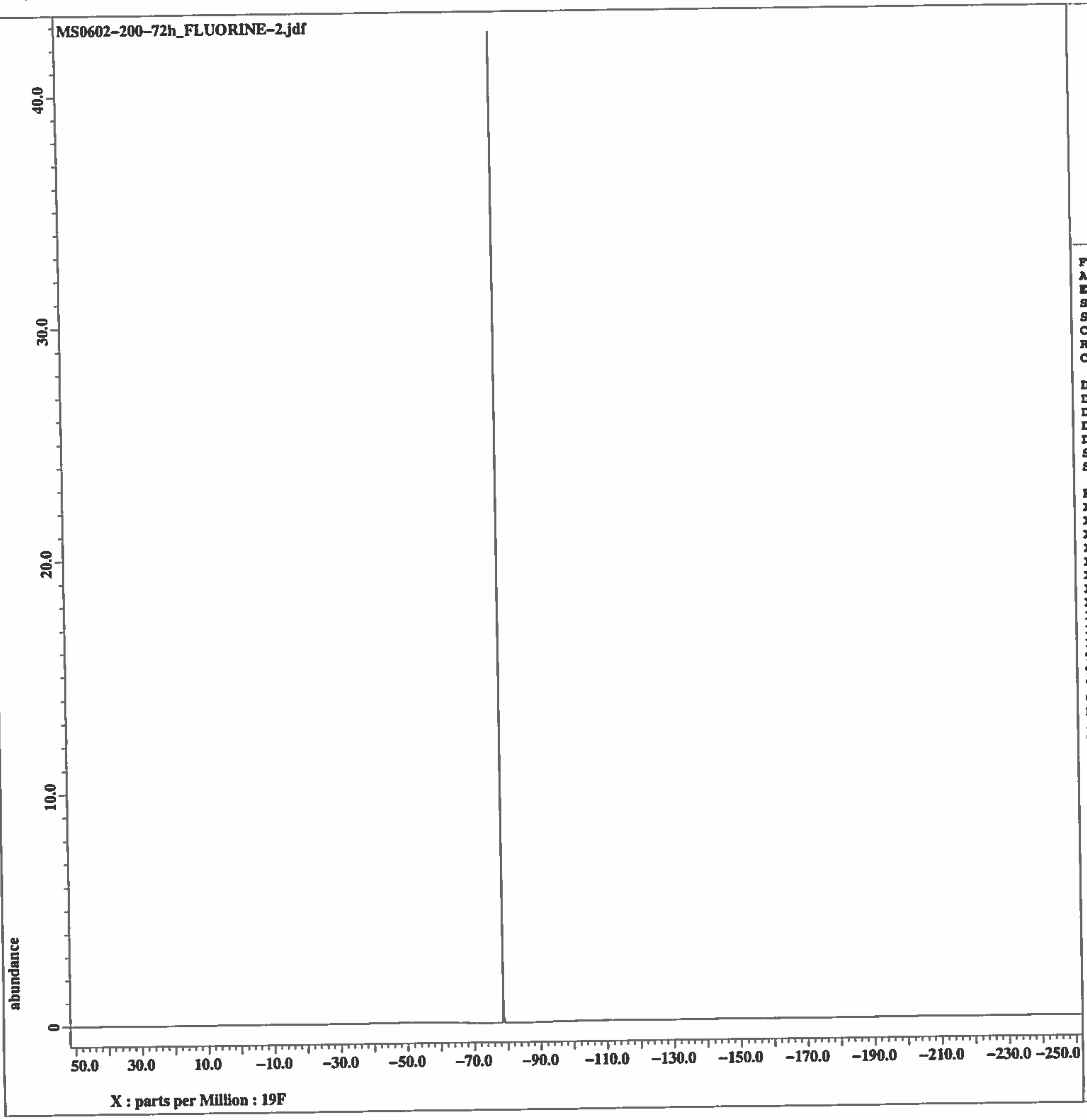


```
Filename           = MS0602-200-72h_FLUORI
Author             = Jim Davis
Experiment         = single_pulse.ex2
Sample_id          = MS0602-200-72h
Solvent            = CHLOROFORM-D
Creation_time      =  8-NOV-2018 19:29:28
Revision_time      =  8-NOV-2018 19:04:45
Current_time       =  8-NOV-2018 19:04:45

Data_format        = 1D COMPLEX
Dim_size           = 104857
Dim_title          = 19F
Dim_units          = [ppm]
Dimensions         = X
Site               = ECA 500
Spectrometer       = JNM-ECA500

Field_strength     = 11.7473579[T] (500[MH
X_acq_duration     = 0.7340032[s]
X_domain           = 19F
X_freq             = 470.62046084[MHz]
X_offset           = -100[ppm]
X_points           = 131072
X_prescans         = 1
X_resolution       = 1.36239188[Hz]
X_sweep            = 178.57142857[kHz]
Irr_domain         = 19F
Irr_freq           = 470.62046084[MHz]
Irr_offset         = 5[ppm]
Tri_domain         = 19F
Tri_freq           = 470.62046084[MHz]
Tri_offset         = 5[ppm]
Clipped            = FALSE
Mod_return         = 1
Scans              = 40
Total_scans        = 40

X_90_width         = 13.1[us]
X_acq_time         = 0.7340032[s]
X_angle            = 45[deg]
X_atn              = 2.5[dB]
X_pulse            = 6.55[us]
Irr_mode           = Off
Tri_mode           = Off
Dante_presat       = FALSE
Initial_wait       = 1[s]
Recvr_gain         = 40
Relaxation_delay   = 4[s]
Repetition_time    = 4.7340032[s]
Temp_get           = 22.9[dC]
```

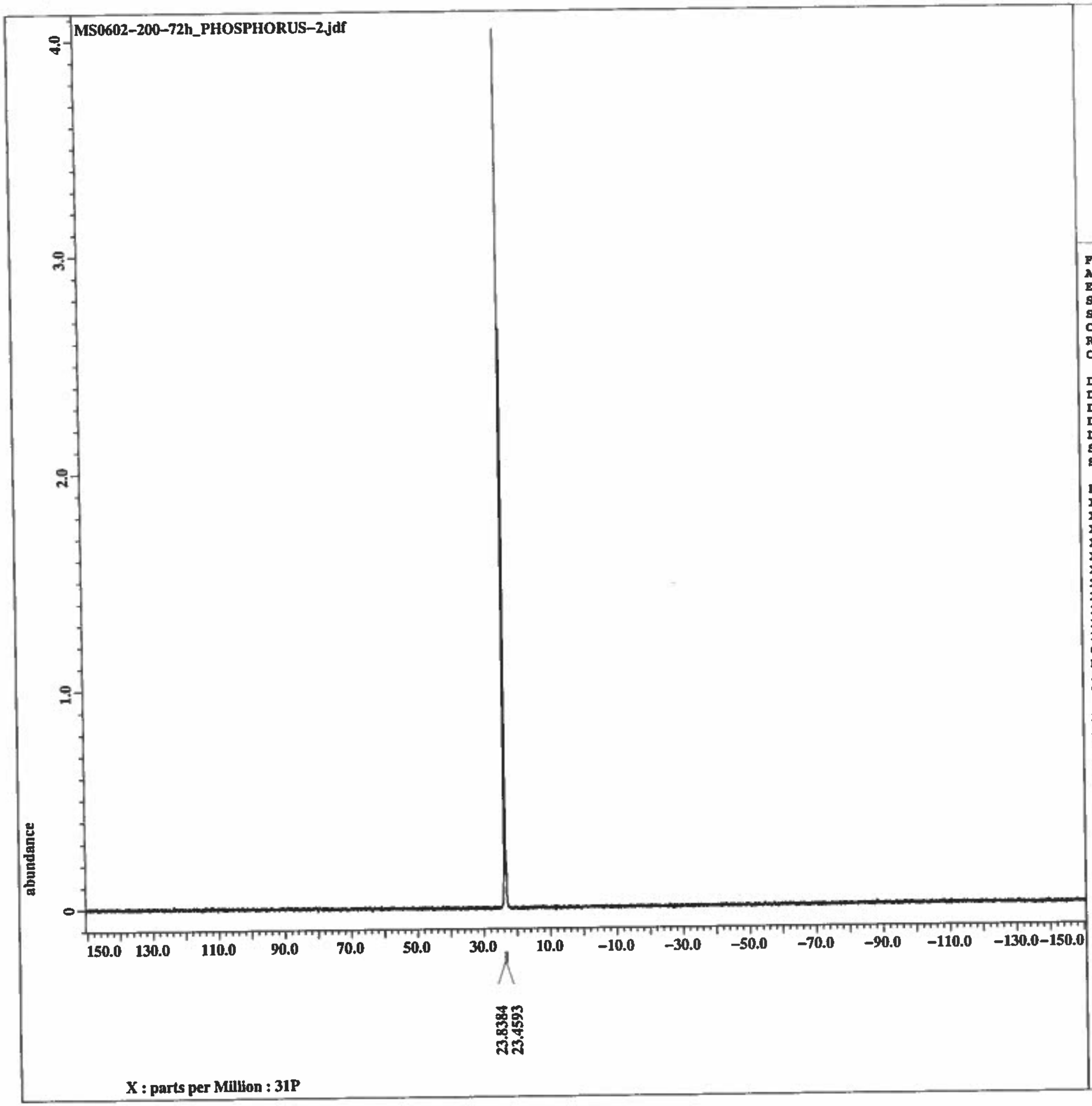

Filename	= MS0602-200-72h_PHOSPH
Author	= Jim Davis
Experiment	= single_pulse_dec
Sample_id	= MS0602-200-72h
Solvent	= CHLOROFORM-D
Creation_time	= 8-NOV-2018 19:34:39
Revision_time	= 8-NOV-2018 19:09:56
Current_time	= 8-NOV-2018 19:09:56
Data_format	= 1D COMPLEX
Dim_size	= 52428
Dim_title	= 31P
Dim_units	= [ppm]
Dimensions	= X
Site	= ECA 500
Spectrometer	= JNM-ECA500
Field_strength	= 11.7473579[T] (500[MH
X_acq_duration	= 0.85983232[s]
X_domain	= 31P
X_freq	= 202.46831075[MHz]
X_offset	= 0[ppm]
X_points	= 65536
X_prescans	= 4
X_resolution	= 1.16301746[Hz]
X_sweep	= 76.2195122[kHz]
Irr_domain	= 1H
Irr_freq	= 500.15991521[MHz]
Irr_offset	= 5.0[ppm]
Clipped	= FALSE
Mod_return	= 1
Scans	= 50
Total_scans	= 50
X_90_width	= 14.687[us]
X_acq_time	= 0.85983232[s]
X_angle	= 30[deg]
X_atn	= 5[dB]
X_pulse	= 4.89566667[us]
Irr_atn_dec	= 20.7[dB]
Irr_atn_noe	= 20.7[dB]
Irr_noise	= WALTZ
Decoupling	= TRUE
Initial_wait	= 1[s]
Noe	= TRUE
Noe_time	= 2[s]
Recvr_gain	= 56
Relaxation_delay	= 2[s]
Repetition_time	= 2.85983232[s]
Temp_get	= 23.2[dC]

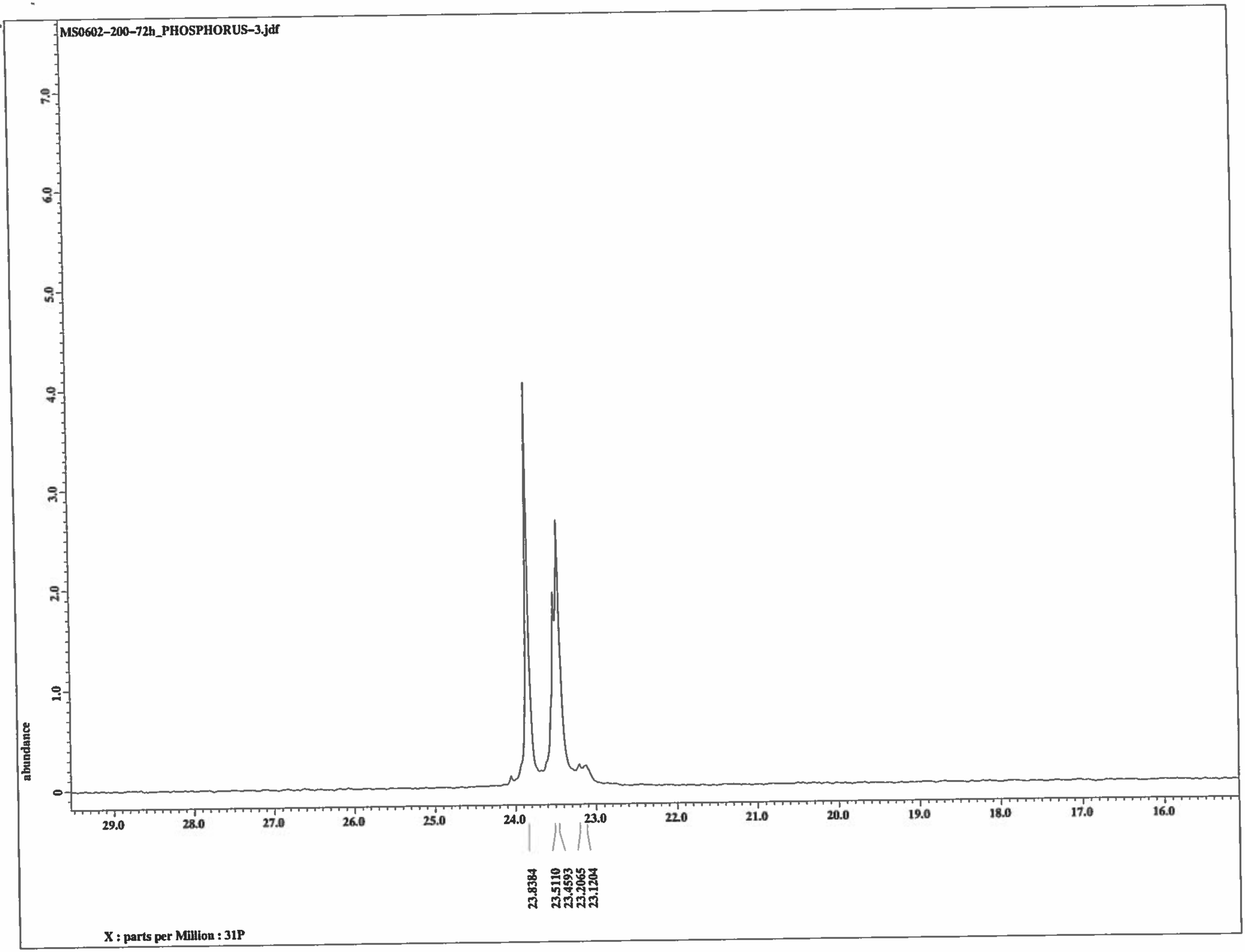

MS0602-200-72h_PHOSPHORUS-3.jdf
abundance
0
1.0
2.0
3.0
4.0
5.0
6.0
7.0
29.0
28.0
27.0
26.0
25.0
24.0
23.0
22.0
21.0
20.0
19.0
18.0
17.0
16.0
23.8384
23.5110
23.4593
23.2065
23.1204
X : parts per Million : 31P

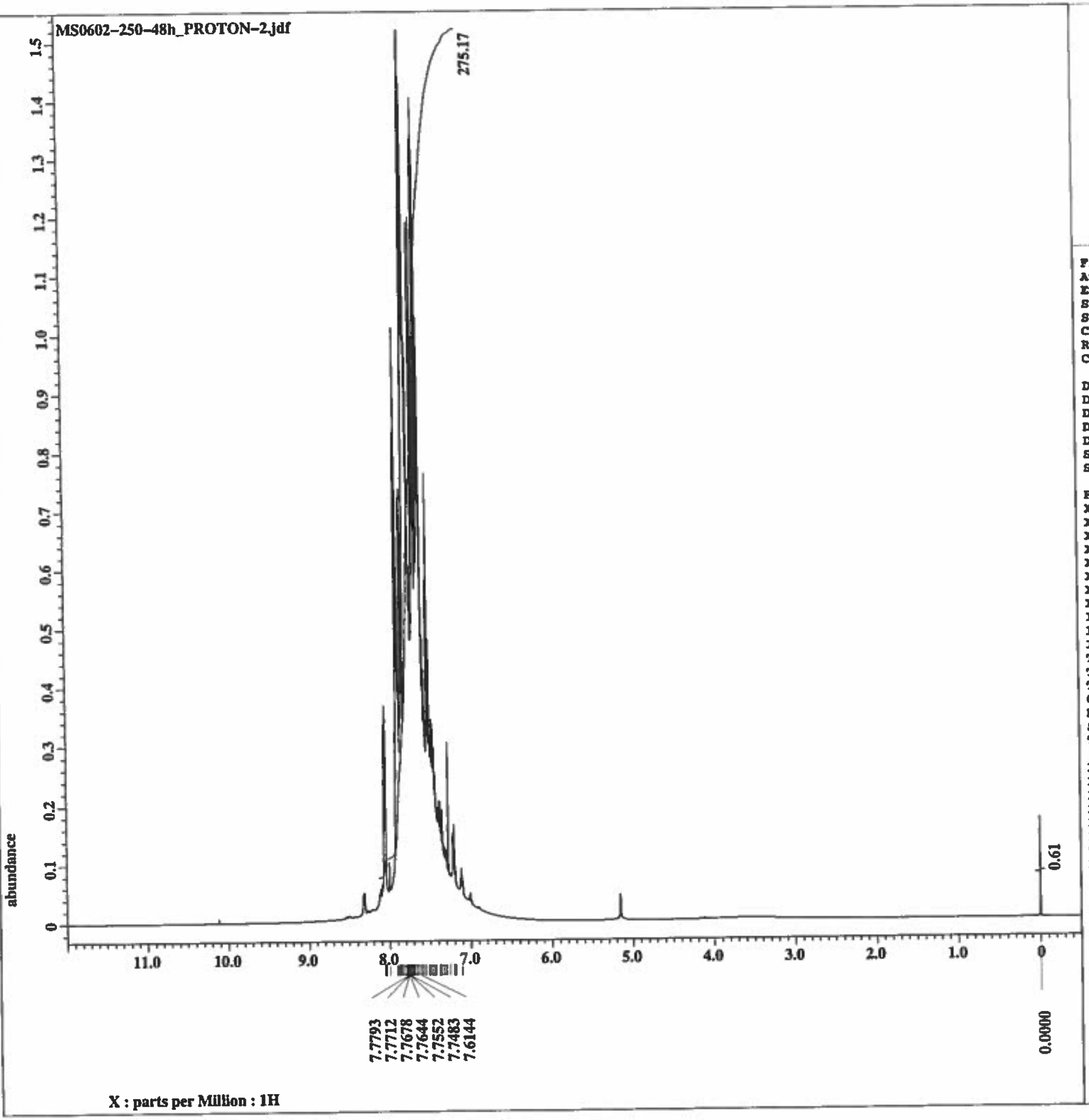

Filename	= MS0602-250-48h_PROTON
Author	= Jim Davis
Experiment	= single_pulse.ex2
Sample_id	= MS0602-250-48h
Solvent	= CHLOROFORM-D
Creation_time	= 8-NOV-2018 19:41:57
Revision_time	= 8-NOV-2018 19:17:14
Current_time	= 8-NOV-2018 19:17:14
Data_format	= 1D COMPLEX
Dim_size	= 13107
Dim_title	= 1H
Dim_units	= [ppm]
Dimensions	= X
Site	= ECA 500
Spectrometer	= JNM-ECA500
Field_strength	= 11.7473579[T] (500[MH
X_acq_duration	= 1.74587904[s]
X_domain	= 1H
X_freq	= 500.15991521[MHz]
X_offset	= 5.0[ppm]
X_points	= 16384
X_prescans	= 1
X_resolution	= 0.57277737[Hz]
X_sweep	= 9.38438438[kHz]
Irr_domain	= 1H
Irr_freq	= 500.15991521[MHz]
Irr_offset	= 5.0[ppm]
Tri_domain	= 1H
Tri_freq	= 500.15991521[MHz]
Tri_offset	= 5.0[ppm]
Clipped	= FALSE
Mod_return	= 1
Scans	= 16
Total_scans	= 16
X_90_width	= 12.4[us]
X_acq_time	= 1.74587904[s]
X_angle	= 45[deg]
X_atn	= 4[dB]
X_pulse	= 6.2[us]
Irr_mode	= Off
Tri_mode	= Off
Dante_presat	= FALSE
Initial_wait	= 1[s]
Recvr_gain	= 28
Relaxation_delay	= 4[s]
Repetition_time	= 5.74587904[s]
Temp_get	= 22.8[dC]

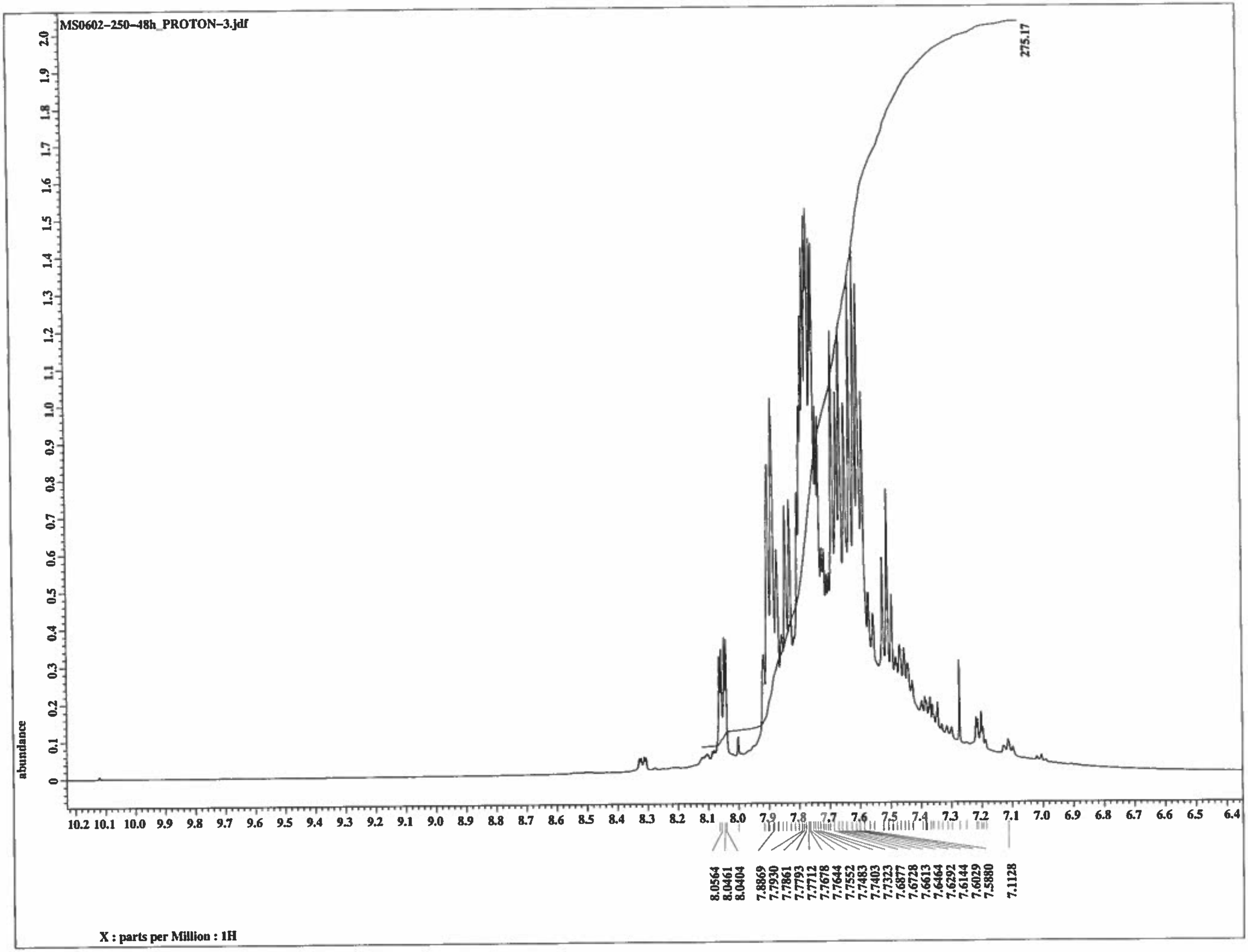

MS0602-250-48h_PROTON-3.jdf
abundance
0 0.1 0.2 0.3 0.4 0.5 0.6 0.7 0.8 0.9 1.0 1.1 1.2 1.3 1.4 1.5 1.6 1.7 1.8 1.9 2.0
275.17
10.2 10.1 10.0 9.9 9.8 9.7 9.6 9.5 9.4 9.3 9.2 9.1 9.0 8.9 8.8 8.7 8.6 8.5 8.4 8.3 8.2 8.1 8.0 7.9 7.8 7.7 7.6 7.5 7.4 7.3 7.2 7.1 7.0 6.9 6.8 6.7 6.6 6.5 6.4
8.0564
8.0461
8.0404
7.8869
7.7930
7.7861
7.7793
7.7712
7.7678
7.7644
7.7552
7.7483
7.7403
7.7323
7.6877
7.6728
7.6613
7.6464
7.6292
7.6144
7.6029
7.5880
7.1128
X : parts per Million : 1H

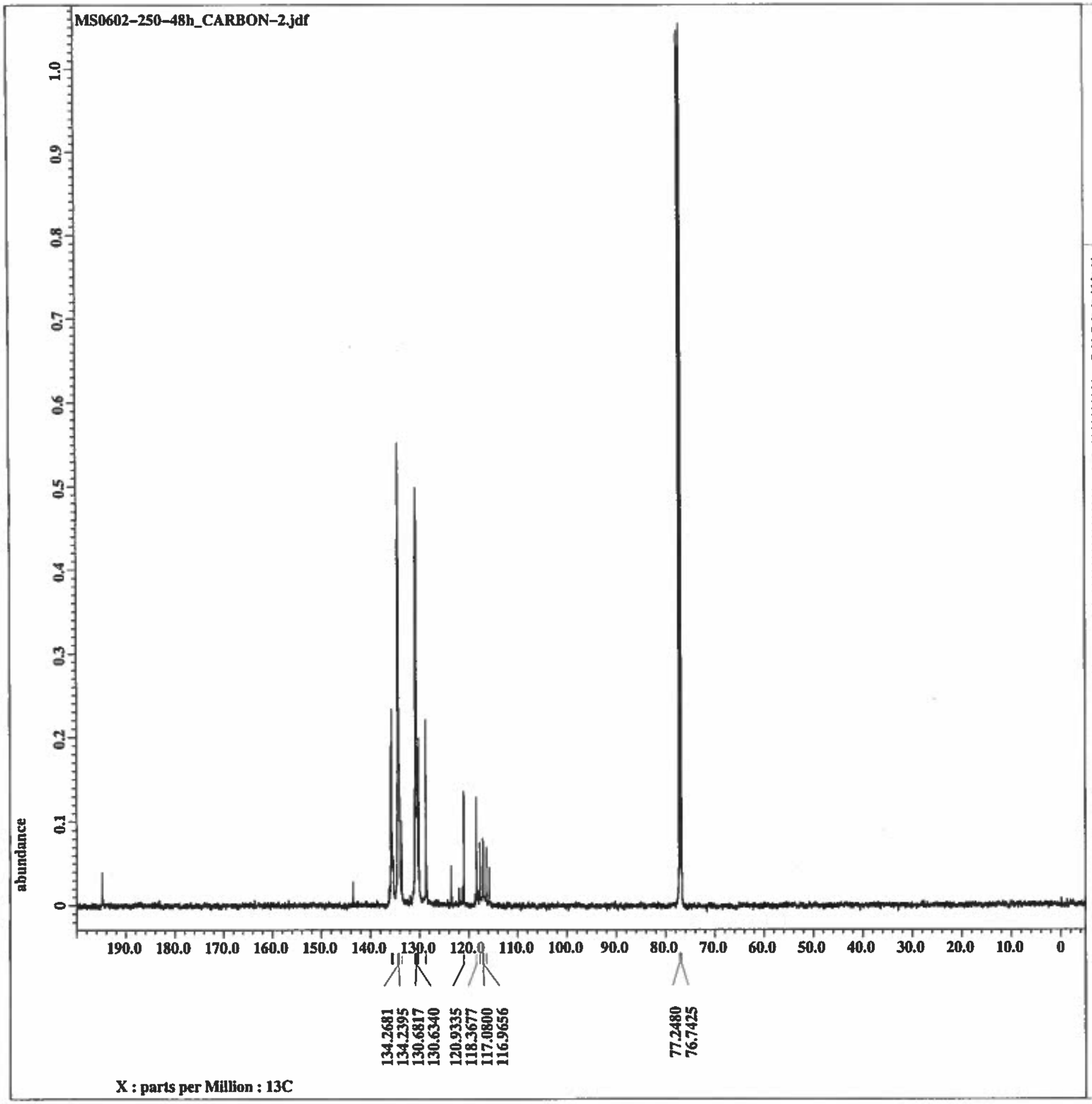

Filename	= MS0602-250-48h_CARBON
Author	= Jim Davis
Experiment	= single_pulse_dec
Sample_id	= MS0602-250-48h
Solvent	= CHLOROFORM-D
Creation_time	= 8-NOV-2018 20:31:36
Revision_time	= 8-NOV-2018 20:06:52
Current_time	= 8-NOV-2018 20:06:53
Data_format	= 1D COMPLEX
Dim_size	= 26214
Dim_title	= 13C
Dim_units	= [ppm]
Dimensions	= X
Site	= ECA 500
Spectrometer	= JNM-ECA500
Field_strength	= 11.7473579[T] (500[MH
X_acq_duration	= 0.83361792[s]
X_domain	= 13C
X_freq	= 125.76529768[MHz]
X_offset	= 100[ppm]
X_points	= 32768
X_prescans	= 4
X_resolution	= 1.19959034[Hz]
X_sweep	= 39.3081761[kHz]
Irr_domain	= 1H
Irr_freq	= 500.15991521[MHz]
Irr_offset	= 5.0[ppm]
Clipped	= FALSE
Mod_return	= 1
Scans	= 1024
Total_scans	= 1024
X_90_width	= 13.2[us]
X_acq_time	= 0.83361792[s]
X_angle	= 30[deg]
X_atn	= 6[dB]
X_pulse	= 4.4[us]
Irr_atn_dec	= 20.7[dB]
Irr_atn_noe	= 20.7[dB]
Irr_noise	= WALTZ
Decoupling	= TRUE
Initial_wait	= 1[s]
Noe	= TRUE
Noe_time	= 2[s]
Recvr_gain	= 60
Relaxation_delay	= 2[s]
Repetition_time	= 2.83361792[s]
Temp_get	= 23.3[dC]

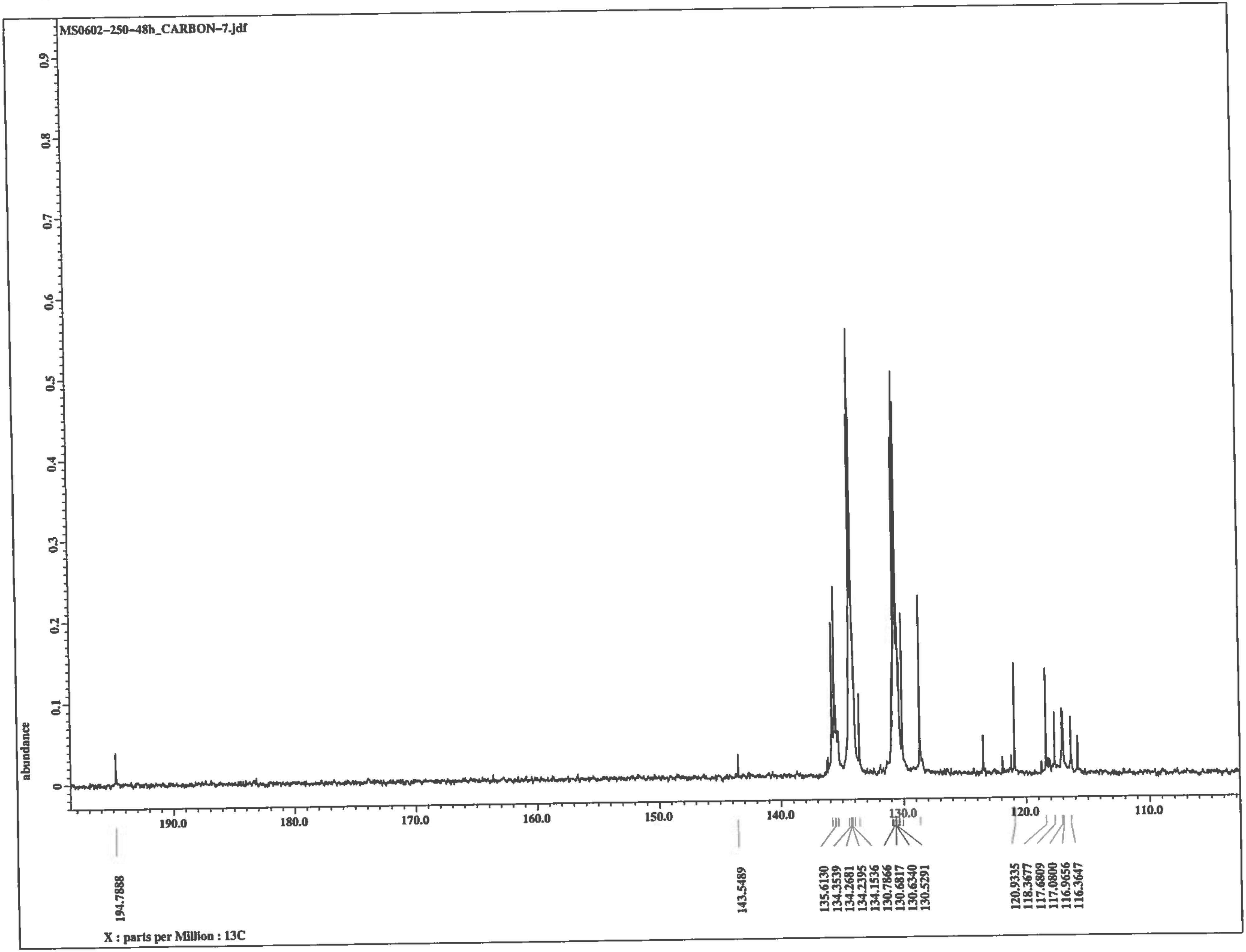
MS0602-250-48h_CARBON-7.jdf
abundance
0
0.1
0.2
0.3
0.4
0.5
0.6
0.7
0.8
0.9
190.0
180.0
170.0
160.0
150.0
140.0
130.0
120.0
110.0
194.7888
143.5489
135.6130
134.3539
134.2681
134.2395
134.1536
130.7866
130.6817
130.6340
130.5291
120.9335
118.3677
117.6809
117.0800
116.9656
116.3647
X : parts per Million : 13C

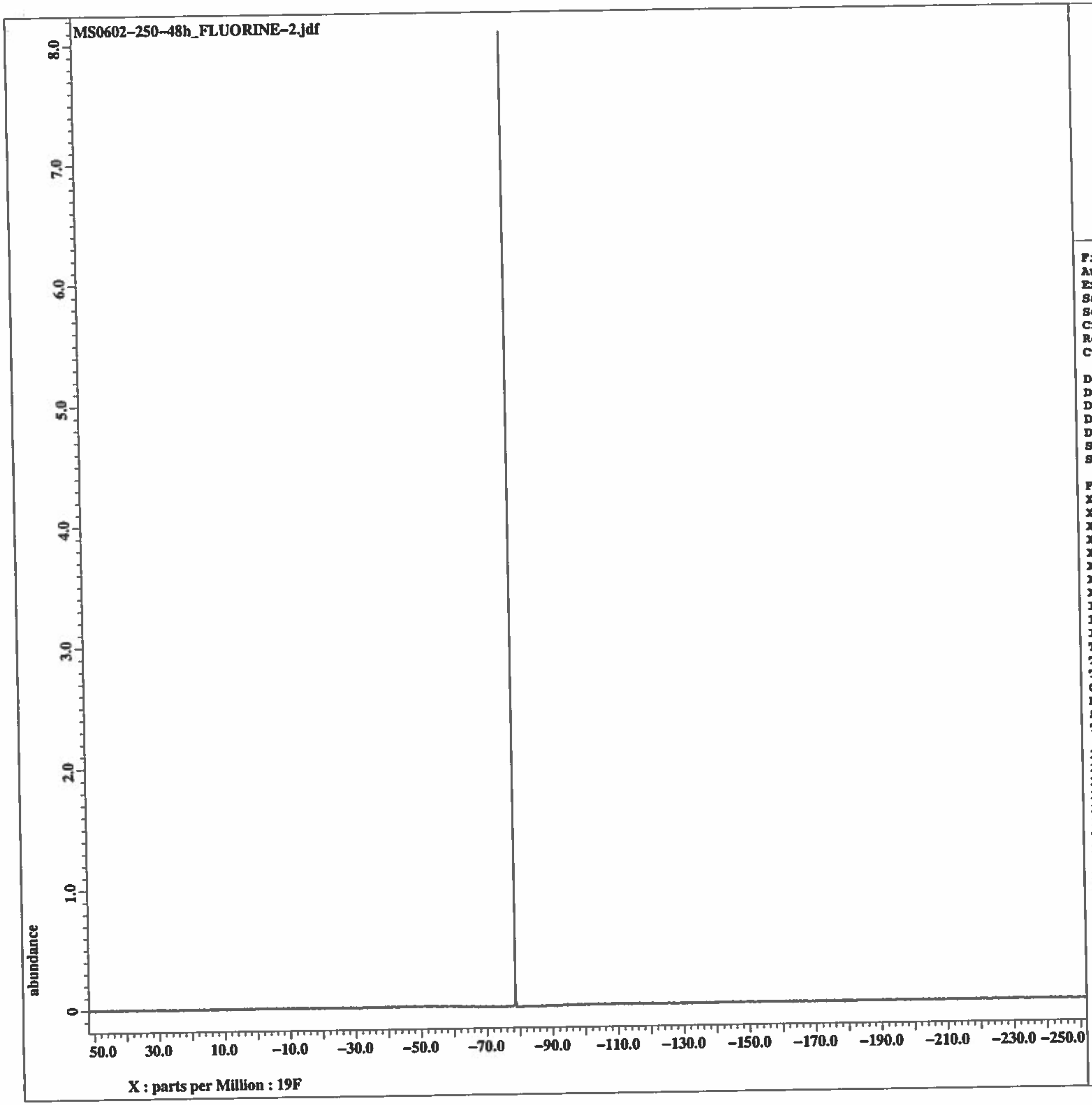

Filename	= MS0602-250-48h_FLUORI
Author	= Jim Davis
Experiment	= single_pulse.ex2
Sample_id	= MS0602-250-48h
Solvent	= CHLOROFORM-D
Creation_time	= 8-NOV-2018 20:37:30
Revision_time	= 8-NOV-2018 20:12:48
Current_time	= 8-NOV-2018 20:12:48
Data_format	= 1D COMPLEX
Dim_size	= 104857
Dim_title	= 19F
Dim_units	= [ppm]
Dimensions	= X
Site	= ECA 500
Spectrometer	= JNM-ECA500
Field_strength	= 11.7473579[T] (500[MH
X_acq_duration	= 0.7340032[s]
X_domain	= 19F
X_freq	= 470.62046084[MHz]
X_offset	= -100[ppm]
X_points	= 131072
X_prescans	= 1
X_resolution	= 1.36239188[Hz]
X_sweep	= 178.57142857[kHz]
Irr_domain	= 19F
Irr_freq	= 470.62046084[MHz]
Irr_offset	= 5[ppm]
Tri_domain	= 19F
Tri_freq	= 470.62046084[MHz]
Tri_offset	= 5[ppm]
Clipped	= FALSE
Mod_return	= 1
Scans	= 40
Total_scans	= 40
X_90_width	= 13.1[us]
X_acq_time	= 0.7340032[s]
X_angle	= 45[deg]
X_atn	= 2.5[dB]
X_pulse	= 6.55[us]
Irr_mode	= Off
Tri_mode	= Off
Dante_presat	= FALSE
Initial_wait	= 1[s]
Recvr_gain	= 70
Relaxation_delay	= 4[s]
Repetition_time	= 4.7340032[s]
Temp_get	= 22.8[dC]

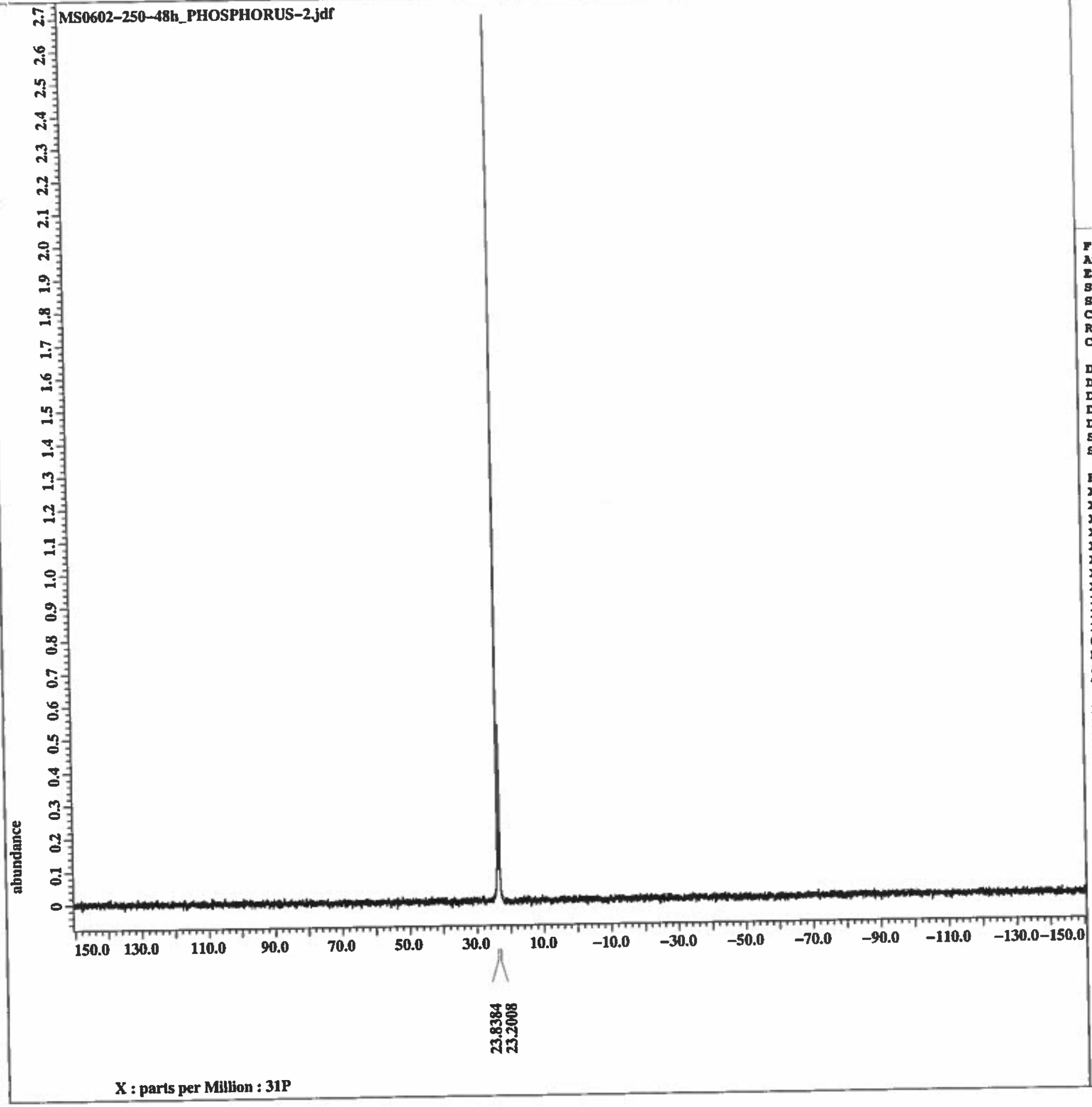

Filename	=	MS0602-250-48h_PHOSPH
Author	=	Jim Davis
Experiment	=	single_pulse_dec
Sample_id	=	MS0602-250-48h
Solvent	=	CHLOROFORM-D
Creation_time	=	8-NOV-2018 20:43:06
Revision_time	=	8-NOV-2018 20:18:23
Current_time	=	8-NOV-2018 20:18:23
Data_format	=	1D COMPLEX
Dim_size	=	52428
Dim_title	=	31P
Dim_units	=	[ppm]
Dimensions	=	X
Site	=	ECA 500
Spectrometer	=	JNM-ECA500
Field_strength	=	11.7473579[T] (500[MH
X_acq_duration	=	0.85983232[s]
X_domain	=	31P
X_freq	=	202.46831075[MHz]
X_offset	=	0[ppm]
X_points	=	65536
X_prescans	=	4
X_resolution	=	1.16301746[Hz]
X_sweep	=	76.2195122[kHz]
Irr_domain	=	1H
Irr_freq	=	500.15991521[MHz]
Irr_offset	=	5.0[ppm]
Clipped	=	FALSE
Mod_return	=	1
Scans	=	50
Total_scans	=	50
X_90_width	=	14.687[us]
X_acq_time	=	0.85983232[s]
X_angle	=	30[deg]
X_atn	=	5[dB]
X_pulse	=	4.89566667[us]
Irr_atn_dec	=	20.7[dB]
Irr_atn_noe	=	20.7[dB]
Irr_noise	=	WALTZ
Decoupling	=	TRUE
Initial_wait	=	1[s]
Noe	=	TRUE
Noe_time	=	2[s]
Recvr_gain	=	58
Relaxation_delay	=	2[s]
Repetition_time	=	2.85983232[s]
Temp_get	=	23[dC]

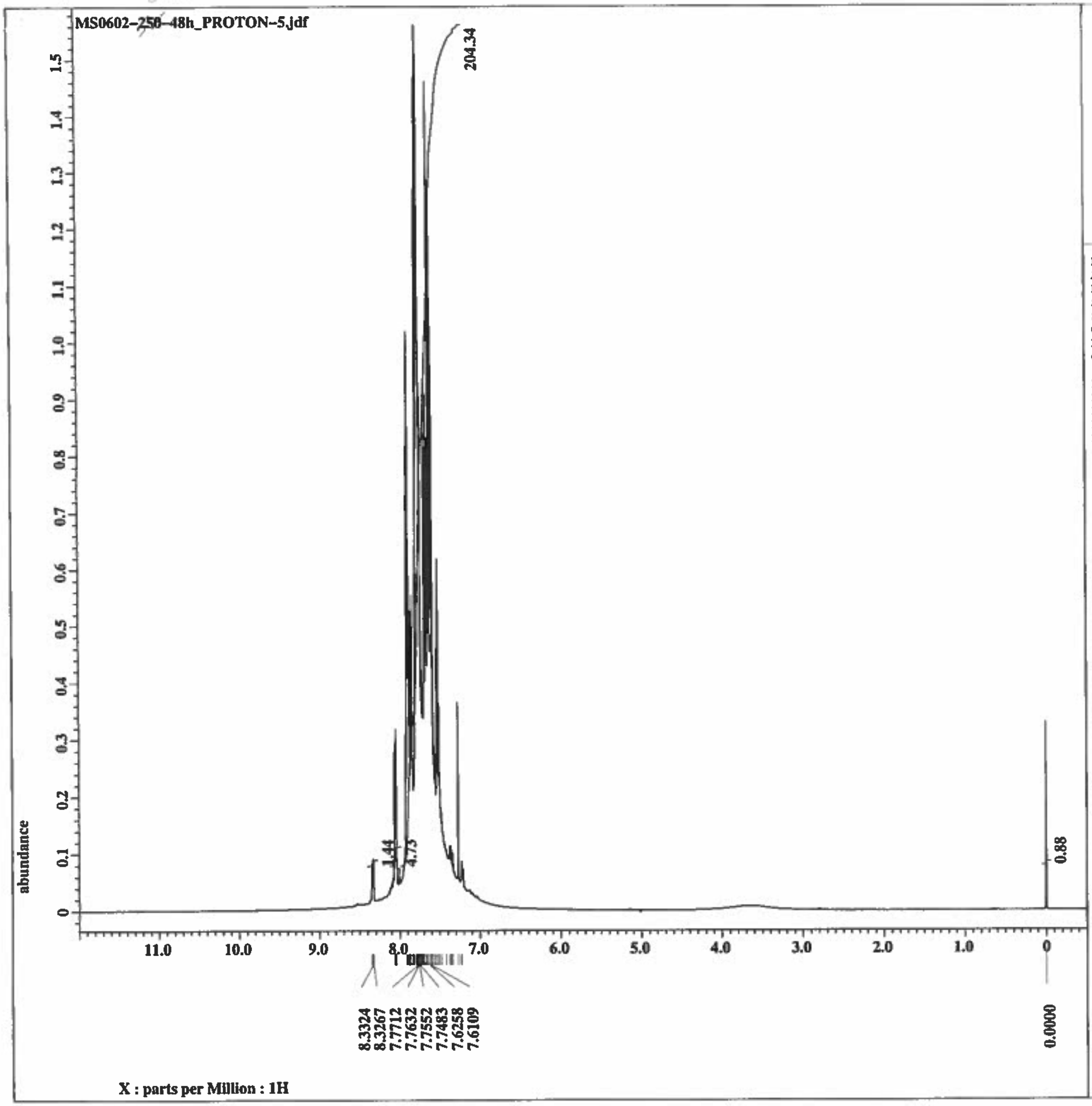

Filename	= MS0602-250-48h_PROTON
Author	= Jim Davis
Experiment	= single_pulse.ex2
Sample_id	= MS0602-250-48h
Solvent	= CHLOROFORM-D
Creation_time	= 8-NOV-2018 20:50:13
Revision_time	= 8-NOV-2018 20:25:29
Current_time	= 8-NOV-2018 20:25:29
Data_format	= 1D COMPLEX
Dim_size	= 13107
Dim_title	= 1H
Dim_units	= [ppm]
Dimensions	= X
Site	= ECA 500
Spectrometer	= JNM-ECA500
Field_strength	= 11.7473579[T] (500[MH
X_acq_duration	= 1.74587904[s]
X_domain	= 1H
X_freq	= 500.15991521[MHz]
X_offset	= 5.0[ppm]
X_points	= 16384
X_prescans	= 1
X_resolution	= 0.57277737[Hz]
X_sweep	= 9.38438438[kHz]
Irr_domain	= 1H
Irr_freq	= 500.15991521[MHz]
Irr_offset	= 5.0[ppm]
Tri_domain	= 1H
Tri_freq	= 500.15991521[MHz]
Tri_offset	= 5.0[ppm]
Clipped	= FALSE
Mod_return	= 1
Scans	= 16
Total_scans	= 16
X_90_width	= 12.4[us]
X_acq_time	= 1.74587904[s]
X_angle	= 45[deg]
X_atn	= 4[dB]
X_pulse	= 6.2[us]
Irr_mode	= Off
Tri_mode	= Off
Dante_presat	= FALSE
Initial_wait	= 1[s]
Recvr_gain	= 30
Relaxation_delay	= 4[s]
Repetition_time	= 5.74587904[s]
Temp_get	= 22.7[dC]

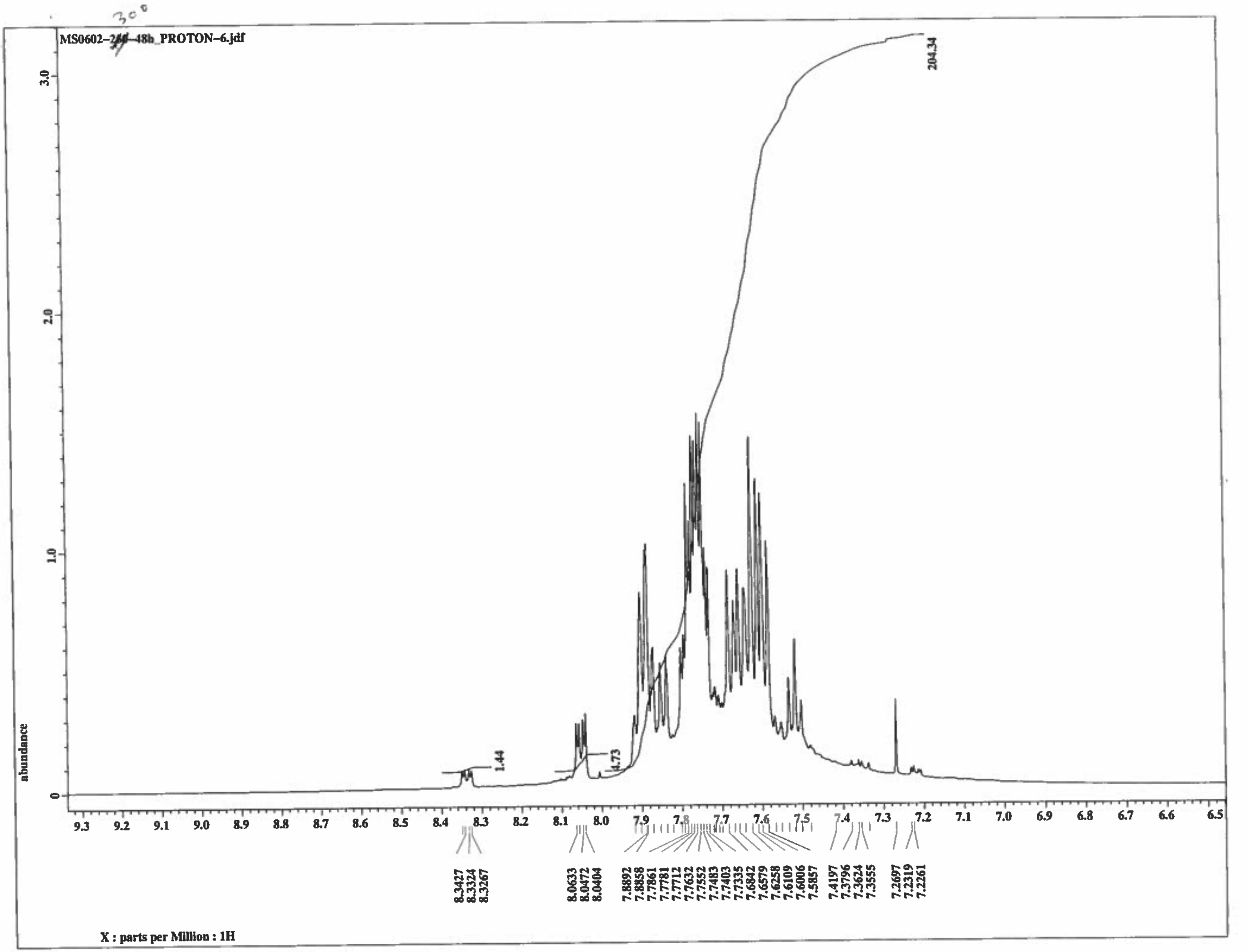

MS0602-2[illegible]-48h_PROTON-6.jdf
abundance
0
1.0
2.0
3.0
9.3 9.2 9.1 9.0 8.9 8.8 8.7 8.6 8.5 8.4 8.3 8.2 8.1 8.0 7.9 7.8 7.7 7.6 7.5 7.4 7.3 7.2 7.1 7.0 6.9 6.8 6.7 6.6 6.5
1.44
4.73
204.34
8.3427
8.3324
8.3267
8.0633
8.0472
8.0404
7.8892
7.8858
7.7861
7.7781
7.7712
7.7632
7.7552
7.7483
7.7403
7.7335
7.6842
7.6579
7.6258
7.6109
7.6006
7.5857
7.4197
7.3796
7.3624
7.3555
7.2697
7.2319
7.2261
X : parts per Million : 1H

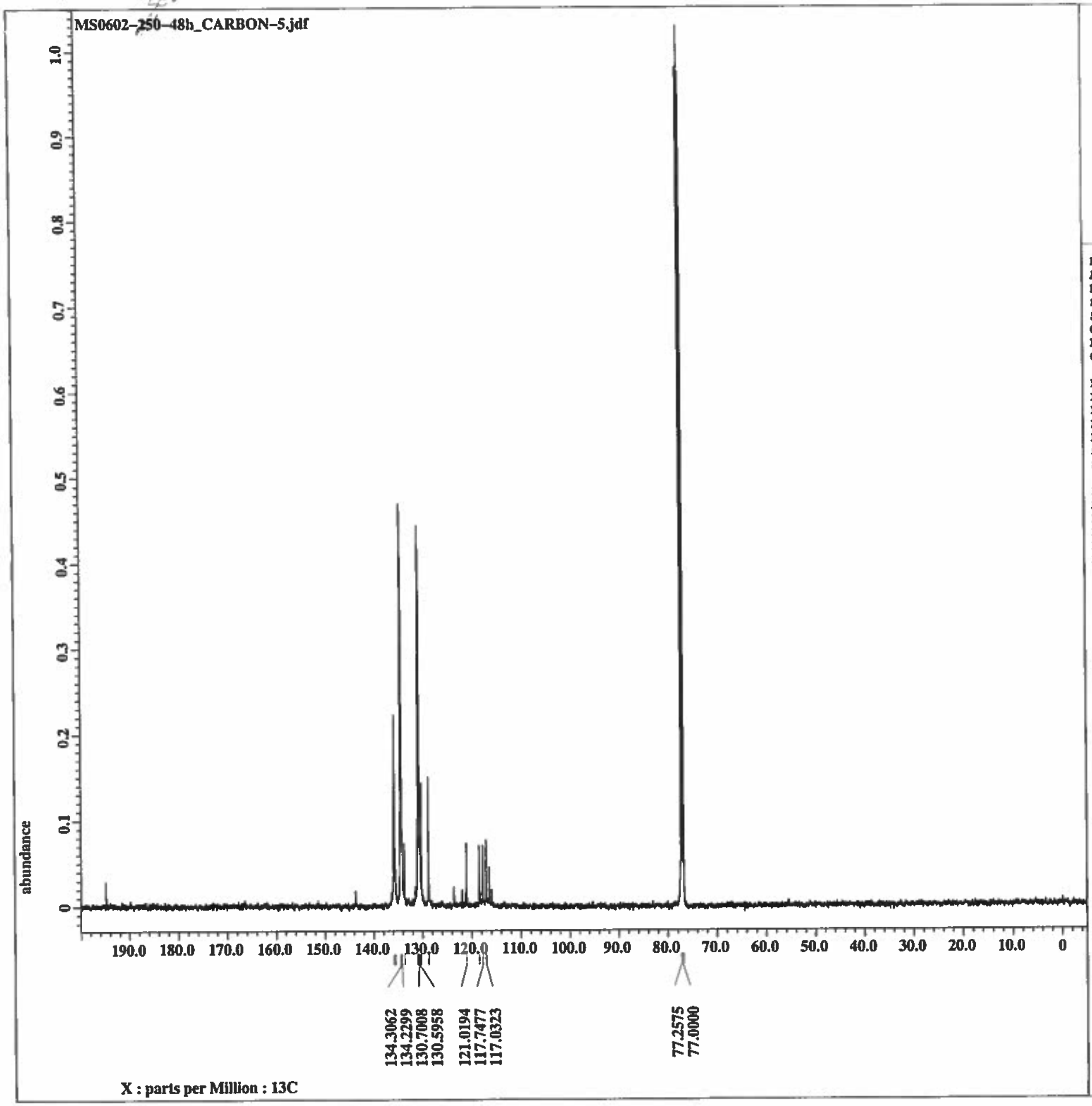


```
Filename           = MS0602-250-48h_CARBON
Author             = Jim Davis
Experiment         = single_pulse_dec
Sample_id          = MS0602-250-48h
Solvent            = CHLOROFORM-D
Creation_time      =  8-NOV-2018 21:39:53
Revision_time      =  8-NOV-2018 21:15:09
Current_time       =  8-NOV-2018 21:15:09

Data_format        = 1D COMPLEX
Dim_size           = 26214
Dim_title          = 13C
Dim_units          = [ppm]
Dimensions         = X
Site               = ECA 500
Spectrometer       = JNM-ECA500

Field_strength     = 11.7473579[T] (500[MH
X_acq_duration     = 0.83361792[s]
X_domain           = 13C
X_freq             = 125.76529768[MHz]
X_offset           = 100[ppm]
X_points           = 32768
X_prescans         = 4
X_resolution       = 1.19959034[Hz]
X_sweep            = 39.3081761[kHz]
Irr_domain         = 1H
Irr_freq           = 500.15991521[MHz]
Irr_offset         = 5.0[ppm]
Clipped            = FALSE
Mod_return         = 1
Scans              = 1024
Total_scans        = 1024

X_90_width         = 13.2[us]
X_acq_time         = 0.83361792[s]
X_angle            = 30[deg]
X_atn              = 6[dB]
X_pulse            = 4.4[us]
Irr_atn_dec        = 20.7[dB]
Irr_atn_noe        = 20.7[dB]
Irr_noise          = WALTZ
Decoupling         = TRUE
Initial_wait       = 1[s]
Noe                = TRUE
Noe_time           = 2[s]
Recvr_gain         = 60
Relaxation_delay   = 2[s]
Repetition_time    = 2.83361792[s]
Temp_get           = 23.1[dC]
```

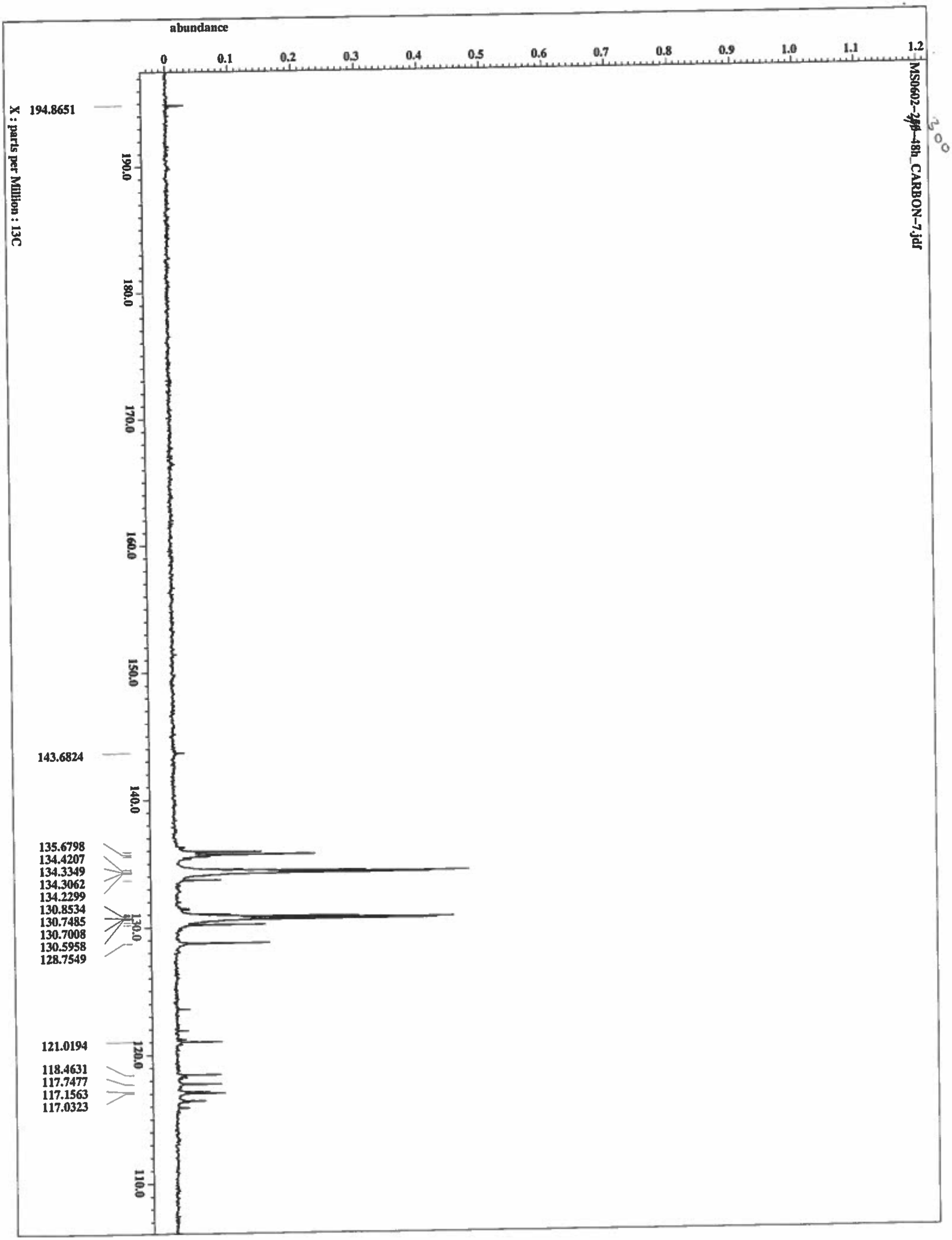

abundance
0
0.1
0.2
0.3
0.4
0.5
0.6
0.7
0.8
0.9
1.0
1.1
1.2
MS0602-2[illegible]-48h_CARBON-7.jdf
X : parts per Million : 13C
194.8651
190.0
180.0
170.0
160.0
150.0
143.6824
140.0
135.6798
134.4207
134.3349
134.3062
134.2299
130.8534
130.7485
130.7008
130.5958
128.7549
130.0
121.0194
120.0
118.4631
117.7477
117.1563
117.0323
110.0

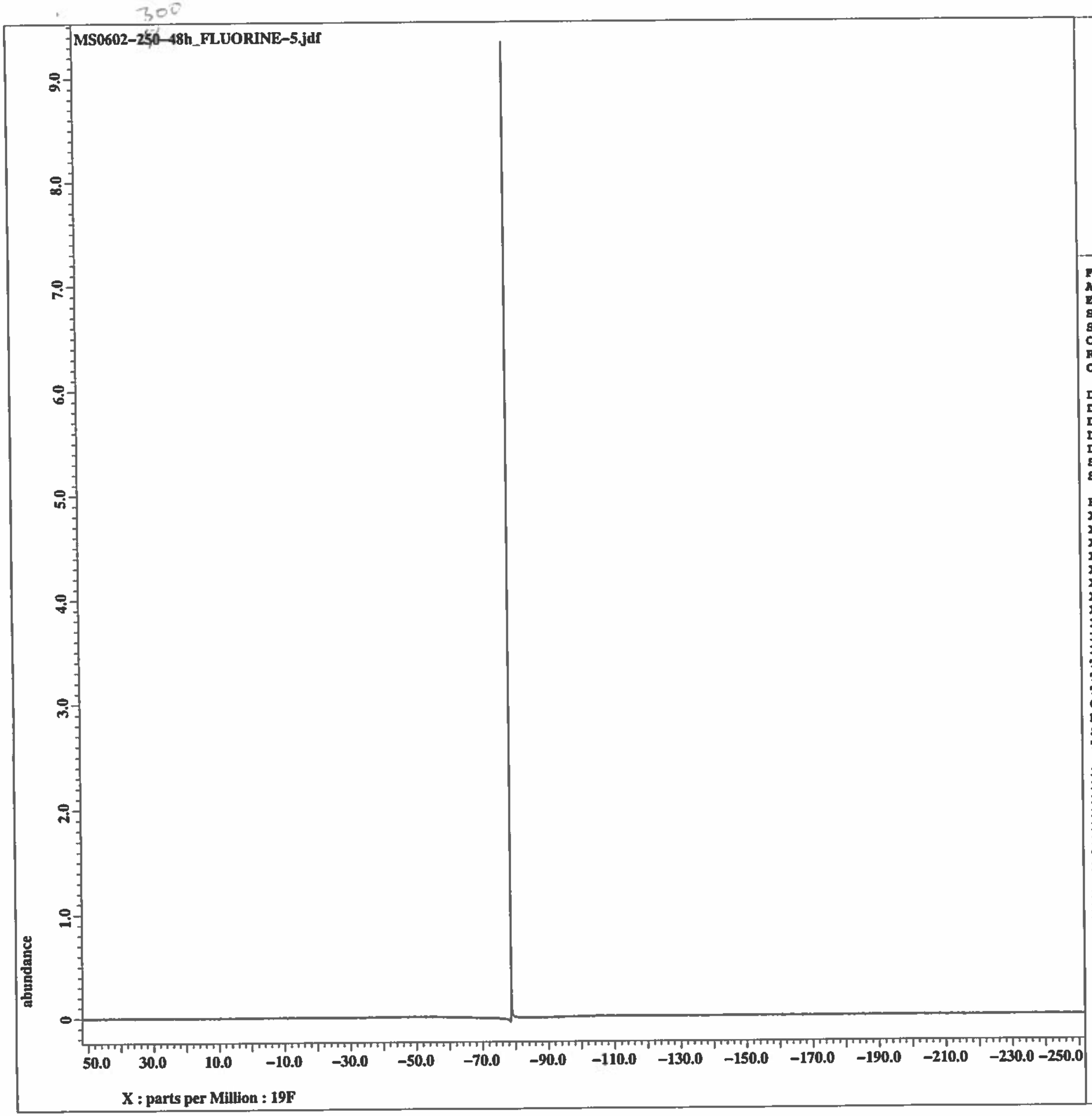

Filename	= MS0602-250-48h_FLUORI
Author	= Jim Davis
Experiment	= single_pulse.ex2
Sample_id	= MS0602-250-48h
Solvent	= CHLOROFORM-D
Creation_time	= 8-NOV-2018 21:45:50
Revision_time	= 8-NOV-2018 21:21:08
Current_time	= 8-NOV-2018 21:21:08
Data_format	= 1D COMPLEX
Dim_size	= 104857
Dim_title	= 19F
Dim_units	= [ppm]
Dimensions	= X
Site	= ECA 500
Spectrometer	= JNM-ECA500
Field_strength	= 11.7473579[T] (500[MH
X_acq_duration	= 0.7340032[s]
X_domain	= 19F
X_freq	= 470.62046084[MHz]
X_offset	= -100[ppm]
X_points	= 131072
X_prescans	= 1
X_resolution	= 1.36239188[Hz]
X_sweep	= 178.57142857[kHz]
Irr_domain	= 19F
Irr_freq	= 470.62046084[MHz]
Irr_offset	= 5[ppm]
Tri_domain	= 19F
Tri_freq	= 470.62046084[MHz]
Tri_offset	= 5[ppm]
Clipped	= FALSE
Mod_return	= 1
Scans	= 40
Total_scans	= 40
X_90_width	= 13.1[us]
X_acq_time	= 0.7340032[s]
X_angle	= 45[deg]
X_atn	= 2.5[dB]
X_pulse	= 6.55[us]
Irr_mode	= Off
Tri_mode	= Off
Dante_presat	= FALSE
Initial_wait	= 1[s]
Recvr_gain	= 68
Relaxation_delay	= 4[s]
Repetition_time	= 4.7340032[s]
Temp_get	= 22.7[dC]

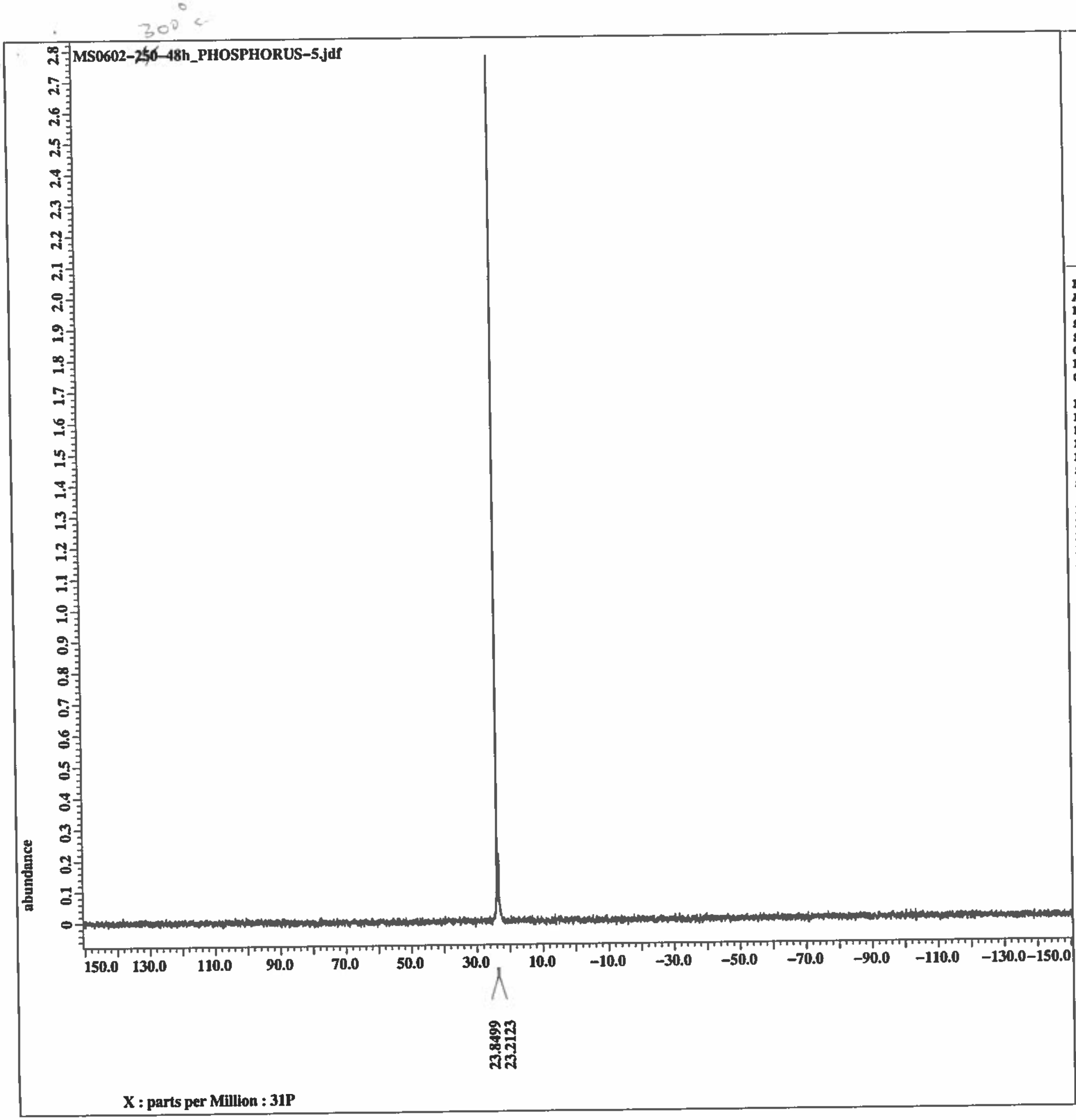

Filename	= MS0602-250-48h_PHOSPH
Author	= Jim Davis
Experiment	= single_pulse_dec
Sample_id	= MS0602-250-48h
Solvent	= CHLOROFORM-D
Creation_time	= 8-NOV-2018 21:50:58
Revision_time	= 8-NOV-2018 21:26:15
Current_time	= 8-NOV-2018 21:26:15
Data_format	= 1D COMPLEX
Dim_size	= 52428
Dim_title	= 31P
Dim_units	= [ppm]
Dimensions	= X
Site	= ECA 500
Spectrometer	= JNM-ECA500
Field_strength	= 11.7473579[T] (500[MH
X_acq_duration	= 0.85983232[s]
X_domain	= 31P
X_freq	= 202.46831075[MHz]
X_offset	= 0[ppm]
X_points	= 65536
X_prescans	= 4
X_resolution	= 1.16301746[Hz]
X_sweep	= 76.2195122[kHz]
Irr_domain	= 1H
Irr_freq	= 500.15991521[MHz]
Irr_offset	= 5.0[ppm]
Clipped	= FALSE
Mod_return	= 1
Scans	= 50
Total_scans	= 50
X_90_width	= 14.687[us]
X_acq_time	= 0.85983232[s]
X_angle	= 30[deg]
X_atn	= 5[dB]
X_pulse	= 4.89566667[us]
Irr_atn_dec	= 20.7[dB]
Irr_atn_noe	= 20.7[dB]
Irr_noise	= WALTZ
Decoupling	= TRUE
Initial_wait	= 1[s]
Noe	= TRUE
Noe_time	= 2[s]
Recvr_gain	= 58
Relaxation_delay	= 2[s]
Repetition_time	= 2.85983232[s]
Temp_get	= 23.1[dC]

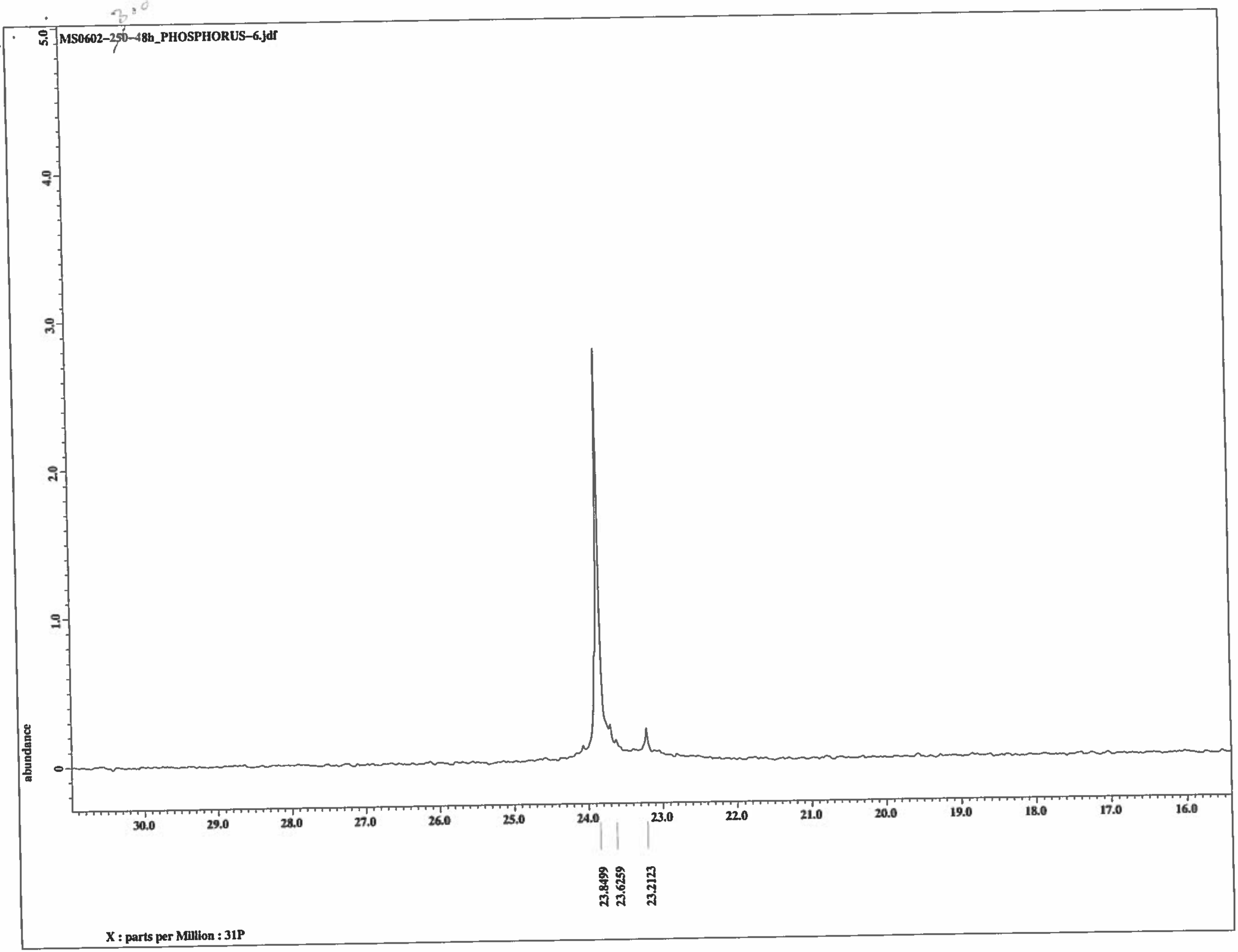
MS0602-250-48h_PHOSPHORUS-6.jdf
abundance
0
1.0
2.0
3.0
4.0
5.0
30.0
29.0
28.0
27.0
26.0
25.0
24.0
23.0
22.0
21.0
20.0
19.0
18.0
17.0
16.0
23.8499
23.6259
23.2123
X : parts per Million : 31P

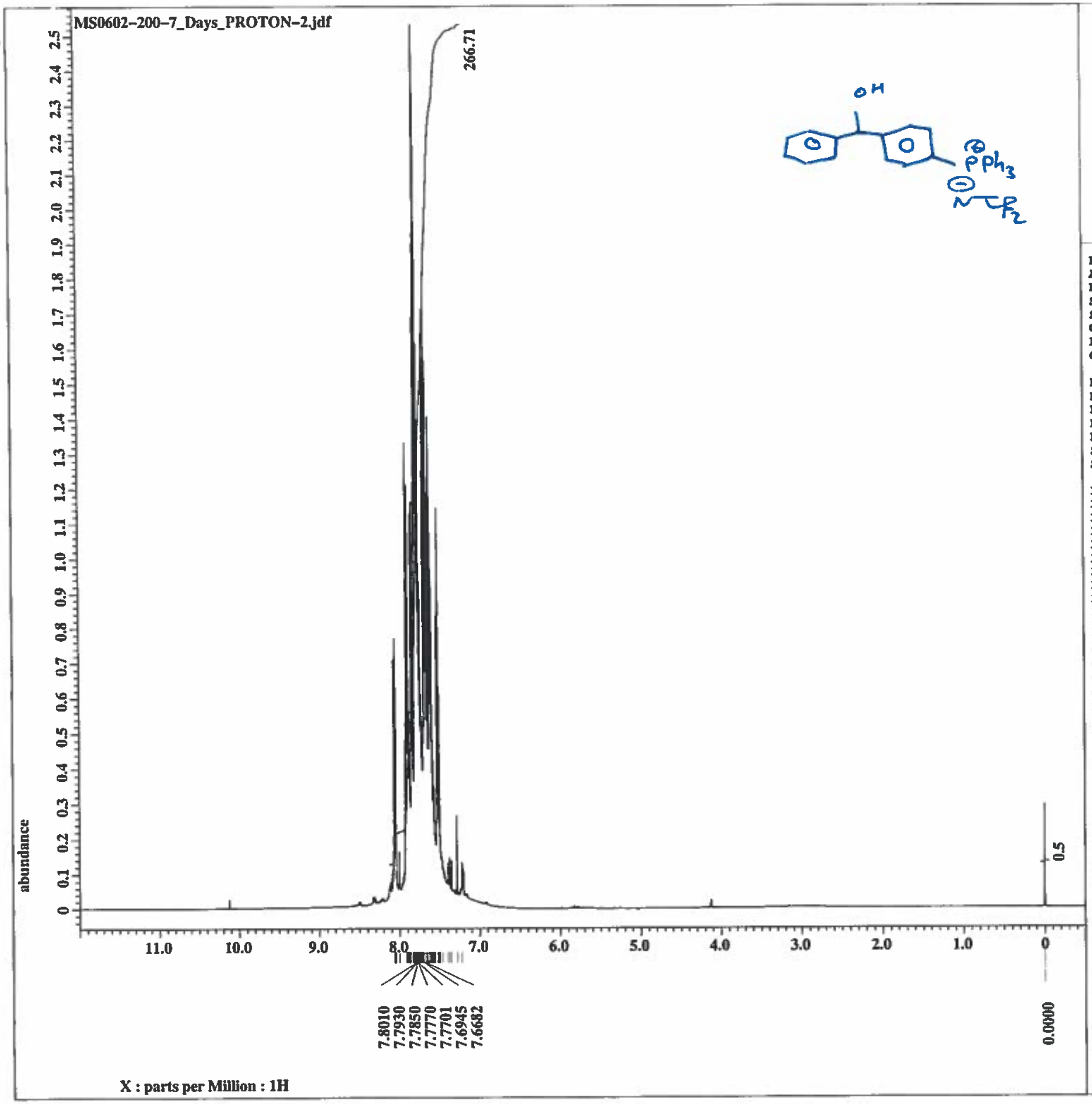

Filename	= MS0602-200-7_Days_PRO
Author	= Jim Davis
Experiment	= single_pulse.ex2
Sample_id	= MS0602-200-7_Days
Solvent	= CHLOROFORM-D
Creation_time	= 19-NOV-2018 16:19:11
Revision_time	= 19-NOV-2018 15:55:02
Current_time	= 19-NOV-2018 15:55:02
Data_format	= 1D COMPLEX
Dim_size	= 13107
Dim_title	= 1H
Dim_units	= [ppm]
Dimensions	= X
Site	= ECA 500
Spectrometer	= JNM-ECA500
Field_strength	= 11.7473579[T] (500[MH
X_acq_duration	= 1.74587904[s]
X_domain	= 1H
X_freq	= 500.15991521[MHz]
X_offset	= 5.0[ppm]
X_points	= 16384
X_prescans	= 1
X_resolution	= 0.57277737[Hz]
X_sweep	= 9.38438438[kHz]
Irr_domain	= 1H
Irr_freq	= 500.15991521[MHz]
Irr_offset	= 5.0[ppm]
Tri_domain	= 1H
Tri_freq	= 500.15991521[MHz]
Tri_offset	= 5.0[ppm]
Clipped	= FALSE
Mod_return	= 1
Scans	= 16
Total_scans	= 16
X_90_width	= 12.4[us]
X_acq_time	= 1.74587904[s]
X_angle	= 45[deg]
X_atn	= 4[dB]
X_pulse	= 6.2[us]
Irr_mode	= Off
Tri_mode	= Off
Dante_presat	= FALSE
Initial_wait	= 1[s]
Recvr_gain	= 26
Relaxation_delay	= 4[s]
Repetition_time	= 5.74587904[s]
Temp_get	= 21.5[dC]

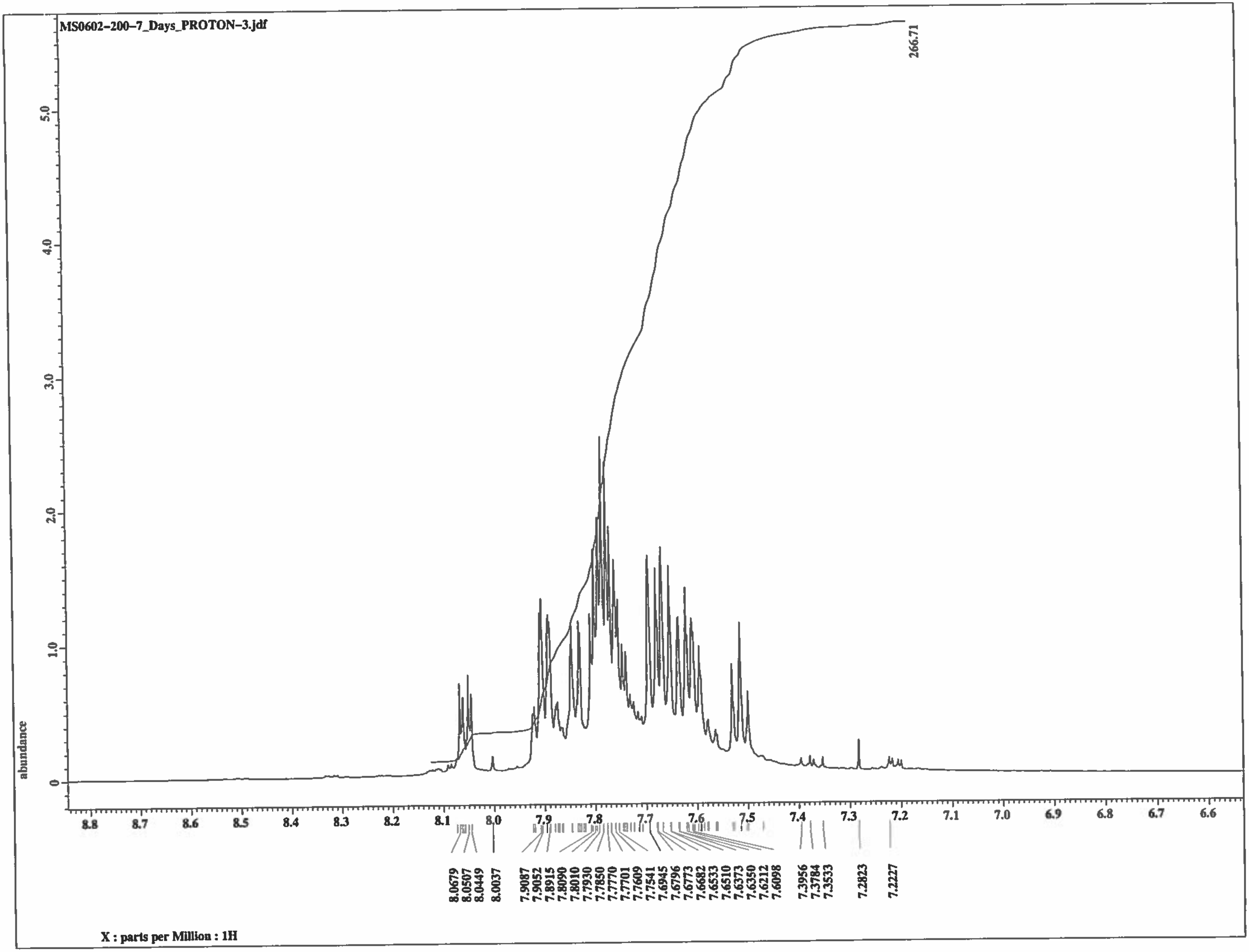

MS0602-200-7_Days_PROTON-3.jdf
abundance
0
1.0
2.0
3.0
4.0
5.0
266.71
8.8
8.7
8.6
8.5
8.4
8.3
8.2
8.1
8.0
7.9
7.8
7.7
7.6
7.5
7.4
7.3
7.2
7.1
7.0
6.9
6.8
6.7
6.6
8.0679
8.0507
8.0449
8.0037
7.9087
7.9052
7.8915
7.8090
7.8010
7.7930
7.7850
7.7770
7.7701
7.7609
7.7541
7.6945
7.6796
7.6773
7.6682
7.6533
7.6510
7.6373
7.6350
7.6212
7.6098
7.3956
7.3784
7.3533
7.2823
7.2227
X : parts per Million : 1H

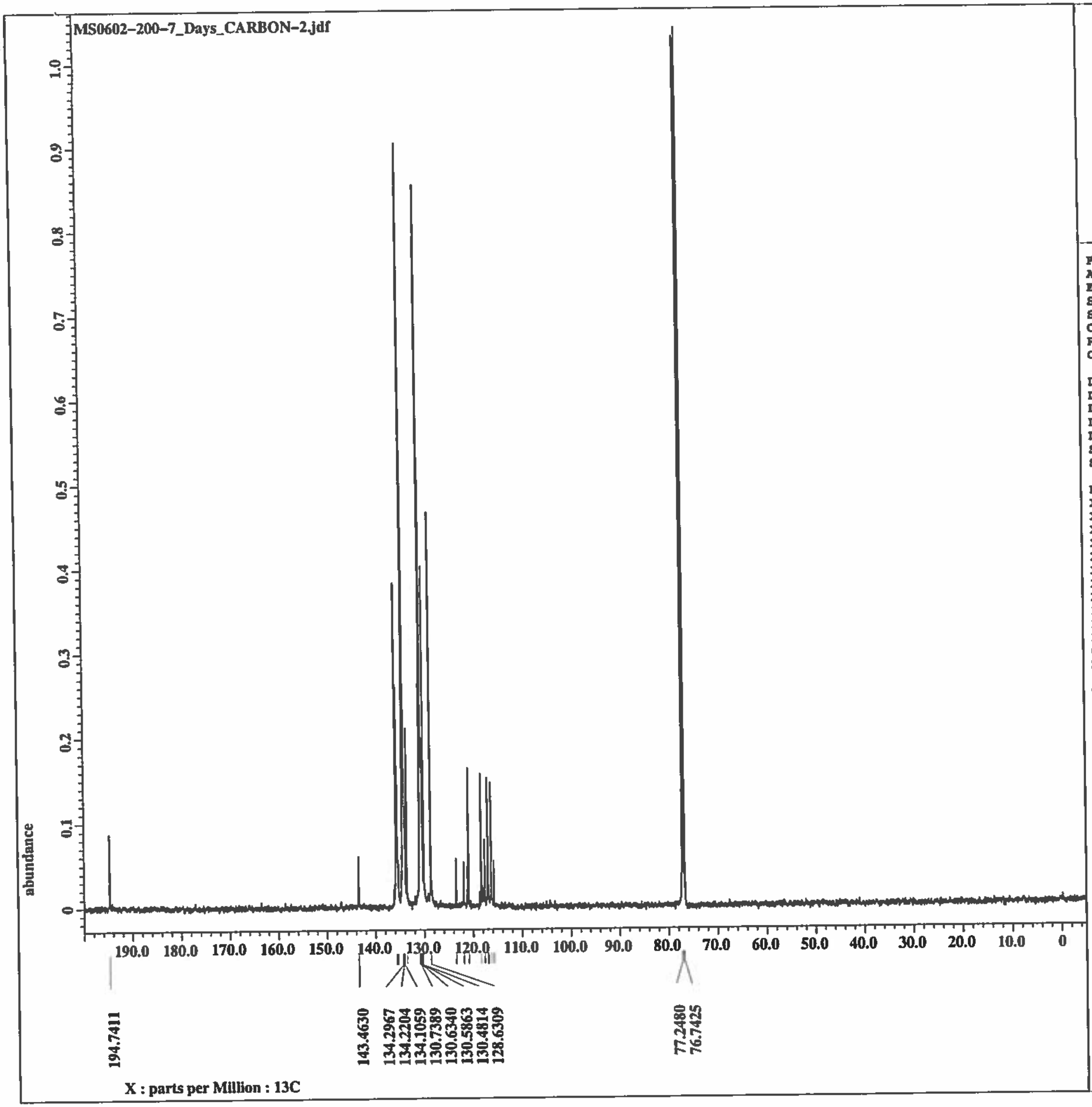

Filename	= MS0602-200-7_Days_CAR
Author	= Jim Davis
Experiment	= single_pulse_dec
Sample_id	= MS0602-200-7_Days
Solvent	= CHLOROFORM-D
Creation_time	= 20-NOV-2018 00:22:06
Revision_time	= 19-NOV-2018 23:57:56
Current_time	= 19-NOV-2018 23:57:56
Data_format	= 1D COMPLEX
Dim_size	= 26214
Dim_title	= 13C
Dim_units	= [ppm]
Dimensions	= X
Site	= ECA 500
Spectrometer	= JNM-ECA500
Field_strength	= 11.7473579[T] (500[MH
X_acq_duration	= 0.83361792[s]
X_domain	= 13C
X_freq	= 125.76529768[MHz]
X_offset	= 100[ppm]
X_points	= 32768
X_prescans	= 4
X_resolution	= 1.19959034[Hz]
X_sweep	= 39.3081761[kHz]
Irr_domain	= 1H
Irr_freq	= 500.15991521[MHz]
Irr_offset	= 5.0[ppm]
Clipped	= FALSE
Mod_return	= 1
Scans	= 1024
Total_scans	= 1024
X_90_width	= 13.2[us]
X_acq_time	= 0.83361792[s]
X_angle	= 30[deg]
X_atn	= 6[dB]
X_pulse	= 4.4[us]
Irr_atn_dec	= 20.7[dB]
Irr_atn_noe	= 20.7[dB]
Irr_noise	= WALTZ
Decoupling	= TRUE
Initial_wait	= 1[s]
Noe	= TRUE
Noe_time	= 2[s]
Recvr_gain	= 60
Relaxation_delay	= 2[s]
Repetition_time	= 2.83361792[s]
Temp_get	= 22.5[dC]

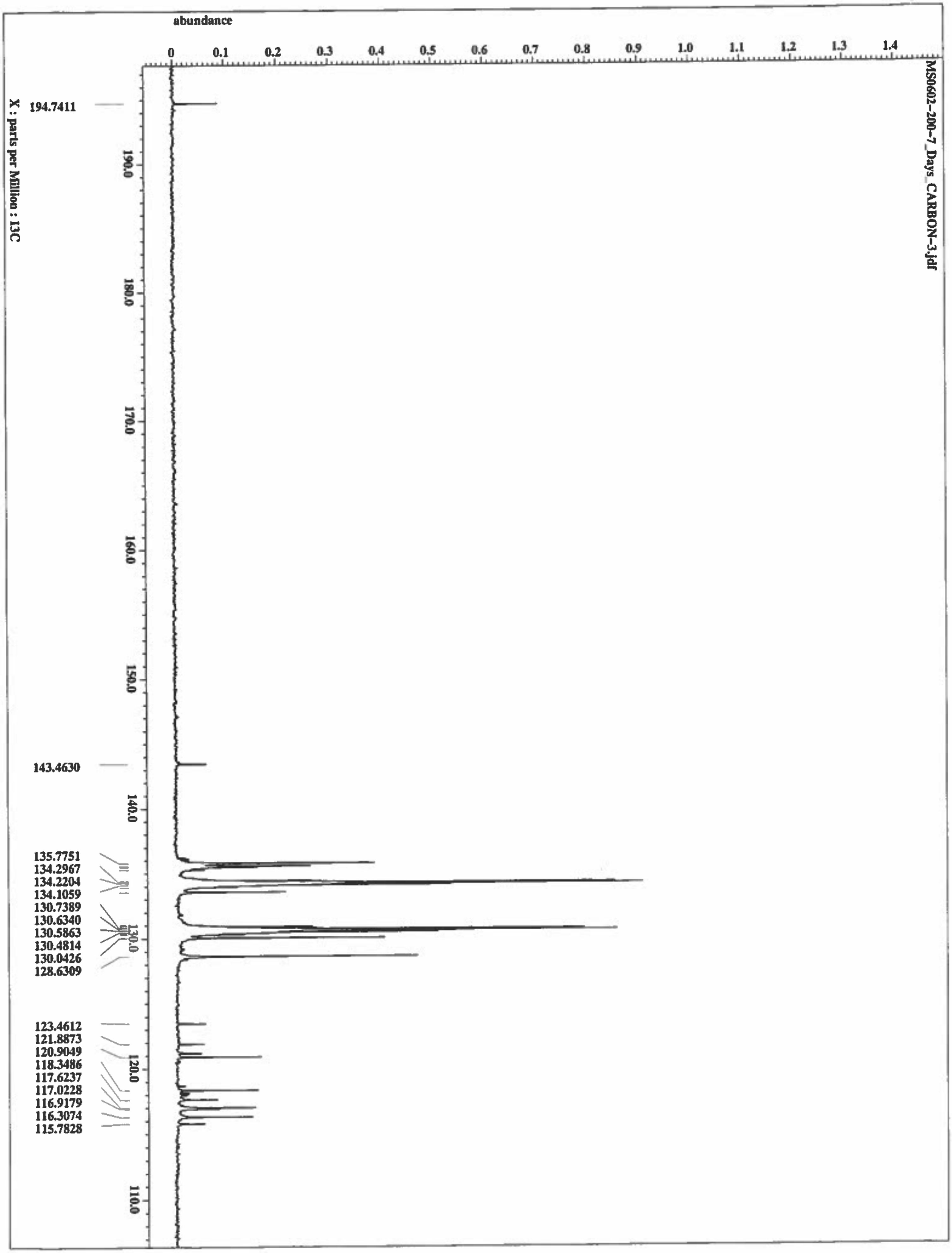

abundance
0
0.1
0.2
0.3
0.4
0.5
0.6
0.7
0.8
0.9
1.0
1.1
1.2
1.3
1.4
MS0602-200-7_Days_CARBON-3.jdf
X : parts per Million : 13C
194.7411
190.0
180.0
170.0
160.0
150.0
143.4630
140.0
135.7751
134.2967
134.2204
134.1059
130.7389
130.6340
130.5863
130.4814
130.0426
128.6309
130.0
123.4612
121.8873
120.9049
118.3486
117.6237
117.0228
116.9179
116.3074
115.7828
120.0
110.0

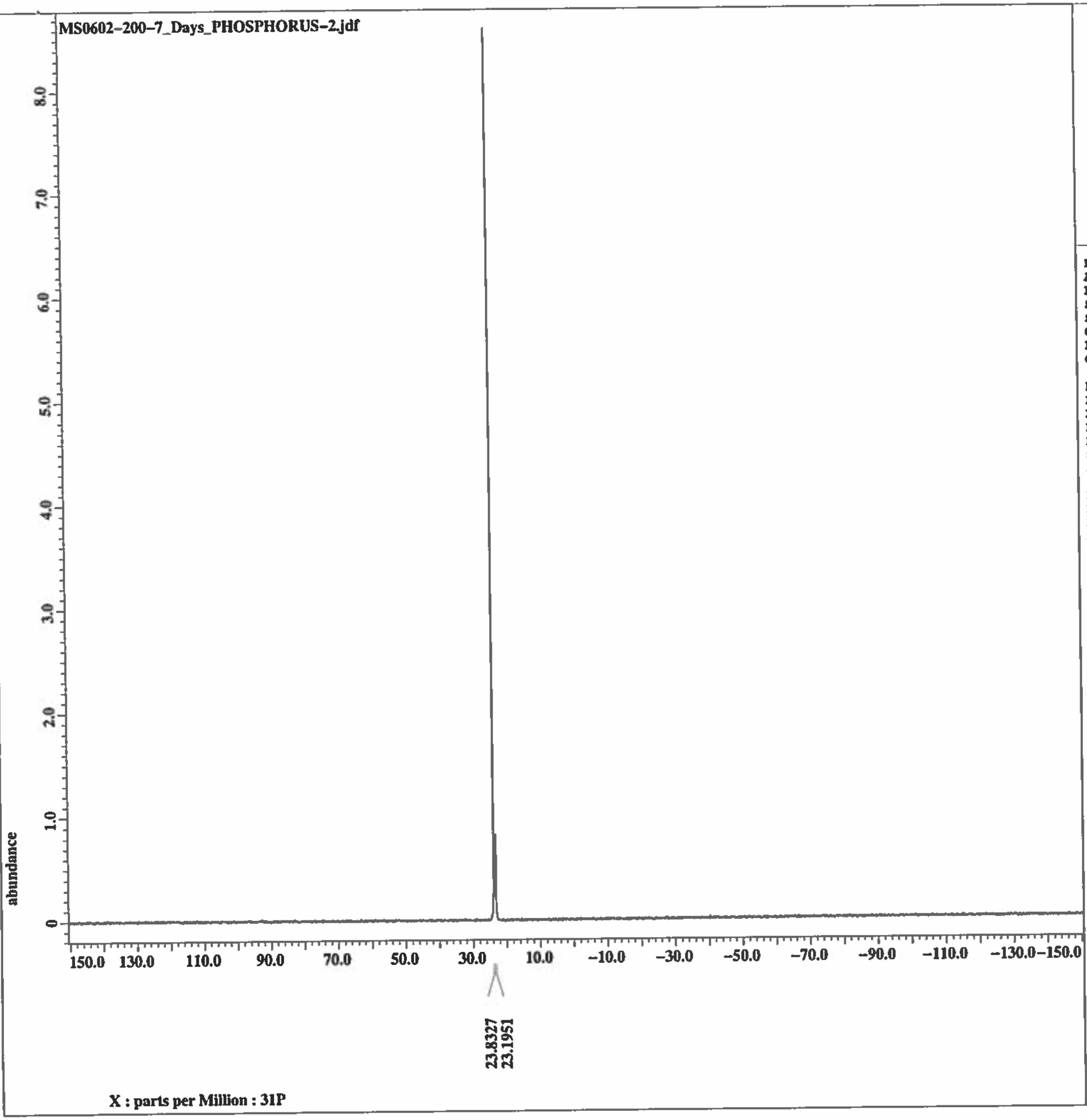

Filename	= MS0602-200-7_Days_PHO
Author	= Jim Davis
Experiment	= single_pulse_dec
Sample_id	= MS0602-200-7_Days
Solvent	= CHLOROFORM-D
Creation_time	= 19-NOV-2018 16:28:59
Revision_time	= 19-NOV-2018 16:04:50
Current_time	= 19-NOV-2018 16:04:50
Data_format	= 1D COMPLEX
Dim_size	= 52428
Dim_title	= 31P
Dim_units	= [ppm]
Dimensions	= X
Site	= ECA 500
Spectrometer	= JNM-ECA500
Field_strength	= 11.7473579[T] (500[MH
X_acq_duration	= 0.85983232[s]
X_domain	= 31P
X_freq	= 202.46831075[MHz]
X_offset	= 0[ppm]
X_points	= 65536
X_prescans	= 4
X_resolution	= 1.16301746[Hz]
X_sweep	= 76.2195122[kHz]
Irr_domain	= 1H
Irr_freq	= 500.15991521[MHz]
Irr_offset	= 5.0[ppm]
Clipped	= FALSE
Mod_return	= 1
Scans	= 40
Total_scans	= 40
X_90_width	= 14.687[us]
X_acq_time	= 0.85983232[s]
X_angle	= 30[deg]
X_atn	= 5[dB]
X_pulse	= 4.89566667[us]
Irr_atn_dec	= 20.7[dB]
Irr_atn_noe	= 20.7[dB]
Irr_noise	= WALTZ
Decoupling	= TRUE
Initial_wait	= 1[s]
Noe	= TRUE
Noe_time	= 2[s]
Recvr_gain	= 58
Relaxation_delay	= 2[s]
Repetition_time	= 2.85983232[s]
Temp_get	= 22[dC]

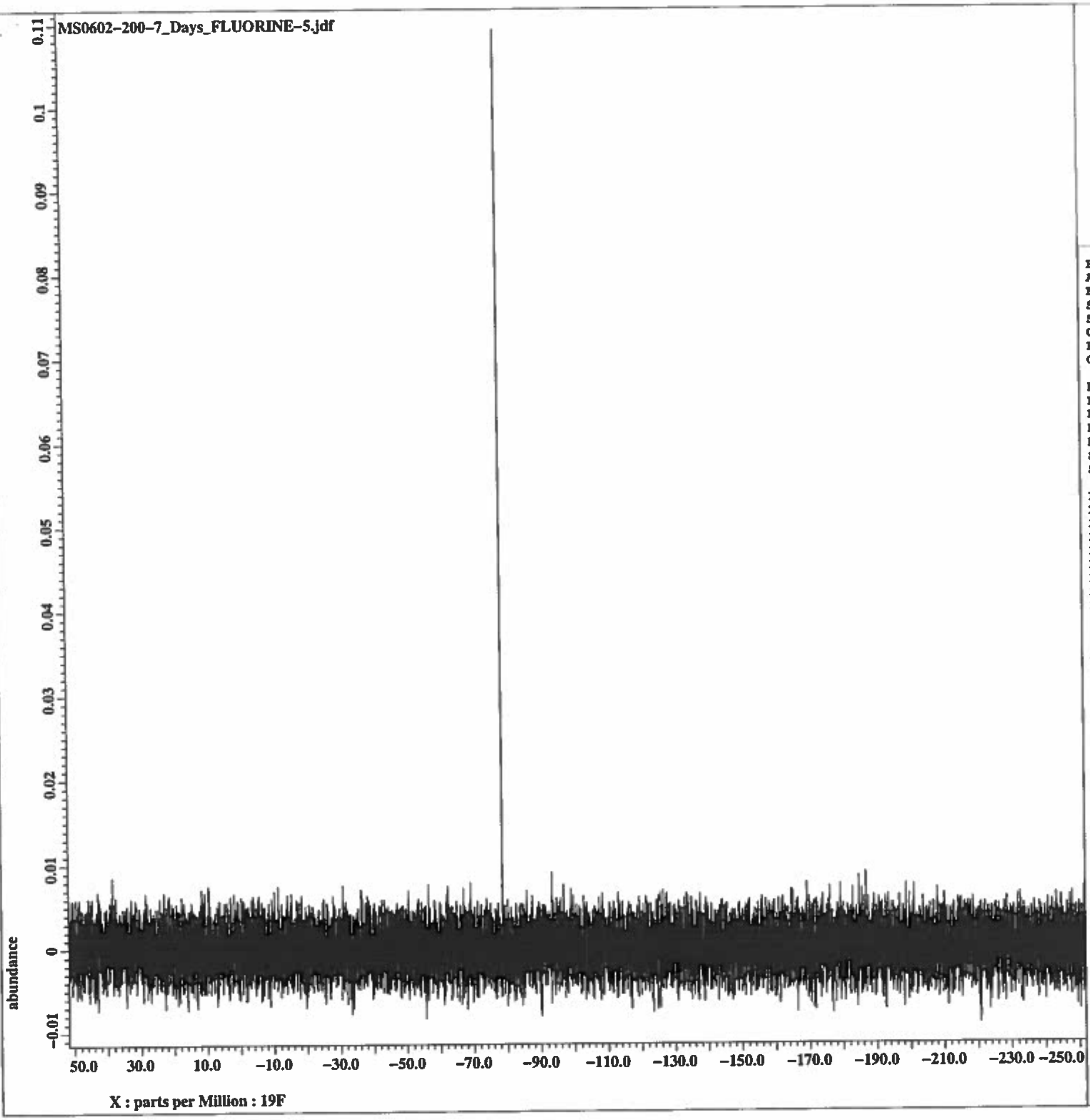

Filename	= MS0602-200-7_Days_FLU
Author	= Jim Davis
Experiment	= single_pulse.ex2
Sample_id	= MS0602-200-7_Days
Solvent	= CHLOROFORM-D
Creation_time	= 20-NOV-2018 09:27:54
Revision_time	= 20-NOV-2018 09:03:42
Current_time	= 20-NOV-2018 09:03:43
Data_format	= 1D COMPLEX
Dim_size	= 104857
Dim_title	= 19F
Dim_units	= [ppm]
Dimensions	= X
Site	= ECA 500
Spectrometer	= JNM-ECA500
Field_strength	= 11.7473579[T] (500[MH
X_acq_duration	= 0.7340032[s]
X_domain	= 19F
X_freq	= 470.62046084[MHz]
X_offset	= -100[ppm]
X_points	= 131072
X_prescans	= 1
X_resolution	= 1.36239188[Hz]
X_sweep	= 178.57142857[kHz]
Irr_domain	= 19F
Irr_freq	= 470.62046084[MHz]
Irr_offset	= 5[ppm]
Tri_domain	= 19F
Tri_freq	= 470.62046084[MHz]
Tri_offset	= 5[ppm]
Clipped	= FALSE
Mod_return	= 1
Scans	= 128
Total_scans	= 128
X_90_width	= 13.1[us]
X_acq_time	= 0.7340032[s]
X_angle	= 45[deg]
X_atn	= 2.5[dB]
X_pulse	= 6.55[us]
Irr_mode	= Off
Tri_mode	= Off
Dante_presat	= FALSE
Initial_wait	= 1[s]
Recvr_gain	= 70
Relaxation_delay	= 4[s]
Repetition_time	= 4.7340032[s]
Temp_get	= 21.9[dC]

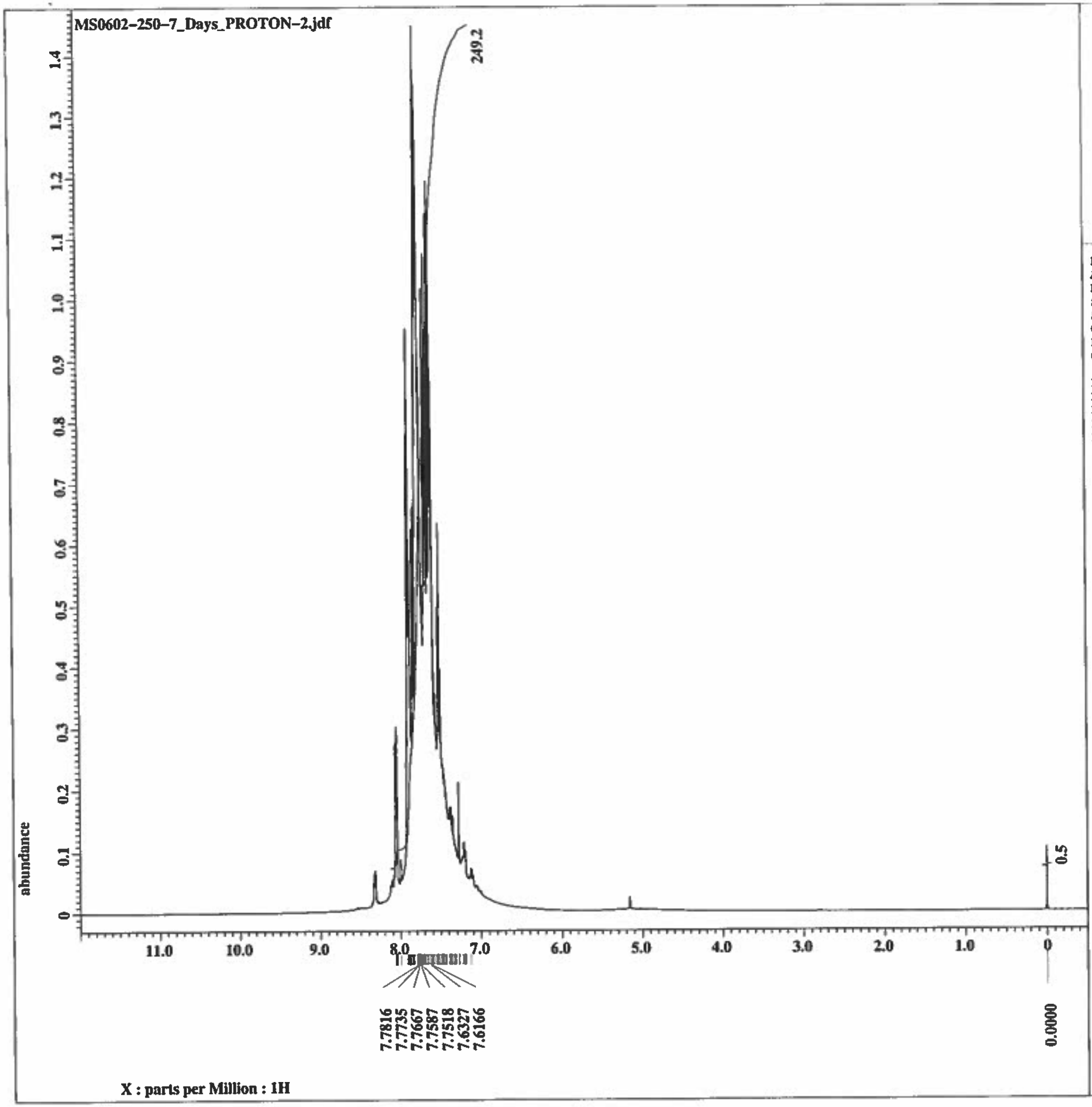

Filename	= MS0602-250-7_Days_PRO
Author	= Jim Davis
Experiment	= single_pulse.ex2
Sample_id	= MS0602-250-7_Days
Solvent	= CHLOROFORM-D
Creation_time	= 19-NOV-2018 16:36:25
Revision_time	= 19-NOV-2018 16:12:14
Current_time	= 19-NOV-2018 16:12:14
Data_format	= 1D COMPLEX
Dim_size	= 13107
Dim_title	= 1H
Dim_units	= [ppm]
Dimensions	= X
Site	= ECA 500
Spectrometer	= JNM-ECA500
Field_strength	= 11.7473579[T] (500[MH
X_acq_duration	= 1.74587904[s]
X_domain	= 1H
X_freq	= 500.15991521[MHz]
X_offset	= 5.0[ppm]
X_points	= 16384
X_prescans	= 1
X_resolution	= 0.57277737[Hz]
X_sweep	= 9.38438438[kHz]
Irr_domain	= 1H
Irr_freq	= 500.15991521[MHz]
Irr_offset	= 5.0[ppm]
Tri_domain	= 1H
Tri_freq	= 500.15991521[MHz]
Tri_offset	= 5.0[ppm]
Clipped	= FALSE
Mod_return	= 1
Scans	= 16
Total_scans	= 16
X_90_width	= 12.4[us]
X_acq_time	= 1.74587904[s]
X_angle	= 45[deg]
X_atn	= 4[dB]
X_pulse	= 6.2[us]
Irr_mode	= Off
Tri_mode	= Off
Dante_presat	= FALSE
Initial_wait	= 1[s]
Recvr_gain	= 26
Relaxation_delay	= 4[s]
Repetition_time	= 5.74587904[s]
Temp_get	= 21.7[dC]

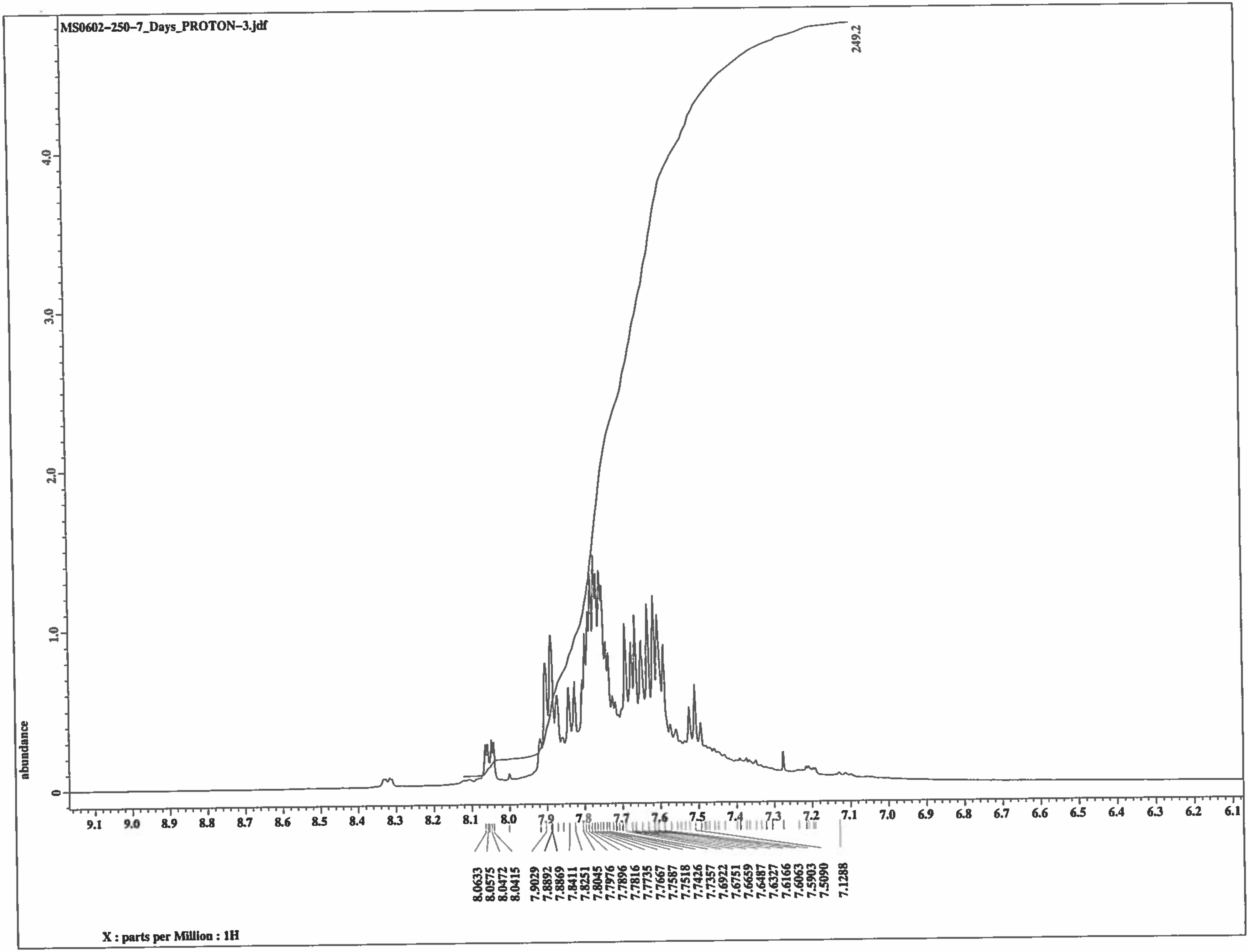
MS0602-250-7_Days_PROTON-3.jdf
249.2
abundance
0
1.0
2.0
3.0
4.0
9.1 9.0 8.9 8.8 8.7 8.6 8.5 8.4 8.3 8.2 8.1 8.0 7.9 7.8 7.7 7.6 7.5 7.4 7.3 7.2 7.1 7.0 6.9 6.8 6.7 6.6 6.5 6.4 6.3 6.2 6.1
8.0633
8.0575
8.0472
8.0415
7.9029
7.8892
7.8869
7.8411
7.8251
7.8045
7.7976
7.7896
7.7816
7.7735
7.7667
7.7587
7.7518
7.7426
7.7357
7.6922
7.6751
7.6659
7.6487
7.6327
7.6166
7.6063
7.5903
7.5090
7.1288
X : parts per Million : 1H

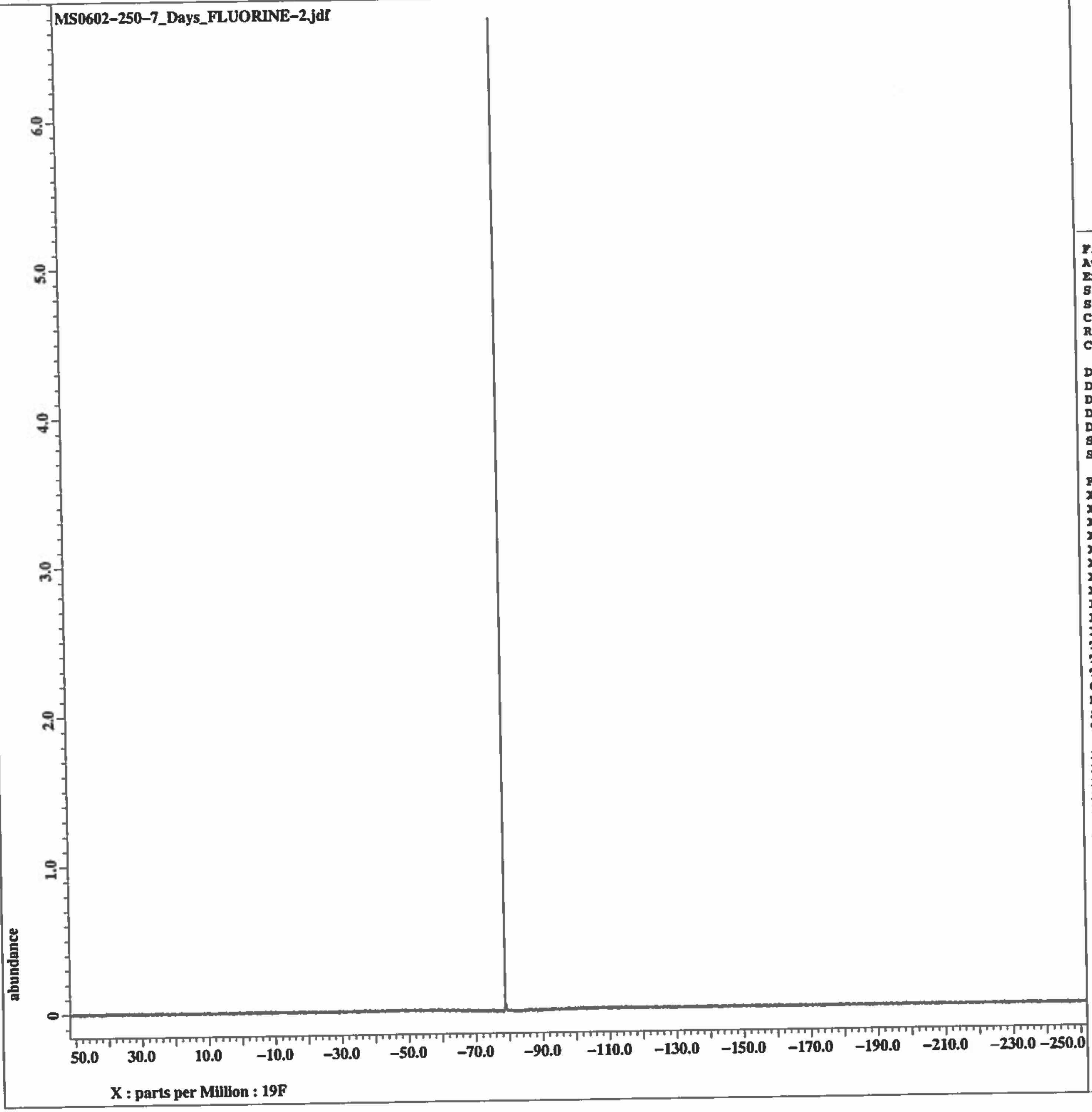

Filename	= MS0602-250-7_Days_FLU
Author	= Jim Davis
Experiment	= single_pulse.ex2
Sample_id	= MS0602-250-7_Days
Solvent	= CHLOROFORM-D
Creation_time	= 19-NOV-2018 16:41:38
Revision_time	= 19-NOV-2018 16:17:31
Current_time	= 19-NOV-2018 16:17:31
Data_format	= 1D COMPLEX
Dim_size	= 104857
Dim_title	= 19F
Dim_units	= [ppm]
Dimensions	= X
Site	= ECA 500
Spectrometer	= JNM-ECA500
Field_strength	= 11.7473579[T] (500[MH
X_acq_duration	= 0.7340032[s]
X_domain	= 19F
X_freq	= 470.62046084[MHz]
X_offset	= -100[ppm]
X_points	= 131072
X_prescans	= 1
X_resolution	= 1.36239188[Hz]
X_sweep	= 178.57142857[kHz]
Irr_domain	= 19F
Irr_freq	= 470.62046084[MHz]
Irr_offset	= 5[ppm]
Tri_domain	= 19F
Tri_freq	= 470.62046084[MHz]
Tri_offset	= 5[ppm]
Clipped	= FALSE
Mod_return	= 1
Scans	= 32
Total_scans	= 32
X_90_width	= 13.1[us]
X_acq_time	= 0.7340032[s]
X_angle	= 45[deg]
X_atn	= 2.5[dB]
X_pulse	= 6.55[us]
Irr_mode	= Off
Tri_mode	= Off
Dante_presat	= FALSE
Initial_wait	= 1[s]
Recvr_gain	= 70
Relaxation_delay	= 4[s]
Repetition_time	= 4.7340032[s]
Temp_get	= 21.7[dC]

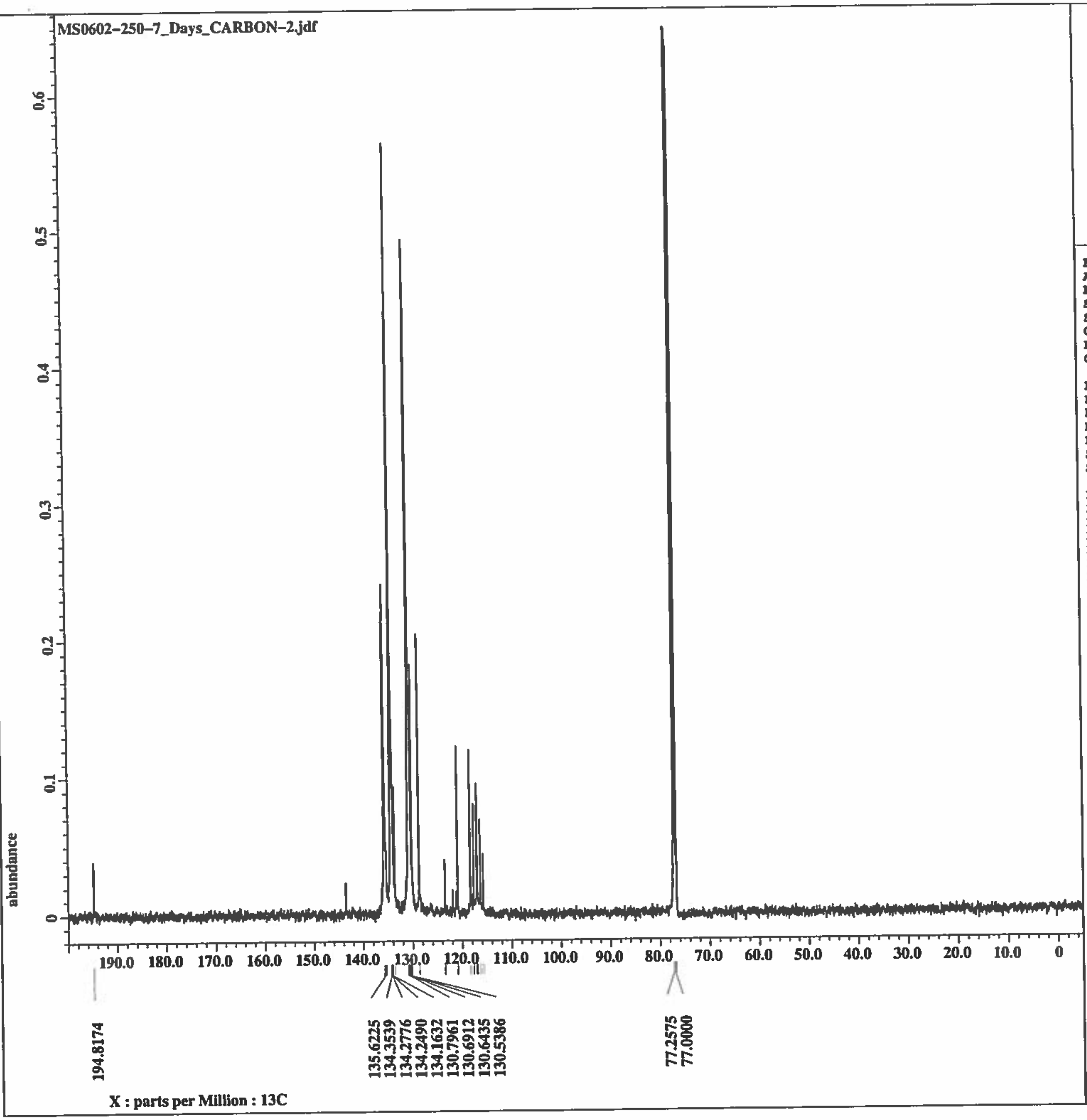

Filename	= MS0602-250-7_Days_CAR
Author	= Jim Davis
Experiment	= single_pulse_dec
Sample_id	= MS0602-250-7_Days
Solvent	= CHLOROFORM-D
Creation_time	= 20-NOV-2018 01:16:33
Revision_time	= 20-NOV-2018 00:52:22
Current_time	= 20-NOV-2018 00:52:22
Data_format	= 1D COMPLEX
Dim_size	= 26214
Dim_title	= 13C
Dim_units	= [ppm]
Dimensions	= X
Site	= ECA 500
Spectrometer	= JNM-ECA500
Field_strength	= 11.7473579[T] (500[MH
X_acq_duration	= 0.83361792[s]
X_domain	= 13C
X_freq	= 125.76529768[MHz]
X_offset	= 100[ppm]
X_points	= 32768
X_prescans	= 4
X_resolution	= 1.19959034[Hz]
X_sweep	= 39.3081761[kHz]
Irr_domain	= 1H
Irr_freq	= 500.15991521[MHz]
Irr_offset	= 5.0[ppm]
Clipped	= FALSE
Mod_return	= 1
Scans	= 1024
Total_scans	= 1024
X_90_width	= 13.2[us]
X_acq_time	= 0.83361792[s]
X_angle	= 30[deg]
X_atn	= 6[dB]
X_pulse	= 4.4[us]
Irr_atn_dec	= 20.7[dB]
Irr_atn_noe	= 20.7[dB]
Irr_noise	= WALTZ
Decoupling	= TRUE
Initial_wait	= 1[s]
Noe	= TRUE
Noe_time	= 2[s]
Recvr_gain	= 60
Relaxation_delay	= 2[s]
Repetition_time	= 2.83361792[s]
Temp_get	= 22.6[dC]

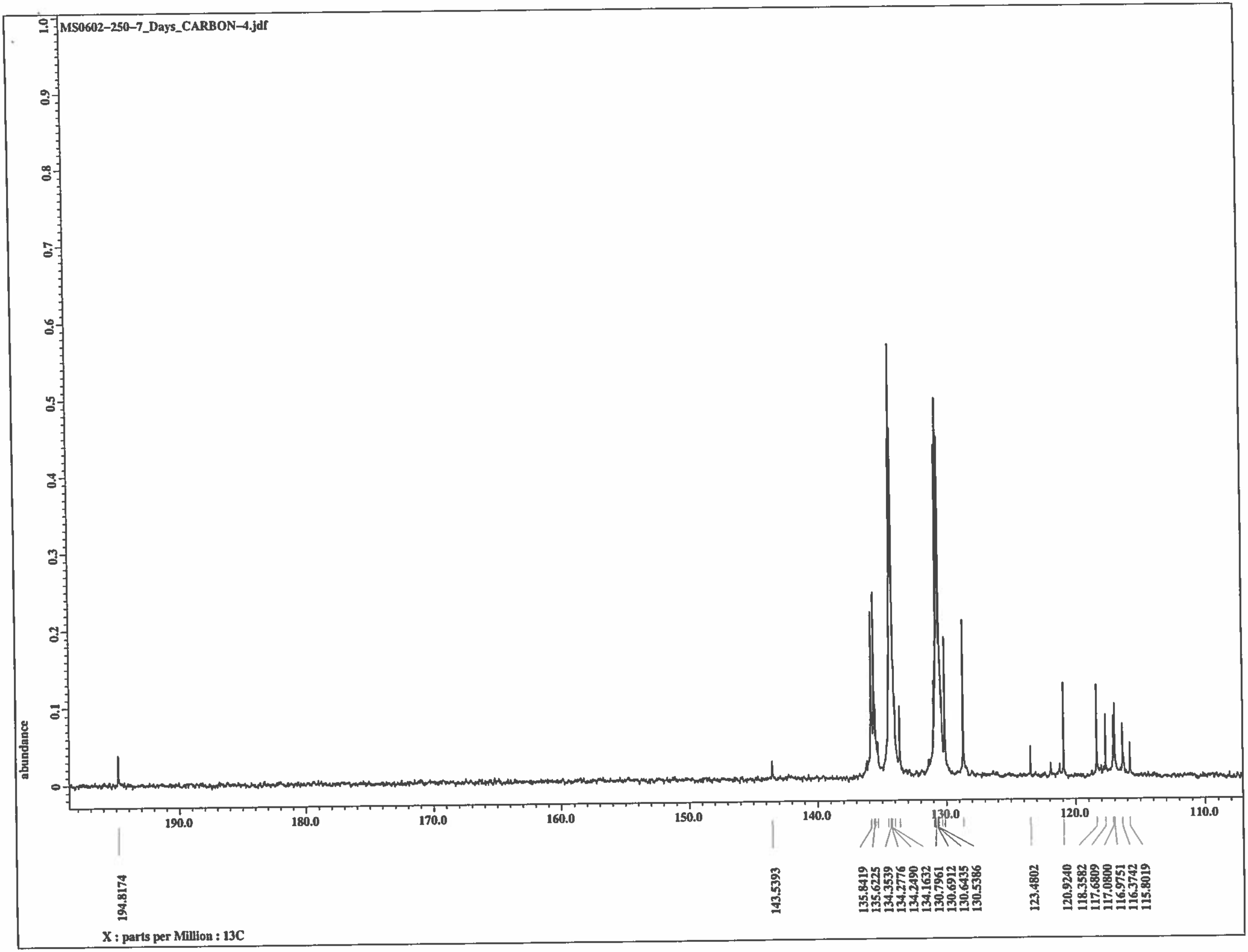
MS0602-250-7_Days_CARBON-4.jdf
abundance
0
0.1
0.2
0.3
0.4
0.5
0.6
0.7
0.8
0.9
1.0
190.0
180.0
170.0
160.0
150.0
140.0
130.0
120.0
110.0
194.8174
143.5393
135.8419
135.6225
134.3539
134.2776
134.2490
134.1632
130.7961
130.6912
130.6435
130.5386
123.4802
120.9240
118.3582
117.6809
117.0800
116.9751
116.3742
115.8019
X : parts per Million : 13C

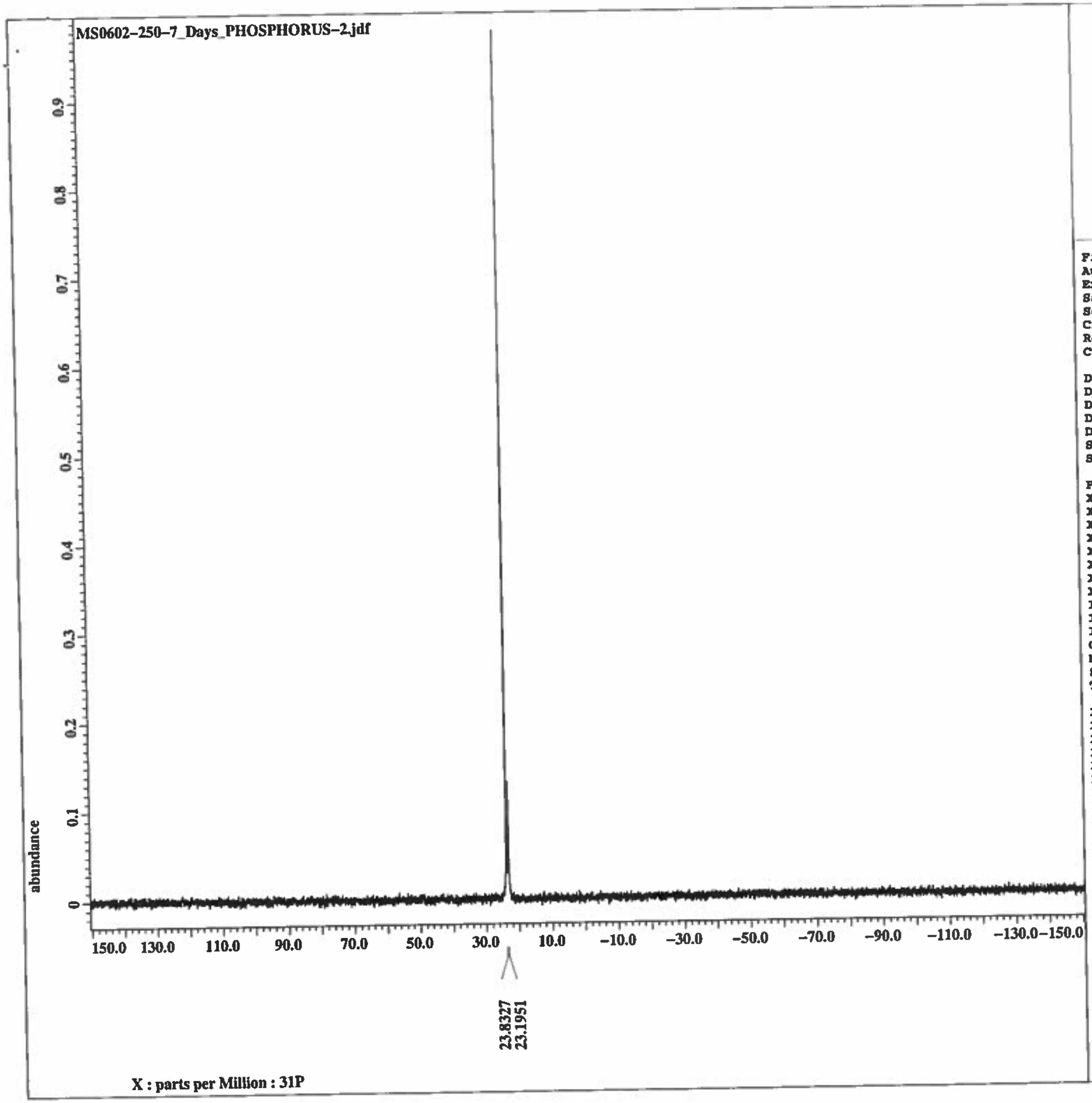

Filename	= MS0602-250-7_Days_PHO
Author	= Jim Davis
Experiment	= single_pulse_dec
Sample_id	= MS0602-250-7_Days
Solvent	= CHLOROFORM-D
Creation_time	= 19-NOV-2018 16:46:11
Revision_time	= 19-NOV-2018 16:22:02
Current_time	= 19-NOV-2018 16:22:02
Data_format	= 1D COMPLEX
Dim_size	= 52428
Dim_title	= 31P
Dim_units	= [ppm]
Dimensions	= X
Site	= ECA 500
Spectrometer	= JNM-ECA500
Field_strength	= 11.7473579[T] (500[MH
X_acq_duration	= 0.85983232[s]
X_domain	= 31P
X_freq	= 202.46831075[MHz]
X_offset	= 0[ppm]
X_points	= 65536
X_prescans	= 4
X_resolution	= 1.16301746[Hz]
X_sweep	= 76.2195122[kHz]
Irr_domain	= 1H
Irr_freq	= 500.15991521[MHz]
Irr_offset	= 5.0[ppm]
Clipped	= TRUE
Mod_return	= 1
Scans	= 40
Total_scans	= 40
X_90_width	= 14.687[us]
X_acq_time	= 0.85983232[s]
X_angle	= 30[deg]
X_atn	= 5[dB]
X_pulse	= 4.89566667[us]
Irr_atn_dec	= 20.7[dB]
Irr_atn_noe	= 20.7[dB]
Irr_noise	= WALTZ
Decoupling	= TRUE
Initial_wait	= 1[s]
Noe	= TRUE
Noe_time	= 2[s]
Recvr_gain	= 50
Relaxation_delay	= 2[s]
Repetition_time	= 2.85983232[s]
Temp_get	= 22.1[dC]

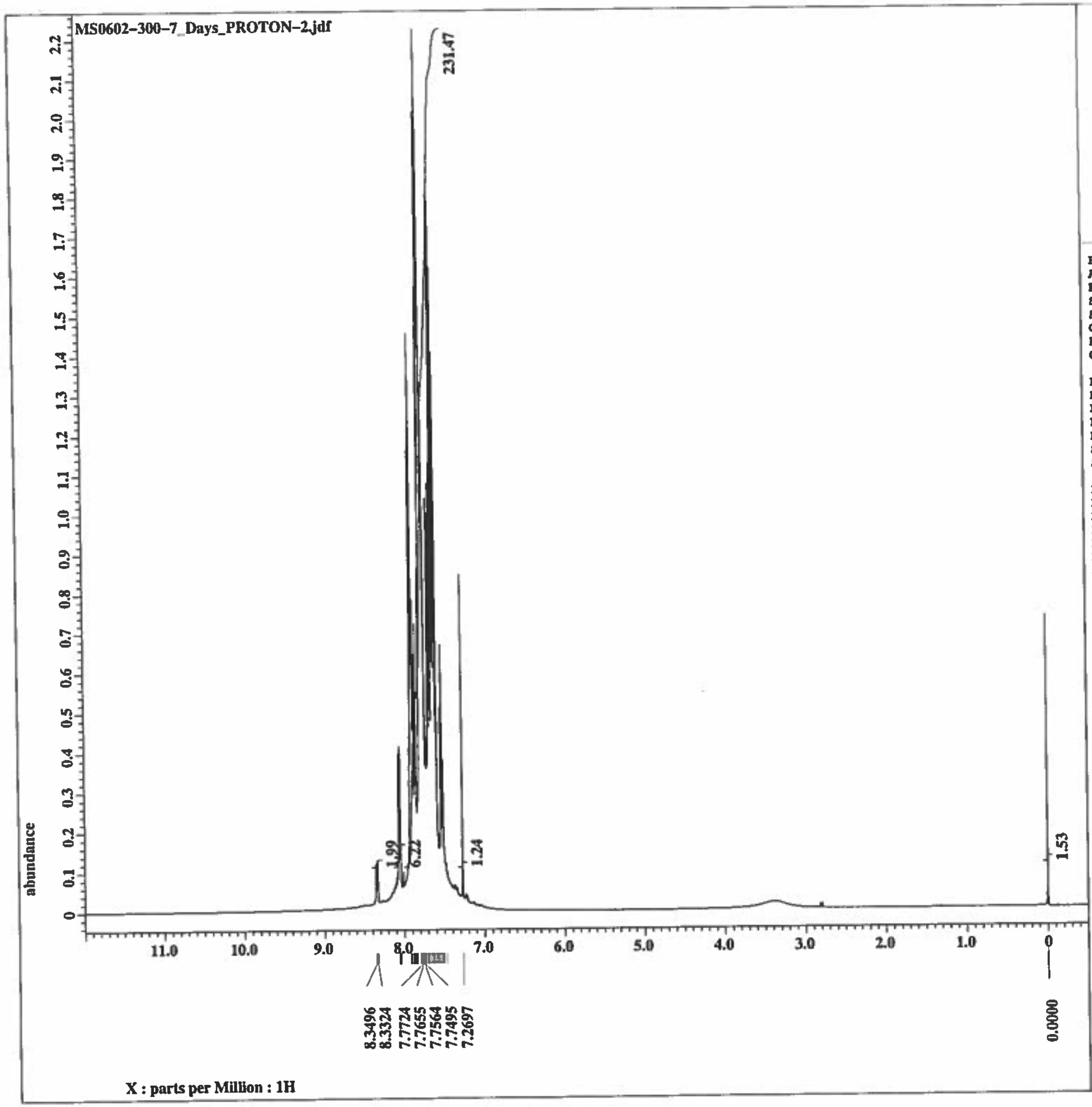

Filename	= MS0602-300-7_Days_PRO
Author	= Jim Davis
Experiment	= single_pulse.ex2
Sample_id	= MS0602-300-7_Days
Solvent	= CHLOROFORM-D
Creation_time	= 19-NOV-2018 16:53:34
Revision_time	= 19-NOV-2018 16:29:25
Current_time	= 19-NOV-2018 16:29:25
Data_format	= 1D COMPLEX
Dim_size	= 13107
Dim_title	= 1H
Dim_units	= [ppm]
Dimensions	= X
Site	= ECA 500
Spectrometer	= JNM-ECA500
Field_strength	= 11.7473579[T] (500[MH
X_acq_duration	= 1.74587904[s]
X_domain	= 1H
X_freq	= 500.15991521[MHz]
X_offset	= 5.0[ppm]
X_points	= 16384
X_prescans	= 1
X_resolution	= 0.57277737[Hz]
X_sweep	= 9.38438438[kHz]
Irr_domain	= 1H
Irr_freq	= 500.15991521[MHz]
Irr_offset	= 5.0[ppm]
Tri_domain	= 1H
Tri_freq	= 500.15991521[MHz]
Tri_offset	= 5.0[ppm]
Clipped	= FALSE
Mod_return	= 1
Scans	= 16
Total_scans	= 16
X_90_width	= 12.4[us]
X_acq_time	= 1.74587904[s]
X_angle	= 45[deg]
X_atn	= 4[dB]
X_pulse	= 6.2[us]
Irr_mode	= Off
Tri_mode	= Off
Dante_presat	= FALSE
Initial_wait	= 1[s]
Recvr_gain	= 36
Relaxation_delay	= 4[s]
Repetition_time	= 5.74587904[s]
Temp_get	= 21.8[dC]

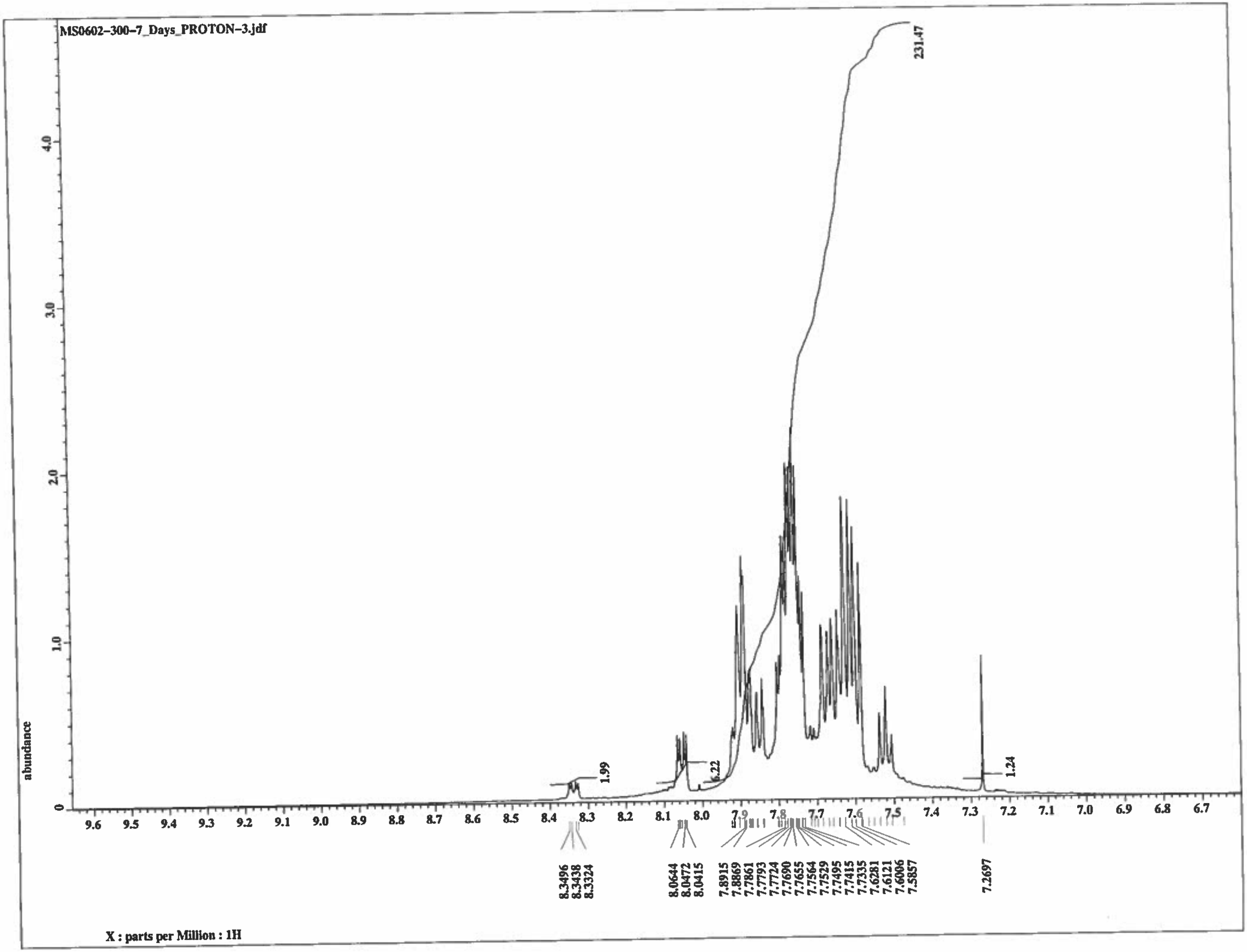
MS0602-300-7_Days_PROTON-3.jdf
abundance
0
1.0
2.0
3.0
4.0
1.99
6.22
231.47
1.24
9.6 9.5 9.4 9.3 9.2 9.1 9.0 8.9 8.8 8.7 8.6 8.5 8.4 8.3 8.2 8.1 8.0 7.9 7.8 7.7 7.6 7.5 7.4 7.3 7.2 7.1 7.0 6.9 6.8 6.7
8.3496
8.3438
8.3324
8.0644
8.0472
8.0415
7.8915
7.8869
7.7861
7.7793
7.7724
7.7690
7.7655
7.7564
7.7529
7.7495
7.7415
7.7335
7.6281
7.6121
7.6006
7.5857
7.2697
X : parts per Million : 1H

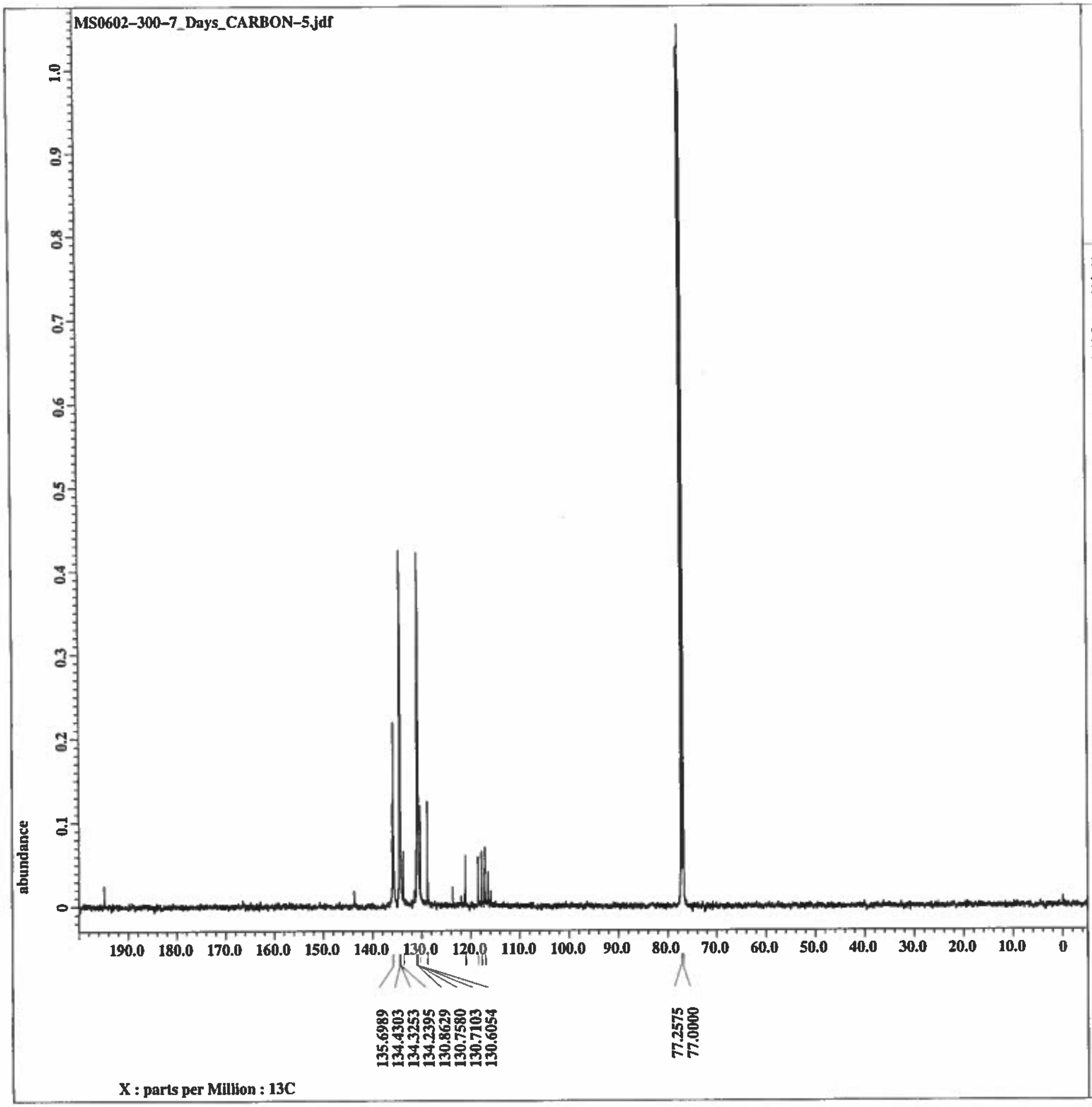

Filename	= MS0602-300-7_Days_CAR
Author	= Jim Davis
Experiment	= single_pulse_dec
Sample_id	= MS0602-300-7_Days
Solvent	= CHLOROFORM-D
Creation_time	= 20-NOV-2018 02:11:07
Revision_time	= 20-NOV-2018 01:46:55
Current_time	= 20-NOV-2018 01:46:55
Data_format	= 1D COMPLEX
Dim_size	= 26214
Dim_title	= 13C
Dim_units	= [ppm]
Dimensions	= X
Site	= ECA 500
Spectrometer	= JNM-ECA500
Field_strength	= 11.7473579[T] (500[MH
X_acq_duration	= 0.83361792[s]
X_domain	= 13C
X_freq	= 125.76529768[MHz]
X_offset	= 100[ppm]
X_points	= 32768
X_prescans	= 4
X_resolution	= 1.19959034[Hz]
X_sweep	= 39.3081761[kHz]
Irr_domain	= 1H
Irr_freq	= 500.15991521[MHz]
Irr_offset	= 5.0[ppm]
Clipped	= FALSE
Mod_return	= 1
Scans	= 1024
Total_scans	= 1024
X_90_width	= 13.2[us]
X_acq_time	= 0.83361792[s]
X_angle	= 30[deg]
X_atn	= 6[dB]
X_pulse	= 4.4[us]
Irr_atn_dec	= 20.7[dB]
Irr_atn_noe	= 20.7[dB]
Irr_noise	= WALTZ
Decoupling	= TRUE
Initial_wait	= 1[s]
Noe	= TRUE
Noe_time	= 2[s]
Recvr_gain	= 60
Relaxation_delay	= 2[s]
Repetition_time	= 2.83361792[s]
Temp_get	= 22.6[dC]

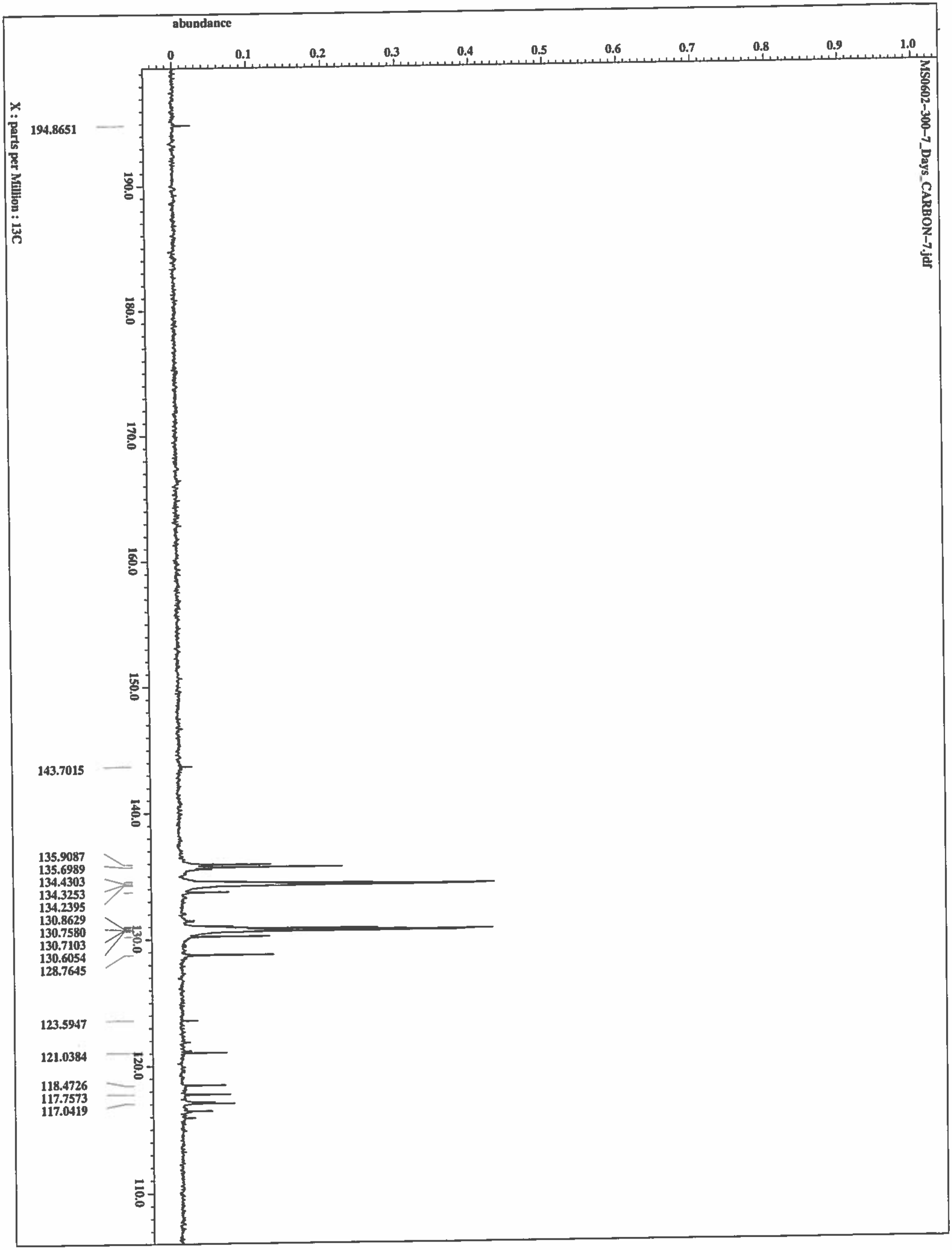
abundance
0
0.1
0.2
0.3
0.4
0.5
0.6
0.7
0.8
0.9
1.0
MS0602-300-7_Days_CARBON-7.jdf
X : parts per Million : 13C
194.8651
190.0
180.0
170.0
160.0
150.0
143.7015
140.0
135.9087
135.6989
134.4303
134.3253
134.2395
130.8629
130.7580
130.7103
130.6054
128.7645
130.0
123.5947
121.0384
120.0
118.4726
117.7573
117.0419
110.0

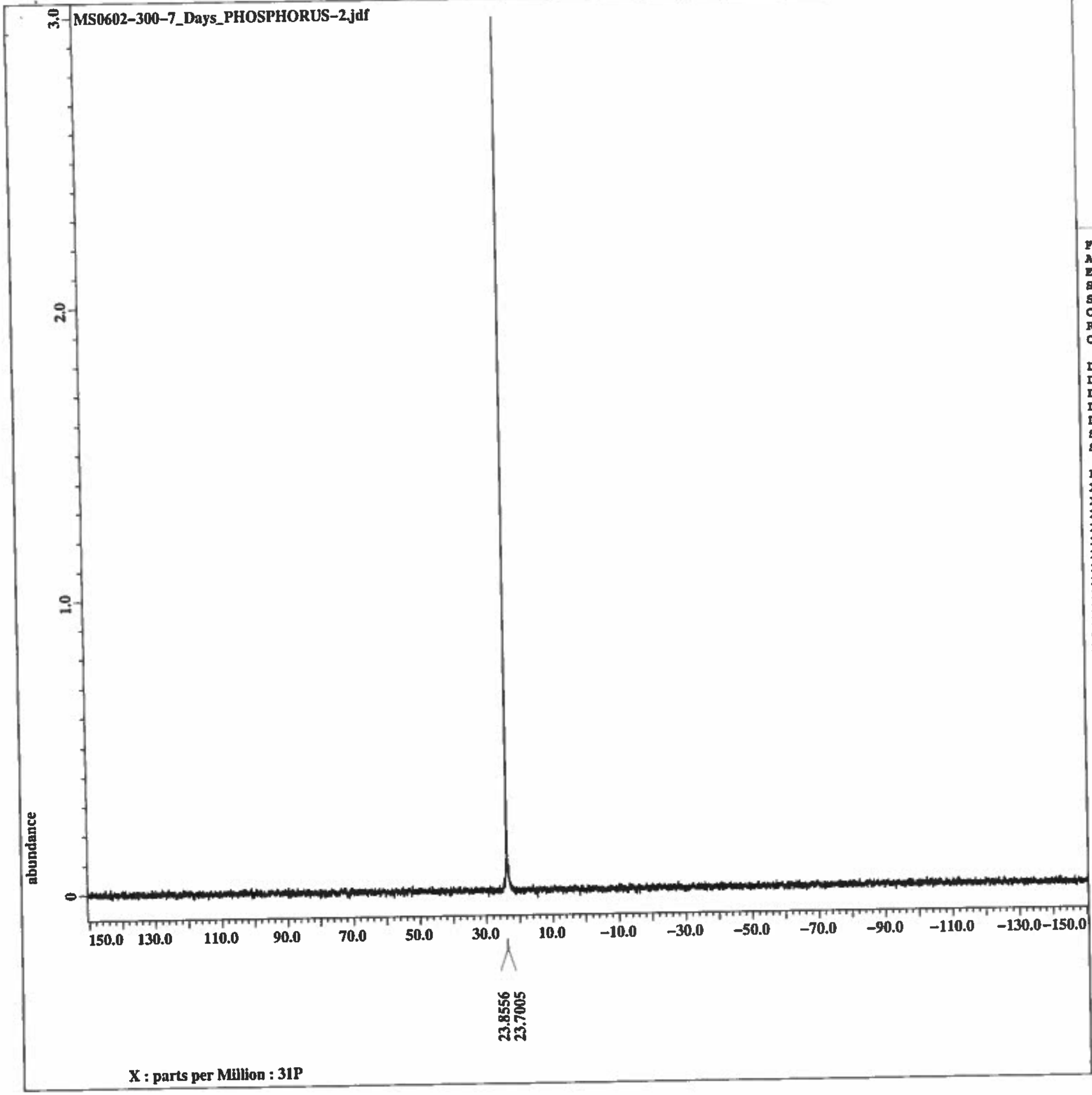


```
Filename          = MS0602-300-7_Days_PHO
Author            = Jim Davis
Experiment        = single_pulse_dec
Sample_id         = MS0602-300-7_Days
Solvent           = CHLOROFORM-D
Creation_time     = 19-NOV-2018 17:03:08
Revision_time     = 19-NOV-2018 16:38:59
Current_time      = 19-NOV-2018 16:38:59

Data_format       = 1D COMPLEX
Dim_size          = 52428
Dim_title         = 31P
Dim_units         = [ppm]
Dimensions        = X
Site              = ECA 500
Spectrometer      = JNM-ECA500

Field_strength    = 11.7473579[T] (500[MH
X_acq_duration    = 0.85983232[s]
X_domain          = 31P
X_freq            = 202.46831075[MHz]
X_offset          = 0[ppm]
X_points          = 65536
X_prescans        = 4
X_resolution      = 1.16301746[Hz]
X_sweep           = 76.2195122[kHz]
Irr_domain        = 1H
Irr_freq          = 500.15991521[MHz]
Irr_offset        = 5.0[ppm]
Clipped           = FALSE
Mod_return        = 1
Scans             = 40
Total_scans       = 40

X_90_width        = 14.687[us]
X_acq_time        = 0.85983232[s]
X_angle           = 30[deg]
X_atn             = 5[dB]
X_pulse           = 4.89566667[us]
Irr_atn_dec       = 20.7[dB]
Irr_atn_noe       = 20.7[dB]
Irr_noise         = WALTZ
Decoupling        = TRUE
Initial_wait      = 1[s]
Noe               = TRUE
Noe_time          = 2[s]
Recvr_gain        = 58
Relaxation_delay  = 2[s]
Repetition_time   = 2.85983232[s]
Temp_get          = 22.2[dC]
```

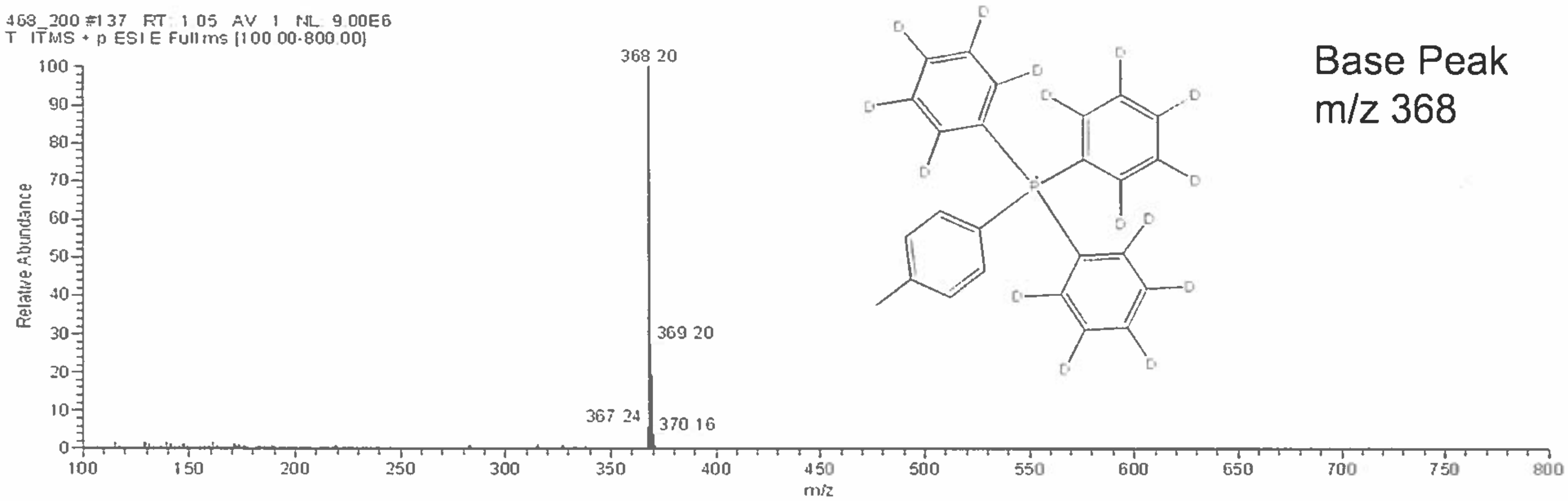
468_200 #137 RT 1 05 AV 1 NL 9.00E6
T ITMS + p ESI E Full ms [100 00-800.00]
368 20
369 20
367 24
370 16
Relative Abundance
m/z
Base Peak
m/z 368

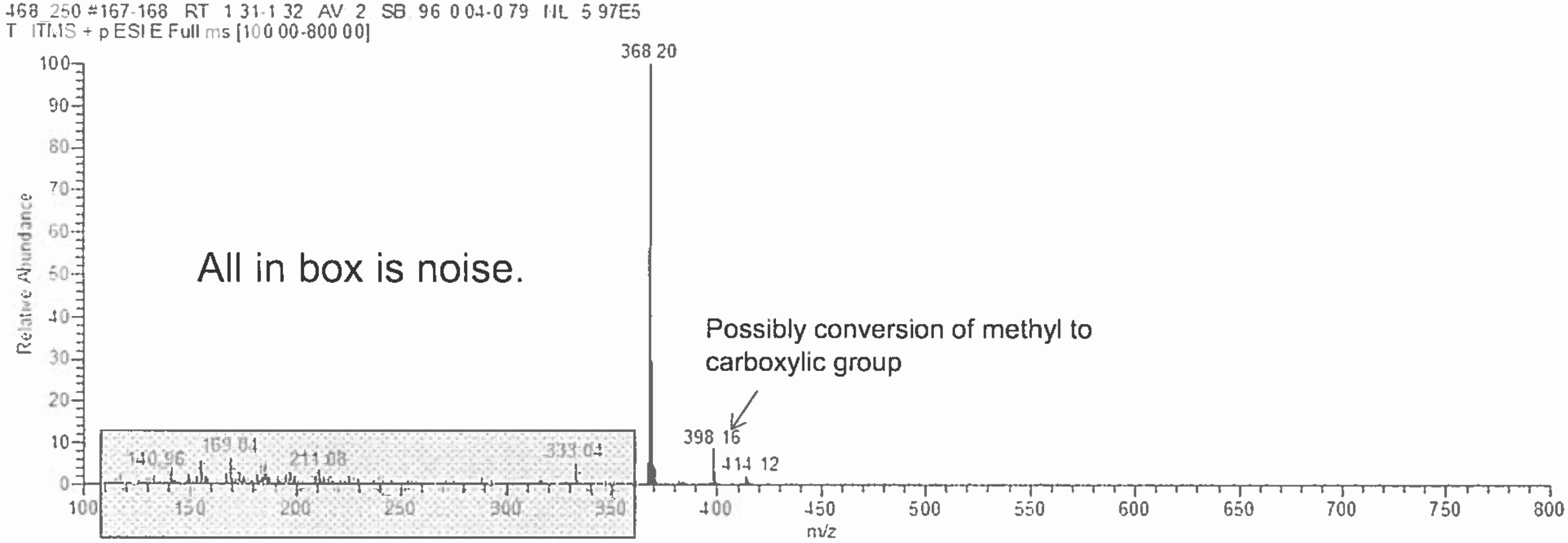
468 250 #167-168 RT 1 31-1 32 AV 2 SB 96 0 04-0 79 NL 5 97E5
T ITMS + p ESI E Full ms [100 00-800 00]
368 20
398 16
414 12
140.96
169.04
211.08
333.04
Relative Abundance
m/z
All in box is noise.
Possibly conversion of methyl to carboxylic group

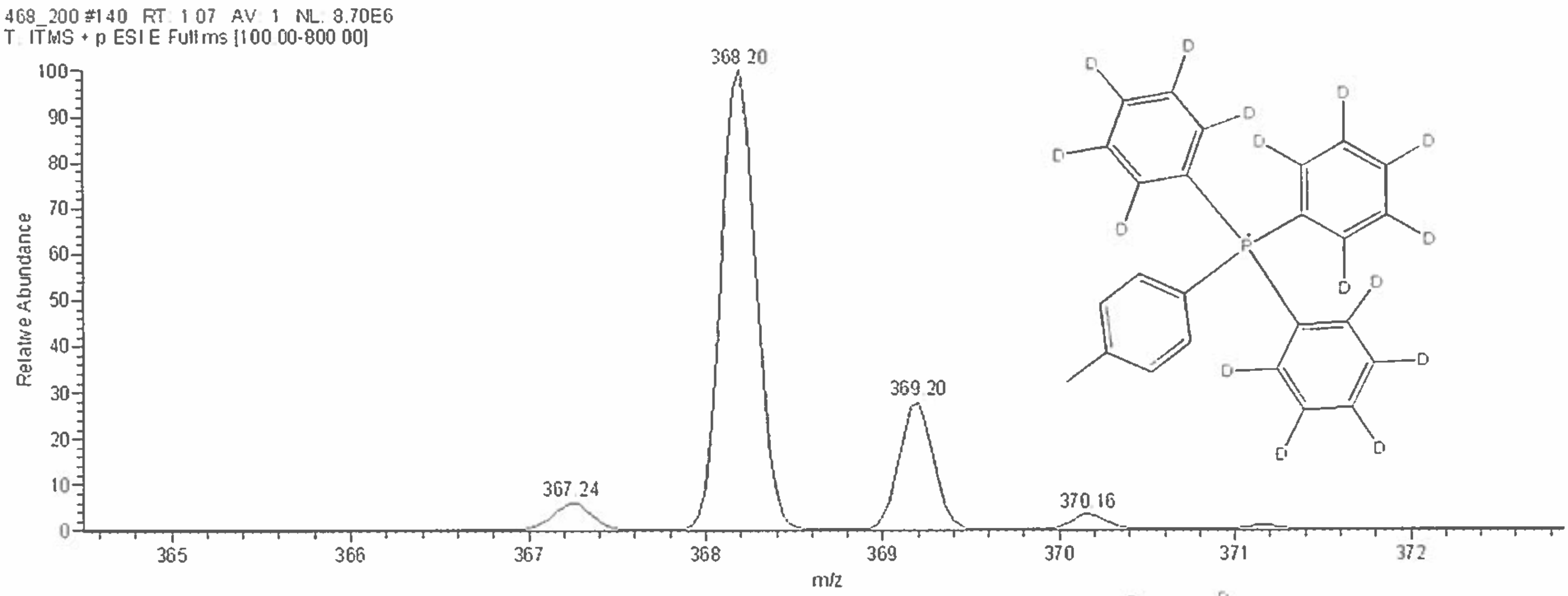
468_200 #140 RT: 1.07 AV: 1 NL: 8.70E6
T: ITMS + p ESI E Full ms [100.00-800.00]
Relative Abundance
m/z
368.20
369.20
367.24
370.16

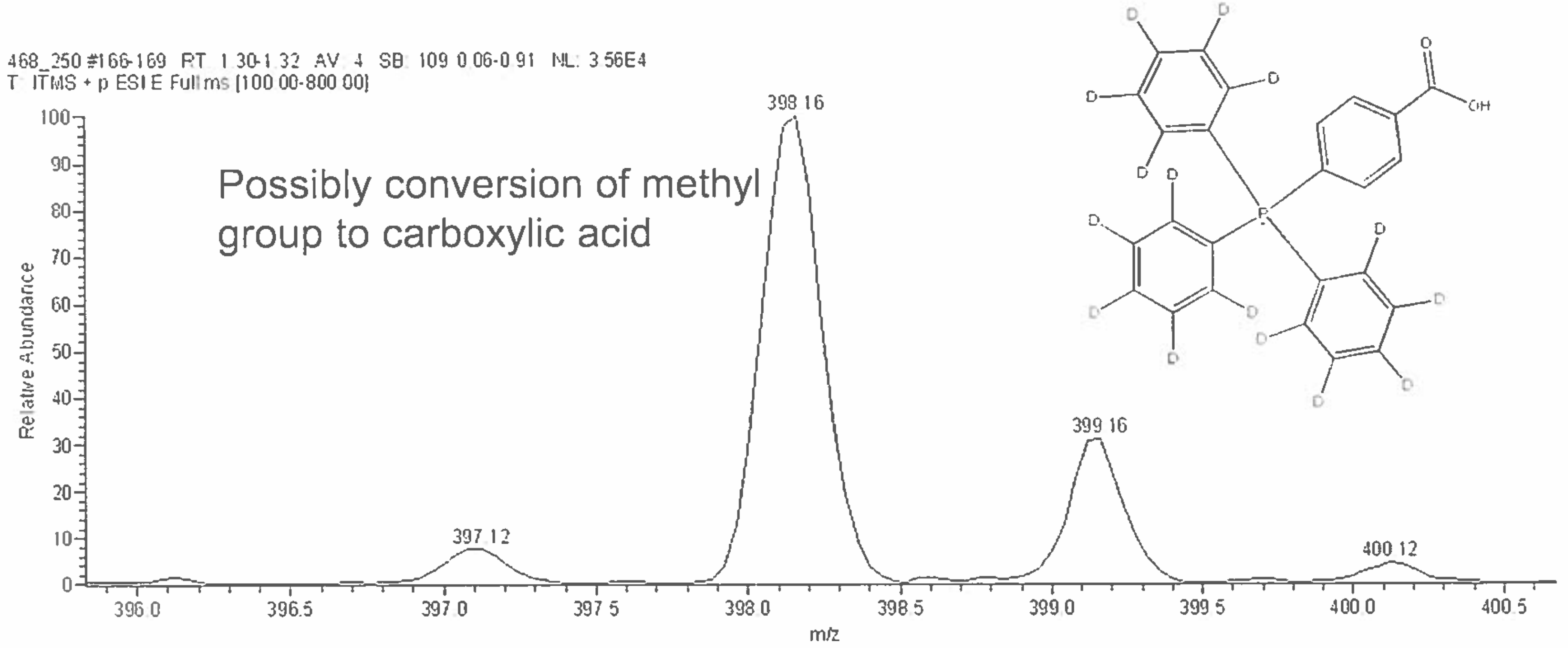
468_250 #166-169 RT: 1.30-1.32 AV: 4 SB: 109 0.06-0.91 NL: 3.56E4
T: ITMS + p ESI E Full ms [100.00-800.00]
Relative Abundance
m/z
Possibly conversion of methyl group to carboxylic acid
398.16
399.16
397.12
400.12

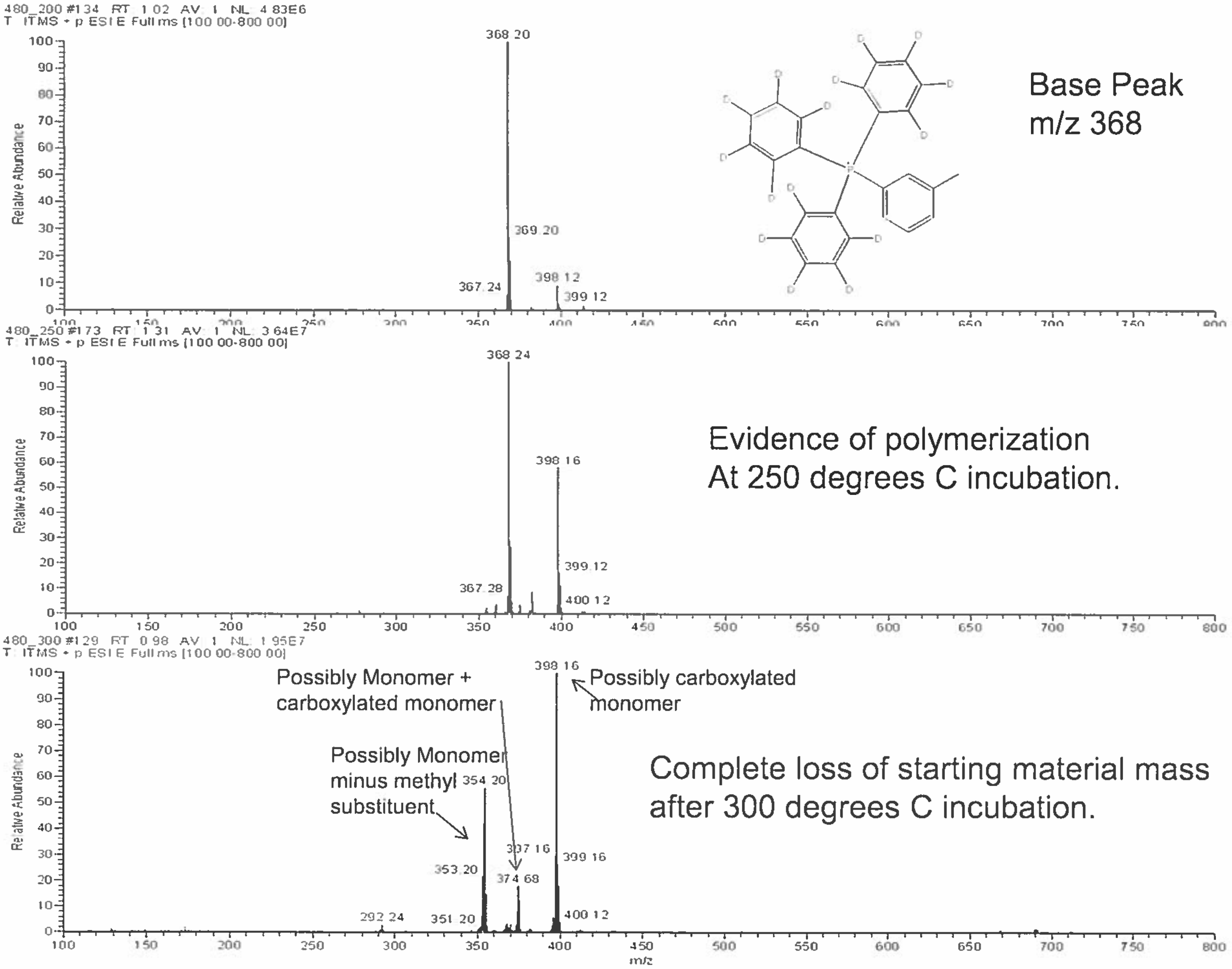
480_200 #134 RT 1.02 AV 1 NL 4.83E6
T ITMS + p ESI E Full ms [100.00-800.00]
Relative Abundance
368.20
369.20
367.24
398.12
399.12
Base Peak
m/z 368
480_250 #173 RT 1.31 AV 1 NL 3.64E7
T ITMS + p ESI E Full ms [100.00-800.00]
368.24
398.16
399.12
367.28
400.12
Evidence of polymerization
At 250 degrees C incubation.
480_300 #129 RT 0.98 AV 1 NL 1.95E7
T ITMS + p ESI E Full ms [100.00-800.00]
398.16
Possibly Monomer +
carboxylated monomer
Possibly carboxylated
monomer
Possibly Monomer
minus methyl
substituent
354.20
353.20
374.68
399.16
292.24
351.20
400.12
Complete loss of starting material mass
after 300 degrees C incubation.
m/z

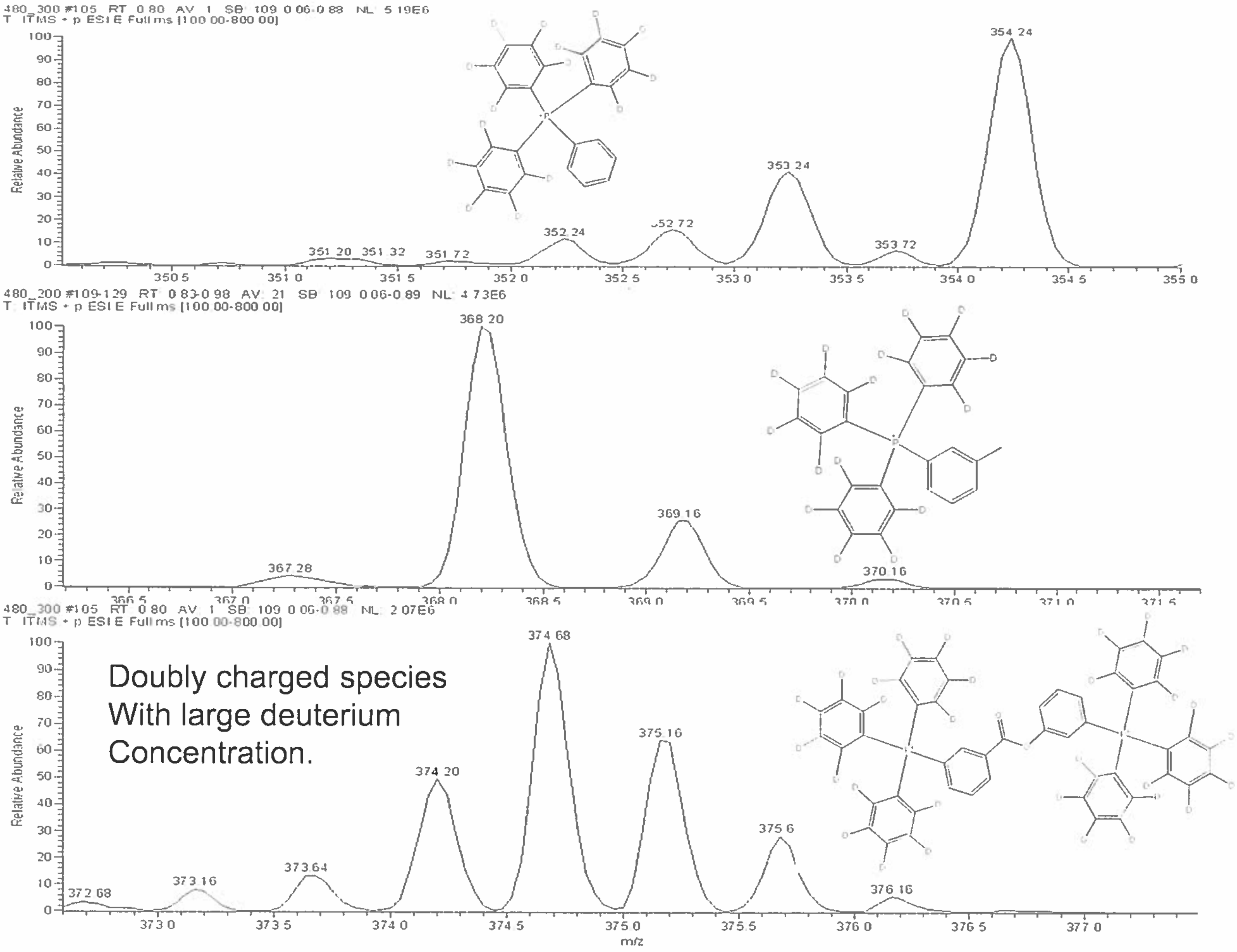
480_300 #105 RT 0 80 AV 1 SB 109 0 06-0 88 NL 5 19E6
T ITMS + p ESI E Full ms [100 00-800 00]
Relative Abundance
351 20
351 32
351 72
352 24
353 24
353 72
354 24
480_200 #109-129 RT 0 83-0 98 AV 21 SB 109 0 06-0 89 NL 4 73E6
T ITMS + p ESI E Full ms [100 00-800 00]
Relative Abundance
367 28
368 20
369 16
370 16
480_300 #105 RT 0 80 AV 1 SB 109 0 06-0 88 NL 2 07E6
T ITMS + p ESI E Full ms [100 00-800 00]
Relative Abundance
Doubly charged species
With large deuterium
Concentration.
372 68
373 16
373 64
374 20
374 68
375 16
375 6
376 16
m/z

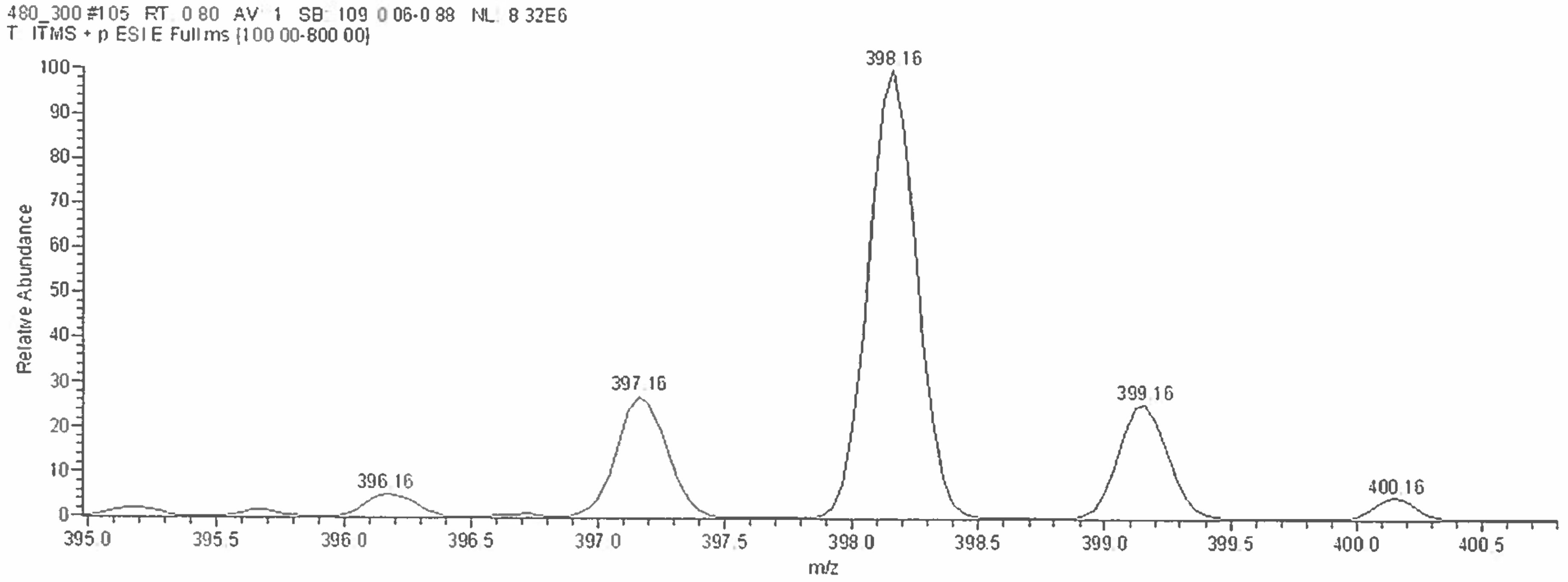

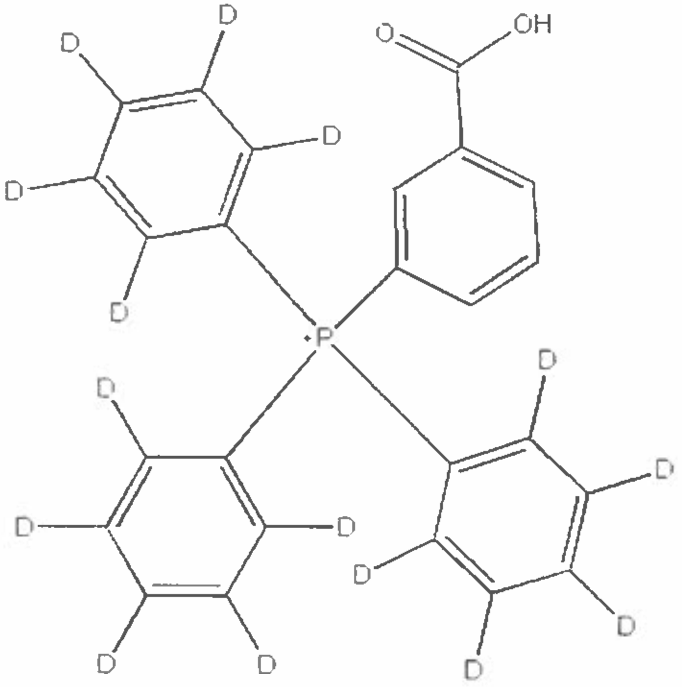

Conversion of methyl group to carboxylic acid

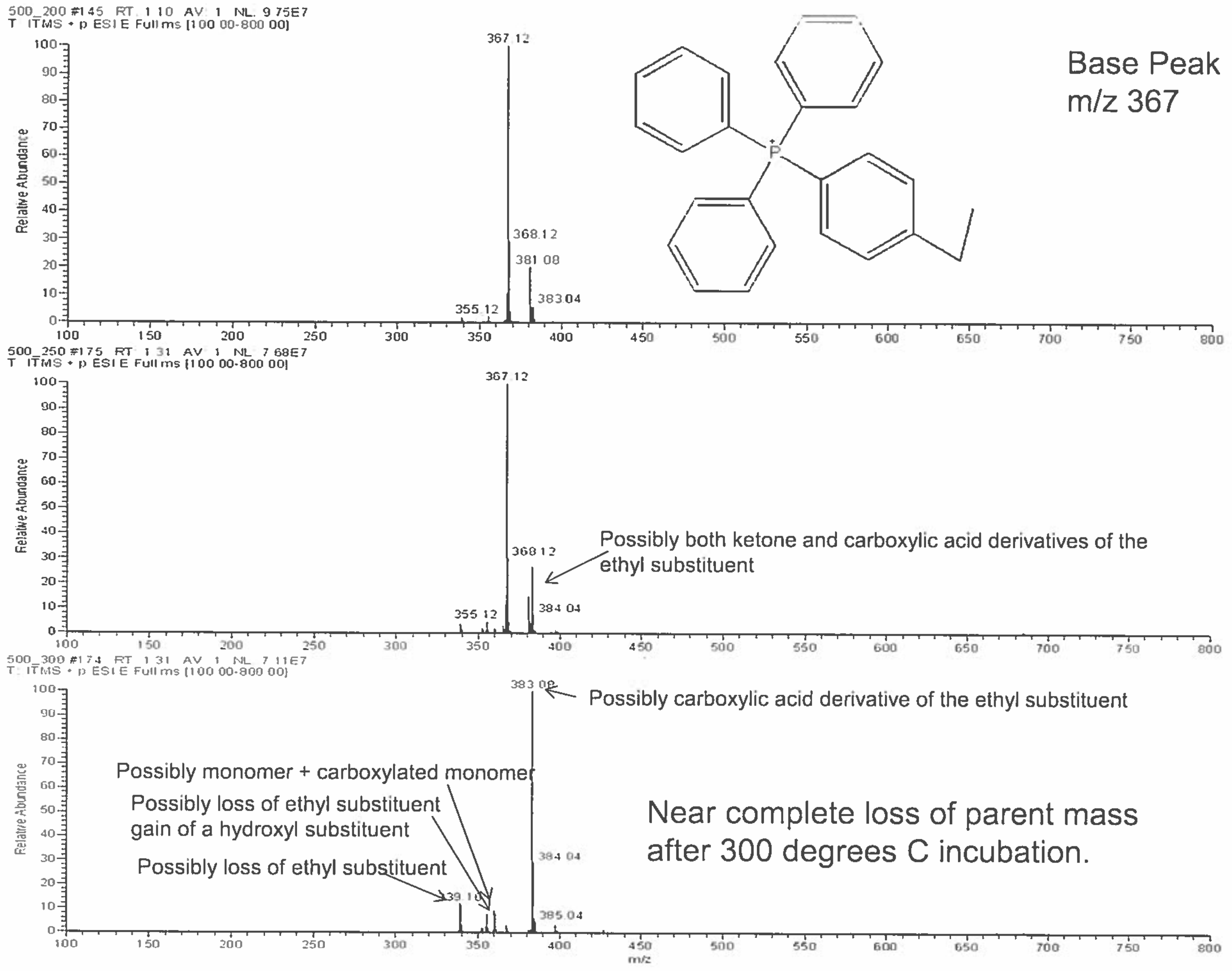

500_200 #145 RT 1 10 AV 1 NL 9 75E7
T ITMS + p ESI E Full ms [100 00-800 00]
Relative Abundance
367 12
368.12
381 08
383.04
355.12
Base Peak
m/z 367
P+
500_250 #175 RT 1 31 AV 1 NL 7 68E7
T ITMS + p ESI E Full ms [100 00-800 00]
367 12
368 12
384 04
355 12
Possibly both ketone and carboxylic acid derivatives of the ethyl substituent
500_300 #174 RT 1 31 AV 1 NL 7 11E7
T ITMS + p ESI E Full ms [100 00-800 00]
383 08
Possibly carboxylic acid derivative of the ethyl substituent
Possibly monomer + carboxylated monomer
Possibly loss of ethyl substituent gain of a hydroxyl substituent
Possibly loss of ethyl substituent
384 04
385 04
Near complete loss of parent mass after 300 degrees C incubation.
m/z

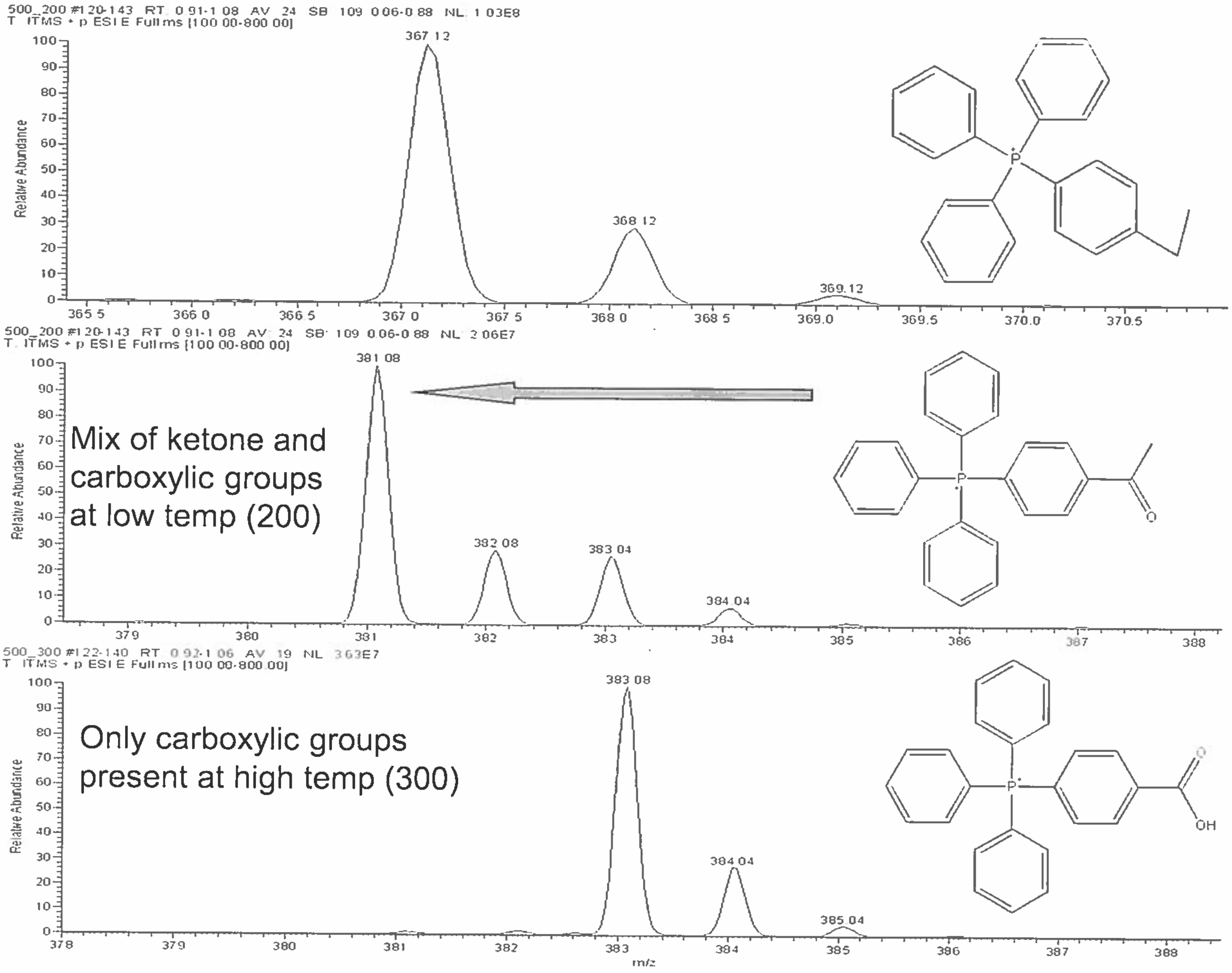
500_200 #120-143 RT 0 91-1 08 AV 24 SB 109 0 06-0 88 NL 1 03E8
T ITMS + p ESI E Full ms [100 00-800 00]
367 12
368 12
369.12
500_200 #120-143 RT 0 91-1 08 AV 24 SB 109 0 06-0 88 NL 2 06E7
T ITMS + p ESI E Full ms [100 00-800 00]
381 08
382 08
383 04
384 04
Mix of ketone and carboxylic groups at low temp (200)
500_300 #122-140 RT 0 92-1 06 AV 19 NL 3 63E7
T ITMS + p ESI E Full ms [100 00-800 00]
383 08
384 04
385 04
Only carboxylic groups present at high temp (300)
Relative Abundance
m/z

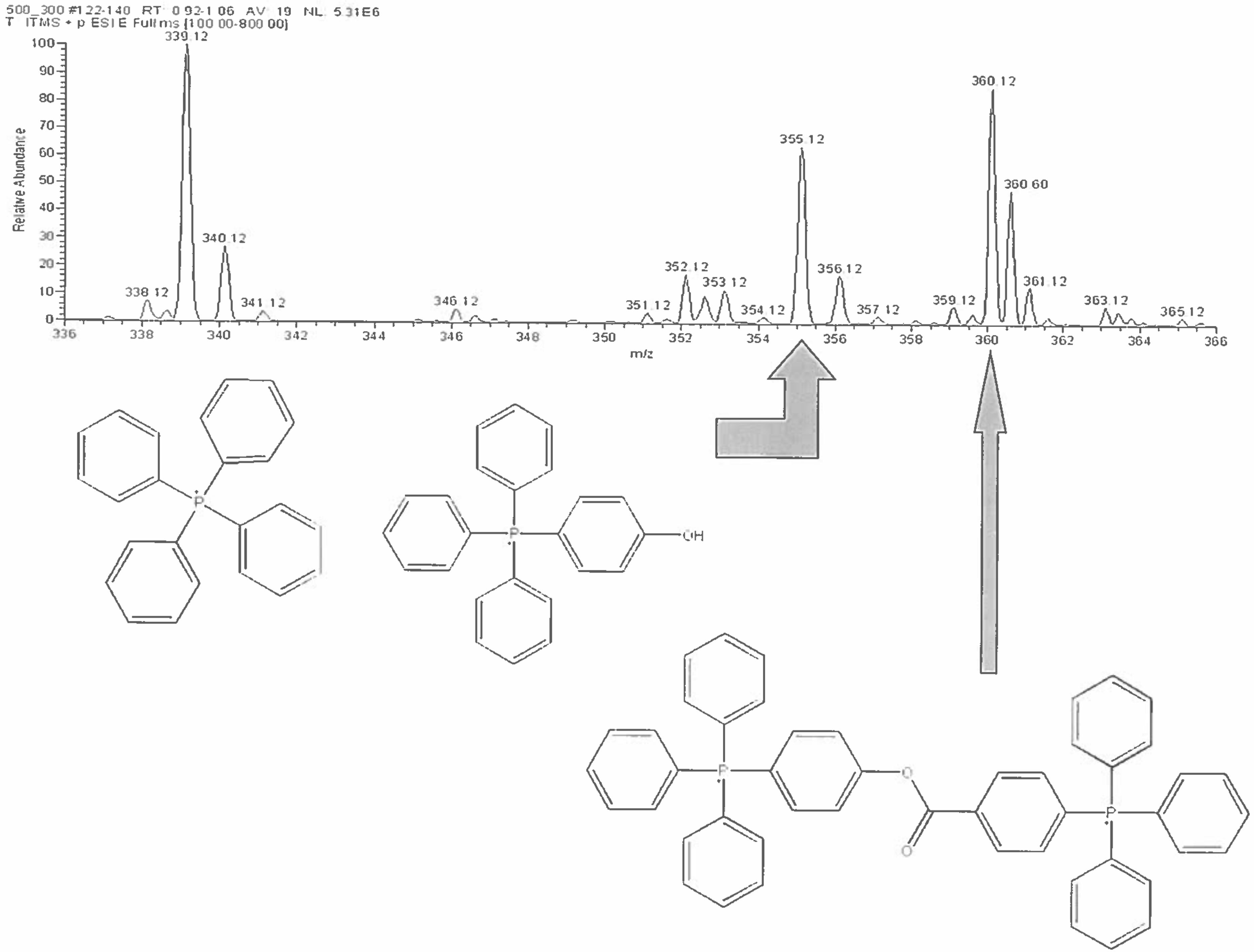
500_300 #122-140 RT 0.92-1.06 AV 19 NL 5.31E6
T ITMS + p ESI E Full ms [100.00-800.00]
Relative Abundance
m/z
339.12
340.12
338.12
341.12
346.12
351.12
352.12
353.12
354.12
355.12
356.12
357.12
359.12
360.12
360.60
361.12
363.12
365.12
P
OH
O

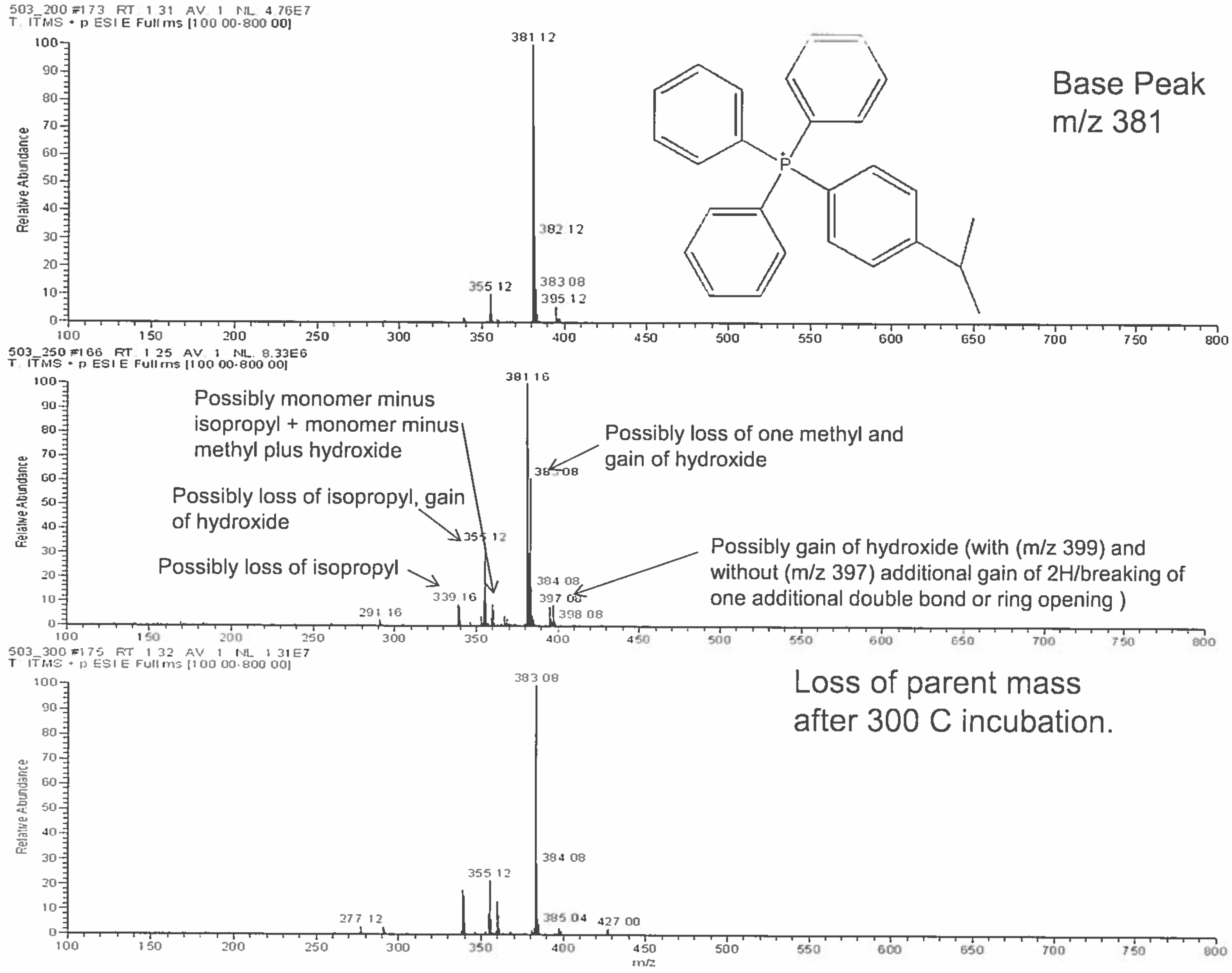

503_200 #173 RT 1.31 AV 1 NL 4.76E7
T ITMS + p ESI E Full ms [100.00-800.00]
Relative Abundance
381.12
382.12
355.12
383.08
395.12
Base Peak
m/z 381
503_250 #166 RT 1.25 AV 1 NL 8.33E6
T ITMS + p ESI E Full ms [100.00-800.00]
381.16
Possibly monomer minus isopropyl + monomer minus methyl plus hydroxide
Possibly loss of one methyl and gain of hydroxide
Possibly loss of isopropyl, gain of hydroxide
Possibly loss of isopropyl
Possibly gain of hydroxide (with (m/z 399) and without (m/z 397) additional gain of 2H/breaking of one additional double bond or ring opening)
339.16
291.16
384.08
397.08
398.08
503_300 #175 RT 1.32 AV 1 NL 1.31E7
T ITMS + p ESI E Full ms [100.00-800.00]
383.08
Loss of parent mass
after 300 C incubation.
384.08
355.12
277.12
385.04
427.00
m/z

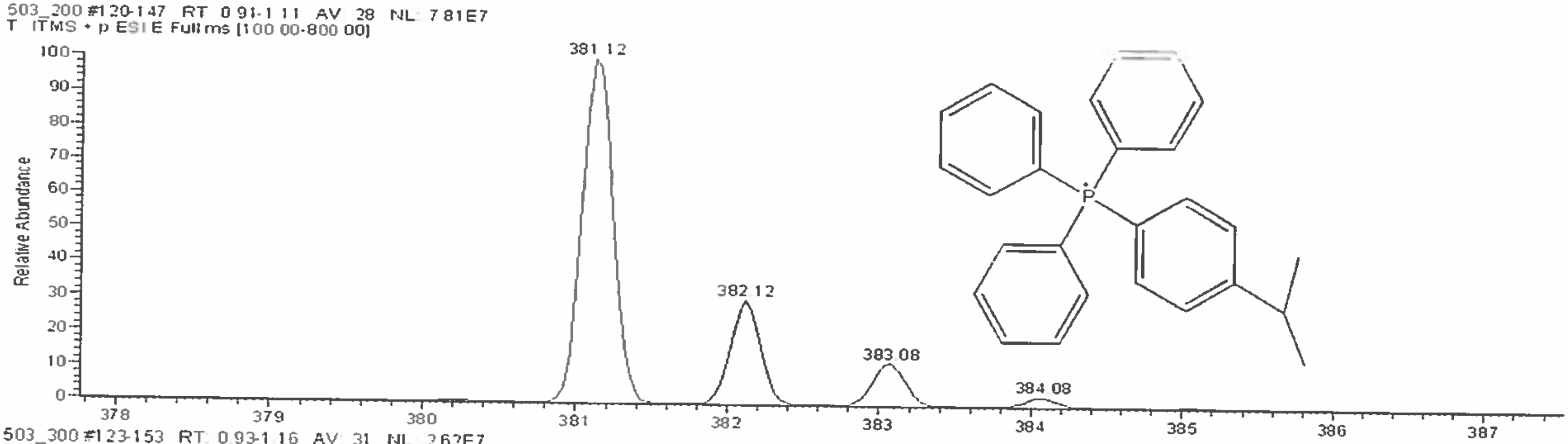
503_200 #120-147 RT 0 91-1 11 AV 28 NL 7 81E7
T ITMS + p ESI E Full ms [100 00-800 00]
Relative Abundance
381 12
382 12
383 08
384 08
P

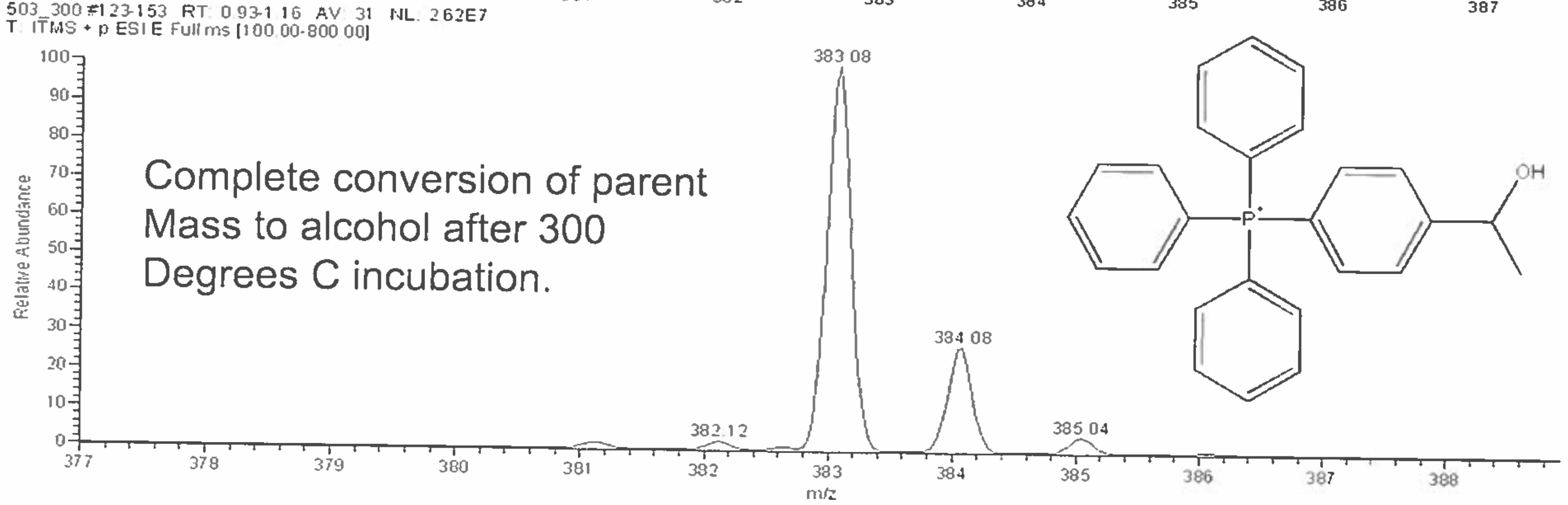
503_300 #123-153 RT 0 93-1 16 AV 31 NL 2 62E7
T ITMS + p ESI E Full ms [100 00-800 00]
Relative Abundance
Complete conversion of parent
Mass to alcohol after 300
Degrees C incubation.
383 08
384 08
382 12
385 04
m/z
P
OH

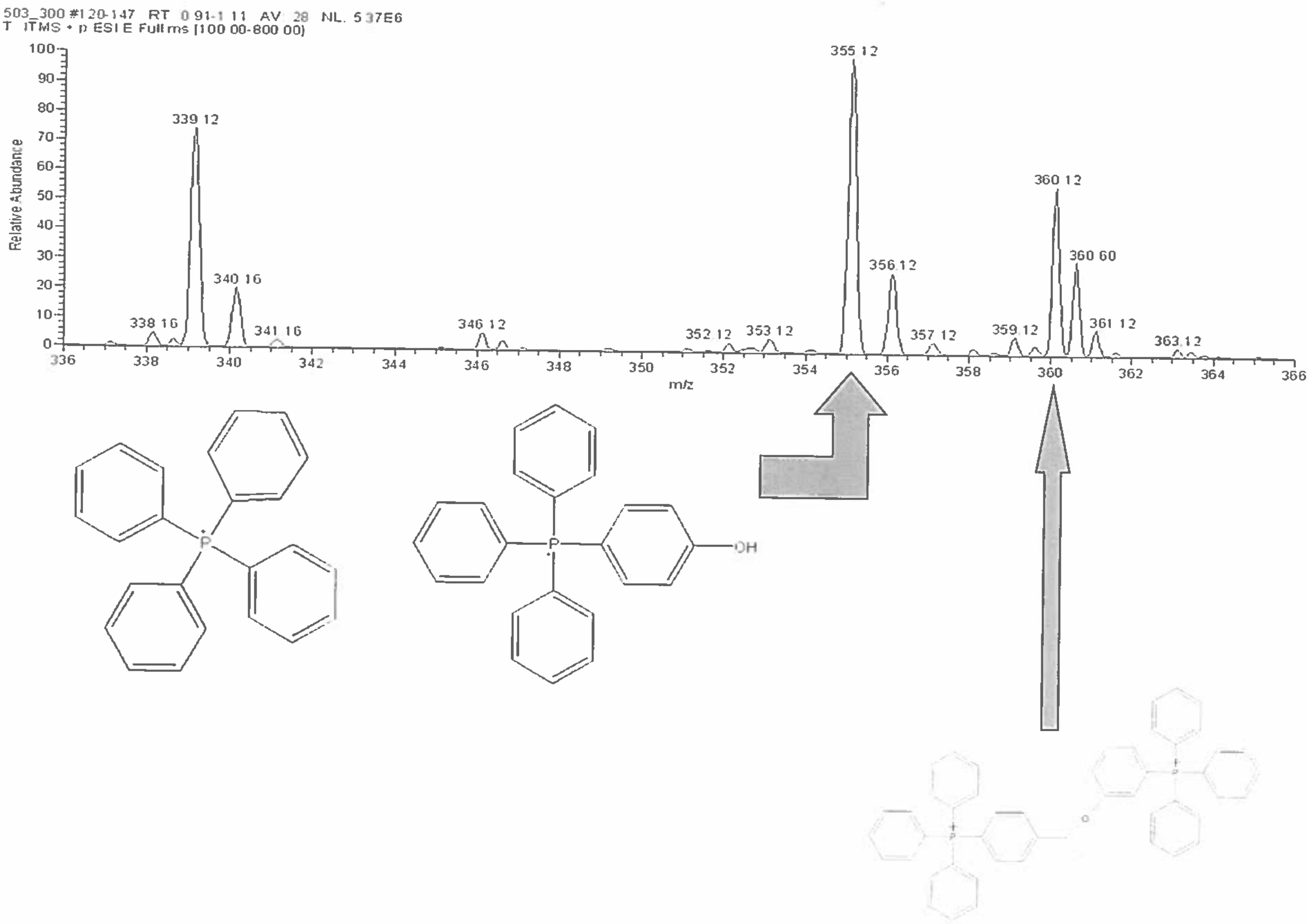

Very similar to 336-366 region for sample MSO 500

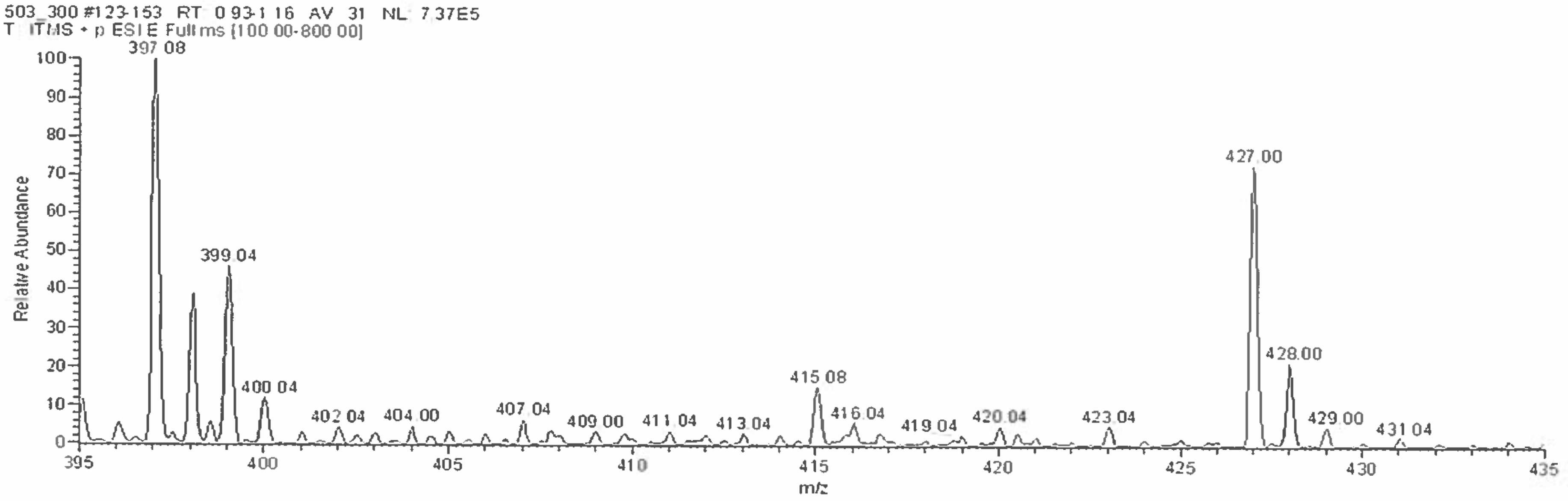

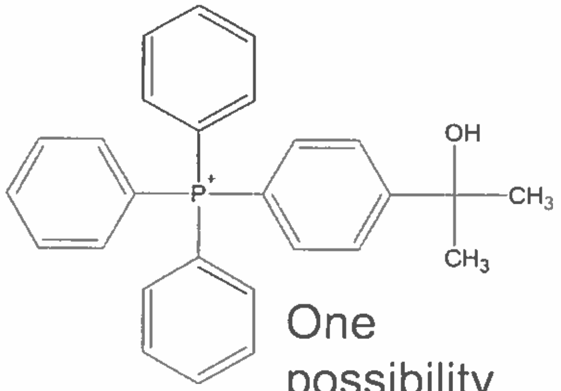

One possibility for 397

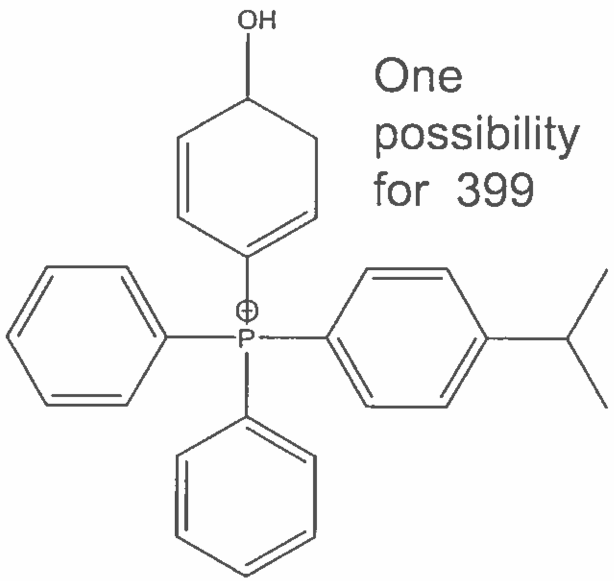

One possibility for 399

c:\xcalibur\...\2018_dec_06\619-300

12/6/2018 2:02:38 PM

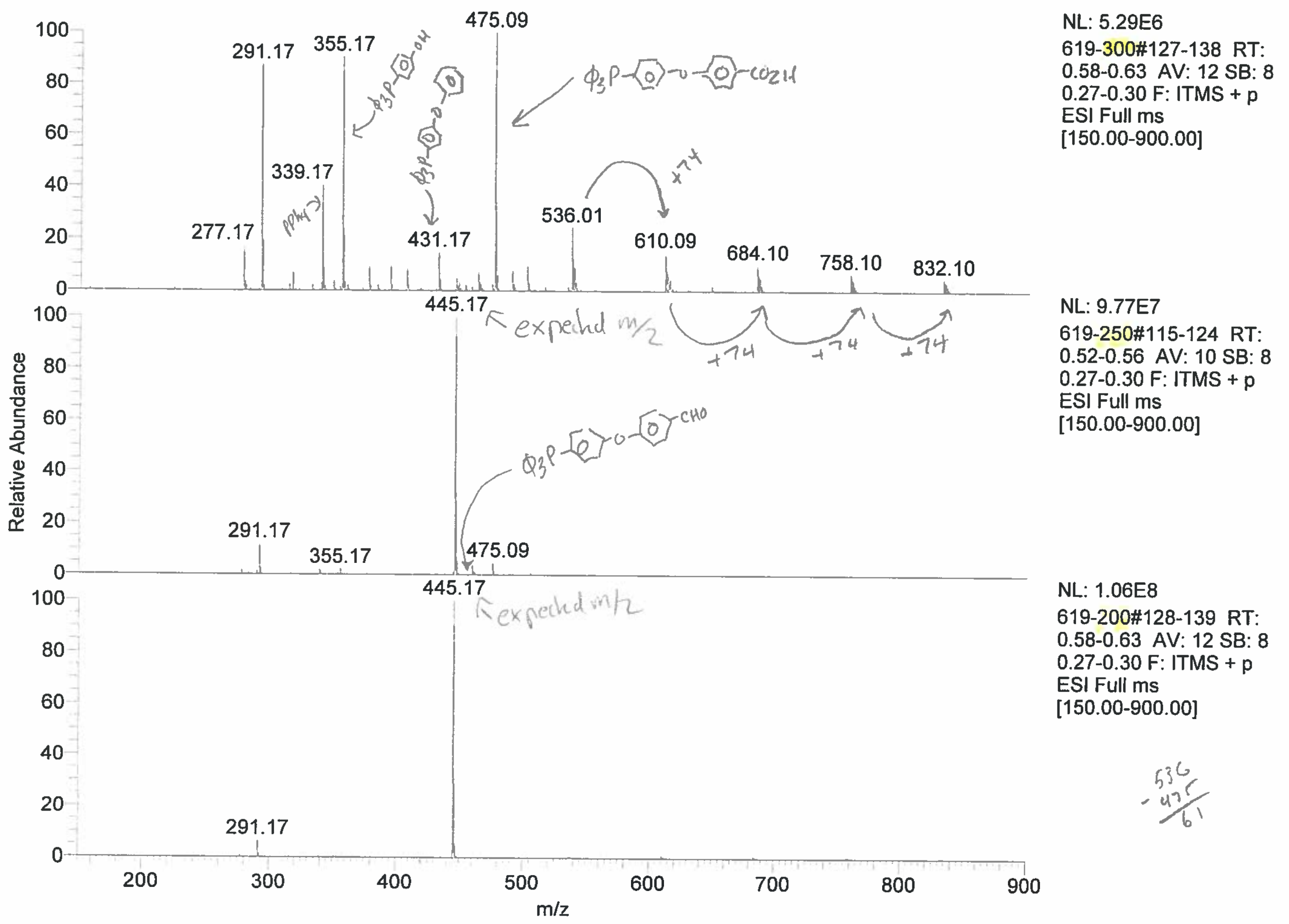

$O=PPh_3 = 278.29$

$262 = PPh_3$
$276 = \phi_2P-tolyl$

MSo494 (collab notebook pg 113)

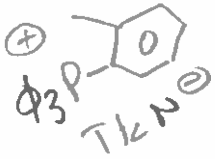

$\phi_2P(O)$-tolyl = 2[illegible]2.10

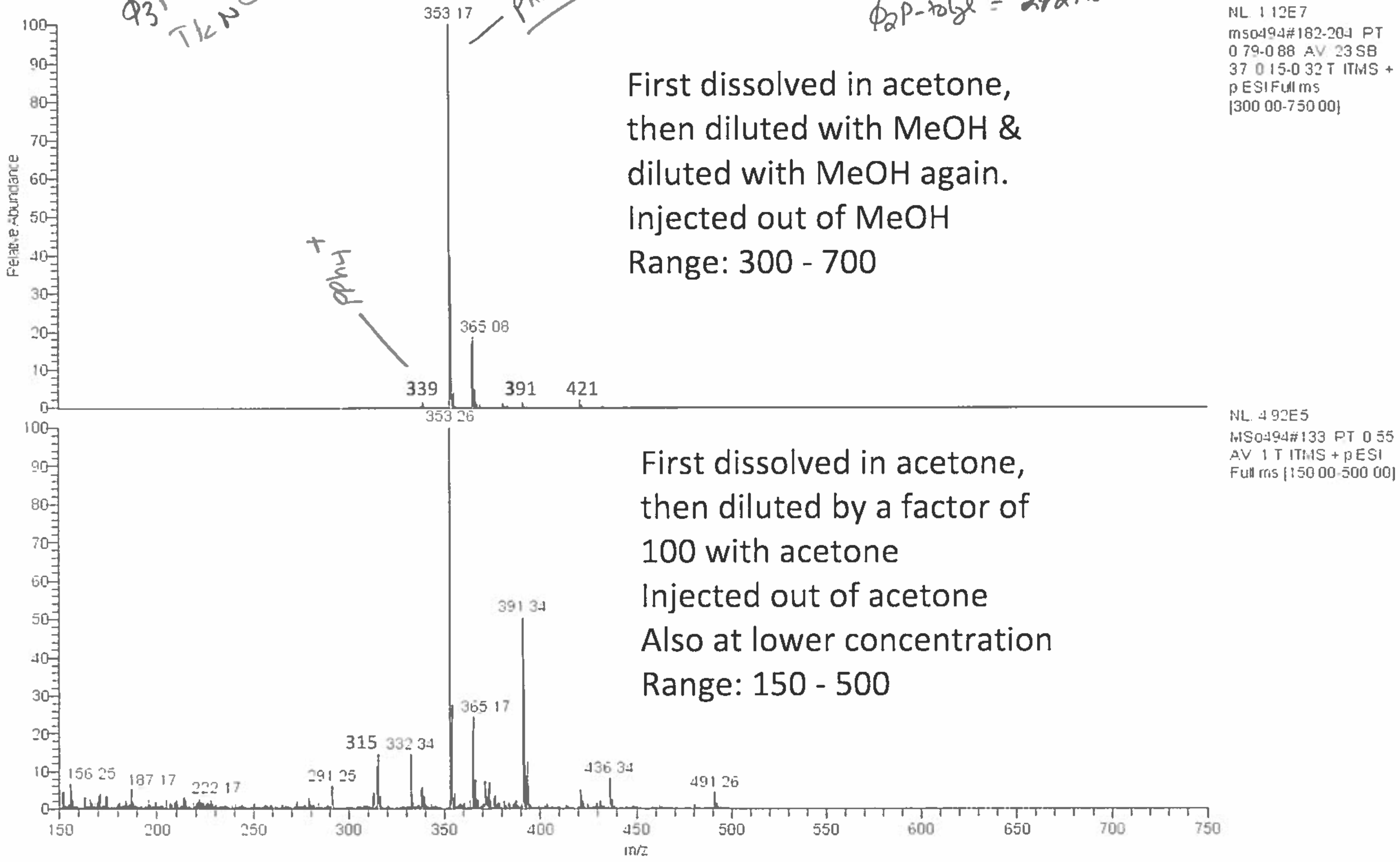

First dissolved in acetone, then diluted with MeOH & diluted with MeOH again.
Injected out of MeOH
Range: 300 - 700

First dissolved in acetone, then diluted by a factor of 100 with acetone
Injected out of acetone
Also at lower concentration
Range: 150 - 500

“aldehyde” (collab notebook pg 111 & 113)

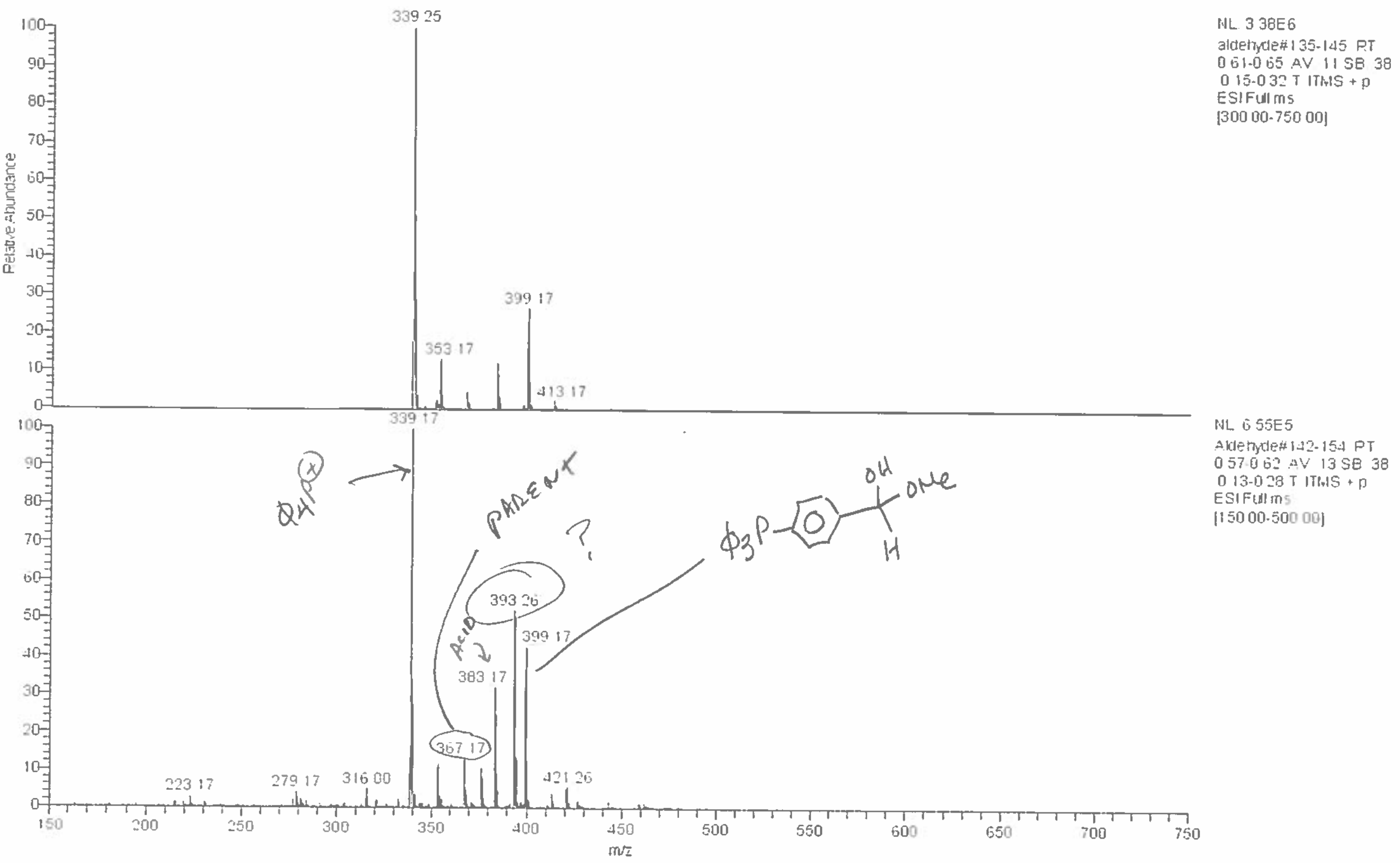

"ketone" (collab notebook pg 111 & 113)

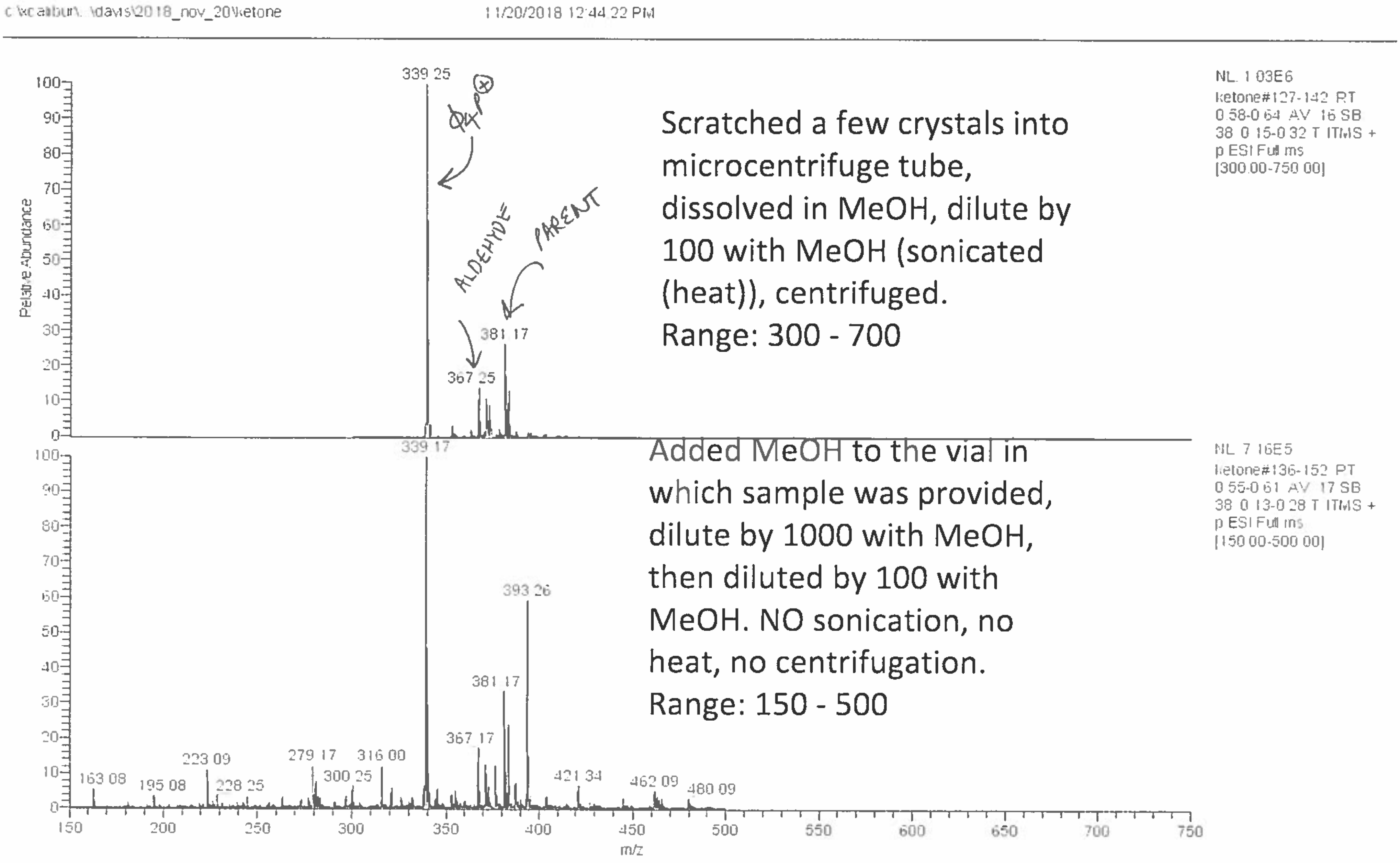

Scratched a few crystals into microcentrifuge tube, dissolved in MeOH, dilute by 100 with MeOH (sonicated (heat)), centrifuged.
Range: 300 - 700

Added MeOH to the vial in which sample was provided, dilute by 1000 with MeOH, then diluted by 100 with MeOH. NO sonication, no heat, no centrifugation.
Range: 150 - 500

“acid” (collab notebook pg 111 & 113)

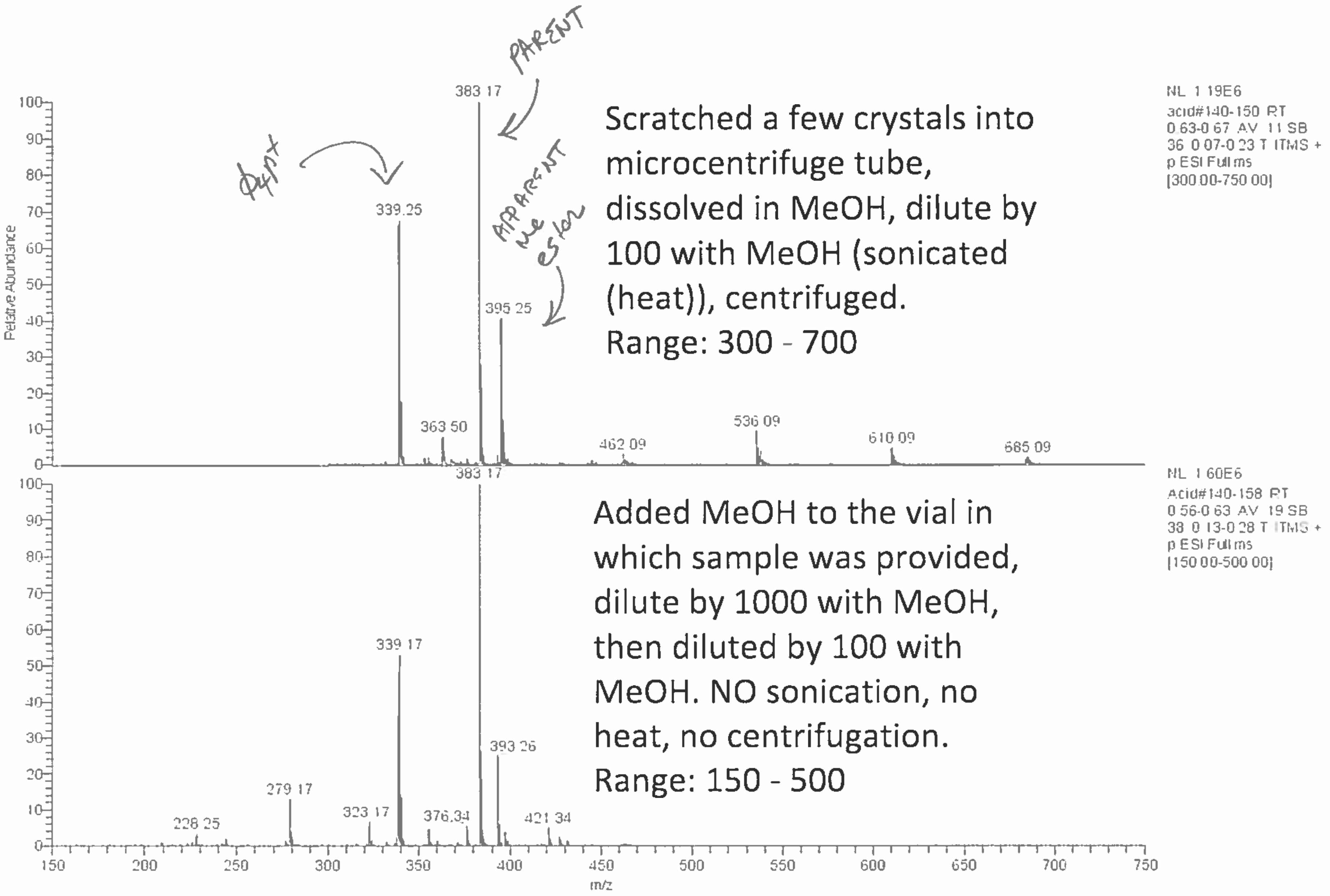

Scratched a few crystals into microcentrifuge tube, dissolved in MeOH, dilute by 100 with MeOH (sonicated (heat)), centrifuged.
Range: 300 - 700

Added MeOH to the vial in which sample was provided, dilute by 1000 with MeOH, then diluted by 100 with MeOH. NO sonication, no heat, no centrifugation.
Range: 150 - 500

C:\Xcalibur\...\2018_Nov_20\Aldehyde 11/20/2018 12:49:58 PM

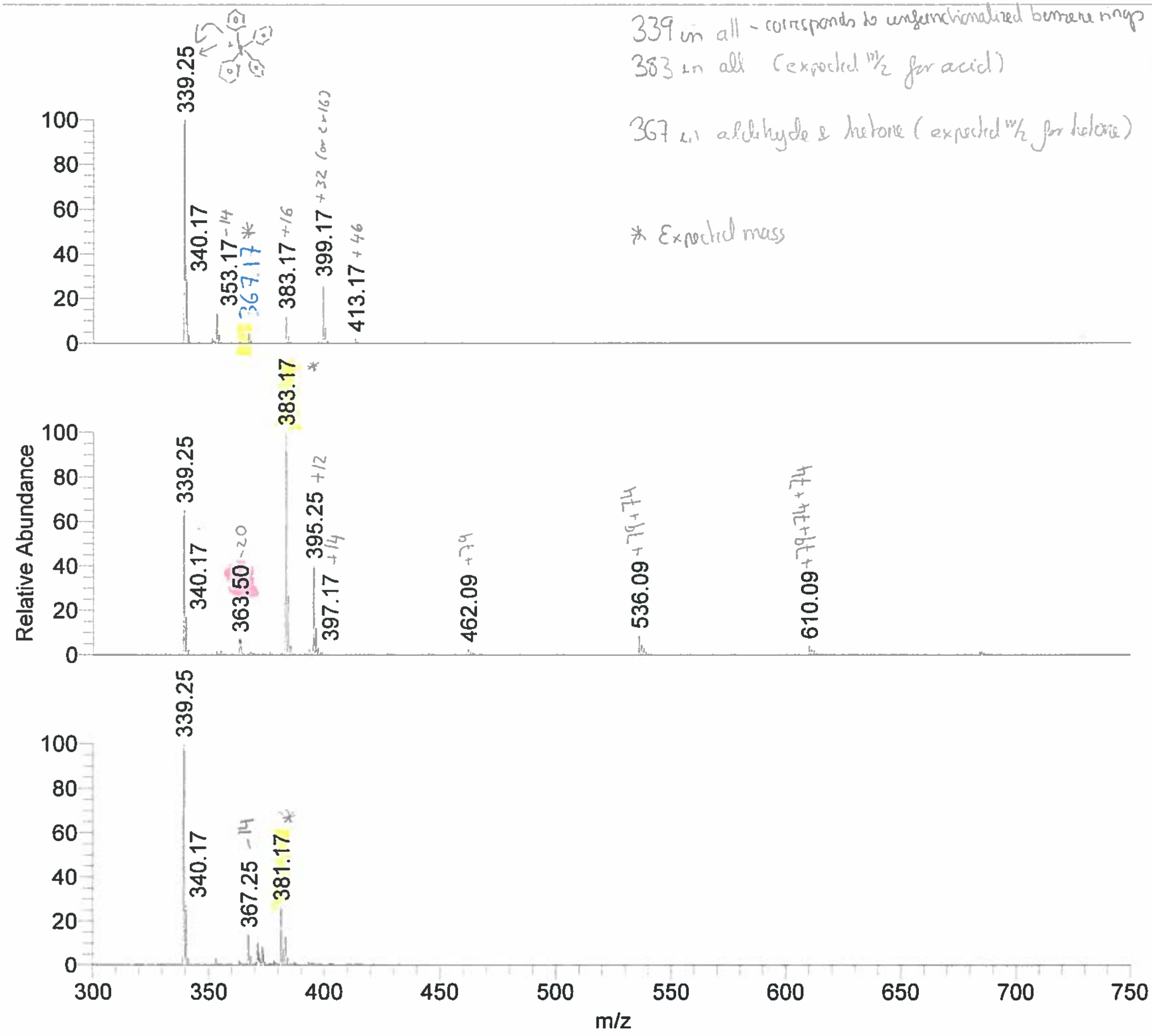

NL: 3.38E6
Aldehyde#133-154 RT: 0.60-0.68 AV: 22 SB: 23
0.15-0.25 T: ITMS + p
ESI Full ms
[300.00-750.00]

462
- 383
79

NL: 1.21E6
acid#140-156 RT: 0.63-0.70 AV: 17 SB: 22
0.15-0.25 T: ITMS + p
ESI Full ms
[300.00-750.00]

NL: 9.77E5
Ketone#122-146 RT: 0.56-0.66 AV: 25 SB: 23
0.15-0.25 T: ITMS + p
ESI Full ms
[300.00-750.00]

79
74

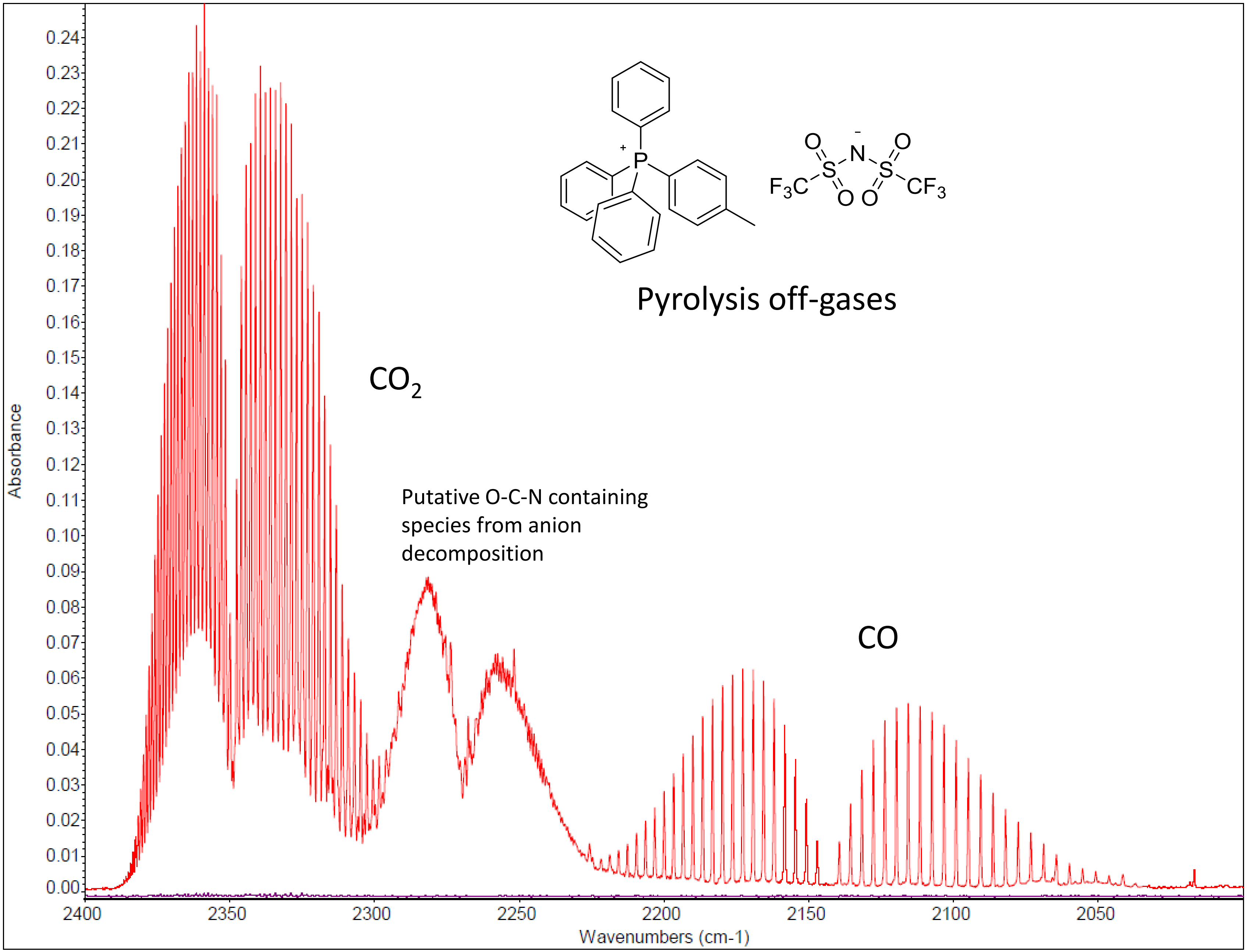

Pyrolysis off-gases
CO_2
Putative O-C-N containing species from anion decomposition
CO
Absorbance
Wavenumbers (cm-1)
0.24
0.23
0.22
0.21
0.20
0.19
0.18
0.17
0.16
0.15
0.14
0.13
0.12
0.11
0.10
0.09
0.08
0.07
0.06
0.05
0.04
0.03
0.02
0.01
0.00
2400
2350
2300
2250
2200
2150
2100
2050

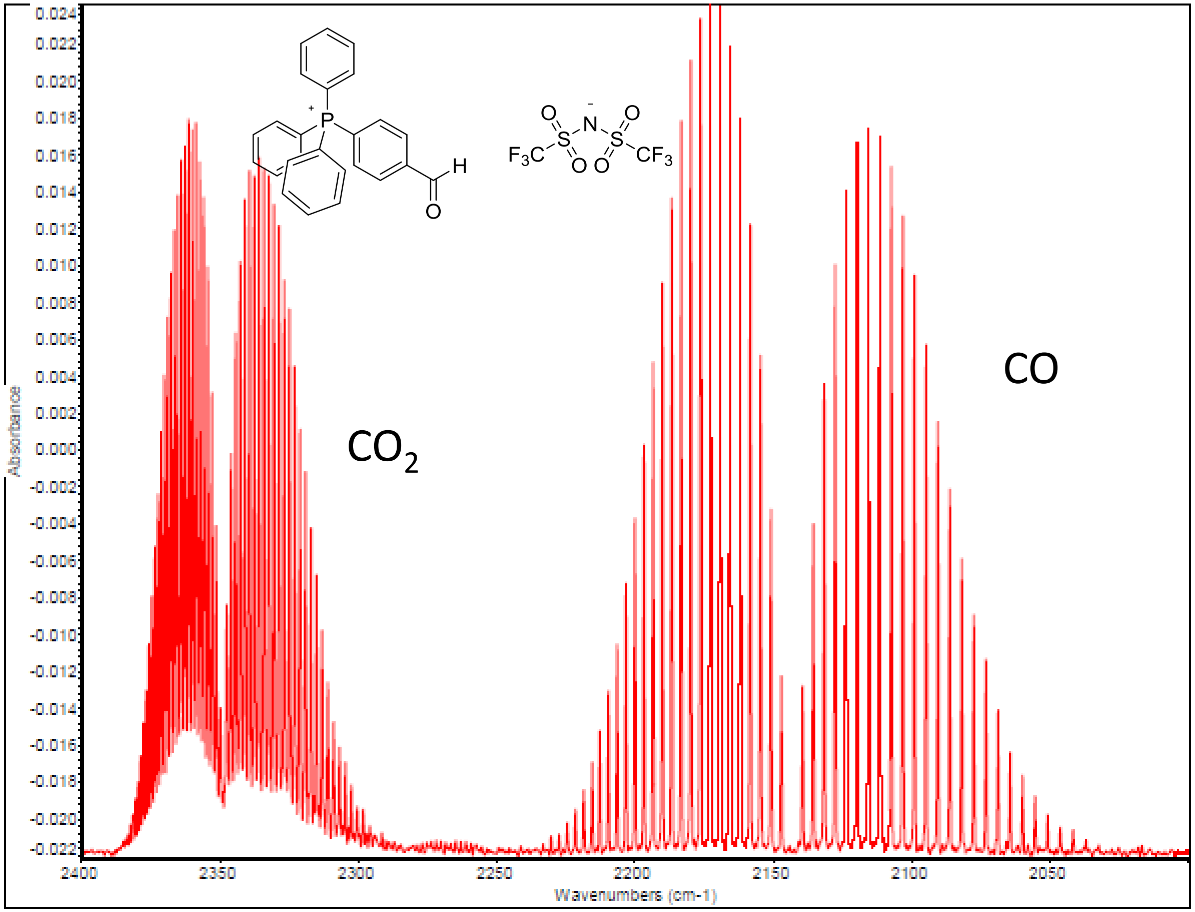

CO$_2$
CO
Absorbance
Wavenumbers (cm-1)

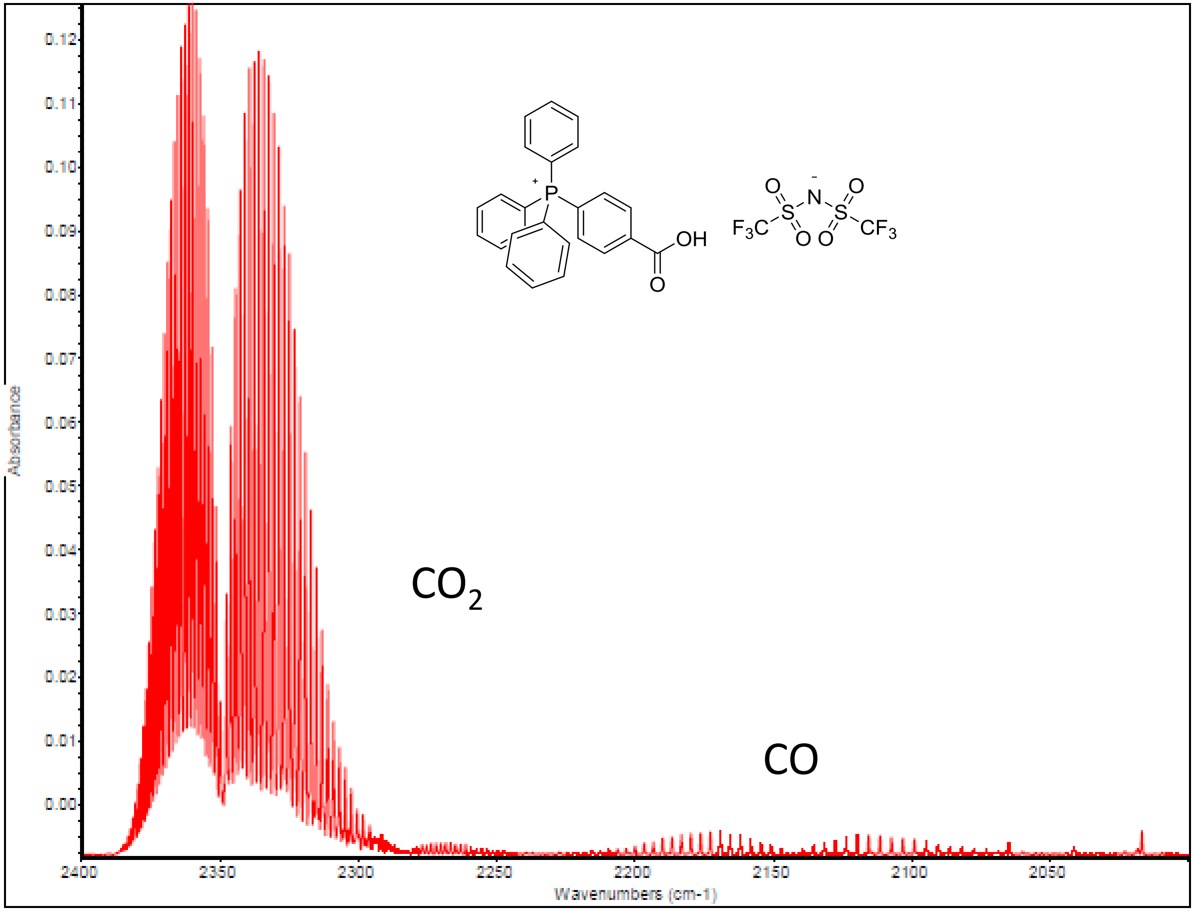

0.12
0.11
0.10
0.09
0.08
0.07
0.06
0.05
0.04
0.03
0.02
0.01
0.00
Absorbance
2400
2350
2300
2250
2200
2150
2100
2050
Wavenumbers (cm-1)
CO_2
CO
OH
O
F_3C
CF_3
S
N
P

Key to structures for TGA scans

MS0441

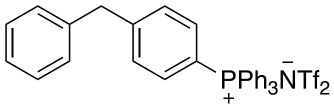

MS0468

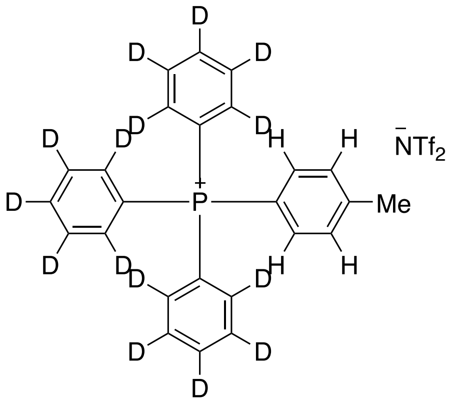

MS0477

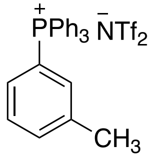

MS0500

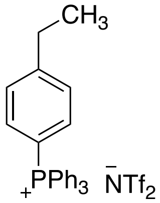

MS0503

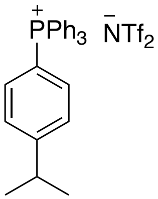

MS0630

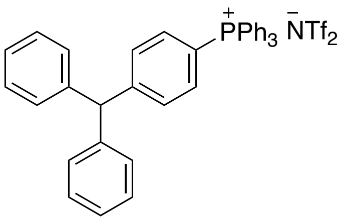

Sample: 441
Size: 12.7630 mg
Method: test

TGA

File: C:...\MS\1 min ramp rate\36 h\441.002

Run Date: 22-Apr-2019 08:28
Instrument: TGA Q500 V20.2 Build 27

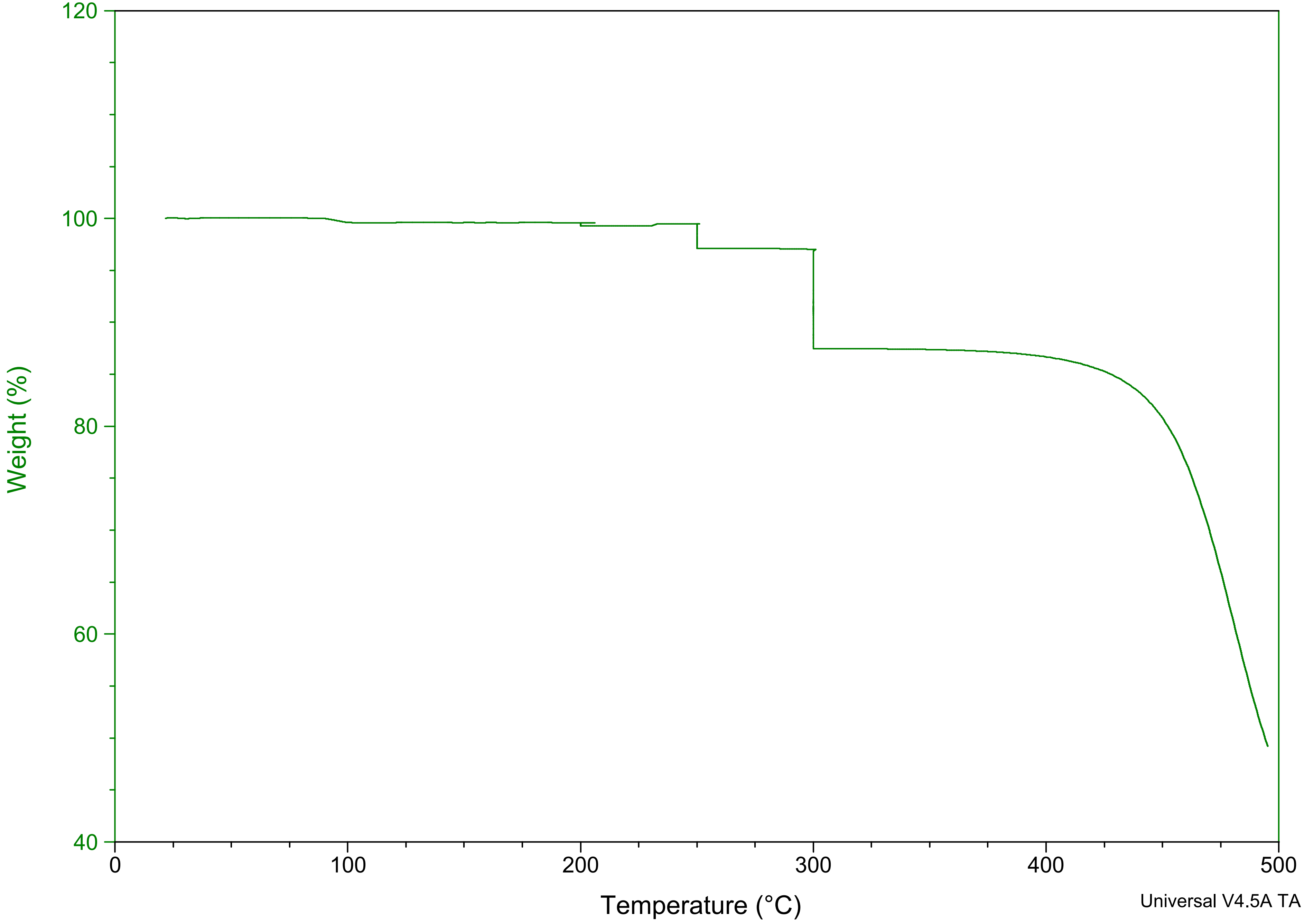

Universal V4.5A TA Instruments

Sample: 441
Size: 12.7630 mg
Method: test

TGA

File: C:...\MS\1 min ramp rate\36 h\441.002

Run Date: 22-Apr-2019 08:28
Instrument: TGA Q500 V20.2 Build 27

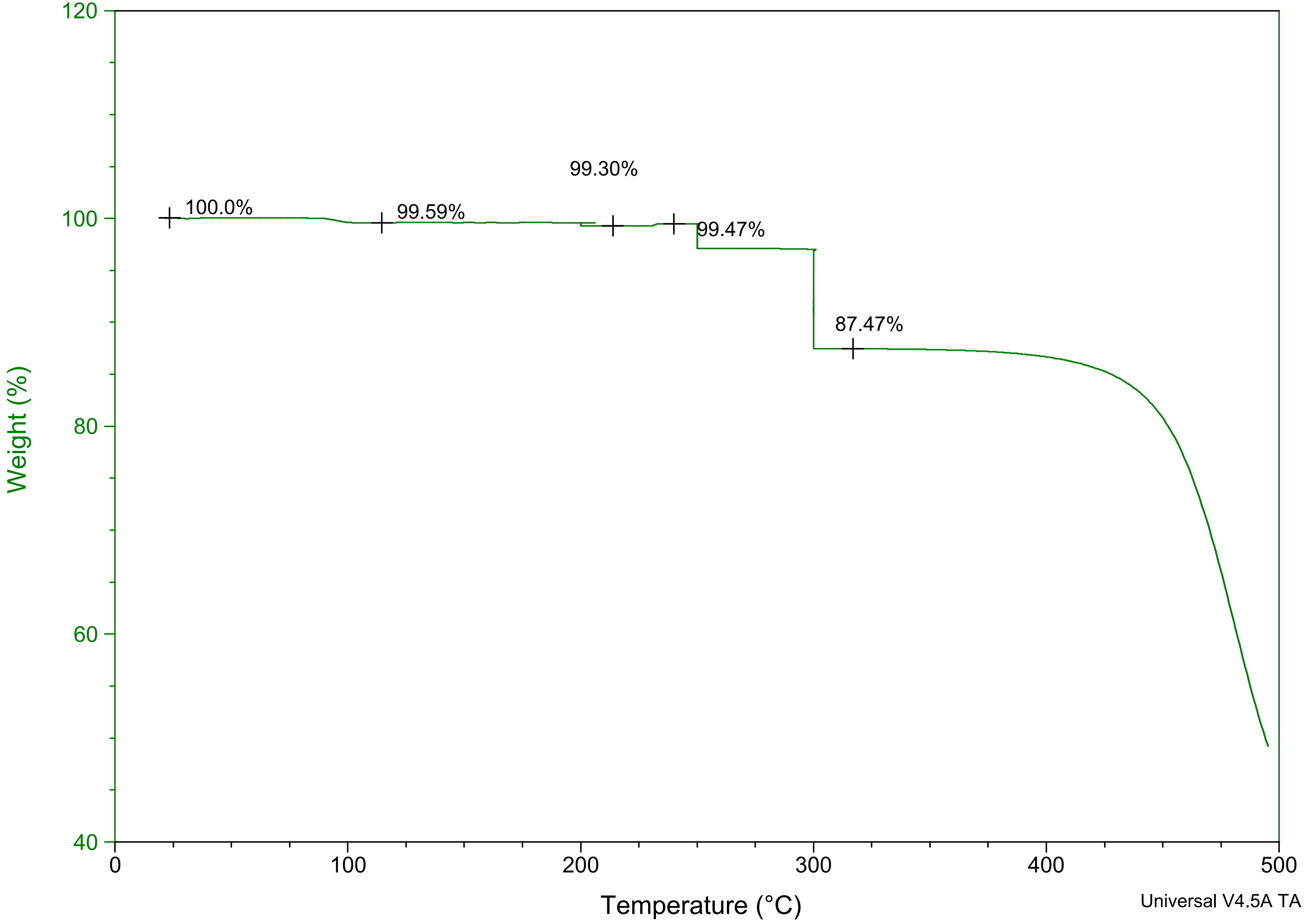

Universal V4.5A TA Instruments

Sample: 441
Size: 12.7630 mg
Method: test

TGA

File: C:...\MS\1 min ramp rate\36 h\441.002

Run Date: 22-Apr-2019 08:28
Instrument: TGA Q500 V20.2 Build 27

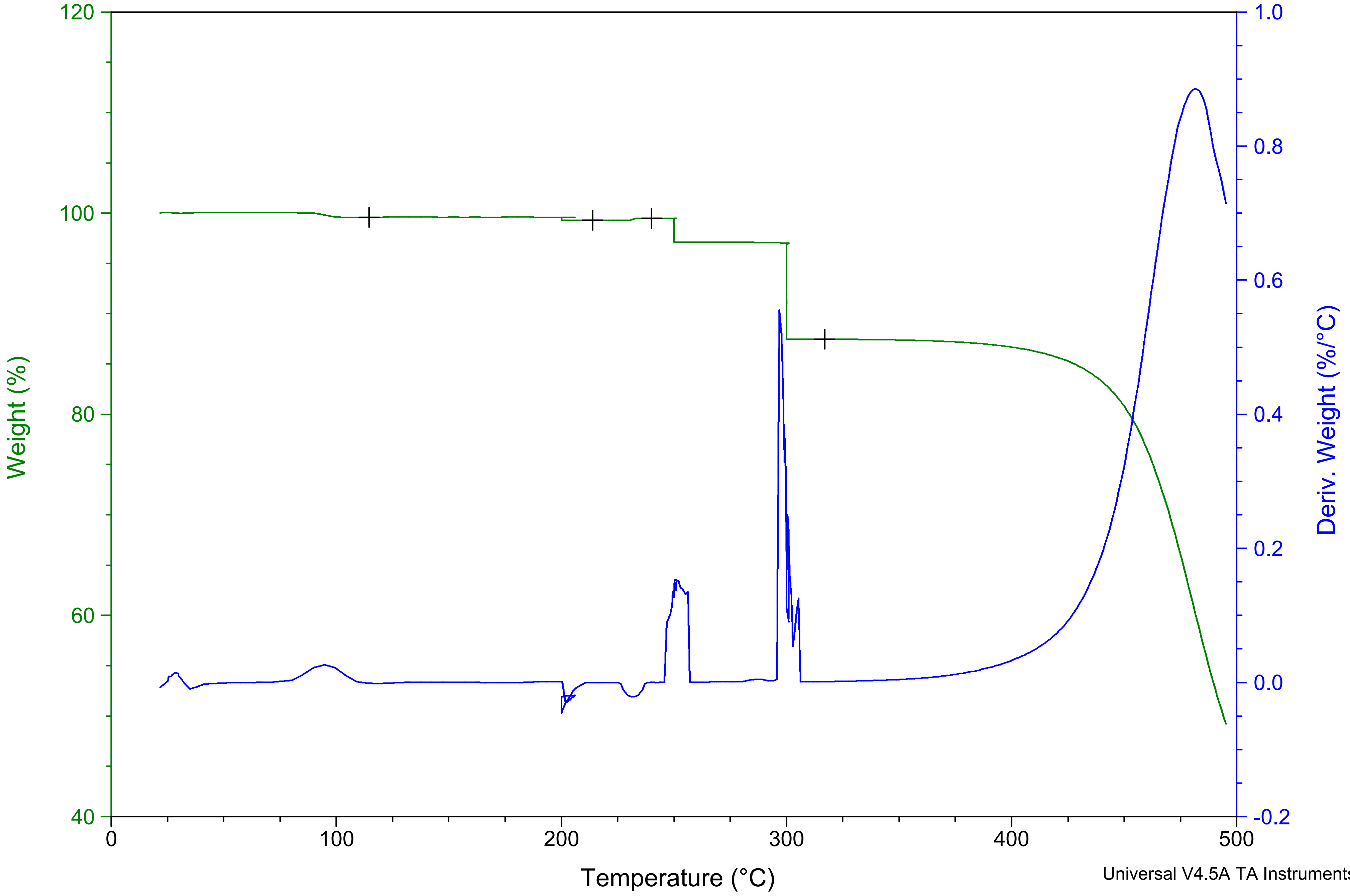

Universal V4.5A TA Instruments

Sample: 468
Size: 12.3470 mg
Method: test

TGA

File: C:...\MS\1 min ramp rate\36 h\468.002

Run Date: 19-Apr-2019 09:07
Instrument: TGA Q500 V20.2 Build 27

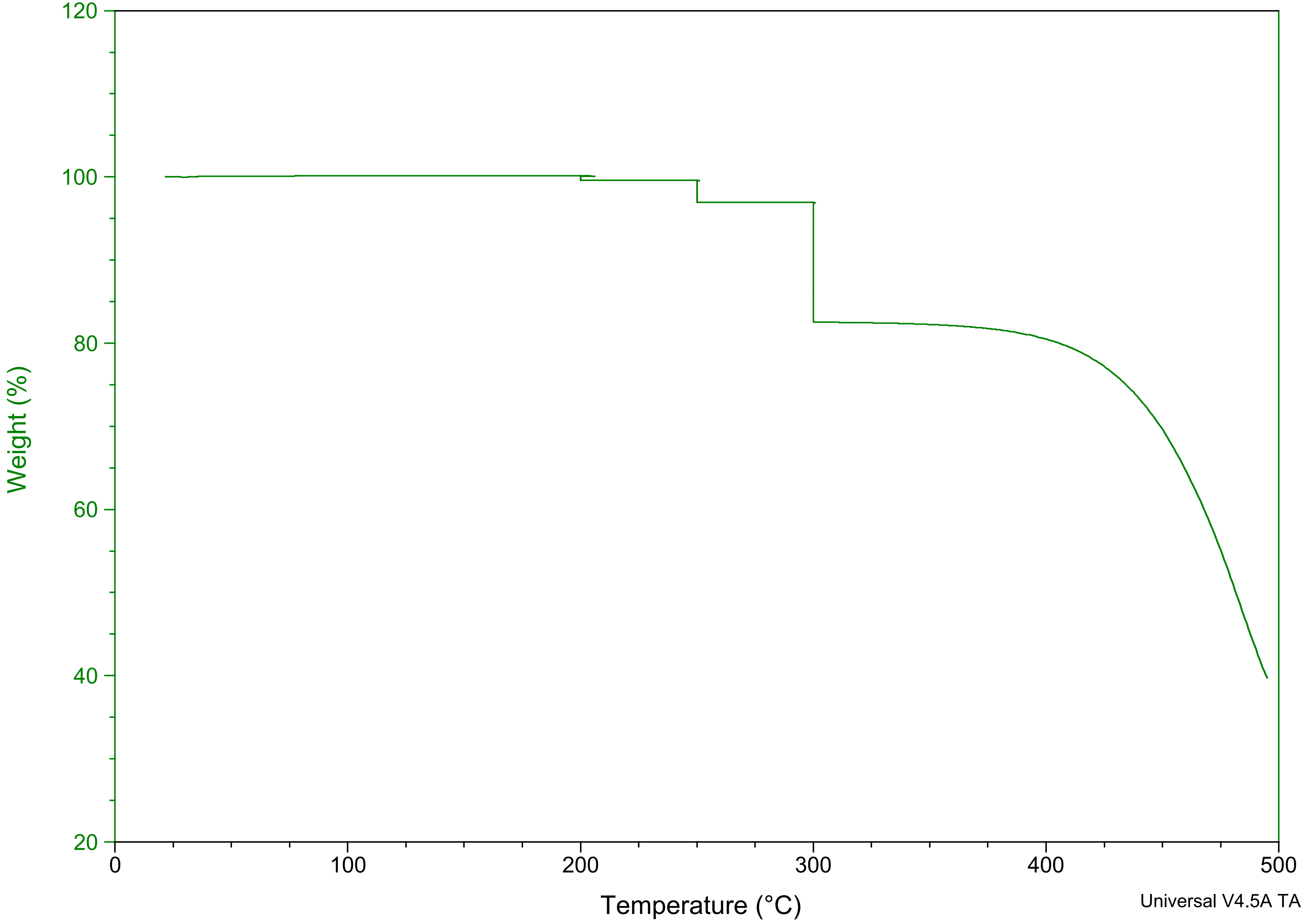

Universal V4.5A TA Instruments

Sample: 468
Size: 12.3470 mg
Method: test

TGA

File: C:...\MS\1 min ramp rate\36 h\468.002

Run Date: 19-Apr-2019 09:07
Instrument: TGA Q500 V20.2 Build 27

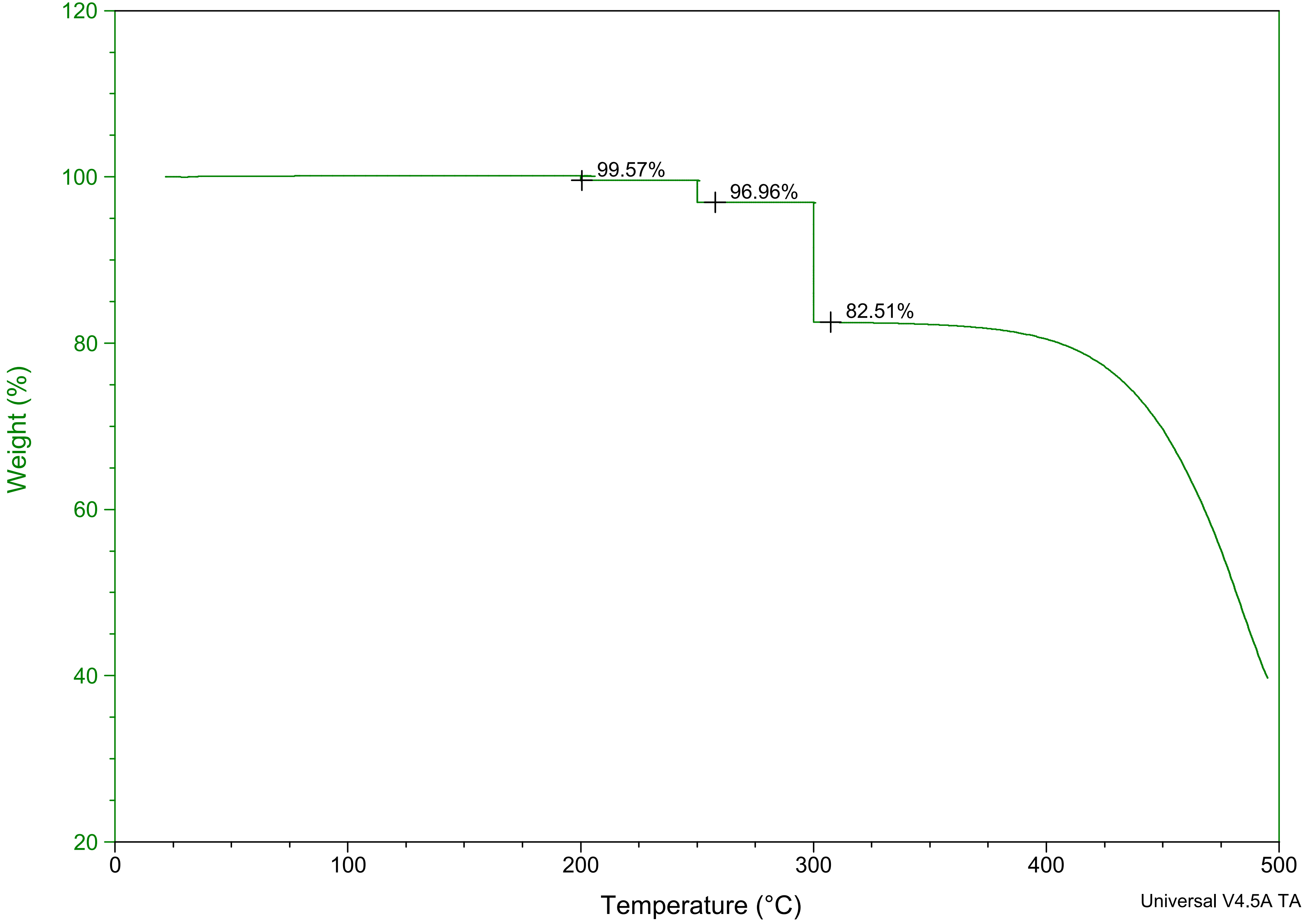

Universal V4.5A TA Instruments

Sample: 468
Size: 12.3470 mg
Method: test

TGA

File: C:...\MS\1 min ramp rate\36 h\468.002

Run Date: 19-Apr-2019 09:07
Instrument: TGA Q500 V20.2 Build 27

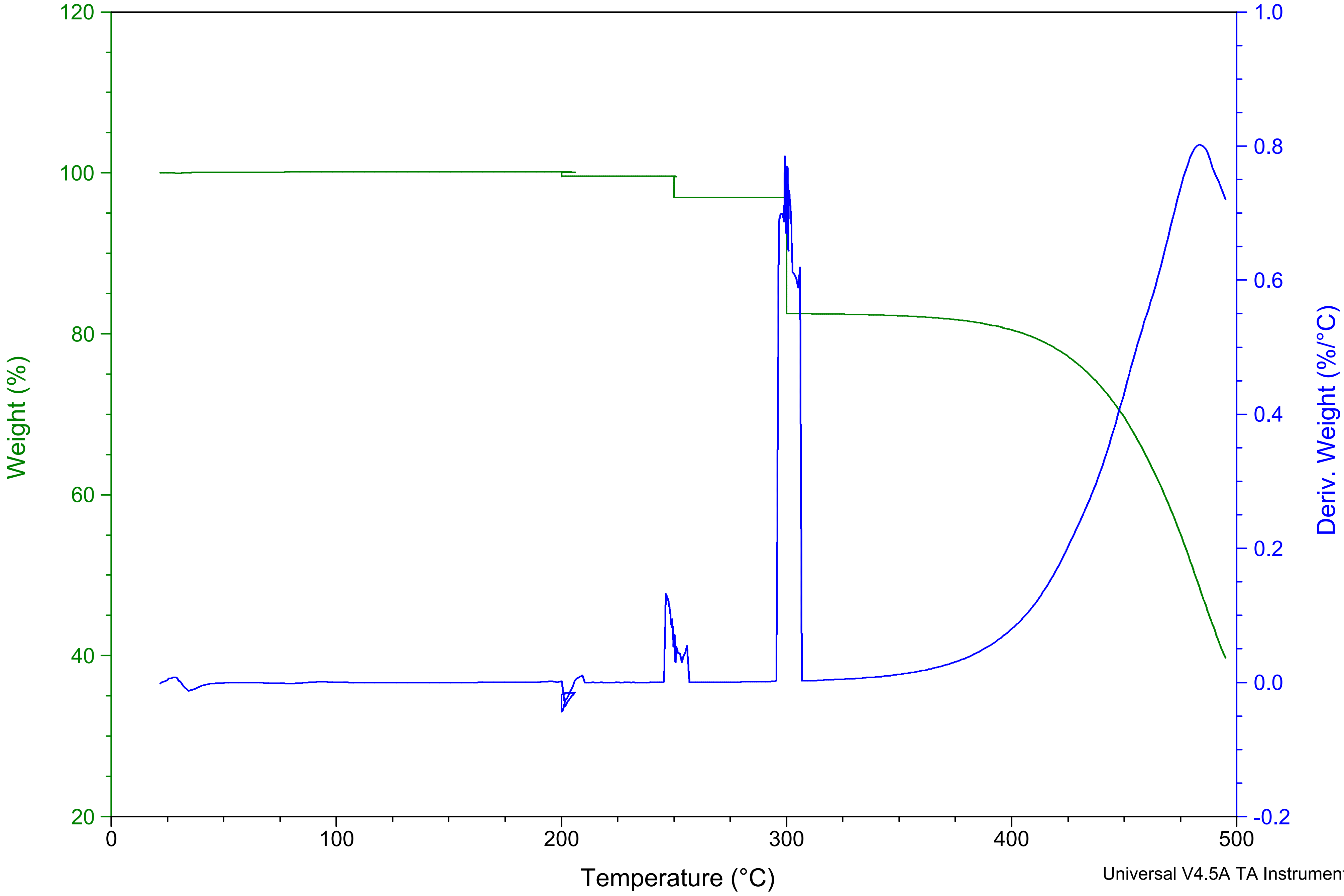

Universal V4.5A TA Instruments

Sample: 477
Size: 16.9800 mg
Method: test

TGA

File: C:...\MS\1 min ramp rate\36 h\477.001

Run Date: 05-Apr-2019 09:34
Instrument: TGA Q500 V20.2 Build 27

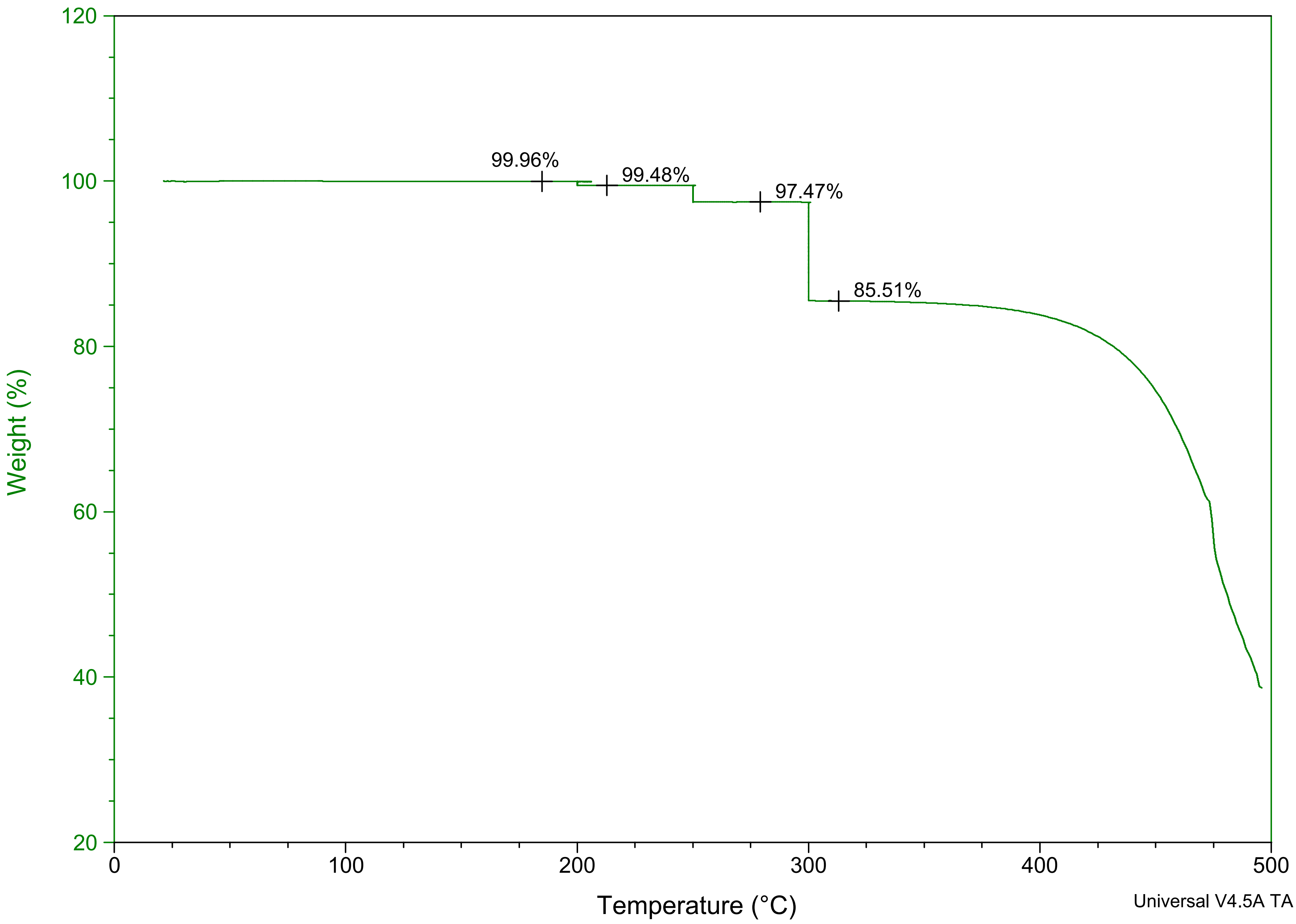

Universal V4.5A TA Instruments

Sample: 477
Size: 16.9800 mg
Method: test

TGA

File: C:...\MS\1 min ramp rate\36 h\477.001

Run Date: 05-Apr-2019 09:34
Instrument: TGA Q500 V20.2 Build 27

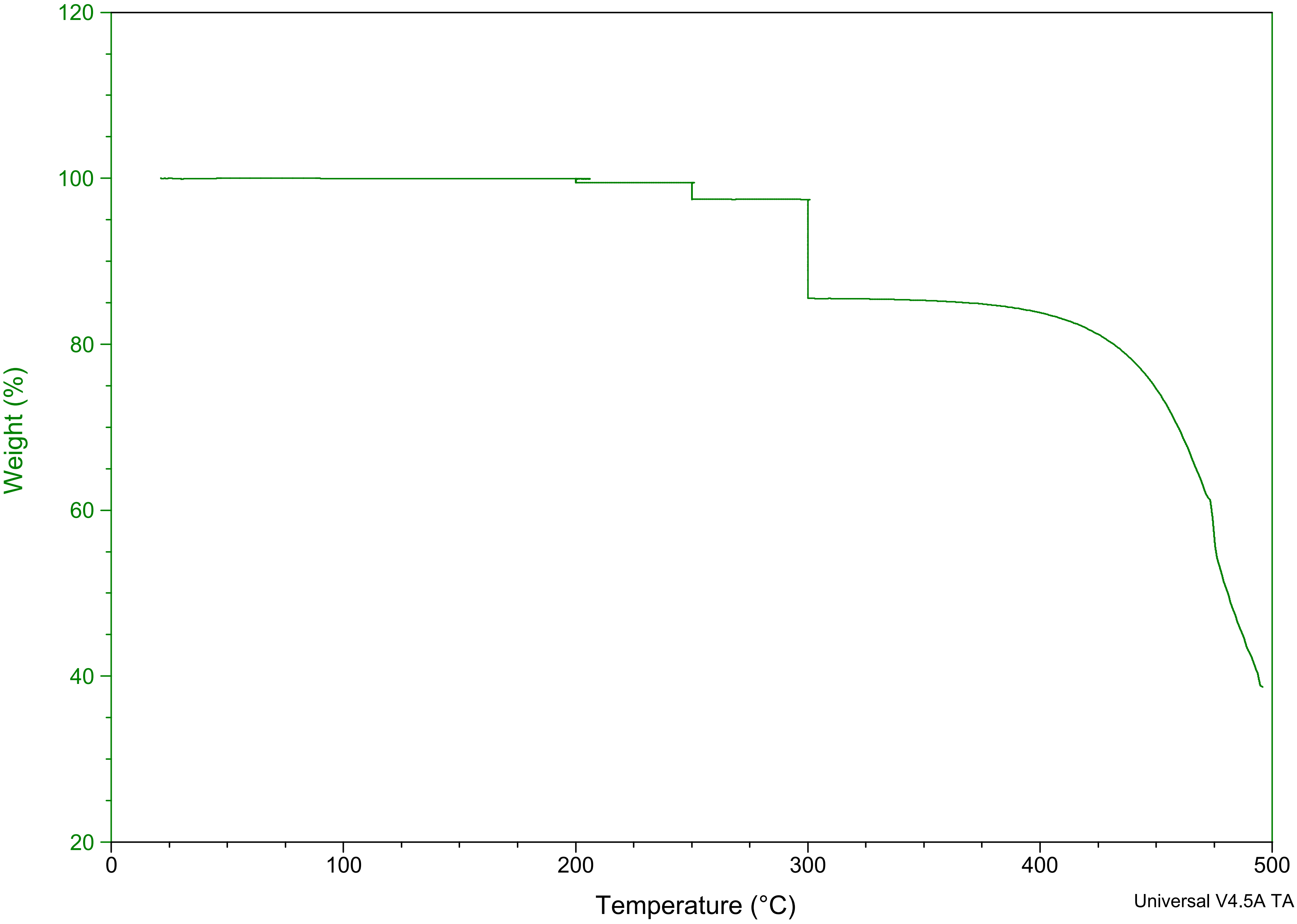

Universal V4.5A TA Instruments

Sample: 477
Size: 16.9800 mg
Method: test

TGA

File: C:...\MS\1 min ramp rate\36 h\477.001

Run Date: 05-Apr-2019 09:34
Instrument: TGA Q500 V20.2 Build 27

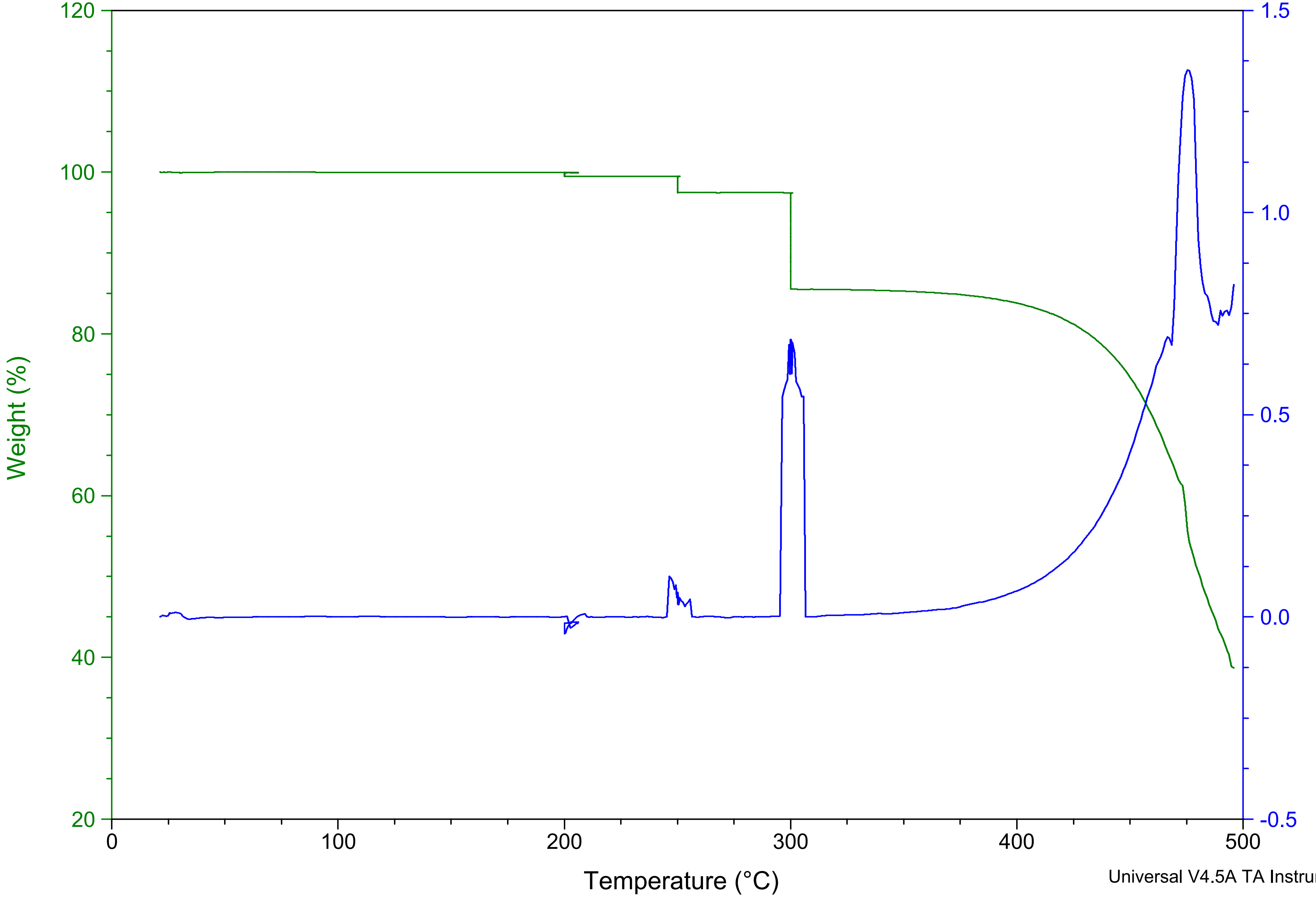

Universal V4.5A TA Instruments

Sample: 477
Size: 16.9800 mg
Method: test

TGA

File: C:...\MS\1 min ramp rate\36 h\477.001

Run Date: 05-Apr-2019 09:34
Instrument: TGA Q500 V20.2 Build 27

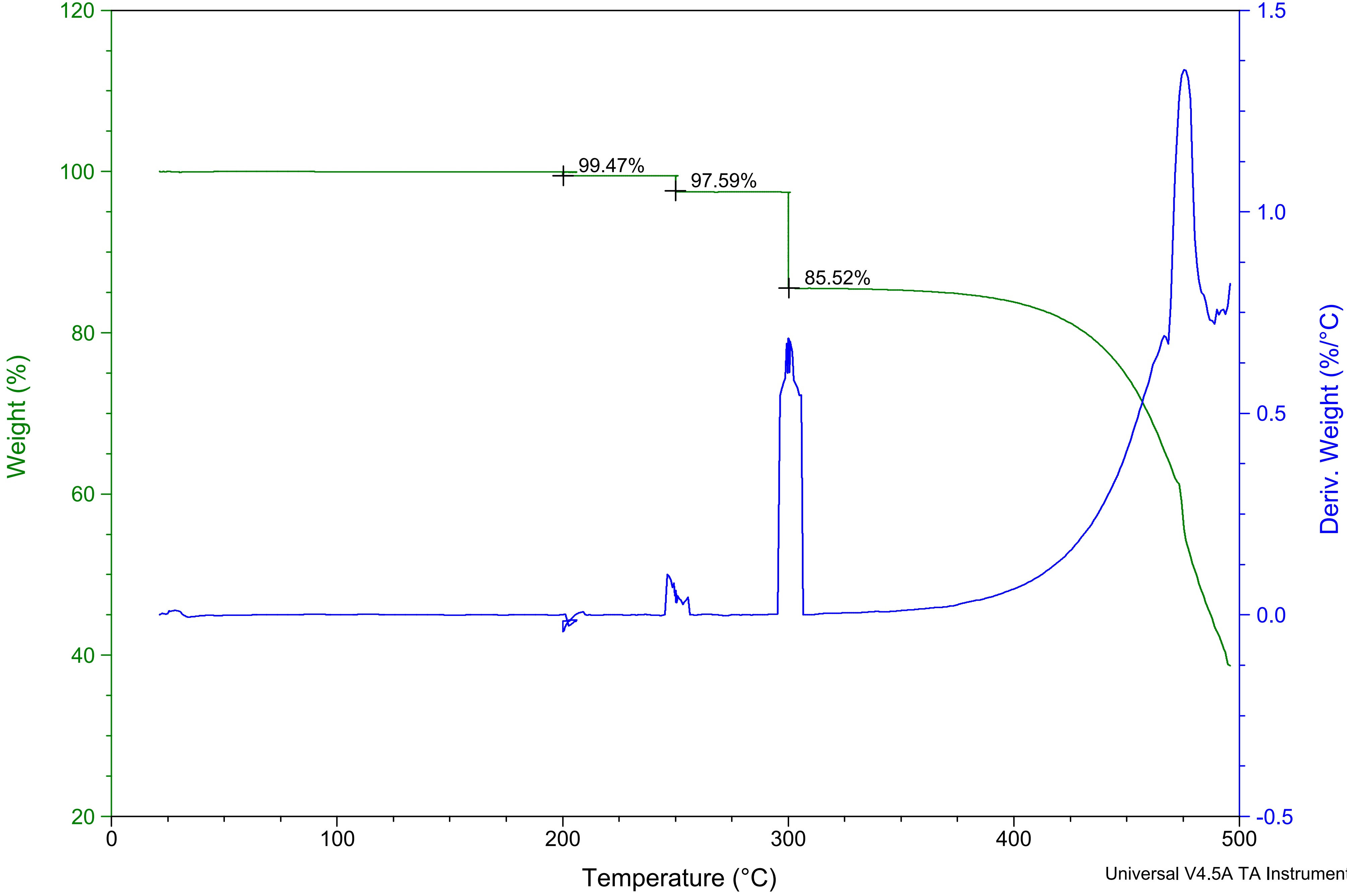

Universal V4.5A TA Instruments

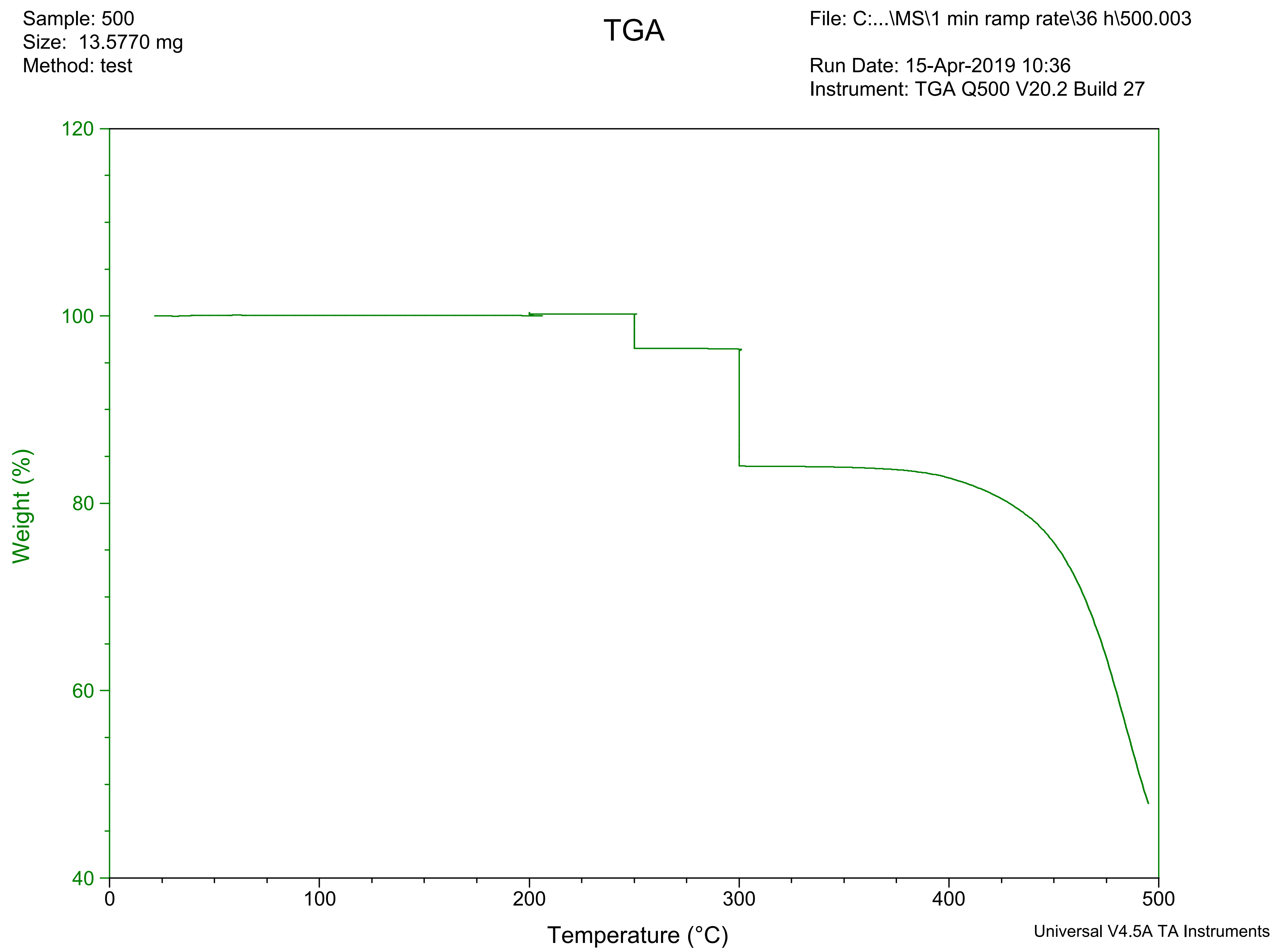
Sample: 500
Size: 13.5770 mg
Method: test
TGA
File: C:...\MS\1 min ramp rate\36 h\500.003
Run Date: 15-Apr-2019 10:36
Instrument: TGA Q500 V20.2 Build 27
Weight (%)
120
100
80
60
40
0
100
200
300
400
500
Temperature (°C)
Universal V4.5A TA Instruments

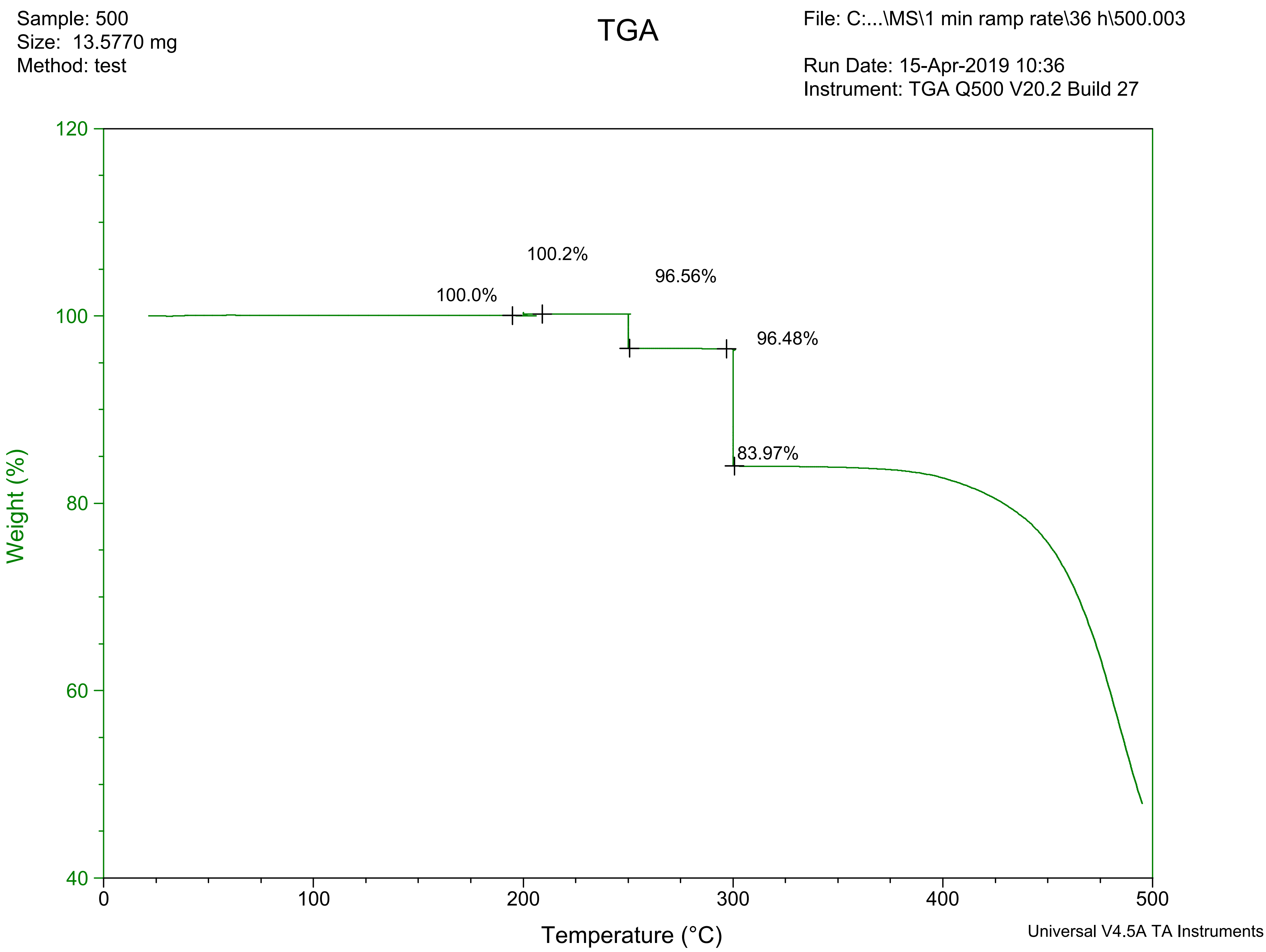
Sample: 500
Size: 13.5770 mg
Method: test
TGA
File: C:...\MS\1 min ramp rate\36 h\500.003
Run Date: 15-Apr-2019 10:36
Instrument: TGA Q500 V20.2 Build 27
100.2%
96.56%
100.0%
96.48%
83.97%
Weight (%)
120
100
80
60
40
0
100
200
300
400
500
Temperature (°C)
Universal V4.5A TA Instruments

Sample: 500
Size: 13.5770 mg
Method: test

TGA

File: C:...\MS\1 min ramp rate\36 h\500.003

Run Date: 15-Apr-2019 10:36
Instrument: TGA Q500 V20.2 Build 27

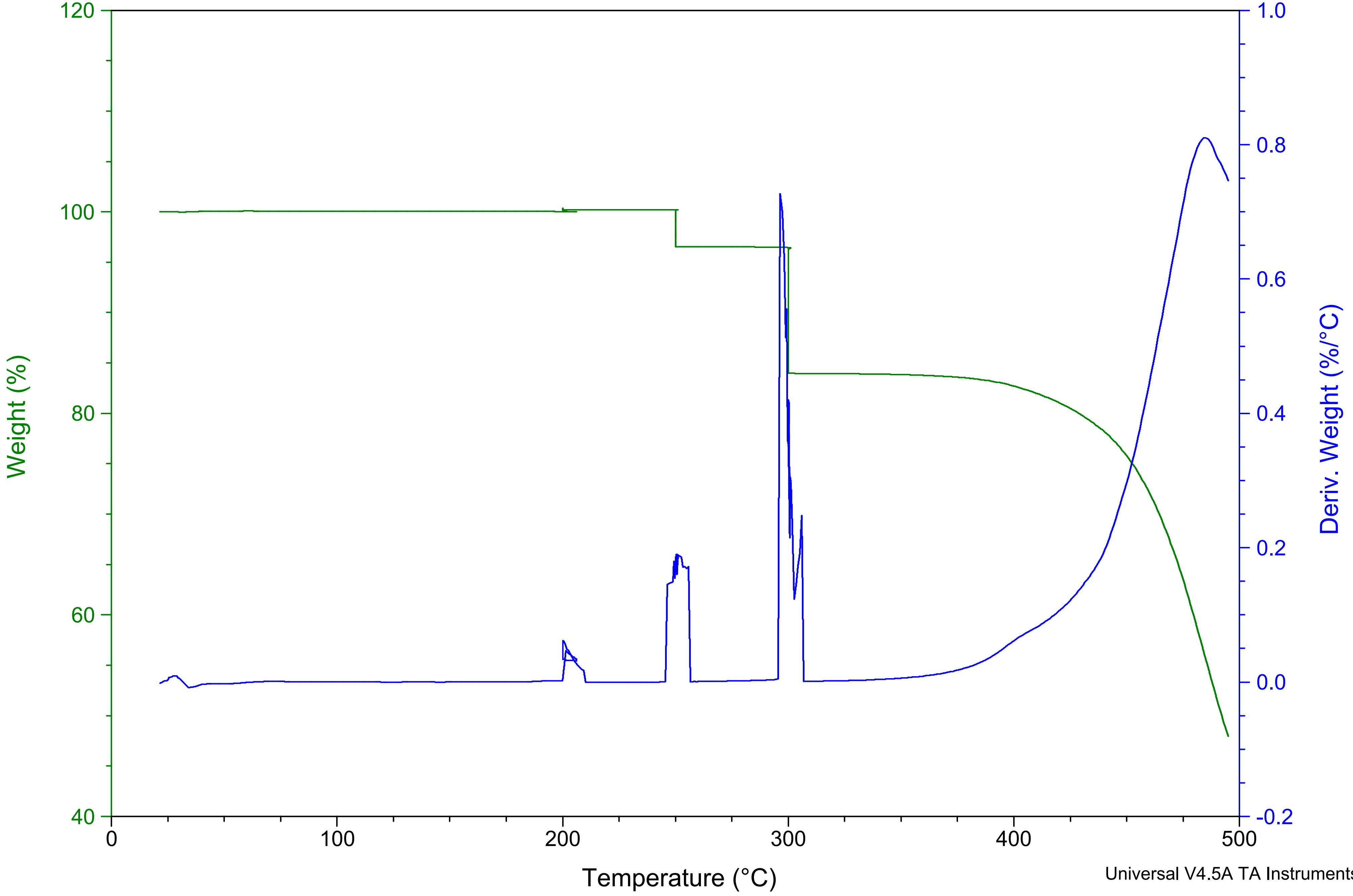

Universal V4.5A TA Instruments

Sample: 503
Size: 10.3000 mg
Method: test

TGA

File: C:...\MS\1 min ramp rate\36 h\503.001

Run Date: 10-Apr-2019 09:17
Instrument: TGA Q500 V20.2 Build 27

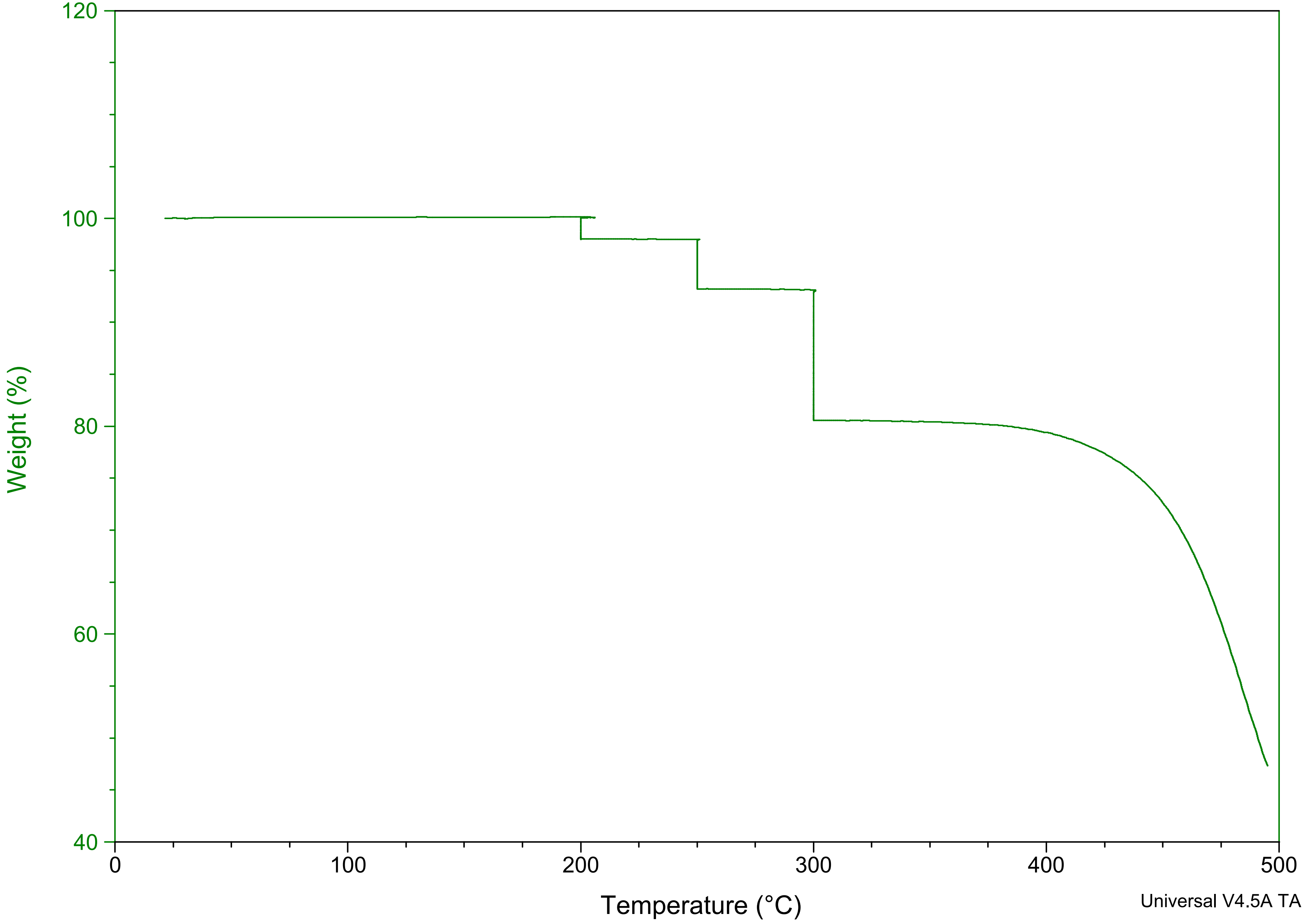

Universal V4.5A TA Instruments

Sample: 503
Size: 10.3000 mg
Method: test

TGA

File: C:...\MS\1 min ramp rate\36 h\503.001

Run Date: 10-Apr-2019 09:17
Instrument: TGA Q500 V20.2 Build 27

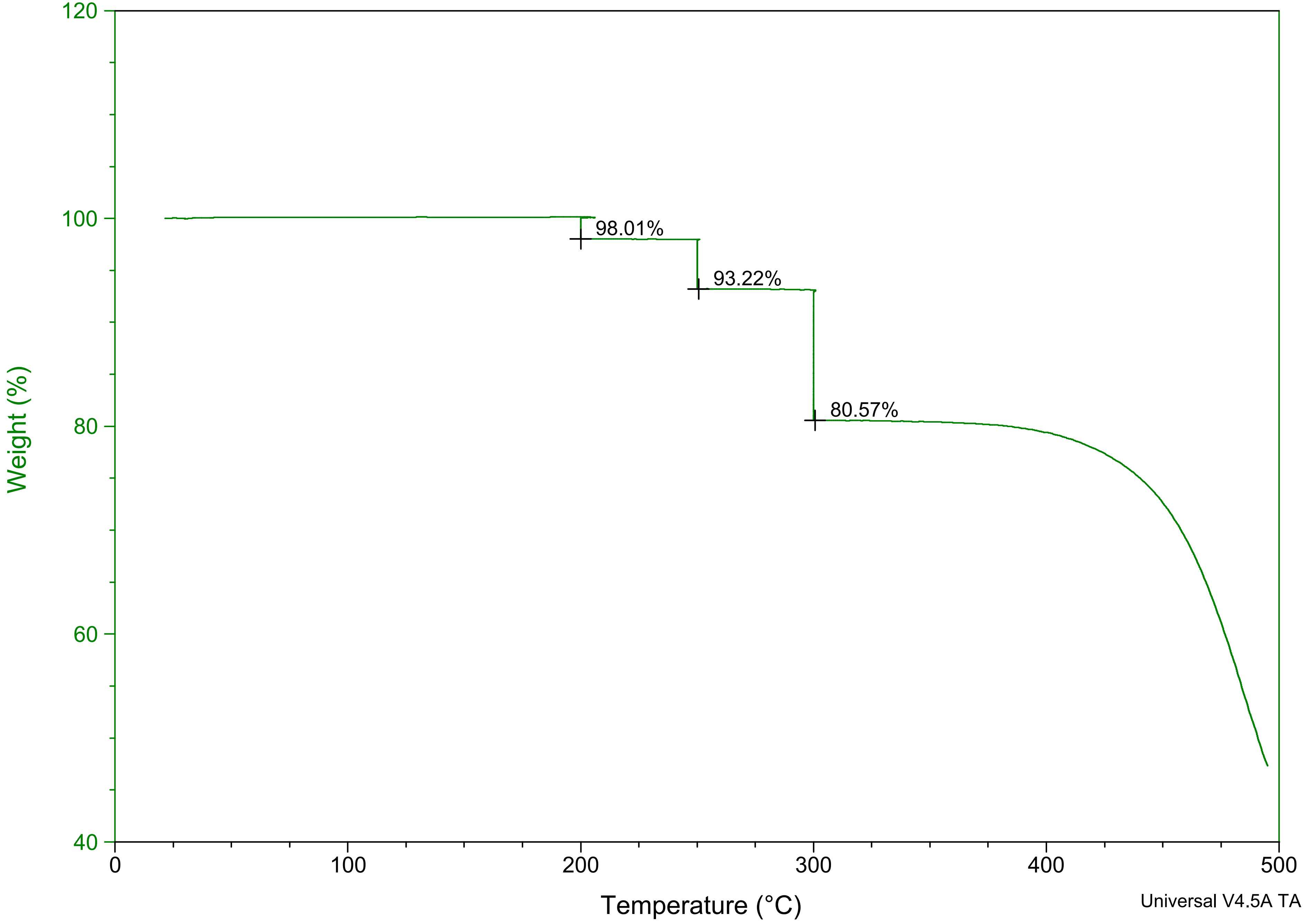

Universal V4.5A TA Instruments

Sample: 503
Size: 10.3000 mg
Method: test

TGA

File: C:...\MS\1 min ramp rate\36 h\503.001

Run Date: 10-Apr-2019 09:17
Instrument: TGA Q500 V20.2 Build 27

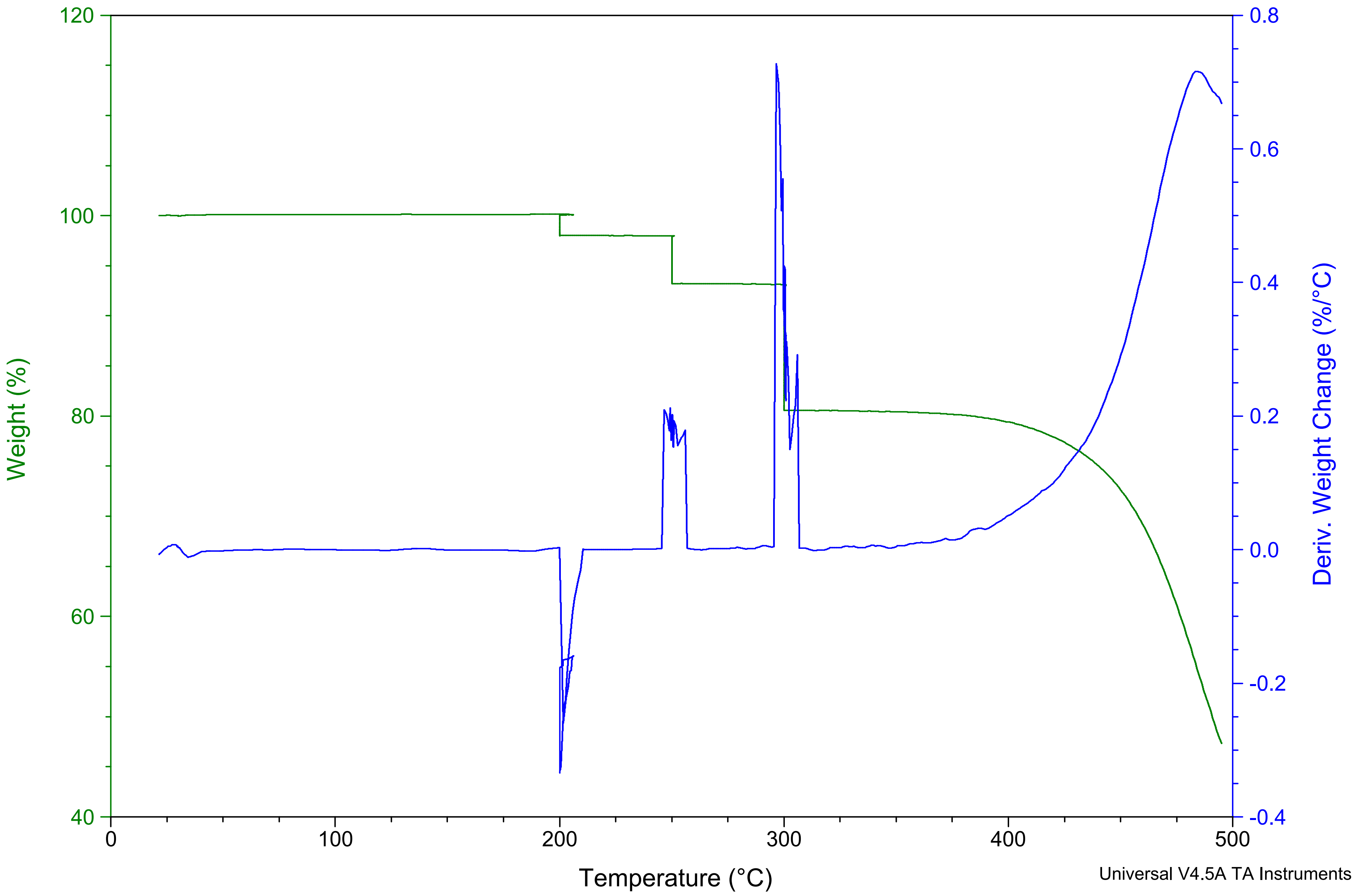

Universal V4.5A TA Instruments

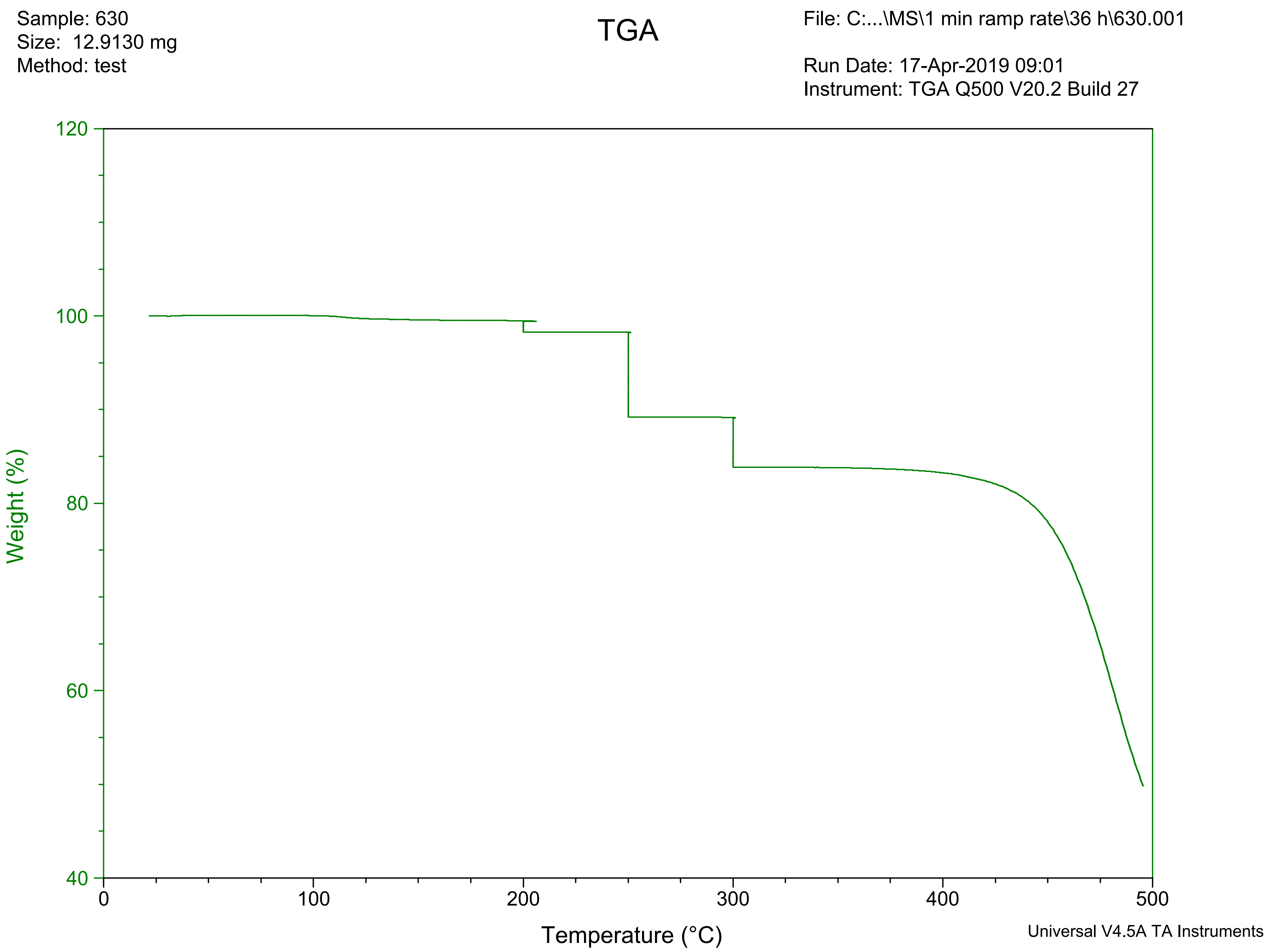

Sample: 630
Size: 12.9130 mg
Method: test
TGA
File: C:...\MS\1 min ramp rate\36 h\630.001
Run Date: 17-Apr-2019 09:01
Instrument: TGA Q500 V20.2 Build 27
120
100
80
60
40
Weight (%)
0
100
200
300
400
500
Temperature (°C)
Universal V4.5A TA Instruments

Sample: 630
Size: 12.9130 mg
Method: test

TGA

File: C:...\MS\1 min ramp rate\36 h\630.001

Run Date: 17-Apr-2019 09:01
Instrument: TGA Q500 V20.2 Build 27

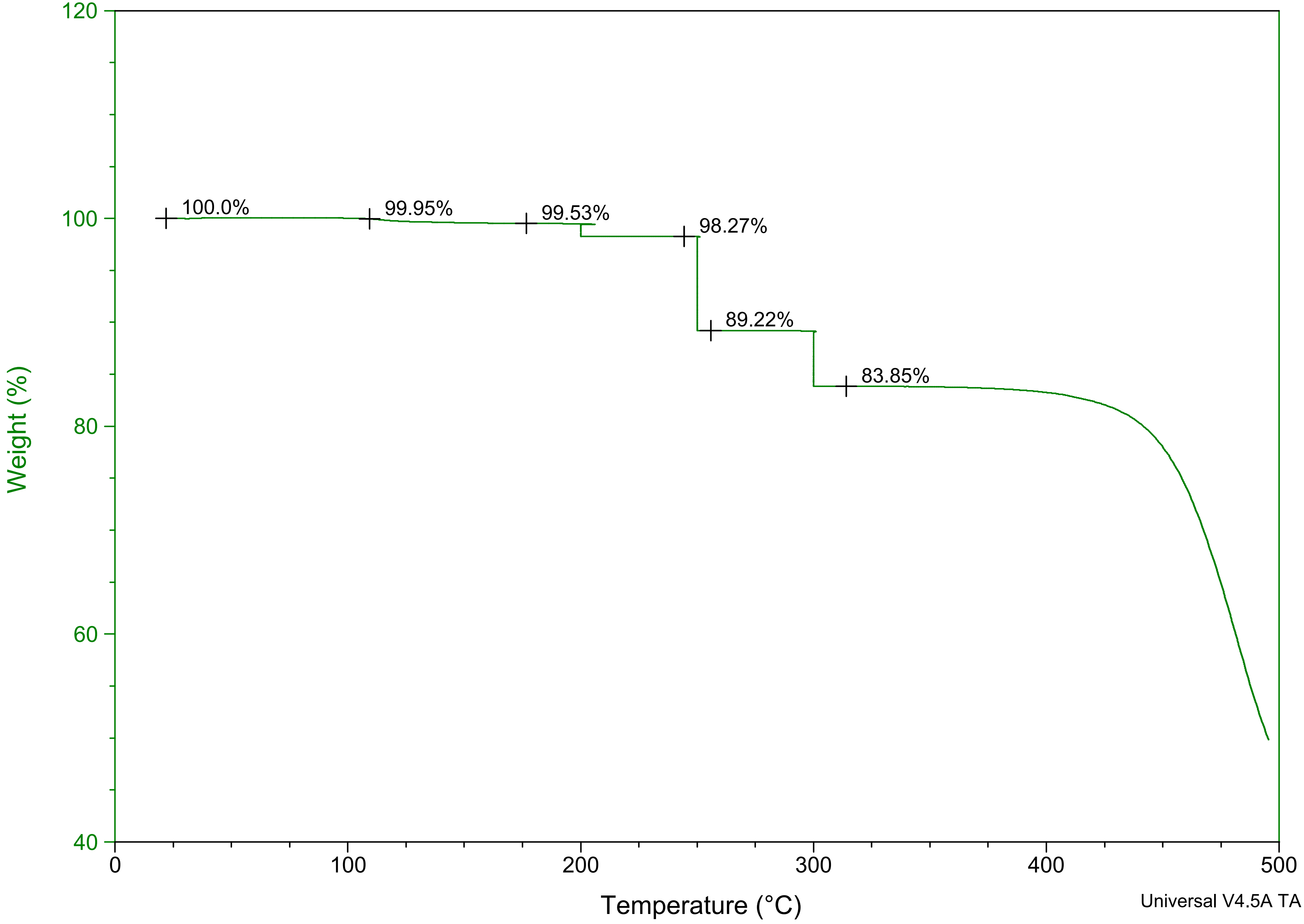

Universal V4.5A TA Instruments

Sample: 630
Size: 12.9130 mg
Method: test

TGA

File: C:...\MS\1 min ramp rate\36 h\630.001

Run Date: 17-Apr-2019 09:01
Instrument: TGA Q500 V20.2 Build 27

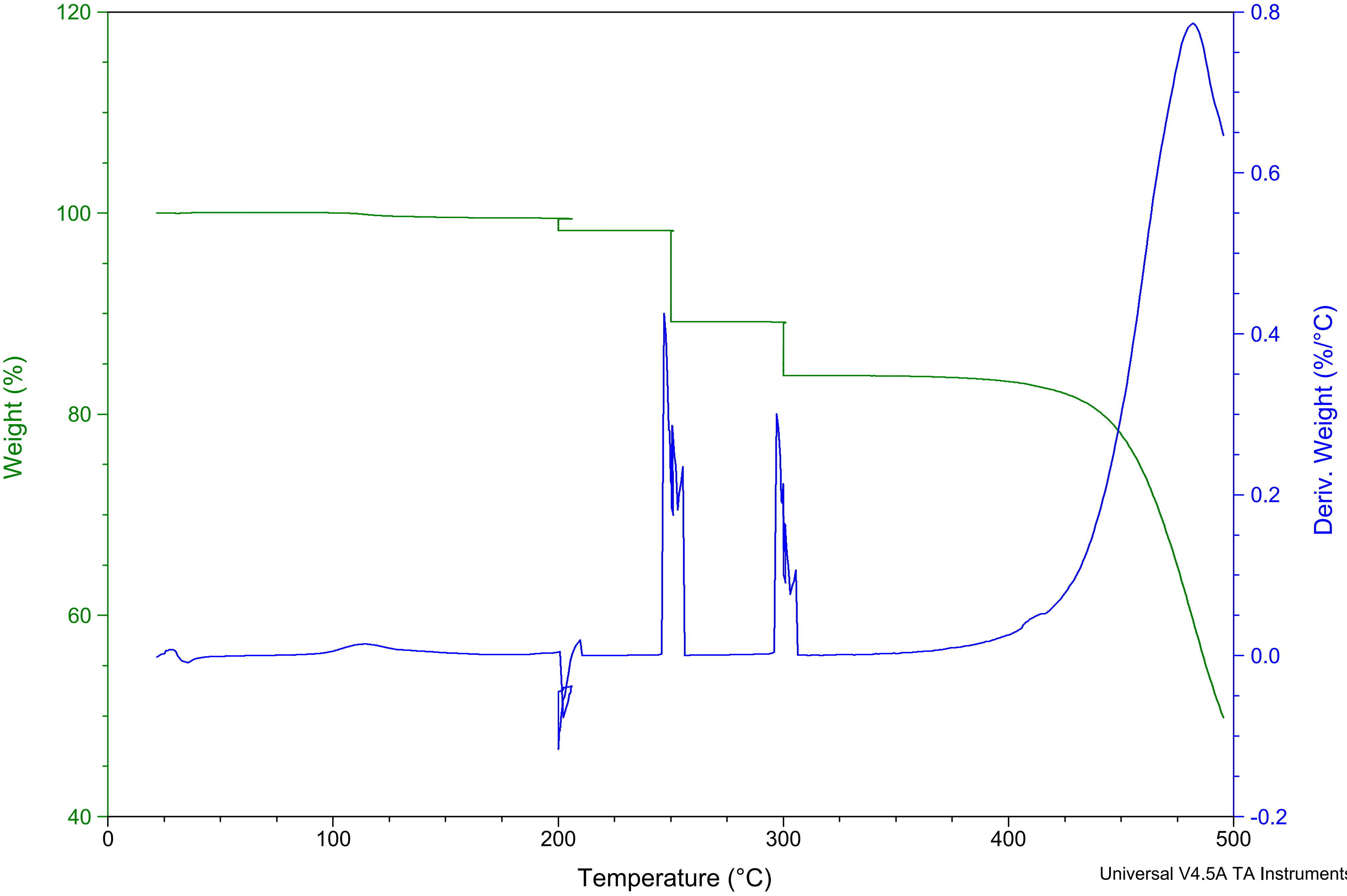

Universal V4.5A TA Instruments
